# Supplementary figures and images for: Regulated microexon alternative splicing in single neurons tunes synaptic function (part 1 of 6)
Source: EMBO Rep. 2025 Jun 9;26(14):3640–62. doi: 10.1038/s44319-025-00493-7 (PMC12287369; doi:10.1038/s44319-025-00493-7)

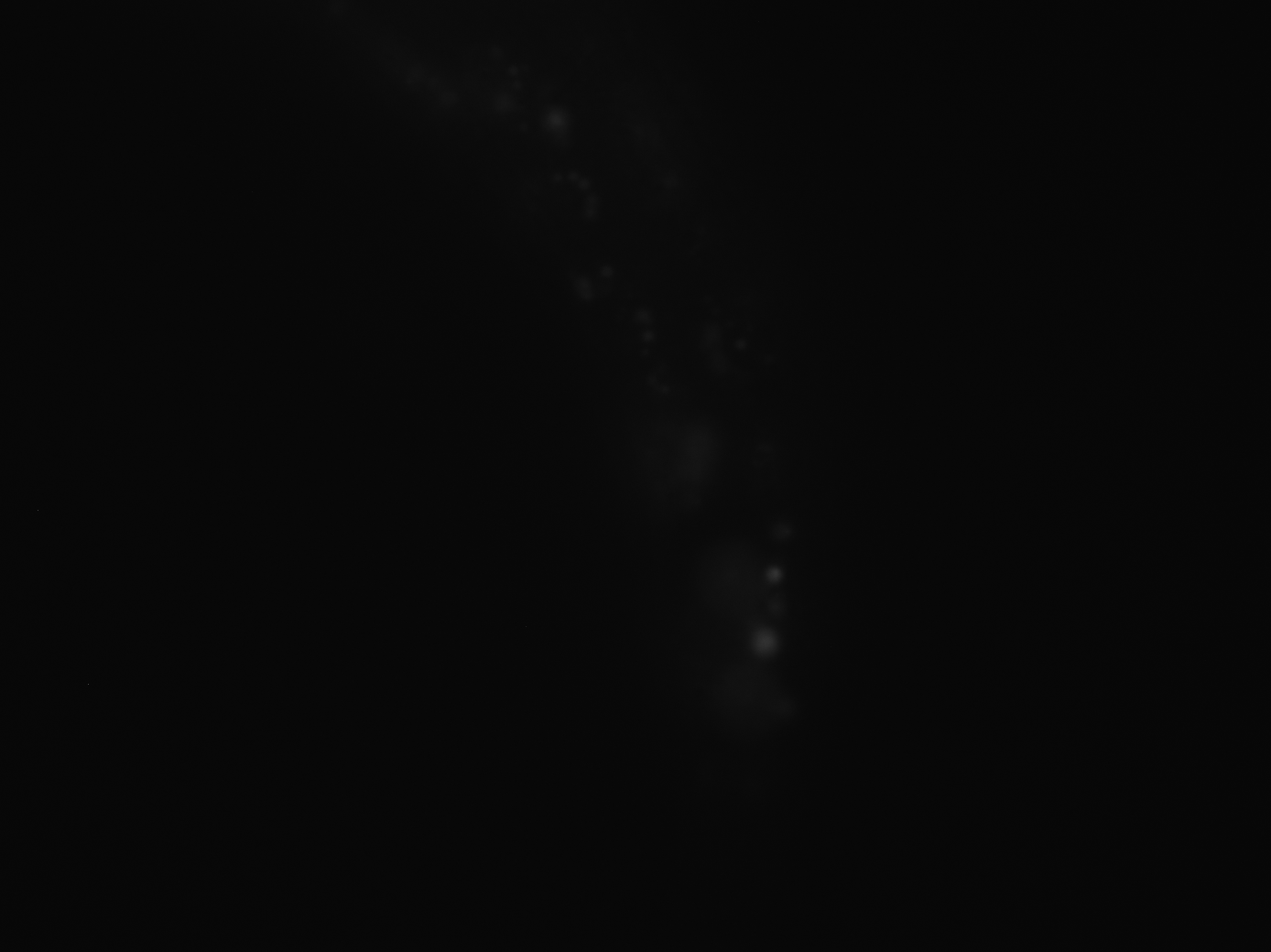

Supplement: Supplementary file 2 — Source data Fig. 1 [file 44319_2025_493_MOESM2_ESM.zip › Figure1/Fig1F/Experiment-11_tail_wildtype.tif_files/Experiment-11_z9c1x0-1388y0-1040.tif]

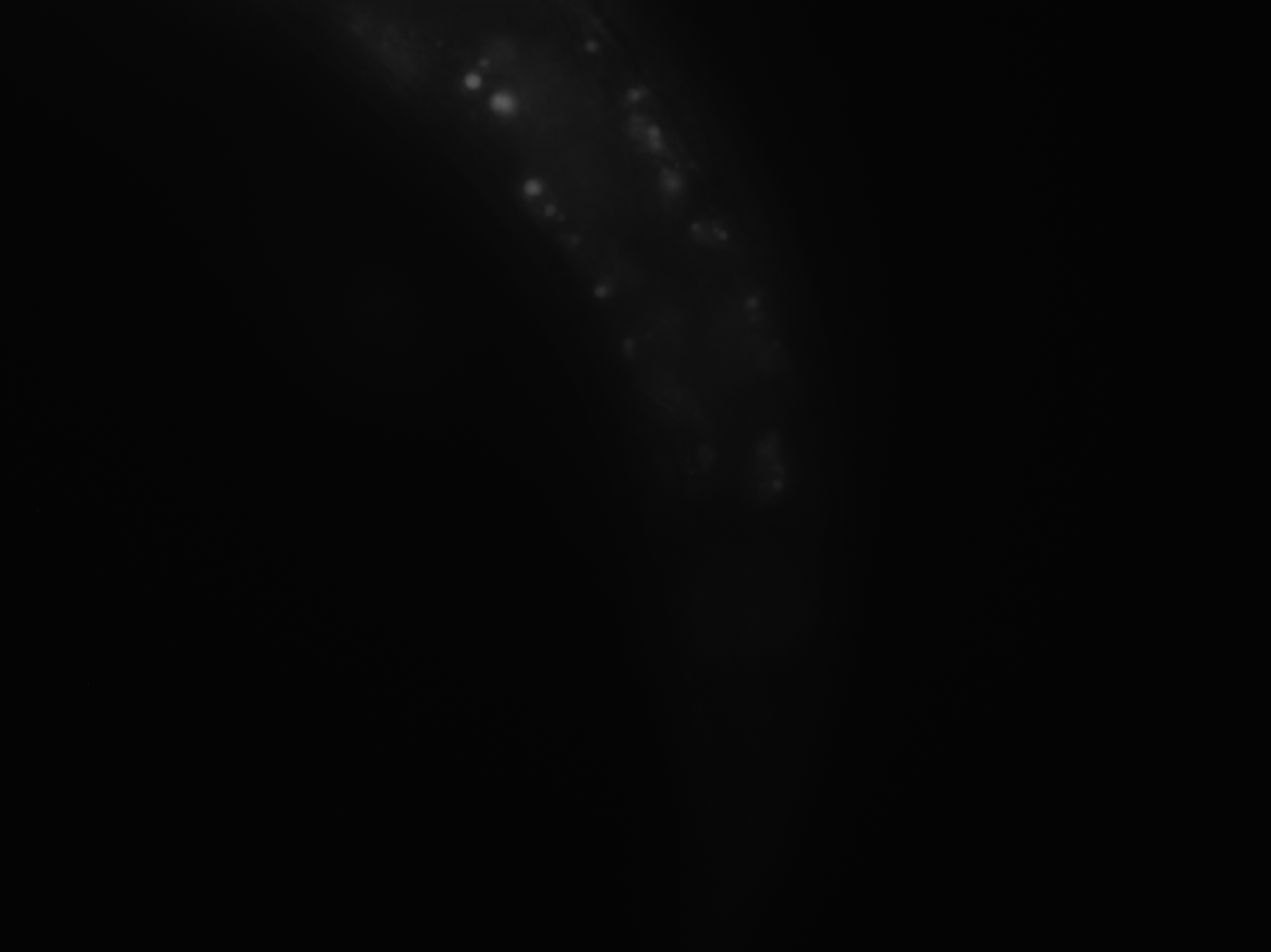

Supplement: Supplementary file 2 — Source data Fig. 1 [file 44319_2025_493_MOESM2_ESM.zip › Figure1/Fig1F/Experiment-11_tail_wildtype.tif_files/Experiment-11_z15c0x0-1388y0-1040.tif]

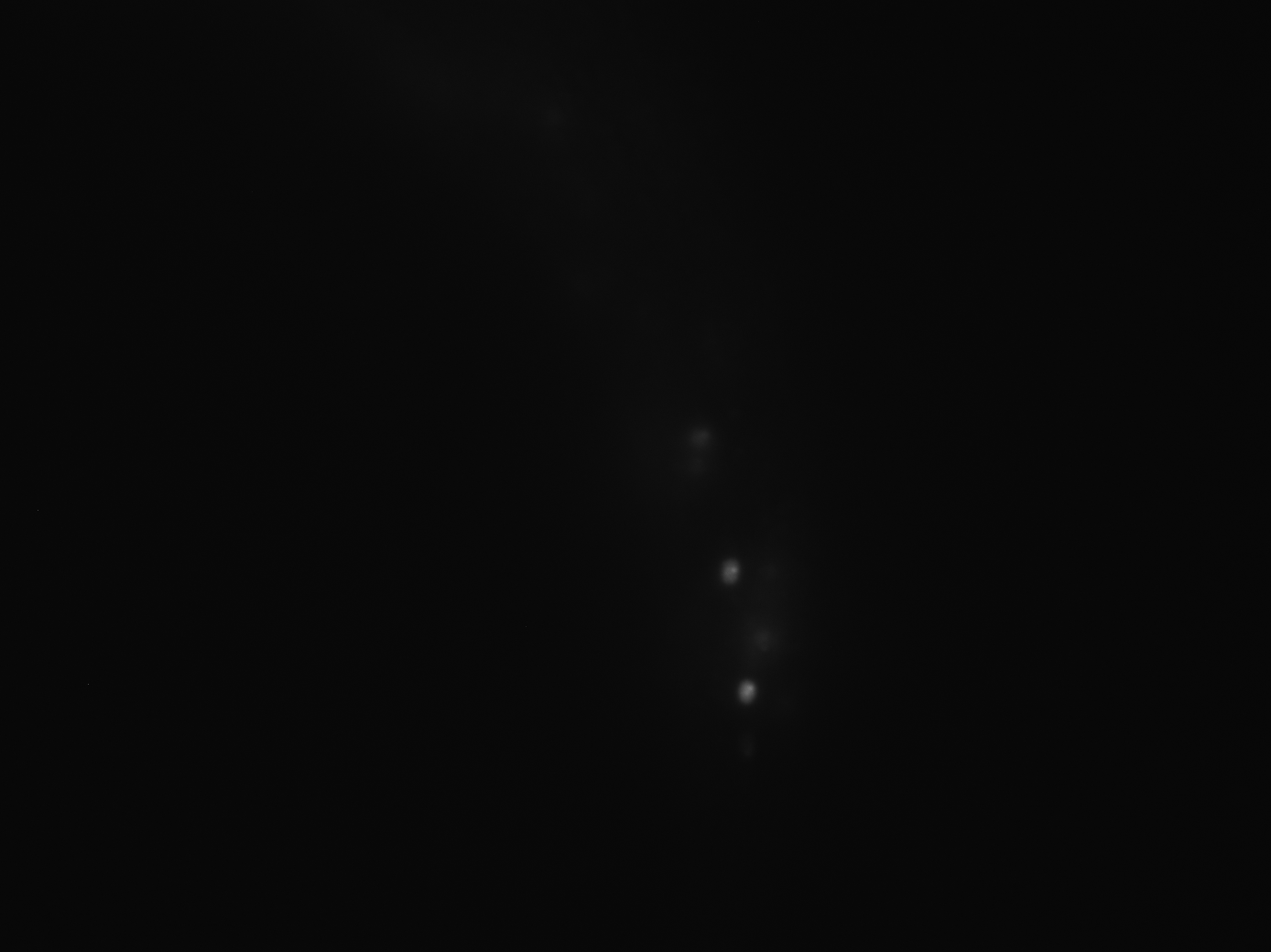

Supplement: Supplementary file 2 — Source data Fig. 1 [file 44319_2025_493_MOESM2_ESM.zip › Figure1/Fig1F/Experiment-11_tail_wildtype.tif_files/Experiment-11_z3c1x0-1388y0-1040.tif]

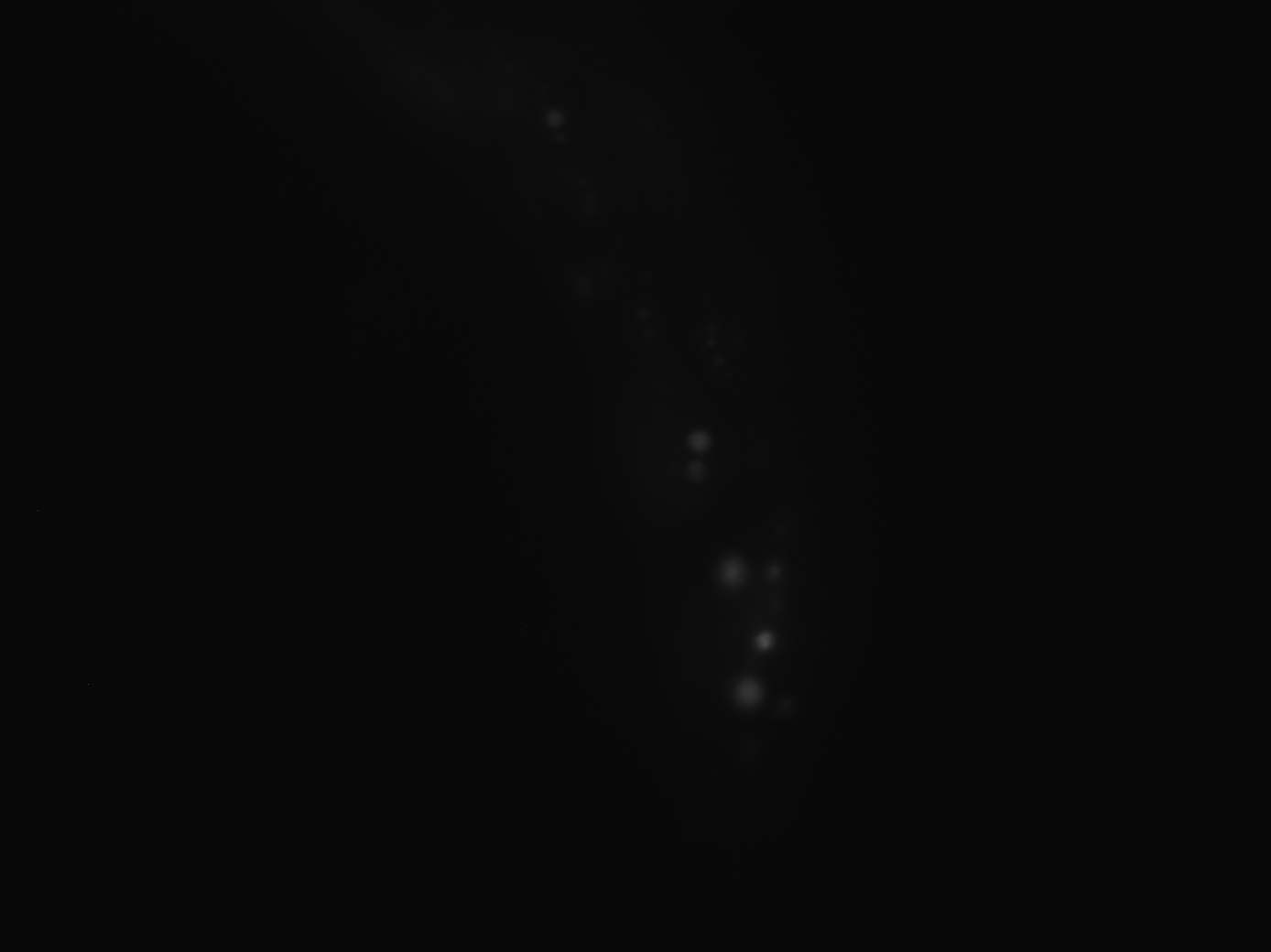

Supplement: Supplementary file 2 — Source data Fig. 1 [file 44319_2025_493_MOESM2_ESM.zip › Figure1/Fig1F/Experiment-11_tail_wildtype.tif_files/Experiment-11_z6c1x0-1388y0-1040.tif]

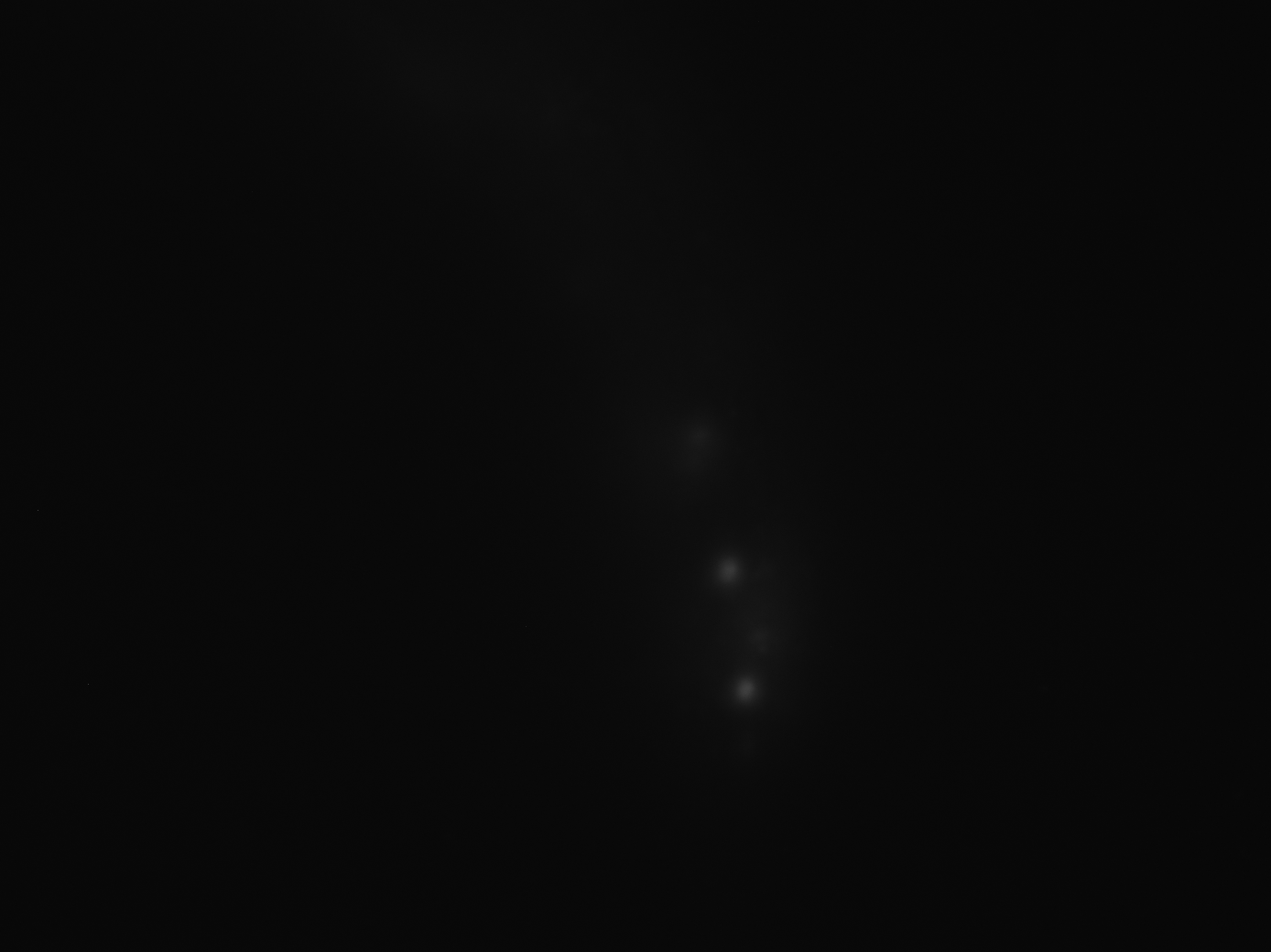

Supplement: Supplementary file 2 — Source data Fig. 1 [file 44319_2025_493_MOESM2_ESM.zip › Figure1/Fig1F/Experiment-11_tail_wildtype.tif_files/Experiment-11_z0c1x0-1388y0-1040.tif]

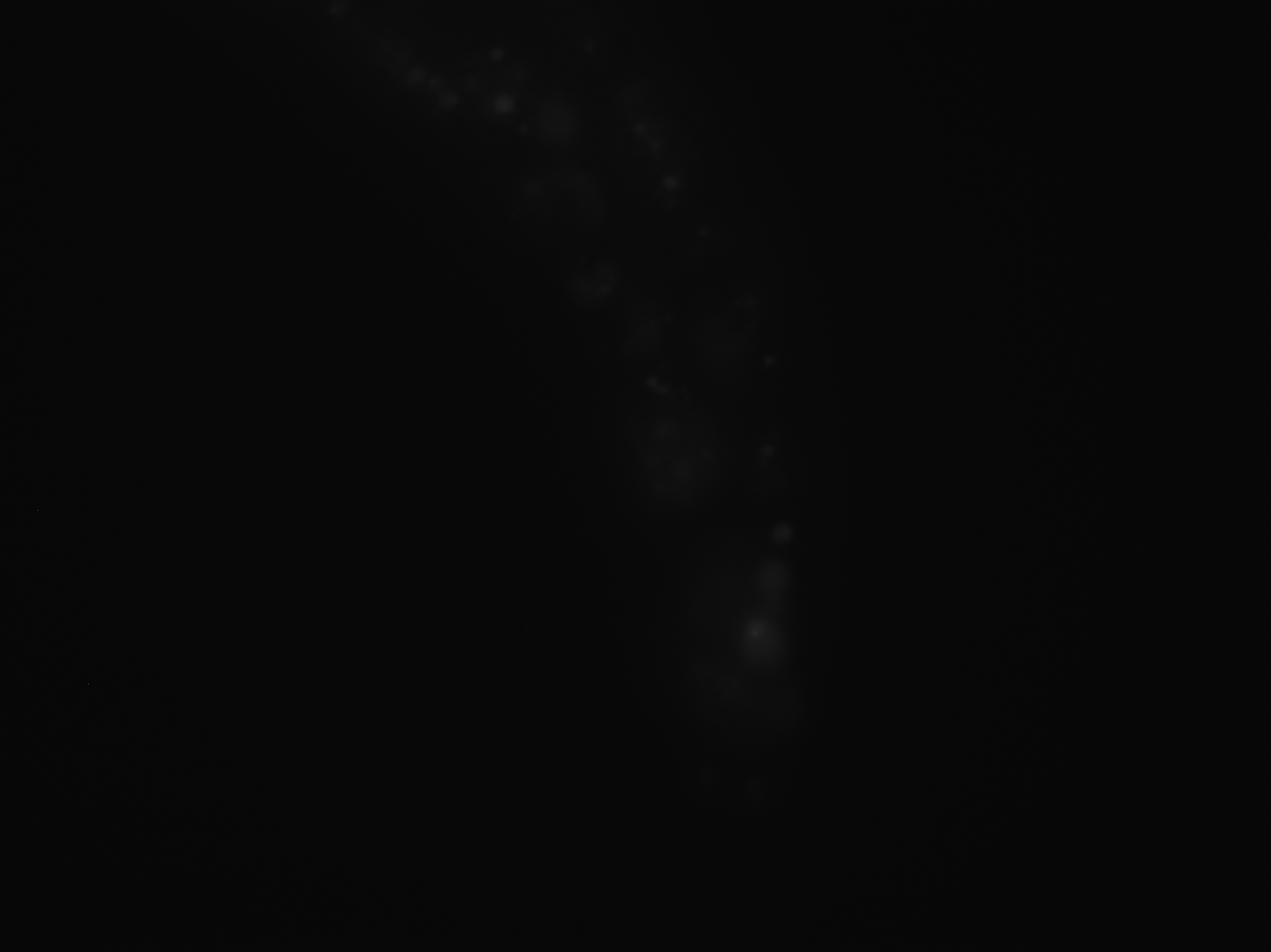

Supplement: Supplementary file 2 — Source data Fig. 1 [file 44319_2025_493_MOESM2_ESM.zip › Figure1/Fig1F/Experiment-11_tail_wildtype.tif_files/Experiment-11_z11c1x0-1388y0-1040.tif]

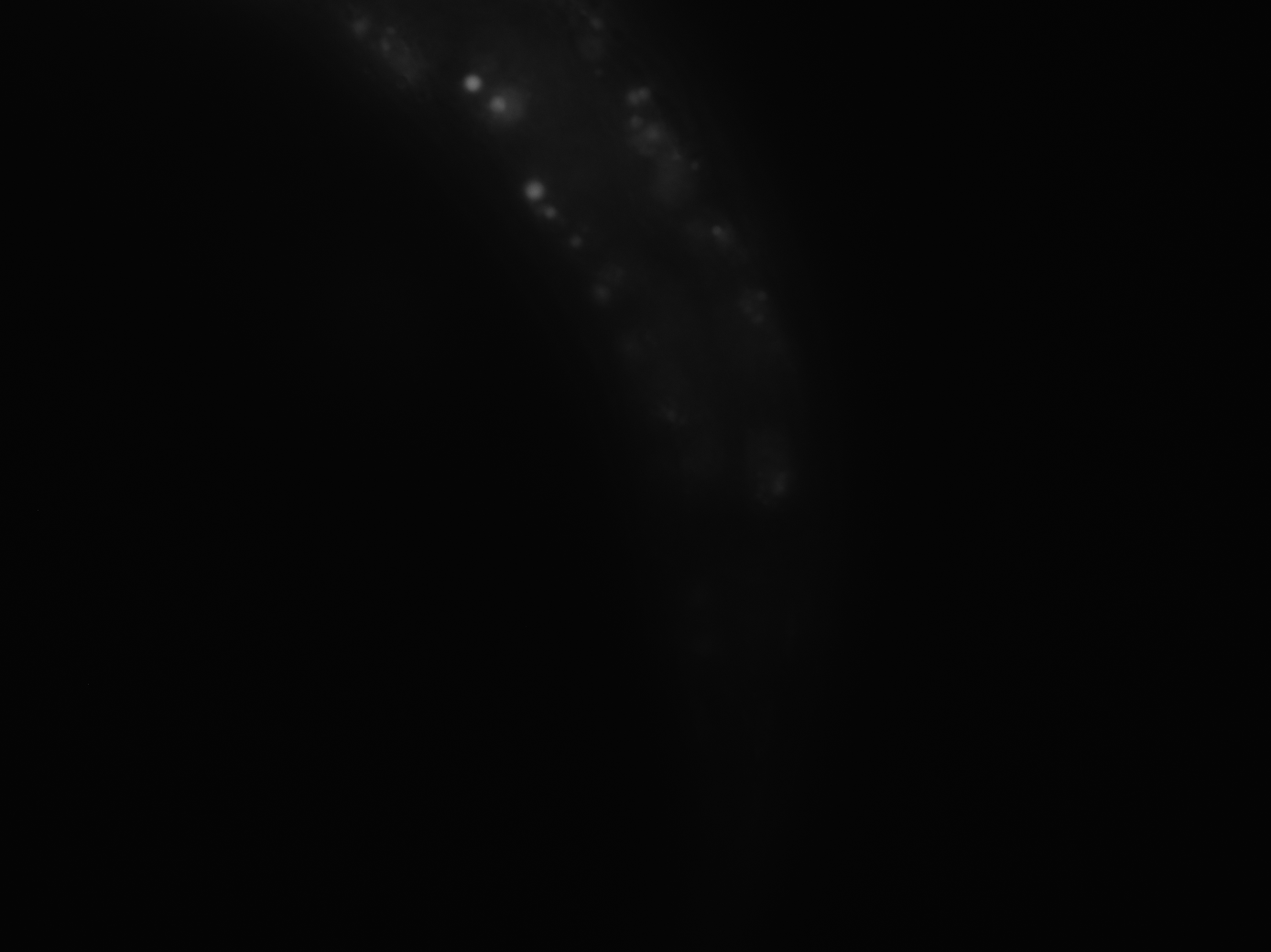

Supplement: Supplementary file 2 — Source data Fig. 1 [file 44319_2025_493_MOESM2_ESM.zip › Figure1/Fig1F/Experiment-11_tail_wildtype.tif_files/Experiment-11_z17c0x0-1388y0-1040.tif]

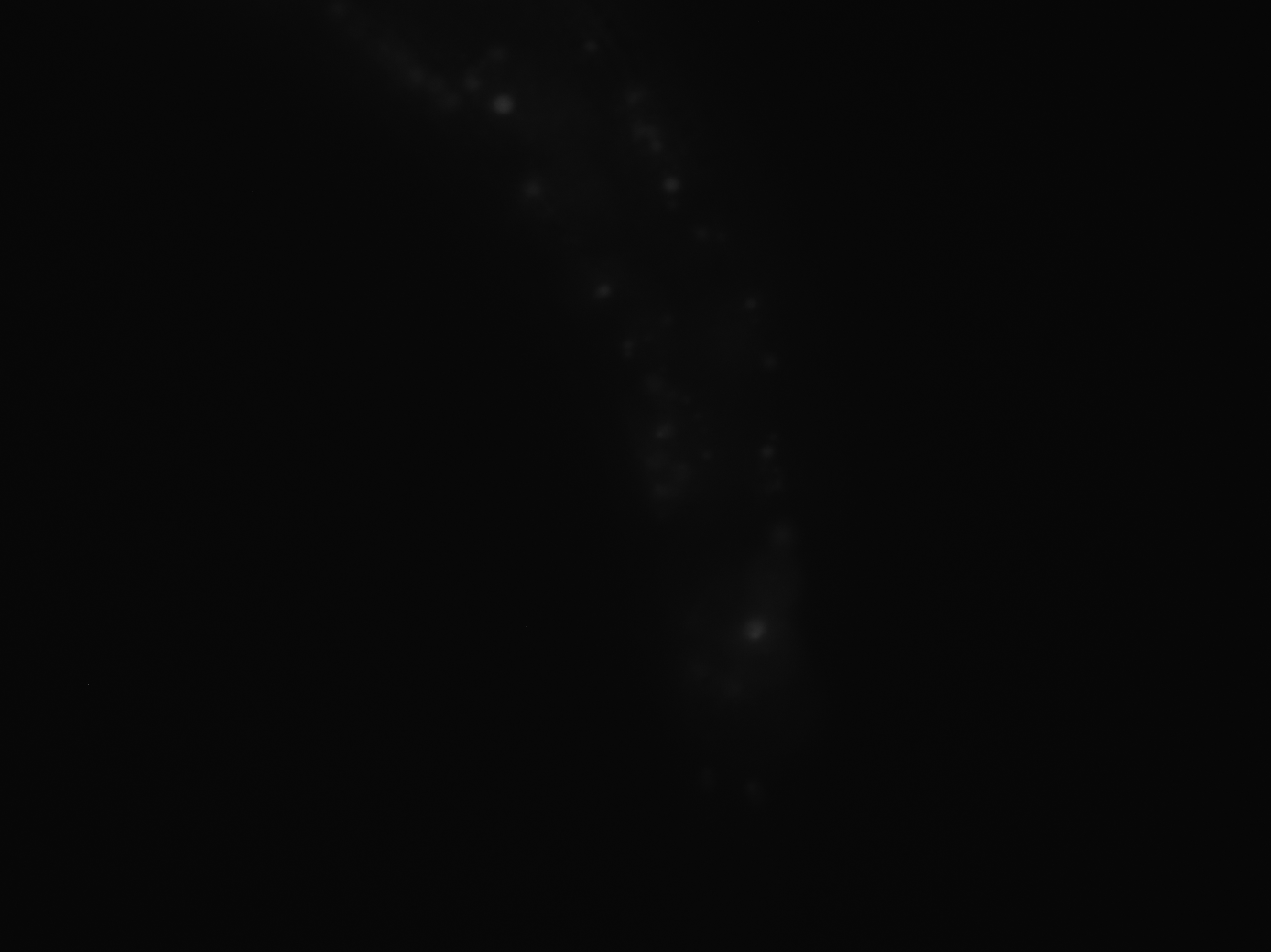

Supplement: Supplementary file 2 — Source data Fig. 1 [file 44319_2025_493_MOESM2_ESM.zip › Figure1/Fig1F/Experiment-11_tail_wildtype.tif_files/Experiment-11_z13c1x0-1388y0-1040.tif]

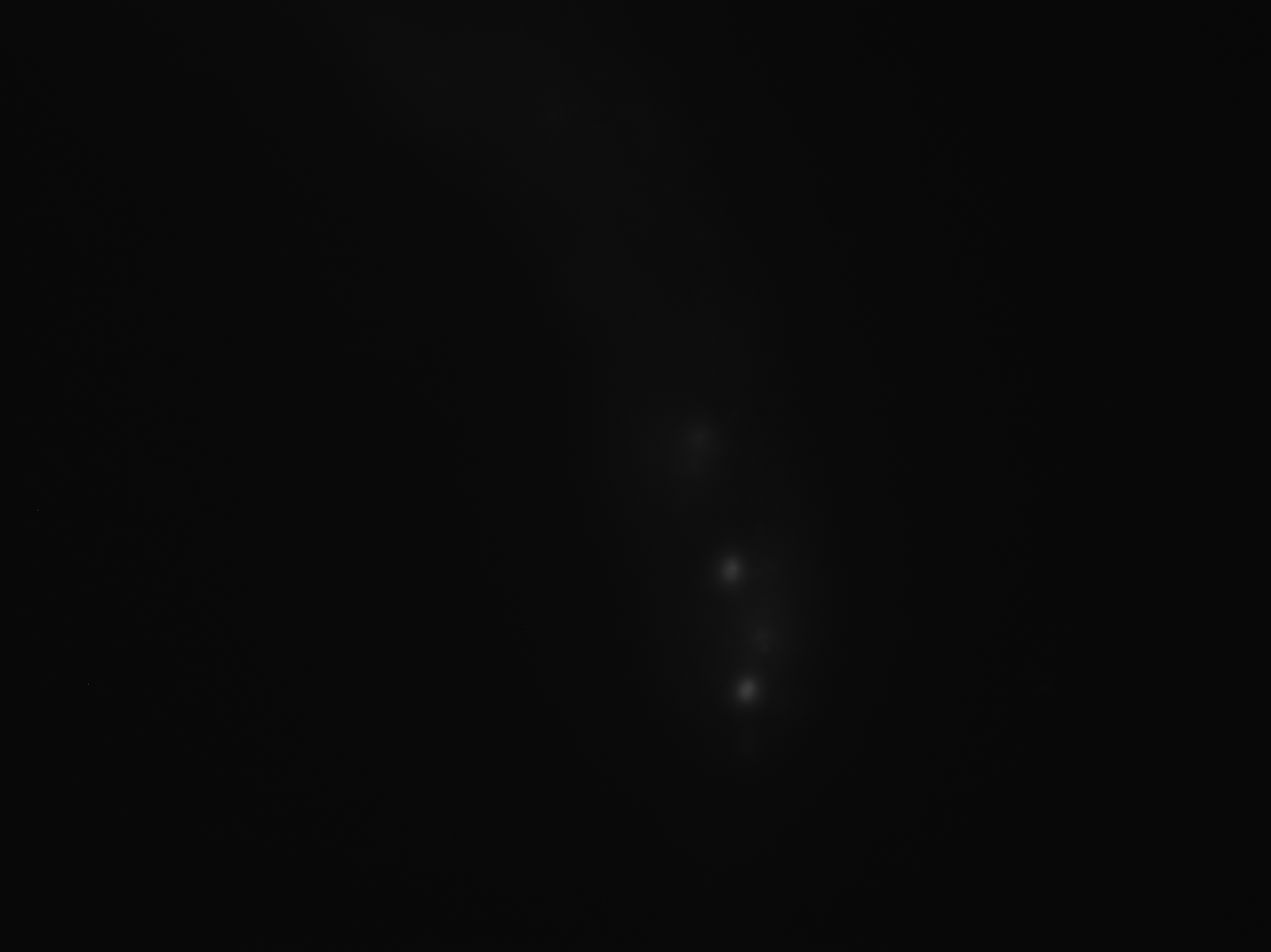

Supplement: Supplementary file 2 — Source data Fig. 1 [file 44319_2025_493_MOESM2_ESM.zip › Figure1/Fig1F/Experiment-11_tail_wildtype.tif_files/Experiment-11_z1c1x0-1388y0-1040.tif]

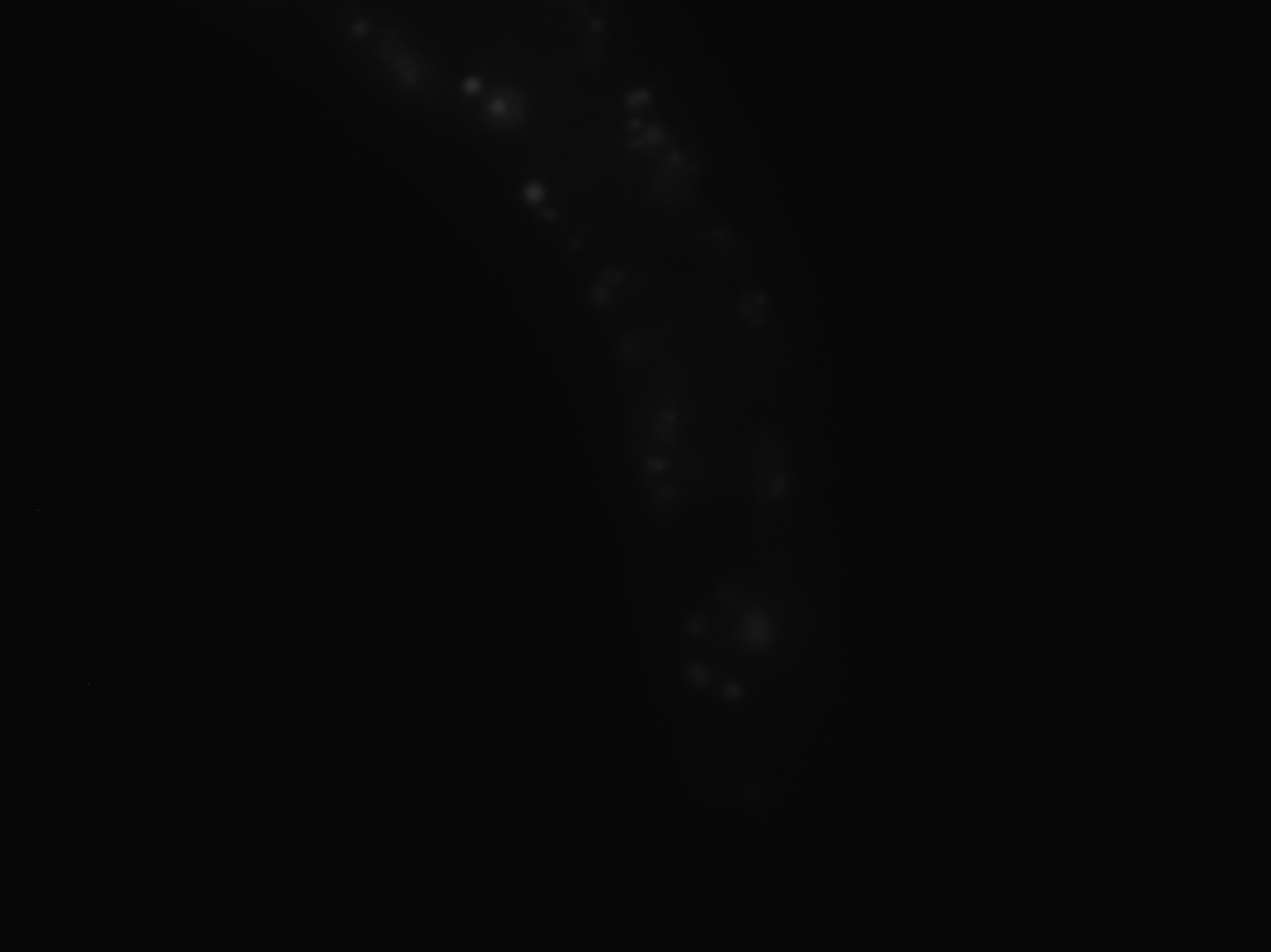

Supplement: Supplementary file 2 — Source data Fig. 1 [file 44319_2025_493_MOESM2_ESM.zip › Figure1/Fig1F/Experiment-11_tail_wildtype.tif_files/Experiment-11_z17c1x0-1388y0-1040.tif]

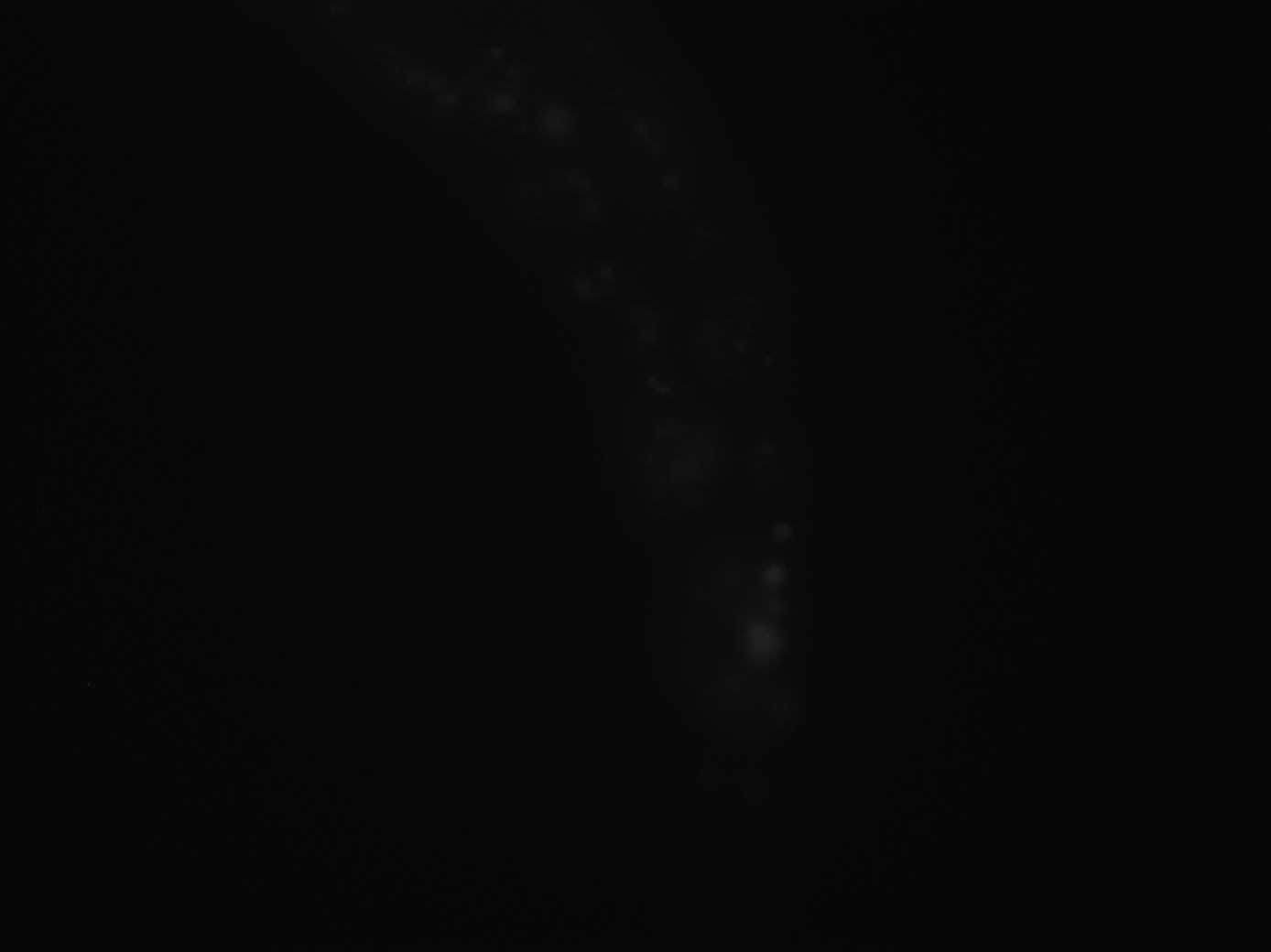

Supplement: Supplementary file 2 — Source data Fig. 1 [file 44319_2025_493_MOESM2_ESM.zip › Figure1/Fig1F/Experiment-11_tail_wildtype.tif_files/Experiment-11_z10c1x0-1388y0-1040.tif]

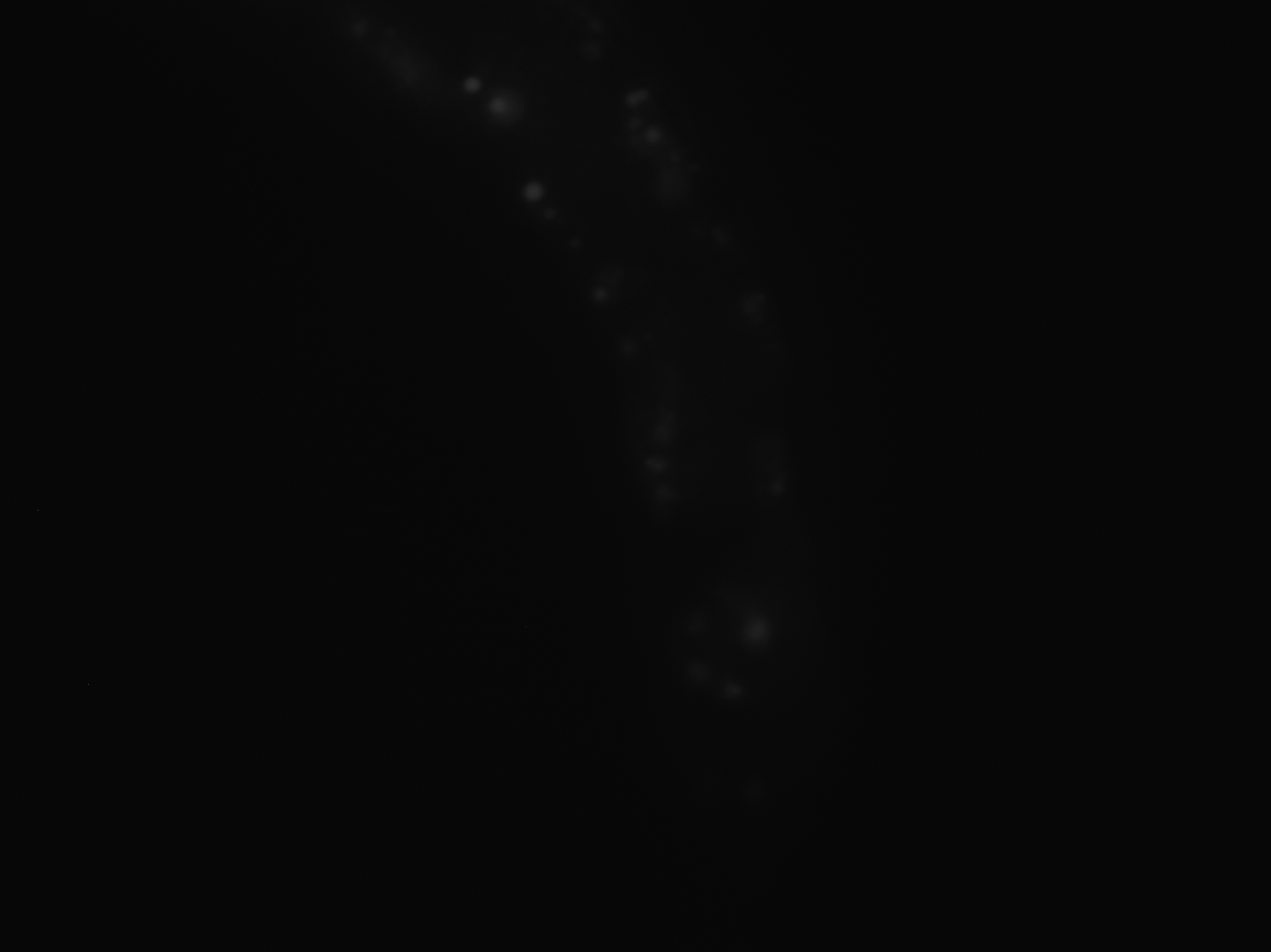

Supplement: Supplementary file 2 — Source data Fig. 1 [file 44319_2025_493_MOESM2_ESM.zip › Figure1/Fig1F/Experiment-11_tail_wildtype.tif_files/Experiment-11_z16c1x0-1388y0-1040.tif]

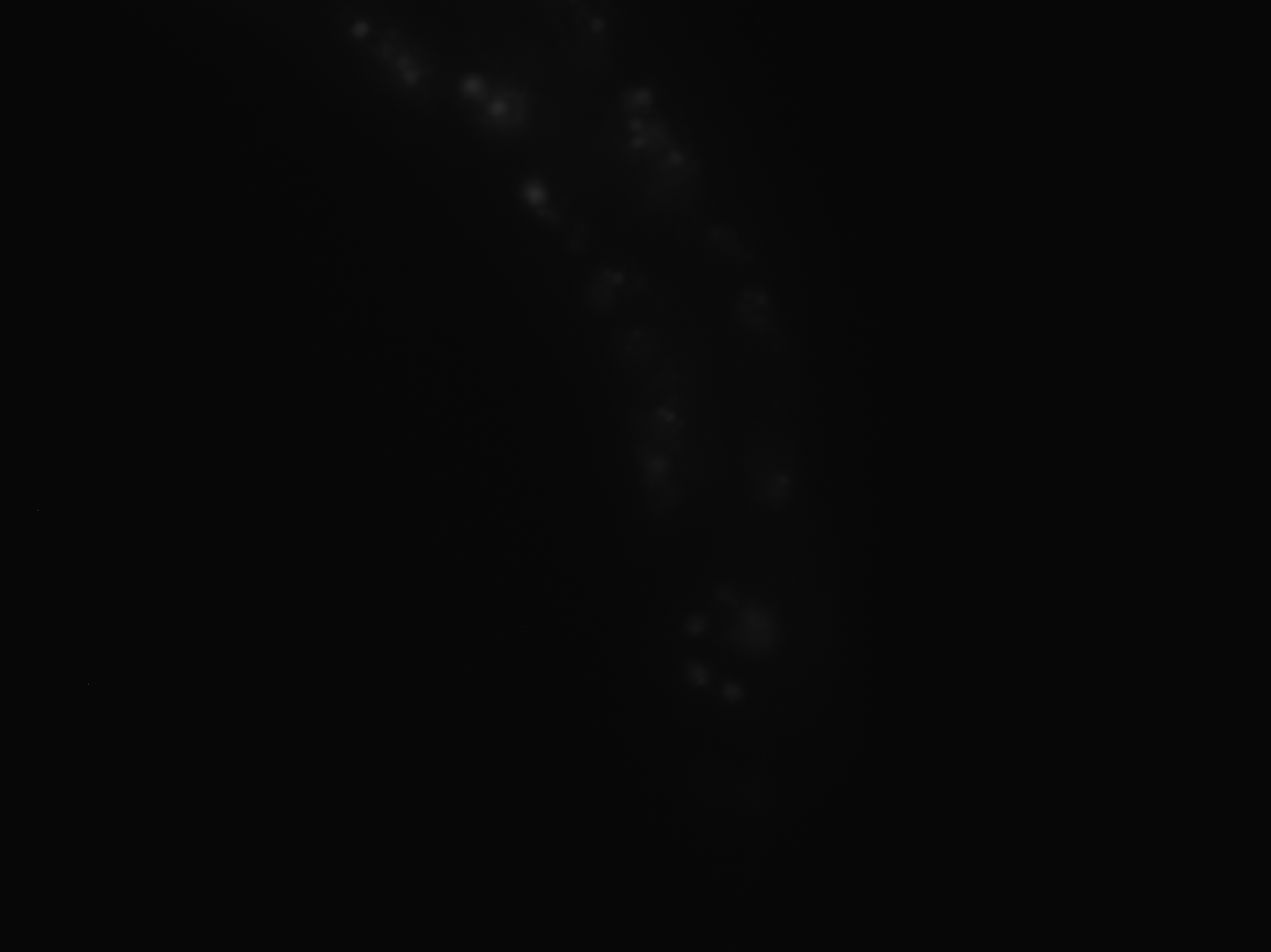

Supplement: Supplementary file 2 — Source data Fig. 1 [file 44319_2025_493_MOESM2_ESM.zip › Figure1/Fig1F/Experiment-11_tail_wildtype.tif_files/Experiment-11_z18c1x0-1388y0-1040.tif]

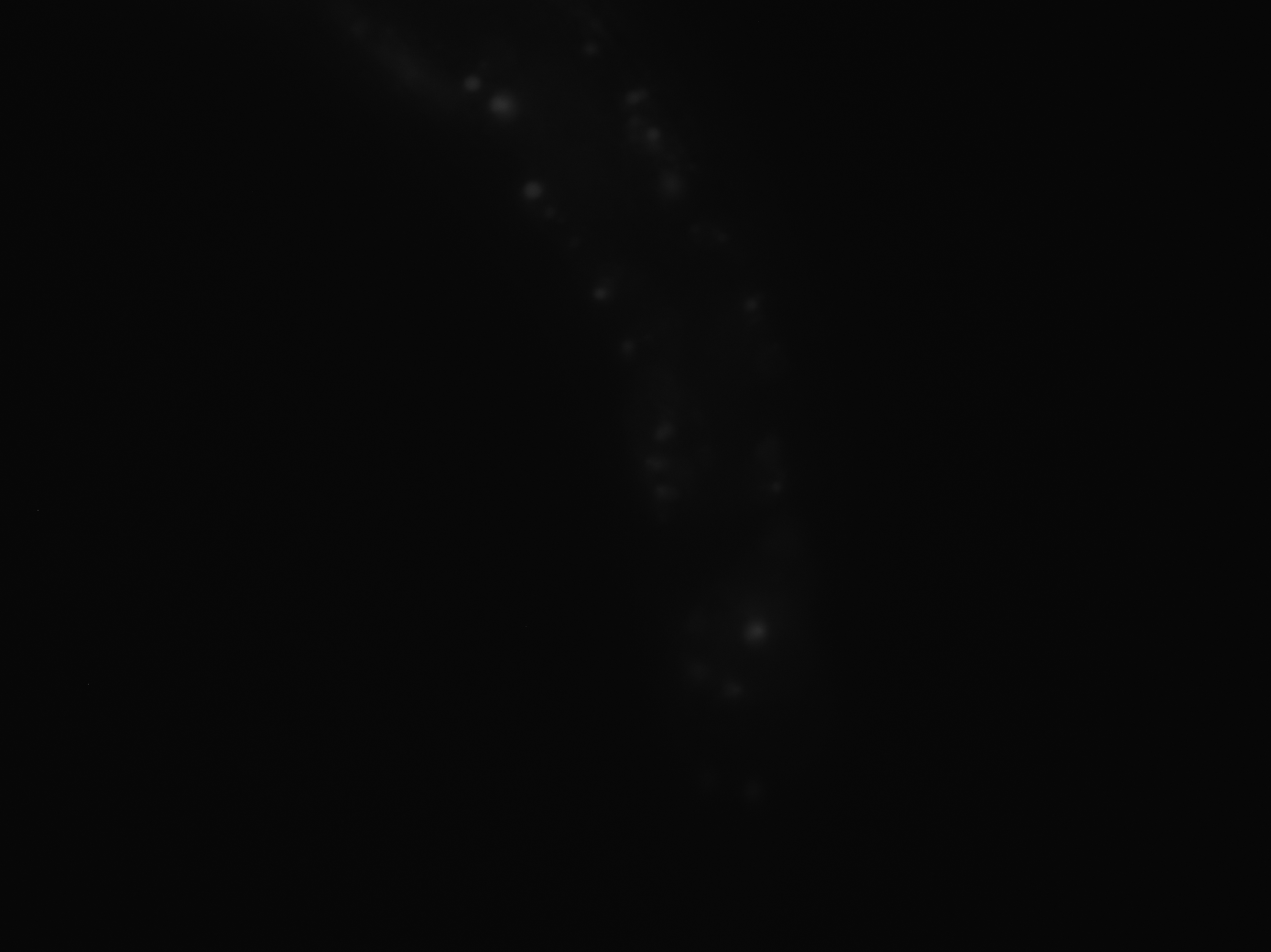

Supplement: Supplementary file 2 — Source data Fig. 1 [file 44319_2025_493_MOESM2_ESM.zip › Figure1/Fig1F/Experiment-11_tail_wildtype.tif_files/Experiment-11_z15c1x0-1388y0-1040.tif]

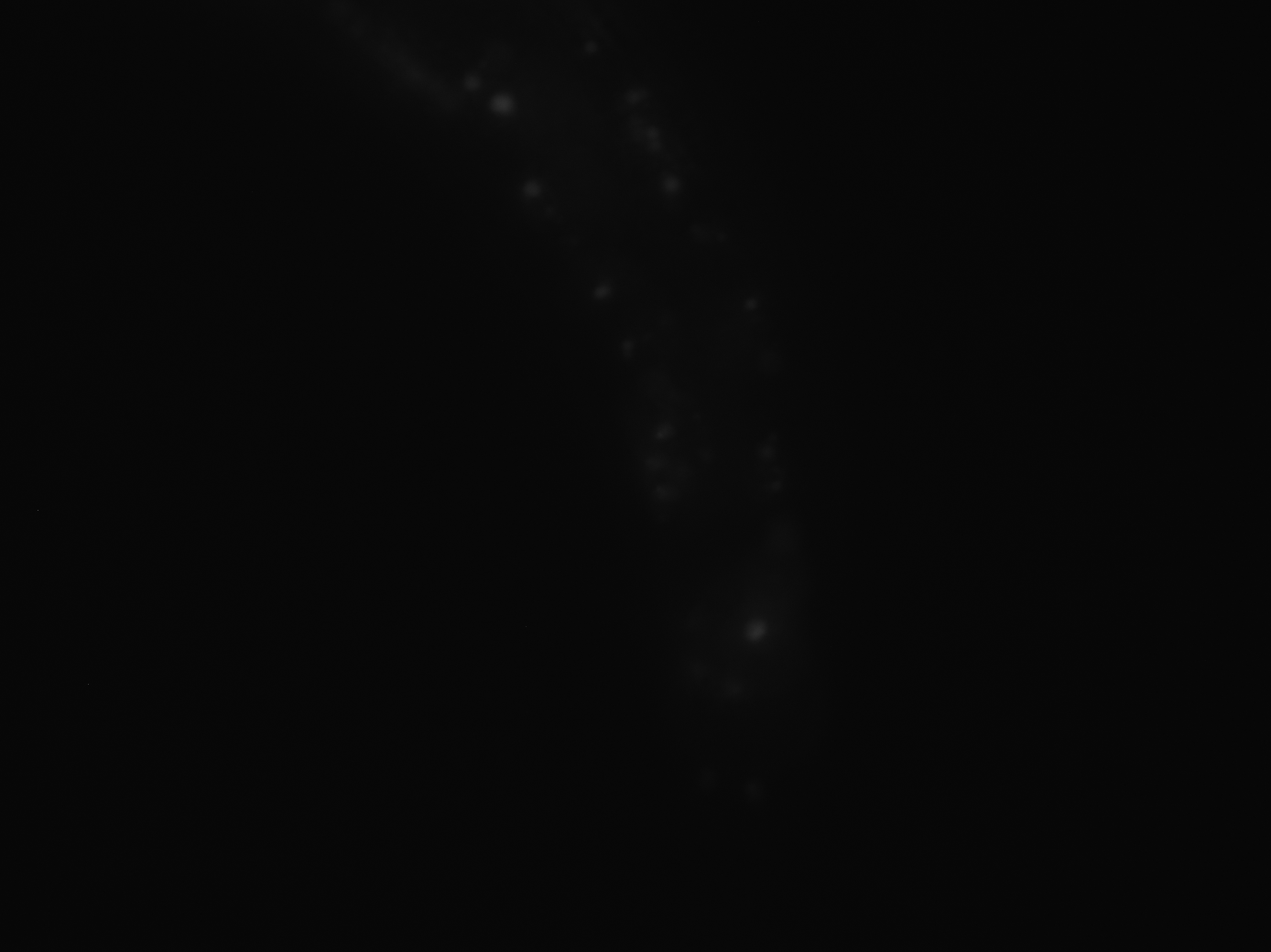

Supplement: Supplementary file 2 — Source data Fig. 1 [file 44319_2025_493_MOESM2_ESM.zip › Figure1/Fig1F/Experiment-11_tail_wildtype.tif_files/Experiment-11_z14c1x0-1388y0-1040.tif]

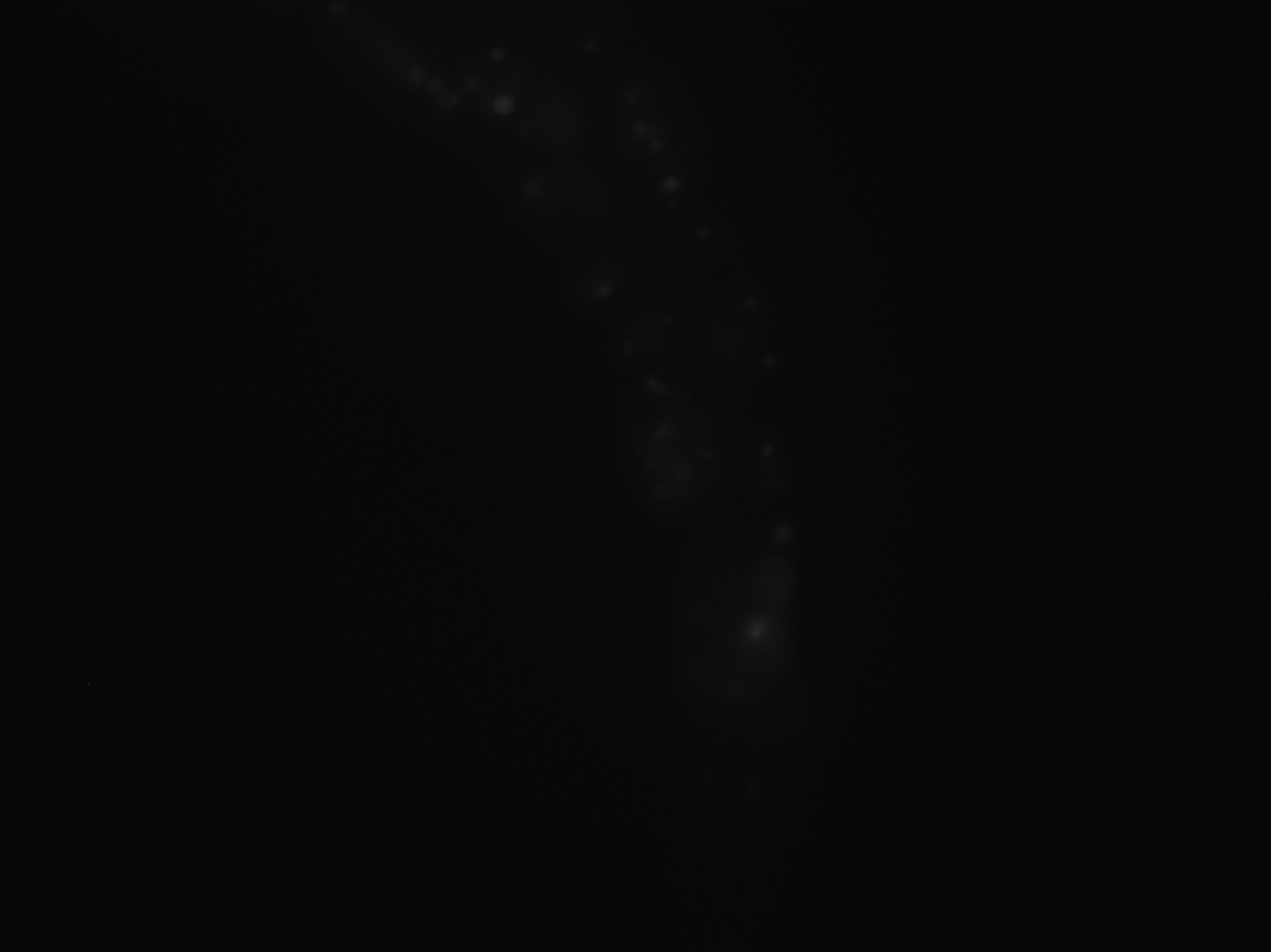

Supplement: Supplementary file 2 — Source data Fig. 1 [file 44319_2025_493_MOESM2_ESM.zip › Figure1/Fig1F/Experiment-11_tail_wildtype.tif_files/Experiment-11_z12c1x0-1388y0-1040.tif]

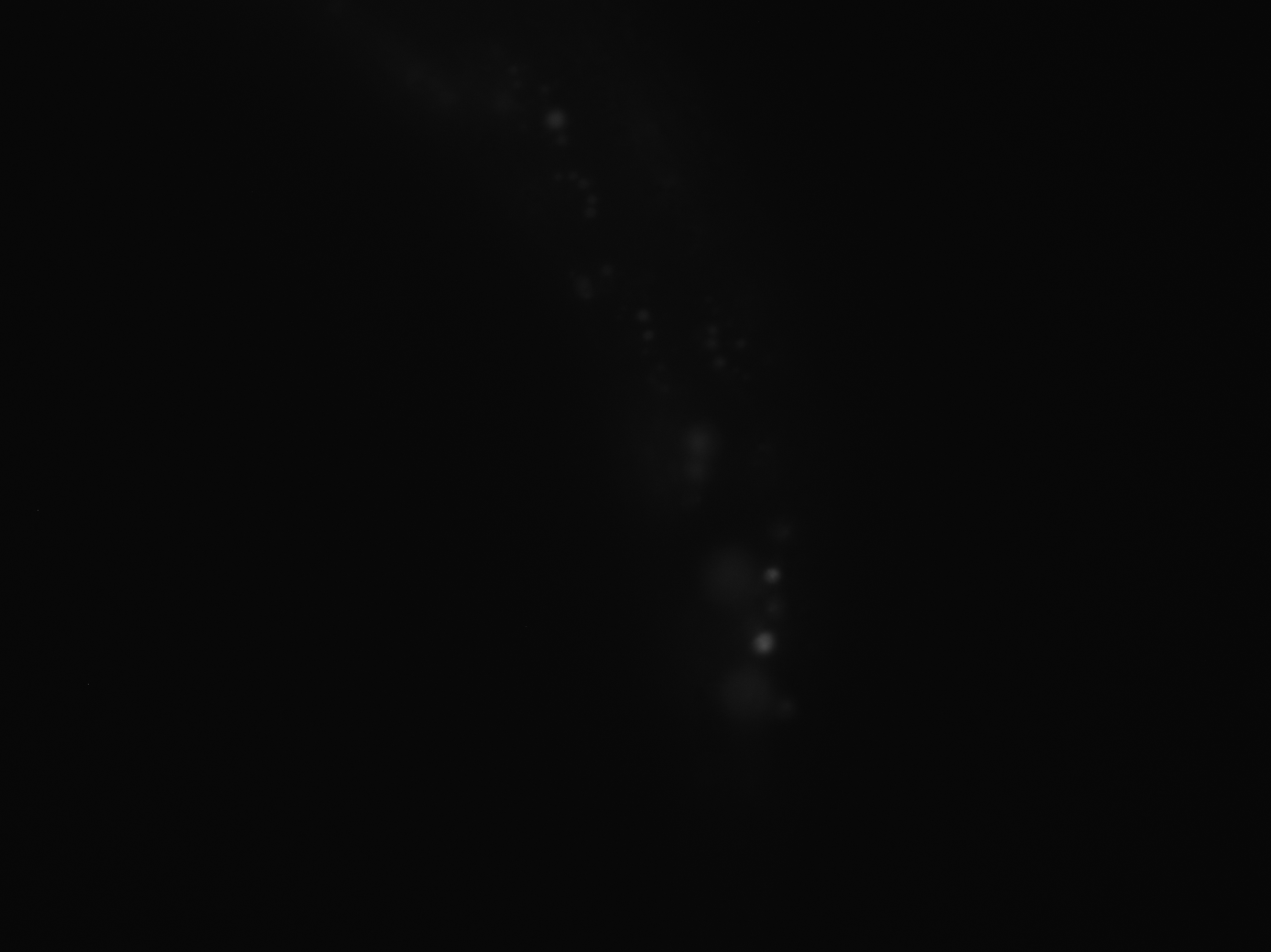

Supplement: Supplementary file 2 — Source data Fig. 1 [file 44319_2025_493_MOESM2_ESM.zip › Figure1/Fig1F/Experiment-11_tail_wildtype.tif_files/Experiment-11_z8c1x0-1388y0-1040.tif]

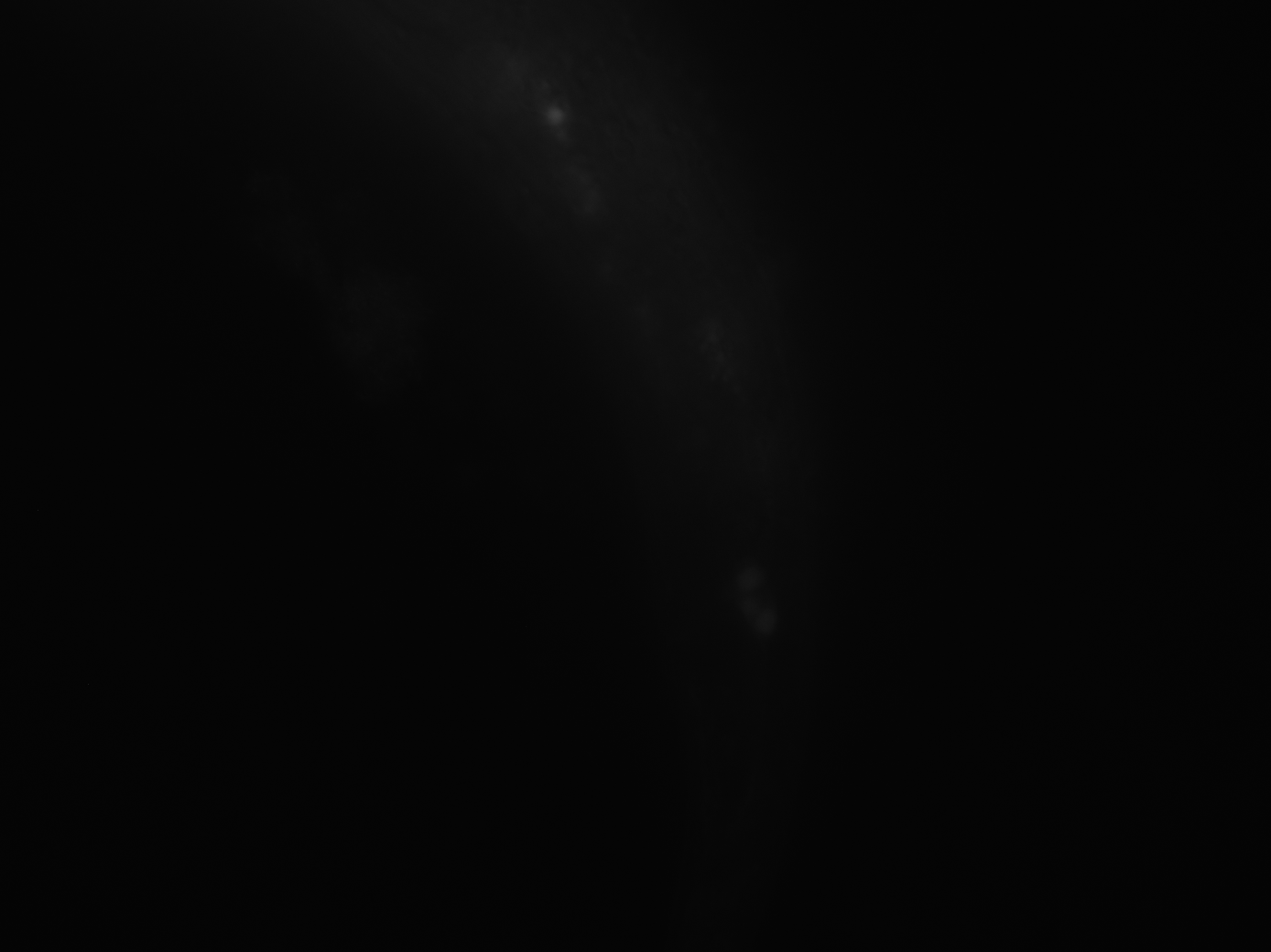

Supplement: Supplementary file 2 — Source data Fig. 1 [file 44319_2025_493_MOESM2_ESM.zip › Figure1/Fig1F/Experiment-11_tail_wildtype.tif_files/Experiment-11_z5c0x0-1388y0-1040.tif]

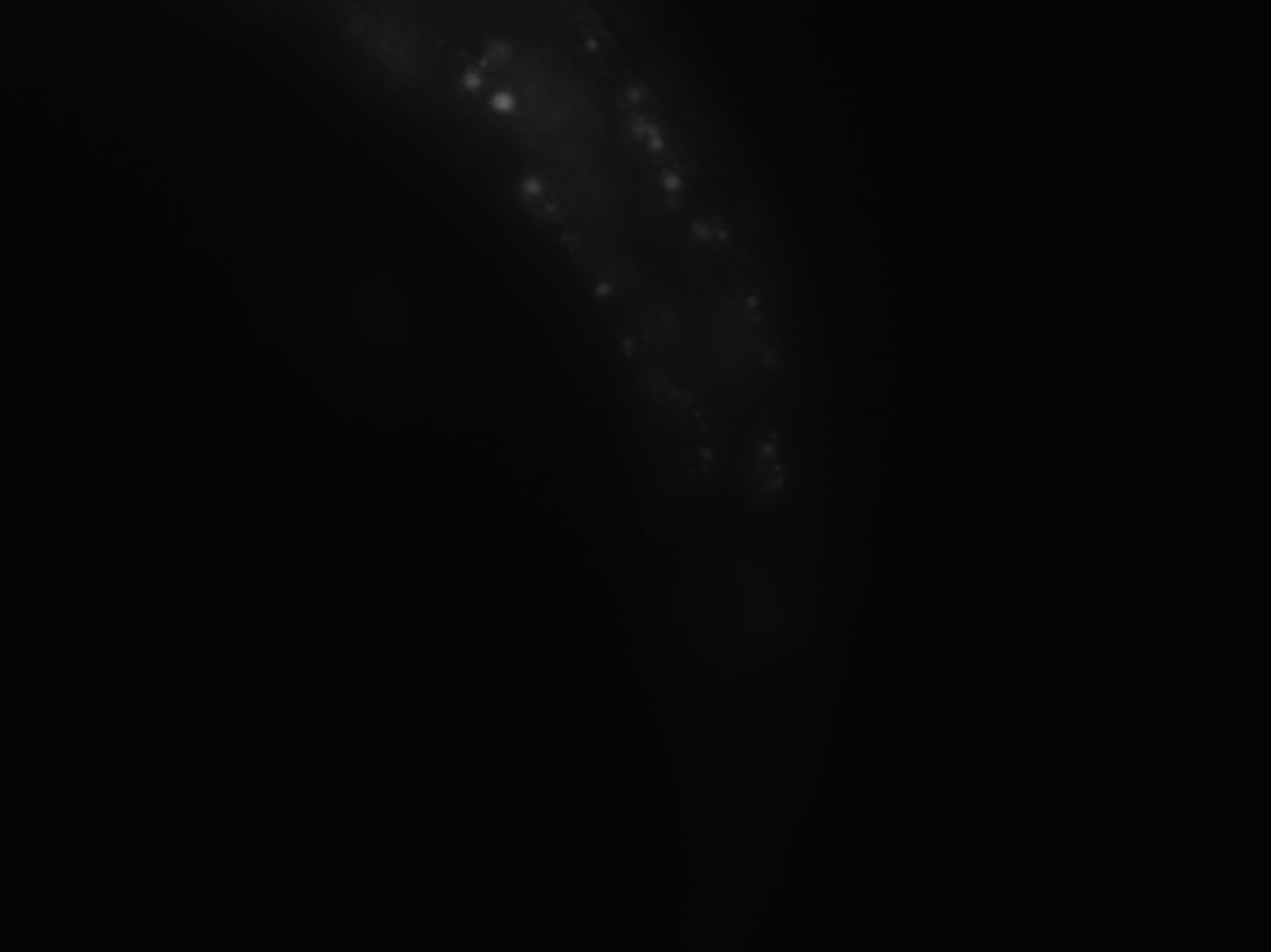

Supplement: Supplementary file 2 — Source data Fig. 1 [file 44319_2025_493_MOESM2_ESM.zip › Figure1/Fig1F/Experiment-11_tail_wildtype.tif_files/Experiment-11_z14c0x0-1388y0-1040.tif]

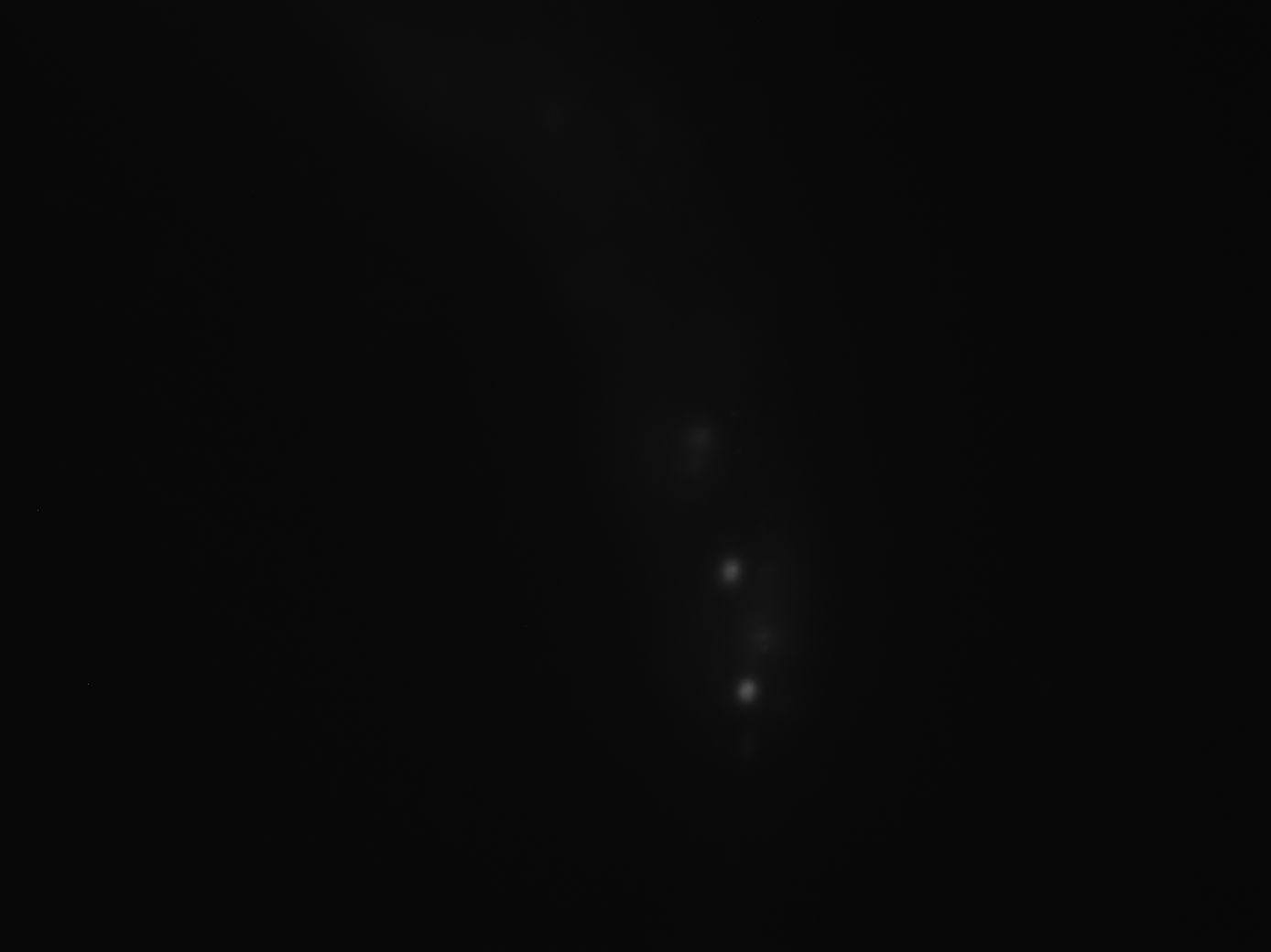

Supplement: Supplementary file 2 — Source data Fig. 1 [file 44319_2025_493_MOESM2_ESM.zip › Figure1/Fig1F/Experiment-11_tail_wildtype.tif_files/Experiment-11_z2c1x0-1388y0-1040.tif]

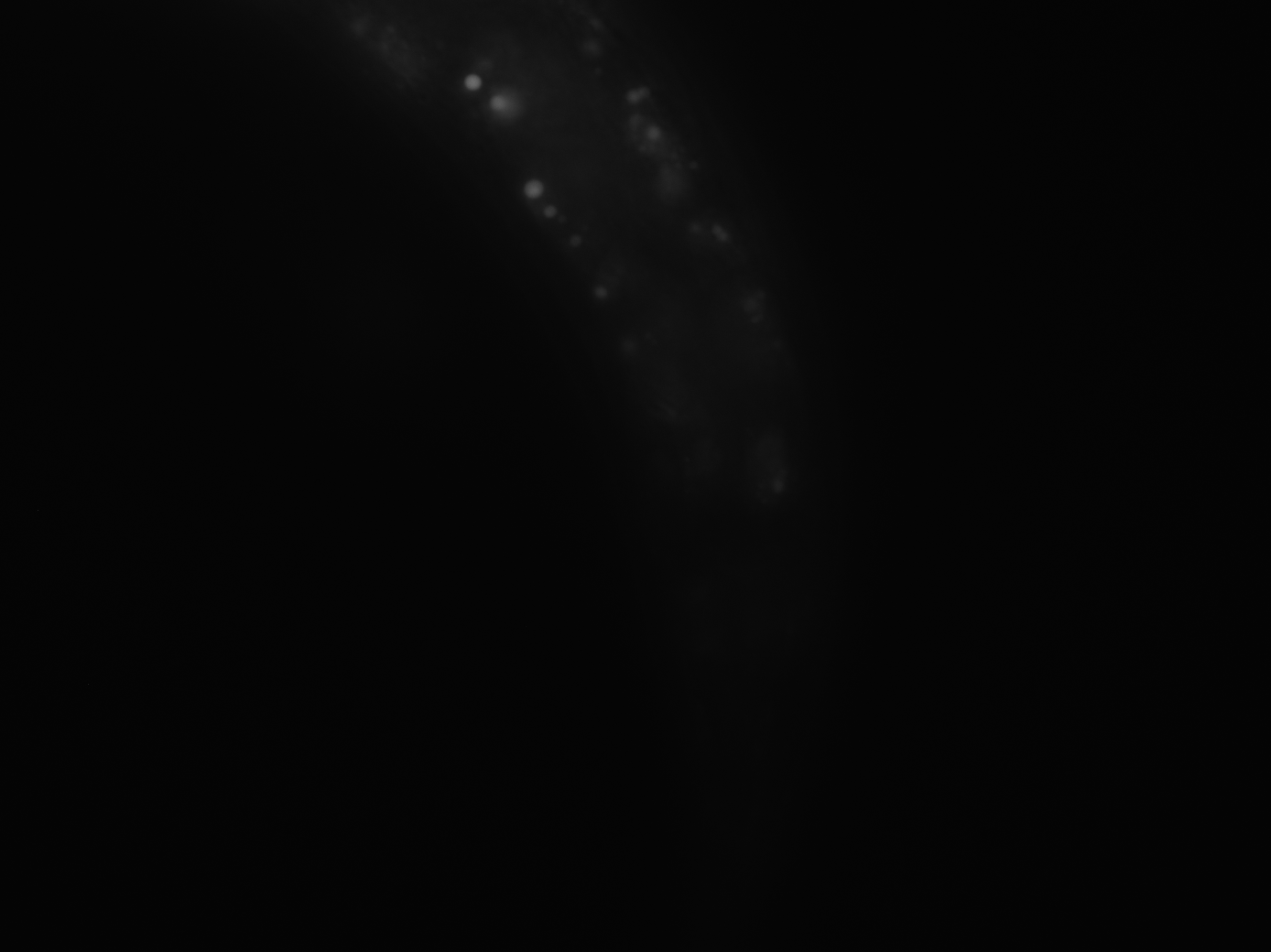

Supplement: Supplementary file 2 — Source data Fig. 1 [file 44319_2025_493_MOESM2_ESM.zip › Figure1/Fig1F/Experiment-11_tail_wildtype.tif_files/Experiment-11_z16c0x0-1388y0-1040.tif]

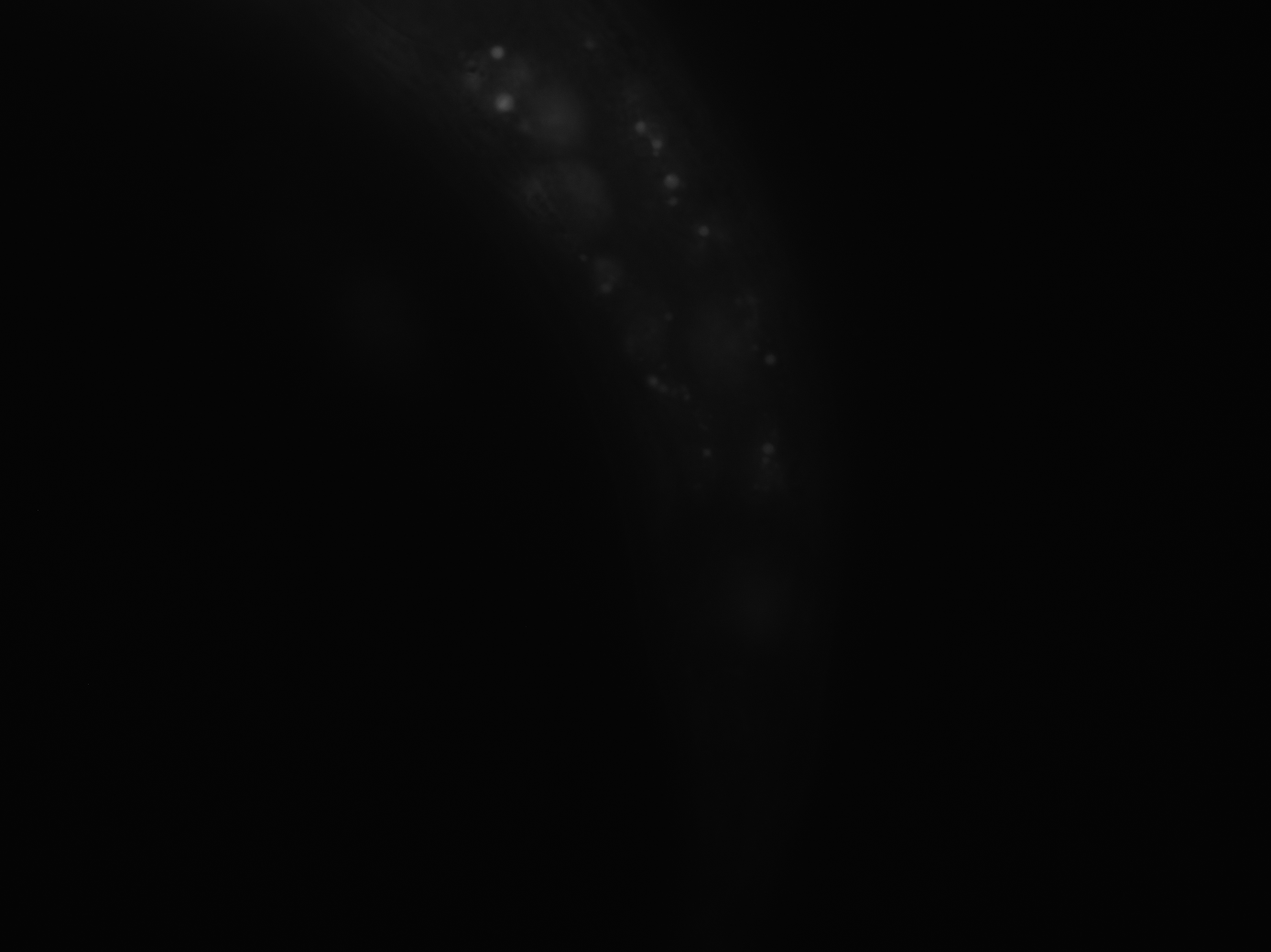

Supplement: Supplementary file 2 — Source data Fig. 1 [file 44319_2025_493_MOESM2_ESM.zip › Figure1/Fig1F/Experiment-11_tail_wildtype.tif_files/Experiment-11_z12c0x0-1388y0-1040.tif]

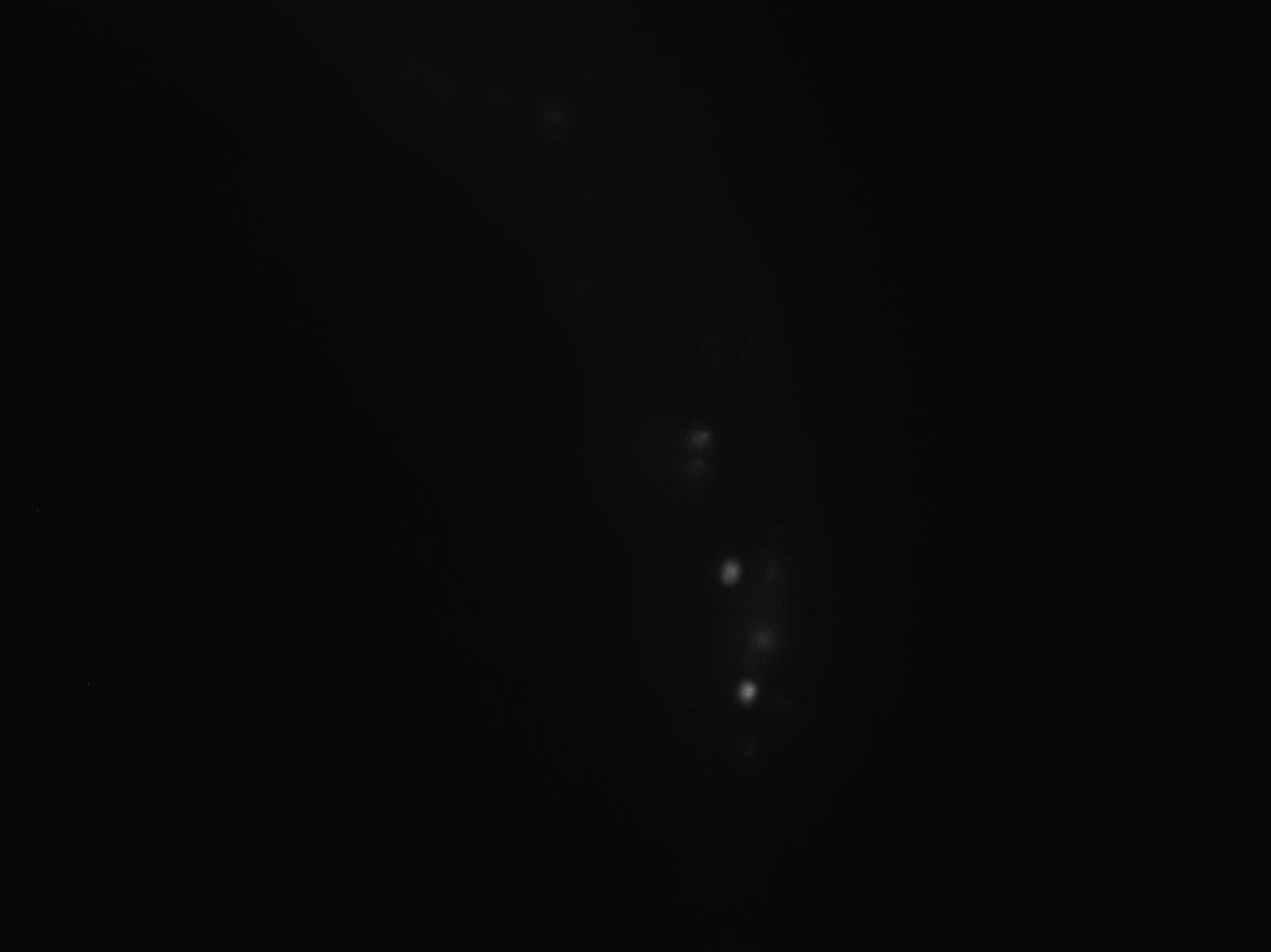

Supplement: Supplementary file 2 — Source data Fig. 1 [file 44319_2025_493_MOESM2_ESM.zip › Figure1/Fig1F/Experiment-11_tail_wildtype.tif_files/Experiment-11_z4c1x0-1388y0-1040.tif]

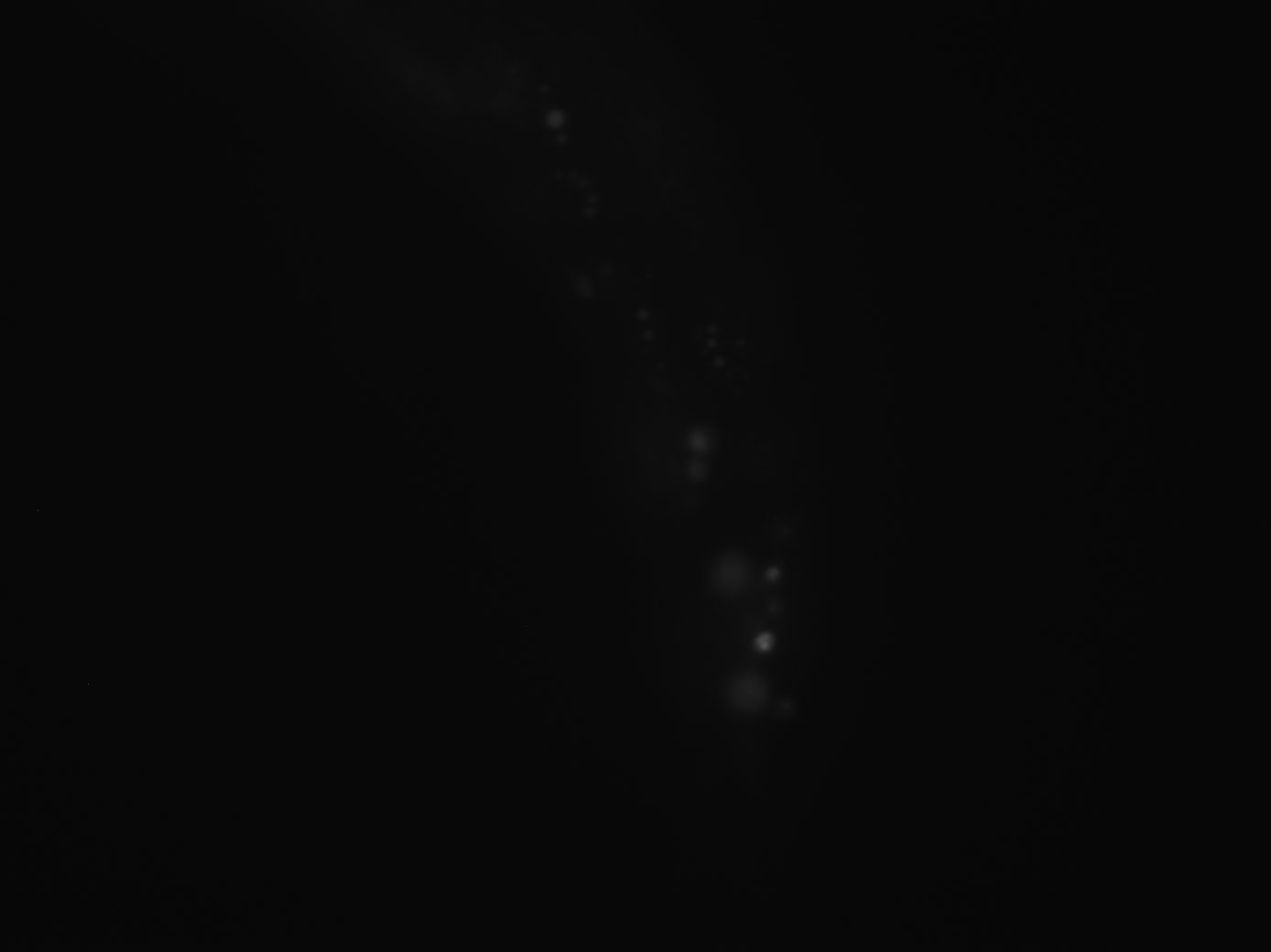

Supplement: Supplementary file 2 — Source data Fig. 1 [file 44319_2025_493_MOESM2_ESM.zip › Figure1/Fig1F/Experiment-11_tail_wildtype.tif_files/Experiment-11_z7c1x0-1388y0-1040.tif]

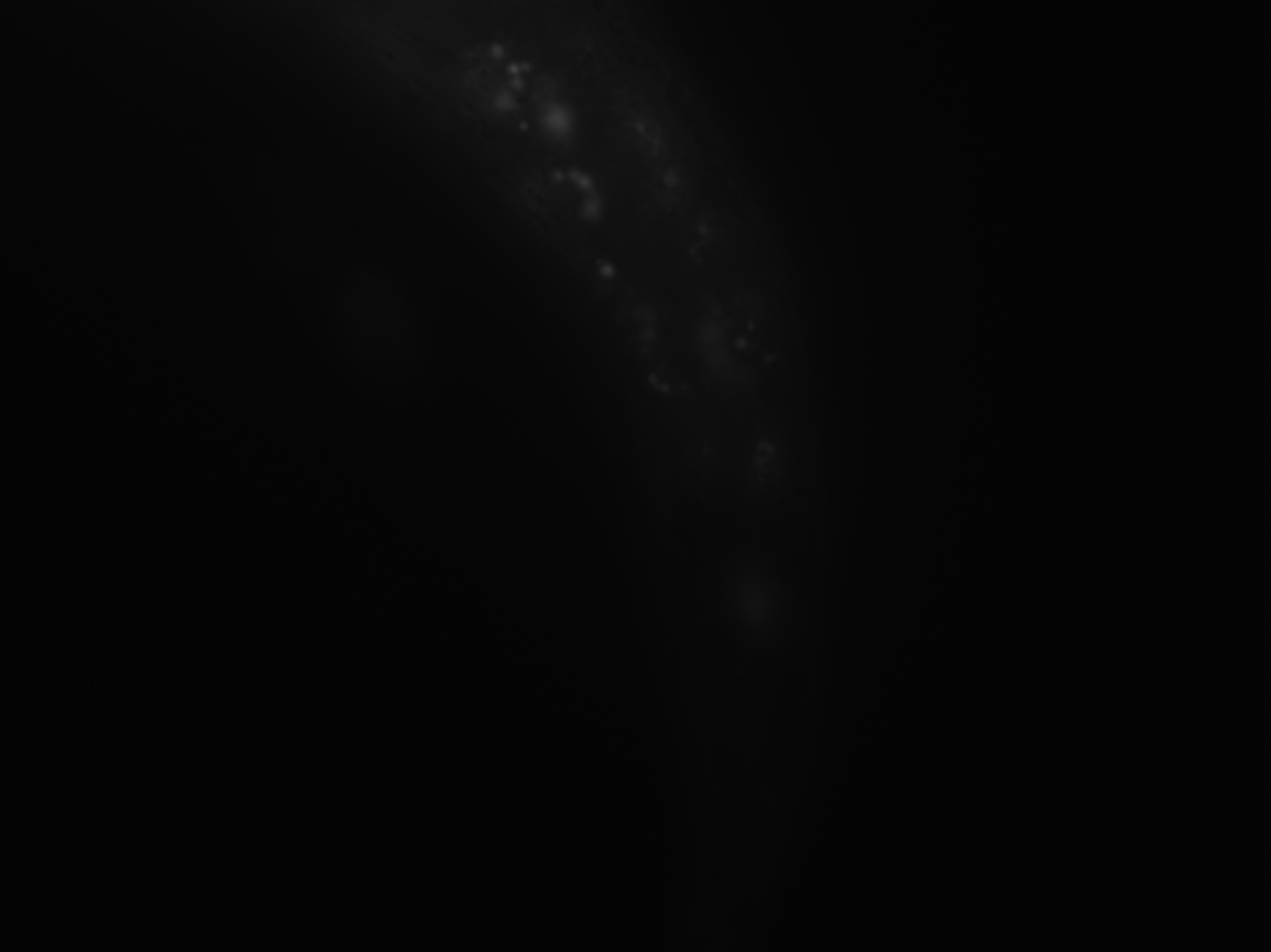

Supplement: Supplementary file 2 — Source data Fig. 1 [file 44319_2025_493_MOESM2_ESM.zip › Figure1/Fig1F/Experiment-11_tail_wildtype.tif_files/Experiment-11_z10c0x0-1388y0-1040.tif]

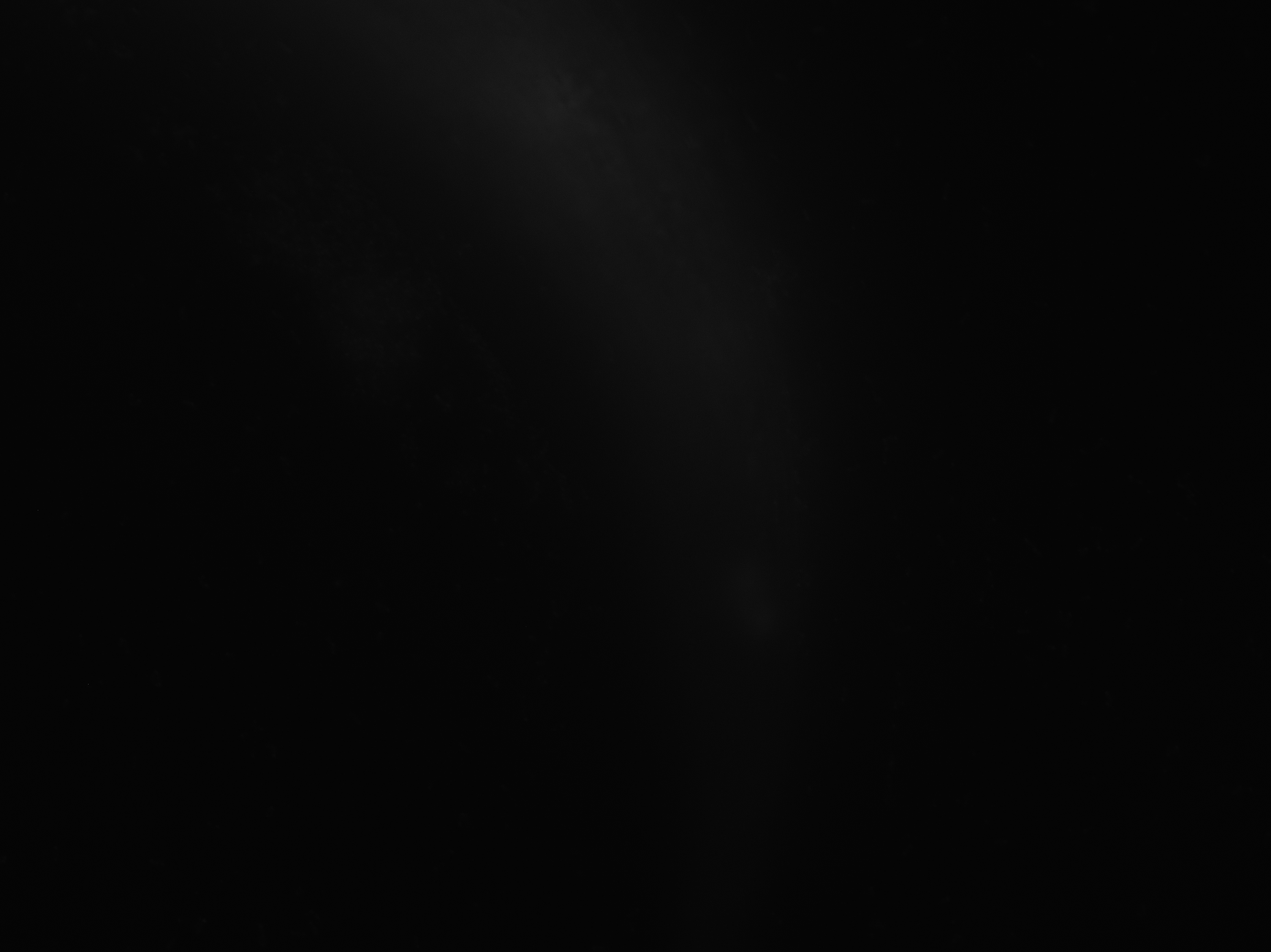

Supplement: Supplementary file 2 — Source data Fig. 1 [file 44319_2025_493_MOESM2_ESM.zip › Figure1/Fig1F/Experiment-11_tail_wildtype.tif_files/Experiment-11_z0c0x0-1388y0-1040.tif]

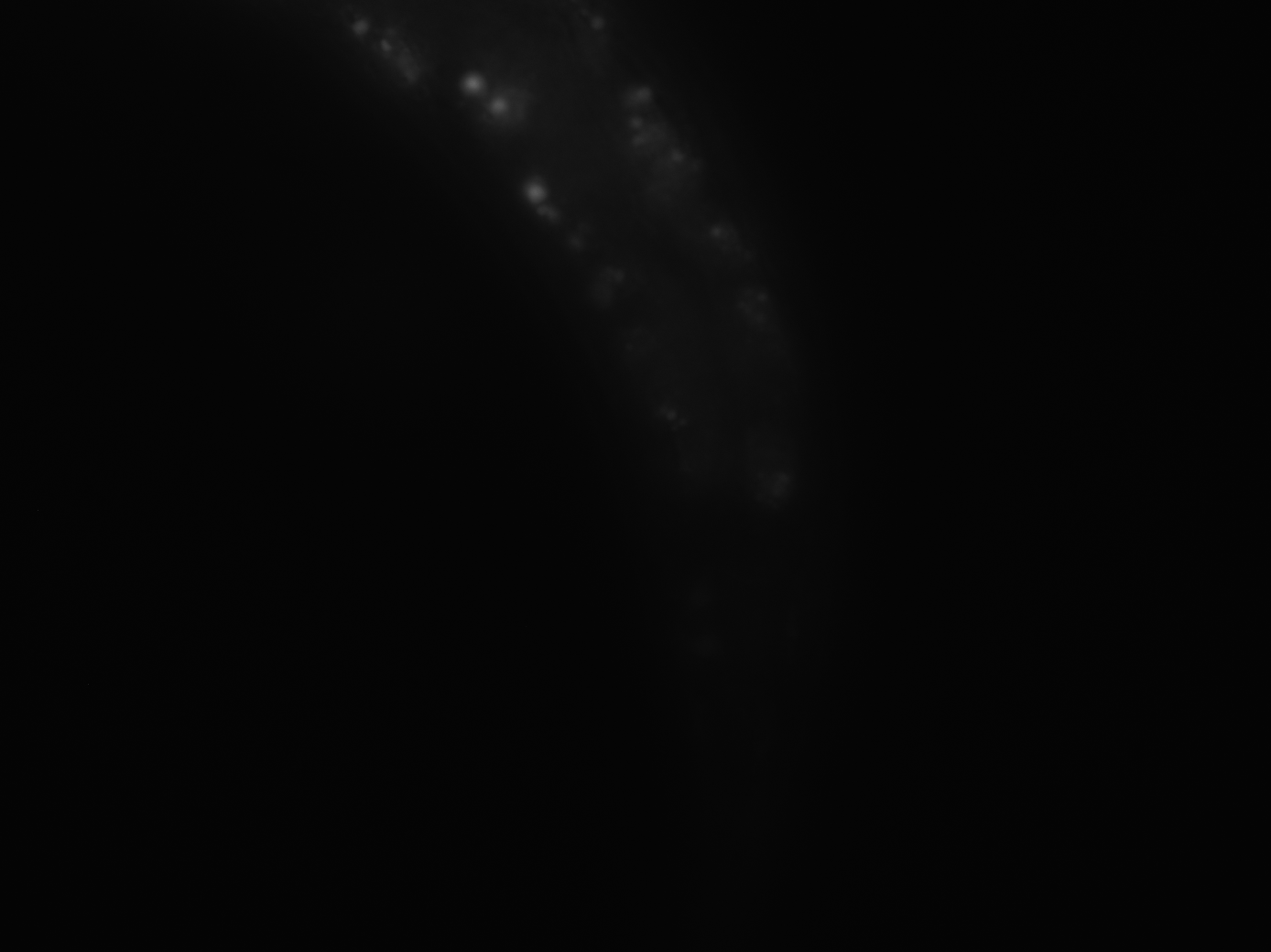

Supplement: Supplementary file 2 — Source data Fig. 1 [file 44319_2025_493_MOESM2_ESM.zip › Figure1/Fig1F/Experiment-11_tail_wildtype.tif_files/Experiment-11_z18c0x0-1388y0-1040.tif]

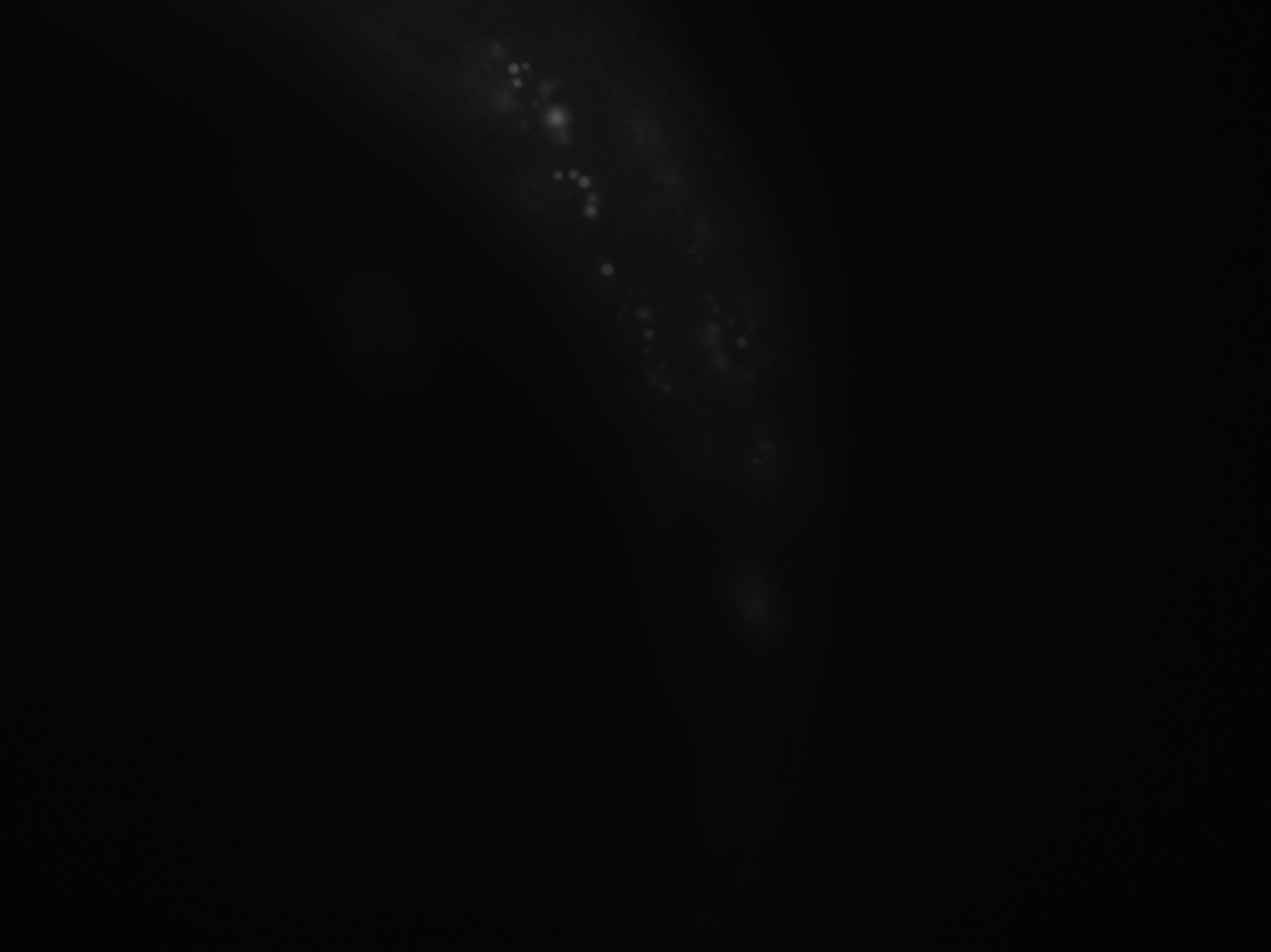

Supplement: Supplementary file 2 — Source data Fig. 1 [file 44319_2025_493_MOESM2_ESM.zip › Figure1/Fig1F/Experiment-11_tail_wildtype.tif_files/Experiment-11_z9c0x0-1388y0-1040.tif]

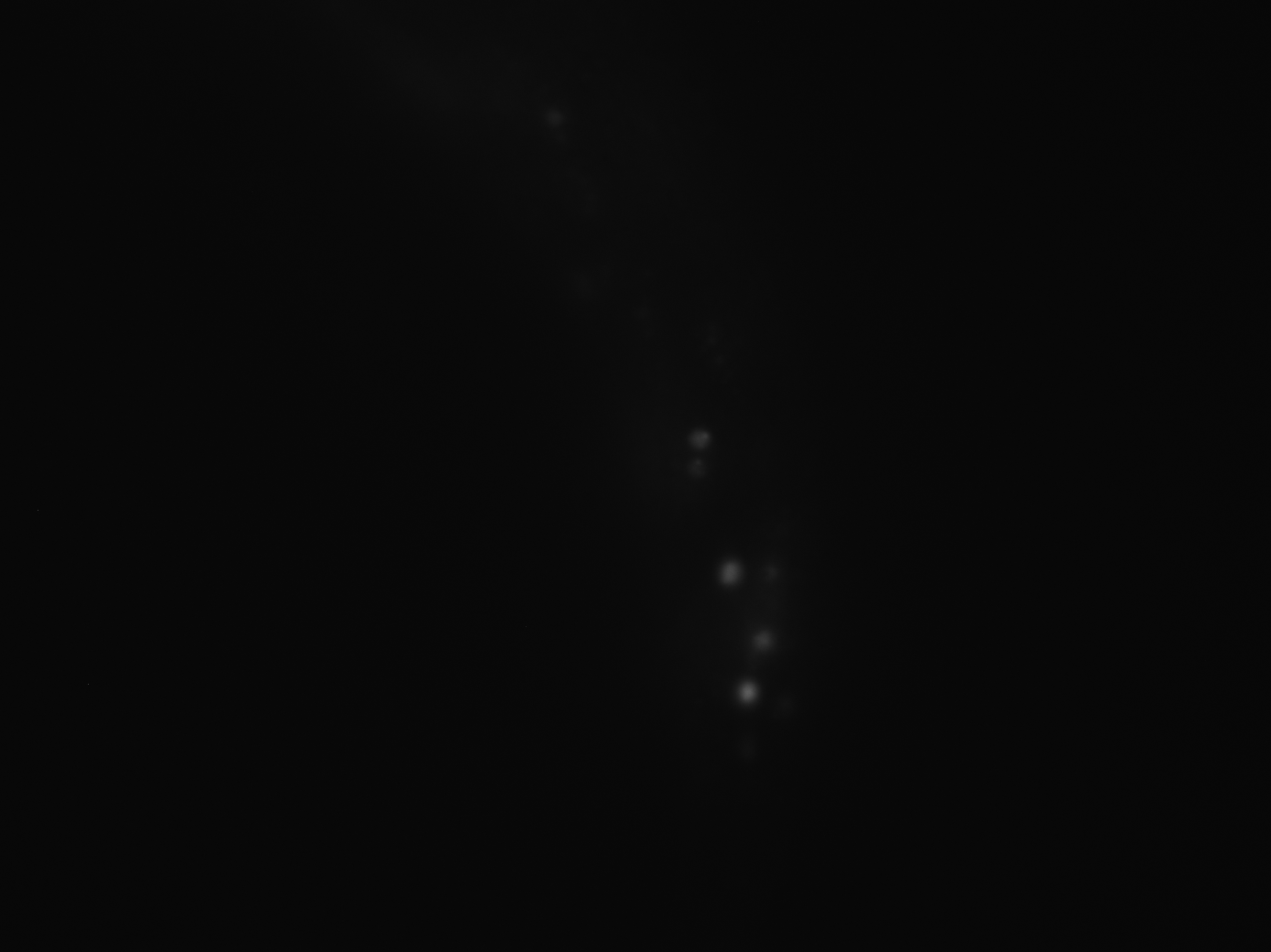

Supplement: Supplementary file 2 — Source data Fig. 1 [file 44319_2025_493_MOESM2_ESM.zip › Figure1/Fig1F/Experiment-11_tail_wildtype.tif_files/Experiment-11_z5c1x0-1388y0-1040.tif]

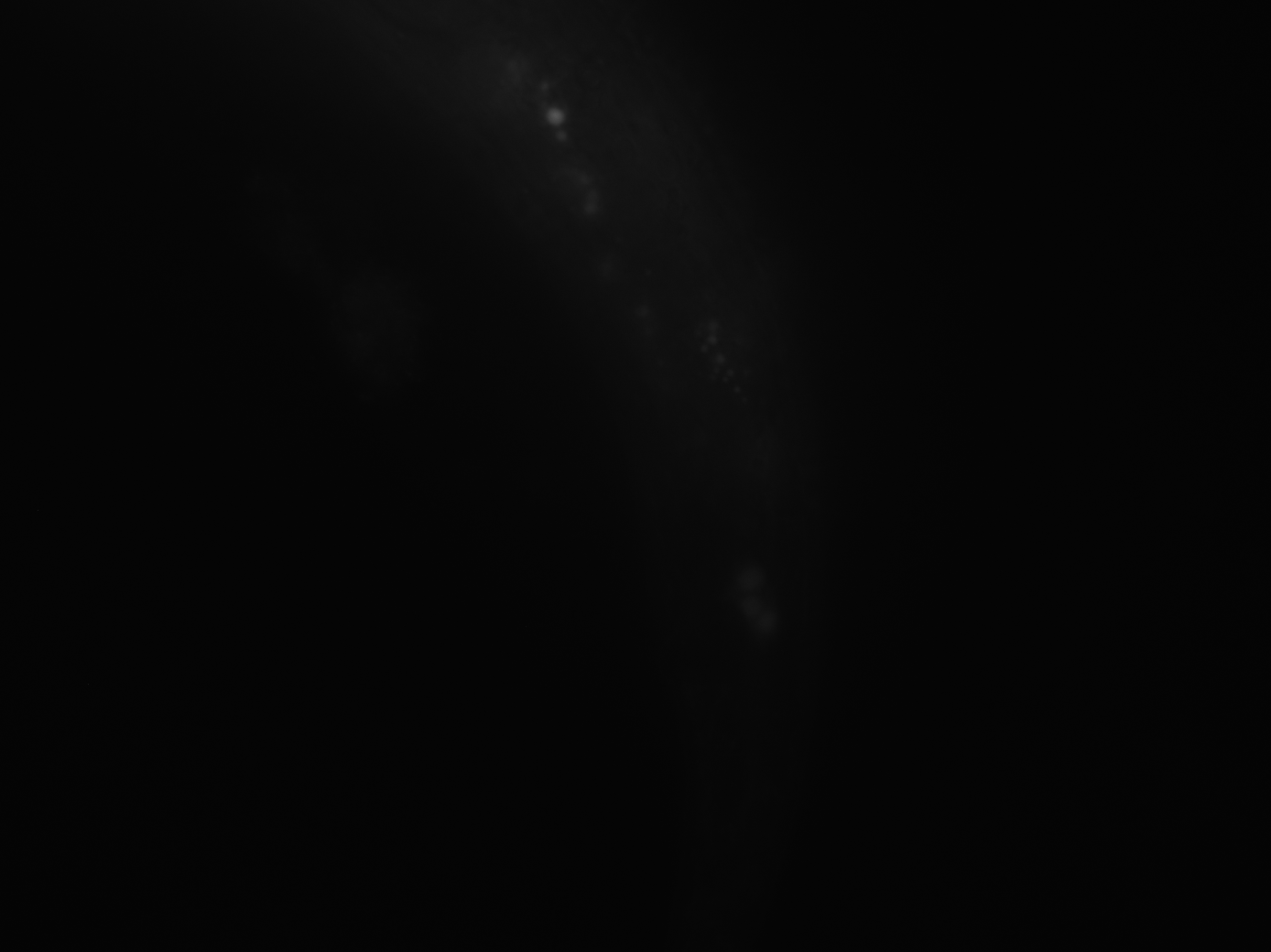

Supplement: Supplementary file 2 — Source data Fig. 1 [file 44319_2025_493_MOESM2_ESM.zip › Figure1/Fig1F/Experiment-11_tail_wildtype.tif_files/Experiment-11_z6c0x0-1388y0-1040.tif]

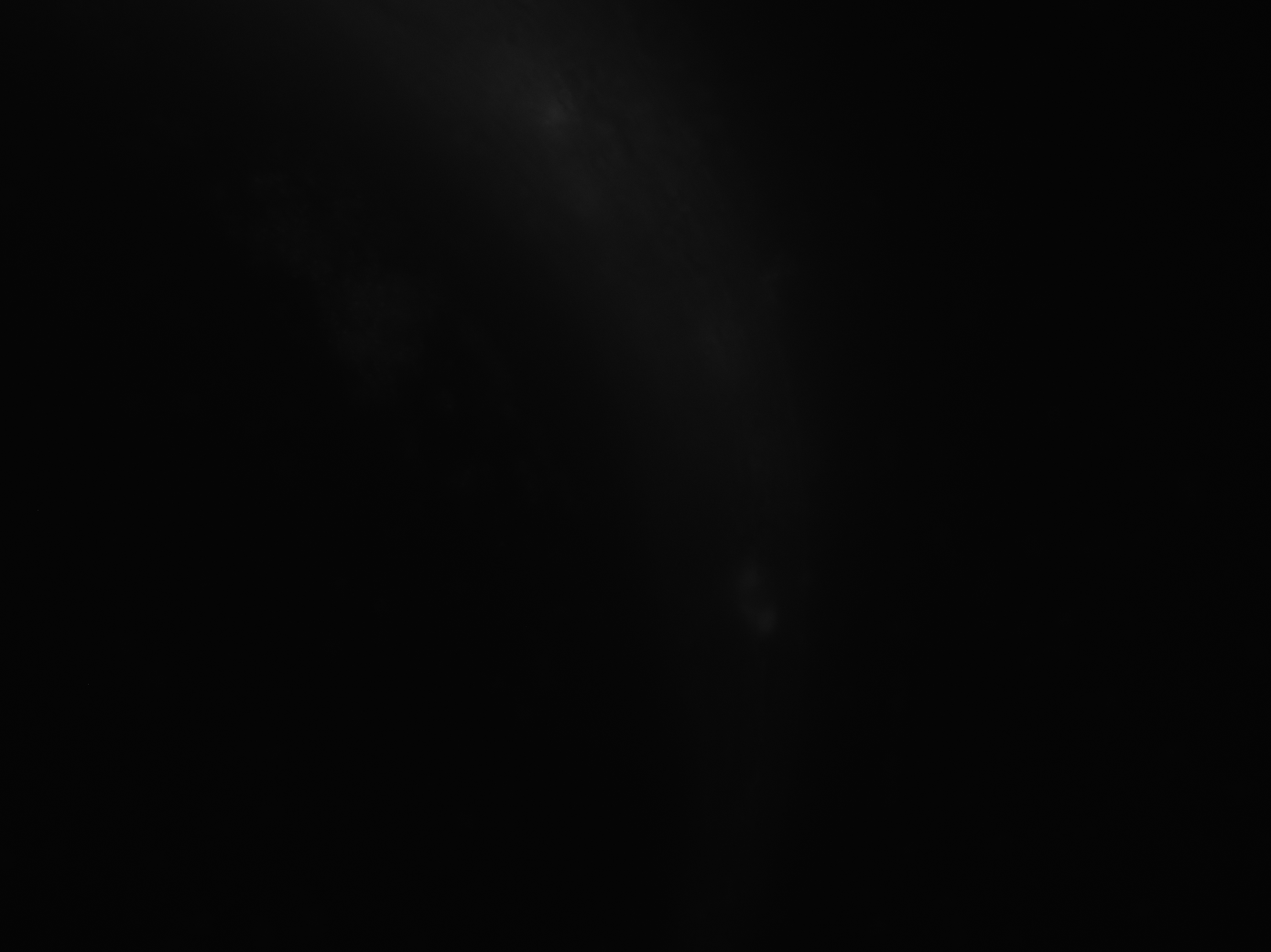

Supplement: Supplementary file 2 — Source data Fig. 1 [file 44319_2025_493_MOESM2_ESM.zip › Figure1/Fig1F/Experiment-11_tail_wildtype.tif_files/Experiment-11_z3c0x0-1388y0-1040.tif]

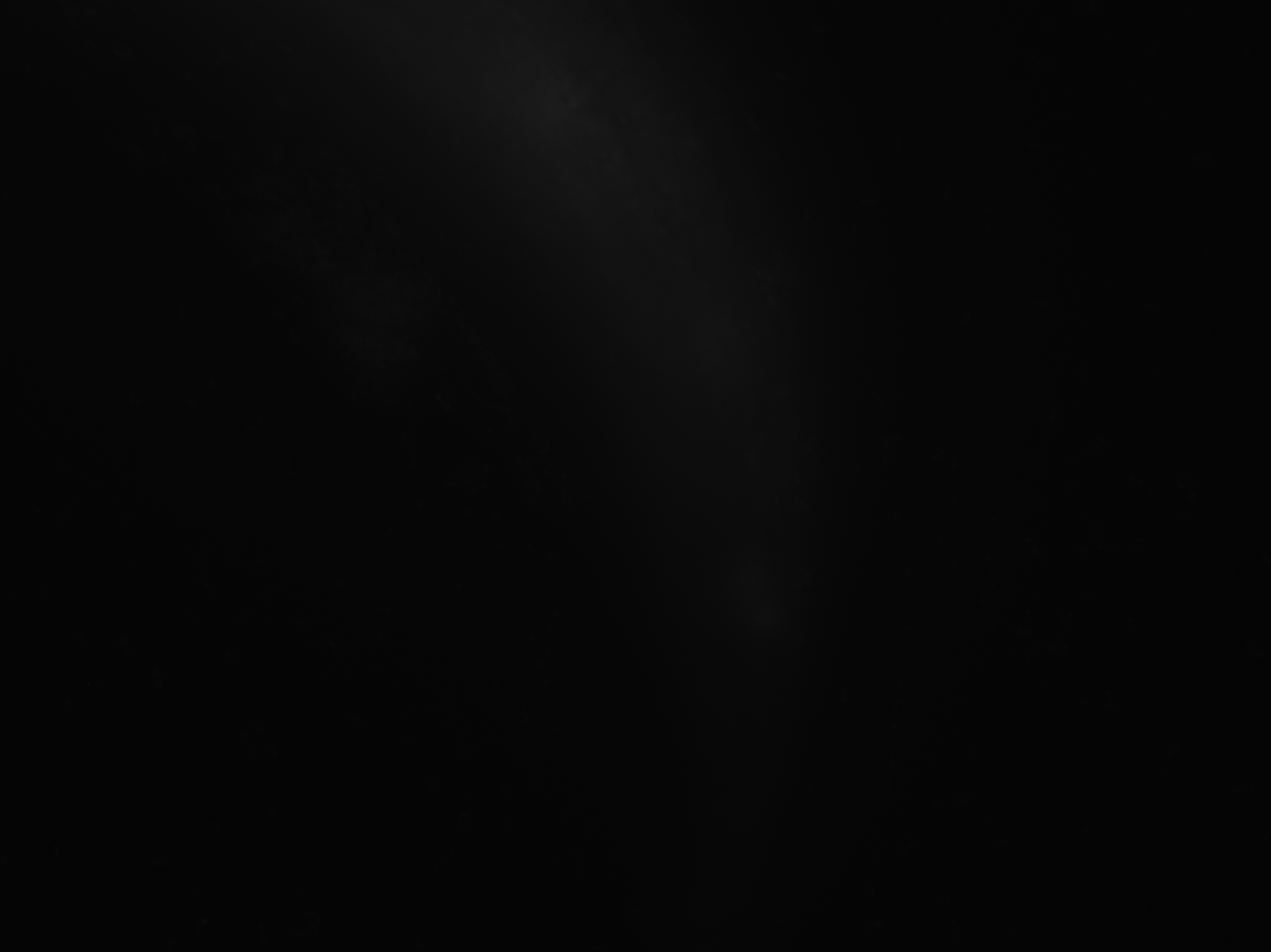

Supplement: Supplementary file 2 — Source data Fig. 1 [file 44319_2025_493_MOESM2_ESM.zip › Figure1/Fig1F/Experiment-11_tail_wildtype.tif_files/Experiment-11_z1c0x0-1388y0-1040.tif]

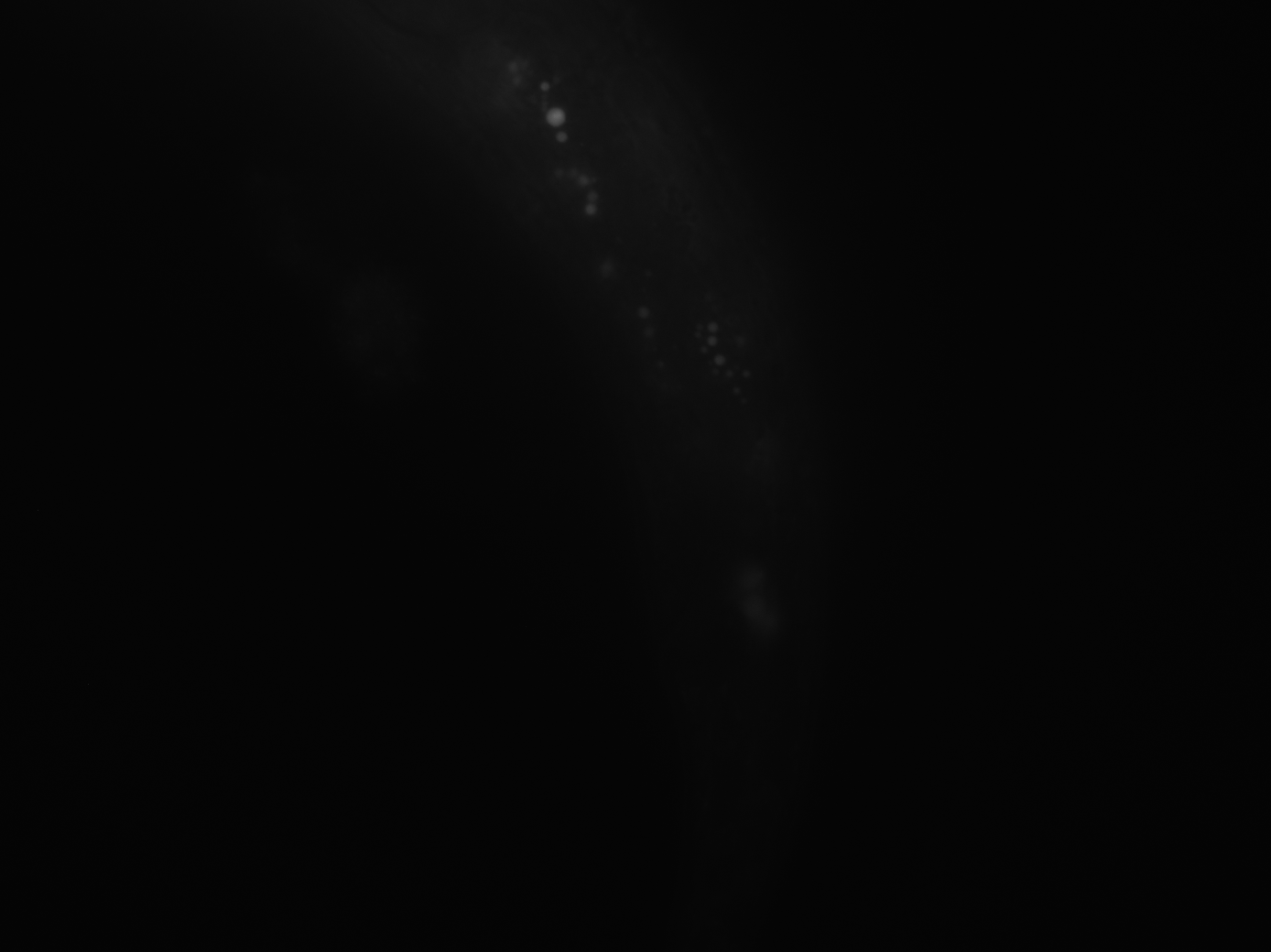

Supplement: Supplementary file 2 — Source data Fig. 1 [file 44319_2025_493_MOESM2_ESM.zip › Figure1/Fig1F/Experiment-11_tail_wildtype.tif_files/Experiment-11_z7c0x0-1388y0-1040.tif]

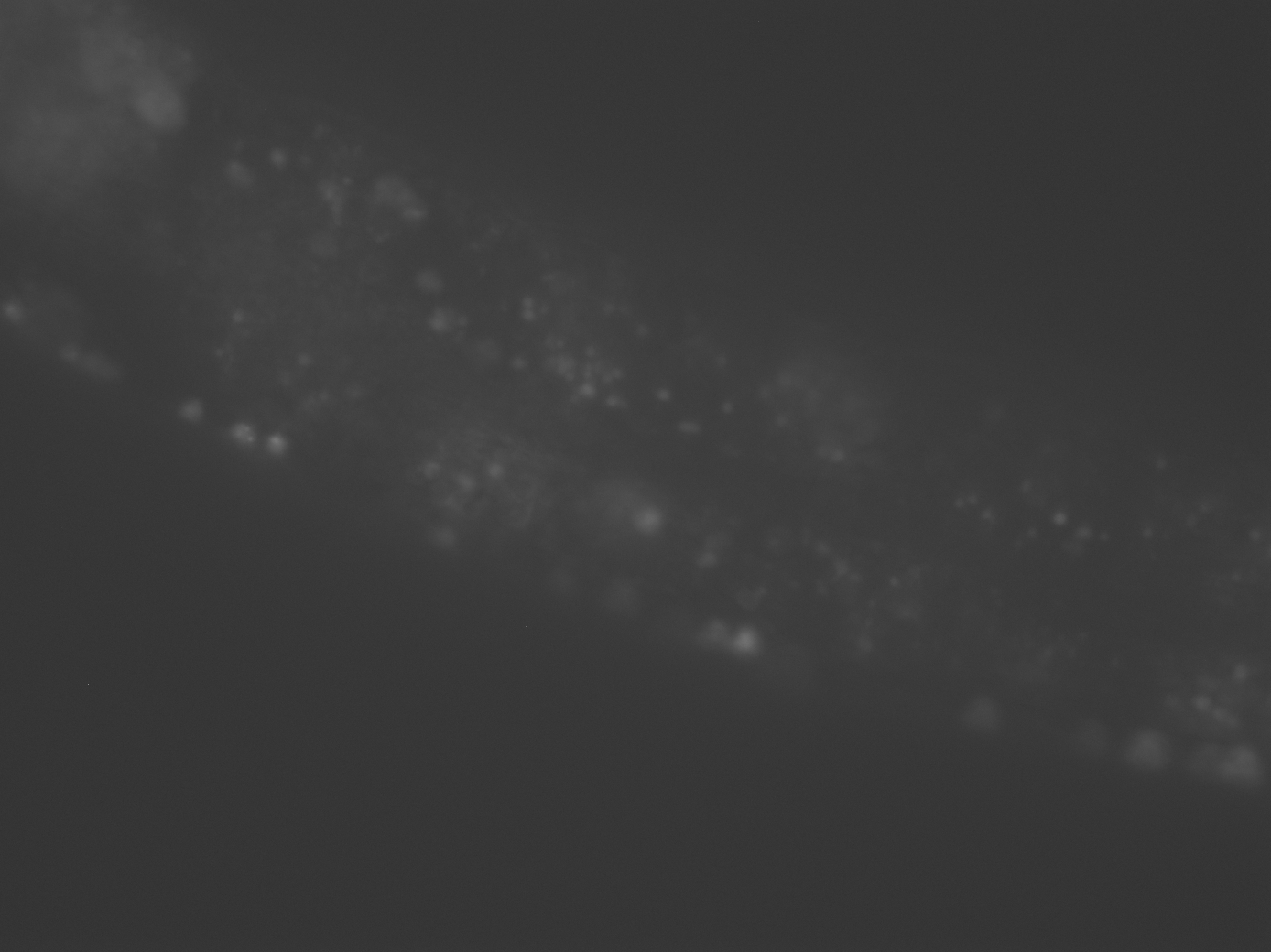

Supplement: Supplementary file 2 — Source data Fig. 1 [file 44319_2025_493_MOESM2_ESM.zip › Figure1/Fig1F/Experiment-04_VC_wiltype.tif_files/Experiment-04_z11c1x0-1388y0-1040.tif]

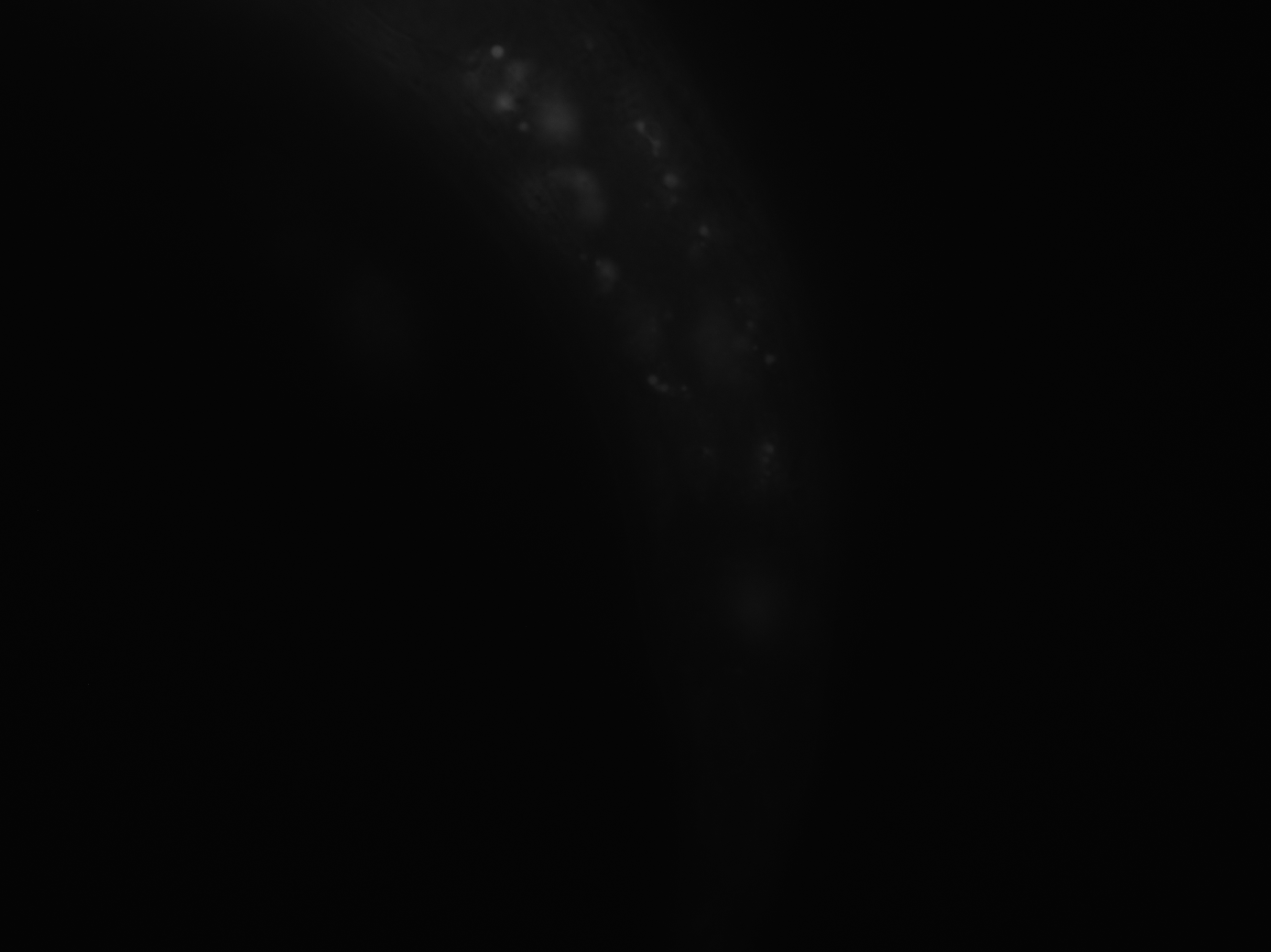

Supplement: Supplementary file 2 — Source data Fig. 1 [file 44319_2025_493_MOESM2_ESM.zip › Figure1/Fig1F/Experiment-11_tail_wildtype.tif_files/Experiment-11_z11c0x0-1388y0-1040.tif]

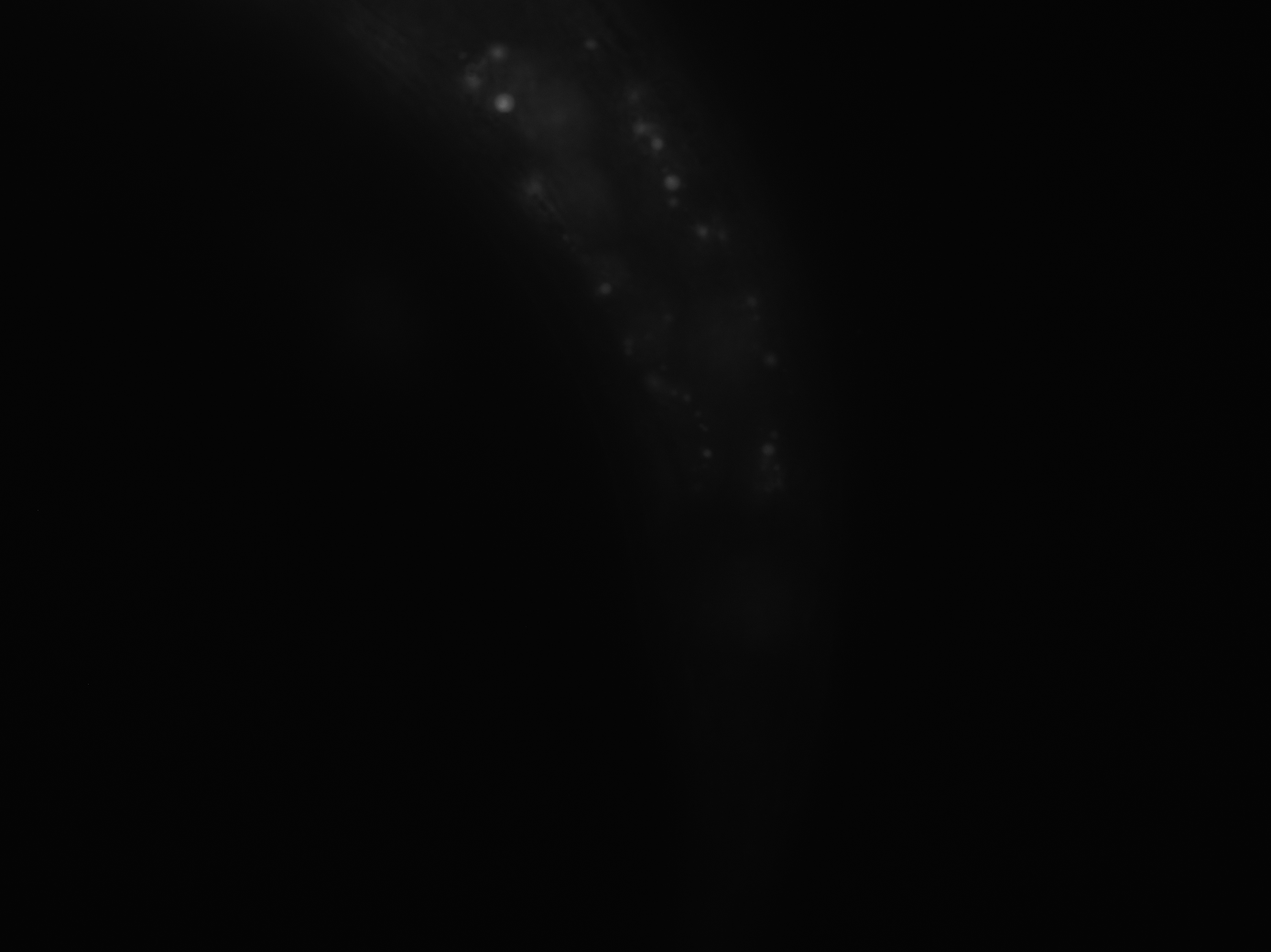

Supplement: Supplementary file 2 — Source data Fig. 1 [file 44319_2025_493_MOESM2_ESM.zip › Figure1/Fig1F/Experiment-11_tail_wildtype.tif_files/Experiment-11_z13c0x0-1388y0-1040.tif]

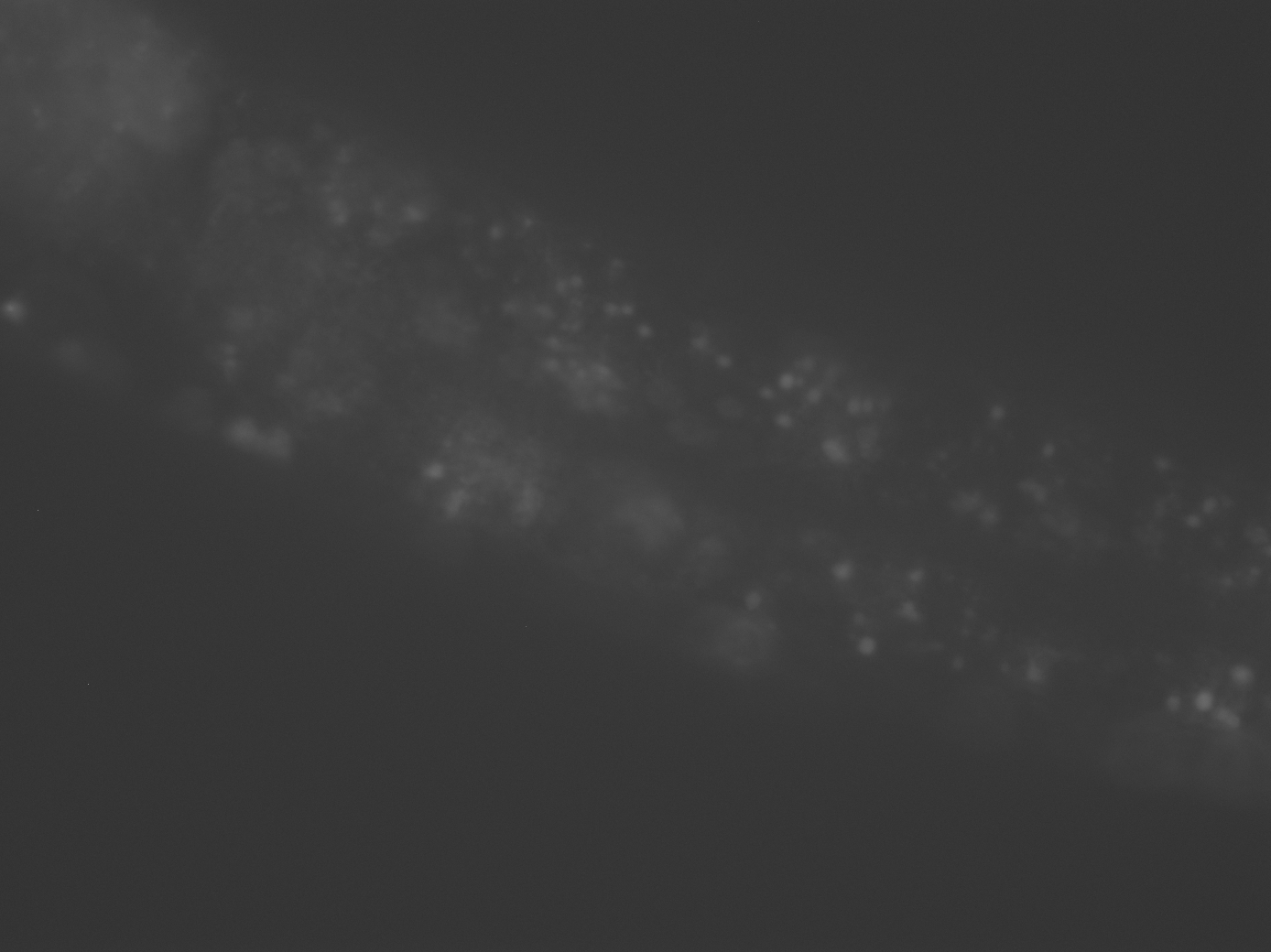

Supplement: Supplementary file 2 — Source data Fig. 1 [file 44319_2025_493_MOESM2_ESM.zip › Figure1/Fig1F/Experiment-04_VC_wiltype.tif_files/Experiment-04_z15c1x0-1388y0-1040.tif]

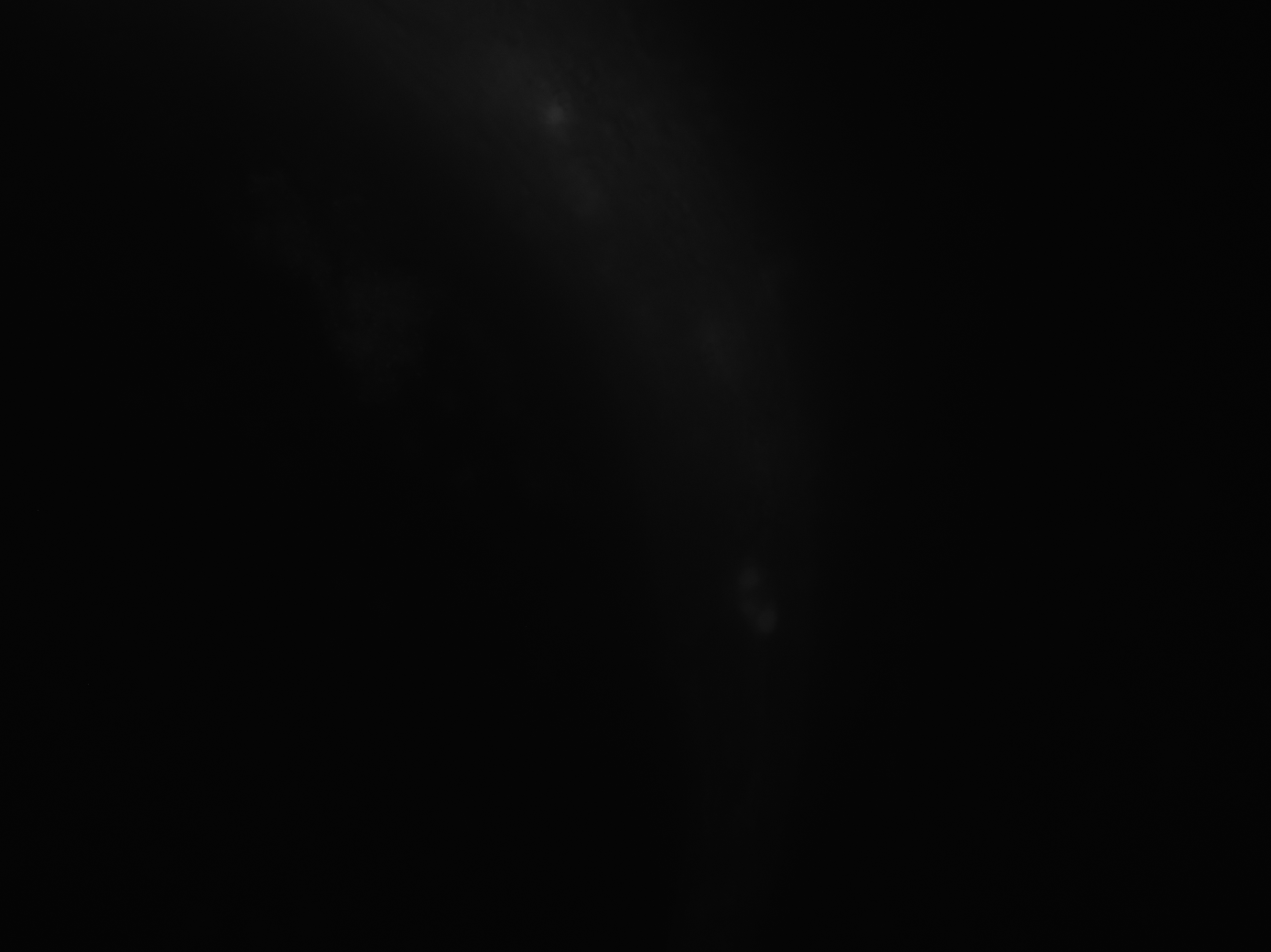

Supplement: Supplementary file 2 — Source data Fig. 1 [file 44319_2025_493_MOESM2_ESM.zip › Figure1/Fig1F/Experiment-11_tail_wildtype.tif_files/Experiment-11_z4c0x0-1388y0-1040.tif]

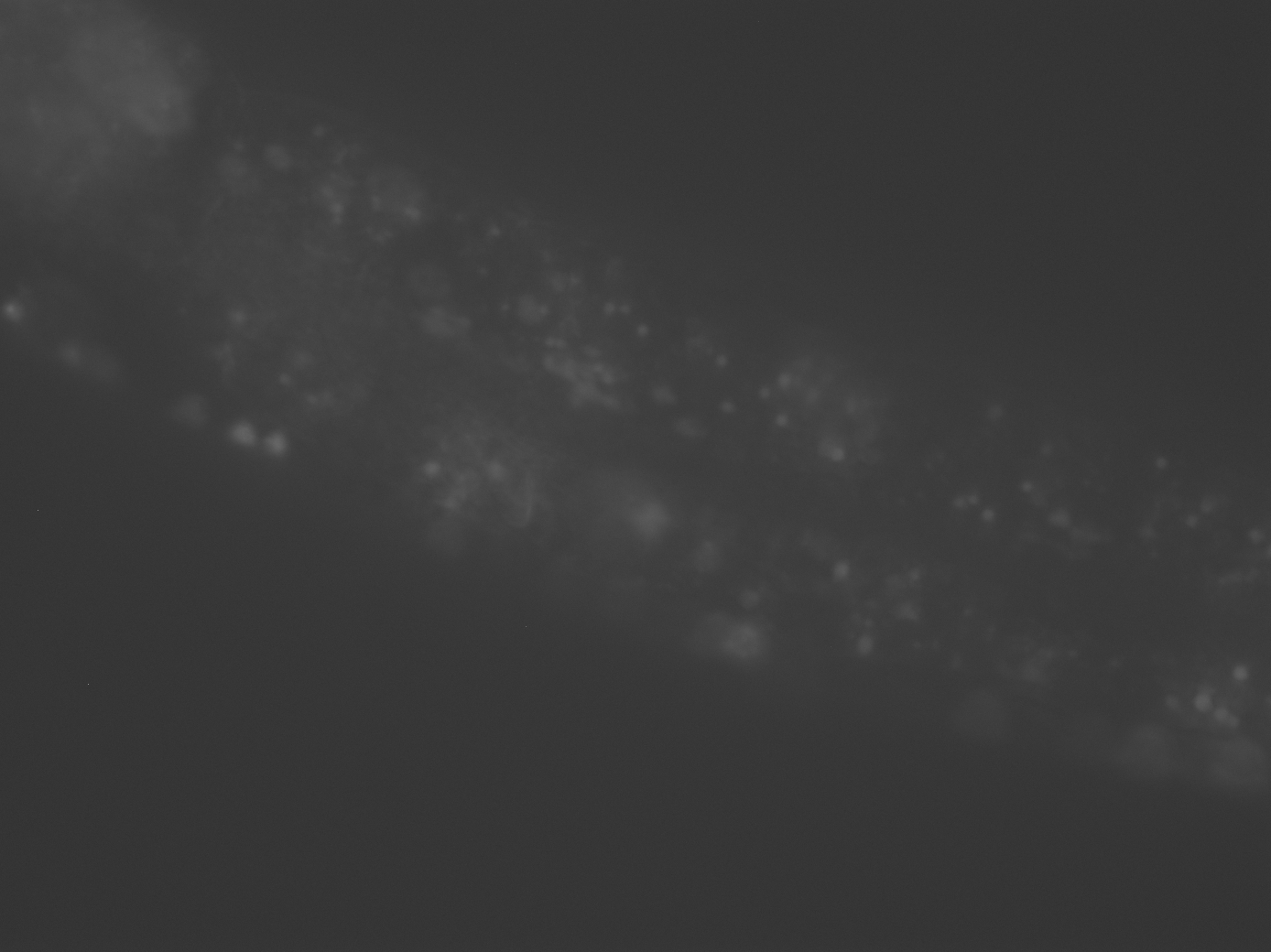

Supplement: Supplementary file 2 — Source data Fig. 1 [file 44319_2025_493_MOESM2_ESM.zip › Figure1/Fig1F/Experiment-04_VC_wiltype.tif_files/Experiment-04_z13c1x0-1388y0-1040.tif]

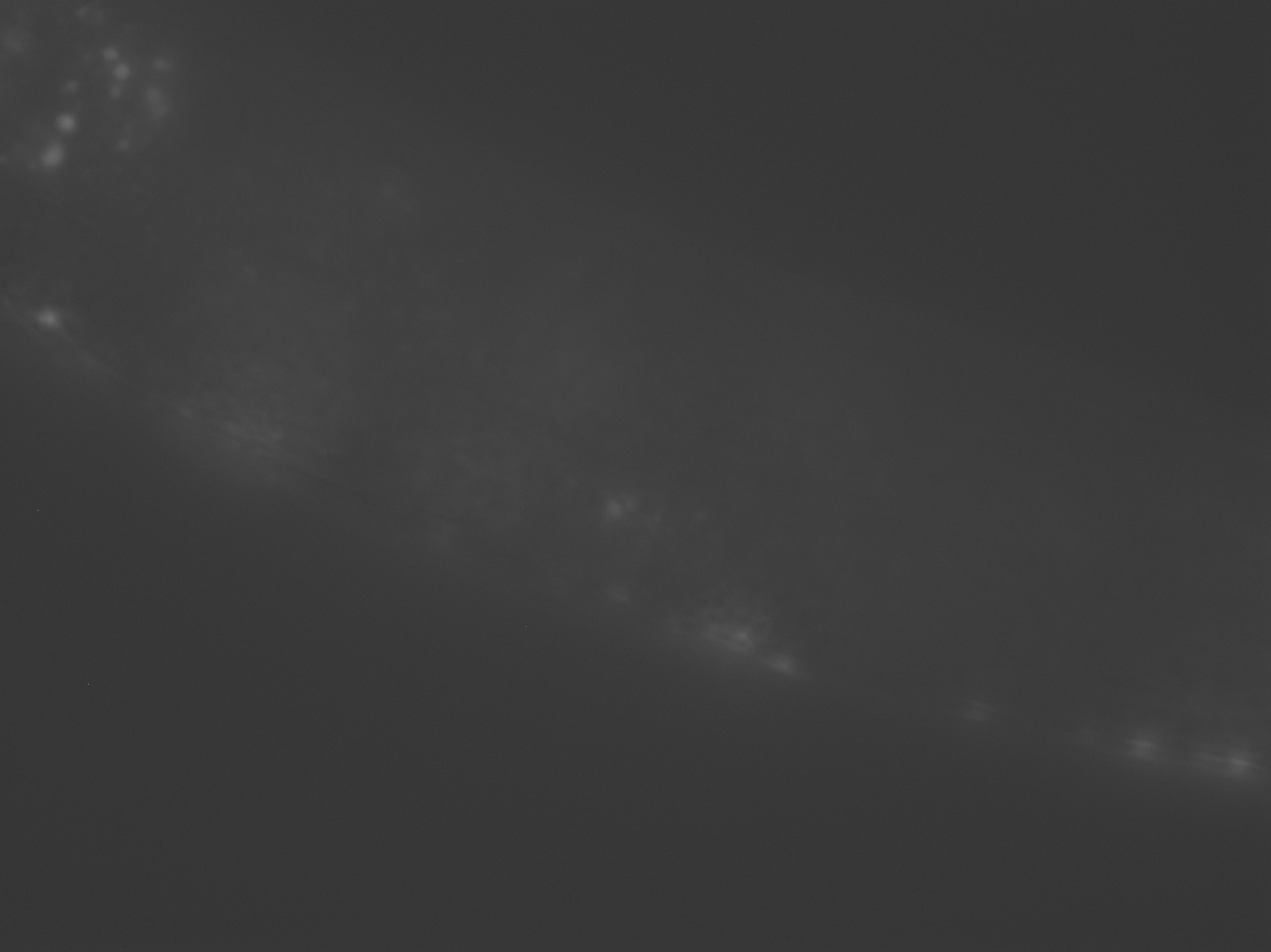

Supplement: Supplementary file 2 — Source data Fig. 1 [file 44319_2025_493_MOESM2_ESM.zip › Figure1/Fig1F/Experiment-04_VC_wiltype.tif_files/Experiment-04_z2c1x0-1388y0-1040.tif]

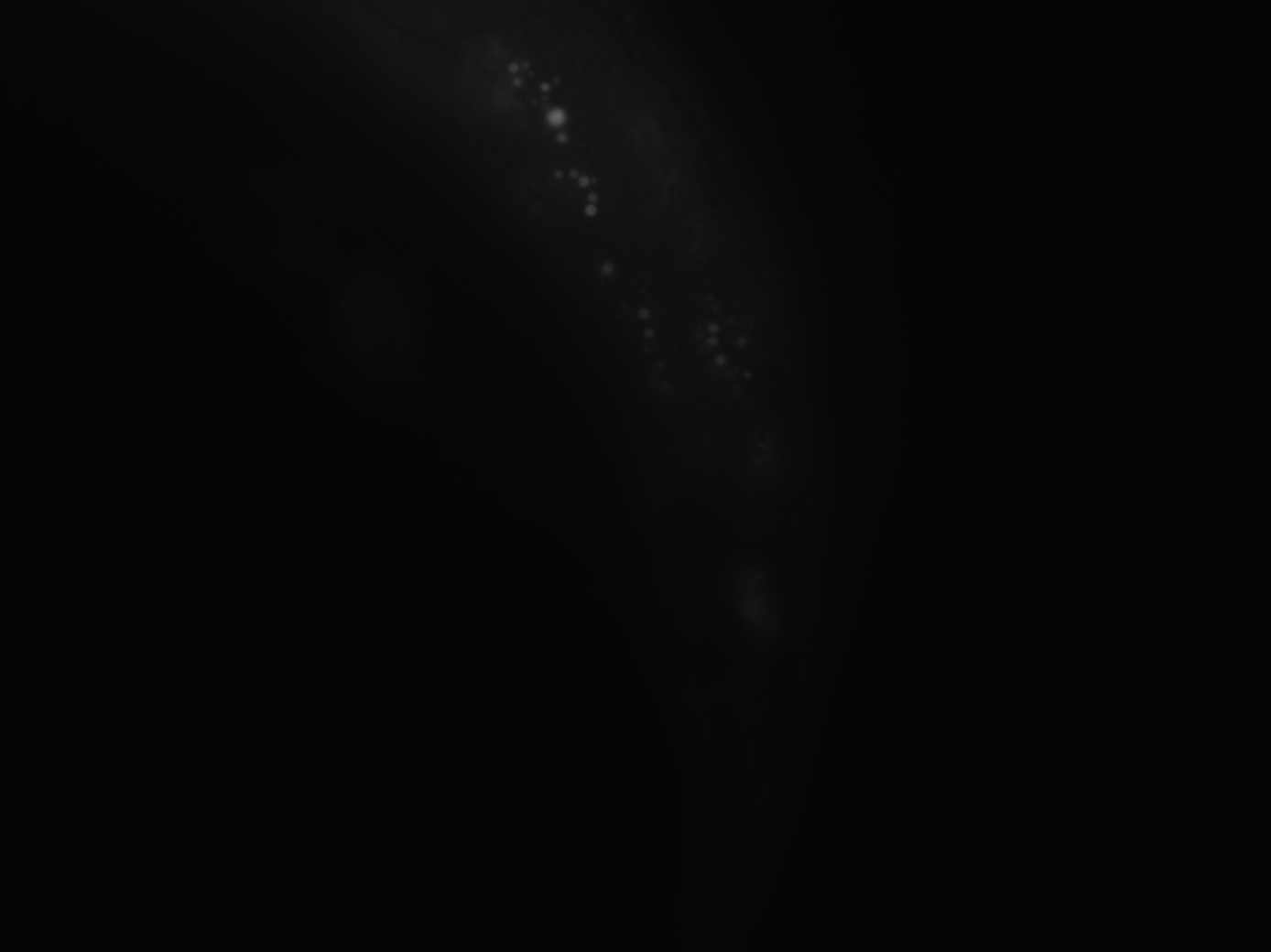

Supplement: Supplementary file 2 — Source data Fig. 1 [file 44319_2025_493_MOESM2_ESM.zip › Figure1/Fig1F/Experiment-11_tail_wildtype.tif_files/Experiment-11_z8c0x0-1388y0-1040.tif]

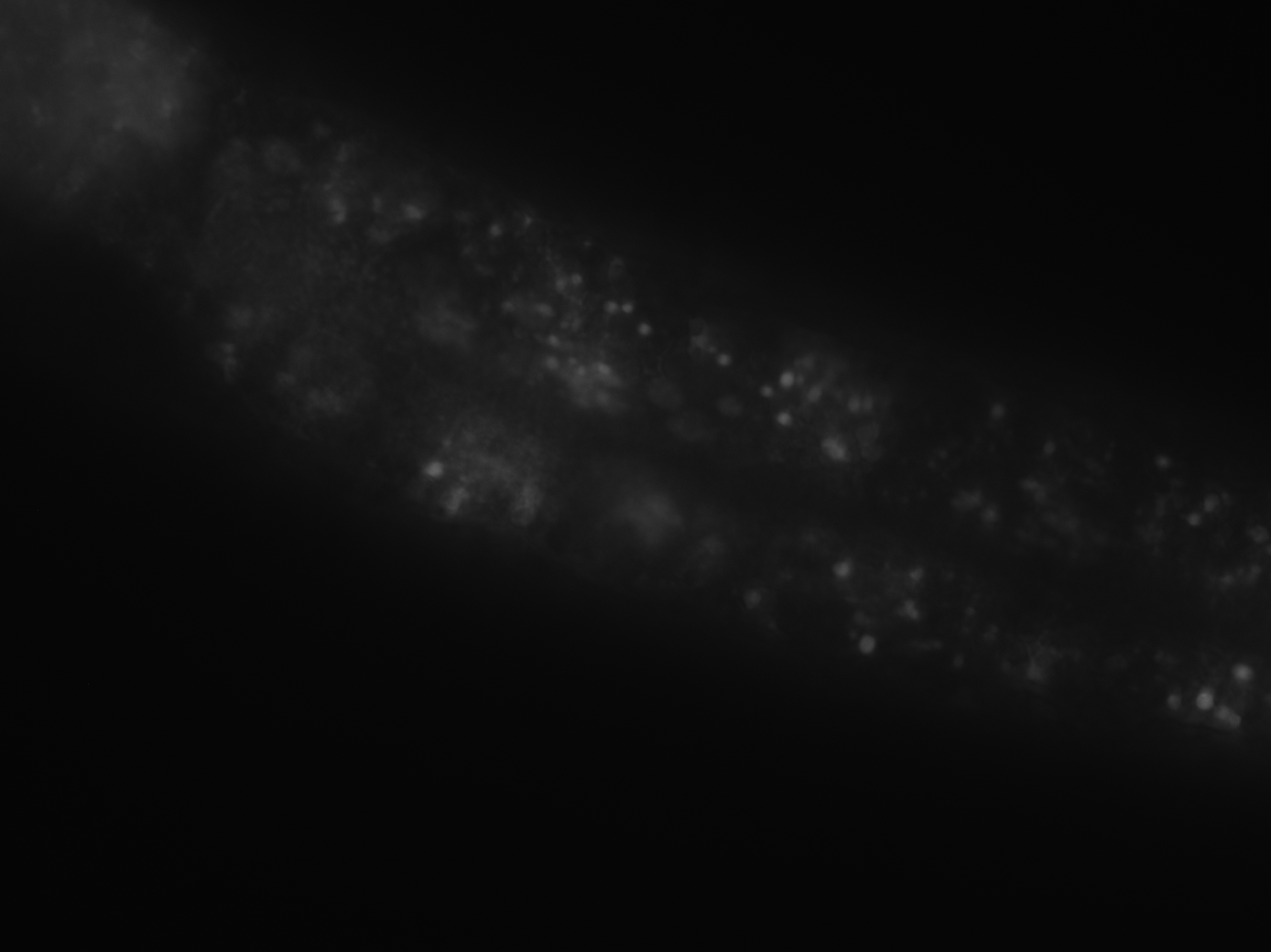

Supplement: Supplementary file 2 — Source data Fig. 1 [file 44319_2025_493_MOESM2_ESM.zip › Figure1/Fig1F/Experiment-04_VC_wiltype.tif_files/Experiment-04_z15c0x0-1388y0-1040.tif]

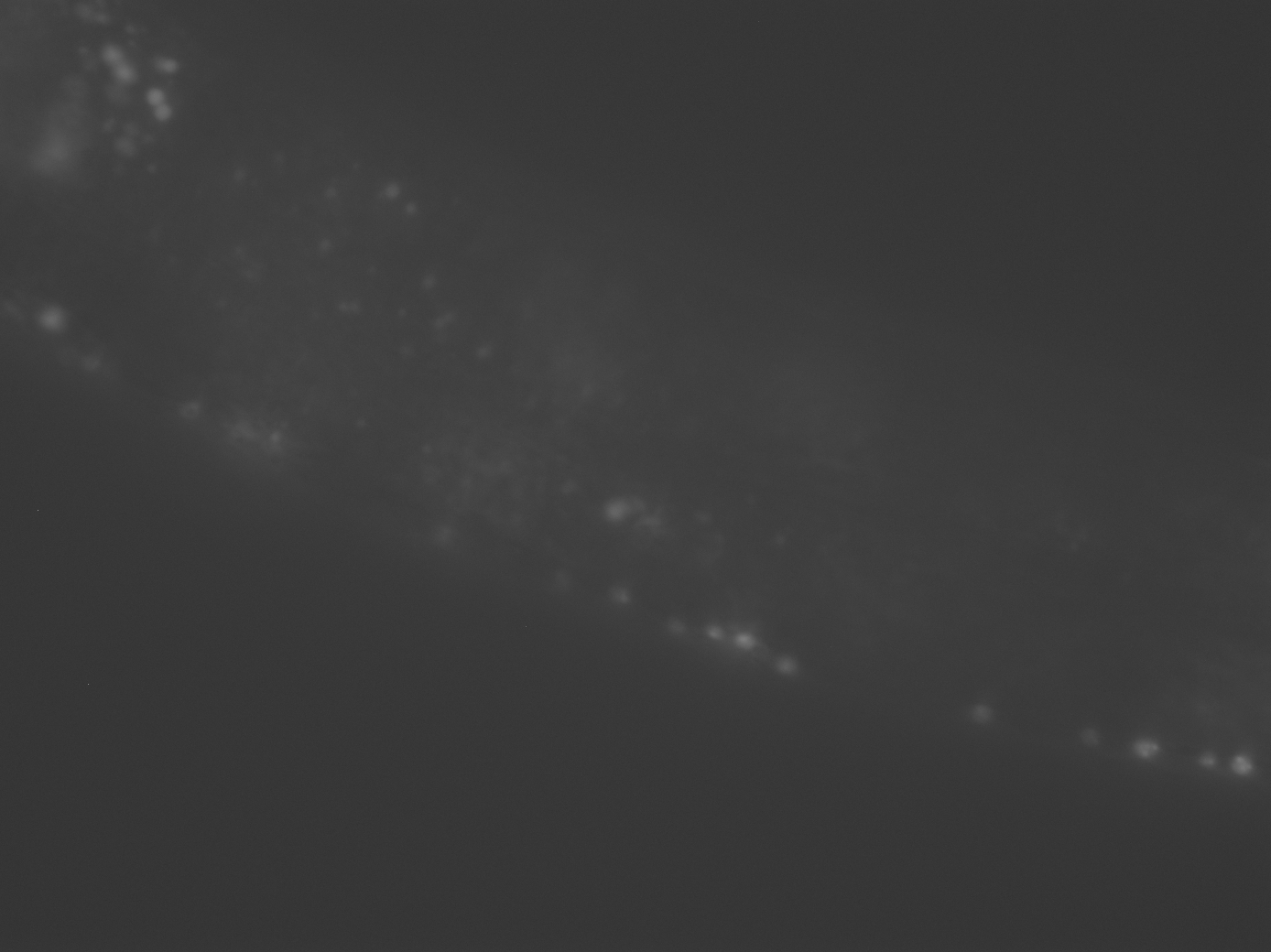

Supplement: Supplementary file 2 — Source data Fig. 1 [file 44319_2025_493_MOESM2_ESM.zip › Figure1/Fig1F/Experiment-04_VC_wiltype.tif_files/Experiment-04_z6c1x0-1388y0-1040.tif]

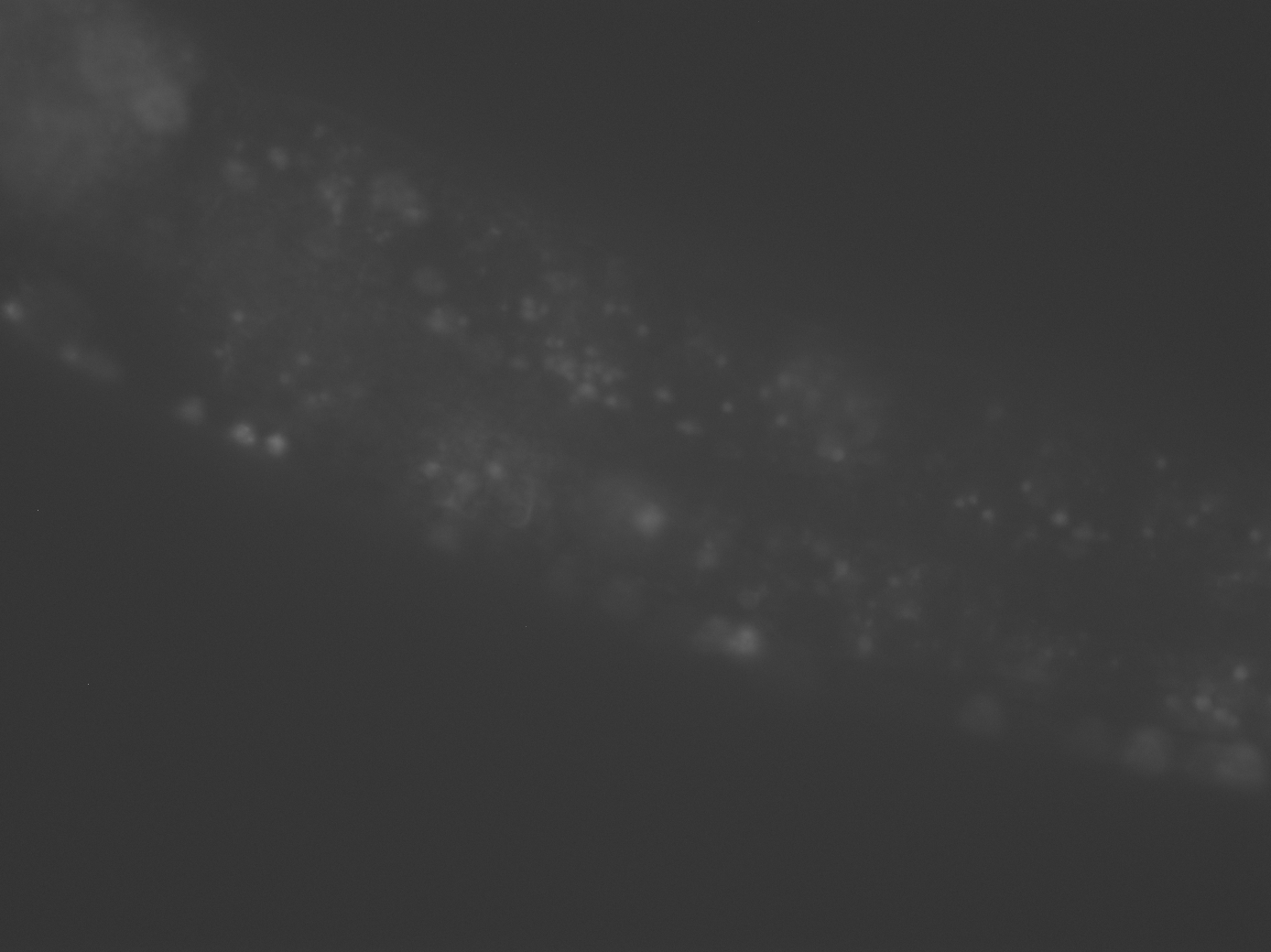

Supplement: Supplementary file 2 — Source data Fig. 1 [file 44319_2025_493_MOESM2_ESM.zip › Figure1/Fig1F/Experiment-04_VC_wiltype.tif_files/Experiment-04_z12c1x0-1388y0-1040.tif]

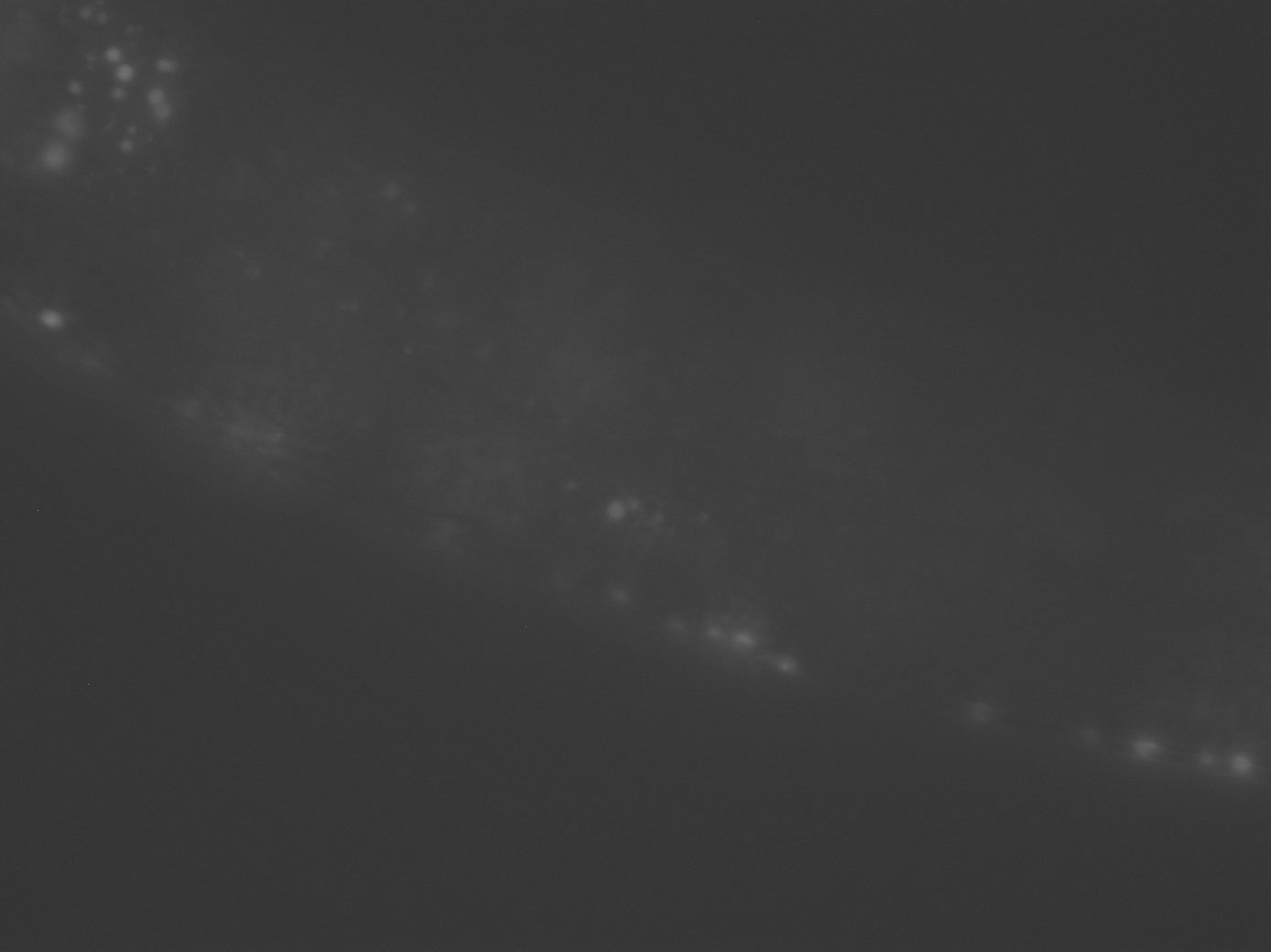

Supplement: Supplementary file 2 — Source data Fig. 1 [file 44319_2025_493_MOESM2_ESM.zip › Figure1/Fig1F/Experiment-04_VC_wiltype.tif_files/Experiment-04_z4c1x0-1388y0-1040.tif]

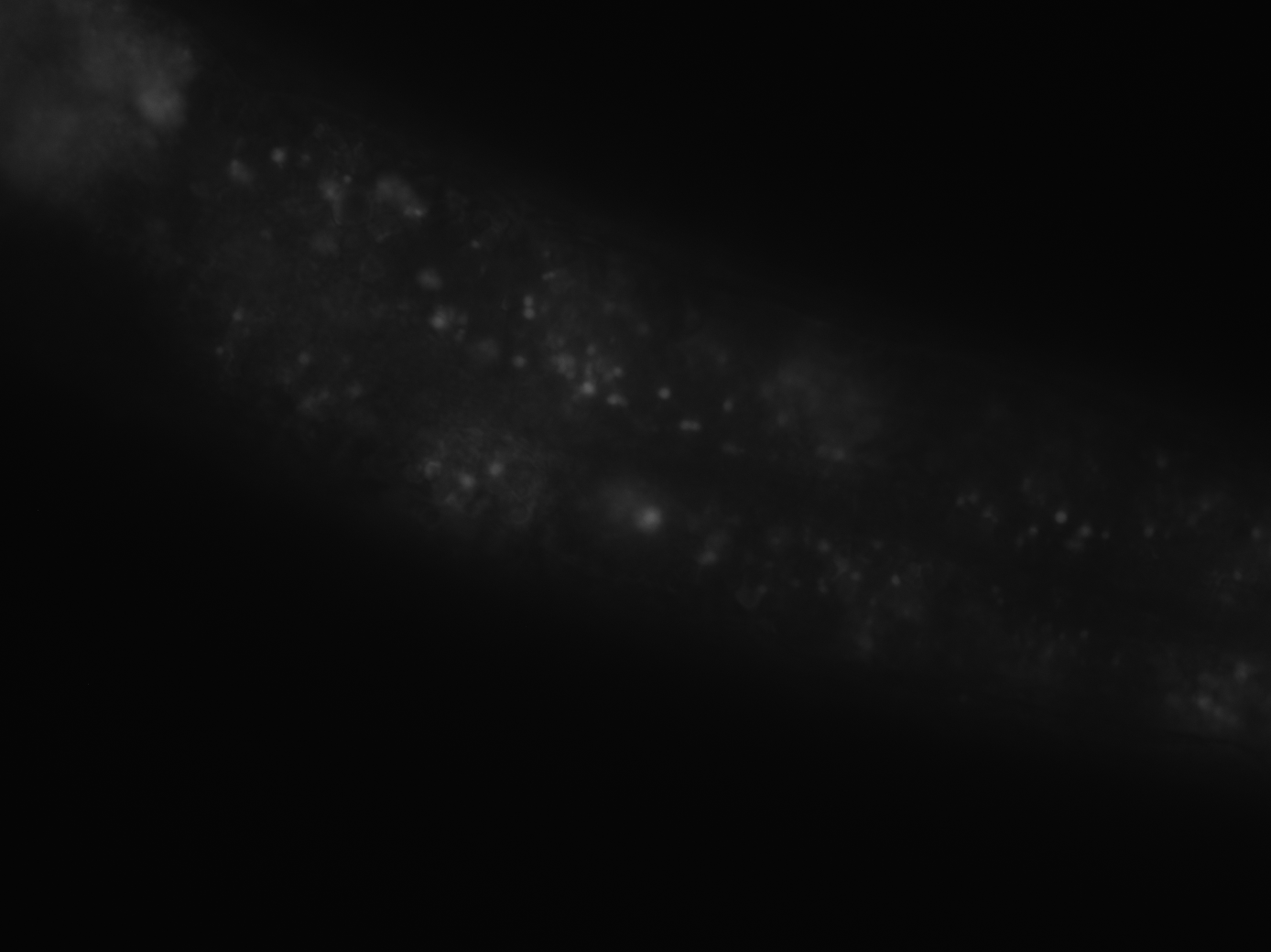

Supplement: Supplementary file 2 — Source data Fig. 1 [file 44319_2025_493_MOESM2_ESM.zip › Figure1/Fig1F/Experiment-04_VC_wiltype.tif_files/Experiment-04_z11c0x0-1388y0-1040.tif]

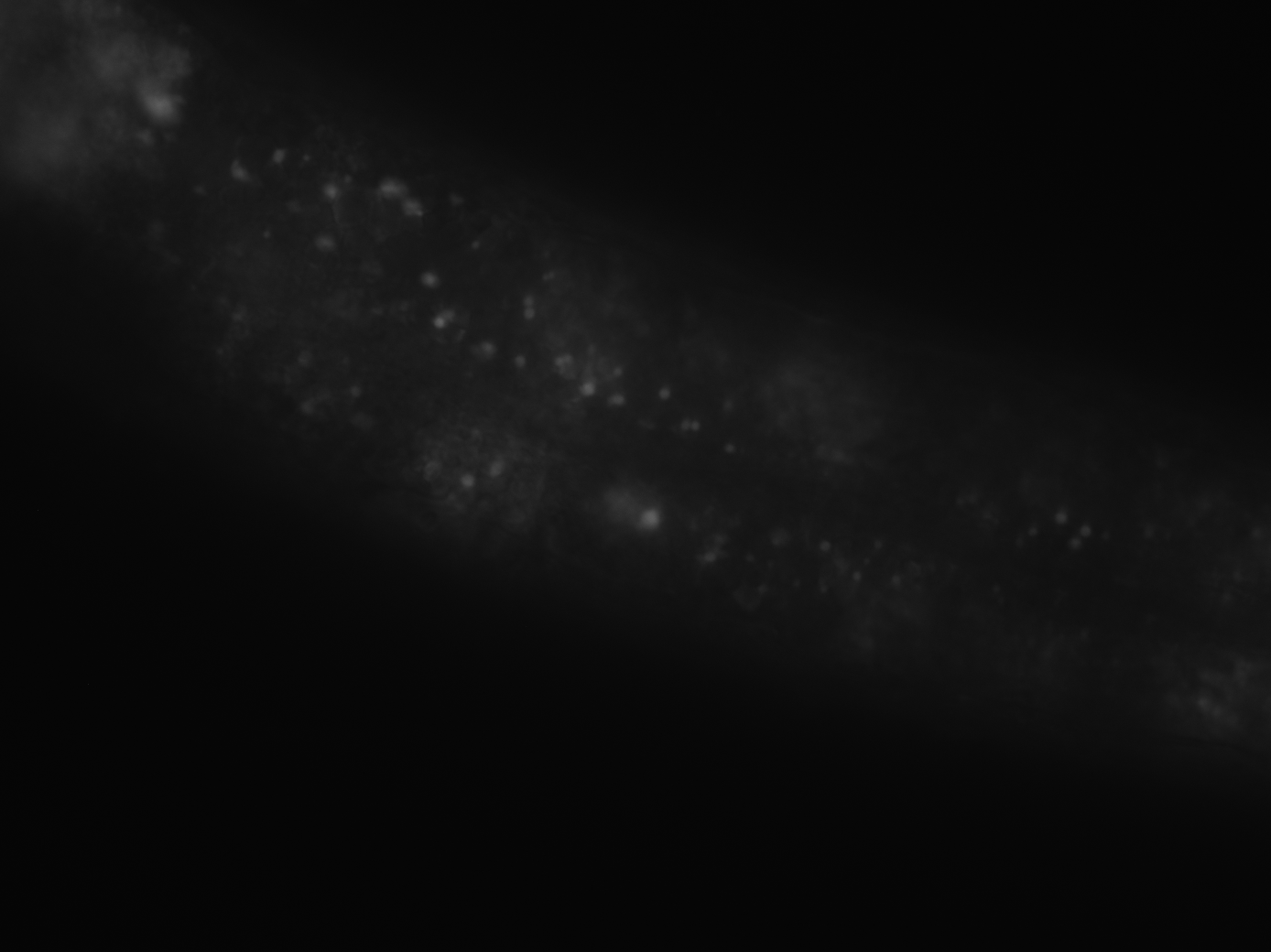

Supplement: Supplementary file 2 — Source data Fig. 1 [file 44319_2025_493_MOESM2_ESM.zip › Figure1/Fig1F/Experiment-04_VC_wiltype.tif_files/Experiment-04_z10c0x0-1388y0-1040.tif]

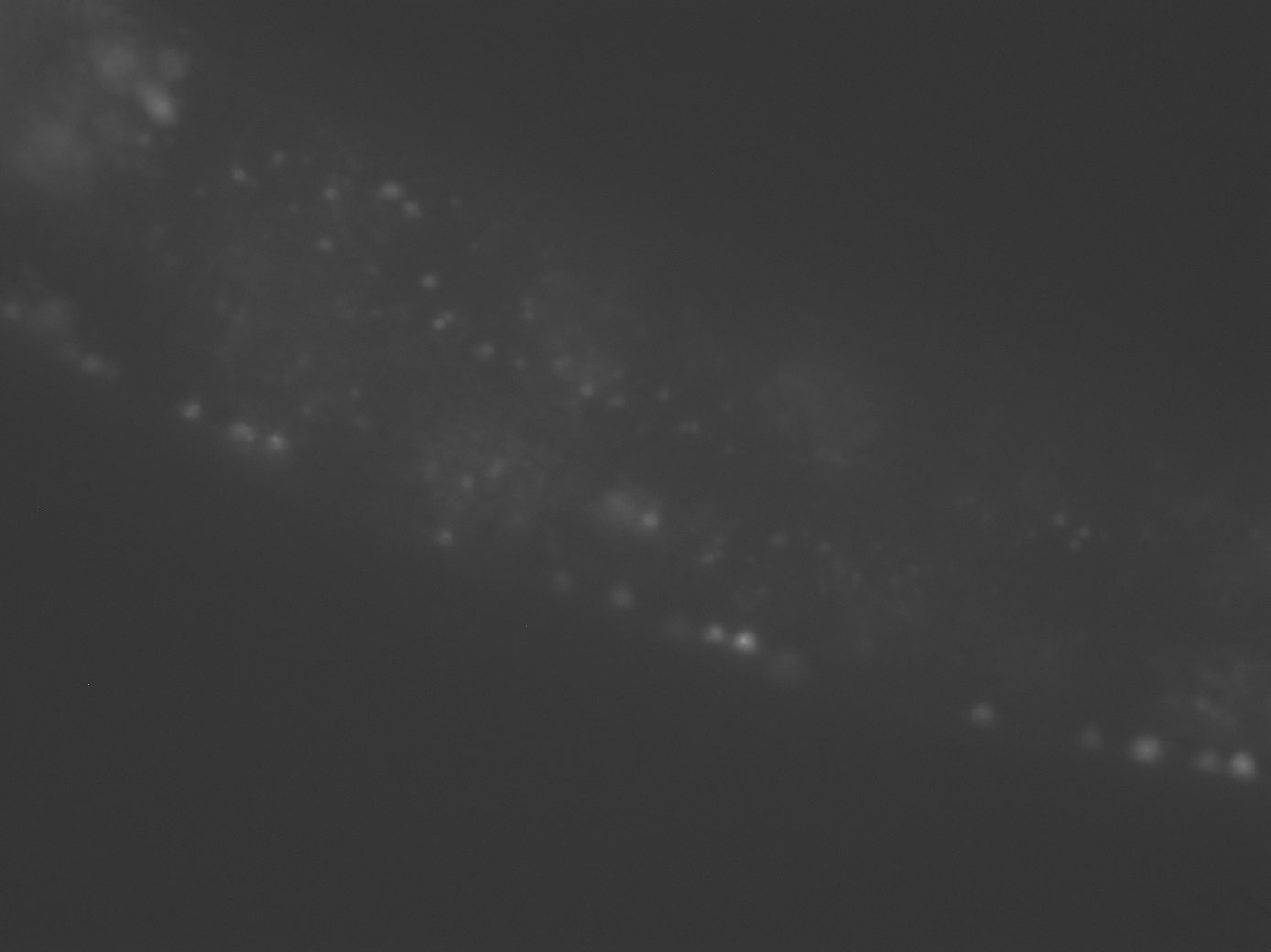

Supplement: Supplementary file 2 — Source data Fig. 1 [file 44319_2025_493_MOESM2_ESM.zip › Figure1/Fig1F/Experiment-04_VC_wiltype.tif_files/Experiment-04_z9c1x0-1388y0-1040.tif]

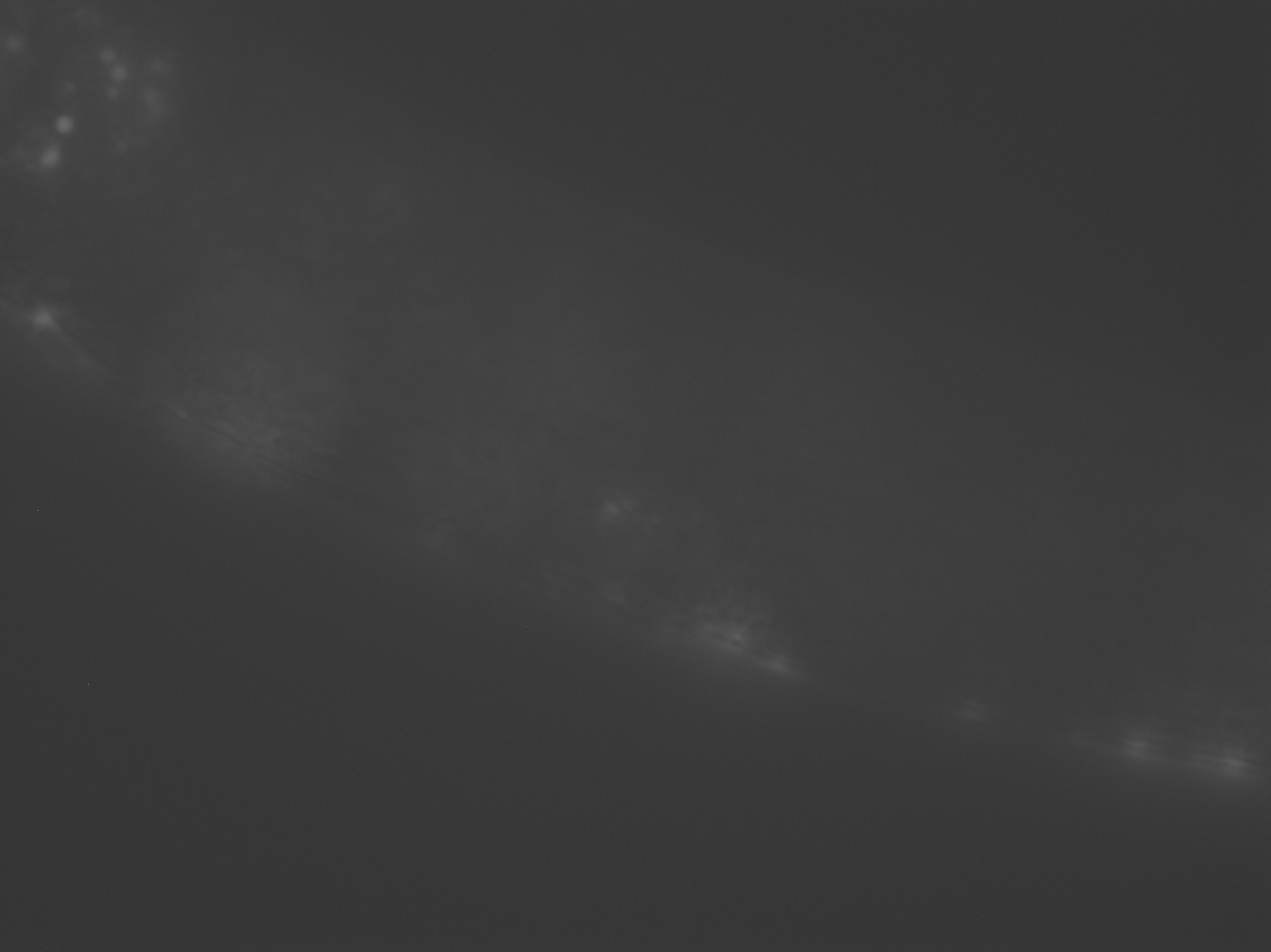

Supplement: Supplementary file 2 — Source data Fig. 1 [file 44319_2025_493_MOESM2_ESM.zip › Figure1/Fig1F/Experiment-04_VC_wiltype.tif_files/Experiment-04_z0c1x0-1388y0-1040.tif]

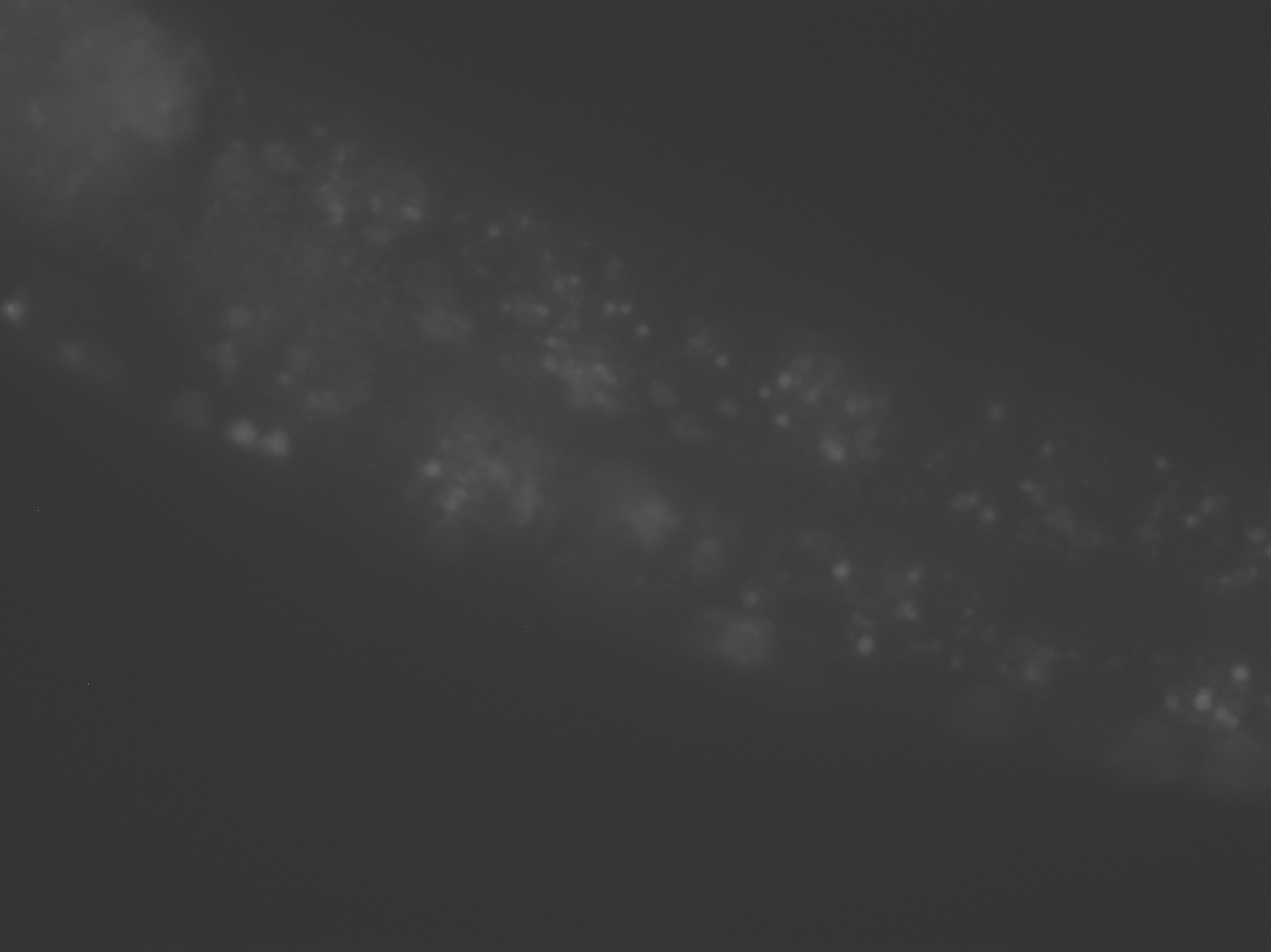

Supplement: Supplementary file 2 — Source data Fig. 1 [file 44319_2025_493_MOESM2_ESM.zip › Figure1/Fig1F/Experiment-04_VC_wiltype.tif_files/Experiment-04_z14c1x0-1388y0-1040.tif]

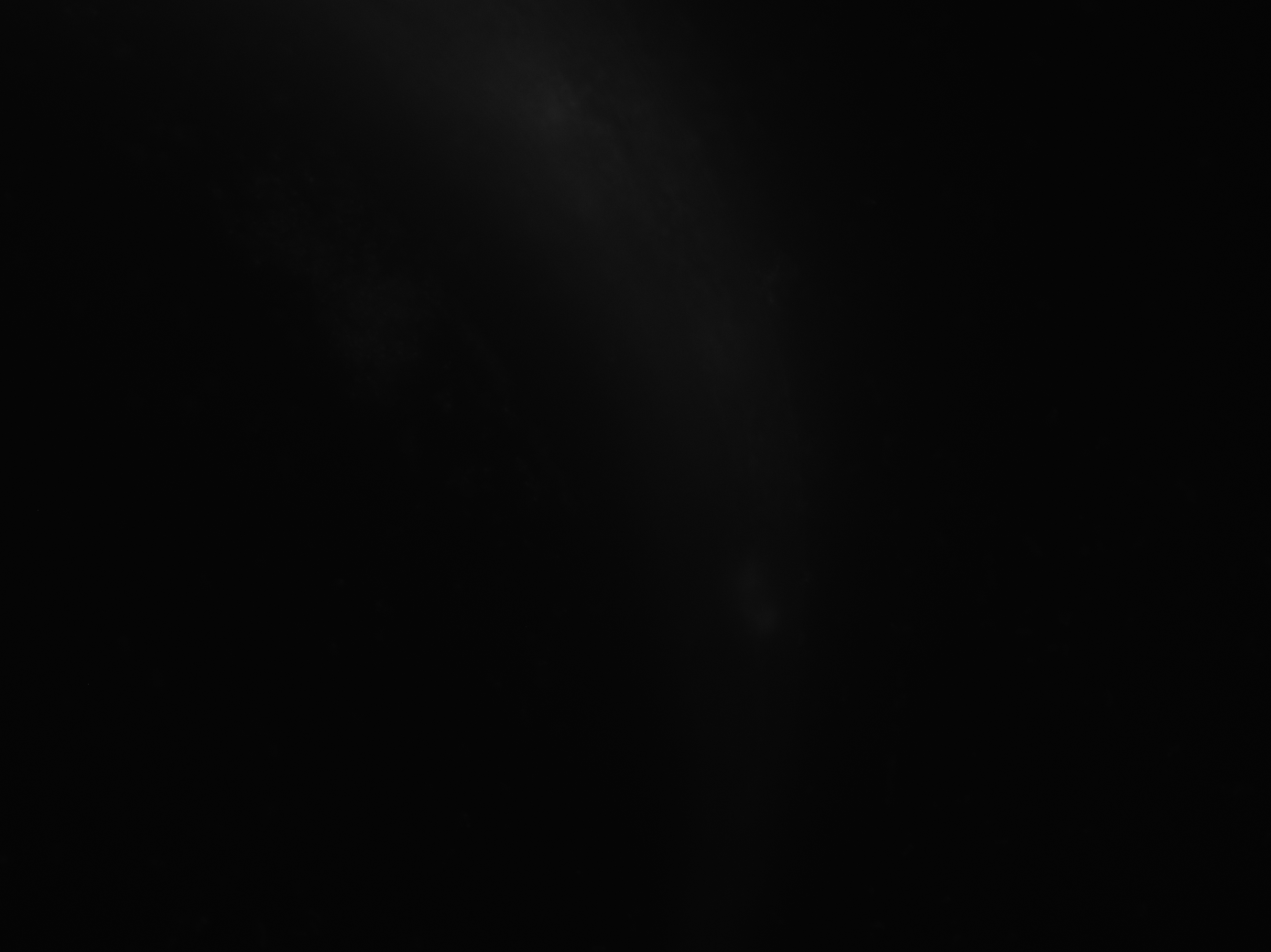

Supplement: Supplementary file 2 — Source data Fig. 1 [file 44319_2025_493_MOESM2_ESM.zip › Figure1/Fig1F/Experiment-11_tail_wildtype.tif_files/Experiment-11_z2c0x0-1388y0-1040.tif]

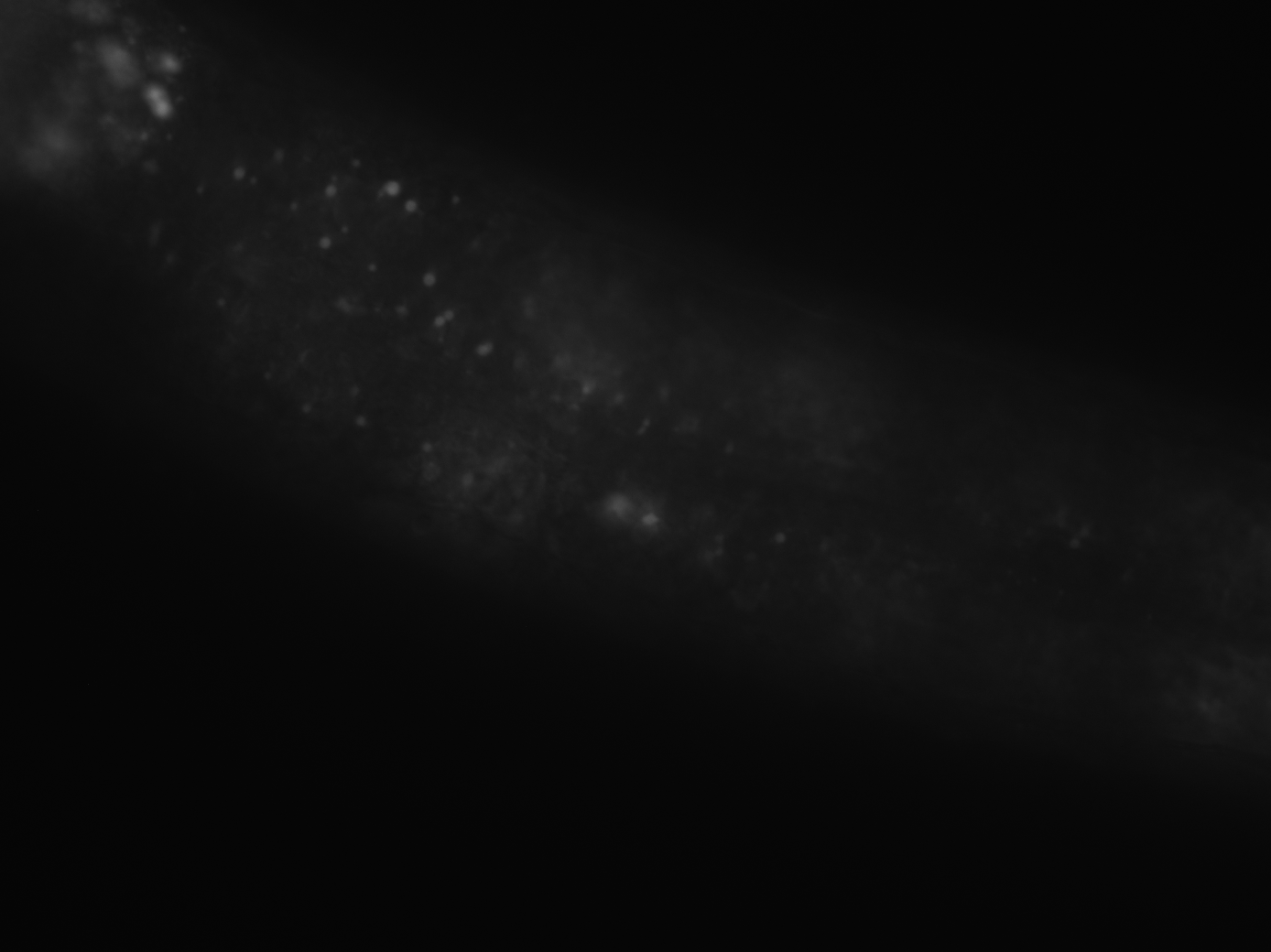

Supplement: Supplementary file 2 — Source data Fig. 1 [file 44319_2025_493_MOESM2_ESM.zip › Figure1/Fig1F/Experiment-04_VC_wiltype.tif_files/Experiment-04_z8c0x0-1388y0-1040.tif]

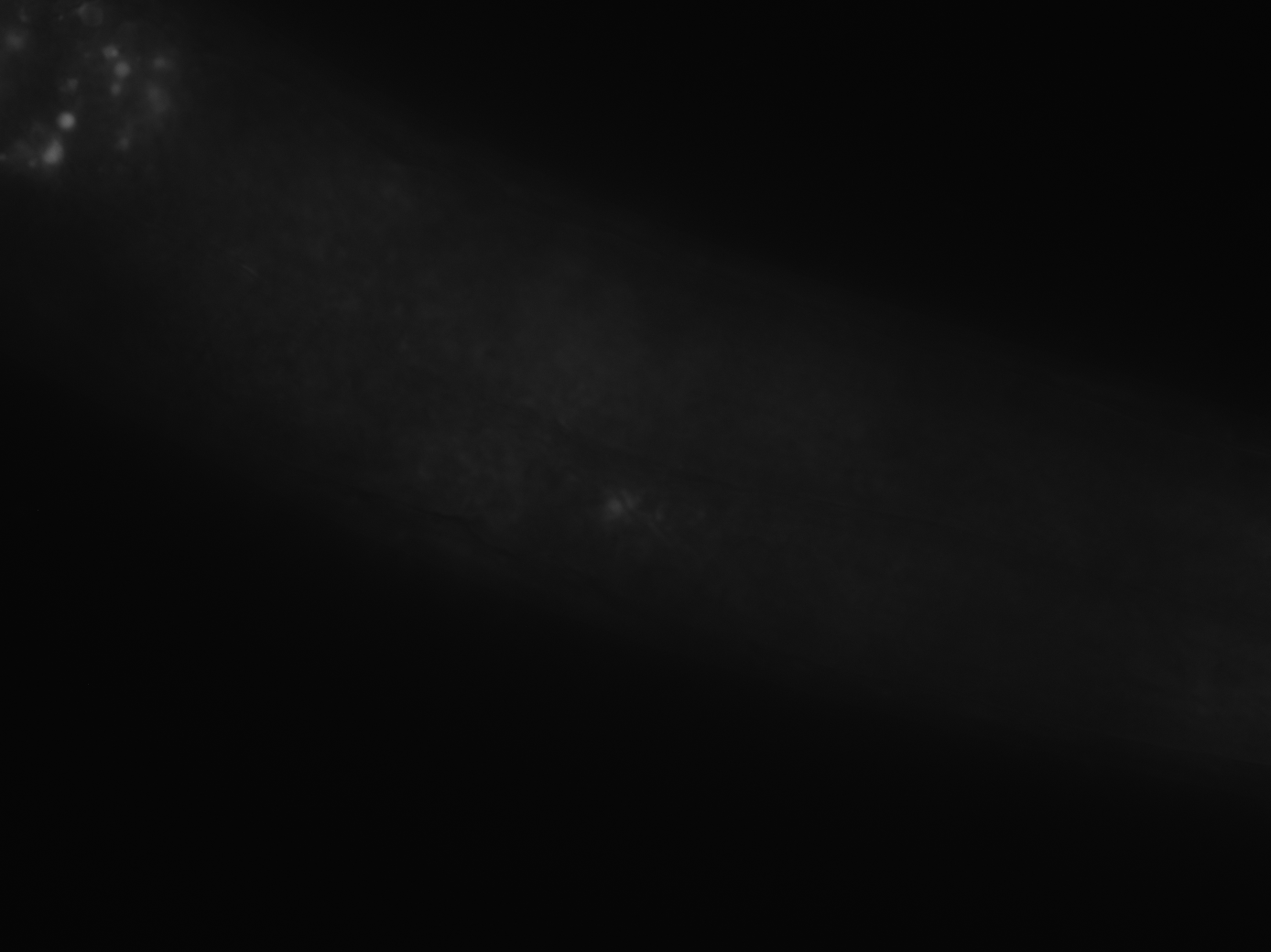

Supplement: Supplementary file 2 — Source data Fig. 1 [file 44319_2025_493_MOESM2_ESM.zip › Figure1/Fig1F/Experiment-04_VC_wiltype.tif_files/Experiment-04_z2c0x0-1388y0-1040.tif]

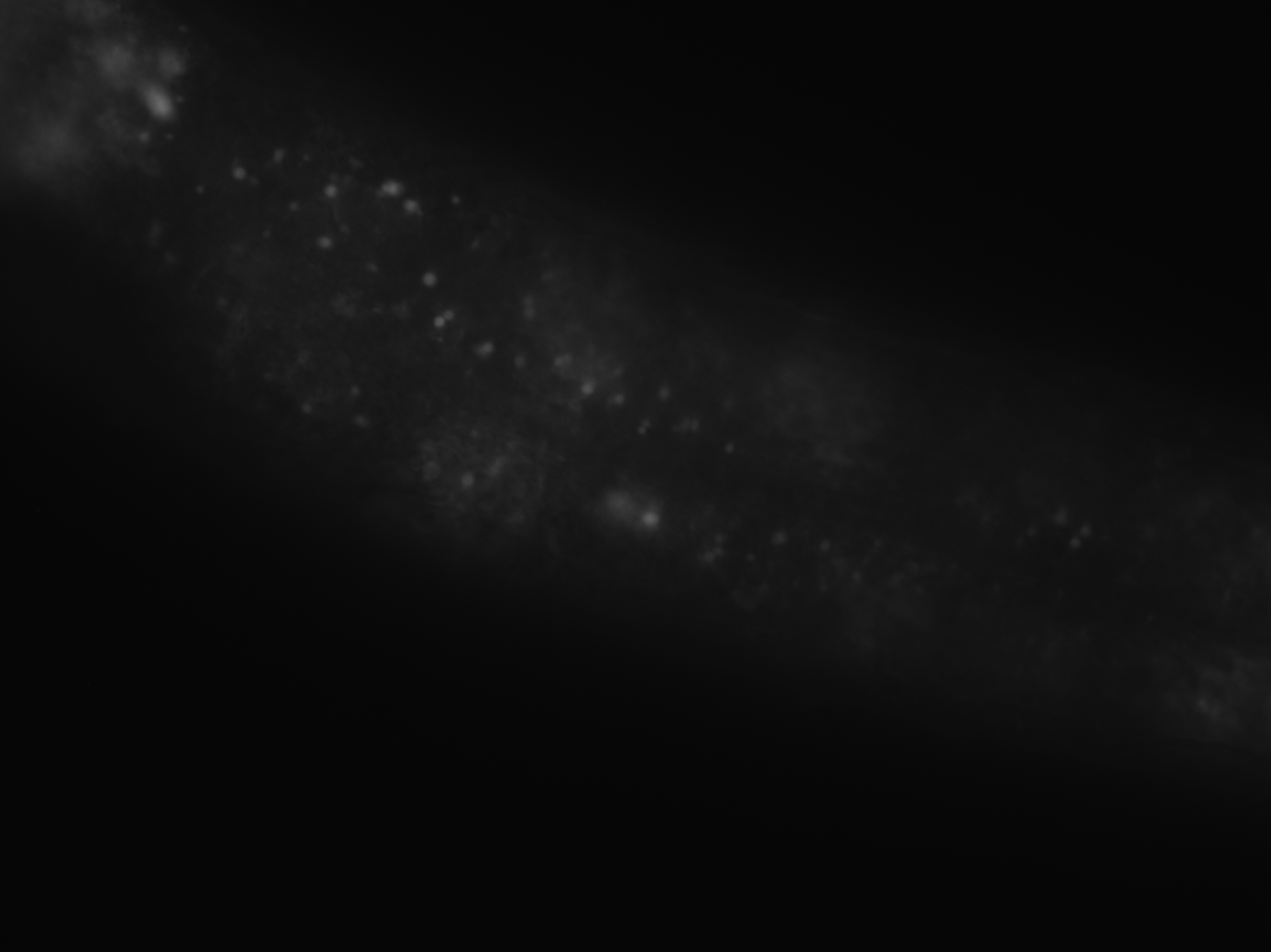

Supplement: Supplementary file 2 — Source data Fig. 1 [file 44319_2025_493_MOESM2_ESM.zip › Figure1/Fig1F/Experiment-04_VC_wiltype.tif_files/Experiment-04_z9c0x0-1388y0-1040.tif]

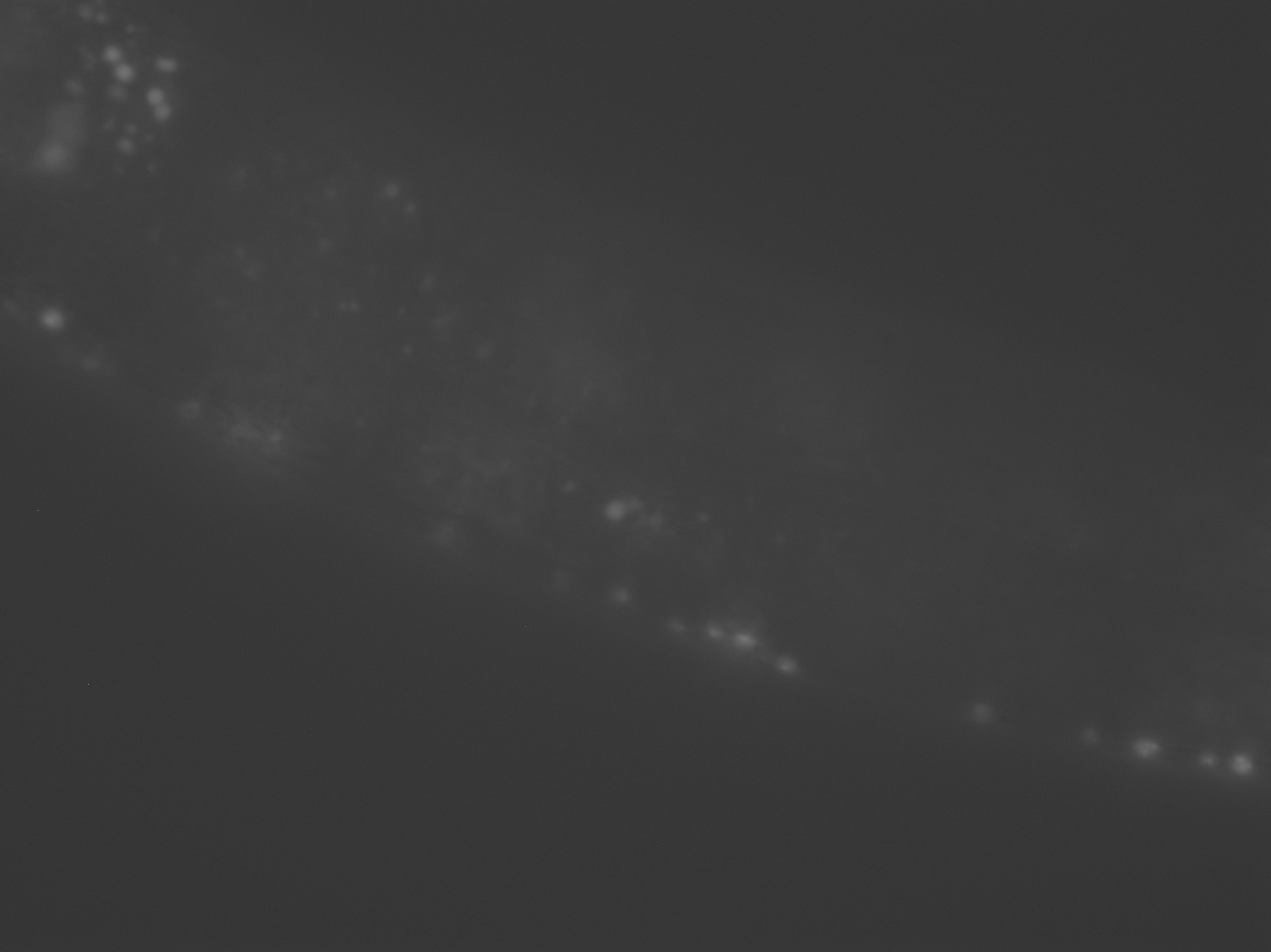

Supplement: Supplementary file 2 — Source data Fig. 1 [file 44319_2025_493_MOESM2_ESM.zip › Figure1/Fig1F/Experiment-04_VC_wiltype.tif_files/Experiment-04_z5c1x0-1388y0-1040.tif]

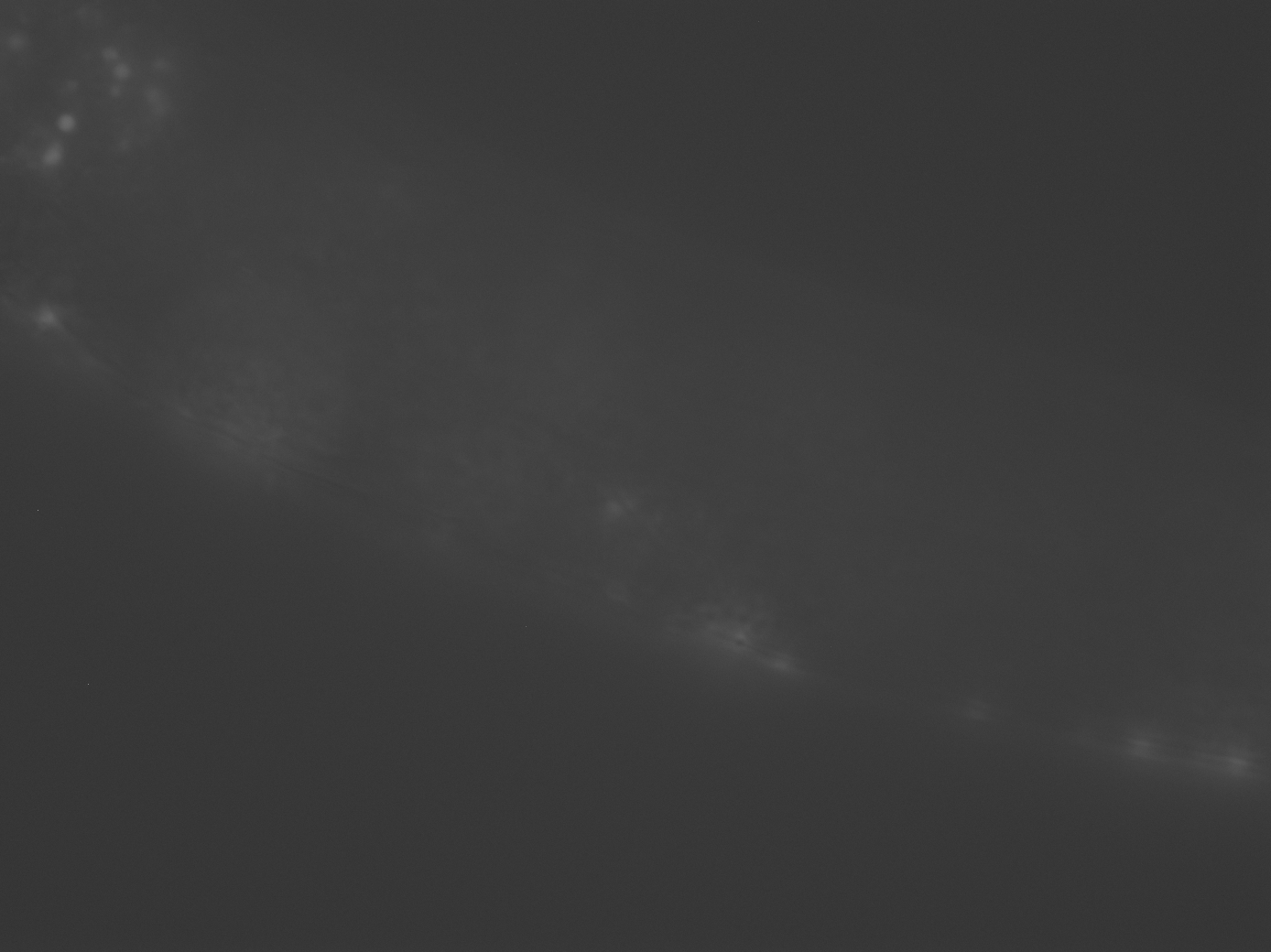

Supplement: Supplementary file 2 — Source data Fig. 1 [file 44319_2025_493_MOESM2_ESM.zip › Figure1/Fig1F/Experiment-04_VC_wiltype.tif_files/Experiment-04_z1c1x0-1388y0-1040.tif]

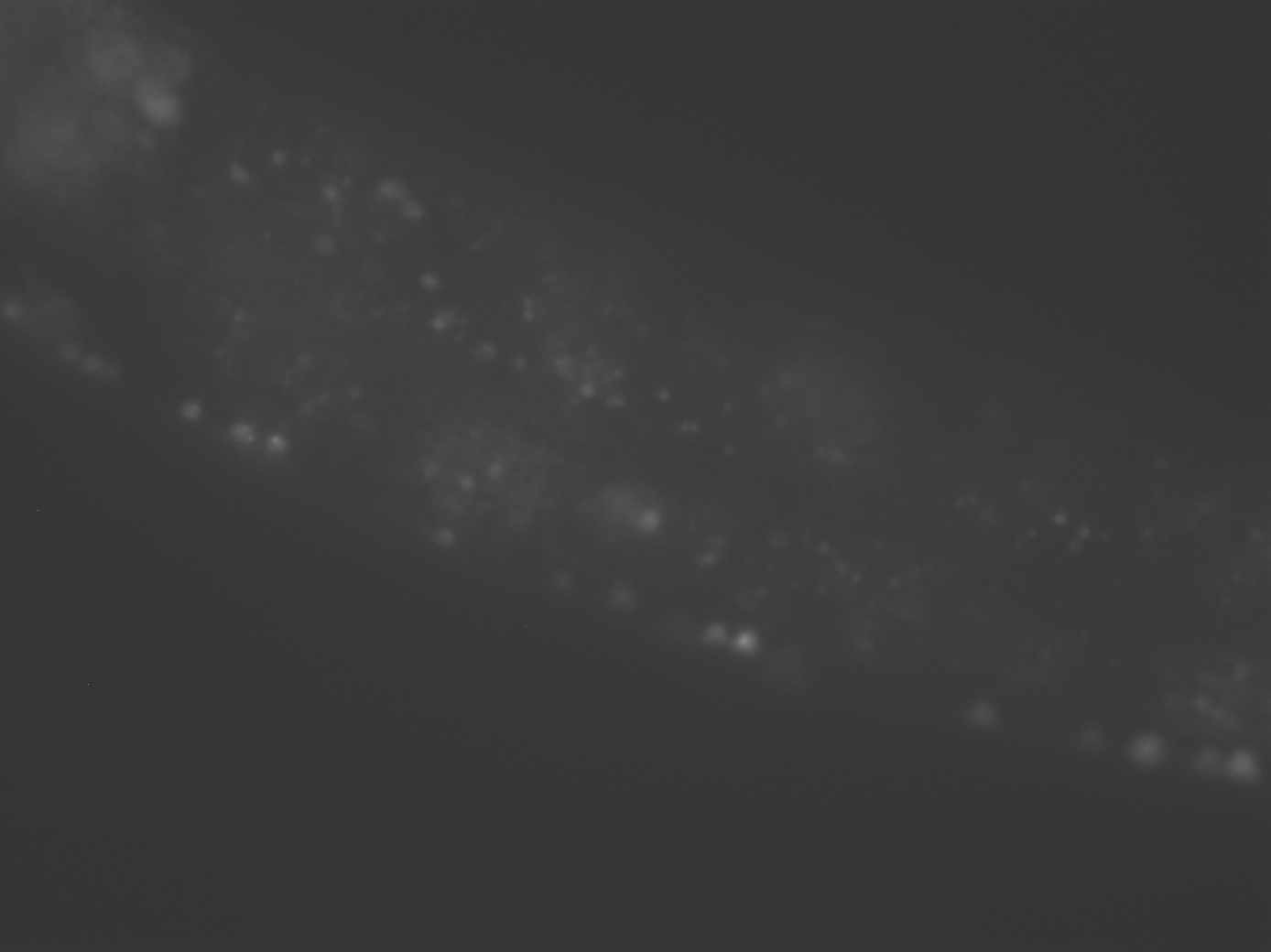

Supplement: Supplementary file 2 — Source data Fig. 1 [file 44319_2025_493_MOESM2_ESM.zip › Figure1/Fig1F/Experiment-04_VC_wiltype.tif_files/Experiment-04_z10c1x0-1388y0-1040.tif]

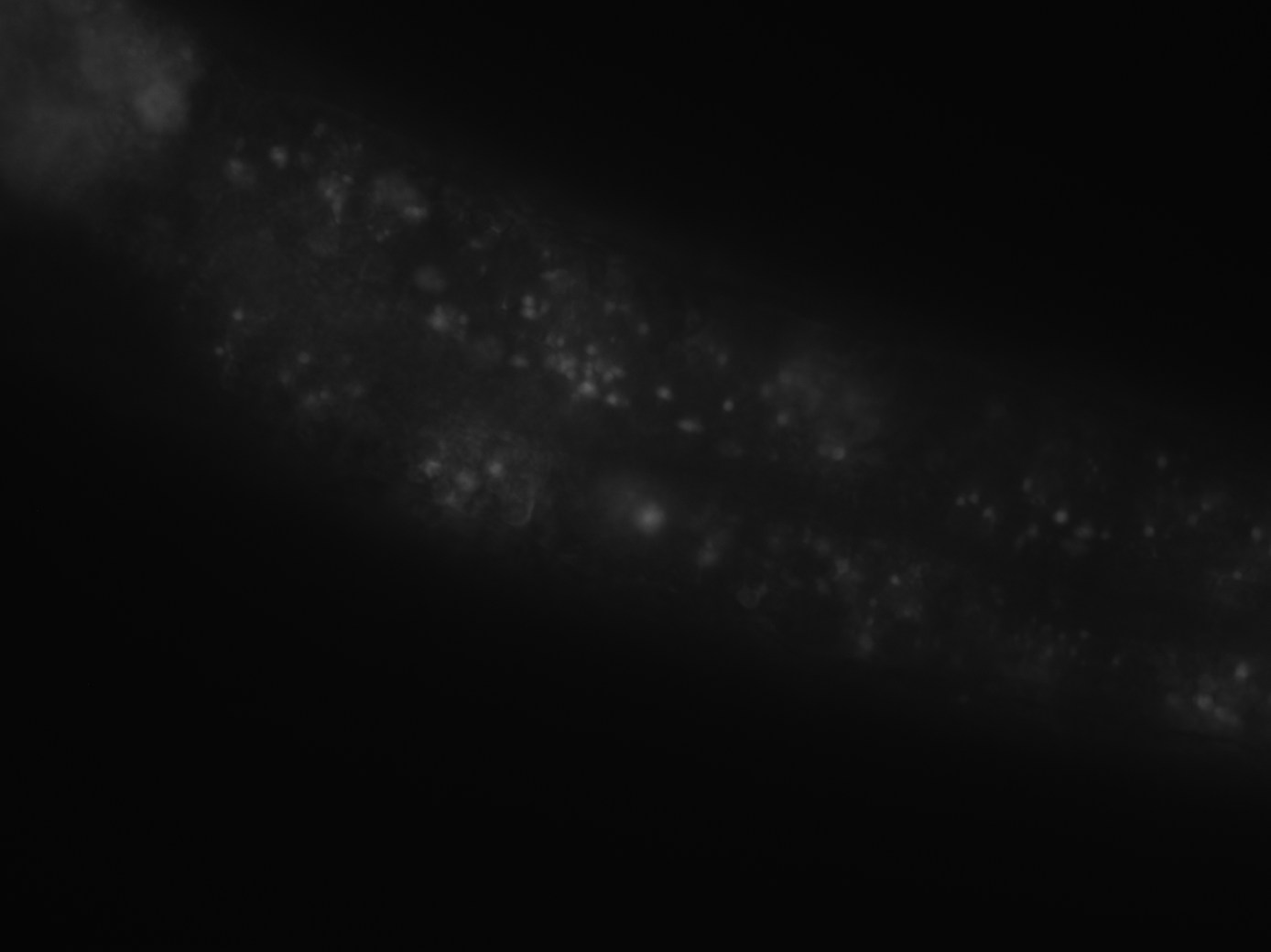

Supplement: Supplementary file 2 — Source data Fig. 1 [file 44319_2025_493_MOESM2_ESM.zip › Figure1/Fig1F/Experiment-04_VC_wiltype.tif_files/Experiment-04_z12c0x0-1388y0-1040.tif]

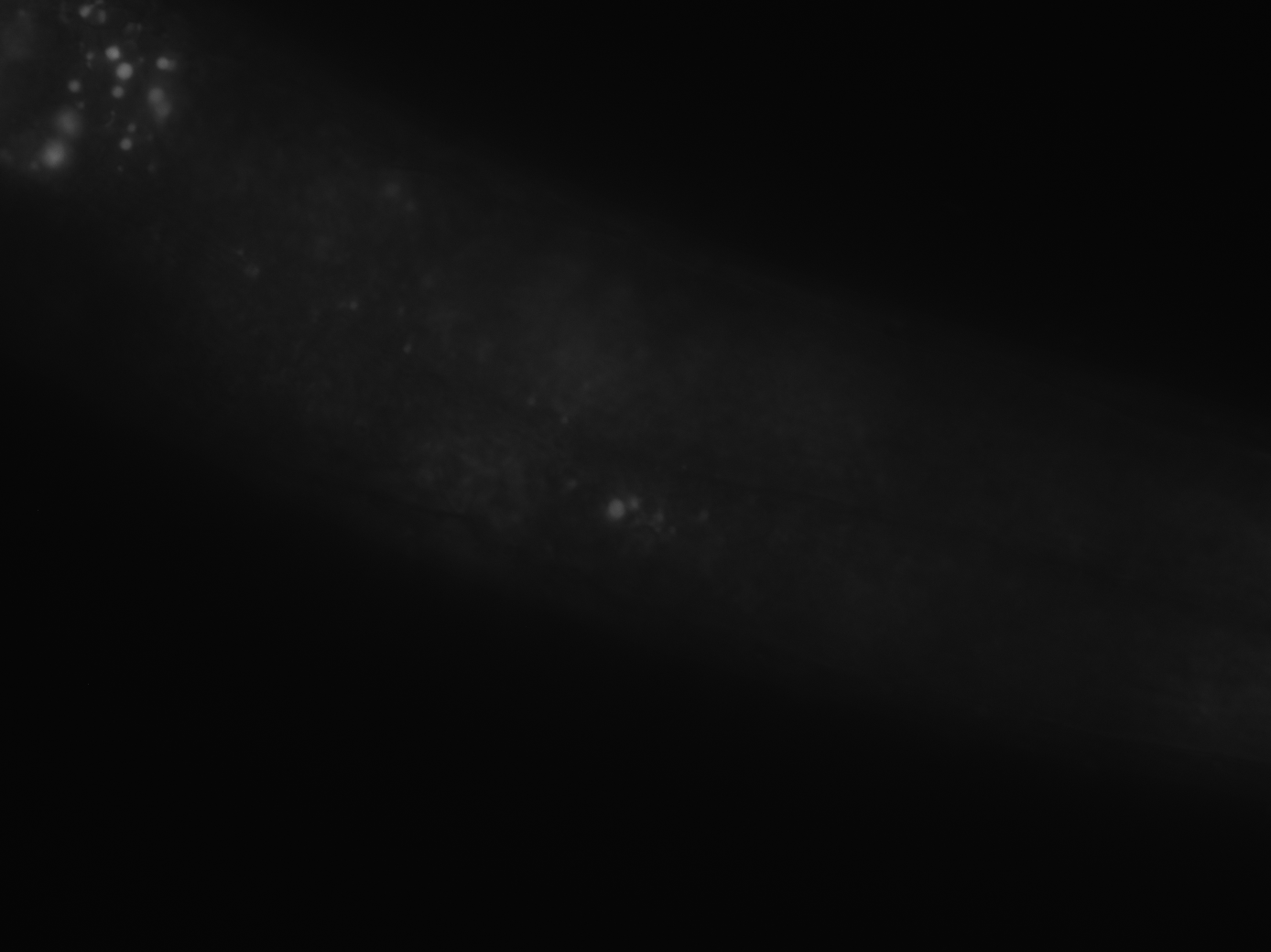

Supplement: Supplementary file 2 — Source data Fig. 1 [file 44319_2025_493_MOESM2_ESM.zip › Figure1/Fig1F/Experiment-04_VC_wiltype.tif_files/Experiment-04_z4c0x0-1388y0-1040.tif]

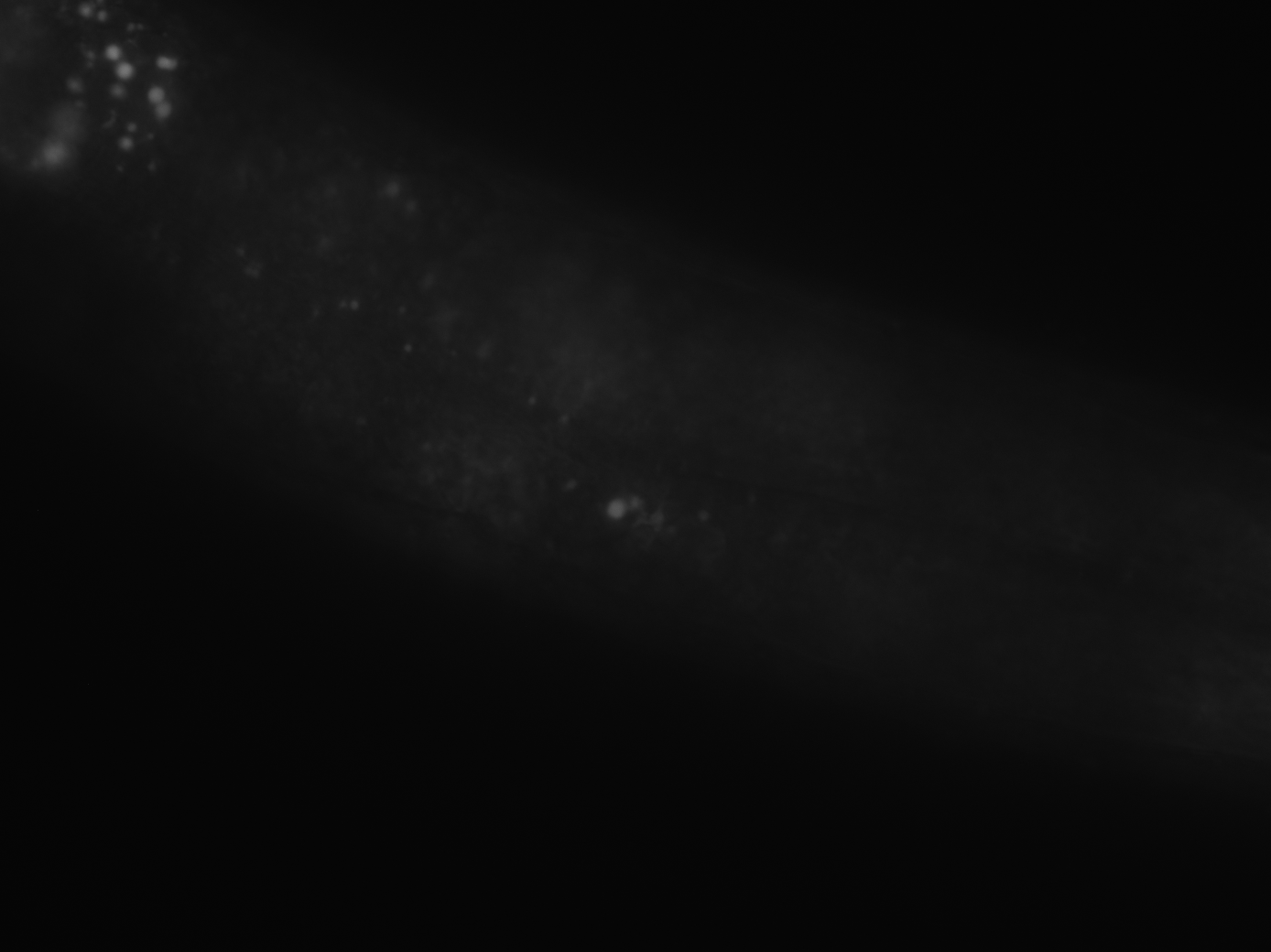

Supplement: Supplementary file 2 — Source data Fig. 1 [file 44319_2025_493_MOESM2_ESM.zip › Figure1/Fig1F/Experiment-04_VC_wiltype.tif_files/Experiment-04_z5c0x0-1388y0-1040.tif]

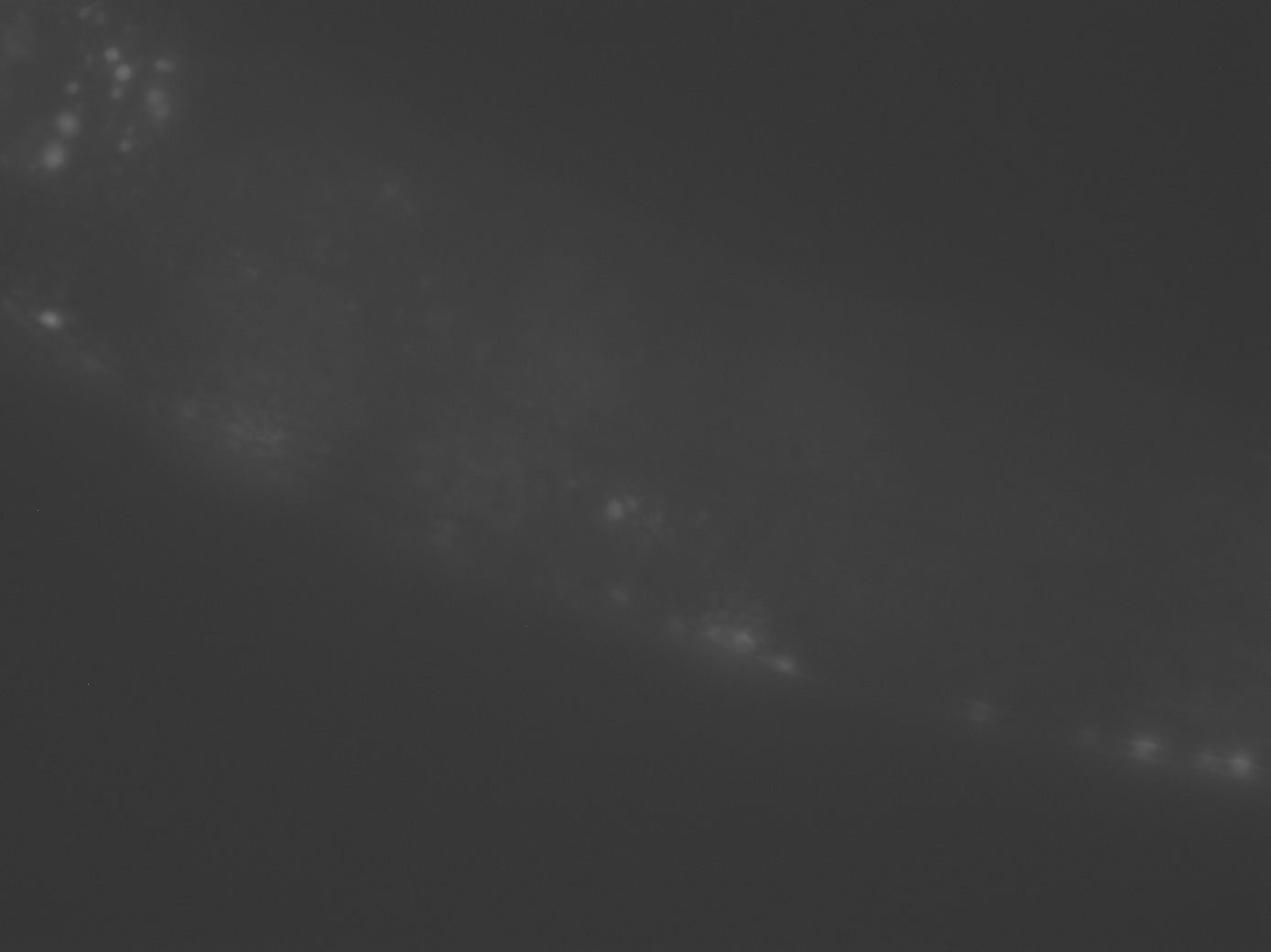

Supplement: Supplementary file 2 — Source data Fig. 1 [file 44319_2025_493_MOESM2_ESM.zip › Figure1/Fig1F/Experiment-04_VC_wiltype.tif_files/Experiment-04_z3c1x0-1388y0-1040.tif]

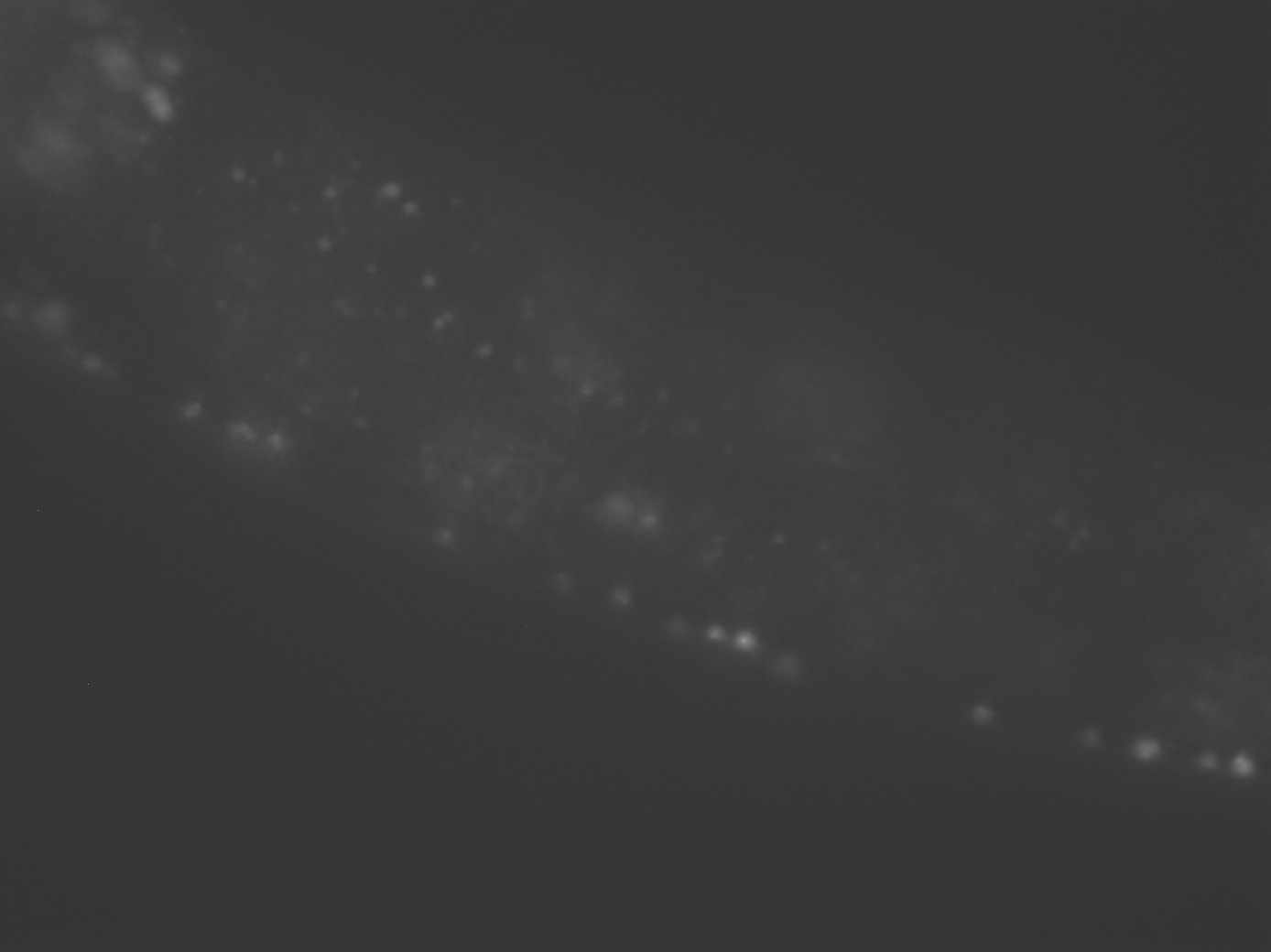

Supplement: Supplementary file 2 — Source data Fig. 1 [file 44319_2025_493_MOESM2_ESM.zip › Figure1/Fig1F/Experiment-04_VC_wiltype.tif_files/Experiment-04_z8c1x0-1388y0-1040.tif]

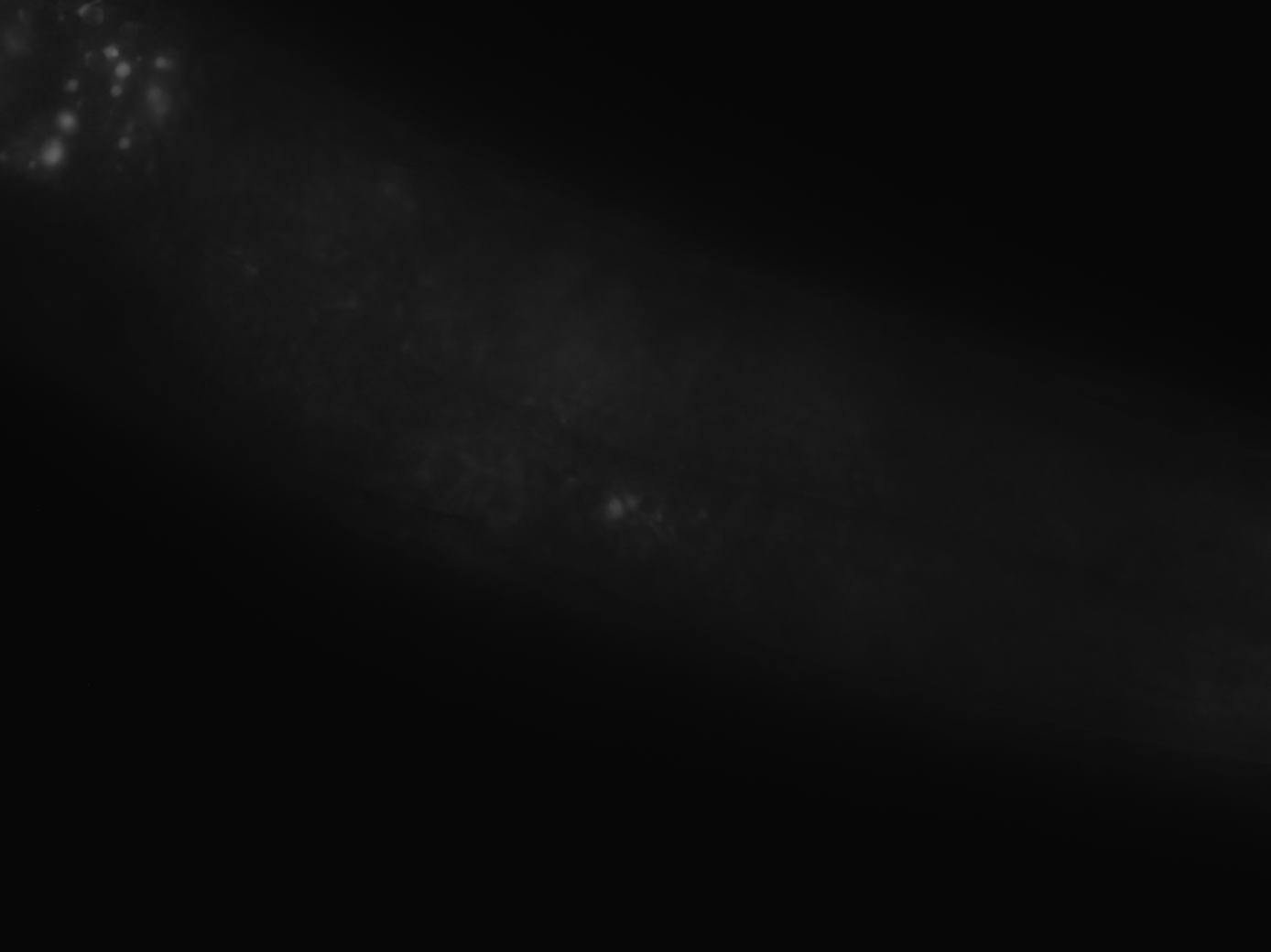

Supplement: Supplementary file 2 — Source data Fig. 1 [file 44319_2025_493_MOESM2_ESM.zip › Figure1/Fig1F/Experiment-04_VC_wiltype.tif_files/Experiment-04_z3c0x0-1388y0-1040.tif]

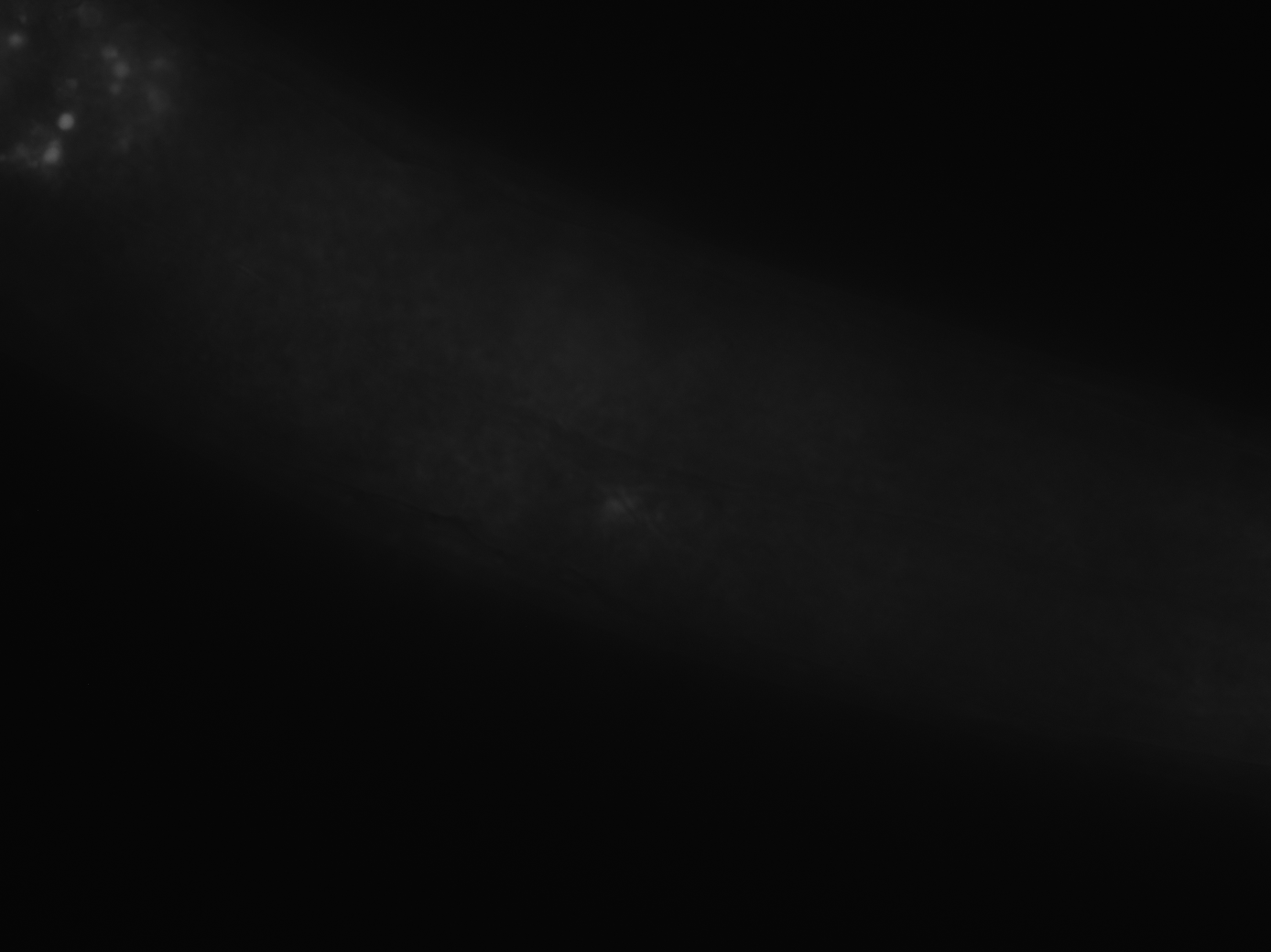

Supplement: Supplementary file 2 — Source data Fig. 1 [file 44319_2025_493_MOESM2_ESM.zip › Figure1/Fig1F/Experiment-04_VC_wiltype.tif_files/Experiment-04_z1c0x0-1388y0-1040.tif]

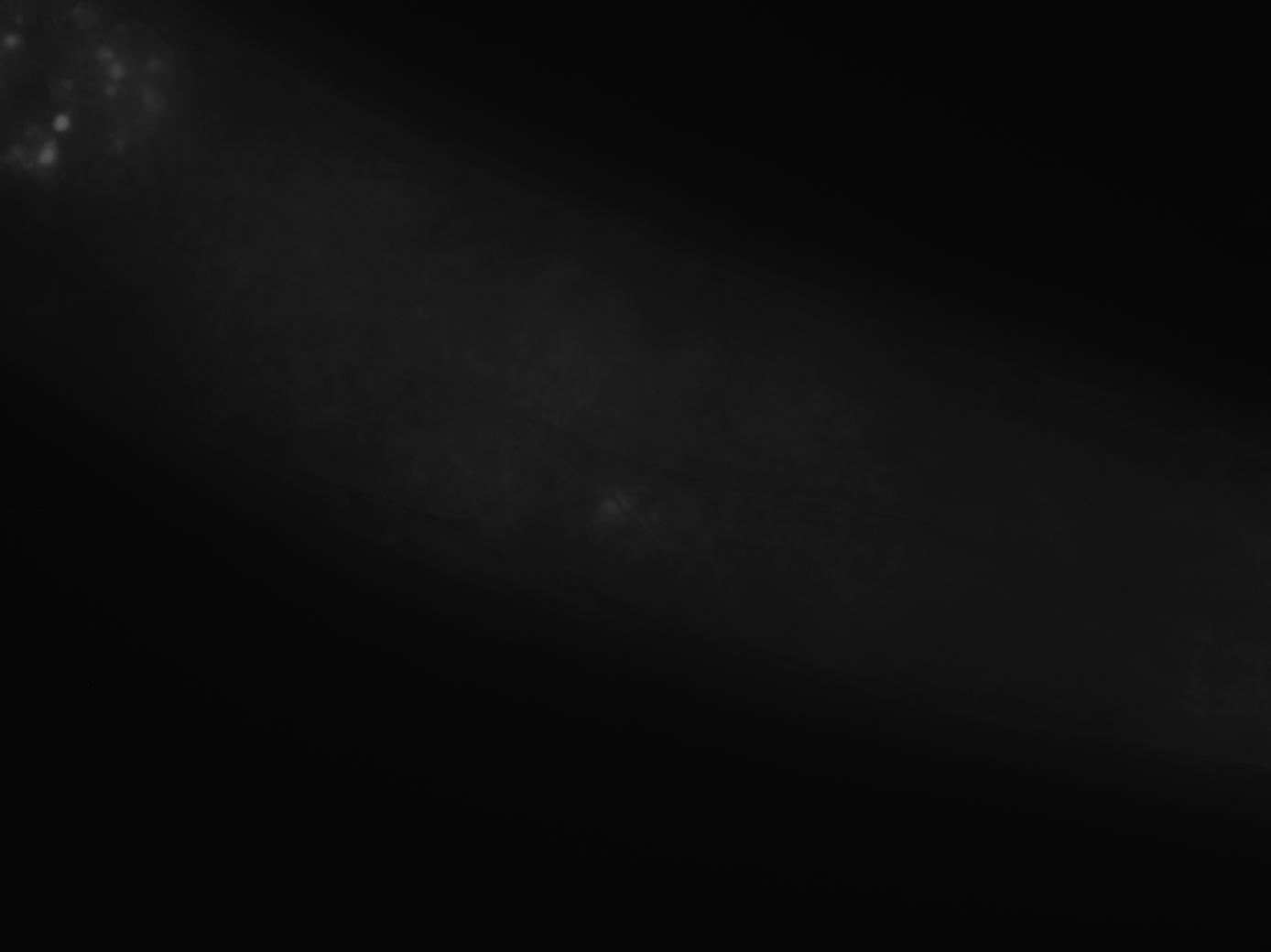

Supplement: Supplementary file 2 — Source data Fig. 1 [file 44319_2025_493_MOESM2_ESM.zip › Figure1/Fig1F/Experiment-04_VC_wiltype.tif_files/Experiment-04_z0c0x0-1388y0-1040.tif]

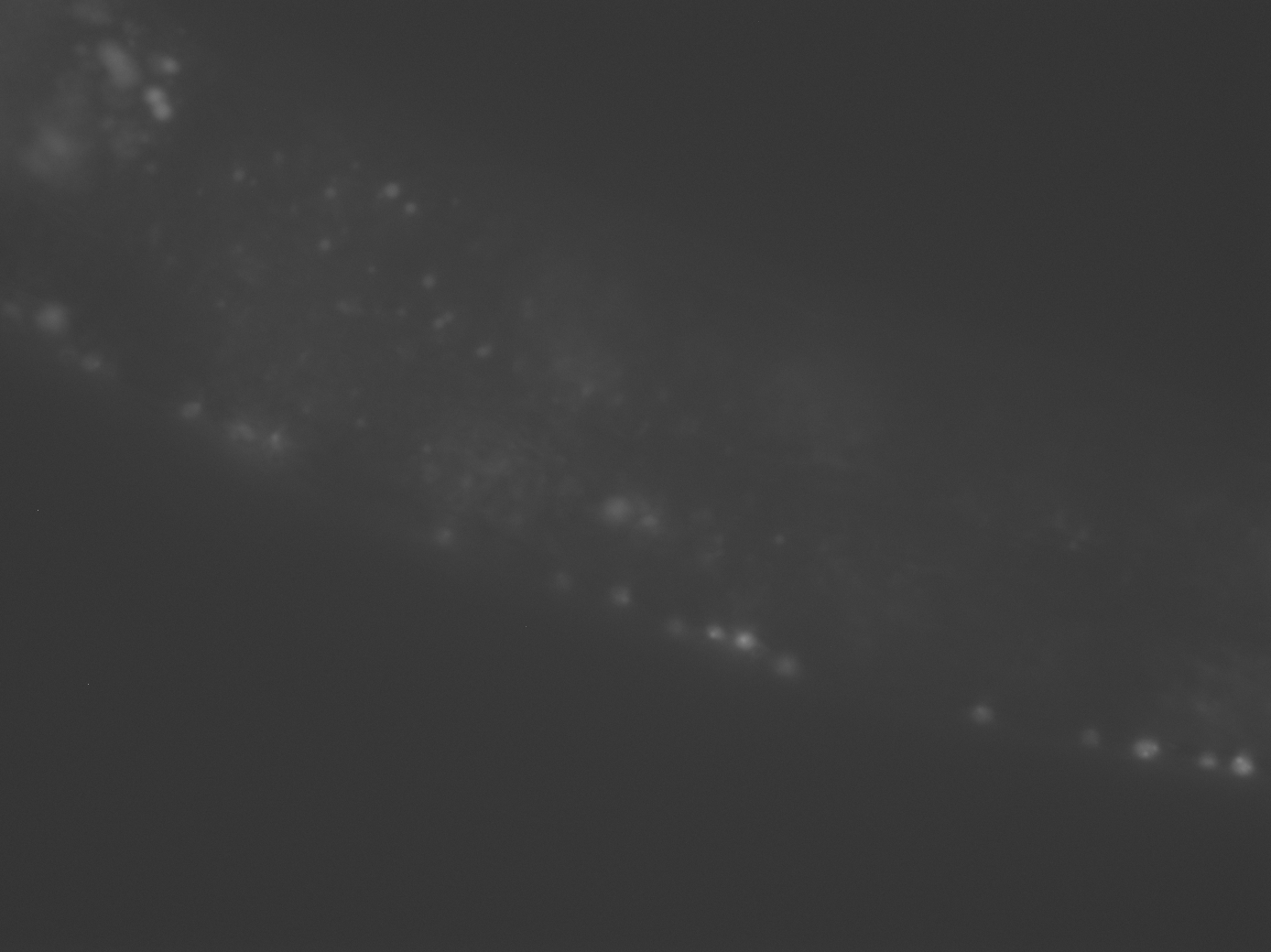

Supplement: Supplementary file 2 — Source data Fig. 1 [file 44319_2025_493_MOESM2_ESM.zip › Figure1/Fig1F/Experiment-04_VC_wiltype.tif_files/Experiment-04_z7c1x0-1388y0-1040.tif]

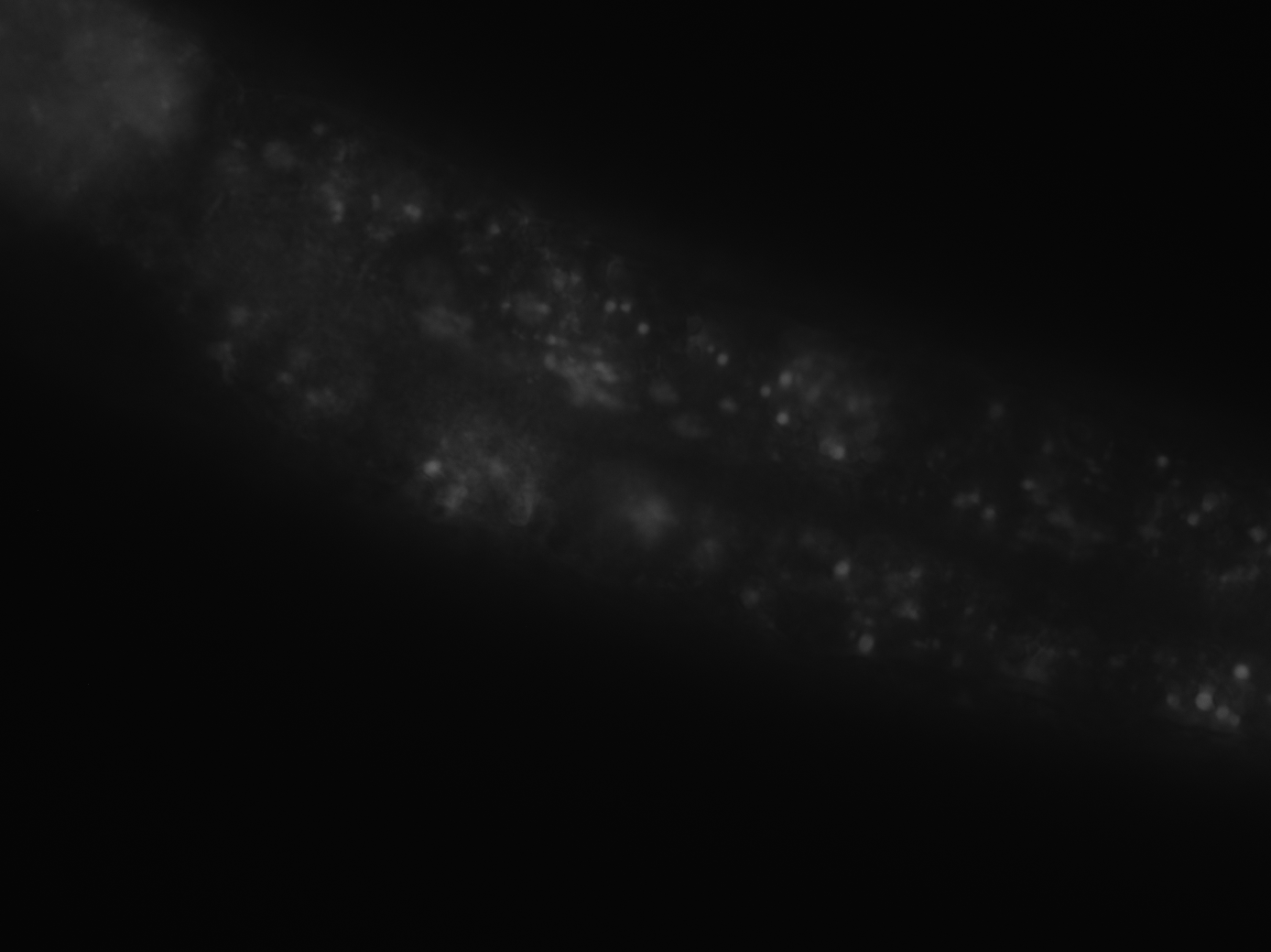

Supplement: Supplementary file 2 — Source data Fig. 1 [file 44319_2025_493_MOESM2_ESM.zip › Figure1/Fig1F/Experiment-04_VC_wiltype.tif_files/Experiment-04_z14c0x0-1388y0-1040.tif]

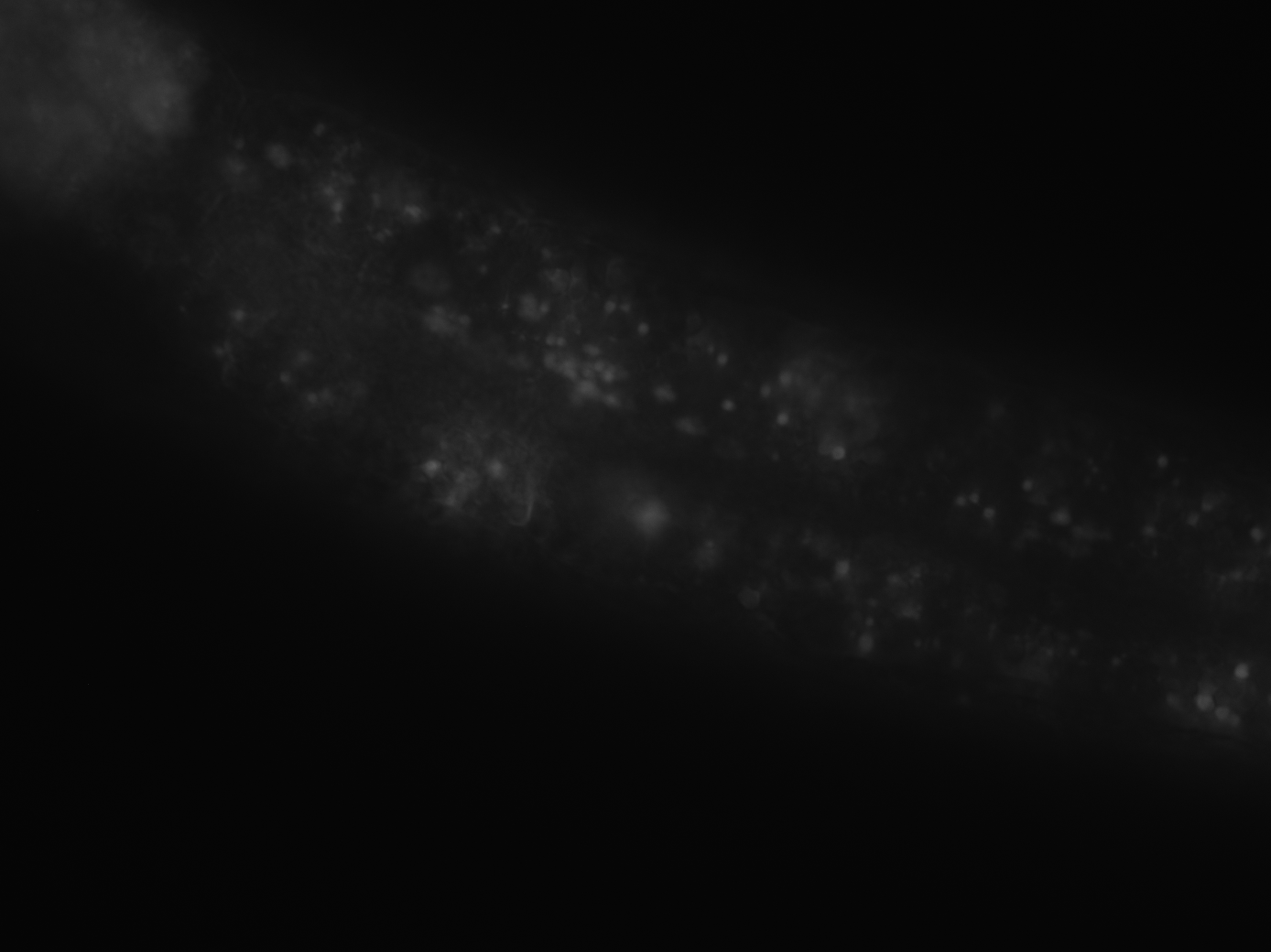

Supplement: Supplementary file 2 — Source data Fig. 1 [file 44319_2025_493_MOESM2_ESM.zip › Figure1/Fig1F/Experiment-04_VC_wiltype.tif_files/Experiment-04_z13c0x0-1388y0-1040.tif]

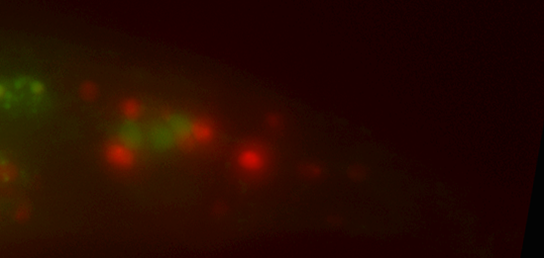

Supplement: Supplementary file 2 — Source data Fig. 1 [file 44319_2025_493_MOESM2_ESM.zip › Figure1/Fig1F/Experiment-11_tail_wildtype.tif_files/processed/tail.tif]

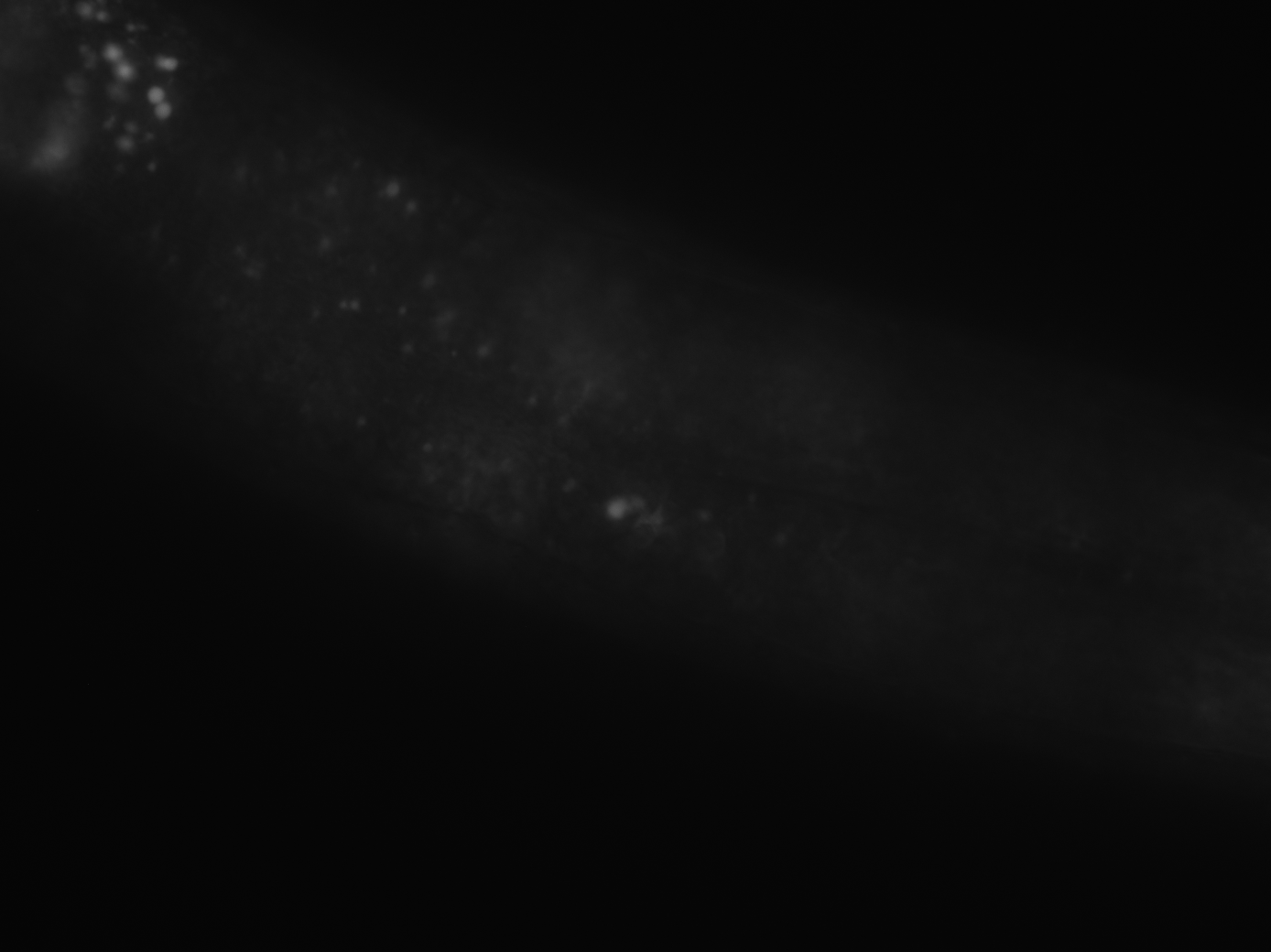

Supplement: Supplementary file 2 — Source data Fig. 1 [file 44319_2025_493_MOESM2_ESM.zip › Figure1/Fig1F/Experiment-04_VC_wiltype.tif_files/Experiment-04_z6c0x0-1388y0-1040.tif]

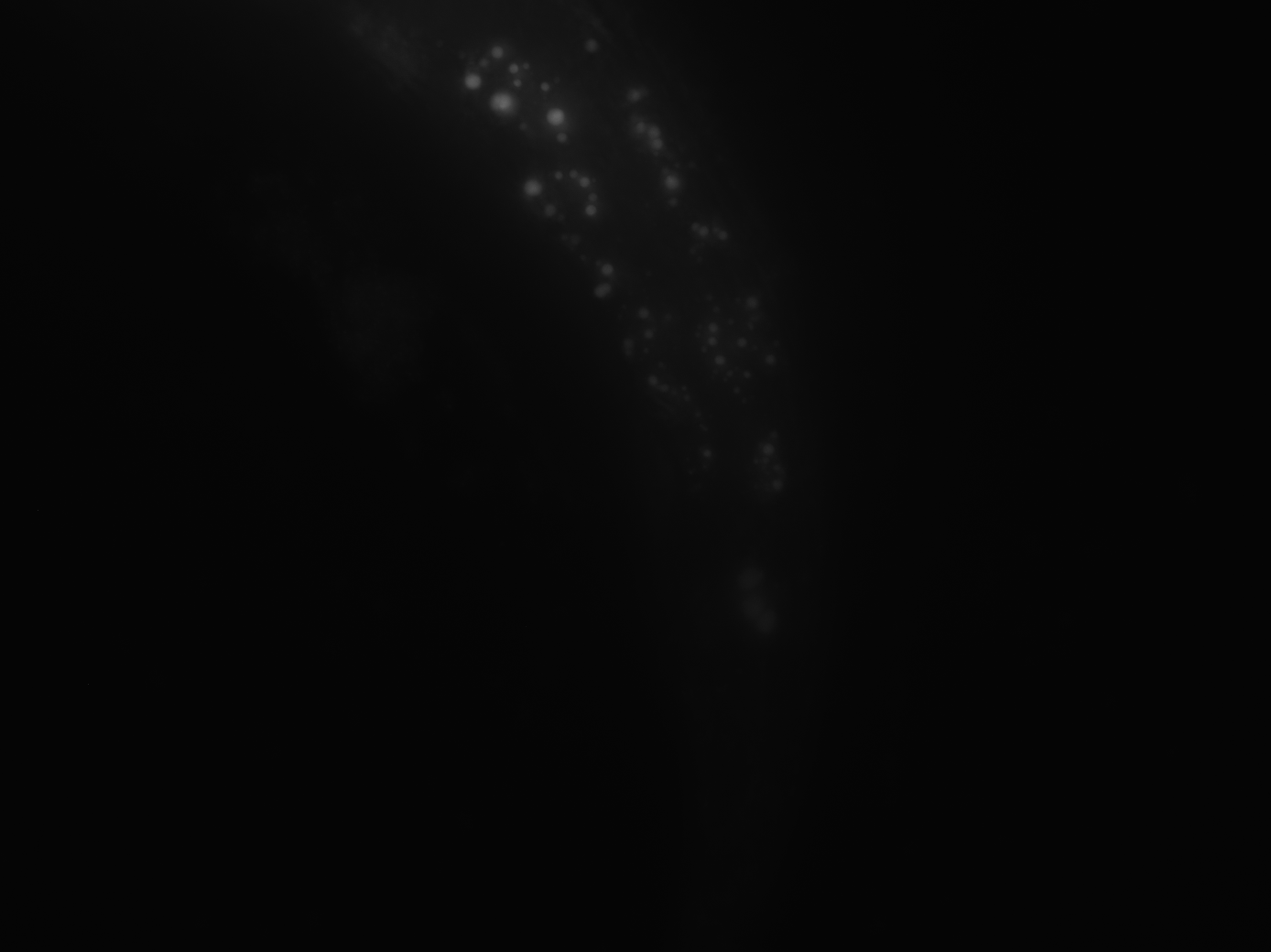

Supplement: Supplementary file 2 — Source data Fig. 1 [file 44319_2025_493_MOESM2_ESM.zip › Figure1/Fig1F/Experiment-11_tail_wildtype.tif_files/processed/MAX_Experiment-11green.tif_files-1.tif]

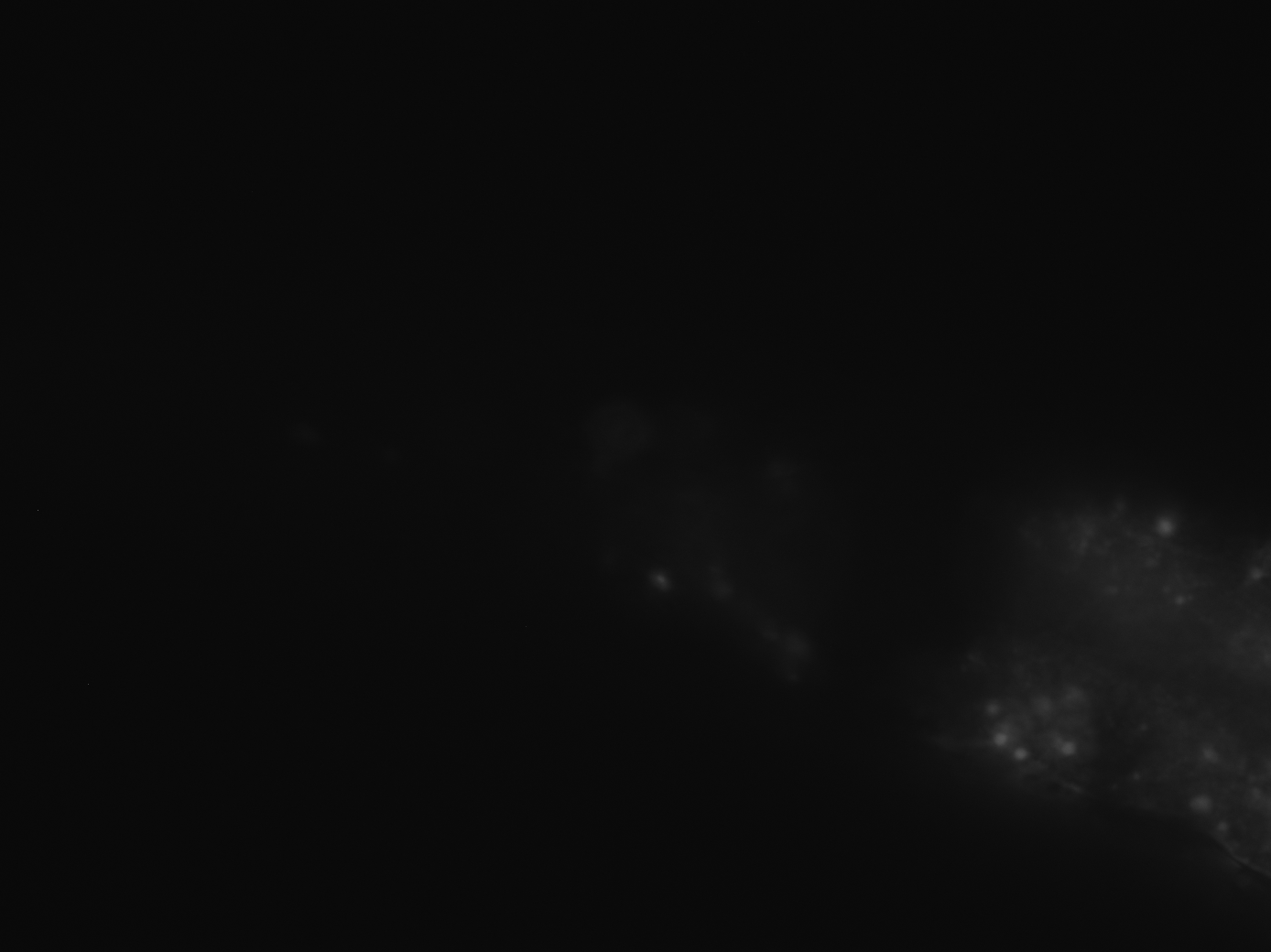

Supplement: Supplementary file 2 — Source data Fig. 1 [file 44319_2025_493_MOESM2_ESM.zip › Figure1/Fig1F/Experiment-06_NR_wildtype.tif_files/Experiment-06_z16c1x0-1388y0-1040.tif]

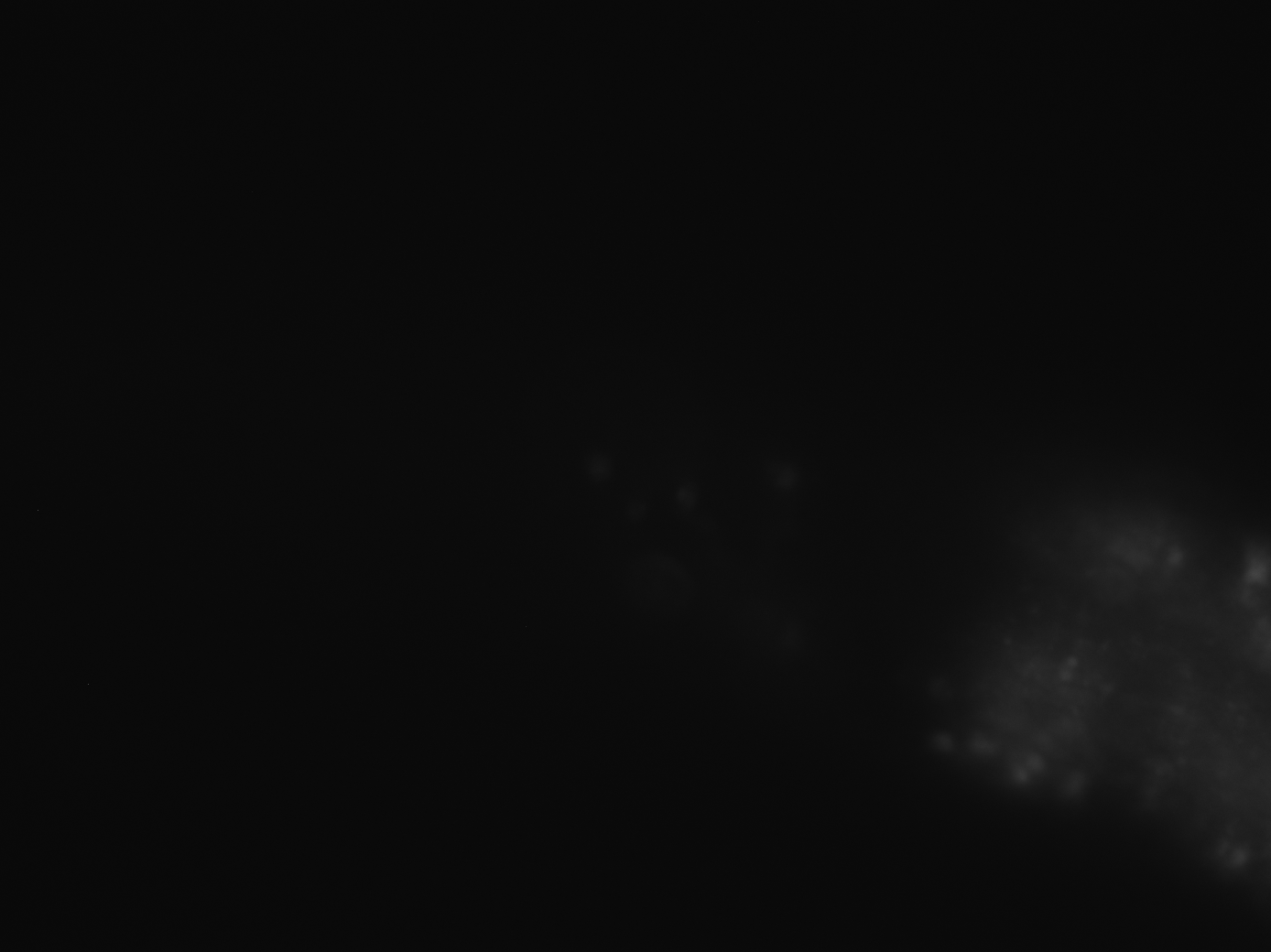

Supplement: Supplementary file 2 — Source data Fig. 1 [file 44319_2025_493_MOESM2_ESM.zip › Figure1/Fig1F/Experiment-06_NR_wildtype.tif_files/Experiment-06_z24c1x0-1388y0-1040.tif]

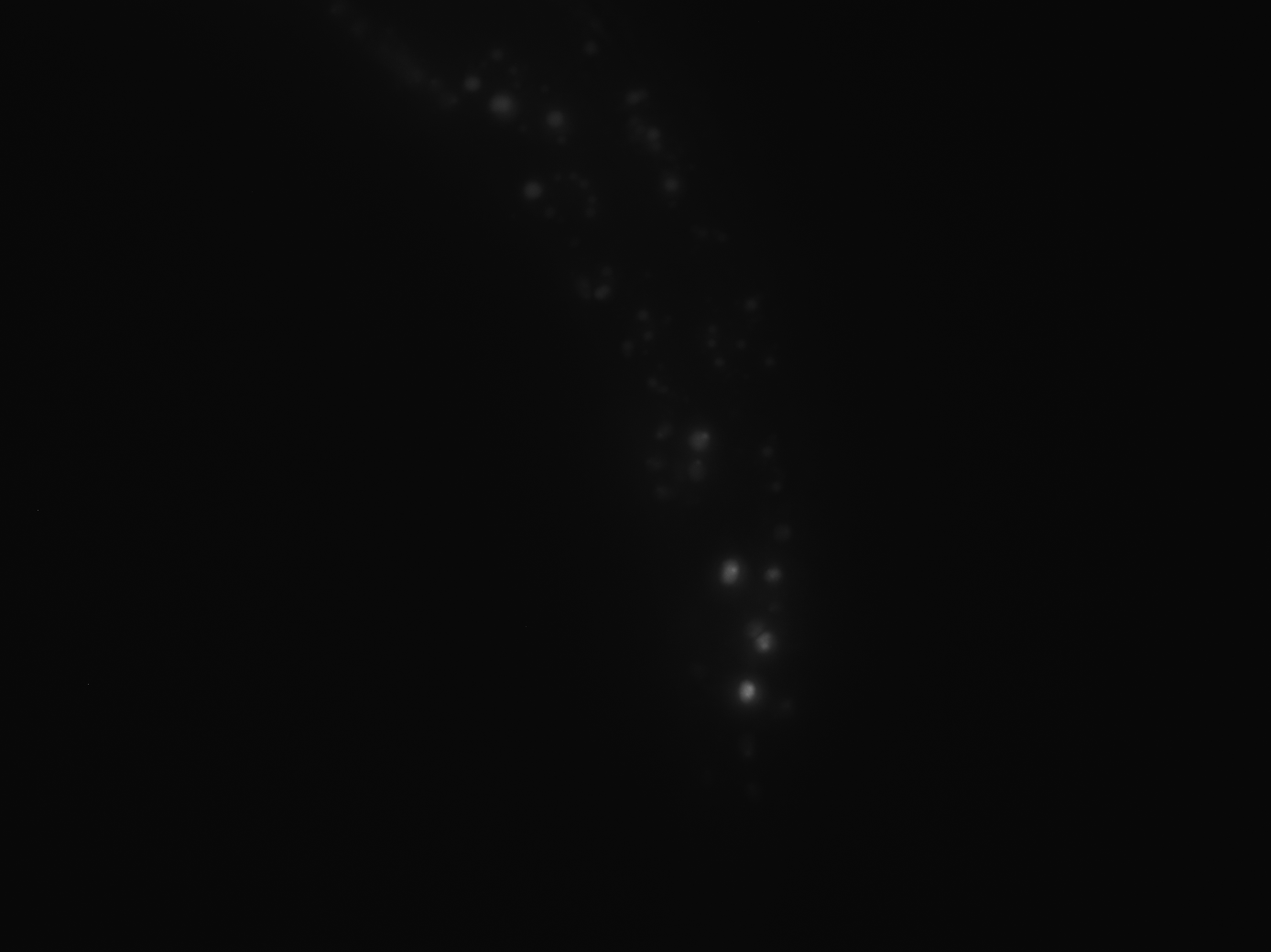

Supplement: Supplementary file 2 — Source data Fig. 1 [file 44319_2025_493_MOESM2_ESM.zip › Figure1/Fig1F/Experiment-11_tail_wildtype.tif_files/processed/MAX_Experiment-11red.tif_files.tif]

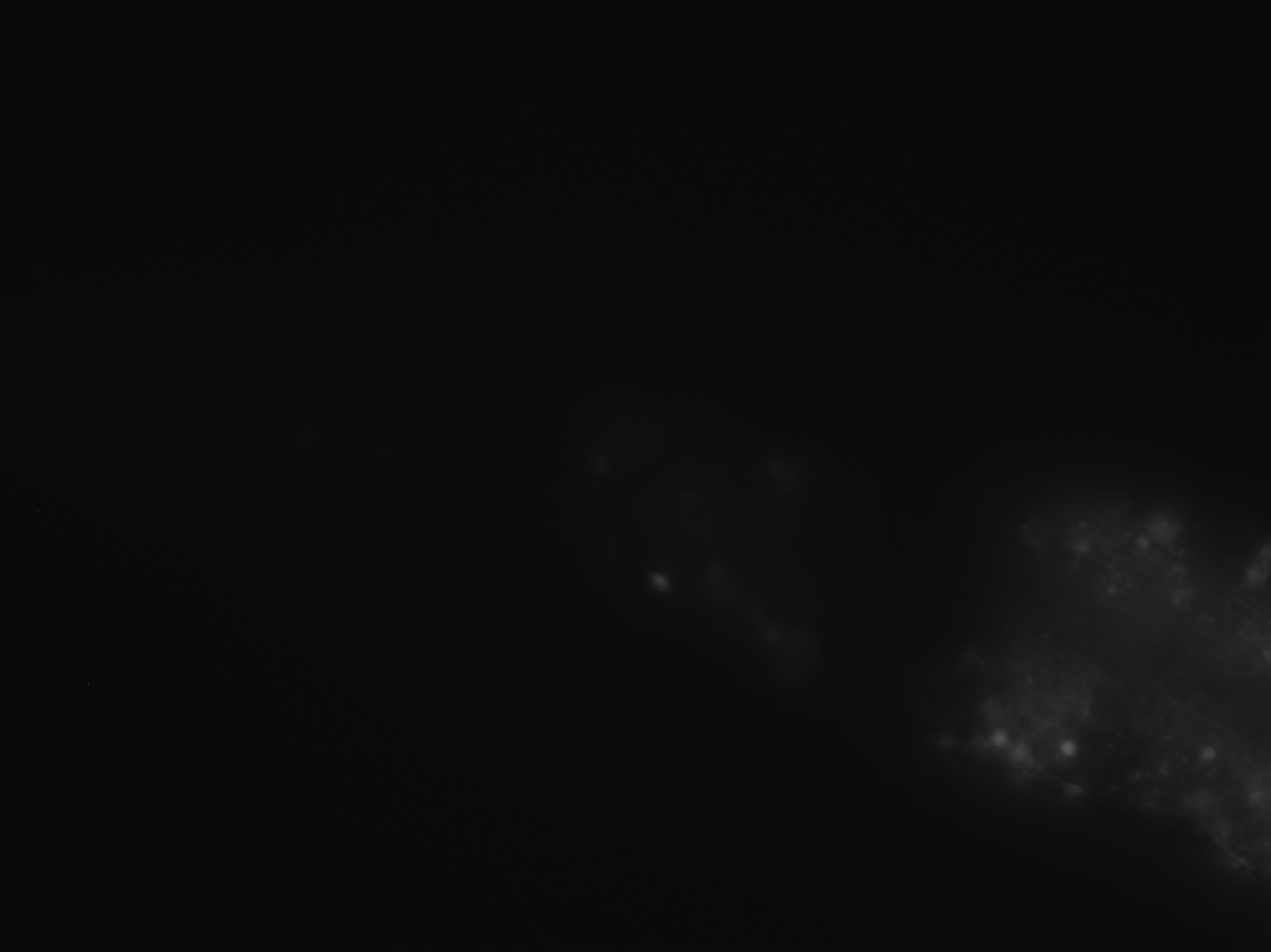

Supplement: Supplementary file 2 — Source data Fig. 1 [file 44319_2025_493_MOESM2_ESM.zip › Figure1/Fig1F/Experiment-06_NR_wildtype.tif_files/Experiment-06_z18c1x0-1388y0-1040.tif]

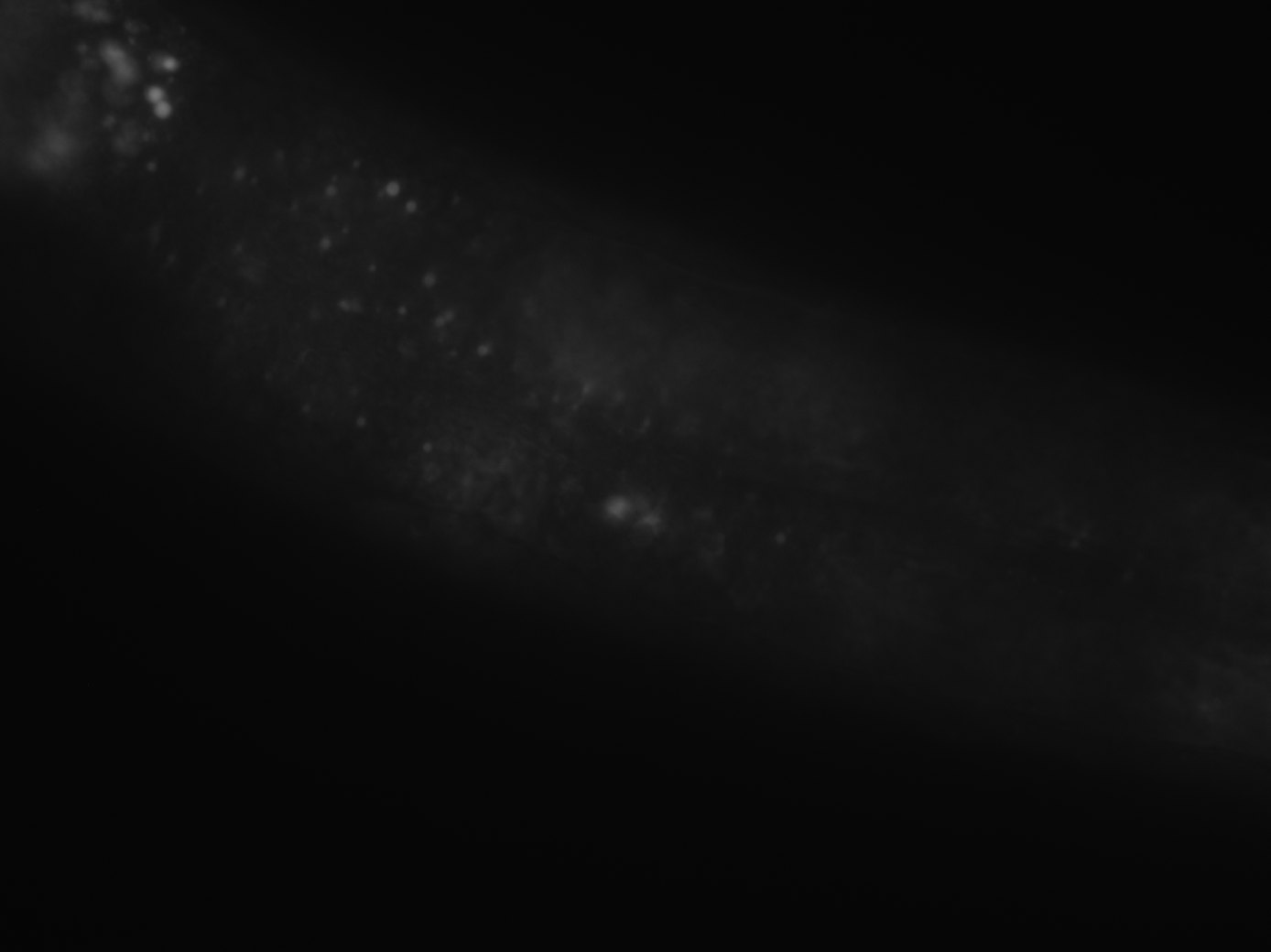

Supplement: Supplementary file 2 — Source data Fig. 1 [file 44319_2025_493_MOESM2_ESM.zip › Figure1/Fig1F/Experiment-04_VC_wiltype.tif_files/Experiment-04_z7c0x0-1388y0-1040.tif]

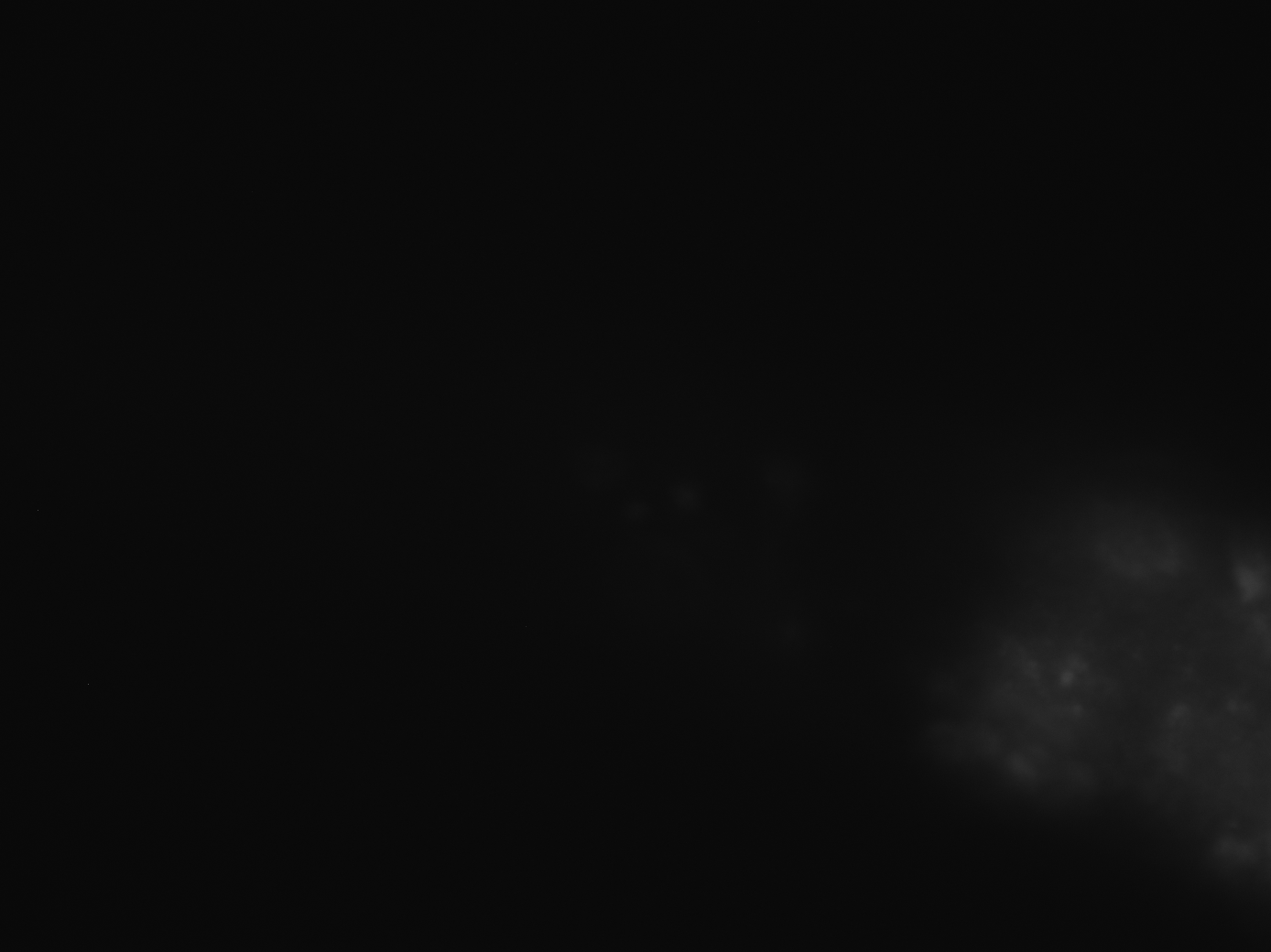

Supplement: Supplementary file 2 — Source data Fig. 1 [file 44319_2025_493_MOESM2_ESM.zip › Figure1/Fig1F/Experiment-06_NR_wildtype.tif_files/Experiment-06_z27c1x0-1388y0-1040.tif]

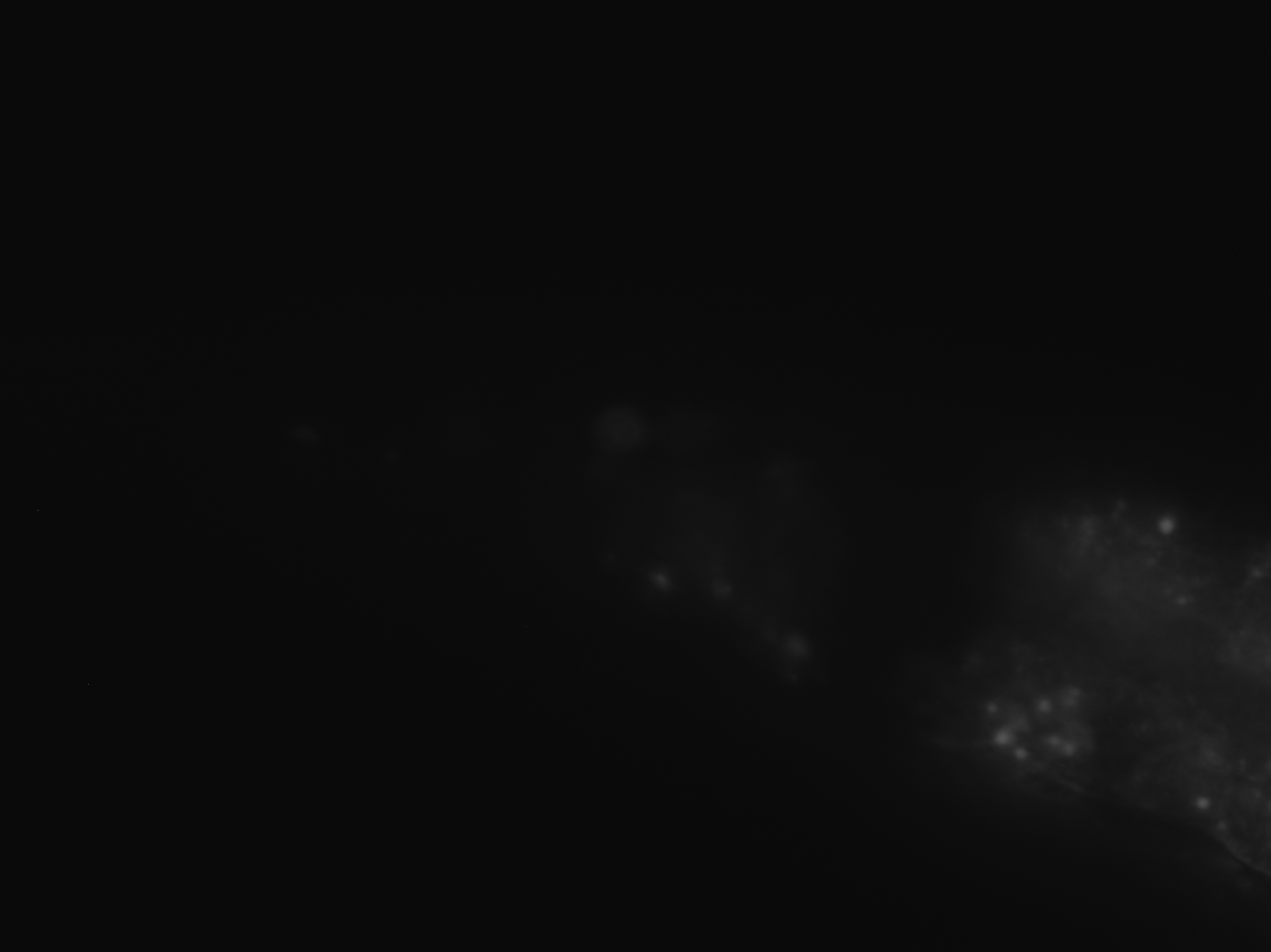

Supplement: Supplementary file 2 — Source data Fig. 1 [file 44319_2025_493_MOESM2_ESM.zip › Figure1/Fig1F/Experiment-06_NR_wildtype.tif_files/Experiment-06_z15c1x0-1388y0-1040.tif]

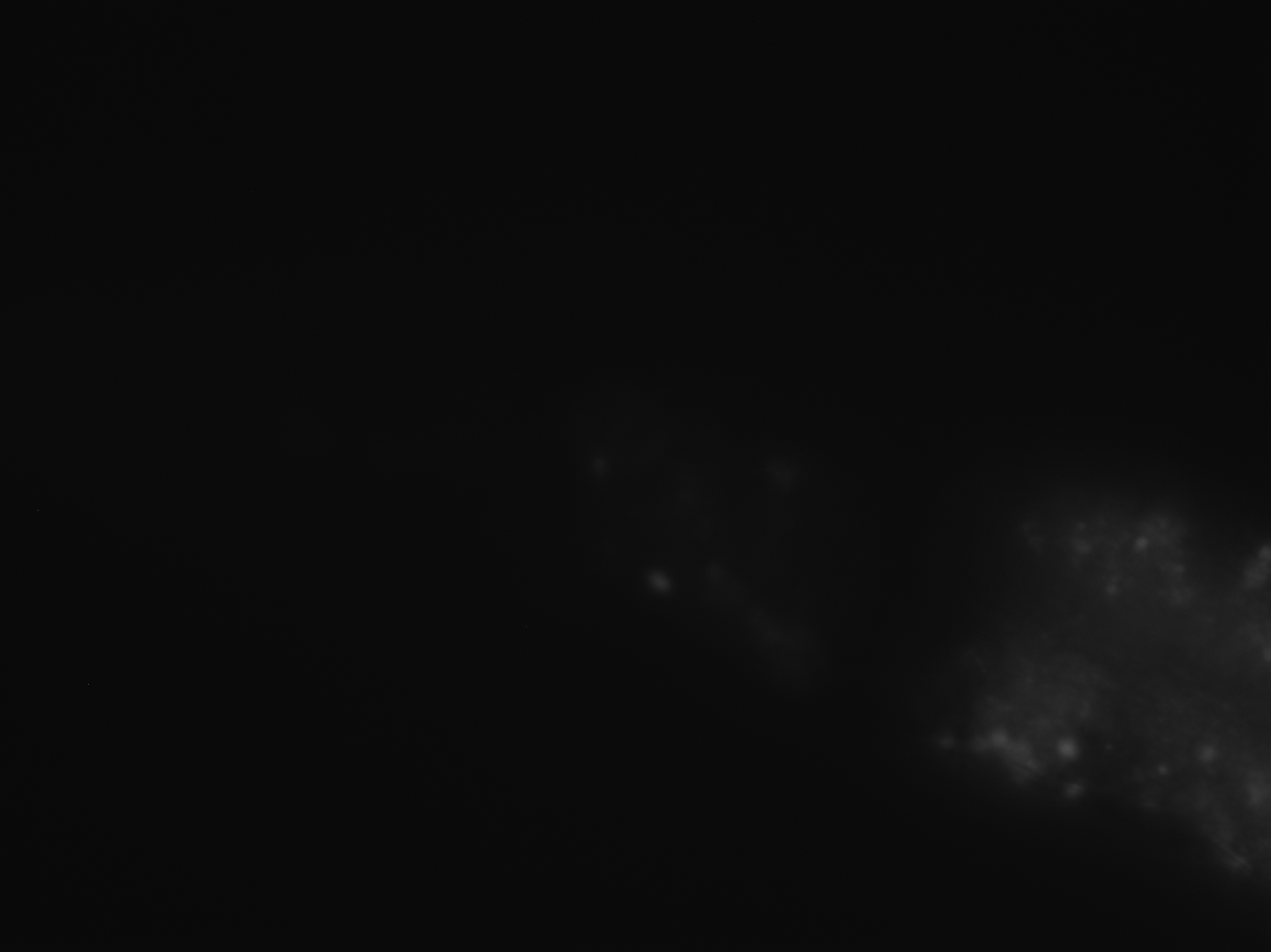

Supplement: Supplementary file 2 — Source data Fig. 1 [file 44319_2025_493_MOESM2_ESM.zip › Figure1/Fig1F/Experiment-06_NR_wildtype.tif_files/Experiment-06_z19c1x0-1388y0-1040.tif]

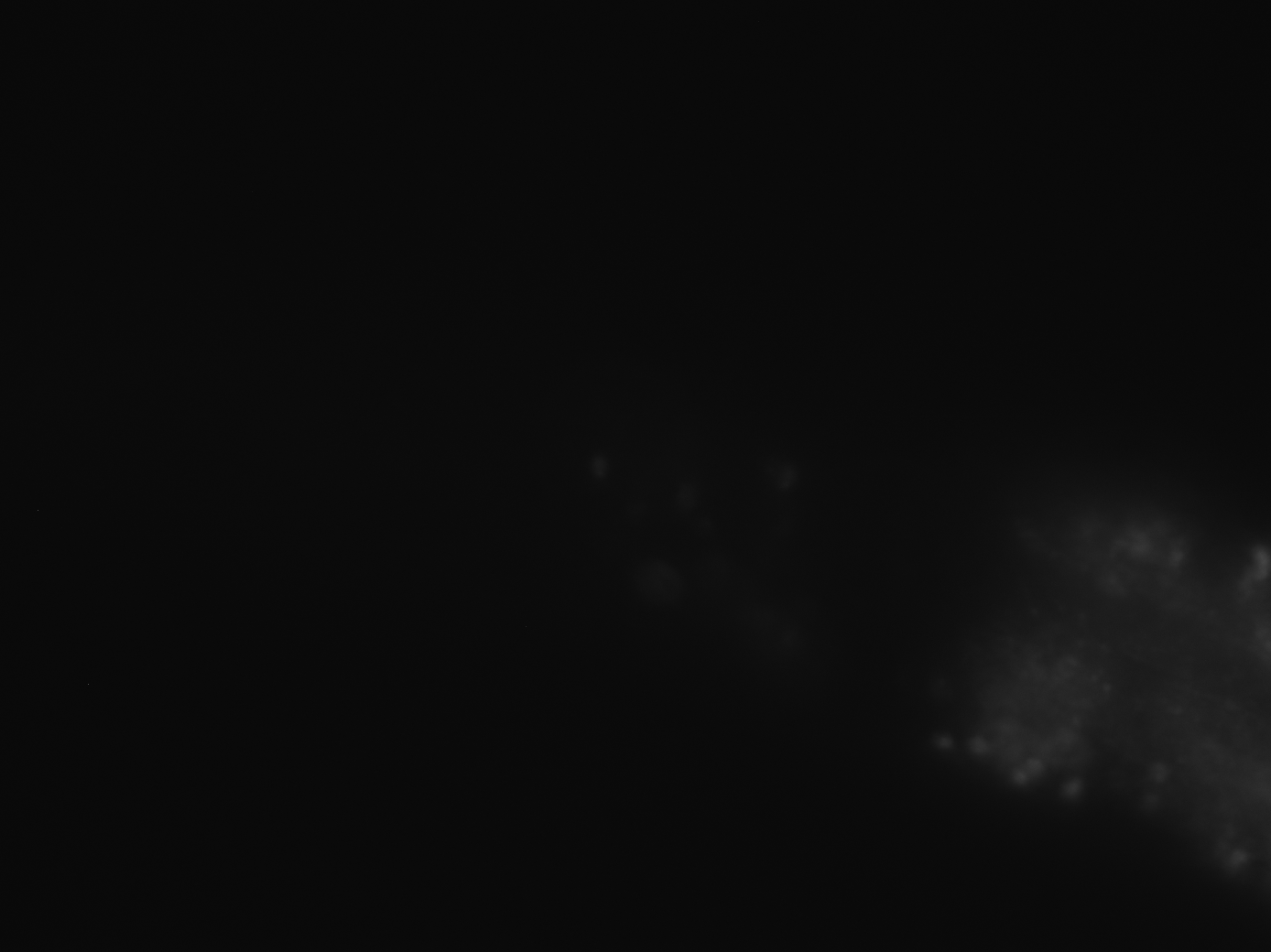

Supplement: Supplementary file 2 — Source data Fig. 1 [file 44319_2025_493_MOESM2_ESM.zip › Figure1/Fig1F/Experiment-06_NR_wildtype.tif_files/Experiment-06_z22c1x0-1388y0-1040.tif]

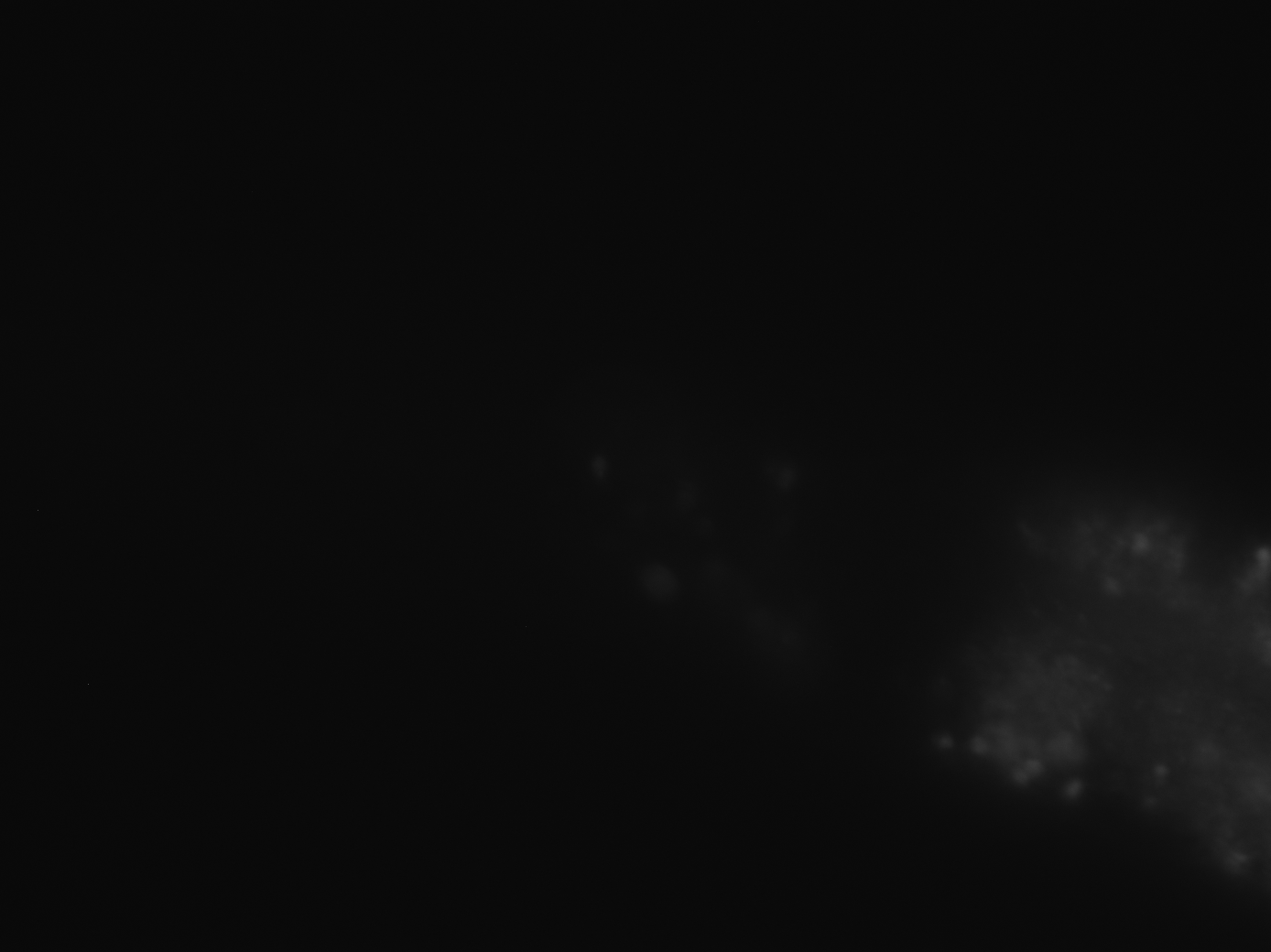

Supplement: Supplementary file 2 — Source data Fig. 1 [file 44319_2025_493_MOESM2_ESM.zip › Figure1/Fig1F/Experiment-06_NR_wildtype.tif_files/Experiment-06_z21c1x0-1388y0-1040.tif]

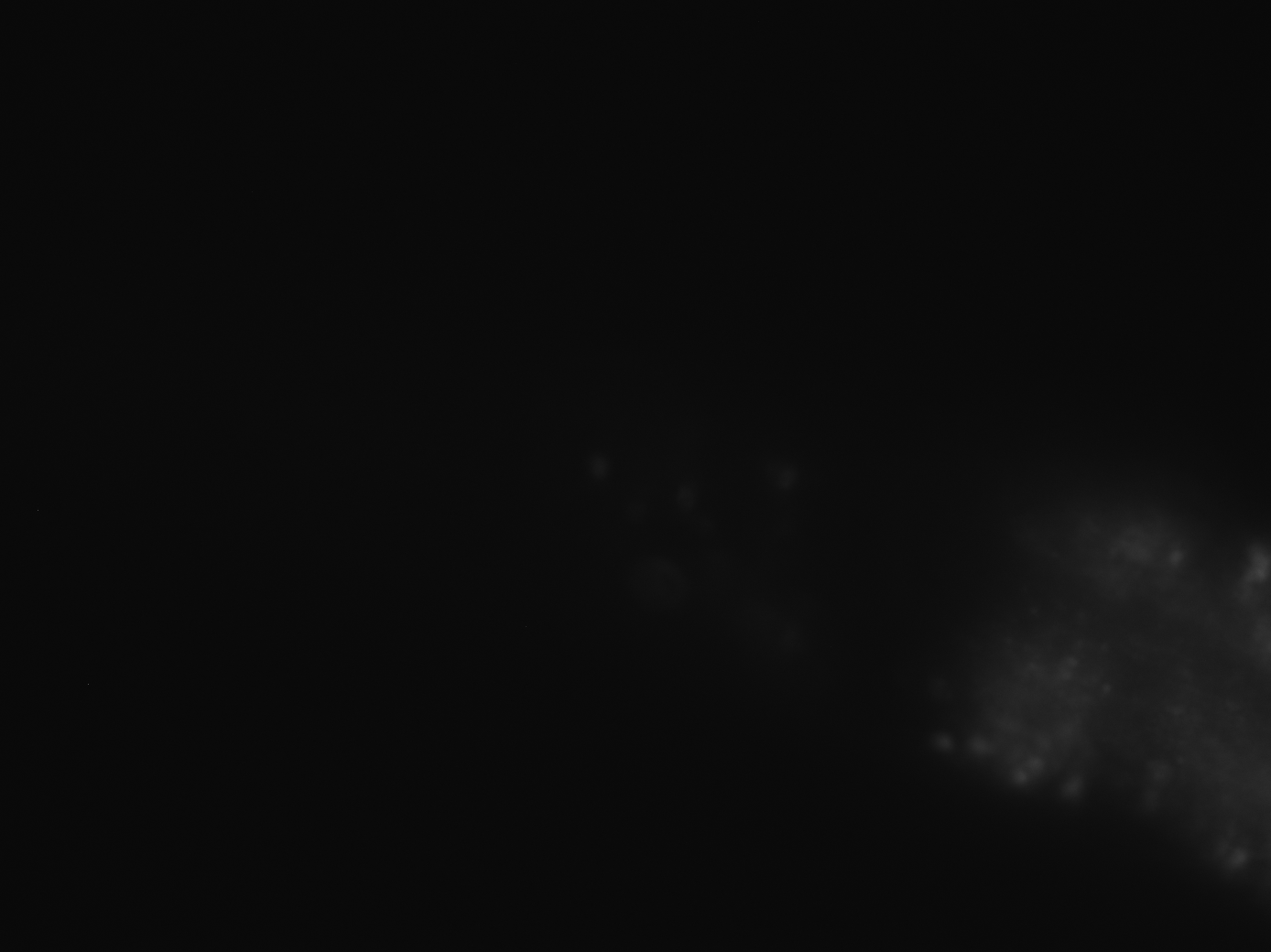

Supplement: Supplementary file 2 — Source data Fig. 1 [file 44319_2025_493_MOESM2_ESM.zip › Figure1/Fig1F/Experiment-06_NR_wildtype.tif_files/Experiment-06_z23c1x0-1388y0-1040.tif]

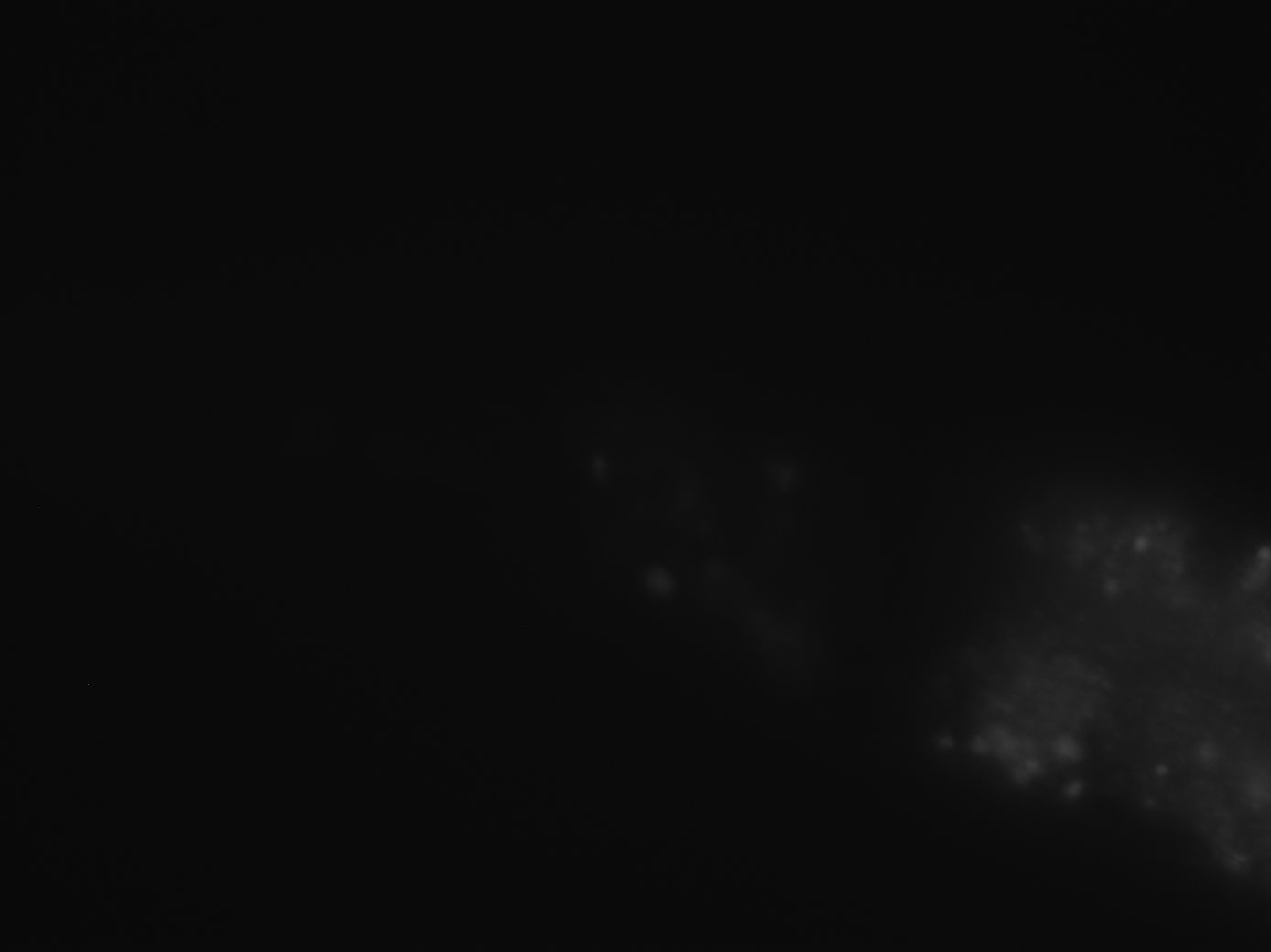

Supplement: Supplementary file 2 — Source data Fig. 1 [file 44319_2025_493_MOESM2_ESM.zip › Figure1/Fig1F/Experiment-06_NR_wildtype.tif_files/Experiment-06_z20c1x0-1388y0-1040.tif]

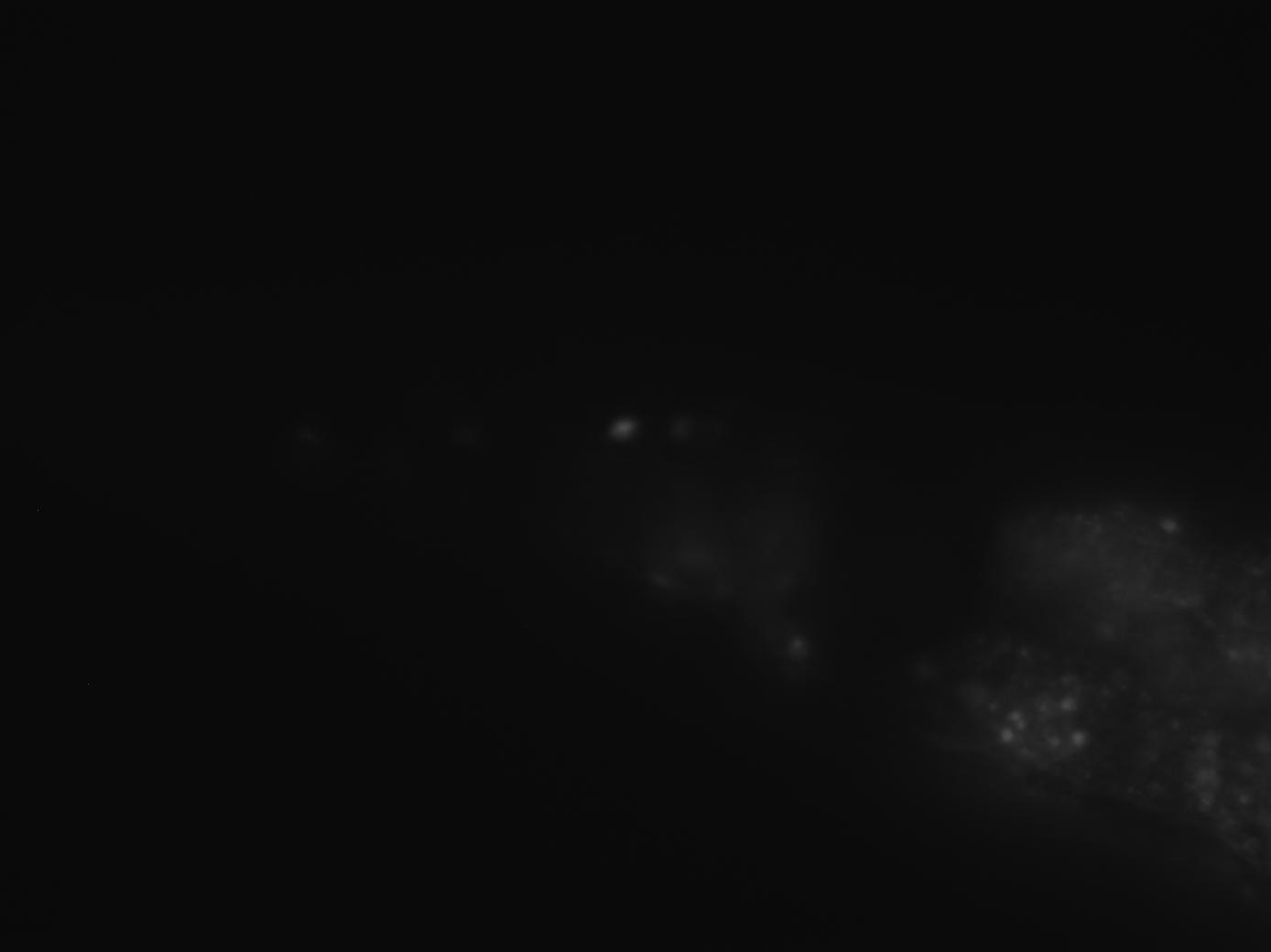

Supplement: Supplementary file 2 — Source data Fig. 1 [file 44319_2025_493_MOESM2_ESM.zip › Figure1/Fig1F/Experiment-06_NR_wildtype.tif_files/Experiment-06_z12c1x0-1388y0-1040.tif]

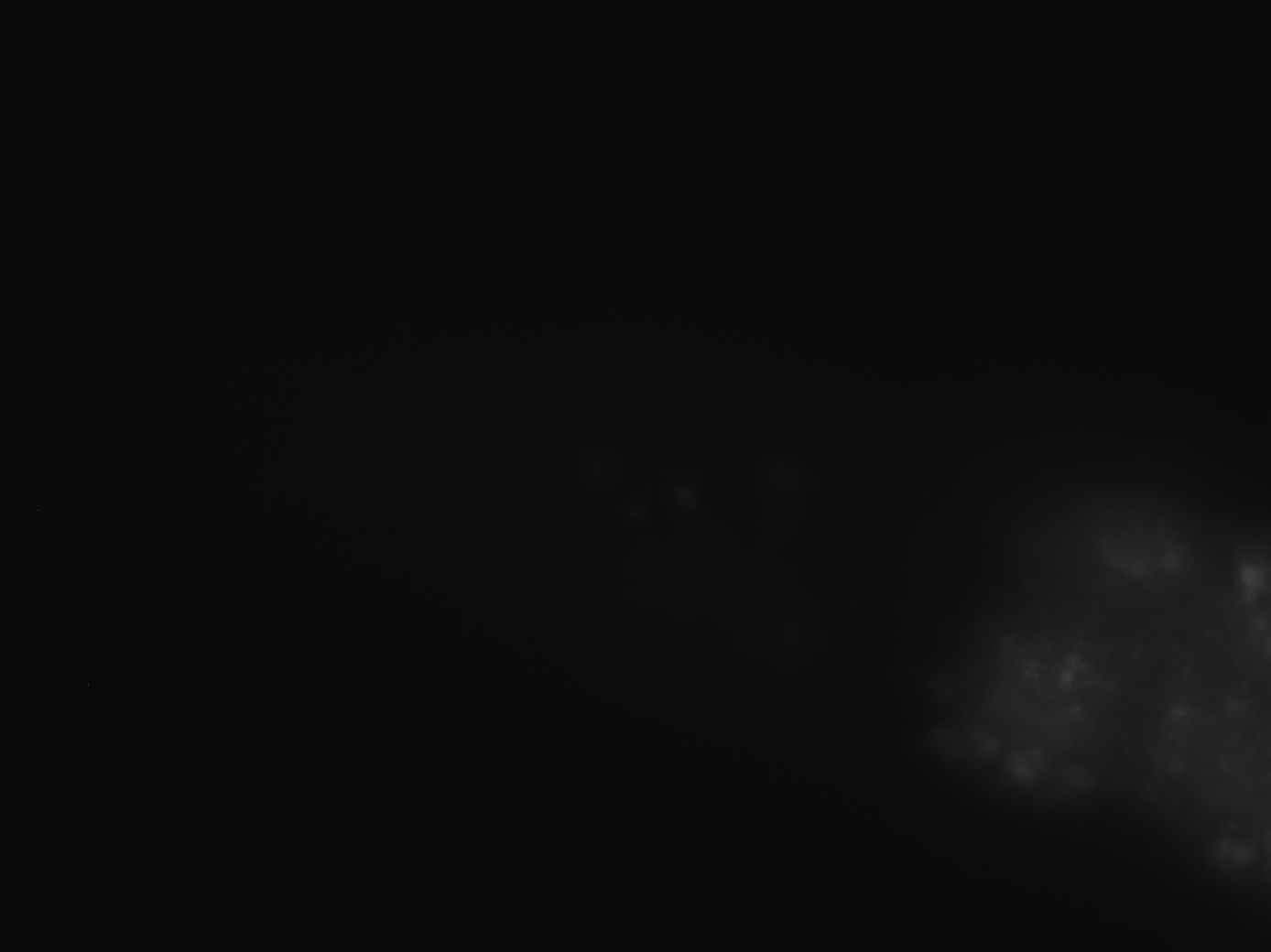

Supplement: Supplementary file 2 — Source data Fig. 1 [file 44319_2025_493_MOESM2_ESM.zip › Figure1/Fig1F/Experiment-06_NR_wildtype.tif_files/Experiment-06_z26c1x0-1388y0-1040.tif]

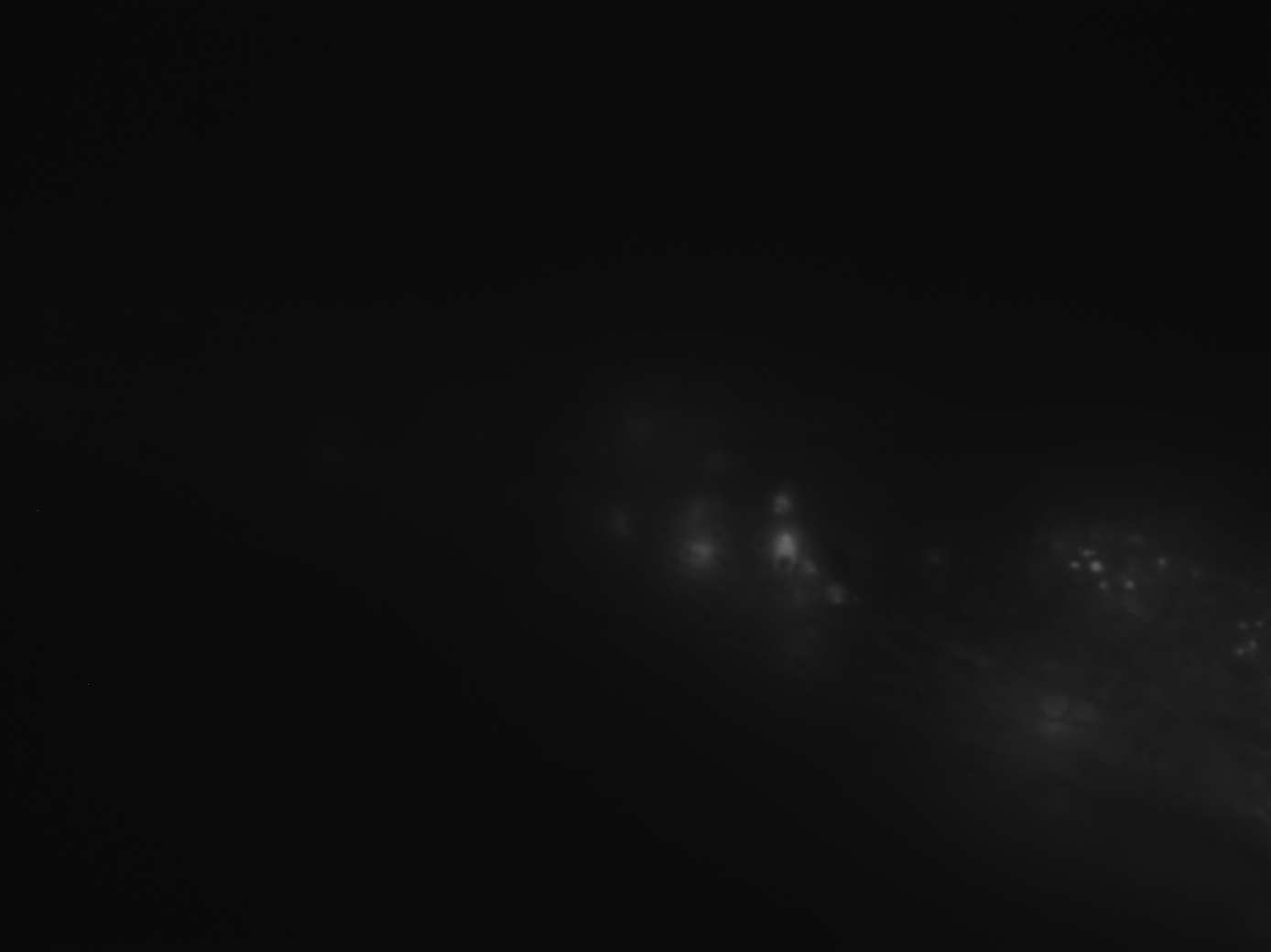

Supplement: Supplementary file 2 — Source data Fig. 1 [file 44319_2025_493_MOESM2_ESM.zip › Figure1/Fig1F/Experiment-06_NR_wildtype.tif_files/Experiment-06_z1c1x0-1388y0-1040.tif]

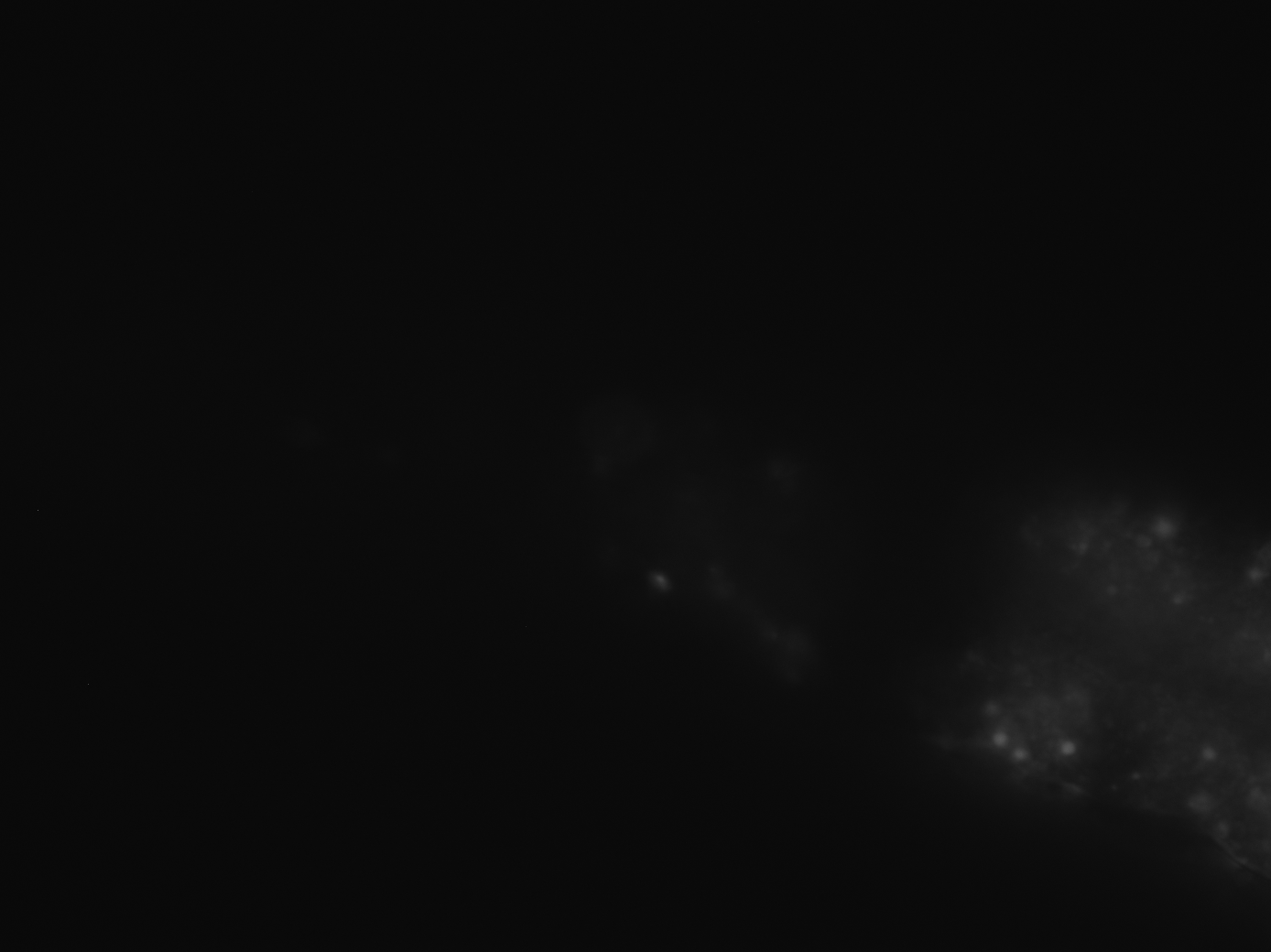

Supplement: Supplementary file 2 — Source data Fig. 1 [file 44319_2025_493_MOESM2_ESM.zip › Figure1/Fig1F/Experiment-06_NR_wildtype.tif_files/Experiment-06_z17c1x0-1388y0-1040.tif]

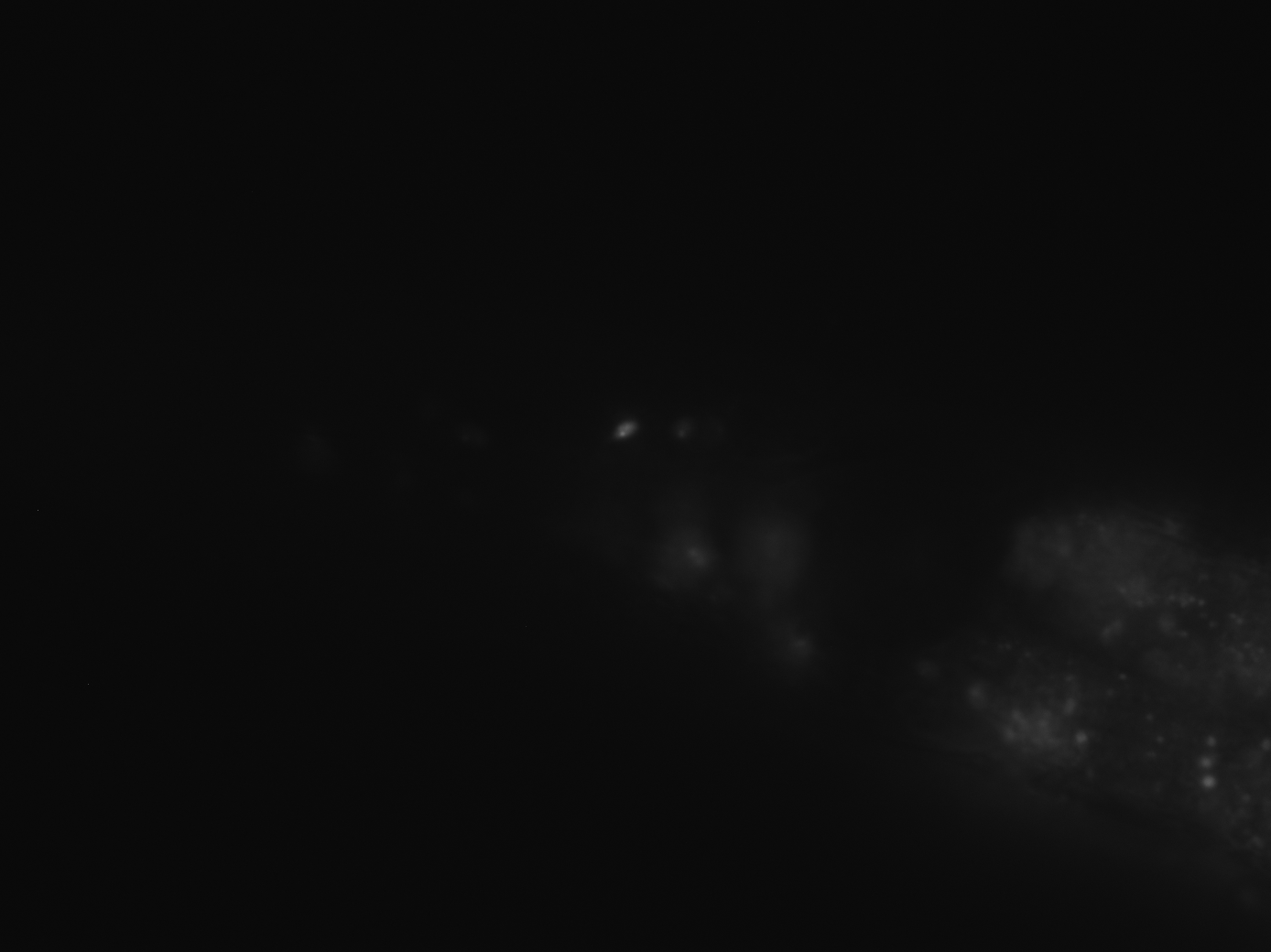

Supplement: Supplementary file 2 — Source data Fig. 1 [file 44319_2025_493_MOESM2_ESM.zip › Figure1/Fig1F/Experiment-06_NR_wildtype.tif_files/Experiment-06_z10c1x0-1388y0-1040.tif]

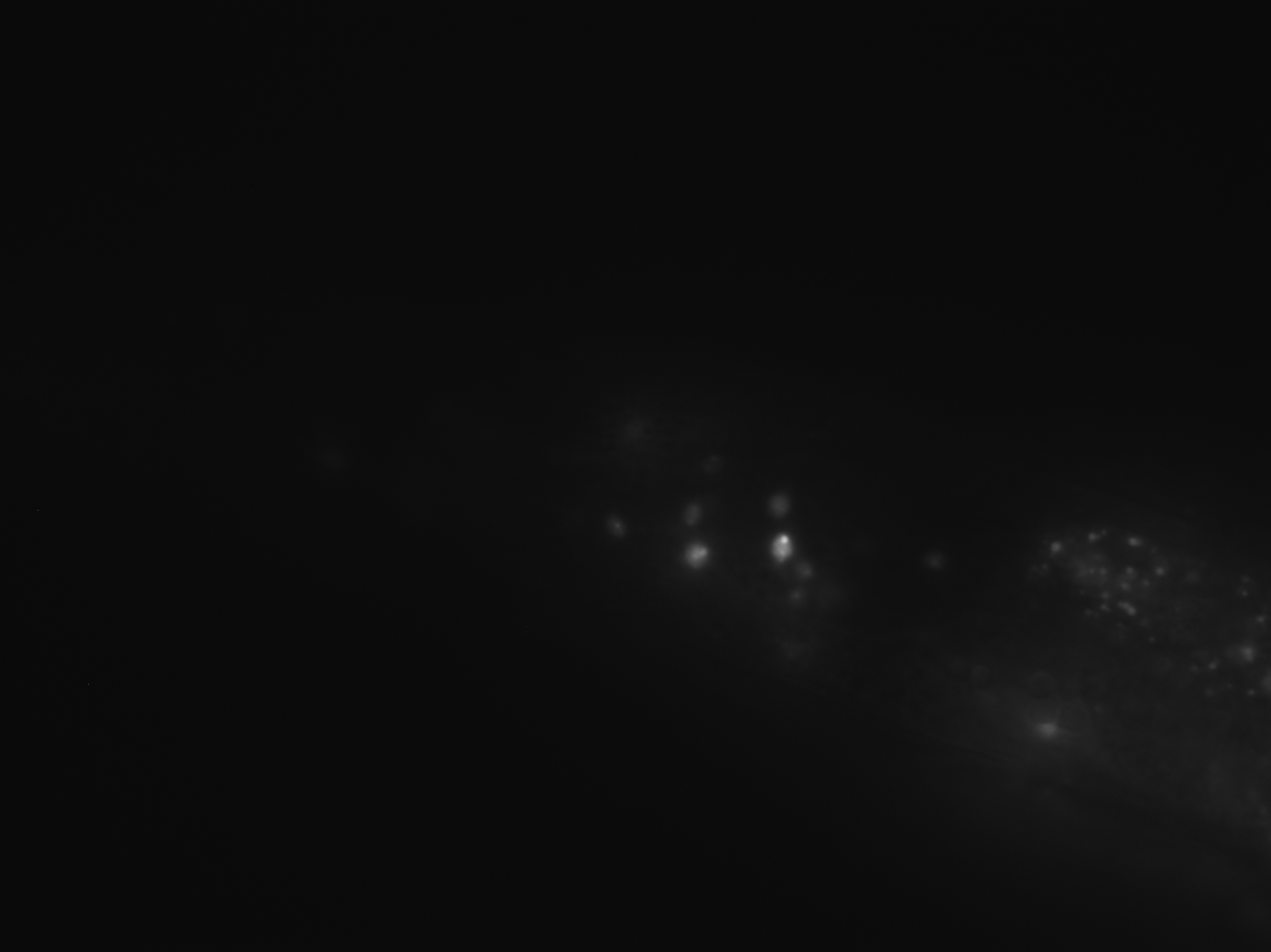

Supplement: Supplementary file 2 — Source data Fig. 1 [file 44319_2025_493_MOESM2_ESM.zip › Figure1/Fig1F/Experiment-06_NR_wildtype.tif_files/Experiment-06_z4c1x0-1388y0-1040.tif]

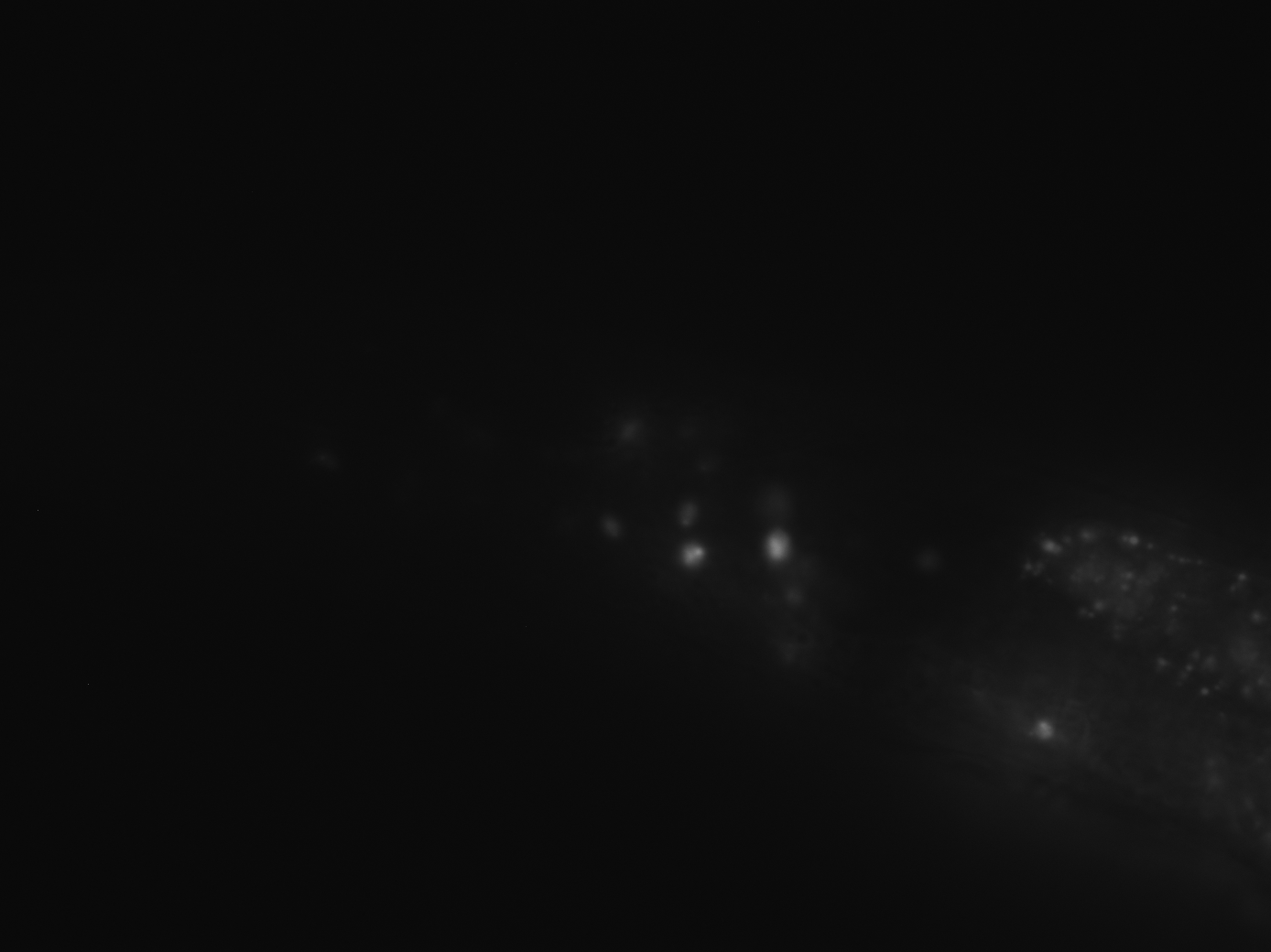

Supplement: Supplementary file 2 — Source data Fig. 1 [file 44319_2025_493_MOESM2_ESM.zip › Figure1/Fig1F/Experiment-06_NR_wildtype.tif_files/Experiment-06_z6c1x0-1388y0-1040.tif]

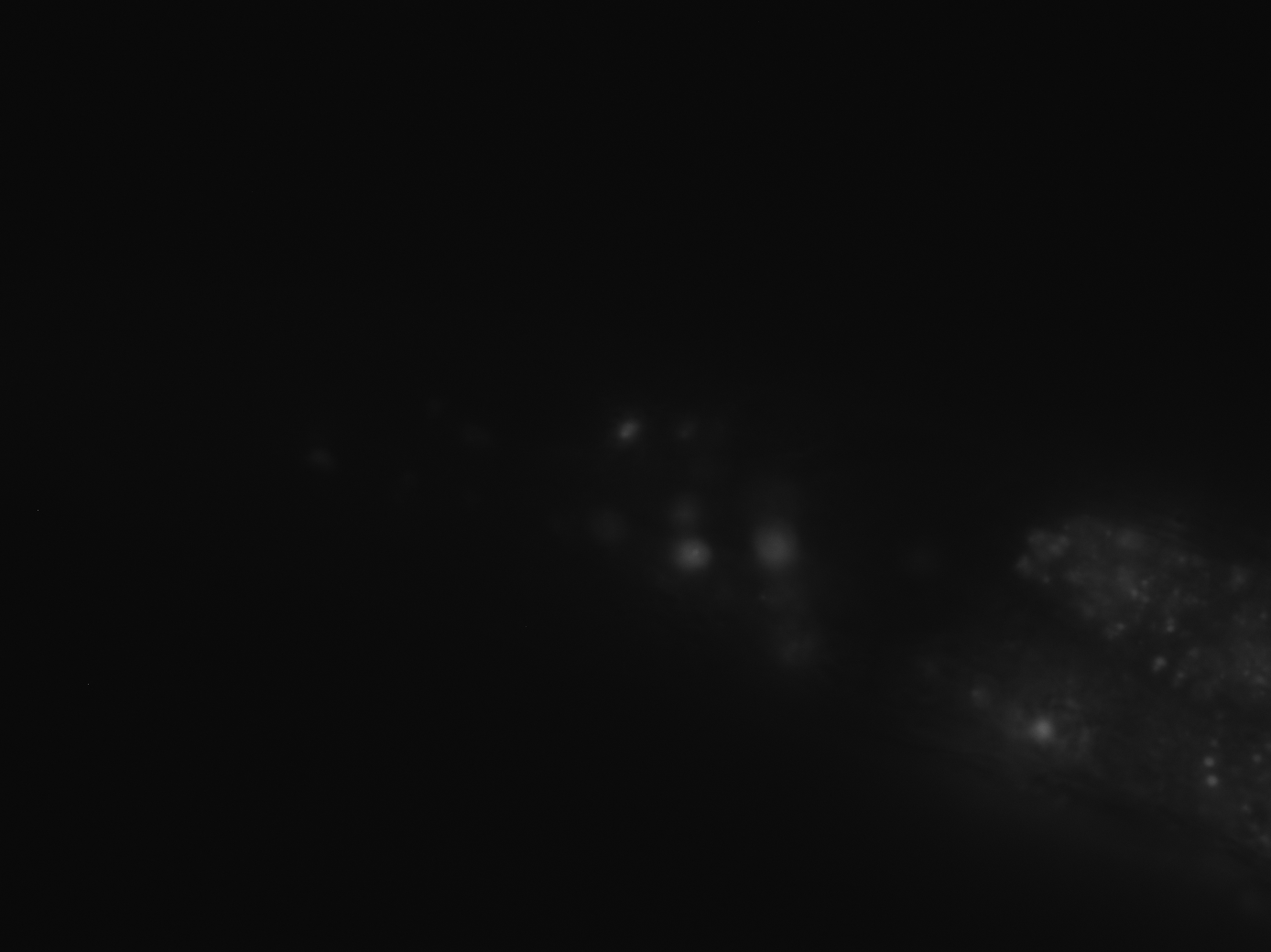

Supplement: Supplementary file 2 — Source data Fig. 1 [file 44319_2025_493_MOESM2_ESM.zip › Figure1/Fig1F/Experiment-06_NR_wildtype.tif_files/Experiment-06_z8c1x0-1388y0-1040.tif]

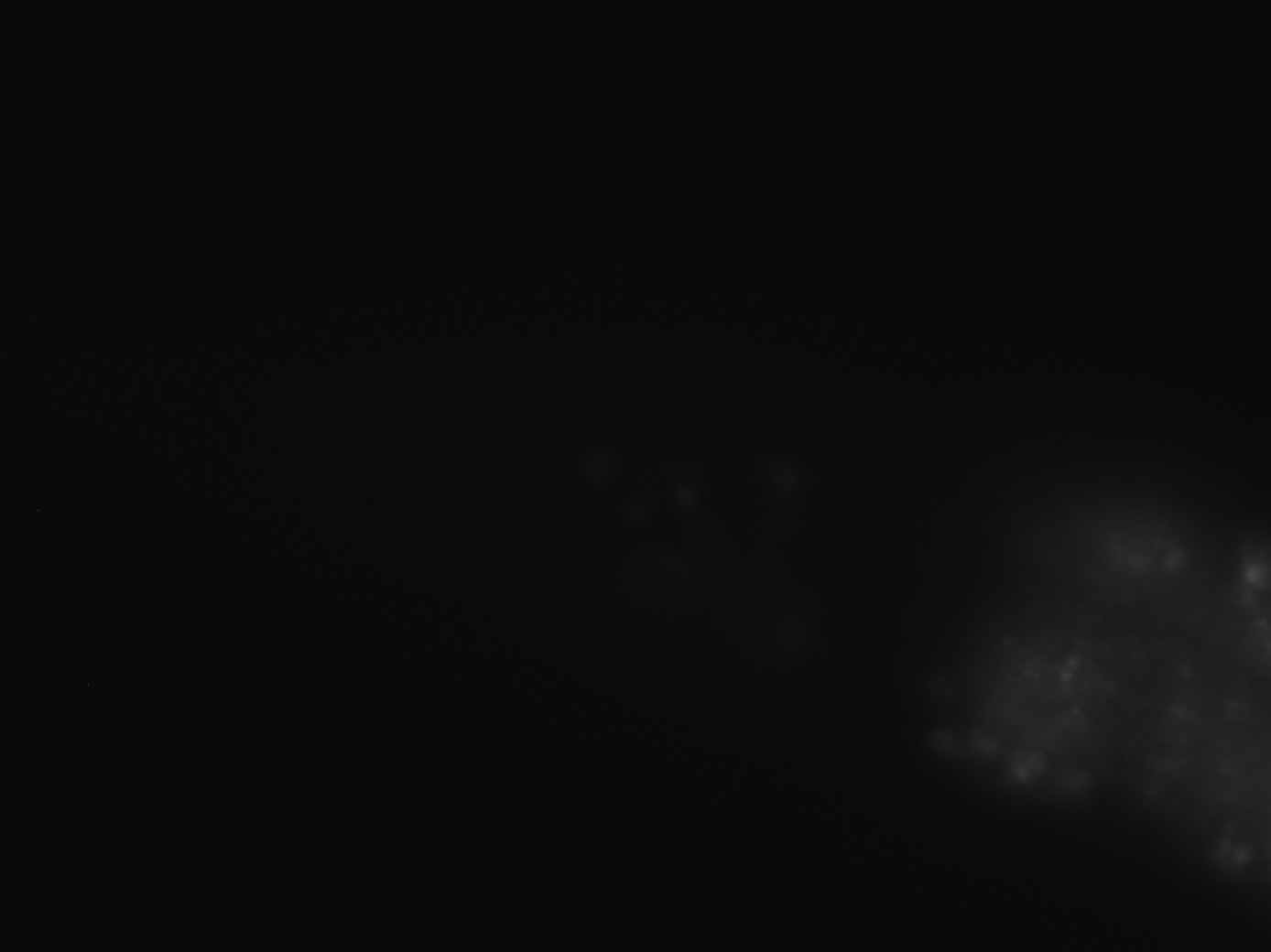

Supplement: Supplementary file 2 — Source data Fig. 1 [file 44319_2025_493_MOESM2_ESM.zip › Figure1/Fig1F/Experiment-06_NR_wildtype.tif_files/Experiment-06_z25c1x0-1388y0-1040.tif]

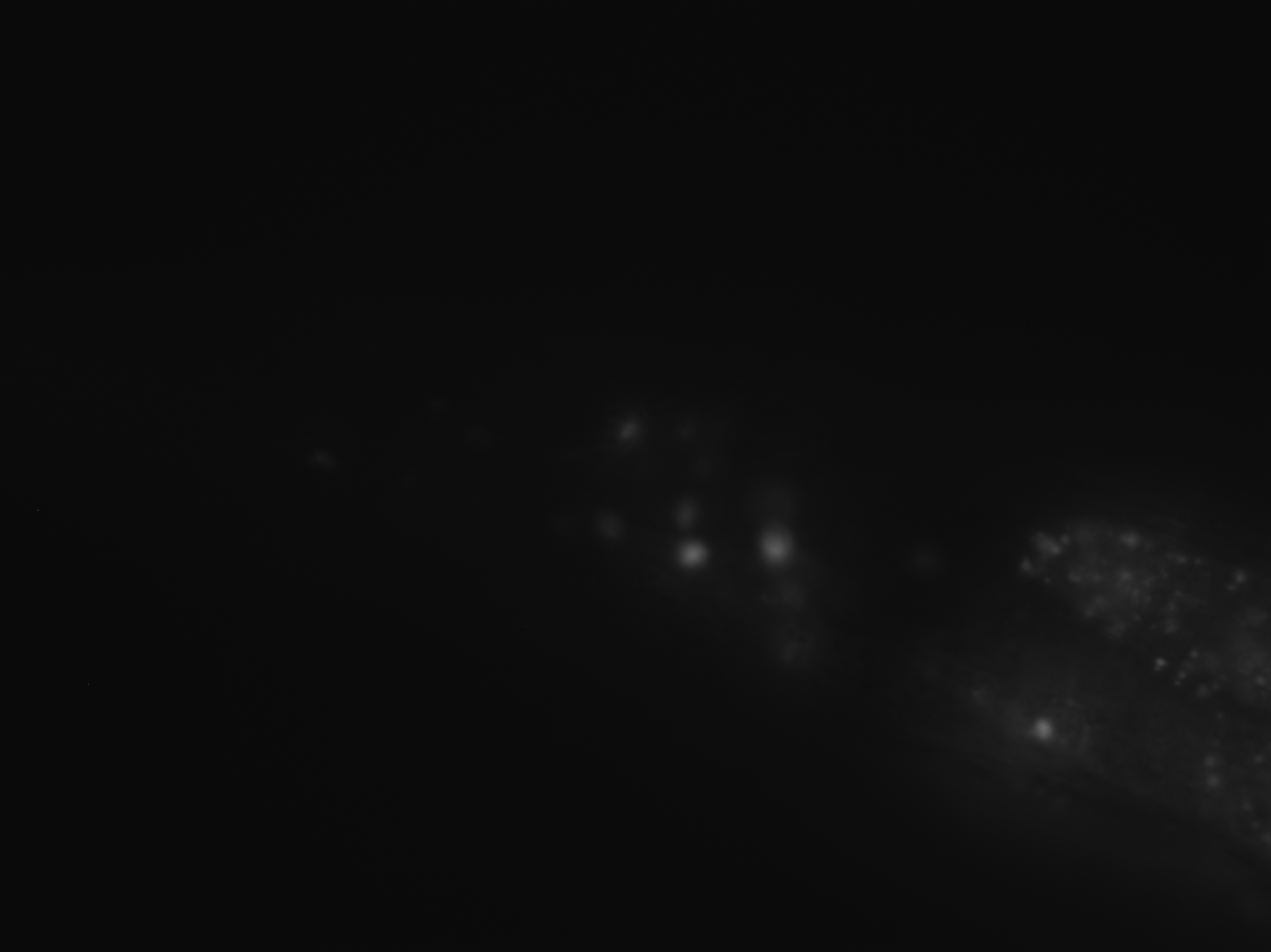

Supplement: Supplementary file 2 — Source data Fig. 1 [file 44319_2025_493_MOESM2_ESM.zip › Figure1/Fig1F/Experiment-06_NR_wildtype.tif_files/Experiment-06_z7c1x0-1388y0-1040.tif]

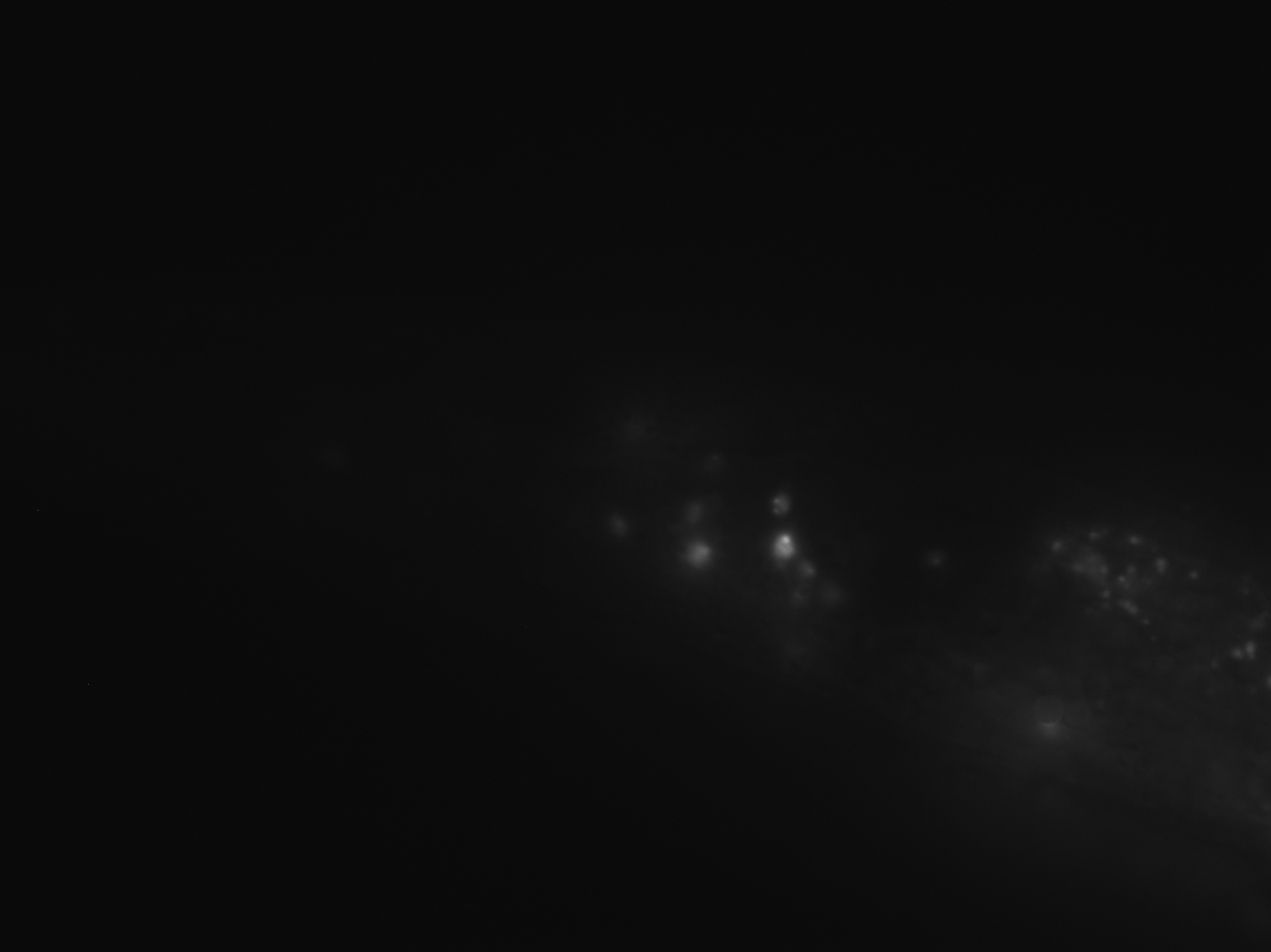

Supplement: Supplementary file 2 — Source data Fig. 1 [file 44319_2025_493_MOESM2_ESM.zip › Figure1/Fig1F/Experiment-06_NR_wildtype.tif_files/Experiment-06_z3c1x0-1388y0-1040.tif]

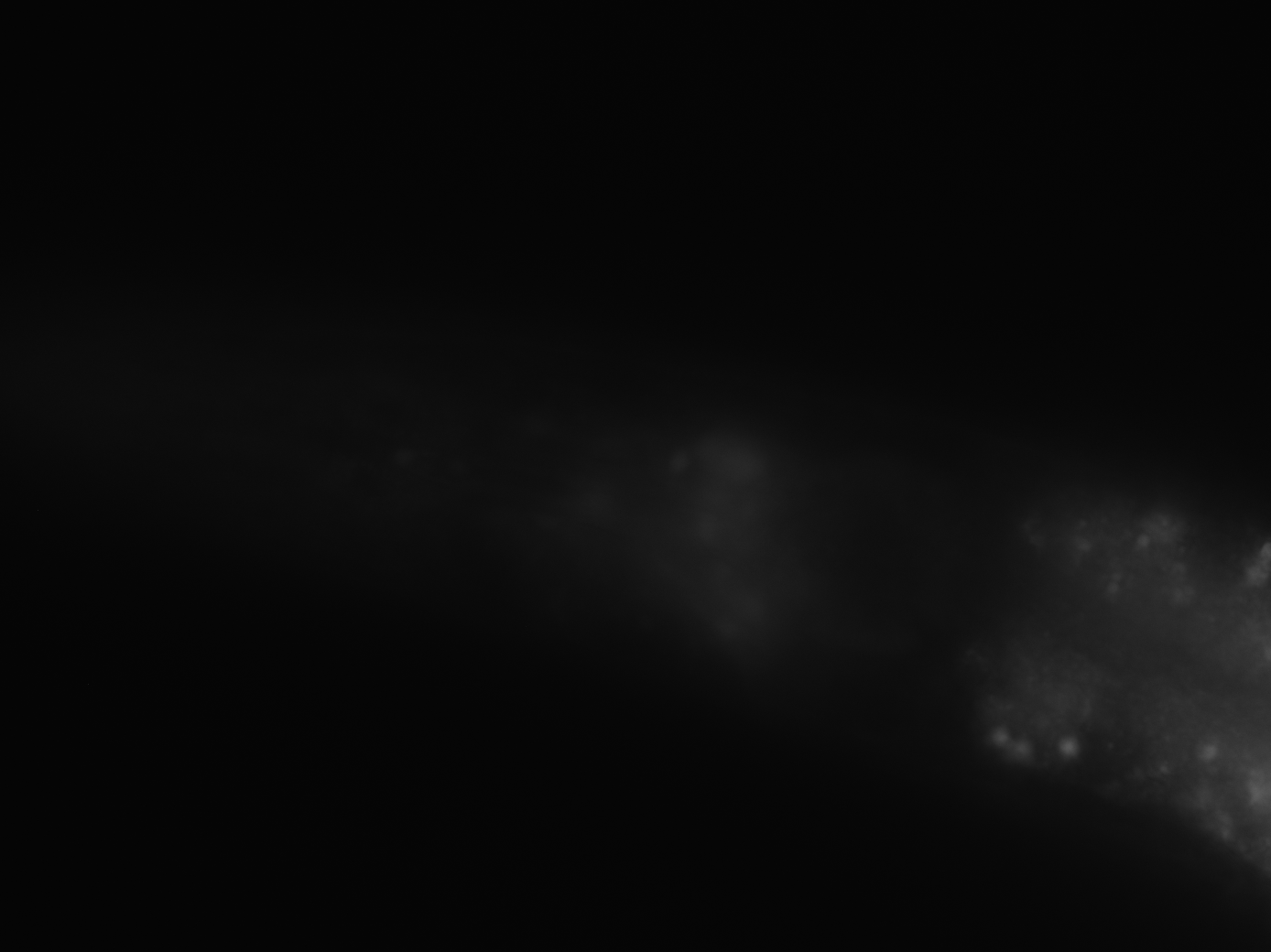

Supplement: Supplementary file 2 — Source data Fig. 1 [file 44319_2025_493_MOESM2_ESM.zip › Figure1/Fig1F/Experiment-06_NR_wildtype.tif_files/Experiment-06_z19c0x0-1388y0-1040.tif]

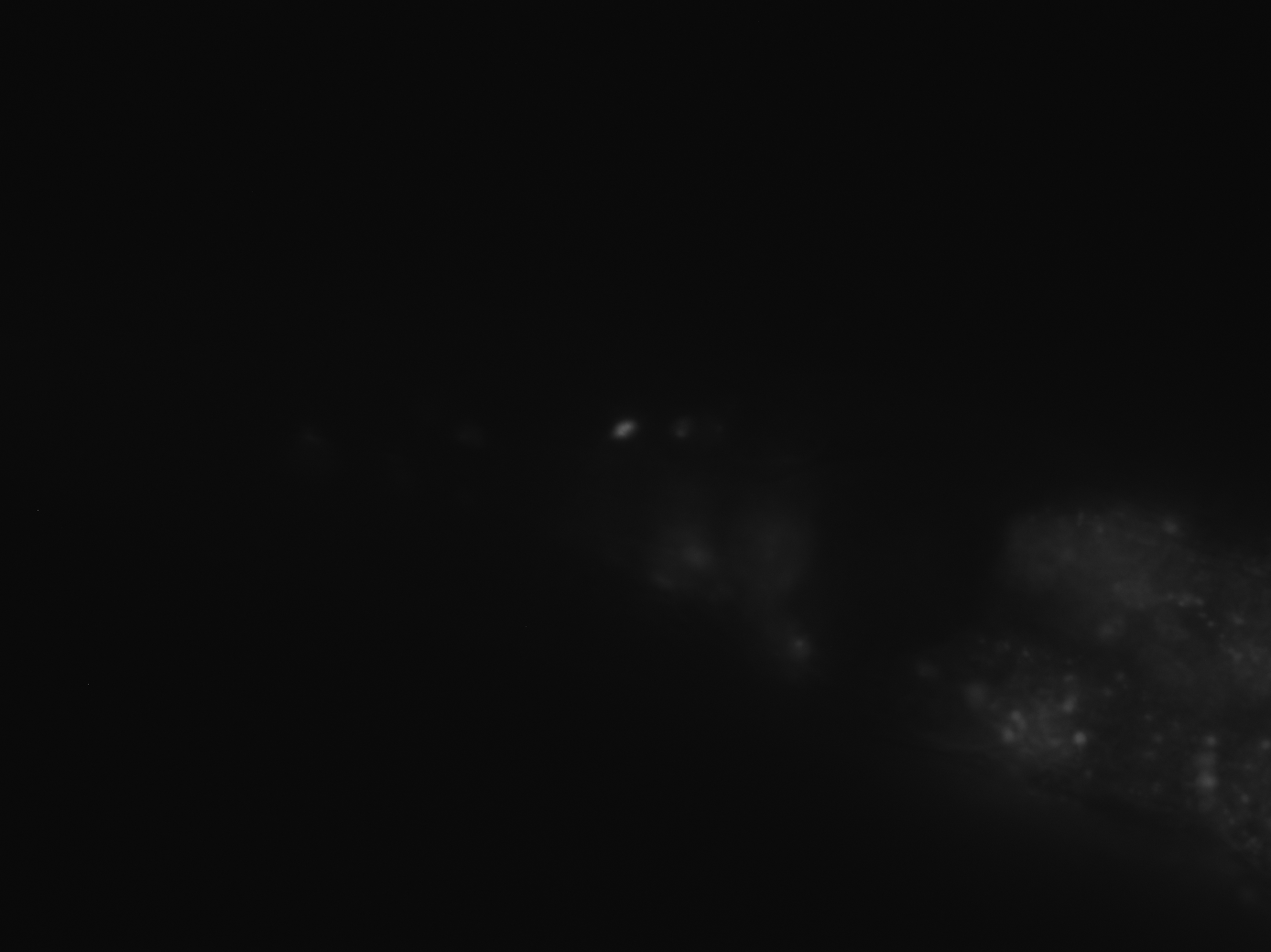

Supplement: Supplementary file 2 — Source data Fig. 1 [file 44319_2025_493_MOESM2_ESM.zip › Figure1/Fig1F/Experiment-06_NR_wildtype.tif_files/Experiment-06_z11c1x0-1388y0-1040.tif]

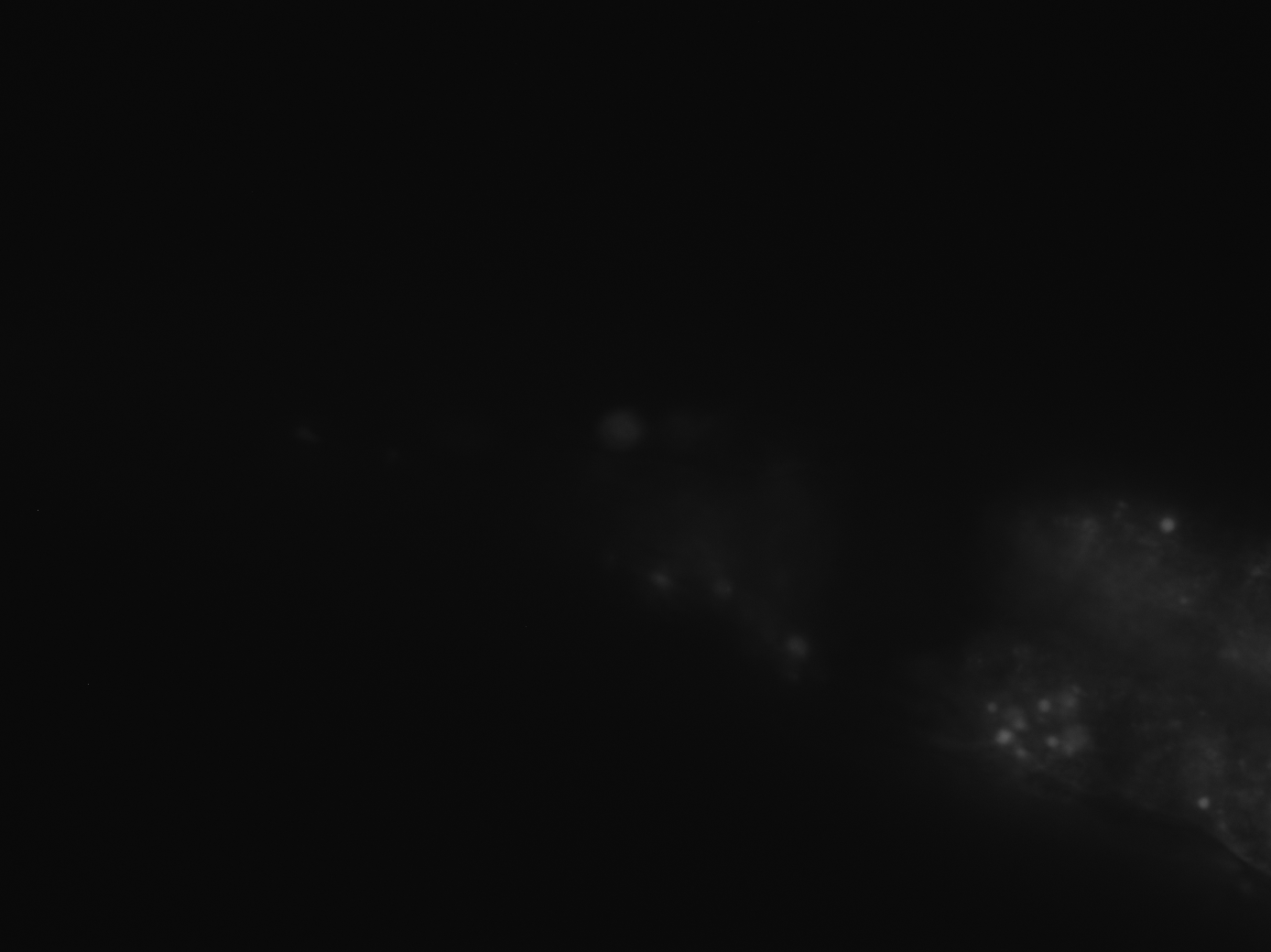

Supplement: Supplementary file 2 — Source data Fig. 1 [file 44319_2025_493_MOESM2_ESM.zip › Figure1/Fig1F/Experiment-06_NR_wildtype.tif_files/Experiment-06_z14c1x0-1388y0-1040.tif]

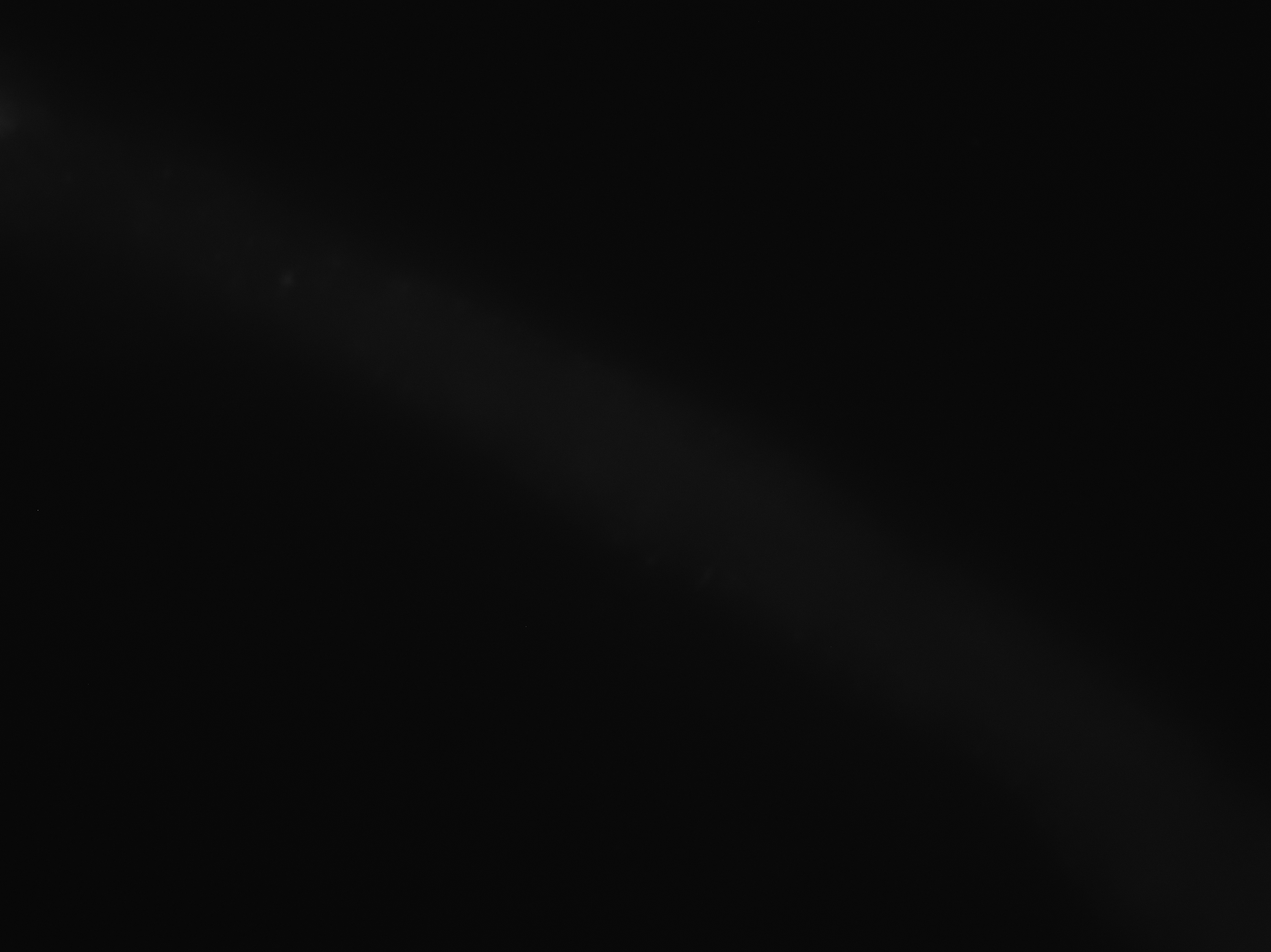

Supplement: Supplementary file 3 — Source data Fig. 2 [file 44319_2025_493_MOESM3_ESM.zip › Figure2/Fig2D/Experiment-383_VC_exc7_mbl1.tif_files/Experiment-383_z14c1x0-1388y0-1040.tif]

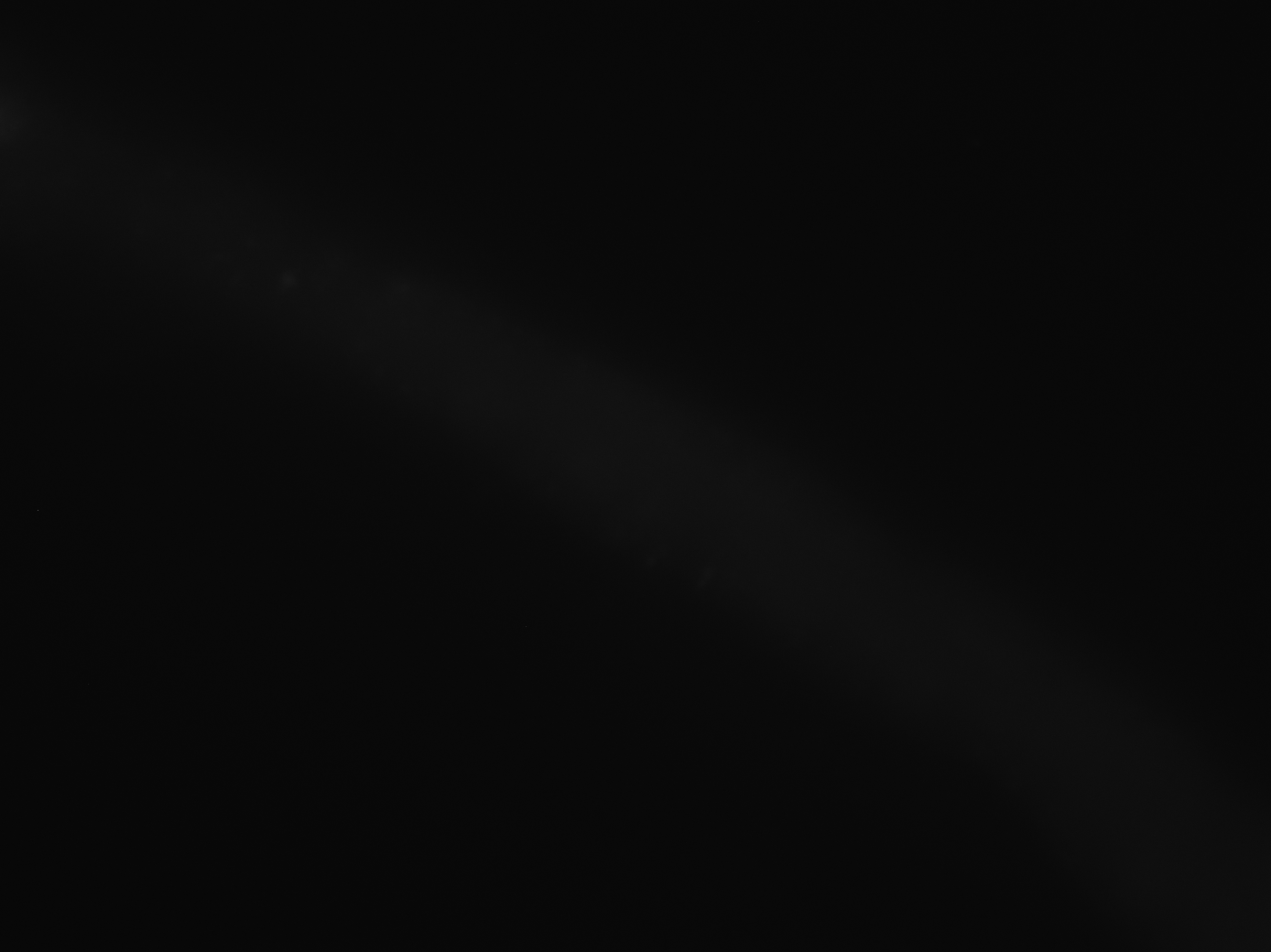

Supplement: Supplementary file 3 — Source data Fig. 2 [file 44319_2025_493_MOESM3_ESM.zip › Figure2/Fig2D/Experiment-383_VC_exc7_mbl1.tif_files/Experiment-383_z15c1x0-1388y0-1040.tif]

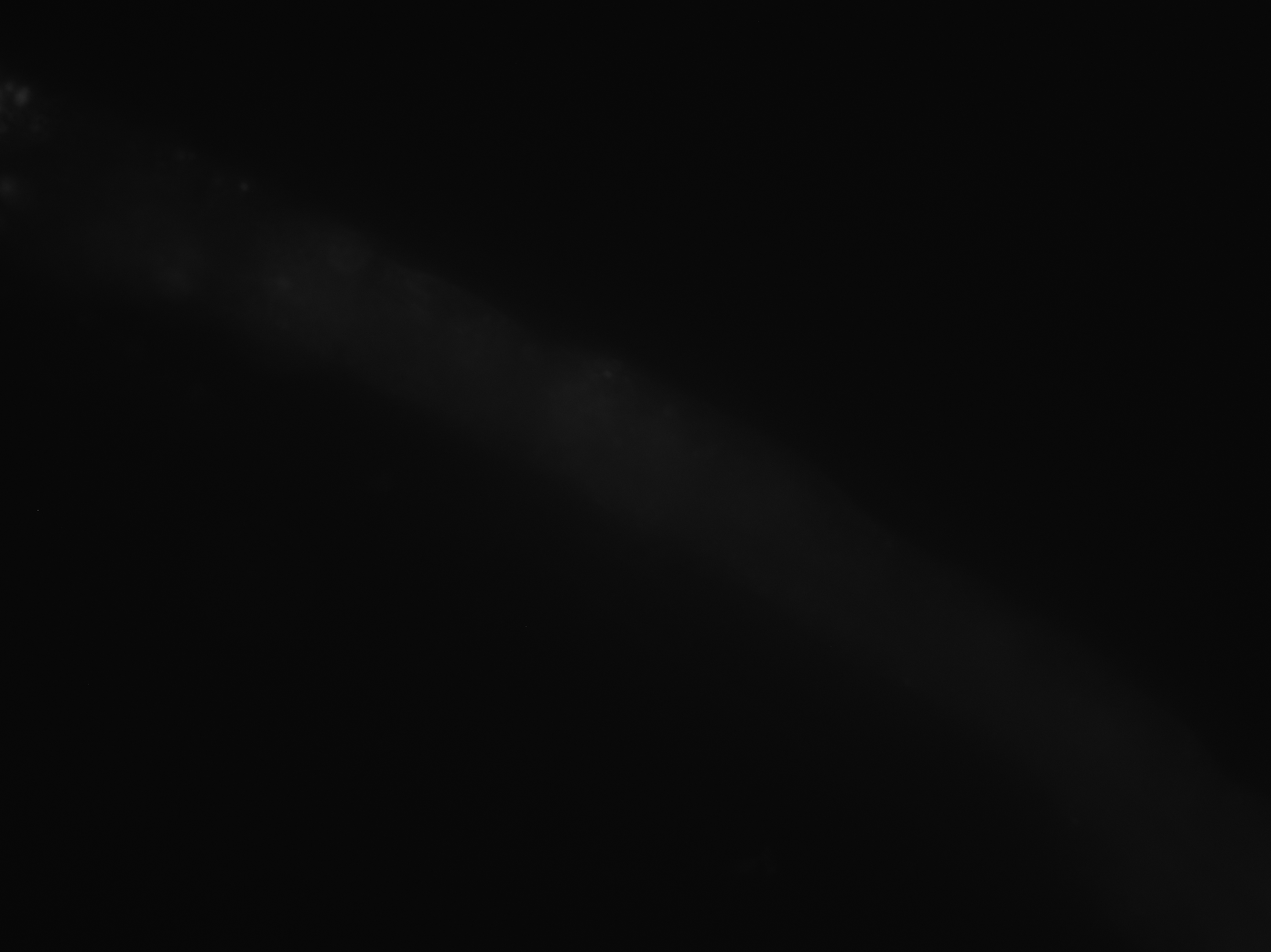

Supplement: Supplementary file 3 — Source data Fig. 2 [file 44319_2025_493_MOESM3_ESM.zip › Figure2/Fig2D/Experiment-383_VC_exc7_mbl1.tif_files/Experiment-383_z7c1x0-1388y0-1040.tif]
