# Supplementary figures and images for: Regulated microexon alternative splicing in single neurons tunes synaptic function (part 2 of 6)
Source: EMBO Rep. 2025 Jun 9;26(14):3640–62. doi: 10.1038/s44319-025-00493-7 (PMC12287369; doi:10.1038/s44319-025-00493-7)

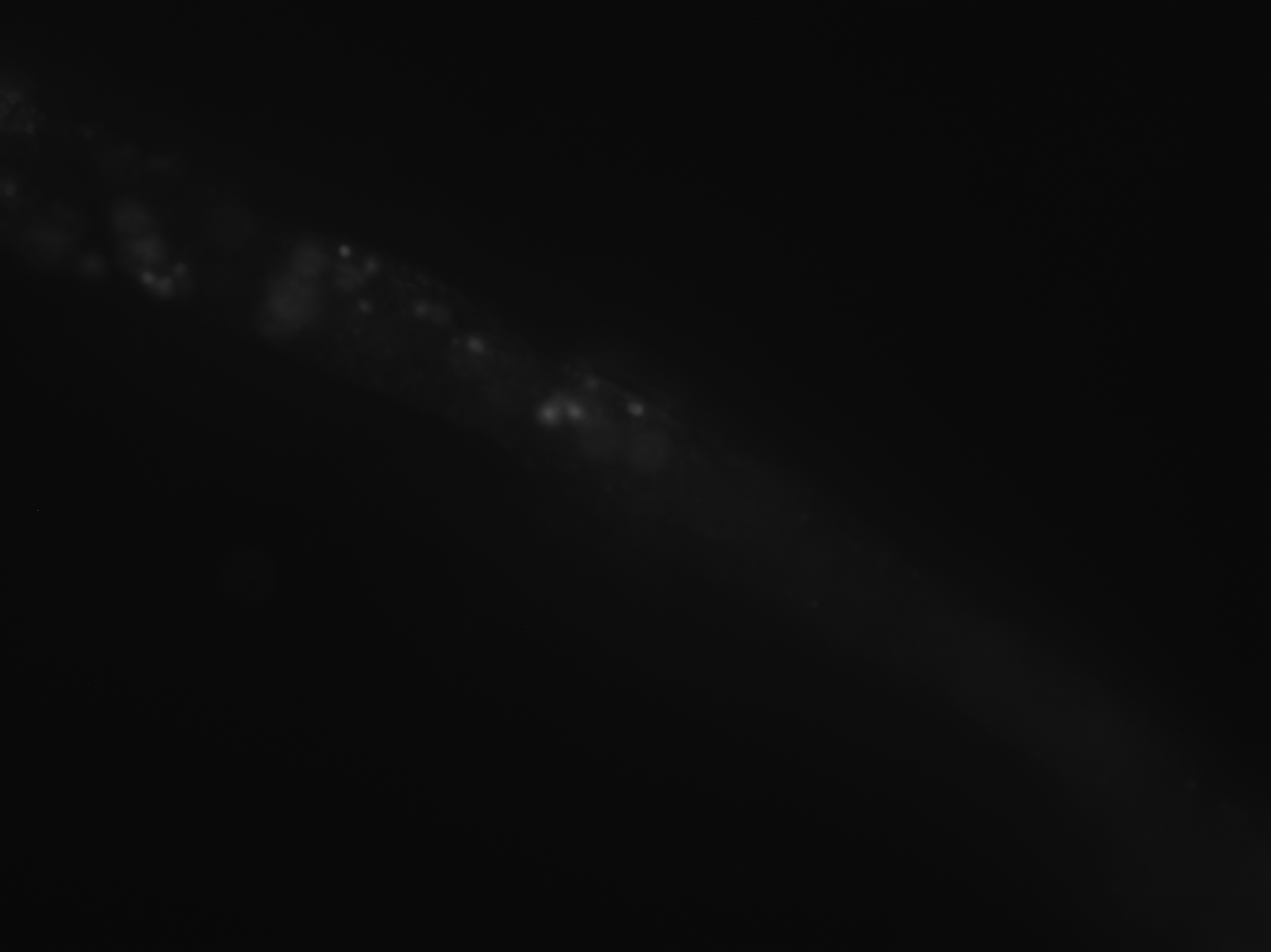

Supplement: Supplementary file 3 — Source data Fig. 2 [file 44319_2025_493_MOESM3_ESM.zip › Figure2/Fig2D/Experiment-383_VC_exc7_mbl1.tif_files/Experiment-383_z2c1x0-1388y0-1040.tif]

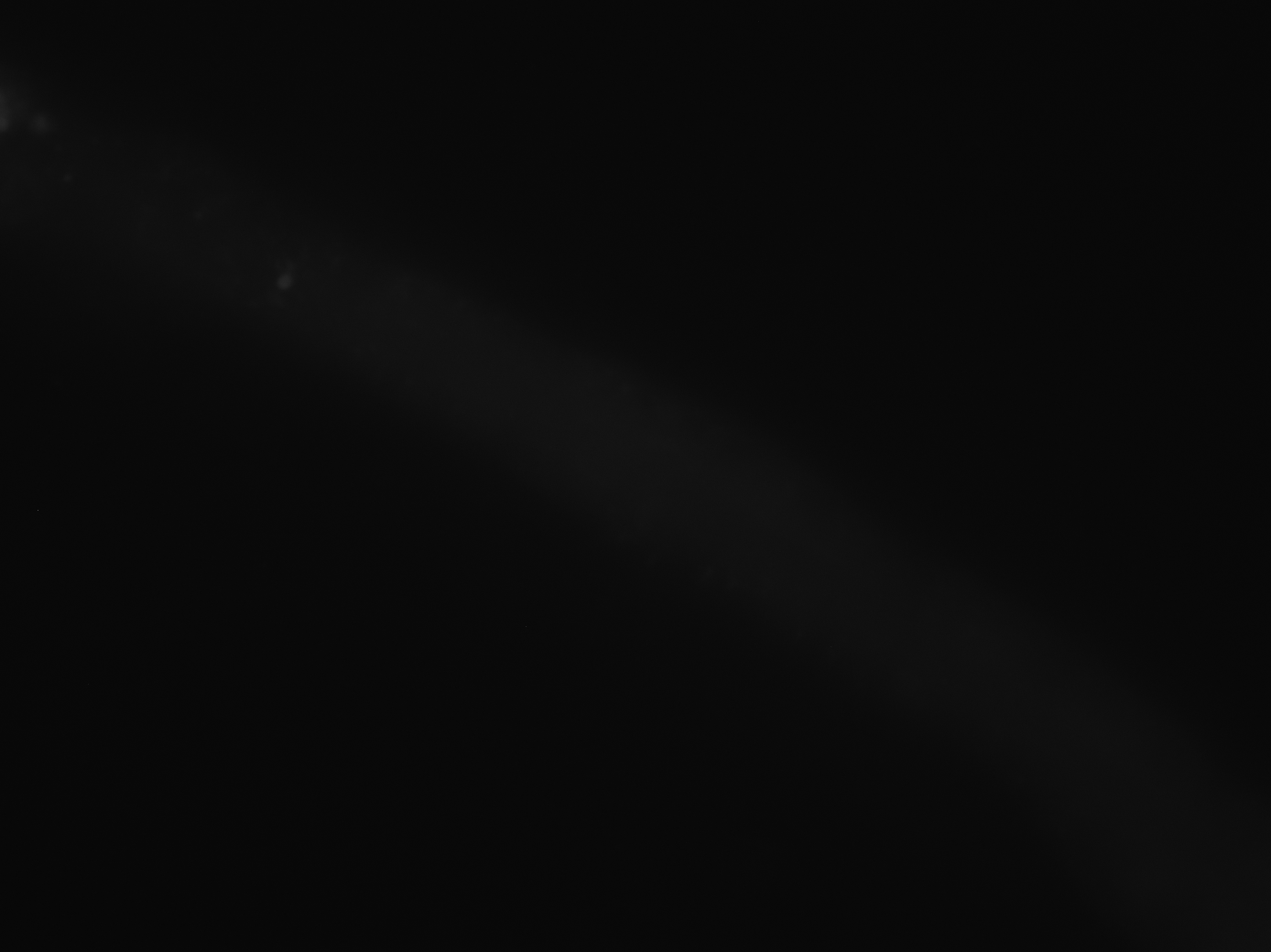

Supplement: Supplementary file 3 — Source data Fig. 2 [file 44319_2025_493_MOESM3_ESM.zip › Figure2/Fig2D/Experiment-383_VC_exc7_mbl1.tif_files/Experiment-383_z12c1x0-1388y0-1040.tif]

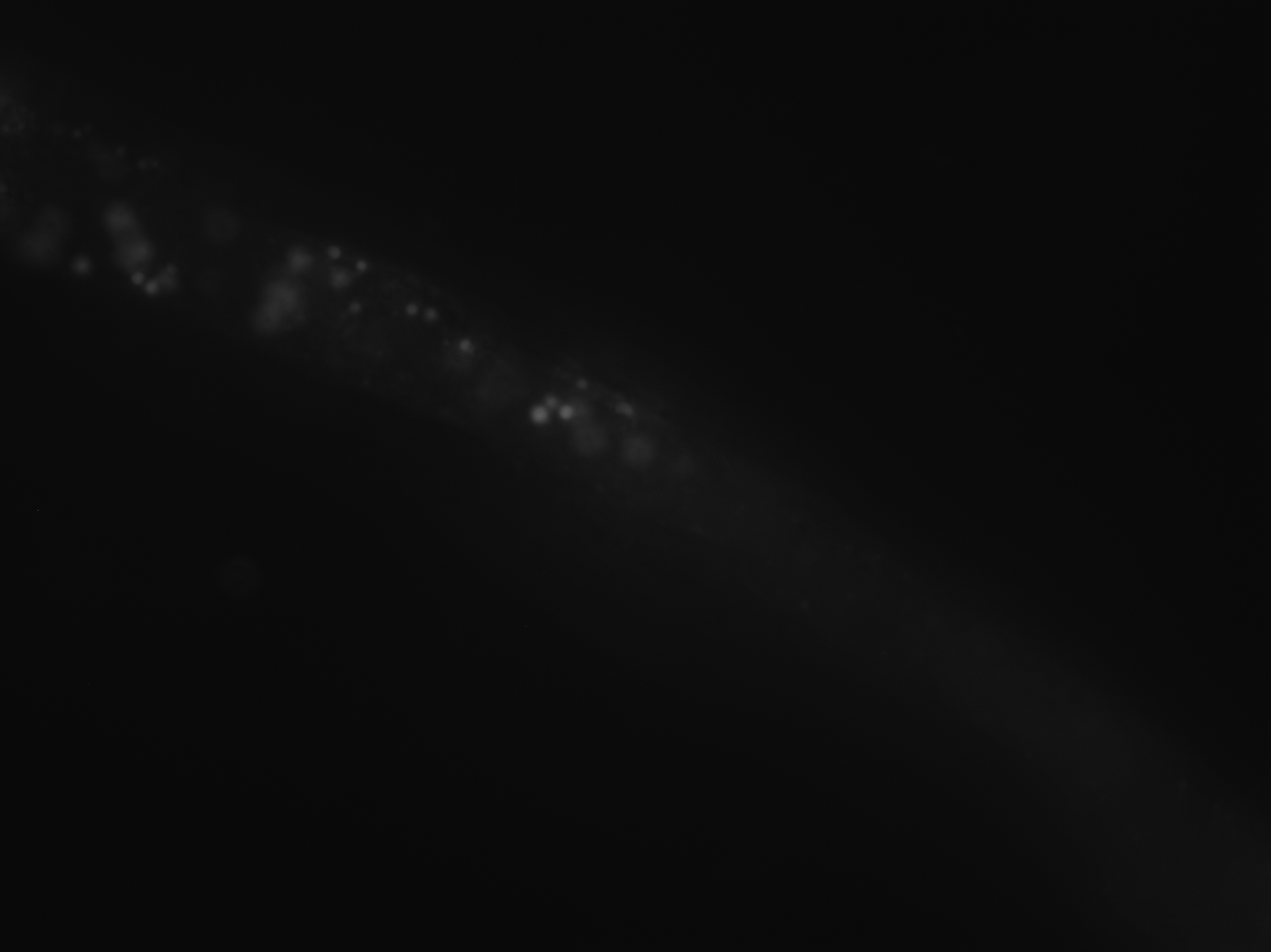

Supplement: Supplementary file 3 — Source data Fig. 2 [file 44319_2025_493_MOESM3_ESM.zip › Figure2/Fig2D/Experiment-383_VC_exc7_mbl1.tif_files/Experiment-383_z0c1x0-1388y0-1040.tif]

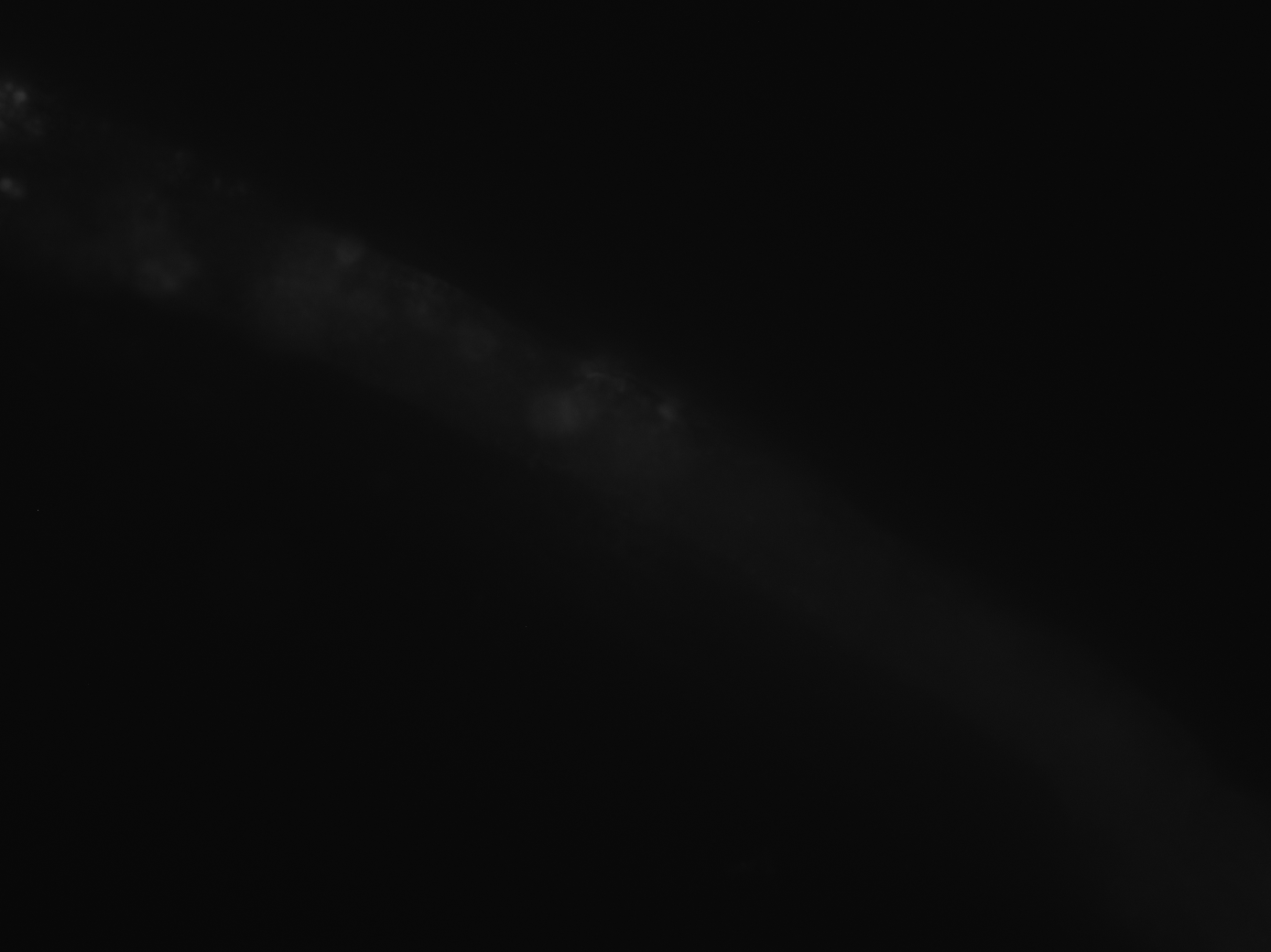

Supplement: Supplementary file 3 — Source data Fig. 2 [file 44319_2025_493_MOESM3_ESM.zip › Figure2/Fig2D/Experiment-383_VC_exc7_mbl1.tif_files/Experiment-383_z5c1x0-1388y0-1040.tif]

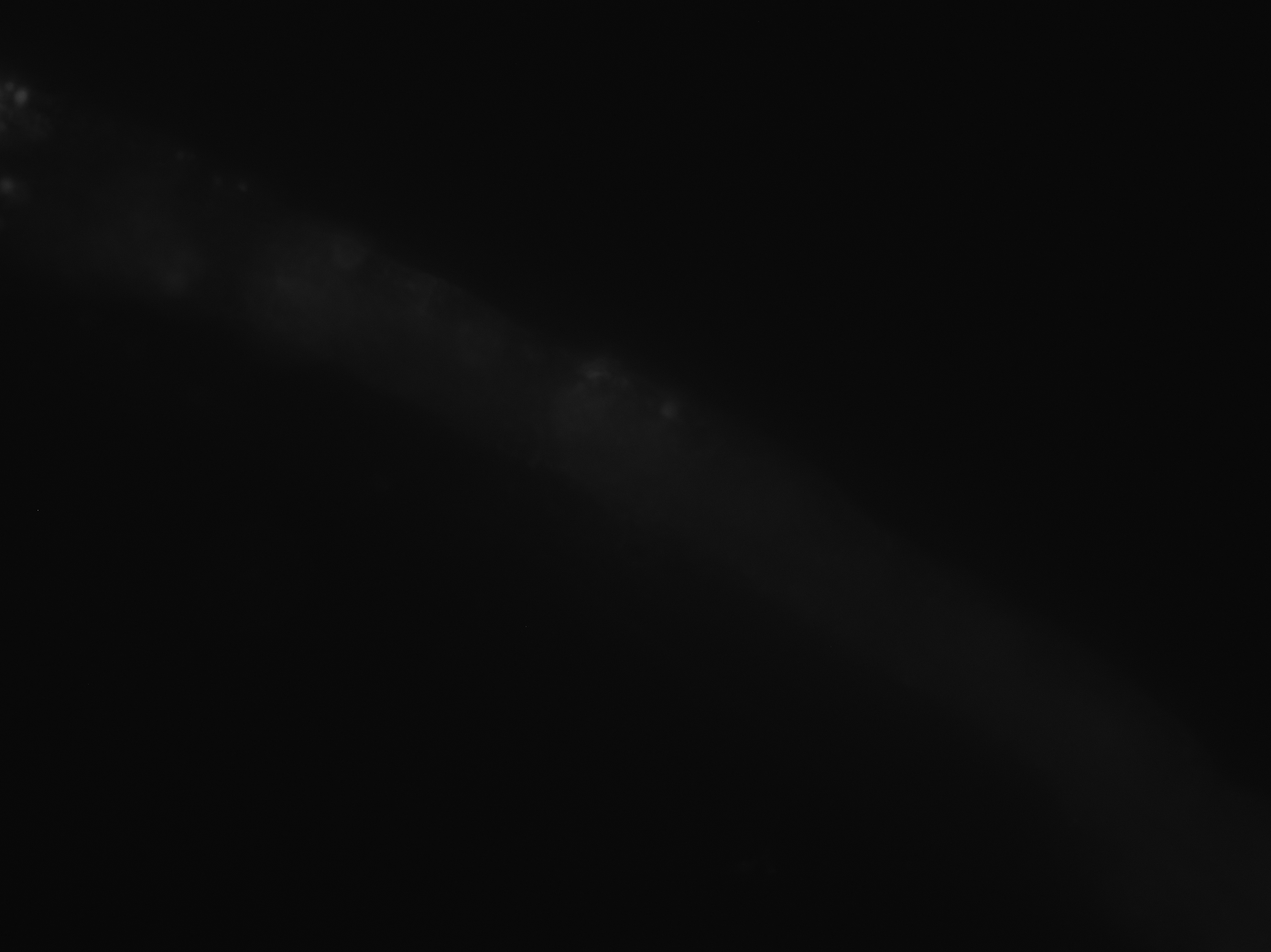

Supplement: Supplementary file 3 — Source data Fig. 2 [file 44319_2025_493_MOESM3_ESM.zip › Figure2/Fig2D/Experiment-383_VC_exc7_mbl1.tif_files/Experiment-383_z6c1x0-1388y0-1040.tif]

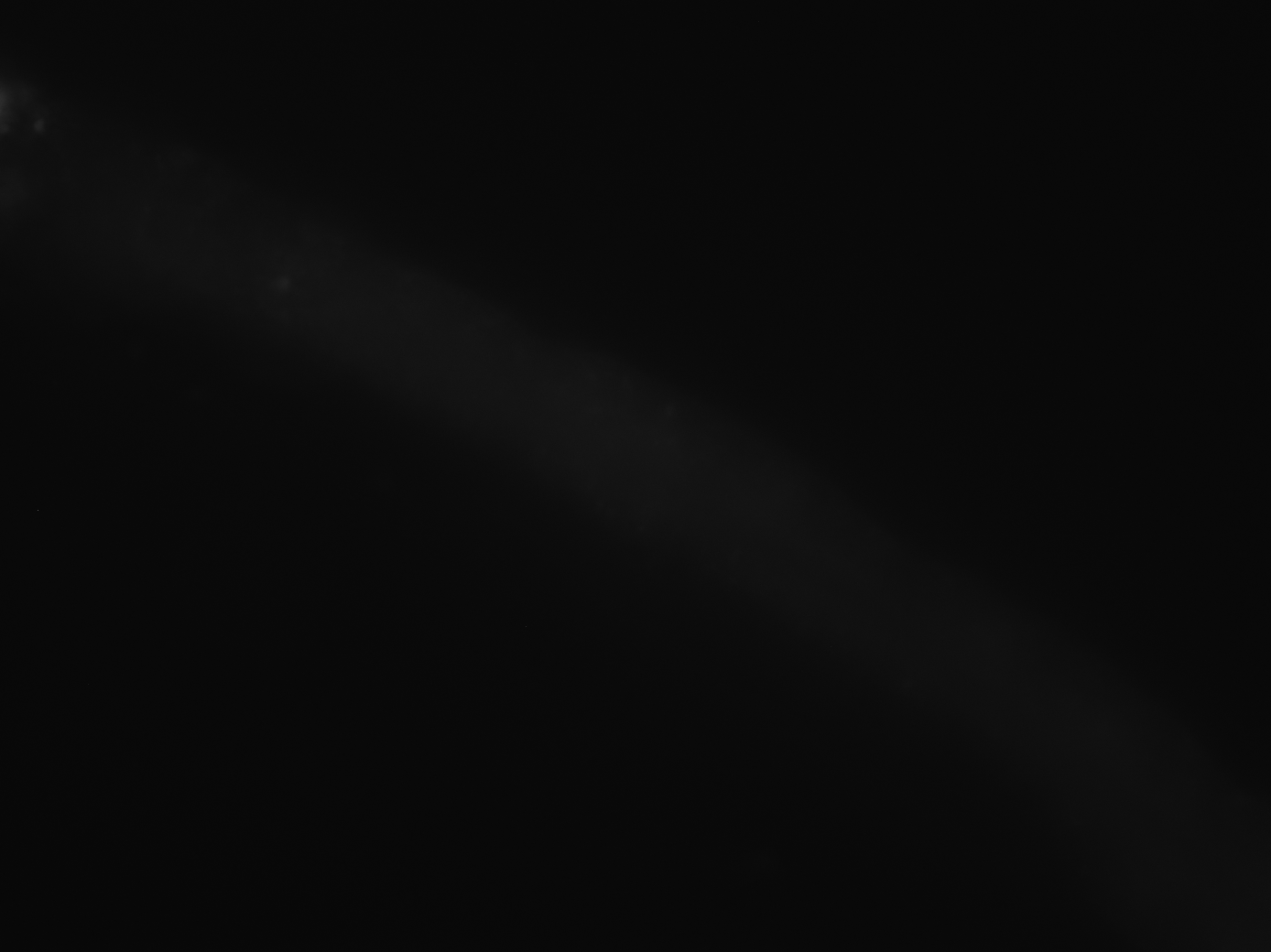

Supplement: Supplementary file 3 — Source data Fig. 2 [file 44319_2025_493_MOESM3_ESM.zip › Figure2/Fig2D/Experiment-383_VC_exc7_mbl1.tif_files/Experiment-383_z9c1x0-1388y0-1040.tif]

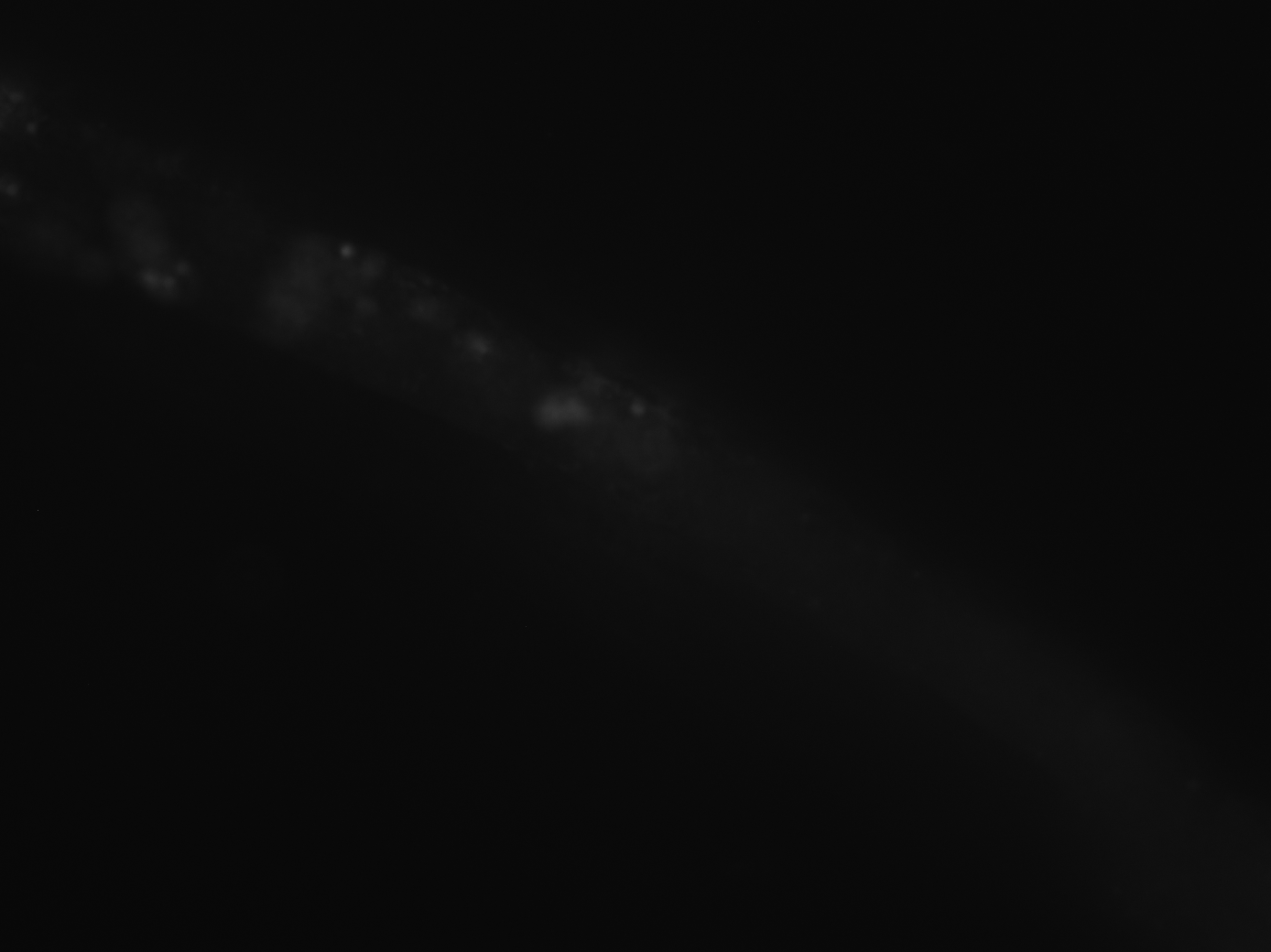

Supplement: Supplementary file 3 — Source data Fig. 2 [file 44319_2025_493_MOESM3_ESM.zip › Figure2/Fig2D/Experiment-383_VC_exc7_mbl1.tif_files/Experiment-383_z3c1x0-1388y0-1040.tif]

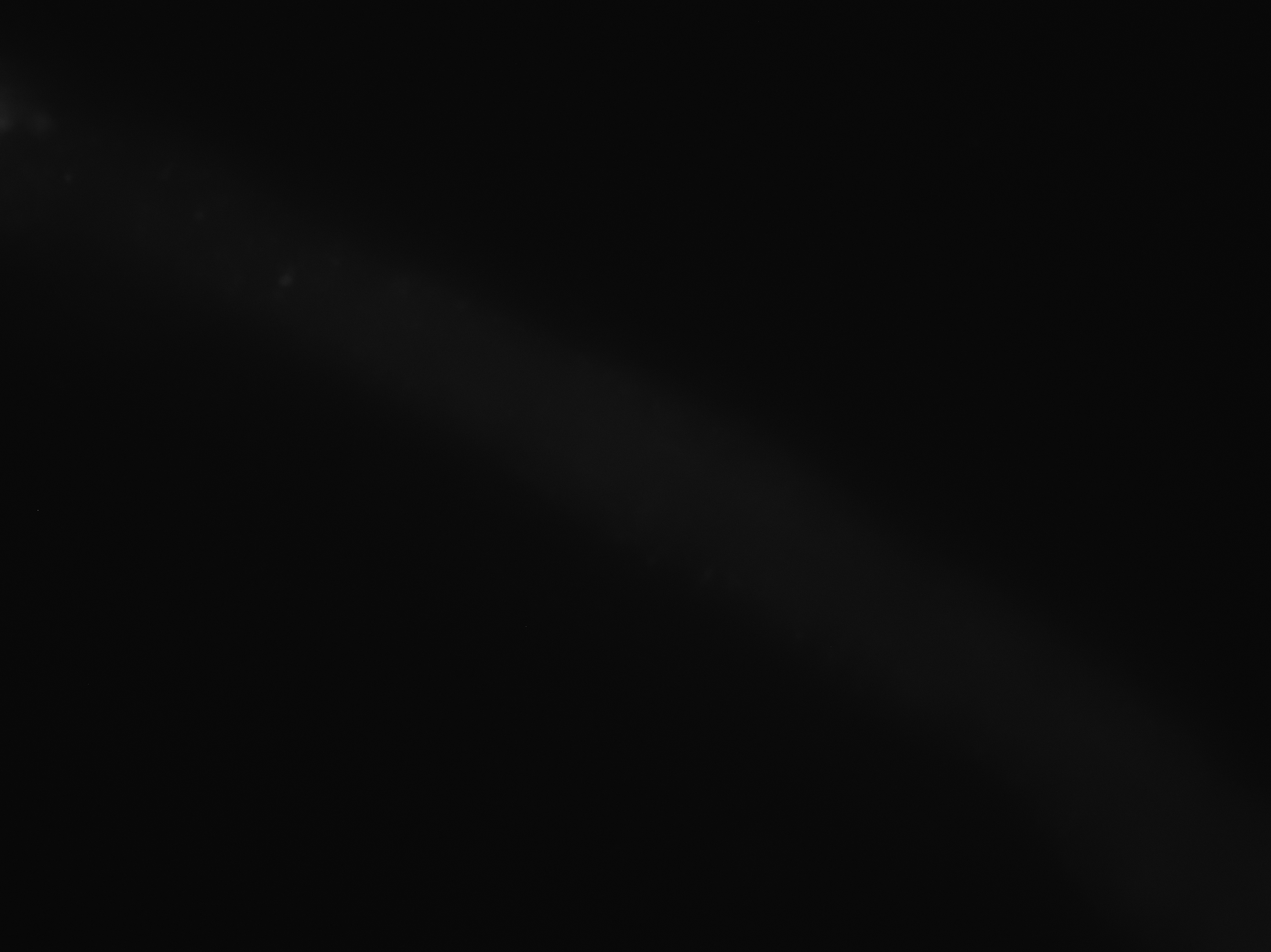

Supplement: Supplementary file 3 — Source data Fig. 2 [file 44319_2025_493_MOESM3_ESM.zip › Figure2/Fig2D/Experiment-383_VC_exc7_mbl1.tif_files/Experiment-383_z13c1x0-1388y0-1040.tif]

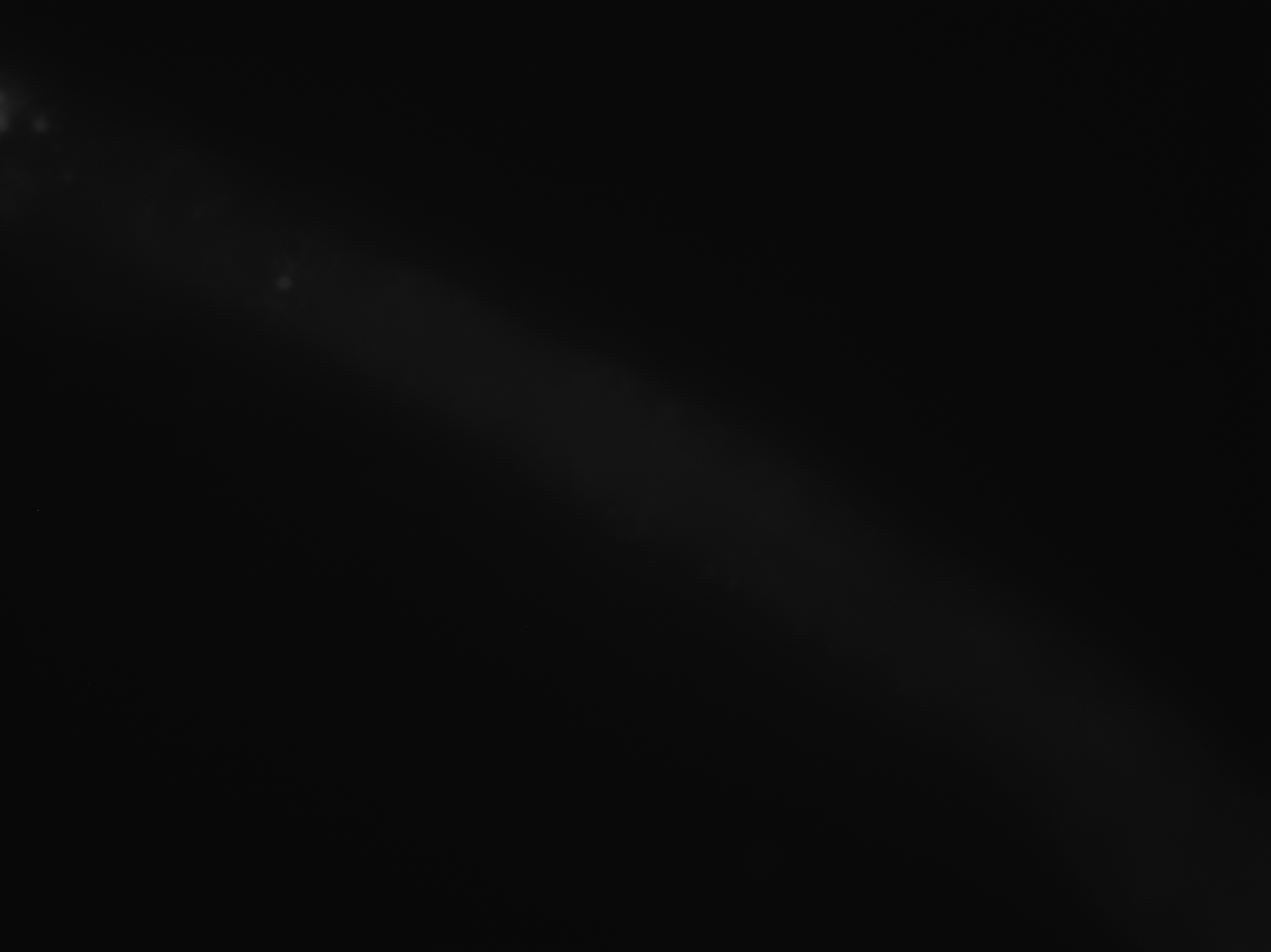

Supplement: Supplementary file 3 — Source data Fig. 2 [file 44319_2025_493_MOESM3_ESM.zip › Figure2/Fig2D/Experiment-383_VC_exc7_mbl1.tif_files/Experiment-383_z11c1x0-1388y0-1040.tif]

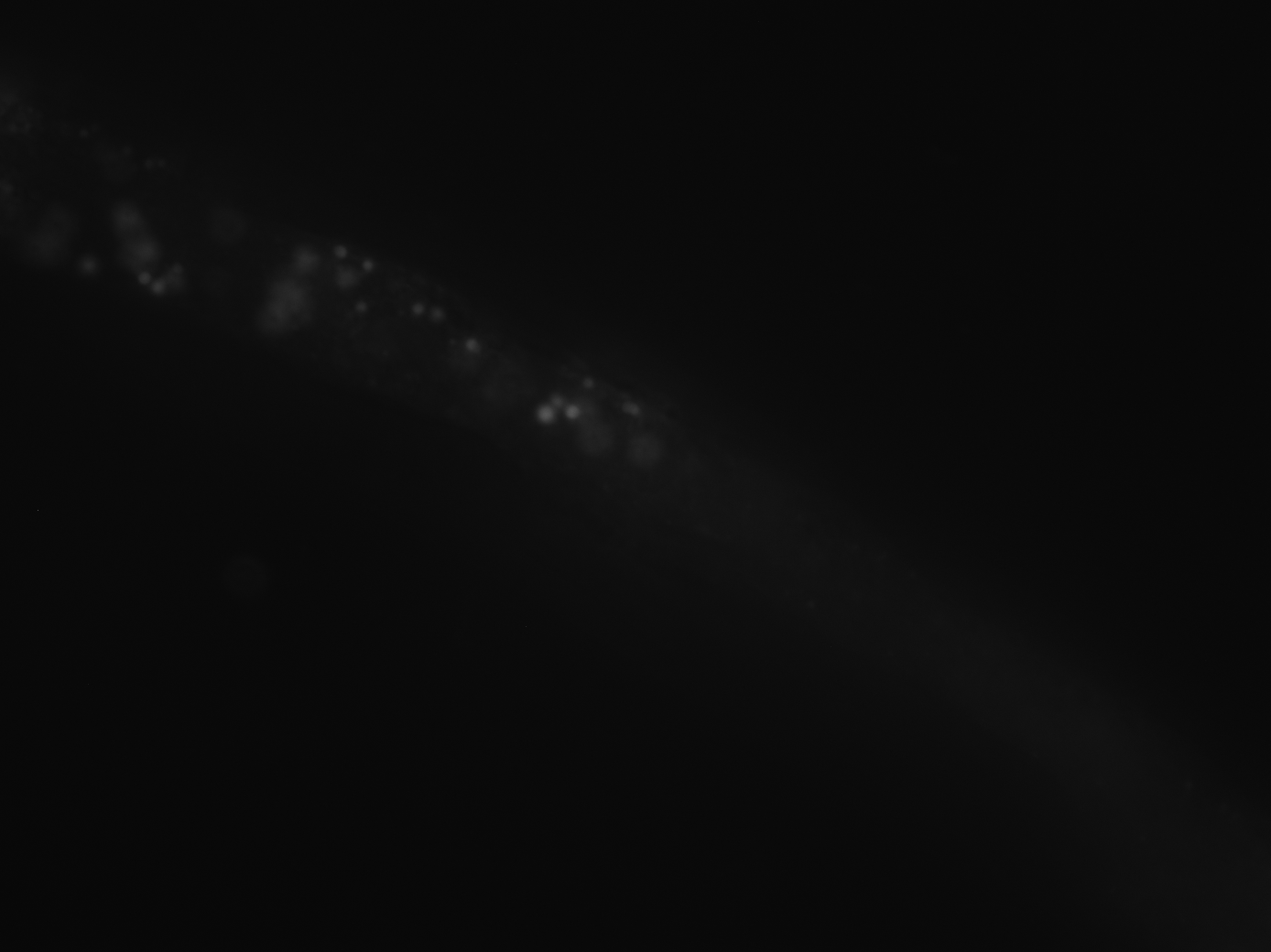

Supplement: Supplementary file 3 — Source data Fig. 2 [file 44319_2025_493_MOESM3_ESM.zip › Figure2/Fig2D/Experiment-383_VC_exc7_mbl1.tif_files/Experiment-383_z1c1x0-1388y0-1040.tif]

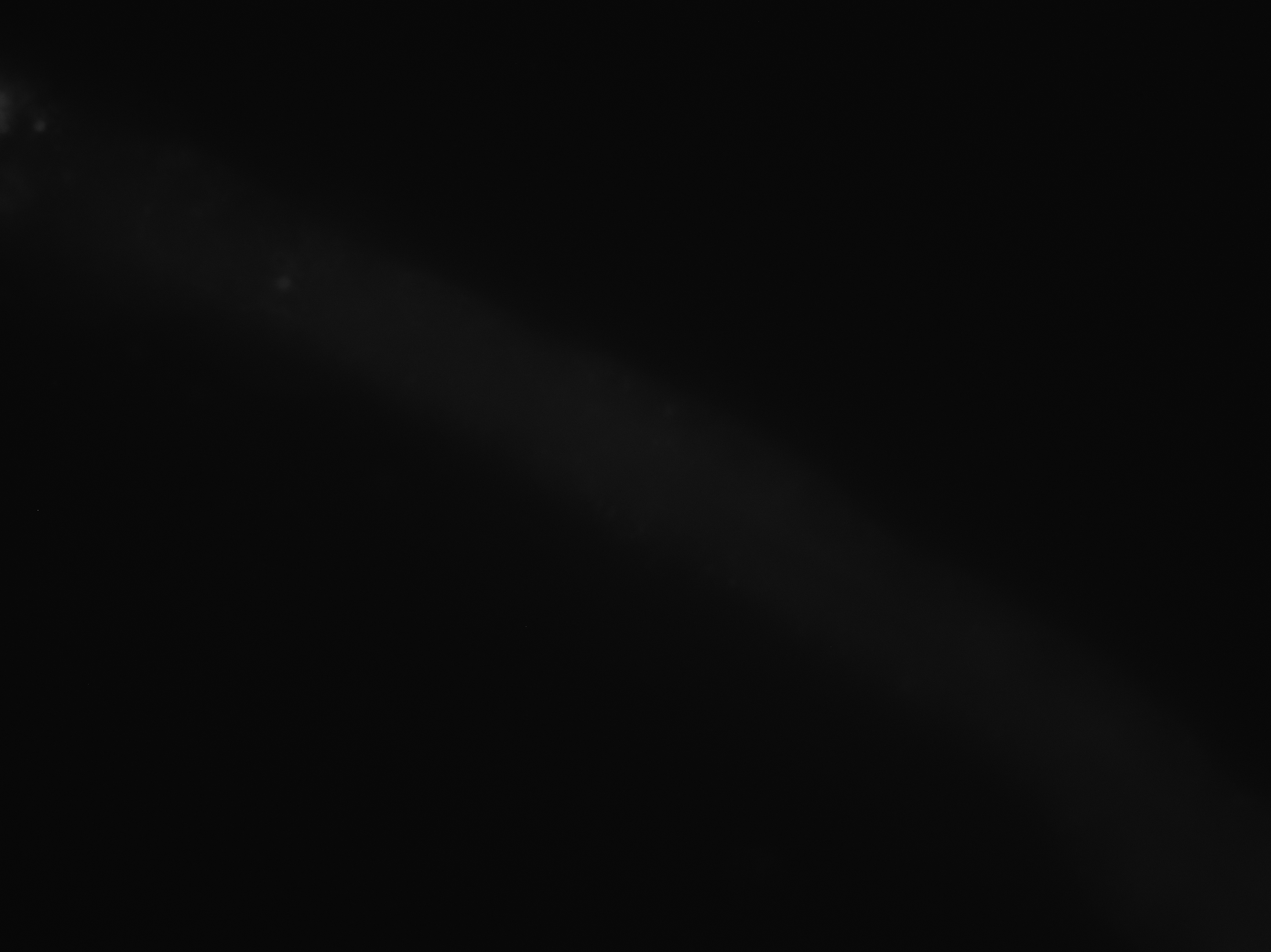

Supplement: Supplementary file 3 — Source data Fig. 2 [file 44319_2025_493_MOESM3_ESM.zip › Figure2/Fig2D/Experiment-383_VC_exc7_mbl1.tif_files/Experiment-383_z10c1x0-1388y0-1040.tif]

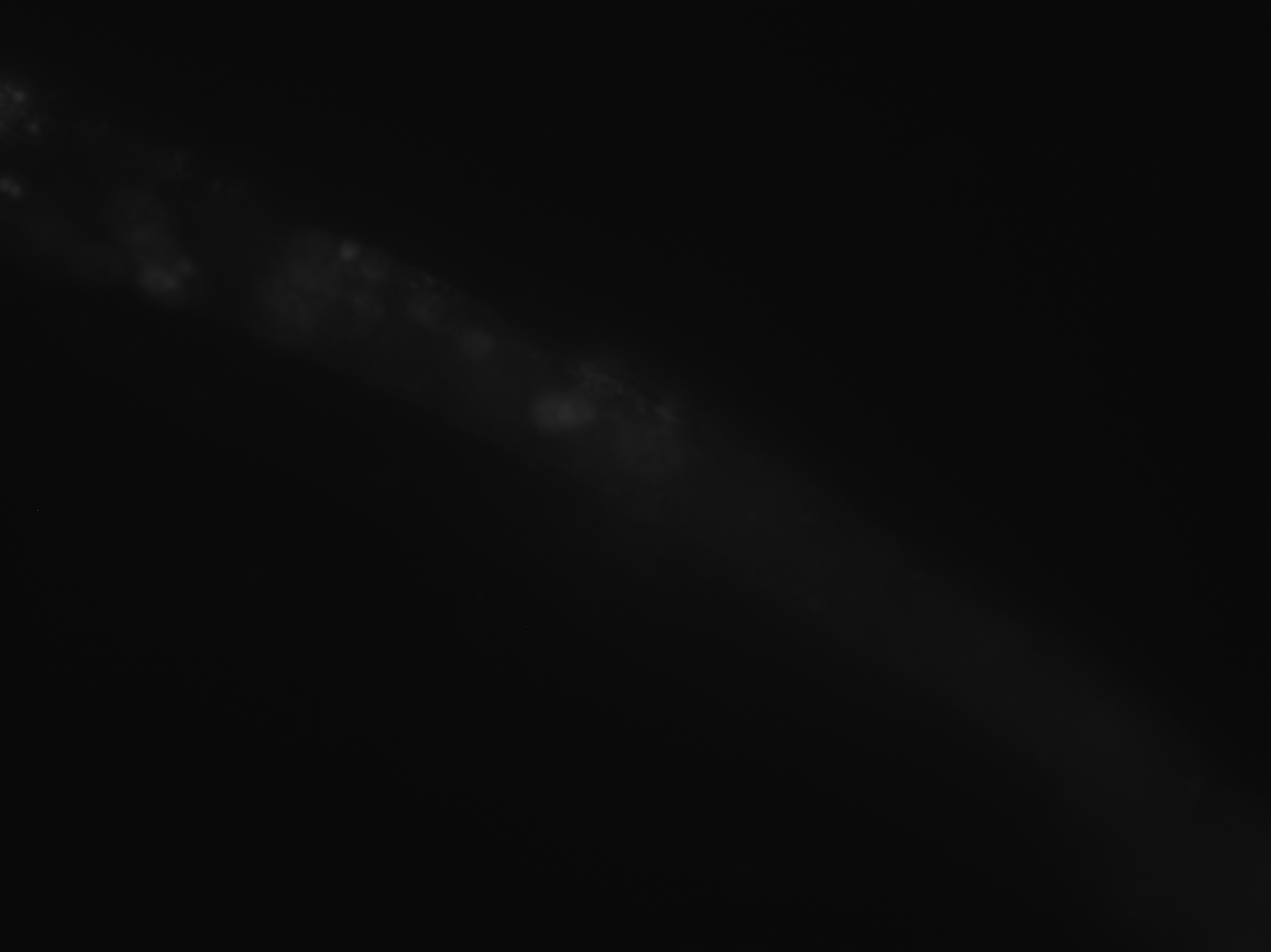

Supplement: Supplementary file 3 — Source data Fig. 2 [file 44319_2025_493_MOESM3_ESM.zip › Figure2/Fig2D/Experiment-383_VC_exc7_mbl1.tif_files/Experiment-383_z4c1x0-1388y0-1040.tif]

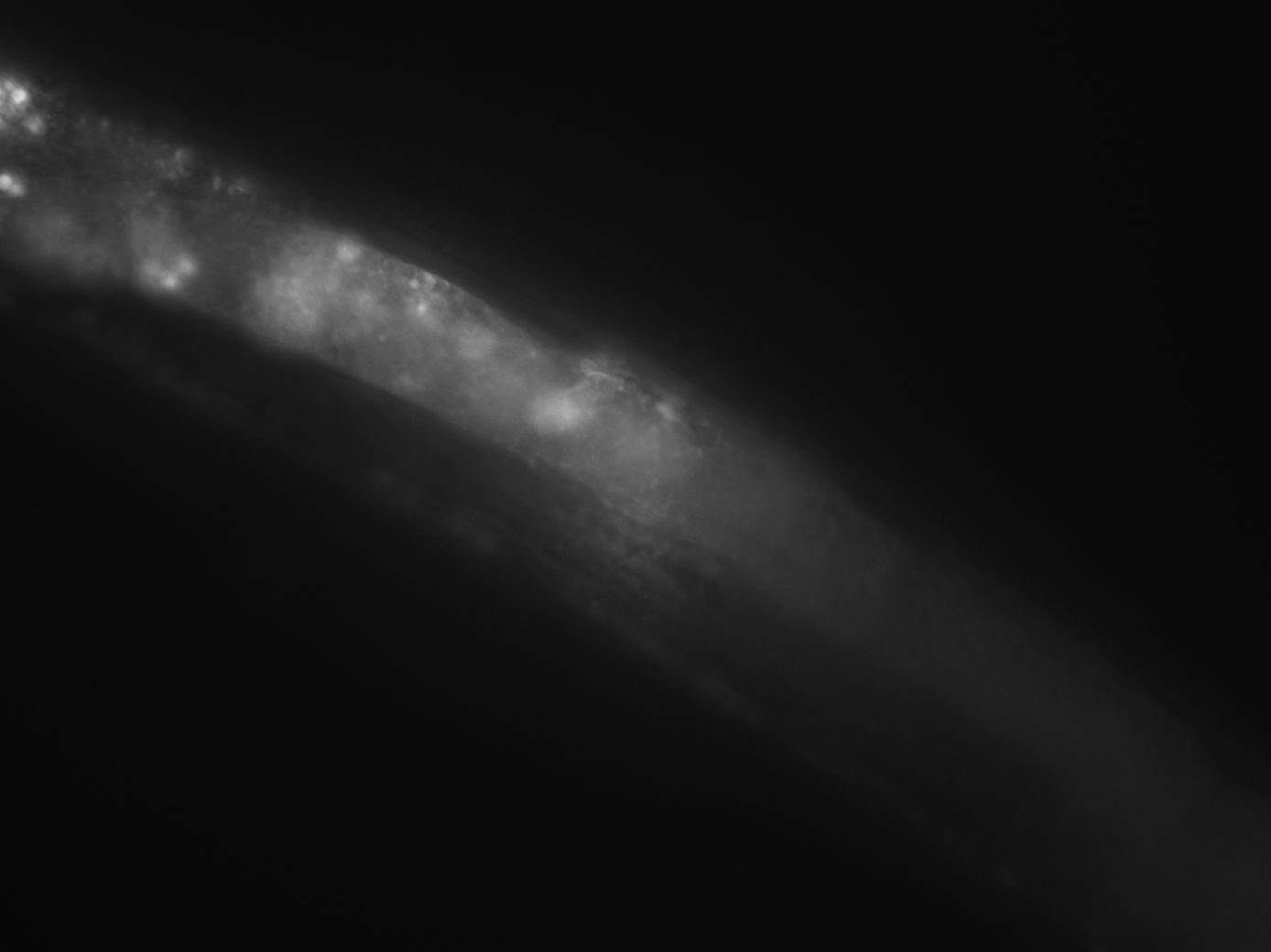

Supplement: Supplementary file 3 — Source data Fig. 2 [file 44319_2025_493_MOESM3_ESM.zip › Figure2/Fig2D/Experiment-383_VC_exc7_mbl1.tif_files/Experiment-383_z5c0x0-1388y0-1040.tif]

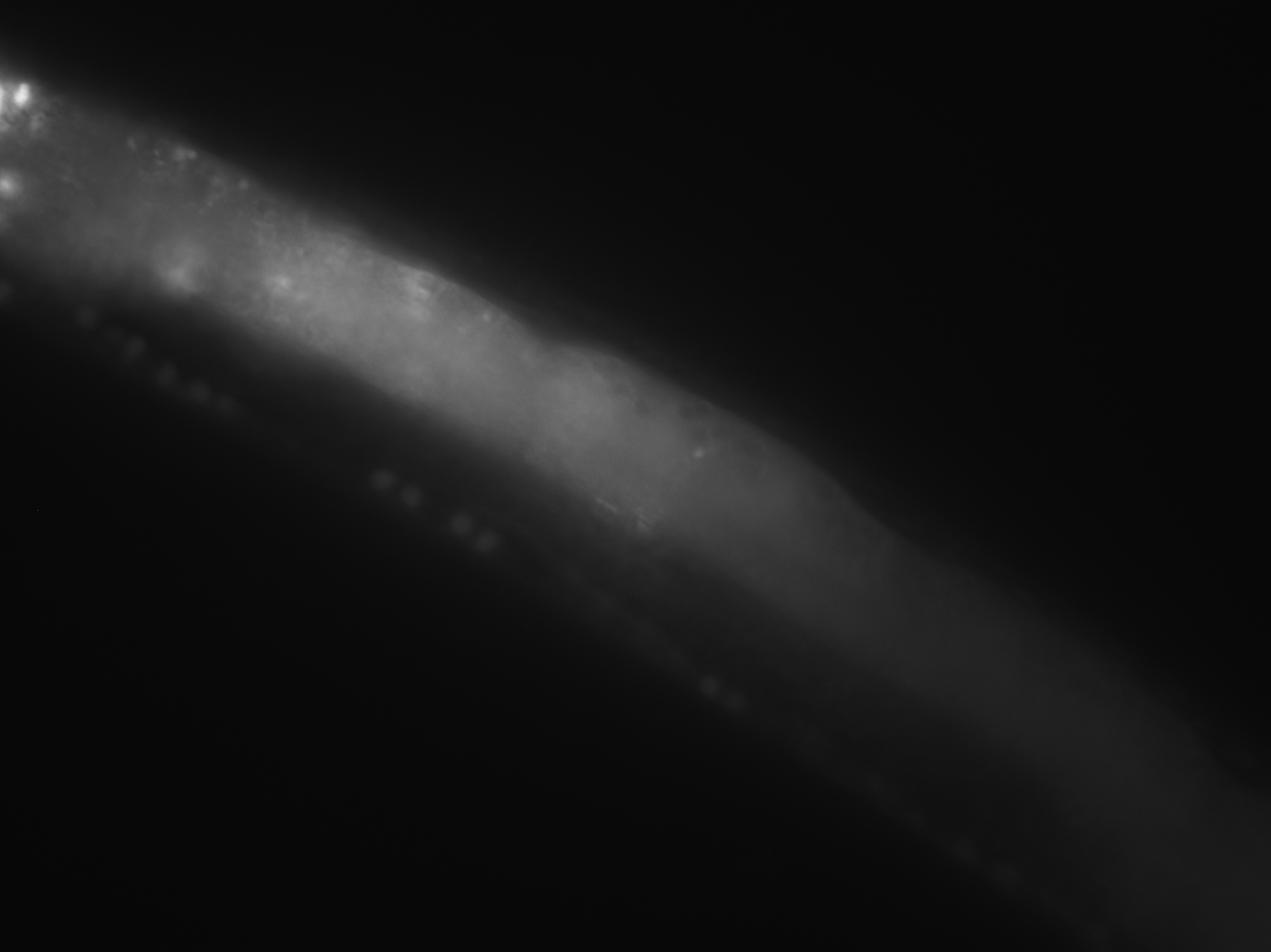

Supplement: Supplementary file 3 — Source data Fig. 2 [file 44319_2025_493_MOESM3_ESM.zip › Figure2/Fig2D/Experiment-383_VC_exc7_mbl1.tif_files/Experiment-383_z8c0x0-1388y0-1040.tif]

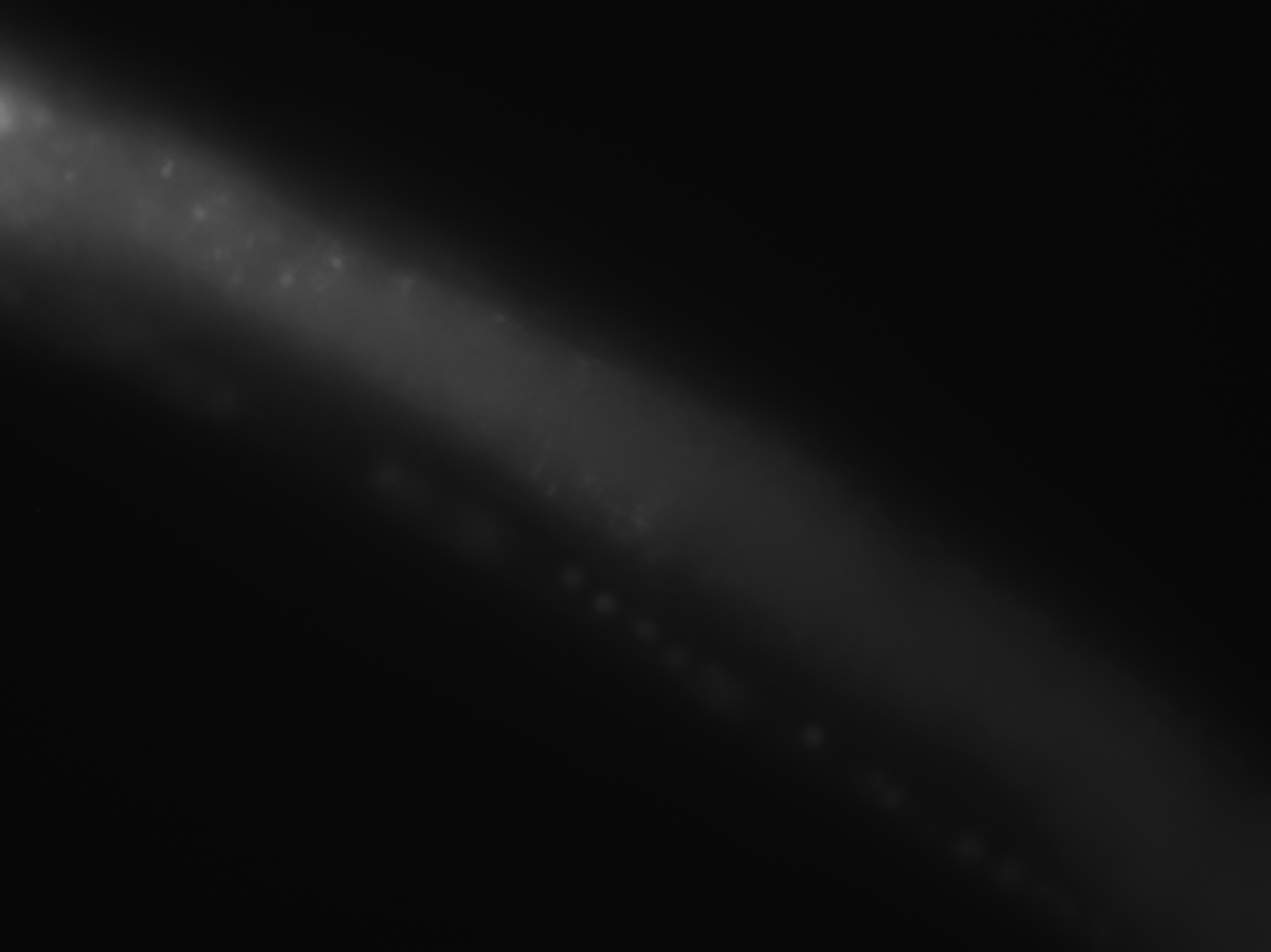

Supplement: Supplementary file 3 — Source data Fig. 2 [file 44319_2025_493_MOESM3_ESM.zip › Figure2/Fig2D/Experiment-383_VC_exc7_mbl1.tif_files/Experiment-383_z14c0x0-1388y0-1040.tif]

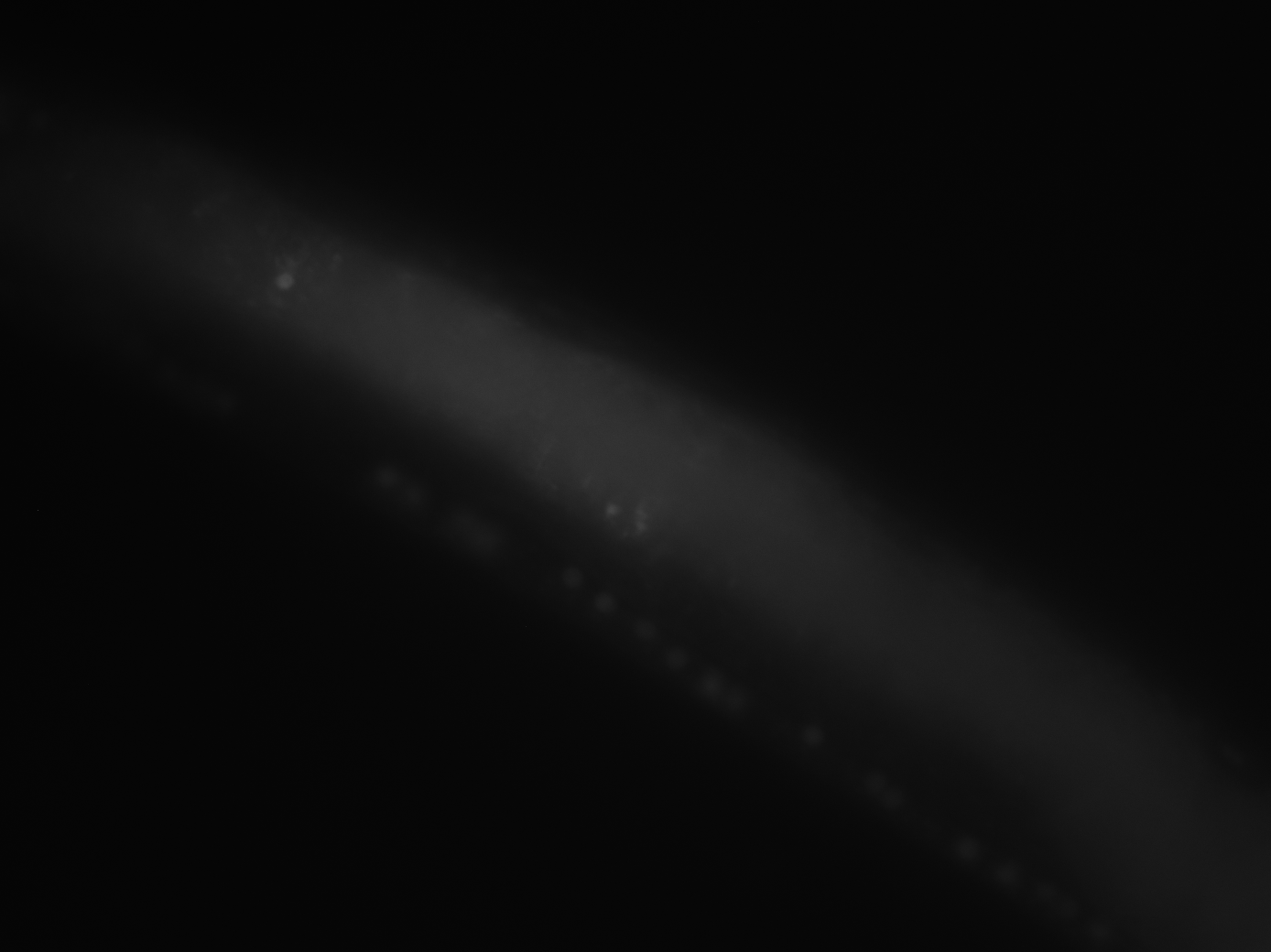

Supplement: Supplementary file 3 — Source data Fig. 2 [file 44319_2025_493_MOESM3_ESM.zip › Figure2/Fig2D/Experiment-383_VC_exc7_mbl1.tif_files/Experiment-383_z12c0x0-1388y0-1040.tif]

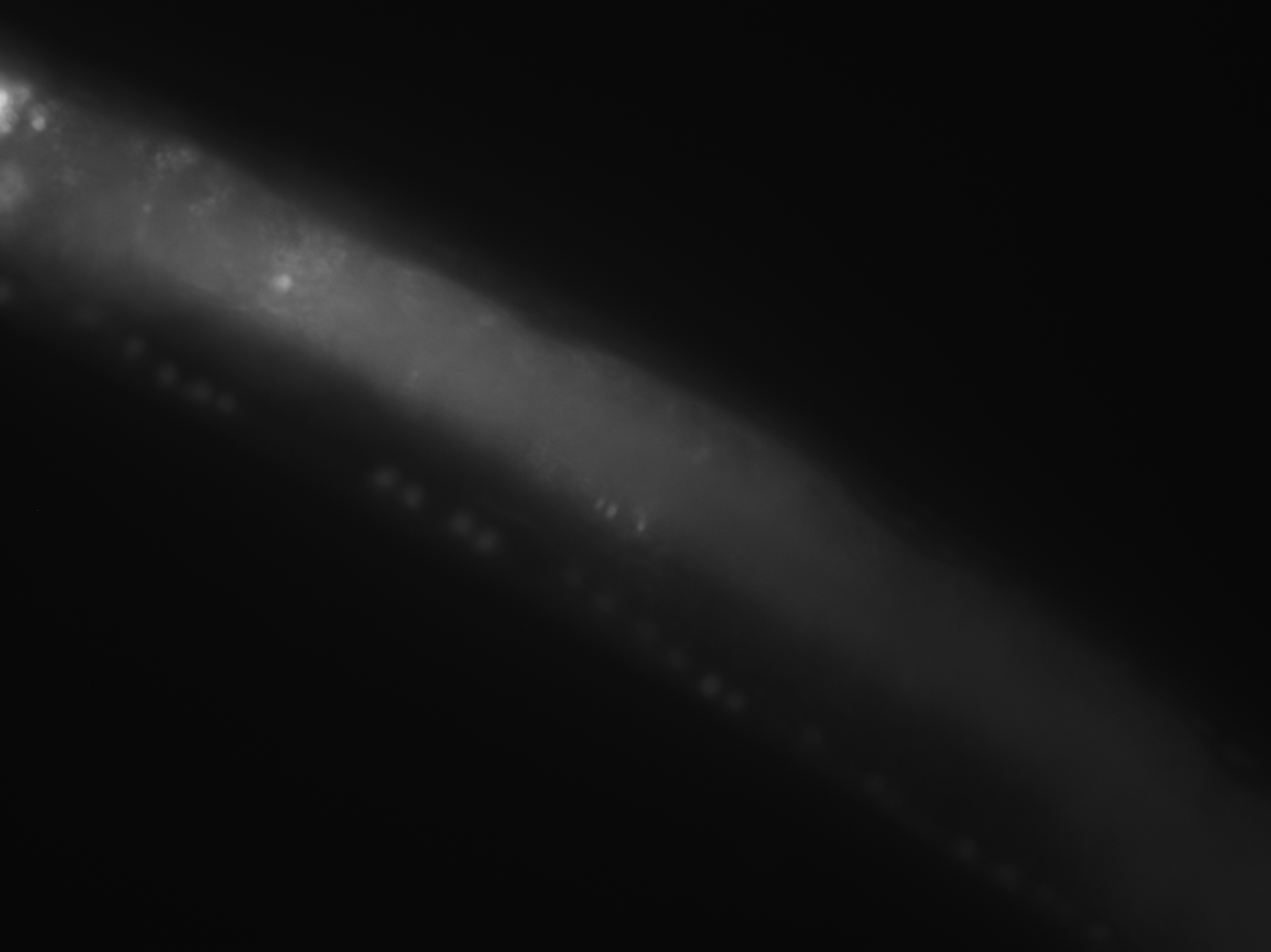

Supplement: Supplementary file 3 — Source data Fig. 2 [file 44319_2025_493_MOESM3_ESM.zip › Figure2/Fig2D/Experiment-383_VC_exc7_mbl1.tif_files/Experiment-383_z10c0x0-1388y0-1040.tif]

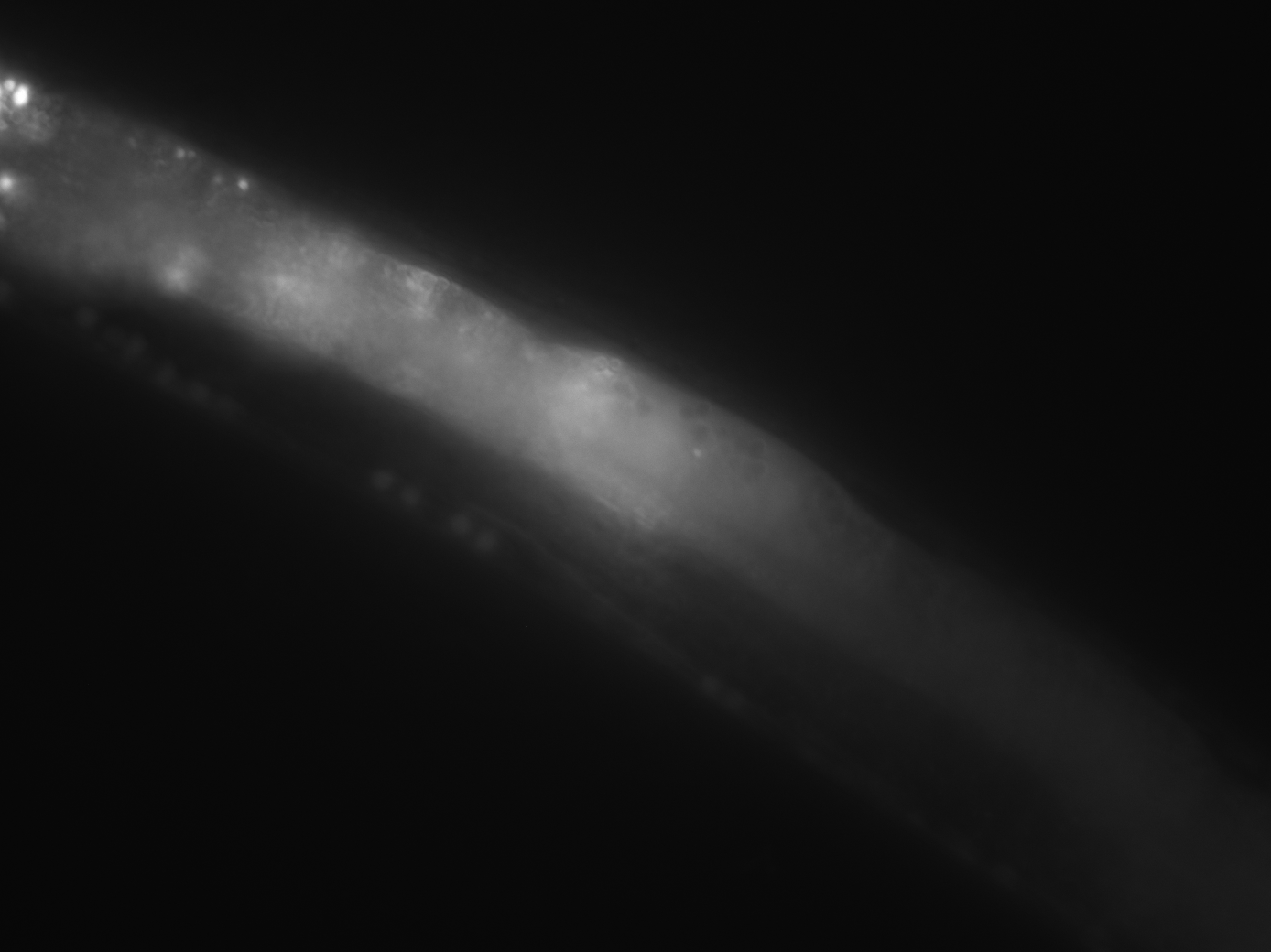

Supplement: Supplementary file 3 — Source data Fig. 2 [file 44319_2025_493_MOESM3_ESM.zip › Figure2/Fig2D/Experiment-383_VC_exc7_mbl1.tif_files/Experiment-383_z7c0x0-1388y0-1040.tif]

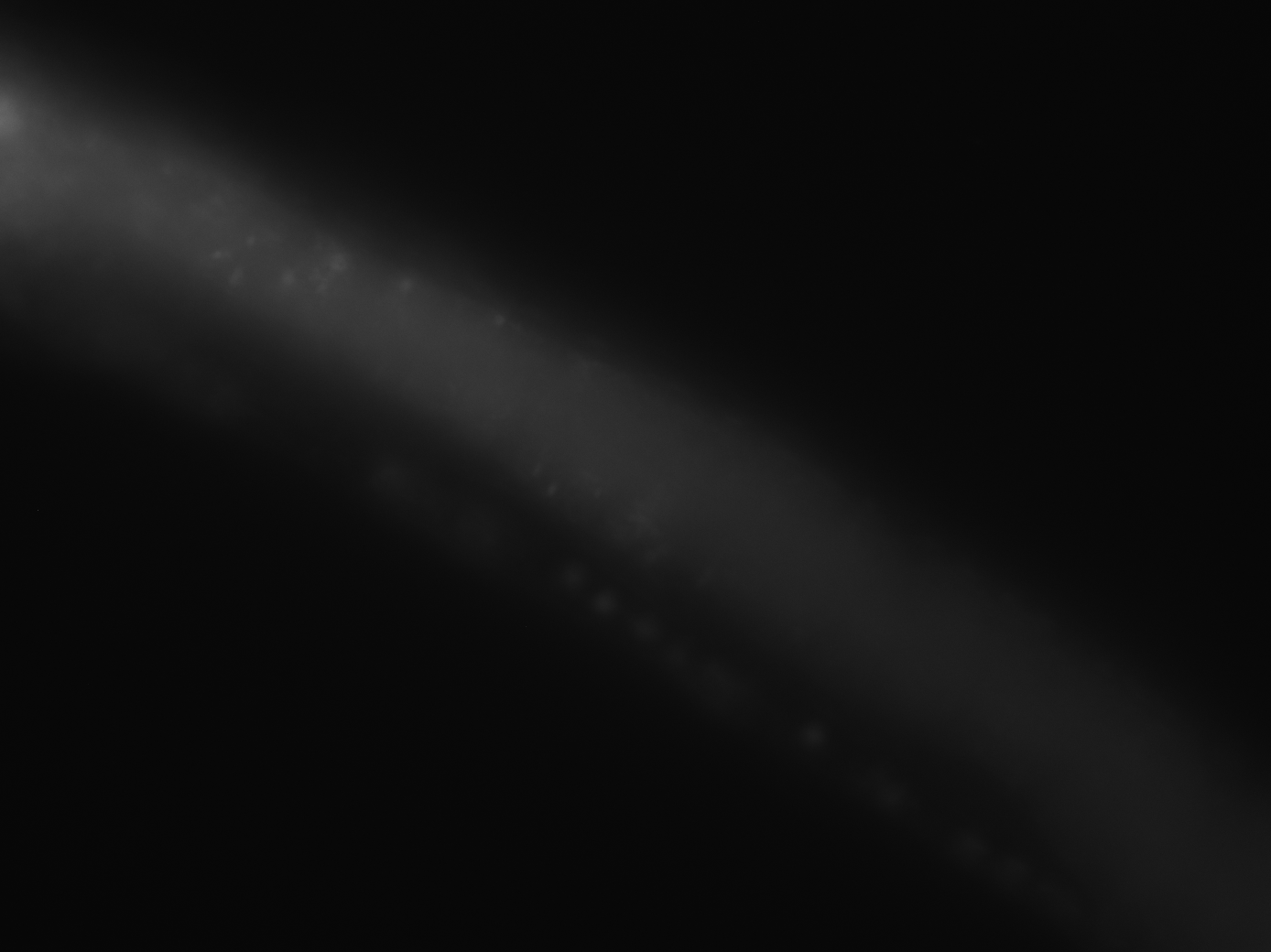

Supplement: Supplementary file 3 — Source data Fig. 2 [file 44319_2025_493_MOESM3_ESM.zip › Figure2/Fig2D/Experiment-383_VC_exc7_mbl1.tif_files/Experiment-383_z15c0x0-1388y0-1040.tif]

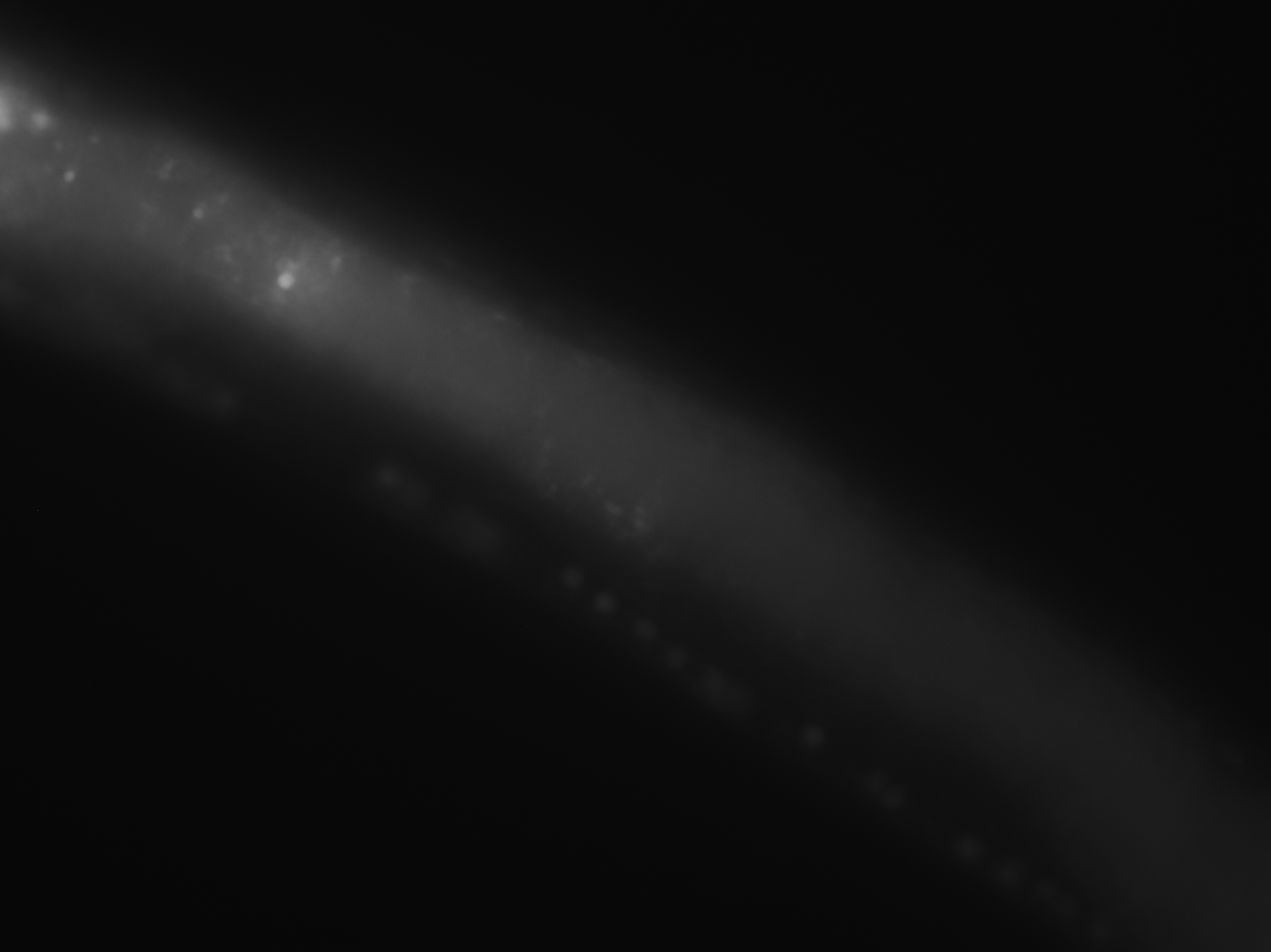

Supplement: Supplementary file 3 — Source data Fig. 2 [file 44319_2025_493_MOESM3_ESM.zip › Figure2/Fig2D/Experiment-383_VC_exc7_mbl1.tif_files/Experiment-383_z13c0x0-1388y0-1040.tif]

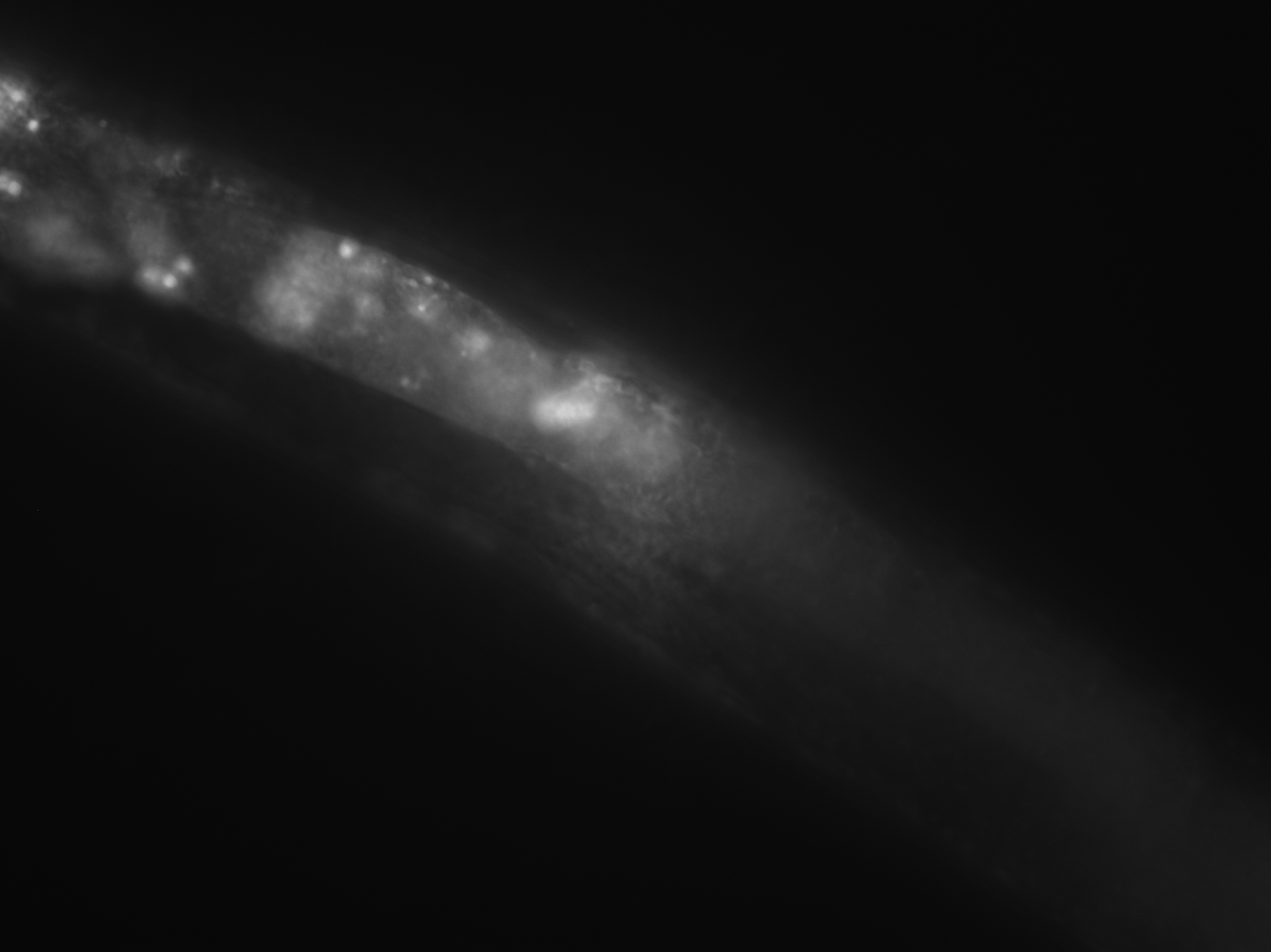

Supplement: Supplementary file 3 — Source data Fig. 2 [file 44319_2025_493_MOESM3_ESM.zip › Figure2/Fig2D/Experiment-383_VC_exc7_mbl1.tif_files/Experiment-383_z4c0x0-1388y0-1040.tif]

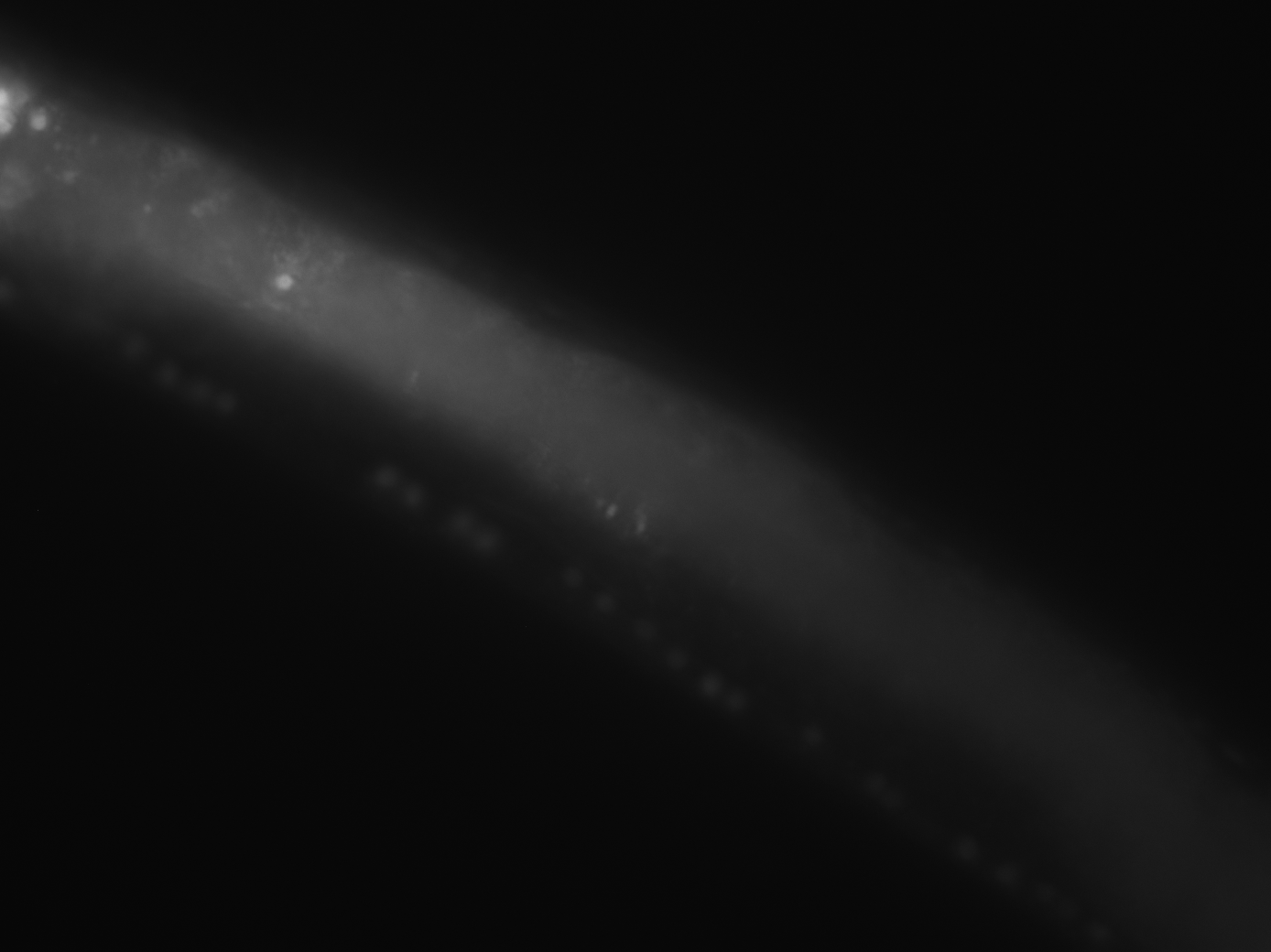

Supplement: Supplementary file 3 — Source data Fig. 2 [file 44319_2025_493_MOESM3_ESM.zip › Figure2/Fig2D/Experiment-383_VC_exc7_mbl1.tif_files/Experiment-383_z11c0x0-1388y0-1040.tif]

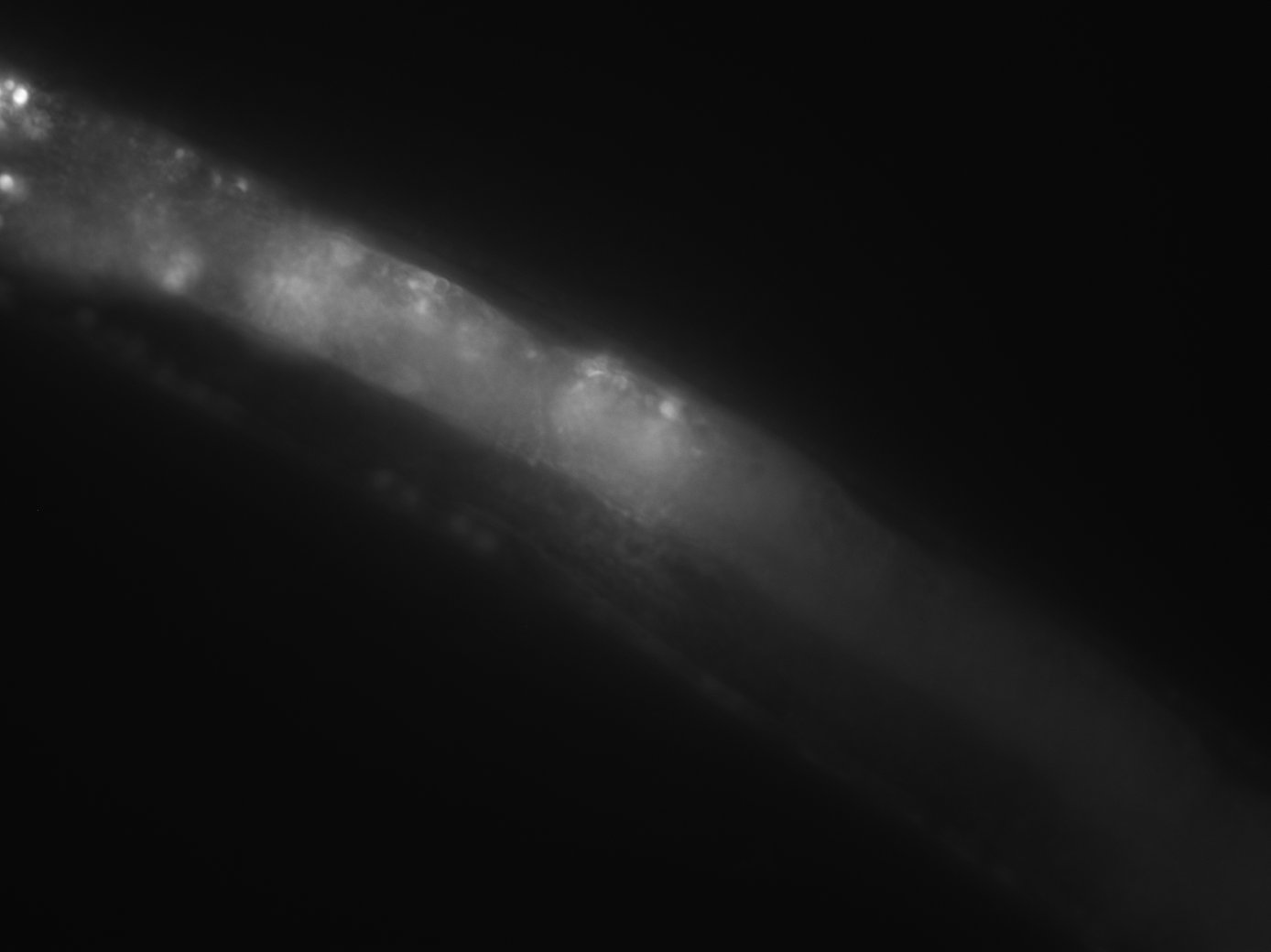

Supplement: Supplementary file 3 — Source data Fig. 2 [file 44319_2025_493_MOESM3_ESM.zip › Figure2/Fig2D/Experiment-383_VC_exc7_mbl1.tif_files/Experiment-383_z6c0x0-1388y0-1040.tif]

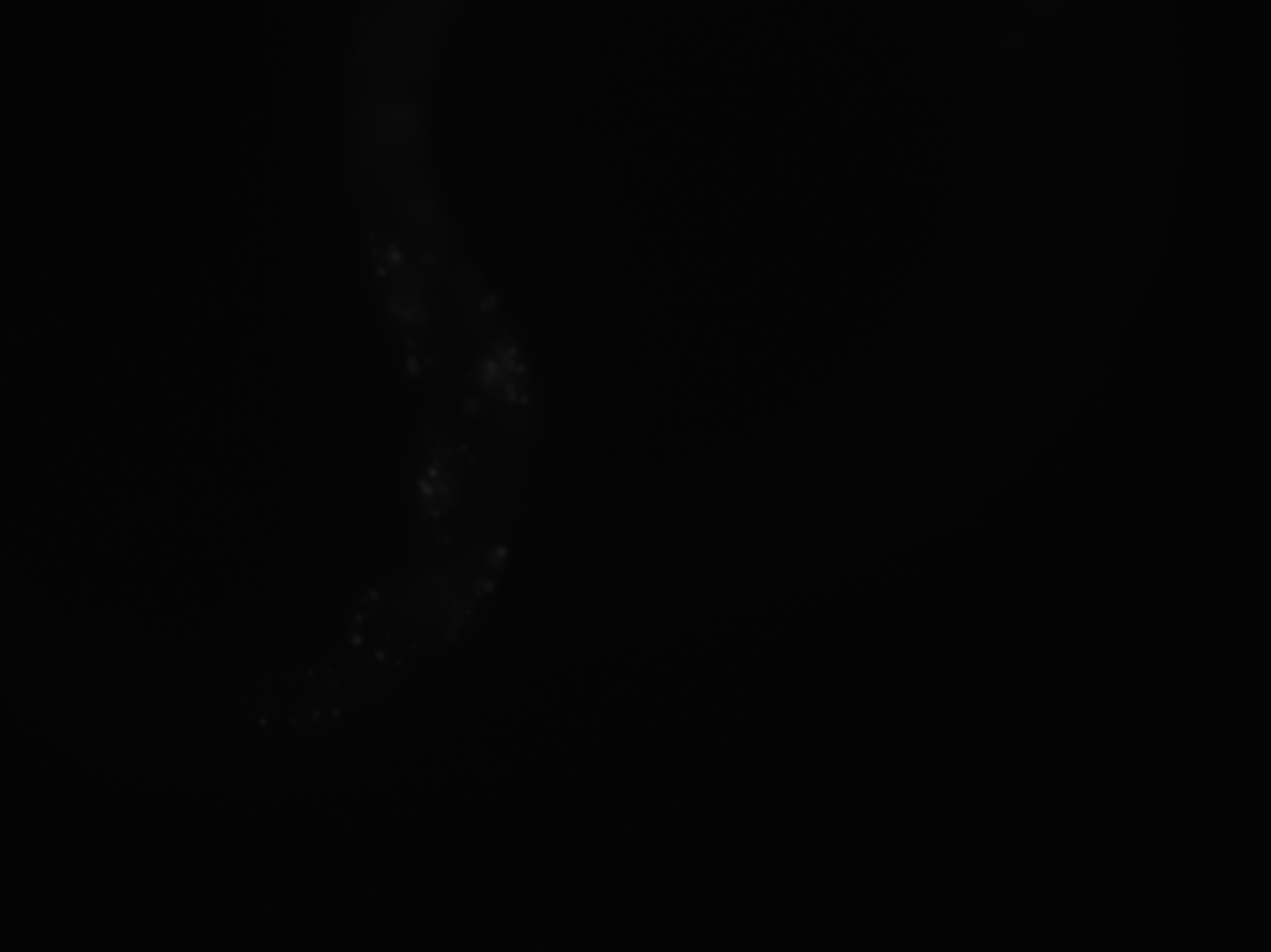

Supplement: Supplementary file 3 — Source data Fig. 2 [file 44319_2025_493_MOESM3_ESM.zip › Figure2/Fig2F/Experiment-01prp40_NR.tif_files/Experiment-01_z10c1x0-1388y0-1040.tif]

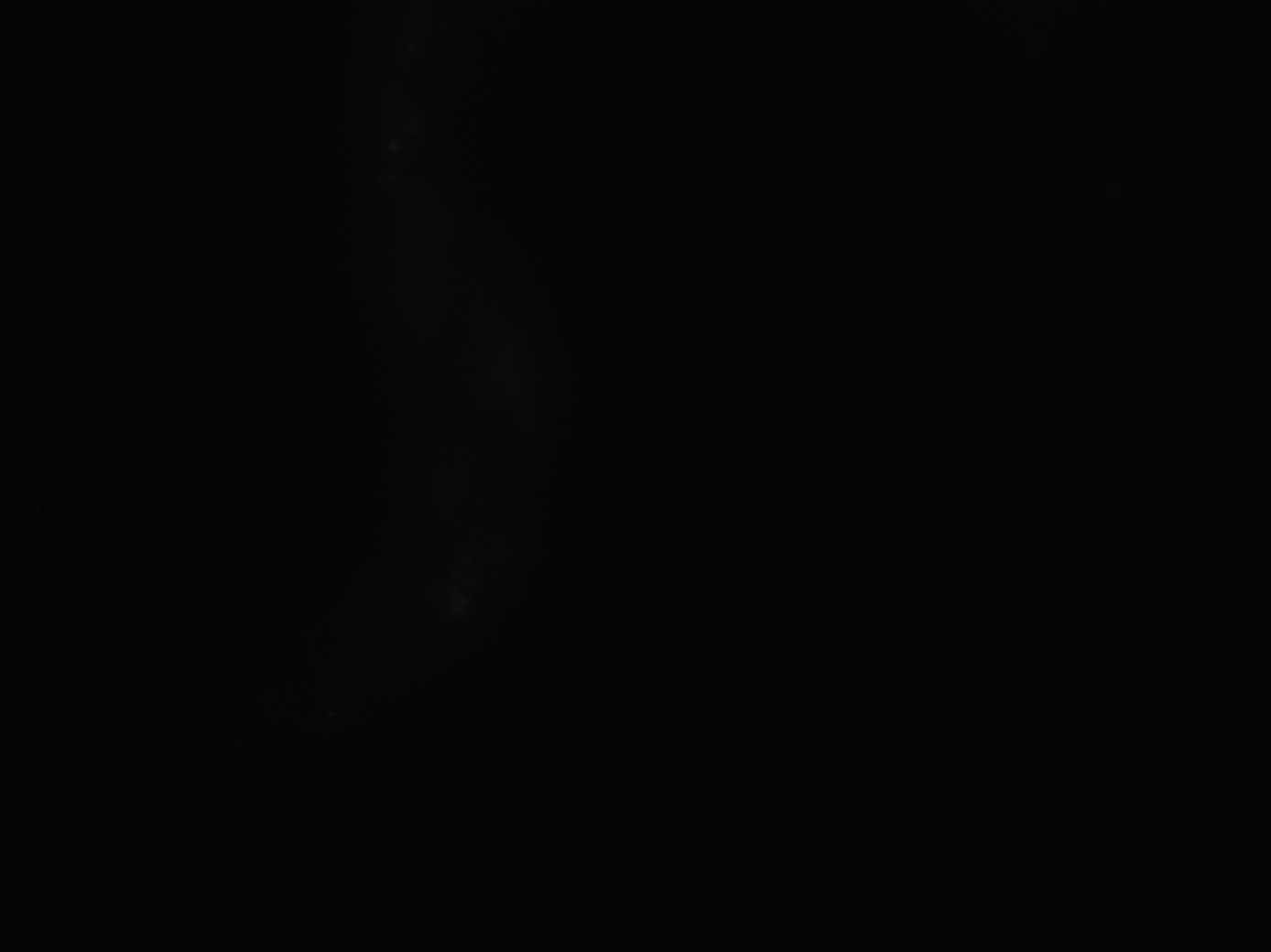

Supplement: Supplementary file 3 — Source data Fig. 2 [file 44319_2025_493_MOESM3_ESM.zip › Figure2/Fig2F/Experiment-01prp40_NR.tif_files/Experiment-01_z2c1x0-1388y0-1040.tif]

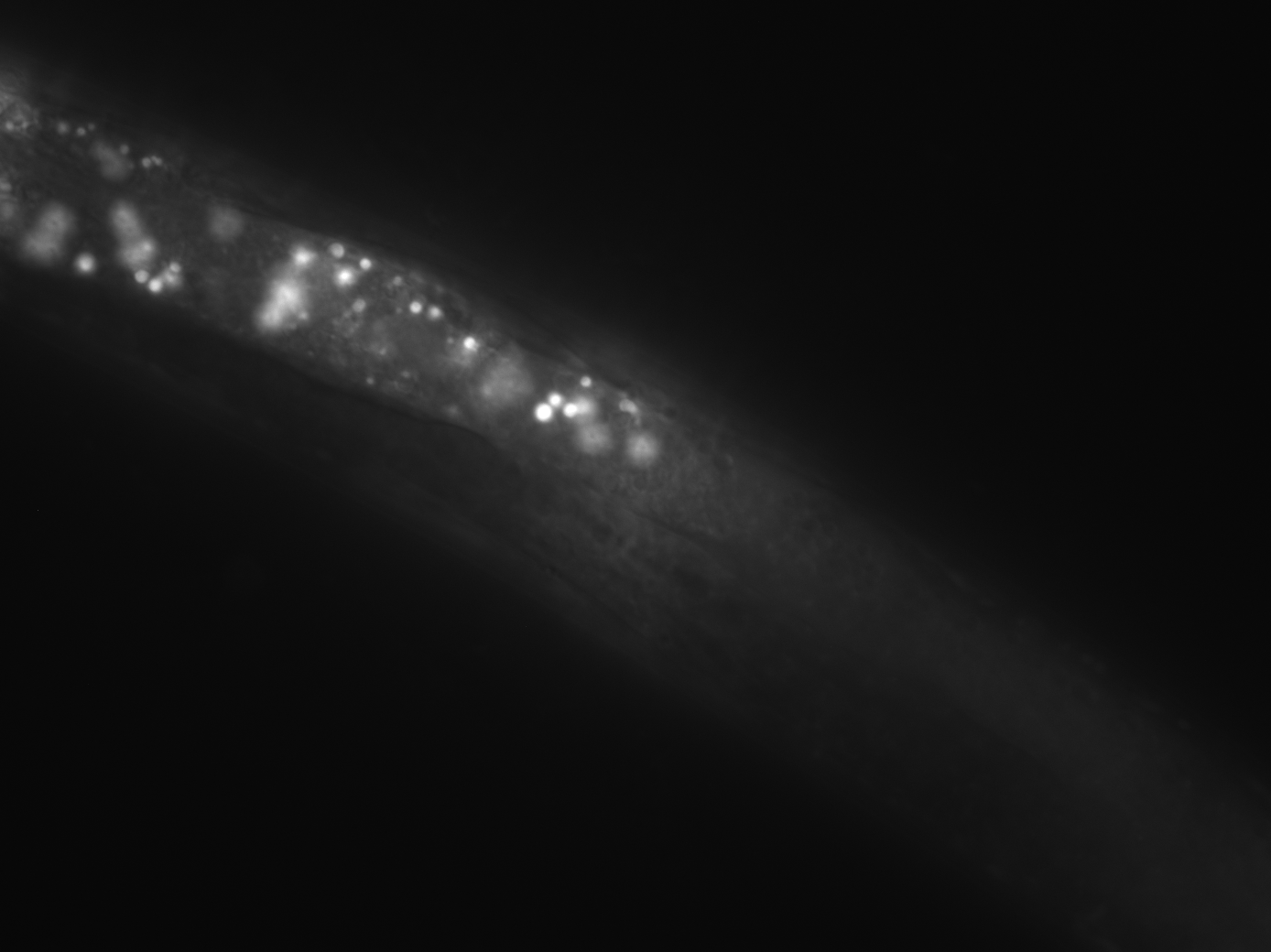

Supplement: Supplementary file 3 — Source data Fig. 2 [file 44319_2025_493_MOESM3_ESM.zip › Figure2/Fig2D/Experiment-383_VC_exc7_mbl1.tif_files/Experiment-383_z1c0x0-1388y0-1040.tif]

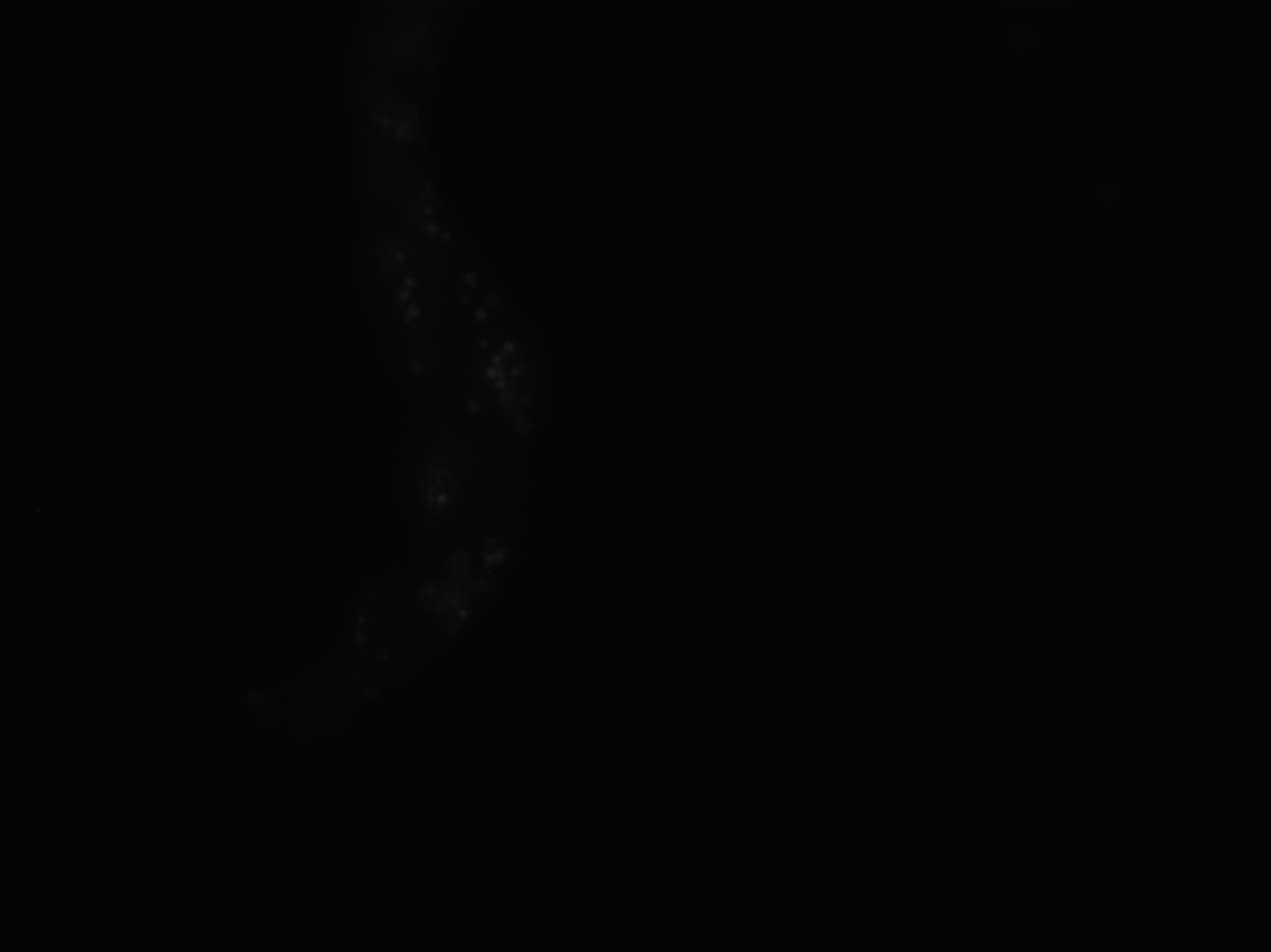

Supplement: Supplementary file 3 — Source data Fig. 2 [file 44319_2025_493_MOESM3_ESM.zip › Figure2/Fig2F/Experiment-01prp40_NR.tif_files/Experiment-01_z7c1x0-1388y0-1040.tif]

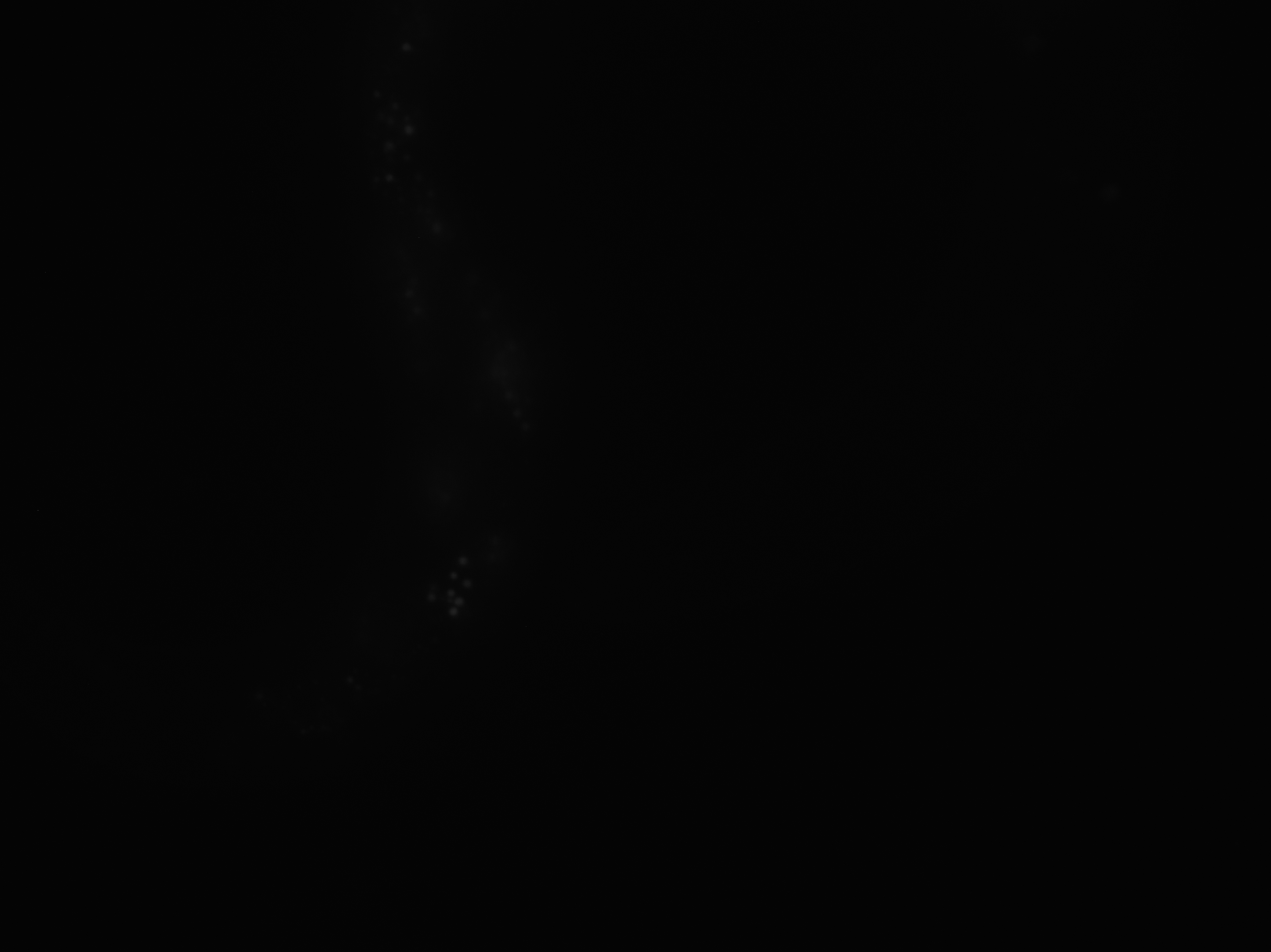

Supplement: Supplementary file 3 — Source data Fig. 2 [file 44319_2025_493_MOESM3_ESM.zip › Figure2/Fig2F/Experiment-01prp40_NR.tif_files/Experiment-01_z4c1x0-1388y0-1040.tif]

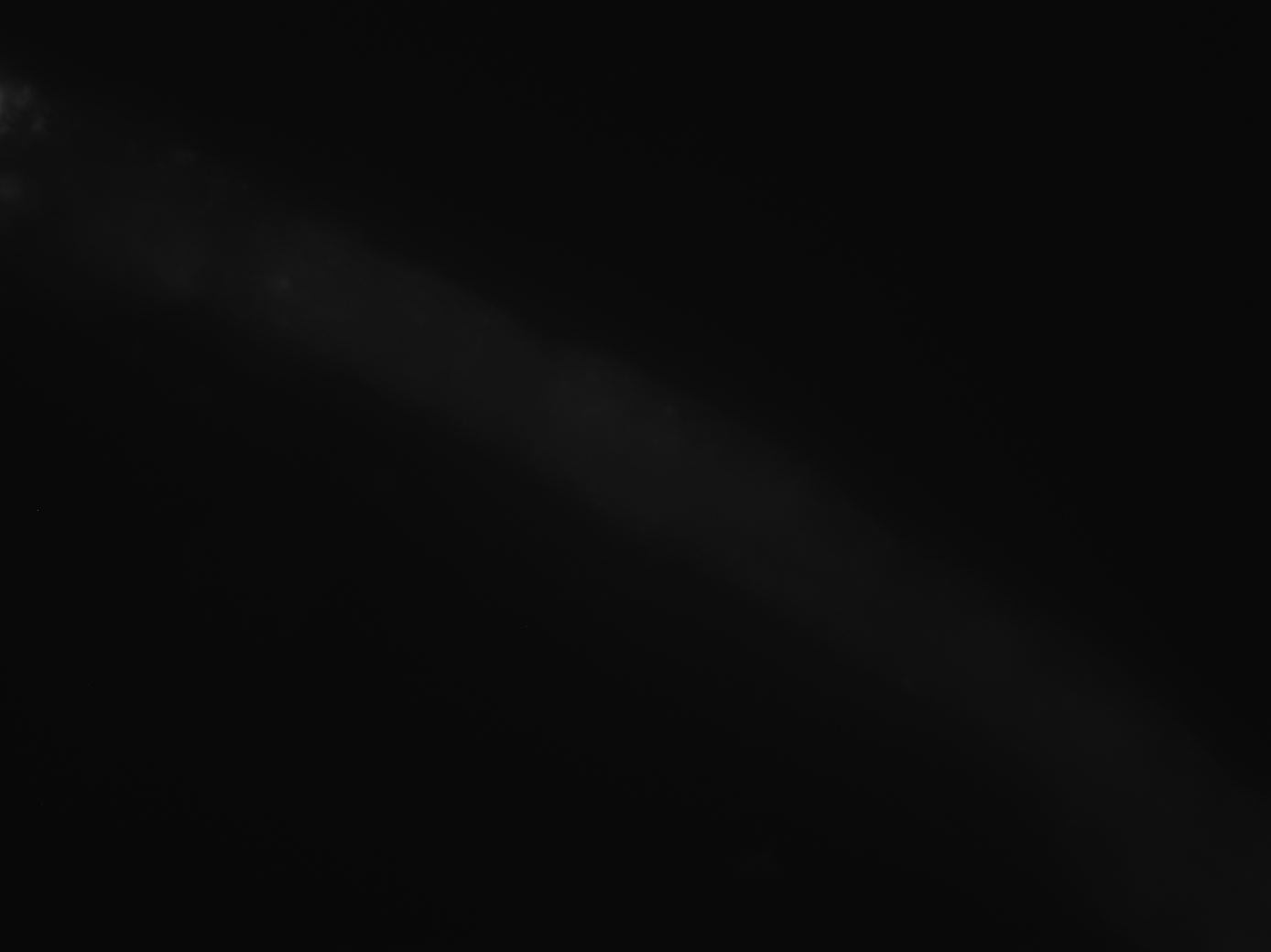

Supplement: Supplementary file 3 — Source data Fig. 2 [file 44319_2025_493_MOESM3_ESM.zip › Figure2/Fig2D/Experiment-383_VC_exc7_mbl1.tif_files/Experiment-383_z8c1x0-1388y0-1040.tif]

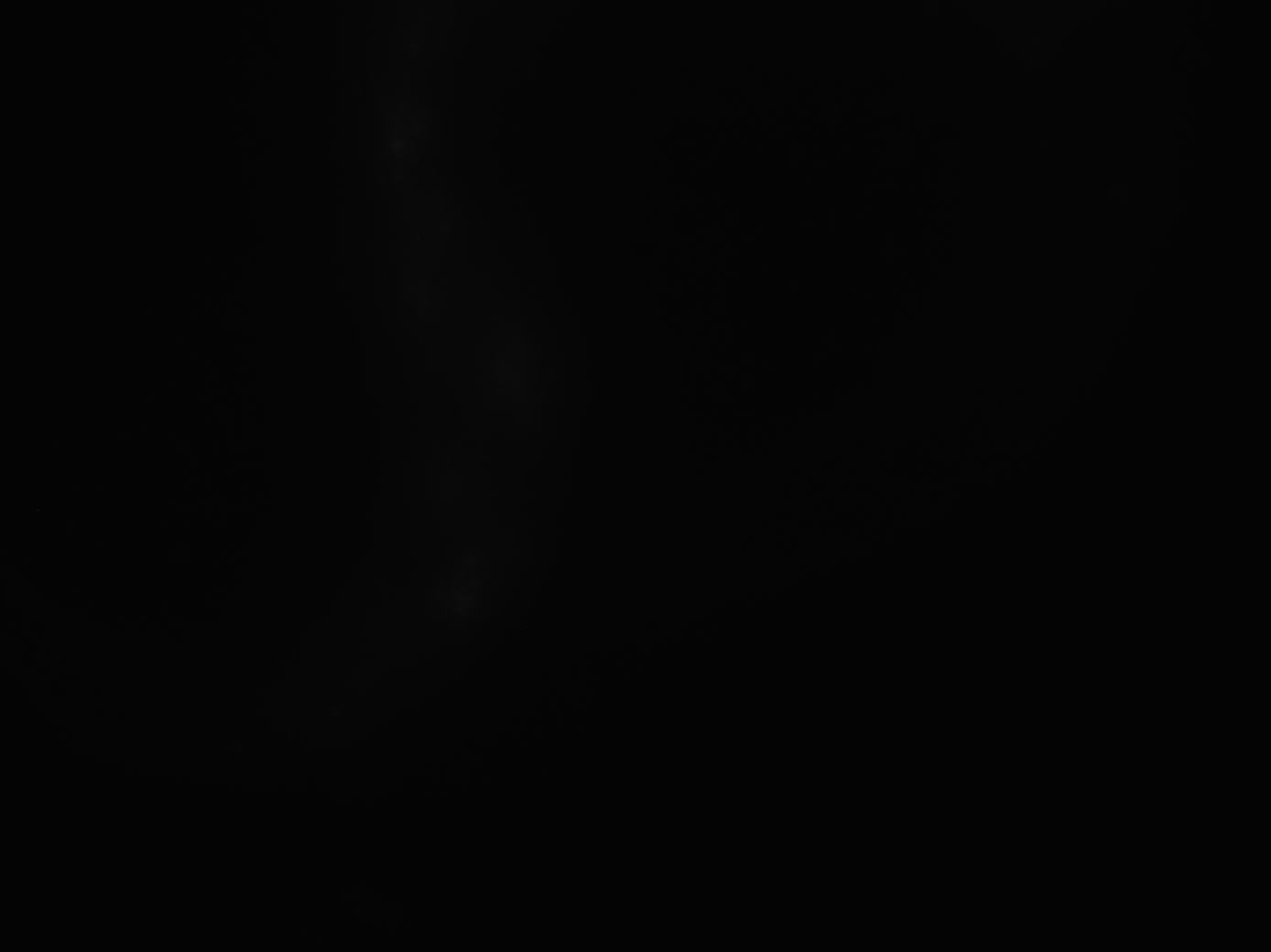

Supplement: Supplementary file 3 — Source data Fig. 2 [file 44319_2025_493_MOESM3_ESM.zip › Figure2/Fig2F/Experiment-01prp40_NR.tif_files/Experiment-01_z0c1x0-1388y0-1040.tif]

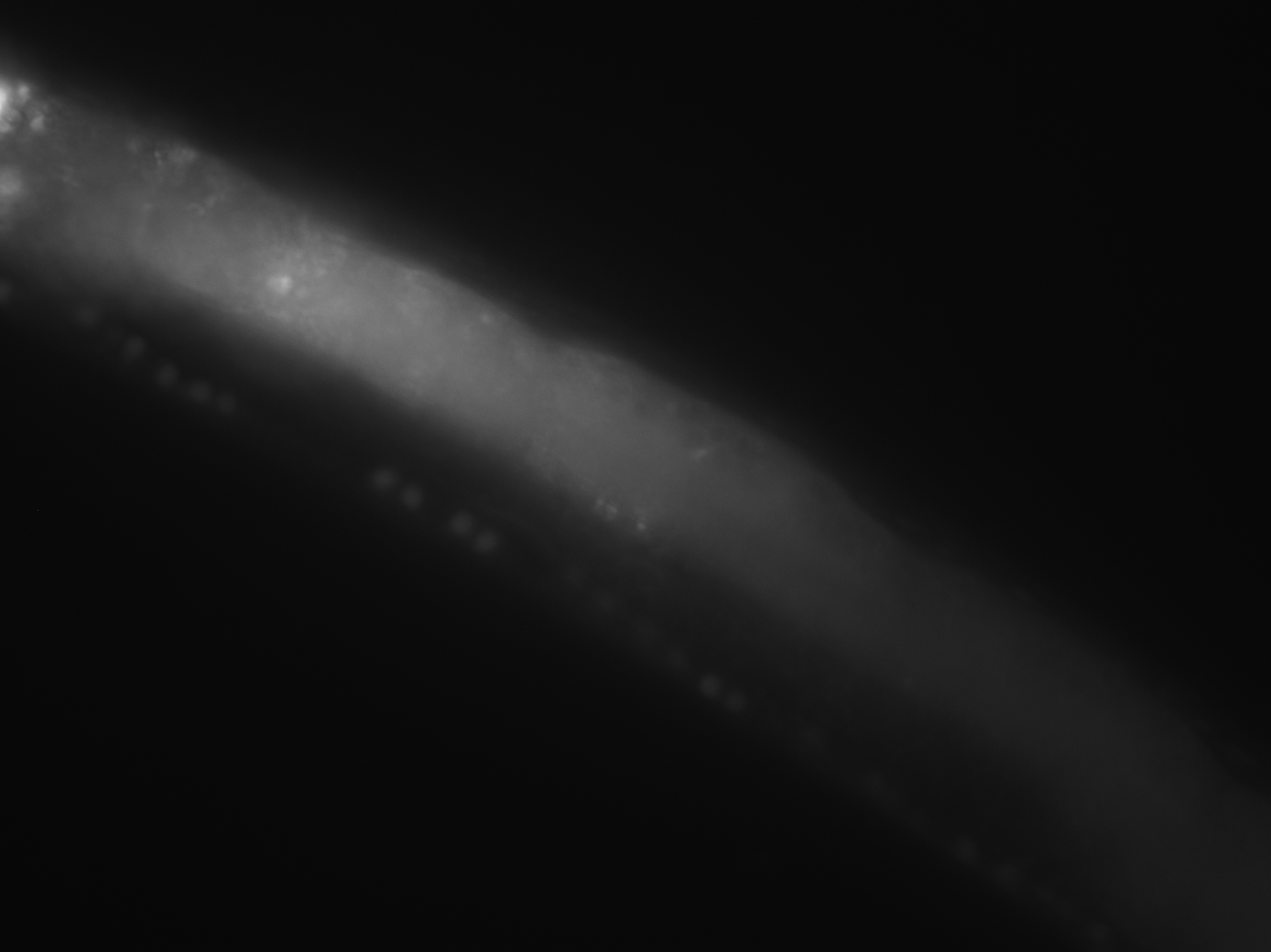

Supplement: Supplementary file 3 — Source data Fig. 2 [file 44319_2025_493_MOESM3_ESM.zip › Figure2/Fig2D/Experiment-383_VC_exc7_mbl1.tif_files/Experiment-383_z9c0x0-1388y0-1040.tif]

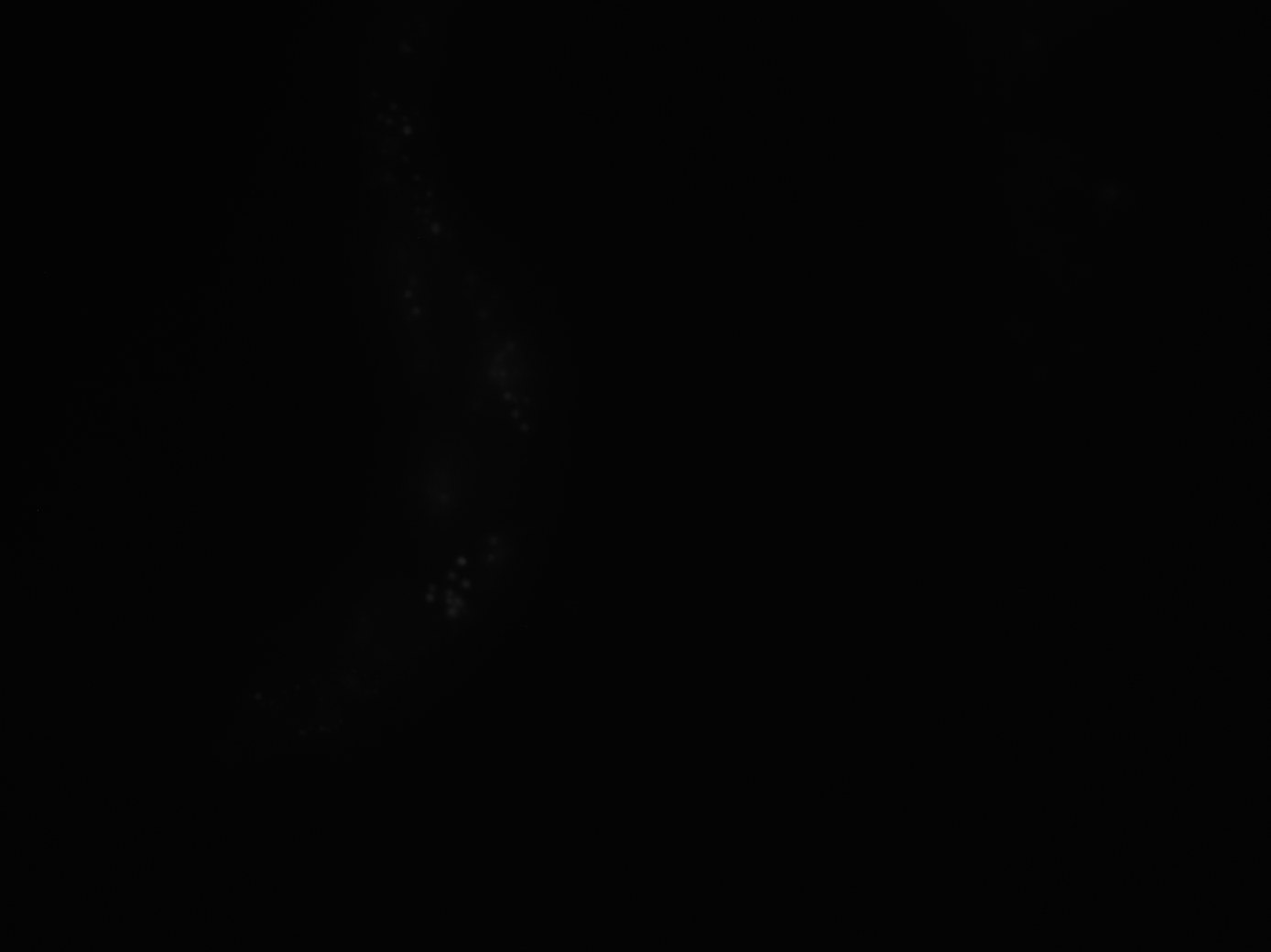

Supplement: Supplementary file 3 — Source data Fig. 2 [file 44319_2025_493_MOESM3_ESM.zip › Figure2/Fig2F/Experiment-01prp40_NR.tif_files/Experiment-01_z5c1x0-1388y0-1040.tif]

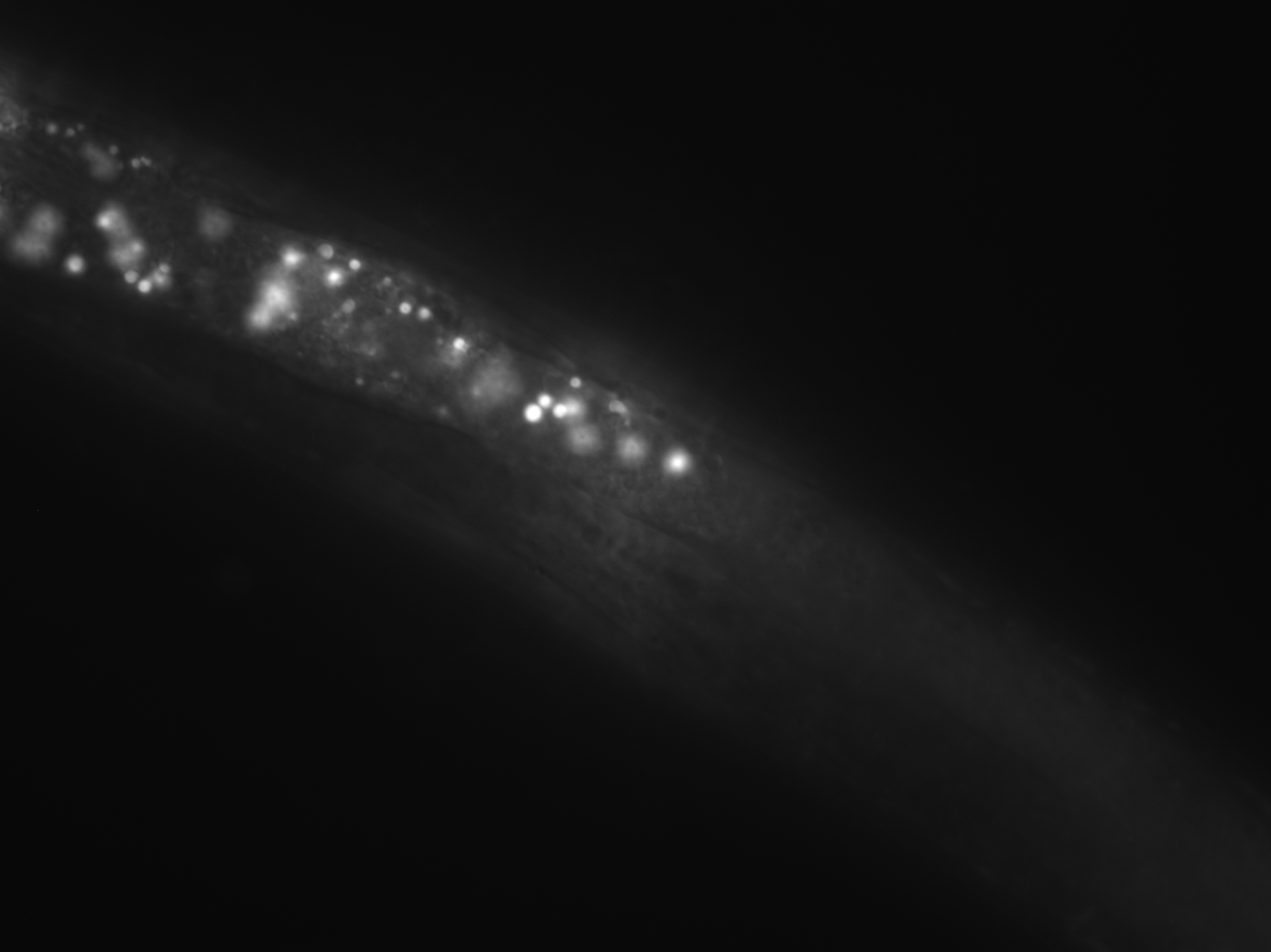

Supplement: Supplementary file 3 — Source data Fig. 2 [file 44319_2025_493_MOESM3_ESM.zip › Figure2/Fig2D/Experiment-383_VC_exc7_mbl1.tif_files/Experiment-383_z0c0x0-1388y0-1040.tif]

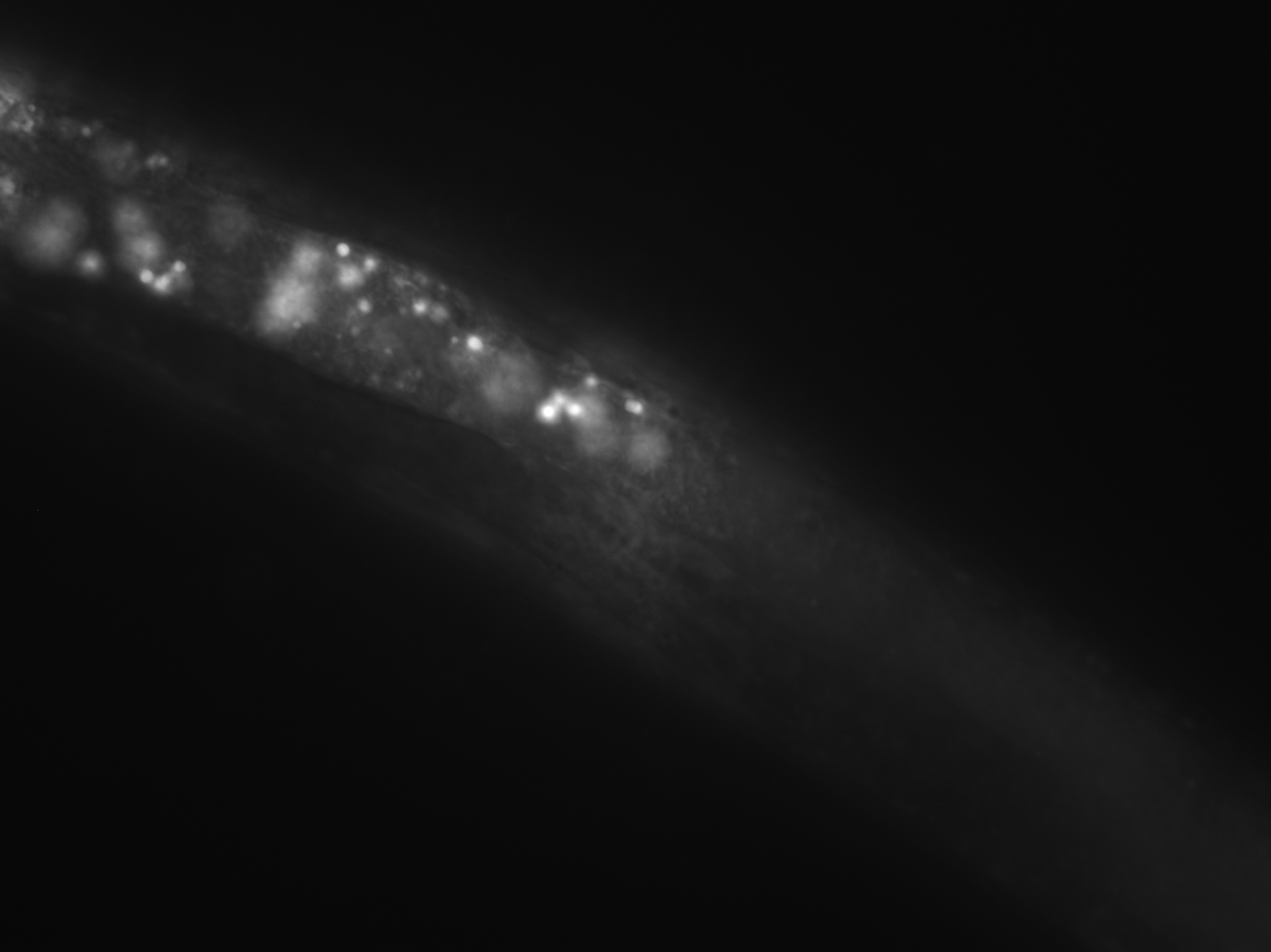

Supplement: Supplementary file 3 — Source data Fig. 2 [file 44319_2025_493_MOESM3_ESM.zip › Figure2/Fig2D/Experiment-383_VC_exc7_mbl1.tif_files/Experiment-383_z2c0x0-1388y0-1040.tif]

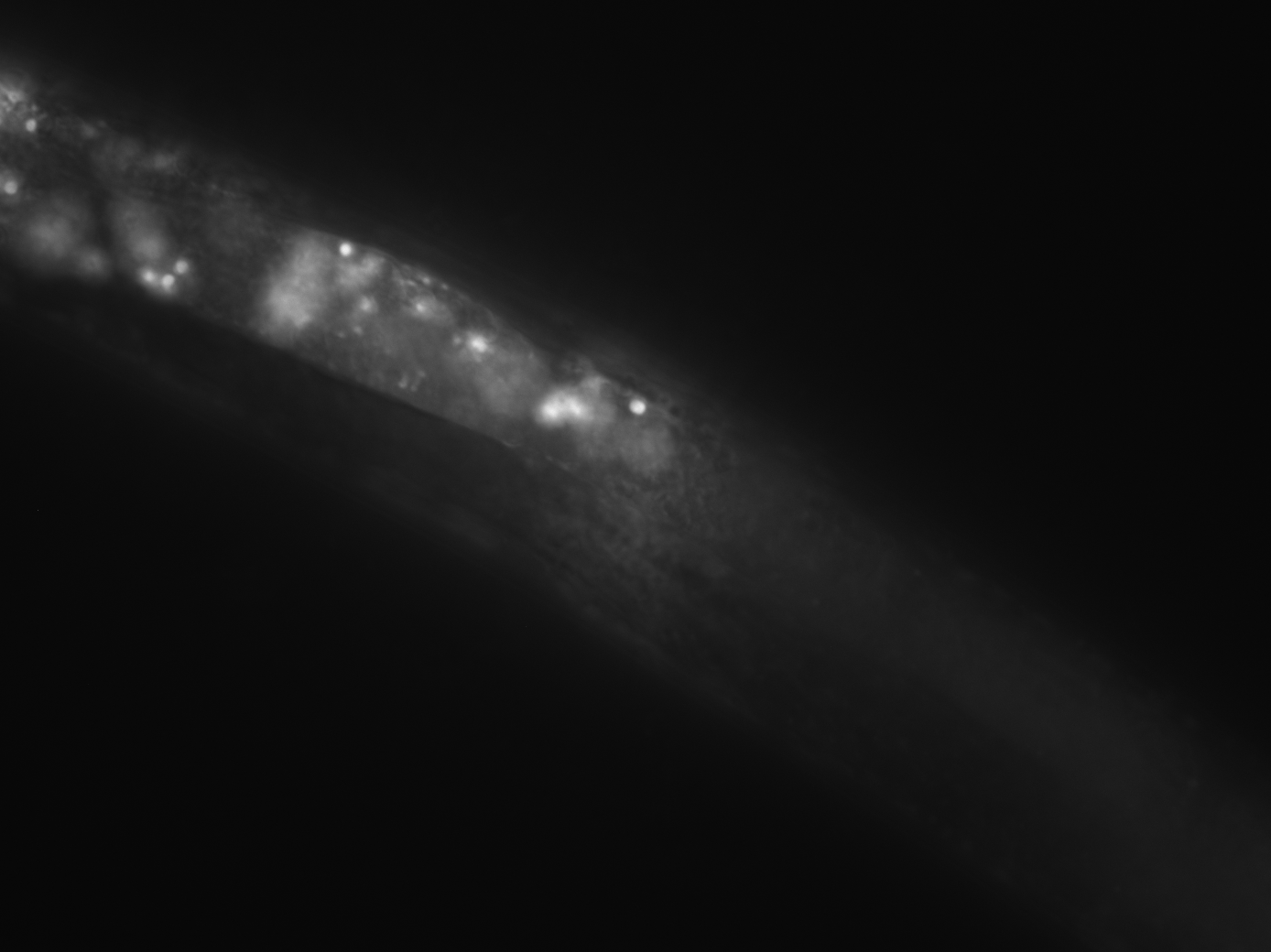

Supplement: Supplementary file 3 — Source data Fig. 2 [file 44319_2025_493_MOESM3_ESM.zip › Figure2/Fig2D/Experiment-383_VC_exc7_mbl1.tif_files/Experiment-383_z3c0x0-1388y0-1040.tif]

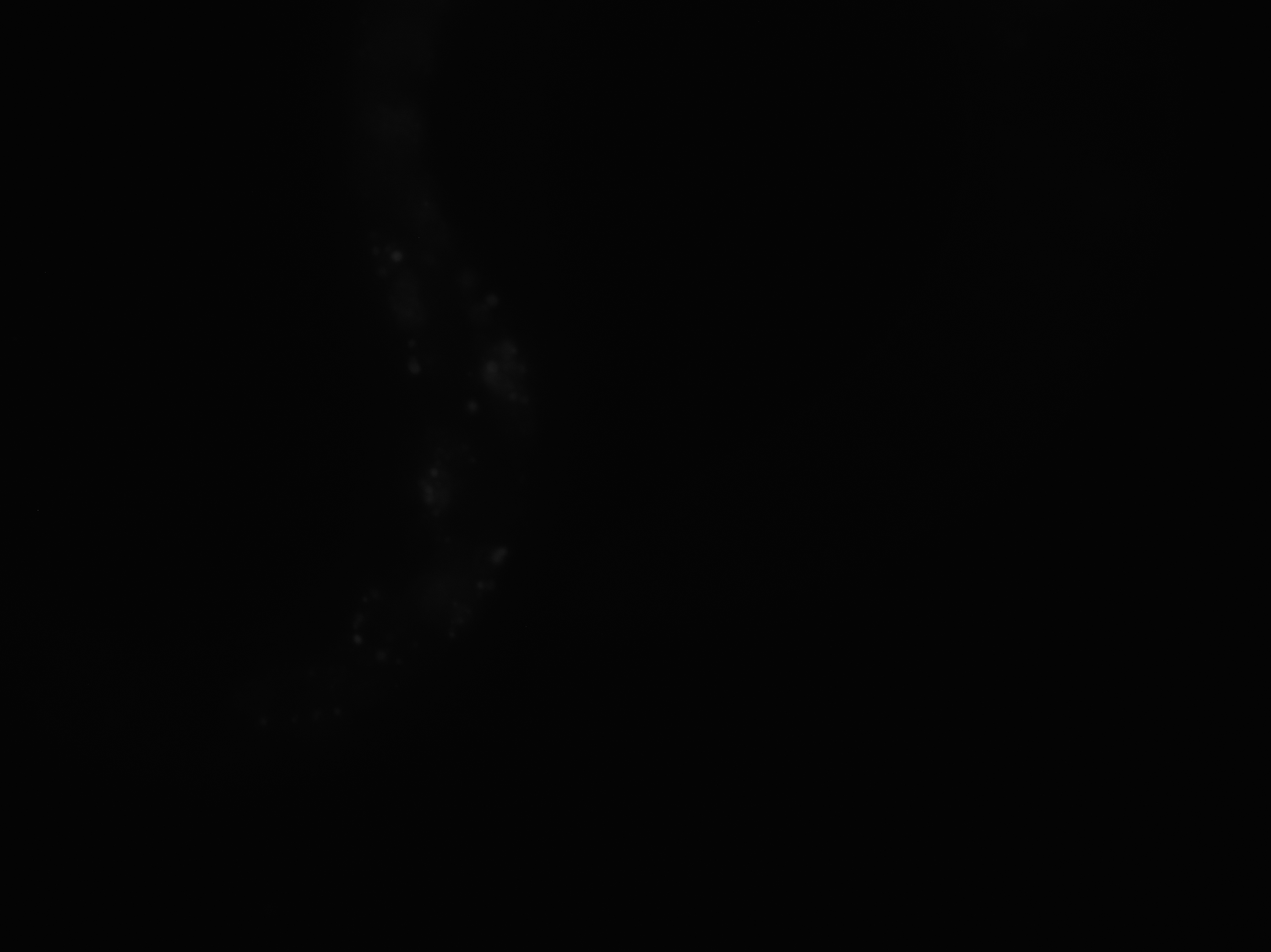

Supplement: Supplementary file 3 — Source data Fig. 2 [file 44319_2025_493_MOESM3_ESM.zip › Figure2/Fig2F/Experiment-01prp40_NR.tif_files/Experiment-01_z9c1x0-1388y0-1040.tif]

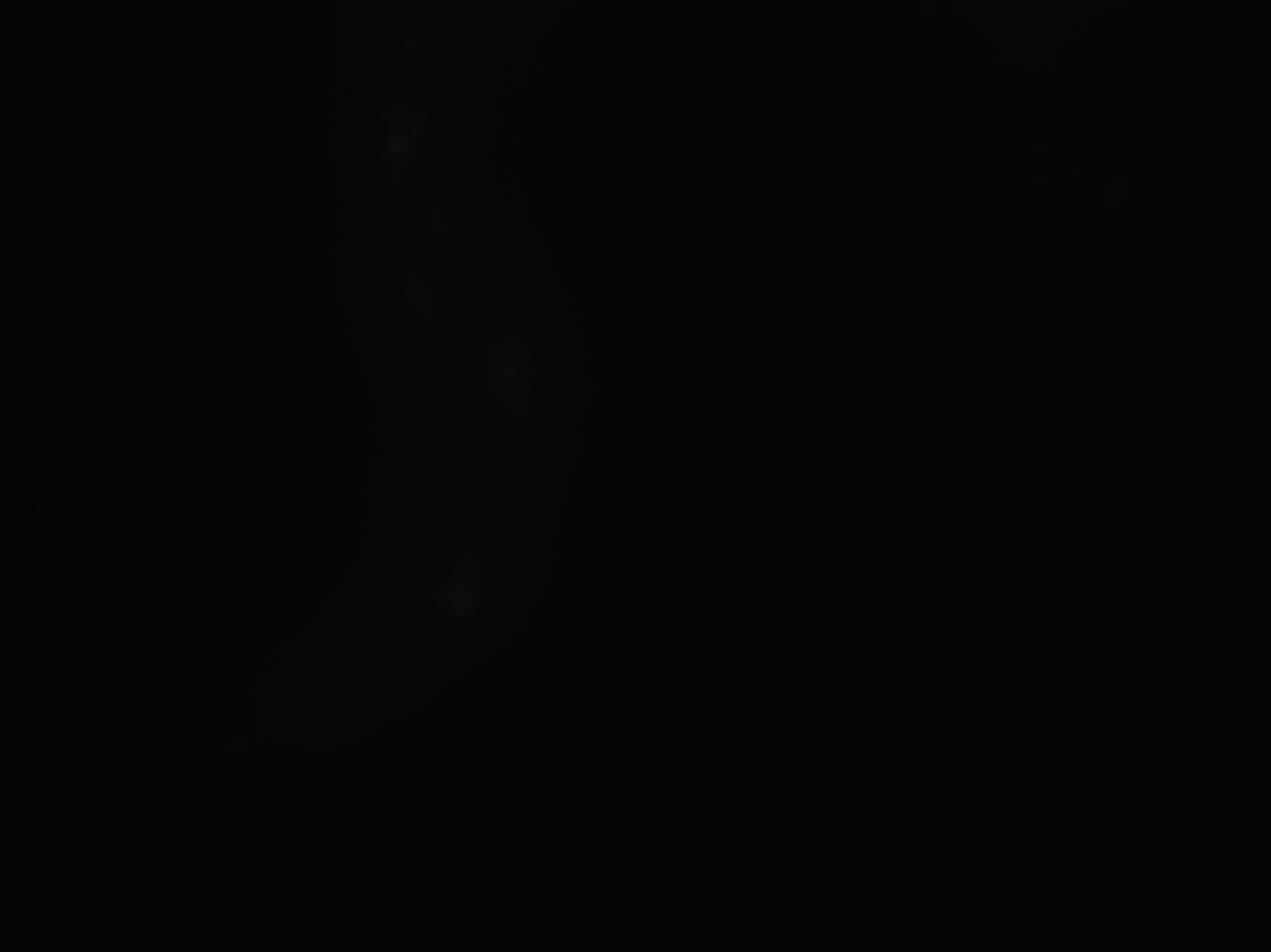

Supplement: Supplementary file 3 — Source data Fig. 2 [file 44319_2025_493_MOESM3_ESM.zip › Figure2/Fig2F/Experiment-01prp40_NR.tif_files/Experiment-01_z1c1x0-1388y0-1040.tif]

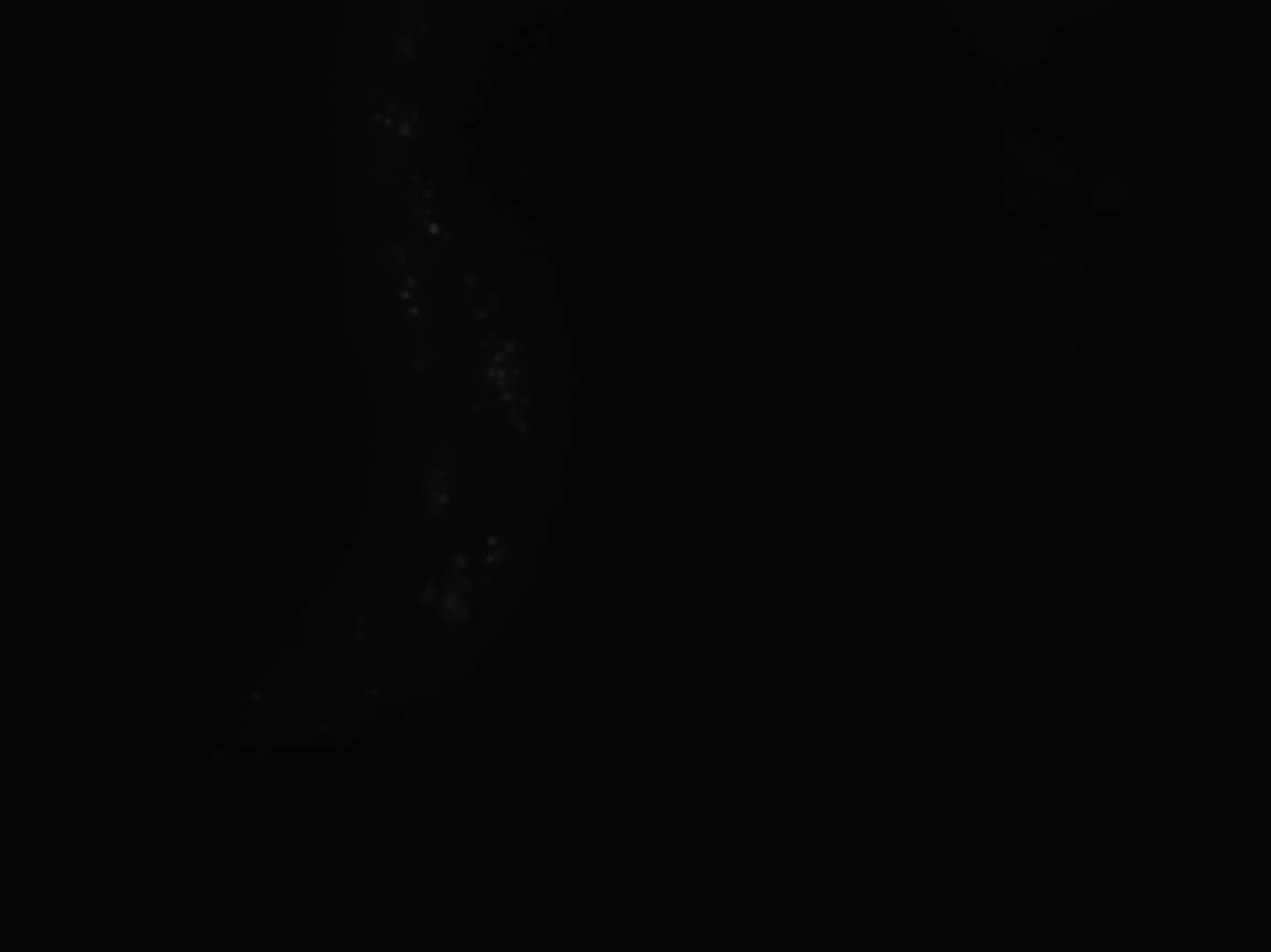

Supplement: Supplementary file 3 — Source data Fig. 2 [file 44319_2025_493_MOESM3_ESM.zip › Figure2/Fig2F/Experiment-01prp40_NR.tif_files/Experiment-01_z6c1x0-1388y0-1040.tif]

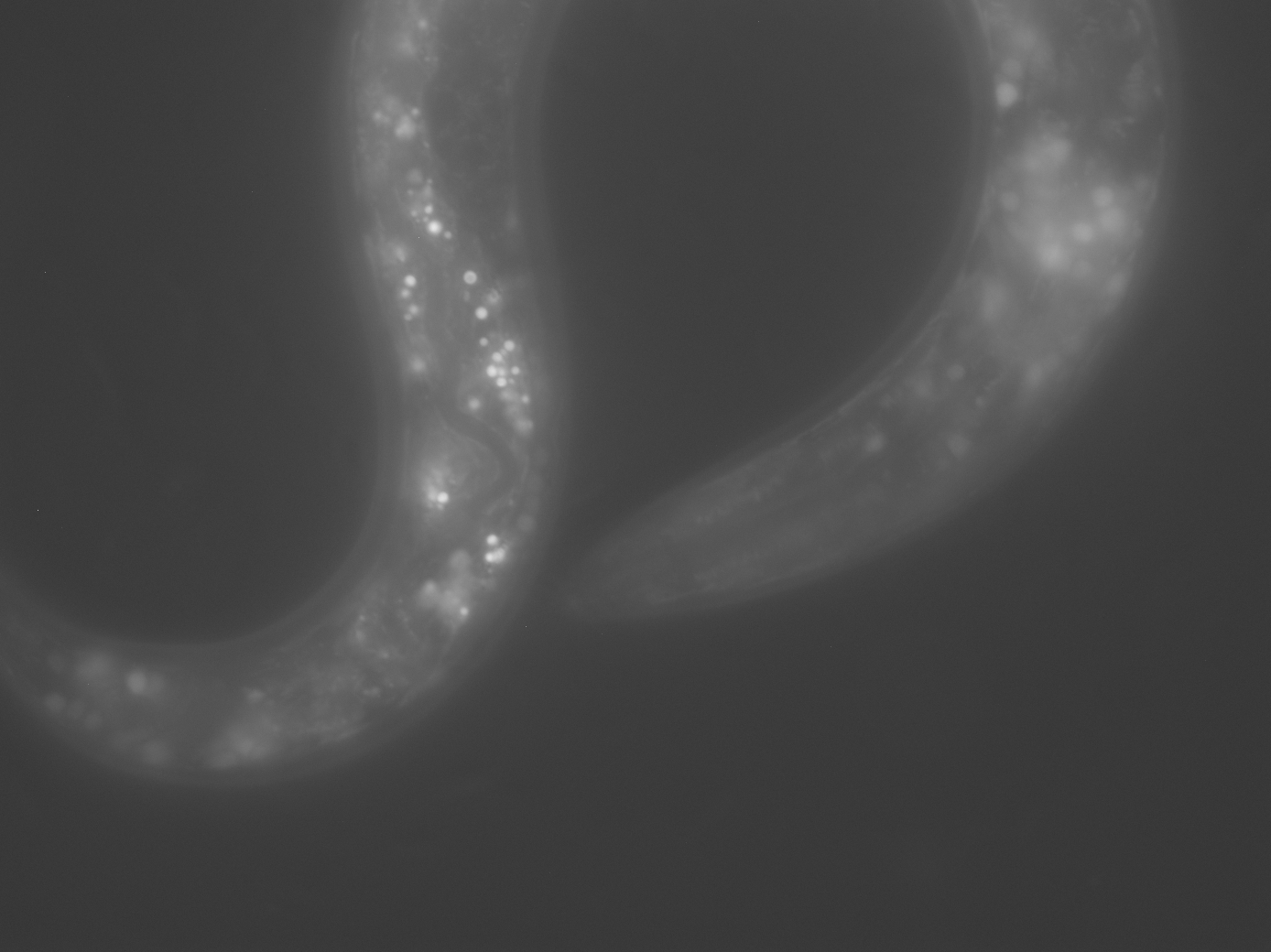

Supplement: Supplementary file 3 — Source data Fig. 2 [file 44319_2025_493_MOESM3_ESM.zip › Figure2/Fig2F/Experiment-01prp40_NR.tif_files/Experiment-01_z7c0x0-1388y0-1040.tif]

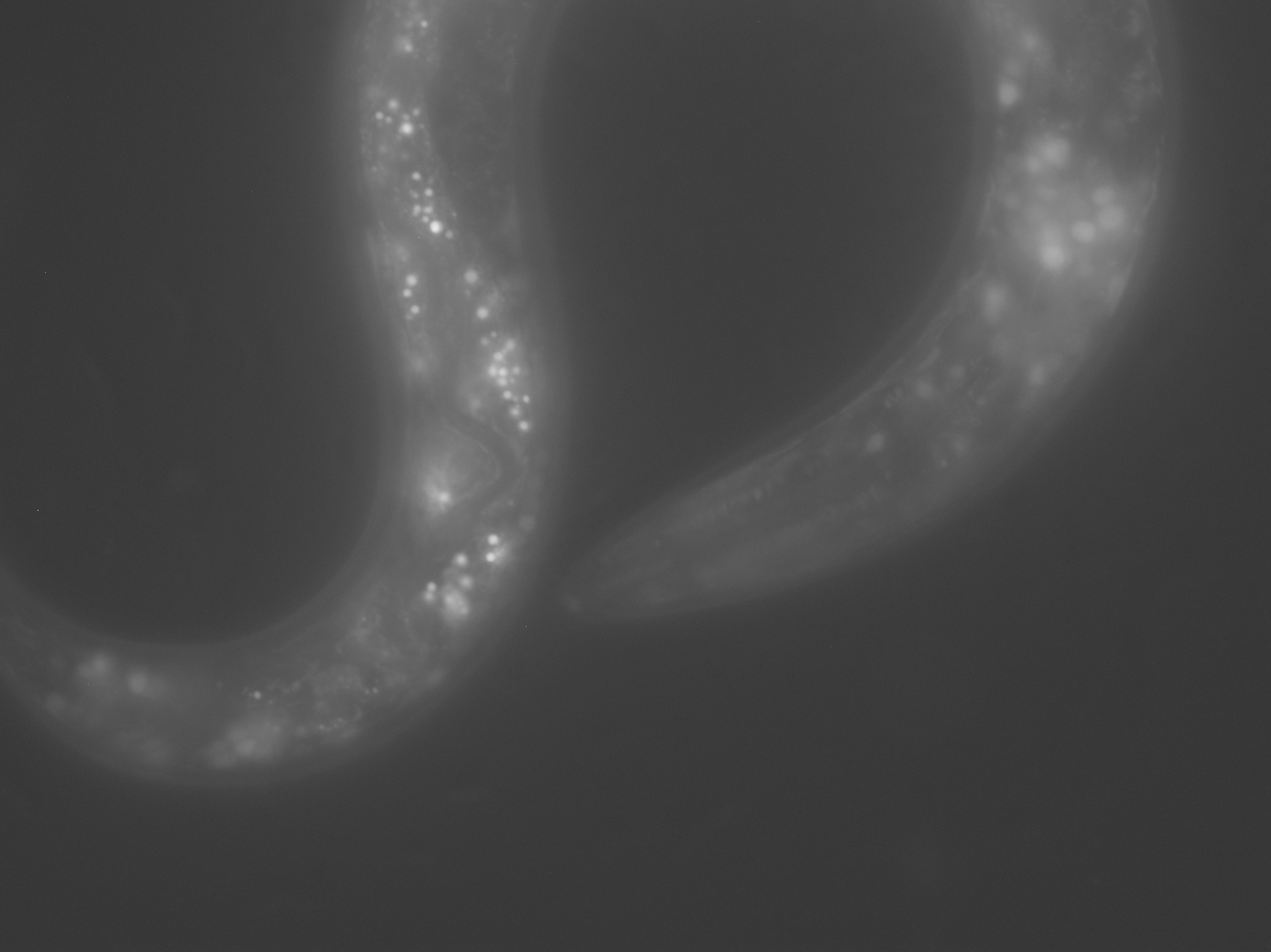

Supplement: Supplementary file 3 — Source data Fig. 2 [file 44319_2025_493_MOESM3_ESM.zip › Figure2/Fig2F/Experiment-01prp40_NR.tif_files/Experiment-01_z6c0x0-1388y0-1040.tif]

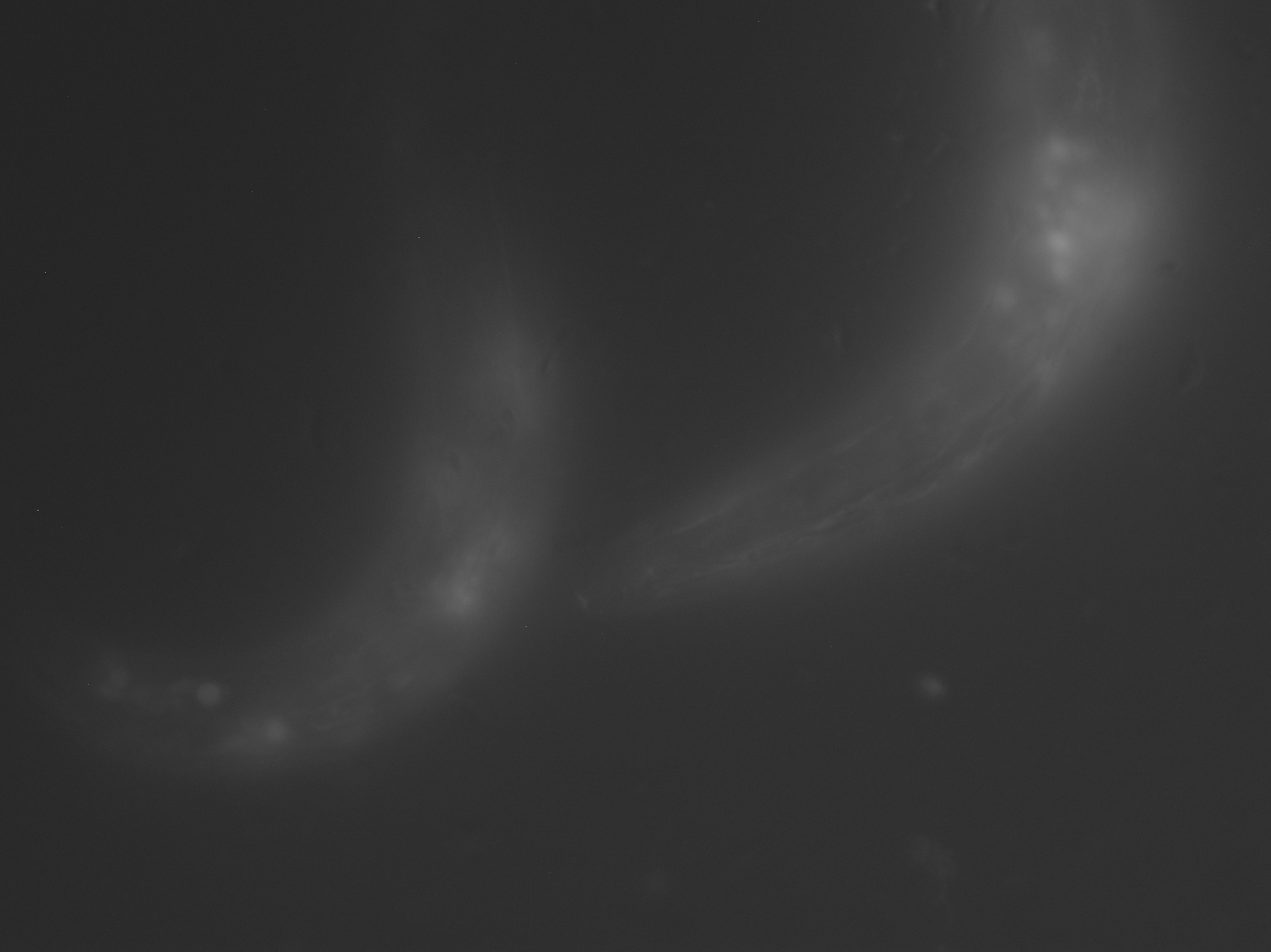

Supplement: Supplementary file 3 — Source data Fig. 2 [file 44319_2025_493_MOESM3_ESM.zip › Figure2/Fig2F/Experiment-01prp40_NR.tif_files/Experiment-01_z1c0x0-1388y0-1040.tif]

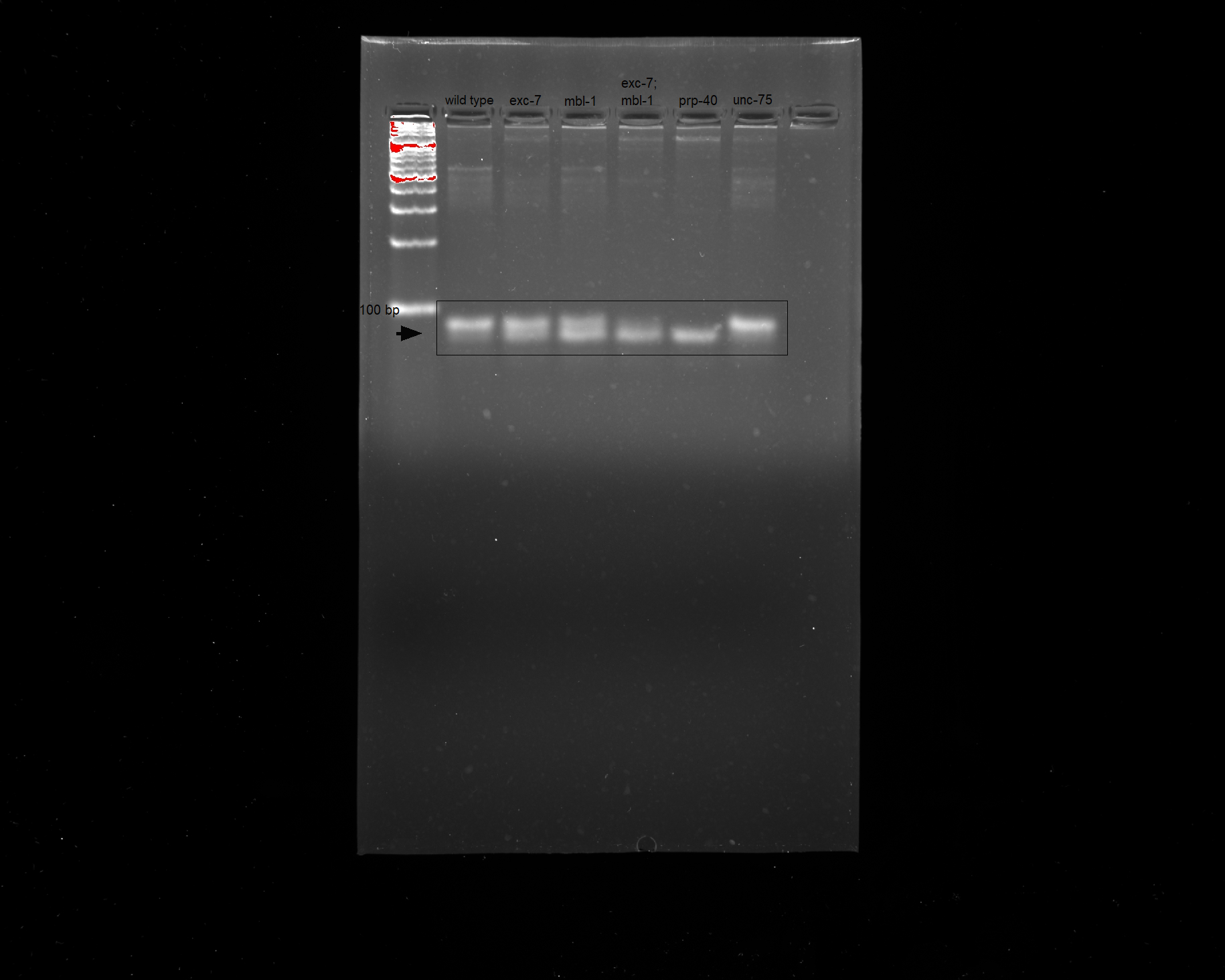

Supplement: Supplementary file 3 — Source data Fig. 2 [file 44319_2025_493_MOESM3_ESM.zip › Figure2/Fig2C/RTPCR_UNC13microexon.tif]

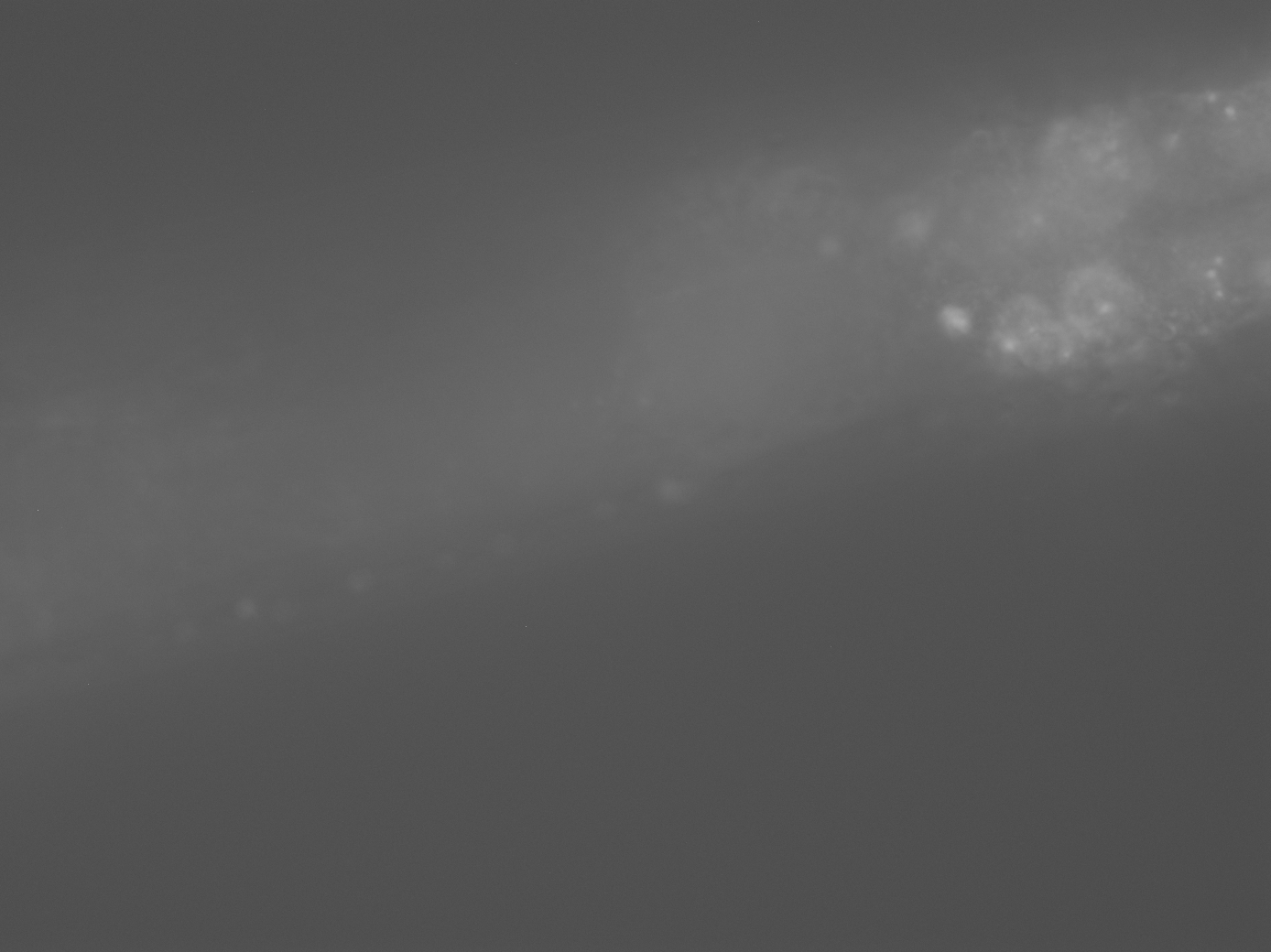

Supplement: Supplementary file 3 — Source data Fig. 2 [file 44319_2025_493_MOESM3_ESM.zip › Figure2/Fig2D/Experiment-77_VC_mbl1.tif_files/Experiment-77_z6c1x0-1388y0-1040.tif]

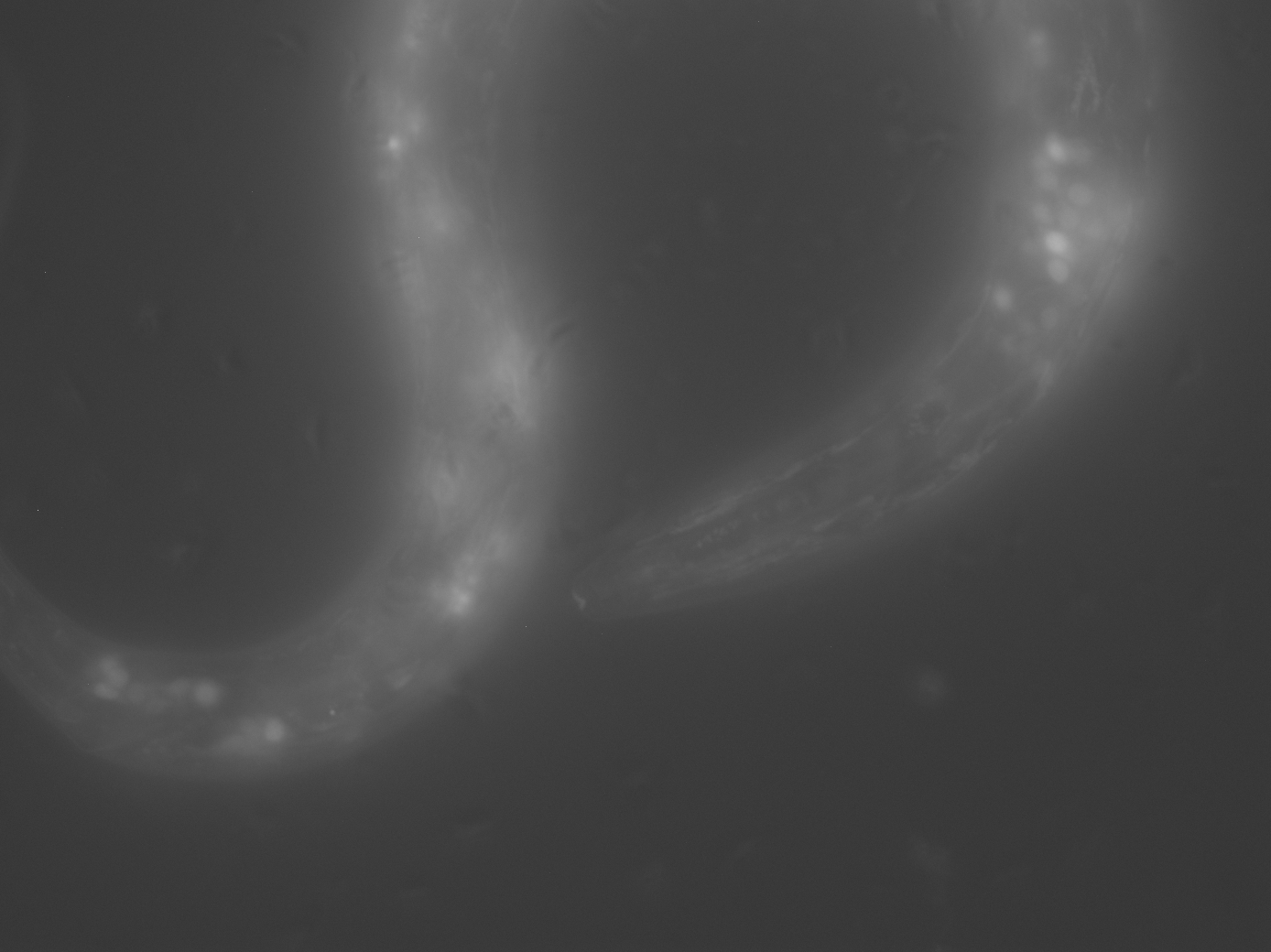

Supplement: Supplementary file 3 — Source data Fig. 2 [file 44319_2025_493_MOESM3_ESM.zip › Figure2/Fig2F/Experiment-01prp40_NR.tif_files/Experiment-01_z2c0x0-1388y0-1040.tif]

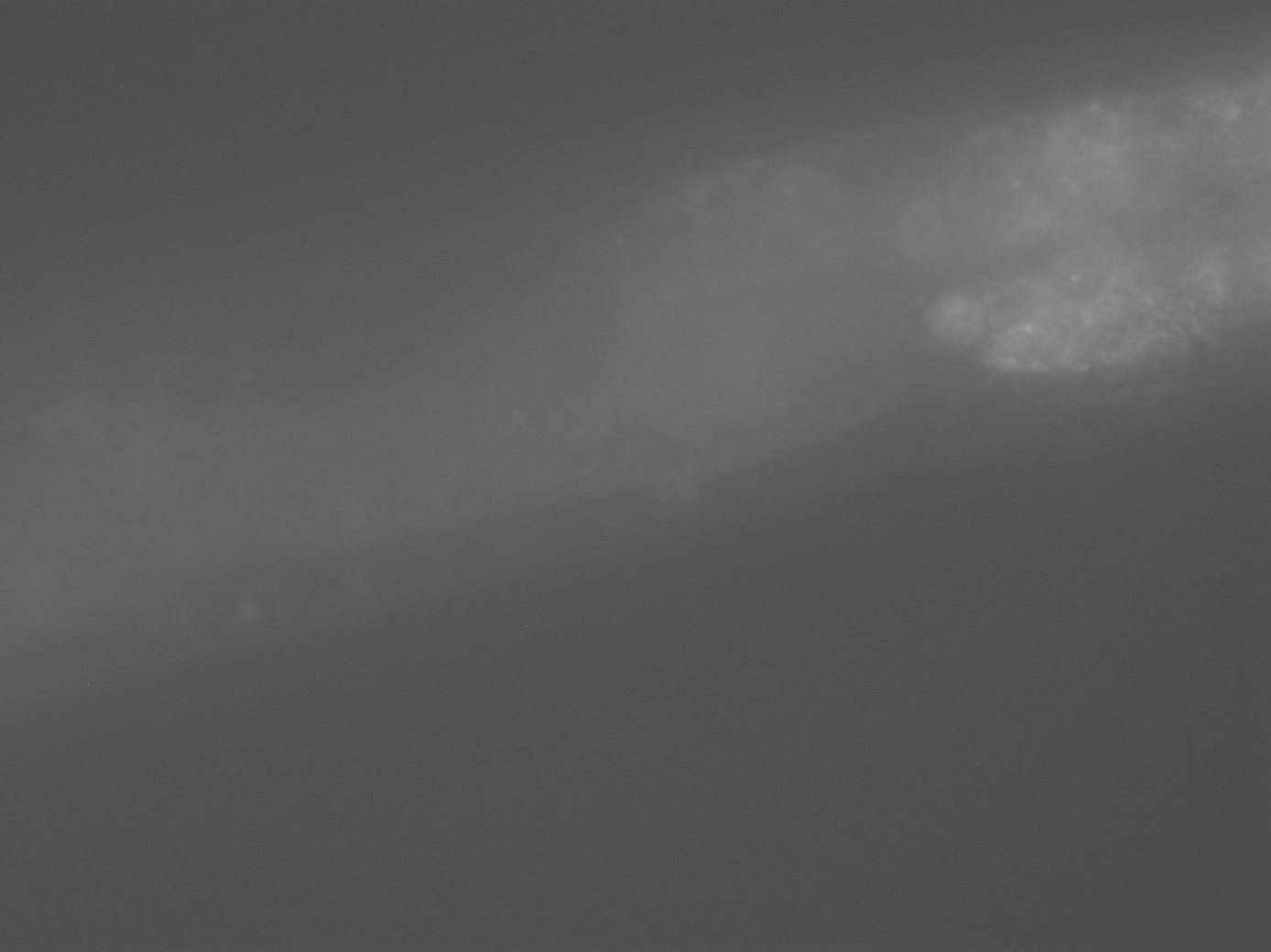

Supplement: Supplementary file 3 — Source data Fig. 2 [file 44319_2025_493_MOESM3_ESM.zip › Figure2/Fig2D/Experiment-77_VC_mbl1.tif_files/Experiment-77_z9c1x0-1388y0-1040.tif]

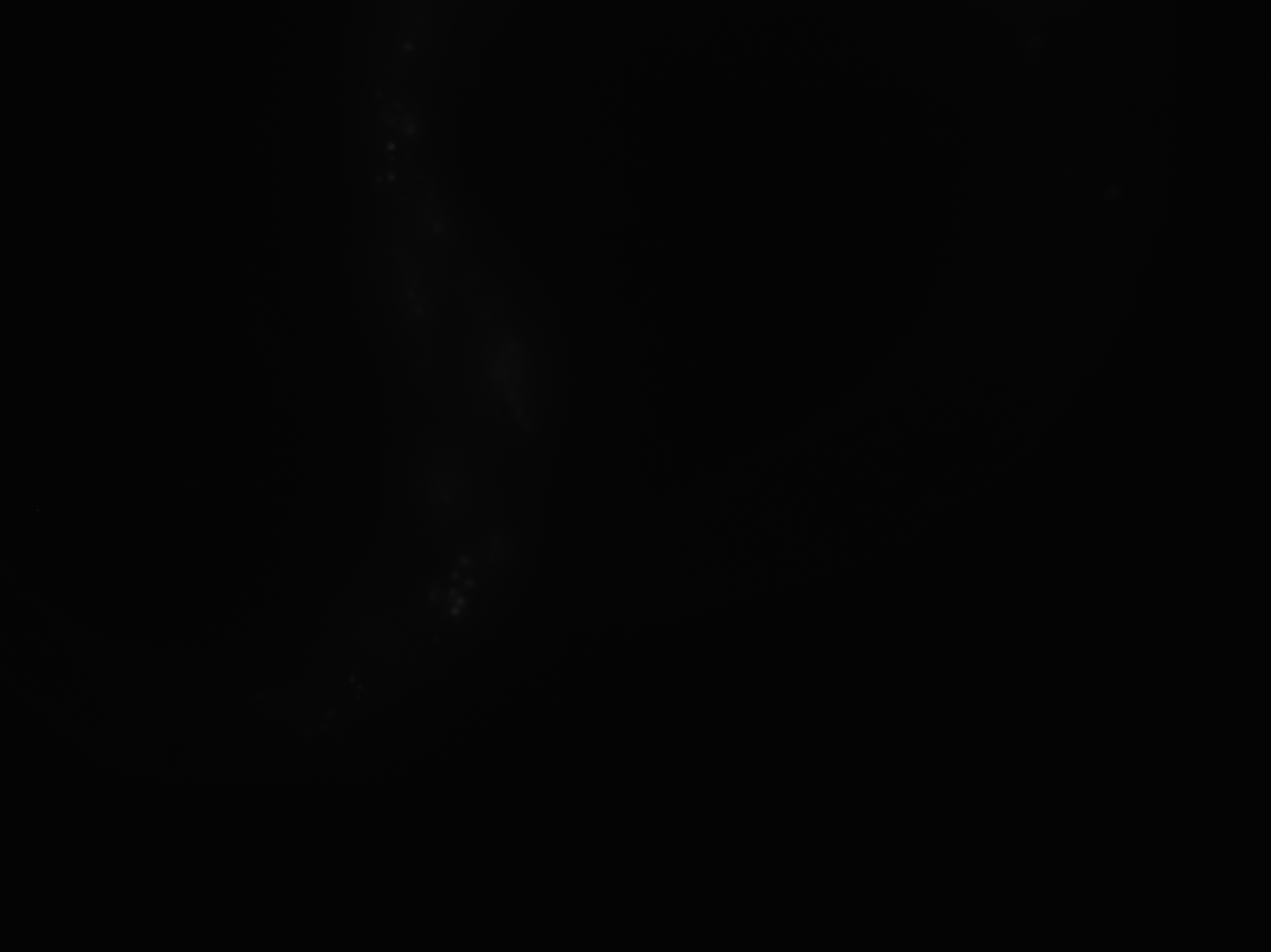

Supplement: Supplementary file 3 — Source data Fig. 2 [file 44319_2025_493_MOESM3_ESM.zip › Figure2/Fig2F/Experiment-01prp40_NR.tif_files/Experiment-01_z3c1x0-1388y0-1040.tif]

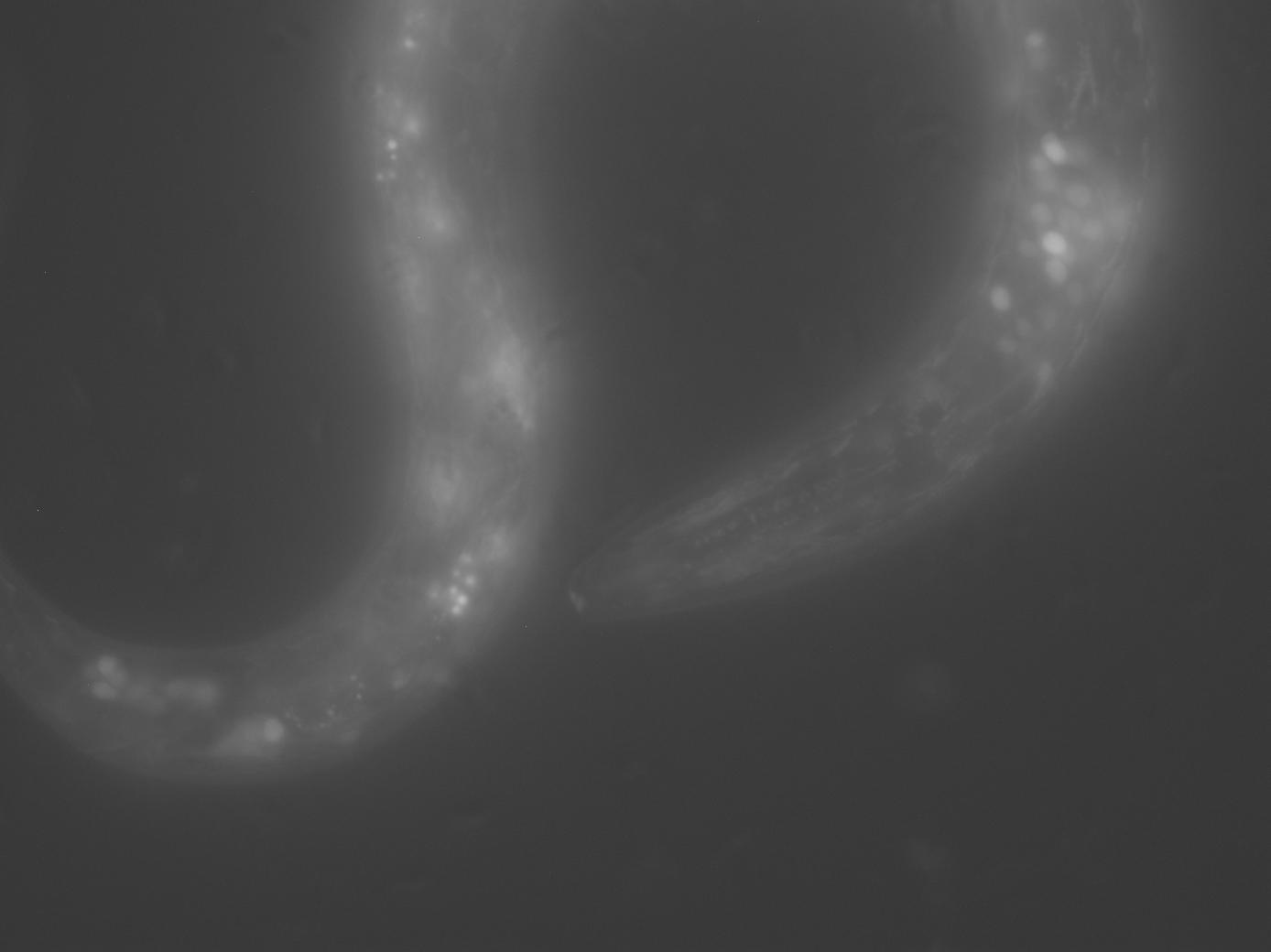

Supplement: Supplementary file 3 — Source data Fig. 2 [file 44319_2025_493_MOESM3_ESM.zip › Figure2/Fig2F/Experiment-01prp40_NR.tif_files/Experiment-01_z3c0x0-1388y0-1040.tif]

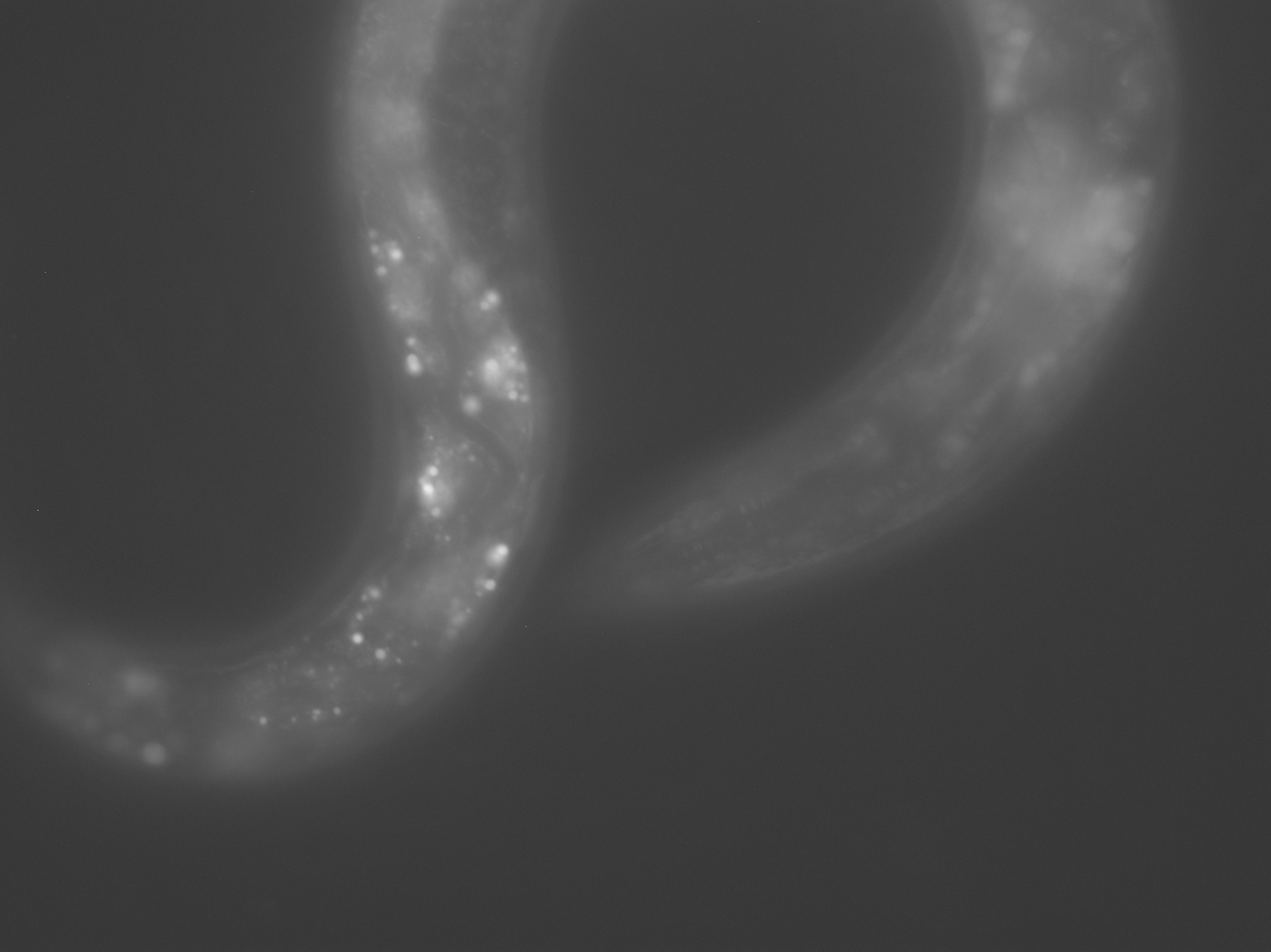

Supplement: Supplementary file 3 — Source data Fig. 2 [file 44319_2025_493_MOESM3_ESM.zip › Figure2/Fig2F/Experiment-01prp40_NR.tif_files/Experiment-01_z10c0x0-1388y0-1040.tif]

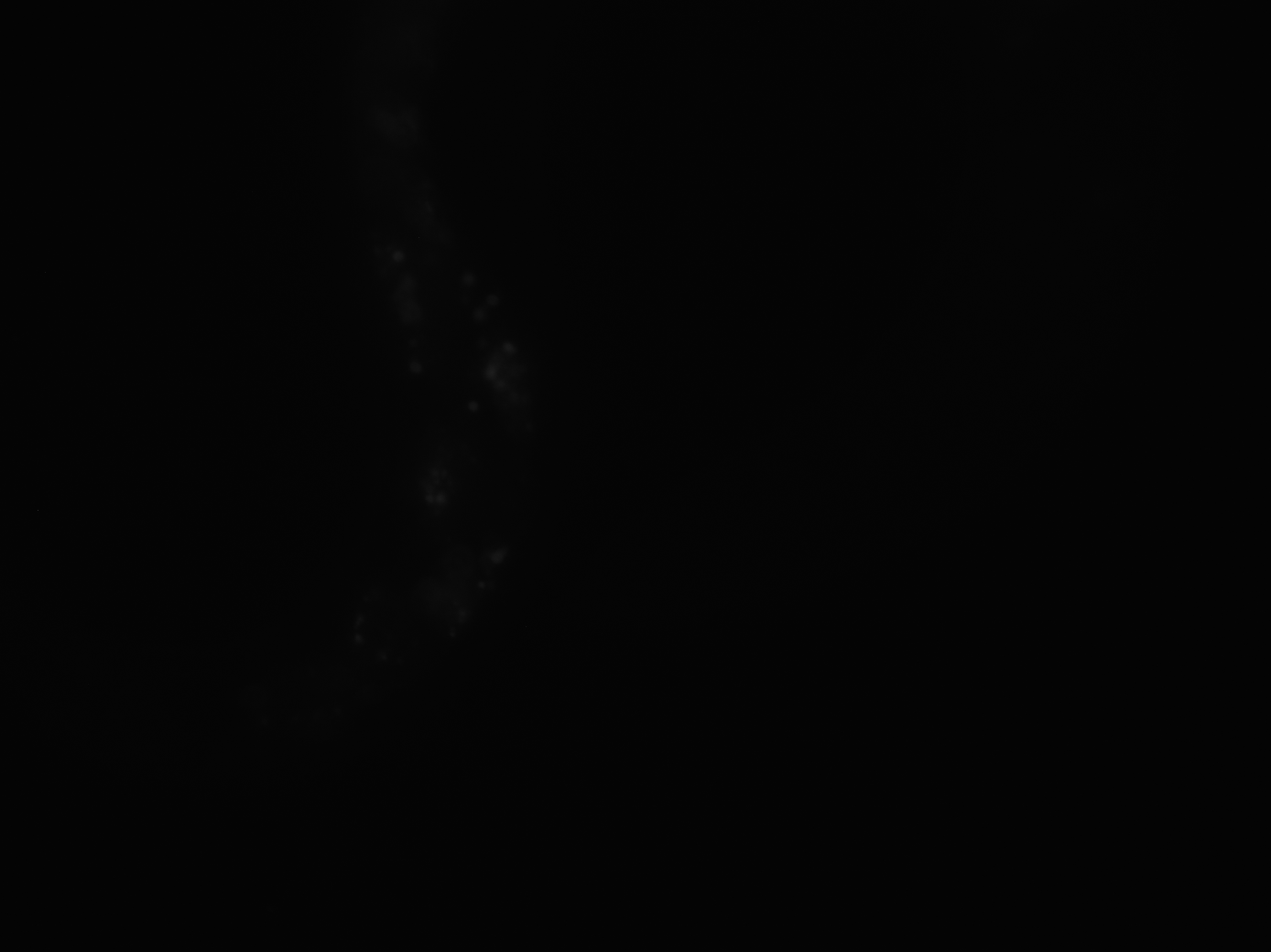

Supplement: Supplementary file 3 — Source data Fig. 2 [file 44319_2025_493_MOESM3_ESM.zip › Figure2/Fig2F/Experiment-01prp40_NR.tif_files/Experiment-01_z8c1x0-1388y0-1040.tif]

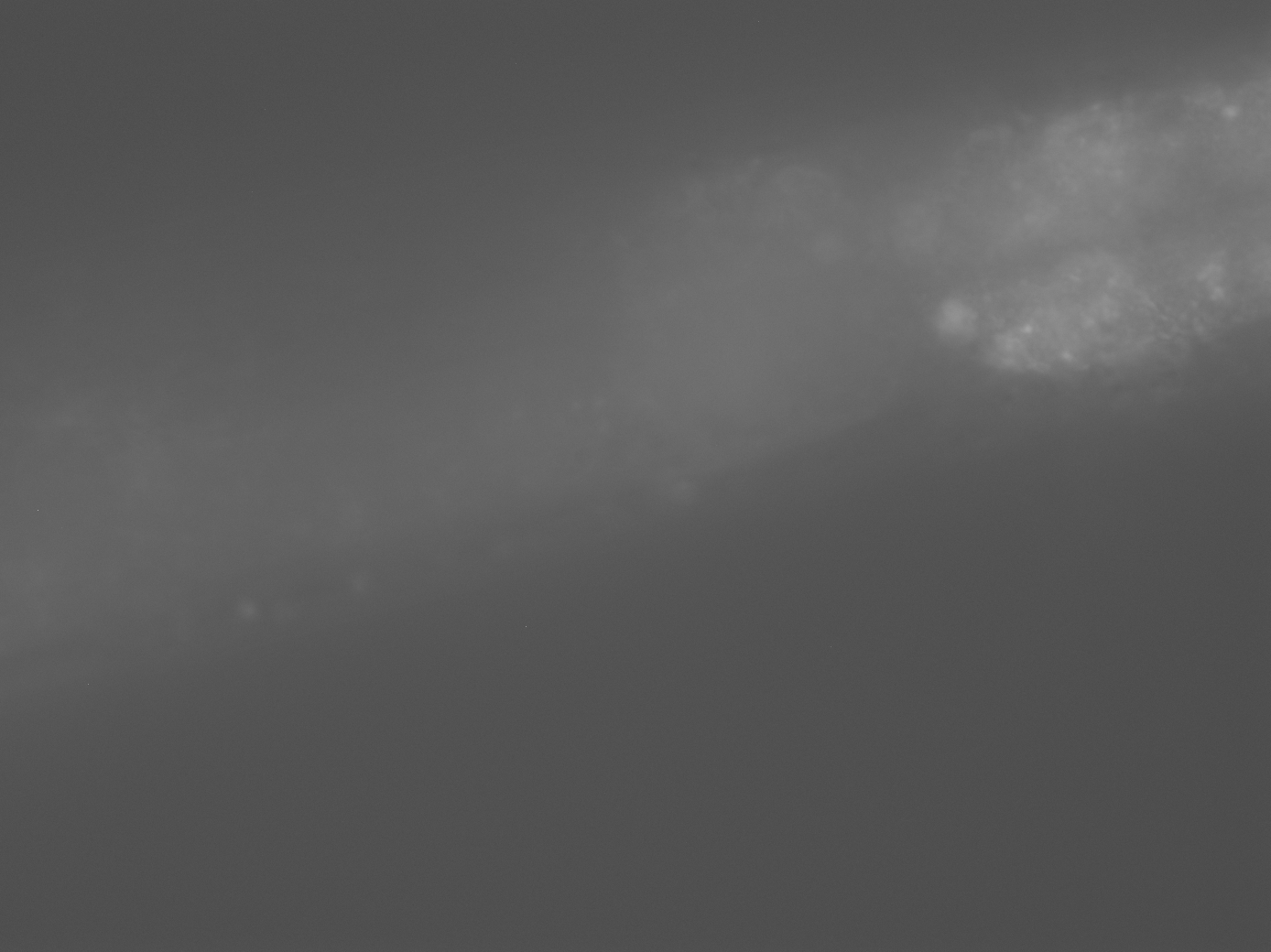

Supplement: Supplementary file 3 — Source data Fig. 2 [file 44319_2025_493_MOESM3_ESM.zip › Figure2/Fig2D/Experiment-77_VC_mbl1.tif_files/Experiment-77_z8c1x0-1388y0-1040.tif]

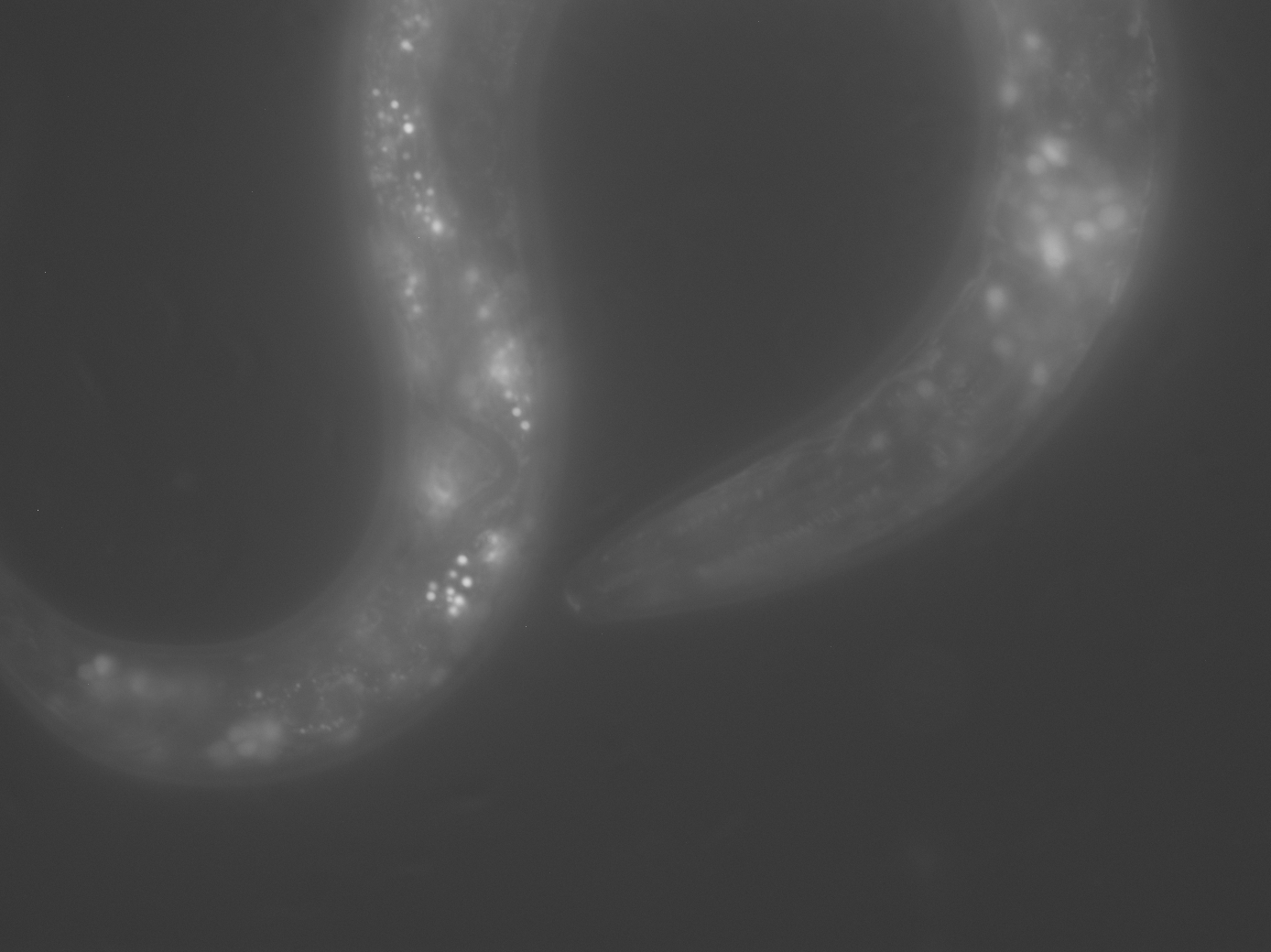

Supplement: Supplementary file 3 — Source data Fig. 2 [file 44319_2025_493_MOESM3_ESM.zip › Figure2/Fig2F/Experiment-01prp40_NR.tif_files/Experiment-01_z5c0x0-1388y0-1040.tif]

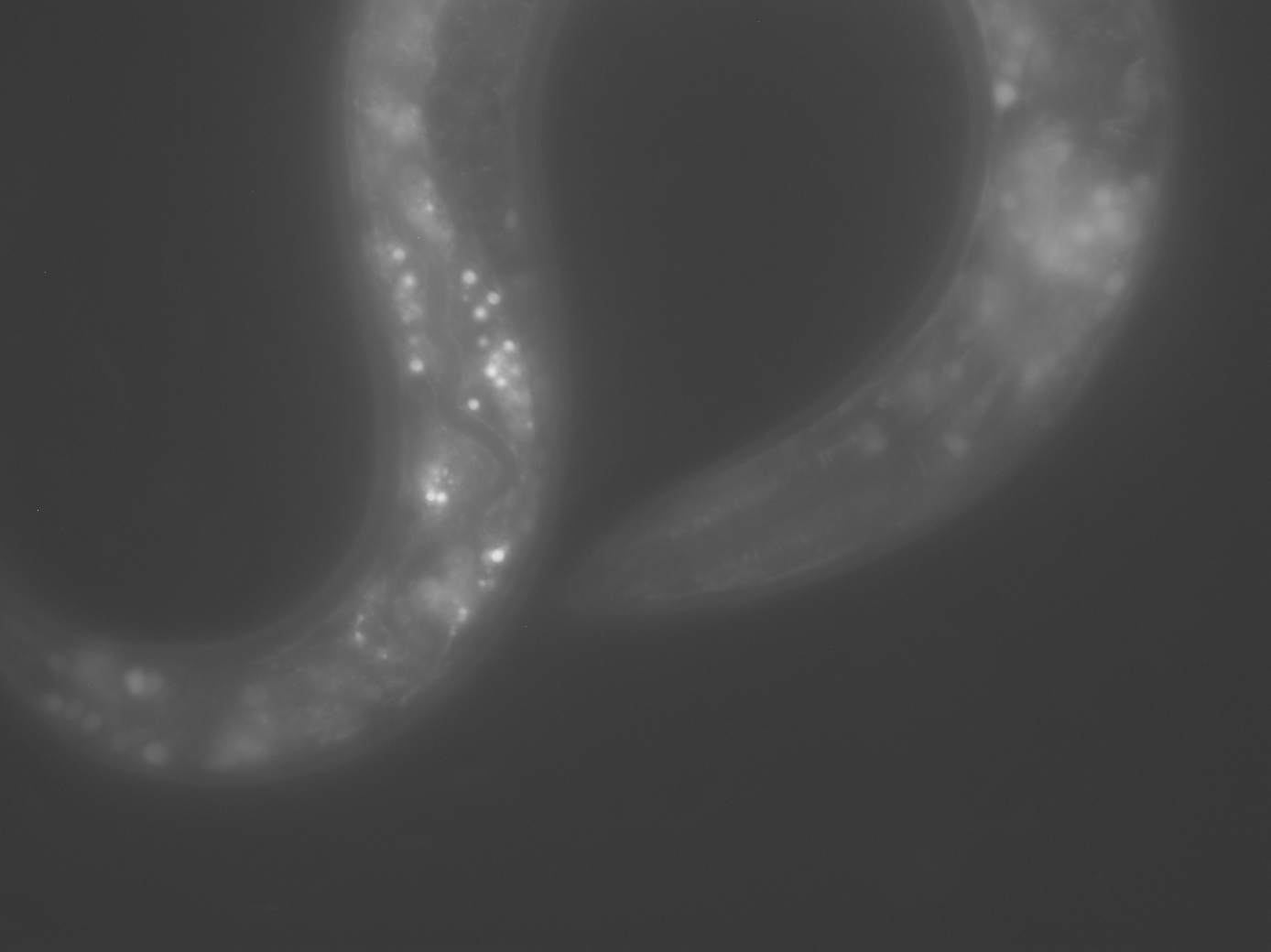

Supplement: Supplementary file 3 — Source data Fig. 2 [file 44319_2025_493_MOESM3_ESM.zip › Figure2/Fig2F/Experiment-01prp40_NR.tif_files/Experiment-01_z8c0x0-1388y0-1040.tif]

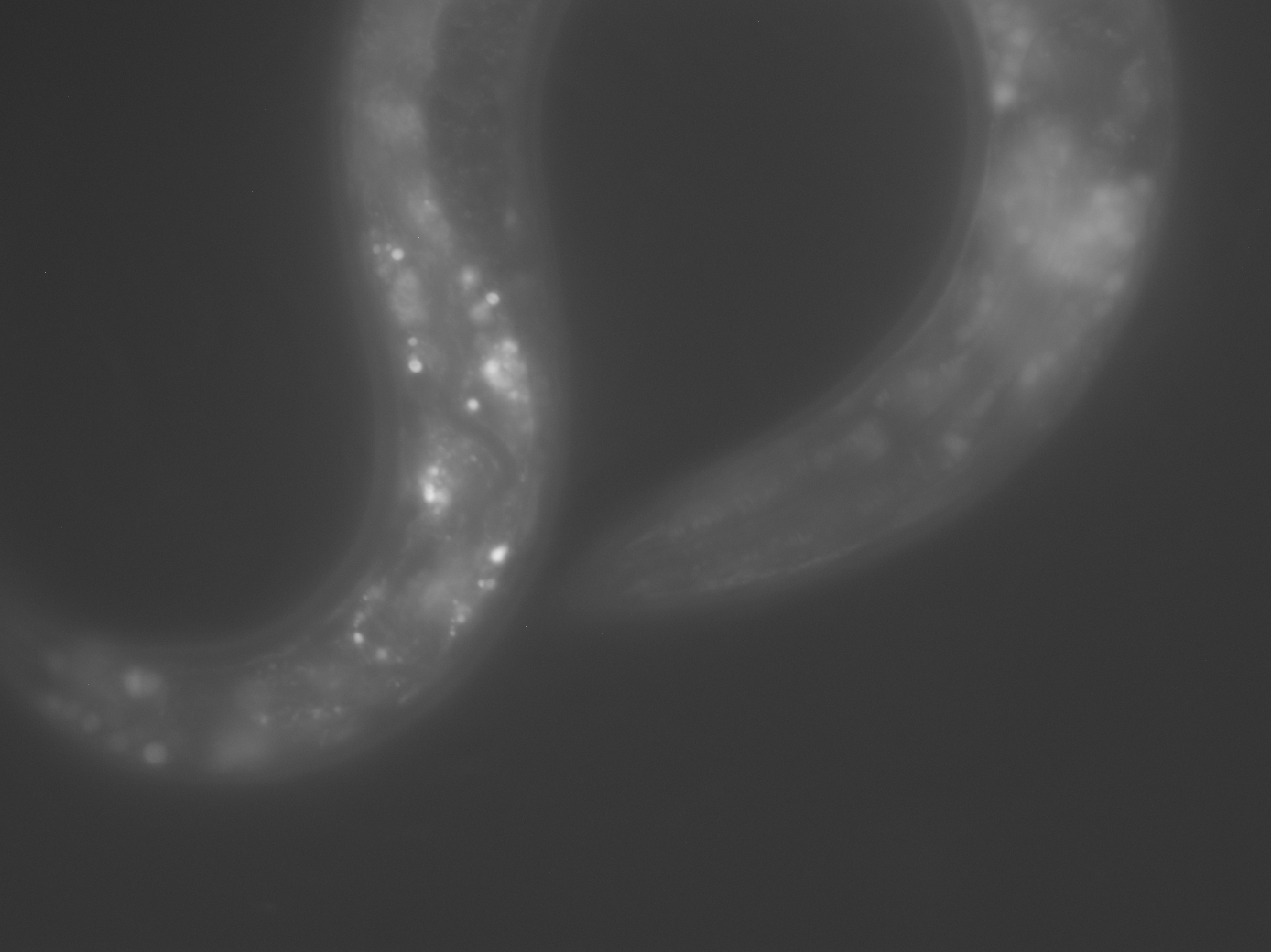

Supplement: Supplementary file 3 — Source data Fig. 2 [file 44319_2025_493_MOESM3_ESM.zip › Figure2/Fig2F/Experiment-01prp40_NR.tif_files/Experiment-01_z9c0x0-1388y0-1040.tif]

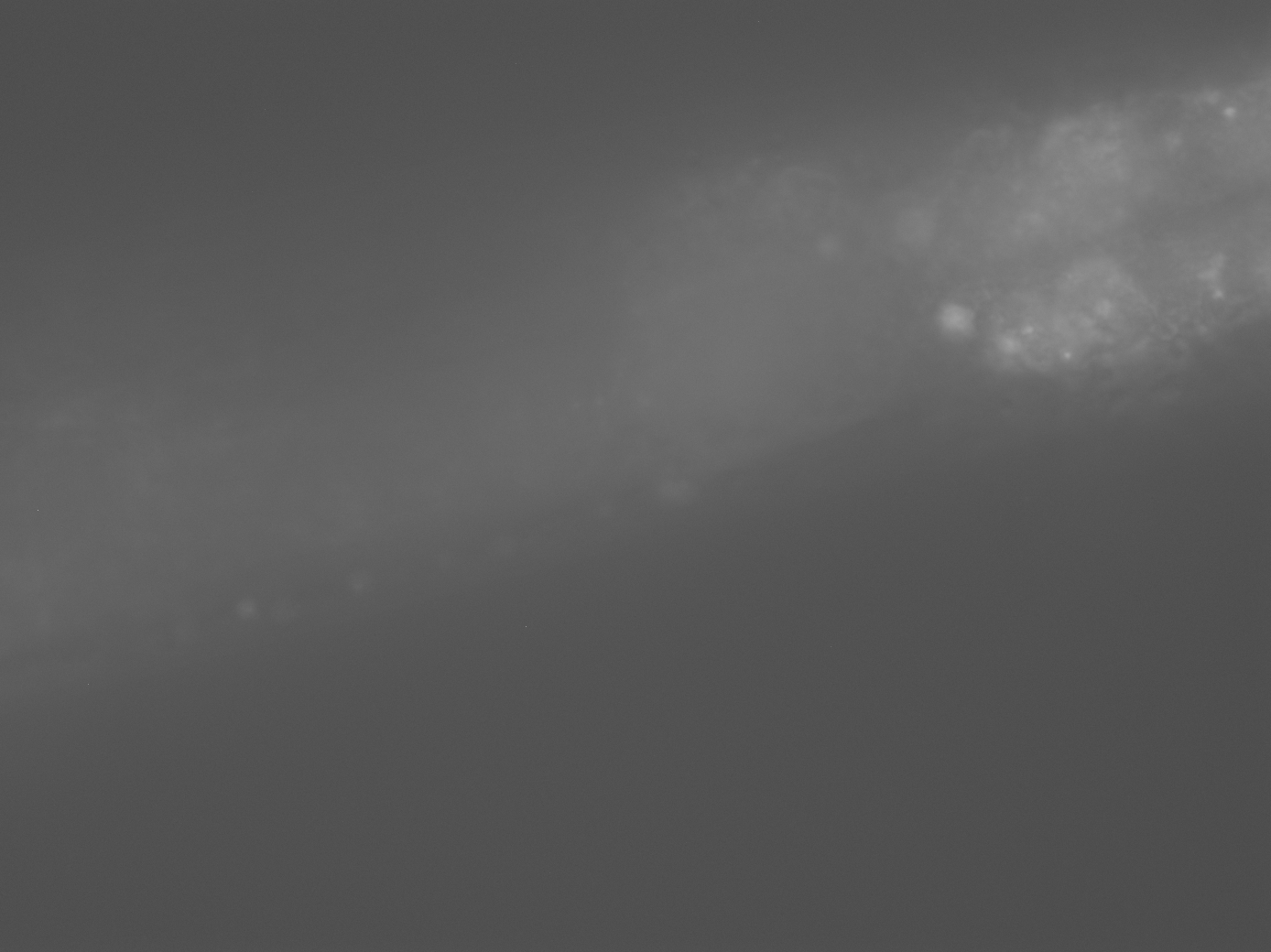

Supplement: Supplementary file 3 — Source data Fig. 2 [file 44319_2025_493_MOESM3_ESM.zip › Figure2/Fig2D/Experiment-77_VC_mbl1.tif_files/Experiment-77_z7c1x0-1388y0-1040.tif]

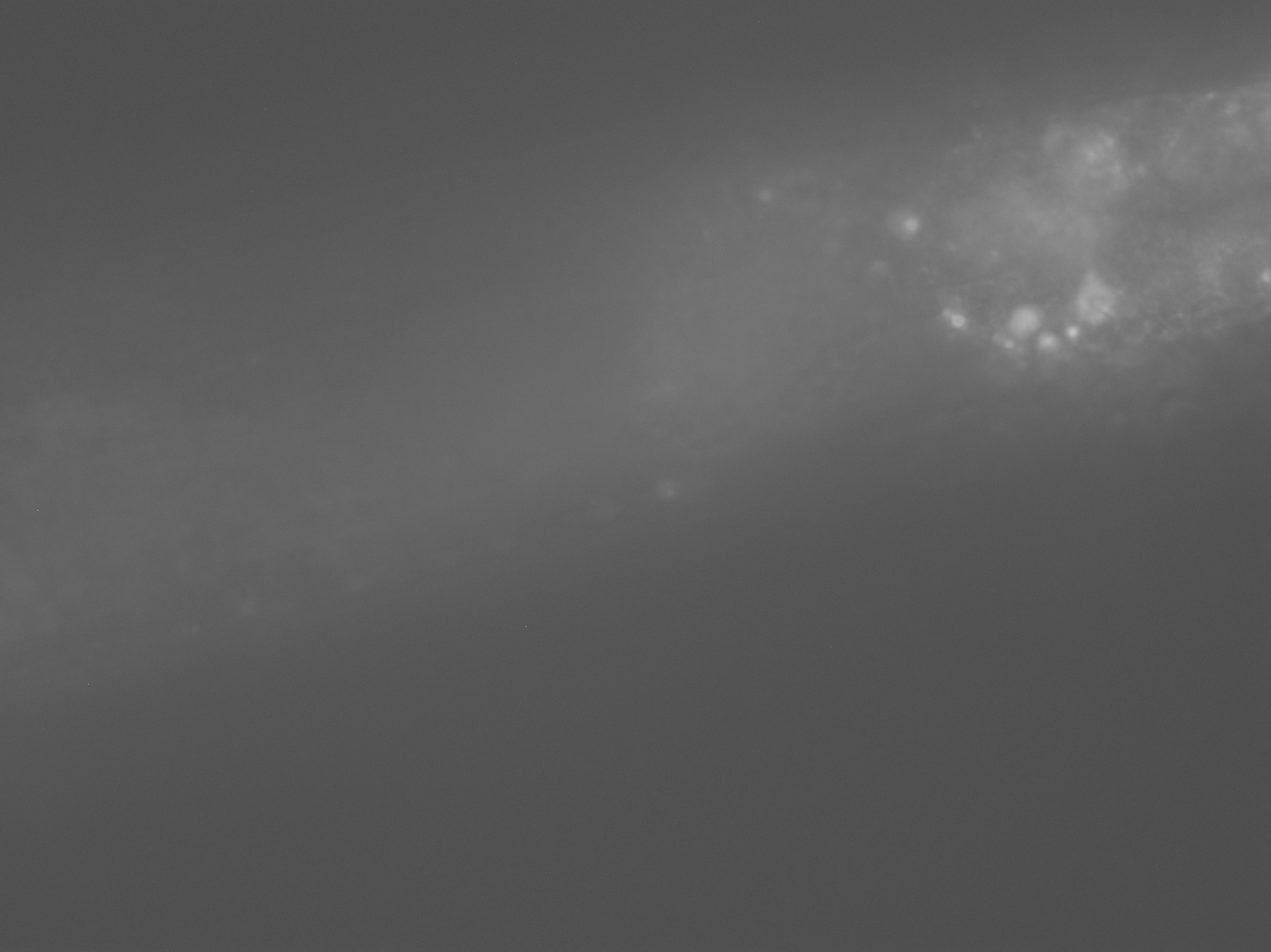

Supplement: Supplementary file 3 — Source data Fig. 2 [file 44319_2025_493_MOESM3_ESM.zip › Figure2/Fig2D/Experiment-77_VC_mbl1.tif_files/Experiment-77_z3c1x0-1388y0-1040.tif]

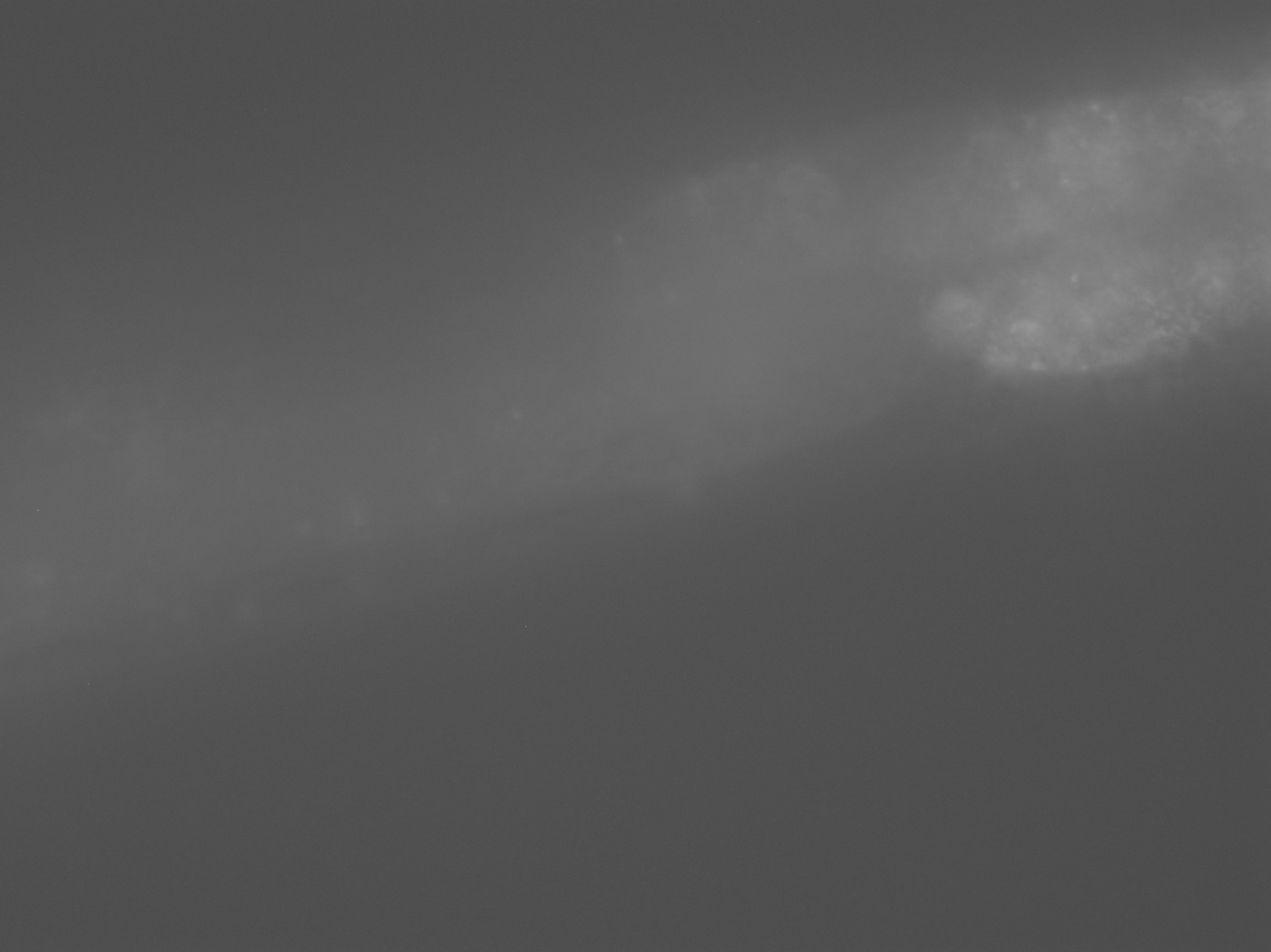

Supplement: Supplementary file 3 — Source data Fig. 2 [file 44319_2025_493_MOESM3_ESM.zip › Figure2/Fig2D/Experiment-77_VC_mbl1.tif_files/Experiment-77_z10c1x0-1388y0-1040.tif]

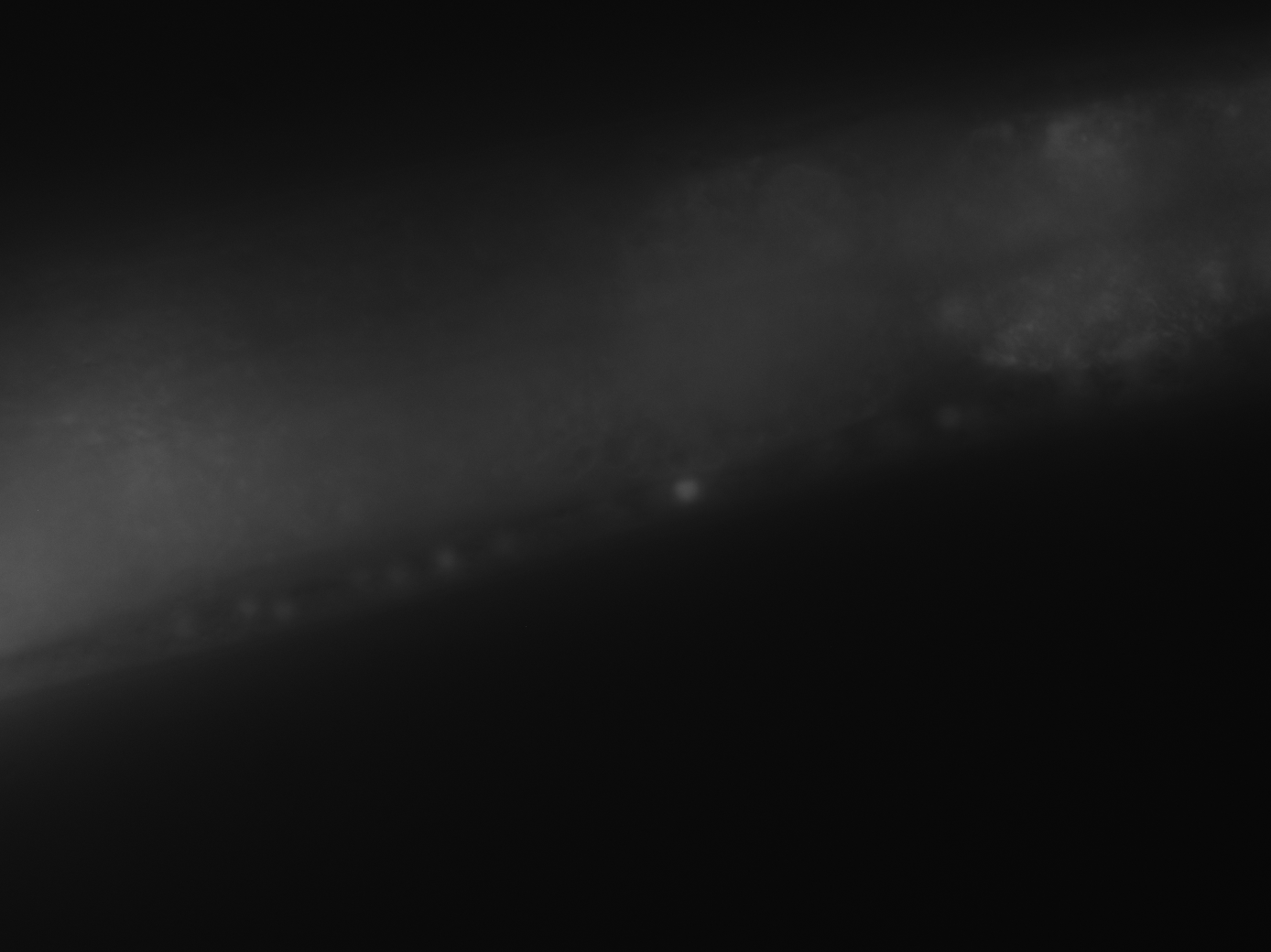

Supplement: Supplementary file 3 — Source data Fig. 2 [file 44319_2025_493_MOESM3_ESM.zip › Figure2/Fig2D/Experiment-77_VC_mbl1.tif_files/Experiment-77_z9c0x0-1388y0-1040.tif]

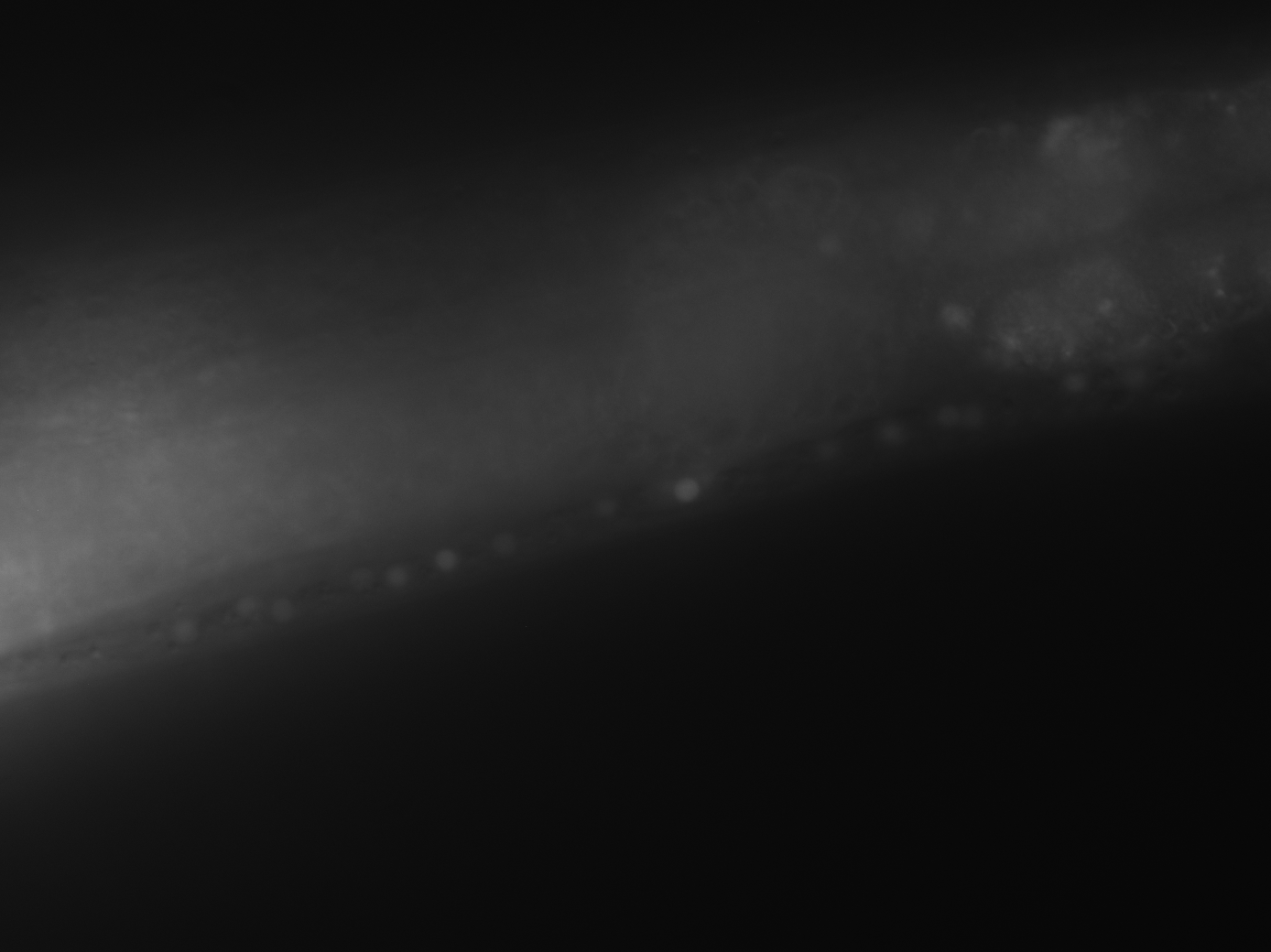

Supplement: Supplementary file 3 — Source data Fig. 2 [file 44319_2025_493_MOESM3_ESM.zip › Figure2/Fig2D/Experiment-77_VC_mbl1.tif_files/Experiment-77_z7c0x0-1388y0-1040.tif]

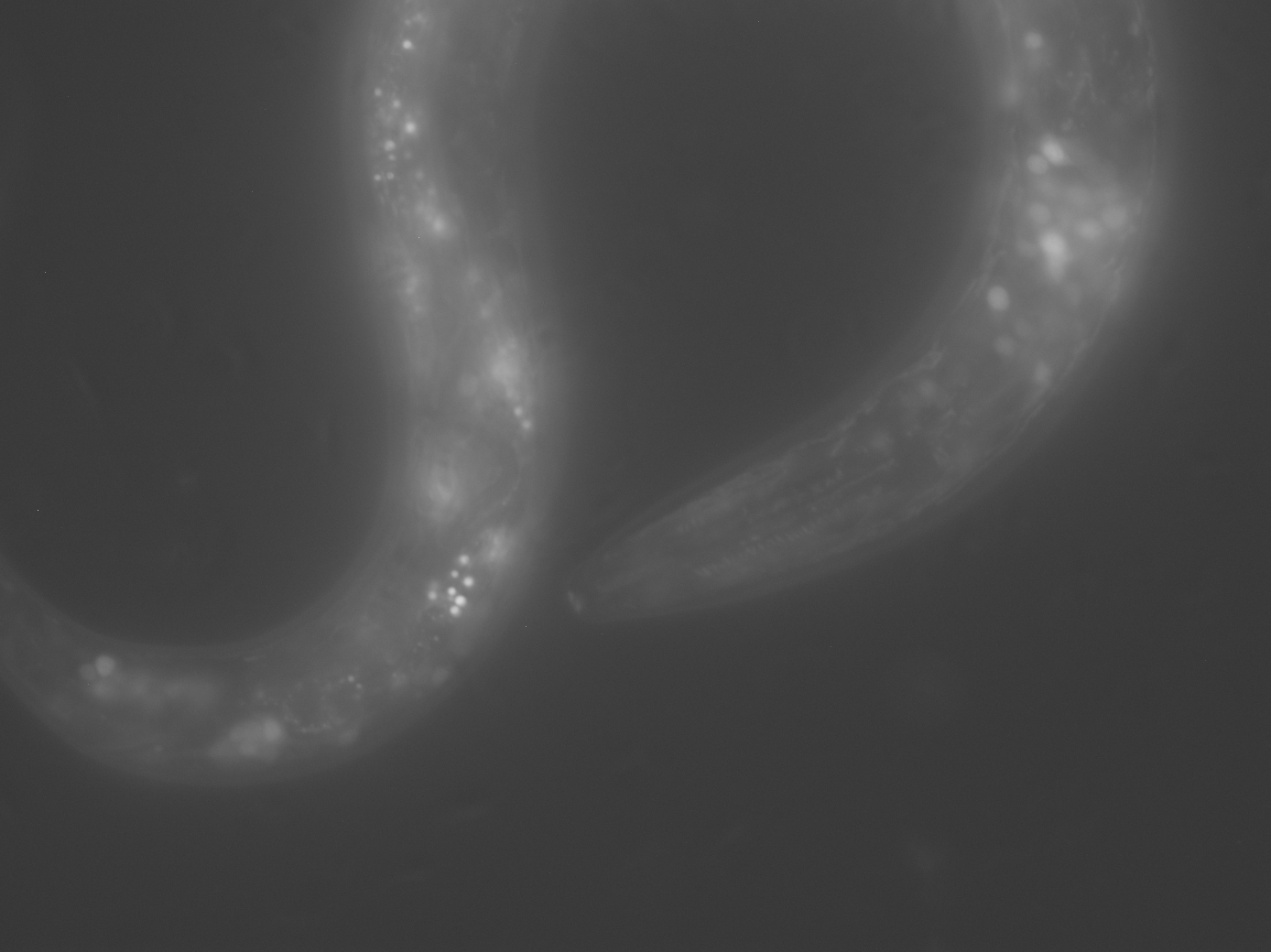

Supplement: Supplementary file 3 — Source data Fig. 2 [file 44319_2025_493_MOESM3_ESM.zip › Figure2/Fig2F/Experiment-01prp40_NR.tif_files/Experiment-01_z4c0x0-1388y0-1040.tif]

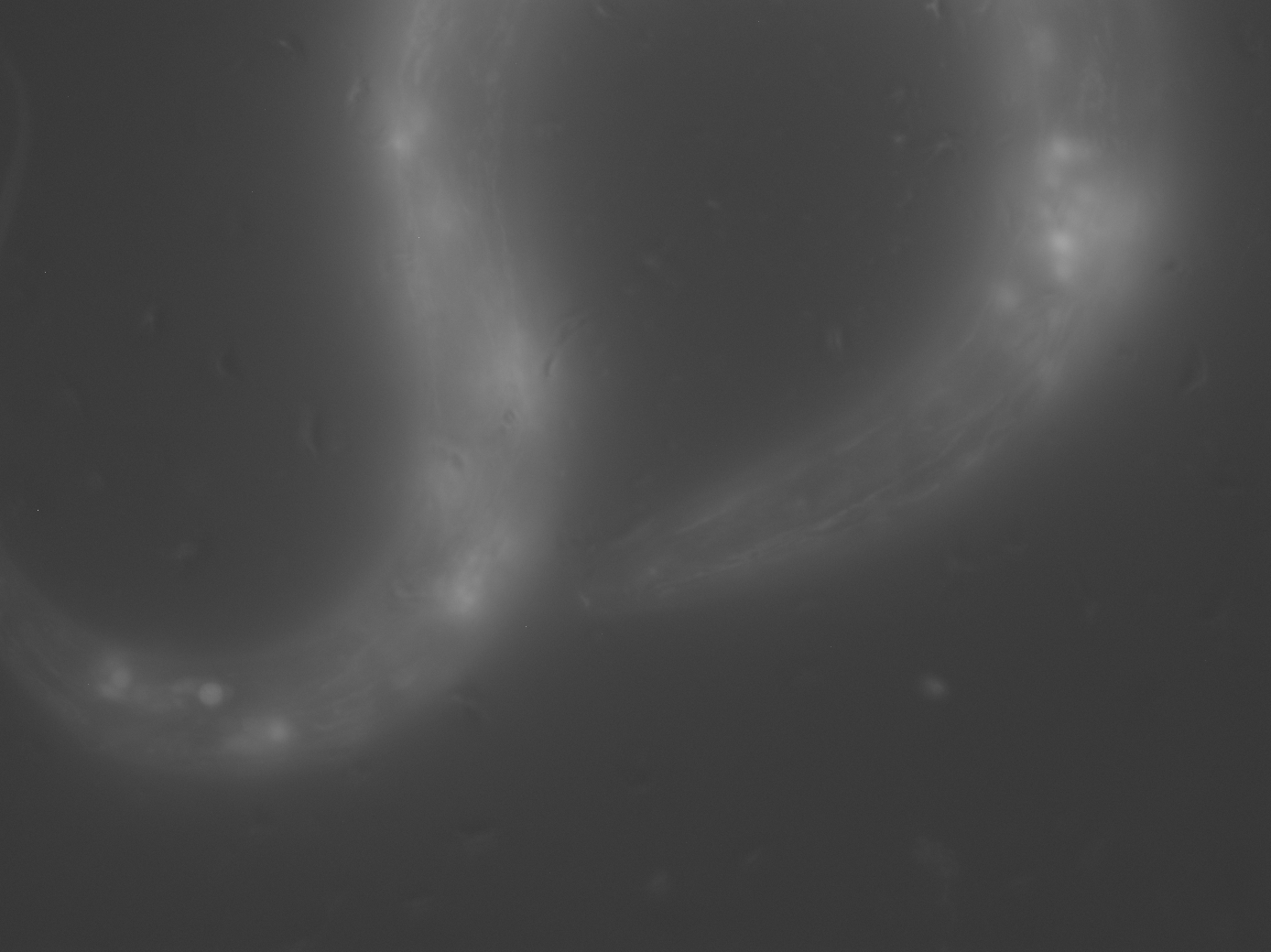

Supplement: Supplementary file 3 — Source data Fig. 2 [file 44319_2025_493_MOESM3_ESM.zip › Figure2/Fig2F/Experiment-01prp40_NR.tif_files/Experiment-01_z0c0x0-1388y0-1040.tif]

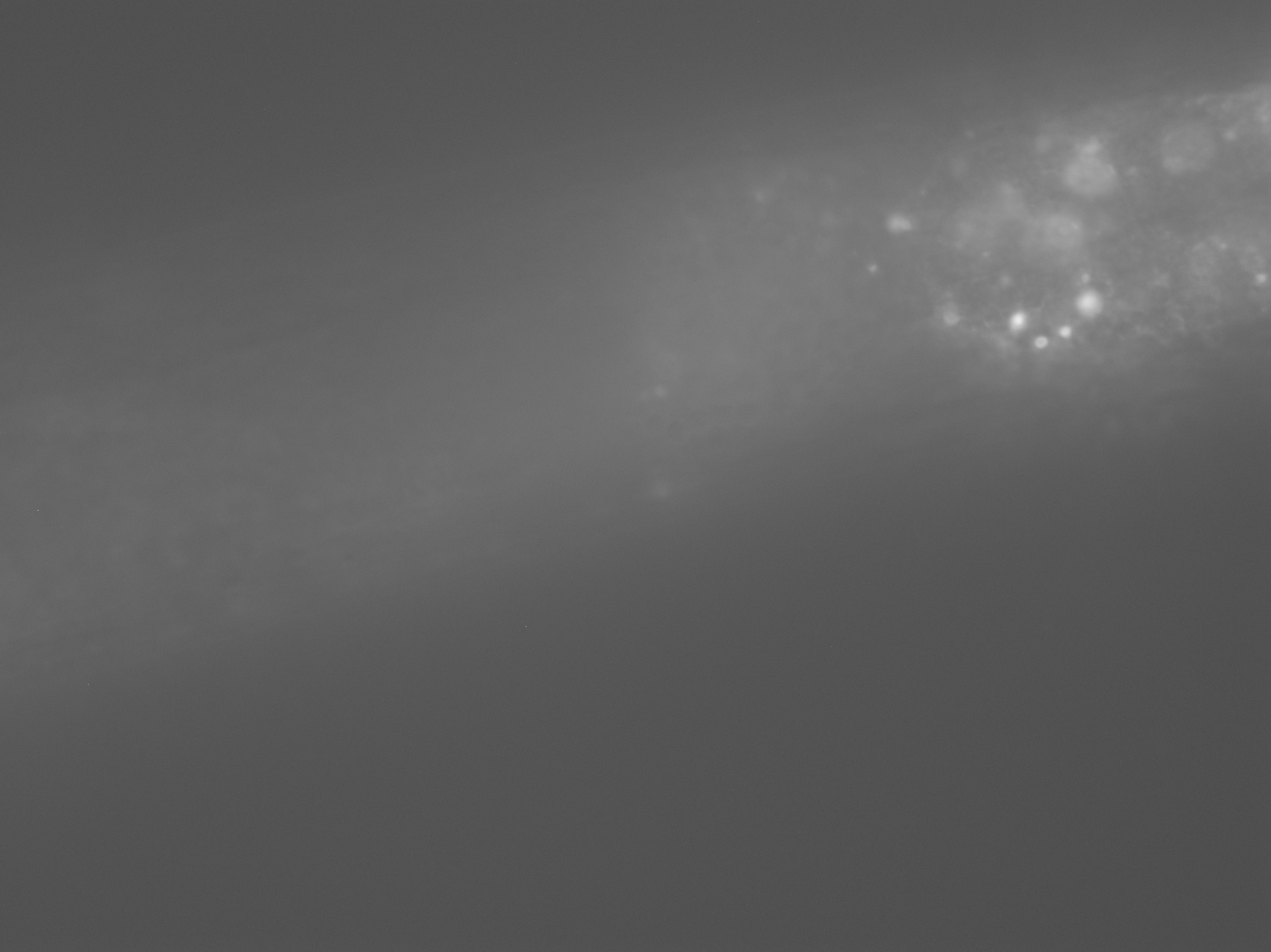

Supplement: Supplementary file 3 — Source data Fig. 2 [file 44319_2025_493_MOESM3_ESM.zip › Figure2/Fig2D/Experiment-77_VC_mbl1.tif_files/Experiment-77_z0c1x0-1388y0-1040.tif]

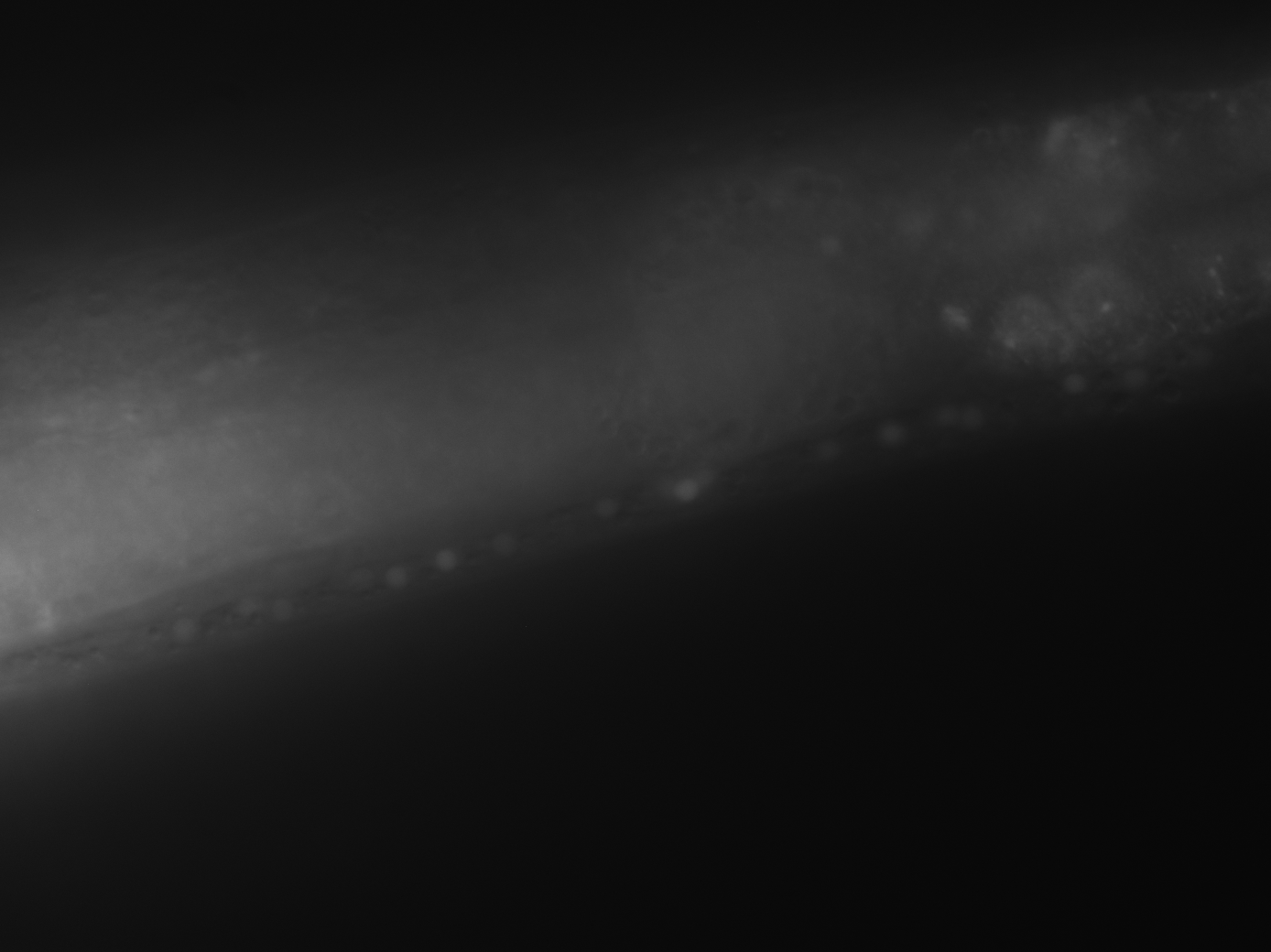

Supplement: Supplementary file 3 — Source data Fig. 2 [file 44319_2025_493_MOESM3_ESM.zip › Figure2/Fig2D/Experiment-77_VC_mbl1.tif_files/Experiment-77_z6c0x0-1388y0-1040.tif]

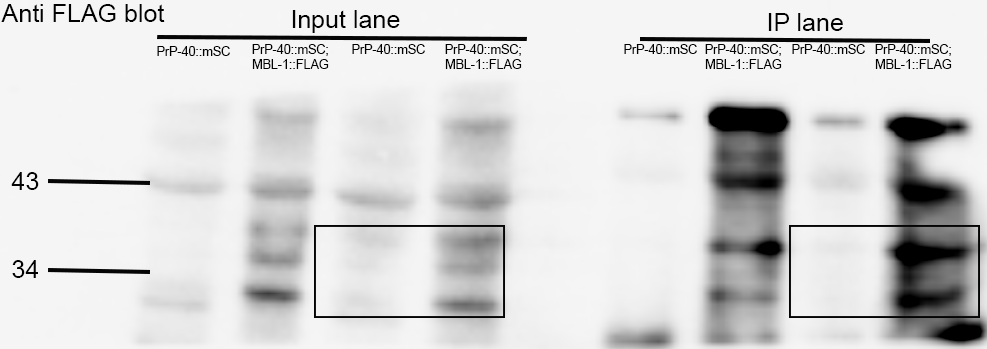

Supplement: Supplementary file 4 — Source data Fig. 3 [file 44319_2025_493_MOESM4_ESM.zip › Figure3/Fig3F/Anti MBL1FLAG_lanedmarked.tif]

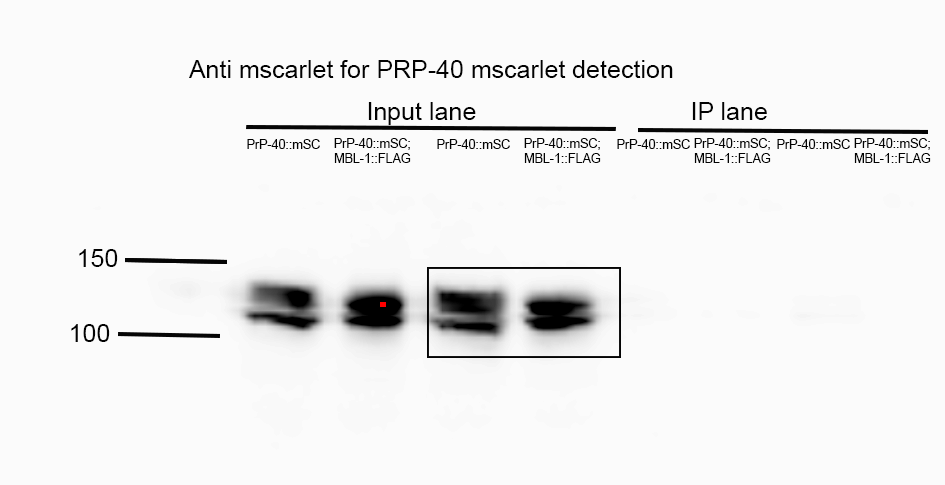

Supplement: Supplementary file 4 — Source data Fig. 3 [file 44319_2025_493_MOESM4_ESM.zip › Figure3/Fig3F/AntiPRP40blot_lowexposure_lanesmarked.tif]

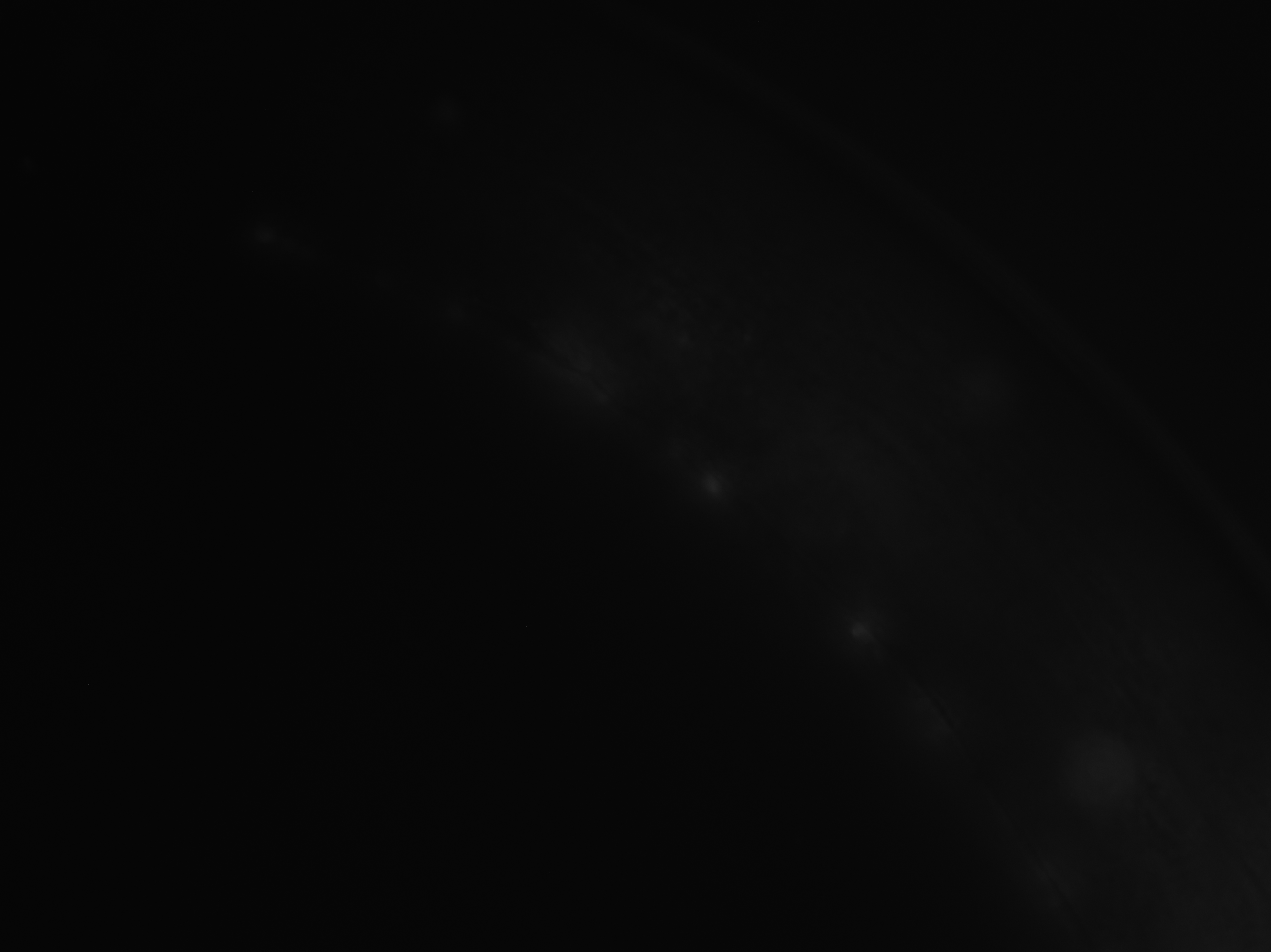

Supplement: Supplementary file 4 — Source data Fig. 3 [file 44319_2025_493_MOESM4_ESM.zip › Figure3/Fig3A/Experiment-14_VC_upstreamdeletion.tif_files/Experiment-14_z9c1x0-1388y0-1040.tif]

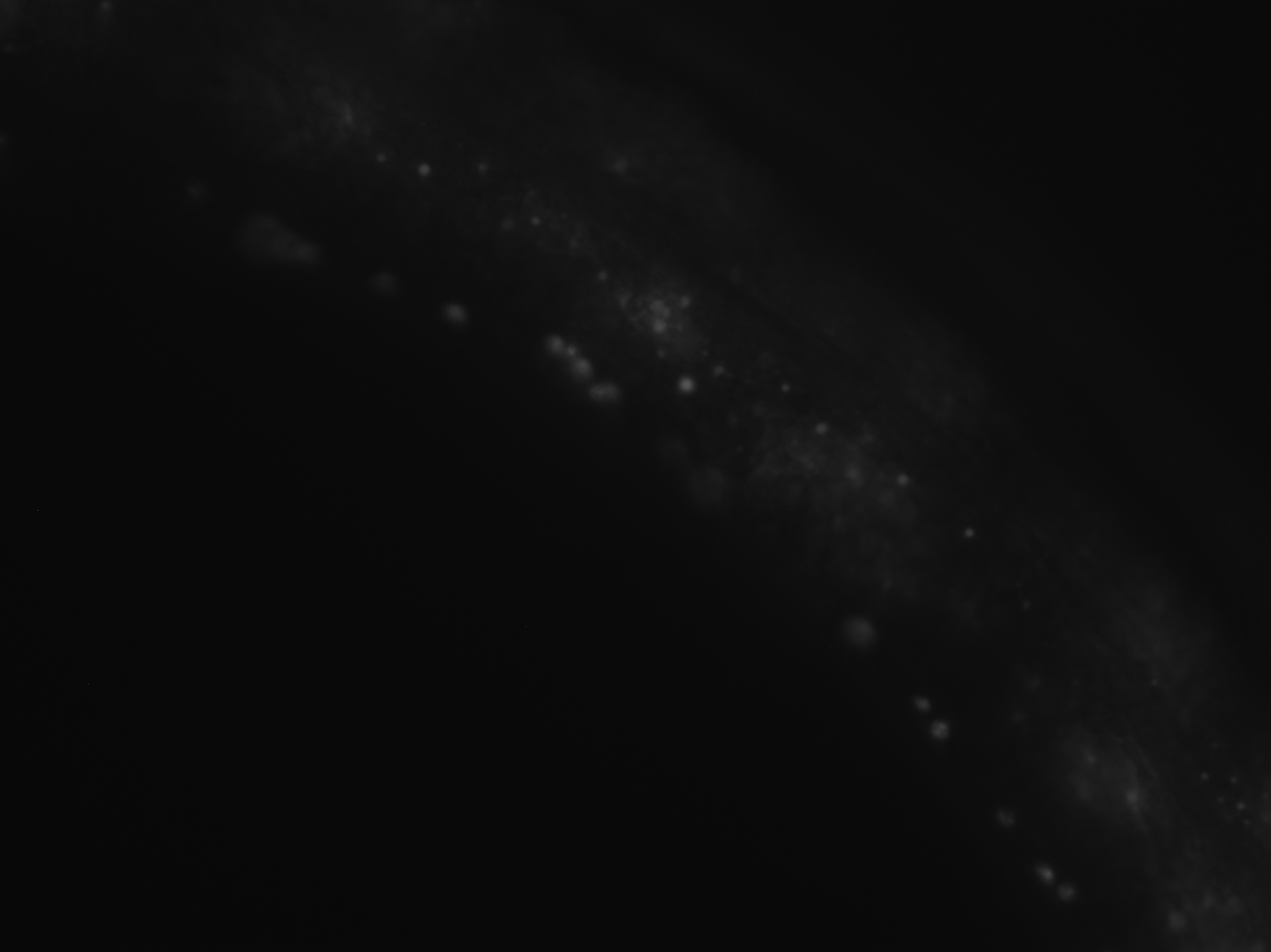

Supplement: Supplementary file 4 — Source data Fig. 3 [file 44319_2025_493_MOESM4_ESM.zip › Figure3/Fig3A/Experiment-14_VC_upstreamdeletion.tif_files/Experiment-14_z17c1x0-1388y0-1040.tif]

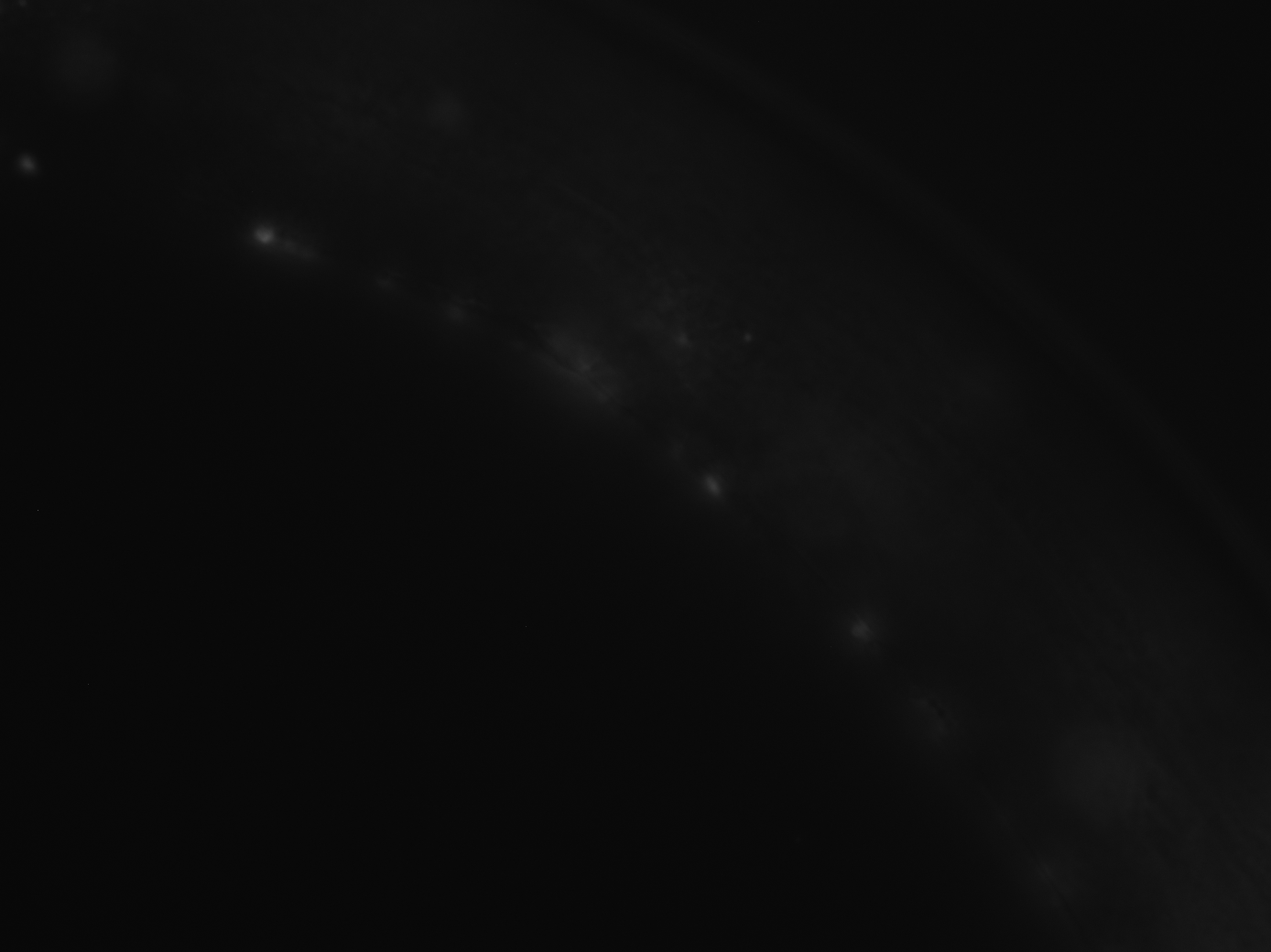

Supplement: Supplementary file 4 — Source data Fig. 3 [file 44319_2025_493_MOESM4_ESM.zip › Figure3/Fig3A/Experiment-14_VC_upstreamdeletion.tif_files/Experiment-14_z10c1x0-1388y0-1040.tif]

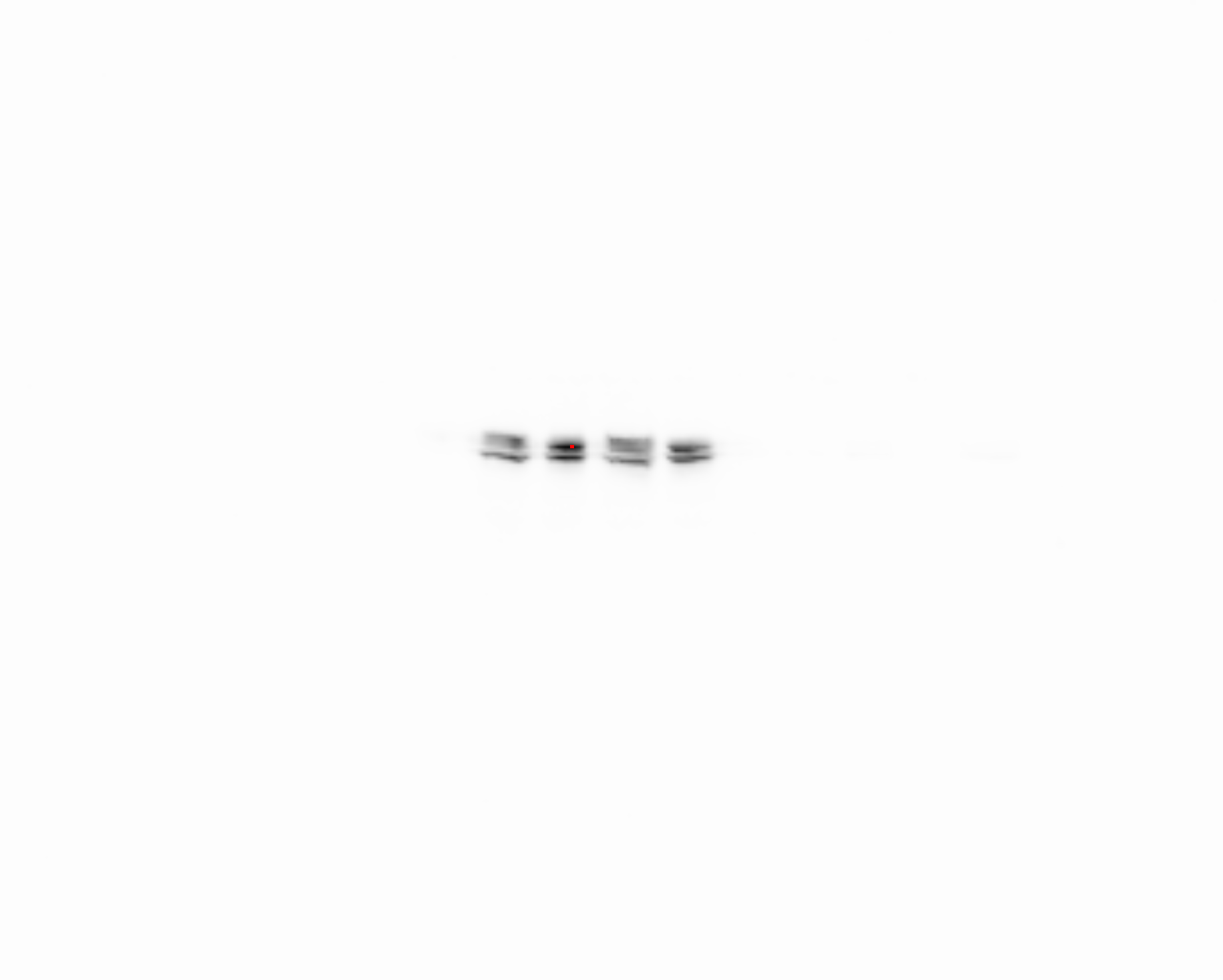

Supplement: Supplementary file 4 — Source data Fig. 3 [file 44319_2025_493_MOESM4_ESM.zip › Figure3/Fig3F/AntiPRP40Blot1_lowexposure.tif]

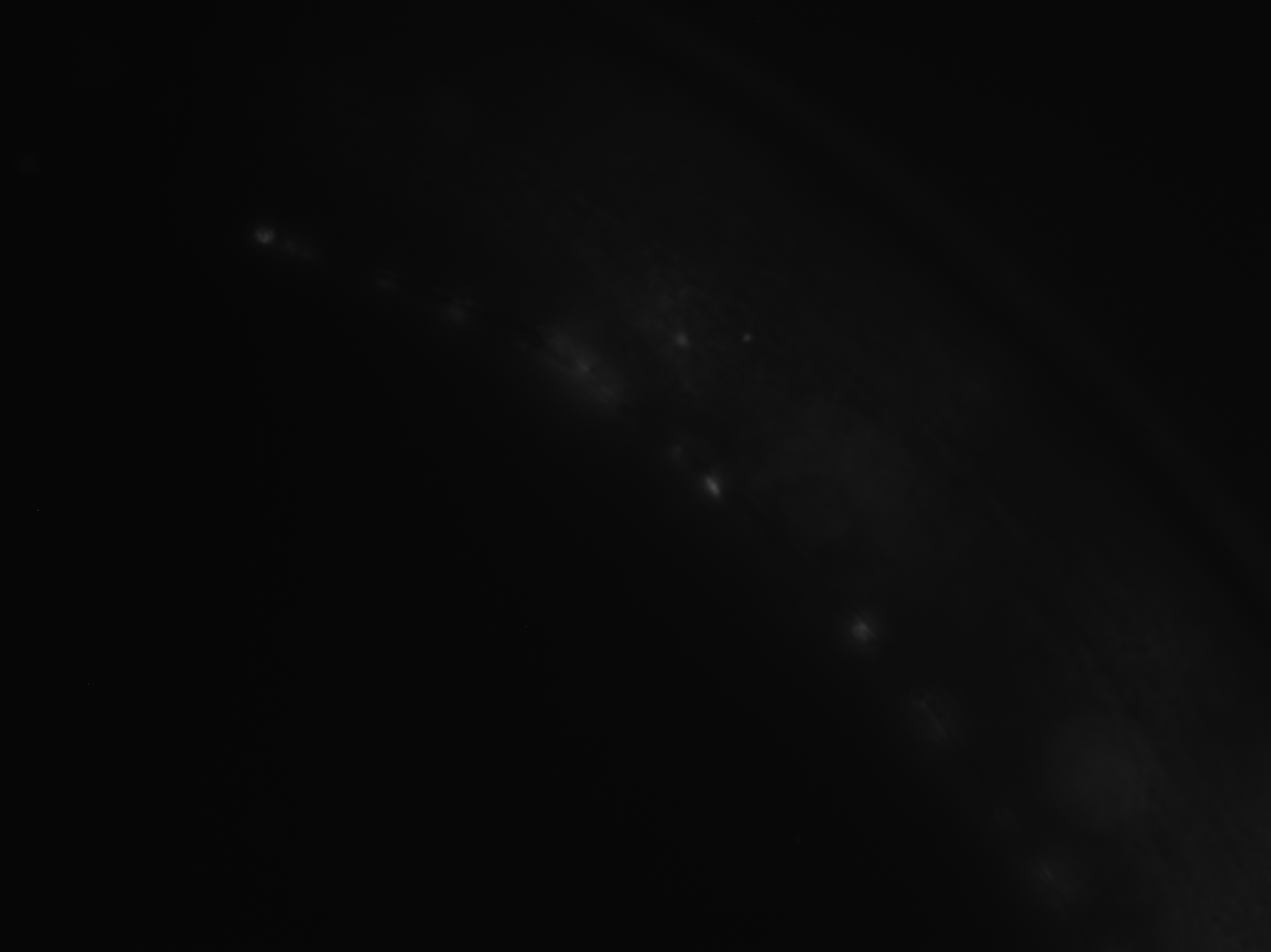

Supplement: Supplementary file 4 — Source data Fig. 3 [file 44319_2025_493_MOESM4_ESM.zip › Figure3/Fig3A/Experiment-14_VC_upstreamdeletion.tif_files/Experiment-14_z11c1x0-1388y0-1040.tif]

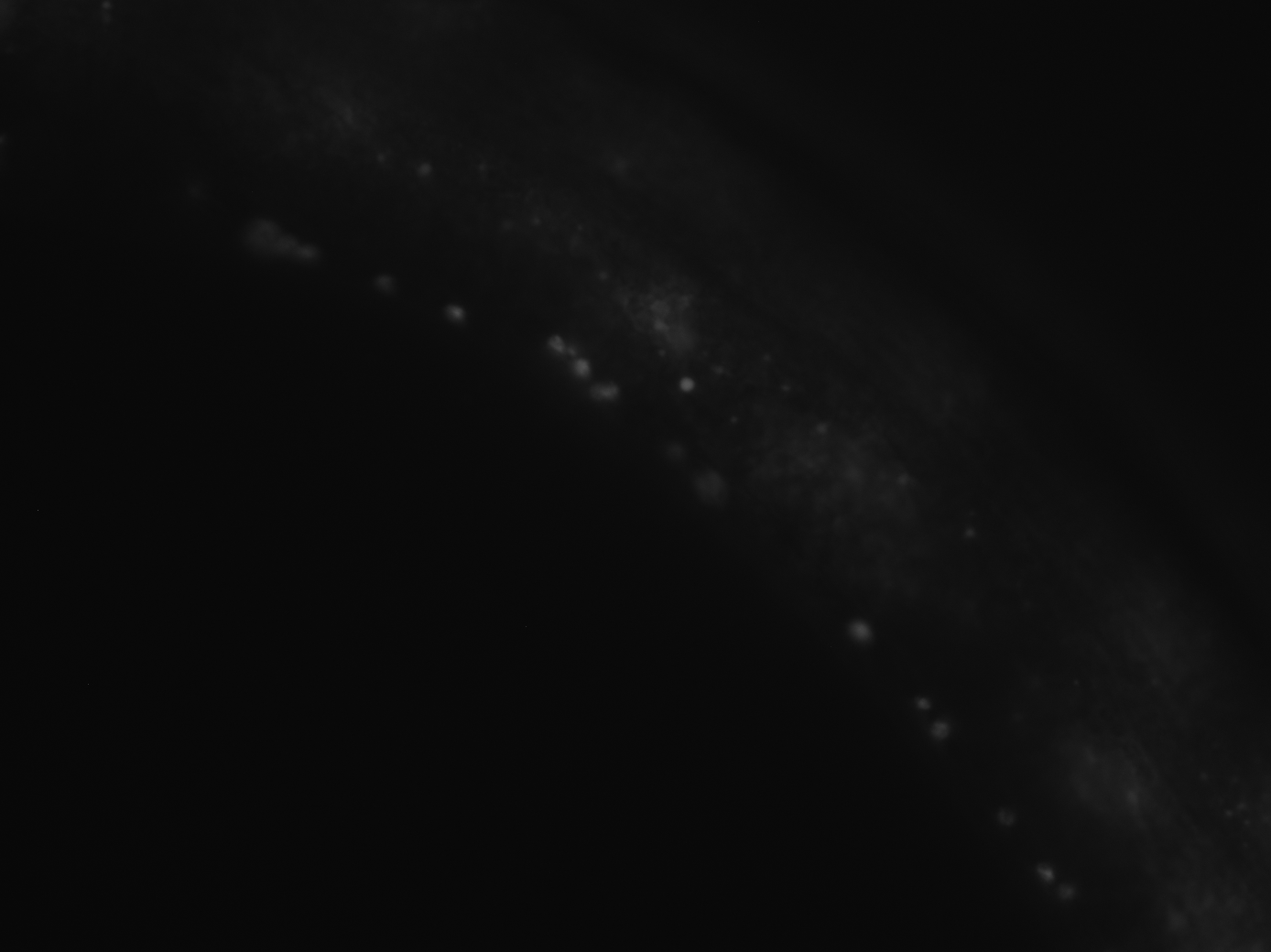

Supplement: Supplementary file 4 — Source data Fig. 3 [file 44319_2025_493_MOESM4_ESM.zip › Figure3/Fig3A/Experiment-14_VC_upstreamdeletion.tif_files/Experiment-14_z16c1x0-1388y0-1040.tif]

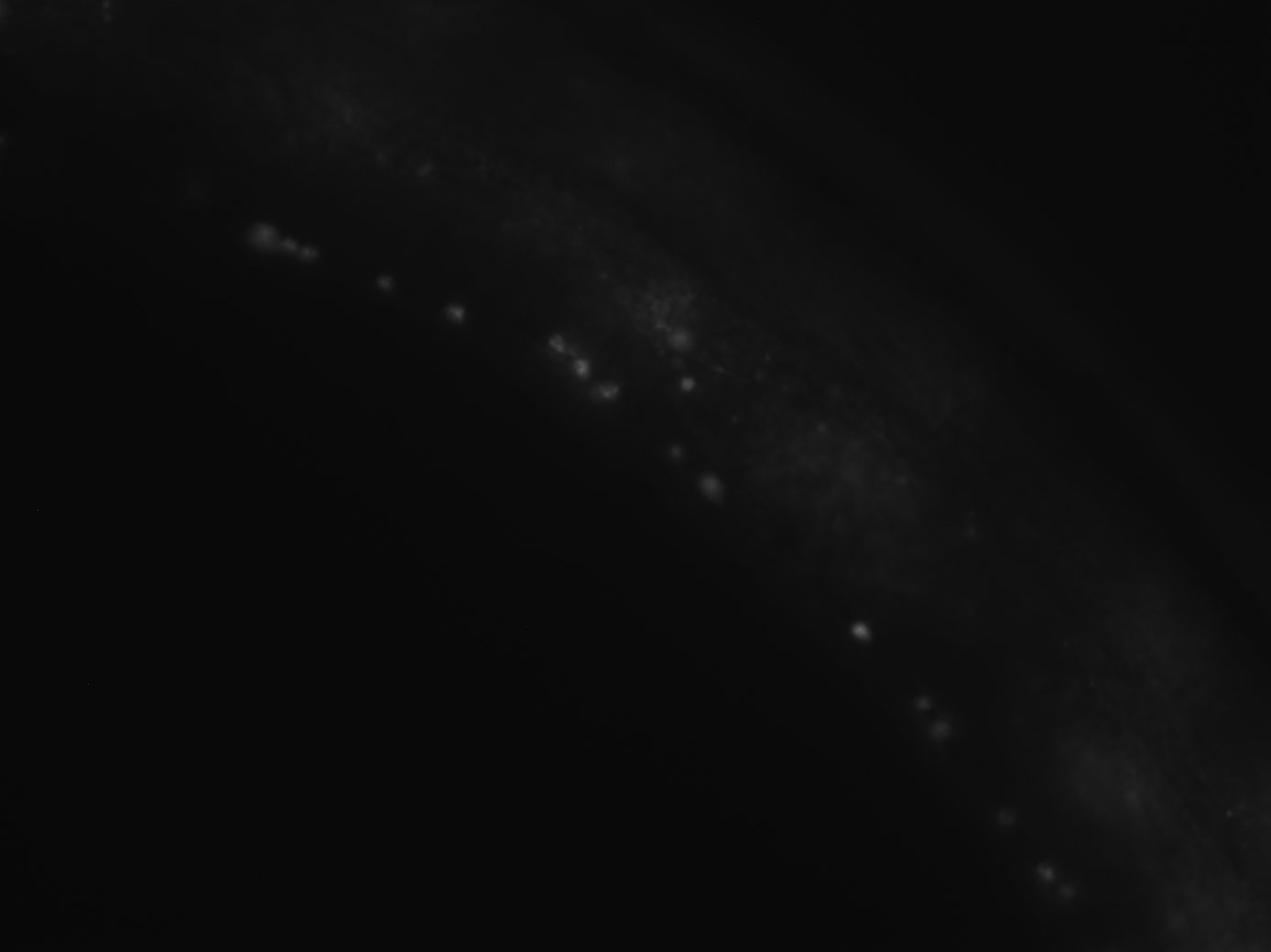

Supplement: Supplementary file 4 — Source data Fig. 3 [file 44319_2025_493_MOESM4_ESM.zip › Figure3/Fig3A/Experiment-14_VC_upstreamdeletion.tif_files/Experiment-14_z15c1x0-1388y0-1040.tif]

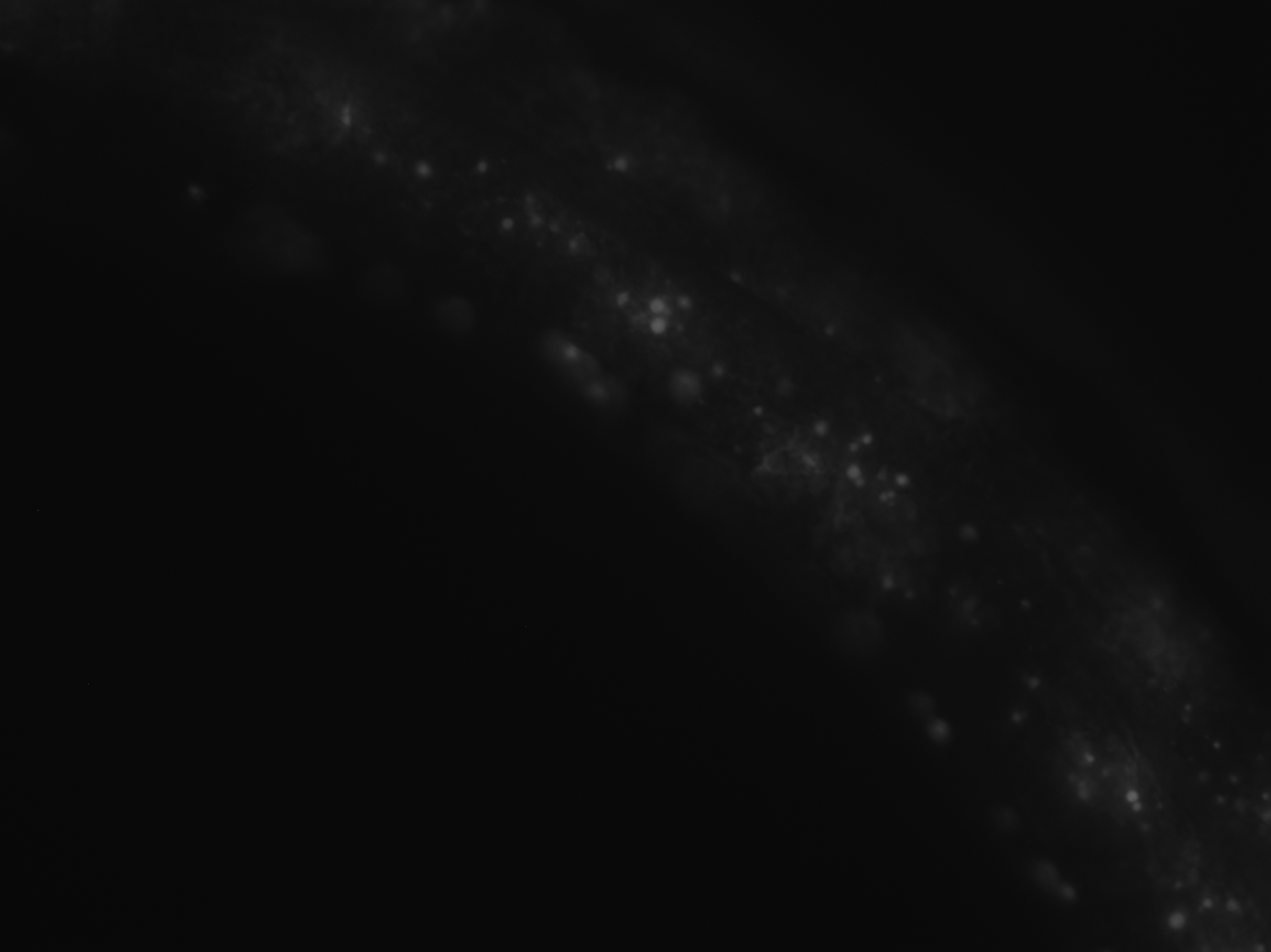

Supplement: Supplementary file 4 — Source data Fig. 3 [file 44319_2025_493_MOESM4_ESM.zip › Figure3/Fig3A/Experiment-14_VC_upstreamdeletion.tif_files/Experiment-14_z19c1x0-1388y0-1040.tif]

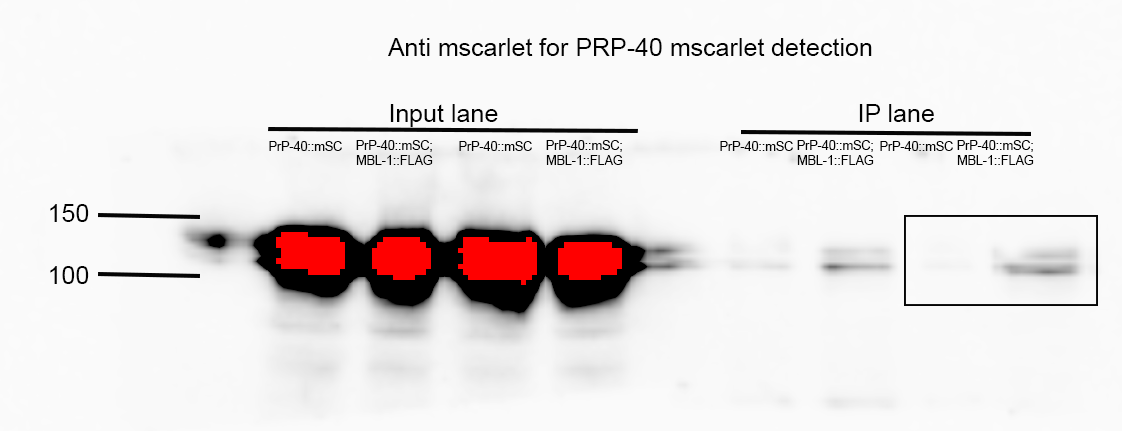

Supplement: Supplementary file 4 — Source data Fig. 3 [file 44319_2025_493_MOESM4_ESM.zip › Figure3/Fig3F/AntiPRP40blot_highexposure_lanesmarked.tif]

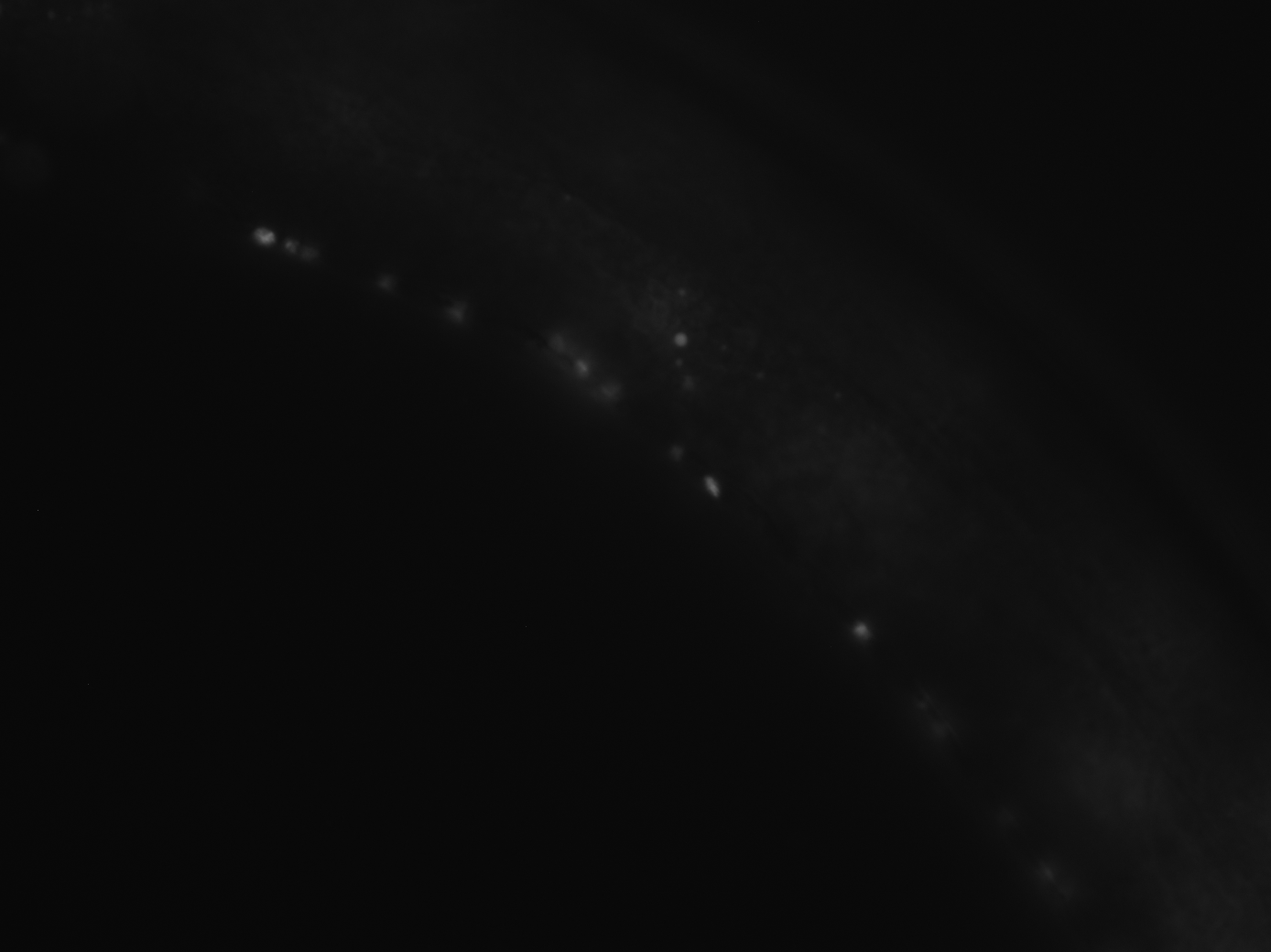

Supplement: Supplementary file 4 — Source data Fig. 3 [file 44319_2025_493_MOESM4_ESM.zip › Figure3/Fig3A/Experiment-14_VC_upstreamdeletion.tif_files/Experiment-14_z13c1x0-1388y0-1040.tif]

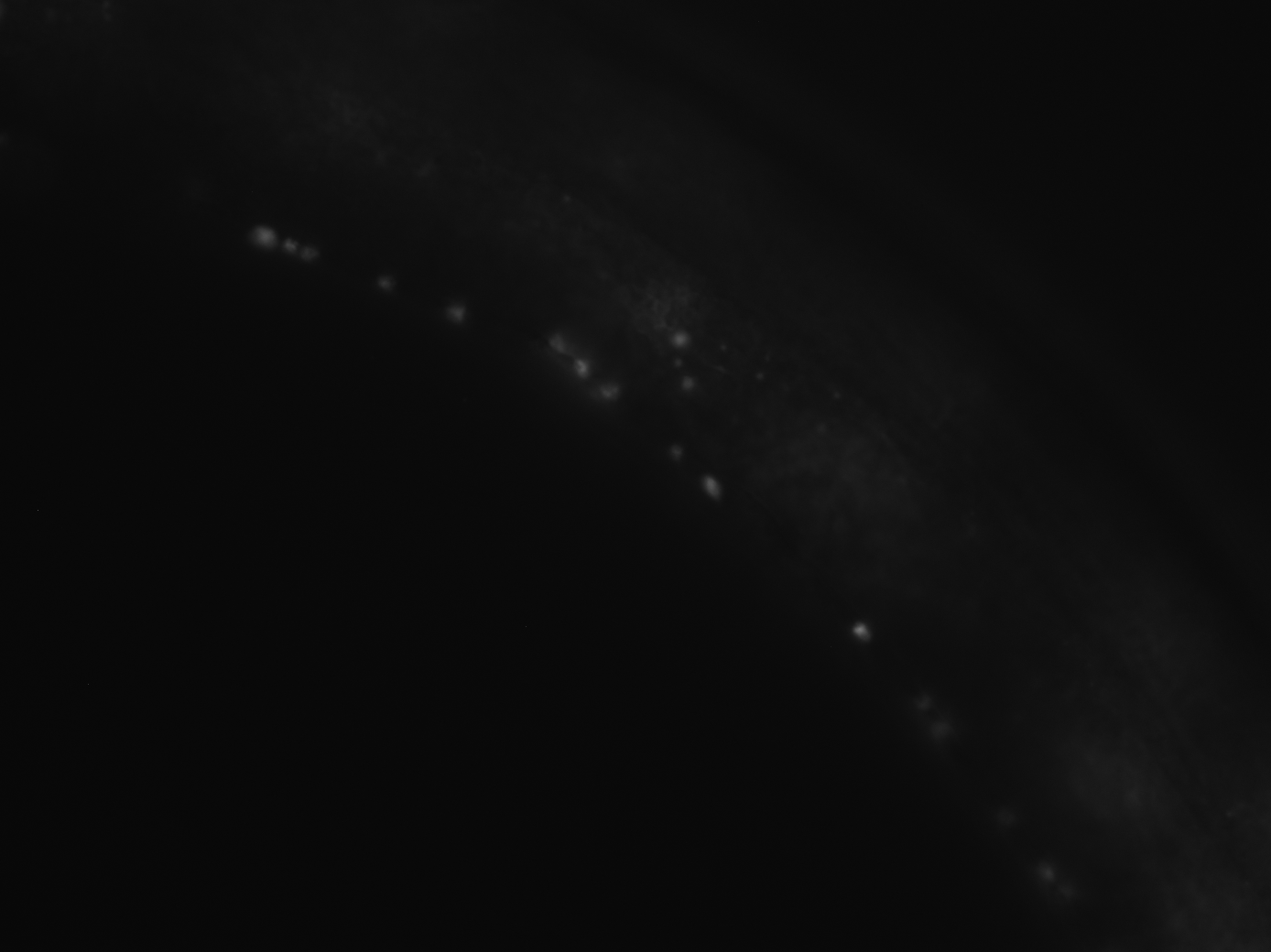

Supplement: Supplementary file 4 — Source data Fig. 3 [file 44319_2025_493_MOESM4_ESM.zip › Figure3/Fig3A/Experiment-14_VC_upstreamdeletion.tif_files/Experiment-14_z14c1x0-1388y0-1040.tif]

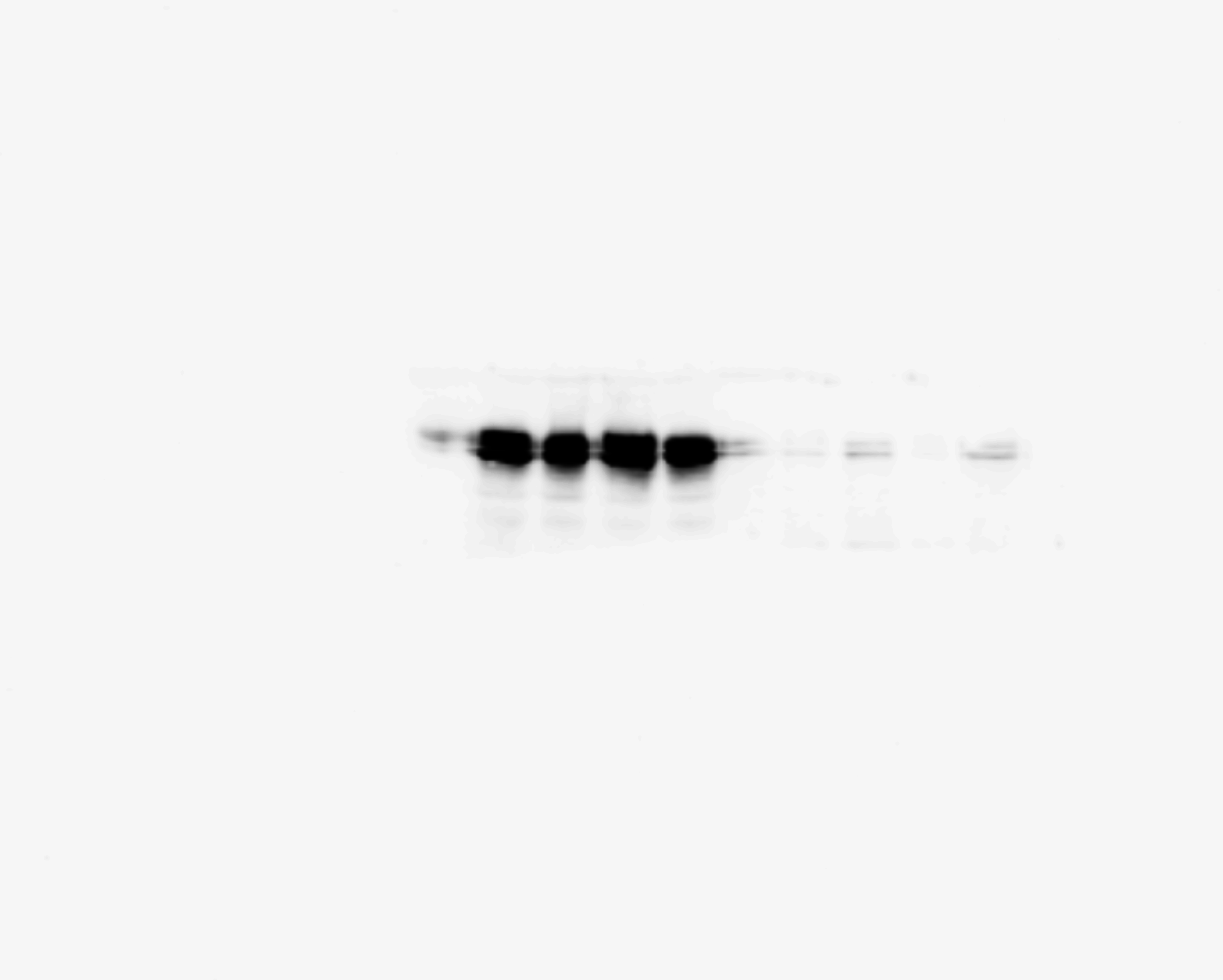

Supplement: Supplementary file 4 — Source data Fig. 3 [file 44319_2025_493_MOESM4_ESM.zip › Figure3/Fig3F/AntiPRP40blotwithadjustedbrightnessANDcontrast_lowexposure.tif]

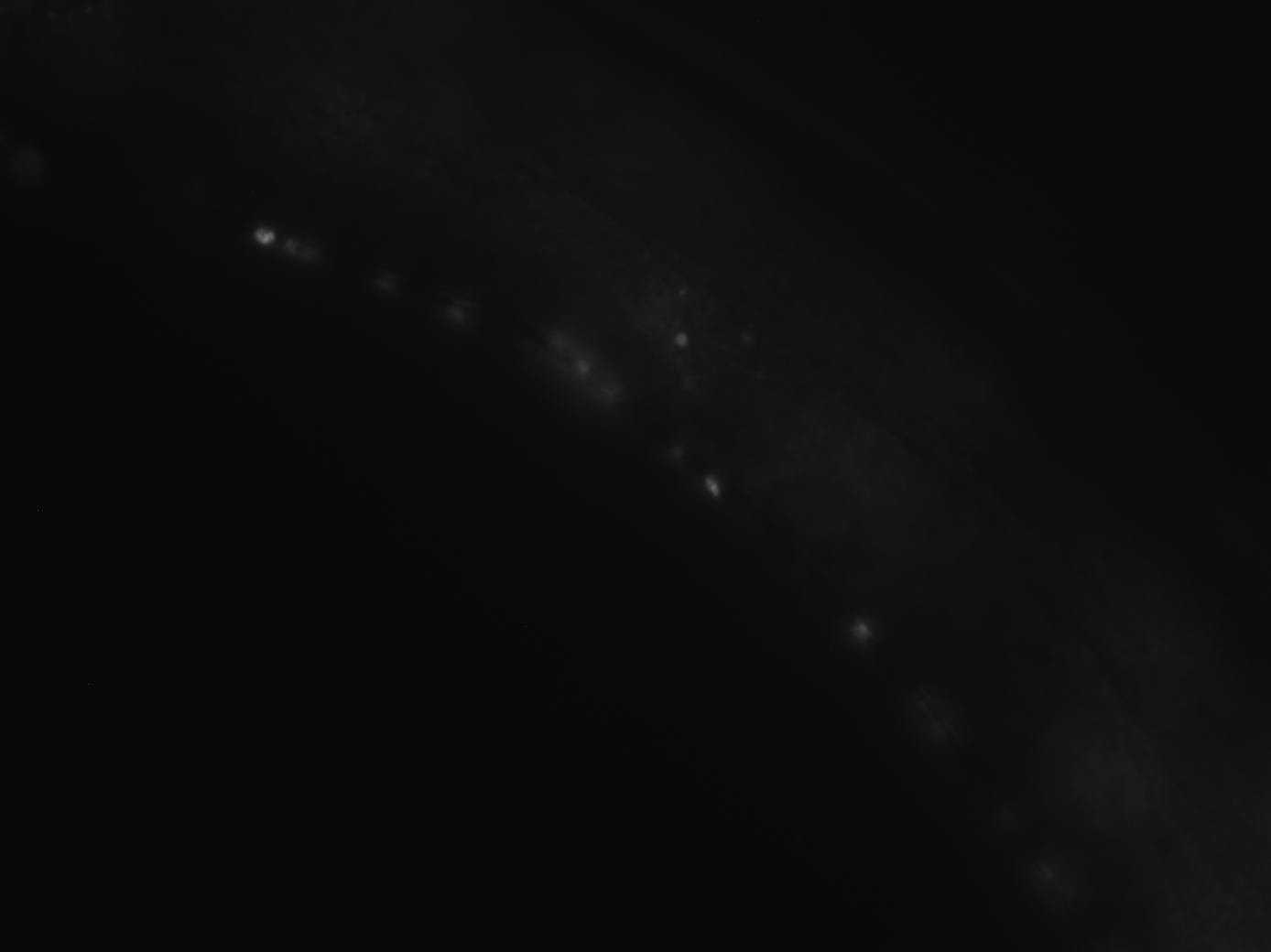

Supplement: Supplementary file 4 — Source data Fig. 3 [file 44319_2025_493_MOESM4_ESM.zip › Figure3/Fig3A/Experiment-14_VC_upstreamdeletion.tif_files/Experiment-14_z12c1x0-1388y0-1040.tif]

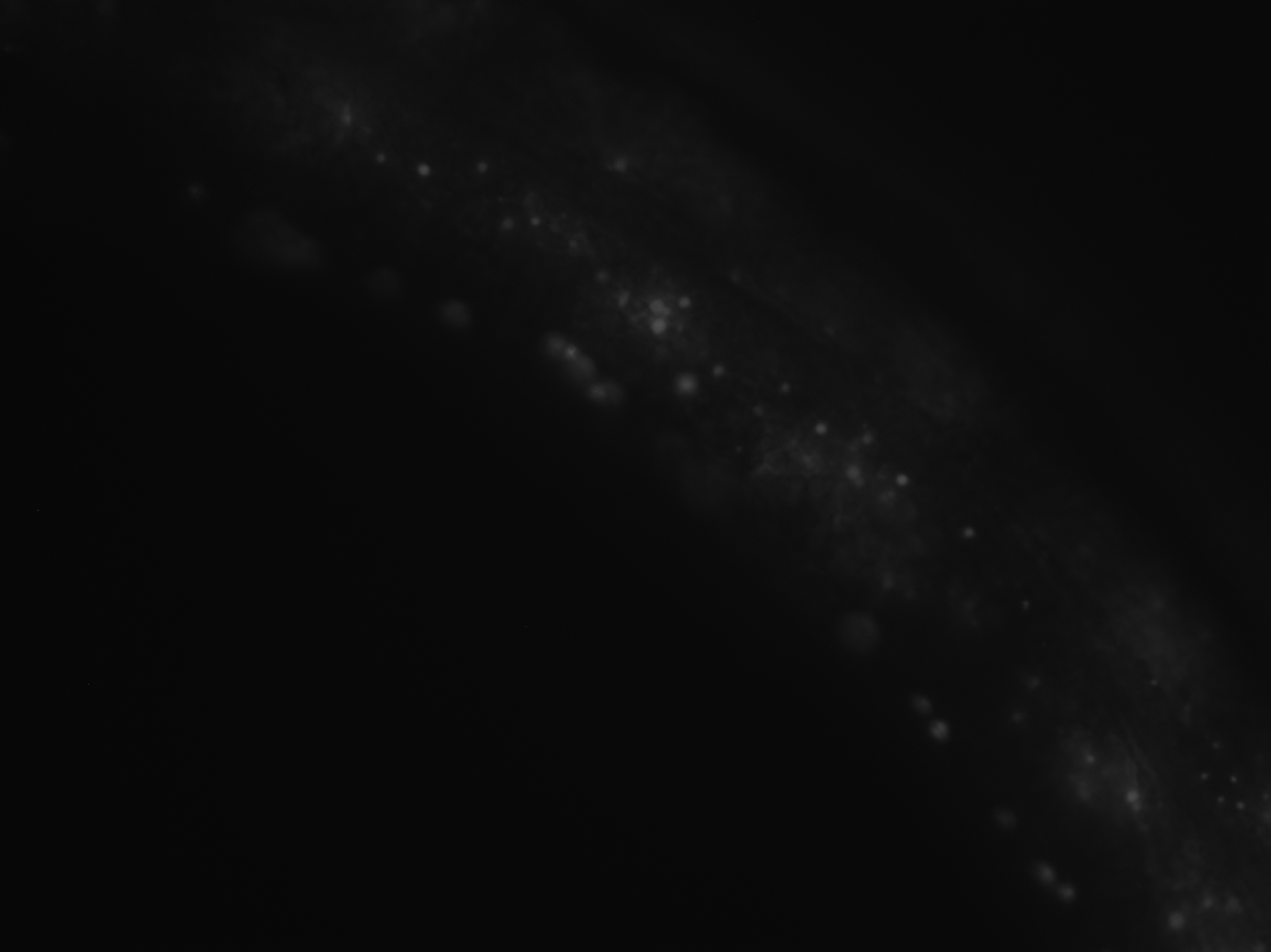

Supplement: Supplementary file 4 — Source data Fig. 3 [file 44319_2025_493_MOESM4_ESM.zip › Figure3/Fig3A/Experiment-14_VC_upstreamdeletion.tif_files/Experiment-14_z18c1x0-1388y0-1040.tif]

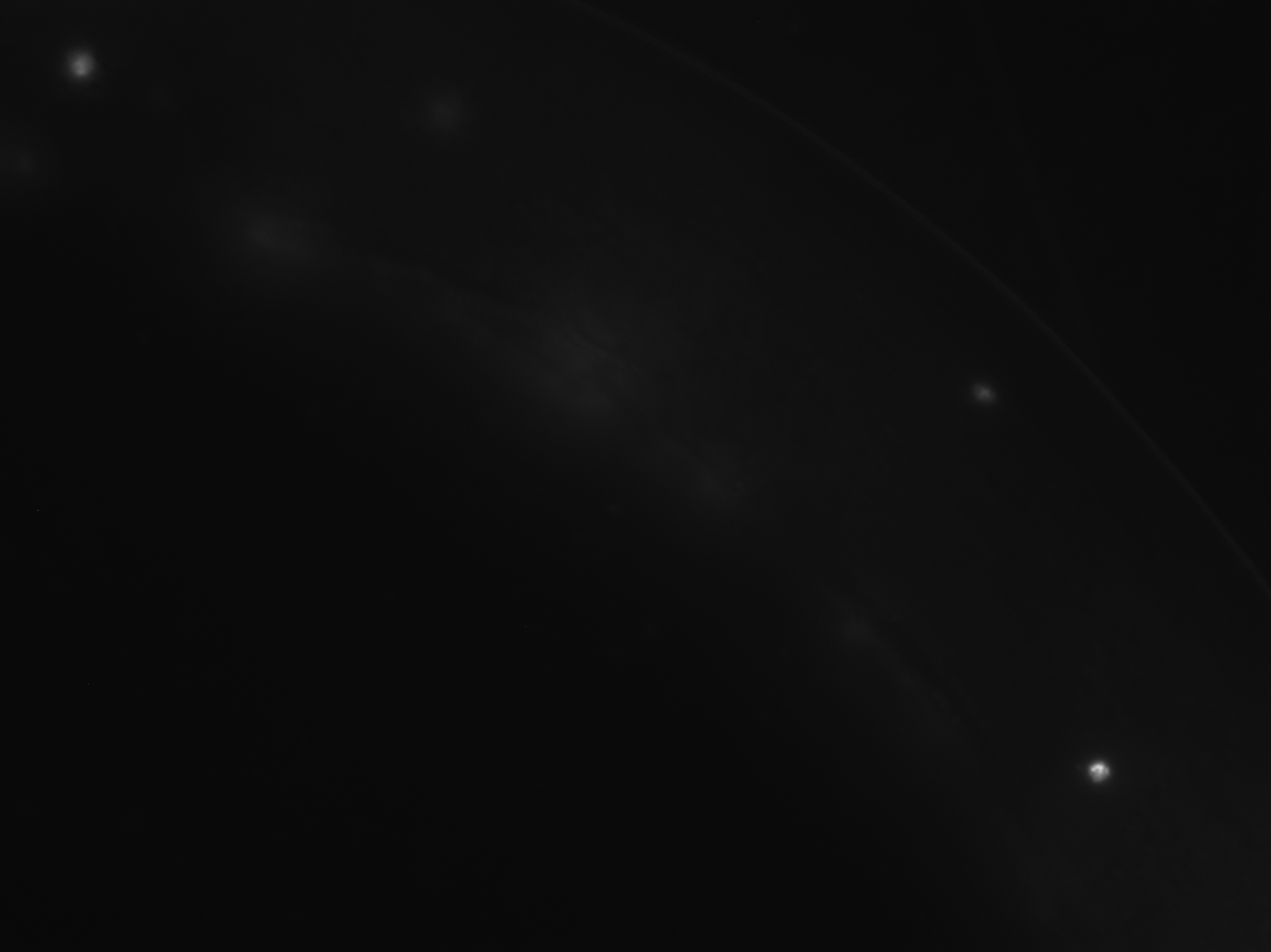

Supplement: Supplementary file 4 — Source data Fig. 3 [file 44319_2025_493_MOESM4_ESM.zip › Figure3/Fig3A/Experiment-14_VC_upstreamdeletion.tif_files/Experiment-14_z3c1x0-1388y0-1040.tif]

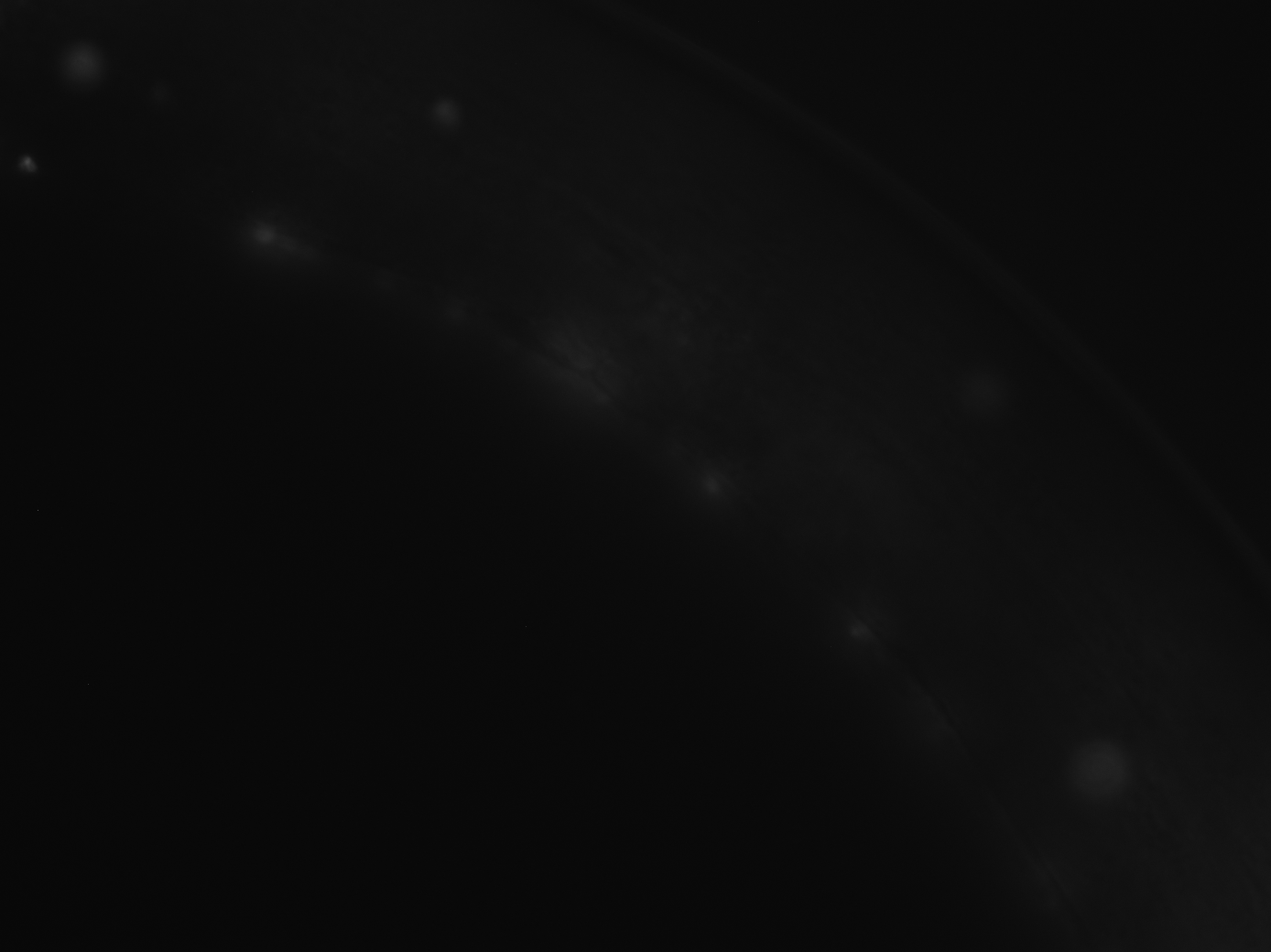

Supplement: Supplementary file 4 — Source data Fig. 3 [file 44319_2025_493_MOESM4_ESM.zip › Figure3/Fig3A/Experiment-14_VC_upstreamdeletion.tif_files/Experiment-14_z8c1x0-1388y0-1040.tif]

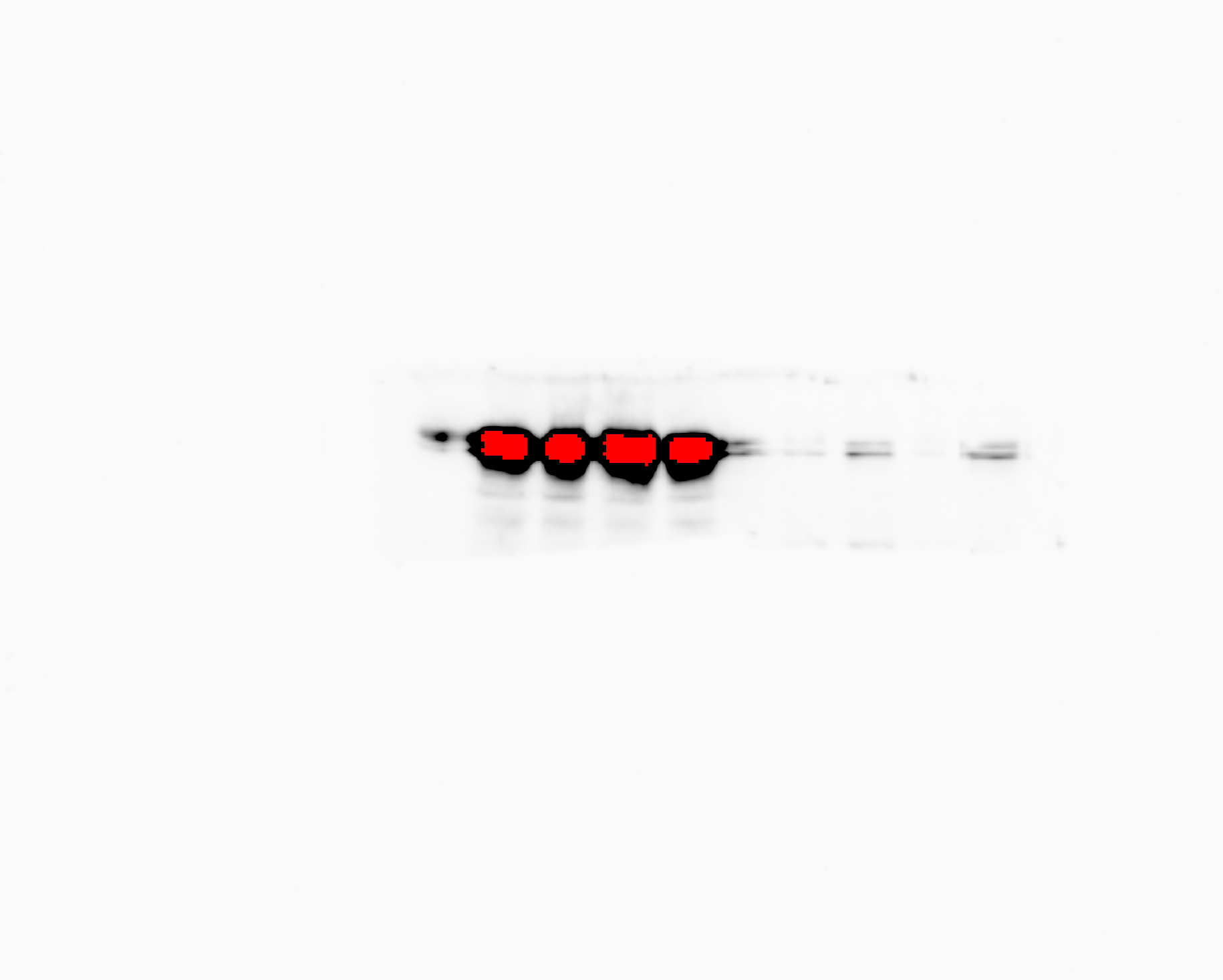

Supplement: Supplementary file 4 — Source data Fig. 3 [file 44319_2025_493_MOESM4_ESM.zip › Figure3/Fig3F/AntiPRP40blot1_highexposure.tif]

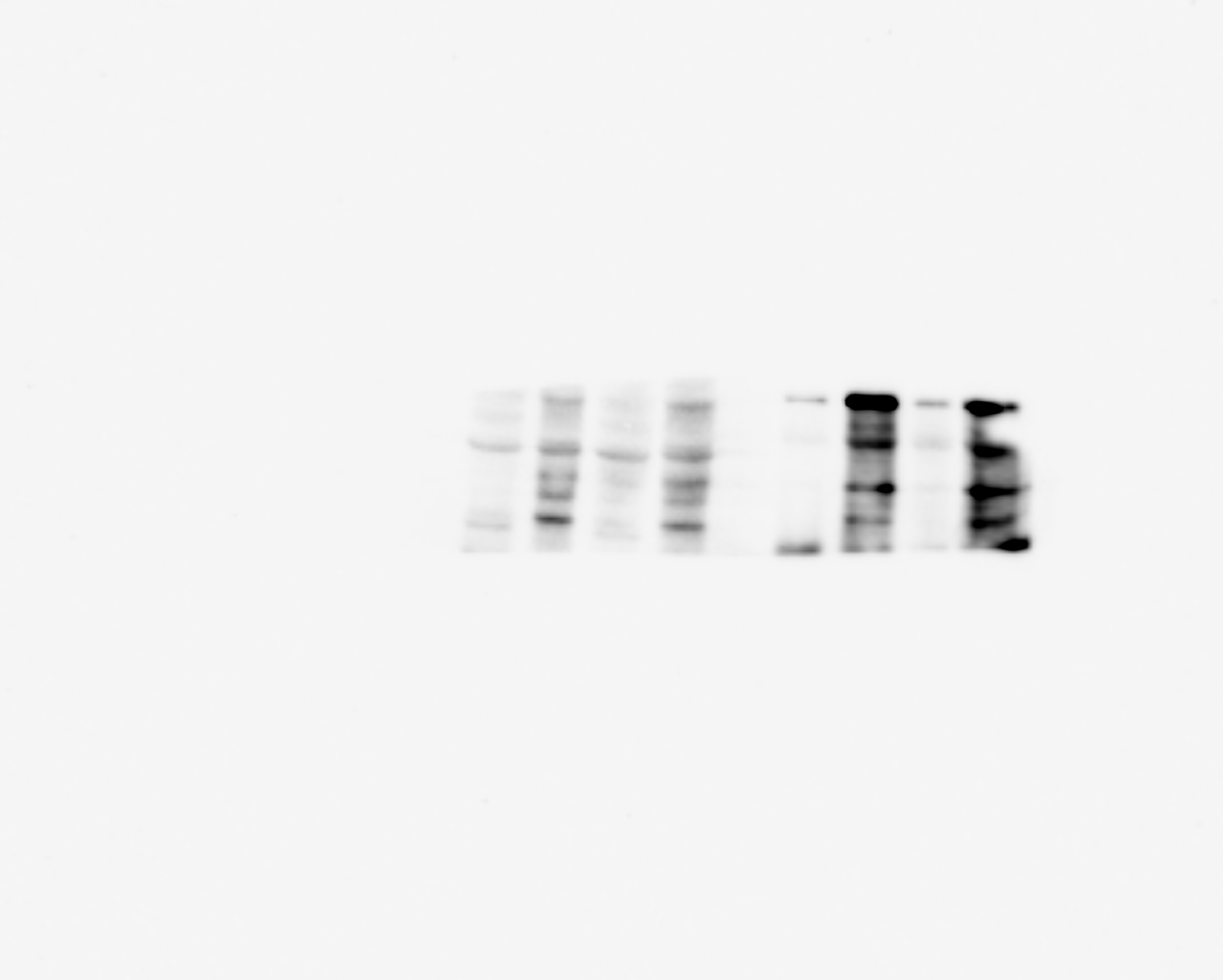

Supplement: Supplementary file 4 — Source data Fig. 3 [file 44319_2025_493_MOESM4_ESM.zip › Figure3/Fig3F/AntiMbl1FLAG.tif]

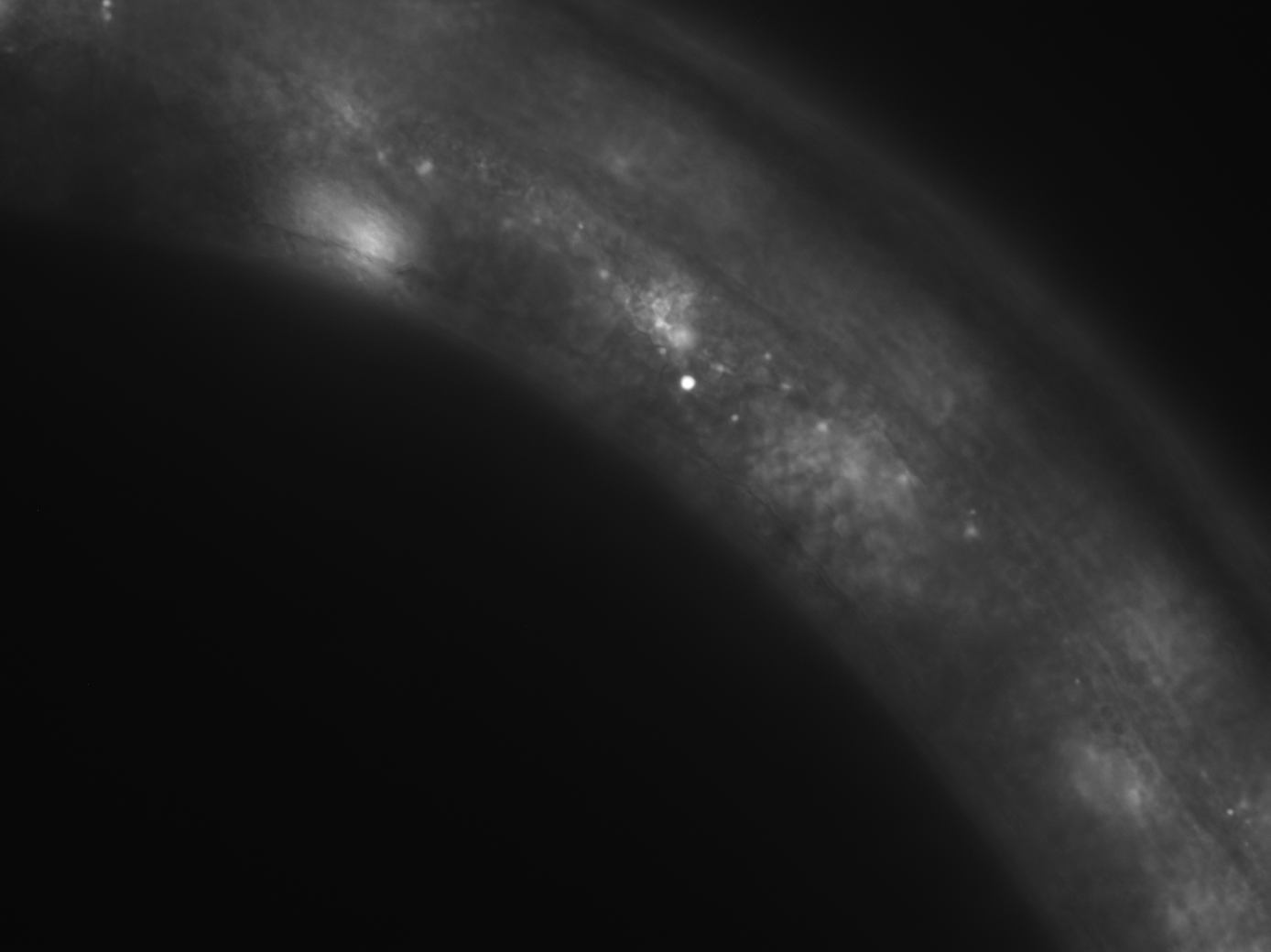

Supplement: Supplementary file 4 — Source data Fig. 3 [file 44319_2025_493_MOESM4_ESM.zip › Figure3/Fig3A/Experiment-14_VC_upstreamdeletion.tif_files/Experiment-14_z16c0x0-1388y0-1040.tif]

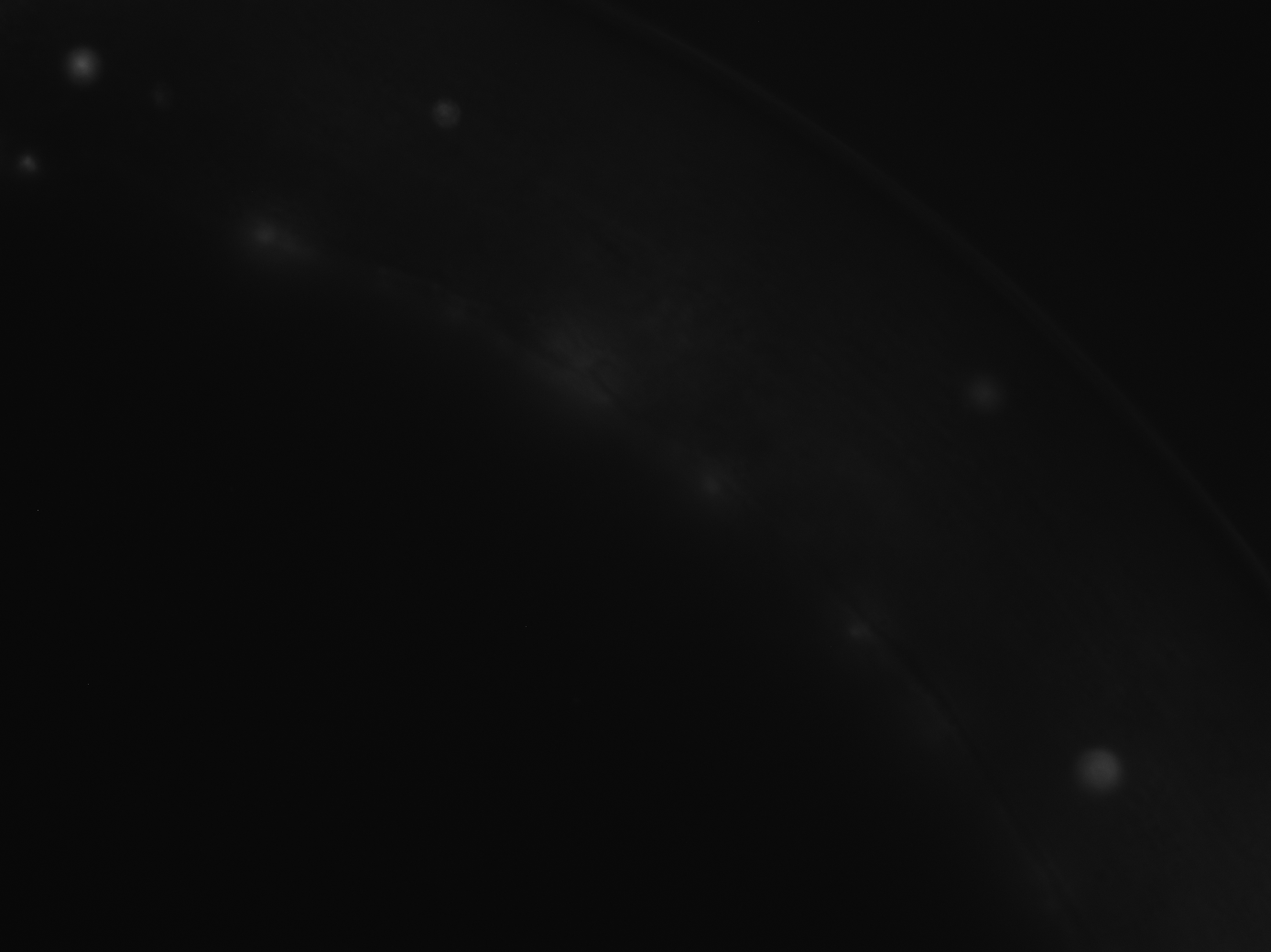

Supplement: Supplementary file 4 — Source data Fig. 3 [file 44319_2025_493_MOESM4_ESM.zip › Figure3/Fig3A/Experiment-14_VC_upstreamdeletion.tif_files/Experiment-14_z7c1x0-1388y0-1040.tif]

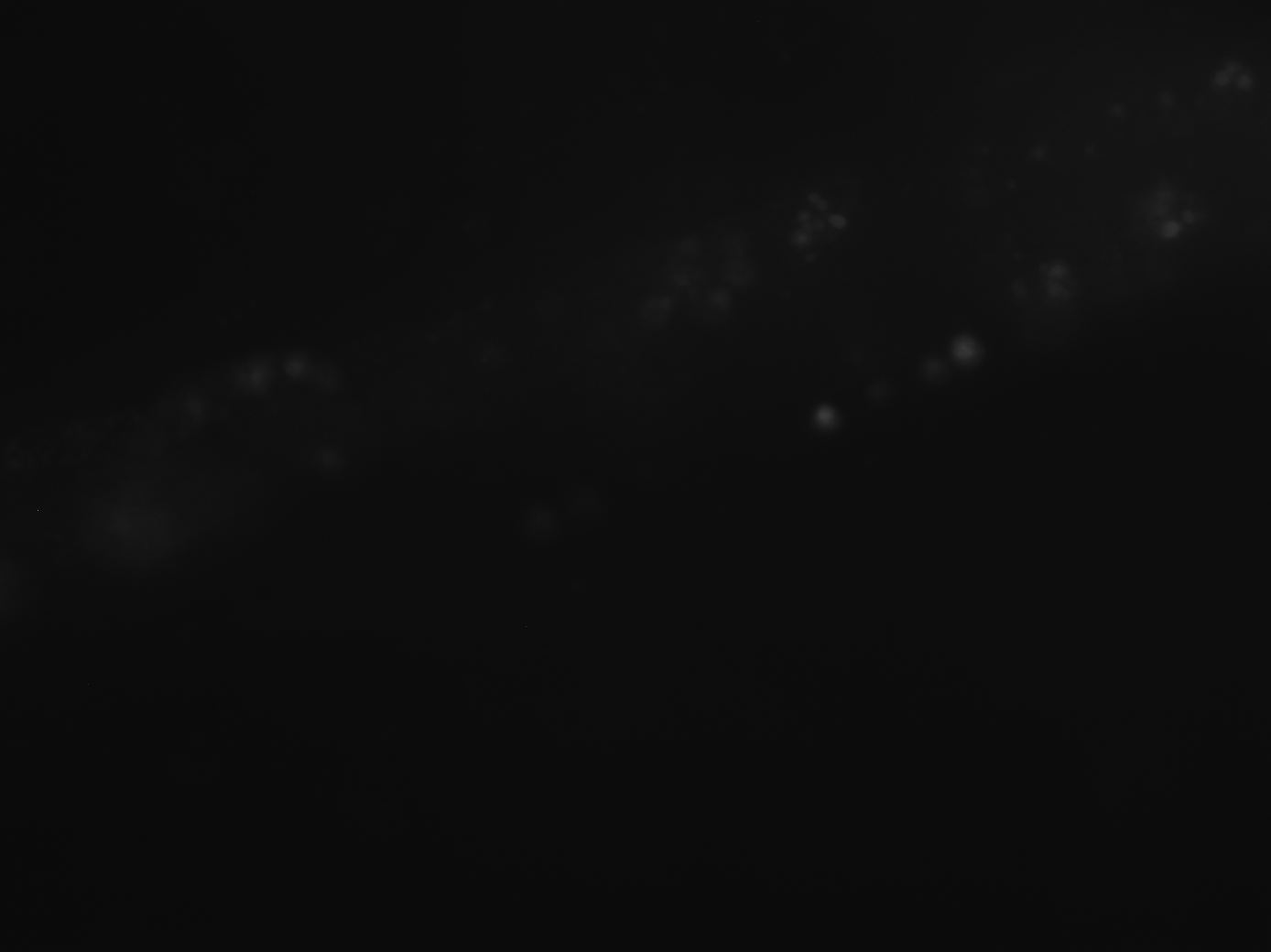

Supplement: Supplementary file 4 — Source data Fig. 3 [file 44319_2025_493_MOESM4_ESM.zip › Figure3/Fig3A/Experiment-63_VC_downstreamdeletion.tif_files/Experiment-63good_z11c1x0-1388y0-1040.tif]

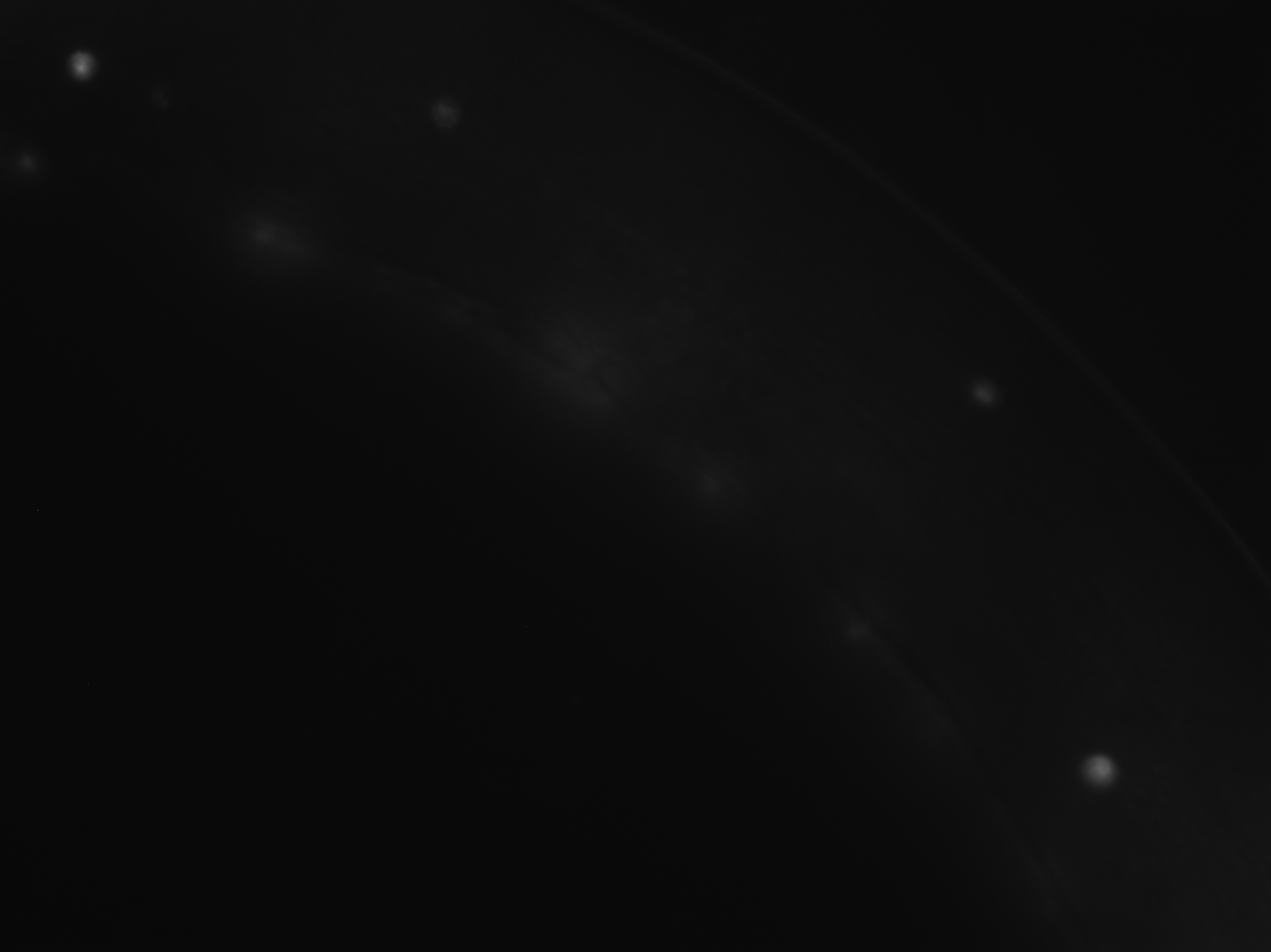

Supplement: Supplementary file 4 — Source data Fig. 3 [file 44319_2025_493_MOESM4_ESM.zip › Figure3/Fig3A/Experiment-14_VC_upstreamdeletion.tif_files/Experiment-14_z6c1x0-1388y0-1040.tif]

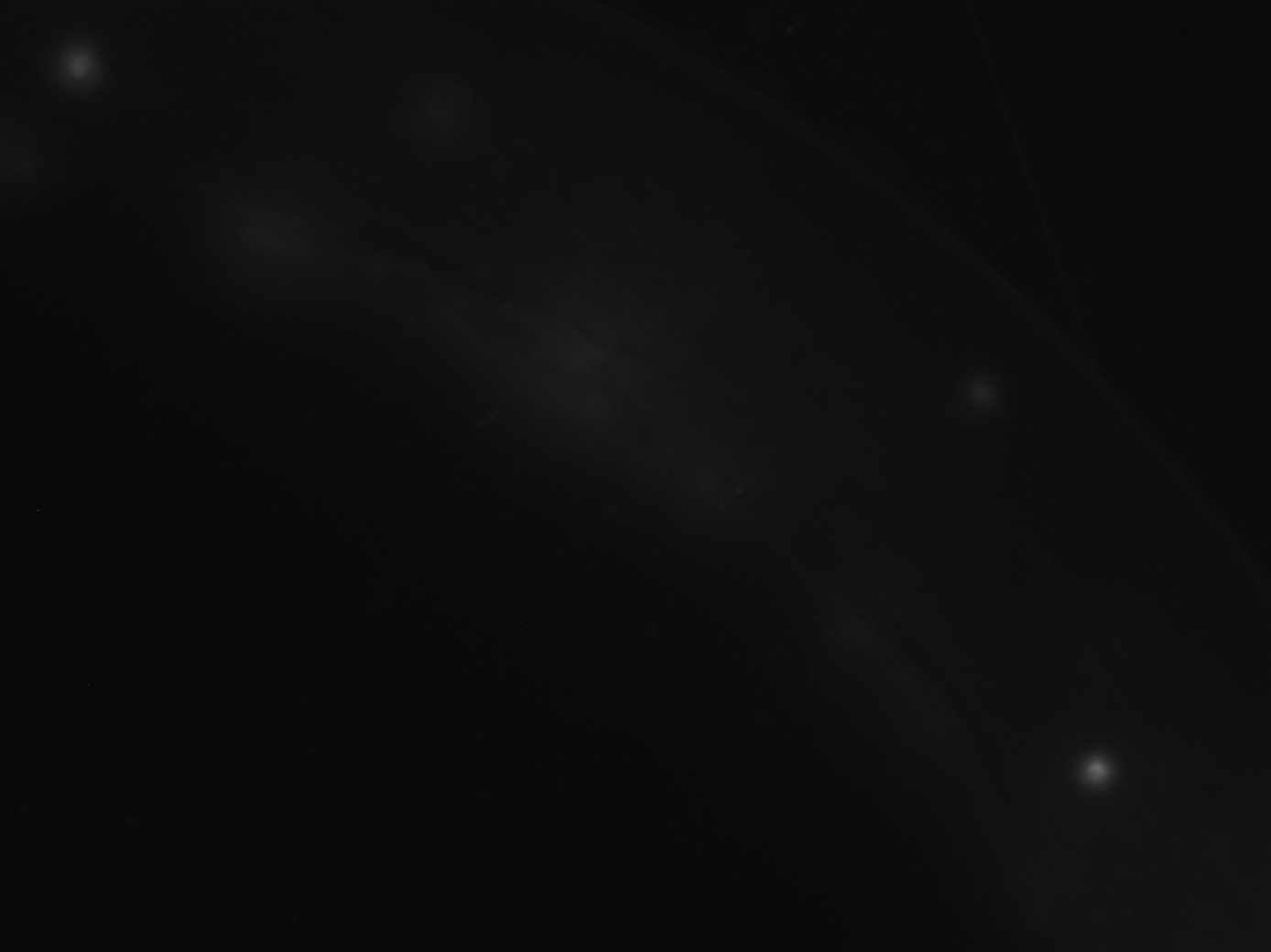

Supplement: Supplementary file 4 — Source data Fig. 3 [file 44319_2025_493_MOESM4_ESM.zip › Figure3/Fig3A/Experiment-14_VC_upstreamdeletion.tif_files/Experiment-14_z1c1x0-1388y0-1040.tif]

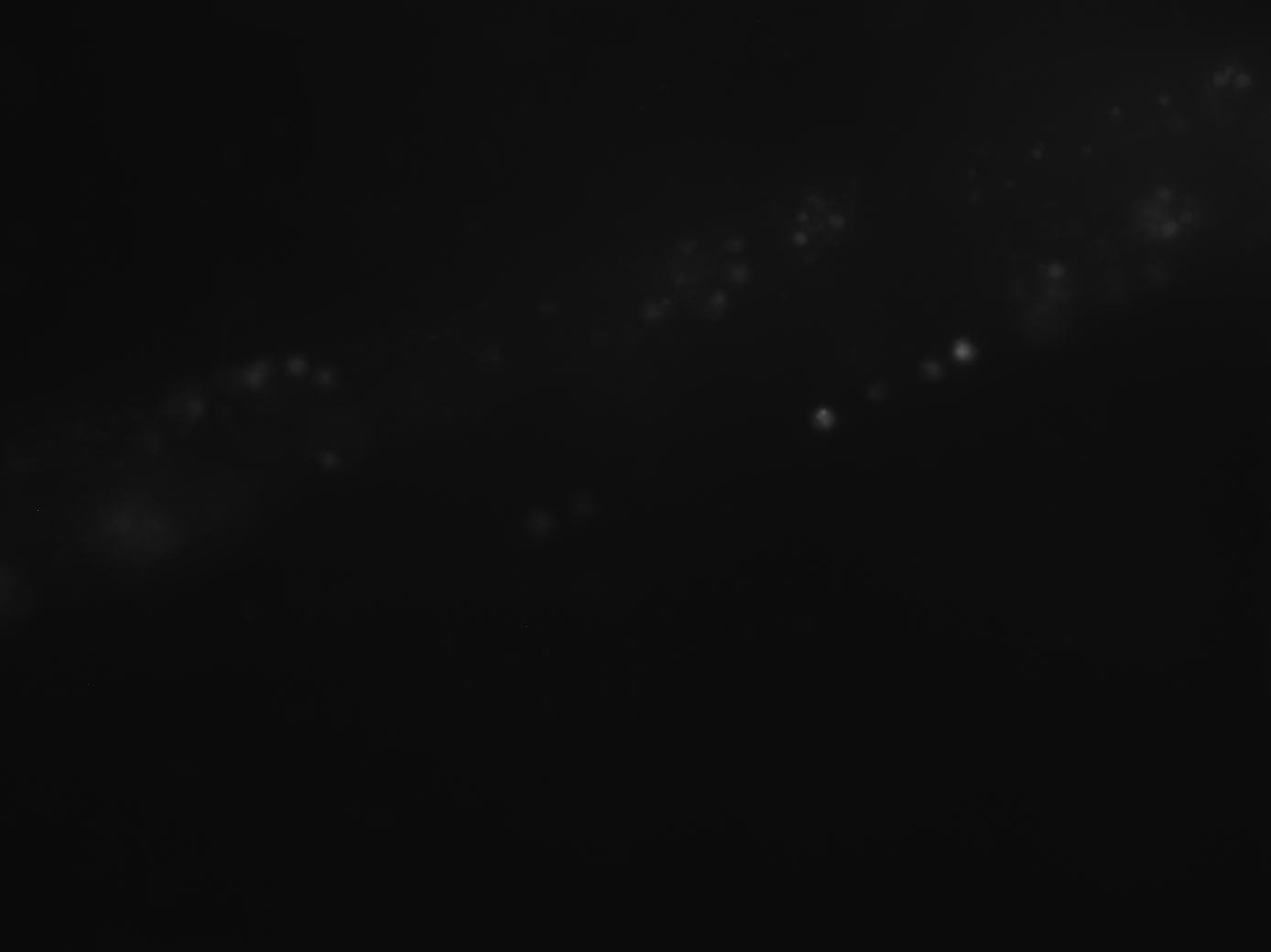

Supplement: Supplementary file 4 — Source data Fig. 3 [file 44319_2025_493_MOESM4_ESM.zip › Figure3/Fig3A/Experiment-63_VC_downstreamdeletion.tif_files/Experiment-63good_z10c1x0-1388y0-1040.tif]

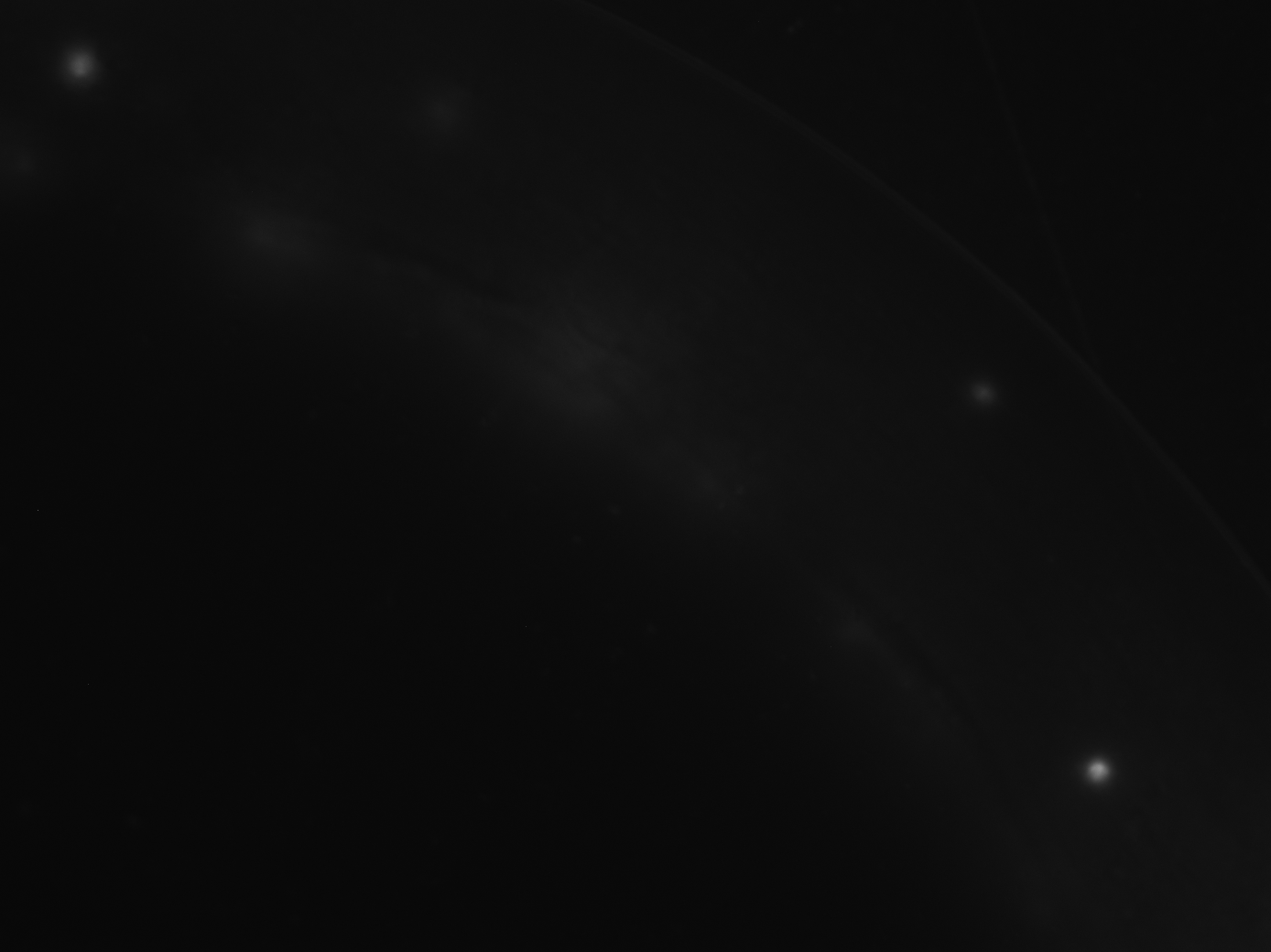

Supplement: Supplementary file 4 — Source data Fig. 3 [file 44319_2025_493_MOESM4_ESM.zip › Figure3/Fig3A/Experiment-14_VC_upstreamdeletion.tif_files/Experiment-14_z2c1x0-1388y0-1040.tif]

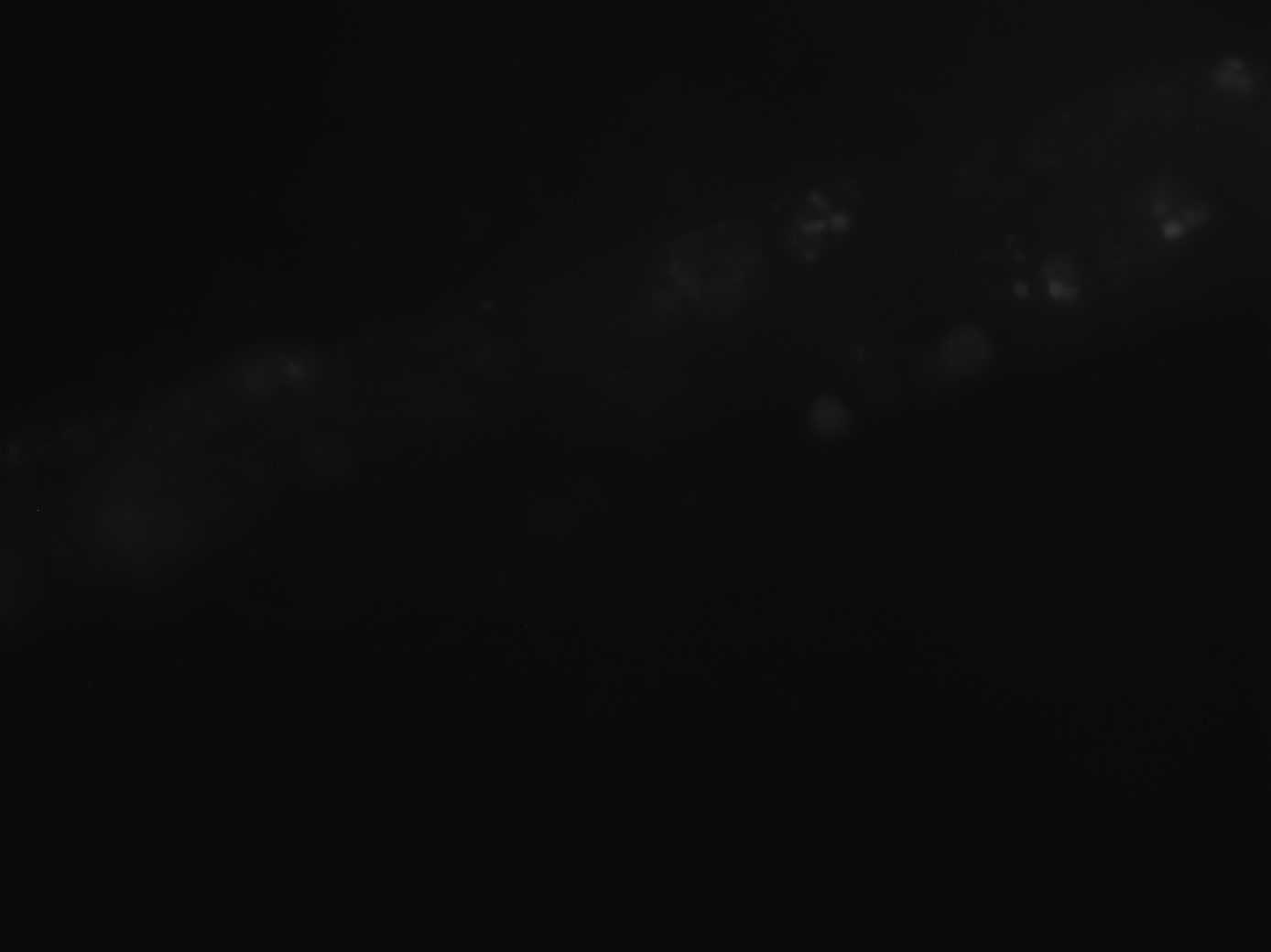

Supplement: Supplementary file 4 — Source data Fig. 3 [file 44319_2025_493_MOESM4_ESM.zip › Figure3/Fig3A/Experiment-63_VC_downstreamdeletion.tif_files/Experiment-63good_z13c1x0-1388y0-1040.tif]

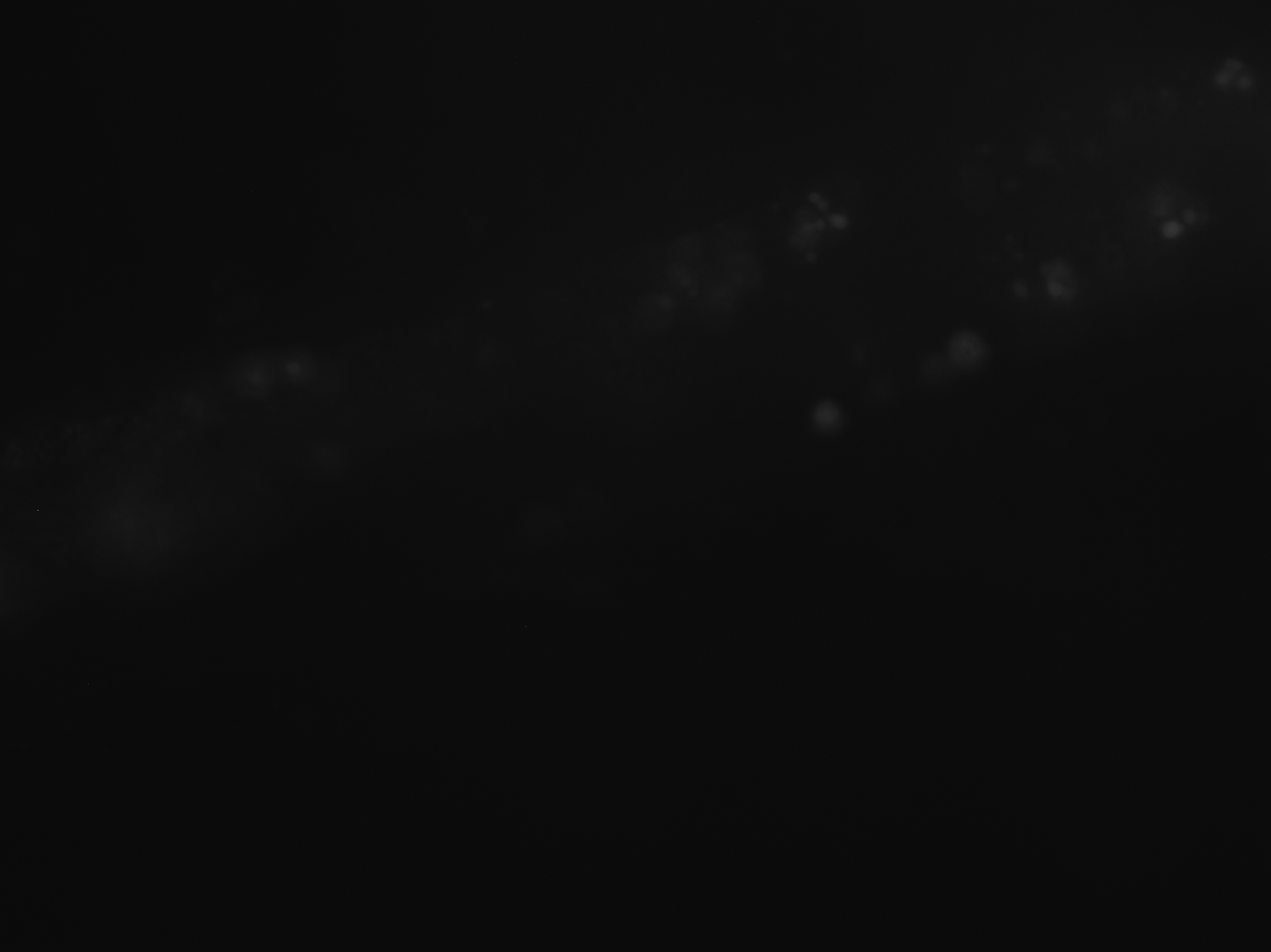

Supplement: Supplementary file 4 — Source data Fig. 3 [file 44319_2025_493_MOESM4_ESM.zip › Figure3/Fig3A/Experiment-63_VC_downstreamdeletion.tif_files/Experiment-63good_z12c1x0-1388y0-1040.tif]

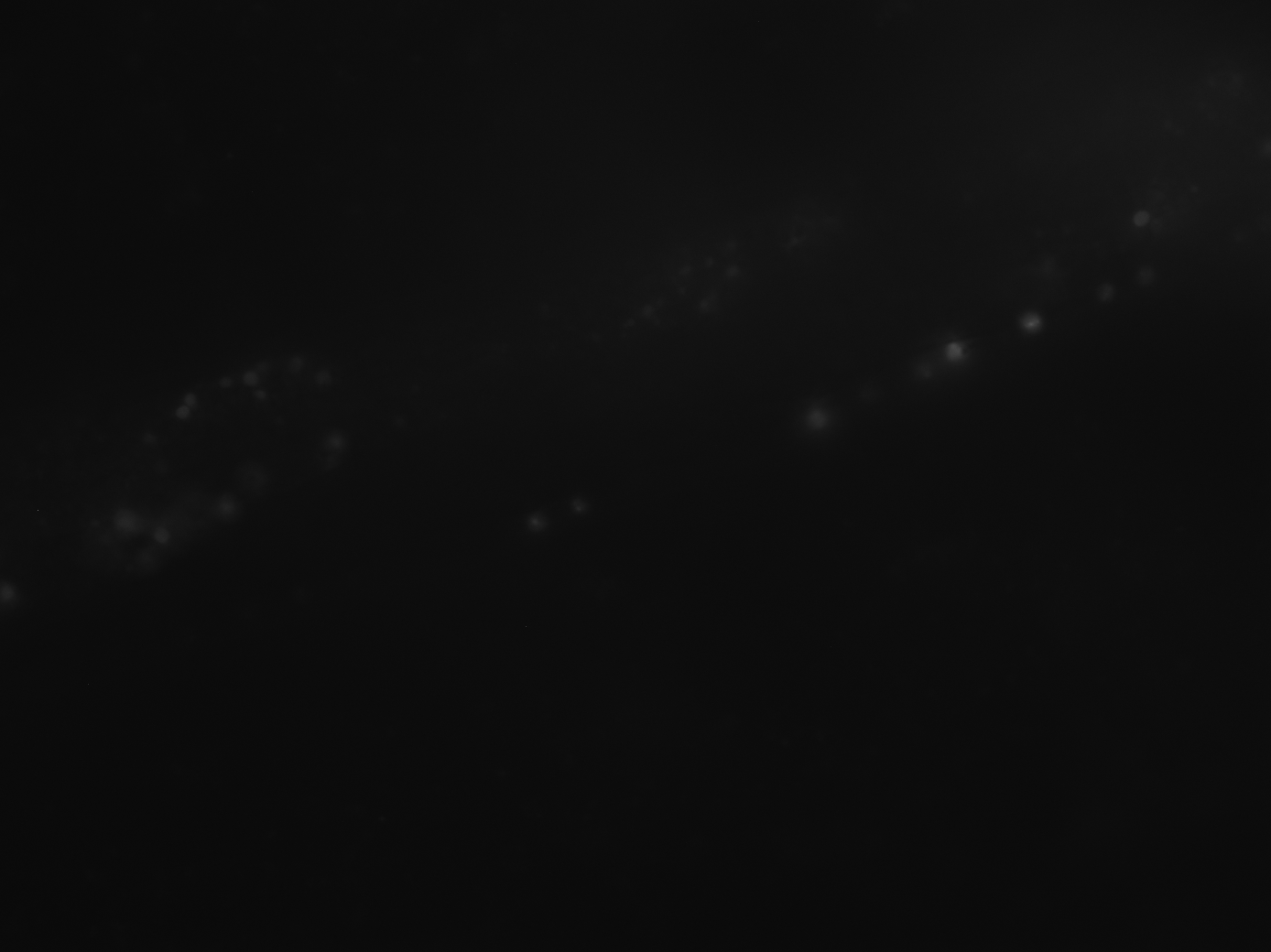

Supplement: Supplementary file 4 — Source data Fig. 3 [file 44319_2025_493_MOESM4_ESM.zip › Figure3/Fig3A/Experiment-63_VC_downstreamdeletion.tif_files/Experiment-63good_z6c1x0-1388y0-1040.tif]

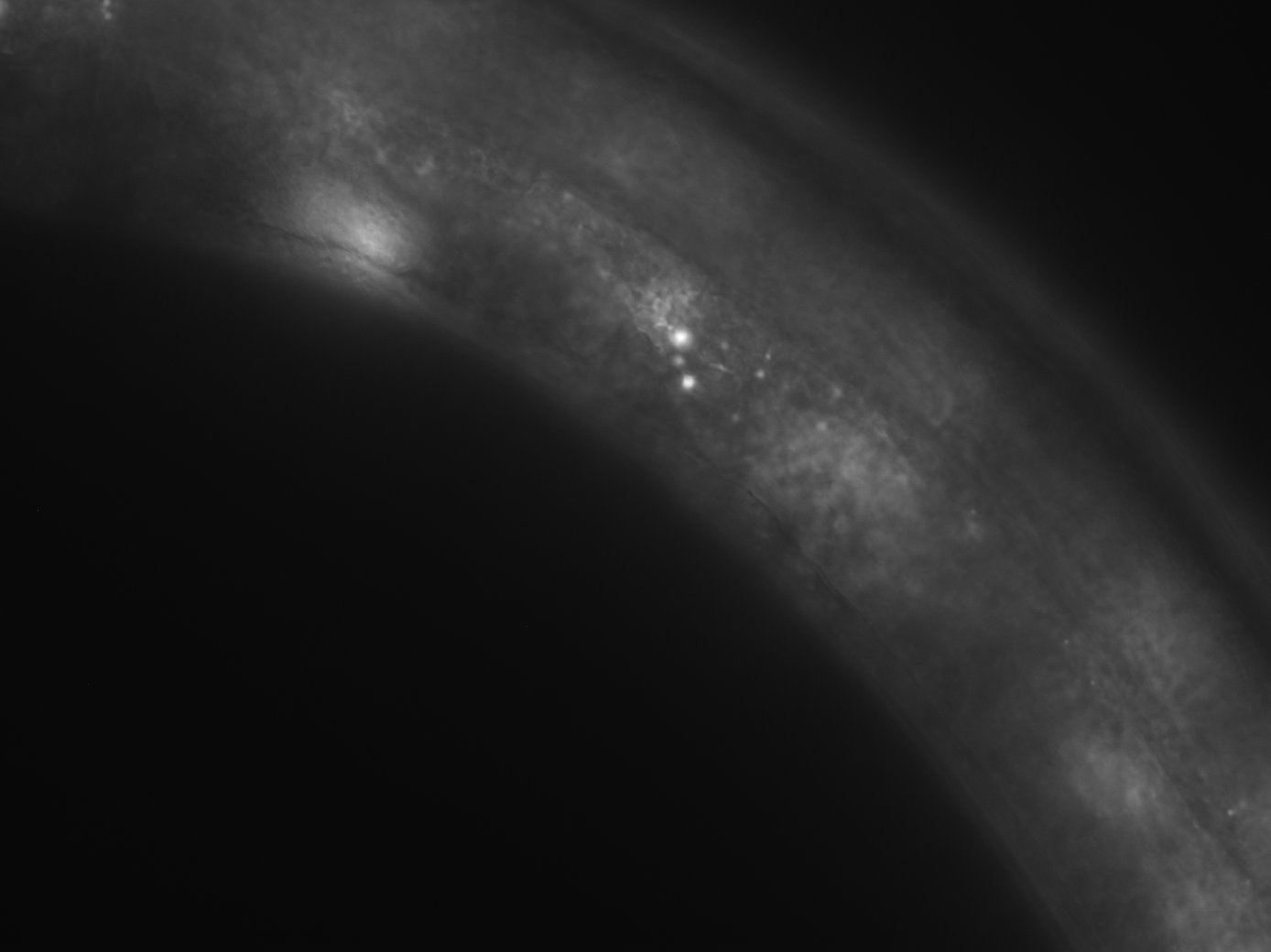

Supplement: Supplementary file 4 — Source data Fig. 3 [file 44319_2025_493_MOESM4_ESM.zip › Figure3/Fig3A/Experiment-14_VC_upstreamdeletion.tif_files/Experiment-14_z15c0x0-1388y0-1040.tif]

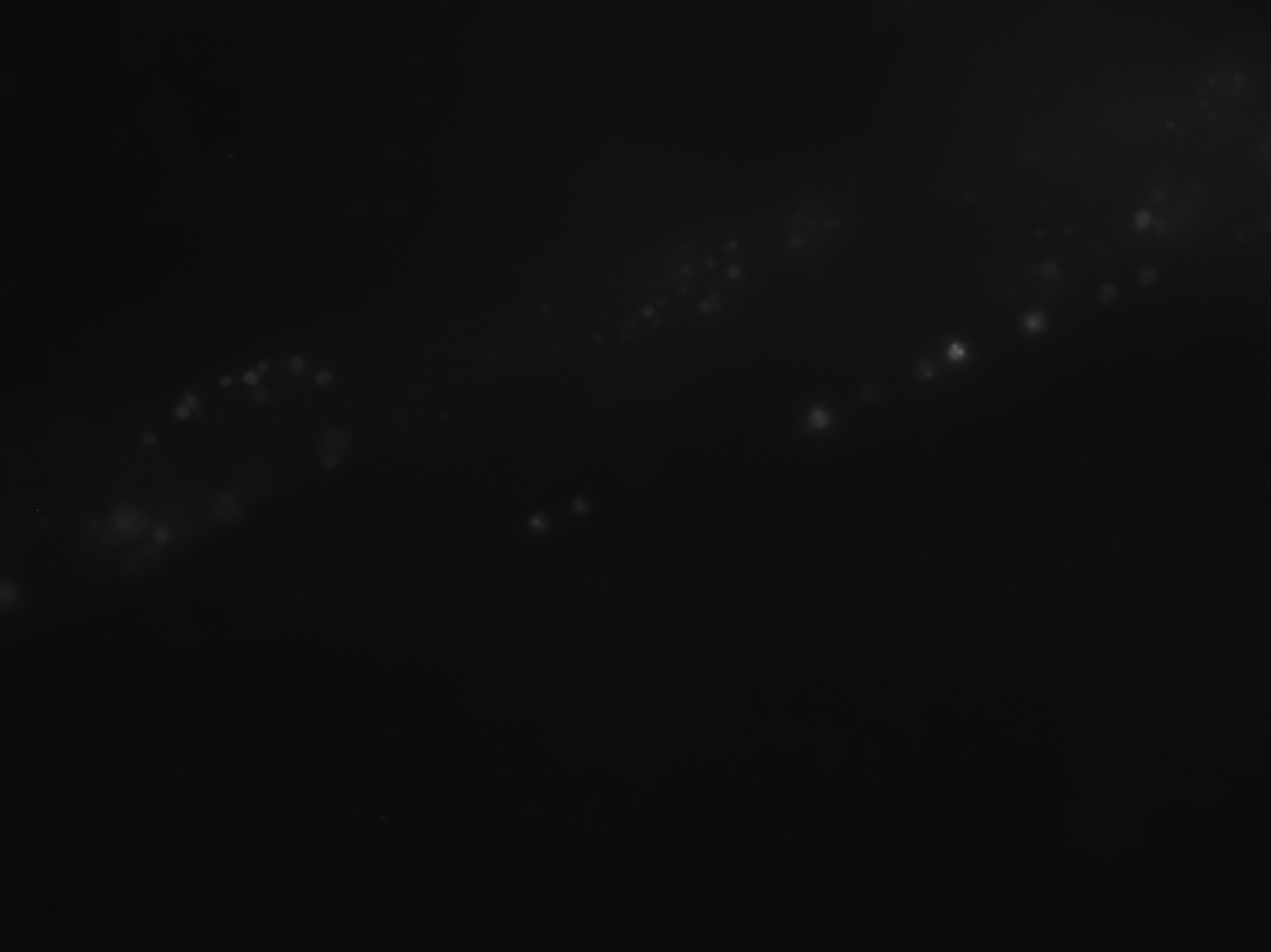

Supplement: Supplementary file 4 — Source data Fig. 3 [file 44319_2025_493_MOESM4_ESM.zip › Figure3/Fig3A/Experiment-63_VC_downstreamdeletion.tif_files/Experiment-63good_z7c1x0-1388y0-1040.tif]

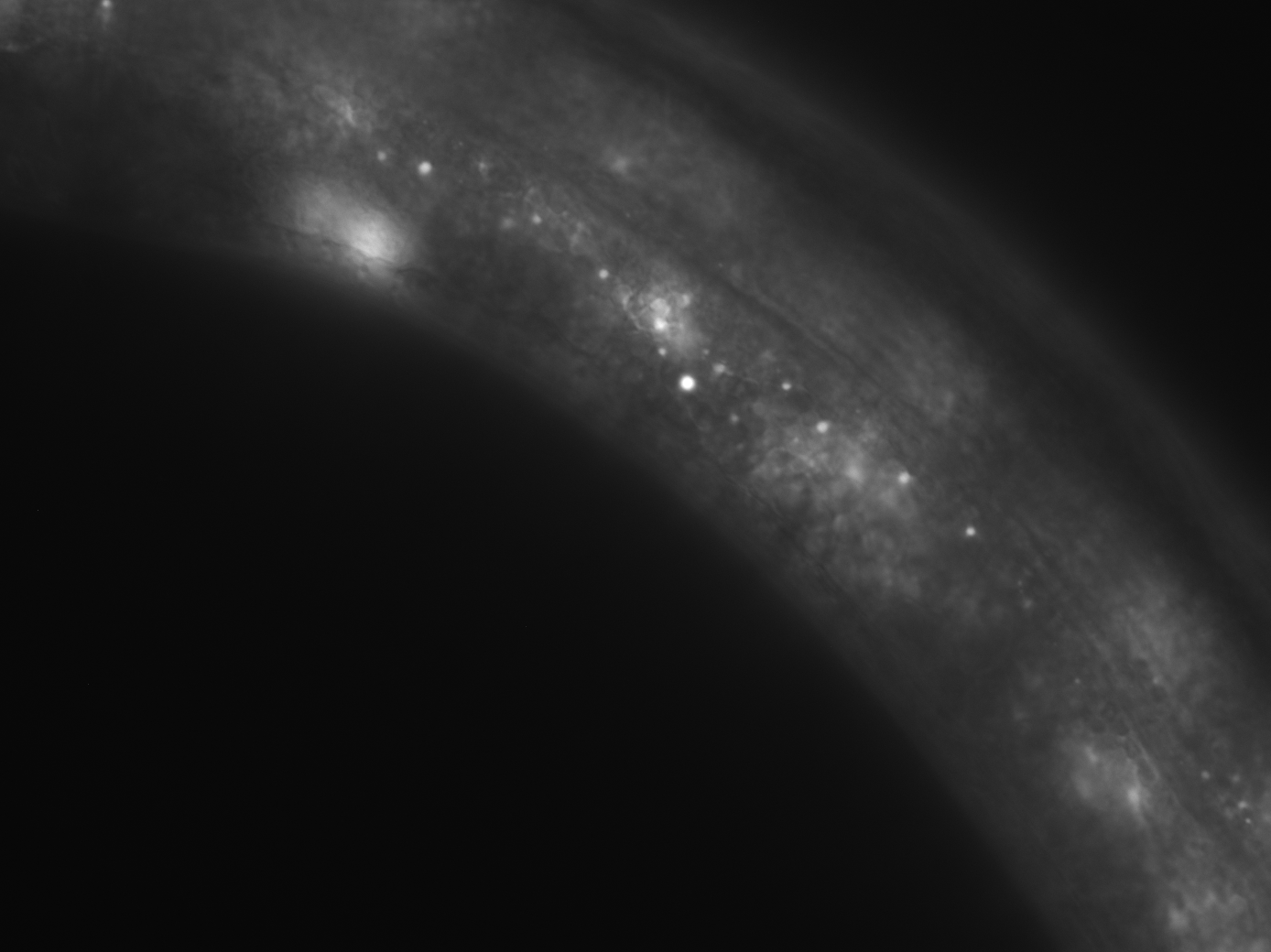

Supplement: Supplementary file 4 — Source data Fig. 3 [file 44319_2025_493_MOESM4_ESM.zip › Figure3/Fig3A/Experiment-14_VC_upstreamdeletion.tif_files/Experiment-14_z17c0x0-1388y0-1040.tif]

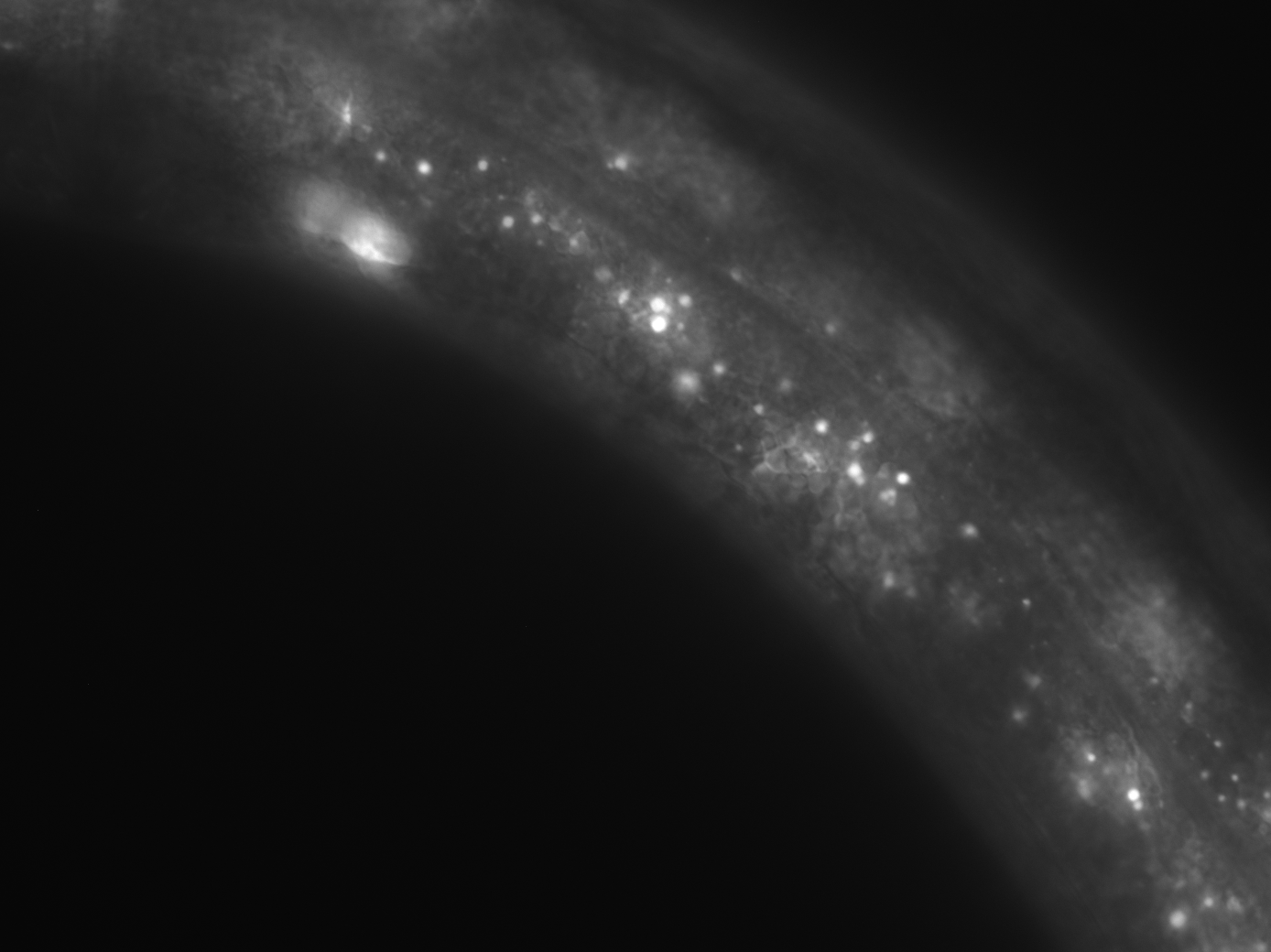

Supplement: Supplementary file 4 — Source data Fig. 3 [file 44319_2025_493_MOESM4_ESM.zip › Figure3/Fig3A/Experiment-14_VC_upstreamdeletion.tif_files/Experiment-14_z19c0x0-1388y0-1040.tif]

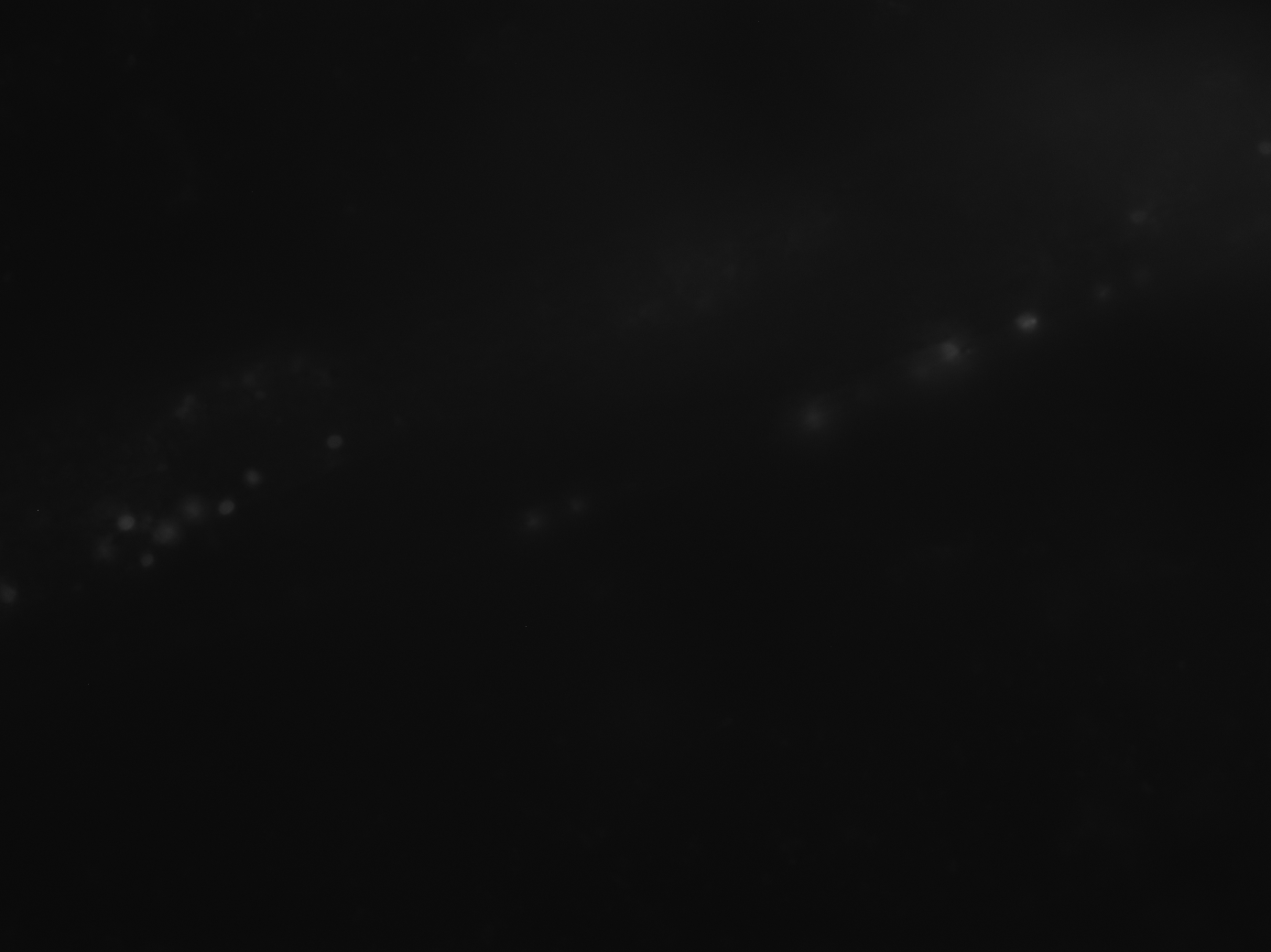

Supplement: Supplementary file 4 — Source data Fig. 3 [file 44319_2025_493_MOESM4_ESM.zip › Figure3/Fig3A/Experiment-63_VC_downstreamdeletion.tif_files/Experiment-63good_z4c1x0-1388y0-1040.tif]

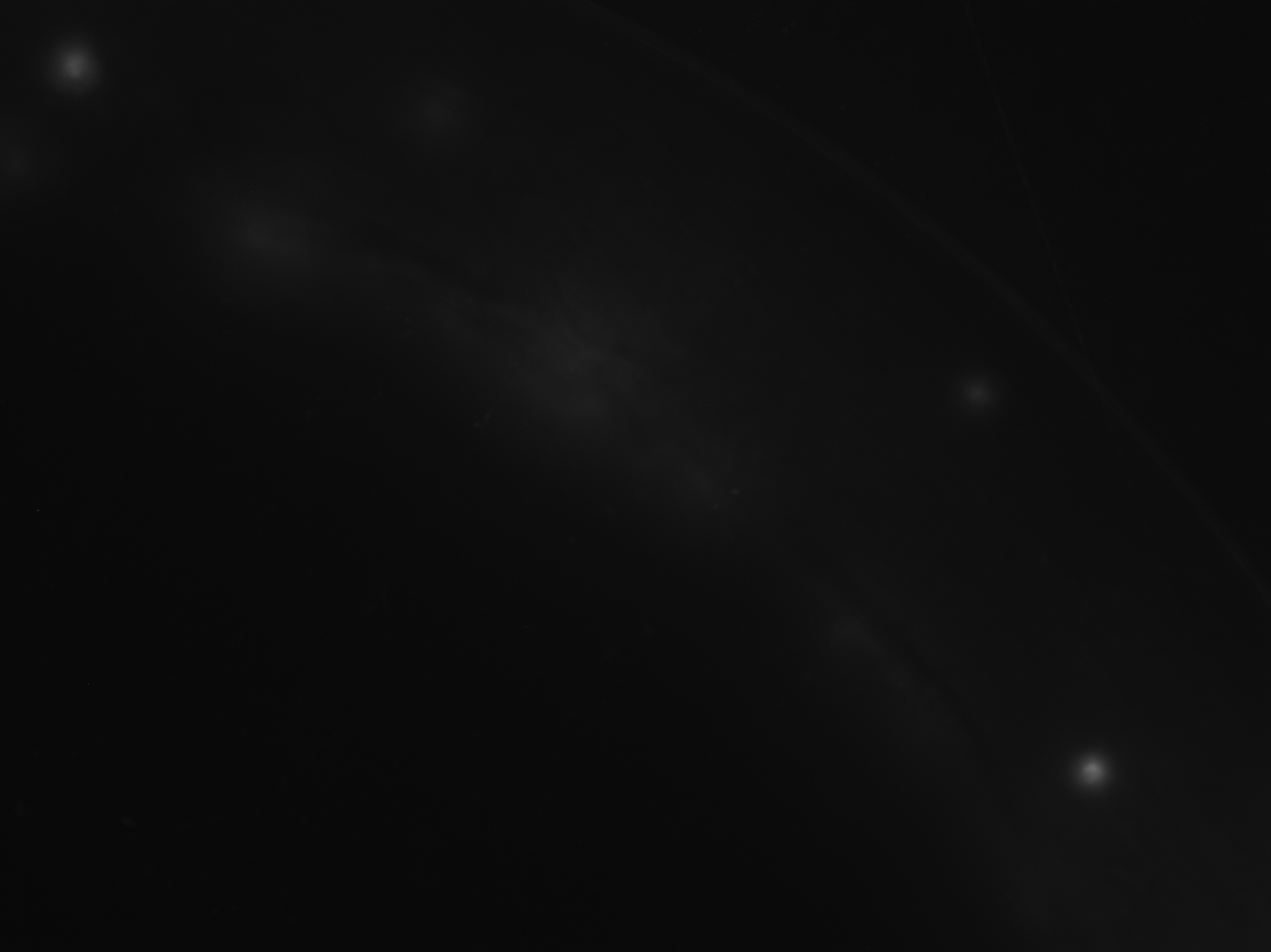

Supplement: Supplementary file 4 — Source data Fig. 3 [file 44319_2025_493_MOESM4_ESM.zip › Figure3/Fig3A/Experiment-14_VC_upstreamdeletion.tif_files/Experiment-14_z0c1x0-1388y0-1040.tif]

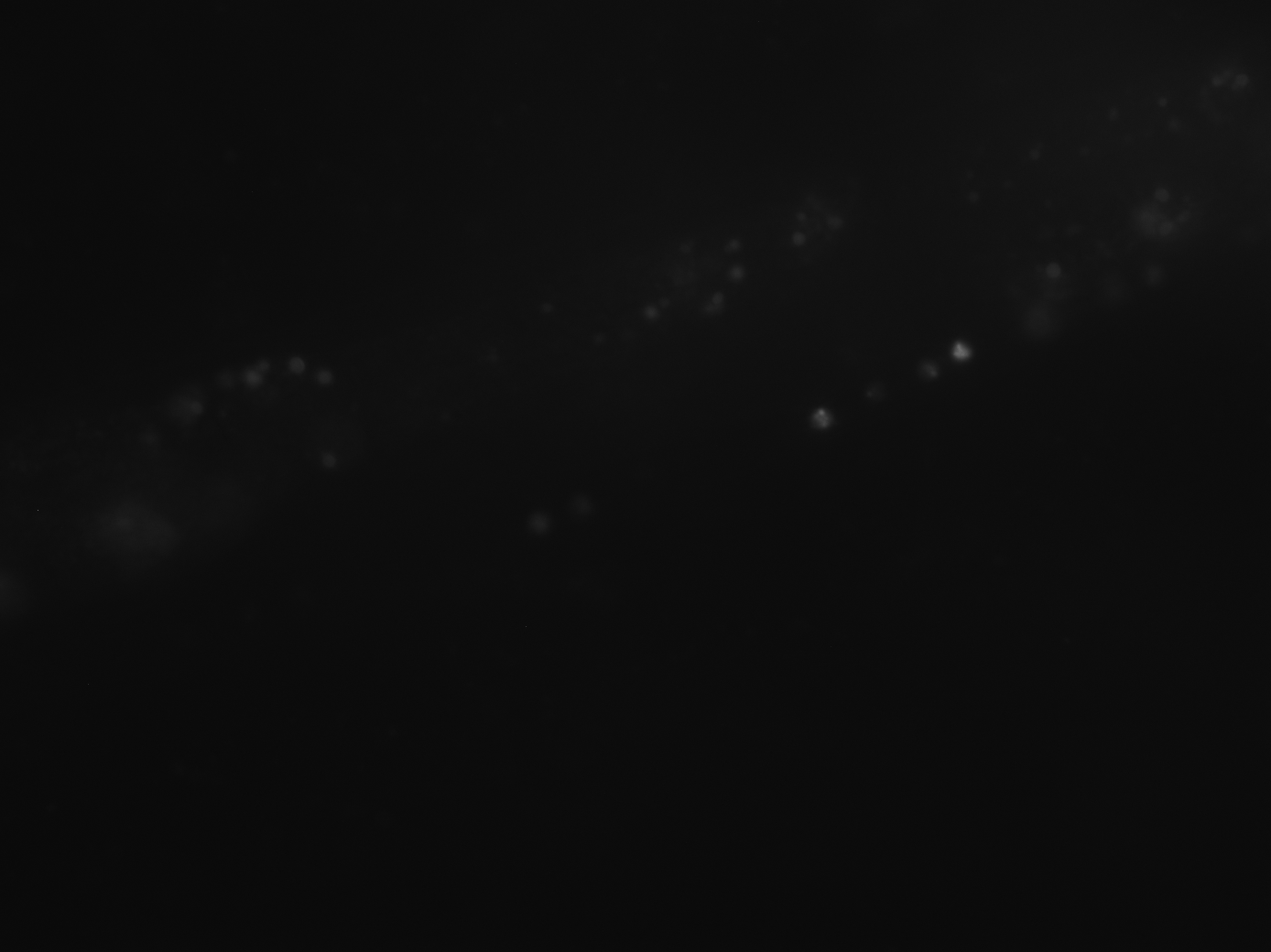

Supplement: Supplementary file 4 — Source data Fig. 3 [file 44319_2025_493_MOESM4_ESM.zip › Figure3/Fig3A/Experiment-63_VC_downstreamdeletion.tif_files/Experiment-63good_z9c1x0-1388y0-1040.tif]

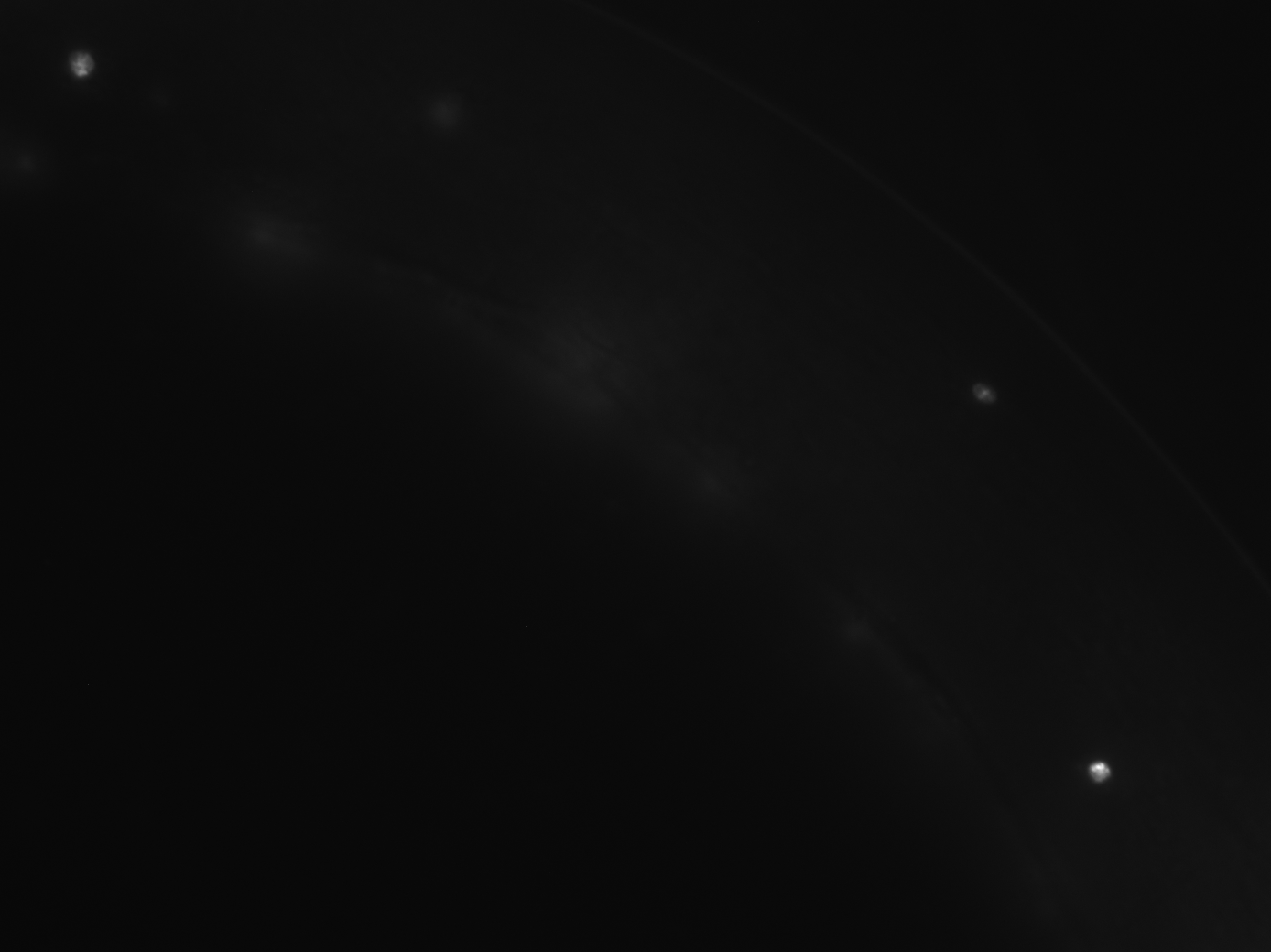

Supplement: Supplementary file 4 — Source data Fig. 3 [file 44319_2025_493_MOESM4_ESM.zip › Figure3/Fig3A/Experiment-14_VC_upstreamdeletion.tif_files/Experiment-14_z4c1x0-1388y0-1040.tif]
