# Supplementary figures and images for: Regulated microexon alternative splicing in single neurons tunes synaptic function (part 3 of 6)
Source: EMBO Rep. 2025 Jun 9;26(14):3640–62. doi: 10.1038/s44319-025-00493-7 (PMC12287369; doi:10.1038/s44319-025-00493-7)

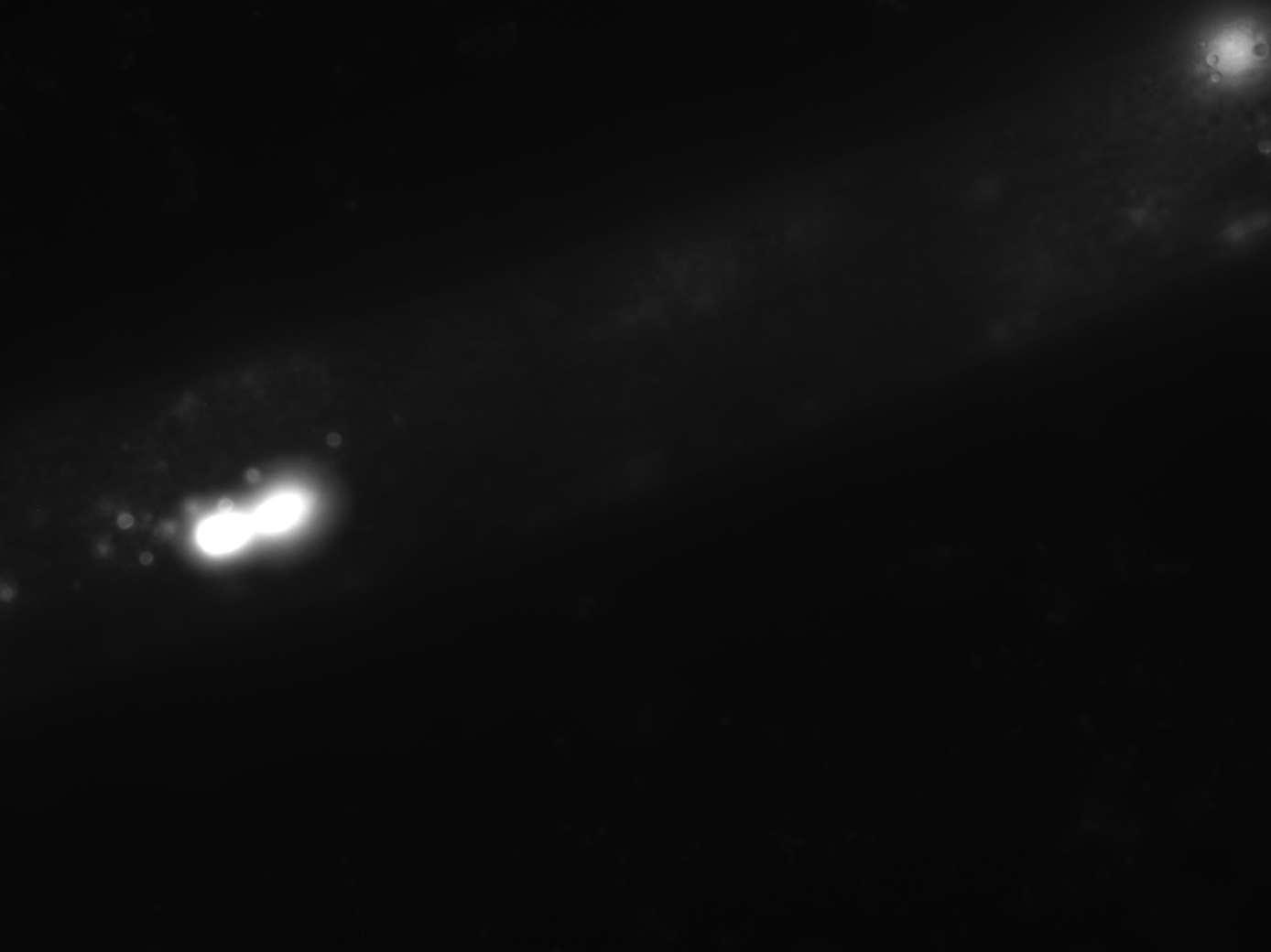

Supplement: Supplementary file 4 — Source data Fig. 3 [file 44319_2025_493_MOESM4_ESM.zip › Figure3/Fig3A/Experiment-63_VC_downstreamdeletion.tif_files/Experiment-63good_z4c0x0-1388y0-1040.tif]

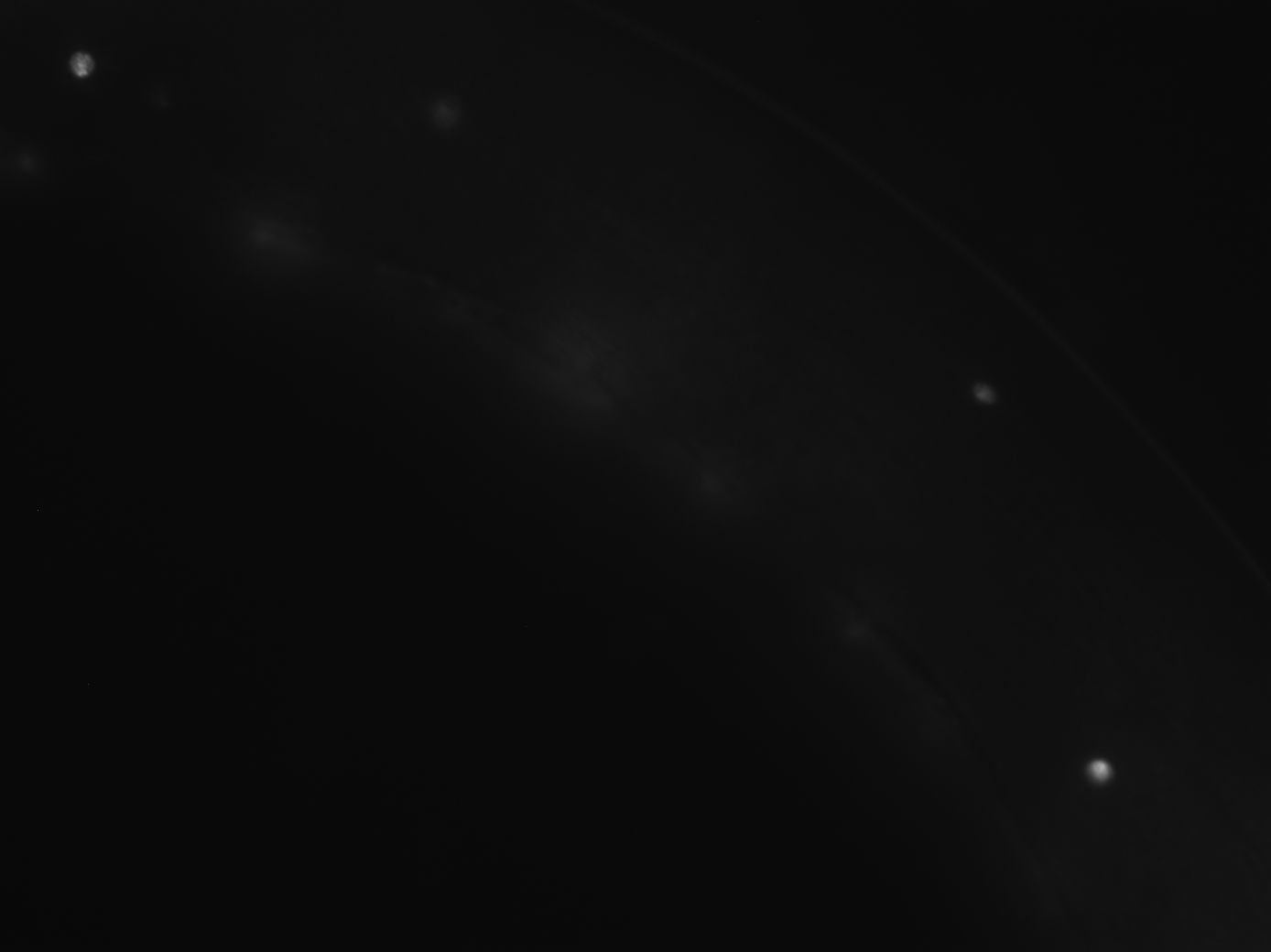

Supplement: Supplementary file 4 — Source data Fig. 3 [file 44319_2025_493_MOESM4_ESM.zip › Figure3/Fig3A/Experiment-14_VC_upstreamdeletion.tif_files/Experiment-14_z5c1x0-1388y0-1040.tif]

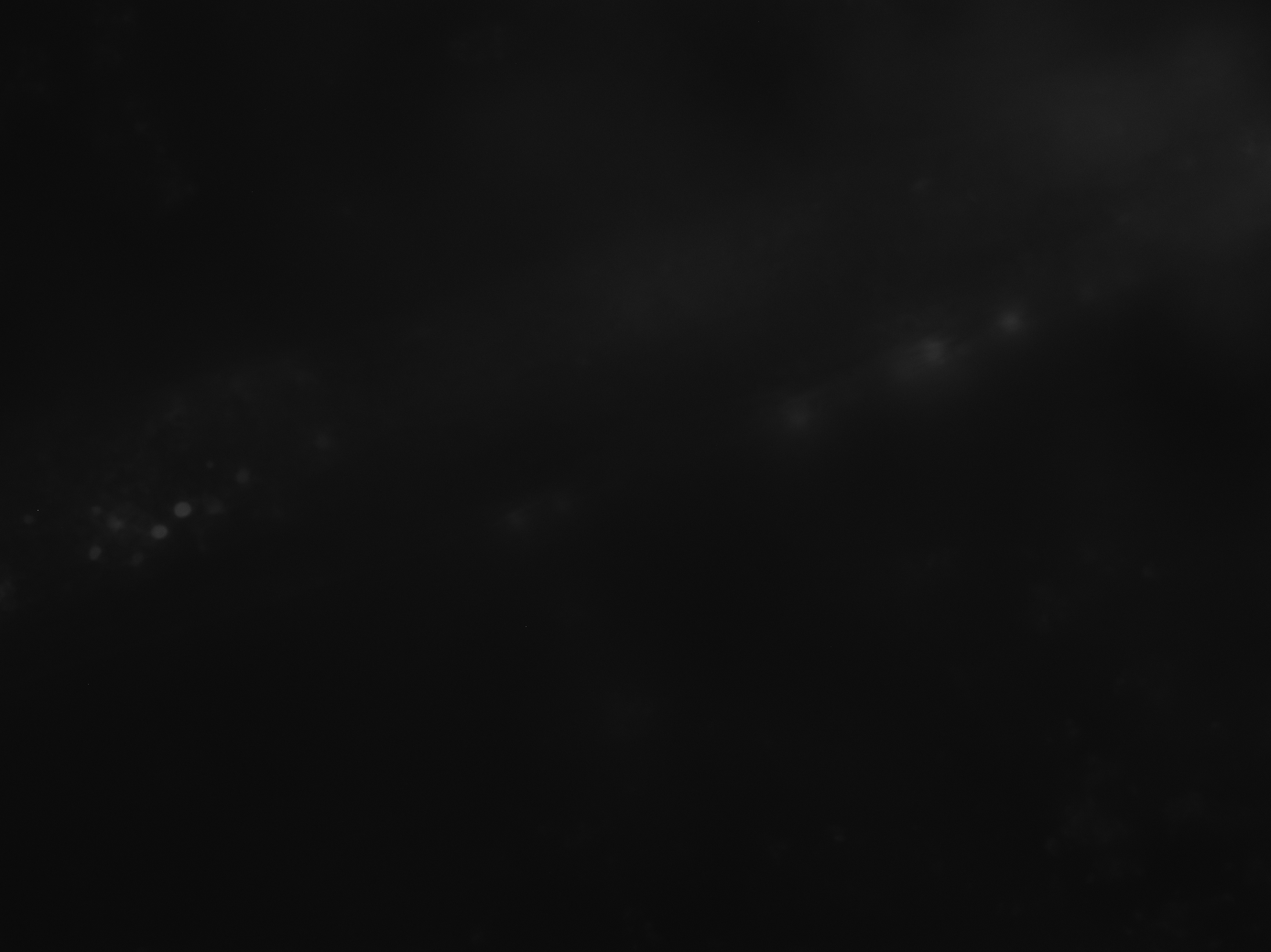

Supplement: Supplementary file 4 — Source data Fig. 3 [file 44319_2025_493_MOESM4_ESM.zip › Figure3/Fig3A/Experiment-63_VC_downstreamdeletion.tif_files/Experiment-63good_z0c1x0-1388y0-1040.tif]

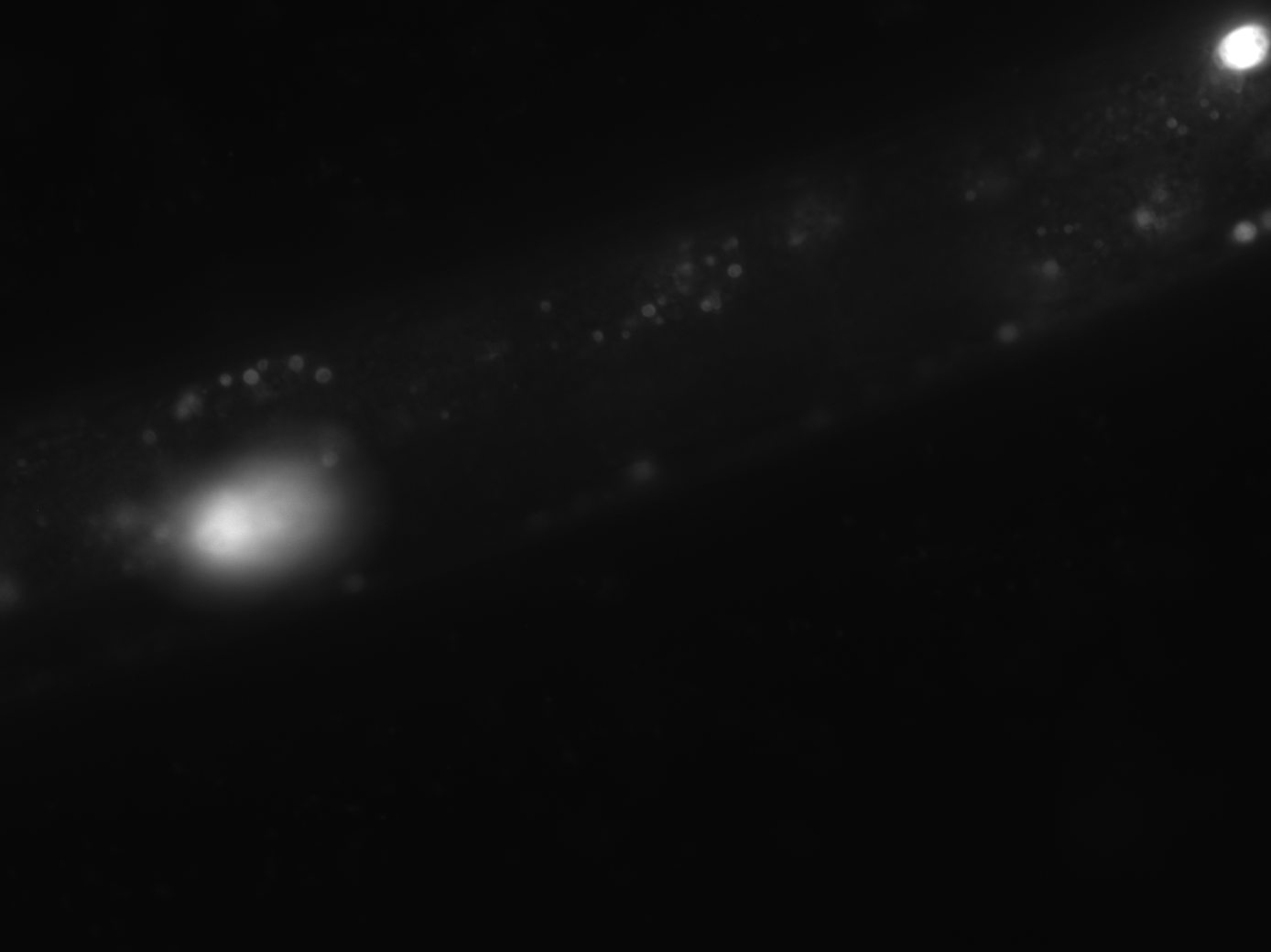

Supplement: Supplementary file 4 — Source data Fig. 3 [file 44319_2025_493_MOESM4_ESM.zip › Figure3/Fig3A/Experiment-63_VC_downstreamdeletion.tif_files/Experiment-63good_z8c0x0-1388y0-1040.tif]

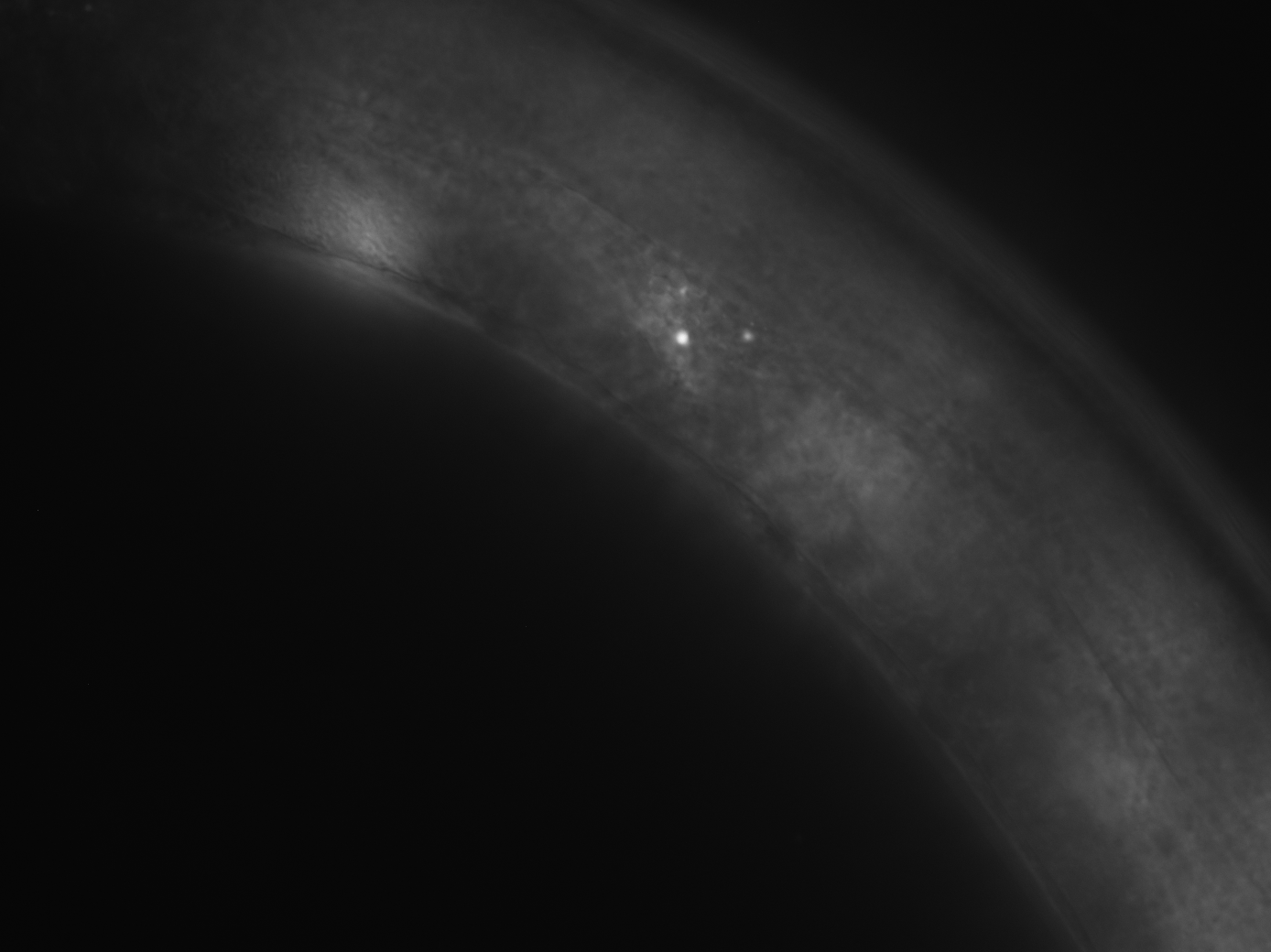

Supplement: Supplementary file 4 — Source data Fig. 3 [file 44319_2025_493_MOESM4_ESM.zip › Figure3/Fig3A/Experiment-14_VC_upstreamdeletion.tif_files/Experiment-14_z12c0x0-1388y0-1040.tif]

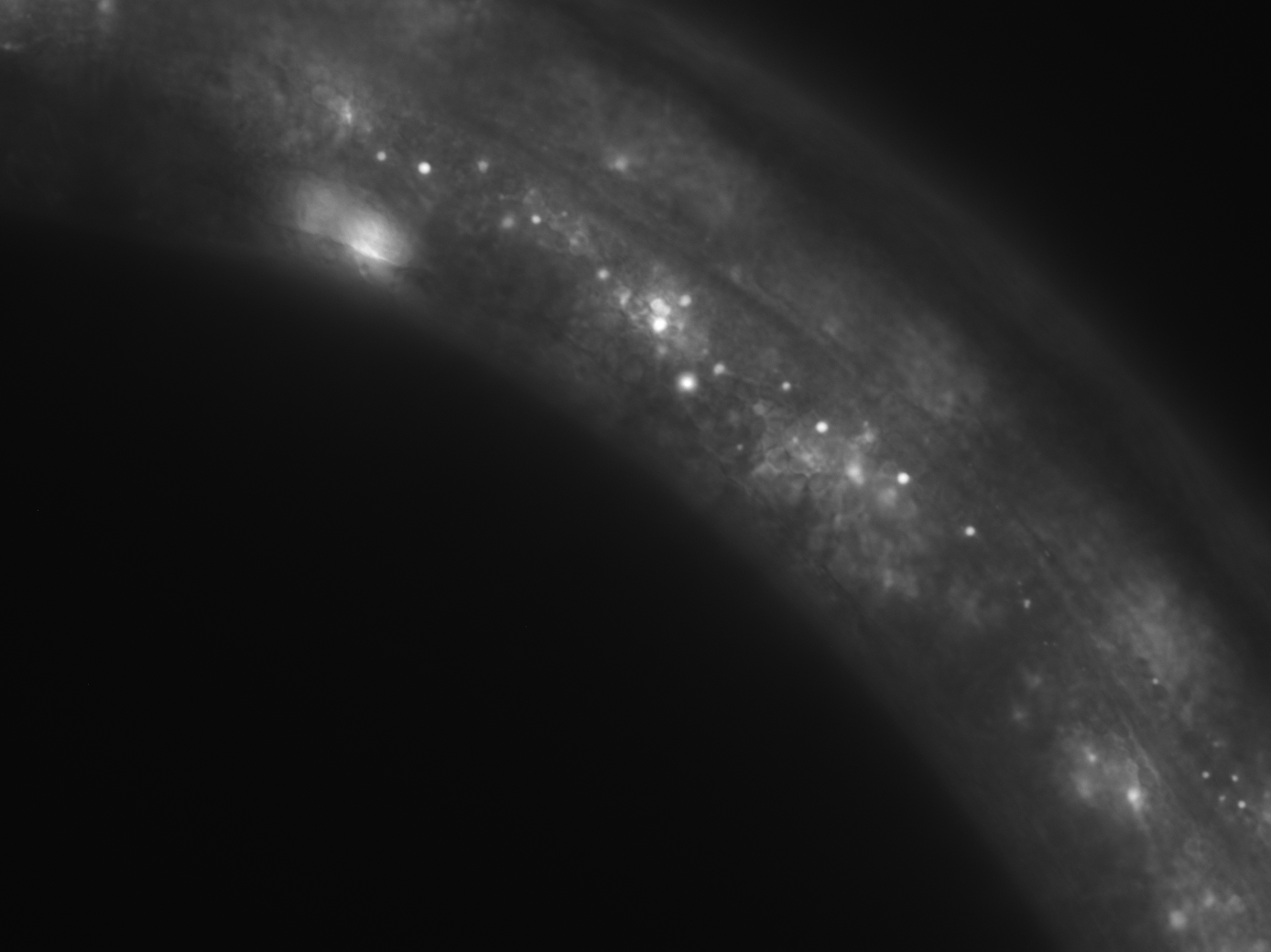

Supplement: Supplementary file 4 — Source data Fig. 3 [file 44319_2025_493_MOESM4_ESM.zip › Figure3/Fig3A/Experiment-14_VC_upstreamdeletion.tif_files/Experiment-14_z18c0x0-1388y0-1040.tif]

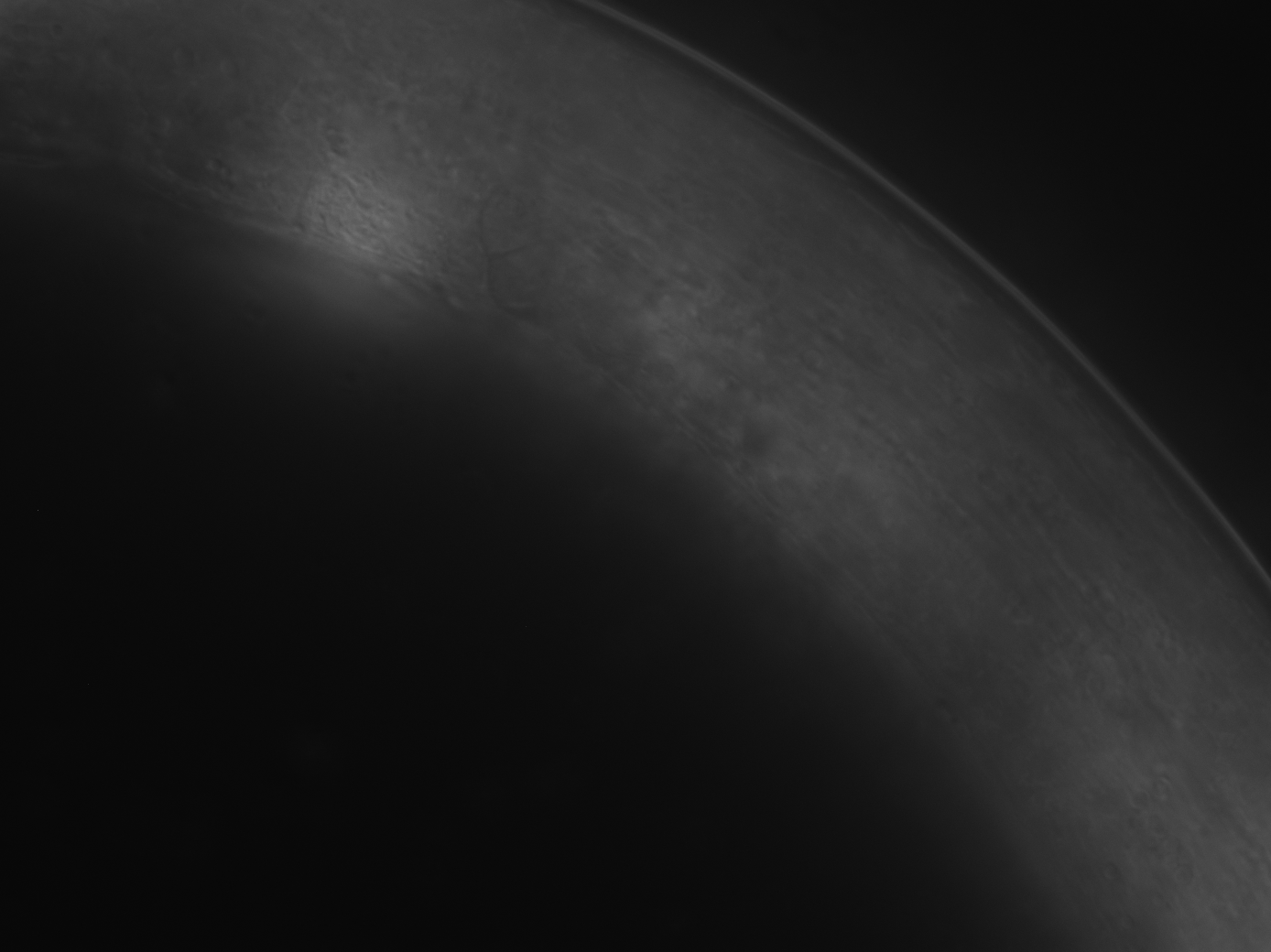

Supplement: Supplementary file 4 — Source data Fig. 3 [file 44319_2025_493_MOESM4_ESM.zip › Figure3/Fig3A/Experiment-14_VC_upstreamdeletion.tif_files/Experiment-14_z6c0x0-1388y0-1040.tif]

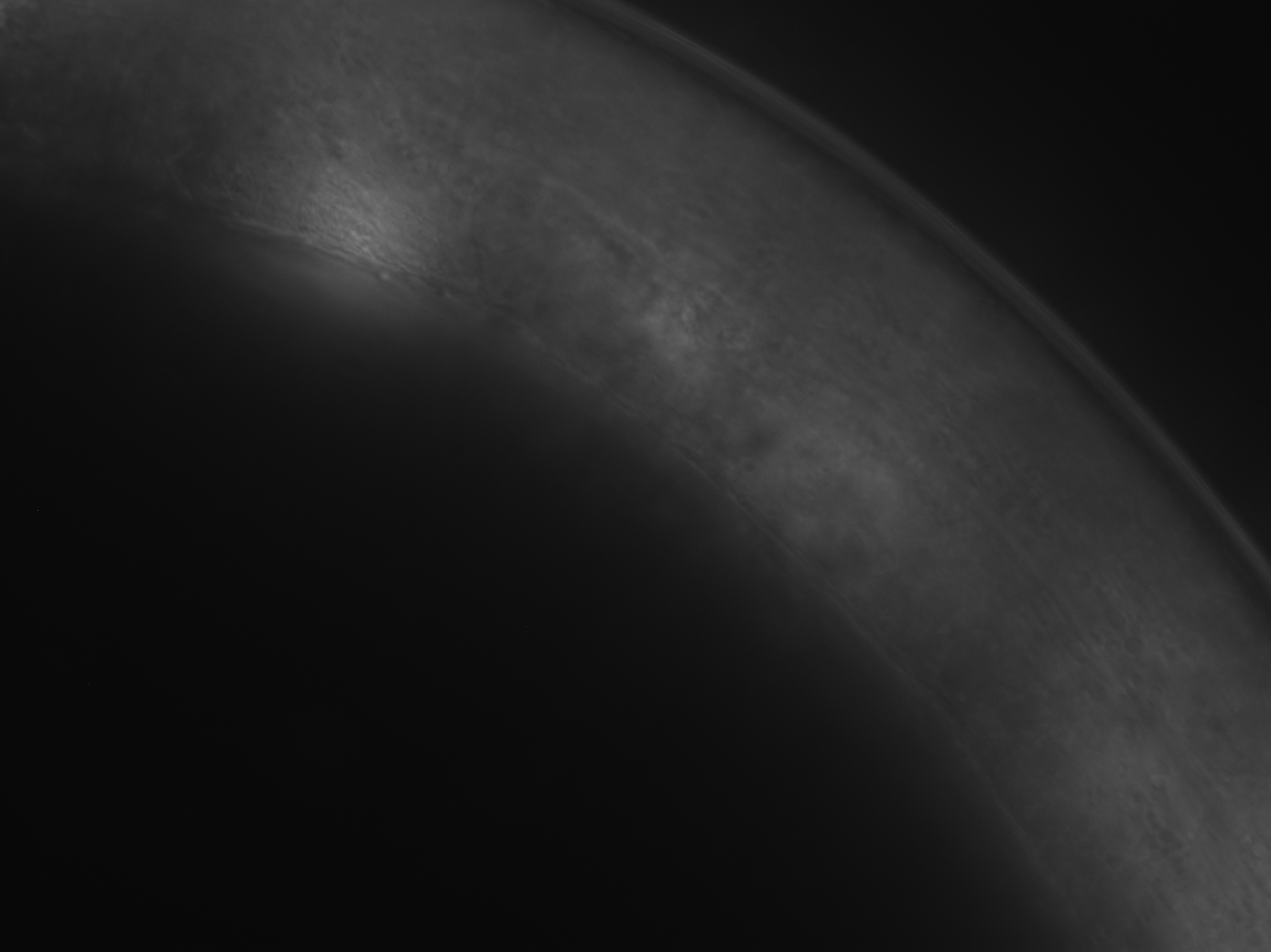

Supplement: Supplementary file 4 — Source data Fig. 3 [file 44319_2025_493_MOESM4_ESM.zip › Figure3/Fig3A/Experiment-14_VC_upstreamdeletion.tif_files/Experiment-14_z8c0x0-1388y0-1040.tif]

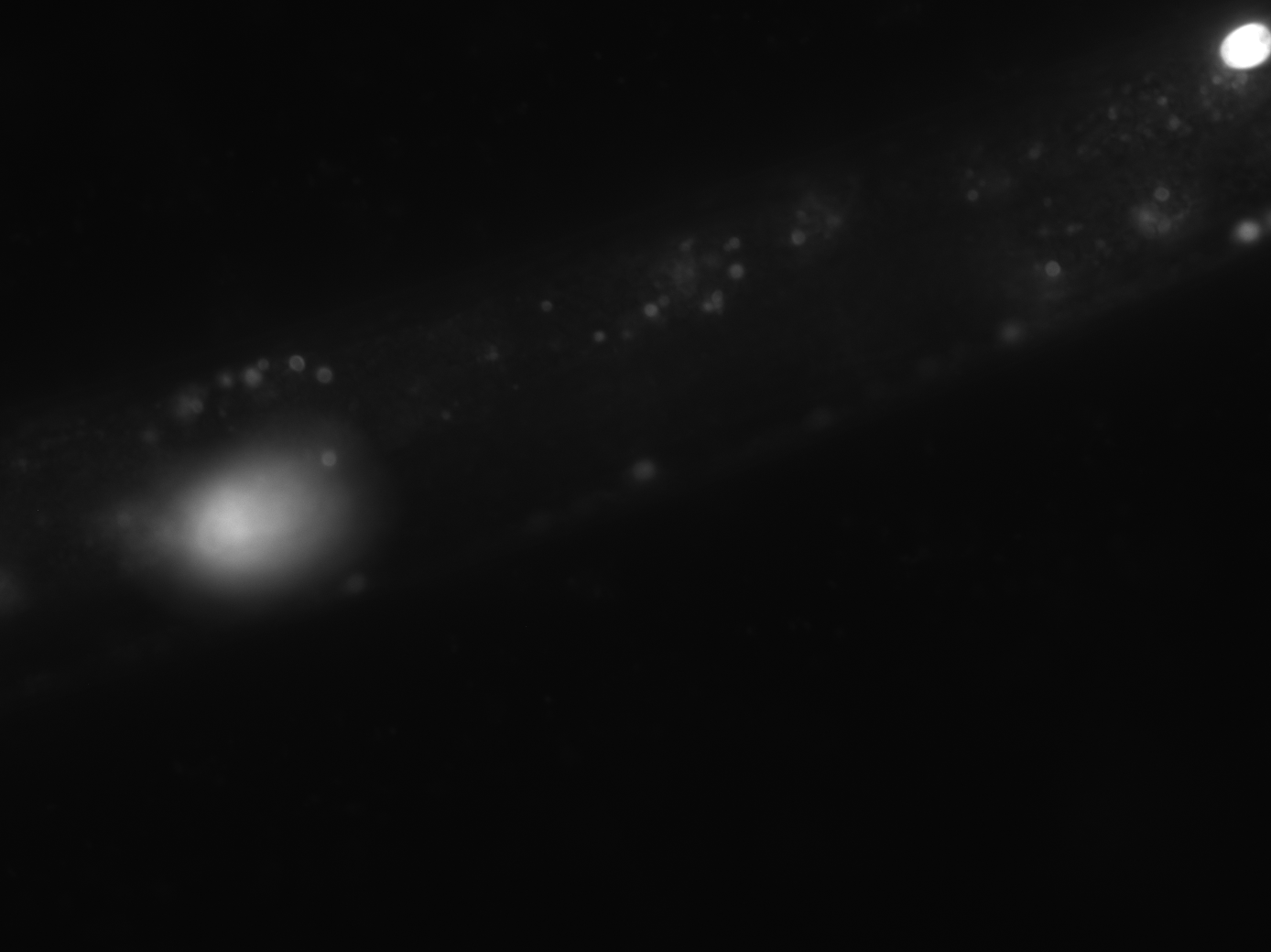

Supplement: Supplementary file 4 — Source data Fig. 3 [file 44319_2025_493_MOESM4_ESM.zip › Figure3/Fig3A/Experiment-63_VC_downstreamdeletion.tif_files/Experiment-63good_z9c0x0-1388y0-1040.tif]

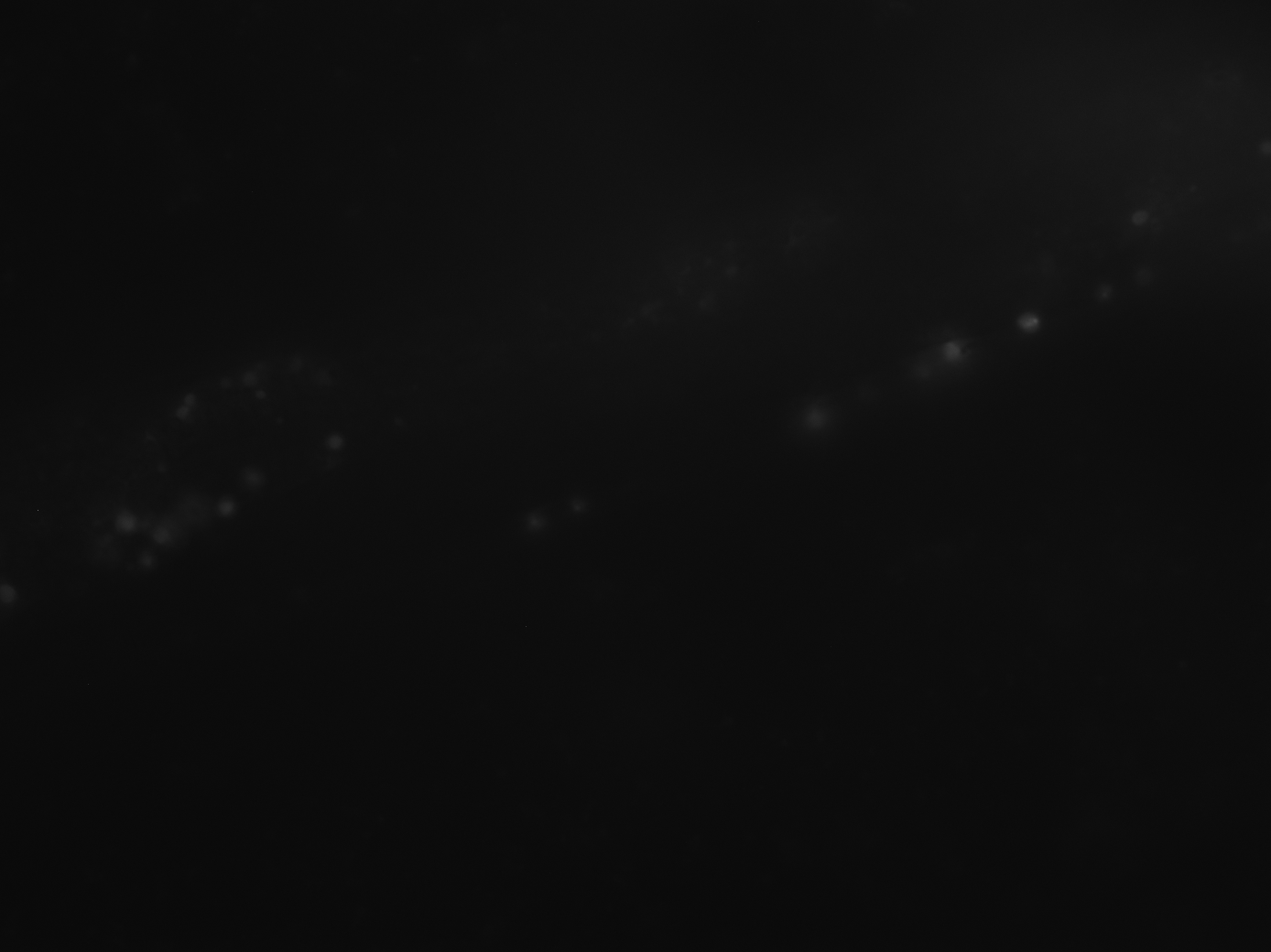

Supplement: Supplementary file 4 — Source data Fig. 3 [file 44319_2025_493_MOESM4_ESM.zip › Figure3/Fig3A/Experiment-63_VC_downstreamdeletion.tif_files/Experiment-63good_z5c1x0-1388y0-1040.tif]

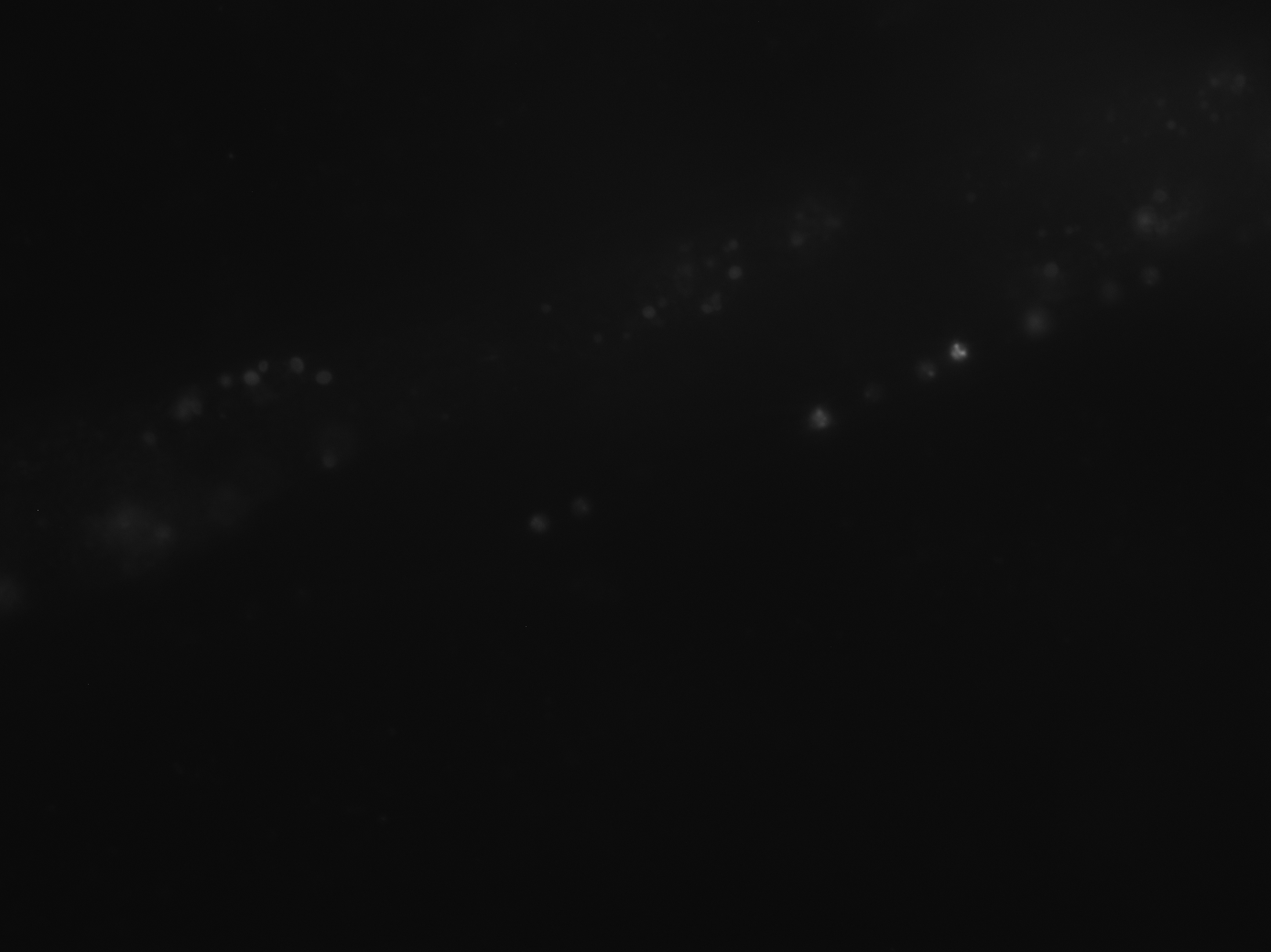

Supplement: Supplementary file 4 — Source data Fig. 3 [file 44319_2025_493_MOESM4_ESM.zip › Figure3/Fig3A/Experiment-63_VC_downstreamdeletion.tif_files/Experiment-63good_z8c1x0-1388y0-1040.tif]

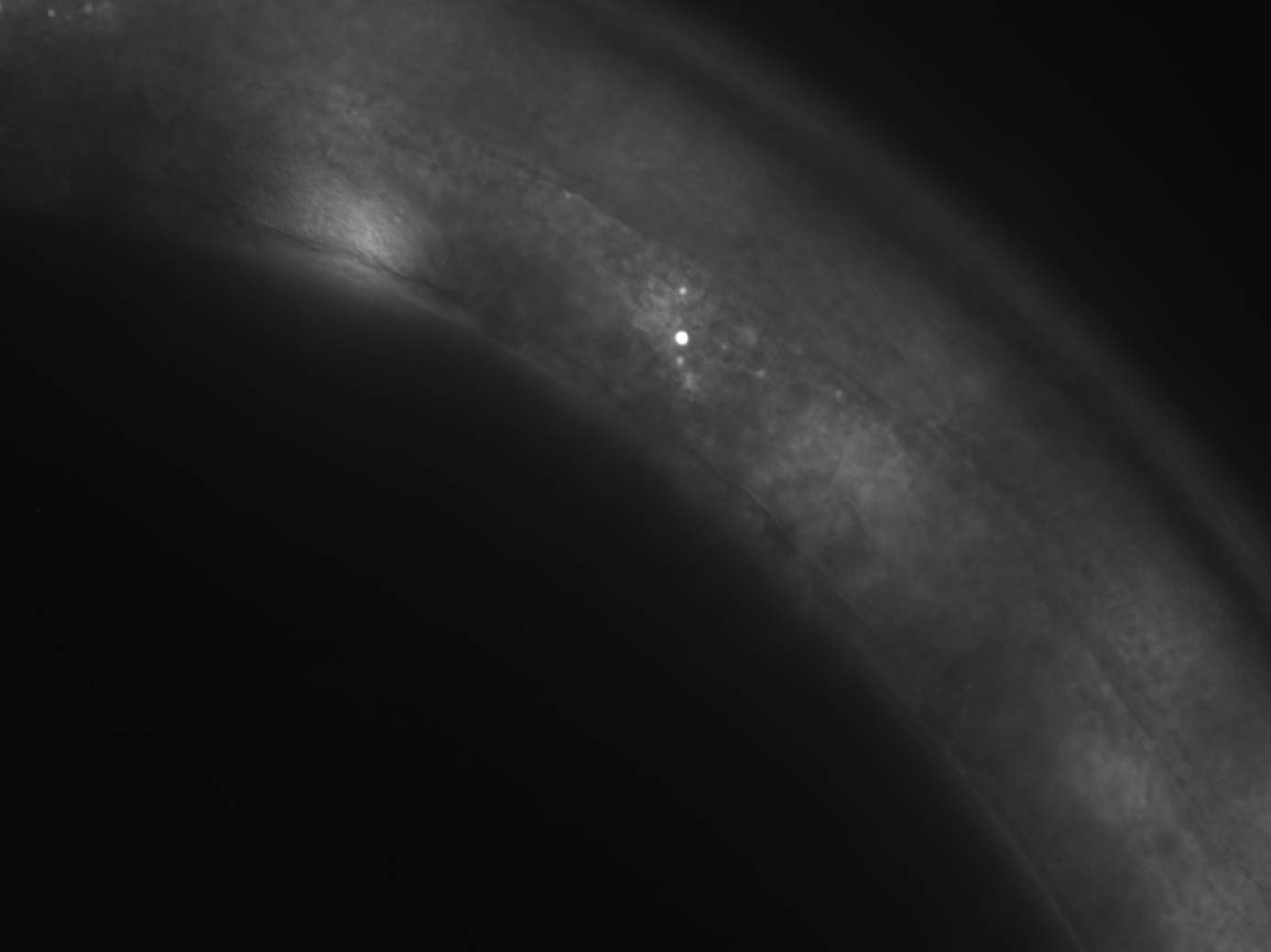

Supplement: Supplementary file 4 — Source data Fig. 3 [file 44319_2025_493_MOESM4_ESM.zip › Figure3/Fig3A/Experiment-14_VC_upstreamdeletion.tif_files/Experiment-14_z13c0x0-1388y0-1040.tif]

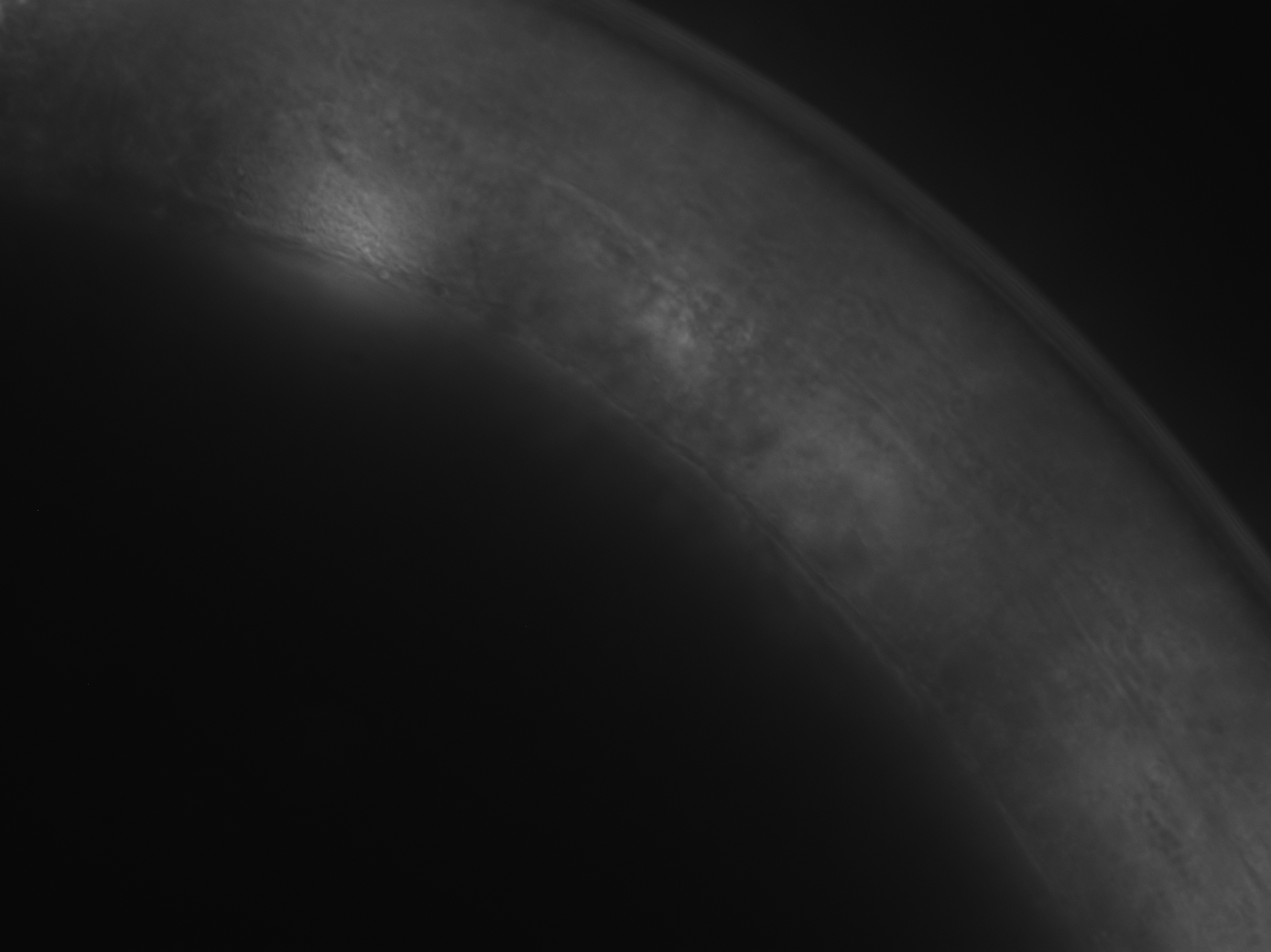

Supplement: Supplementary file 4 — Source data Fig. 3 [file 44319_2025_493_MOESM4_ESM.zip › Figure3/Fig3A/Experiment-14_VC_upstreamdeletion.tif_files/Experiment-14_z9c0x0-1388y0-1040.tif]

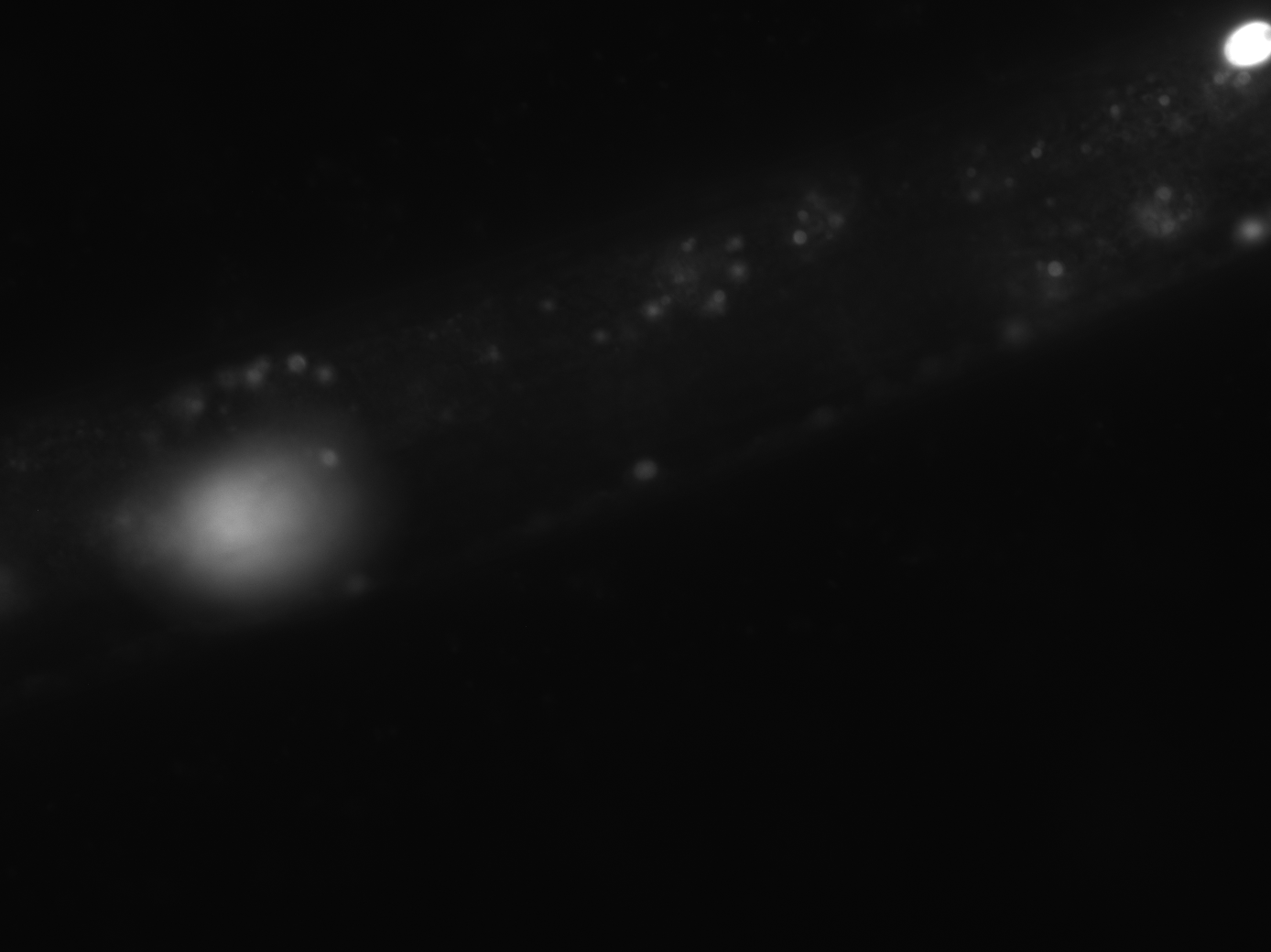

Supplement: Supplementary file 4 — Source data Fig. 3 [file 44319_2025_493_MOESM4_ESM.zip › Figure3/Fig3A/Experiment-63_VC_downstreamdeletion.tif_files/Experiment-63good_z10c0x0-1388y0-1040.tif]

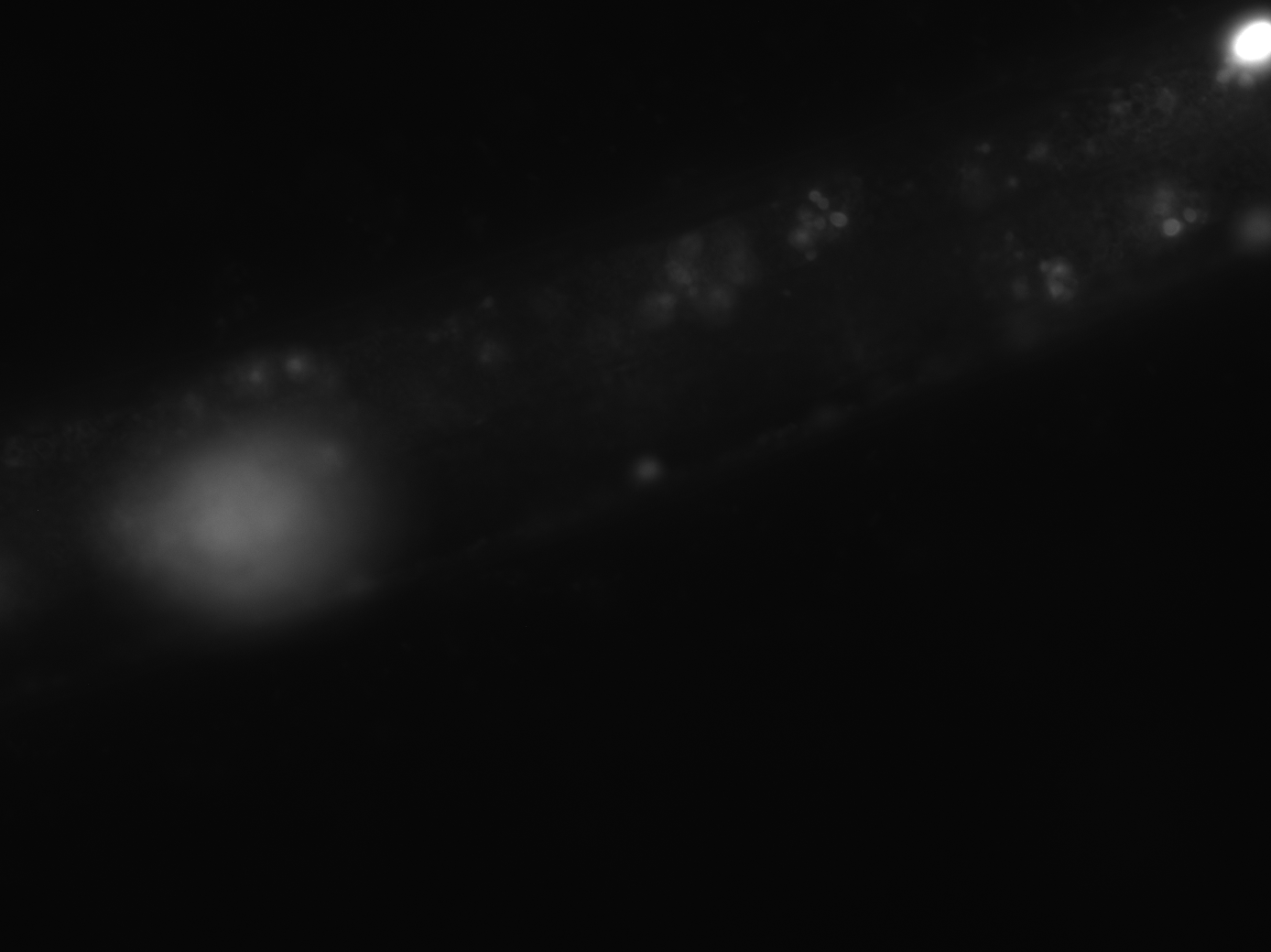

Supplement: Supplementary file 4 — Source data Fig. 3 [file 44319_2025_493_MOESM4_ESM.zip › Figure3/Fig3A/Experiment-63_VC_downstreamdeletion.tif_files/Experiment-63good_z12c0x0-1388y0-1040.tif]

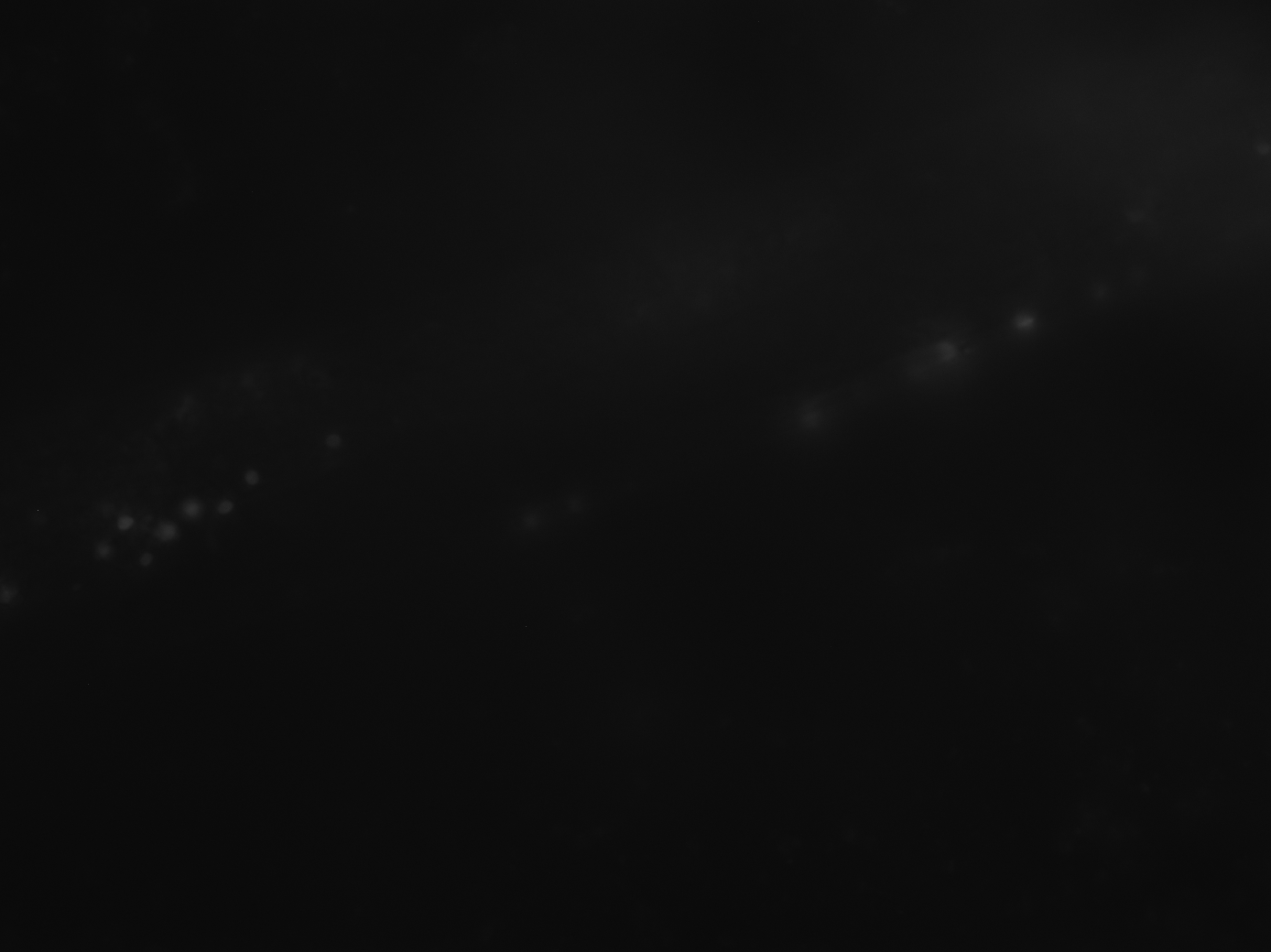

Supplement: Supplementary file 4 — Source data Fig. 3 [file 44319_2025_493_MOESM4_ESM.zip › Figure3/Fig3A/Experiment-63_VC_downstreamdeletion.tif_files/Experiment-63good_z3c1x0-1388y0-1040.tif]

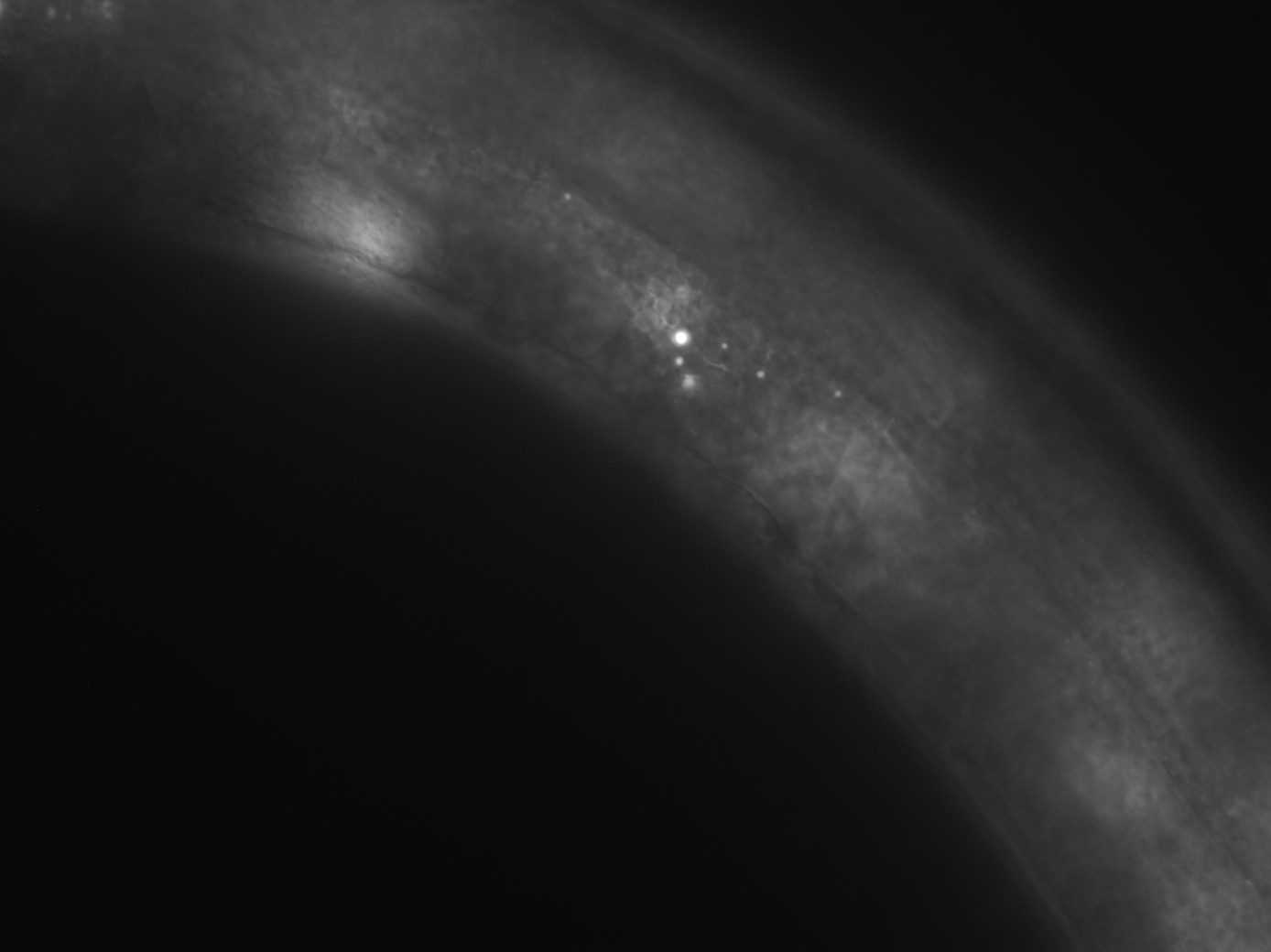

Supplement: Supplementary file 4 — Source data Fig. 3 [file 44319_2025_493_MOESM4_ESM.zip › Figure3/Fig3A/Experiment-14_VC_upstreamdeletion.tif_files/Experiment-14_z14c0x0-1388y0-1040.tif]

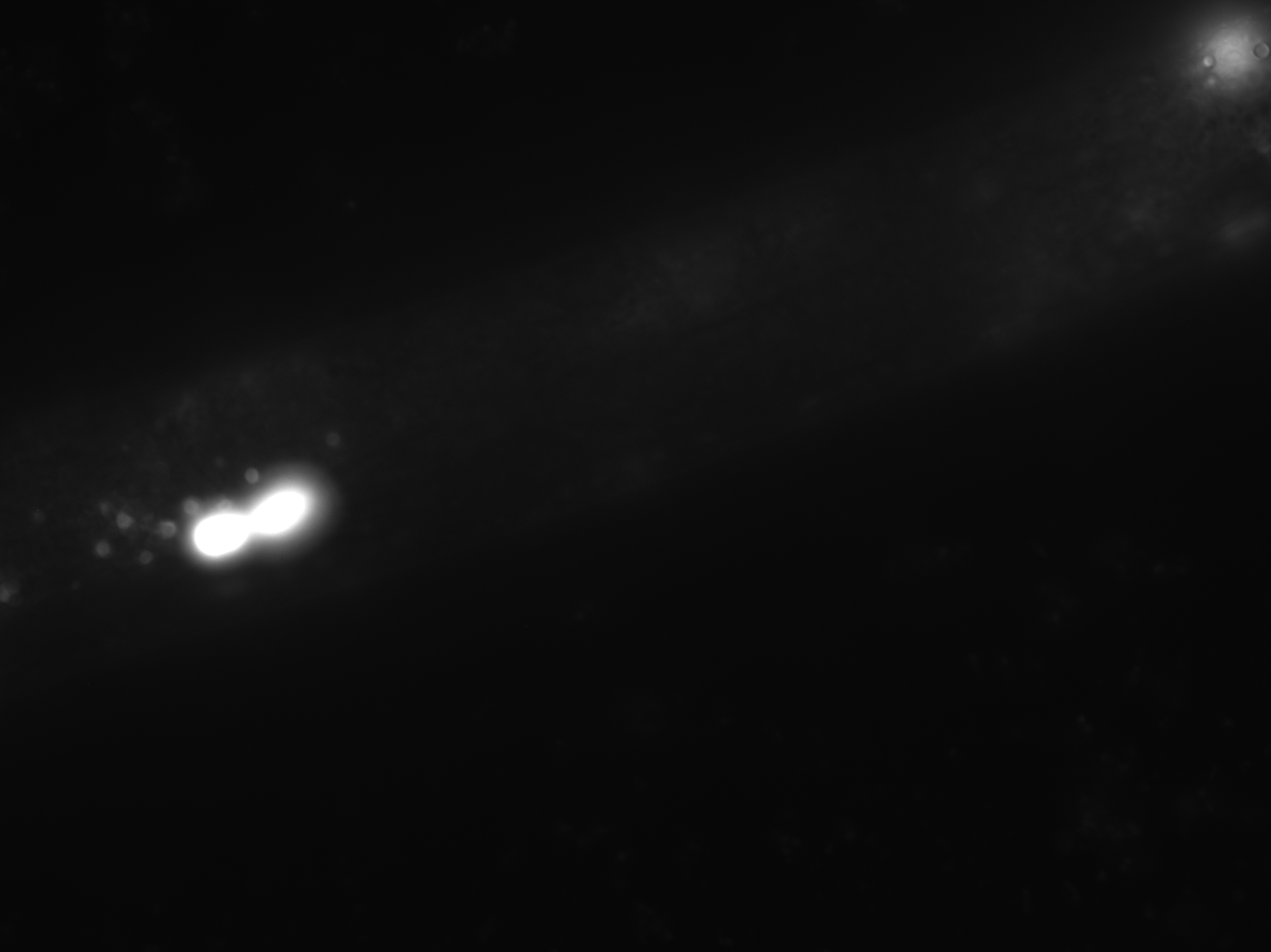

Supplement: Supplementary file 4 — Source data Fig. 3 [file 44319_2025_493_MOESM4_ESM.zip › Figure3/Fig3A/Experiment-63_VC_downstreamdeletion.tif_files/Experiment-63good_z3c0x0-1388y0-1040.tif]

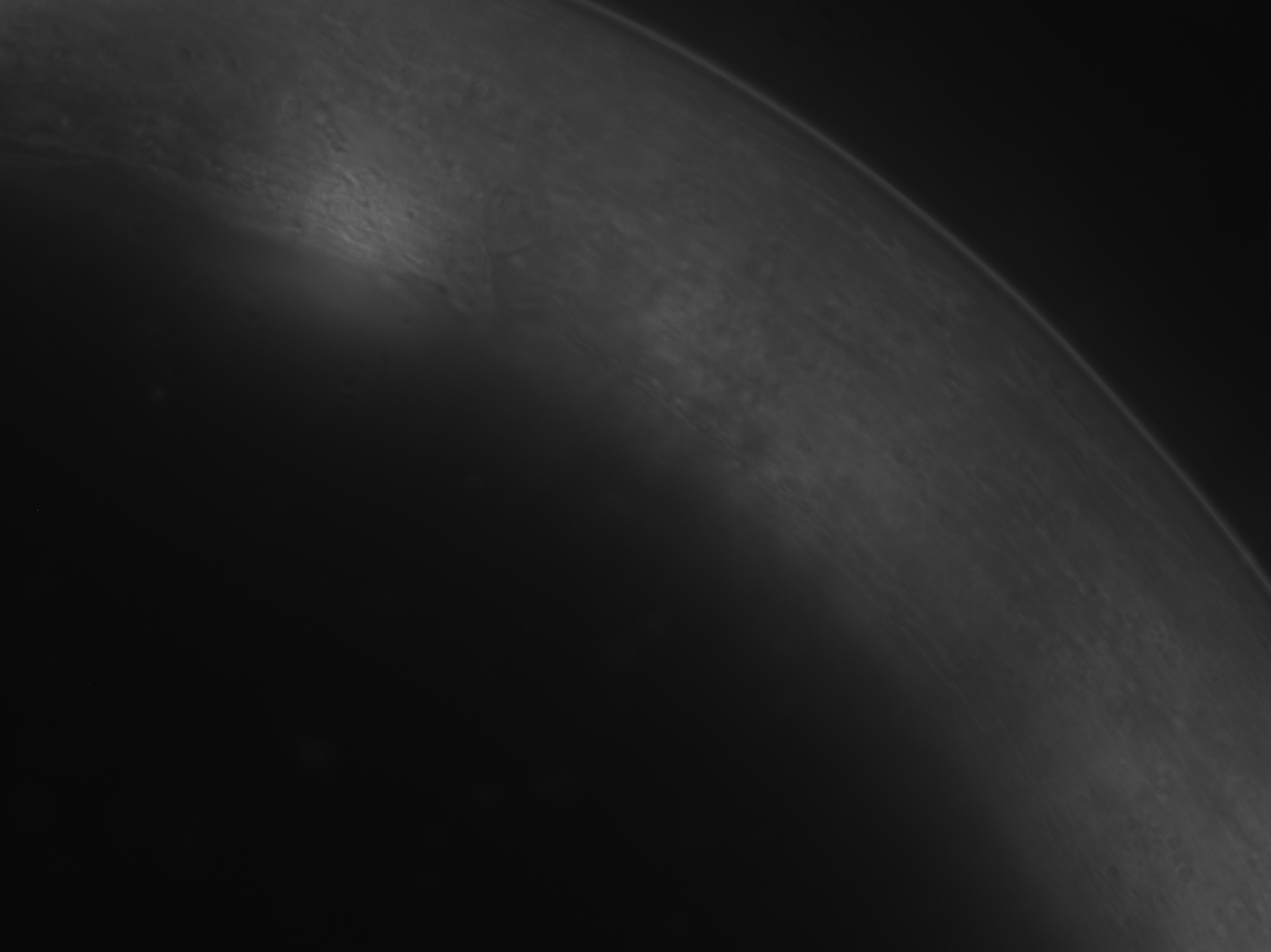

Supplement: Supplementary file 4 — Source data Fig. 3 [file 44319_2025_493_MOESM4_ESM.zip › Figure3/Fig3A/Experiment-14_VC_upstreamdeletion.tif_files/Experiment-14_z5c0x0-1388y0-1040.tif]

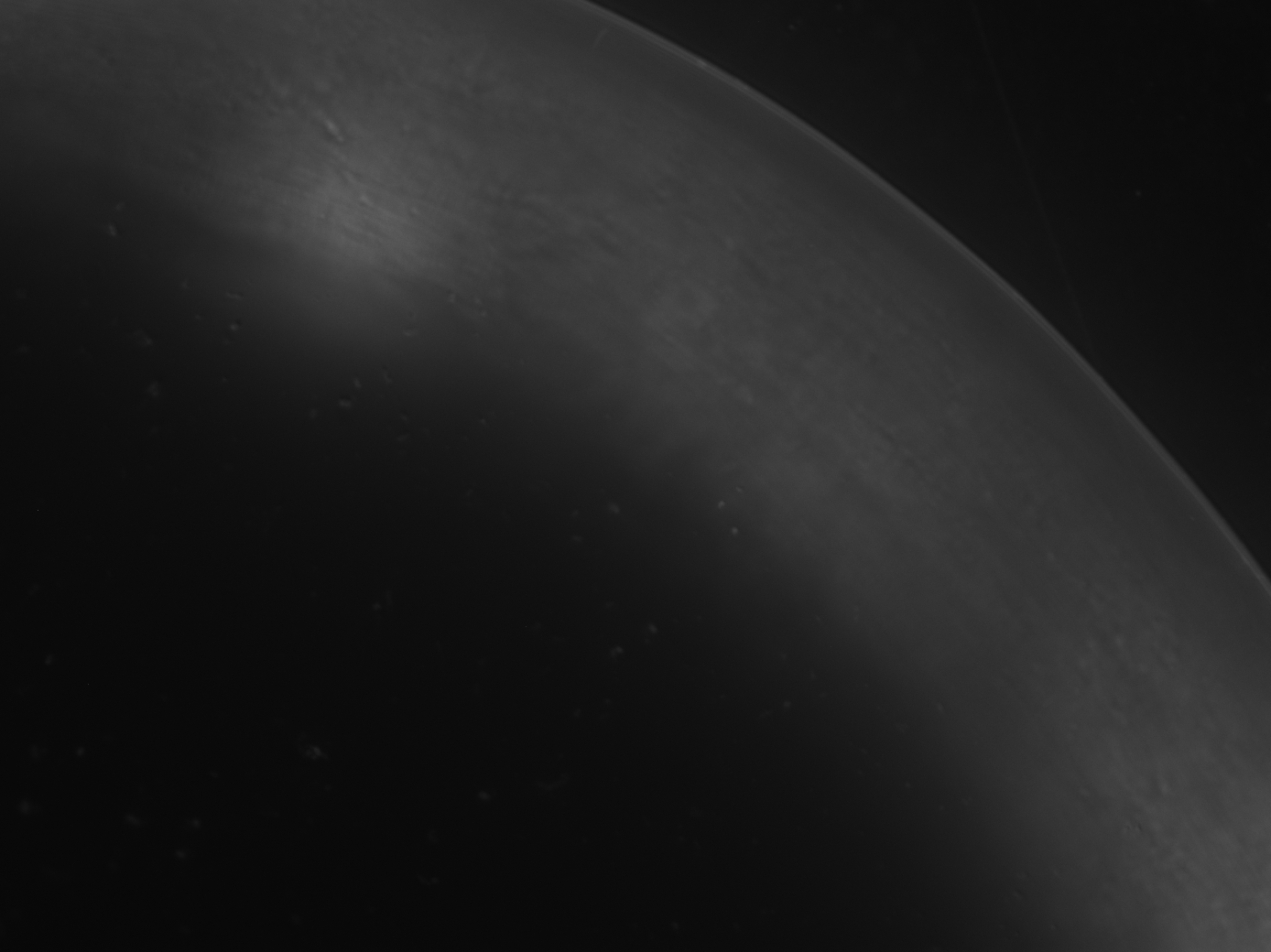

Supplement: Supplementary file 4 — Source data Fig. 3 [file 44319_2025_493_MOESM4_ESM.zip › Figure3/Fig3A/Experiment-14_VC_upstreamdeletion.tif_files/Experiment-14_z2c0x0-1388y0-1040.tif]

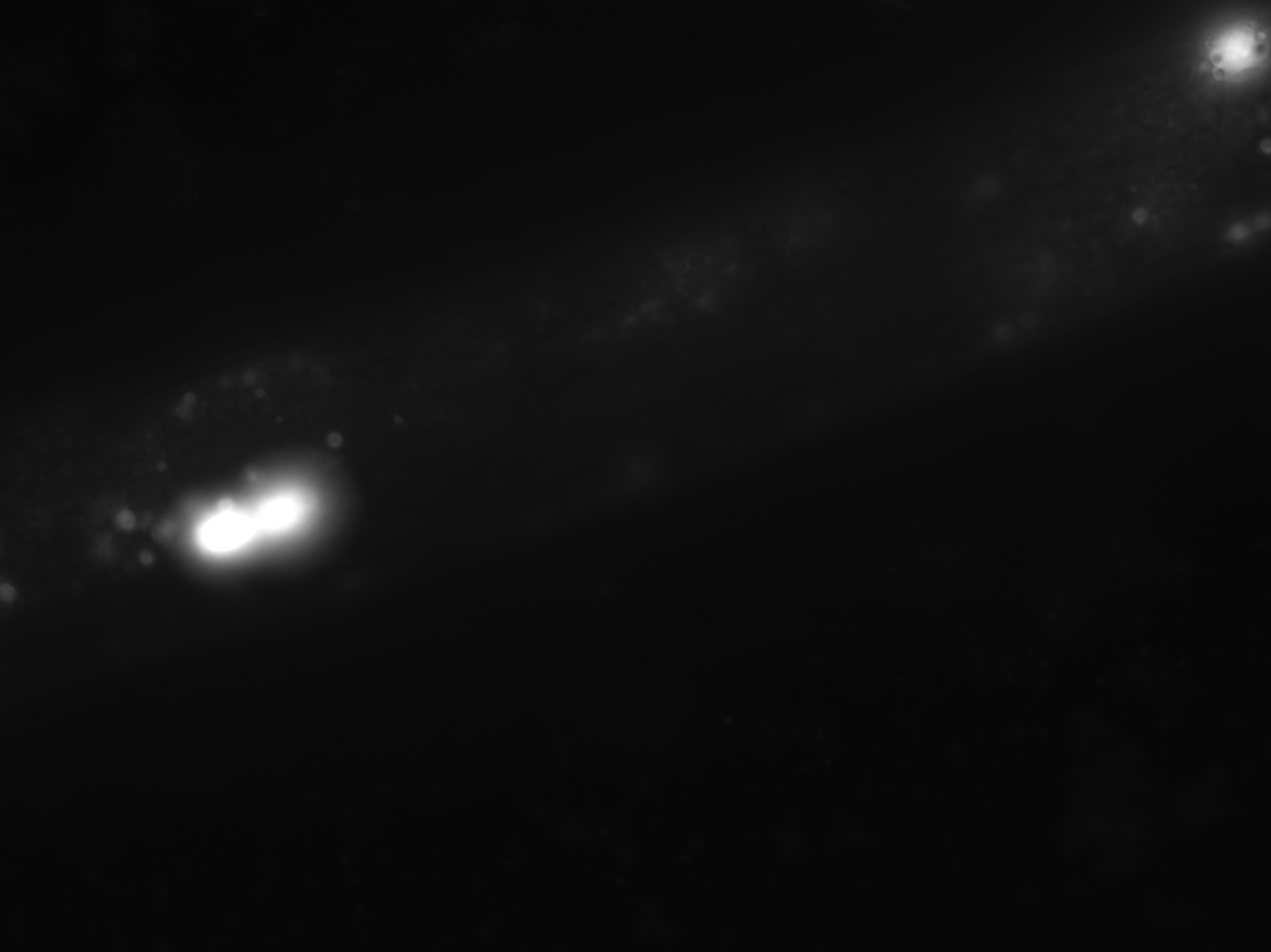

Supplement: Supplementary file 4 — Source data Fig. 3 [file 44319_2025_493_MOESM4_ESM.zip › Figure3/Fig3A/Experiment-63_VC_downstreamdeletion.tif_files/Experiment-63good_z5c0x0-1388y0-1040.tif]

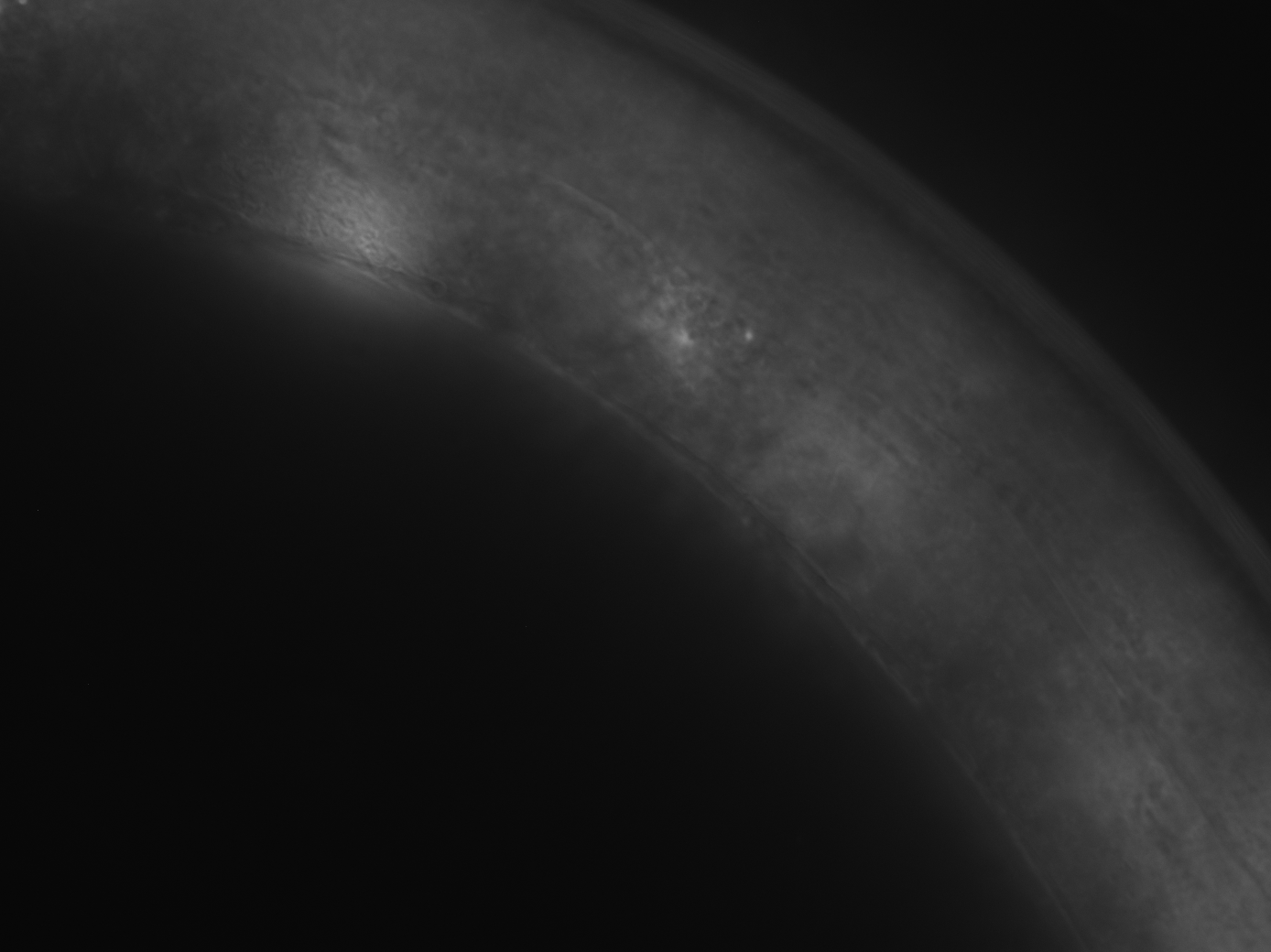

Supplement: Supplementary file 4 — Source data Fig. 3 [file 44319_2025_493_MOESM4_ESM.zip › Figure3/Fig3A/Experiment-14_VC_upstreamdeletion.tif_files/Experiment-14_z10c0x0-1388y0-1040.tif]

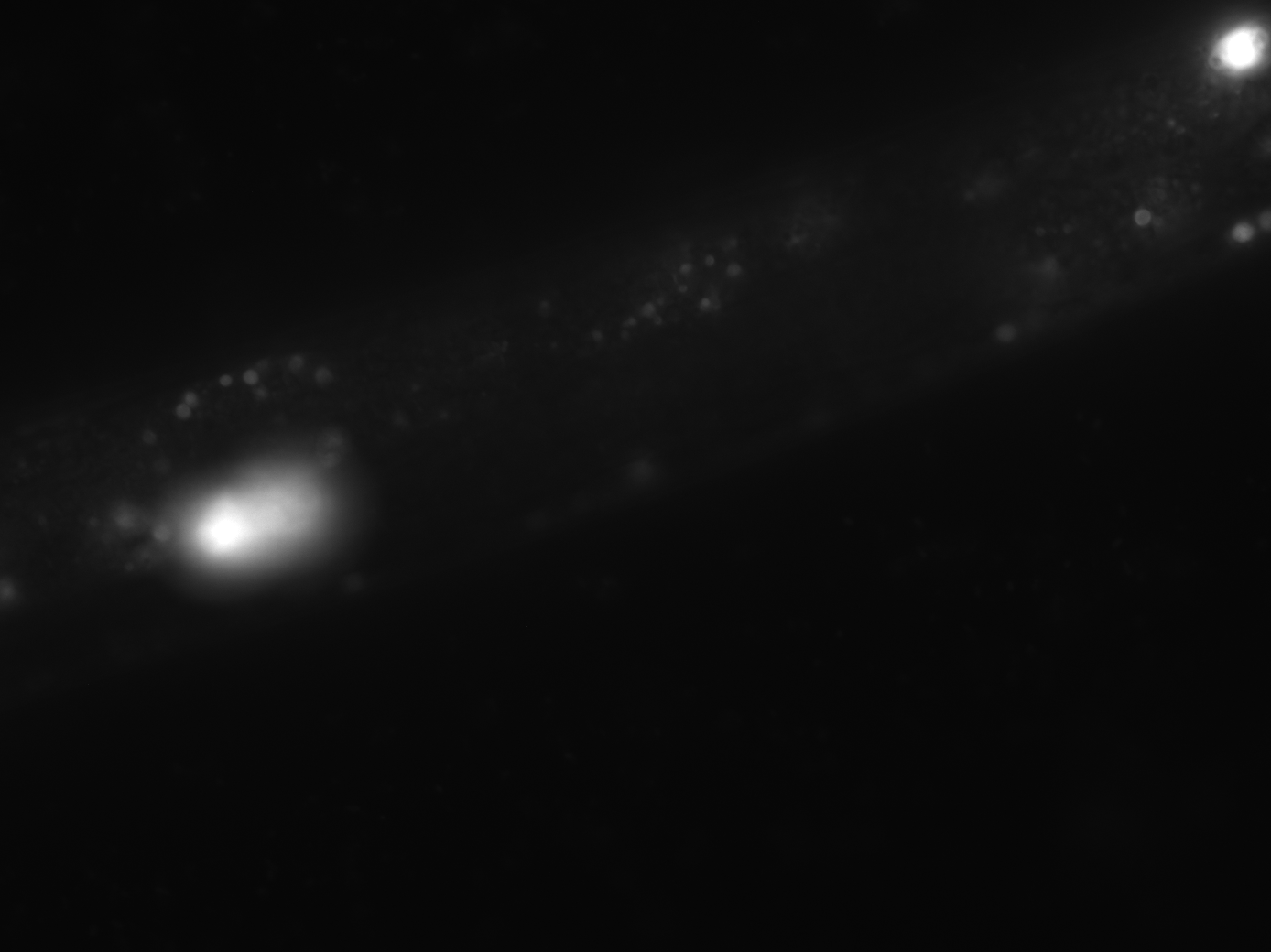

Supplement: Supplementary file 4 — Source data Fig. 3 [file 44319_2025_493_MOESM4_ESM.zip › Figure3/Fig3A/Experiment-63_VC_downstreamdeletion.tif_files/Experiment-63good_z7c0x0-1388y0-1040.tif]

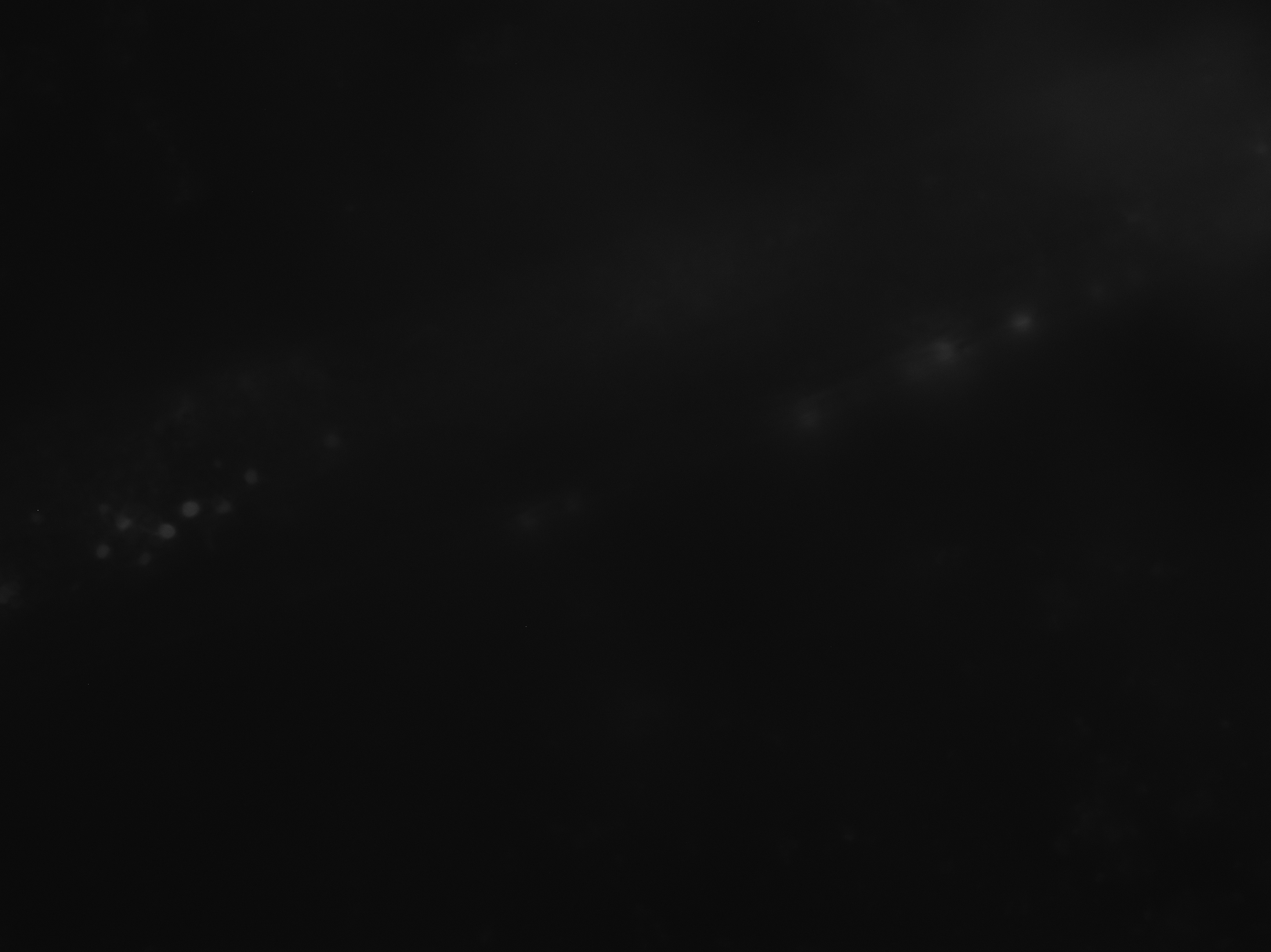

Supplement: Supplementary file 4 — Source data Fig. 3 [file 44319_2025_493_MOESM4_ESM.zip › Figure3/Fig3A/Experiment-63_VC_downstreamdeletion.tif_files/Experiment-63good_z2c1x0-1388y0-1040.tif]

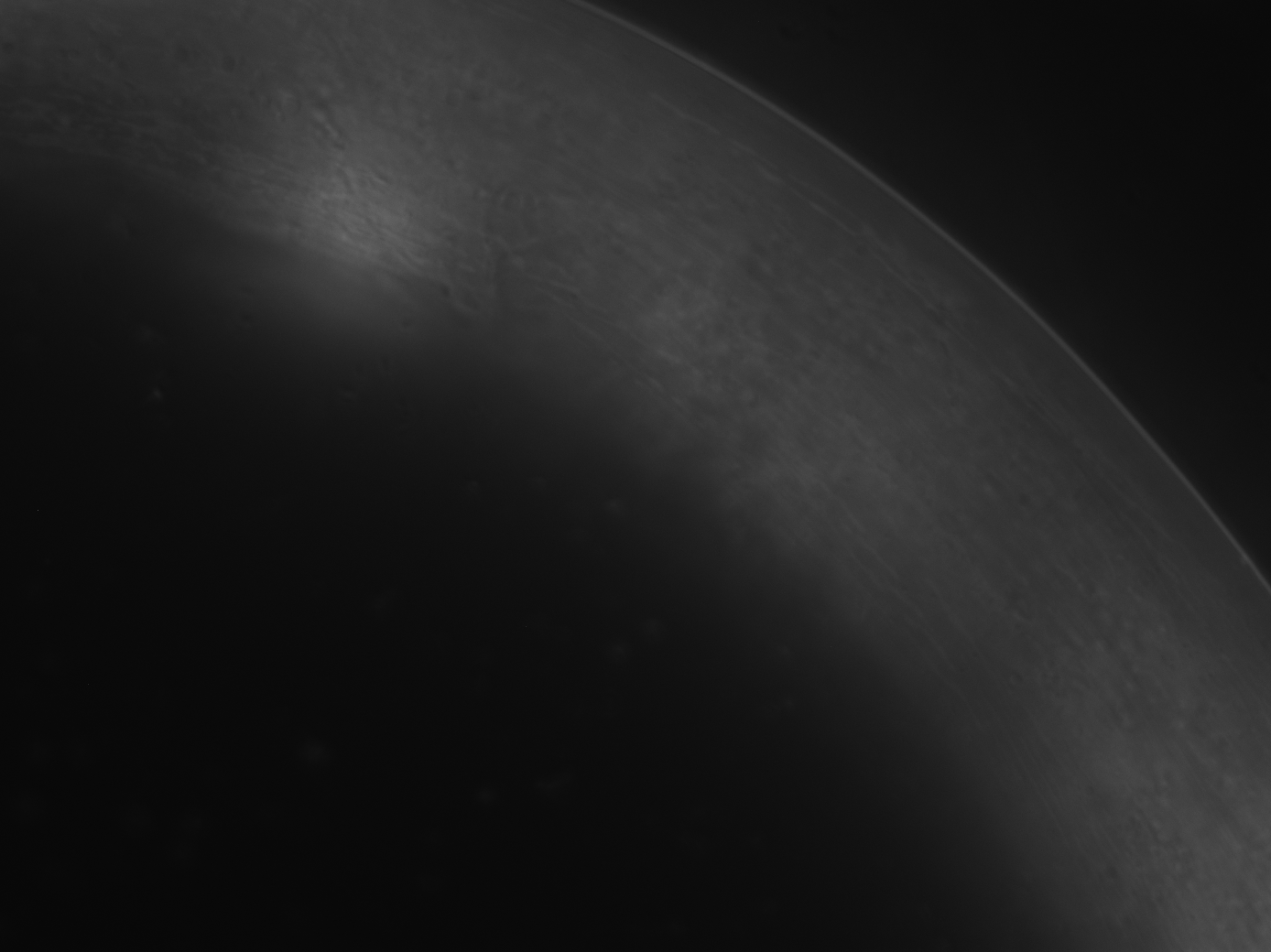

Supplement: Supplementary file 4 — Source data Fig. 3 [file 44319_2025_493_MOESM4_ESM.zip › Figure3/Fig3A/Experiment-14_VC_upstreamdeletion.tif_files/Experiment-14_z4c0x0-1388y0-1040.tif]

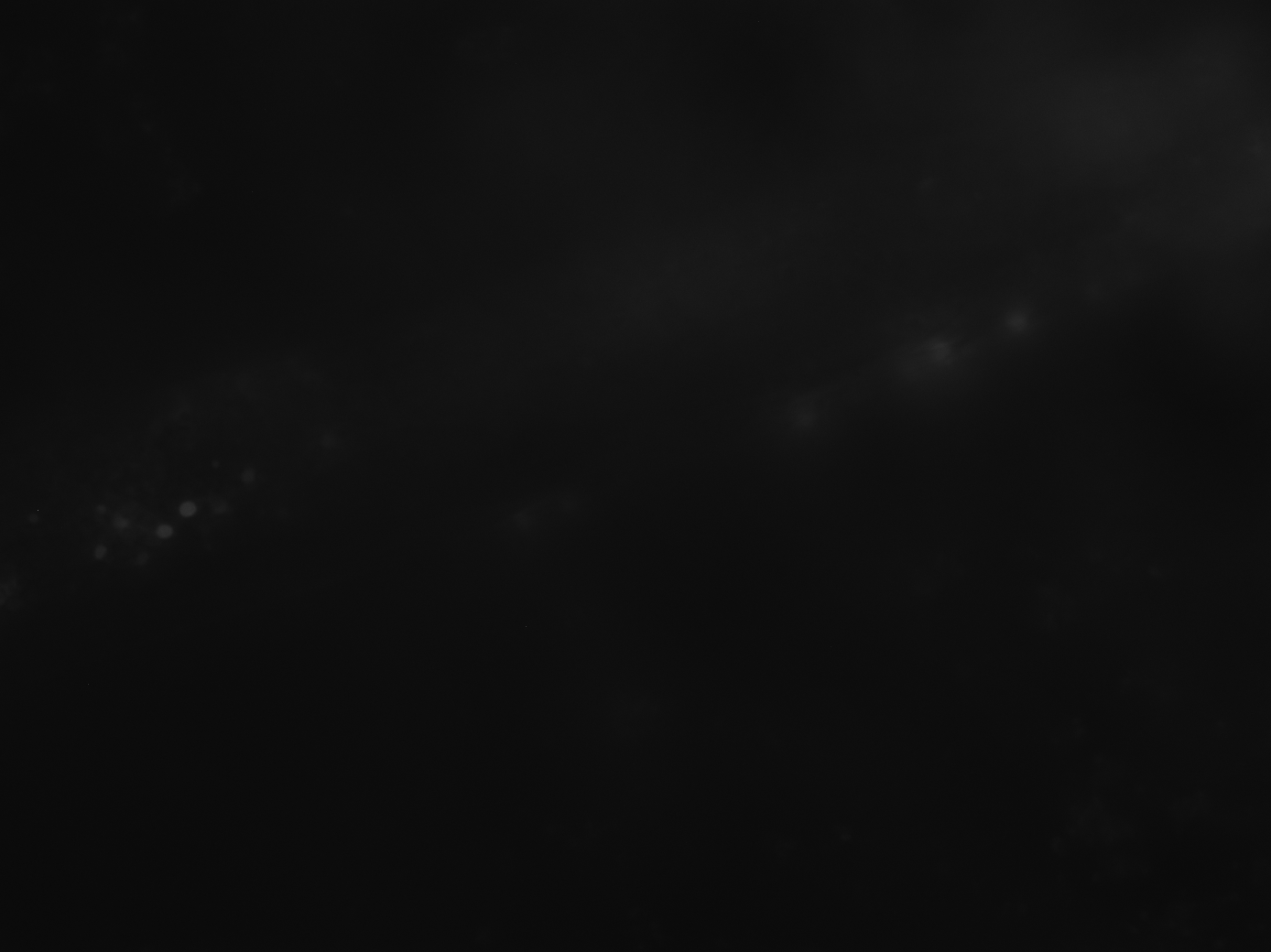

Supplement: Supplementary file 4 — Source data Fig. 3 [file 44319_2025_493_MOESM4_ESM.zip › Figure3/Fig3A/Experiment-63_VC_downstreamdeletion.tif_files/Experiment-63good_z1c1x0-1388y0-1040.tif]

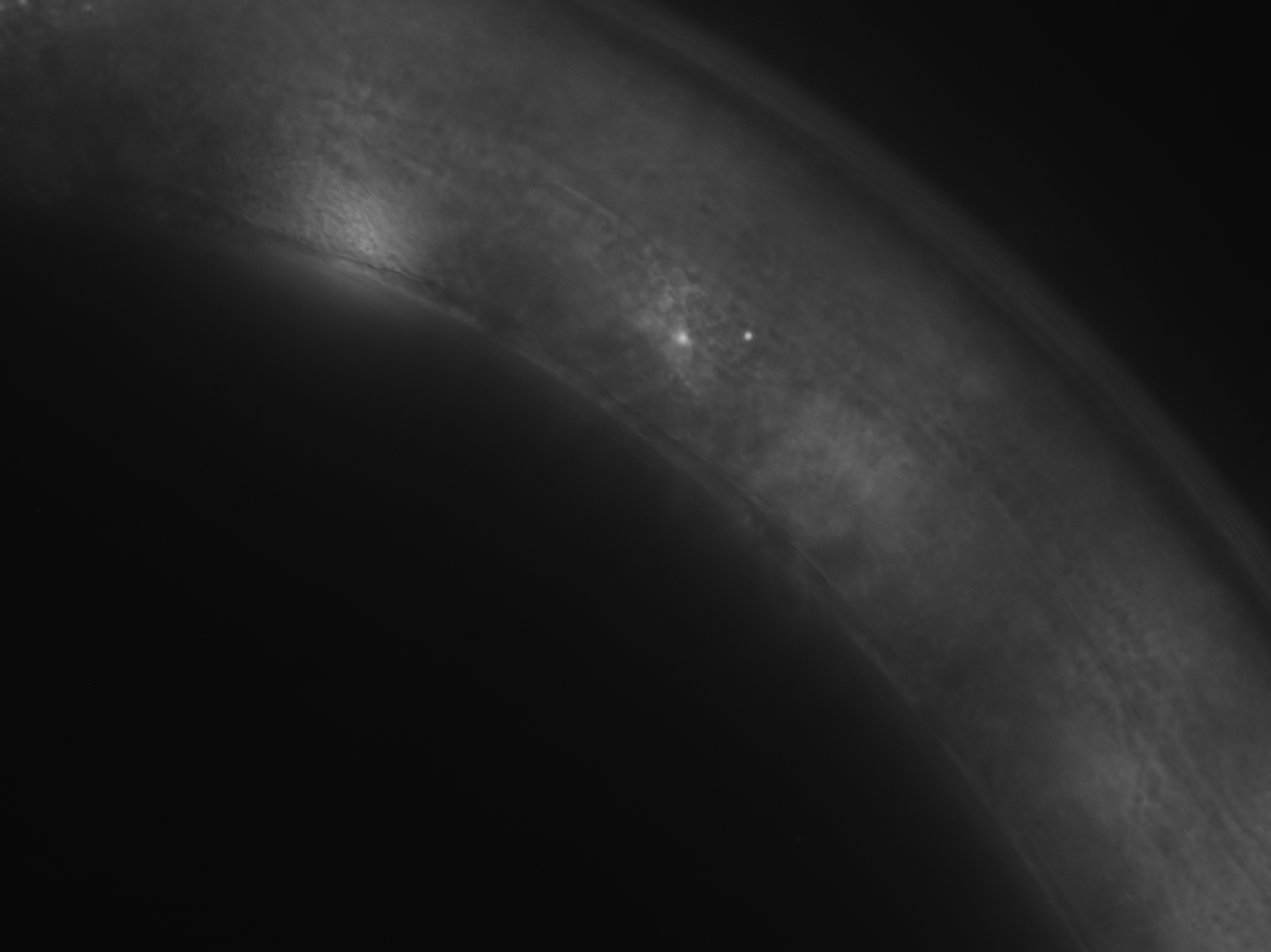

Supplement: Supplementary file 4 — Source data Fig. 3 [file 44319_2025_493_MOESM4_ESM.zip › Figure3/Fig3A/Experiment-14_VC_upstreamdeletion.tif_files/Experiment-14_z11c0x0-1388y0-1040.tif]

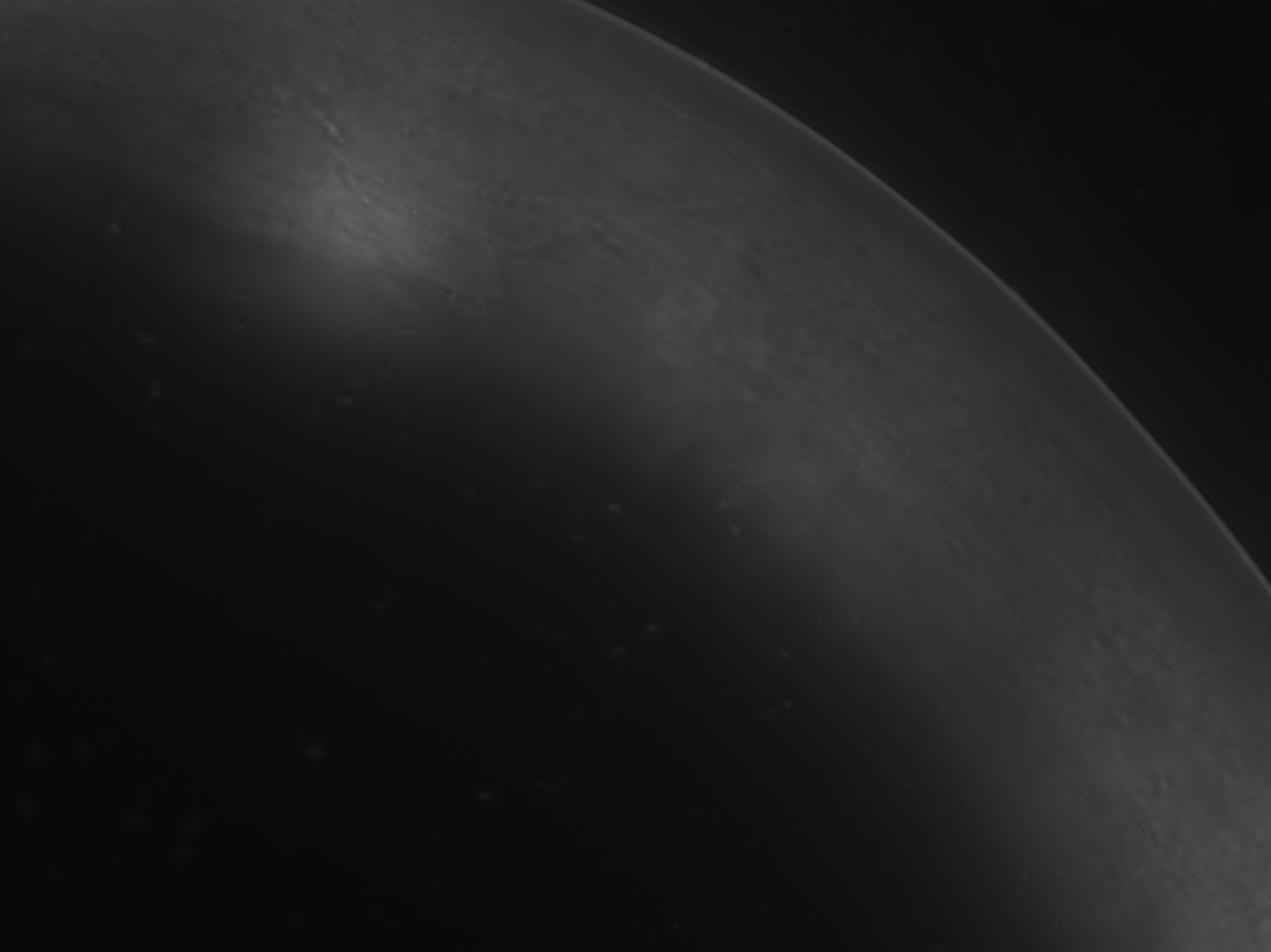

Supplement: Supplementary file 4 — Source data Fig. 3 [file 44319_2025_493_MOESM4_ESM.zip › Figure3/Fig3A/Experiment-14_VC_upstreamdeletion.tif_files/Experiment-14_z3c0x0-1388y0-1040.tif]

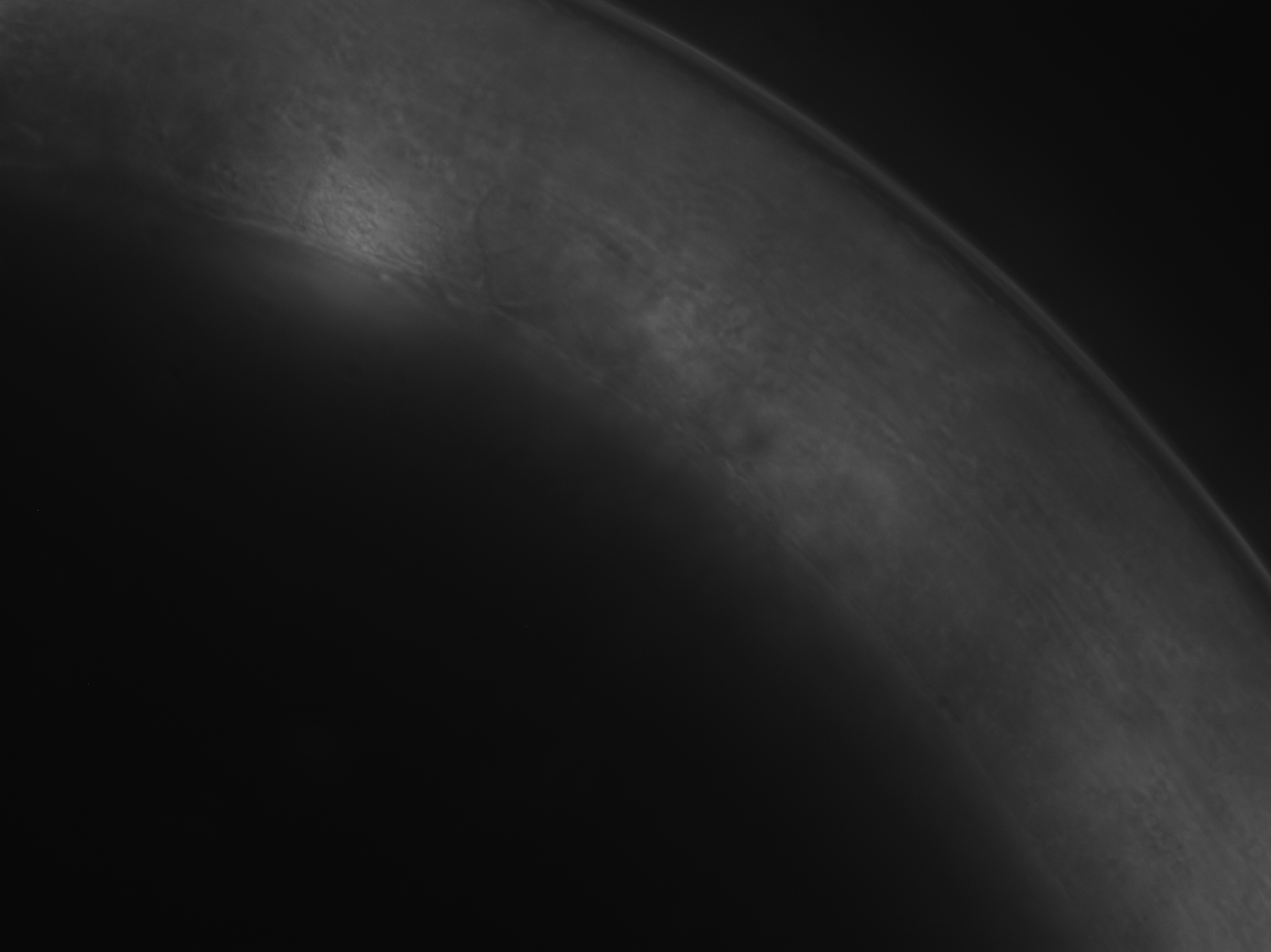

Supplement: Supplementary file 4 — Source data Fig. 3 [file 44319_2025_493_MOESM4_ESM.zip › Figure3/Fig3A/Experiment-14_VC_upstreamdeletion.tif_files/Experiment-14_z7c0x0-1388y0-1040.tif]

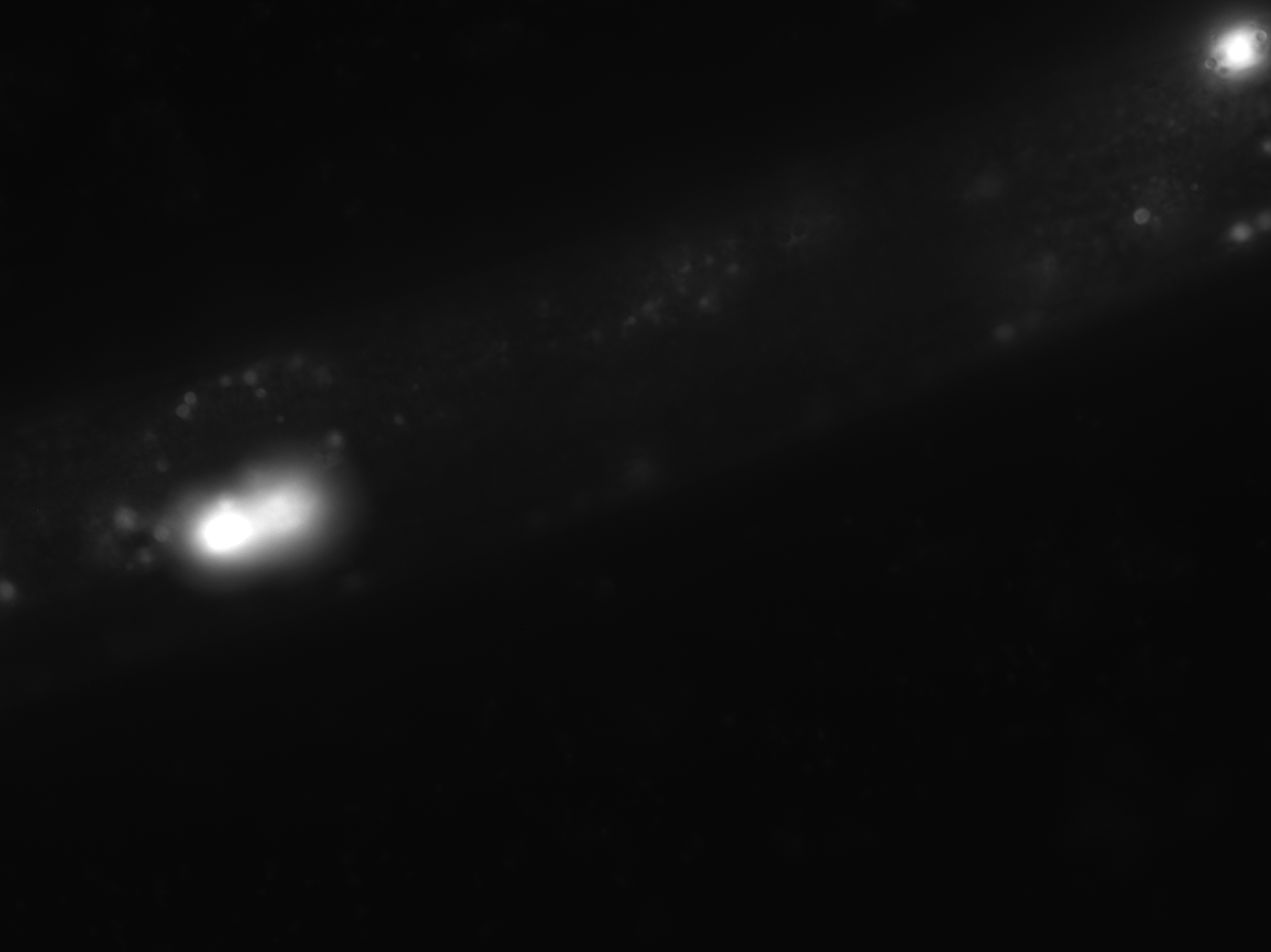

Supplement: Supplementary file 4 — Source data Fig. 3 [file 44319_2025_493_MOESM4_ESM.zip › Figure3/Fig3A/Experiment-63_VC_downstreamdeletion.tif_files/Experiment-63good_z6c0x0-1388y0-1040.tif]

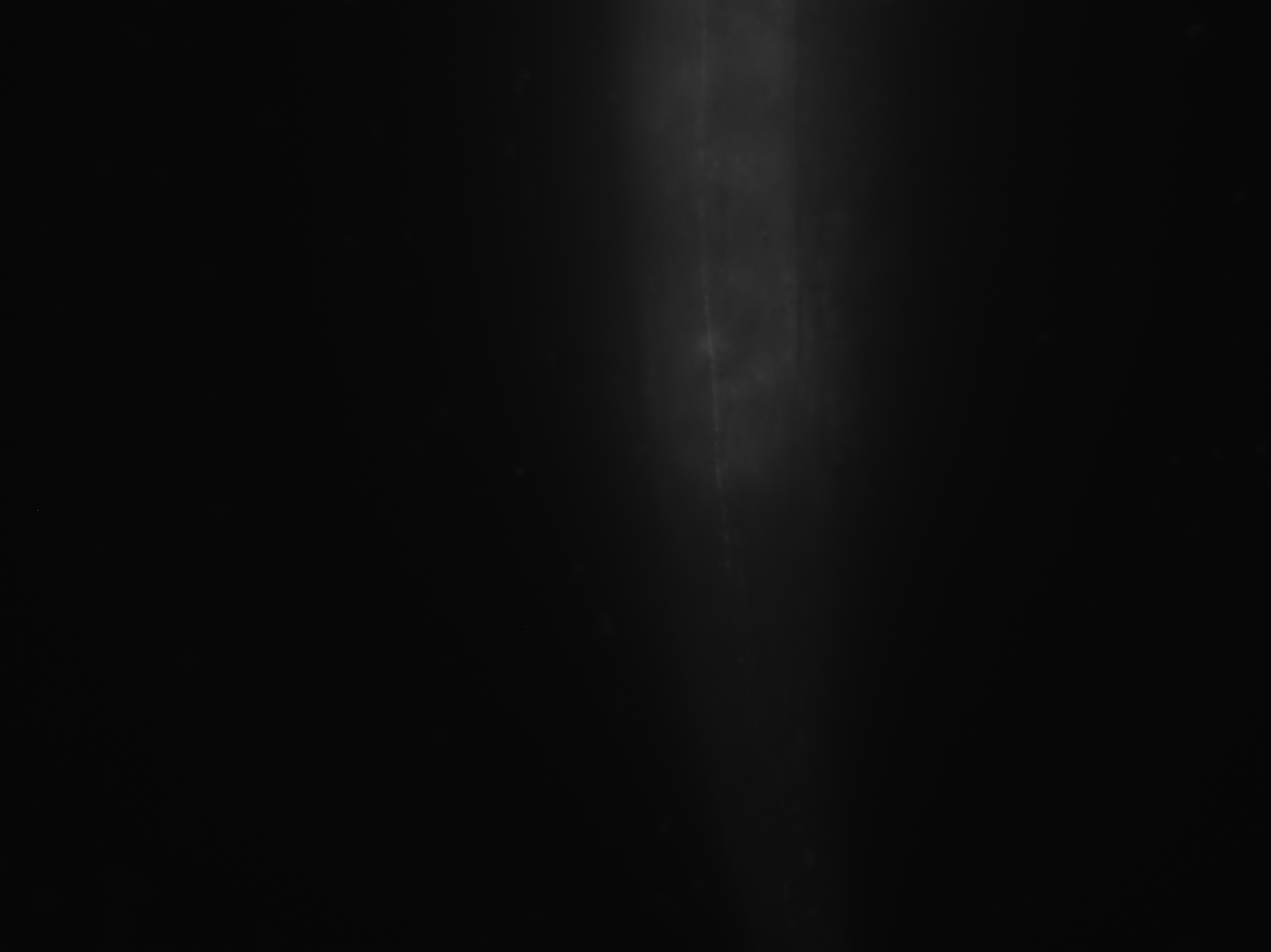

Supplement: Supplementary file 5 — Source data Fig. 4 [file 44319_2025_493_MOESM5_ESM.zip › Figure4/Fig4D/Experiment-96_wildtype.tif_files/Experiment-96_z3c0x0-1388y0-1040.tif]

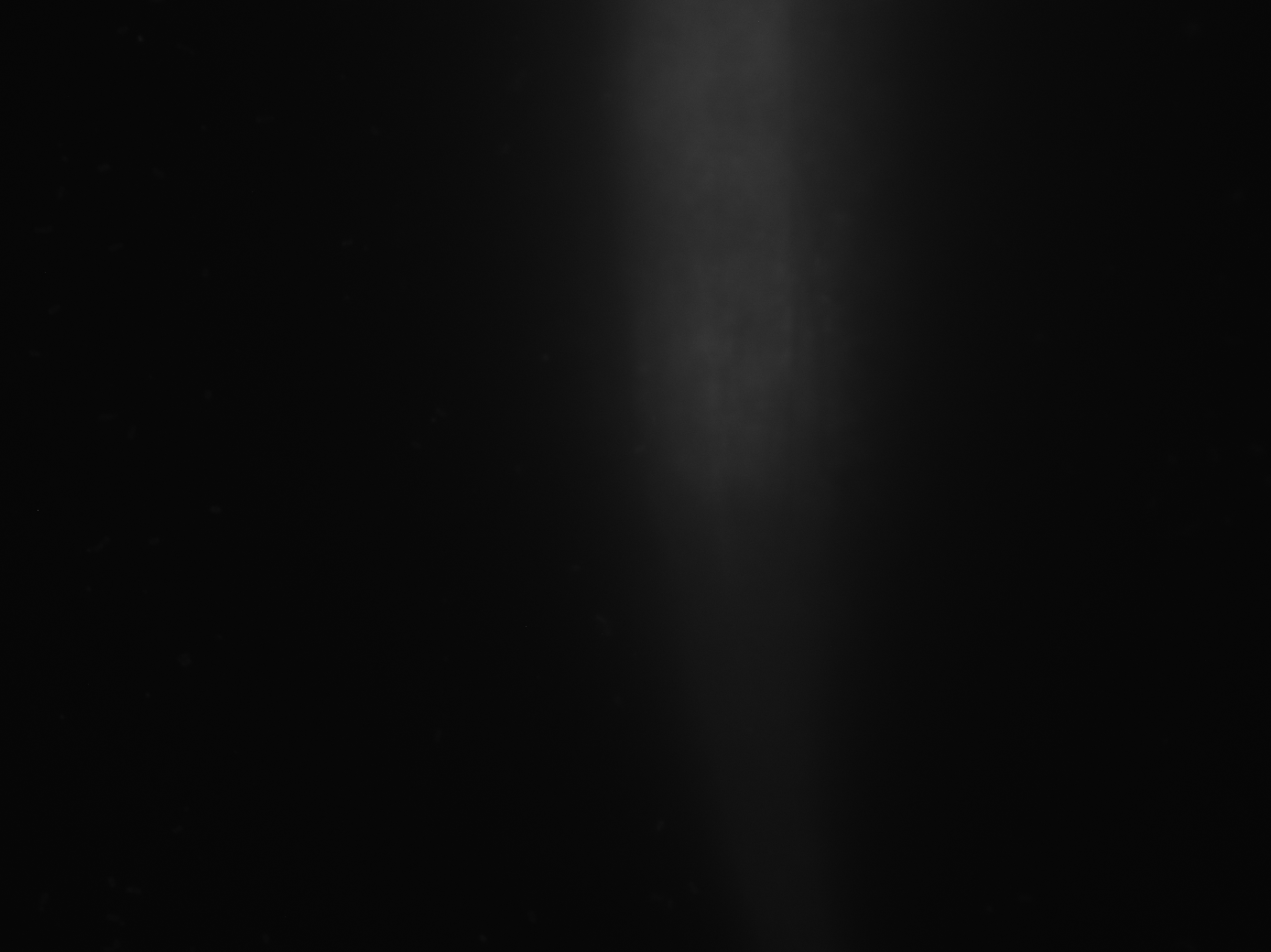

Supplement: Supplementary file 5 — Source data Fig. 4 [file 44319_2025_493_MOESM5_ESM.zip › Figure4/Fig4D/Experiment-96_wildtype.tif_files/Experiment-96_z0c0x0-1388y0-1040.tif]

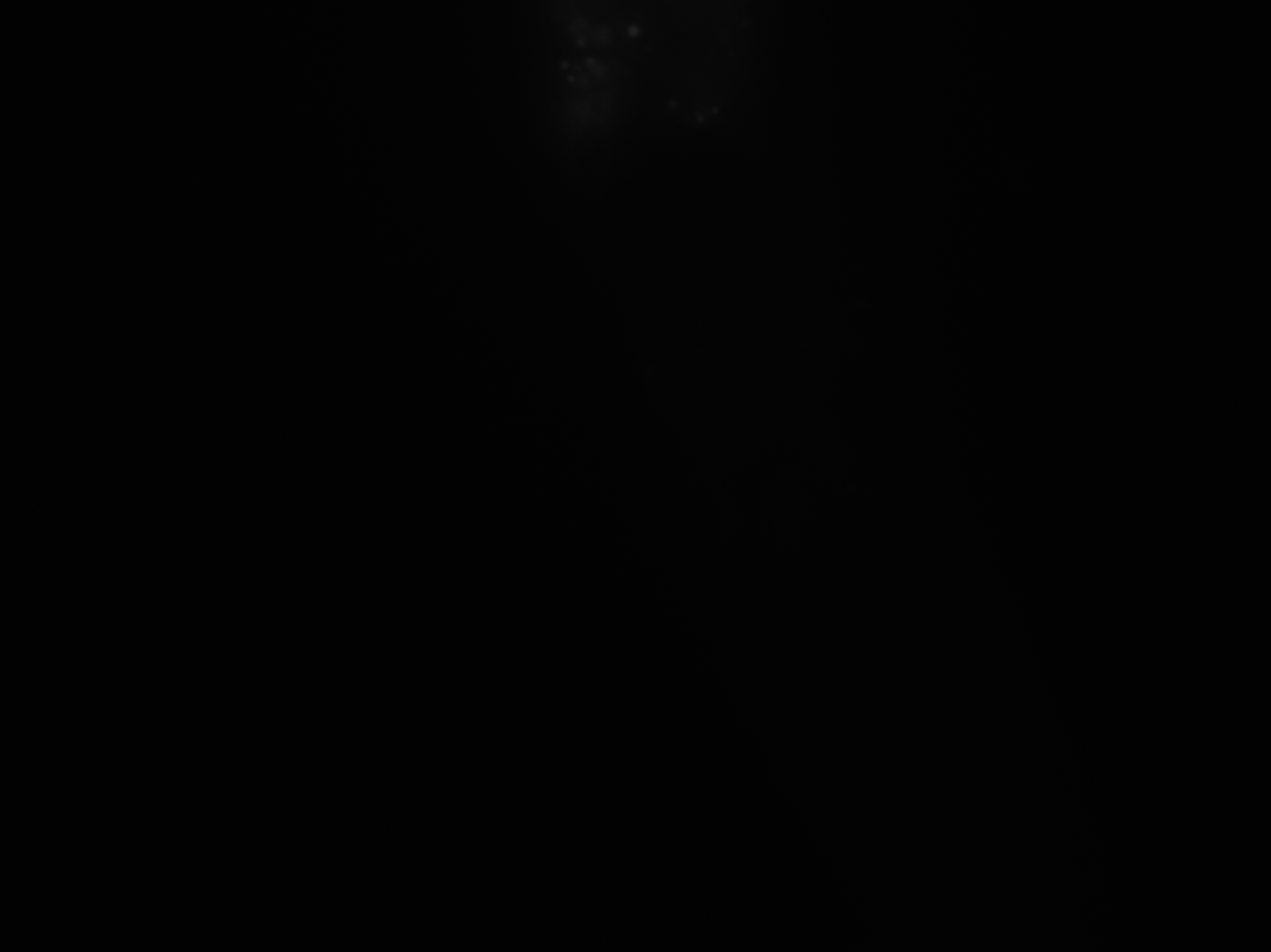

Supplement: Supplementary file 5 — Source data Fig. 4 [file 44319_2025_493_MOESM5_ESM.zip › Figure4/Fig4G/Experiment-62gooddup_AWA.tif_files/Experiment-62gooddup_z3c1x0-1388y0-1040.tif]

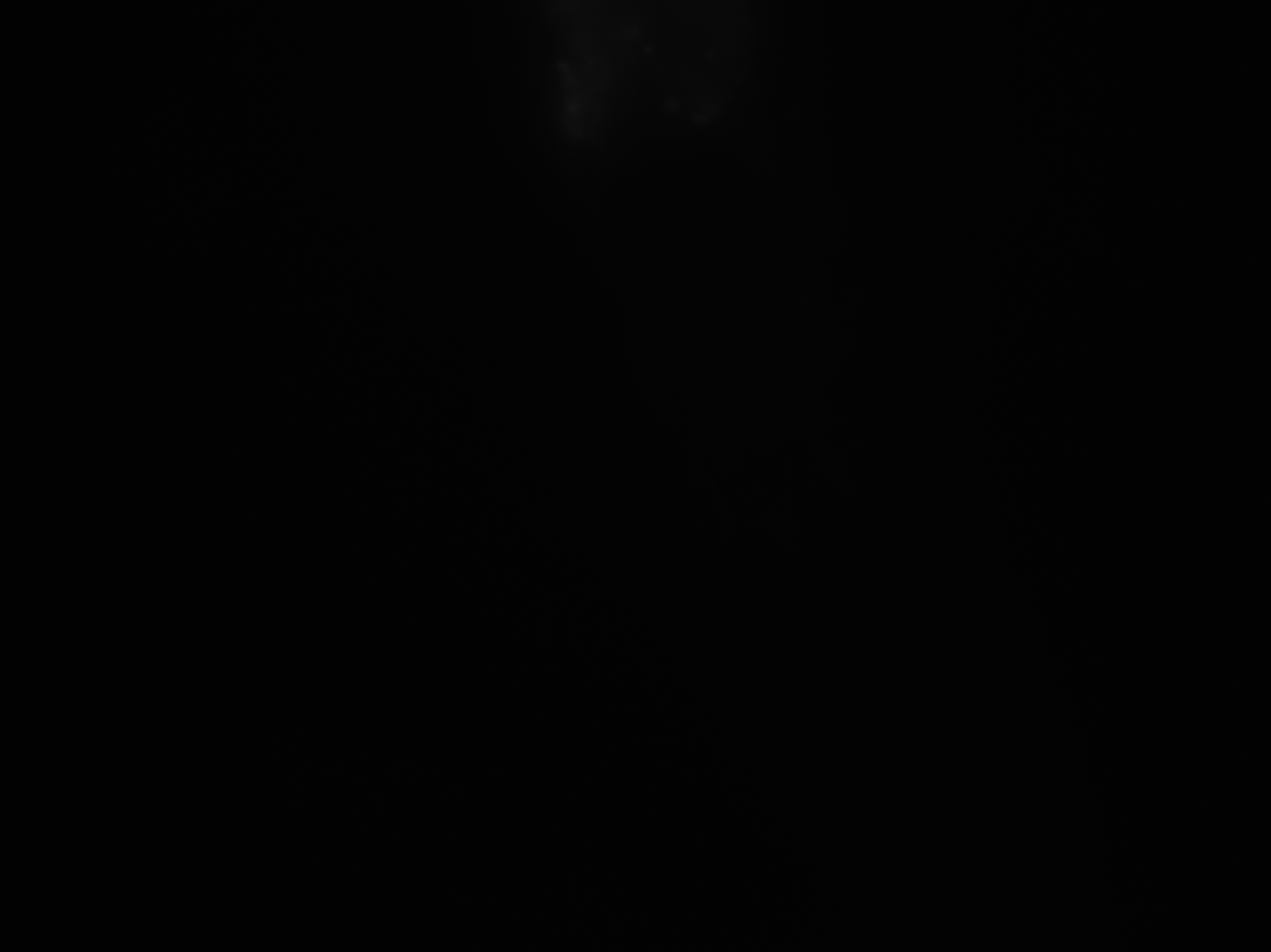

Supplement: Supplementary file 5 — Source data Fig. 4 [file 44319_2025_493_MOESM5_ESM.zip › Figure4/Fig4G/Experiment-62gooddup_AWA.tif_files/Experiment-62gooddup_z5c1x0-1388y0-1040.tif]

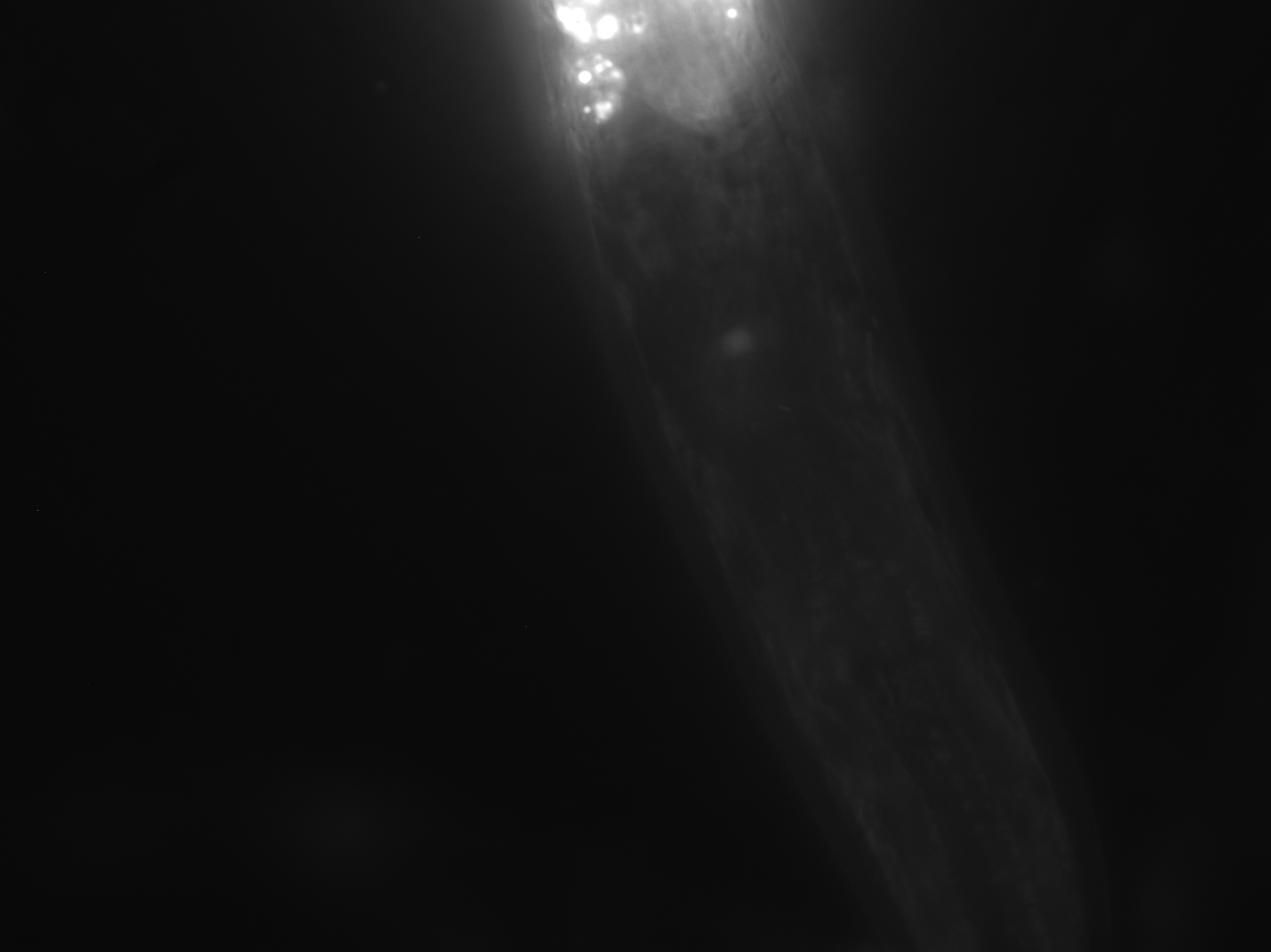

Supplement: Supplementary file 5 — Source data Fig. 4 [file 44319_2025_493_MOESM5_ESM.zip › Figure4/Fig4G/Experiment-62gooddup_AWA.tif_files/Experiment-62gooddup_z0c0x0-1388y0-1040.tif]

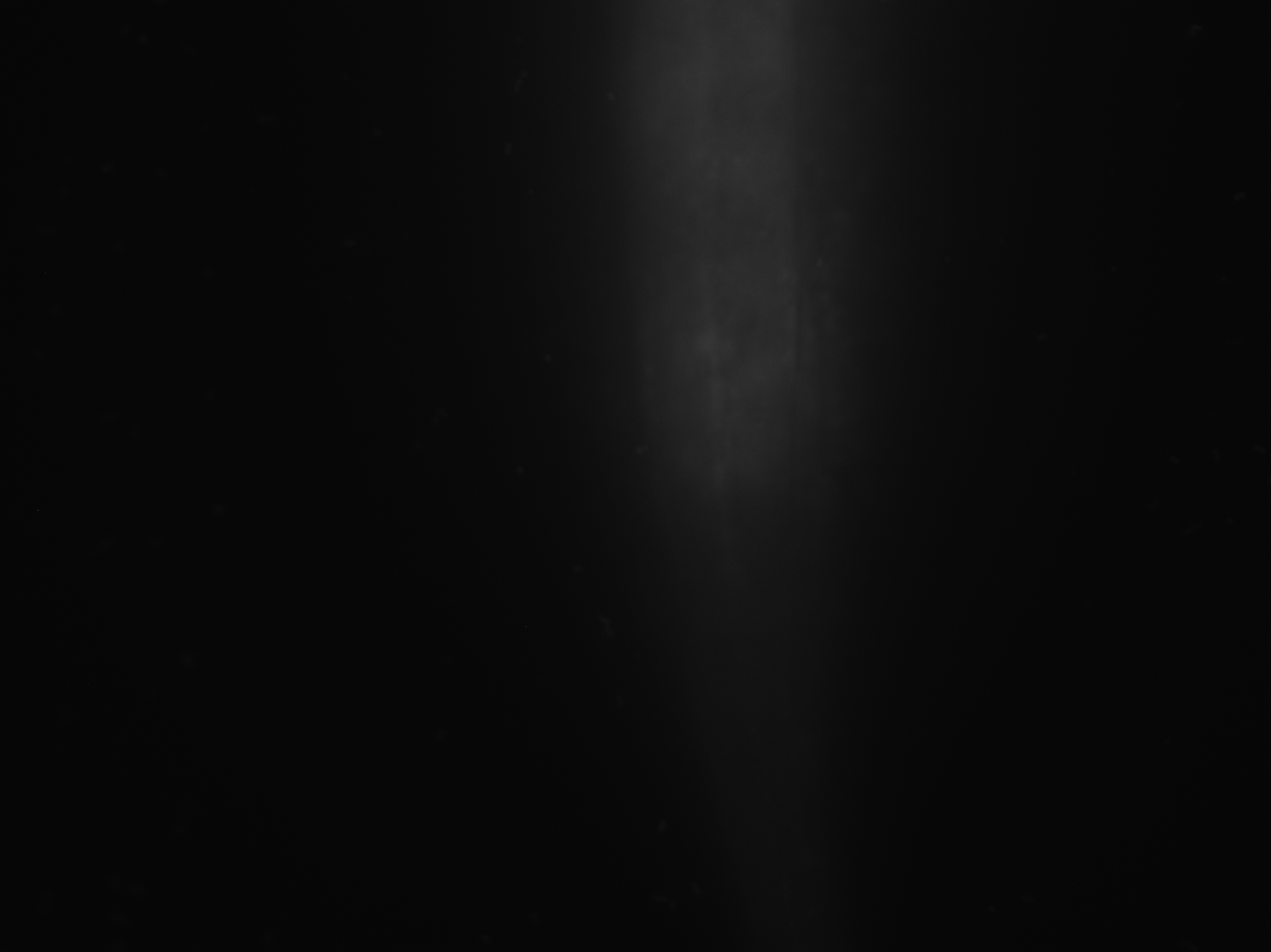

Supplement: Supplementary file 5 — Source data Fig. 4 [file 44319_2025_493_MOESM5_ESM.zip › Figure4/Fig4D/Experiment-96_wildtype.tif_files/Experiment-96_z2c0x0-1388y0-1040.tif]

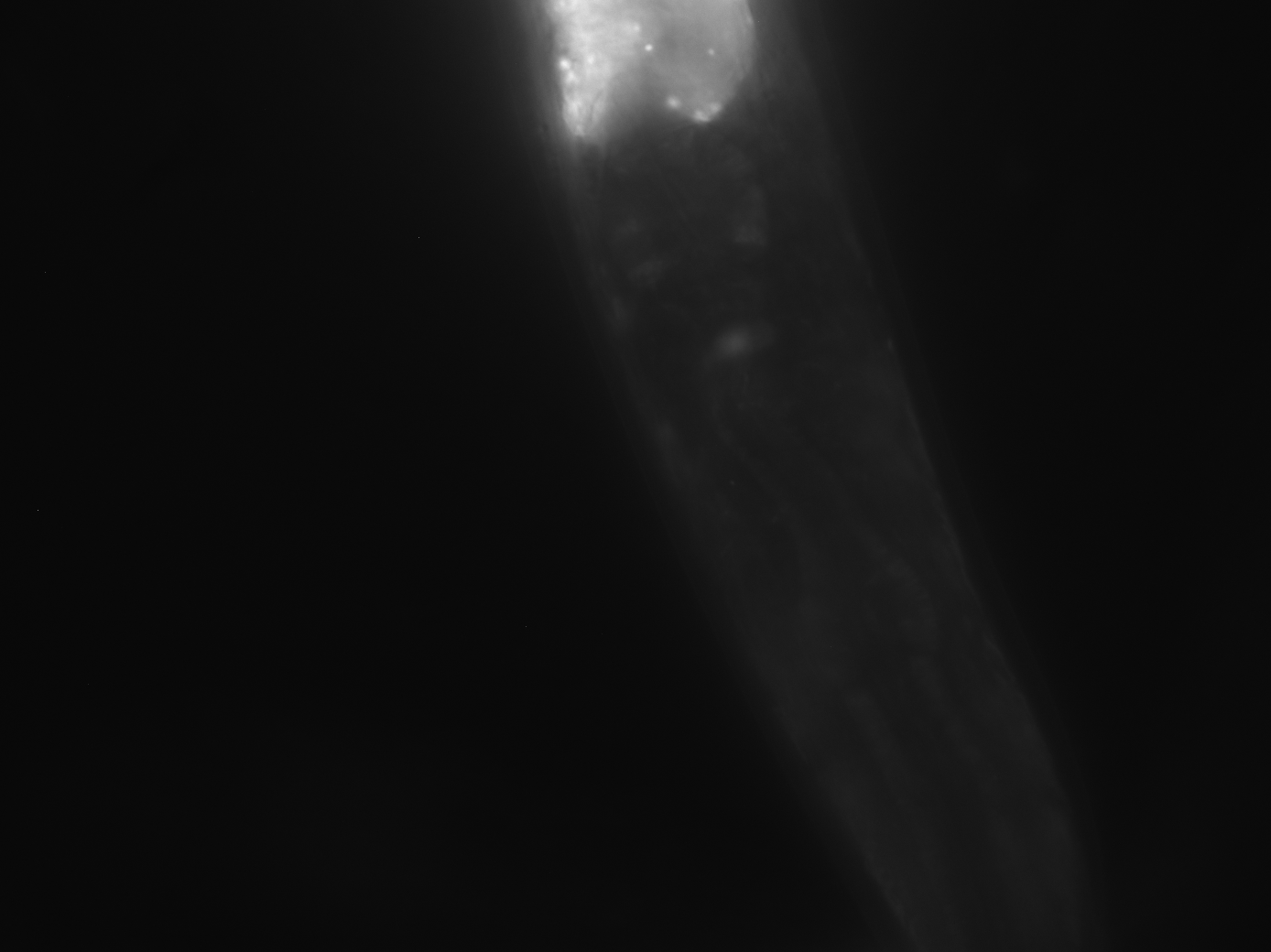

Supplement: Supplementary file 5 — Source data Fig. 4 [file 44319_2025_493_MOESM5_ESM.zip › Figure4/Fig4G/Experiment-62gooddup_AWA.tif_files/Experiment-62gooddup_z5c0x0-1388y0-1040.tif]

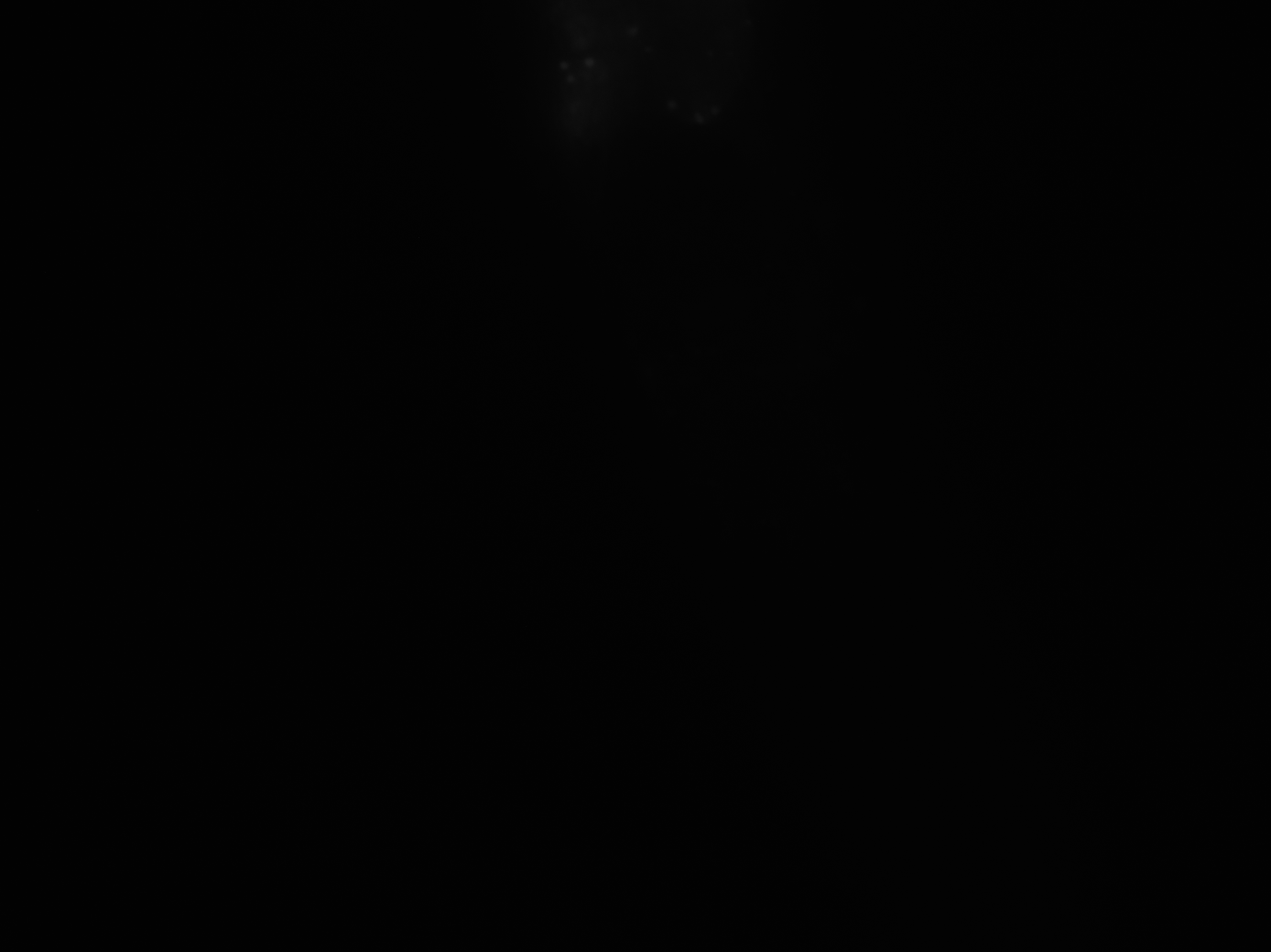

Supplement: Supplementary file 5 — Source data Fig. 4 [file 44319_2025_493_MOESM5_ESM.zip › Figure4/Fig4G/Experiment-62gooddup_AWA.tif_files/Experiment-62gooddup_z4c1x0-1388y0-1040.tif]

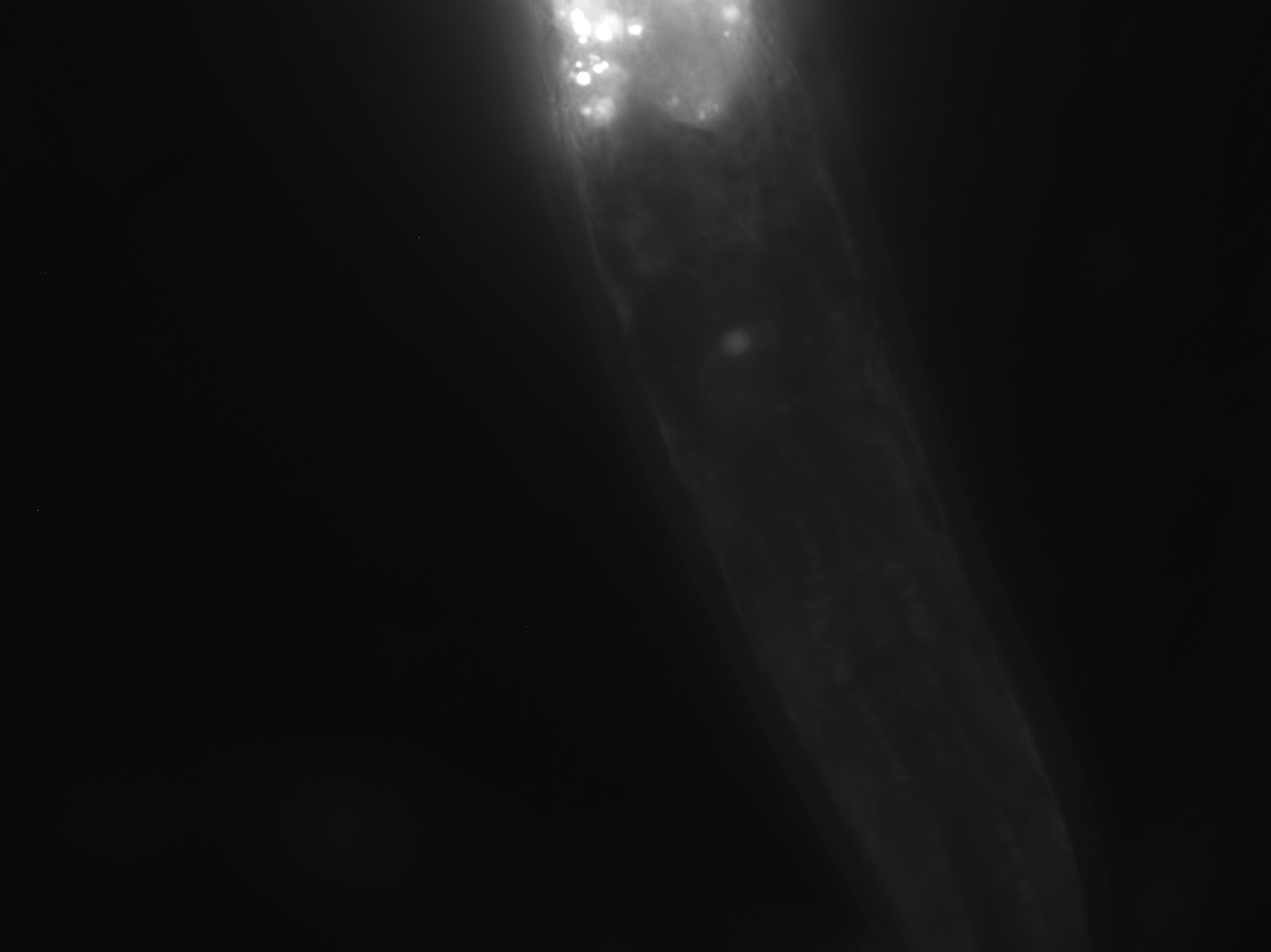

Supplement: Supplementary file 5 — Source data Fig. 4 [file 44319_2025_493_MOESM5_ESM.zip › Figure4/Fig4G/Experiment-62gooddup_AWA.tif_files/Experiment-62gooddup_z2c0x0-1388y0-1040.tif]

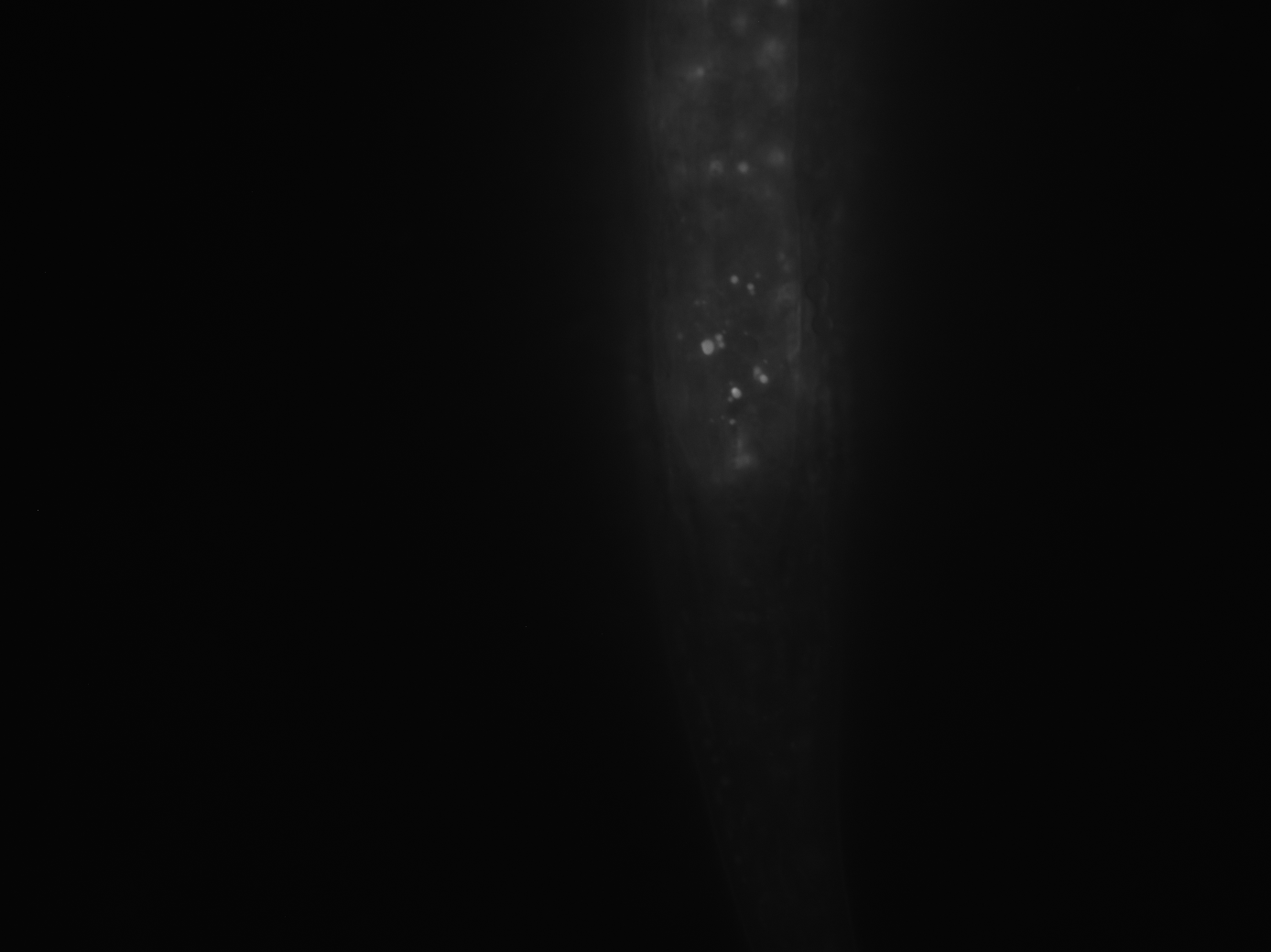

Supplement: Supplementary file 5 — Source data Fig. 4 [file 44319_2025_493_MOESM5_ESM.zip › Figure4/Fig4D/Experiment-96_wildtype.tif_files/Experiment-96_z6c0x0-1388y0-1040.tif]

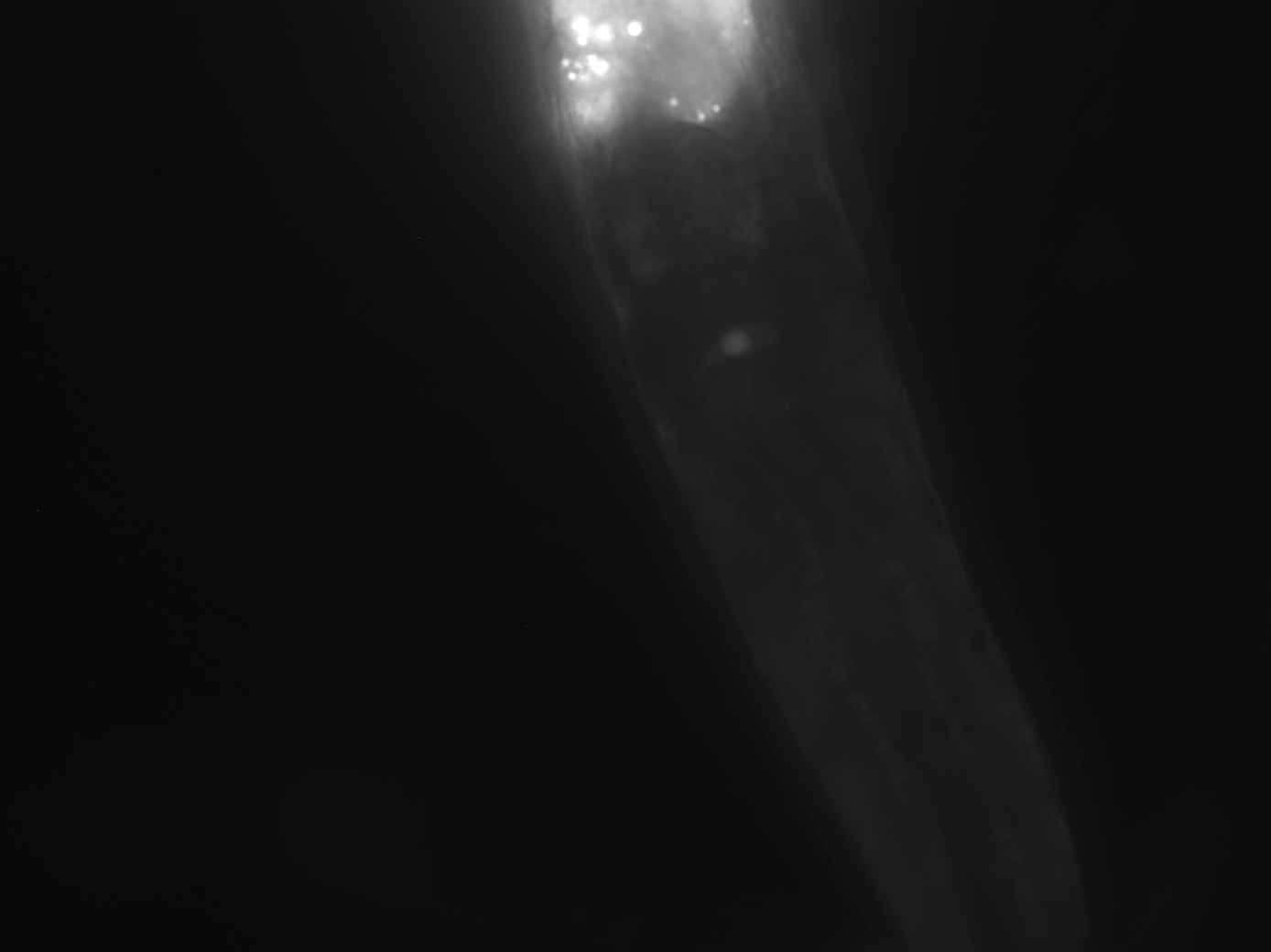

Supplement: Supplementary file 5 — Source data Fig. 4 [file 44319_2025_493_MOESM5_ESM.zip › Figure4/Fig4G/Experiment-62gooddup_AWA.tif_files/Experiment-62gooddup_z3c0x0-1388y0-1040.tif]

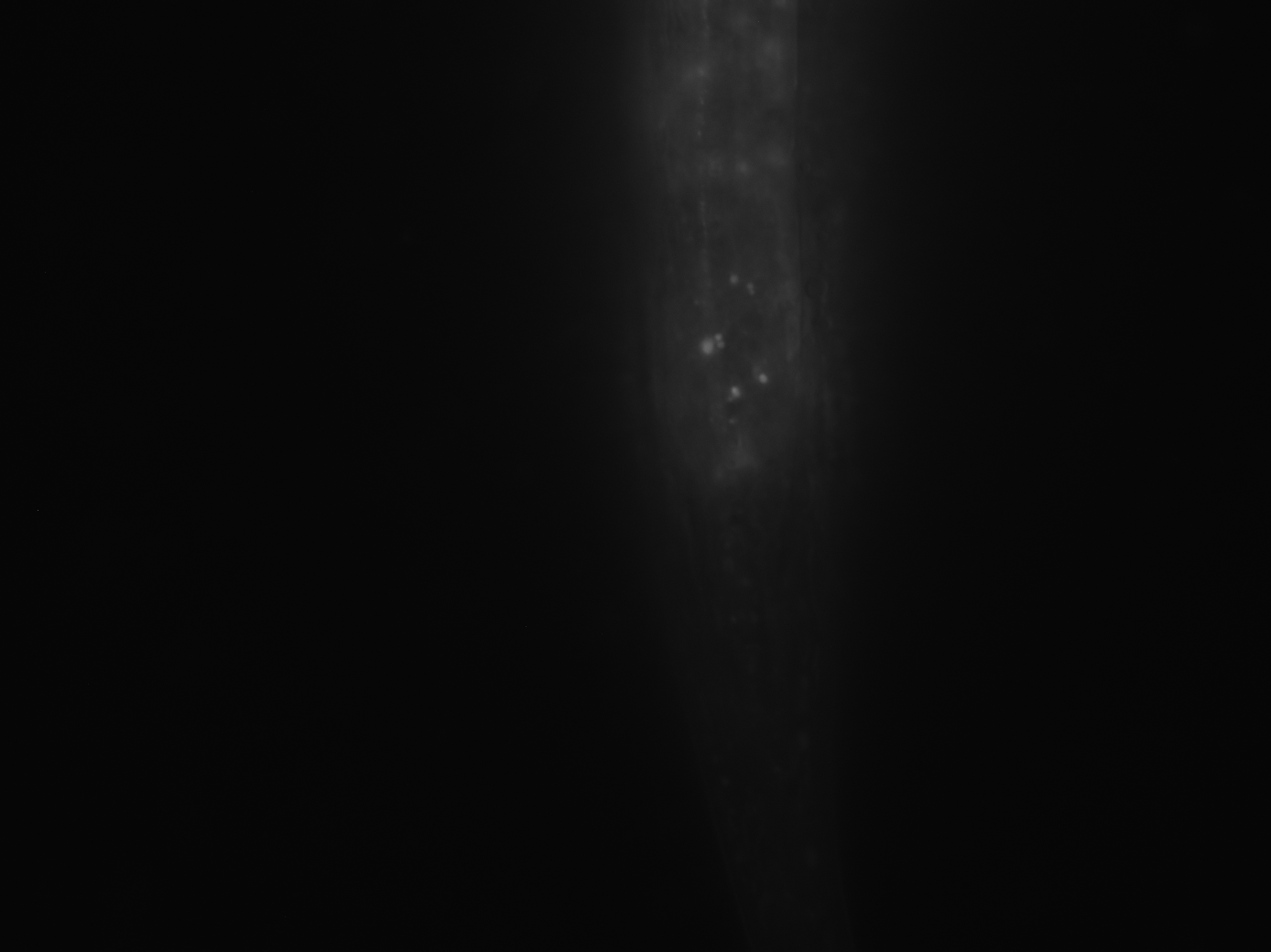

Supplement: Supplementary file 5 — Source data Fig. 4 [file 44319_2025_493_MOESM5_ESM.zip › Figure4/Fig4D/Experiment-96_wildtype.tif_files/Experiment-96_z5c0x0-1388y0-1040.tif]

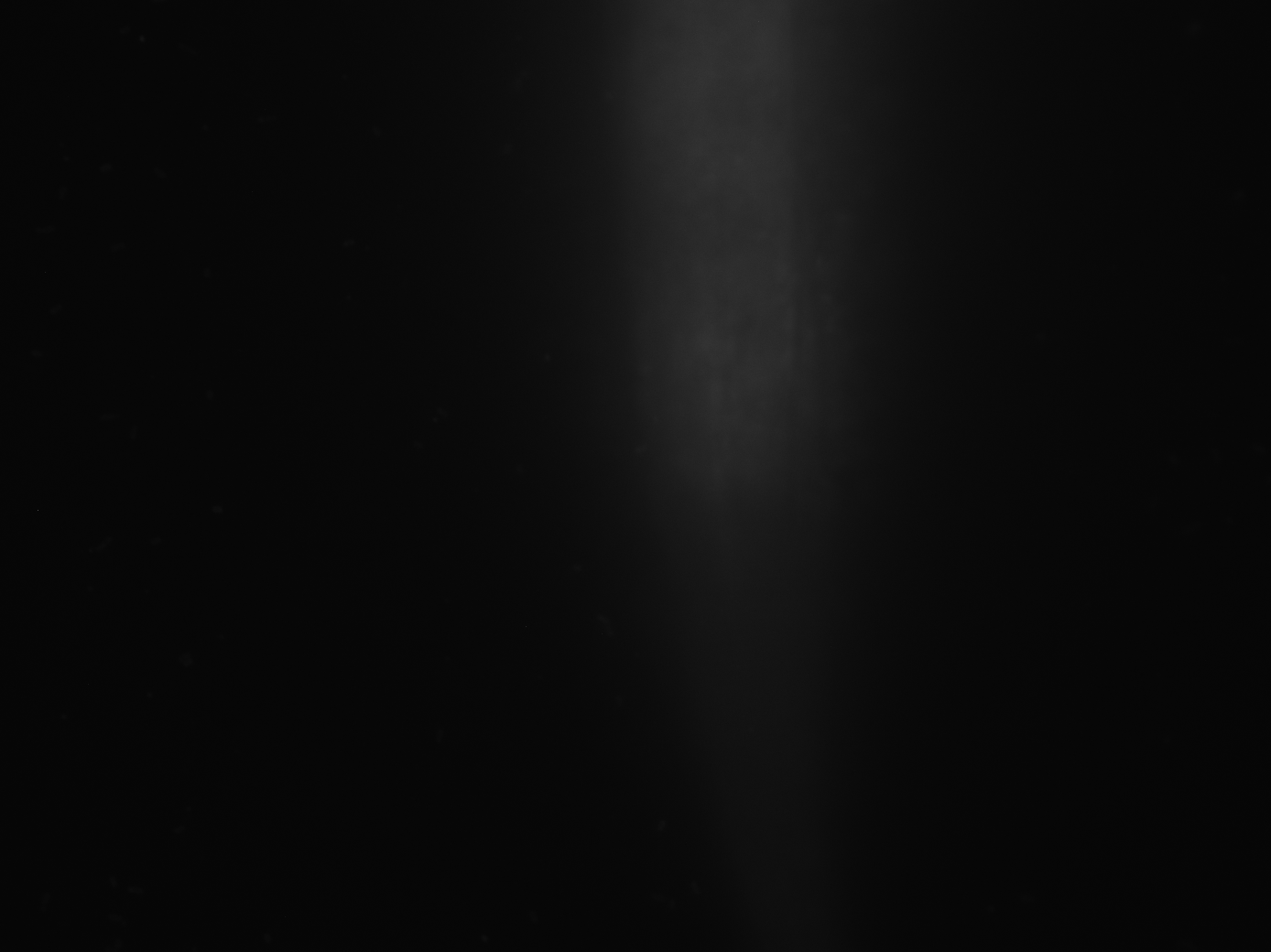

Supplement: Supplementary file 5 — Source data Fig. 4 [file 44319_2025_493_MOESM5_ESM.zip › Figure4/Fig4D/Experiment-96_wildtype.tif_files/Experiment-96_z1c0x0-1388y0-1040.tif]

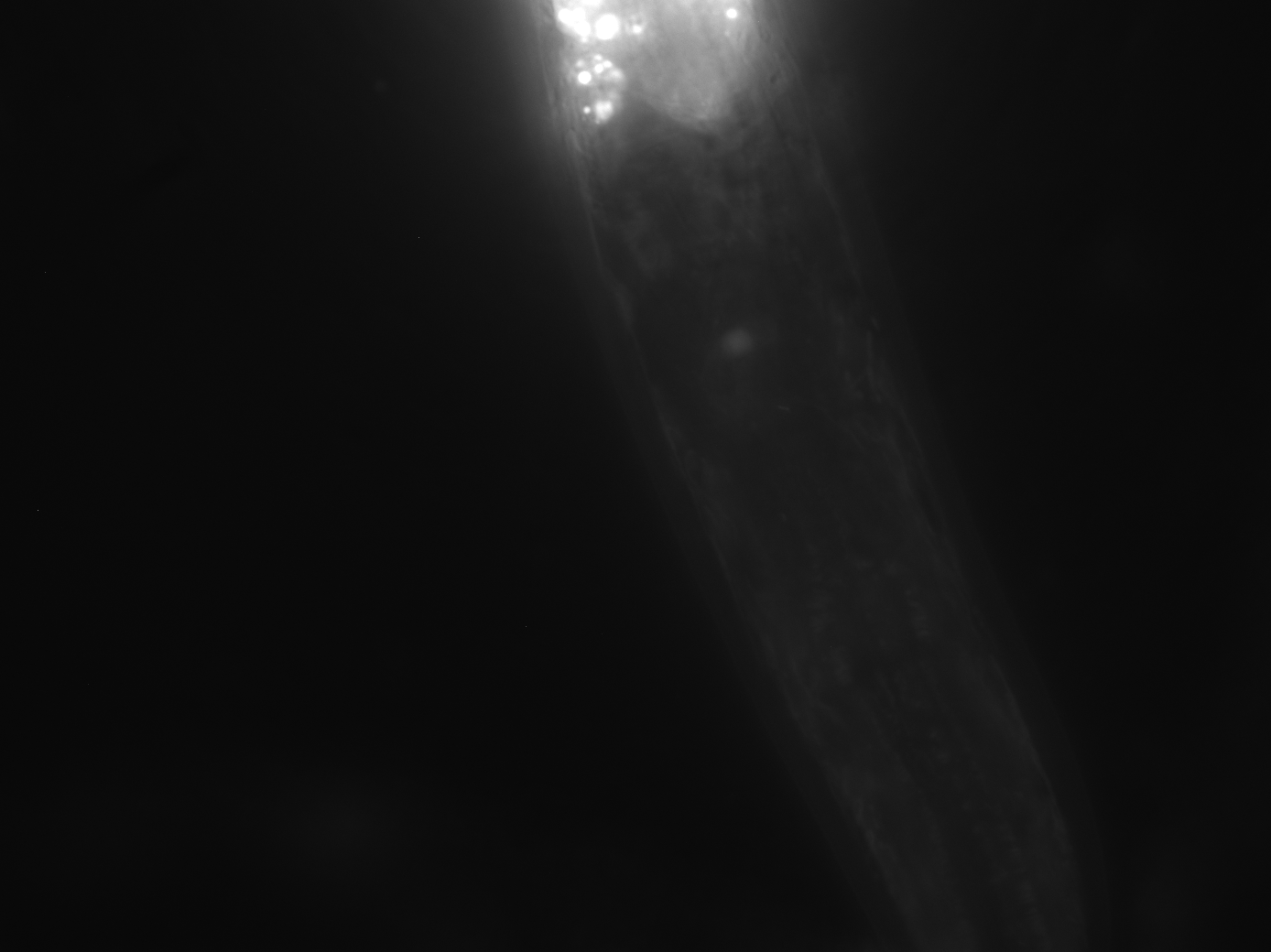

Supplement: Supplementary file 5 — Source data Fig. 4 [file 44319_2025_493_MOESM5_ESM.zip › Figure4/Fig4G/Experiment-62gooddup_AWA.tif_files/Experiment-62gooddup_z1c0x0-1388y0-1040.tif]

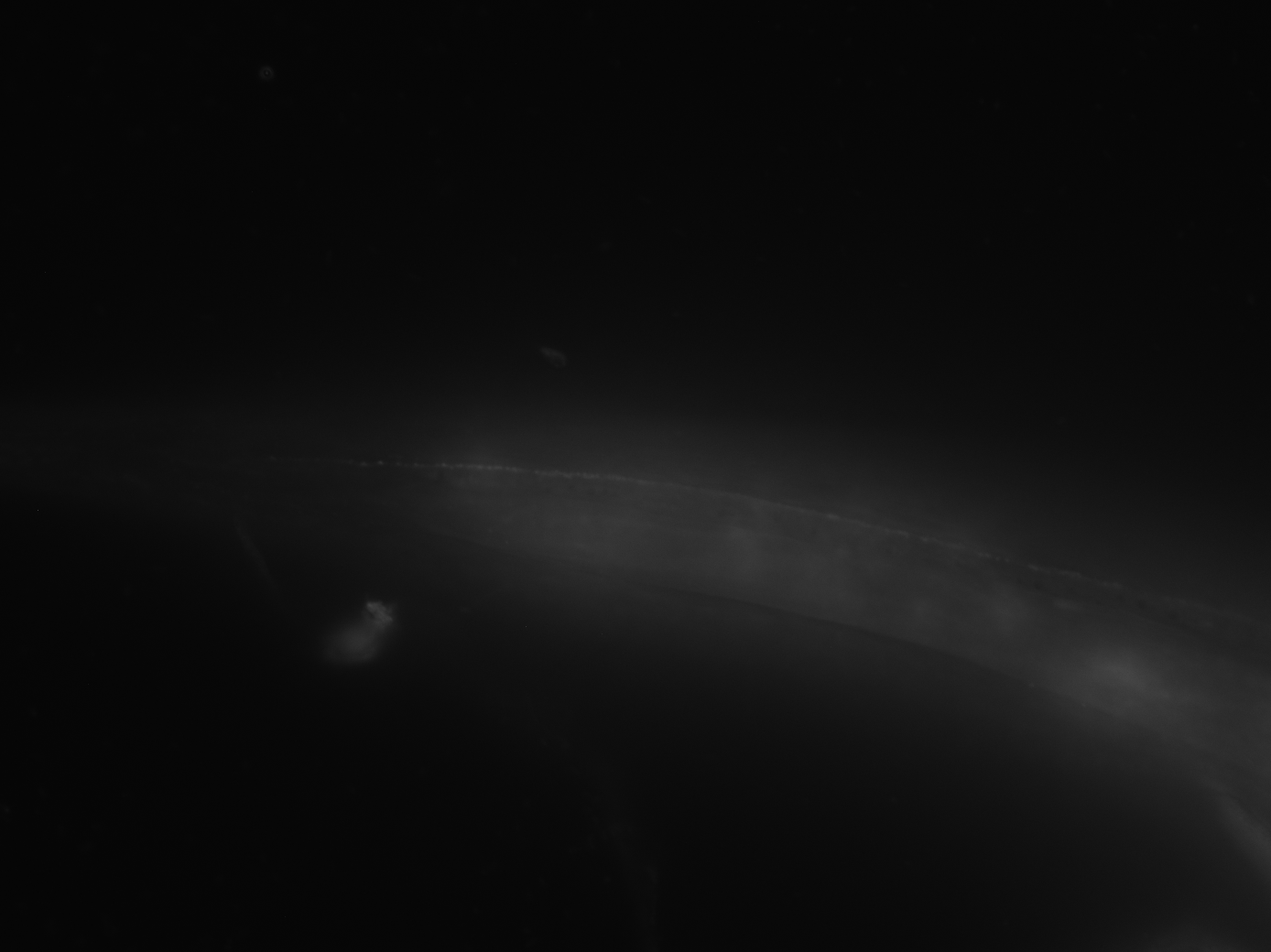

Supplement: Supplementary file 5 — Source data Fig. 4 [file 44319_2025_493_MOESM5_ESM.zip › Figure4/Fig4D/Experiment-106_skipped.tif_files/Experiment-106_z2c0x0-1388y0-1040.tif]

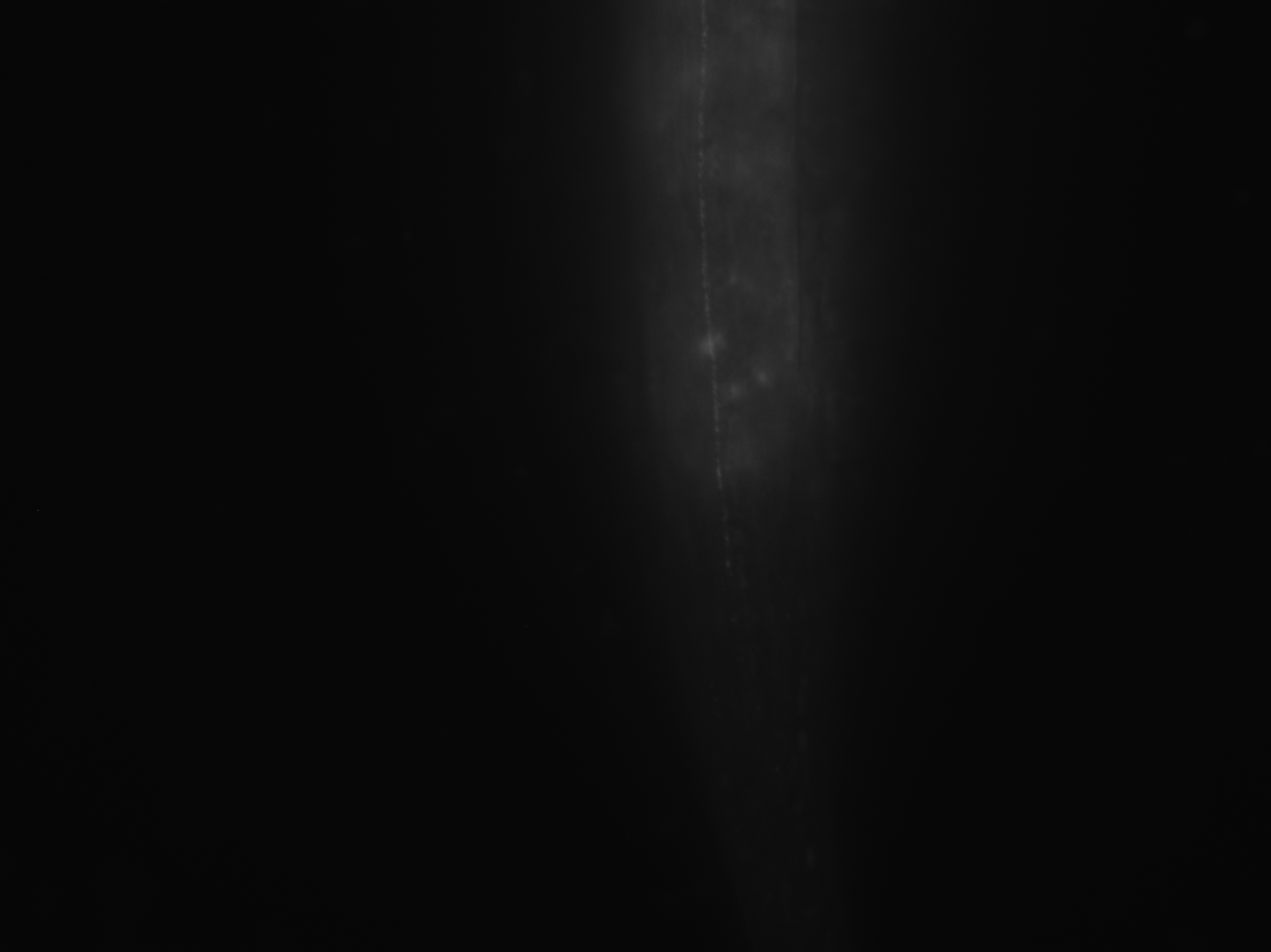

Supplement: Supplementary file 5 — Source data Fig. 4 [file 44319_2025_493_MOESM5_ESM.zip › Figure4/Fig4D/Experiment-96_wildtype.tif_files/Experiment-96_z4c0x0-1388y0-1040.tif]

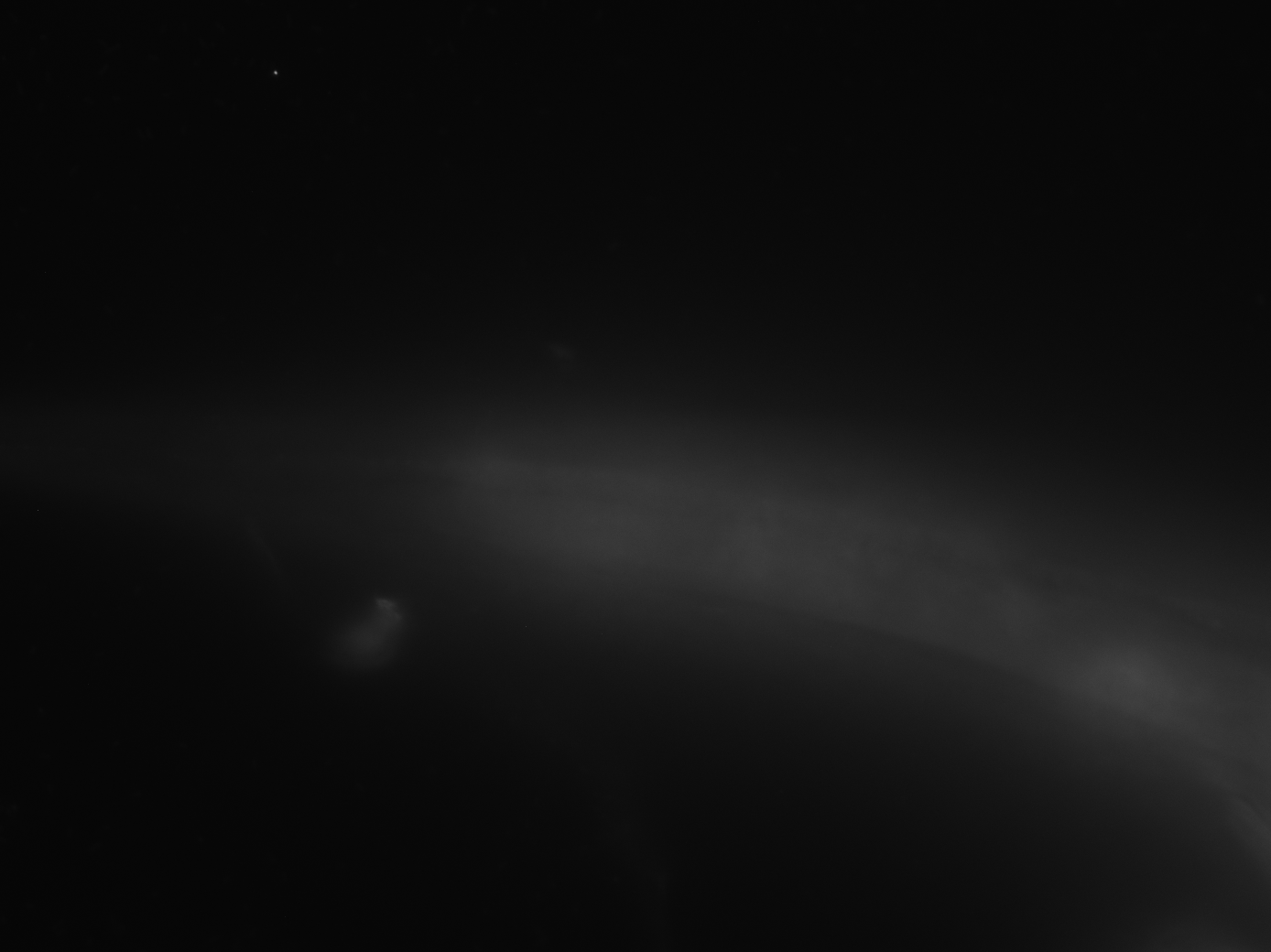

Supplement: Supplementary file 5 — Source data Fig. 4 [file 44319_2025_493_MOESM5_ESM.zip › Figure4/Fig4D/Experiment-106_skipped.tif_files/Experiment-106_z0c0x0-1388y0-1040.tif]

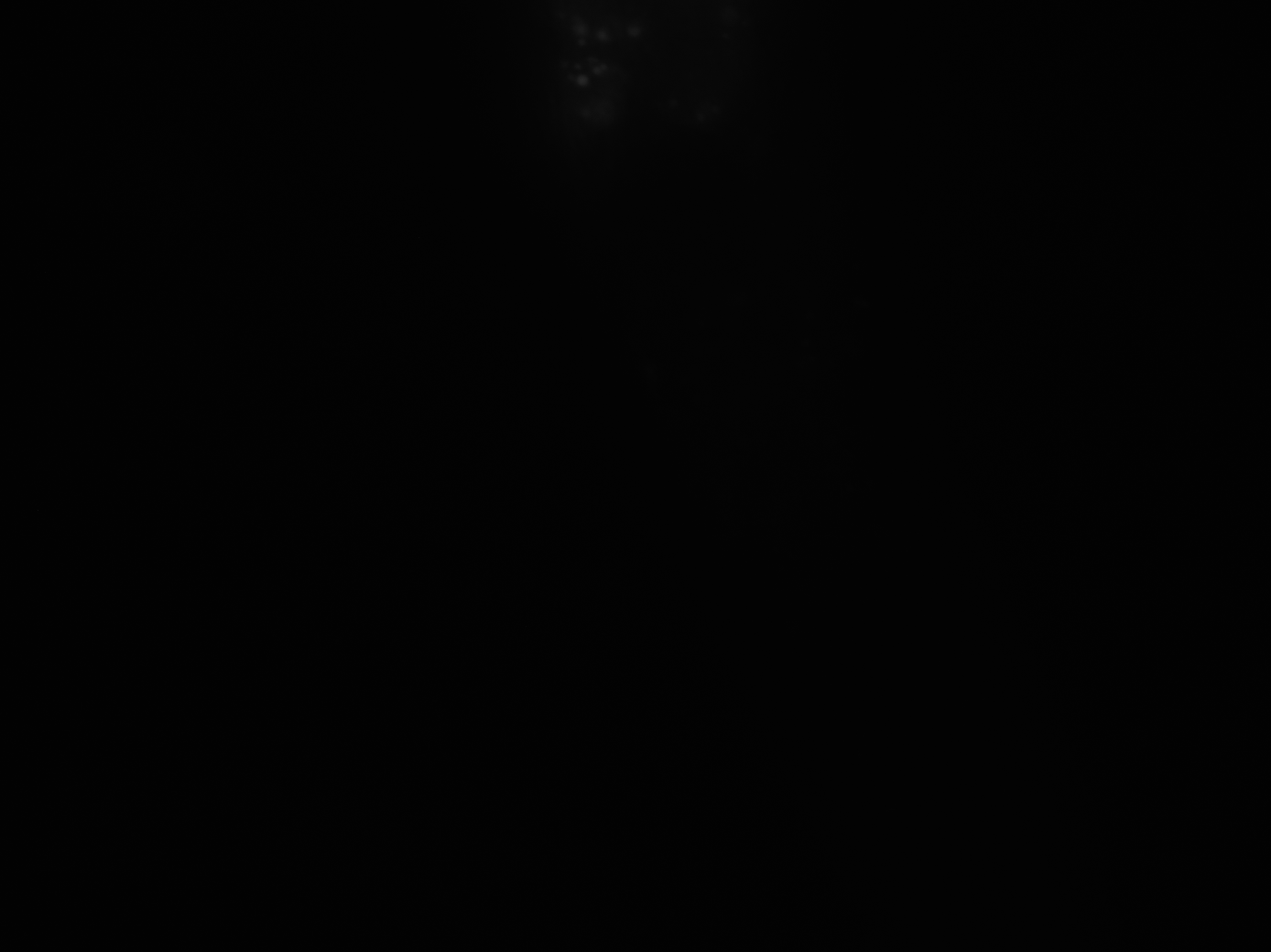

Supplement: Supplementary file 5 — Source data Fig. 4 [file 44319_2025_493_MOESM5_ESM.zip › Figure4/Fig4G/Experiment-62gooddup_AWA.tif_files/Experiment-62gooddup_z2c1x0-1388y0-1040.tif]

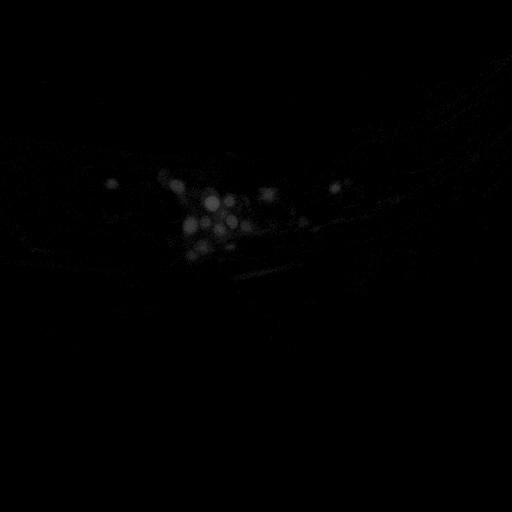

Supplement: Supplementary file 5 — Source data Fig. 4 [file 44319_2025_493_MOESM5_ESM.zip › Figure4/Fig4G/Experiment-645wildtype_NR.czi.tif_files/Experiment-645.czi_h0b0t0z3c1x0-512y0-512.tif]

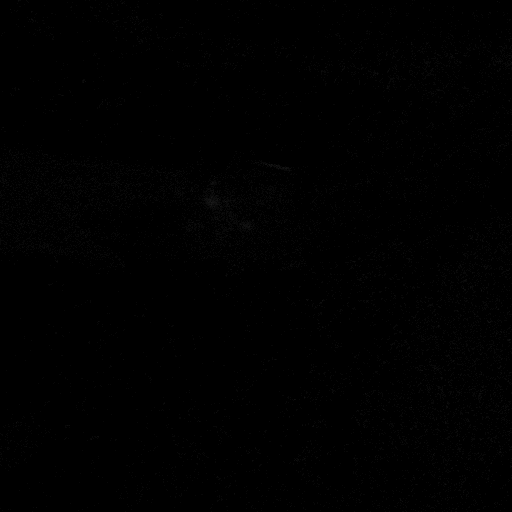

Supplement: Supplementary file 5 — Source data Fig. 4 [file 44319_2025_493_MOESM5_ESM.zip › Figure4/Fig4G/Experiment-645wildtype_NR.czi.tif_files/Experiment-645.czi_h0b0t0z0c1x0-512y0-512.tif]

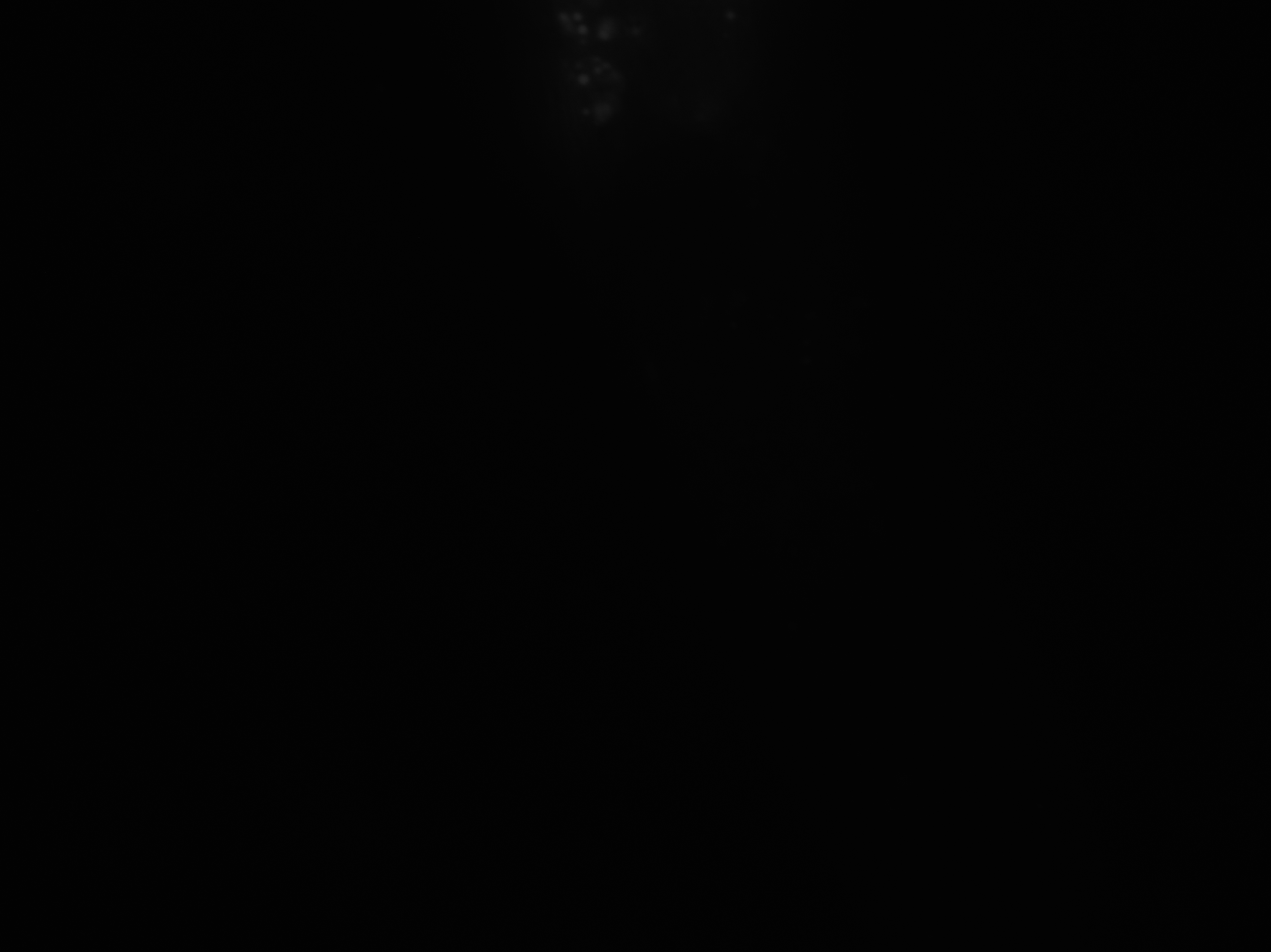

Supplement: Supplementary file 5 — Source data Fig. 4 [file 44319_2025_493_MOESM5_ESM.zip › Figure4/Fig4G/Experiment-62gooddup_AWA.tif_files/Experiment-62gooddup_z0c1x0-1388y0-1040.tif]

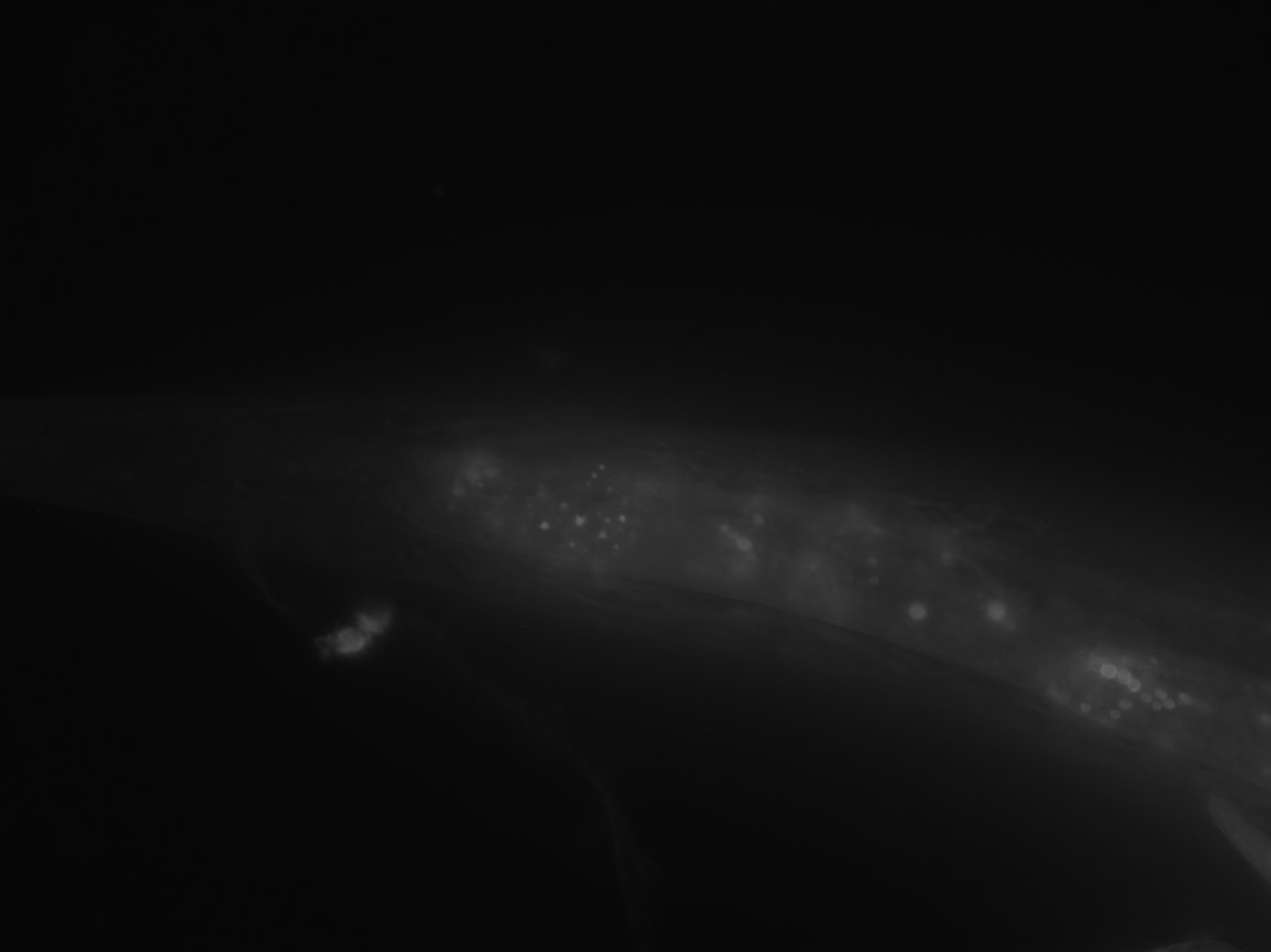

Supplement: Supplementary file 5 — Source data Fig. 4 [file 44319_2025_493_MOESM5_ESM.zip › Figure4/Fig4D/Experiment-106_skipped.tif_files/Experiment-106_z5c0x0-1388y0-1040.tif]

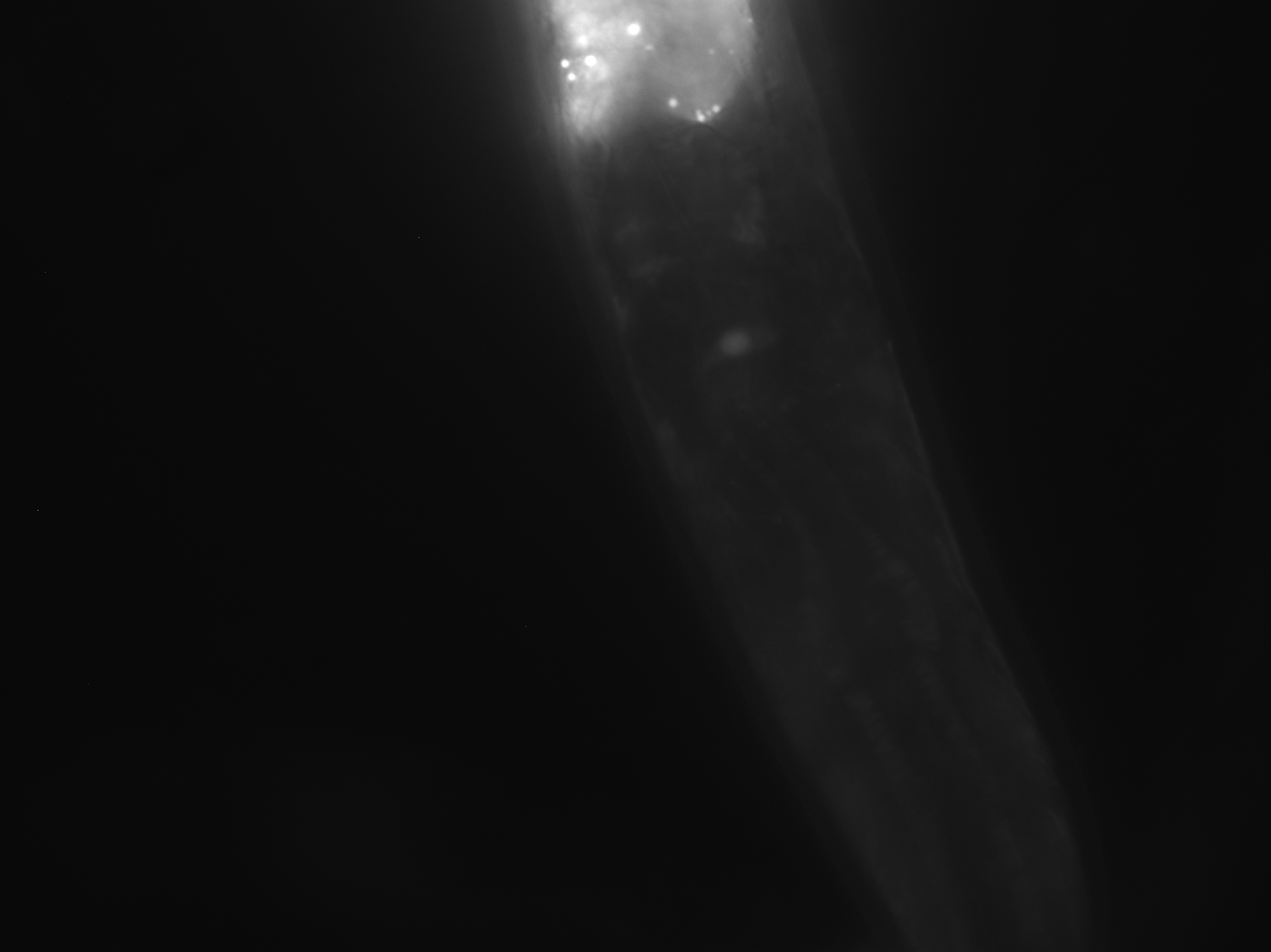

Supplement: Supplementary file 5 — Source data Fig. 4 [file 44319_2025_493_MOESM5_ESM.zip › Figure4/Fig4G/Experiment-62gooddup_AWA.tif_files/Experiment-62gooddup_z4c0x0-1388y0-1040.tif]

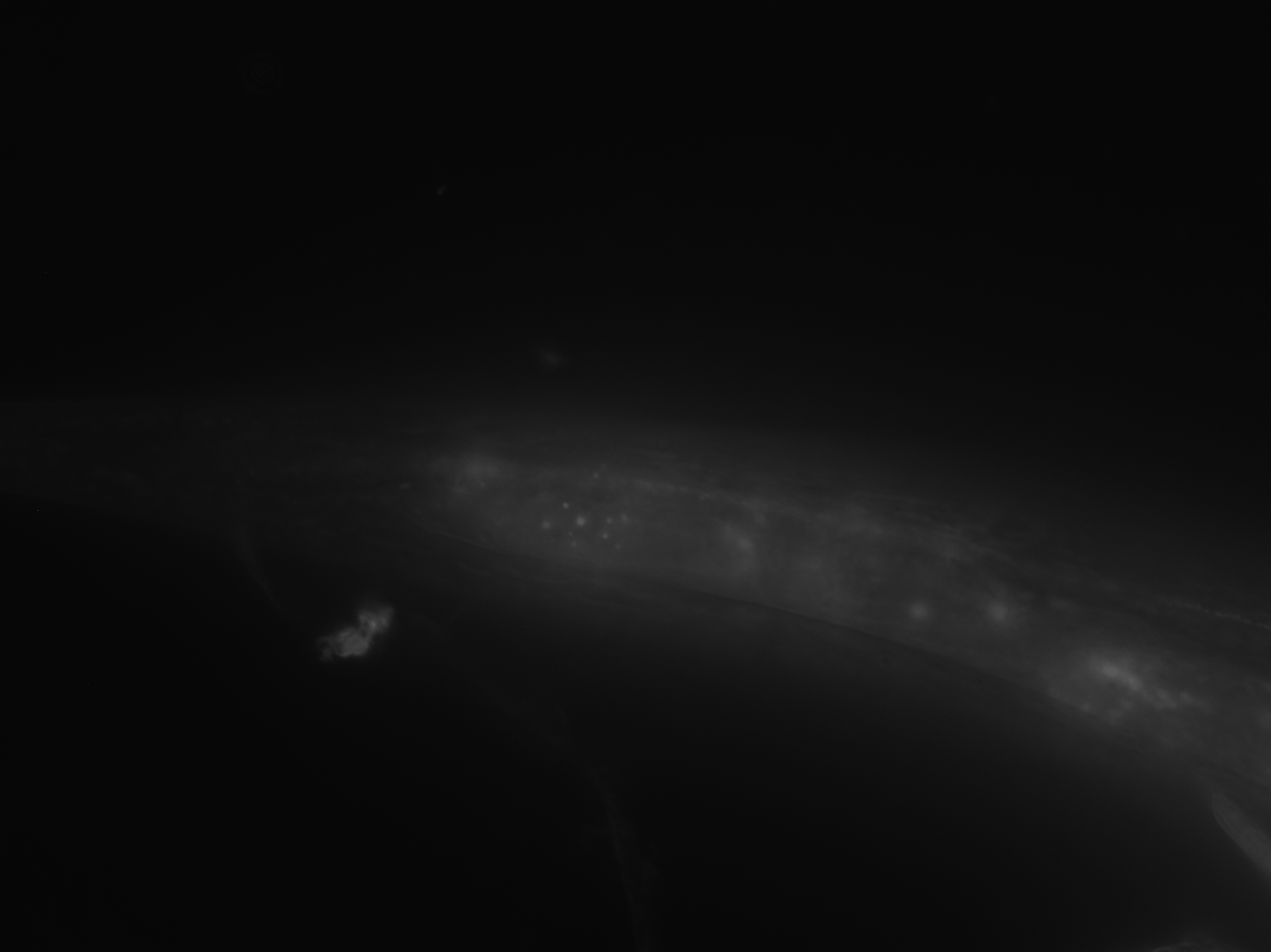

Supplement: Supplementary file 5 — Source data Fig. 4 [file 44319_2025_493_MOESM5_ESM.zip › Figure4/Fig4D/Experiment-106_skipped.tif_files/Experiment-106_z4c0x0-1388y0-1040.tif]

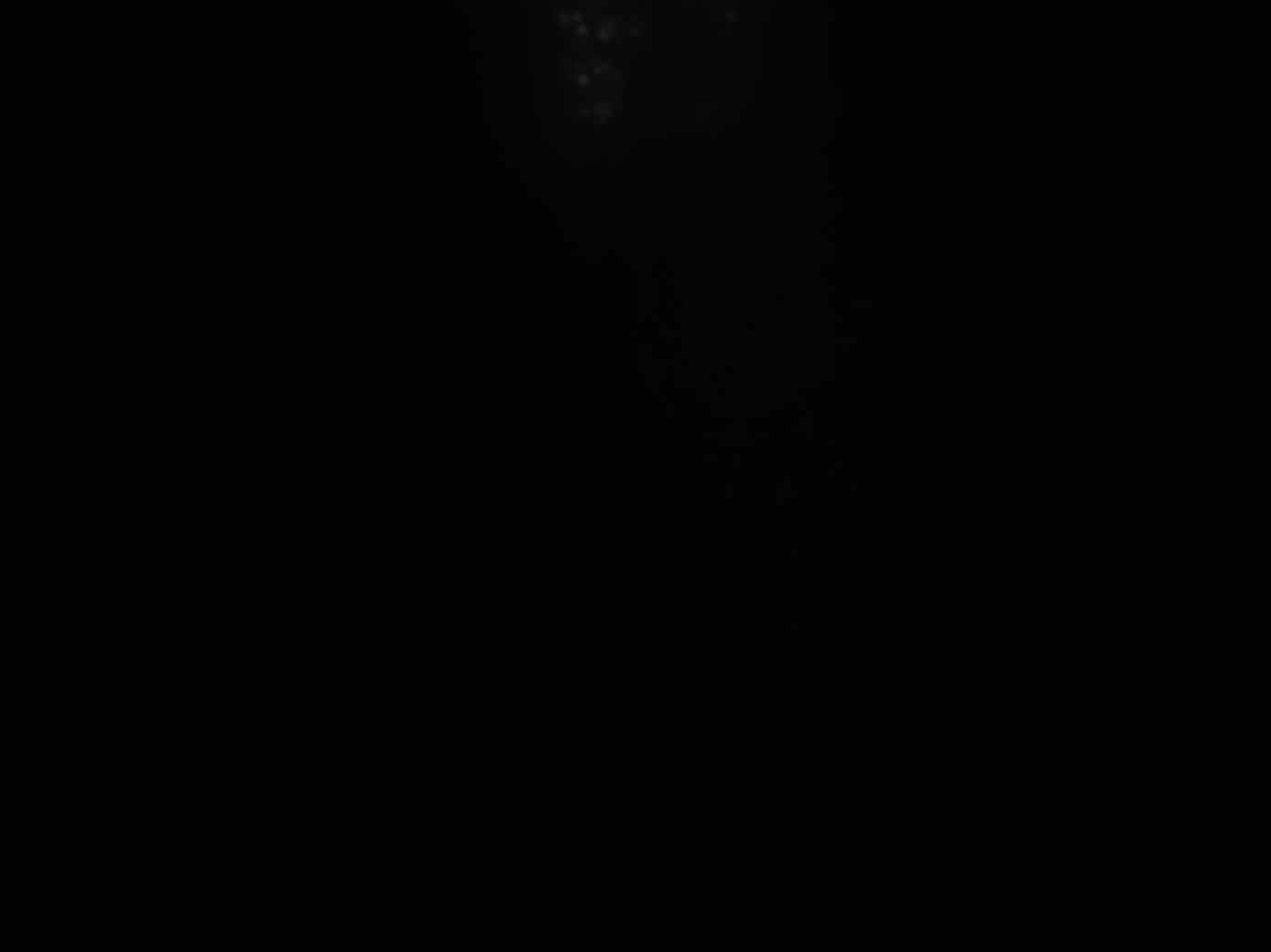

Supplement: Supplementary file 5 — Source data Fig. 4 [file 44319_2025_493_MOESM5_ESM.zip › Figure4/Fig4G/Experiment-62gooddup_AWA.tif_files/Experiment-62gooddup_z1c1x0-1388y0-1040.tif]

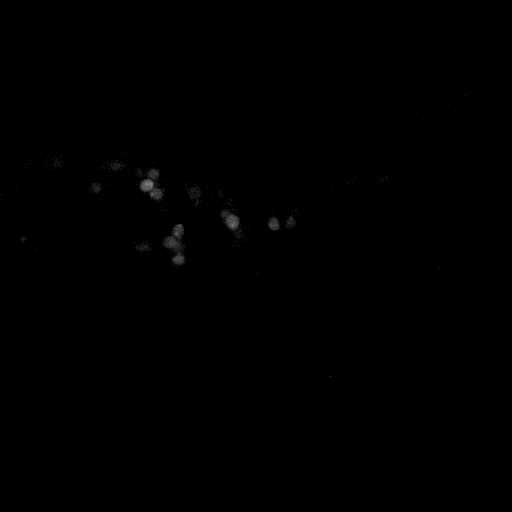

Supplement: Supplementary file 5 — Source data Fig. 4 [file 44319_2025_493_MOESM5_ESM.zip › Figure4/Fig4G/Experiment-645wildtype_NR.czi.tif_files/Experiment-645.czi_h0b0t0z3c0x0-512y0-512.tif]

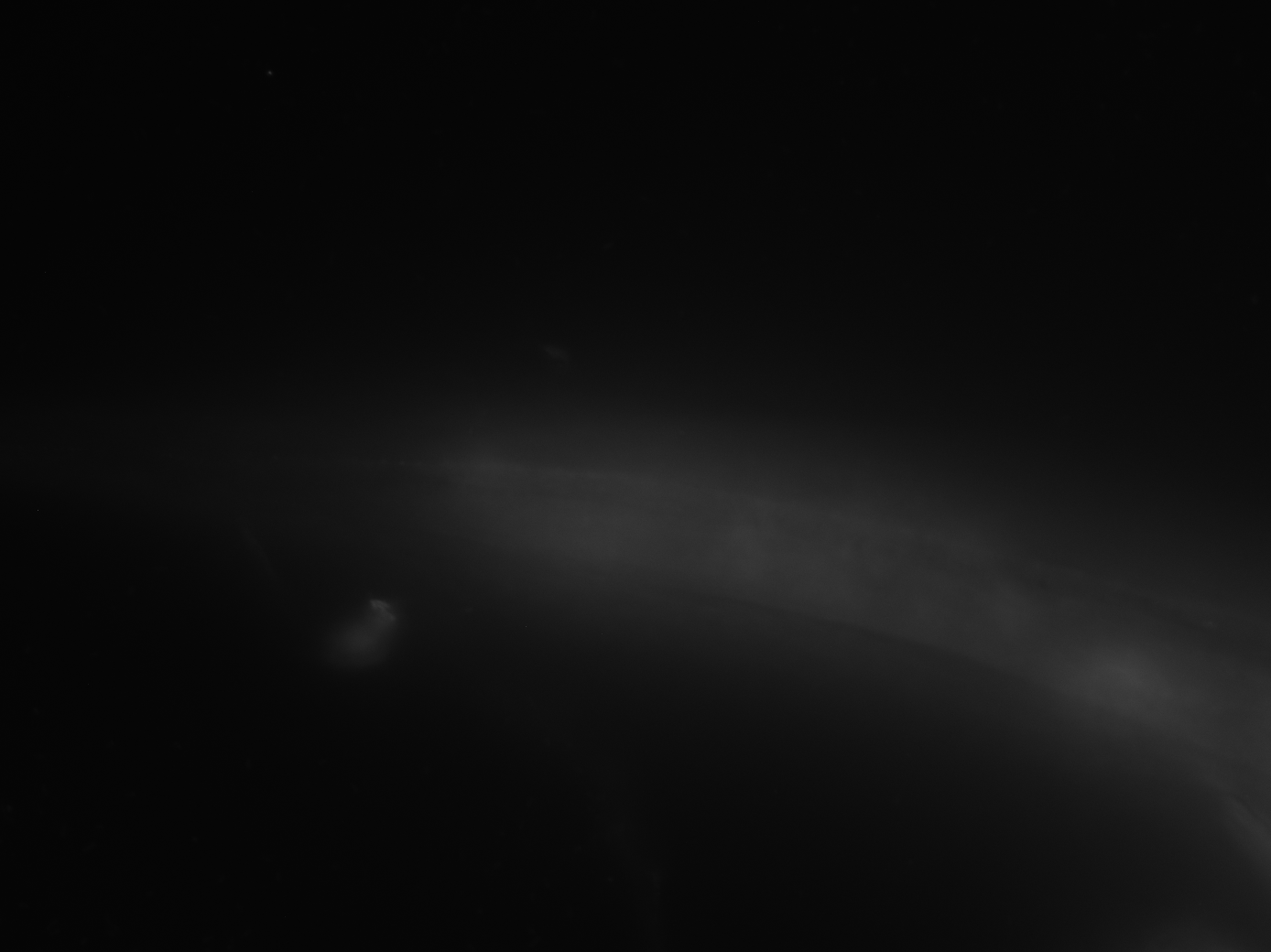

Supplement: Supplementary file 5 — Source data Fig. 4 [file 44319_2025_493_MOESM5_ESM.zip › Figure4/Fig4D/Experiment-106_skipped.tif_files/Experiment-106_z1c0x0-1388y0-1040.tif]

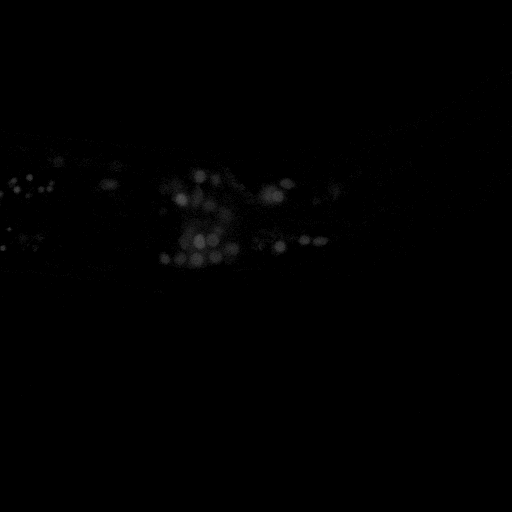

Supplement: Supplementary file 5 — Source data Fig. 4 [file 44319_2025_493_MOESM5_ESM.zip › Figure4/Fig4G/Experiment-645wildtype_NR.czi.tif_files/Experiment-645.czi_h0b0t0z7c1x0-512y0-512.tif]

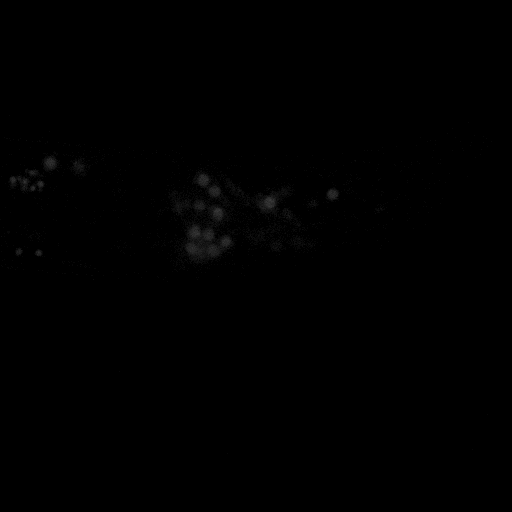

Supplement: Supplementary file 5 — Source data Fig. 4 [file 44319_2025_493_MOESM5_ESM.zip › Figure4/Fig4G/Experiment-645wildtype_NR.czi.tif_files/Experiment-645.czi_h0b0t0z15c1x0-512y0-512.tif]

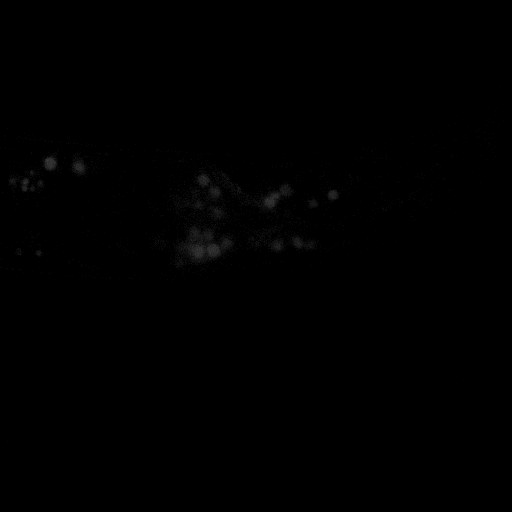

Supplement: Supplementary file 5 — Source data Fig. 4 [file 44319_2025_493_MOESM5_ESM.zip › Figure4/Fig4G/Experiment-645wildtype_NR.czi.tif_files/Experiment-645.czi_h0b0t0z14c1x0-512y0-512.tif]

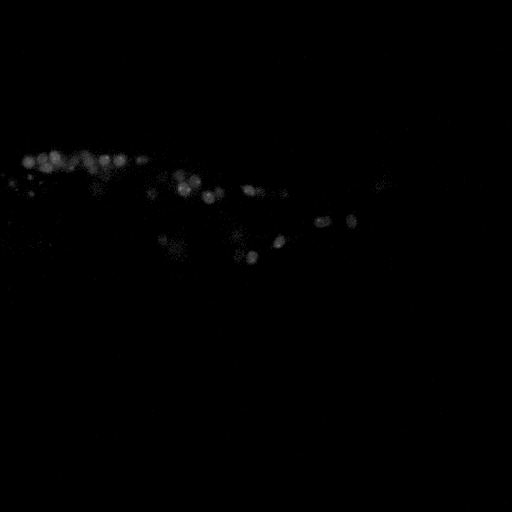

Supplement: Supplementary file 5 — Source data Fig. 4 [file 44319_2025_493_MOESM5_ESM.zip › Figure4/Fig4G/Experiment-645wildtype_NR.czi.tif_files/Experiment-645.czi_h0b0t0z9c0x0-512y0-512.tif]

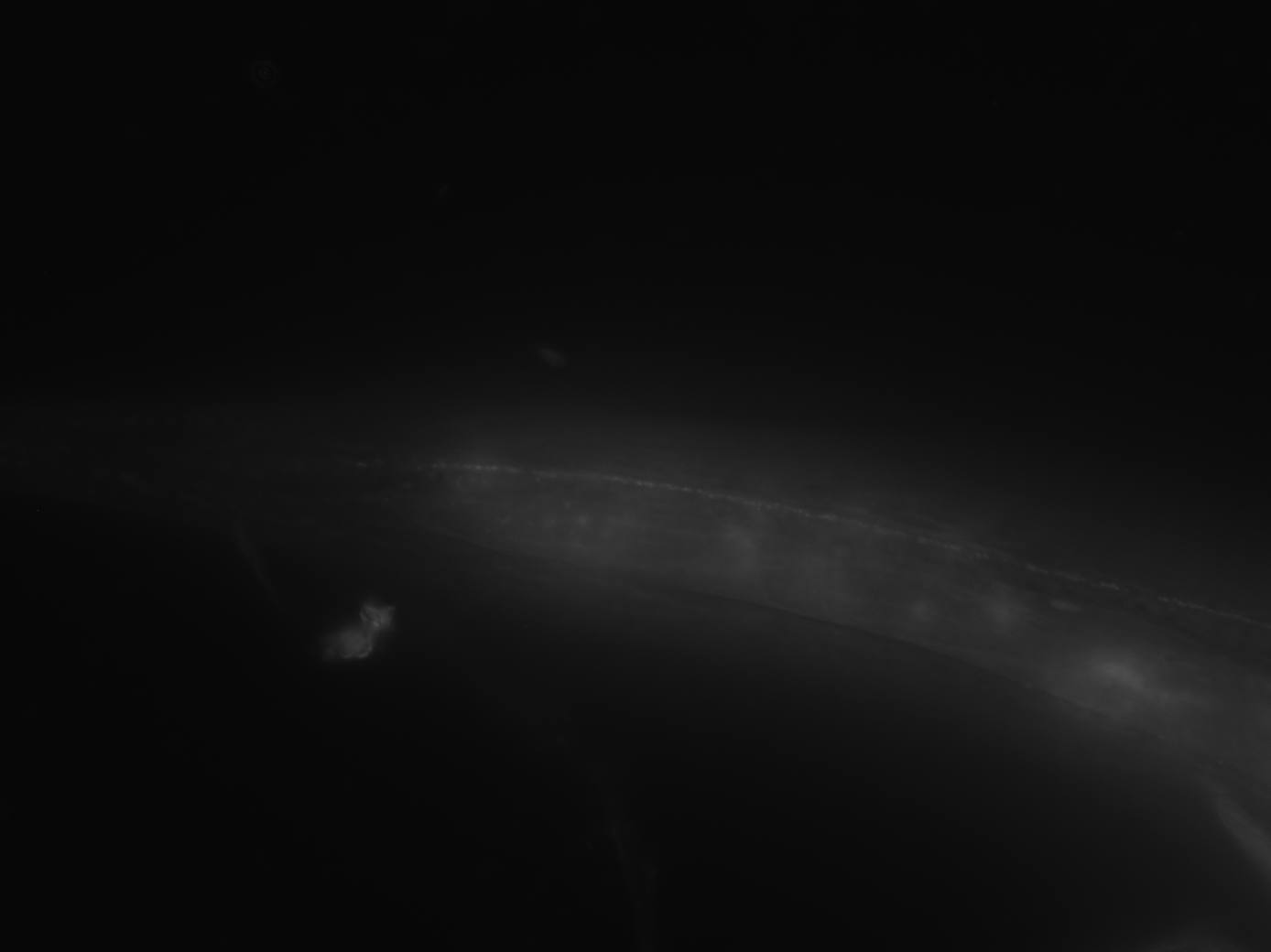

Supplement: Supplementary file 5 — Source data Fig. 4 [file 44319_2025_493_MOESM5_ESM.zip › Figure4/Fig4D/Experiment-106_skipped.tif_files/Experiment-106_z3c0x0-1388y0-1040.tif]

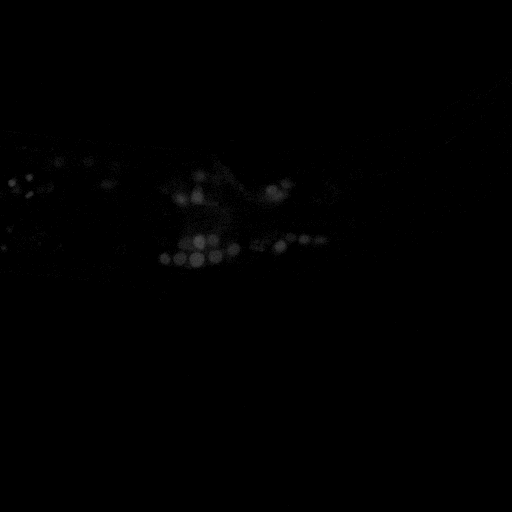

Supplement: Supplementary file 5 — Source data Fig. 4 [file 44319_2025_493_MOESM5_ESM.zip › Figure4/Fig4G/Experiment-645wildtype_NR.czi.tif_files/Experiment-645.czi_h0b0t0z8c1x0-512y0-512.tif]

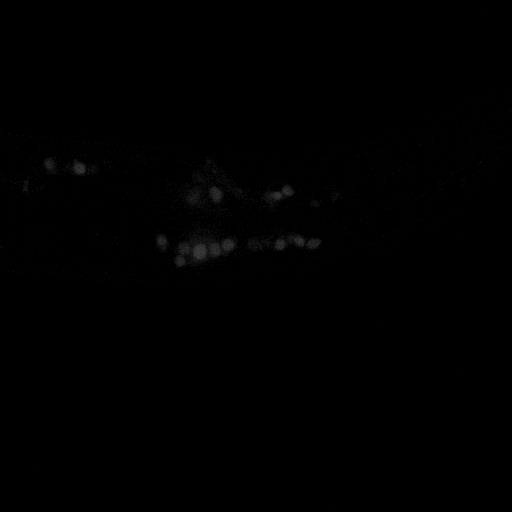

Supplement: Supplementary file 5 — Source data Fig. 4 [file 44319_2025_493_MOESM5_ESM.zip › Figure4/Fig4G/Experiment-645wildtype_NR.czi.tif_files/Experiment-645.czi_h0b0t0z12c1x0-512y0-512.tif]

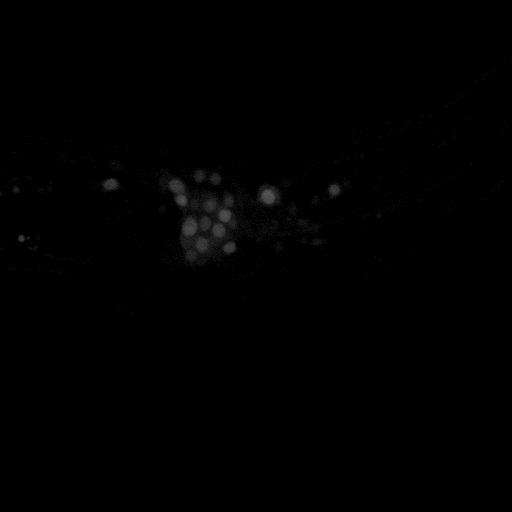

Supplement: Supplementary file 5 — Source data Fig. 4 [file 44319_2025_493_MOESM5_ESM.zip › Figure4/Fig4G/Experiment-645wildtype_NR.czi.tif_files/Experiment-645.czi_h0b0t0z5c1x0-512y0-512.tif]

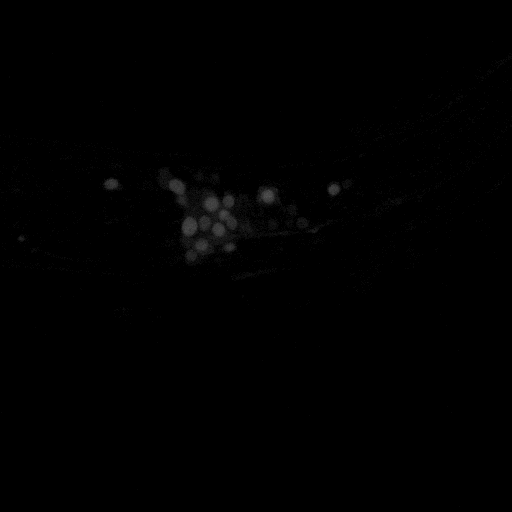

Supplement: Supplementary file 5 — Source data Fig. 4 [file 44319_2025_493_MOESM5_ESM.zip › Figure4/Fig4G/Experiment-645wildtype_NR.czi.tif_files/Experiment-645.czi_h0b0t0z4c1x0-512y0-512.tif]

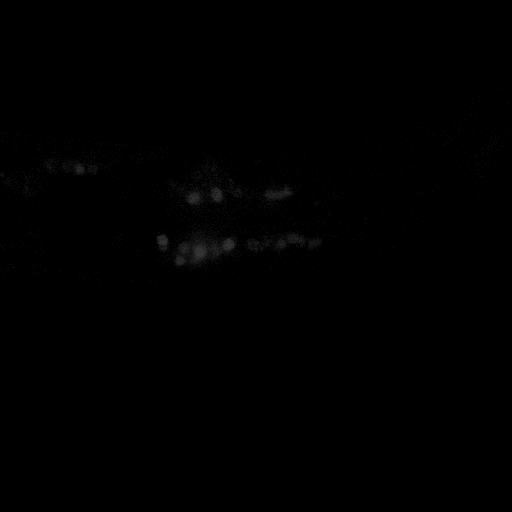

Supplement: Supplementary file 5 — Source data Fig. 4 [file 44319_2025_493_MOESM5_ESM.zip › Figure4/Fig4G/Experiment-645wildtype_NR.czi.tif_files/Experiment-645.czi_h0b0t0z11c1x0-512y0-512.tif]

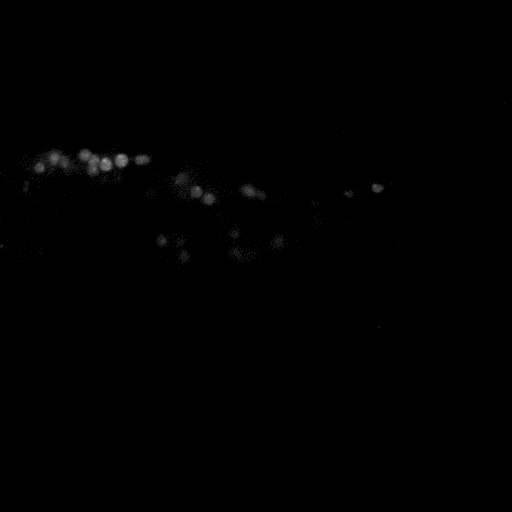

Supplement: Supplementary file 5 — Source data Fig. 4 [file 44319_2025_493_MOESM5_ESM.zip › Figure4/Fig4G/Experiment-645wildtype_NR.czi.tif_files/Experiment-645.czi_h0b0t0z12c0x0-512y0-512.tif]

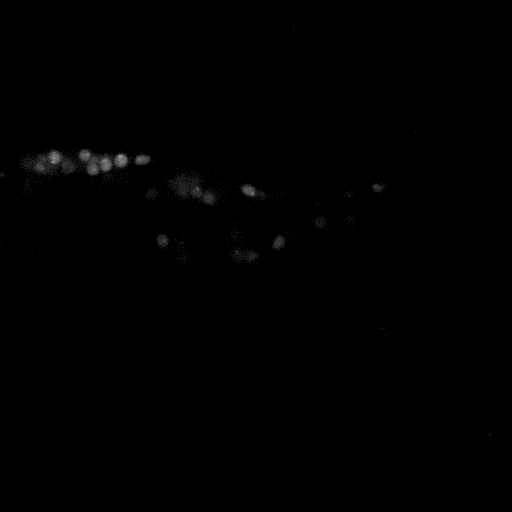

Supplement: Supplementary file 5 — Source data Fig. 4 [file 44319_2025_493_MOESM5_ESM.zip › Figure4/Fig4G/Experiment-645wildtype_NR.czi.tif_files/Experiment-645.czi_h0b0t0z11c0x0-512y0-512.tif]

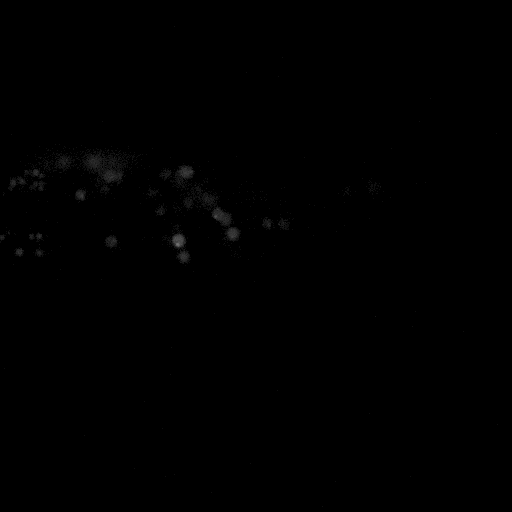

Supplement: Supplementary file 5 — Source data Fig. 4 [file 44319_2025_493_MOESM5_ESM.zip › Figure4/Fig4G/Experiment-645wildtype_NR.czi.tif_files/Experiment-645.czi_h0b0t0z16c0x0-512y0-512.tif]

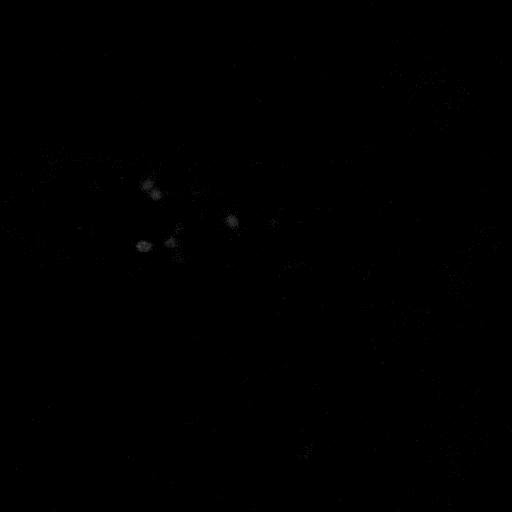

Supplement: Supplementary file 5 — Source data Fig. 4 [file 44319_2025_493_MOESM5_ESM.zip › Figure4/Fig4G/Experiment-645wildtype_NR.czi.tif_files/Experiment-645.czi_h0b0t0z1c0x0-512y0-512.tif]

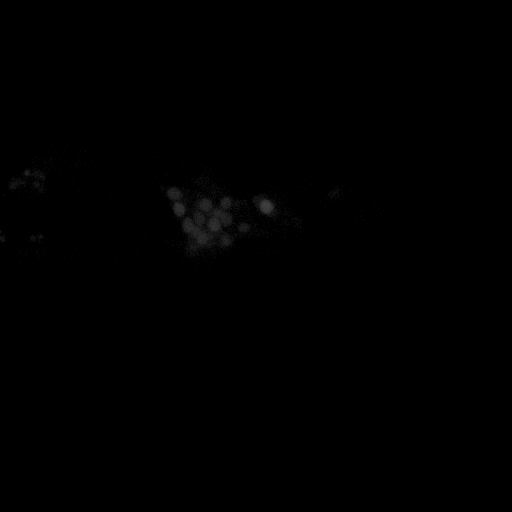

Supplement: Supplementary file 5 — Source data Fig. 4 [file 44319_2025_493_MOESM5_ESM.zip › Figure4/Fig4G/Experiment-645wildtype_NR.czi.tif_files/Experiment-645.czi_h0b0t0z18c1x0-512y0-512.tif]

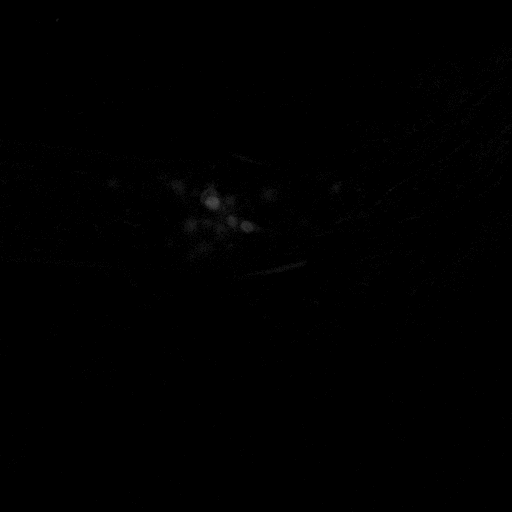

Supplement: Supplementary file 5 — Source data Fig. 4 [file 44319_2025_493_MOESM5_ESM.zip › Figure4/Fig4G/Experiment-645wildtype_NR.czi.tif_files/Experiment-645.czi_h0b0t0z2c1x0-512y0-512.tif]

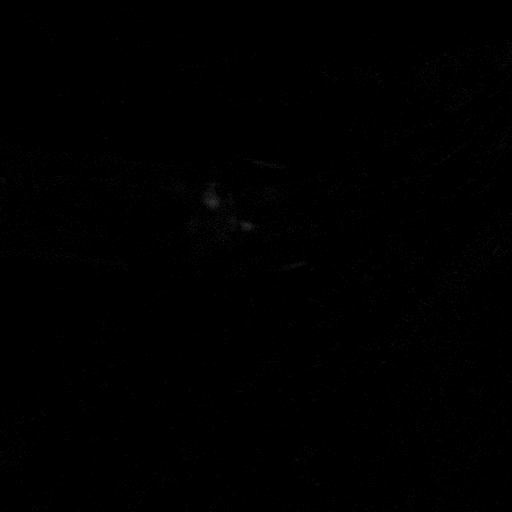

Supplement: Supplementary file 5 — Source data Fig. 4 [file 44319_2025_493_MOESM5_ESM.zip › Figure4/Fig4G/Experiment-645wildtype_NR.czi.tif_files/Experiment-645.czi_h0b0t0z1c1x0-512y0-512.tif]

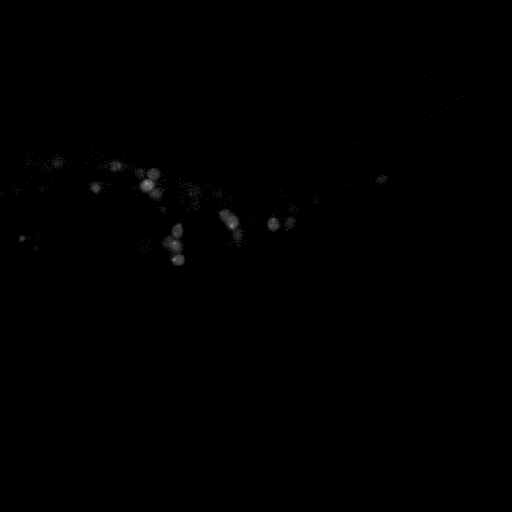

Supplement: Supplementary file 5 — Source data Fig. 4 [file 44319_2025_493_MOESM5_ESM.zip › Figure4/Fig4G/Experiment-645wildtype_NR.czi.tif_files/Experiment-645.czi_h0b0t0z4c0x0-512y0-512.tif]

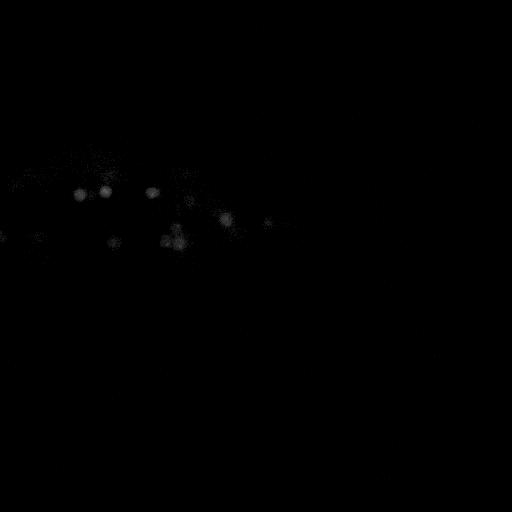

Supplement: Supplementary file 5 — Source data Fig. 4 [file 44319_2025_493_MOESM5_ESM.zip › Figure4/Fig4G/Experiment-645wildtype_NR.czi.tif_files/Experiment-645.czi_h0b0t0z19c0x0-512y0-512.tif]

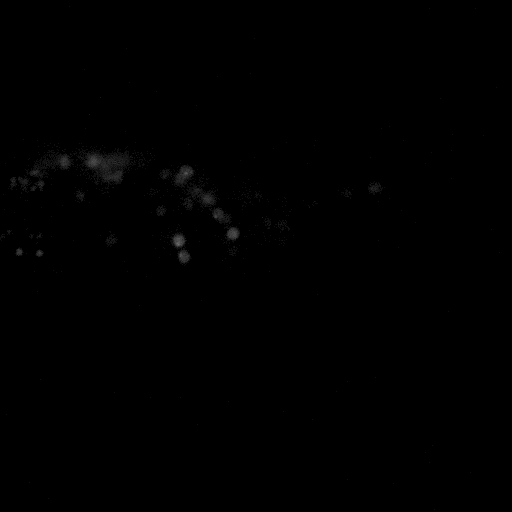

Supplement: Supplementary file 5 — Source data Fig. 4 [file 44319_2025_493_MOESM5_ESM.zip › Figure4/Fig4G/Experiment-645wildtype_NR.czi.tif_files/Experiment-645.czi_h0b0t0z15c0x0-512y0-512.tif]

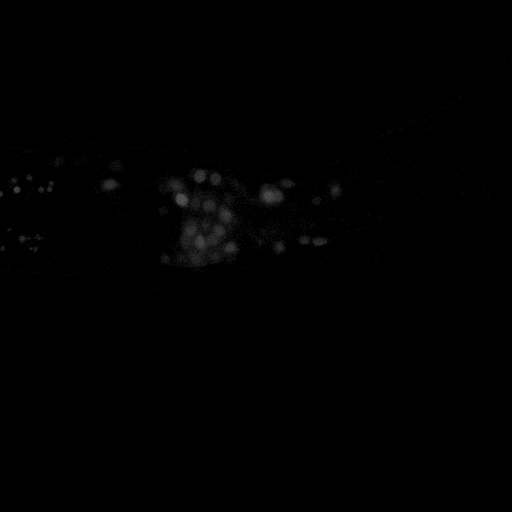

Supplement: Supplementary file 5 — Source data Fig. 4 [file 44319_2025_493_MOESM5_ESM.zip › Figure4/Fig4G/Experiment-645wildtype_NR.czi.tif_files/Experiment-645.czi_h0b0t0z6c1x0-512y0-512.tif]

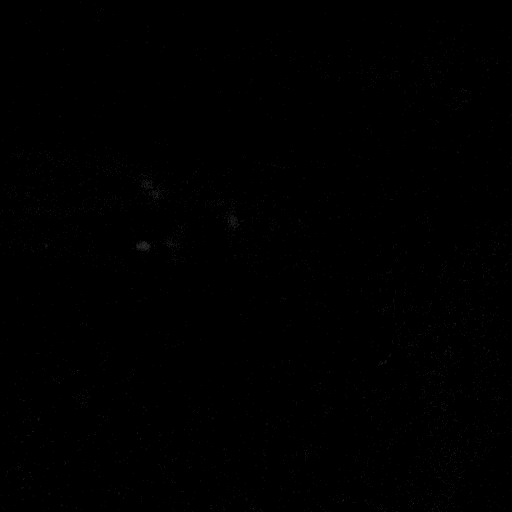

Supplement: Supplementary file 5 — Source data Fig. 4 [file 44319_2025_493_MOESM5_ESM.zip › Figure4/Fig4G/Experiment-645wildtype_NR.czi.tif_files/Experiment-645.czi_h0b0t0z0c0x0-512y0-512.tif]

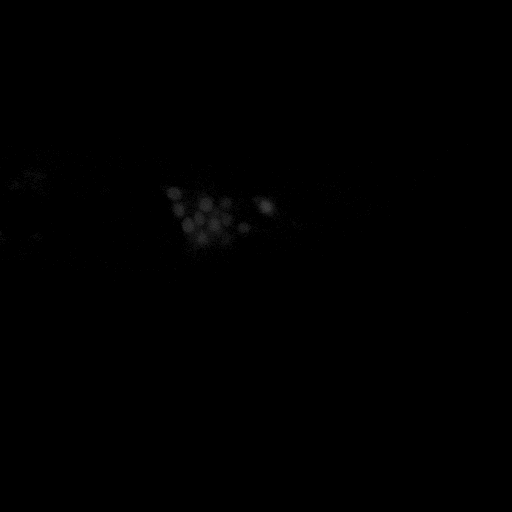

Supplement: Supplementary file 5 — Source data Fig. 4 [file 44319_2025_493_MOESM5_ESM.zip › Figure4/Fig4G/Experiment-645wildtype_NR.czi.tif_files/Experiment-645.czi_h0b0t0z19c1x0-512y0-512.tif]

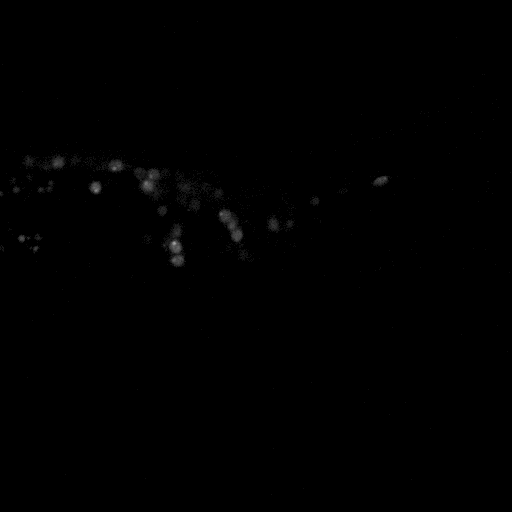

Supplement: Supplementary file 5 — Source data Fig. 4 [file 44319_2025_493_MOESM5_ESM.zip › Figure4/Fig4G/Experiment-645wildtype_NR.czi.tif_files/Experiment-645.czi_h0b0t0z5c0x0-512y0-512.tif]

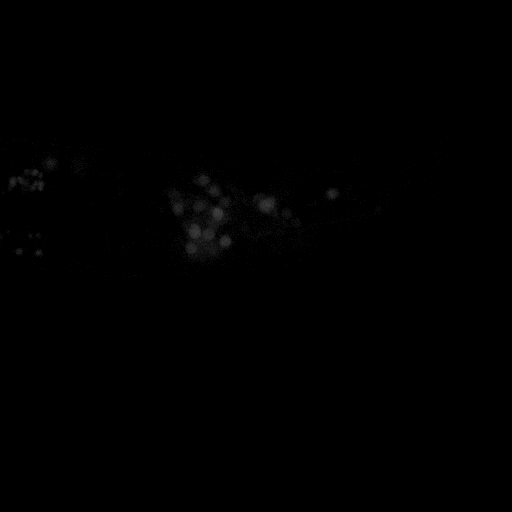

Supplement: Supplementary file 5 — Source data Fig. 4 [file 44319_2025_493_MOESM5_ESM.zip › Figure4/Fig4G/Experiment-645wildtype_NR.czi.tif_files/Experiment-645.czi_h0b0t0z16c1x0-512y0-512.tif]

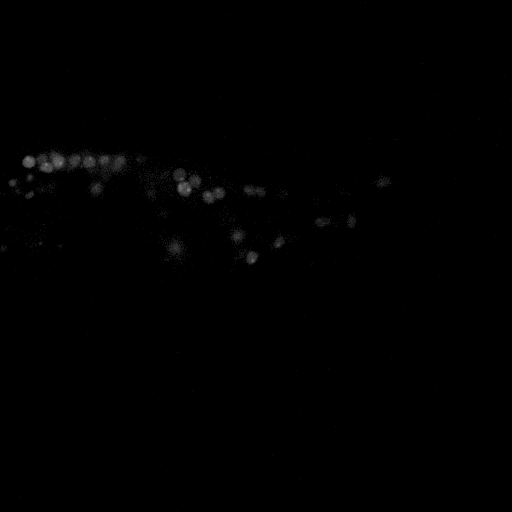

Supplement: Supplementary file 5 — Source data Fig. 4 [file 44319_2025_493_MOESM5_ESM.zip › Figure4/Fig4G/Experiment-645wildtype_NR.czi.tif_files/Experiment-645.czi_h0b0t0z8c0x0-512y0-512.tif]

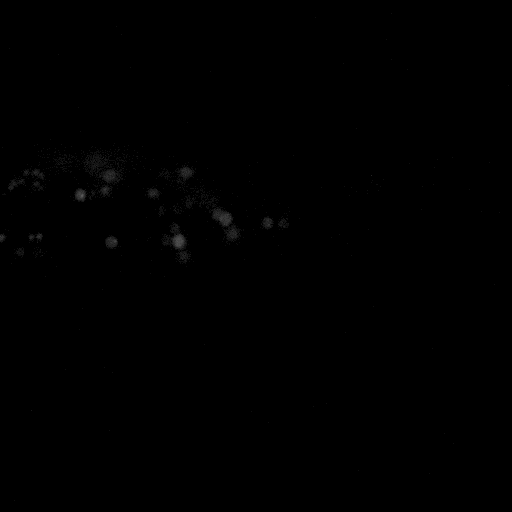

Supplement: Supplementary file 5 — Source data Fig. 4 [file 44319_2025_493_MOESM5_ESM.zip › Figure4/Fig4G/Experiment-645wildtype_NR.czi.tif_files/Experiment-645.czi_h0b0t0z17c0x0-512y0-512.tif]

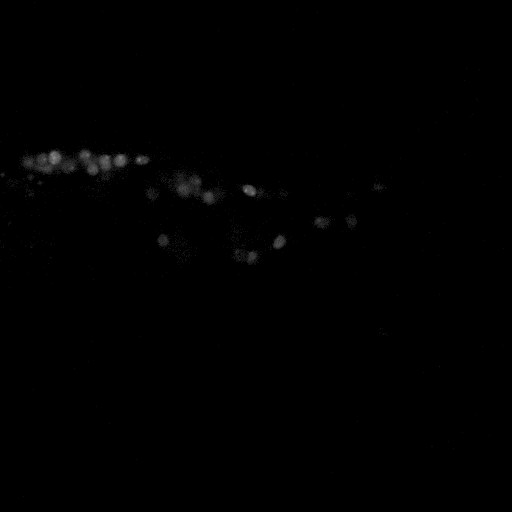

Supplement: Supplementary file 5 — Source data Fig. 4 [file 44319_2025_493_MOESM5_ESM.zip › Figure4/Fig4G/Experiment-645wildtype_NR.czi.tif_files/Experiment-645.czi_h0b0t0z10c0x0-512y0-512.tif]

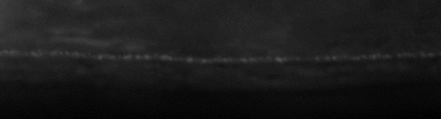

Supplement: Supplementary file 5 — Source data Fig. 4 [file 44319_2025_493_MOESM5_ESM.zip › Figure4/Fig4D/Experiment-122_included.tif_files/processed/includedprocessed.tif]

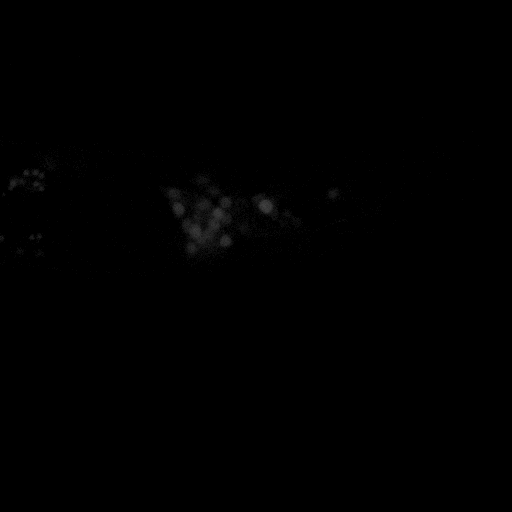

Supplement: Supplementary file 5 — Source data Fig. 4 [file 44319_2025_493_MOESM5_ESM.zip › Figure4/Fig4G/Experiment-645wildtype_NR.czi.tif_files/Experiment-645.czi_h0b0t0z17c1x0-512y0-512.tif]

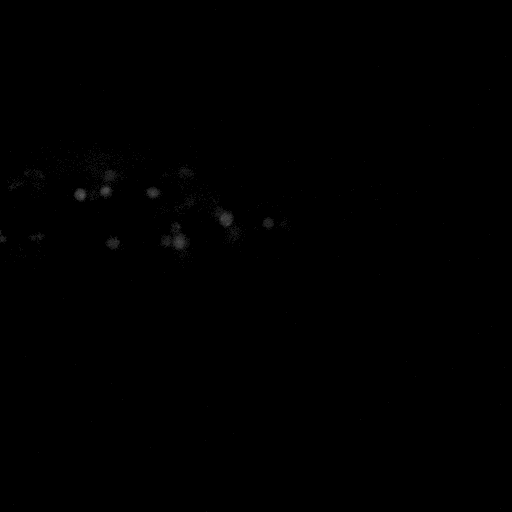

Supplement: Supplementary file 5 — Source data Fig. 4 [file 44319_2025_493_MOESM5_ESM.zip › Figure4/Fig4G/Experiment-645wildtype_NR.czi.tif_files/Experiment-645.czi_h0b0t0z18c0x0-512y0-512.tif]

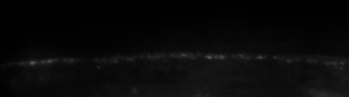

Supplement: Supplementary file 5 — Source data Fig. 4 [file 44319_2025_493_MOESM5_ESM.zip › Figure4/Fig4D/Experiment-106_skipped.tif_files/processed/UNC13skippedprocessed1.tif]

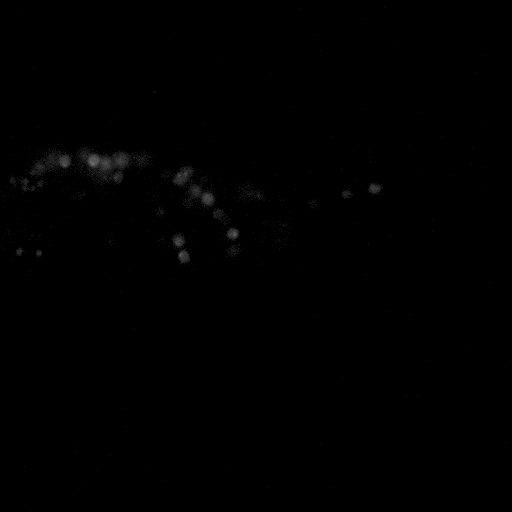

Supplement: Supplementary file 5 — Source data Fig. 4 [file 44319_2025_493_MOESM5_ESM.zip › Figure4/Fig4G/Experiment-645wildtype_NR.czi.tif_files/Experiment-645.czi_h0b0t0z14c0x0-512y0-512.tif]

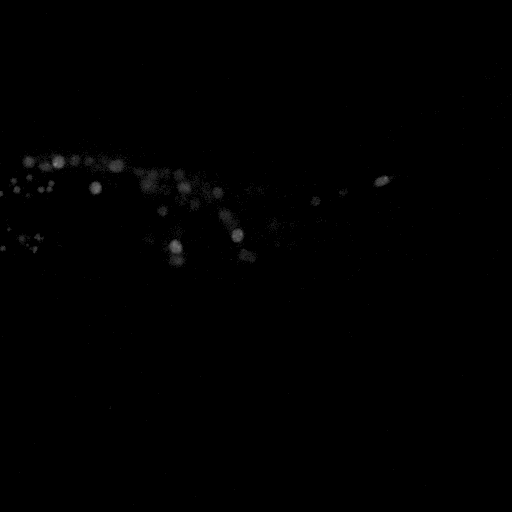

Supplement: Supplementary file 5 — Source data Fig. 4 [file 44319_2025_493_MOESM5_ESM.zip › Figure4/Fig4G/Experiment-645wildtype_NR.czi.tif_files/Experiment-645.czi_h0b0t0z6c0x0-512y0-512.tif]

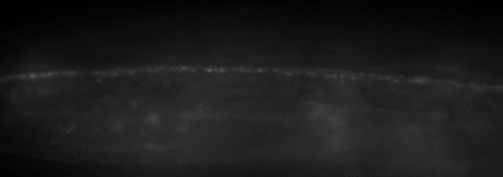

Supplement: Supplementary file 5 — Source data Fig. 4 [file 44319_2025_493_MOESM5_ESM.zip › Figure4/Fig4D/Experiment-106_skipped.tif_files/processed/UNC13skippedprocessed.tif]

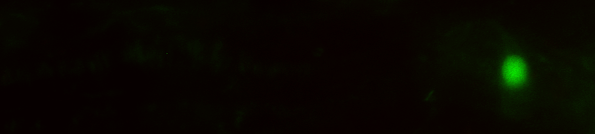

Supplement: Supplementary file 5 — Source data Fig. 4 [file 44319_2025_493_MOESM5_ESM.zip › Figure4/Fig4G/Experiment-62gooddup_AWA.tif_files/processed/processed.tif]

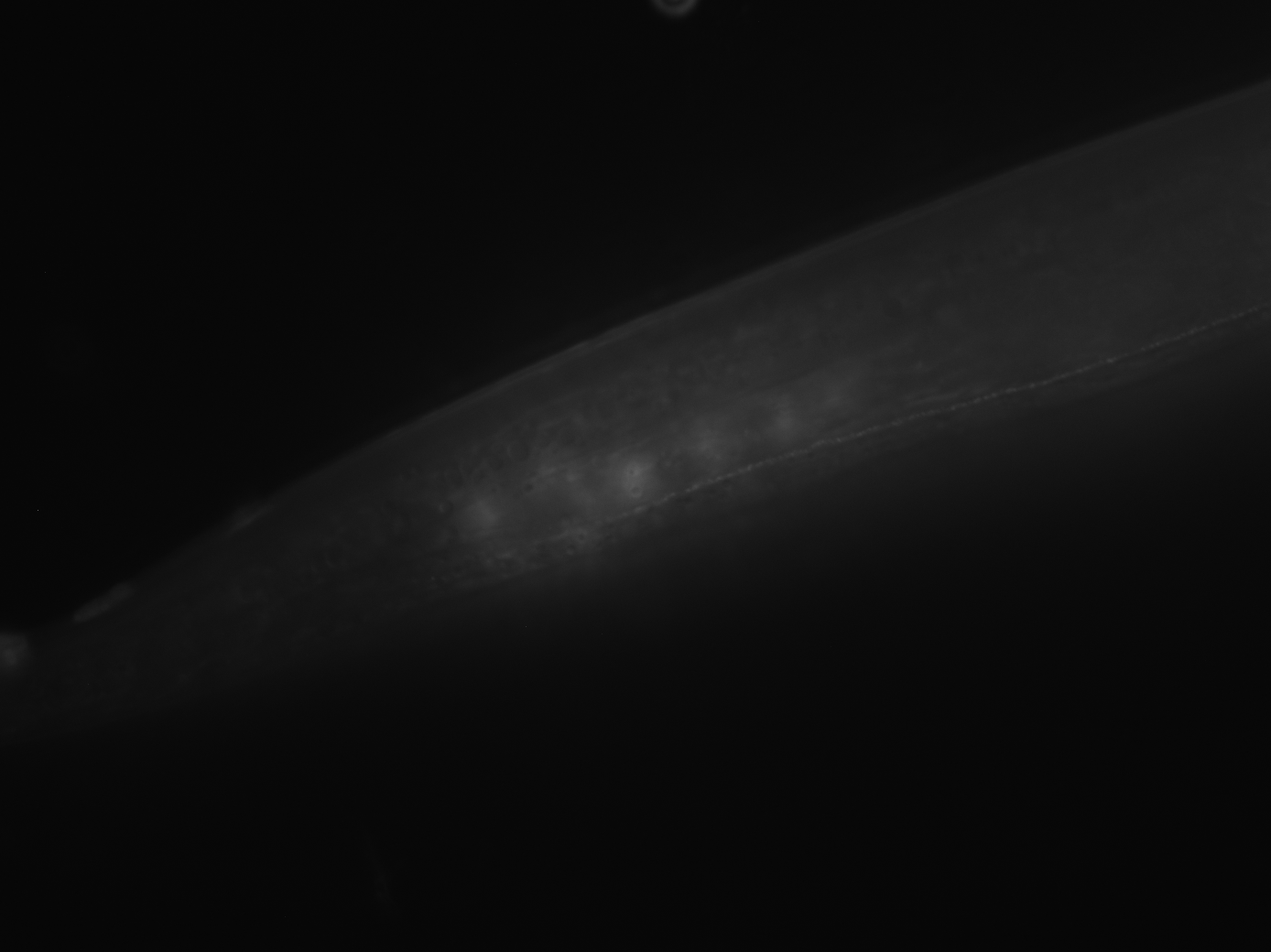

Supplement: Supplementary file 5 — Source data Fig. 4 [file 44319_2025_493_MOESM5_ESM.zip › Figure4/Fig4D/Experiment-122_included.tif_files/processed/included.tif]

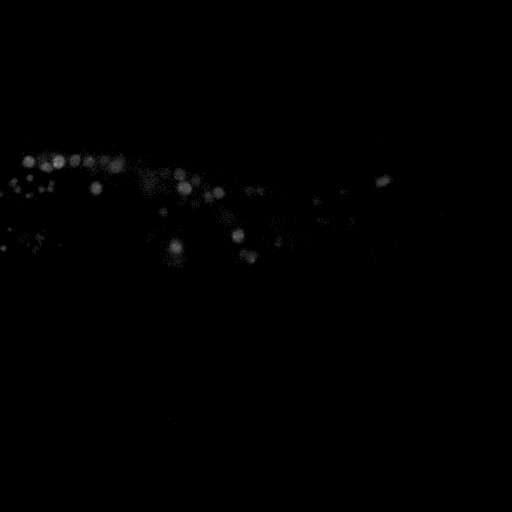

Supplement: Supplementary file 5 — Source data Fig. 4 [file 44319_2025_493_MOESM5_ESM.zip › Figure4/Fig4G/Experiment-645wildtype_NR.czi.tif_files/Experiment-645.czi_h0b0t0z7c0x0-512y0-512.tif]

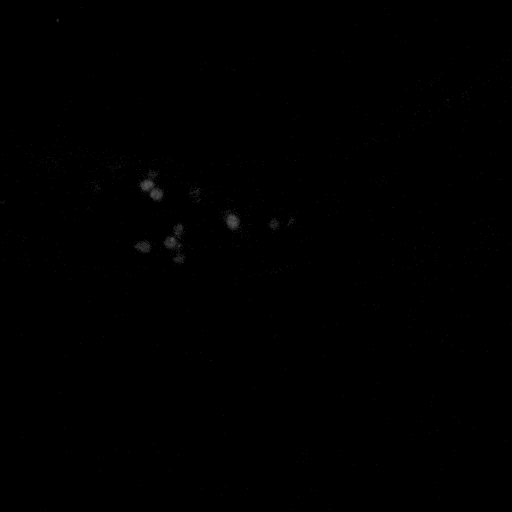

Supplement: Supplementary file 5 — Source data Fig. 4 [file 44319_2025_493_MOESM5_ESM.zip › Figure4/Fig4G/Experiment-645wildtype_NR.czi.tif_files/Experiment-645.czi_h0b0t0z2c0x0-512y0-512.tif]

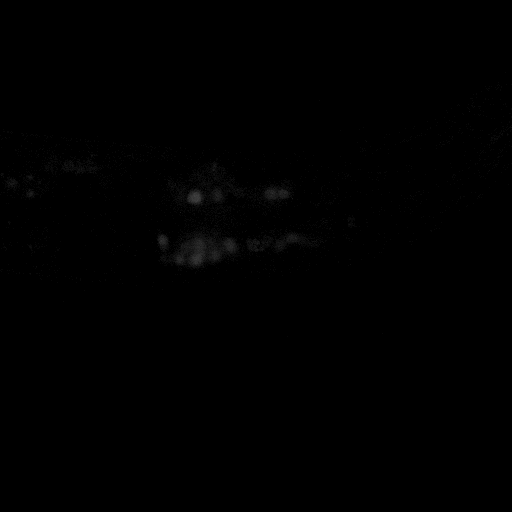

Supplement: Supplementary file 5 — Source data Fig. 4 [file 44319_2025_493_MOESM5_ESM.zip › Figure4/Fig4G/Experiment-645wildtype_NR.czi.tif_files/Experiment-645.czi_h0b0t0z10c1x0-512y0-512.tif]

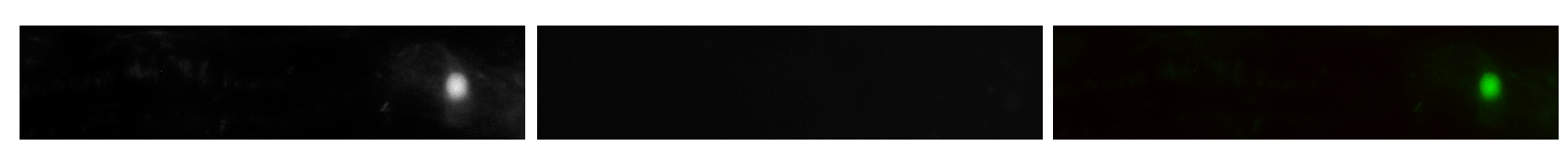

Supplement: Supplementary file 5 — Source data Fig. 4 [file 44319_2025_493_MOESM5_ESM.zip › Figure4/Fig4G/Experiment-62gooddup_AWA.tif_files/processed/processed_compile.tif]

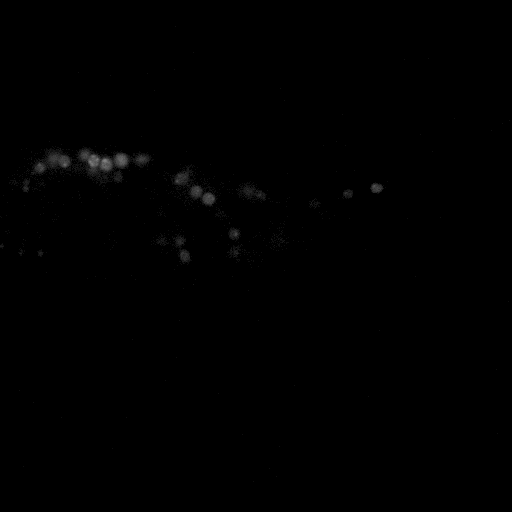

Supplement: Supplementary file 5 — Source data Fig. 4 [file 44319_2025_493_MOESM5_ESM.zip › Figure4/Fig4G/Experiment-645wildtype_NR.czi.tif_files/Experiment-645.czi_h0b0t0z13c0x0-512y0-512.tif]

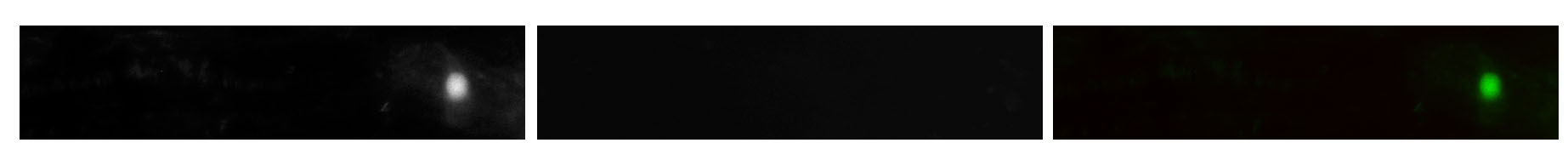

Supplement: Supplementary file 5 — Source data Fig. 4 [file 44319_2025_493_MOESM5_ESM.zip › Figure4/Fig4G/Experiment-62gooddup_AWA.tif_files/processed/processed_compile.jpg]
