# Supplementary figures and images for: Regulated microexon alternative splicing in single neurons tunes synaptic function (part 4 of 6)
Source: EMBO Rep. 2025 Jun 9;26(14):3640–62. doi: 10.1038/s44319-025-00493-7 (PMC12287369; doi:10.1038/s44319-025-00493-7)

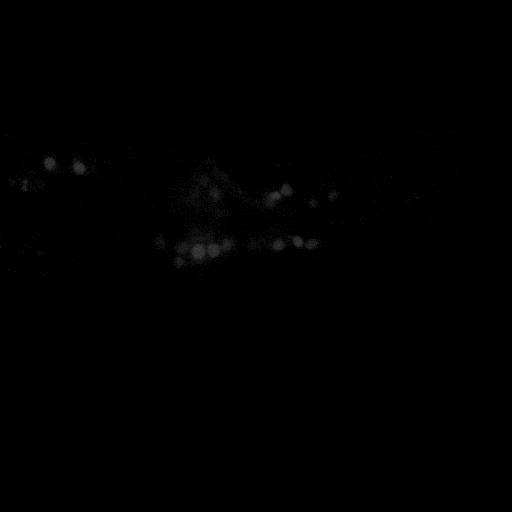

Supplement: Supplementary file 5 — Source data Fig. 4 [file 44319_2025_493_MOESM5_ESM.zip › Figure4/Fig4G/Experiment-645wildtype_NR.czi.tif_files/Experiment-645.czi_h0b0t0z13c1x0-512y0-512.tif]

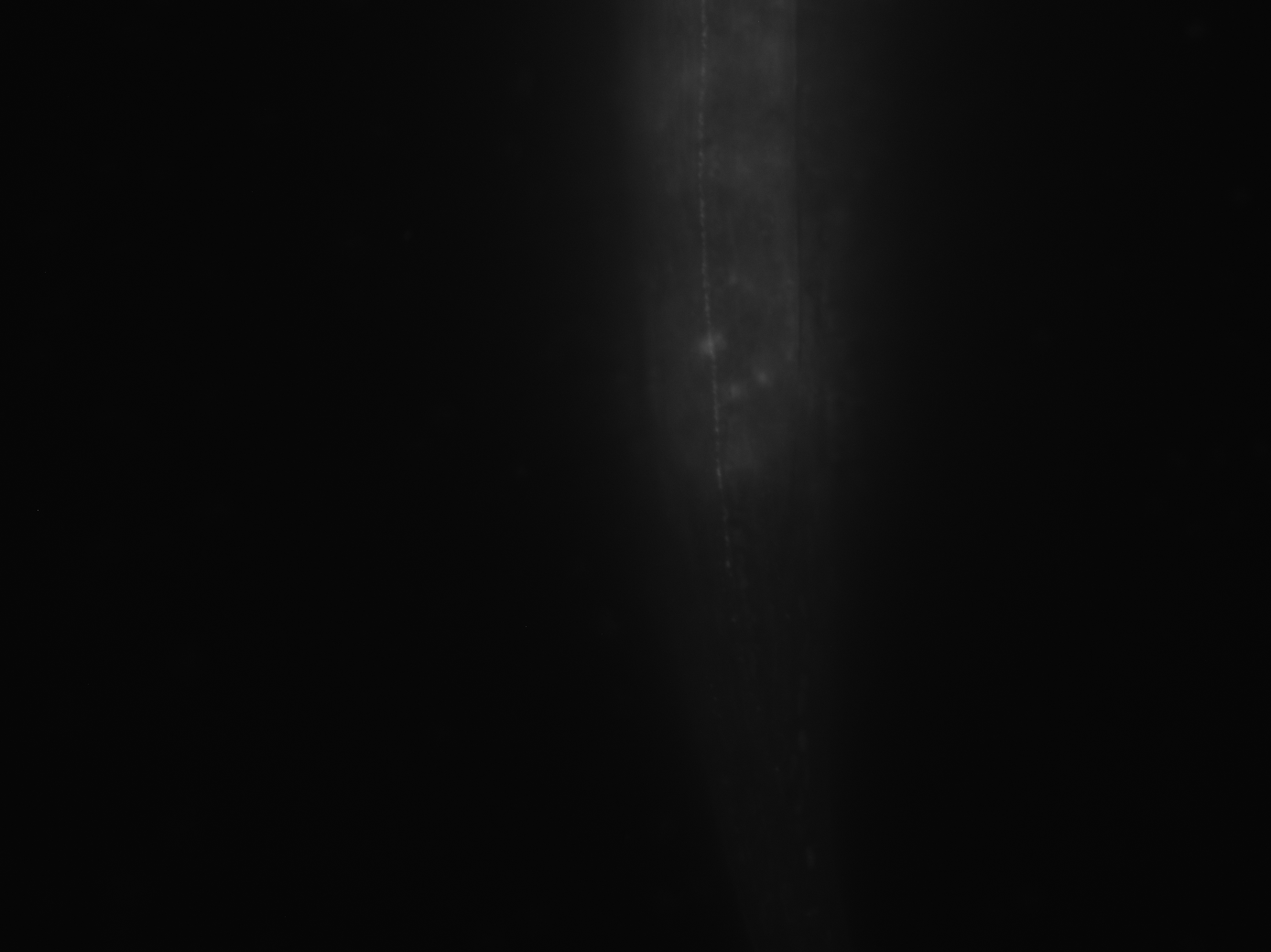

Supplement: Supplementary file 5 — Source data Fig. 4 [file 44319_2025_493_MOESM5_ESM.zip › Figure4/Fig4D/Experiment-96_wildtype.tif_files/processed/wt.tif]

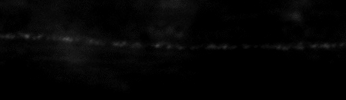

Supplement: Supplementary file 5 — Source data Fig. 4 [file 44319_2025_493_MOESM5_ESM.zip › Figure4/Fig4D/Experiment-96_wildtype.tif_files/processed/wtprocessed.tif]

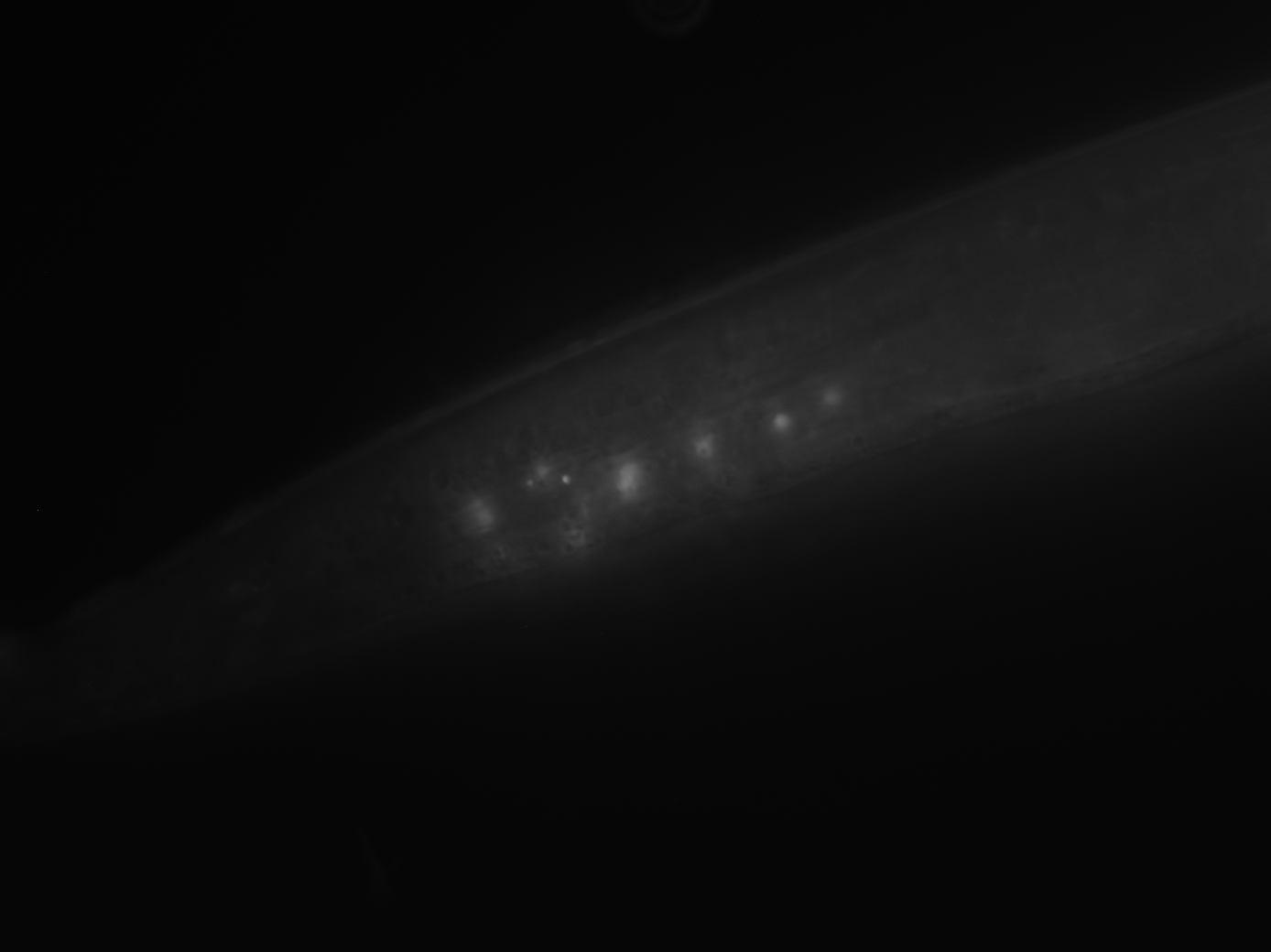

Supplement: Supplementary file 5 — Source data Fig. 4 [file 44319_2025_493_MOESM5_ESM.zip › Figure4/Fig4D/Experiment-122_included.tif_files/Experiment-122_z5c0x0-1388y0-1040.tif]

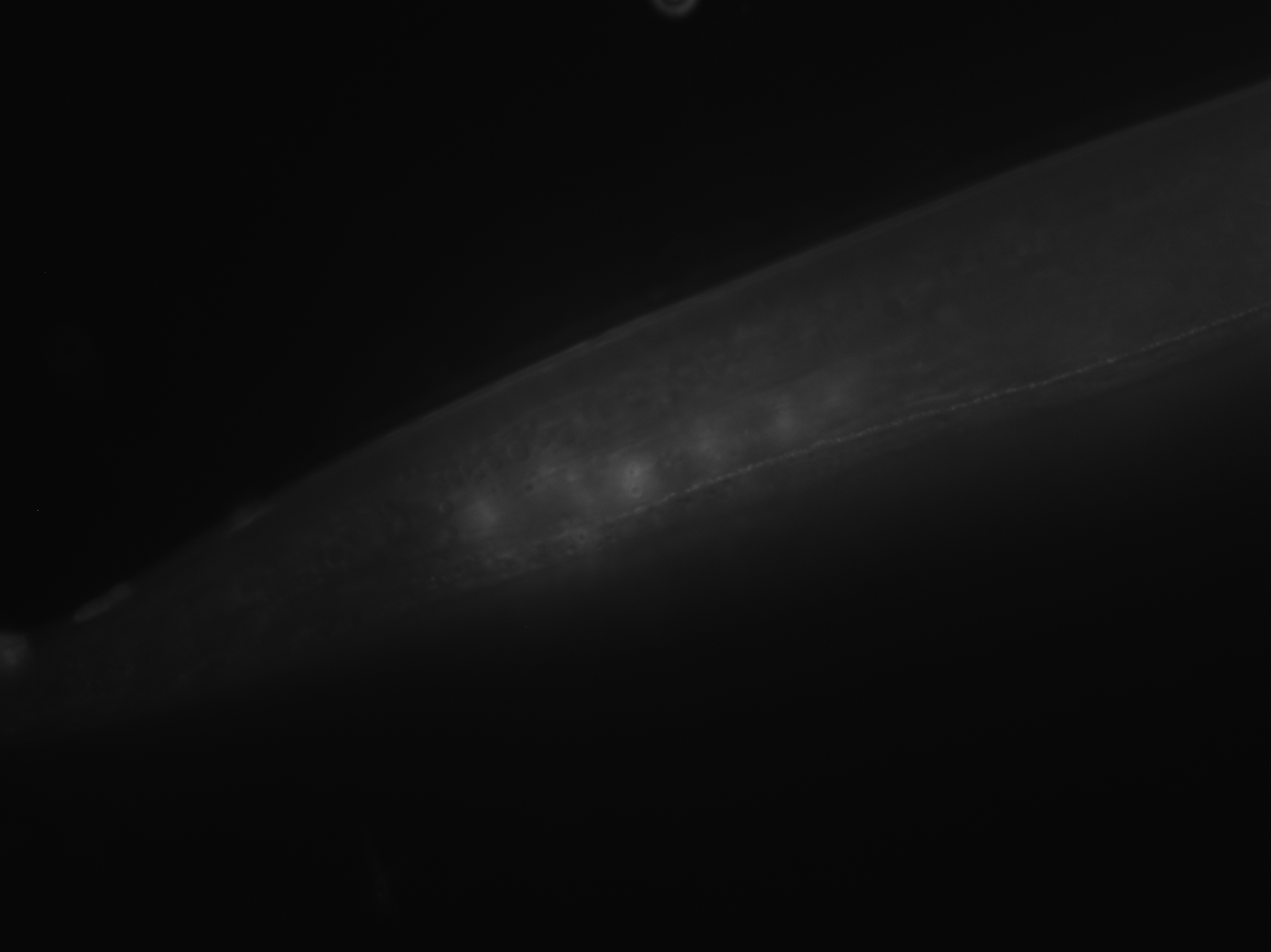

Supplement: Supplementary file 5 — Source data Fig. 4 [file 44319_2025_493_MOESM5_ESM.zip › Figure4/Fig4D/Experiment-122_included.tif_files/Experiment-122_z3c0x0-1388y0-1040.tif]

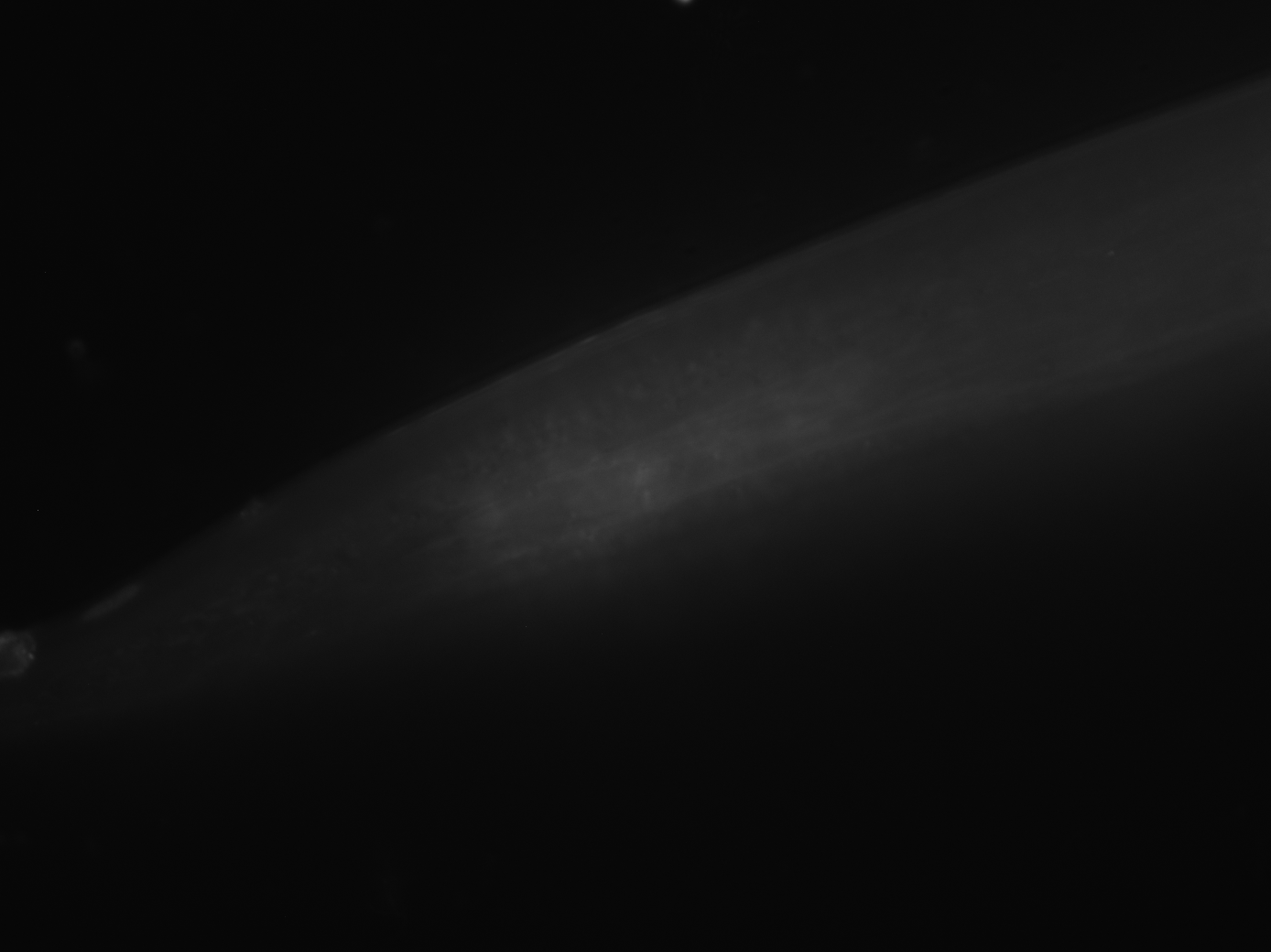

Supplement: Supplementary file 5 — Source data Fig. 4 [file 44319_2025_493_MOESM5_ESM.zip › Figure4/Fig4D/Experiment-122_included.tif_files/Experiment-122_z1c0x0-1388y0-1040.tif]

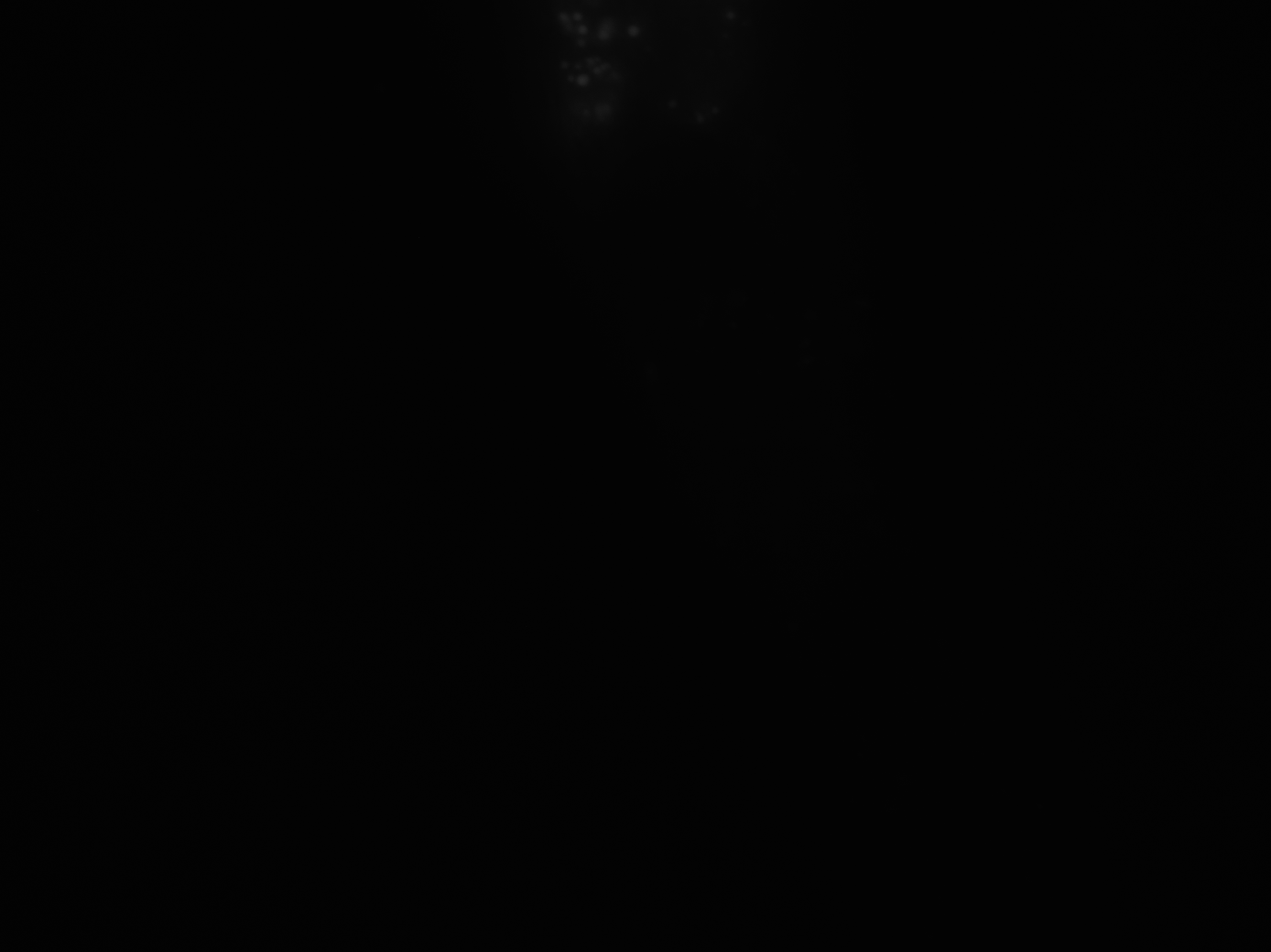

Supplement: Supplementary file 5 — Source data Fig. 4 [file 44319_2025_493_MOESM5_ESM.zip › Figure4/Fig4G/Experiment-62gooddup_AWA.tif_files/processed/MAX_Experiment-62gooddupred.tif_files-1.tif]

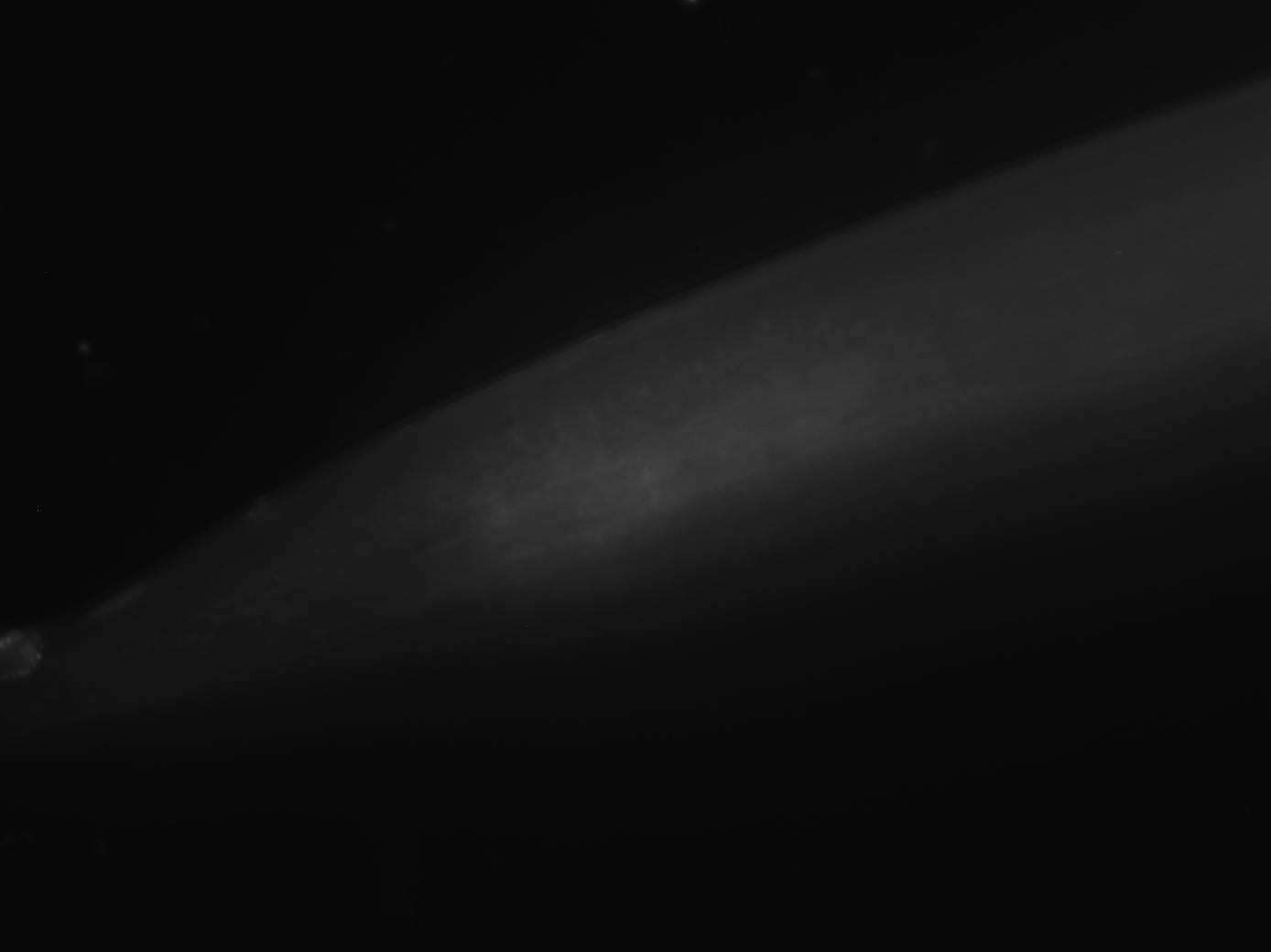

Supplement: Supplementary file 5 — Source data Fig. 4 [file 44319_2025_493_MOESM5_ESM.zip › Figure4/Fig4D/Experiment-122_included.tif_files/Experiment-122_z0c0x0-1388y0-1040.tif]

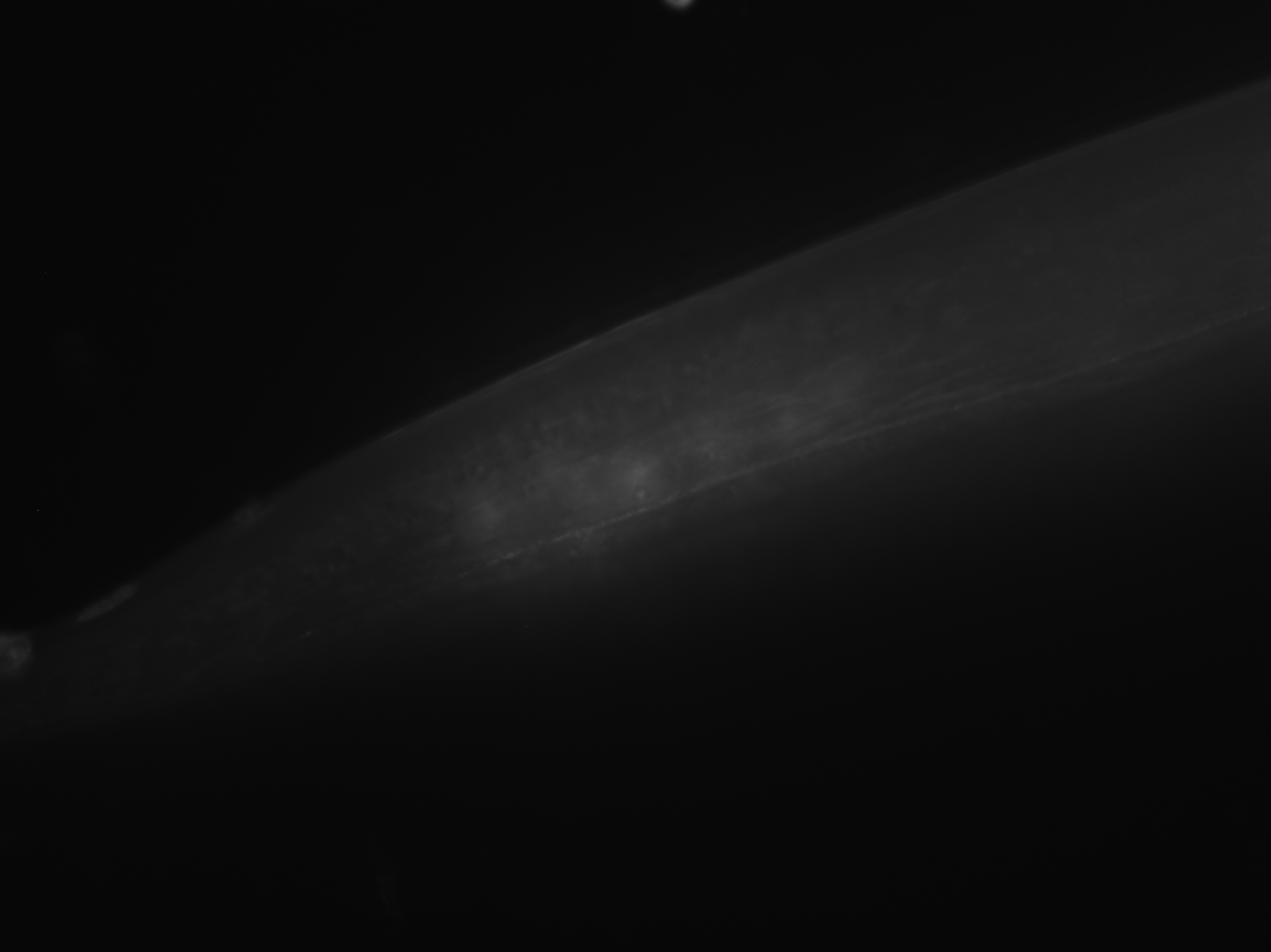

Supplement: Supplementary file 5 — Source data Fig. 4 [file 44319_2025_493_MOESM5_ESM.zip › Figure4/Fig4D/Experiment-122_included.tif_files/Experiment-122_z2c0x0-1388y0-1040.tif]

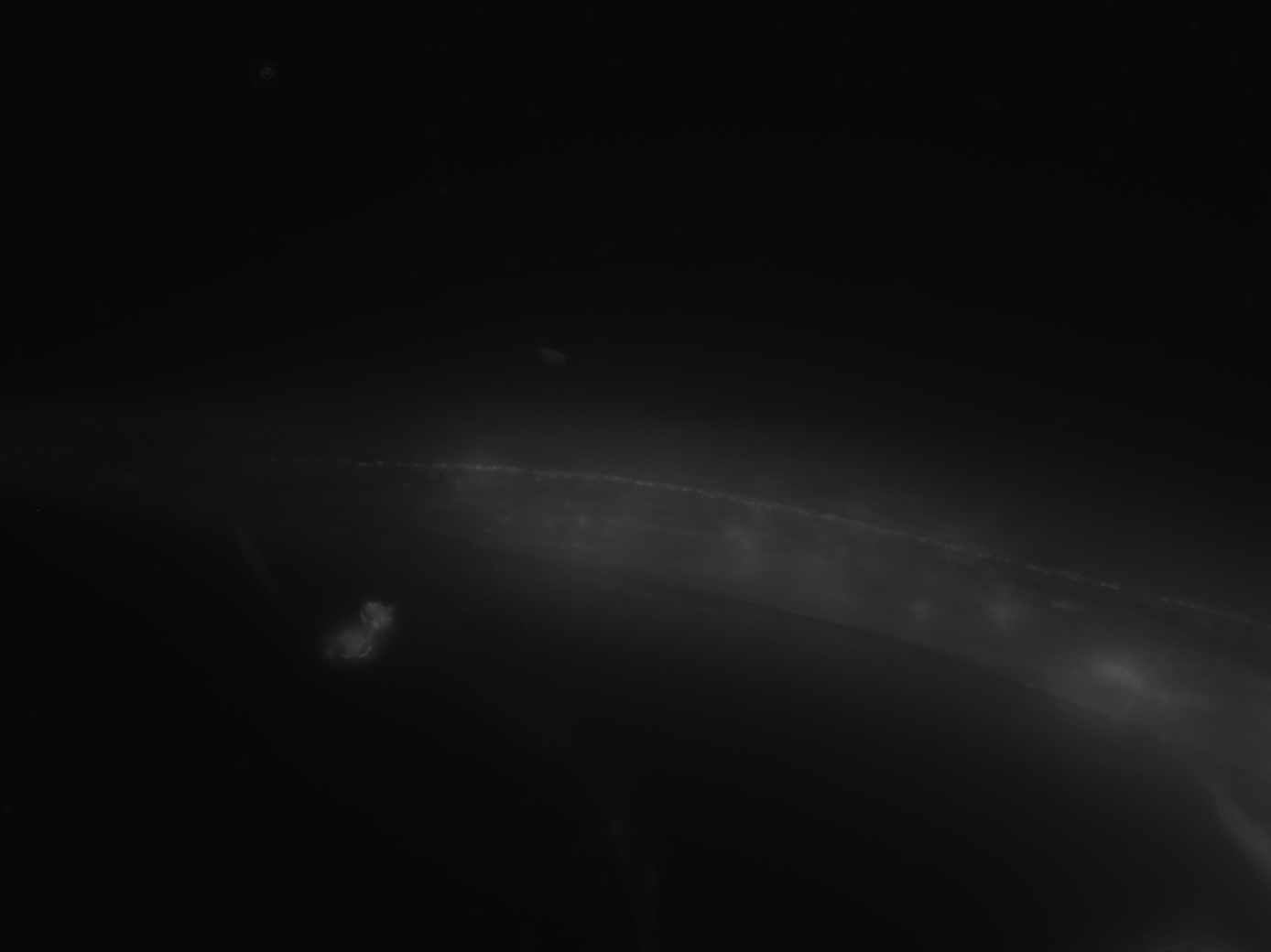

Supplement: Supplementary file 5 — Source data Fig. 4 [file 44319_2025_493_MOESM5_ESM.zip › Figure4/Fig4D/Experiment-106_skipped.tif_files/processed/UNC13skipped.tif]

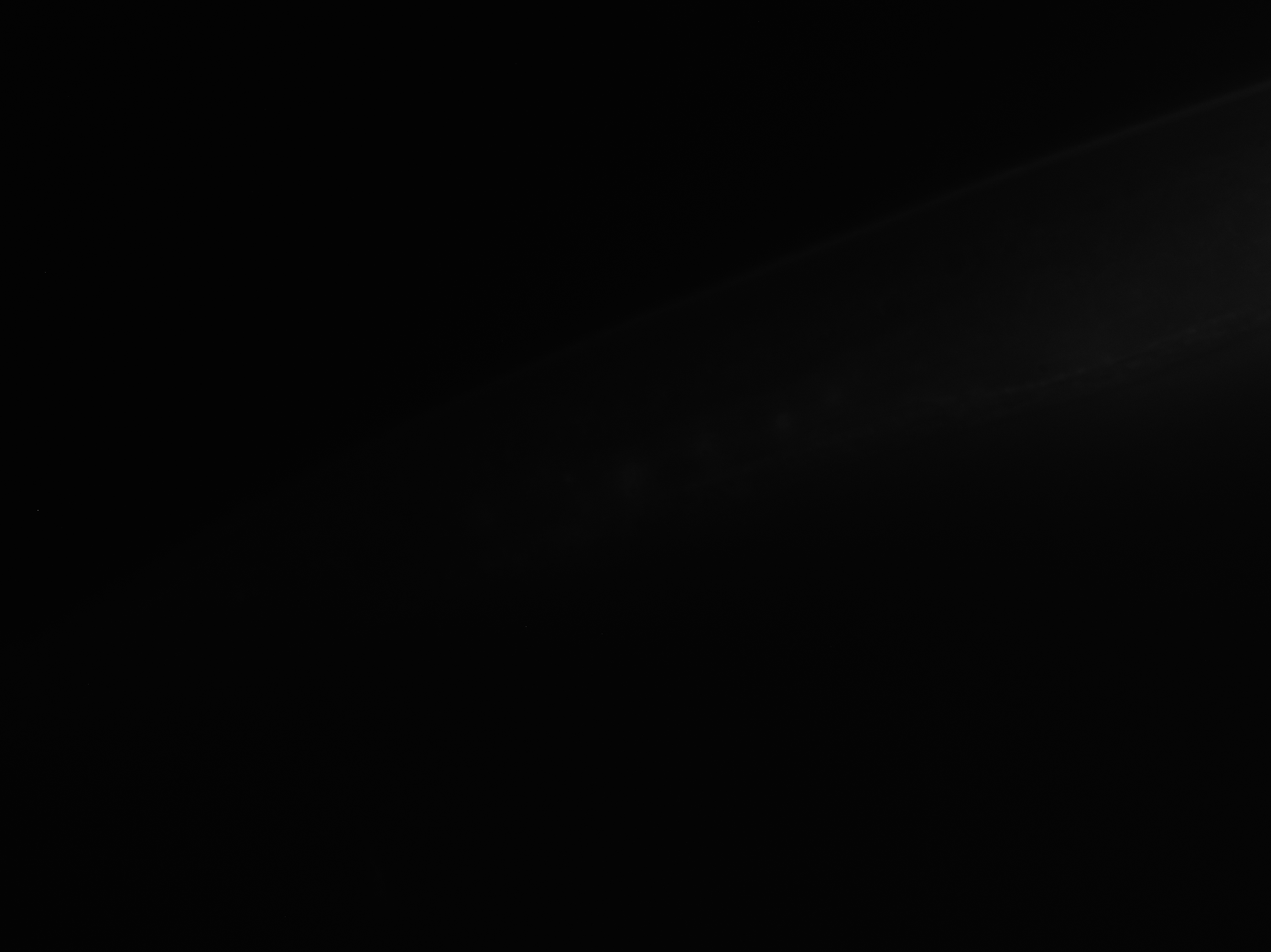

Supplement: Supplementary file 5 — Source data Fig. 4 [file 44319_2025_493_MOESM5_ESM.zip › Figure4/Fig4D/Experiment-122_included.tif_files/Experiment-122_z4c0x0-1388y0-1040.tif]

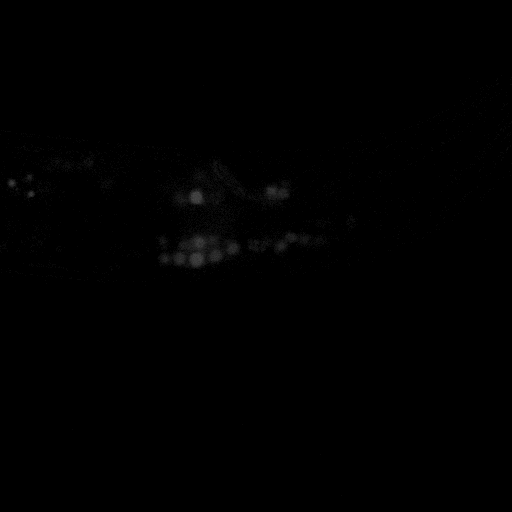

Supplement: Supplementary file 5 — Source data Fig. 4 [file 44319_2025_493_MOESM5_ESM.zip › Figure4/Fig4G/Experiment-645wildtype_NR.czi.tif_files/Experiment-645.czi_h0b0t0z9c1x0-512y0-512.tif]

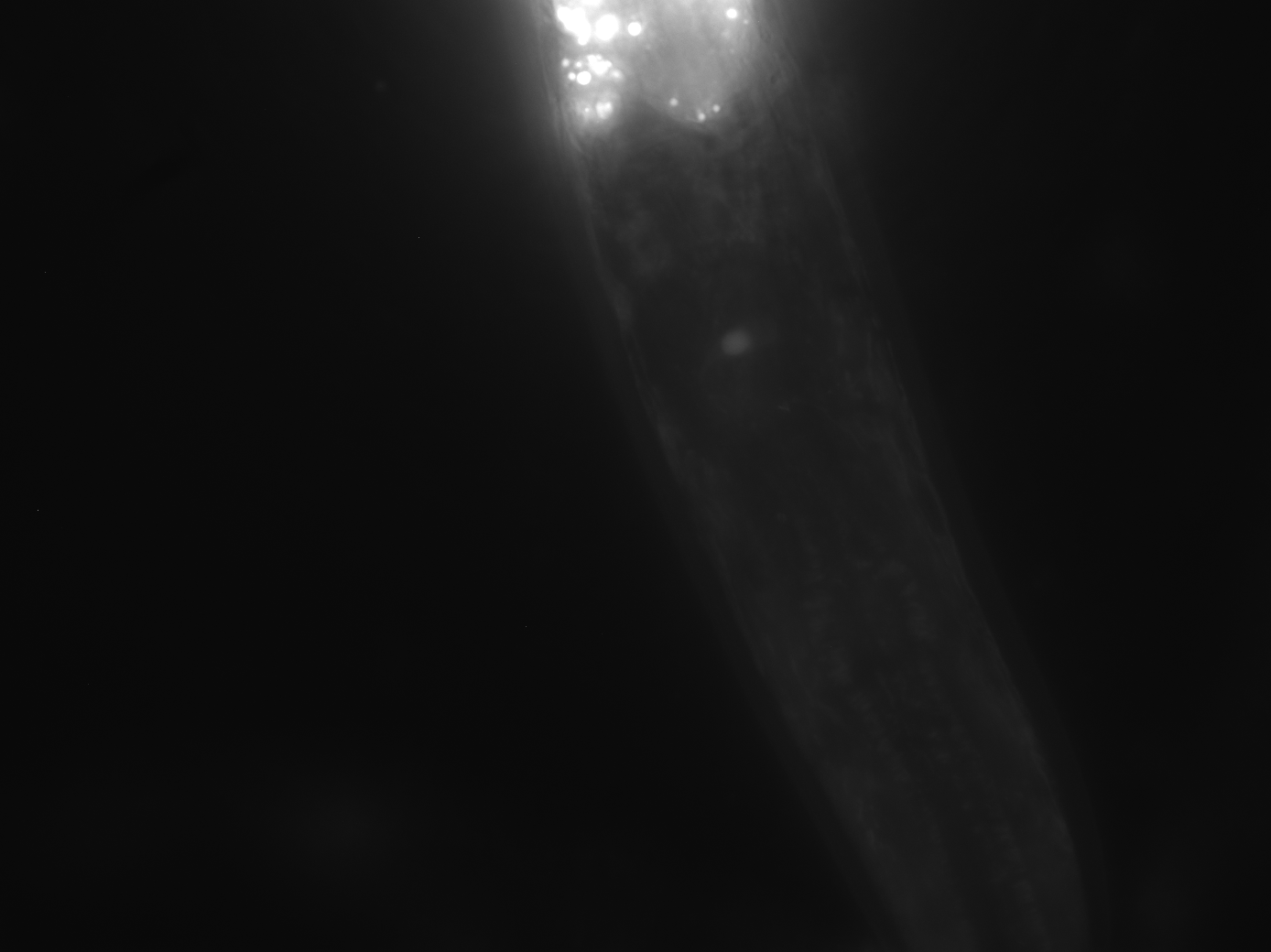

Supplement: Supplementary file 5 — Source data Fig. 4 [file 44319_2025_493_MOESM5_ESM.zip › Figure4/Fig4G/Experiment-62gooddup_AWA.tif_files/processed/MAX_Experiment-62gooddupgreen.tif_files.tif]

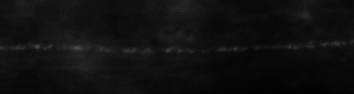

Supplement: Supplementary file 5 — Source data Fig. 4 [file 44319_2025_493_MOESM5_ESM.zip › Figure4/Fig4D/Experiment-96_wildtype.tif_files/processed/wt1.tif]

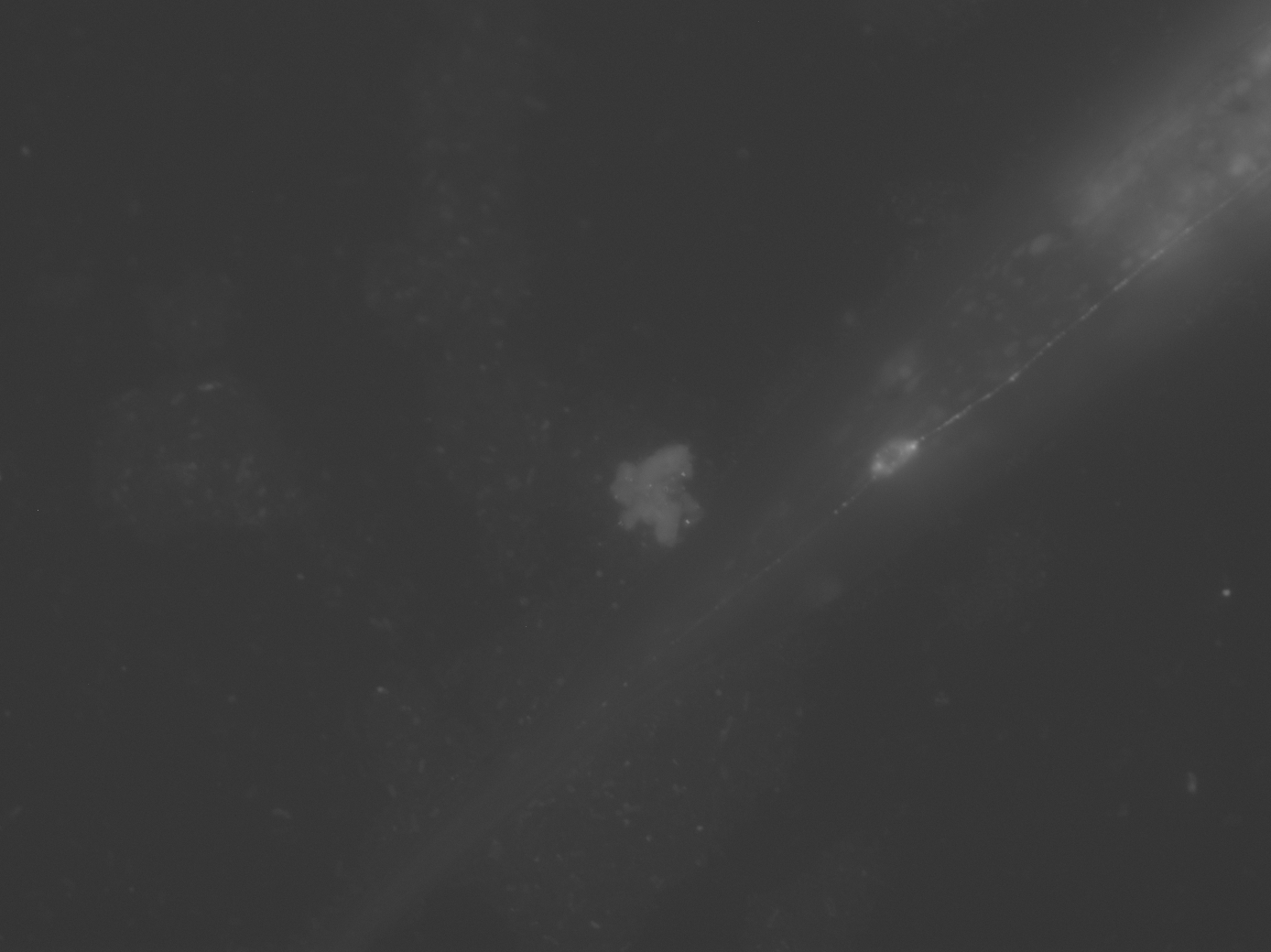

Supplement: Supplementary file 6 — Source data Fig. 5 [file 44319_2025_493_MOESM6_ESM.zip › Figure5/Fig5F/Experiment-31_cellbody_skipped.tif_files/Experiment-31_z2c0x0-1388y0-1040.tif]

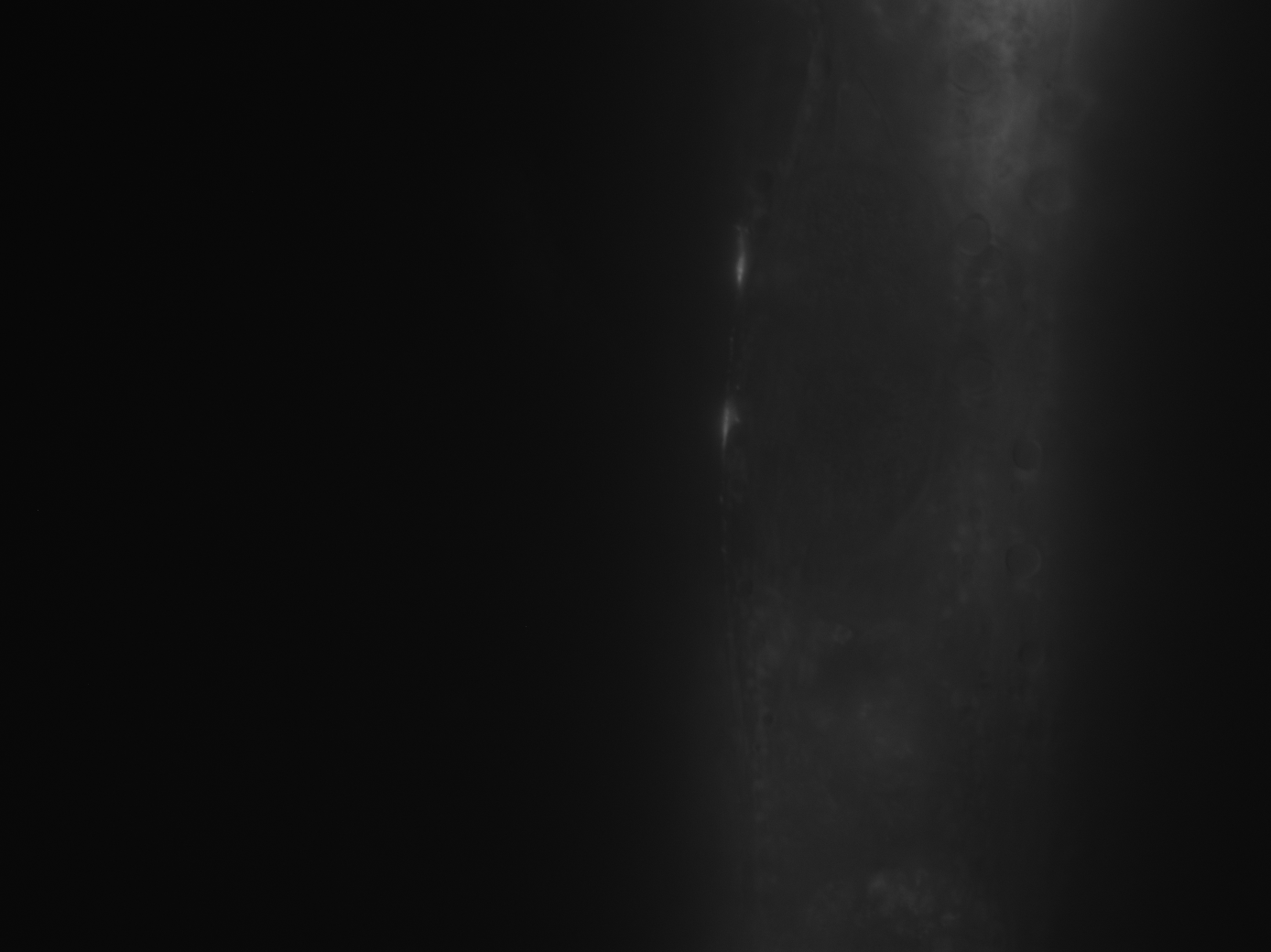

Supplement: Supplementary file 6 — Source data Fig. 5 [file 44319_2025_493_MOESM6_ESM.zip › Figure5/Fig5F/Experiment-70_synapse_skipped.tif_files/Experiment-70_z3c0x0-1388y0-1040.tif]

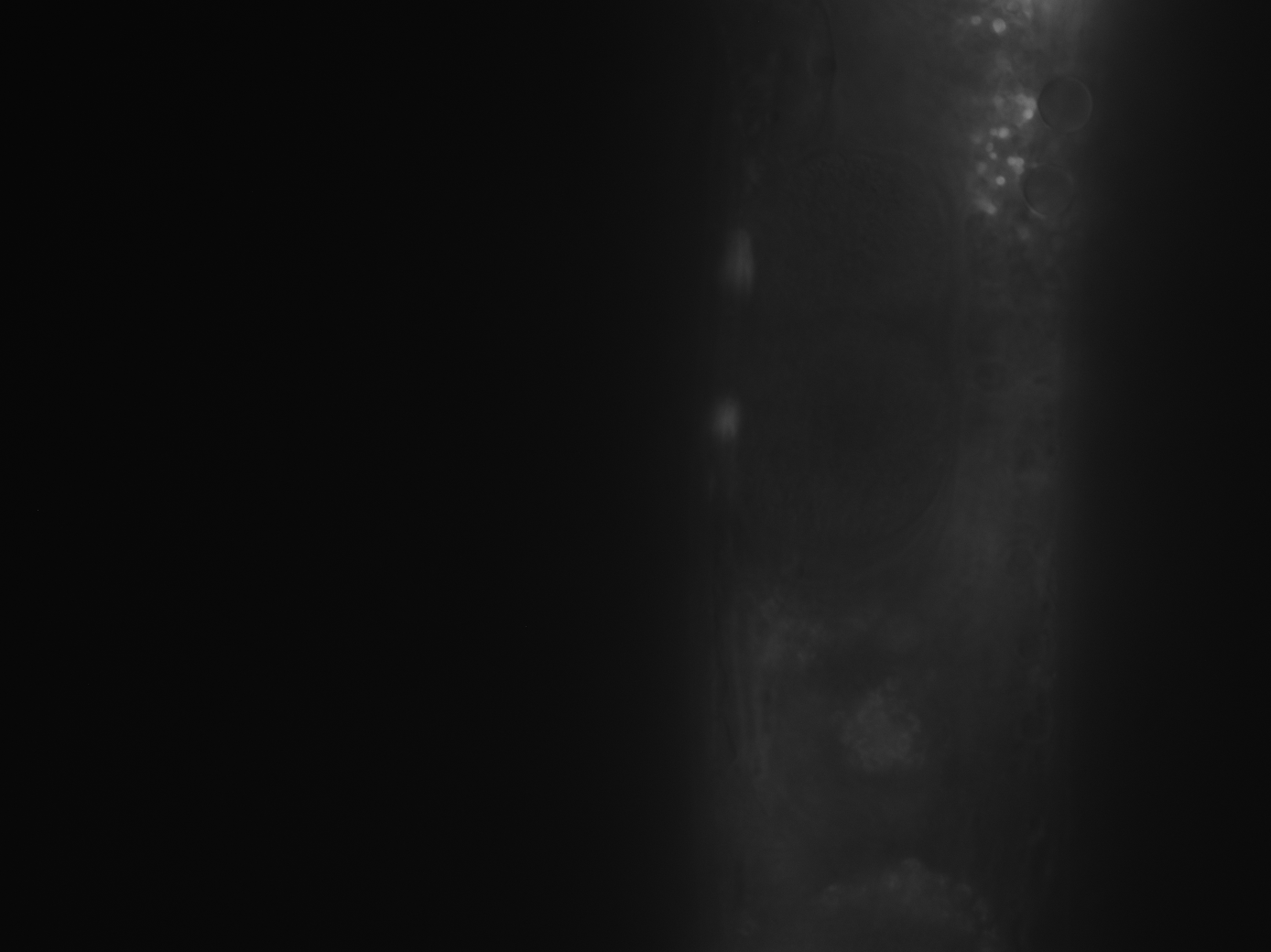

Supplement: Supplementary file 6 — Source data Fig. 5 [file 44319_2025_493_MOESM6_ESM.zip › Figure5/Fig5F/Experiment-70_synapse_skipped.tif_files/Experiment-70_z7c0x0-1388y0-1040.tif]

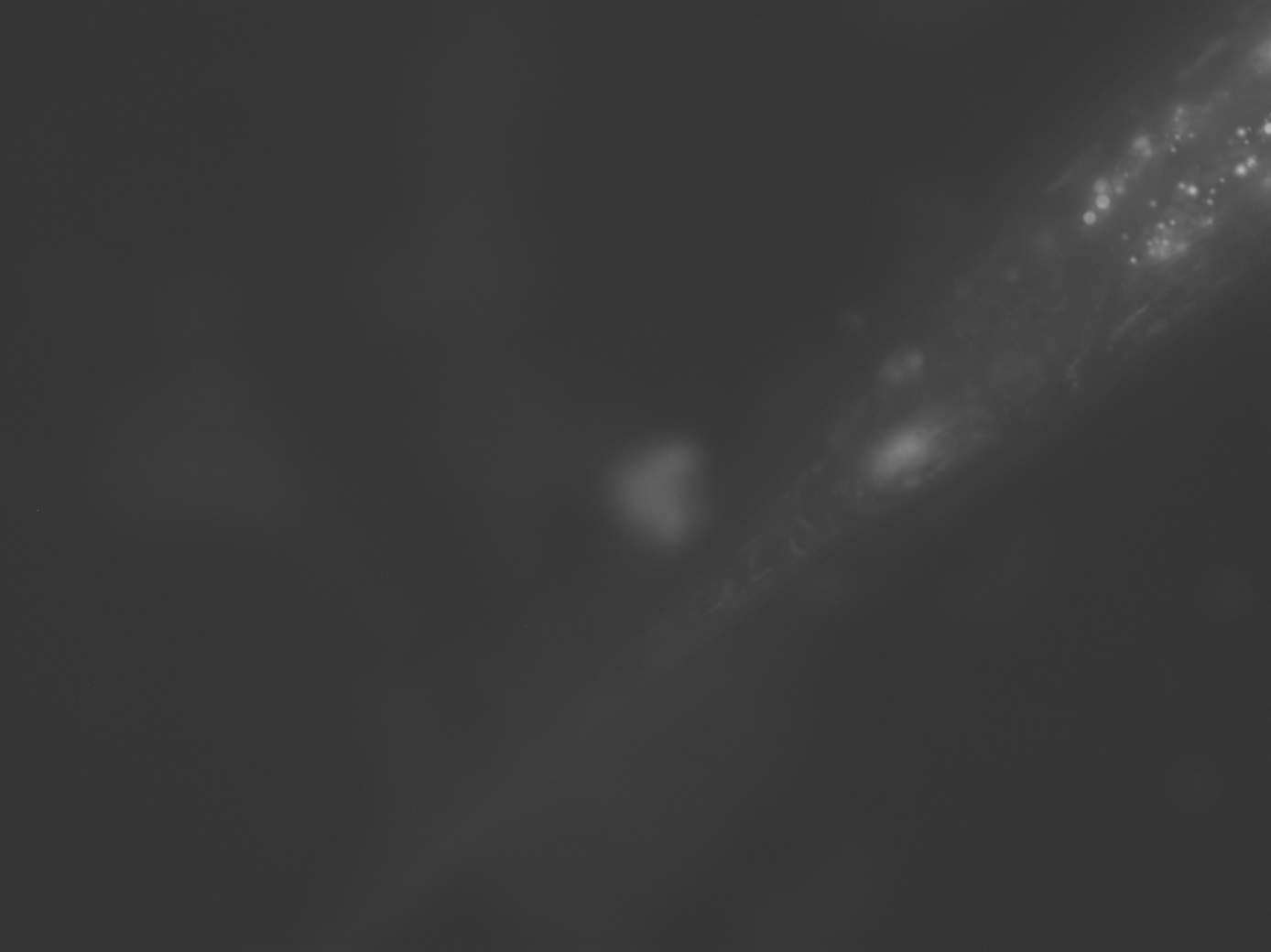

Supplement: Supplementary file 6 — Source data Fig. 5 [file 44319_2025_493_MOESM6_ESM.zip › Figure5/Fig5F/Experiment-31_cellbody_skipped.tif_files/Experiment-31_z6c0x0-1388y0-1040.tif]

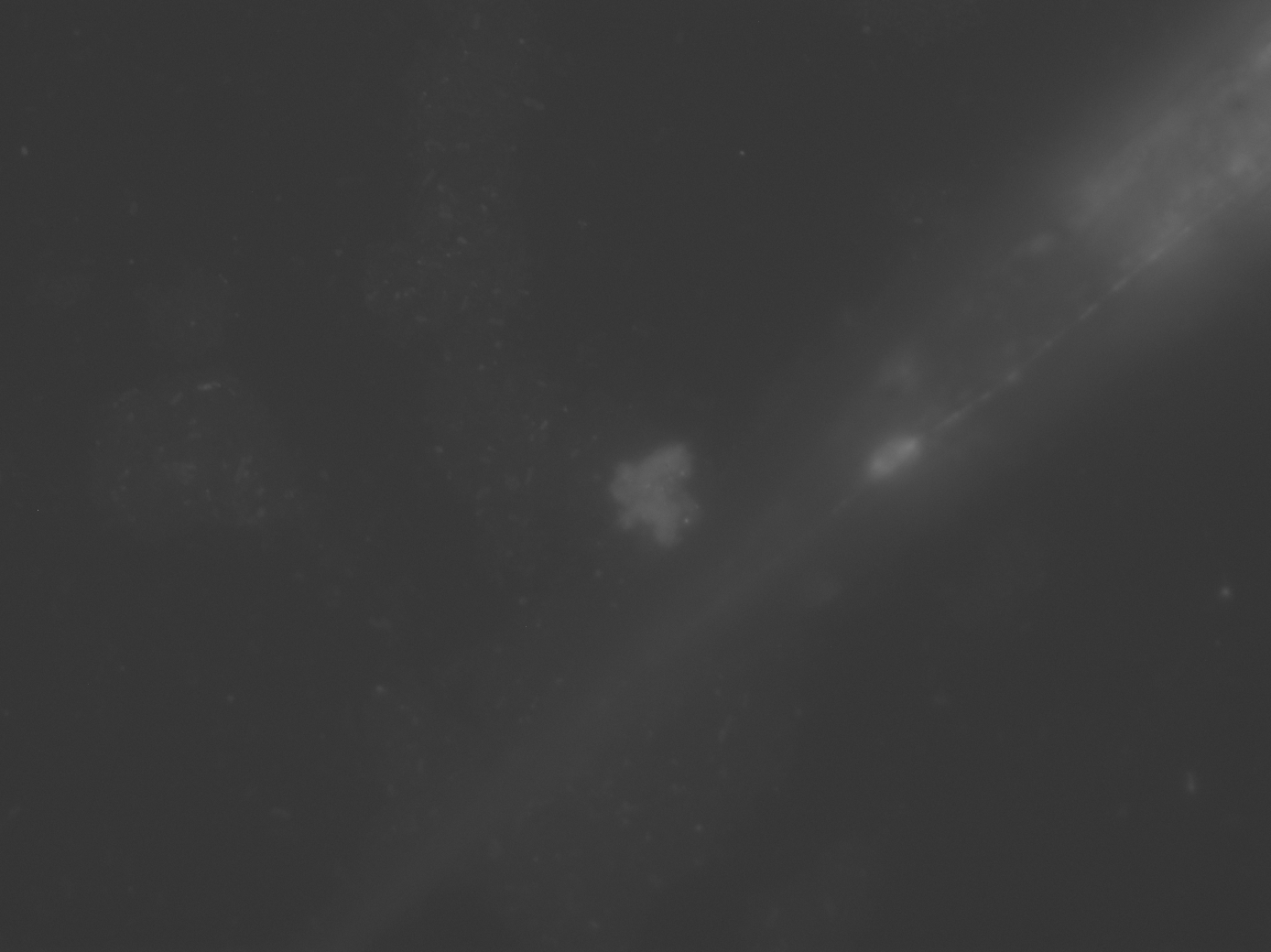

Supplement: Supplementary file 6 — Source data Fig. 5 [file 44319_2025_493_MOESM6_ESM.zip › Figure5/Fig5F/Experiment-31_cellbody_skipped.tif_files/Experiment-31_z1c0x0-1388y0-1040.tif]

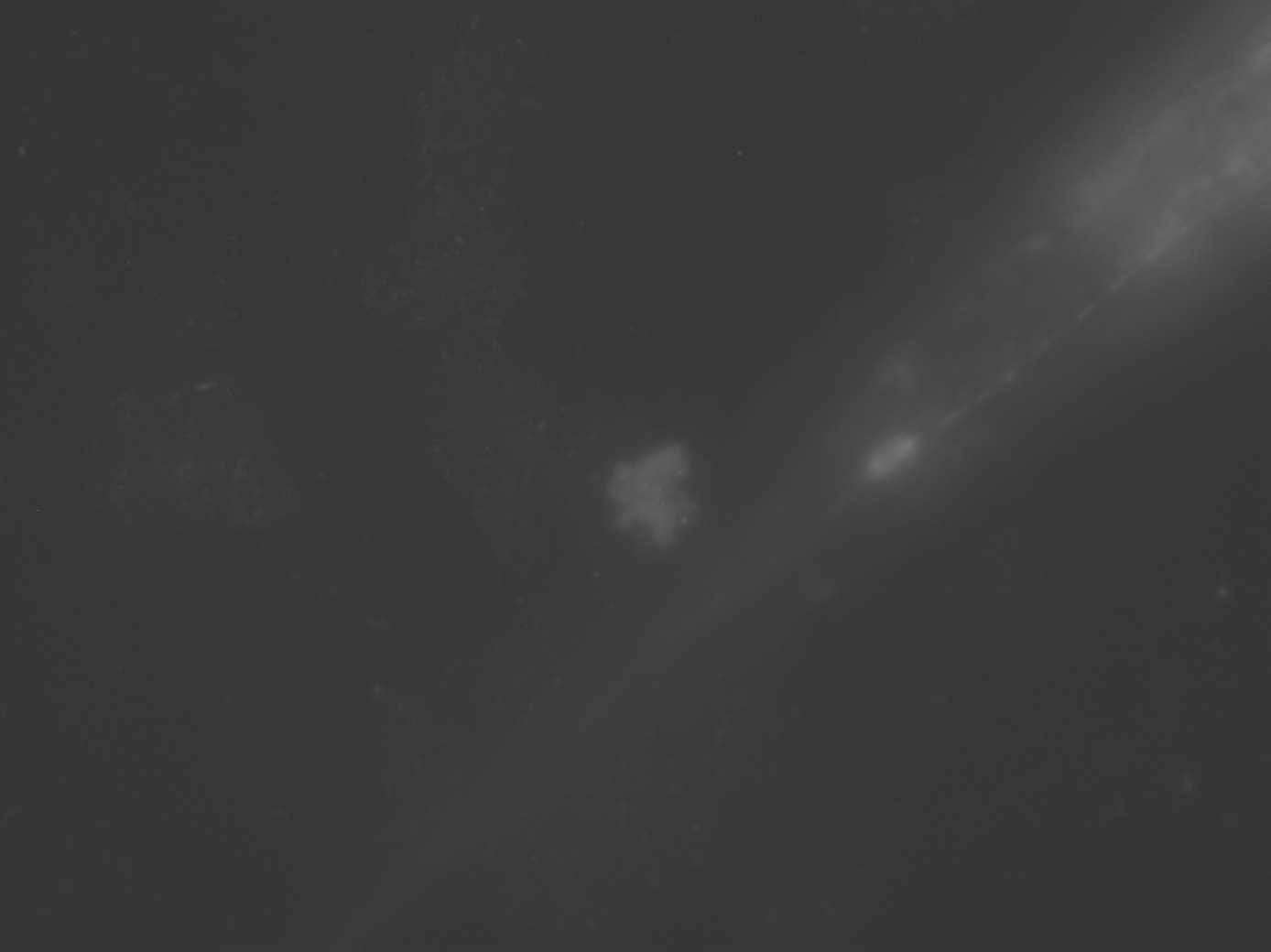

Supplement: Supplementary file 6 — Source data Fig. 5 [file 44319_2025_493_MOESM6_ESM.zip › Figure5/Fig5F/Experiment-31_cellbody_skipped.tif_files/Experiment-31_z0c0x0-1388y0-1040.tif]

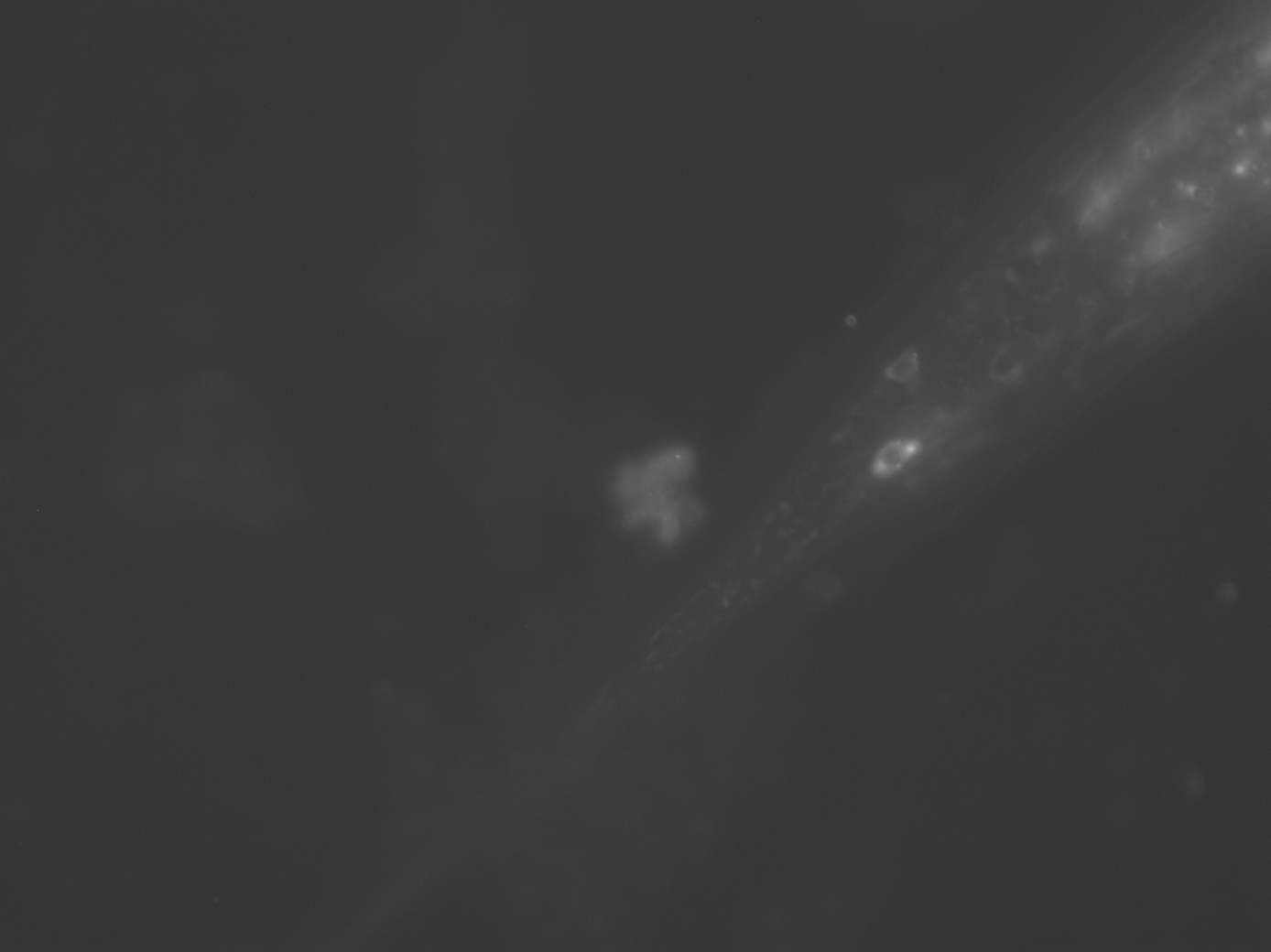

Supplement: Supplementary file 6 — Source data Fig. 5 [file 44319_2025_493_MOESM6_ESM.zip › Figure5/Fig5F/Experiment-31_cellbody_skipped.tif_files/Experiment-31_z4c0x0-1388y0-1040.tif]

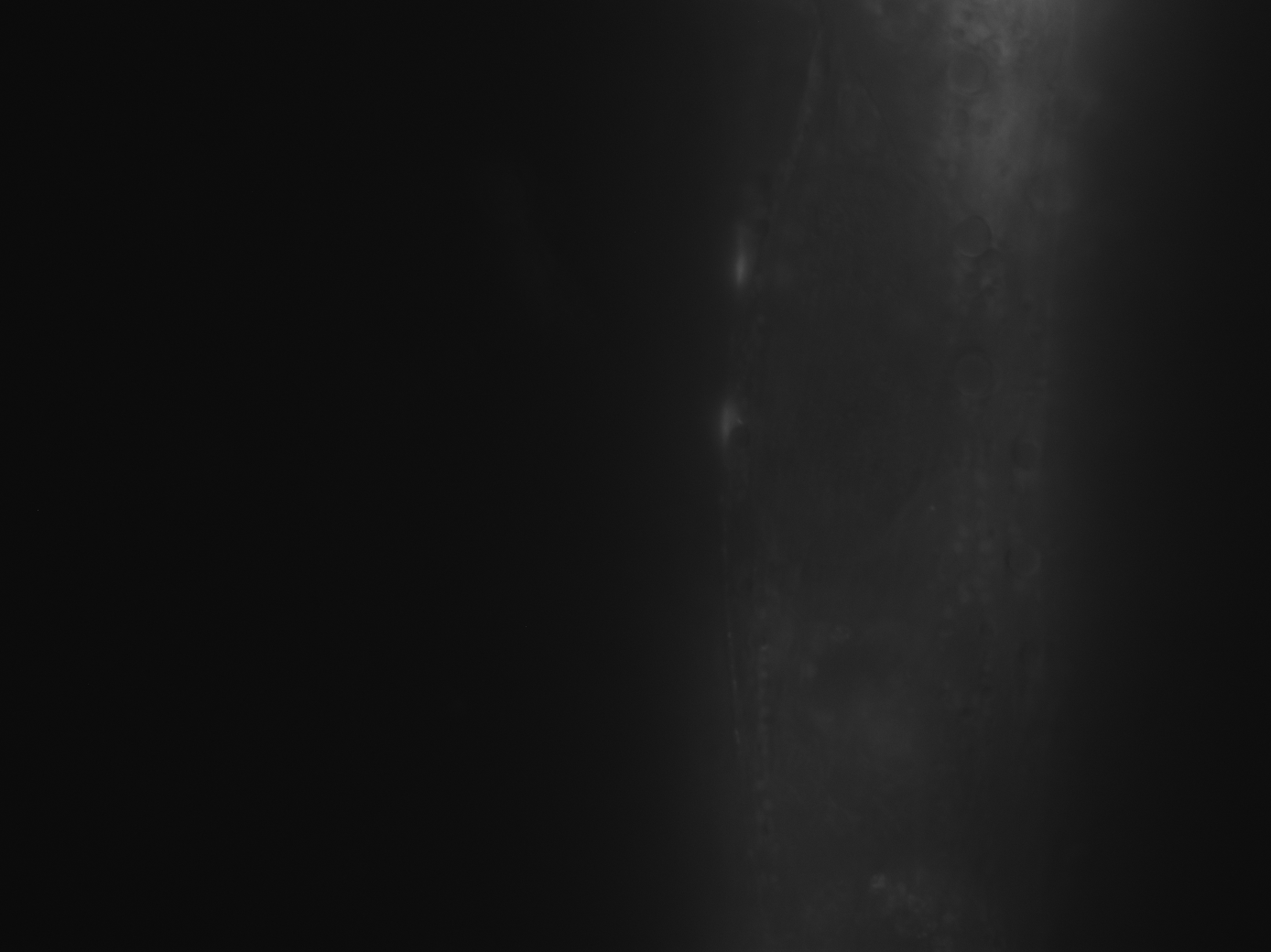

Supplement: Supplementary file 6 — Source data Fig. 5 [file 44319_2025_493_MOESM6_ESM.zip › Figure5/Fig5F/Experiment-70_synapse_skipped.tif_files/Experiment-70_z2c0x0-1388y0-1040.tif]

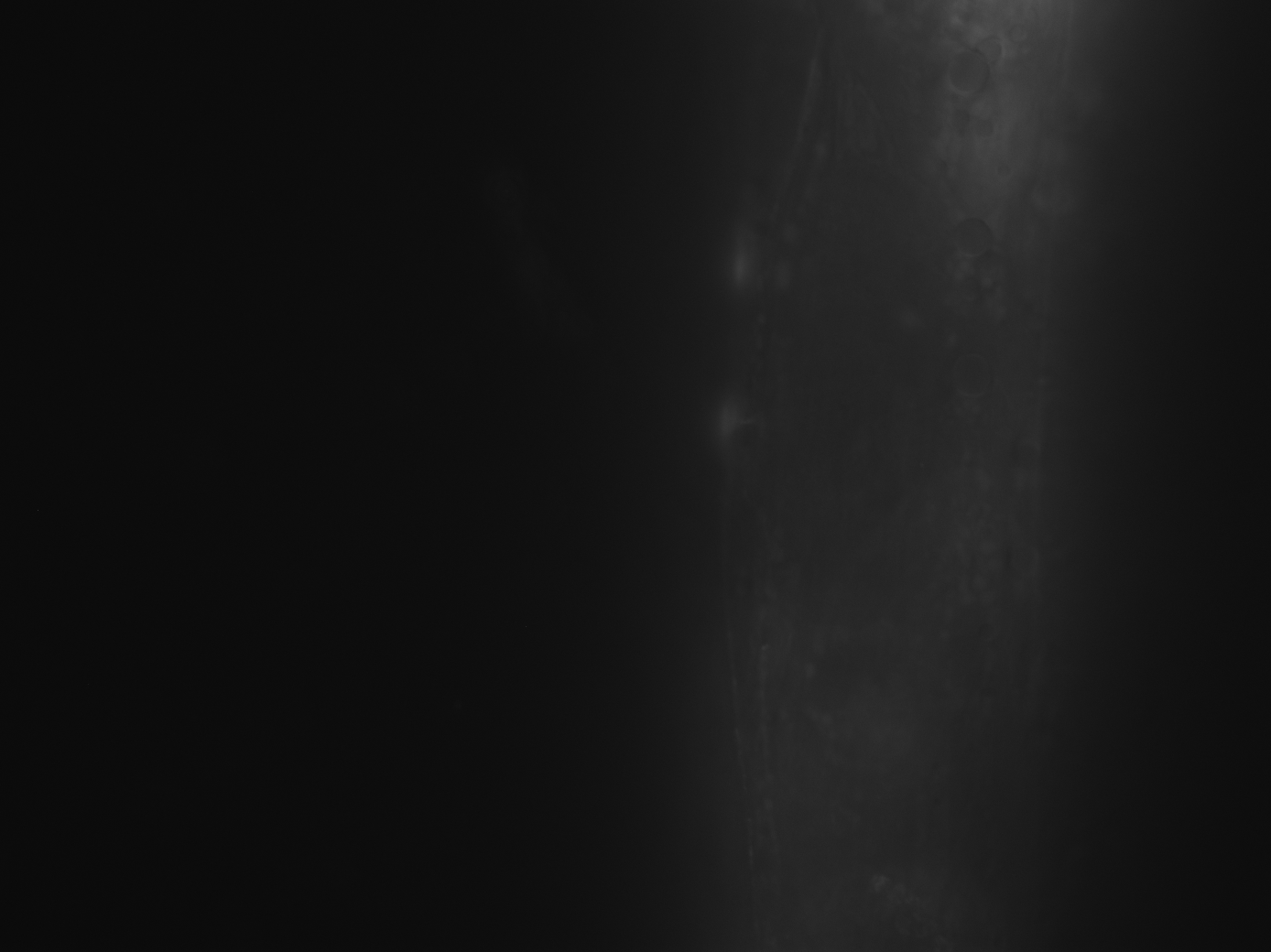

Supplement: Supplementary file 6 — Source data Fig. 5 [file 44319_2025_493_MOESM6_ESM.zip › Figure5/Fig5F/Experiment-70_synapse_skipped.tif_files/Experiment-70_z1c0x0-1388y0-1040.tif]

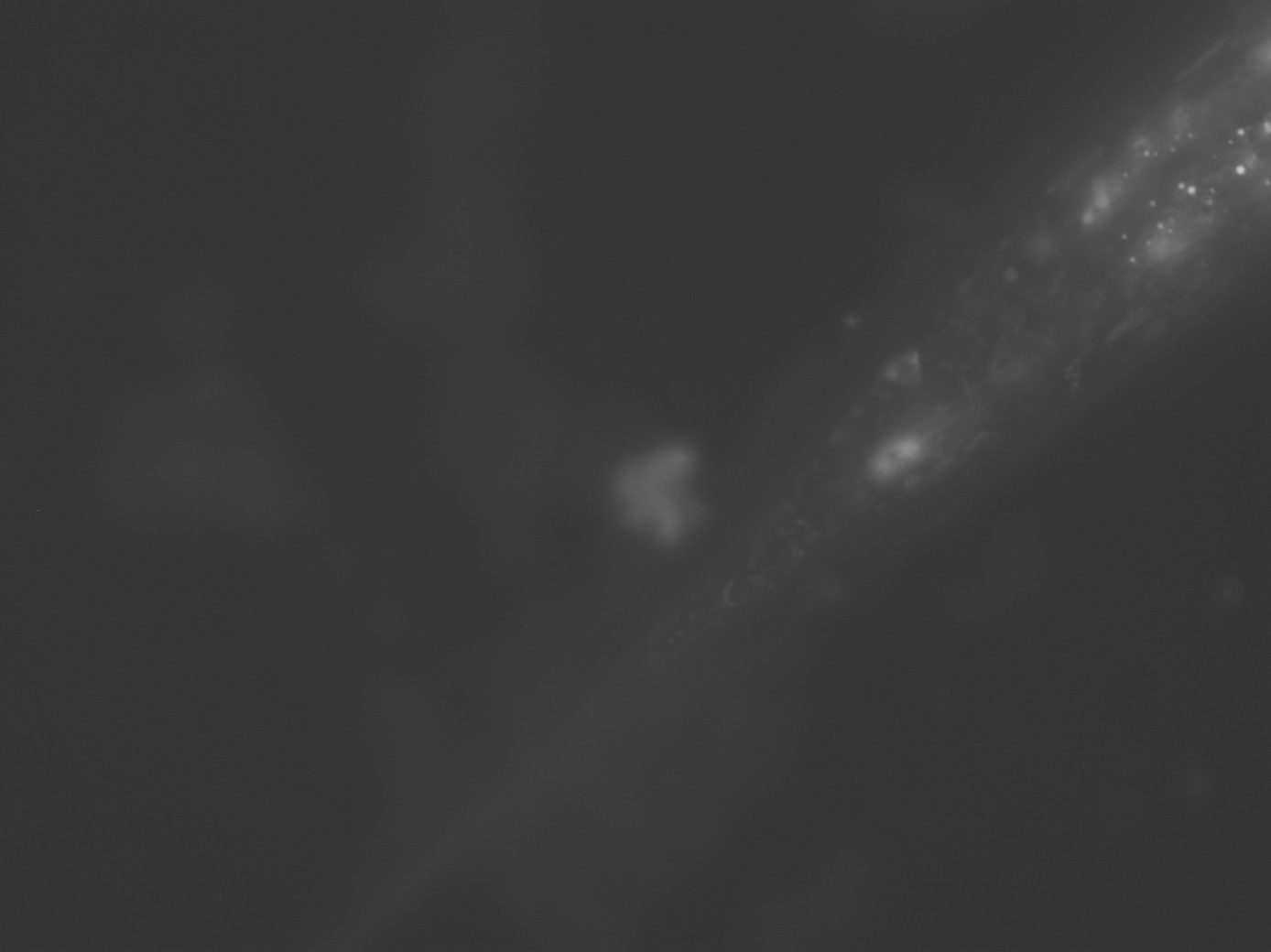

Supplement: Supplementary file 6 — Source data Fig. 5 [file 44319_2025_493_MOESM6_ESM.zip › Figure5/Fig5F/Experiment-31_cellbody_skipped.tif_files/Experiment-31_z5c0x0-1388y0-1040.tif]

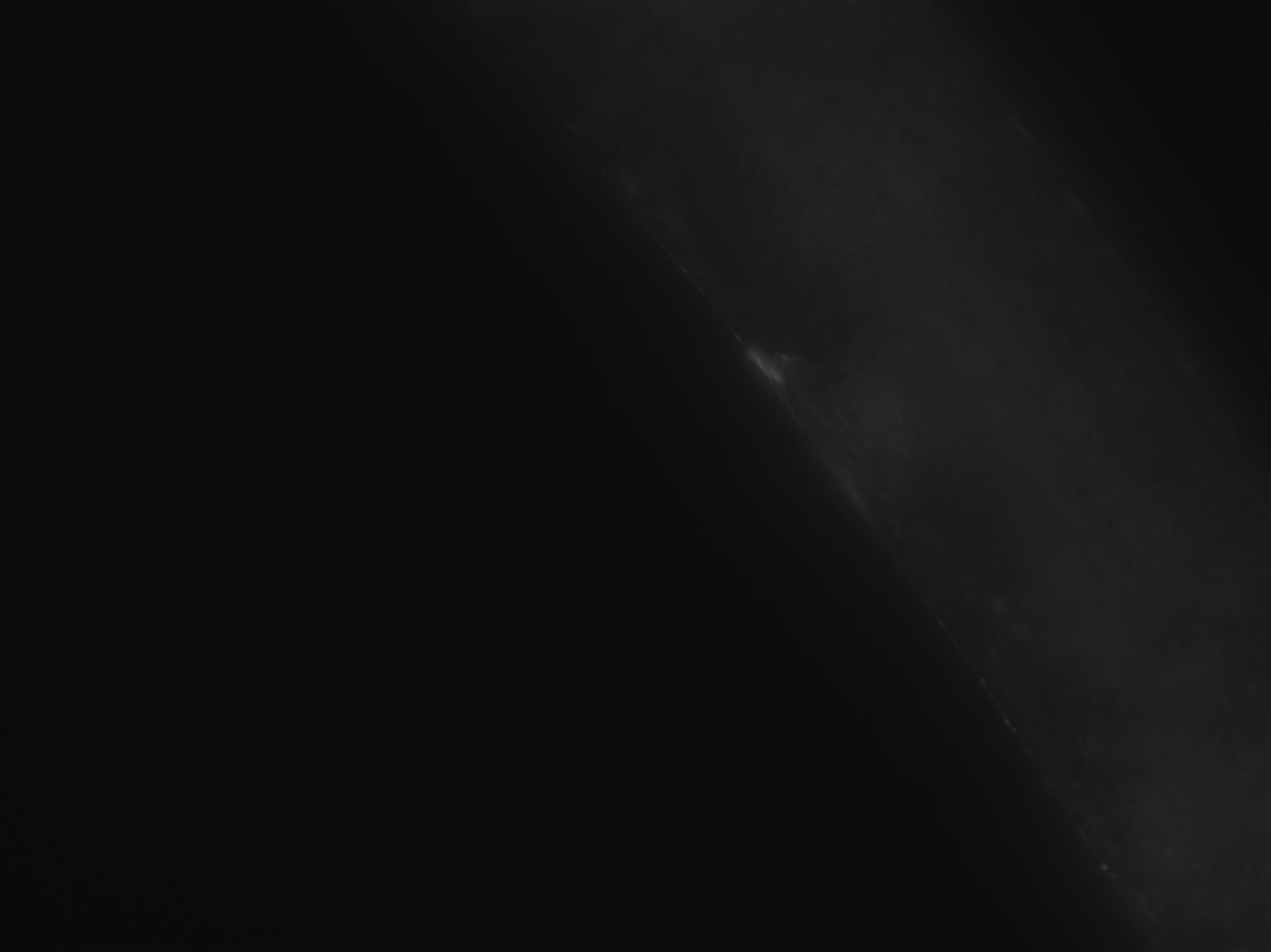

Supplement: Supplementary file 6 — Source data Fig. 5 [file 44319_2025_493_MOESM6_ESM.zip › Figure5/Fig5F/Experiment-46_synapse_wt.tif_files/Experiment-46_z3c0x0-1388y0-1040.tif]

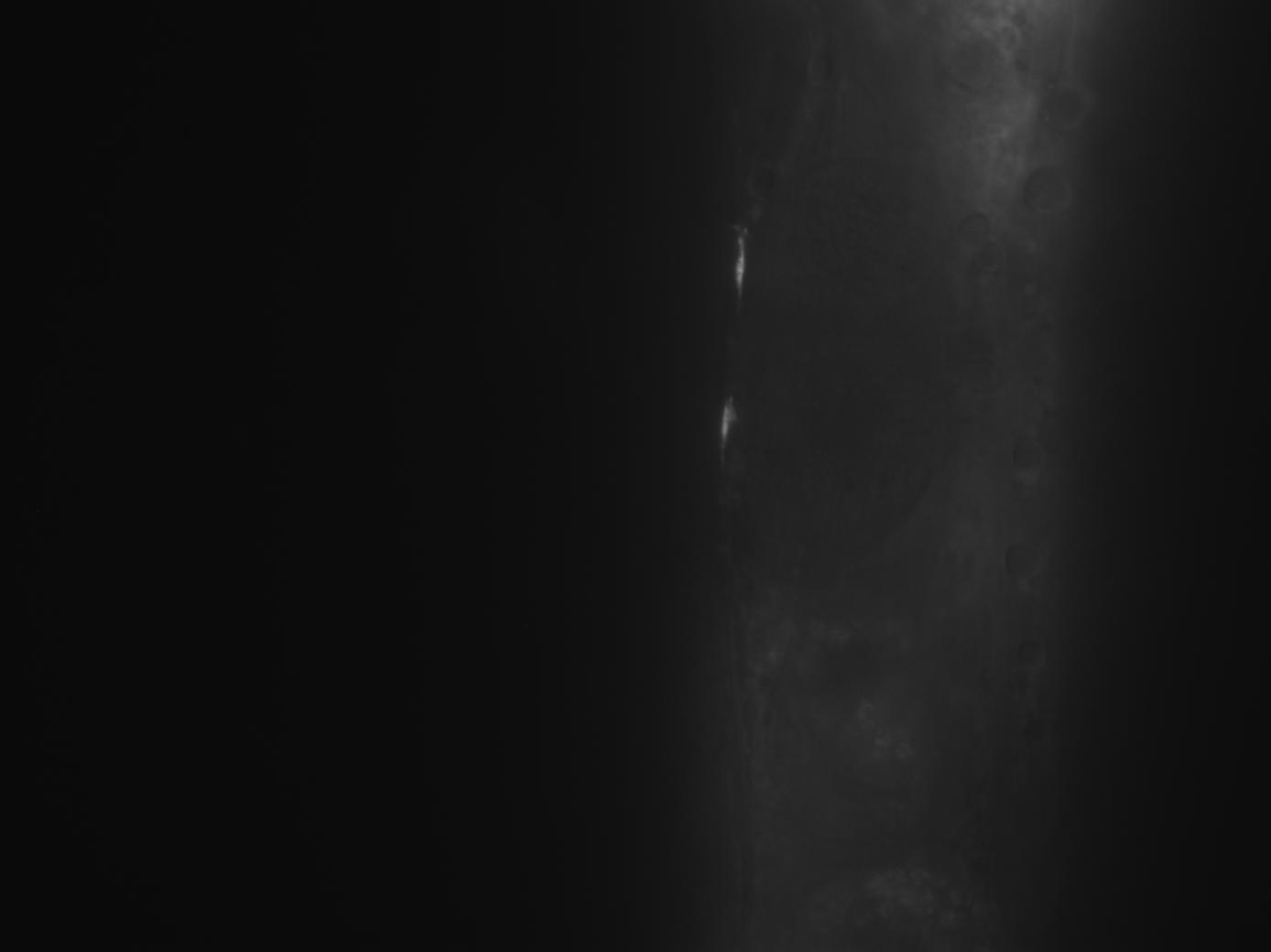

Supplement: Supplementary file 6 — Source data Fig. 5 [file 44319_2025_493_MOESM6_ESM.zip › Figure5/Fig5F/Experiment-70_synapse_skipped.tif_files/Experiment-70_z4c0x0-1388y0-1040.tif]

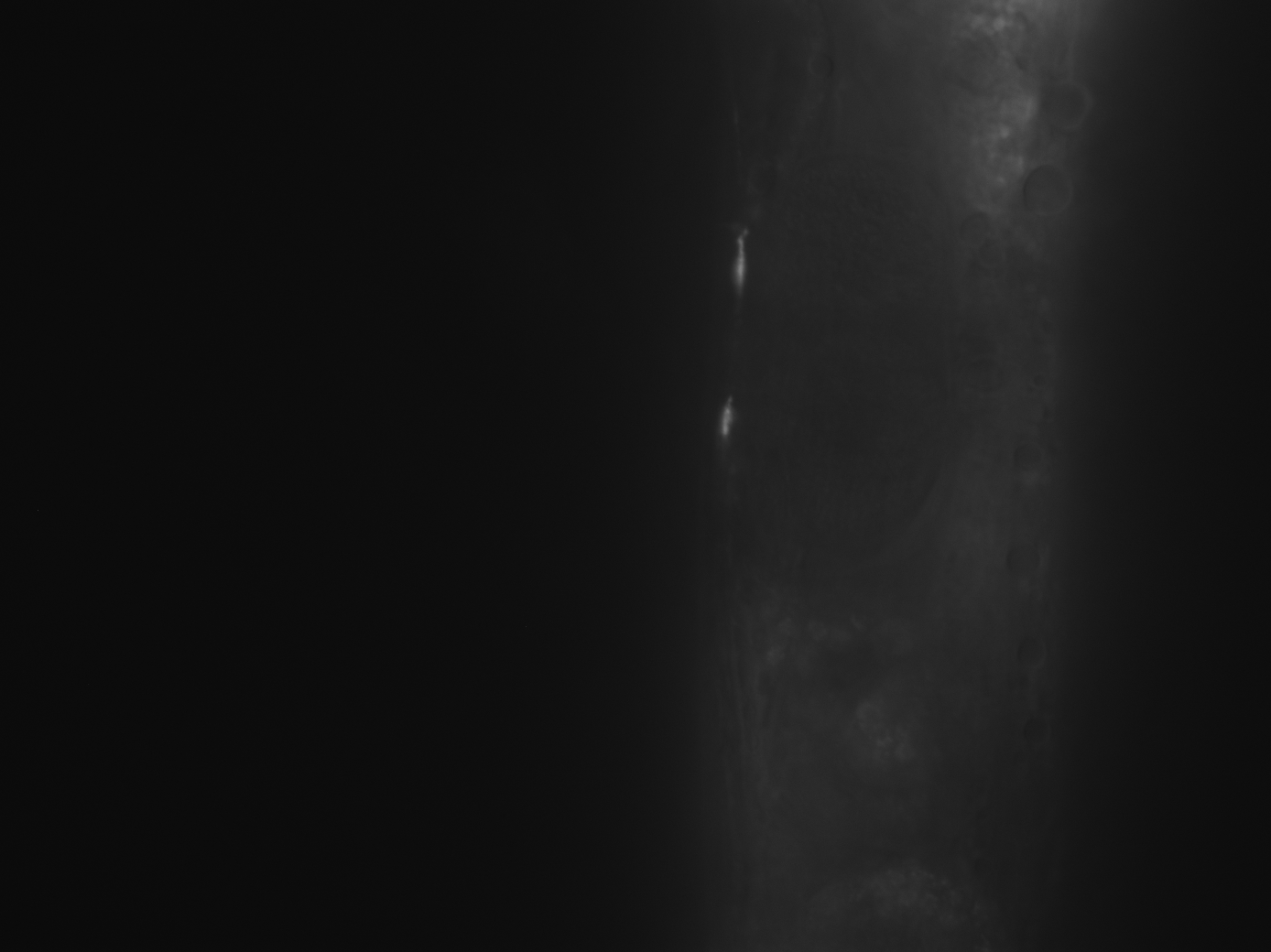

Supplement: Supplementary file 6 — Source data Fig. 5 [file 44319_2025_493_MOESM6_ESM.zip › Figure5/Fig5F/Experiment-70_synapse_skipped.tif_files/Experiment-70_z5c0x0-1388y0-1040.tif]

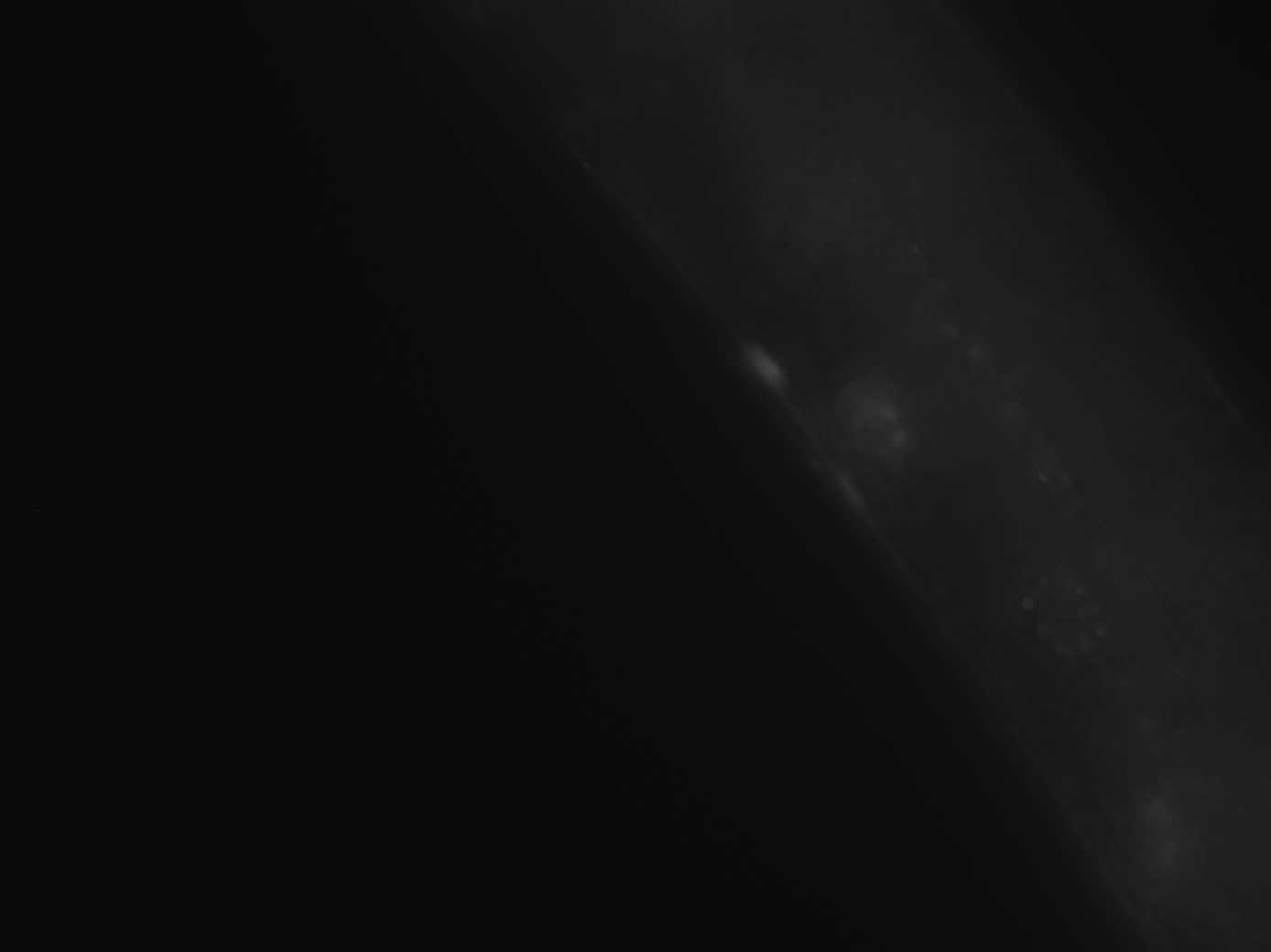

Supplement: Supplementary file 6 — Source data Fig. 5 [file 44319_2025_493_MOESM6_ESM.zip › Figure5/Fig5F/Experiment-46_synapse_wt.tif_files/Experiment-46_z7c0x0-1388y0-1040.tif]

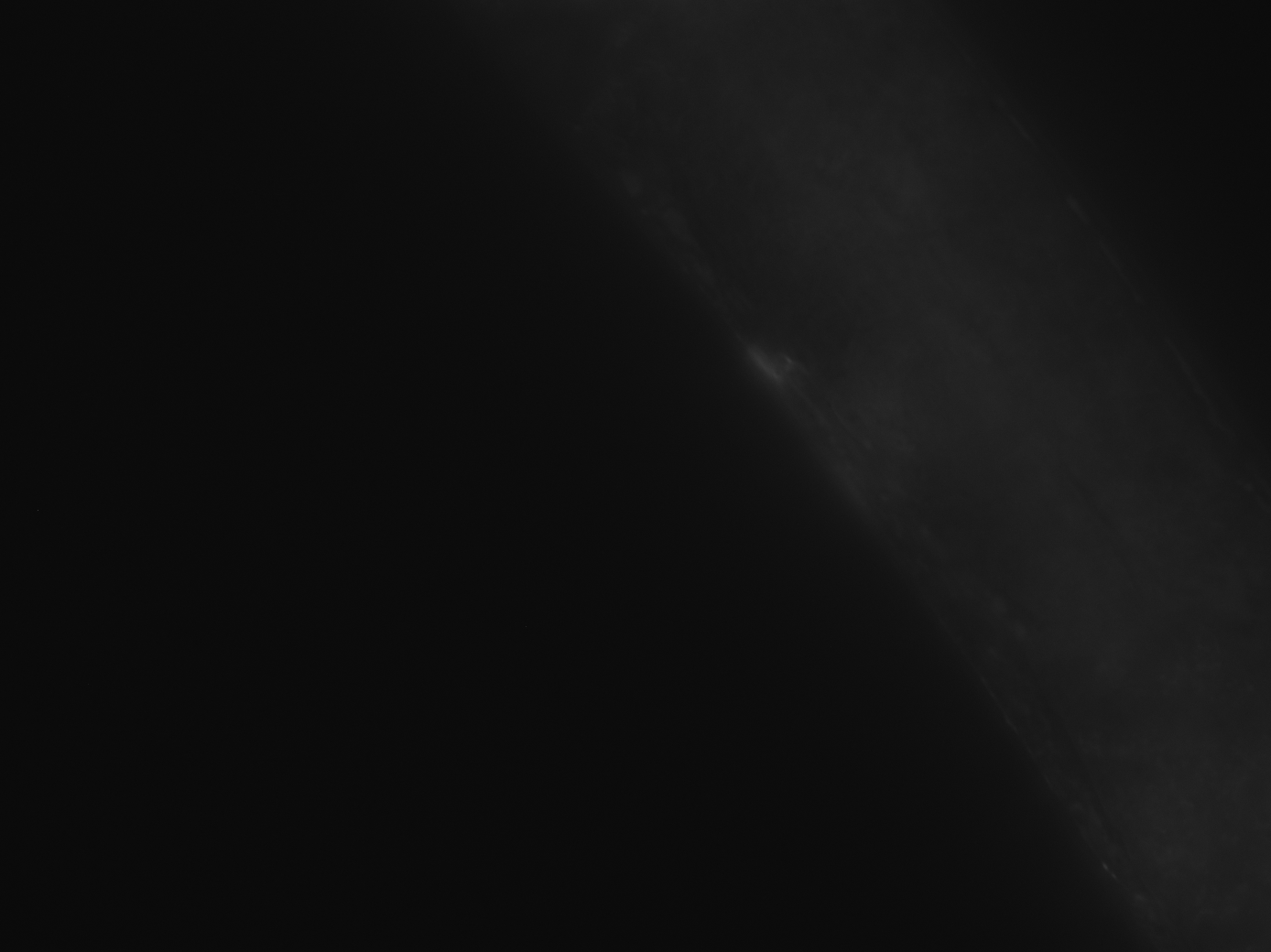

Supplement: Supplementary file 6 — Source data Fig. 5 [file 44319_2025_493_MOESM6_ESM.zip › Figure5/Fig5F/Experiment-46_synapse_wt.tif_files/Experiment-46_z2c0x0-1388y0-1040.tif]

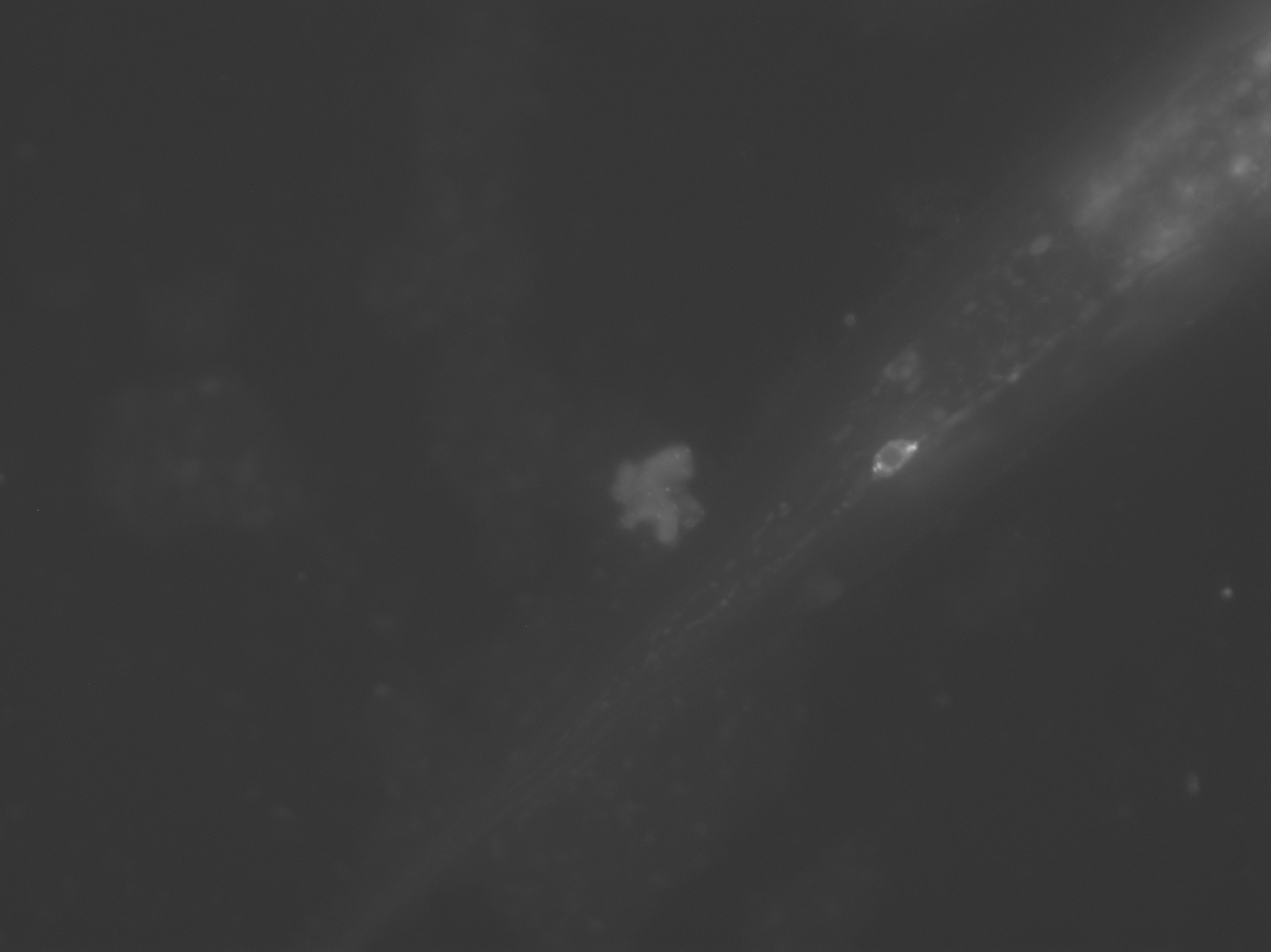

Supplement: Supplementary file 6 — Source data Fig. 5 [file 44319_2025_493_MOESM6_ESM.zip › Figure5/Fig5F/Experiment-31_cellbody_skipped.tif_files/Experiment-31_z3c0x0-1388y0-1040.tif]

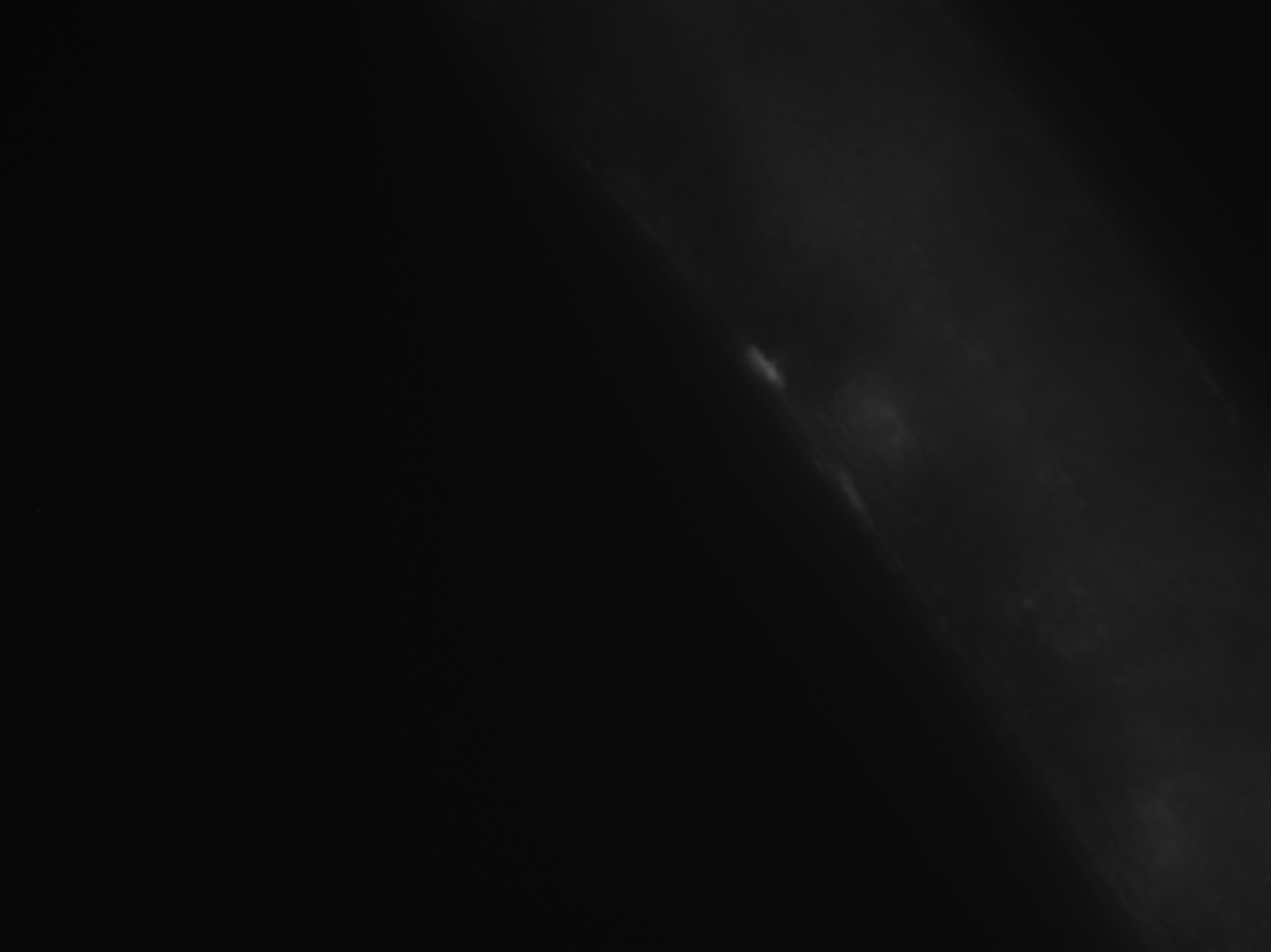

Supplement: Supplementary file 6 — Source data Fig. 5 [file 44319_2025_493_MOESM6_ESM.zip › Figure5/Fig5F/Experiment-46_synapse_wt.tif_files/Experiment-46_z6c0x0-1388y0-1040.tif]

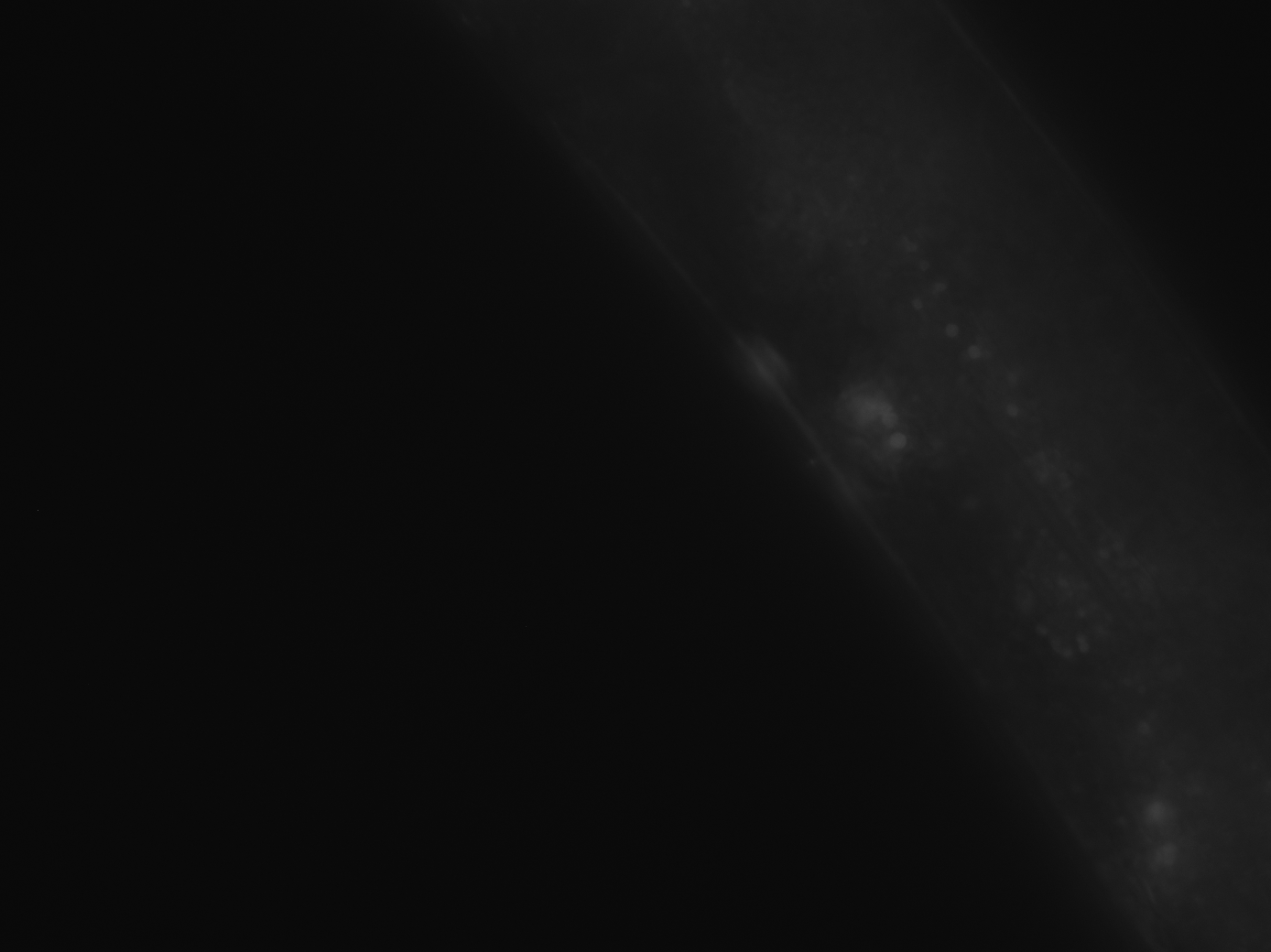

Supplement: Supplementary file 6 — Source data Fig. 5 [file 44319_2025_493_MOESM6_ESM.zip › Figure5/Fig5F/Experiment-46_synapse_wt.tif_files/Experiment-46_z9c0x0-1388y0-1040.tif]

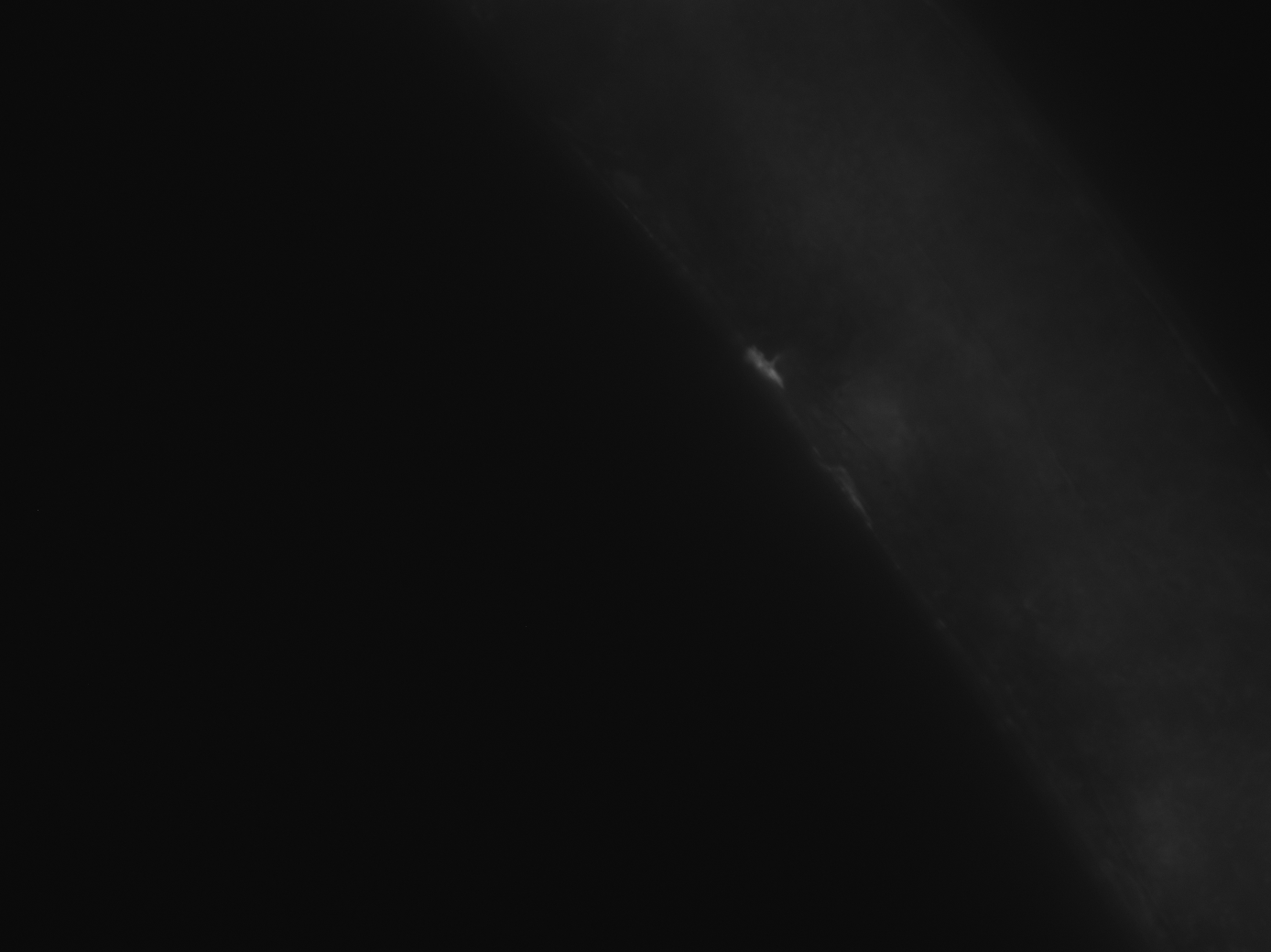

Supplement: Supplementary file 6 — Source data Fig. 5 [file 44319_2025_493_MOESM6_ESM.zip › Figure5/Fig5F/Experiment-46_synapse_wt.tif_files/Experiment-46_z5c0x0-1388y0-1040.tif]

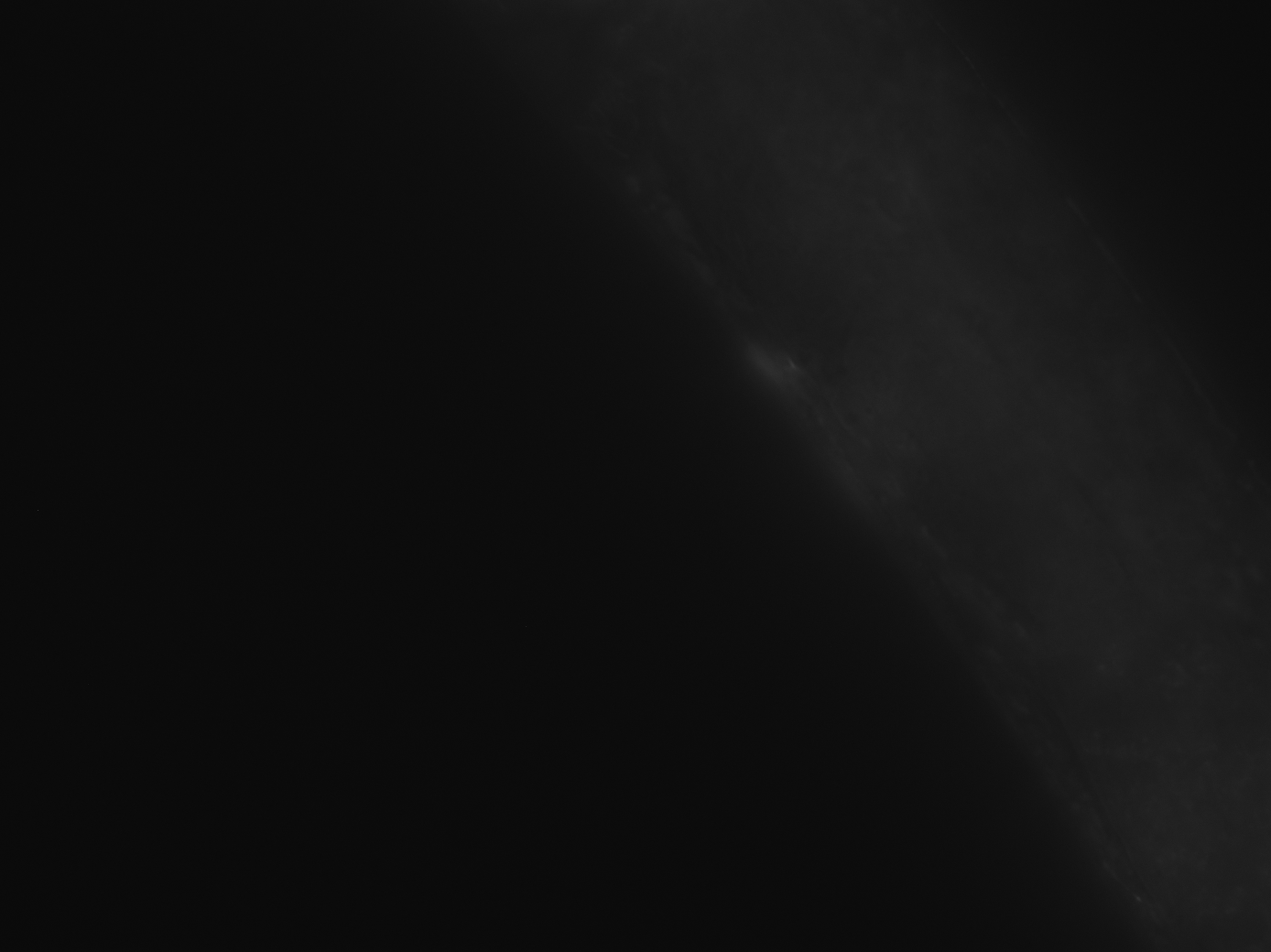

Supplement: Supplementary file 6 — Source data Fig. 5 [file 44319_2025_493_MOESM6_ESM.zip › Figure5/Fig5F/Experiment-46_synapse_wt.tif_files/Experiment-46_z1c0x0-1388y0-1040.tif]

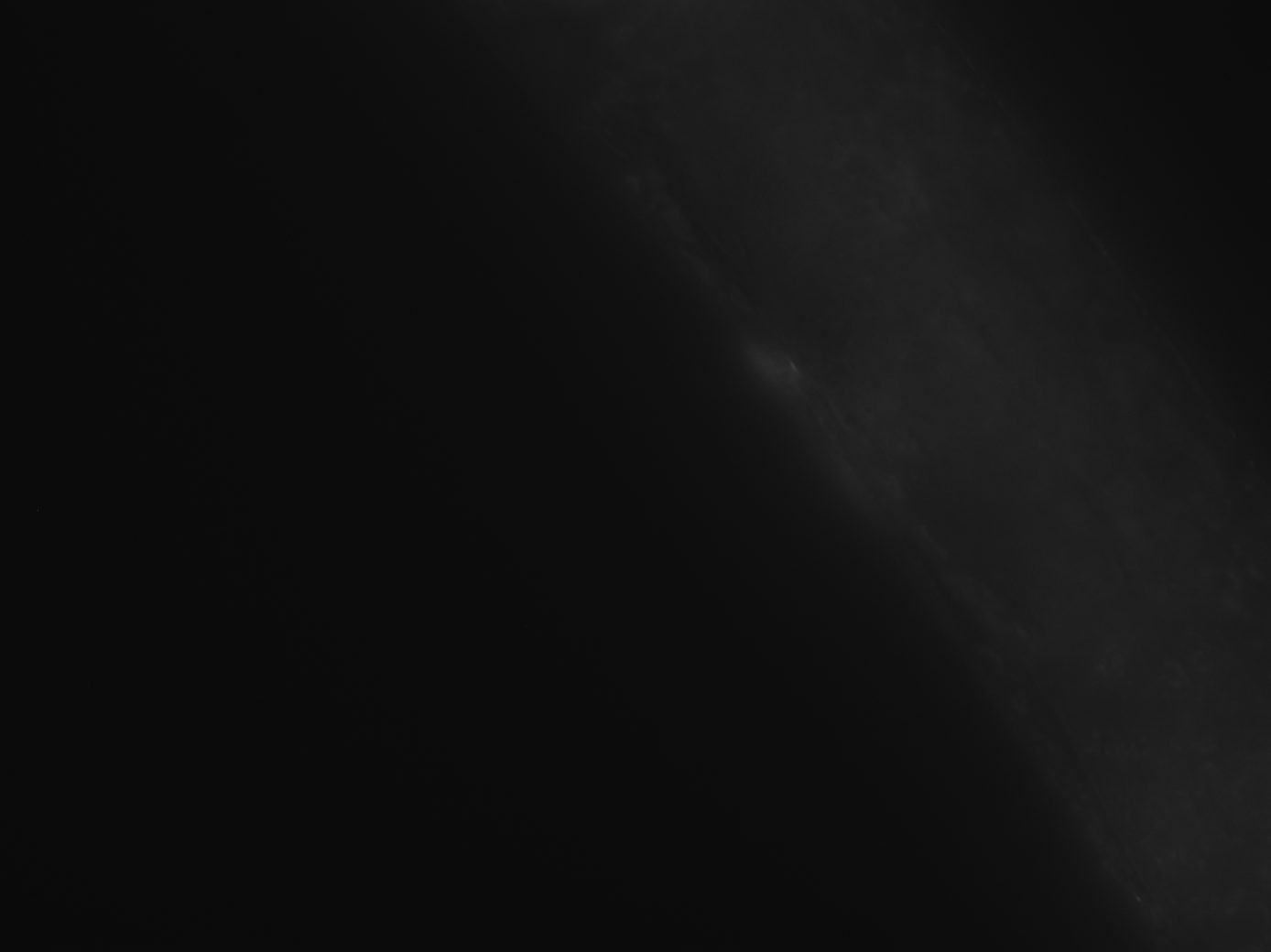

Supplement: Supplementary file 6 — Source data Fig. 5 [file 44319_2025_493_MOESM6_ESM.zip › Figure5/Fig5F/Experiment-46_synapse_wt.tif_files/Experiment-46_z0c0x0-1388y0-1040.tif]

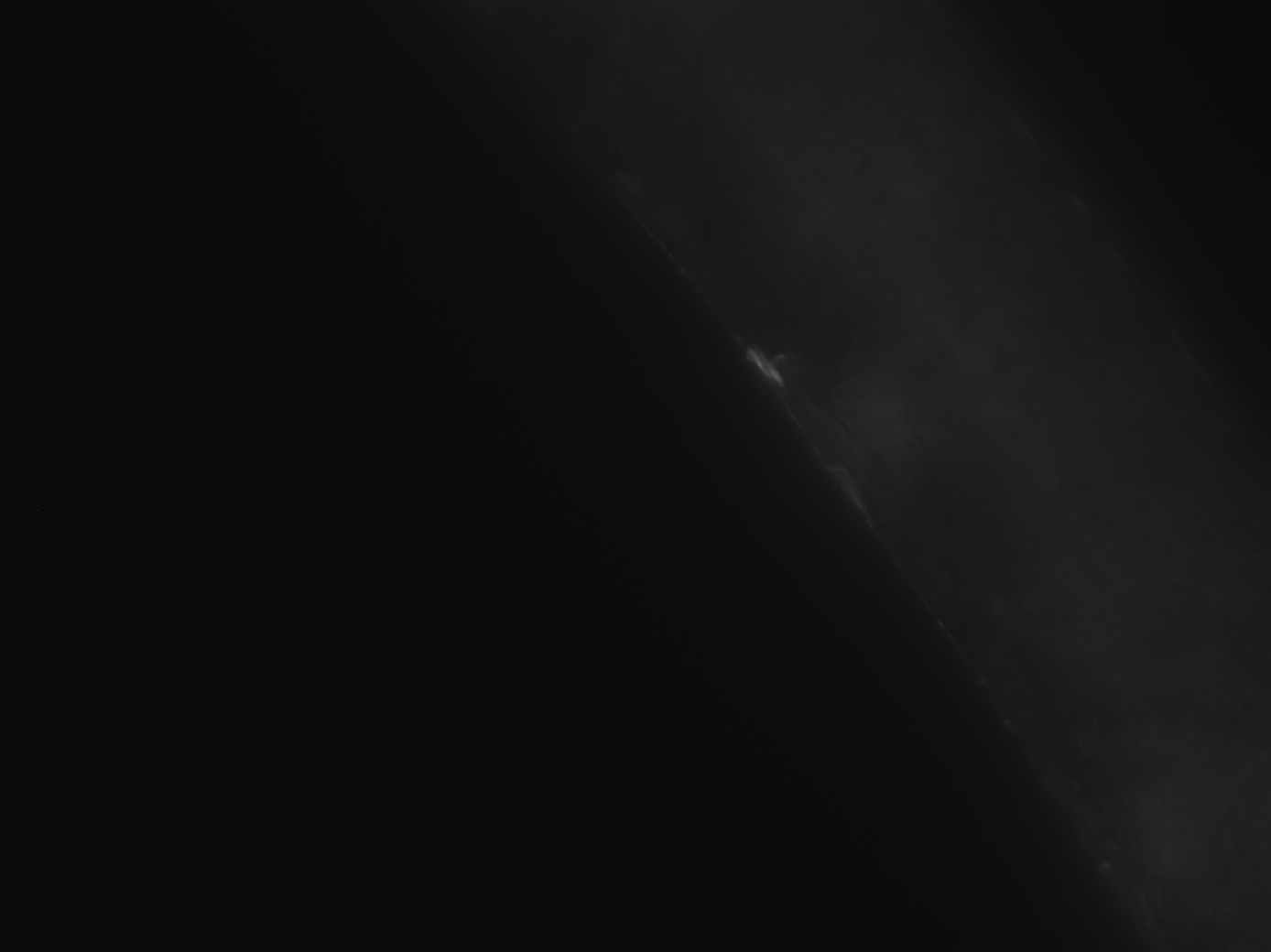

Supplement: Supplementary file 6 — Source data Fig. 5 [file 44319_2025_493_MOESM6_ESM.zip › Figure5/Fig5F/Experiment-46_synapse_wt.tif_files/Experiment-46_z4c0x0-1388y0-1040.tif]

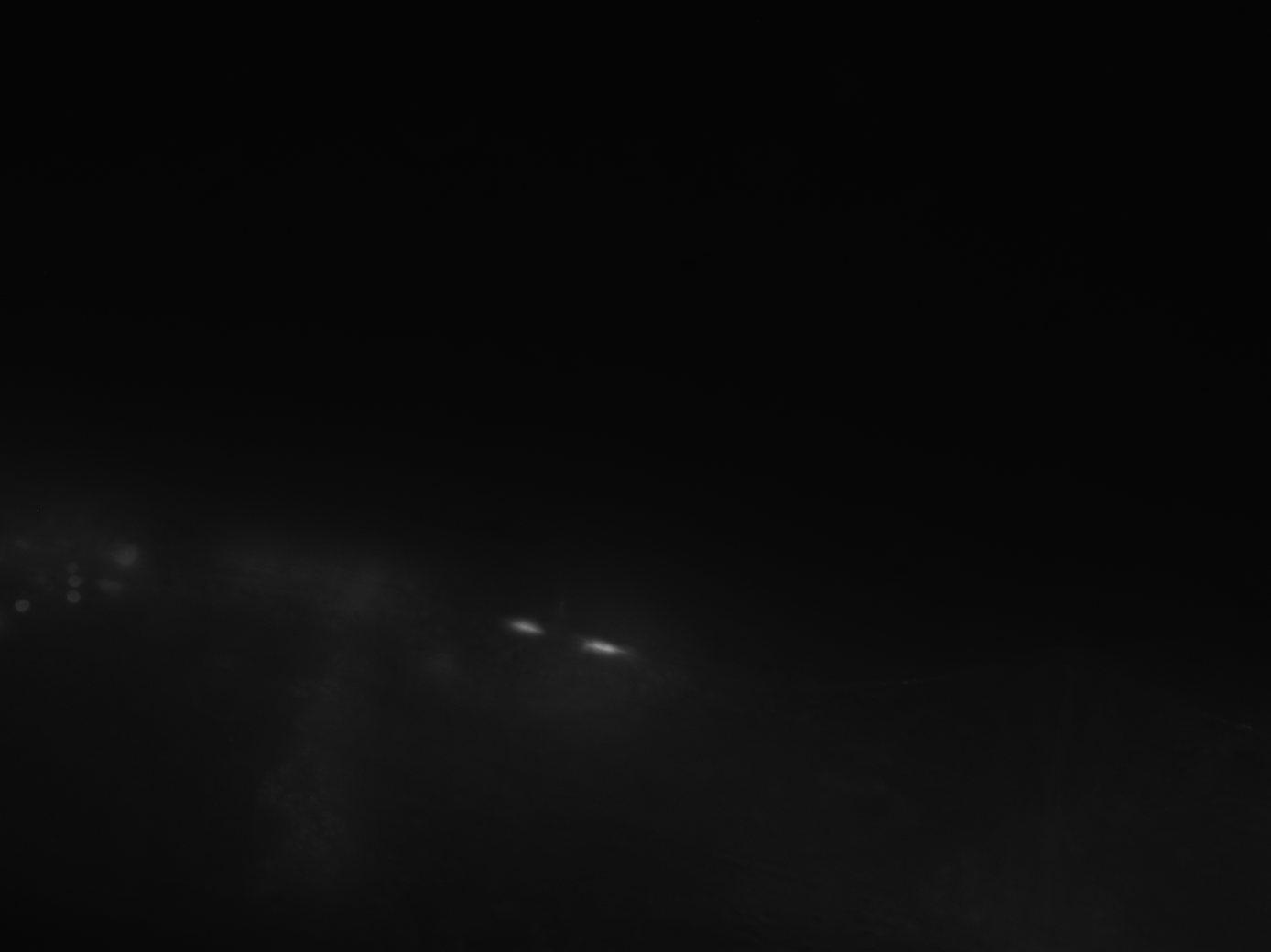

Supplement: Supplementary file 6 — Source data Fig. 5 [file 44319_2025_493_MOESM6_ESM.zip › Figure5/Fig5F/Experiment-979_synapse_n2813.tif_files/Experiment-979_z6c0x0-1388y0-1040.tif]

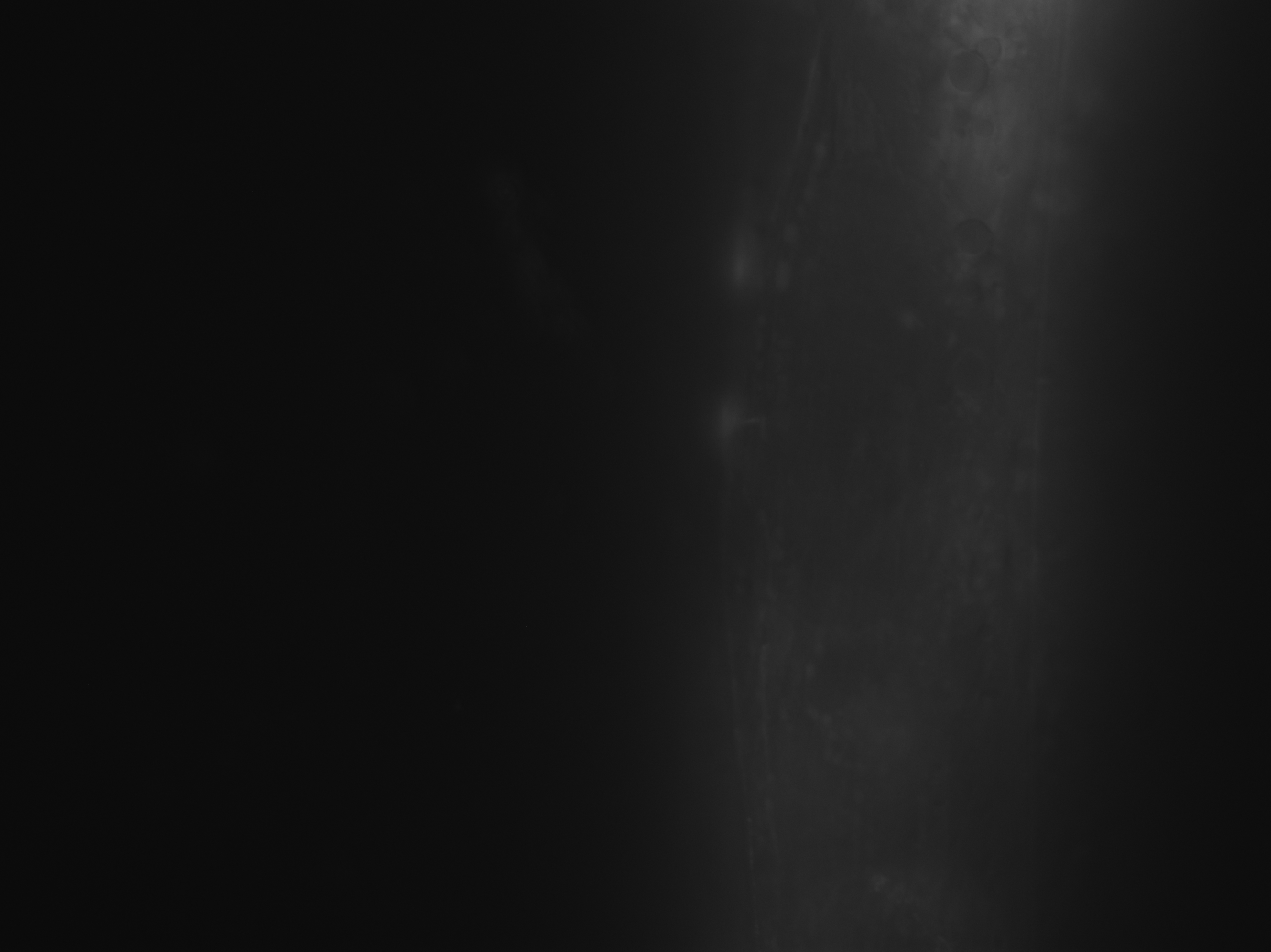

Supplement: Supplementary file 6 — Source data Fig. 5 [file 44319_2025_493_MOESM6_ESM.zip › Figure5/Fig5F/Experiment-70_synapse_skipped.tif_files/Experiment-70_z0c0x0-1388y0-1040.tif]

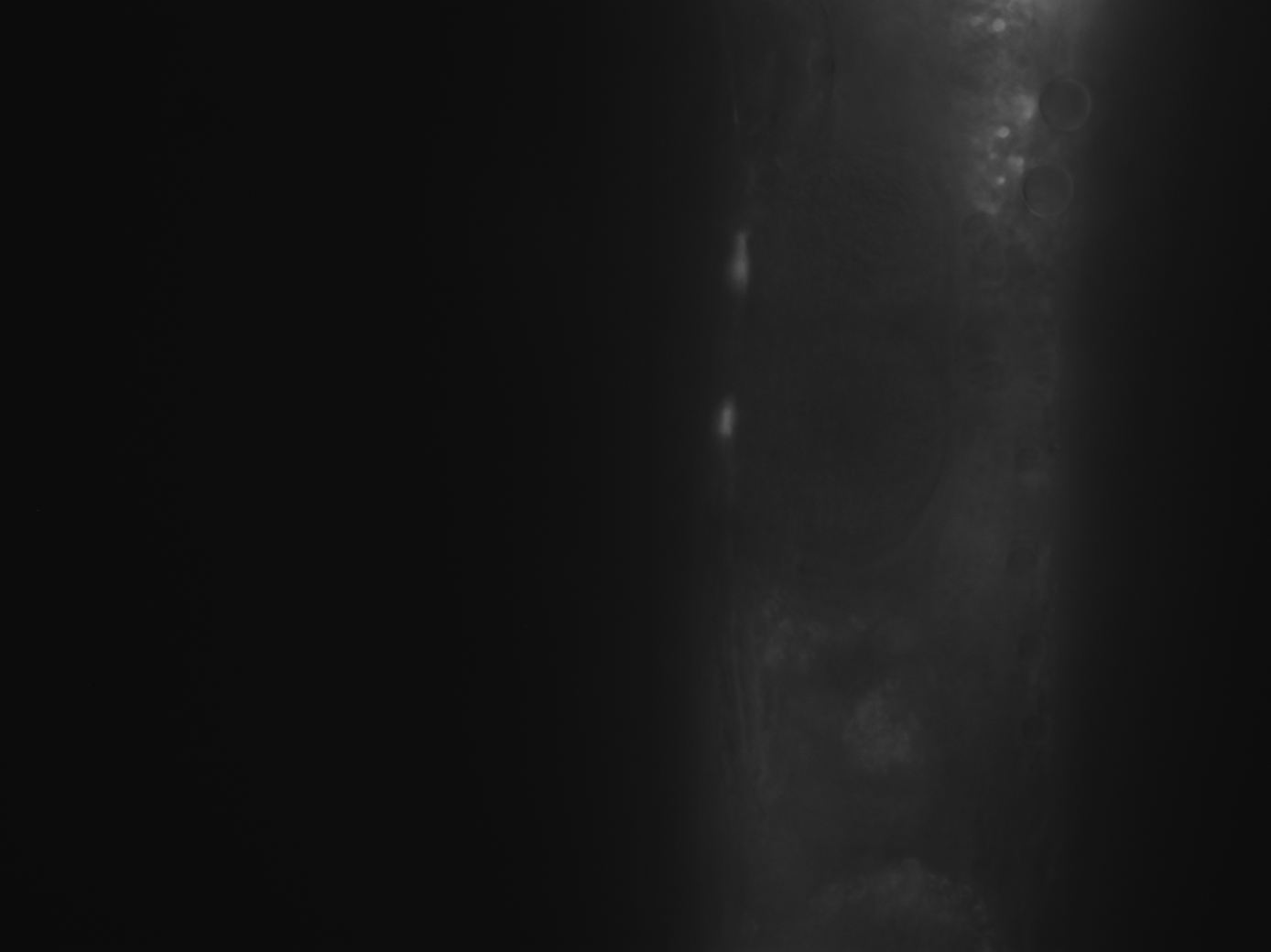

Supplement: Supplementary file 6 — Source data Fig. 5 [file 44319_2025_493_MOESM6_ESM.zip › Figure5/Fig5F/Experiment-70_synapse_skipped.tif_files/Experiment-70_z6c0x0-1388y0-1040.tif]

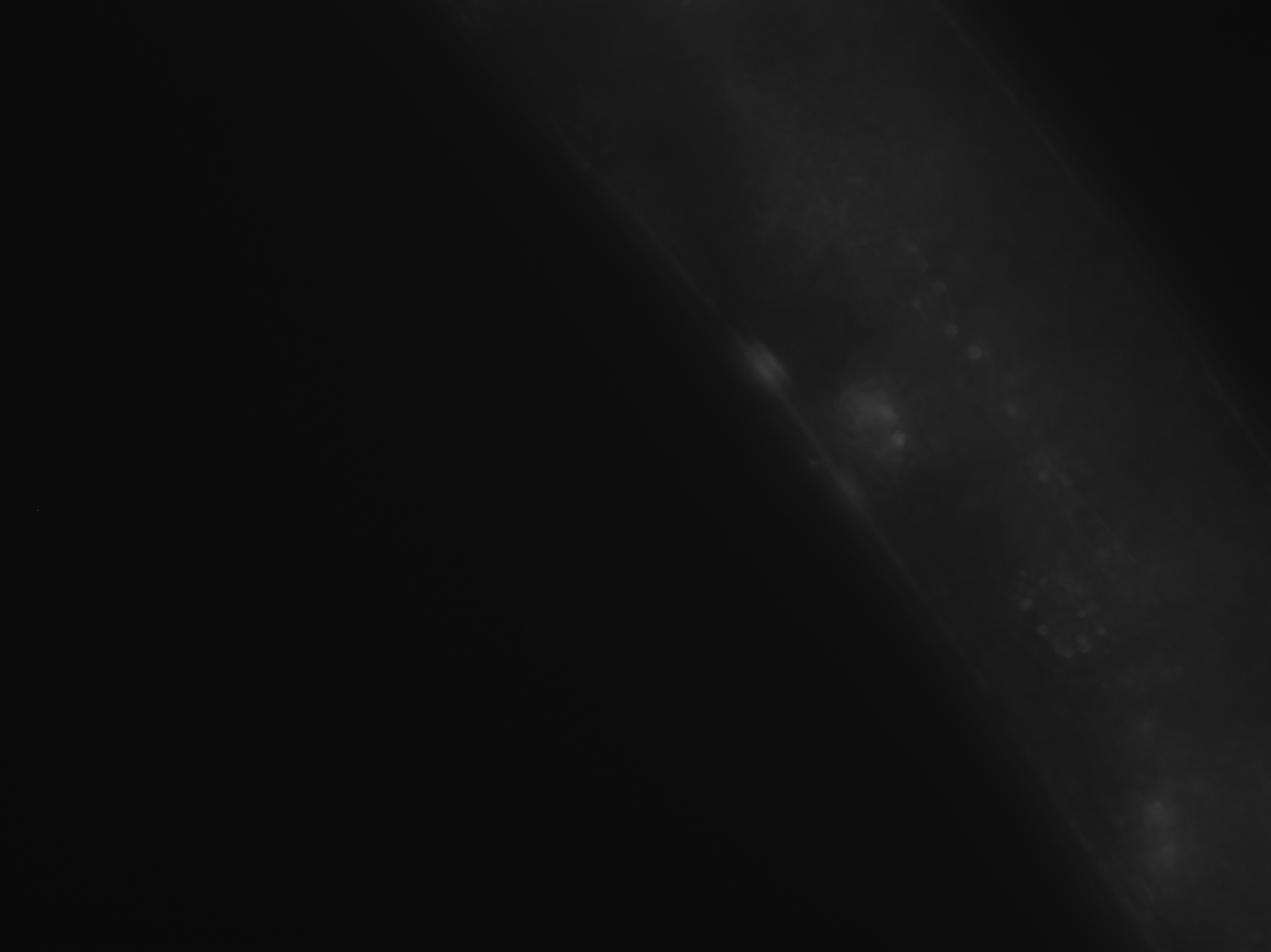

Supplement: Supplementary file 6 — Source data Fig. 5 [file 44319_2025_493_MOESM6_ESM.zip › Figure5/Fig5F/Experiment-46_synapse_wt.tif_files/Experiment-46_z8c0x0-1388y0-1040.tif]

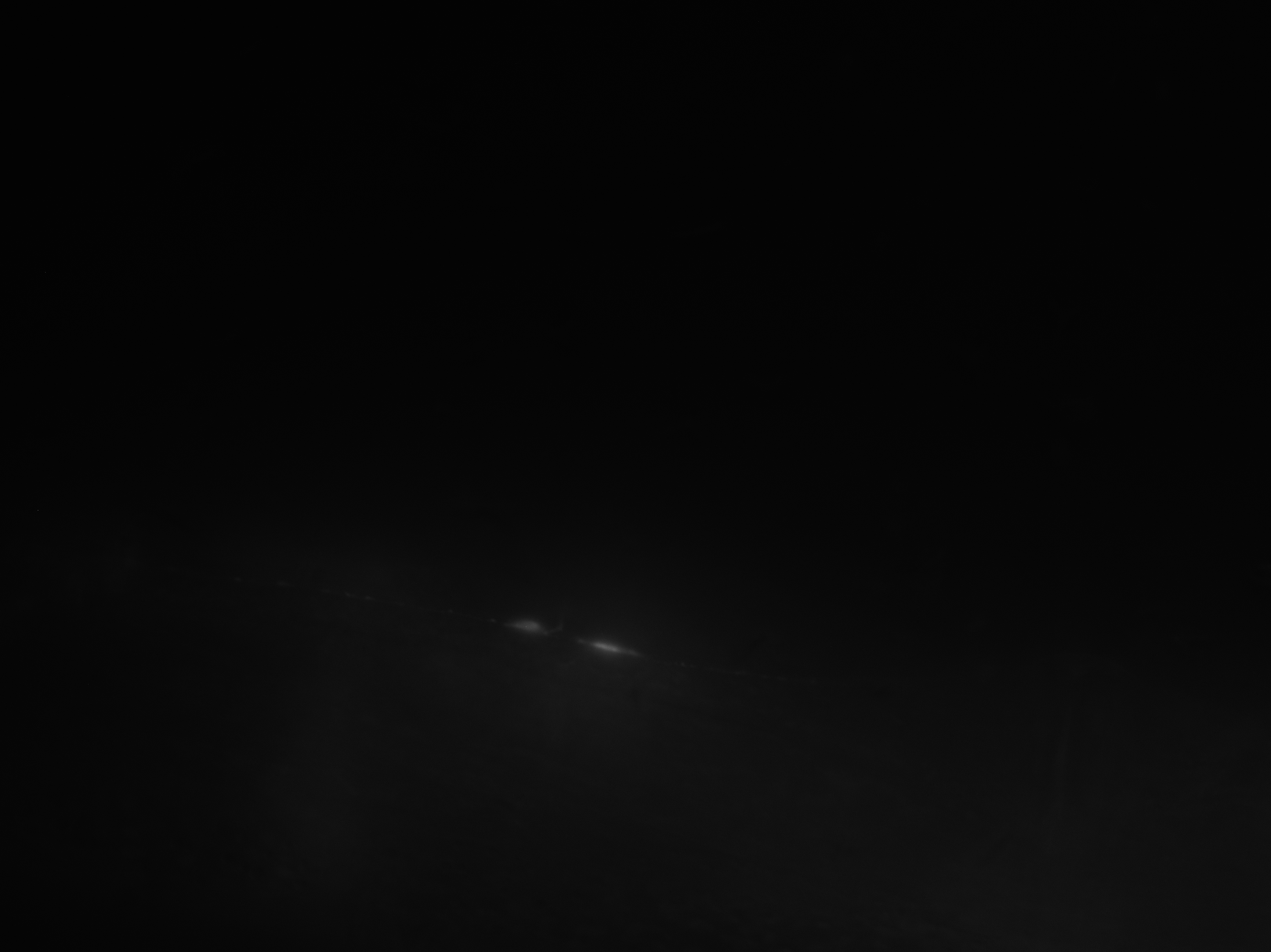

Supplement: Supplementary file 6 — Source data Fig. 5 [file 44319_2025_493_MOESM6_ESM.zip › Figure5/Fig5F/Experiment-979_synapse_n2813.tif_files/Experiment-979_z3c0x0-1388y0-1040.tif]

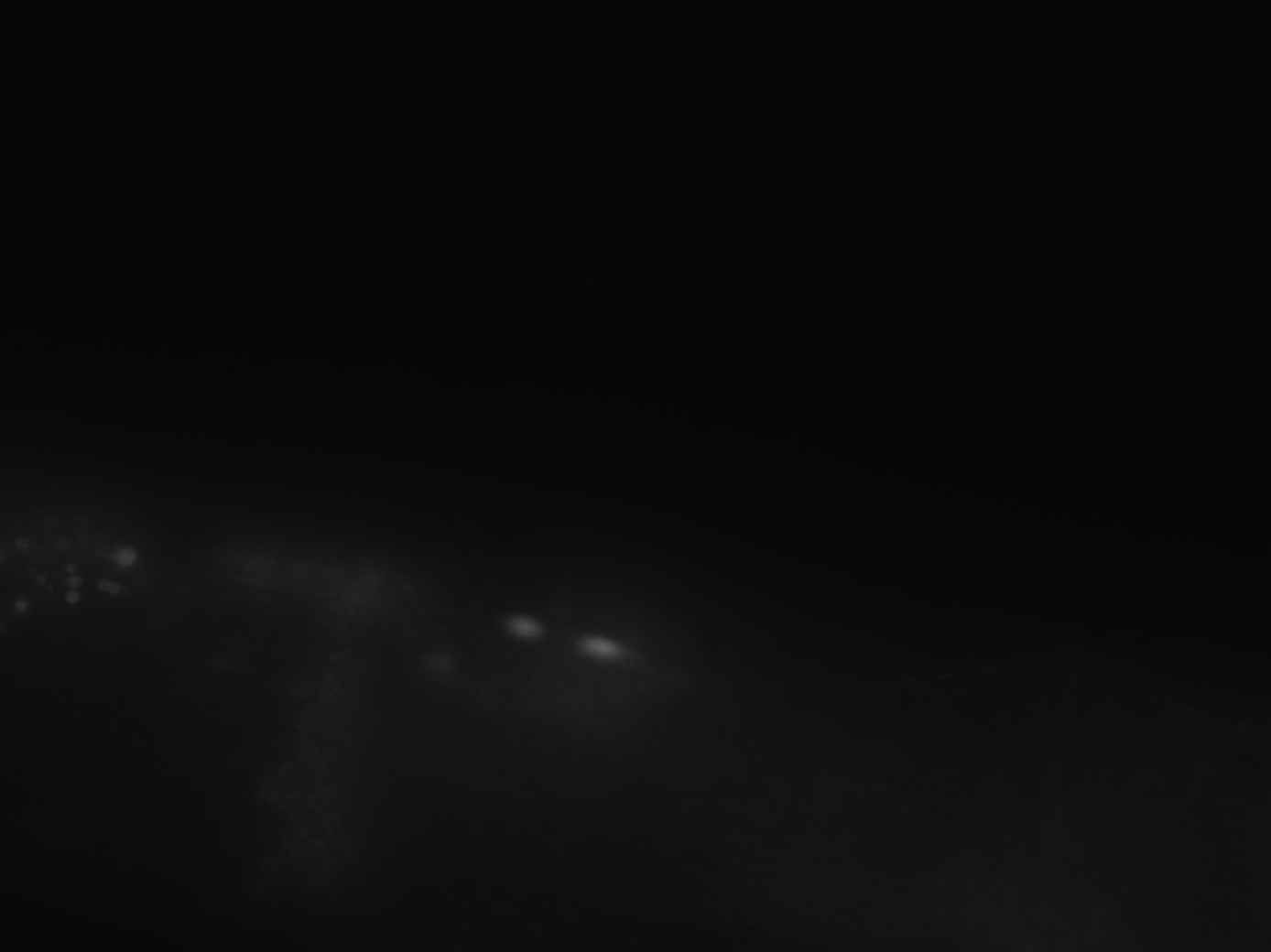

Supplement: Supplementary file 6 — Source data Fig. 5 [file 44319_2025_493_MOESM6_ESM.zip › Figure5/Fig5F/Experiment-979_synapse_n2813.tif_files/Experiment-979_z7c0x0-1388y0-1040.tif]

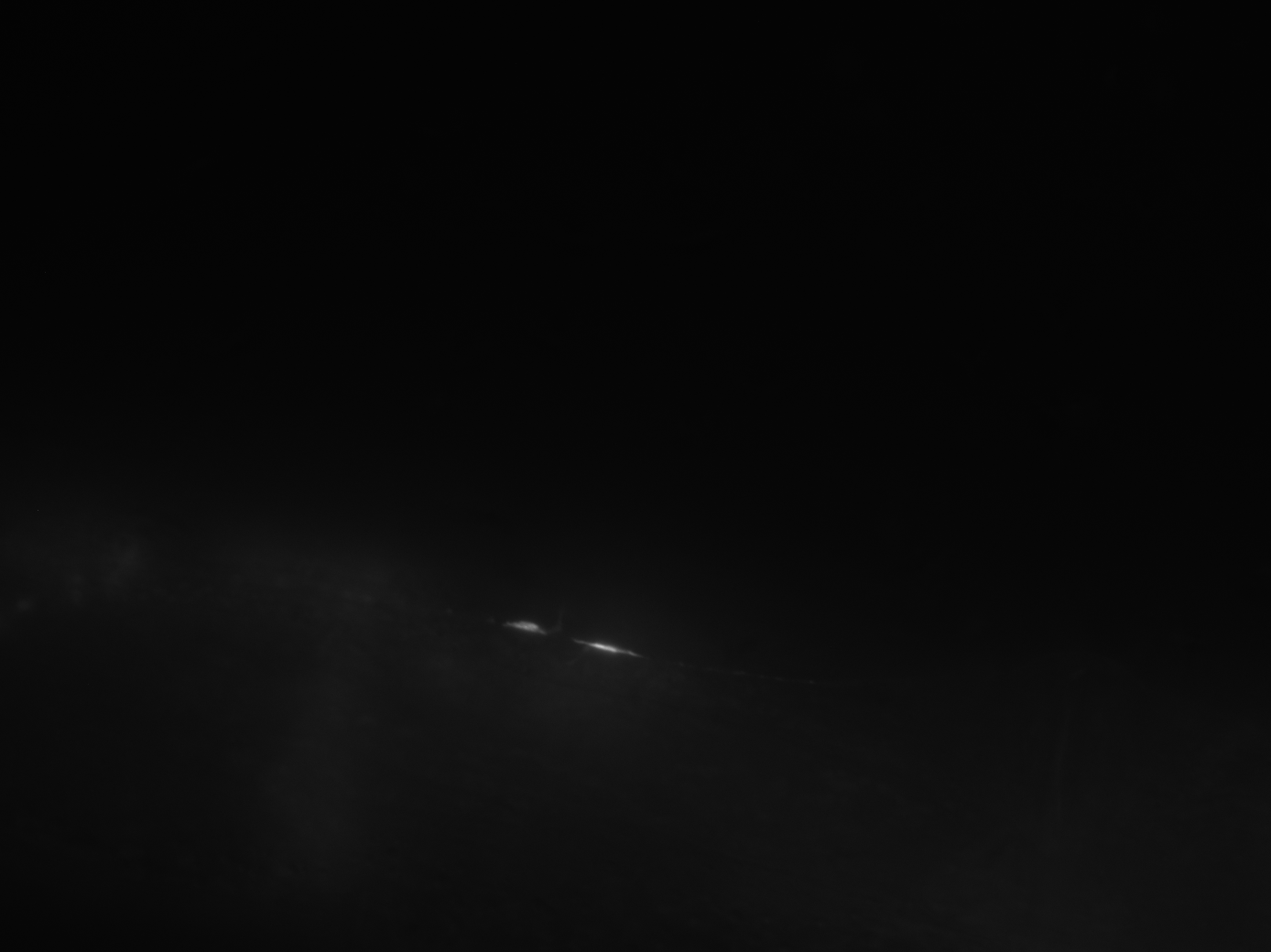

Supplement: Supplementary file 6 — Source data Fig. 5 [file 44319_2025_493_MOESM6_ESM.zip › Figure5/Fig5F/Experiment-979_synapse_n2813.tif_files/Experiment-979_z4c0x0-1388y0-1040.tif]

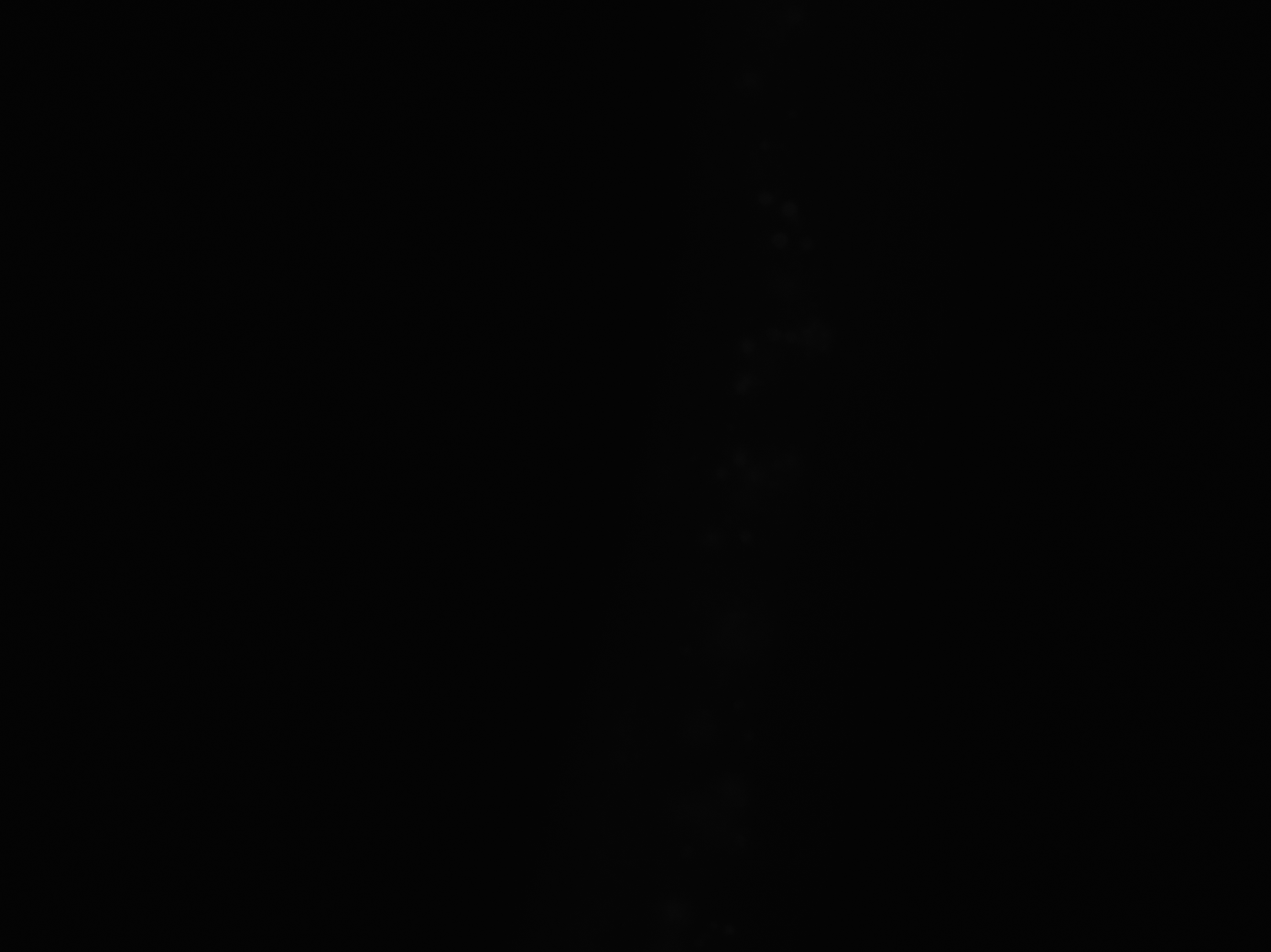

Supplement: Supplementary file 6 — Source data Fig. 5 [file 44319_2025_493_MOESM6_ESM.zip › Figure5/Fig5E/goodALM.tif_files/goodALM_z1c1x0-1388y0-1040.tif]

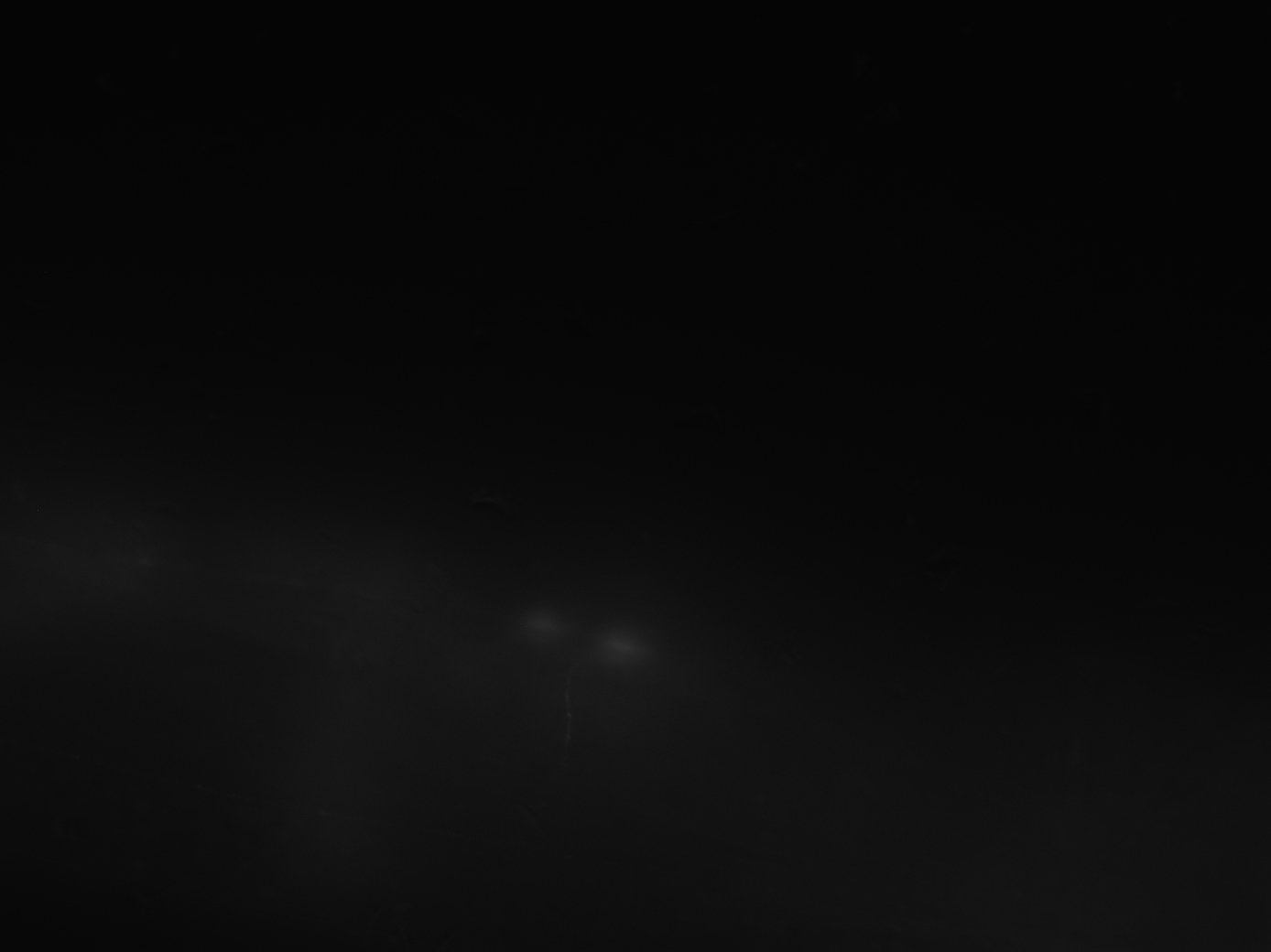

Supplement: Supplementary file 6 — Source data Fig. 5 [file 44319_2025_493_MOESM6_ESM.zip › Figure5/Fig5F/Experiment-979_synapse_n2813.tif_files/Experiment-979_z0c0x0-1388y0-1040.tif]

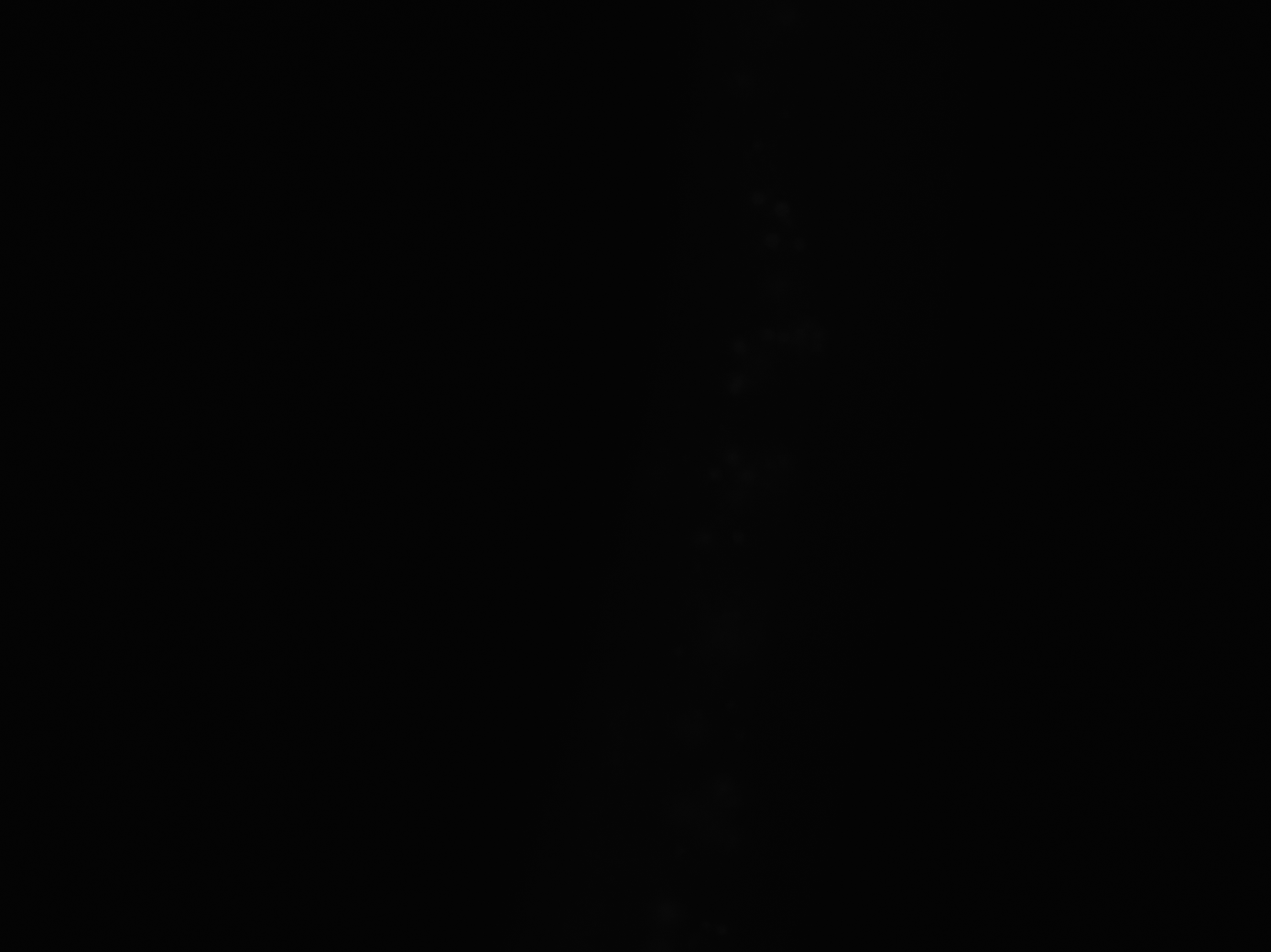

Supplement: Supplementary file 6 — Source data Fig. 5 [file 44319_2025_493_MOESM6_ESM.zip › Figure5/Fig5E/goodALM.tif_files/goodALM_z0c1x0-1388y0-1040.tif]

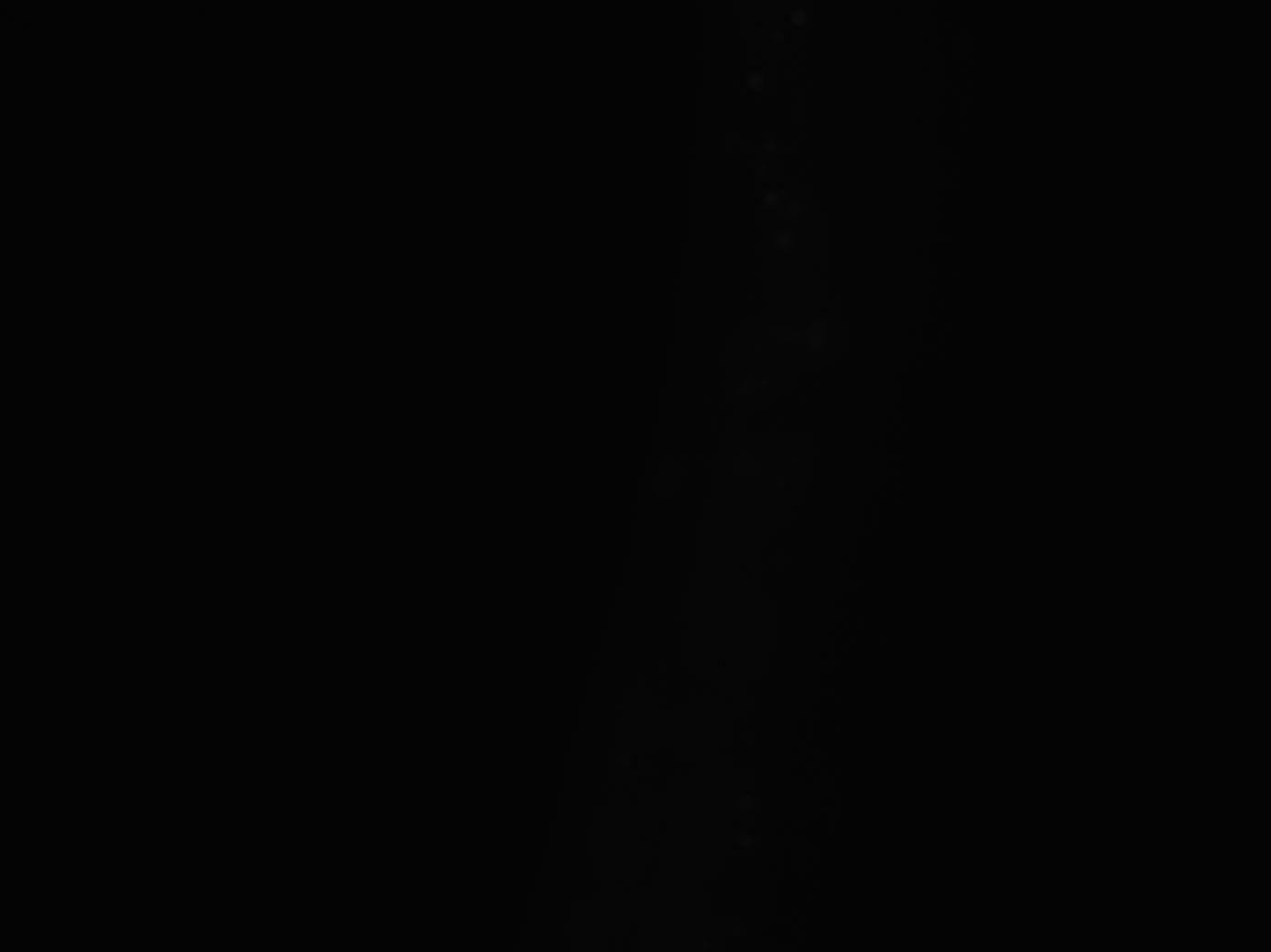

Supplement: Supplementary file 6 — Source data Fig. 5 [file 44319_2025_493_MOESM6_ESM.zip › Figure5/Fig5E/goodALM.tif_files/goodALM_z3c1x0-1388y0-1040.tif]

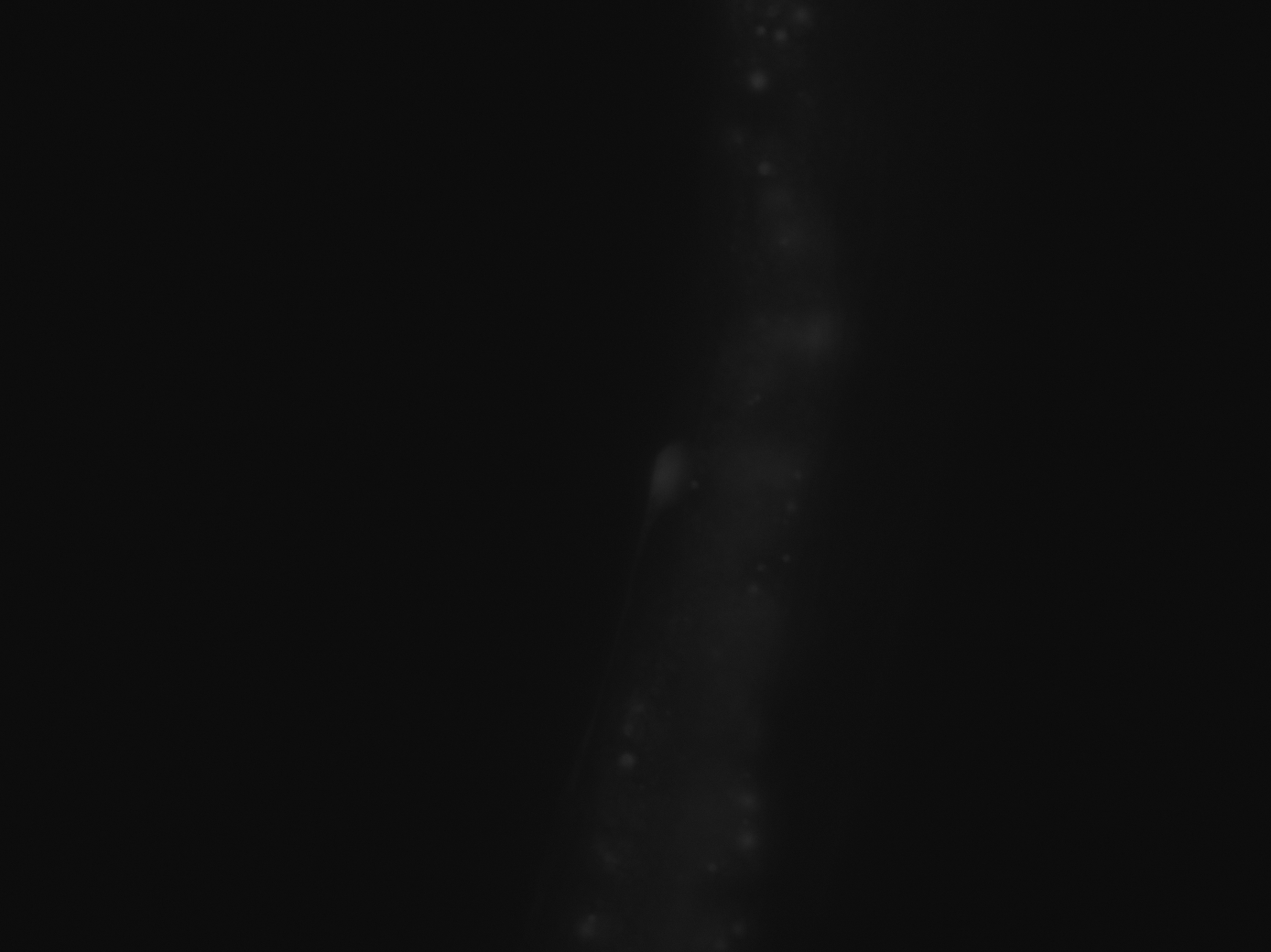

Supplement: Supplementary file 6 — Source data Fig. 5 [file 44319_2025_493_MOESM6_ESM.zip › Figure5/Fig5E/goodALM.tif_files/goodALM_z5c2x0-1388y0-1040.tif]

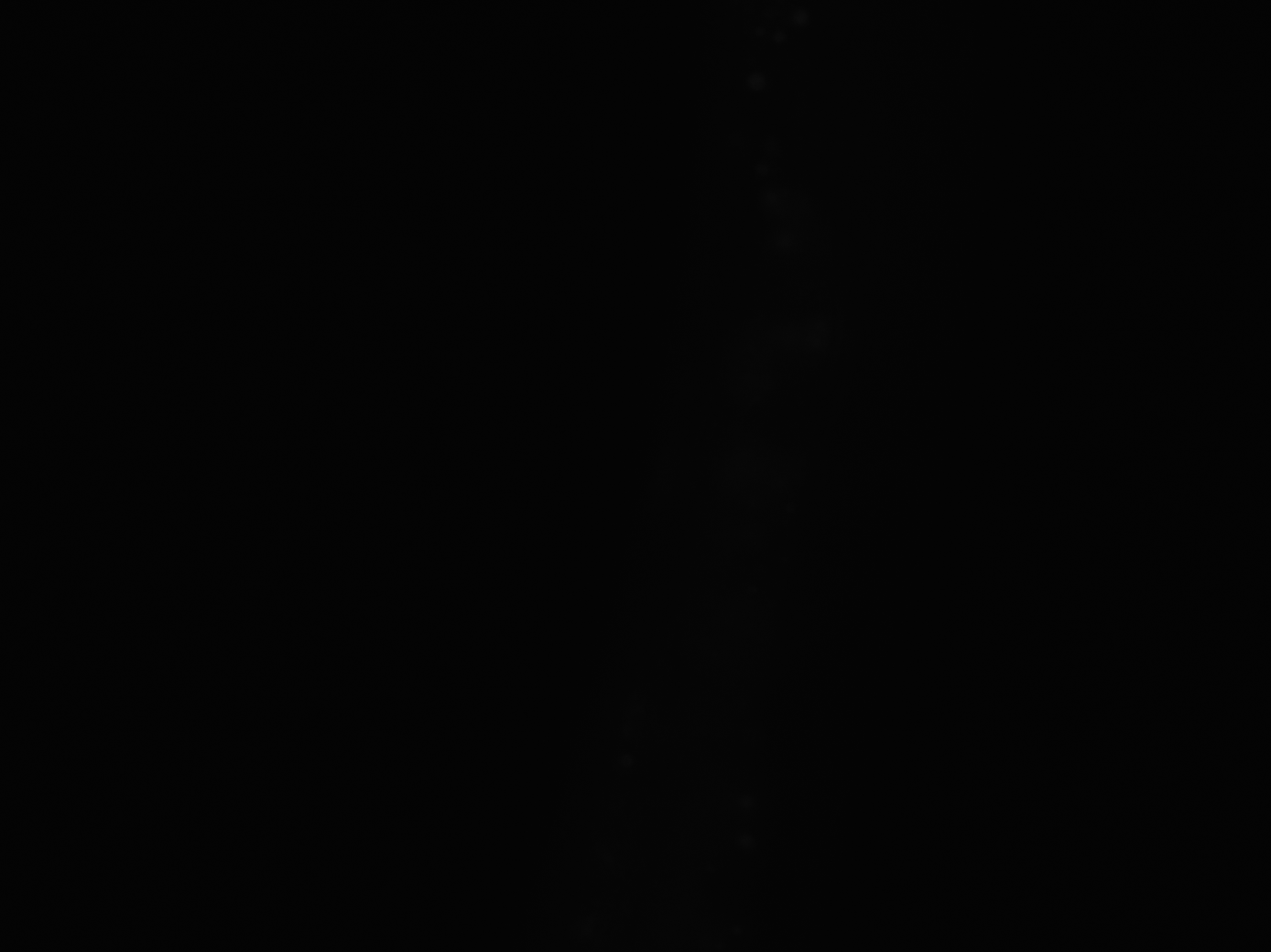

Supplement: Supplementary file 6 — Source data Fig. 5 [file 44319_2025_493_MOESM6_ESM.zip › Figure5/Fig5E/goodALM.tif_files/goodALM_z4c1x0-1388y0-1040.tif]

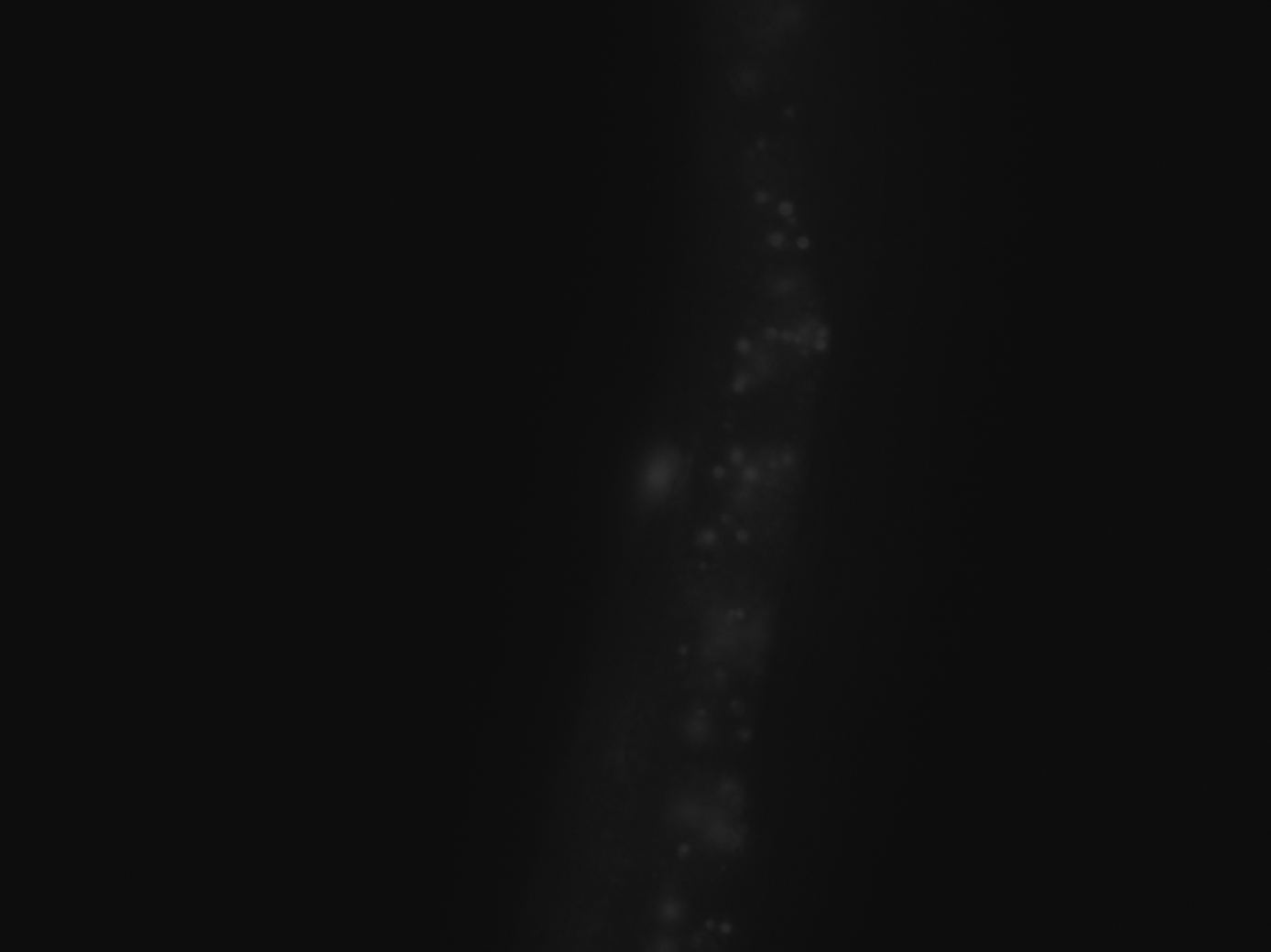

Supplement: Supplementary file 6 — Source data Fig. 5 [file 44319_2025_493_MOESM6_ESM.zip › Figure5/Fig5E/goodALM.tif_files/goodALM_z0c2x0-1388y0-1040.tif]

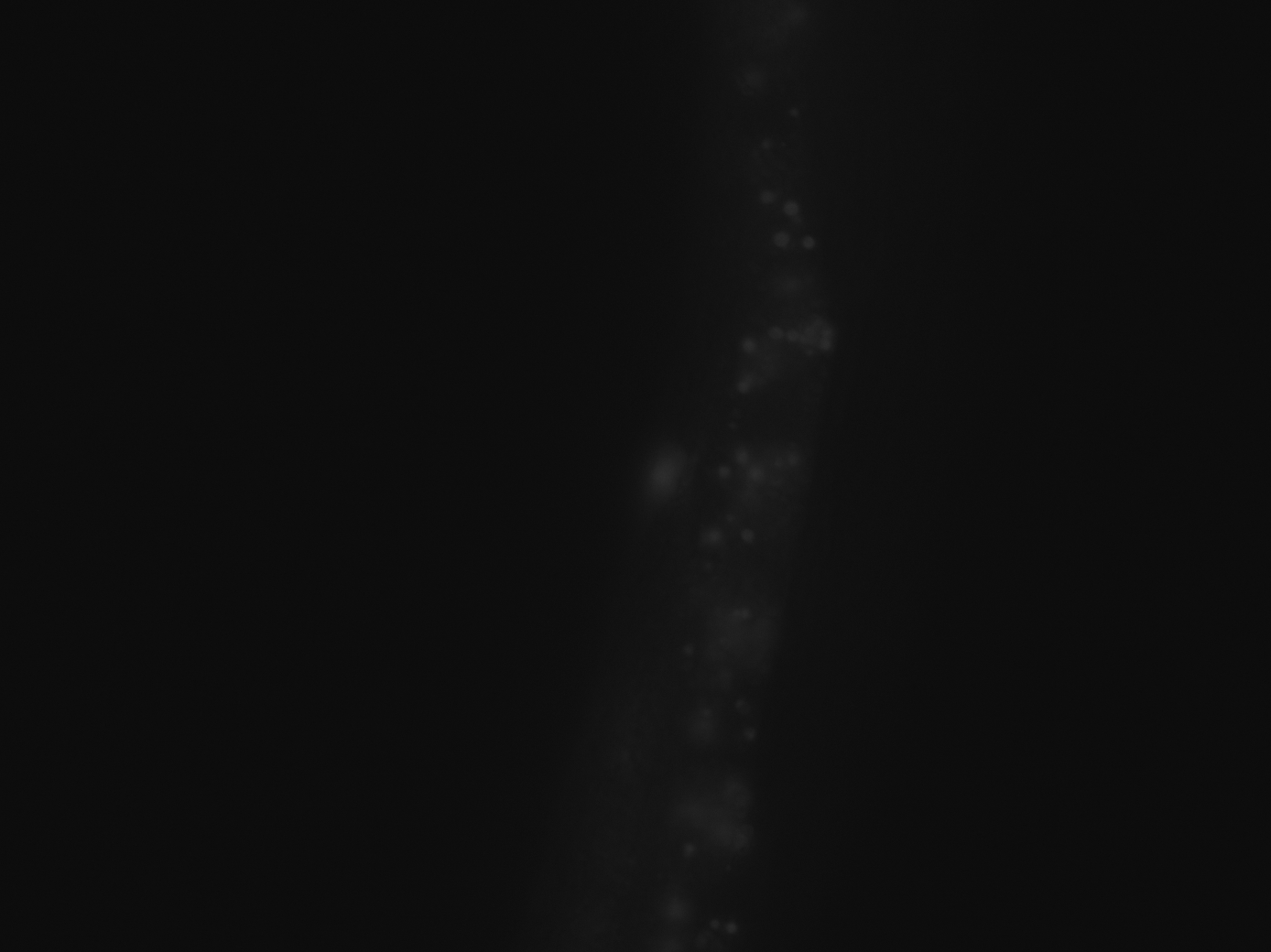

Supplement: Supplementary file 6 — Source data Fig. 5 [file 44319_2025_493_MOESM6_ESM.zip › Figure5/Fig5E/goodALM.tif_files/goodALM_z1c2x0-1388y0-1040.tif]

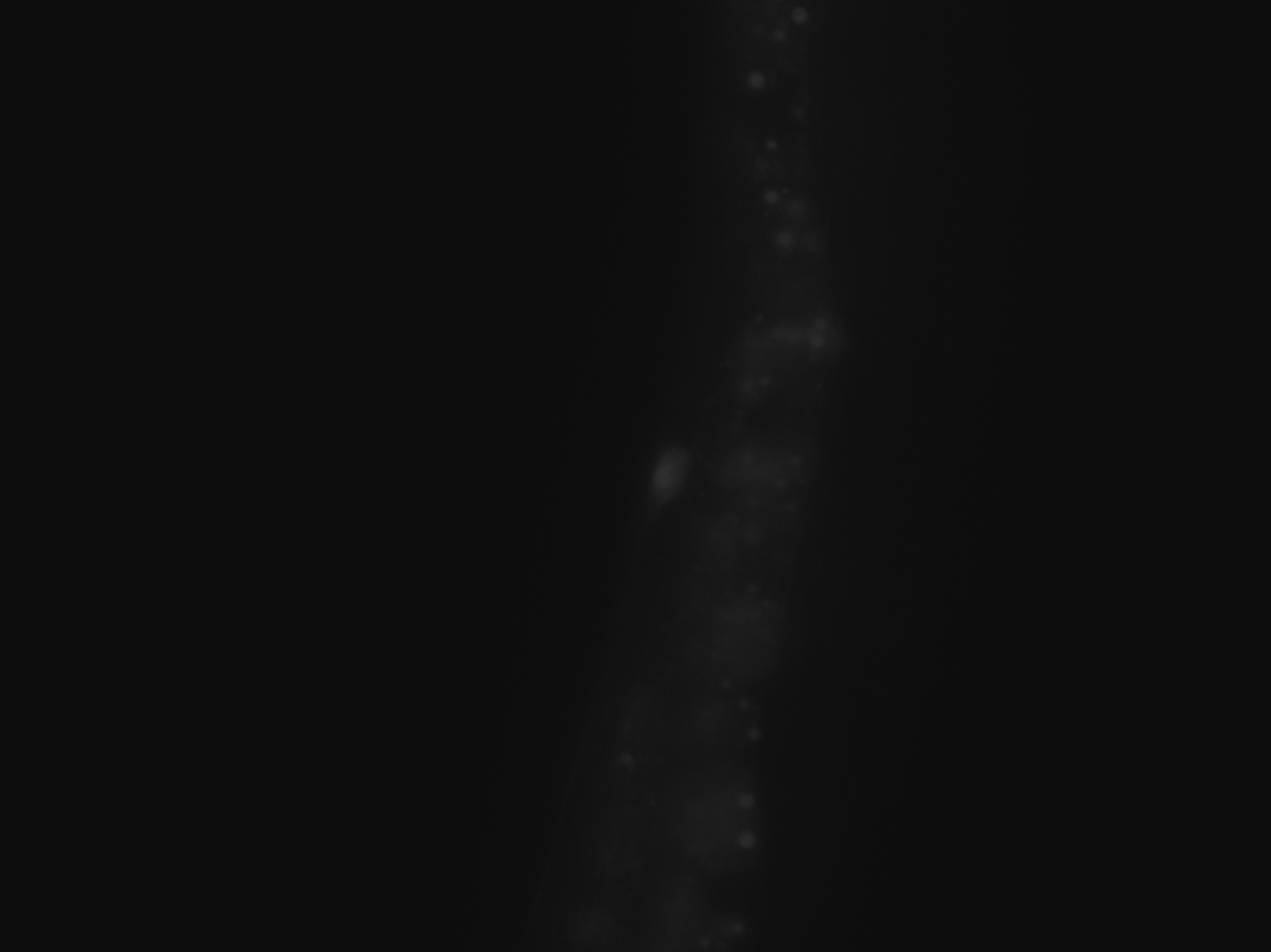

Supplement: Supplementary file 6 — Source data Fig. 5 [file 44319_2025_493_MOESM6_ESM.zip › Figure5/Fig5E/goodALM.tif_files/goodALM_z3c2x0-1388y0-1040.tif]

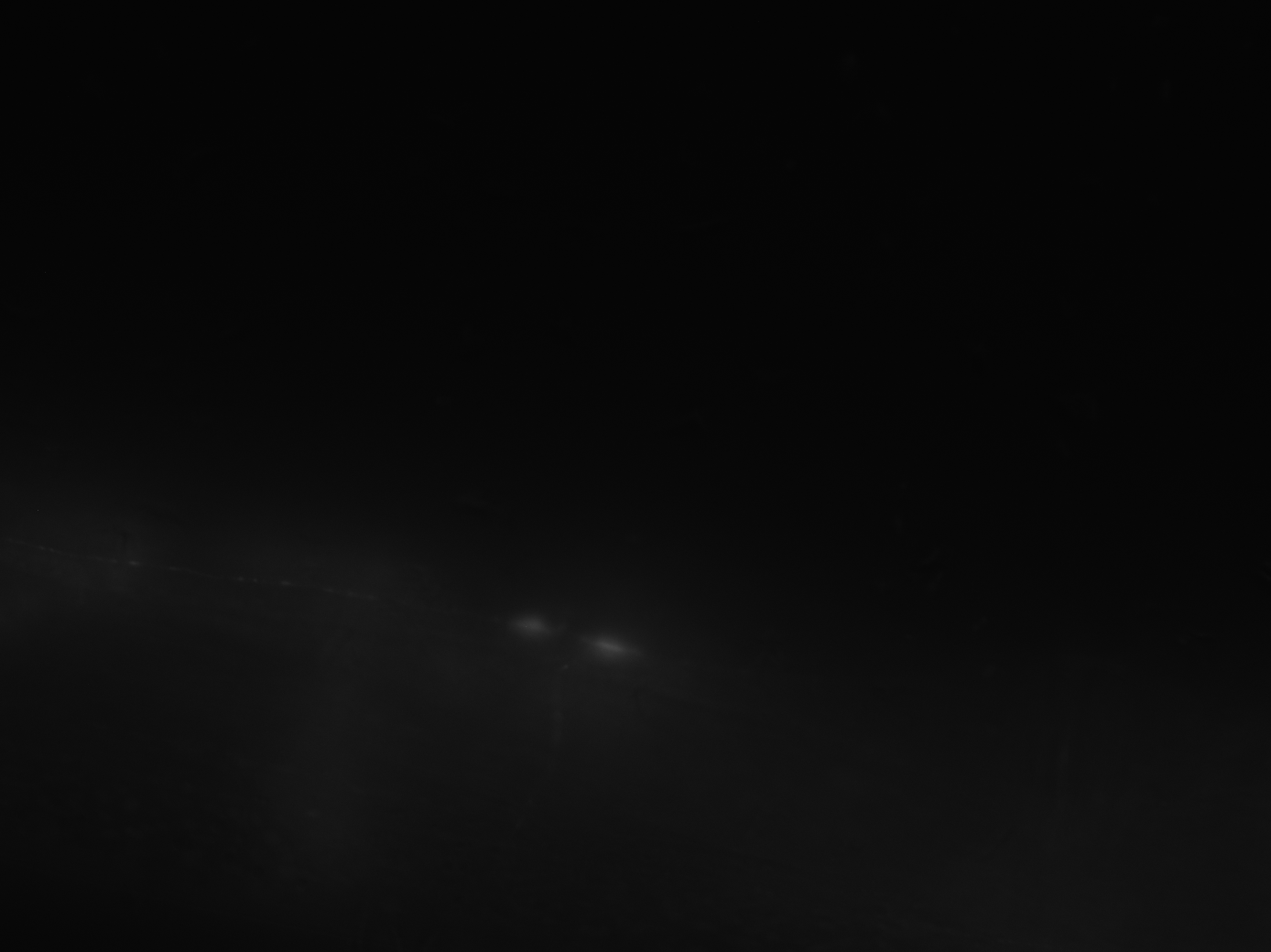

Supplement: Supplementary file 6 — Source data Fig. 5 [file 44319_2025_493_MOESM6_ESM.zip › Figure5/Fig5F/Experiment-979_synapse_n2813.tif_files/Experiment-979_z2c0x0-1388y0-1040.tif]

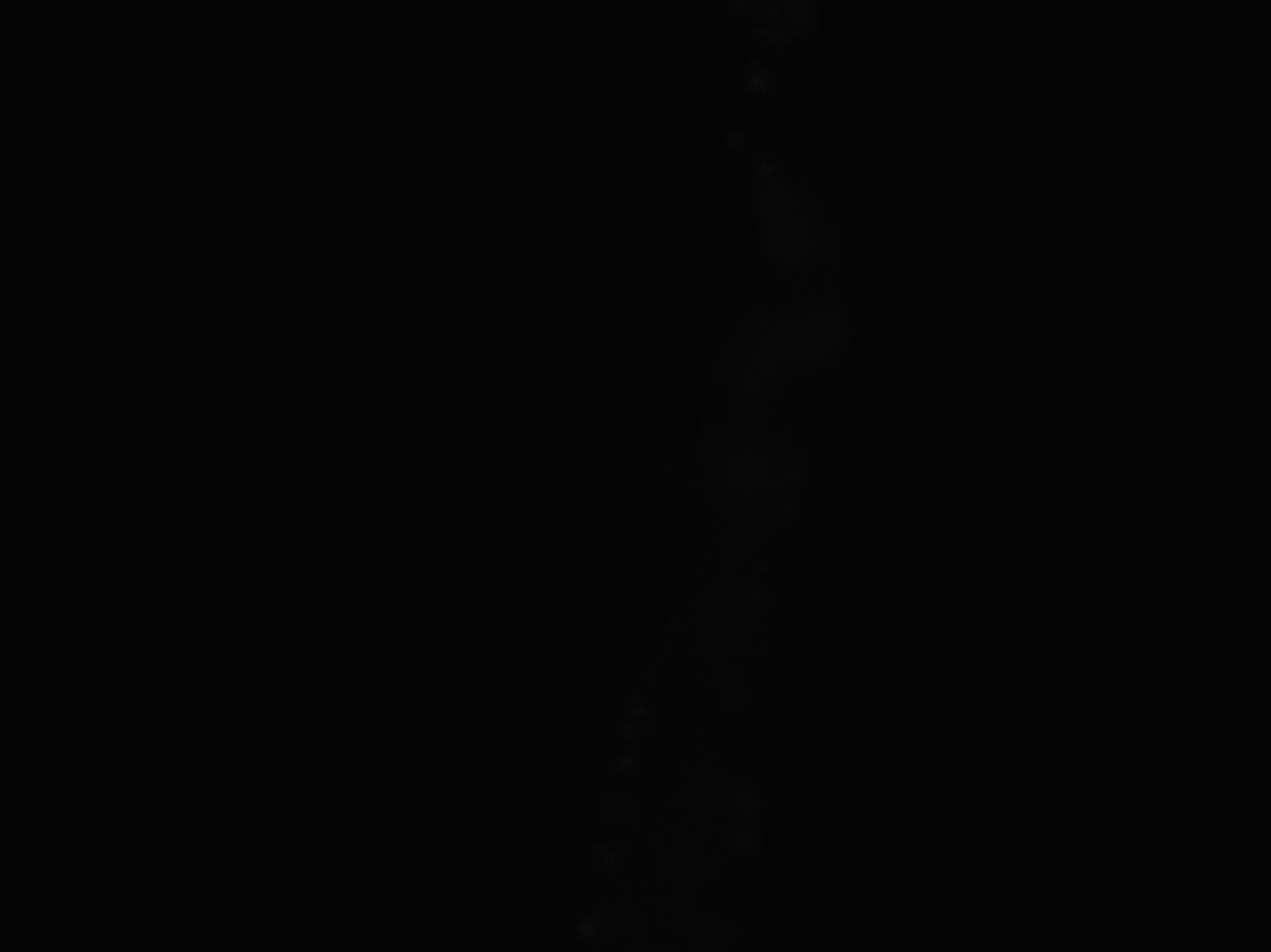

Supplement: Supplementary file 6 — Source data Fig. 5 [file 44319_2025_493_MOESM6_ESM.zip › Figure5/Fig5E/goodALM.tif_files/goodALM_z6c1x0-1388y0-1040.tif]

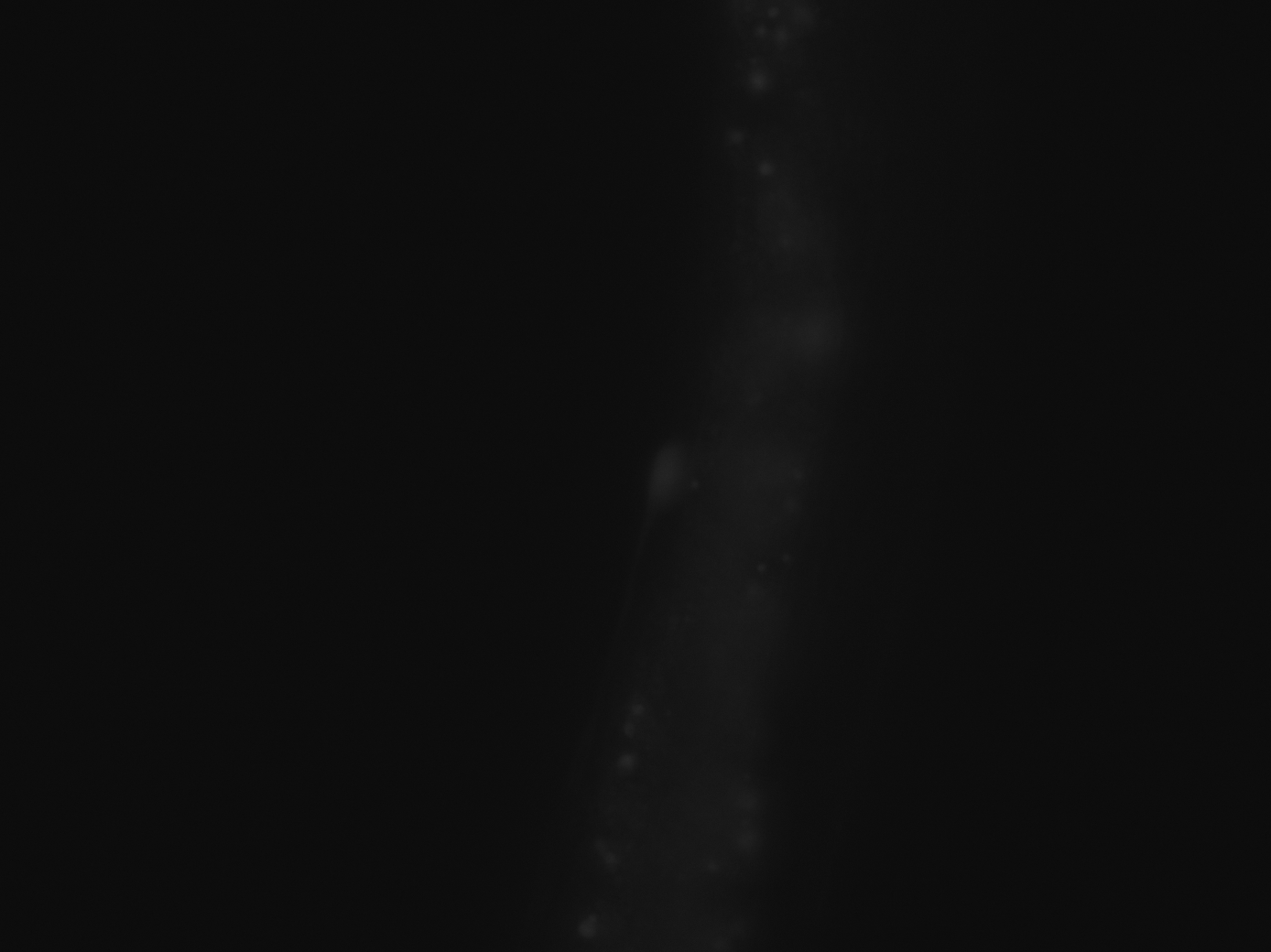

Supplement: Supplementary file 6 — Source data Fig. 5 [file 44319_2025_493_MOESM6_ESM.zip › Figure5/Fig5E/goodALM.tif_files/goodALM_z6c2x0-1388y0-1040.tif]

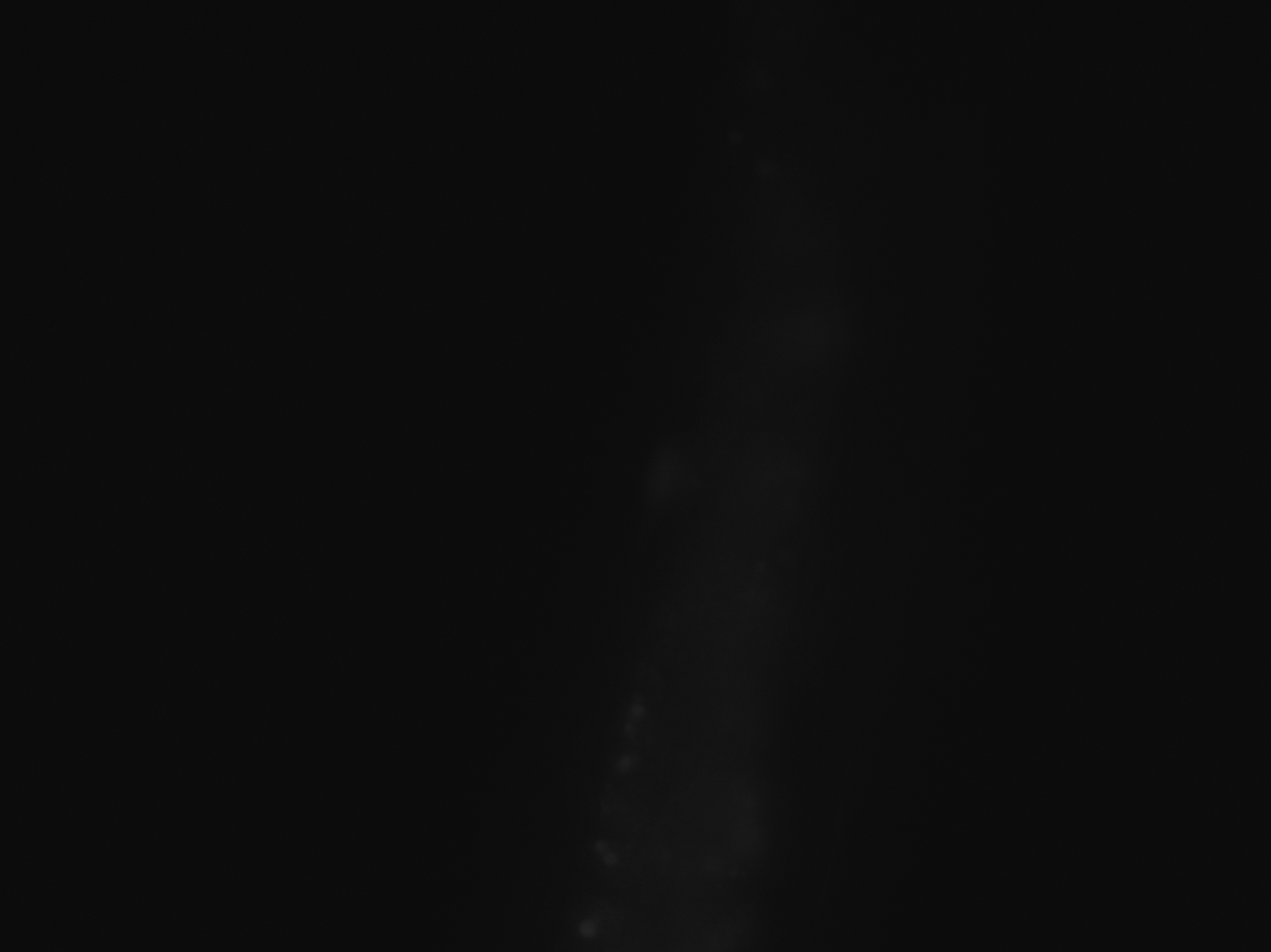

Supplement: Supplementary file 6 — Source data Fig. 5 [file 44319_2025_493_MOESM6_ESM.zip › Figure5/Fig5E/goodALM.tif_files/goodALM_z7c2x0-1388y0-1040.tif]

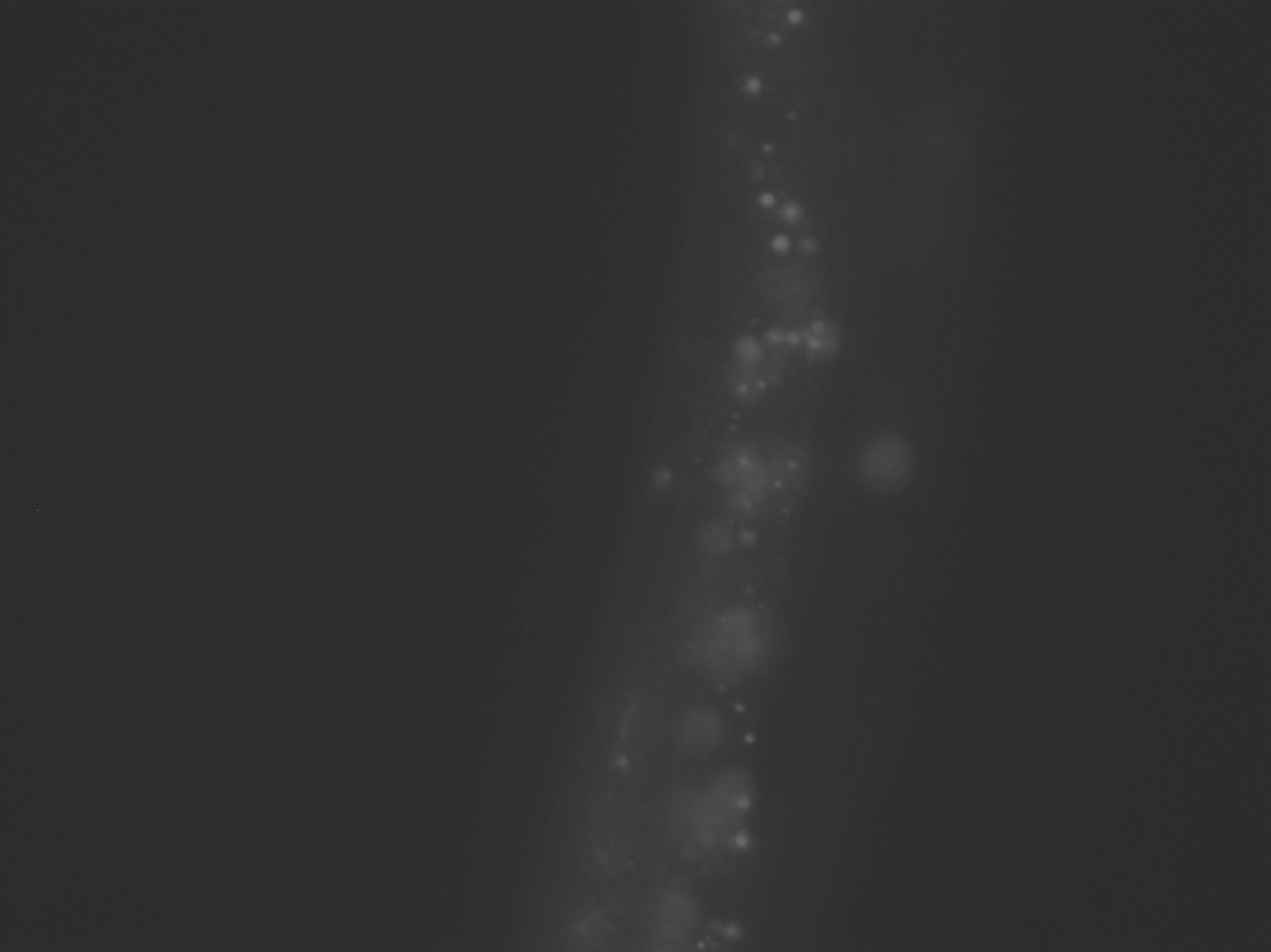

Supplement: Supplementary file 6 — Source data Fig. 5 [file 44319_2025_493_MOESM6_ESM.zip › Figure5/Fig5E/goodALM.tif_files/goodALM_z2c0x0-1388y0-1040.tif]

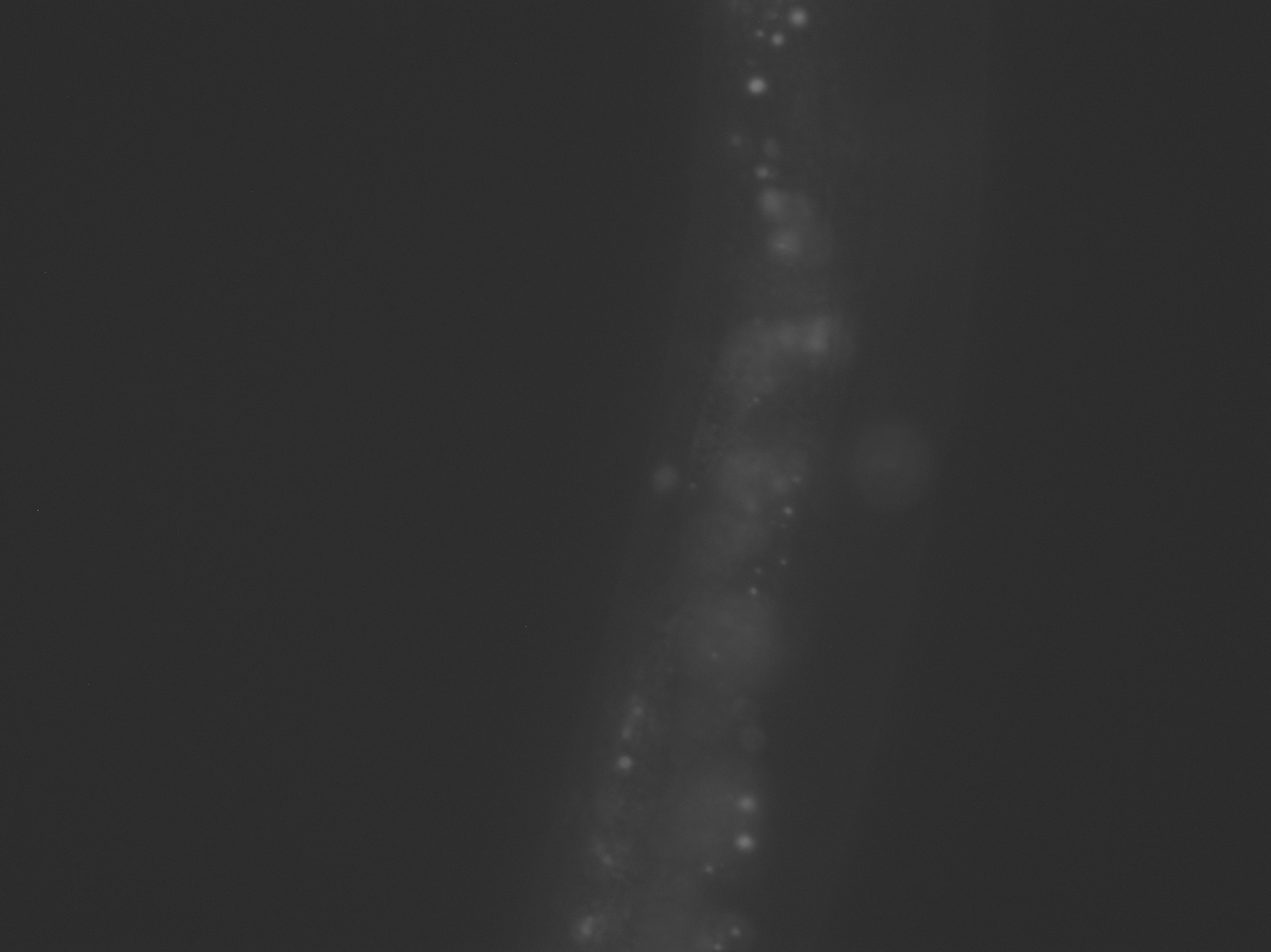

Supplement: Supplementary file 6 — Source data Fig. 5 [file 44319_2025_493_MOESM6_ESM.zip › Figure5/Fig5E/goodALM.tif_files/goodALM_z4c0x0-1388y0-1040.tif]

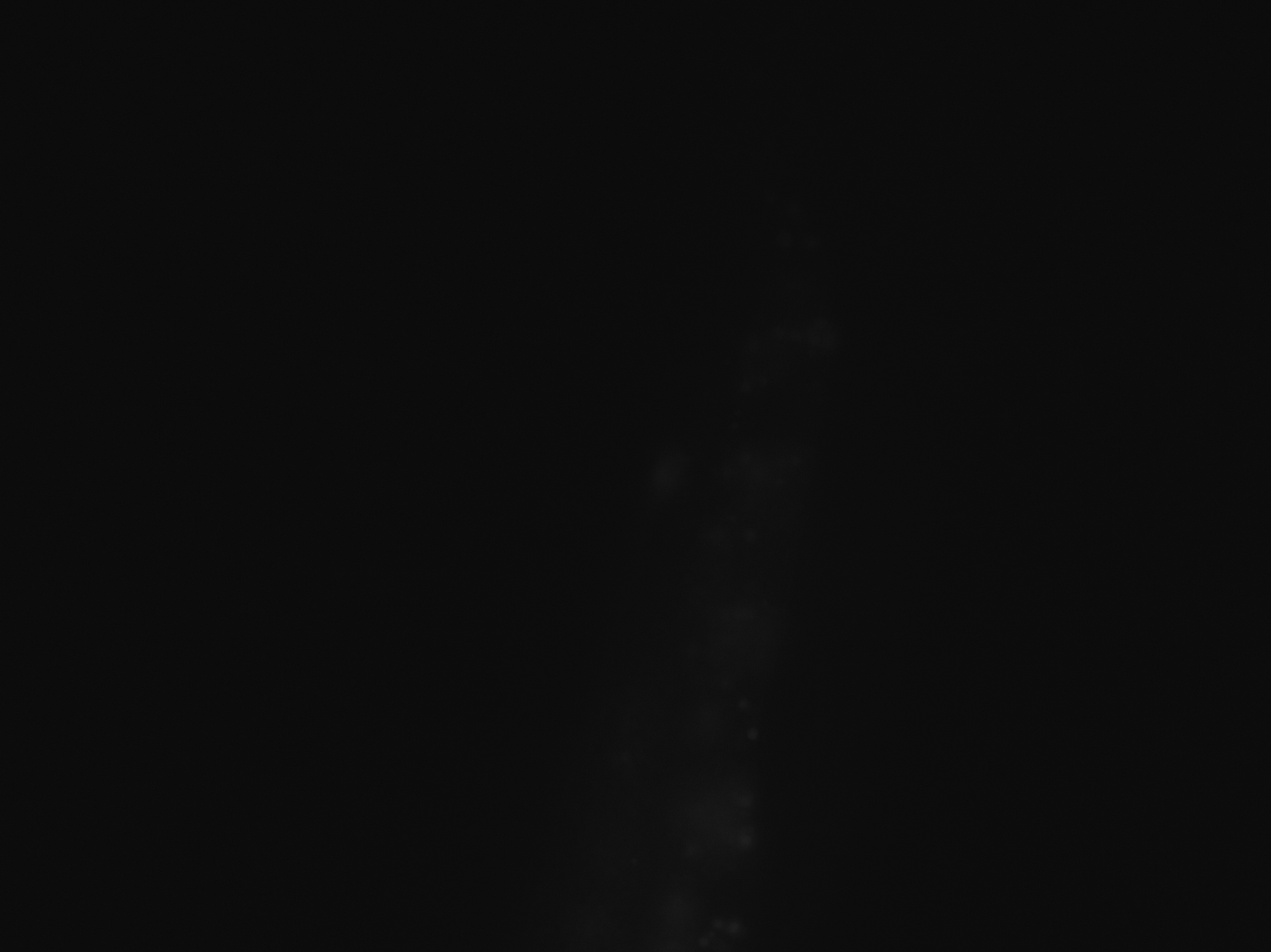

Supplement: Supplementary file 6 — Source data Fig. 5 [file 44319_2025_493_MOESM6_ESM.zip › Figure5/Fig5E/goodALM.tif_files/goodALM_z2c2x0-1388y0-1040.tif]

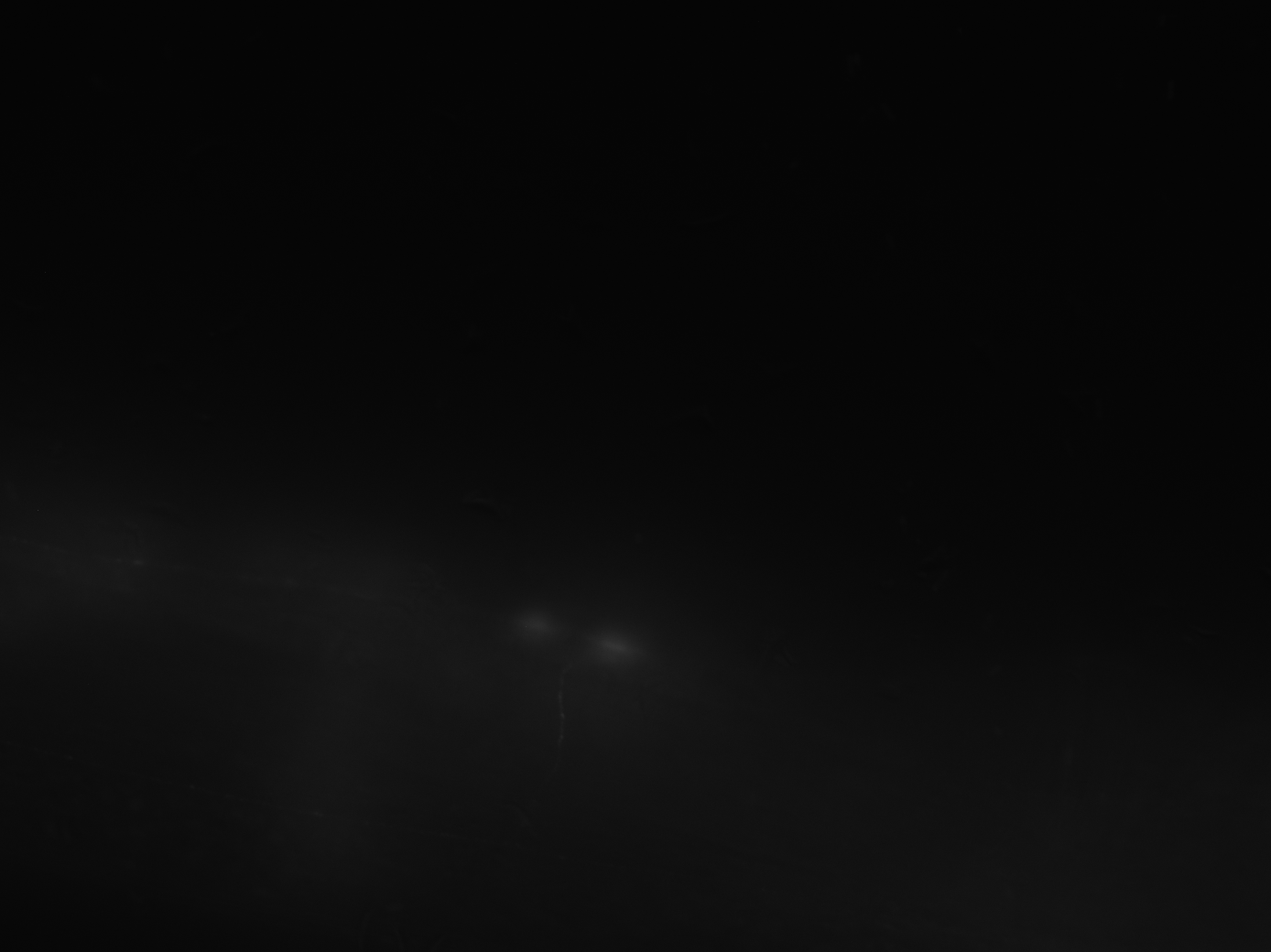

Supplement: Supplementary file 6 — Source data Fig. 5 [file 44319_2025_493_MOESM6_ESM.zip › Figure5/Fig5F/Experiment-979_synapse_n2813.tif_files/Experiment-979_z1c0x0-1388y0-1040.tif]

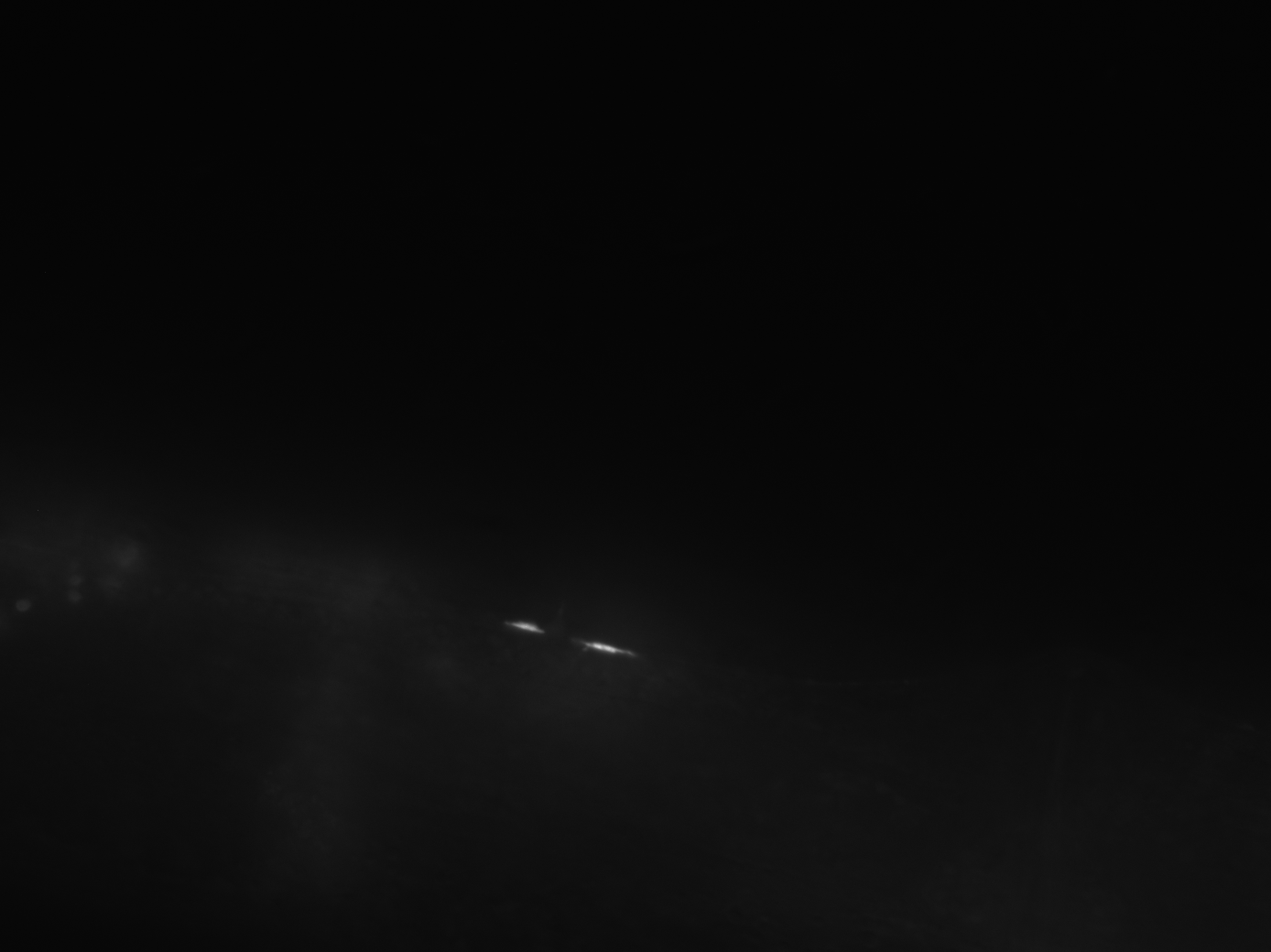

Supplement: Supplementary file 6 — Source data Fig. 5 [file 44319_2025_493_MOESM6_ESM.zip › Figure5/Fig5F/Experiment-979_synapse_n2813.tif_files/Experiment-979_z5c0x0-1388y0-1040.tif]

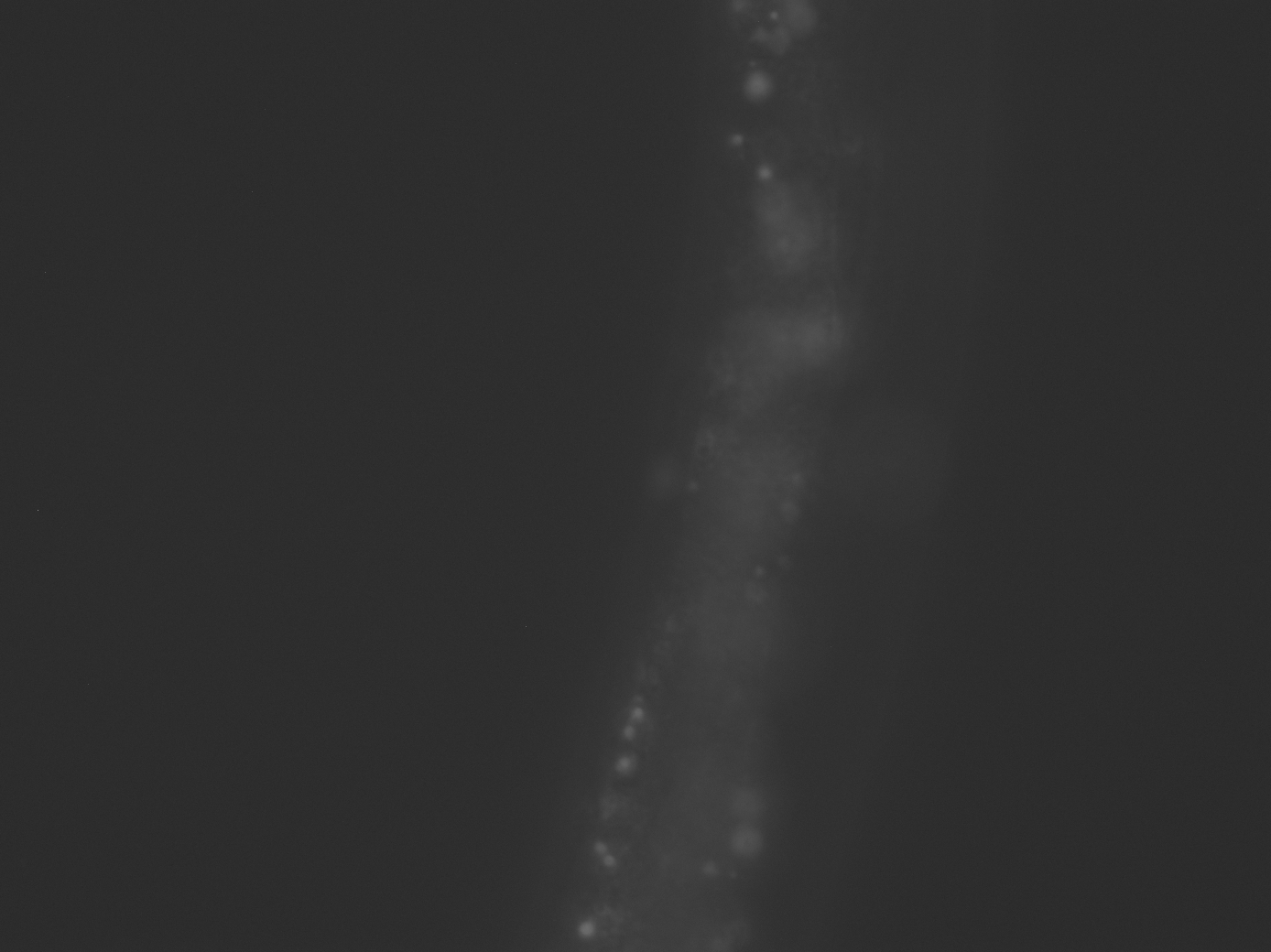

Supplement: Supplementary file 6 — Source data Fig. 5 [file 44319_2025_493_MOESM6_ESM.zip › Figure5/Fig5E/goodALM.tif_files/goodALM_z6c0x0-1388y0-1040.tif]

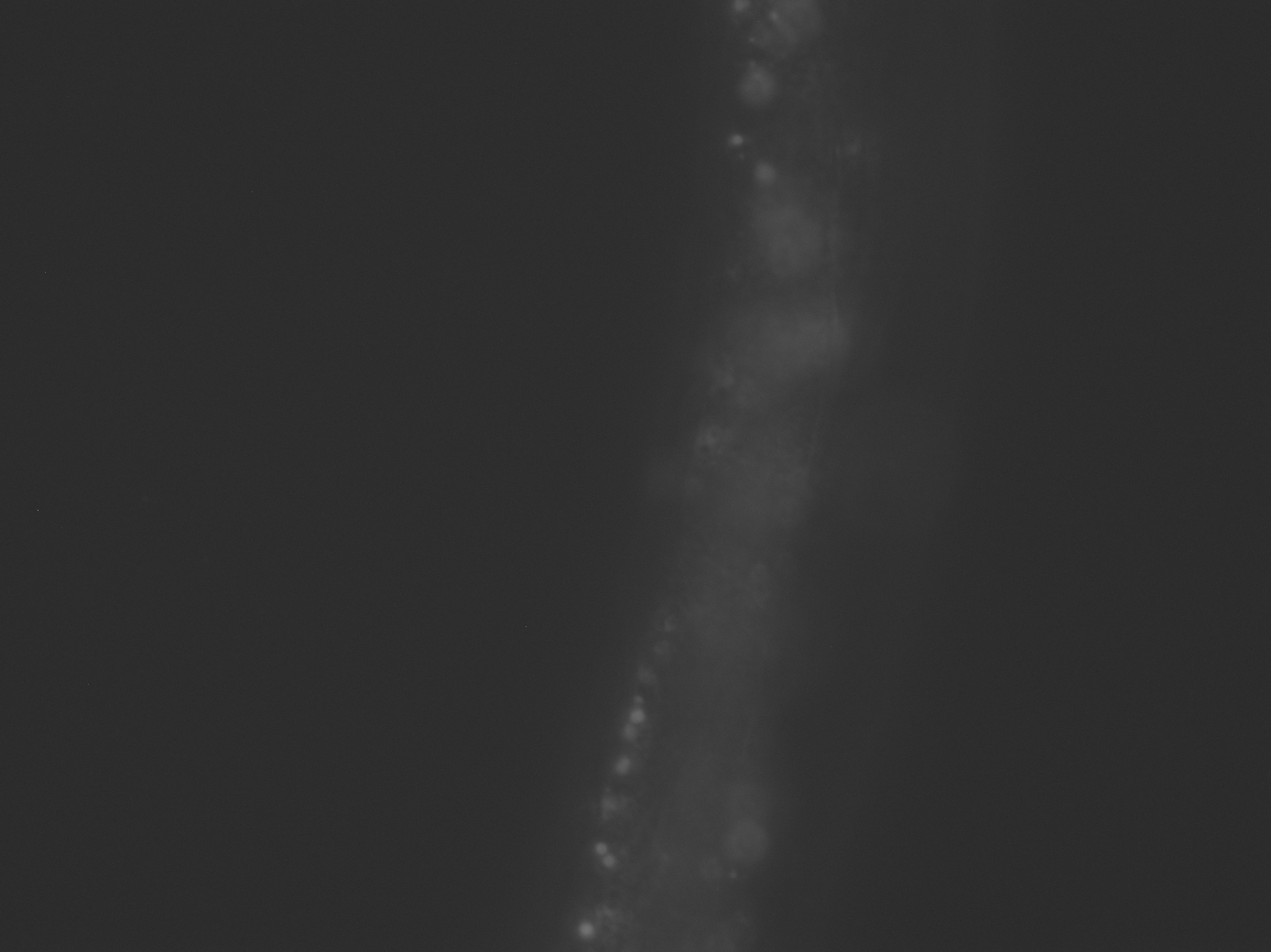

Supplement: Supplementary file 6 — Source data Fig. 5 [file 44319_2025_493_MOESM6_ESM.zip › Figure5/Fig5E/goodALM.tif_files/goodALM_z7c0x0-1388y0-1040.tif]

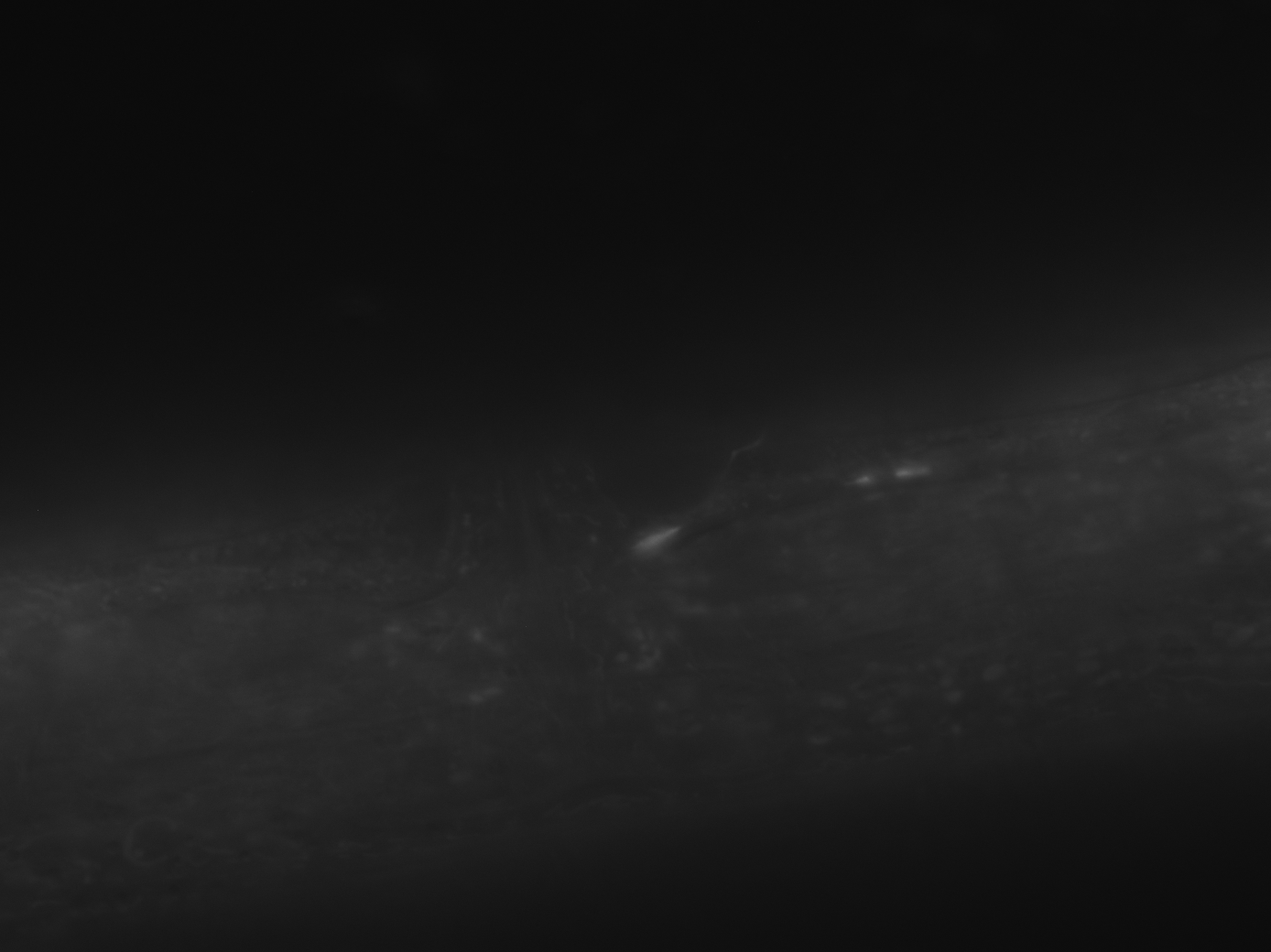

Supplement: Supplementary file 6 — Source data Fig. 5 [file 44319_2025_493_MOESM6_ESM.zip › Figure5/Fig5F/Experiment-122_synapse_included.tif_files/Experiment-122_z5c0x0-1388y0-1040.tif]

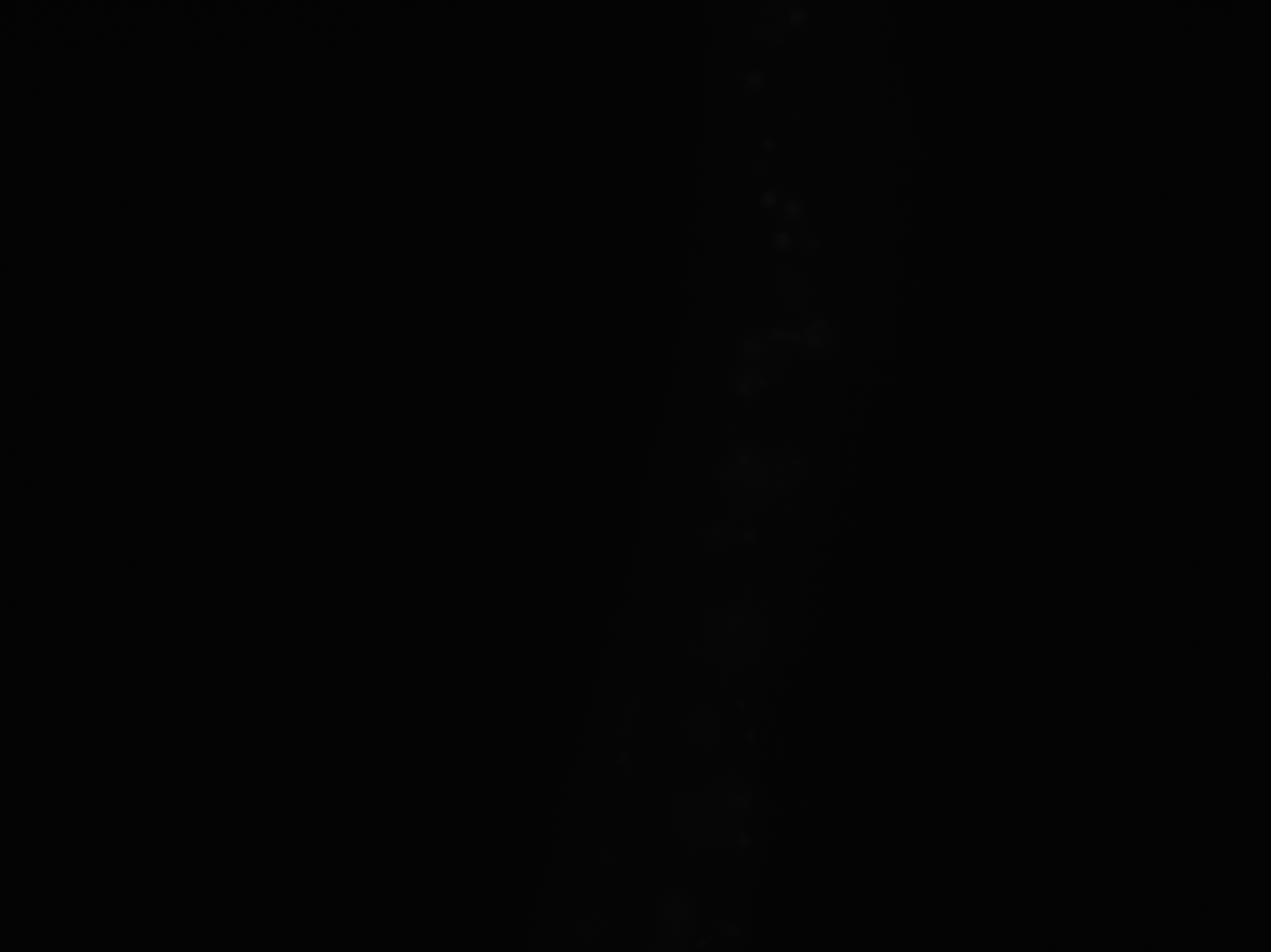

Supplement: Supplementary file 6 — Source data Fig. 5 [file 44319_2025_493_MOESM6_ESM.zip › Figure5/Fig5E/goodALM.tif_files/goodALM_z2c1x0-1388y0-1040.tif]

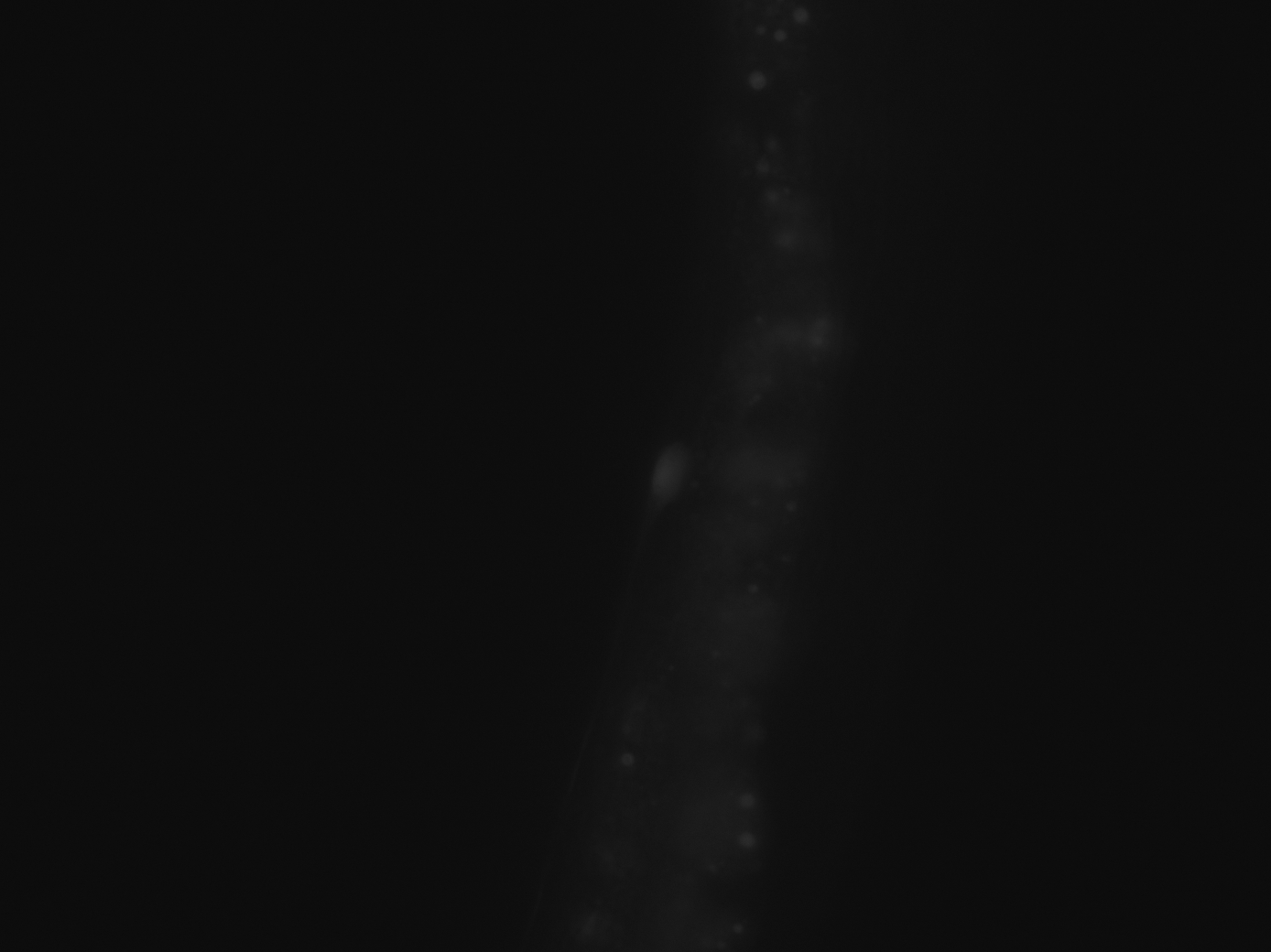

Supplement: Supplementary file 6 — Source data Fig. 5 [file 44319_2025_493_MOESM6_ESM.zip › Figure5/Fig5E/goodALM.tif_files/goodALM_z4c2x0-1388y0-1040.tif]

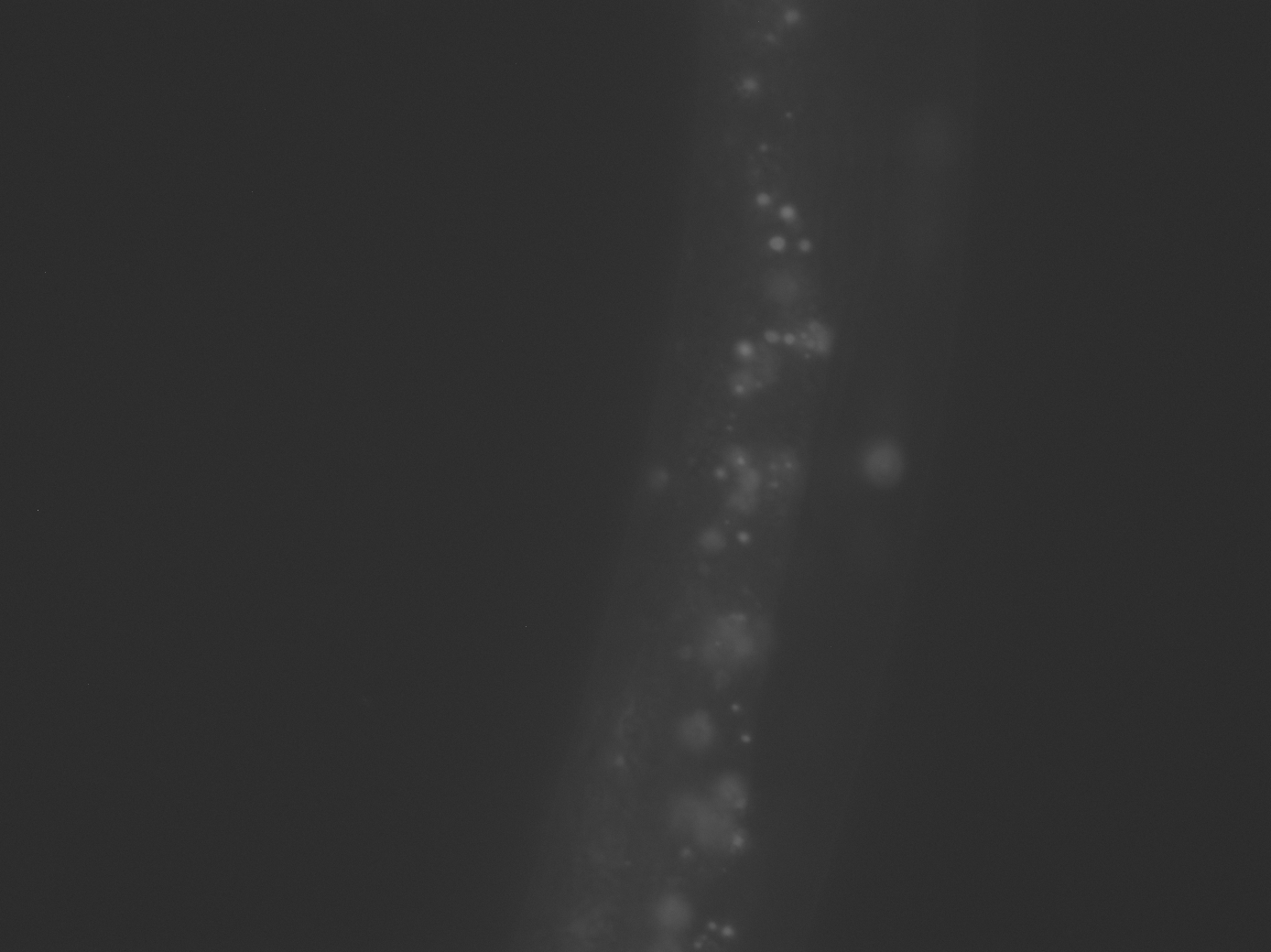

Supplement: Supplementary file 6 — Source data Fig. 5 [file 44319_2025_493_MOESM6_ESM.zip › Figure5/Fig5E/goodALM.tif_files/goodALM_z1c0x0-1388y0-1040.tif]

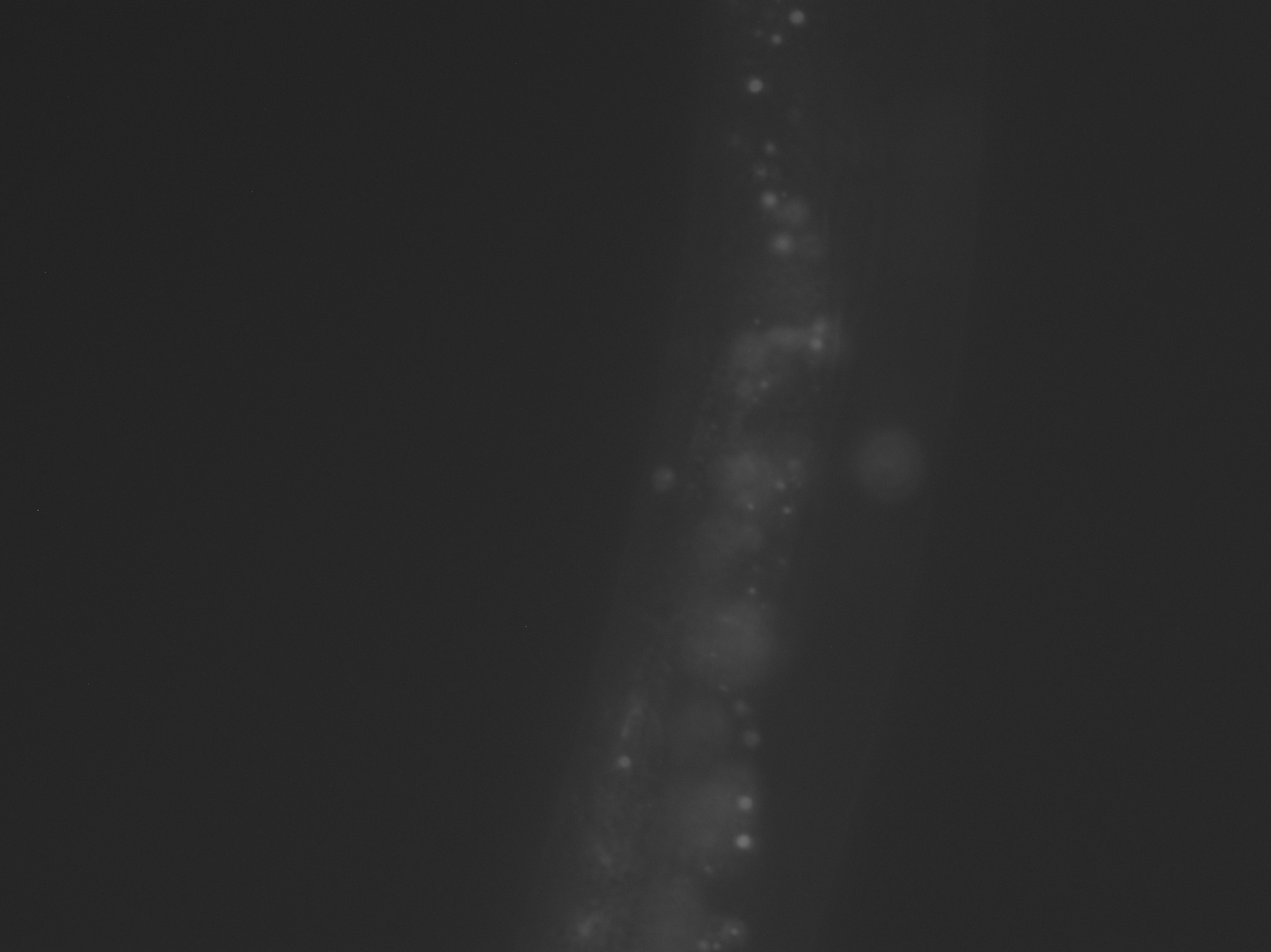

Supplement: Supplementary file 6 — Source data Fig. 5 [file 44319_2025_493_MOESM6_ESM.zip › Figure5/Fig5E/goodALM.tif_files/goodALM_z3c0x0-1388y0-1040.tif]

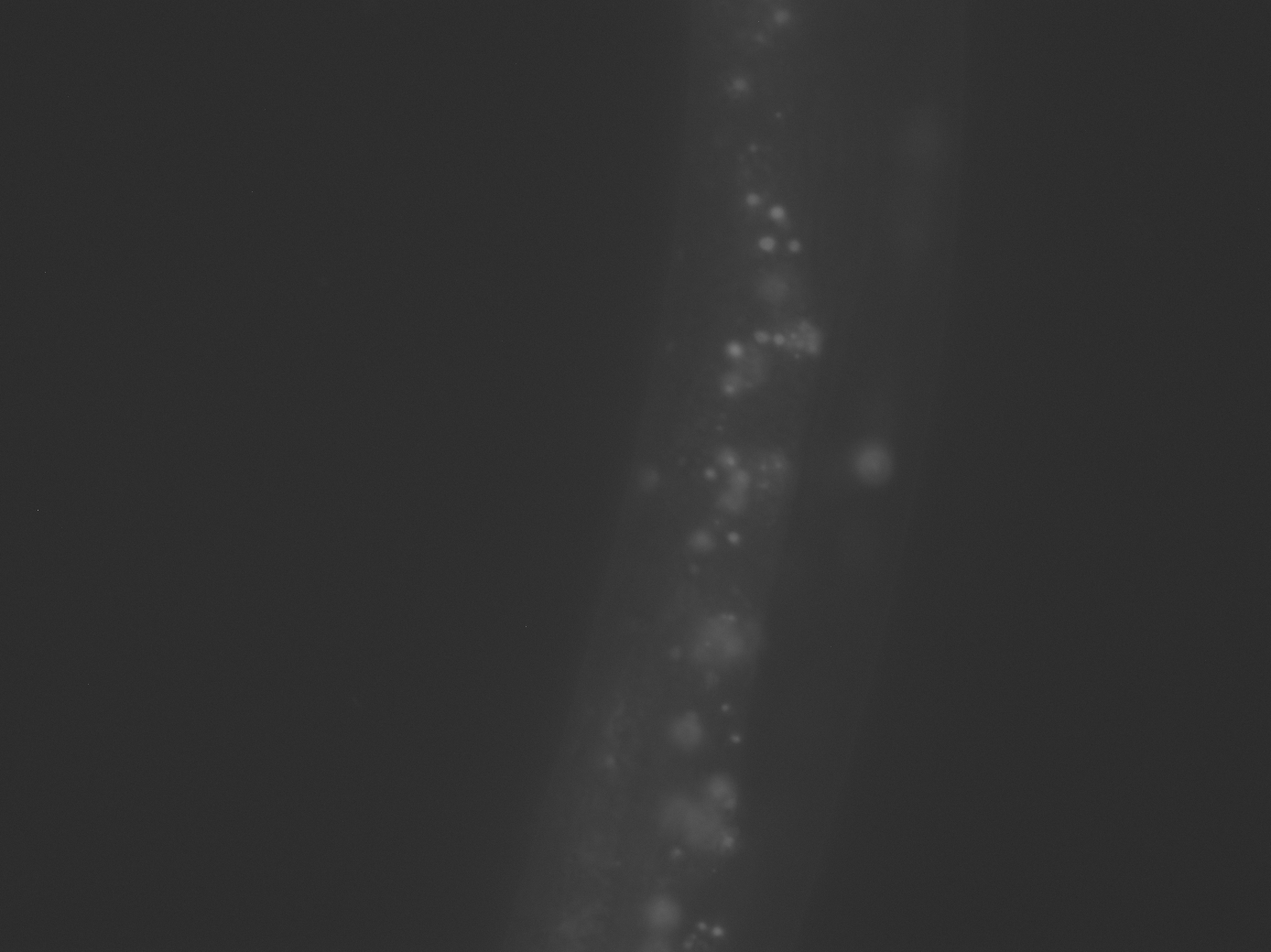

Supplement: Supplementary file 6 — Source data Fig. 5 [file 44319_2025_493_MOESM6_ESM.zip › Figure5/Fig5E/goodALM.tif_files/goodALM_z0c0x0-1388y0-1040.tif]

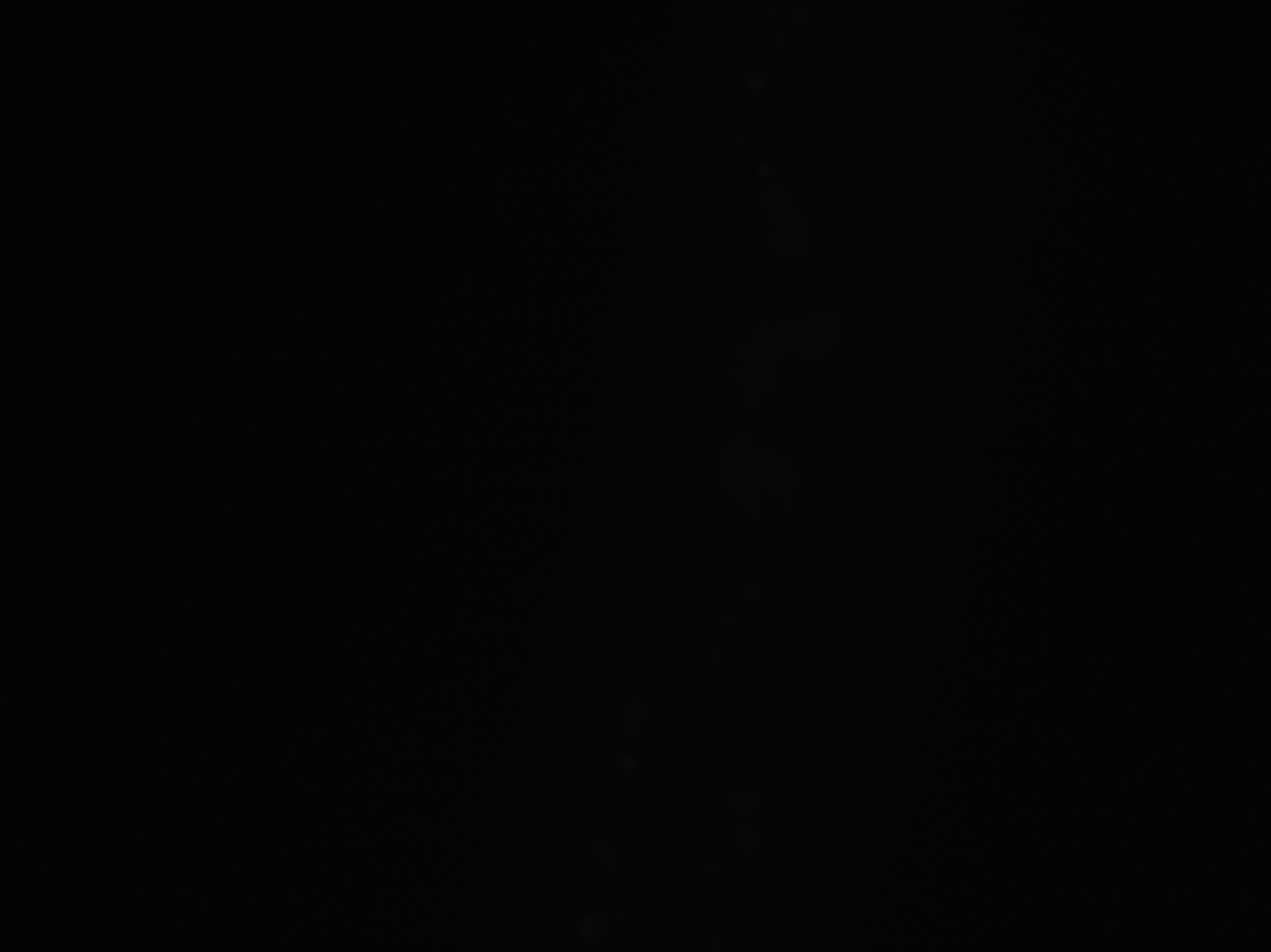

Supplement: Supplementary file 6 — Source data Fig. 5 [file 44319_2025_493_MOESM6_ESM.zip › Figure5/Fig5E/goodALM.tif_files/goodALM_z5c1x0-1388y0-1040.tif]

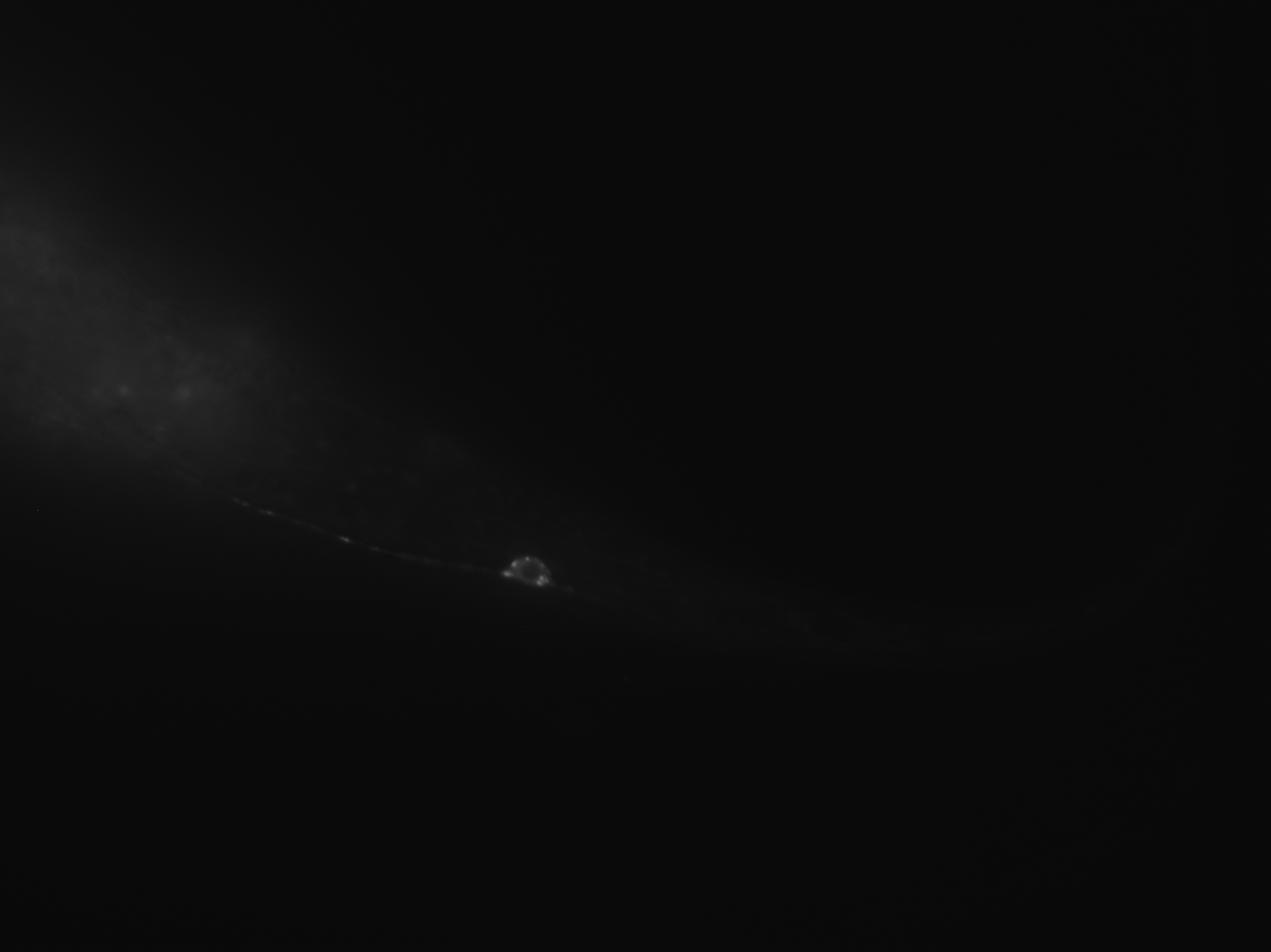

Supplement: Supplementary file 6 — Source data Fig. 5 [file 44319_2025_493_MOESM6_ESM.zip › Figure5/Fig5F/Experiment-35_Cellbody_wt.tif_files/Experiment-35_z3c0x0-1388y0-1040.tif]

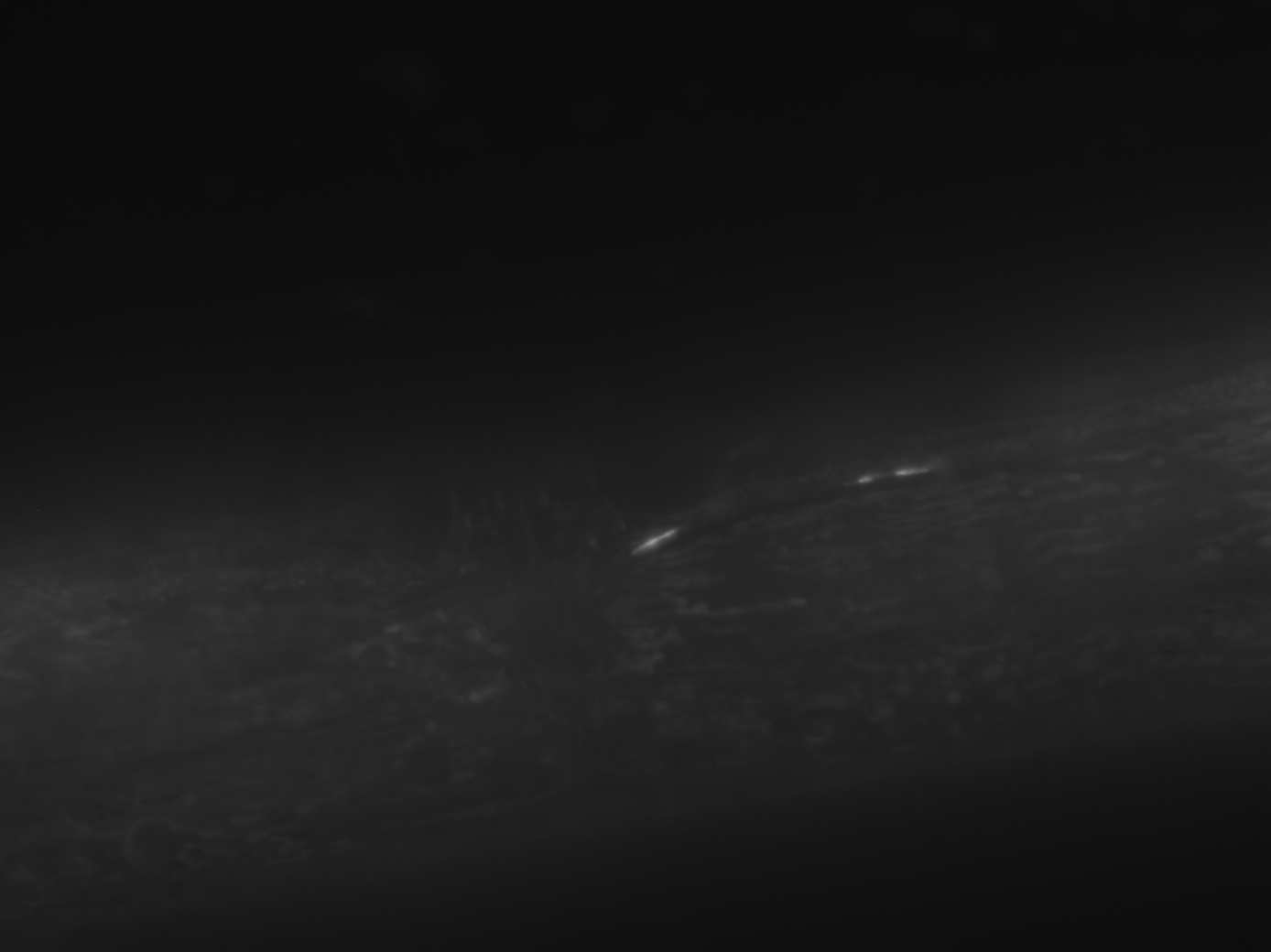

Supplement: Supplementary file 6 — Source data Fig. 5 [file 44319_2025_493_MOESM6_ESM.zip › Figure5/Fig5F/Experiment-122_synapse_included.tif_files/Experiment-122_z4c0x0-1388y0-1040.tif]

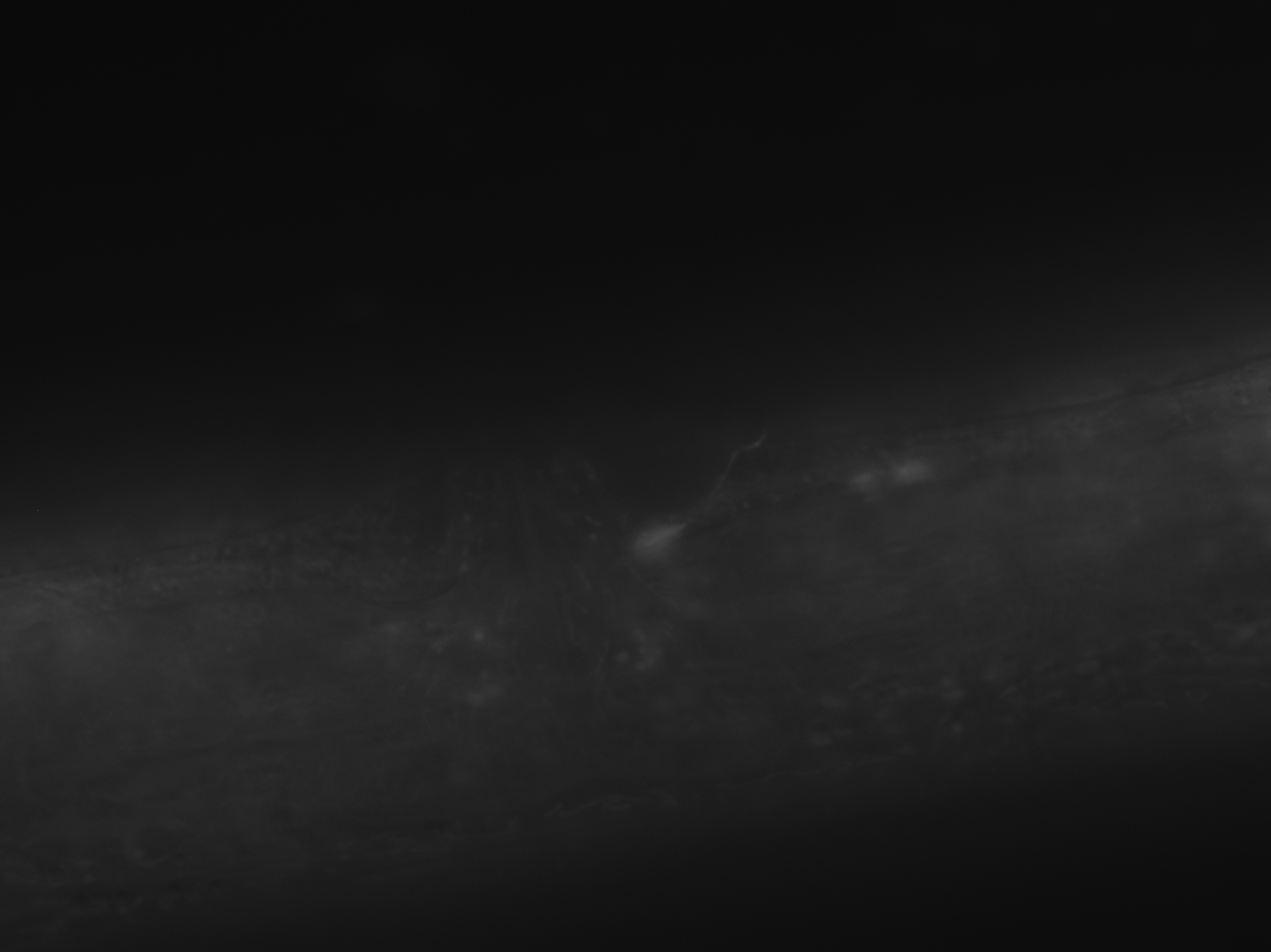

Supplement: Supplementary file 6 — Source data Fig. 5 [file 44319_2025_493_MOESM6_ESM.zip › Figure5/Fig5F/Experiment-122_synapse_included.tif_files/Experiment-122_z6c0x0-1388y0-1040.tif]

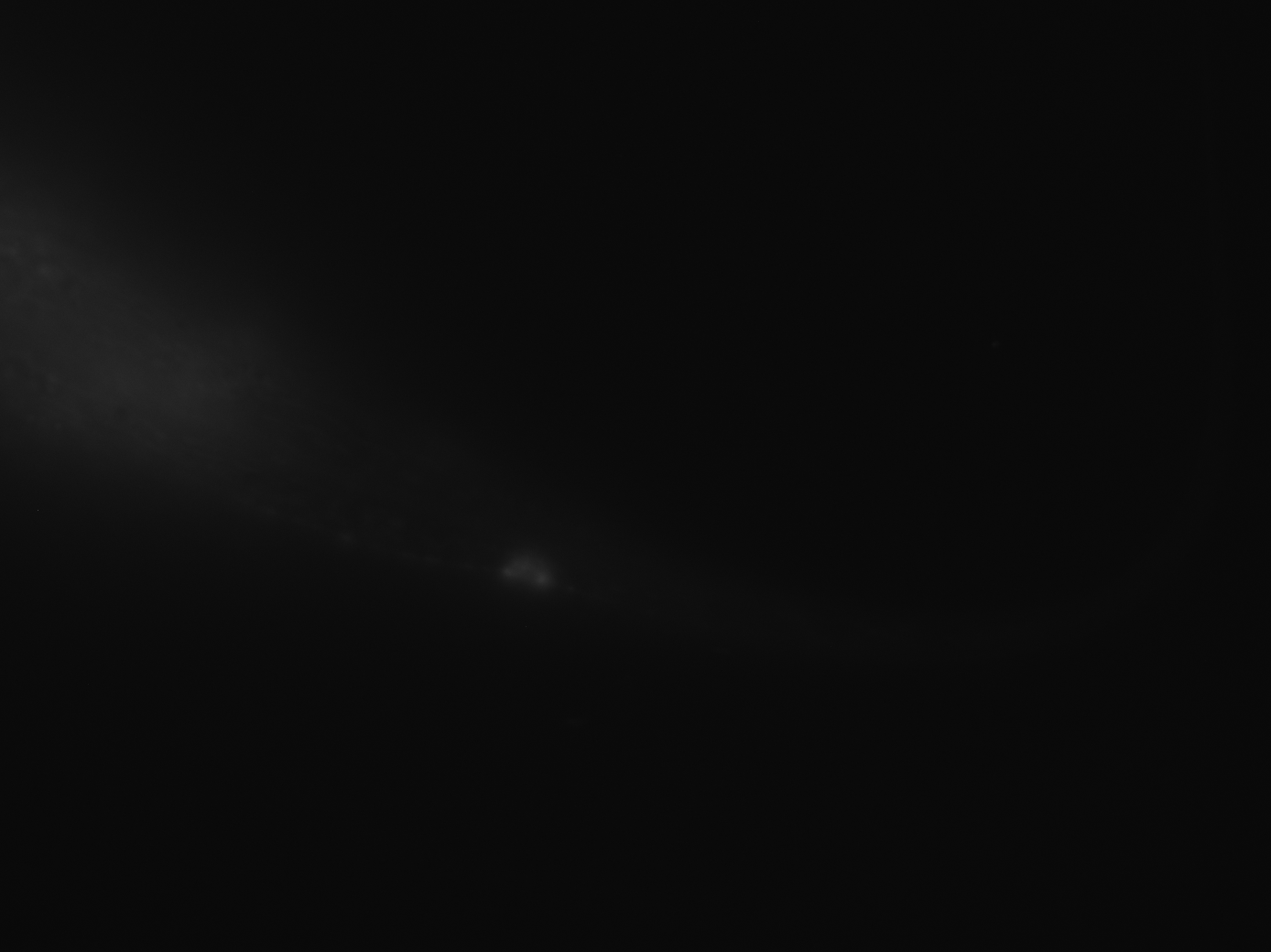

Supplement: Supplementary file 6 — Source data Fig. 5 [file 44319_2025_493_MOESM6_ESM.zip › Figure5/Fig5F/Experiment-35_Cellbody_wt.tif_files/Experiment-35_z1c0x0-1388y0-1040.tif]

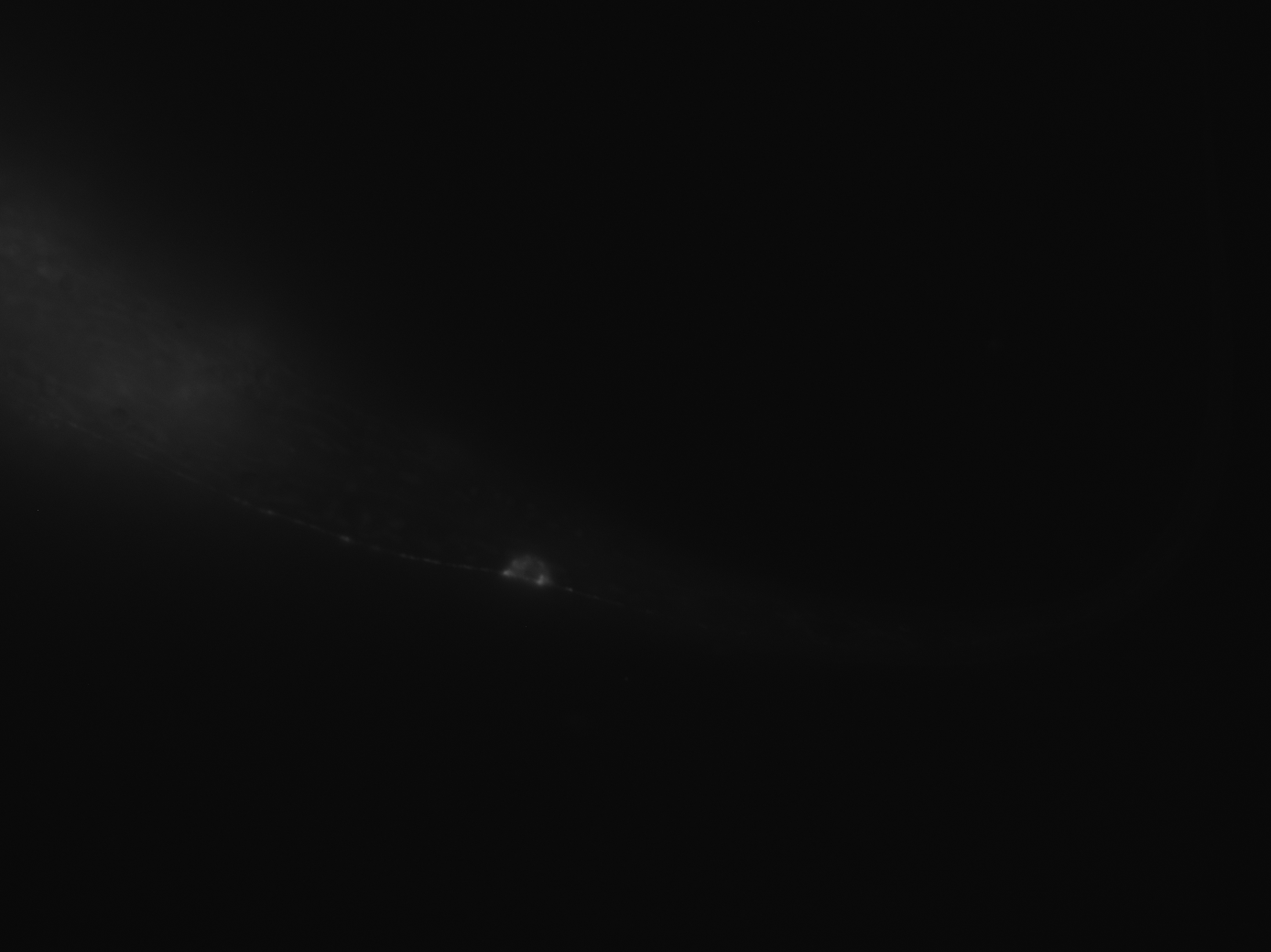

Supplement: Supplementary file 6 — Source data Fig. 5 [file 44319_2025_493_MOESM6_ESM.zip › Figure5/Fig5F/Experiment-35_Cellbody_wt.tif_files/Experiment-35_z2c0x0-1388y0-1040.tif]

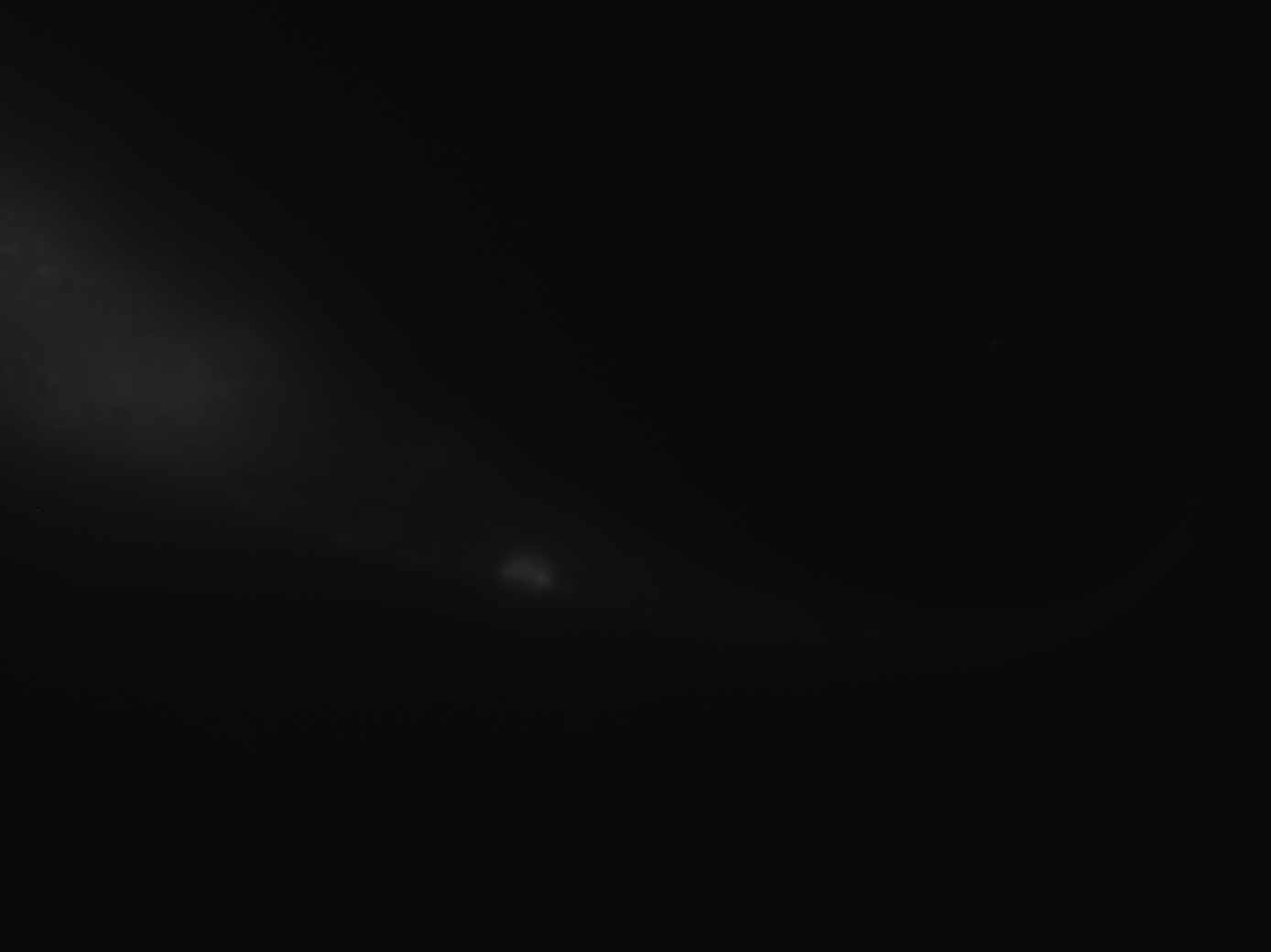

Supplement: Supplementary file 6 — Source data Fig. 5 [file 44319_2025_493_MOESM6_ESM.zip › Figure5/Fig5F/Experiment-35_Cellbody_wt.tif_files/Experiment-35_z0c0x0-1388y0-1040.tif]

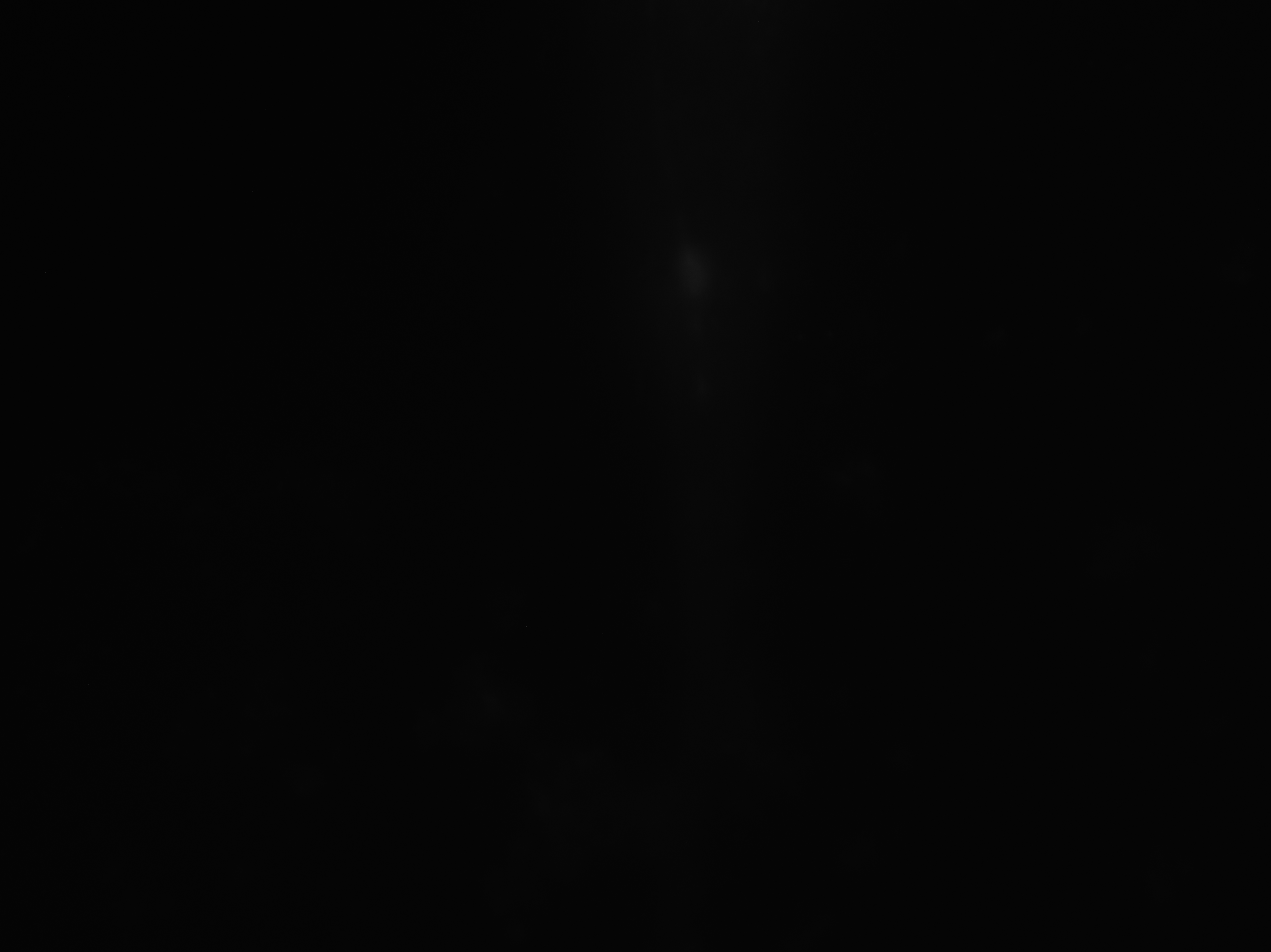

Supplement: Supplementary file 6 — Source data Fig. 5 [file 44319_2025_493_MOESM6_ESM.zip › Figure5/Fig5F/Experiment-962_cellbody_n2813.tif_files/Experiment-962_z0c0x0-1388y0-1040.tif]

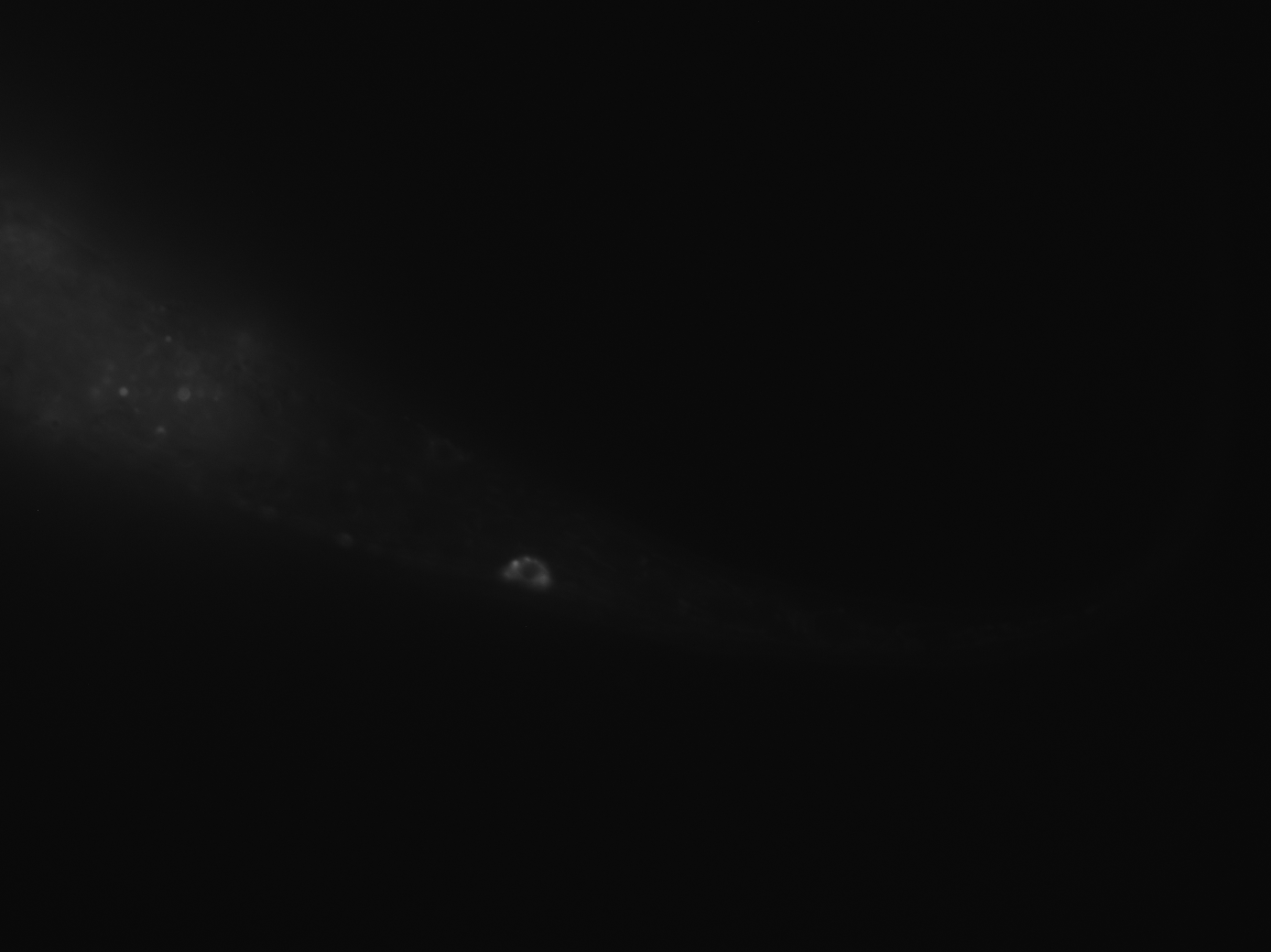

Supplement: Supplementary file 6 — Source data Fig. 5 [file 44319_2025_493_MOESM6_ESM.zip › Figure5/Fig5F/Experiment-35_Cellbody_wt.tif_files/Experiment-35_z4c0x0-1388y0-1040.tif]

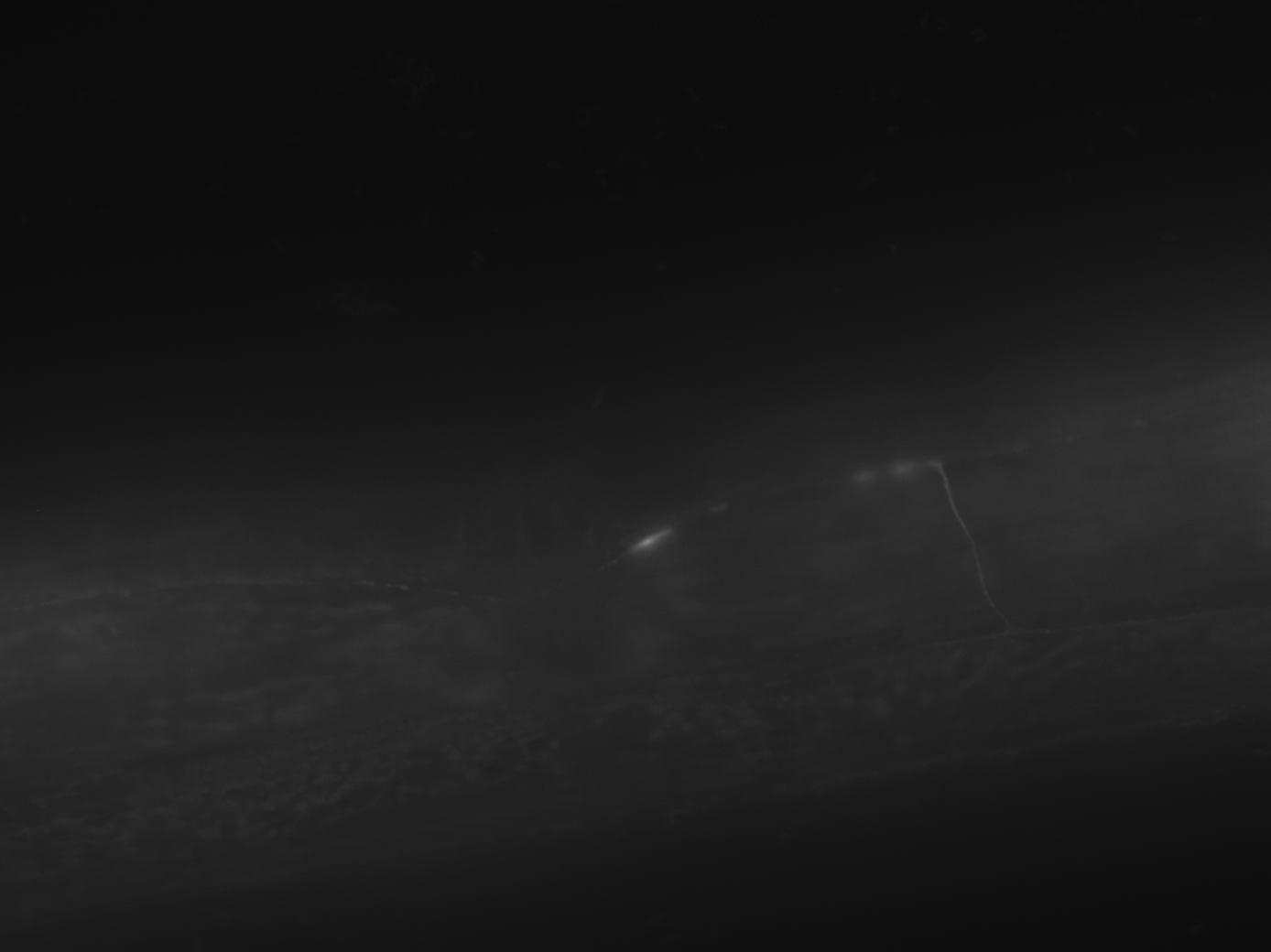

Supplement: Supplementary file 6 — Source data Fig. 5 [file 44319_2025_493_MOESM6_ESM.zip › Figure5/Fig5F/Experiment-122_synapse_included.tif_files/Experiment-122_z0c0x0-1388y0-1040.tif]

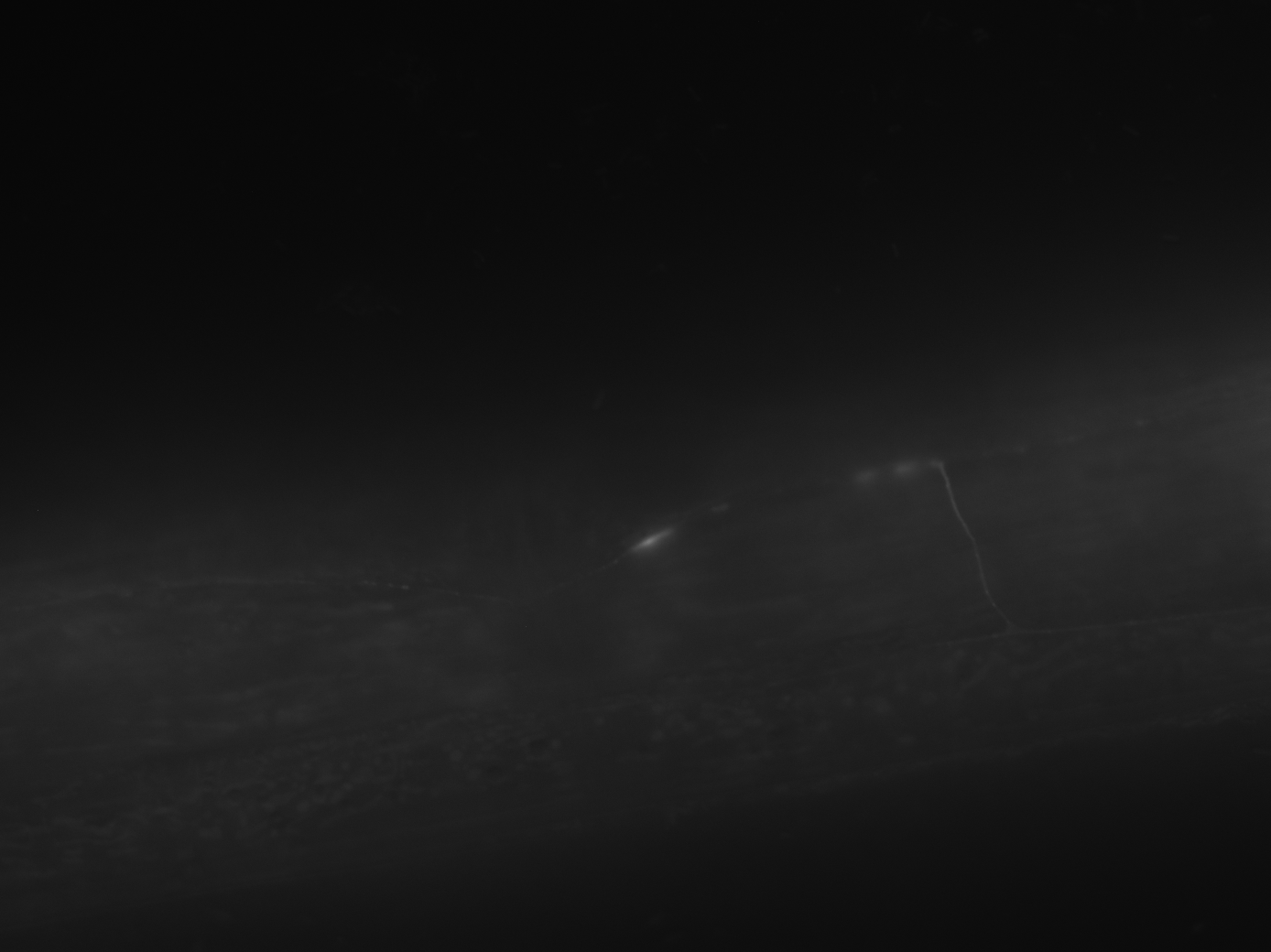

Supplement: Supplementary file 6 — Source data Fig. 5 [file 44319_2025_493_MOESM6_ESM.zip › Figure5/Fig5F/Experiment-122_synapse_included.tif_files/Experiment-122_z1c0x0-1388y0-1040.tif]

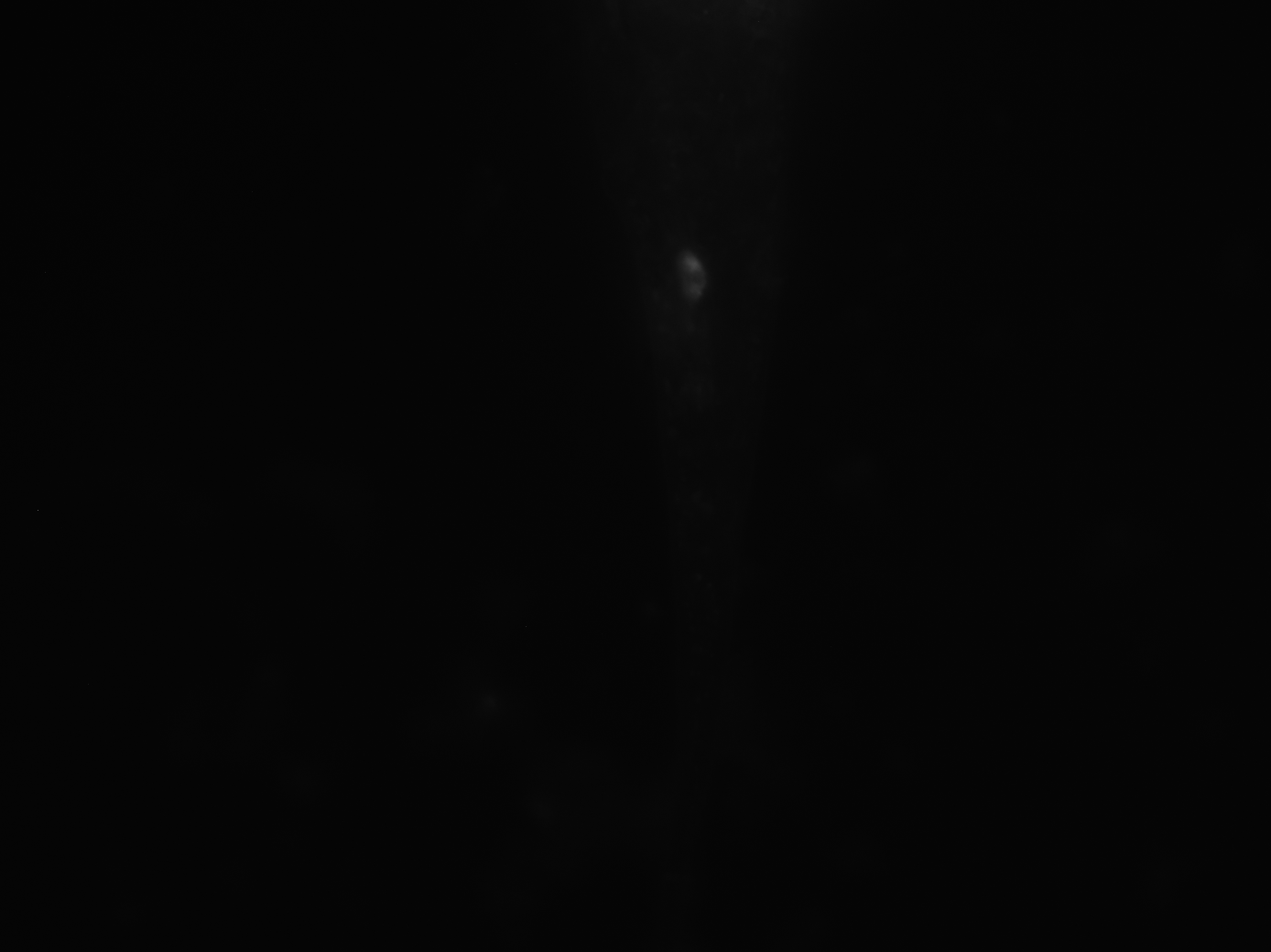

Supplement: Supplementary file 6 — Source data Fig. 5 [file 44319_2025_493_MOESM6_ESM.zip › Figure5/Fig5F/Experiment-962_cellbody_n2813.tif_files/Experiment-962_z6c0x0-1388y0-1040.tif]

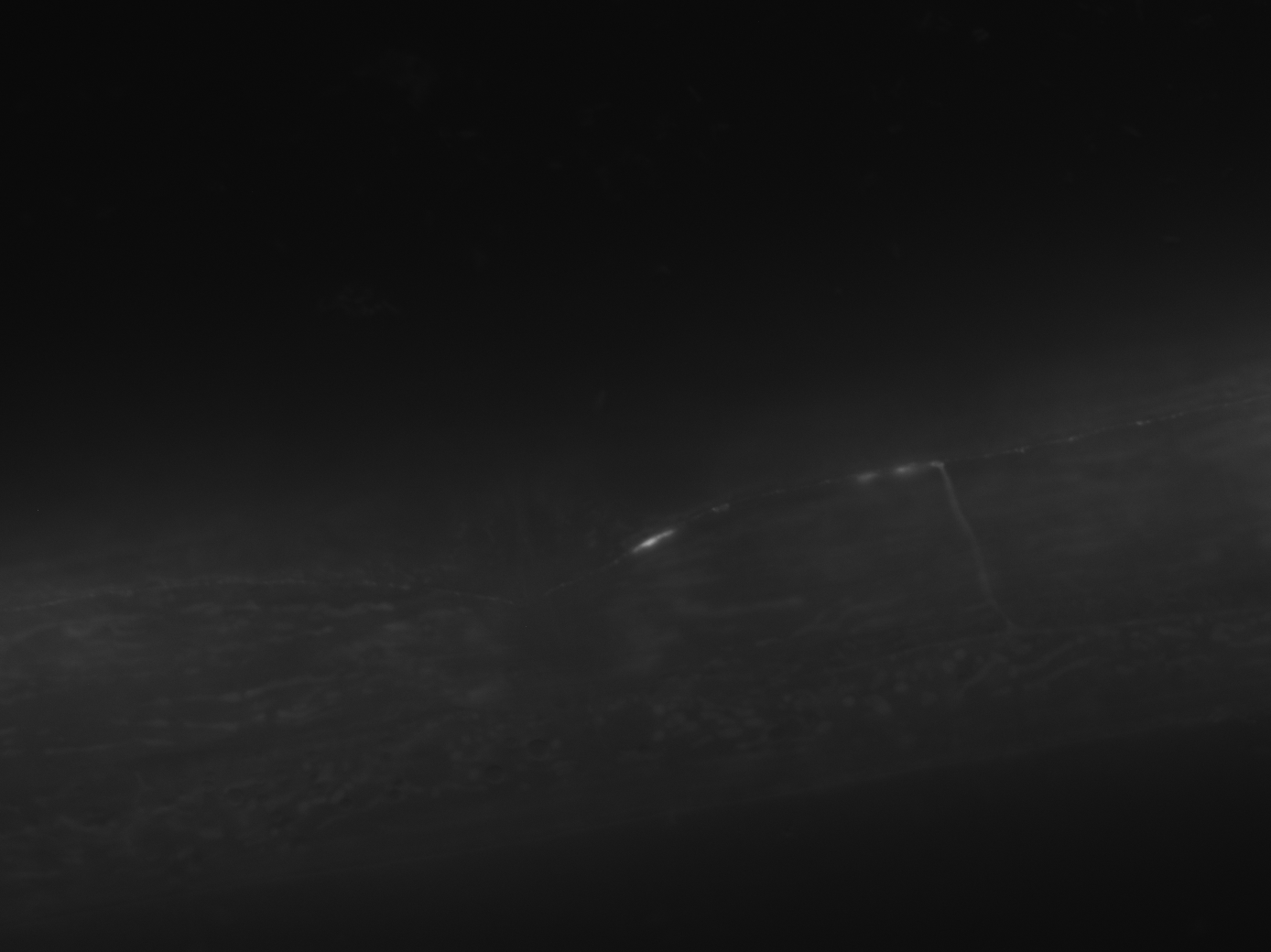

Supplement: Supplementary file 6 — Source data Fig. 5 [file 44319_2025_493_MOESM6_ESM.zip › Figure5/Fig5F/Experiment-122_synapse_included.tif_files/Experiment-122_z2c0x0-1388y0-1040.tif]

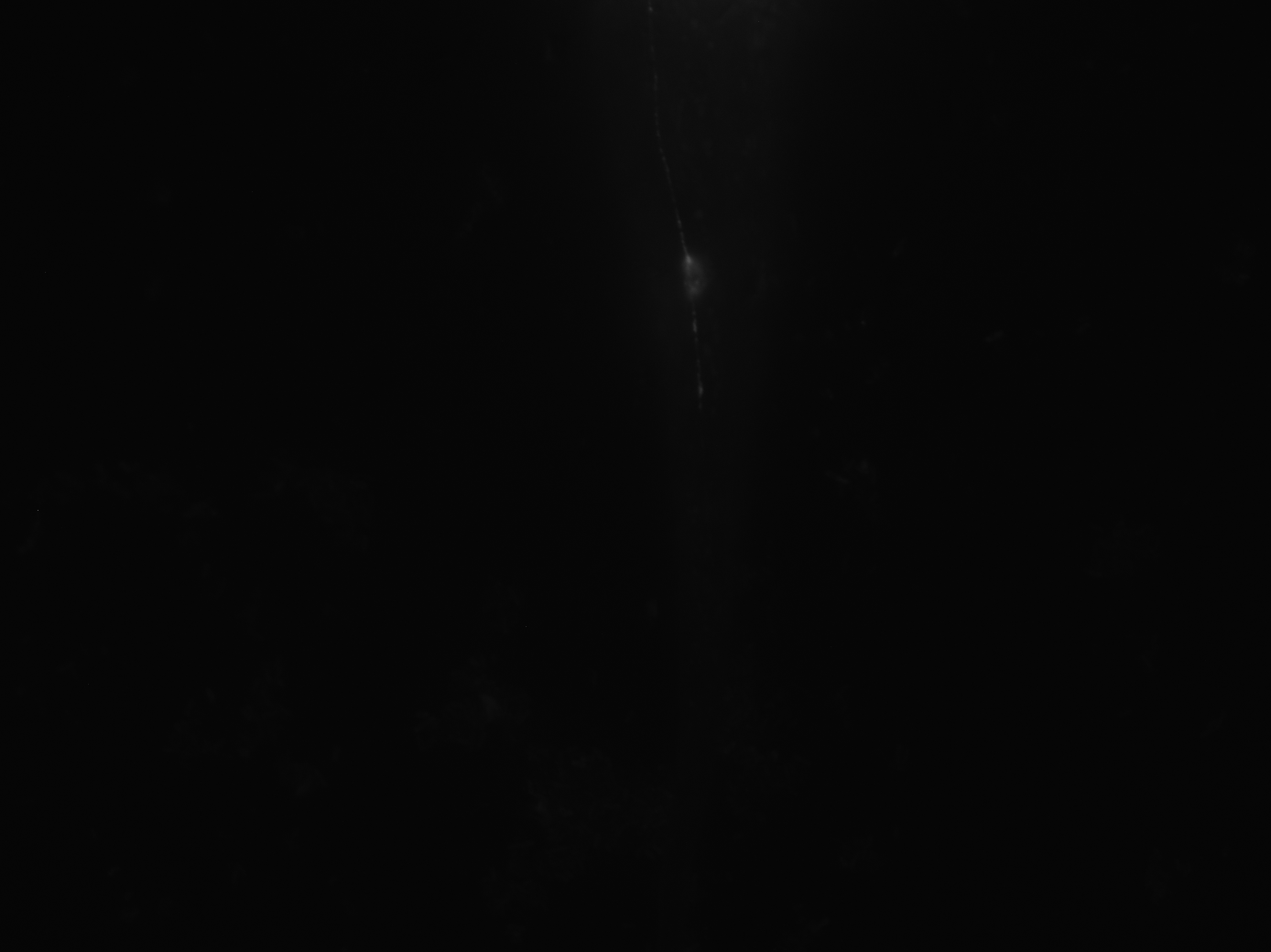

Supplement: Supplementary file 6 — Source data Fig. 5 [file 44319_2025_493_MOESM6_ESM.zip › Figure5/Fig5F/Experiment-962_cellbody_n2813.tif_files/Experiment-962_z3c0x0-1388y0-1040.tif]

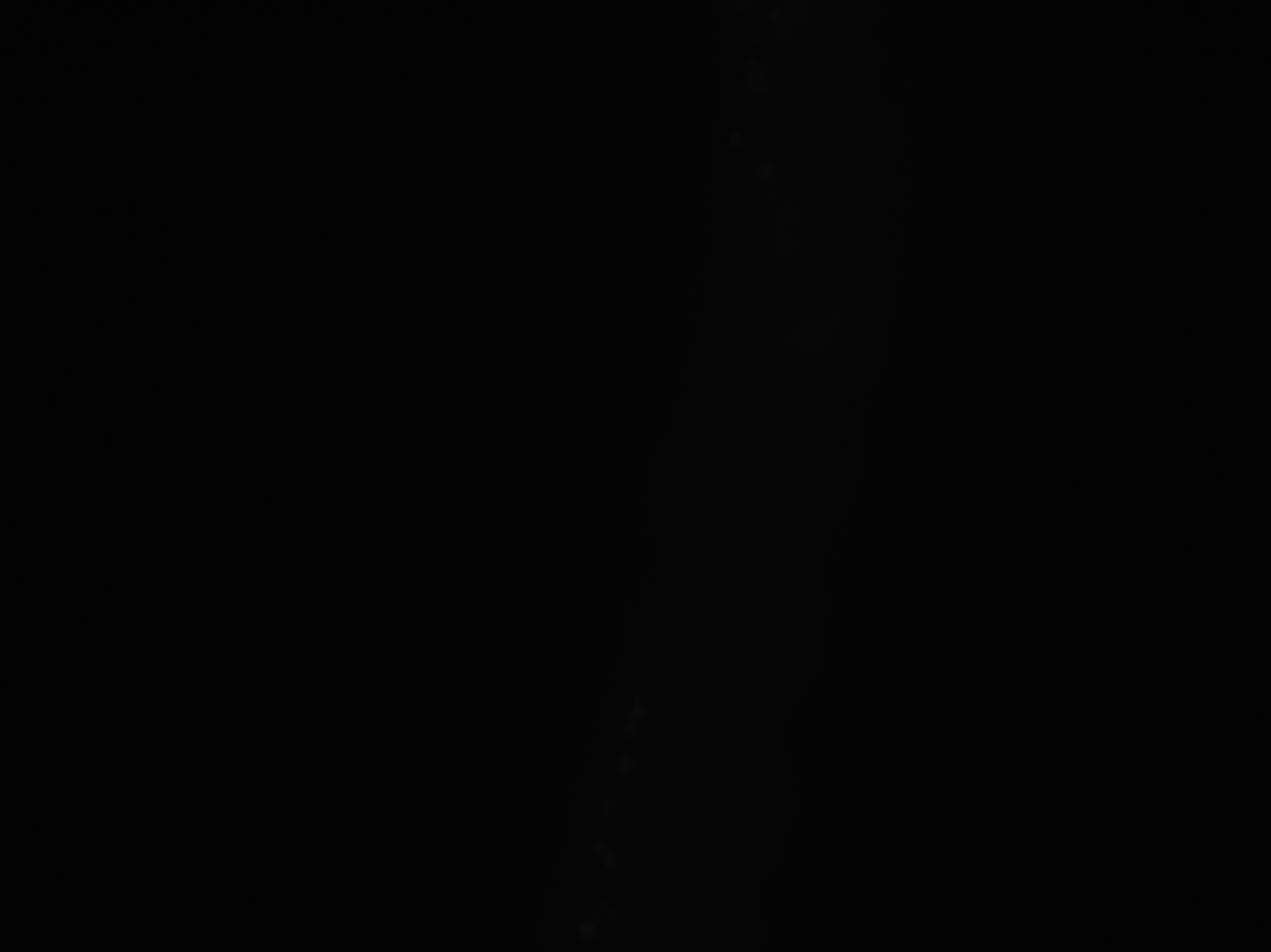

Supplement: Supplementary file 6 — Source data Fig. 5 [file 44319_2025_493_MOESM6_ESM.zip › Figure5/Fig5E/goodALM.tif_files/goodALM_z7c1x0-1388y0-1040.tif]

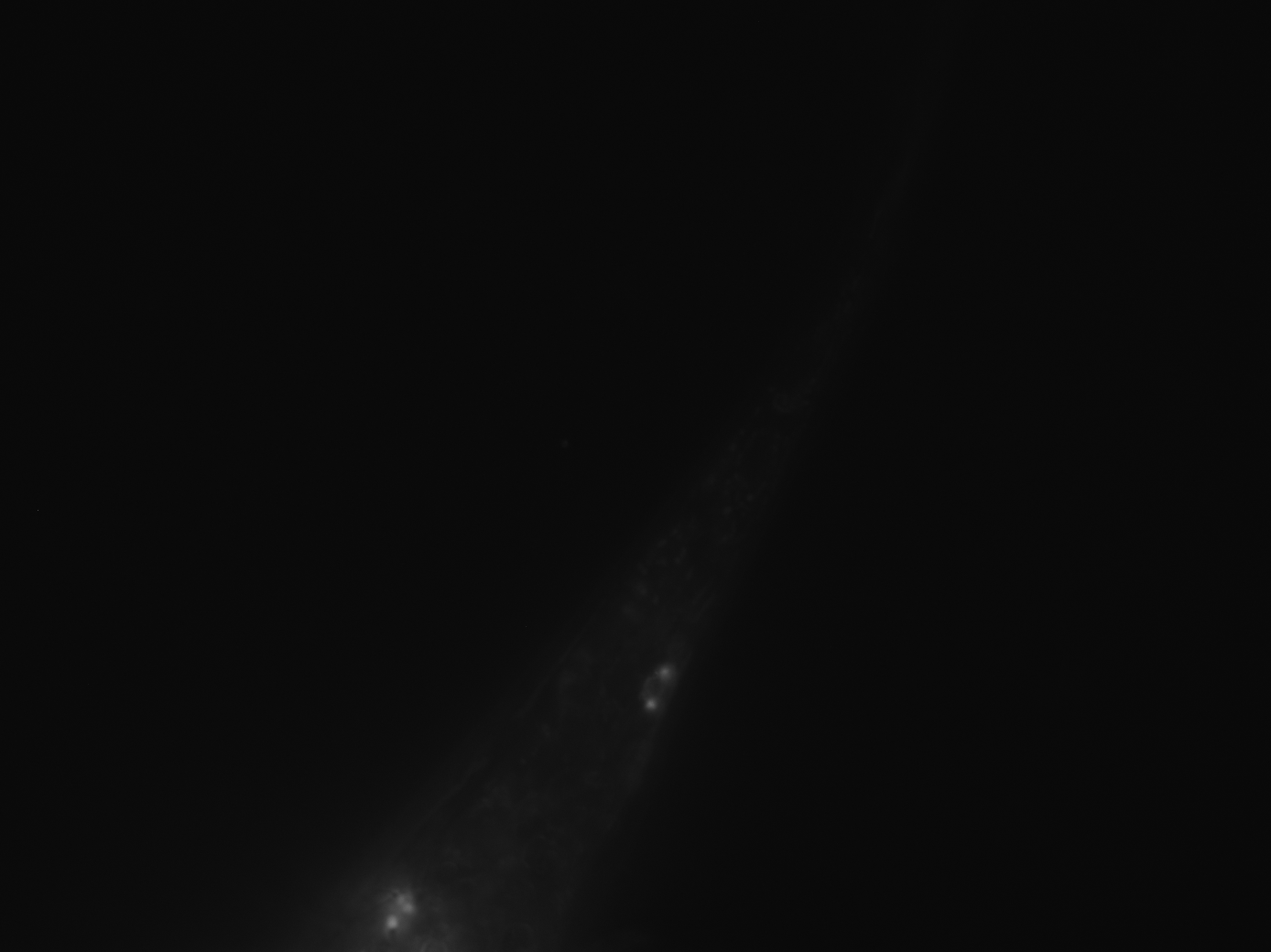

Supplement: Supplementary file 6 — Source data Fig. 5 [file 44319_2025_493_MOESM6_ESM.zip › Figure5/Fig5F/Experiment-110_cellbody_included.tif_files/Experiment-110_z5c0x0-1388y0-1040.tif]

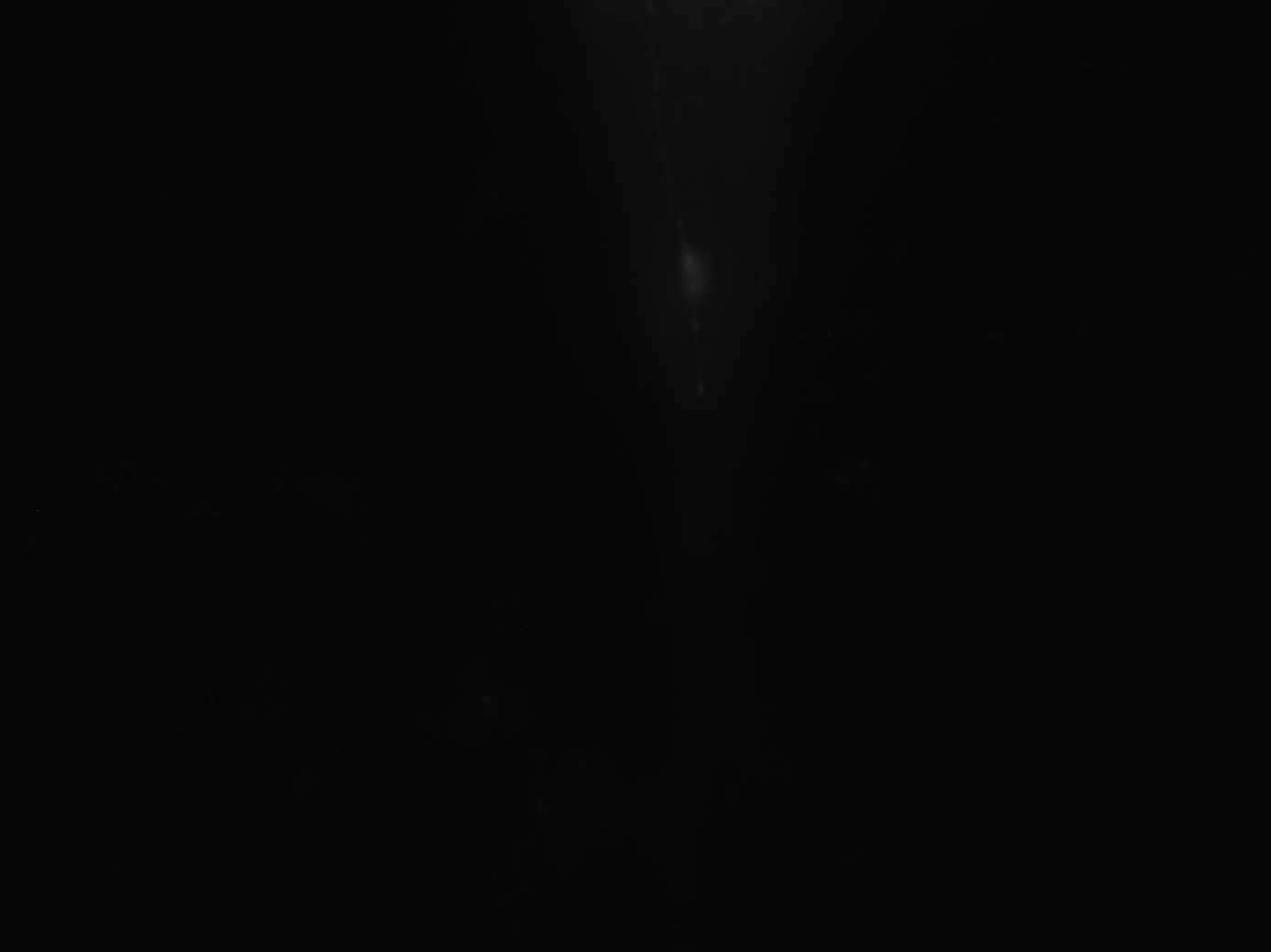

Supplement: Supplementary file 6 — Source data Fig. 5 [file 44319_2025_493_MOESM6_ESM.zip › Figure5/Fig5F/Experiment-962_cellbody_n2813.tif_files/Experiment-962_z2c0x0-1388y0-1040.tif]

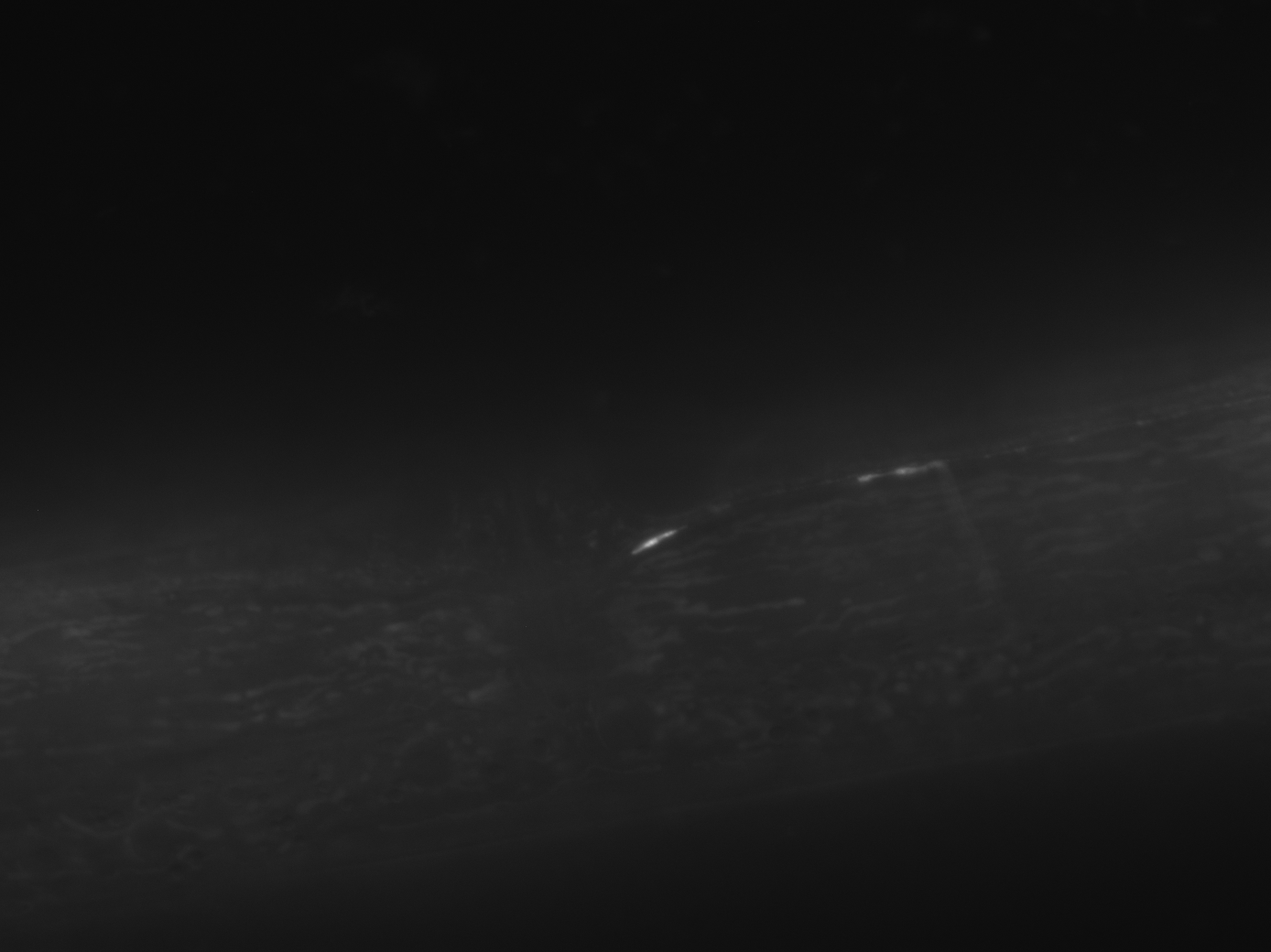

Supplement: Supplementary file 6 — Source data Fig. 5 [file 44319_2025_493_MOESM6_ESM.zip › Figure5/Fig5F/Experiment-122_synapse_included.tif_files/Experiment-122_z3c0x0-1388y0-1040.tif]

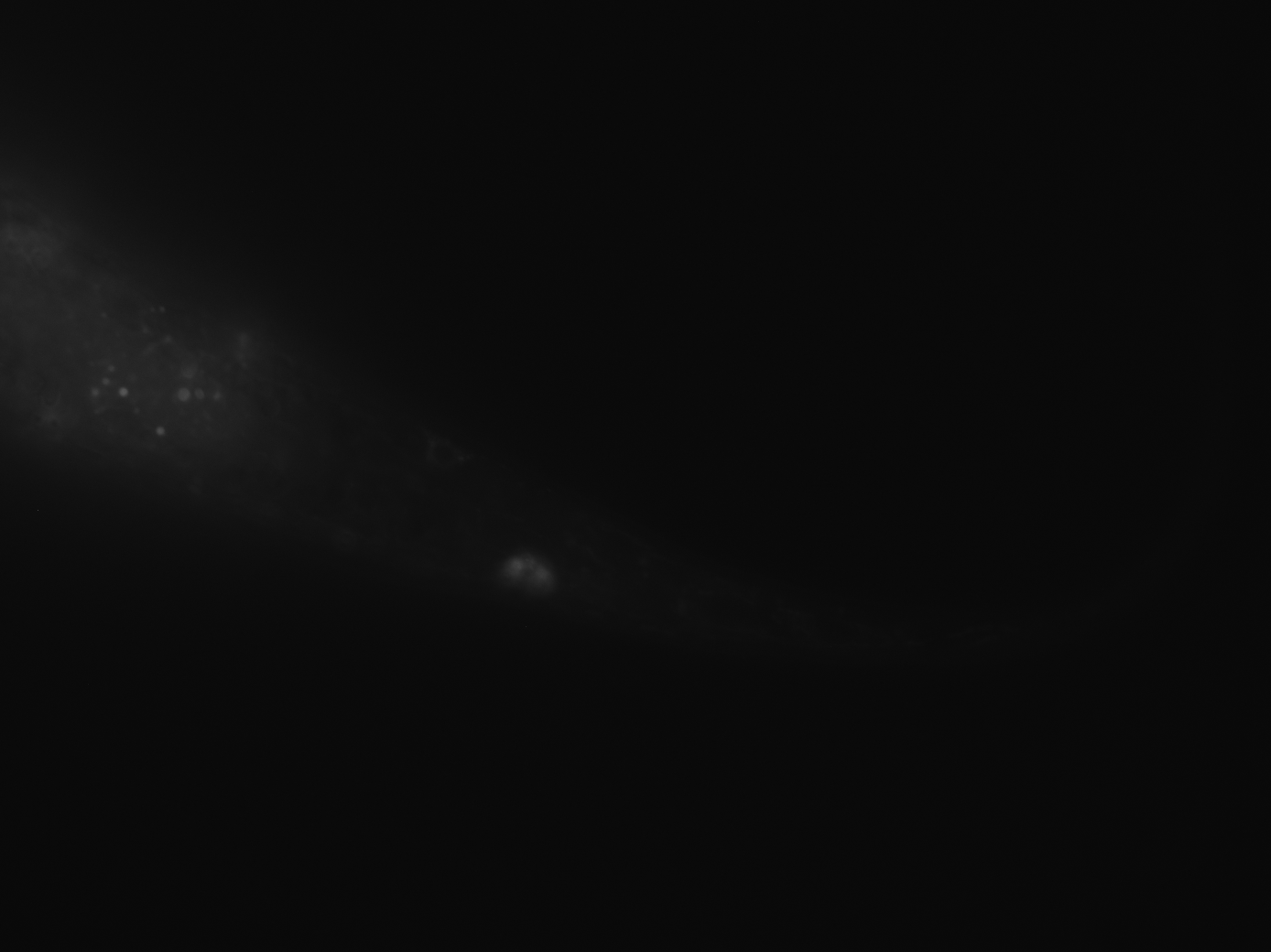

Supplement: Supplementary file 6 — Source data Fig. 5 [file 44319_2025_493_MOESM6_ESM.zip › Figure5/Fig5F/Experiment-35_Cellbody_wt.tif_files/Experiment-35_z5c0x0-1388y0-1040.tif]

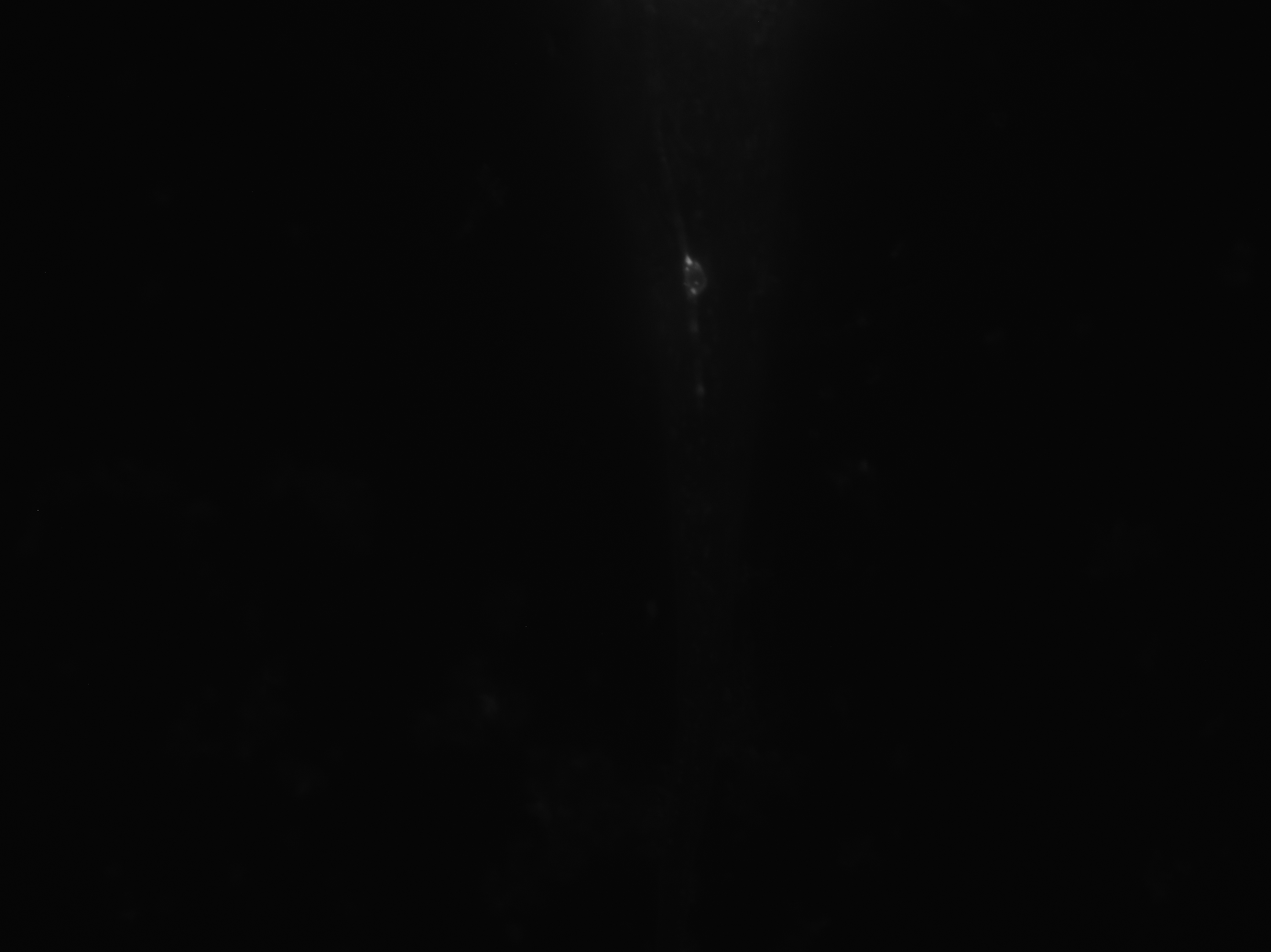

Supplement: Supplementary file 6 — Source data Fig. 5 [file 44319_2025_493_MOESM6_ESM.zip › Figure5/Fig5F/Experiment-962_cellbody_n2813.tif_files/Experiment-962_z4c0x0-1388y0-1040.tif]

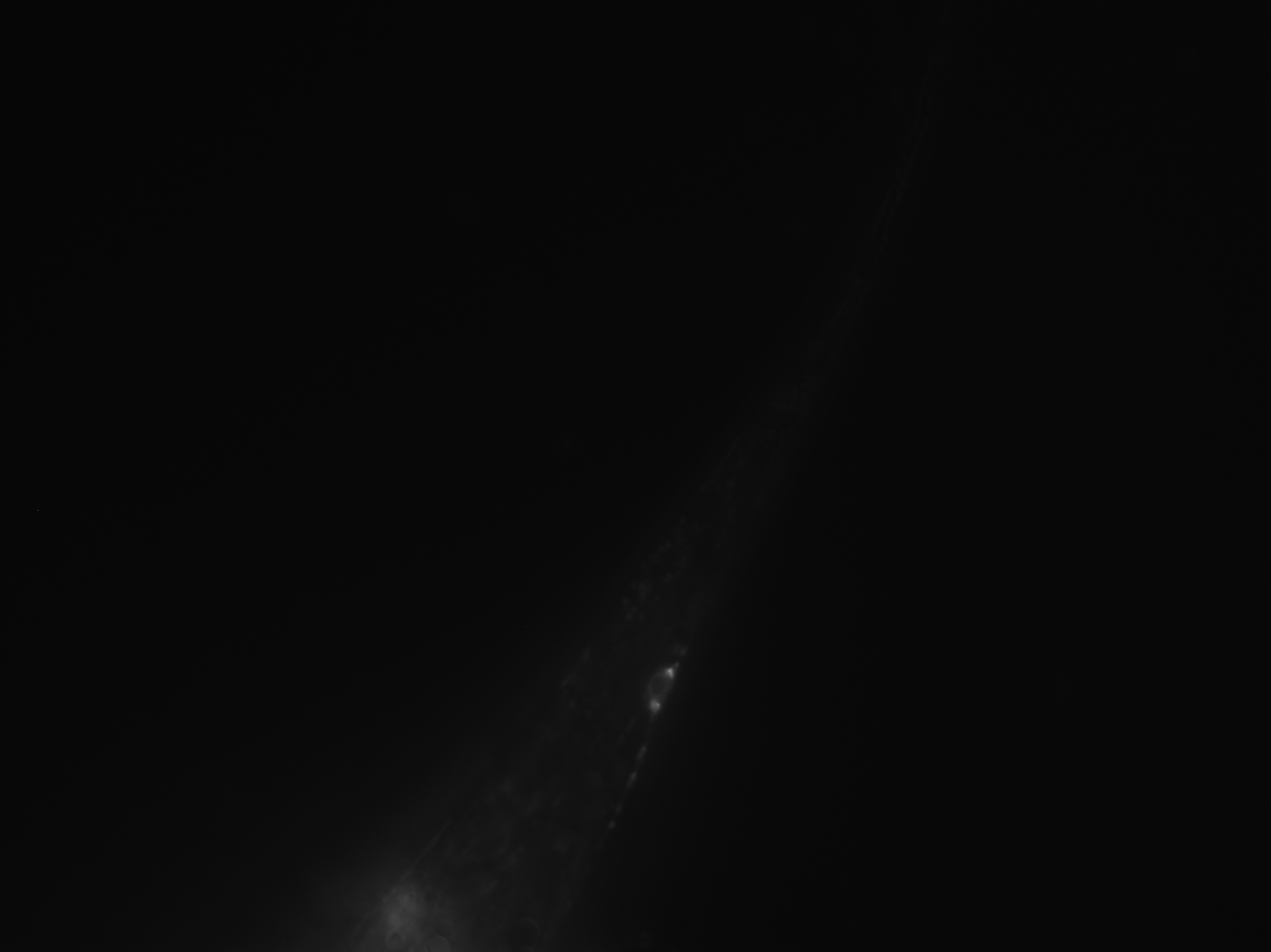

Supplement: Supplementary file 6 — Source data Fig. 5 [file 44319_2025_493_MOESM6_ESM.zip › Figure5/Fig5F/Experiment-110_cellbody_included.tif_files/Experiment-110_z3c0x0-1388y0-1040.tif]

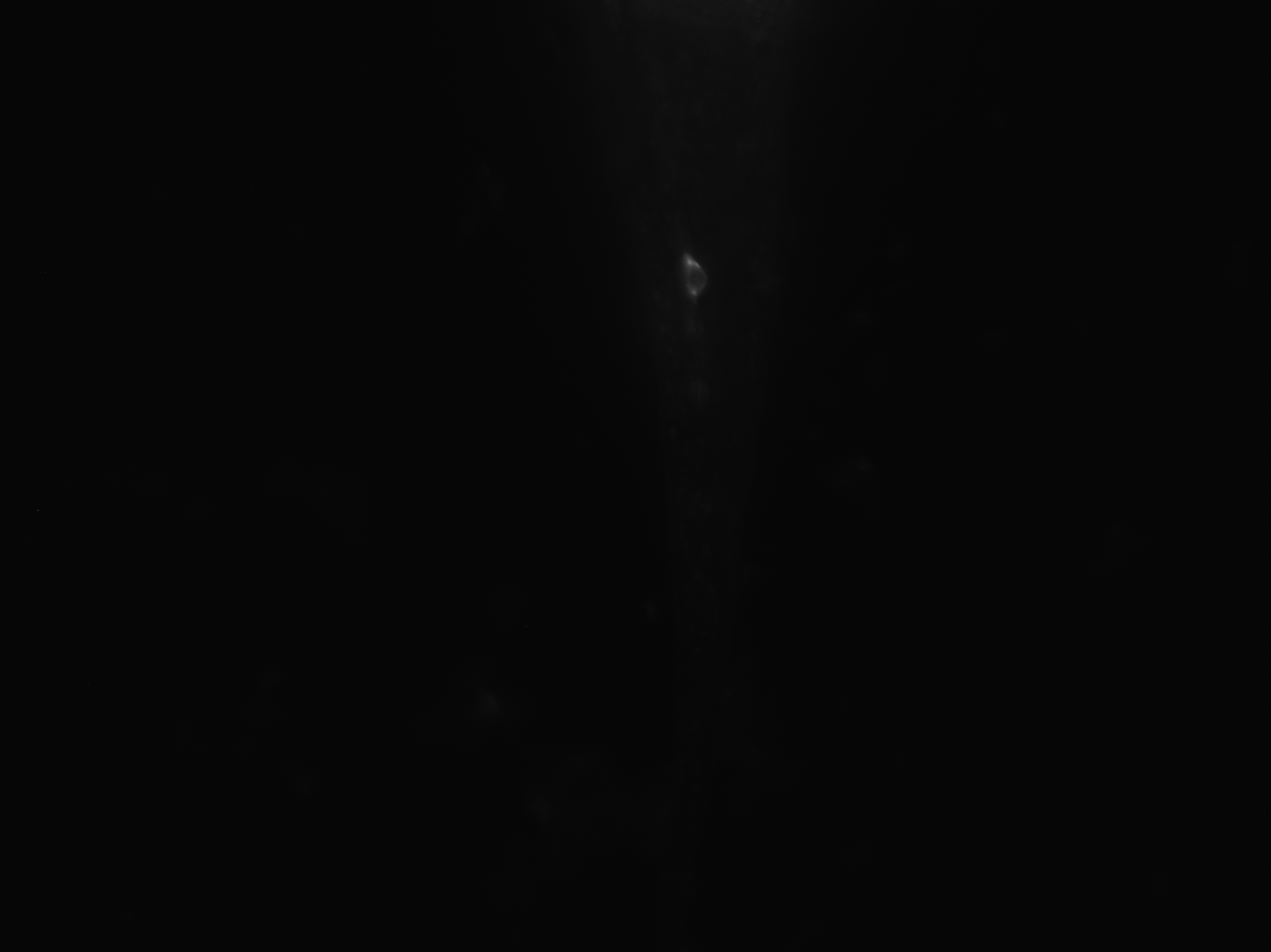

Supplement: Supplementary file 6 — Source data Fig. 5 [file 44319_2025_493_MOESM6_ESM.zip › Figure5/Fig5F/Experiment-962_cellbody_n2813.tif_files/Experiment-962_z5c0x0-1388y0-1040.tif]

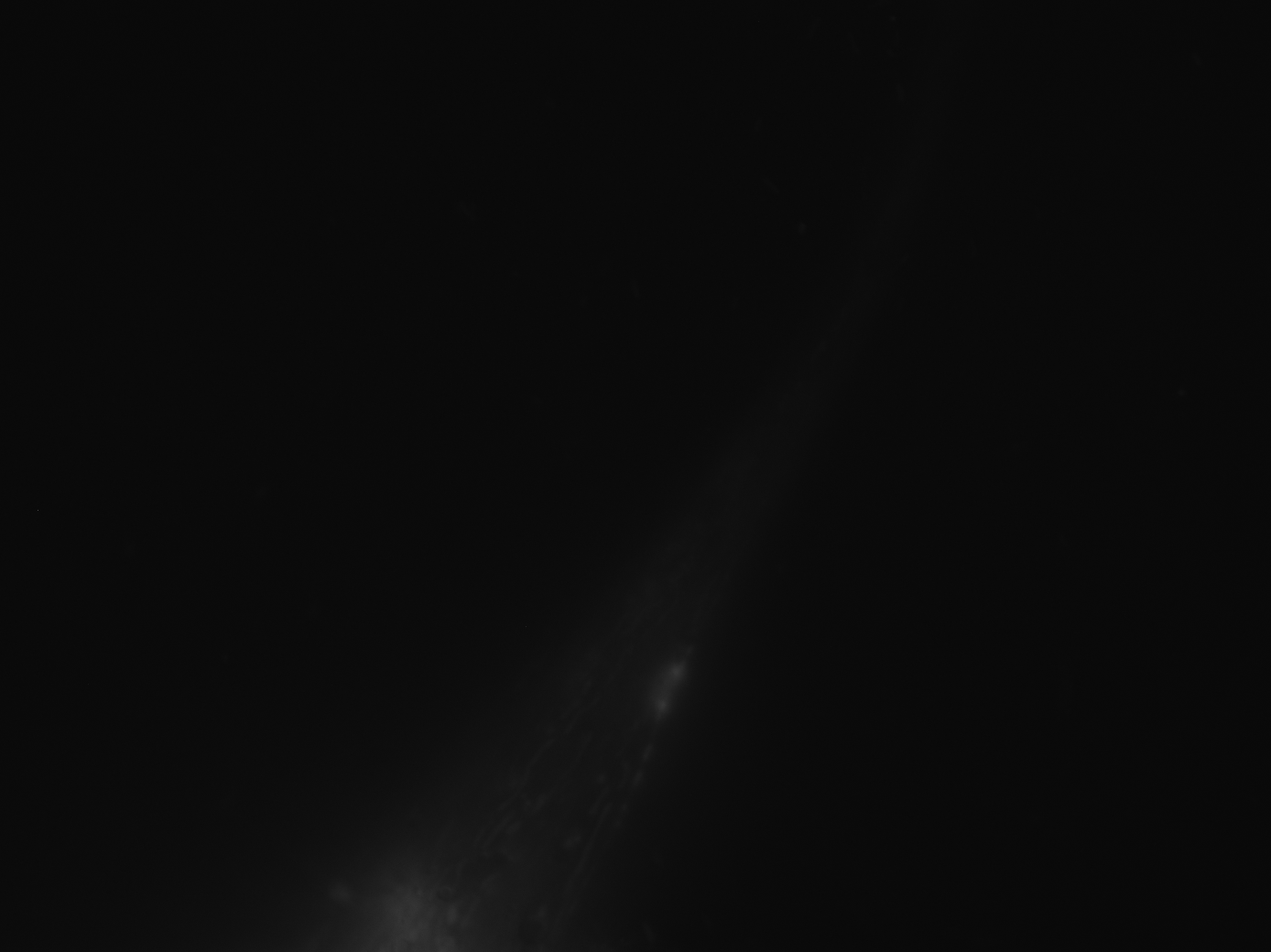

Supplement: Supplementary file 6 — Source data Fig. 5 [file 44319_2025_493_MOESM6_ESM.zip › Figure5/Fig5F/Experiment-110_cellbody_included.tif_files/Experiment-110_z0c0x0-1388y0-1040.tif]

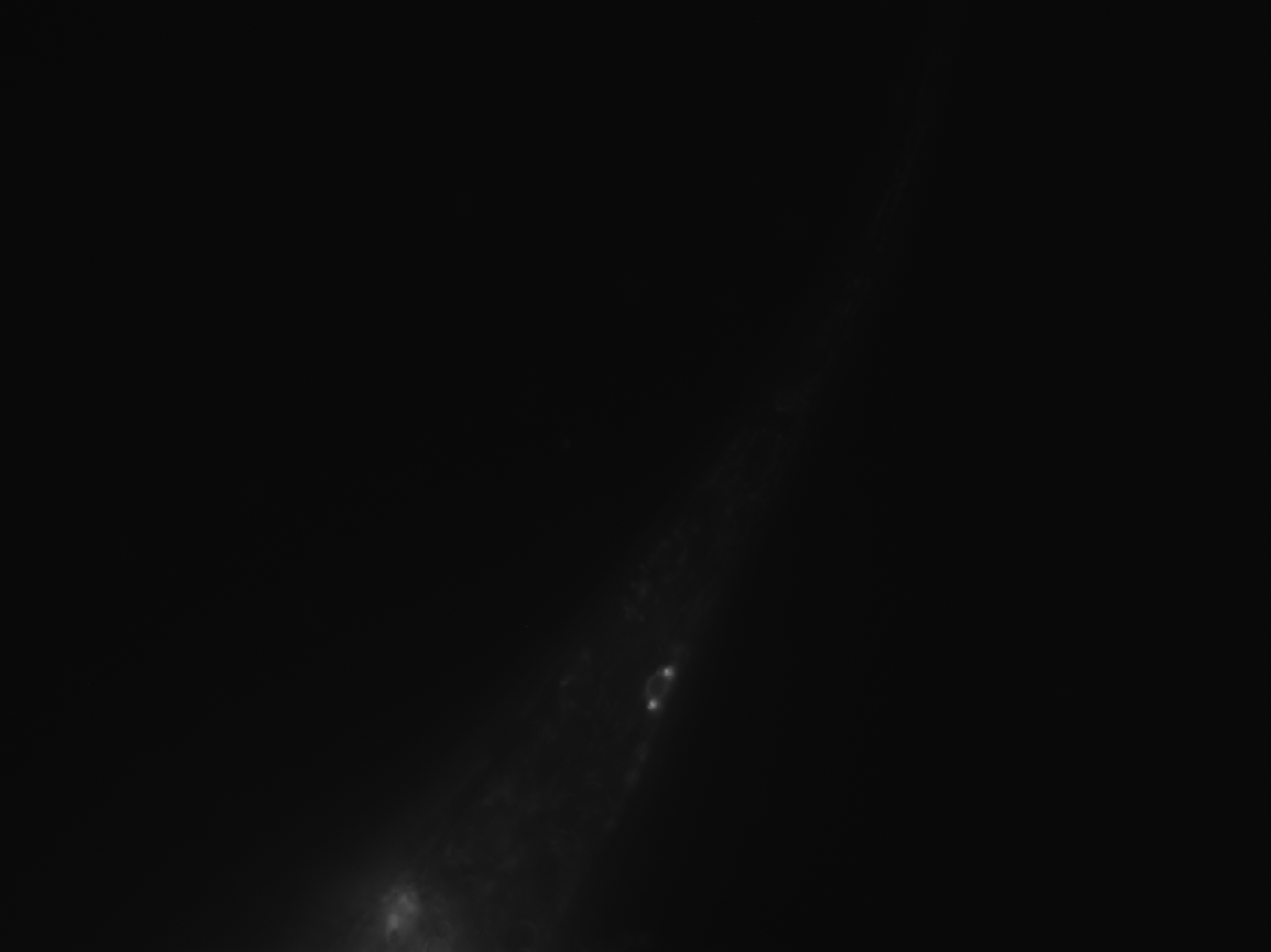

Supplement: Supplementary file 6 — Source data Fig. 5 [file 44319_2025_493_MOESM6_ESM.zip › Figure5/Fig5F/Experiment-110_cellbody_included.tif_files/Experiment-110_z4c0x0-1388y0-1040.tif]

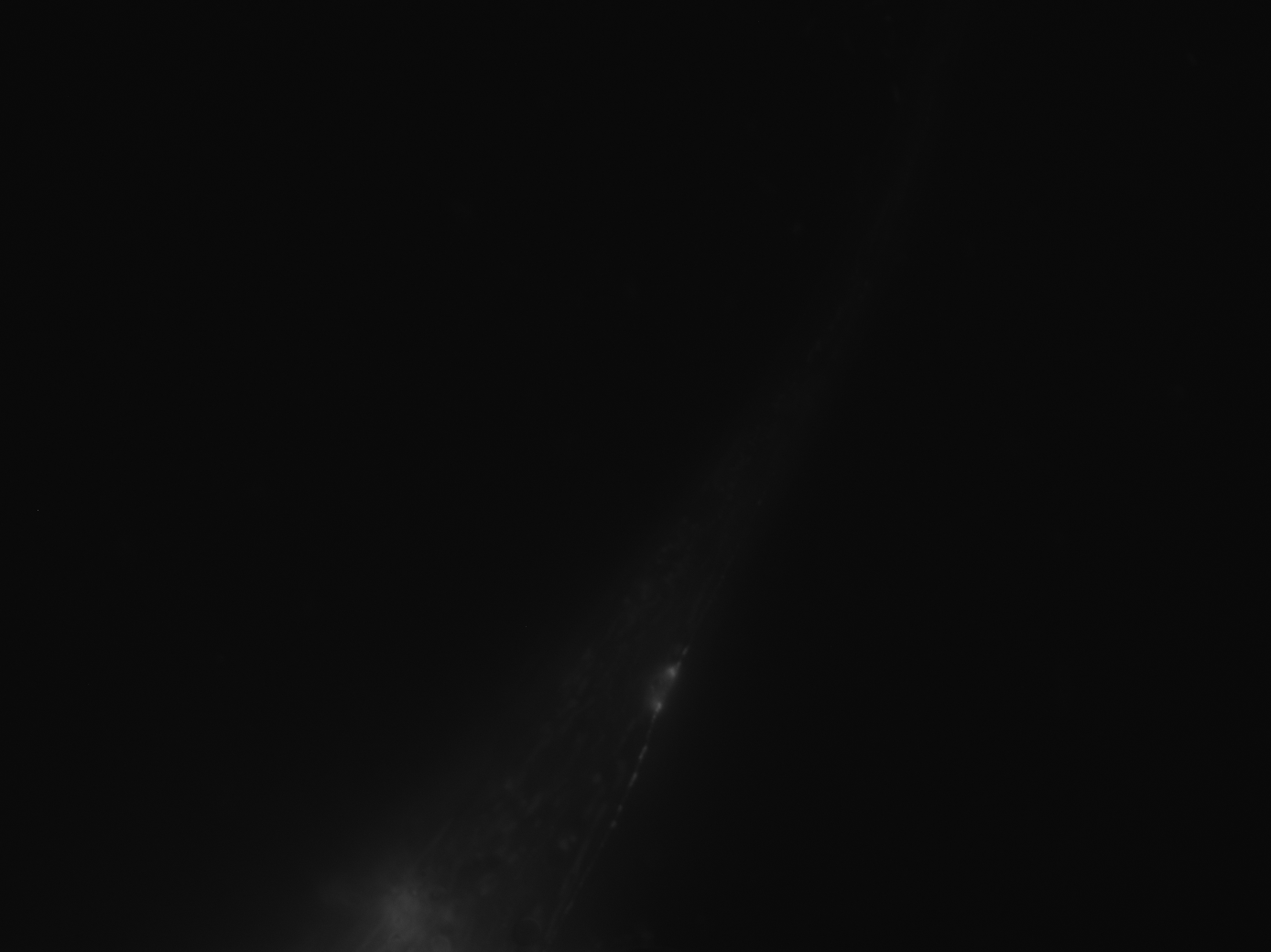

Supplement: Supplementary file 6 — Source data Fig. 5 [file 44319_2025_493_MOESM6_ESM.zip › Figure5/Fig5F/Experiment-110_cellbody_included.tif_files/Experiment-110_z2c0x0-1388y0-1040.tif]

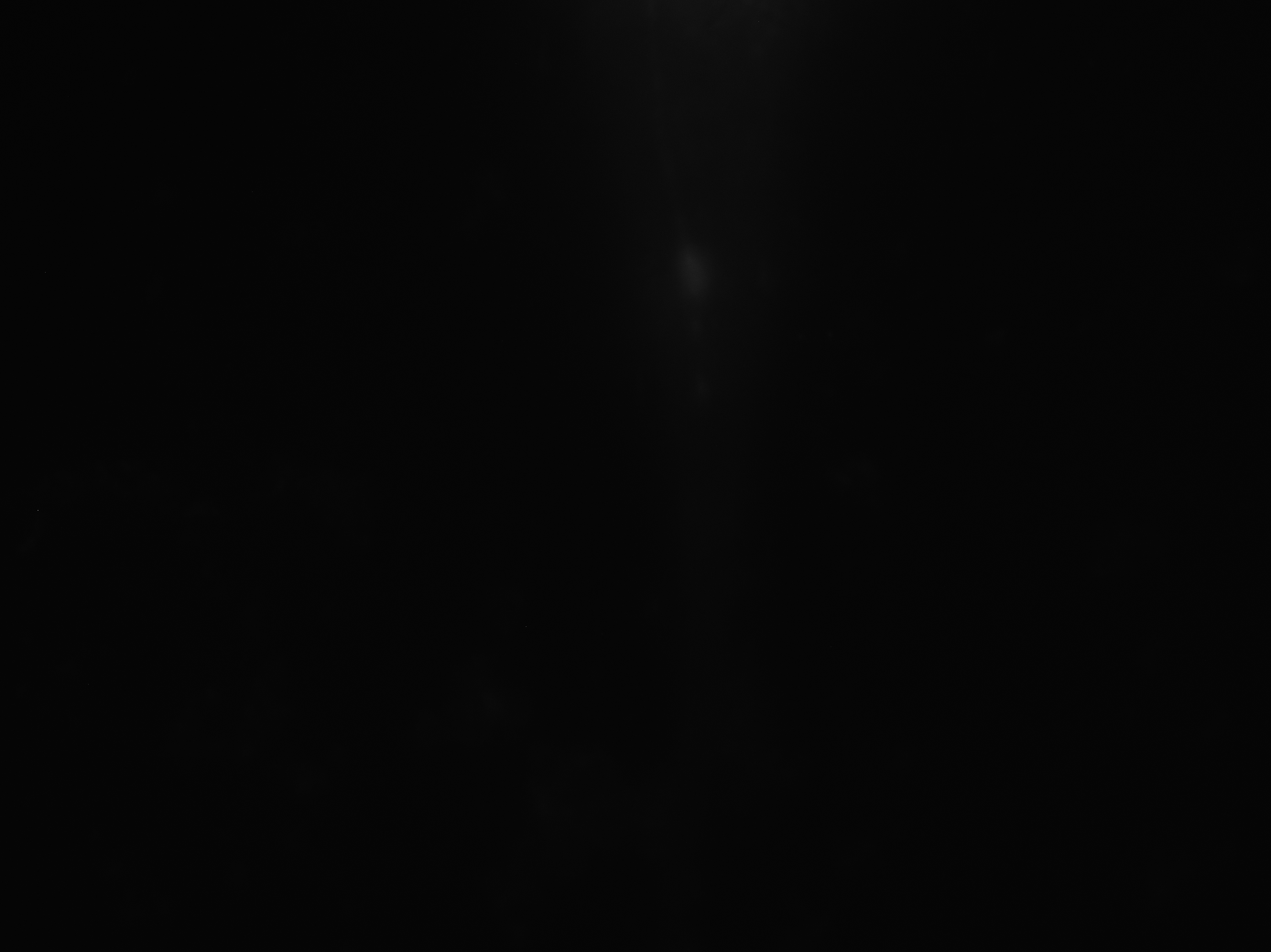

Supplement: Supplementary file 6 — Source data Fig. 5 [file 44319_2025_493_MOESM6_ESM.zip › Figure5/Fig5F/Experiment-962_cellbody_n2813.tif_files/Experiment-962_z1c0x0-1388y0-1040.tif]

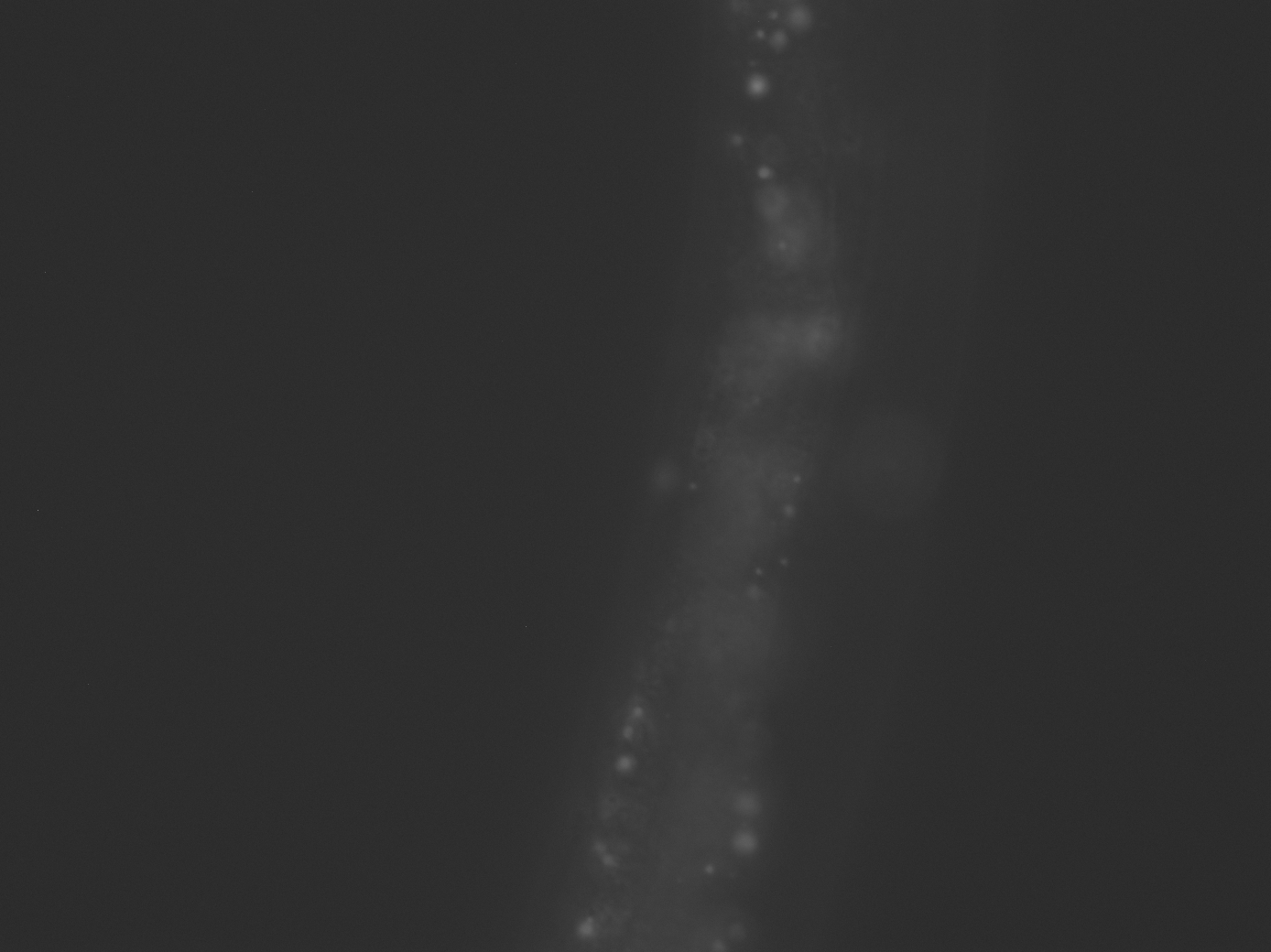

Supplement: Supplementary file 6 — Source data Fig. 5 [file 44319_2025_493_MOESM6_ESM.zip › Figure5/Fig5E/goodALM.tif_files/goodALM_z5c0x0-1388y0-1040.tif]

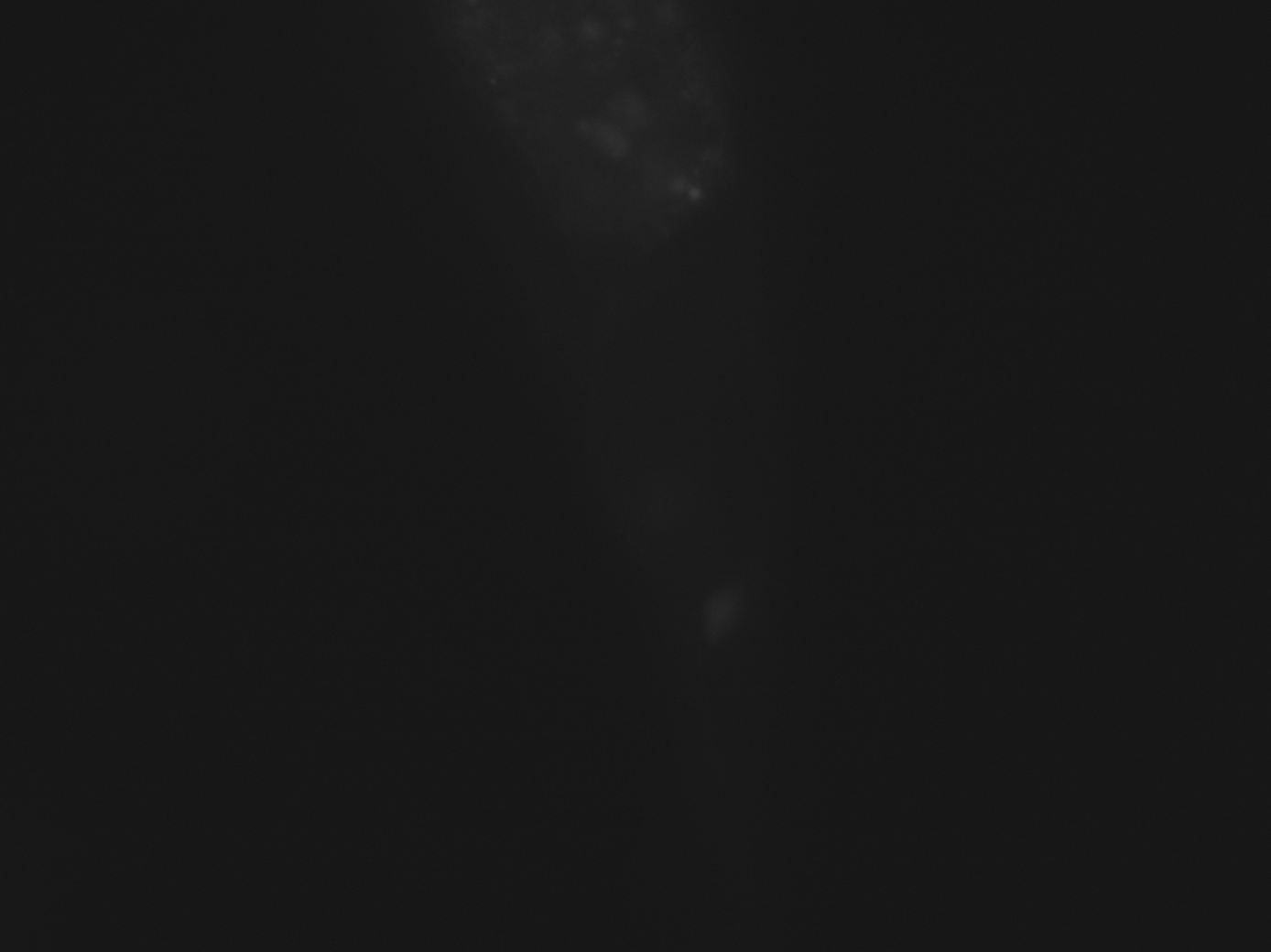

Supplement: Supplementary file 6 — Source data Fig. 5 [file 44319_2025_493_MOESM6_ESM.zip › Figure5/Fig5E/good_PLM.tif_files/good_z12c2x0-1388y0-1040.tif]

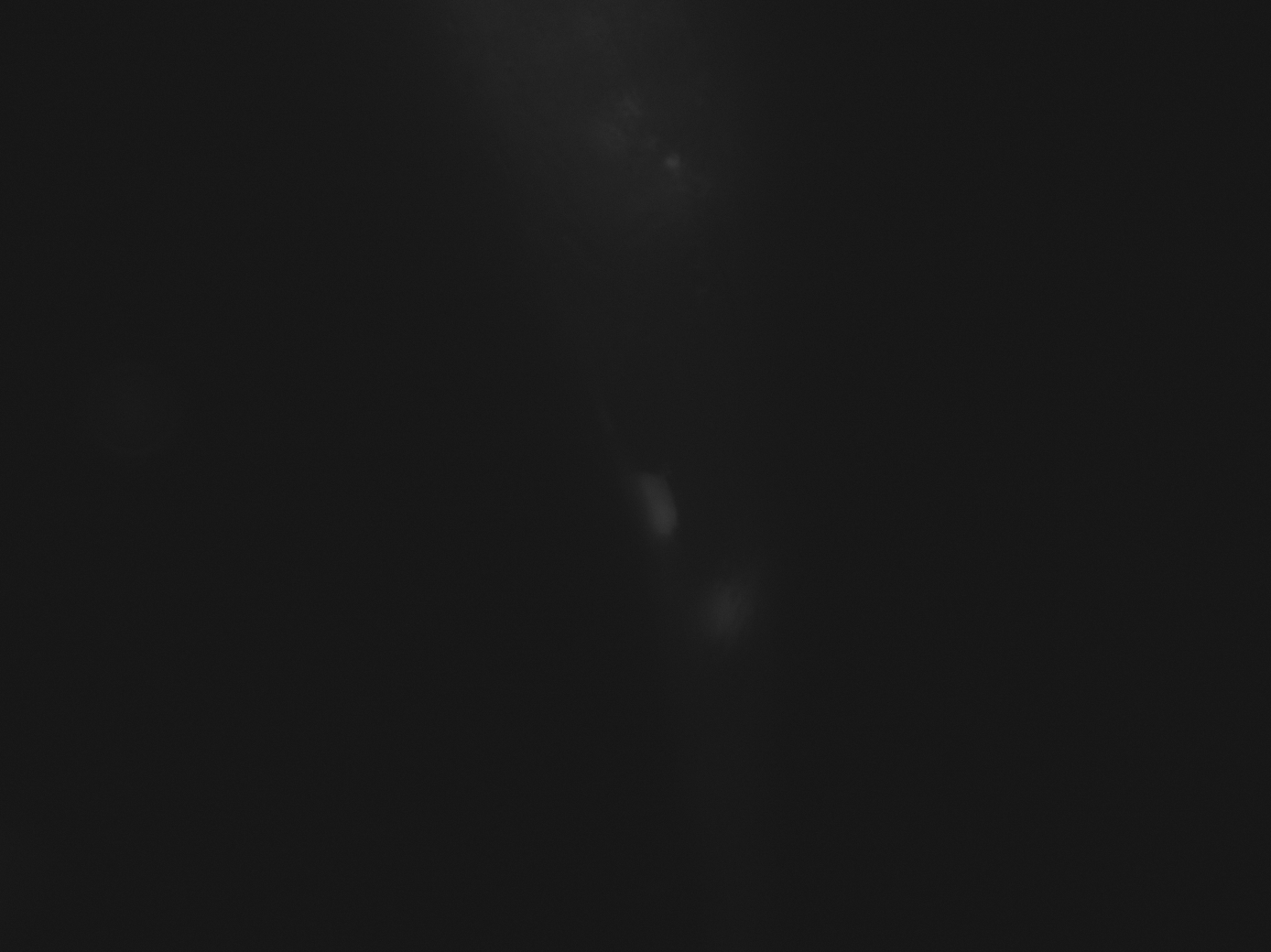

Supplement: Supplementary file 6 — Source data Fig. 5 [file 44319_2025_493_MOESM6_ESM.zip › Figure5/Fig5E/good_PLM.tif_files/good_z6c2x0-1388y0-1040.tif]

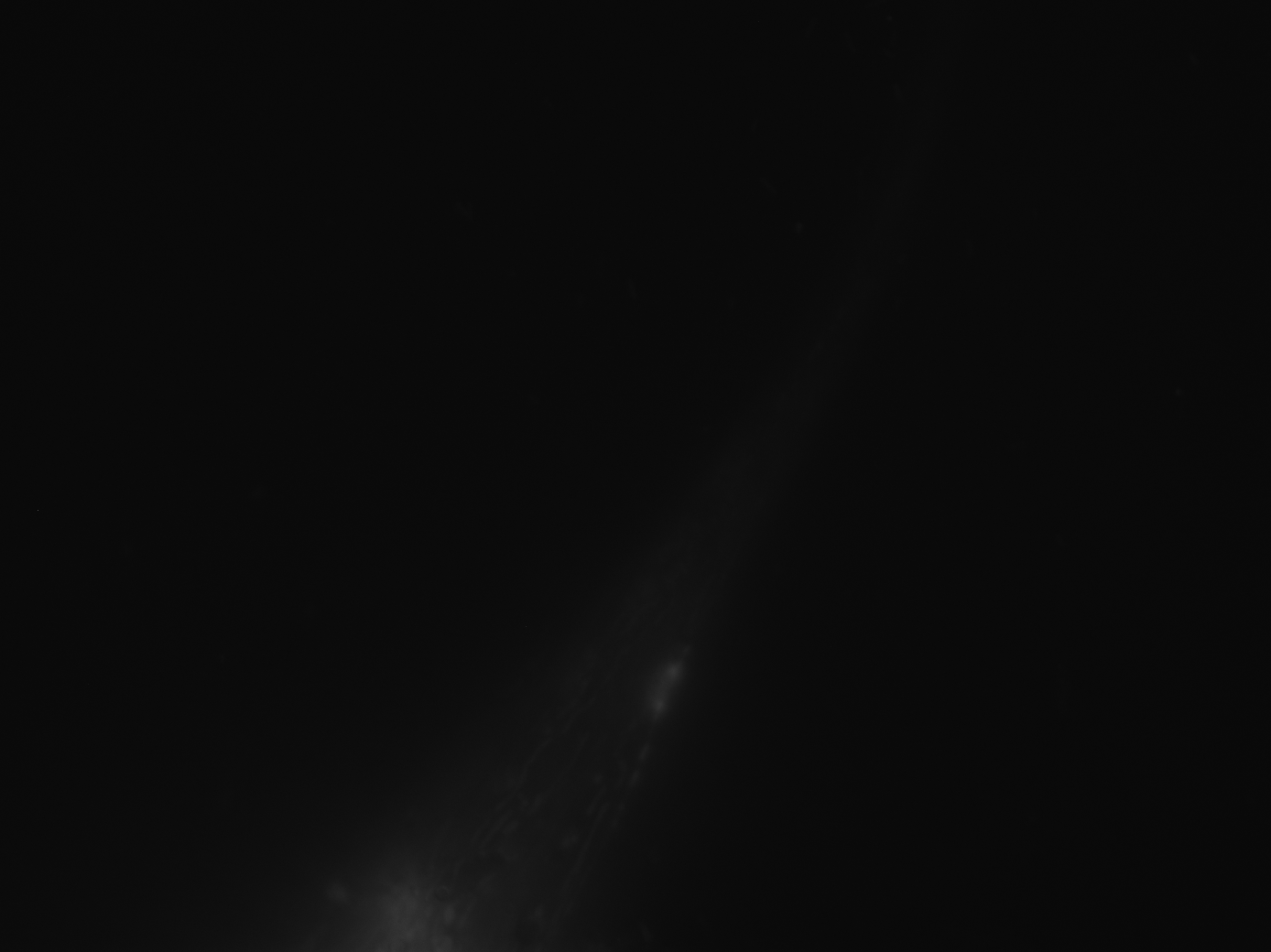

Supplement: Supplementary file 6 — Source data Fig. 5 [file 44319_2025_493_MOESM6_ESM.zip › Figure5/Fig5F/Experiment-110_cellbody_included.tif_files/Experiment-110_z1c0x0-1388y0-1040.tif]

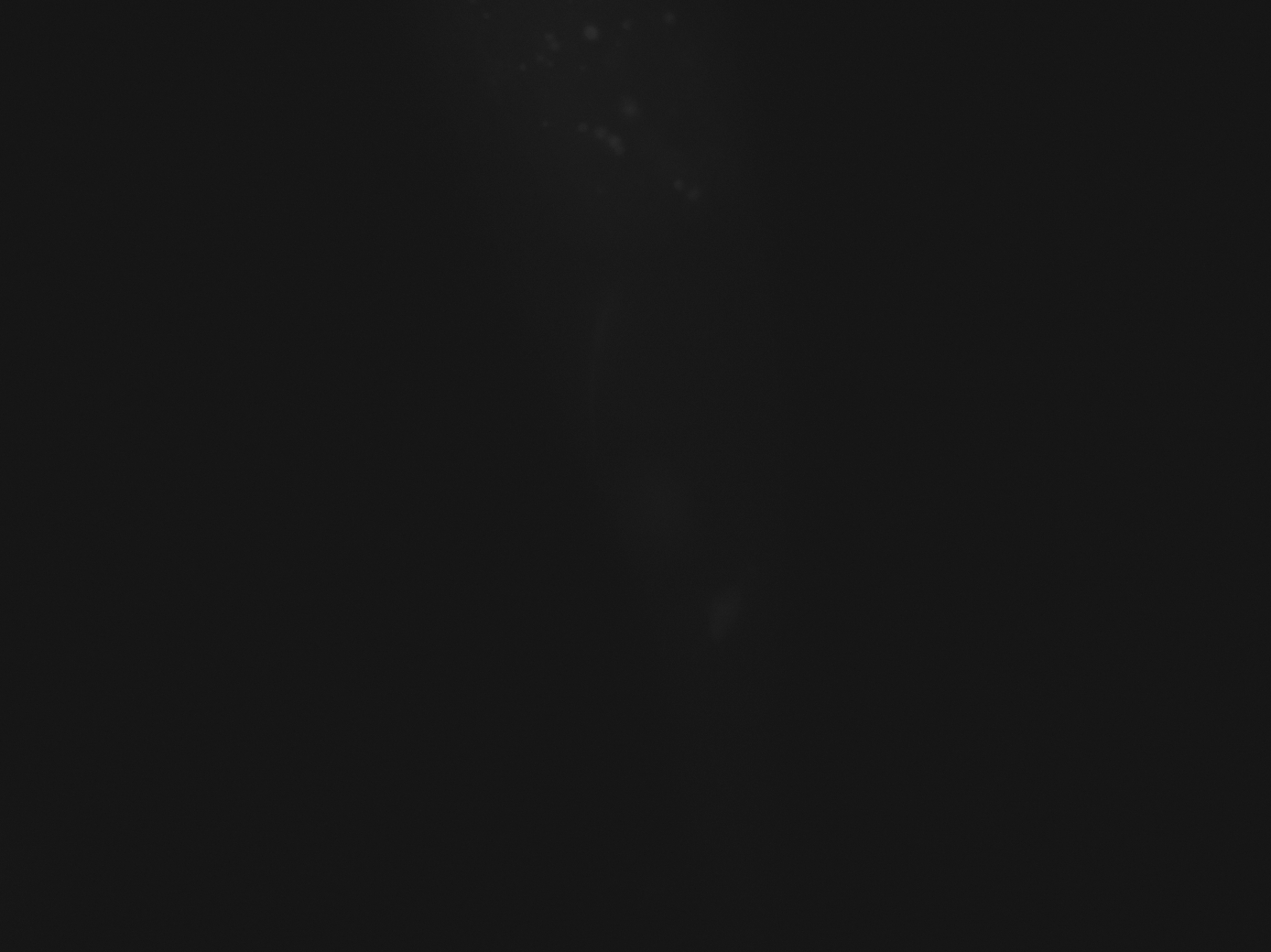

Supplement: Supplementary file 6 — Source data Fig. 5 [file 44319_2025_493_MOESM6_ESM.zip › Figure5/Fig5E/good_PLM.tif_files/good_z10c1x0-1388y0-1040.tif]
