# Supplementary figures and images for: Regulated microexon alternative splicing in single neurons tunes synaptic function (part 5 of 6)
Source: EMBO Rep. 2025 Jun 9;26(14):3640–62. doi: 10.1038/s44319-025-00493-7 (PMC12287369; doi:10.1038/s44319-025-00493-7)

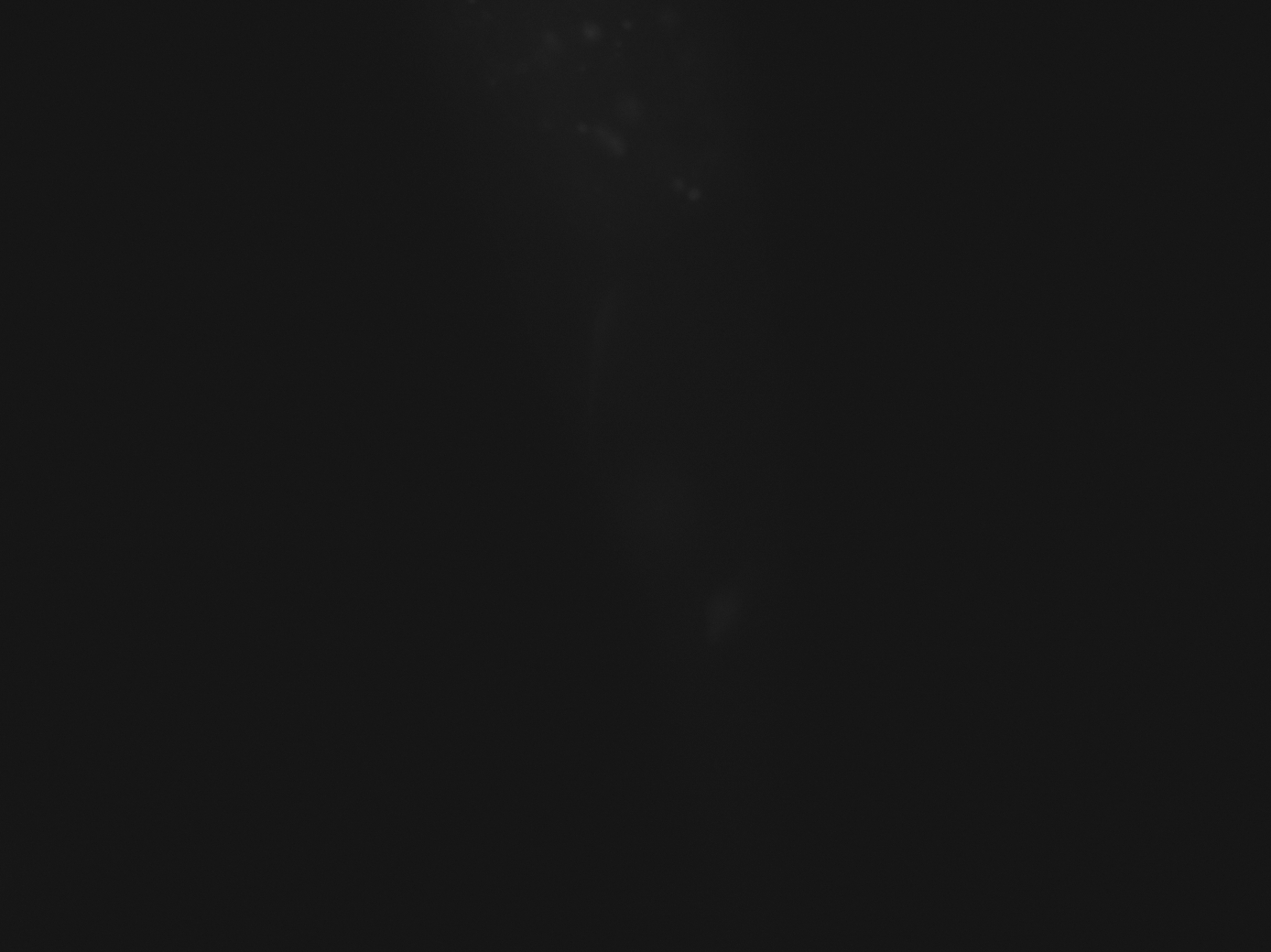

Supplement: Supplementary file 6 — Source data Fig. 5 [file 44319_2025_493_MOESM6_ESM.zip › Figure5/Fig5E/good_PLM.tif_files/good_z11c1x0-1388y0-1040.tif]

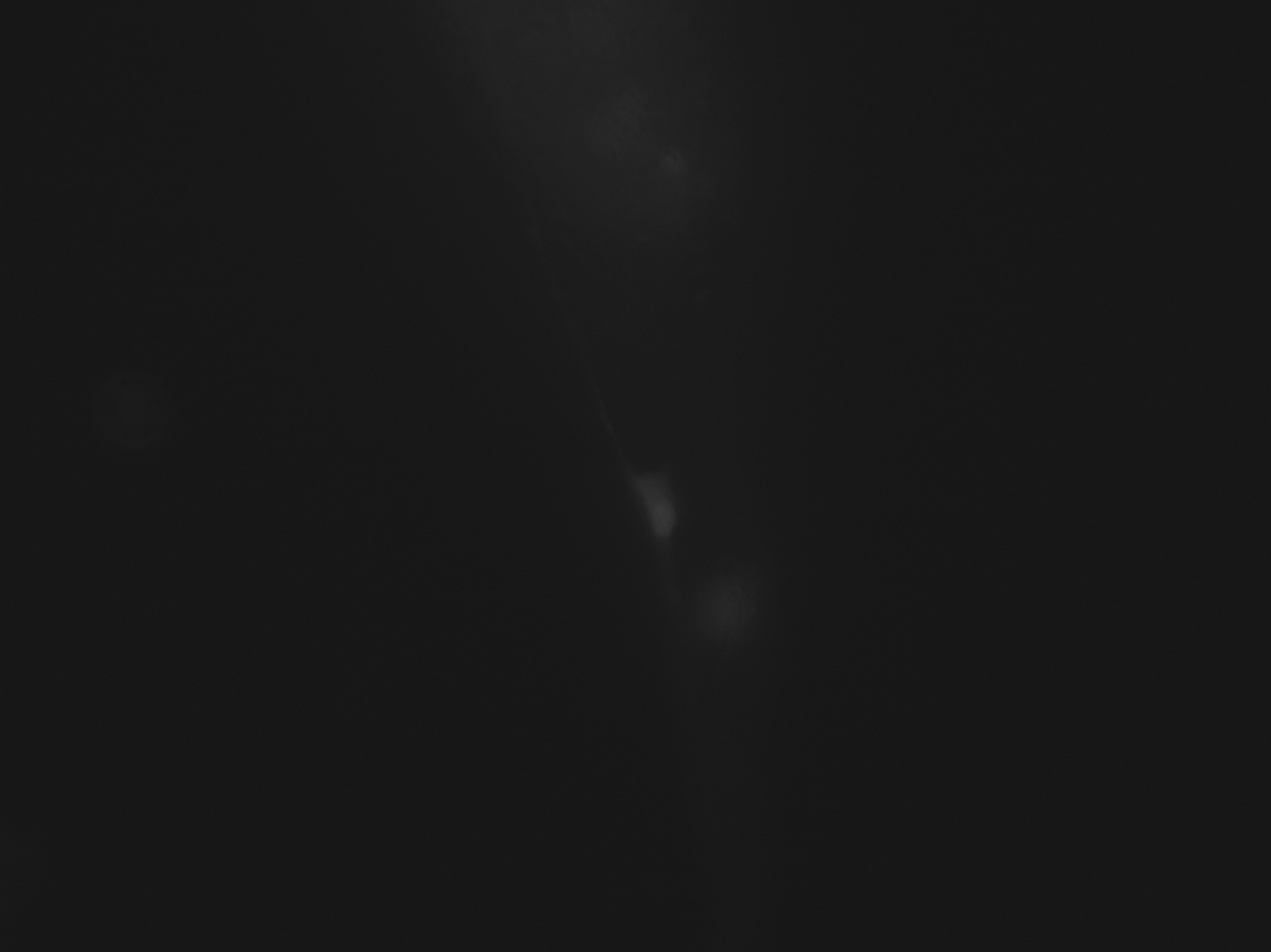

Supplement: Supplementary file 6 — Source data Fig. 5 [file 44319_2025_493_MOESM6_ESM.zip › Figure5/Fig5E/good_PLM.tif_files/good_z5c2x0-1388y0-1040.tif]

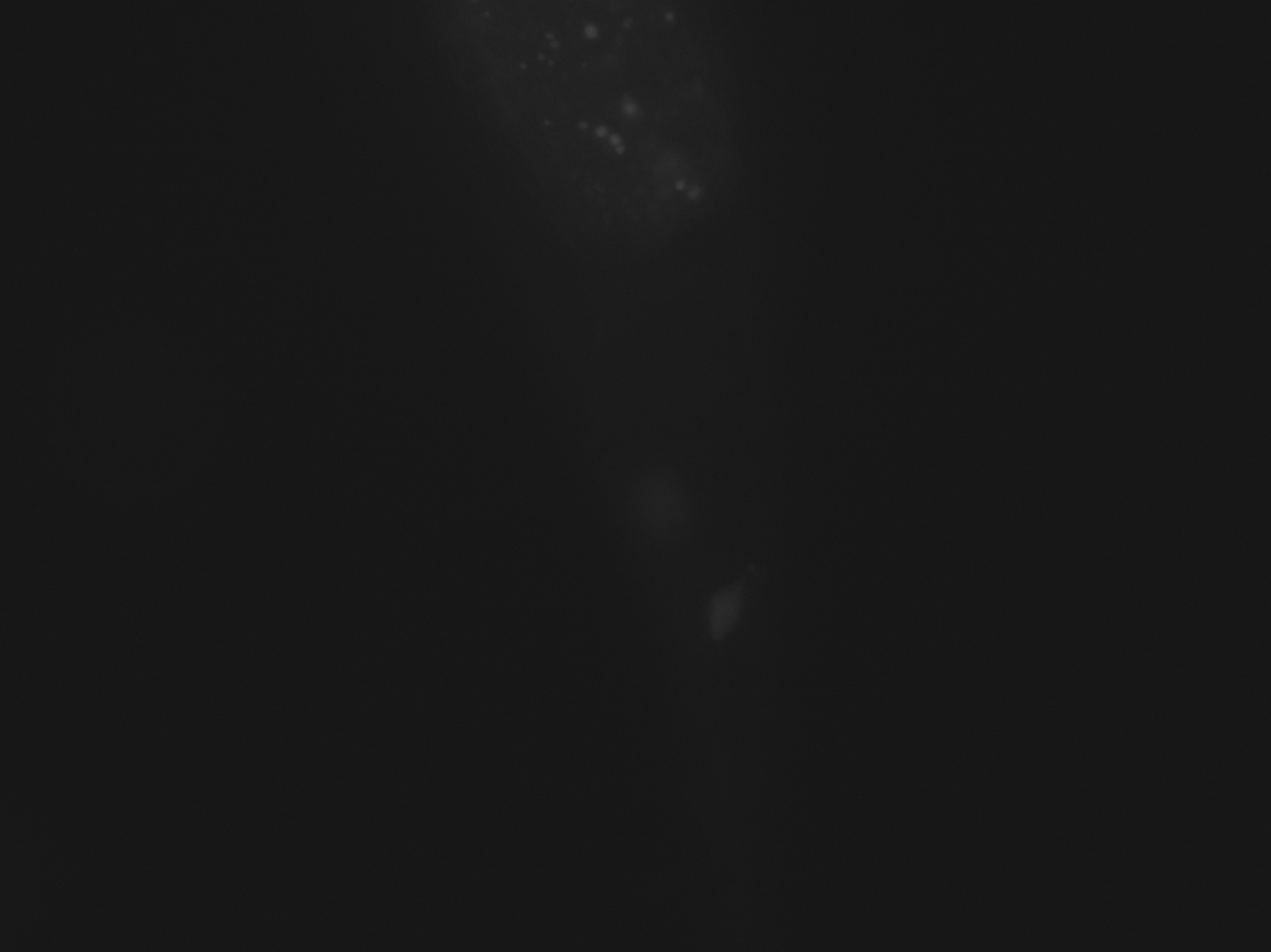

Supplement: Supplementary file 6 — Source data Fig. 5 [file 44319_2025_493_MOESM6_ESM.zip › Figure5/Fig5E/good_PLM.tif_files/good_z10c2x0-1388y0-1040.tif]

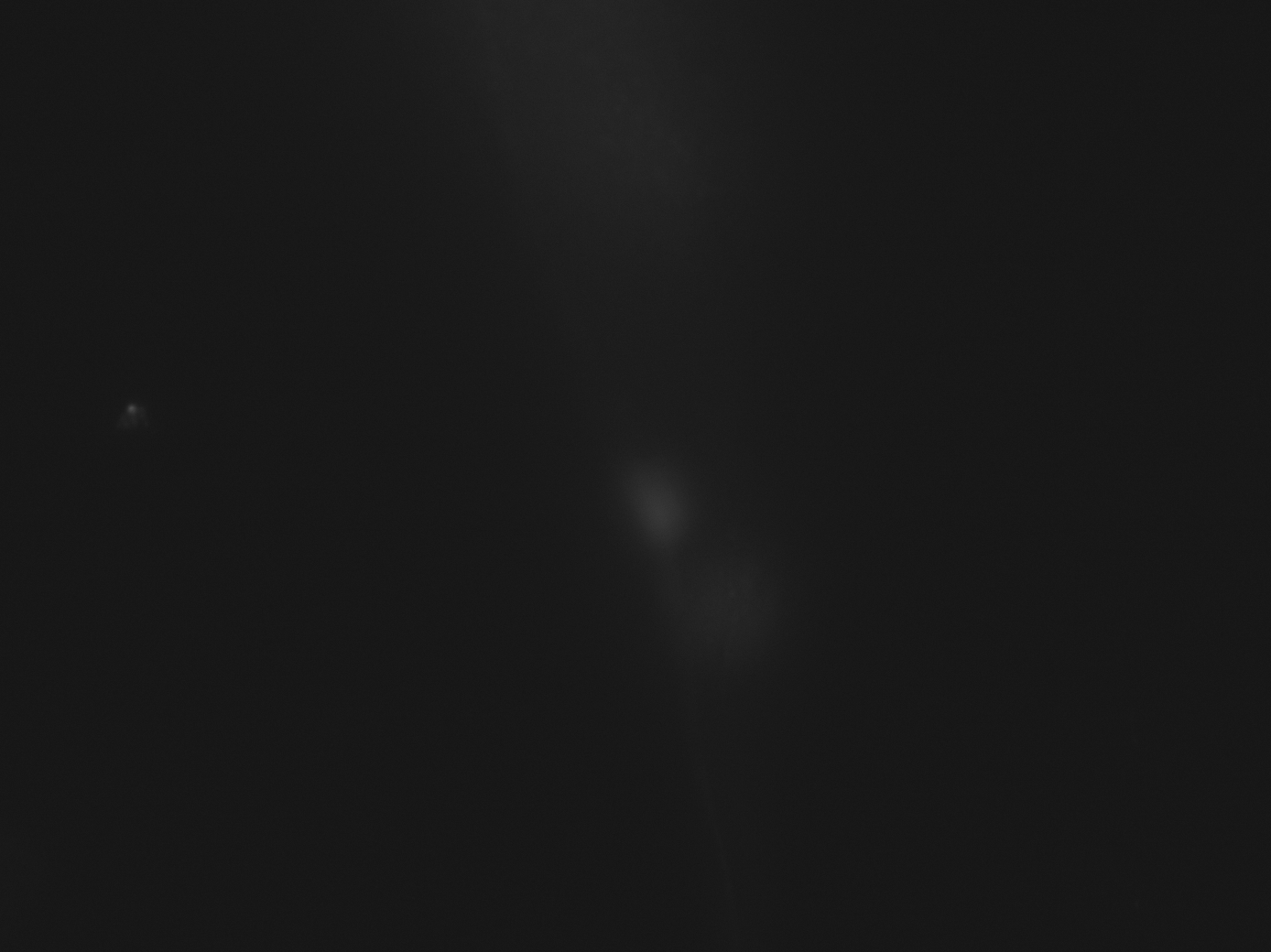

Supplement: Supplementary file 6 — Source data Fig. 5 [file 44319_2025_493_MOESM6_ESM.zip › Figure5/Fig5E/good_PLM.tif_files/good_z0c2x0-1388y0-1040.tif]

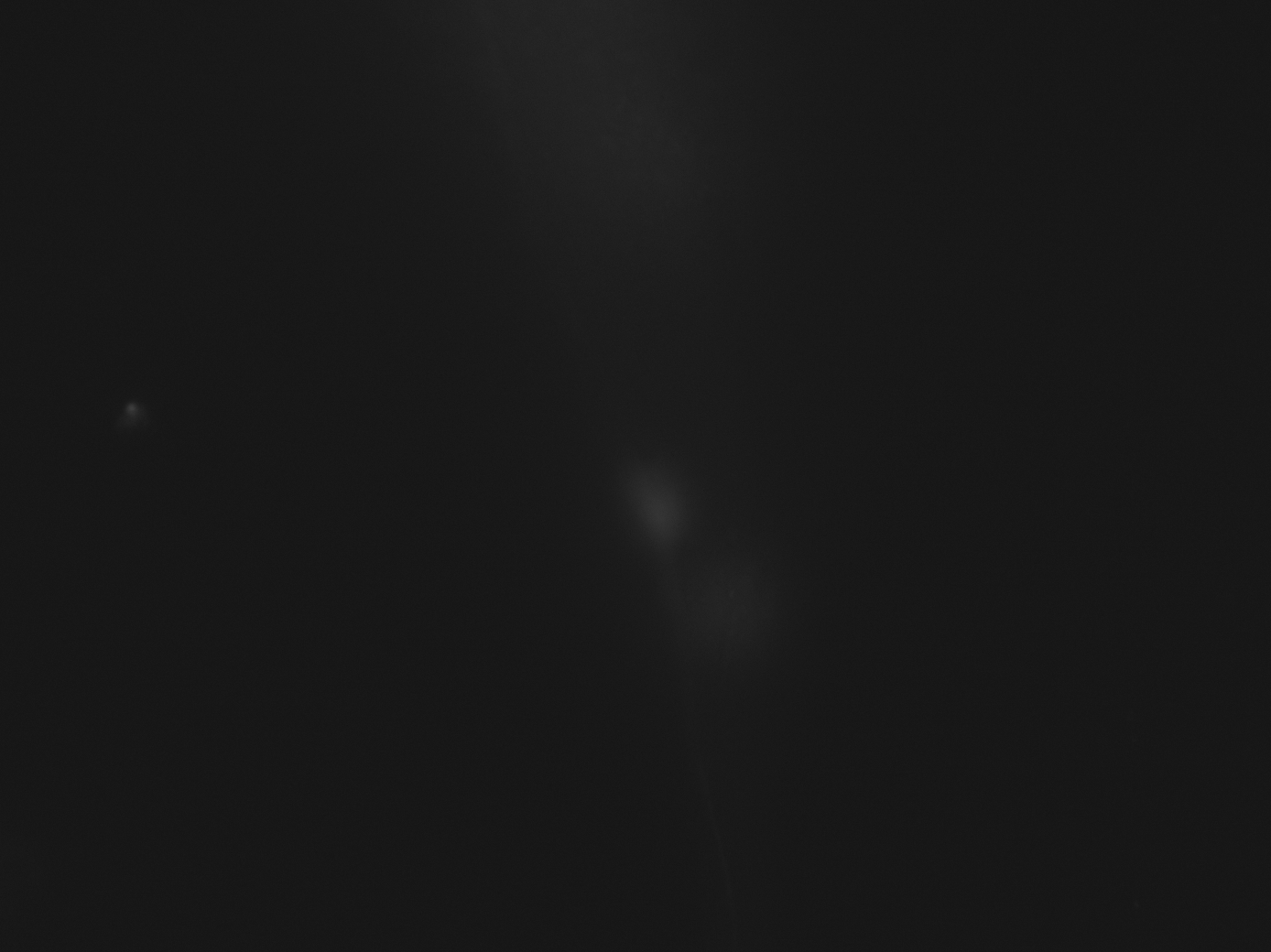

Supplement: Supplementary file 6 — Source data Fig. 5 [file 44319_2025_493_MOESM6_ESM.zip › Figure5/Fig5E/good_PLM.tif_files/good_z1c2x0-1388y0-1040.tif]

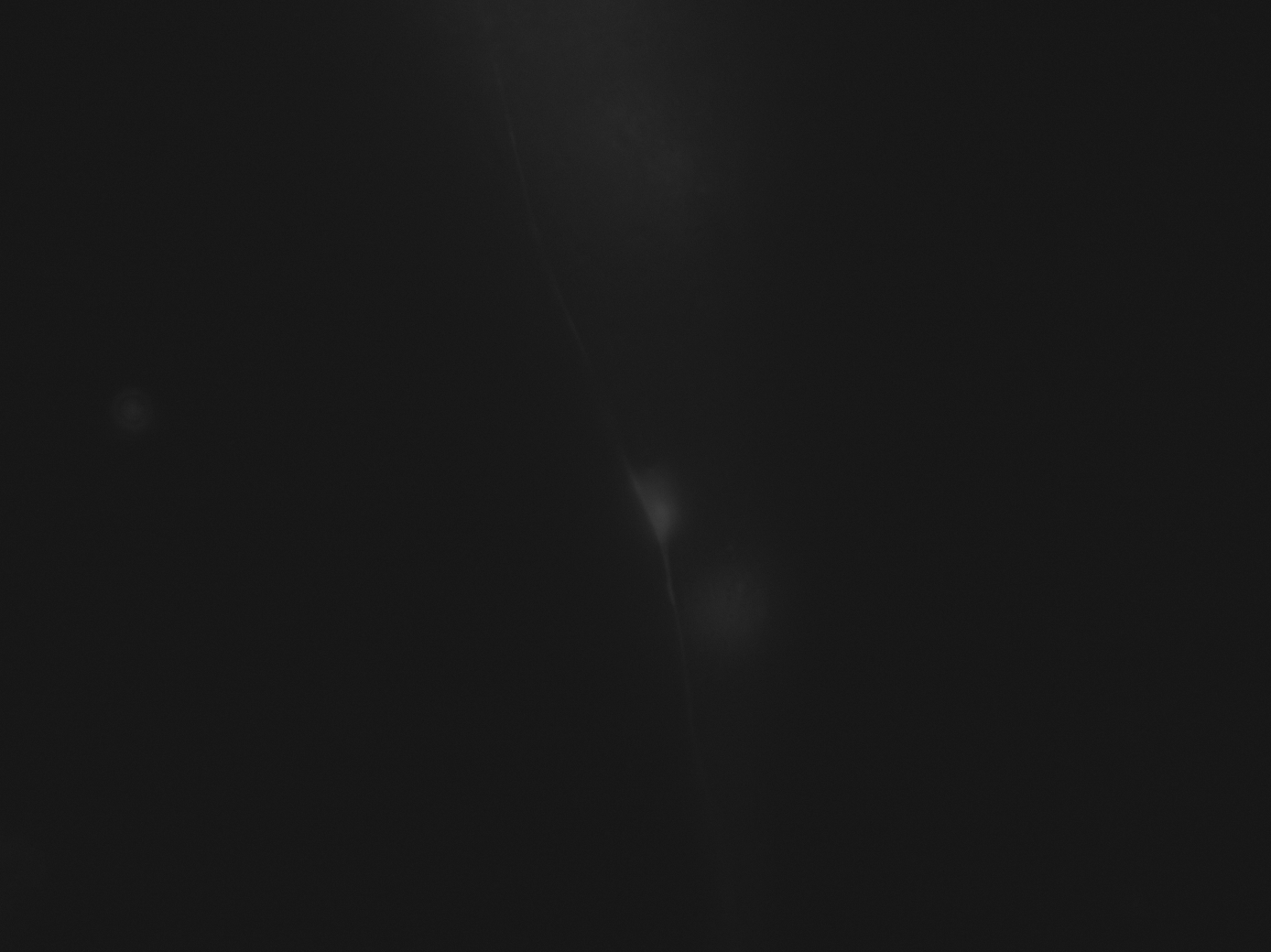

Supplement: Supplementary file 6 — Source data Fig. 5 [file 44319_2025_493_MOESM6_ESM.zip › Figure5/Fig5E/good_PLM.tif_files/good_z3c2x0-1388y0-1040.tif]

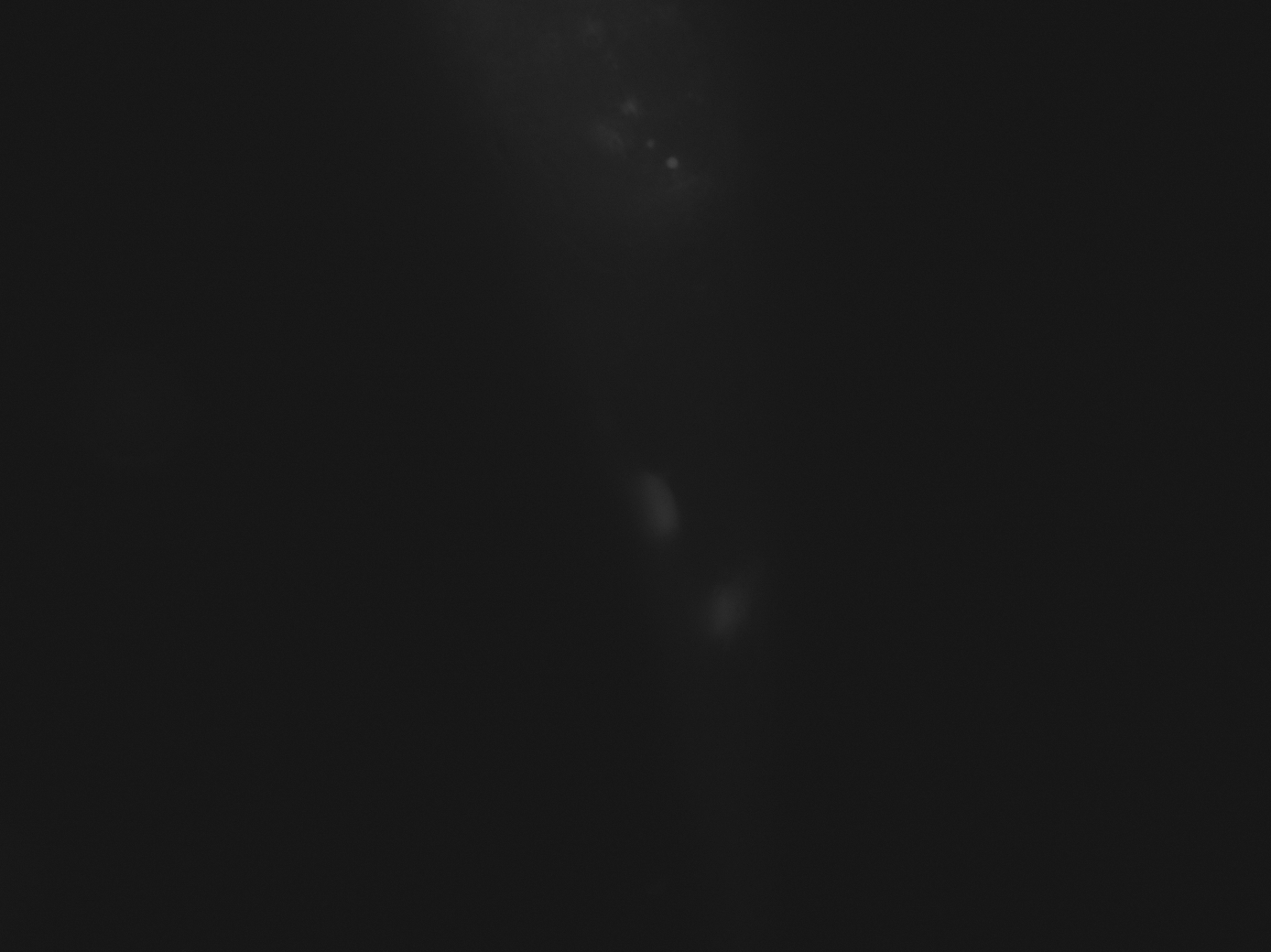

Supplement: Supplementary file 6 — Source data Fig. 5 [file 44319_2025_493_MOESM6_ESM.zip › Figure5/Fig5E/good_PLM.tif_files/good_z7c2x0-1388y0-1040.tif]

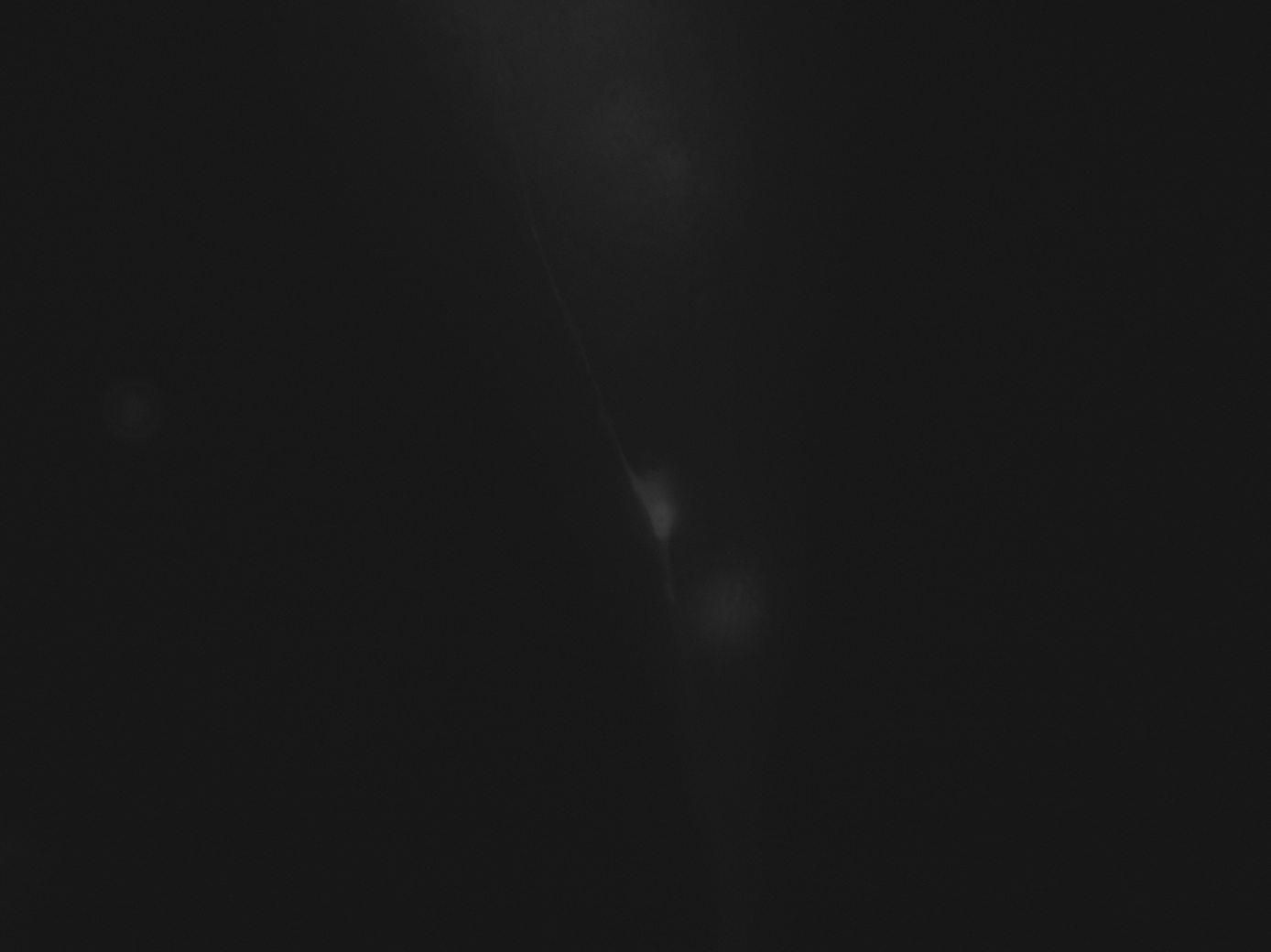

Supplement: Supplementary file 6 — Source data Fig. 5 [file 44319_2025_493_MOESM6_ESM.zip › Figure5/Fig5E/good_PLM.tif_files/good_z4c2x0-1388y0-1040.tif]

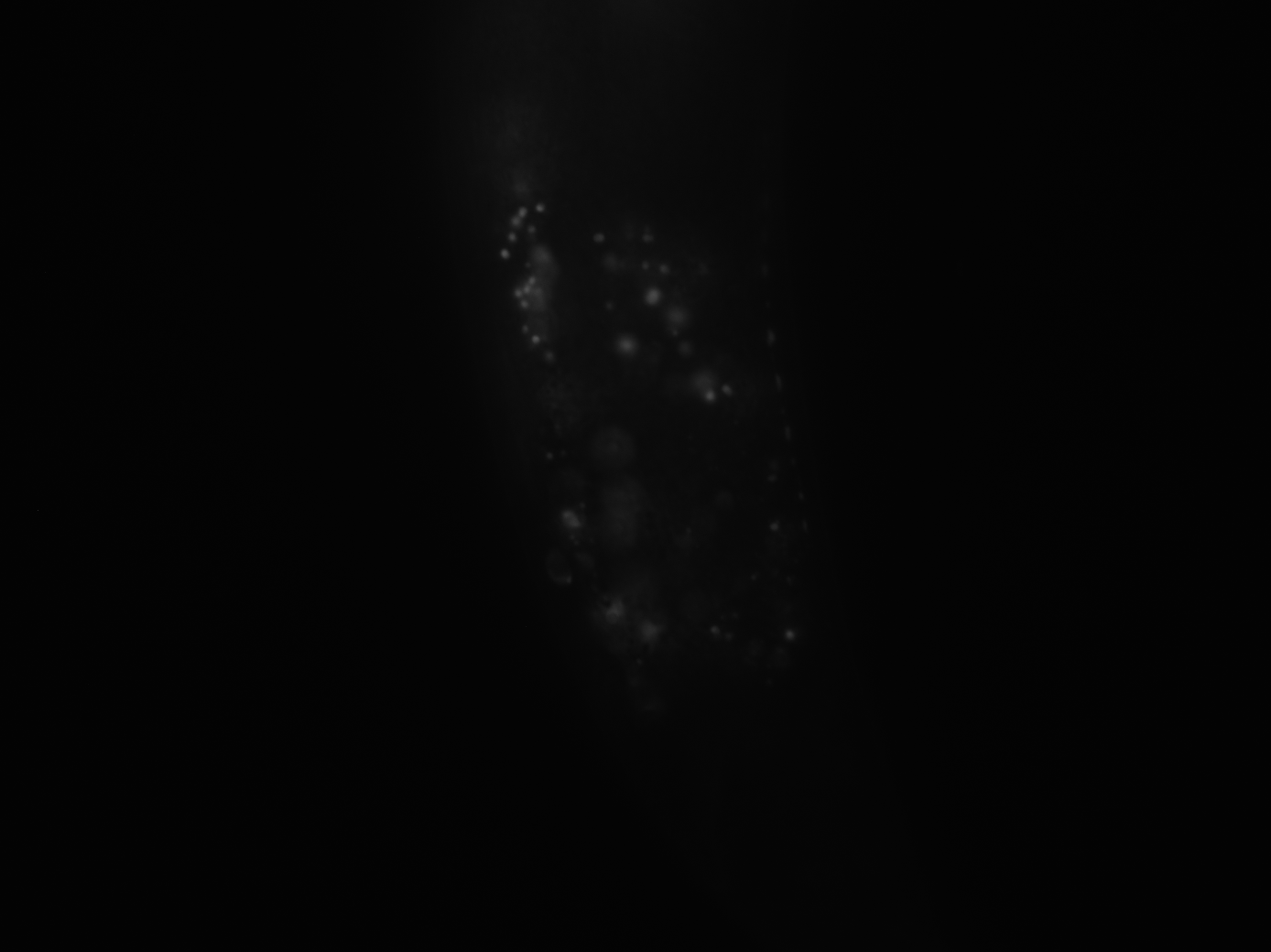

Supplement: Supplementary file 7 — Source data Fig. 6 [file 44319_2025_493_MOESM7_ESM.zip › Figure6/Fig6H/Experiment-07_GABA_s69.tif_files/Experiment-07_z2c0x0-1388y0-1040.tif]

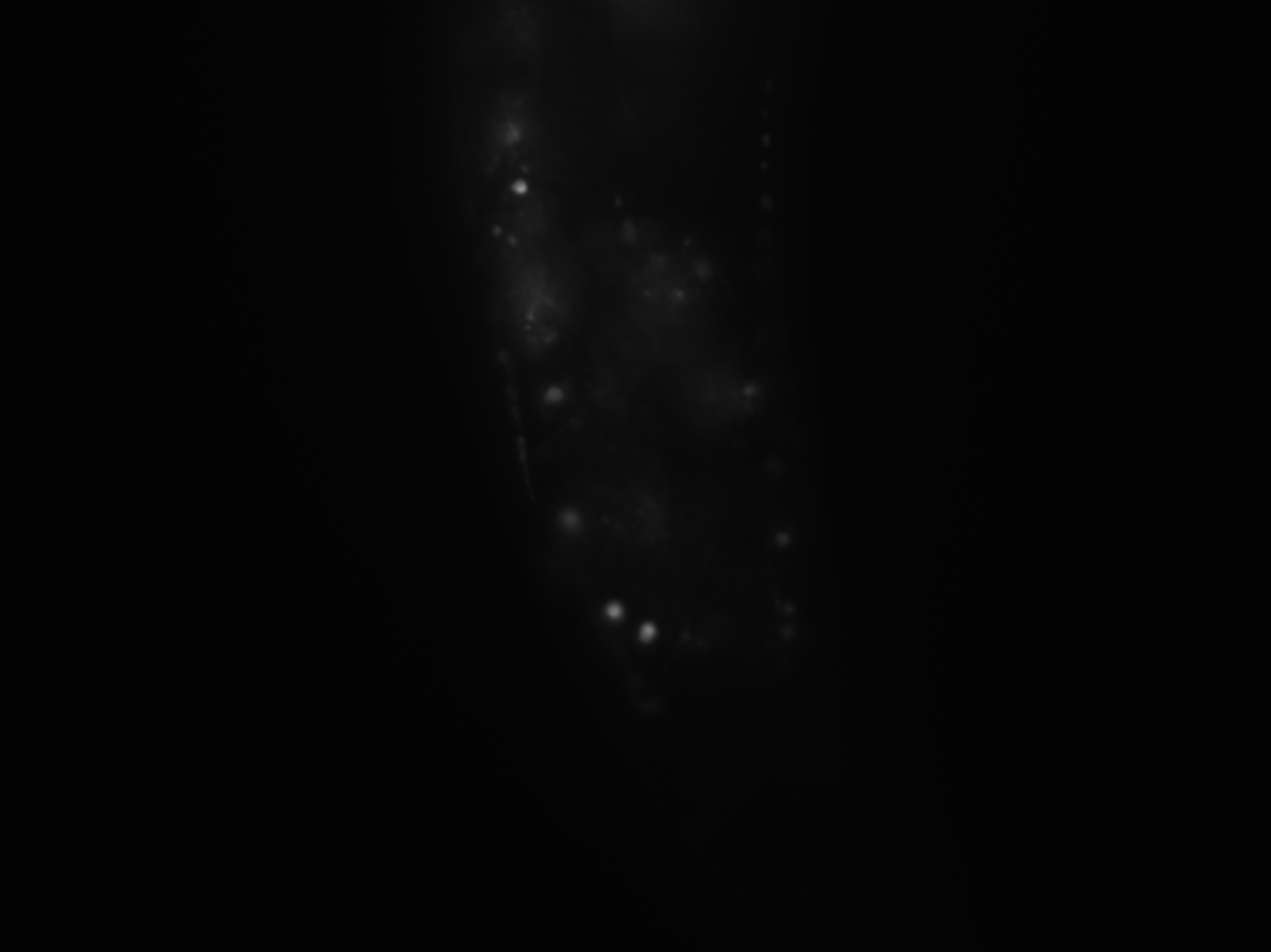

Supplement: Supplementary file 7 — Source data Fig. 6 [file 44319_2025_493_MOESM7_ESM.zip › Figure6/Fig6H/Experiment-07_GABA_s69.tif_files/Experiment-07_z6c0x0-1388y0-1040.tif]

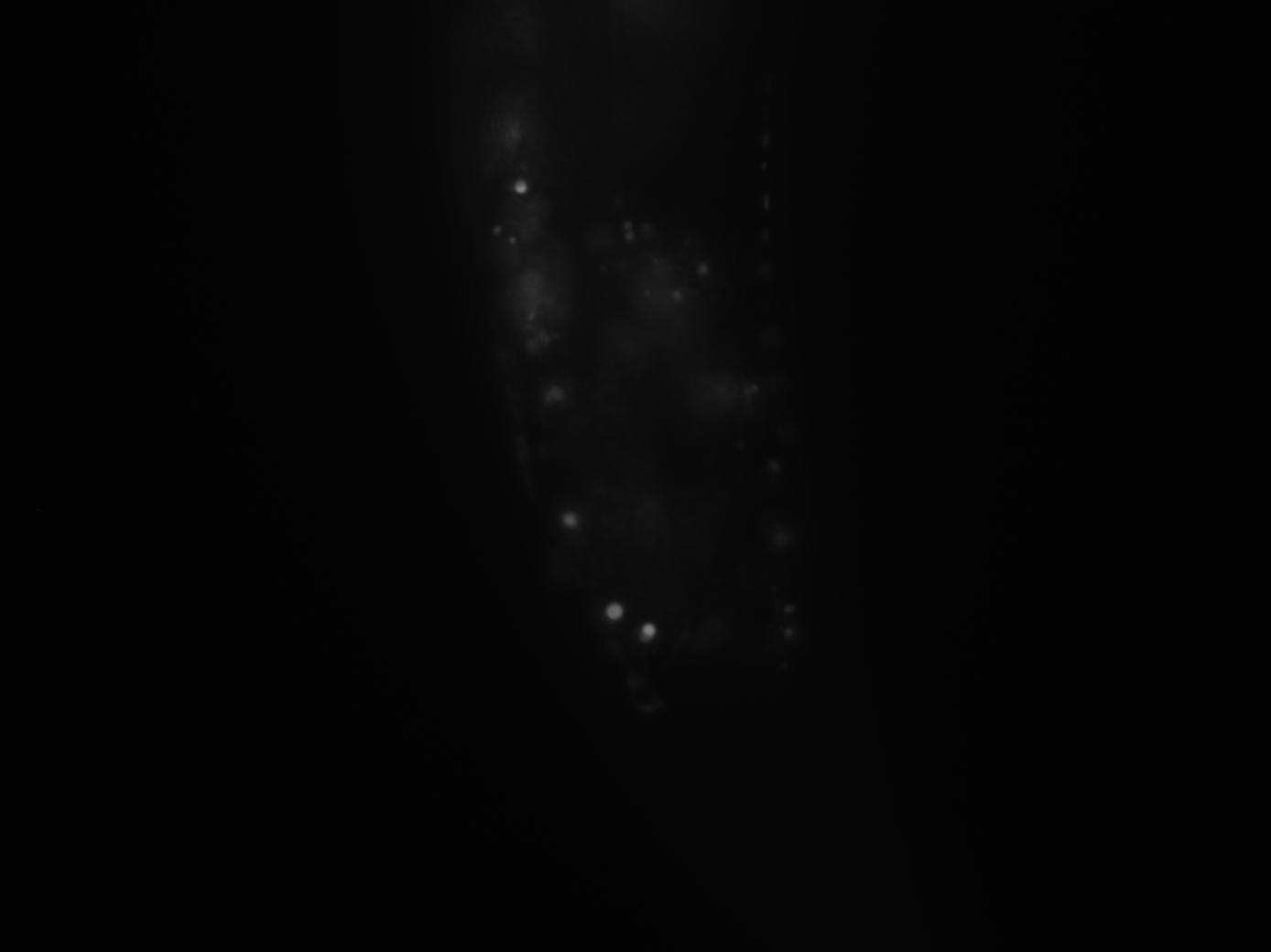

Supplement: Supplementary file 7 — Source data Fig. 6 [file 44319_2025_493_MOESM7_ESM.zip › Figure6/Fig6H/Experiment-07_GABA_s69.tif_files/Experiment-07_z5c0x0-1388y0-1040.tif]

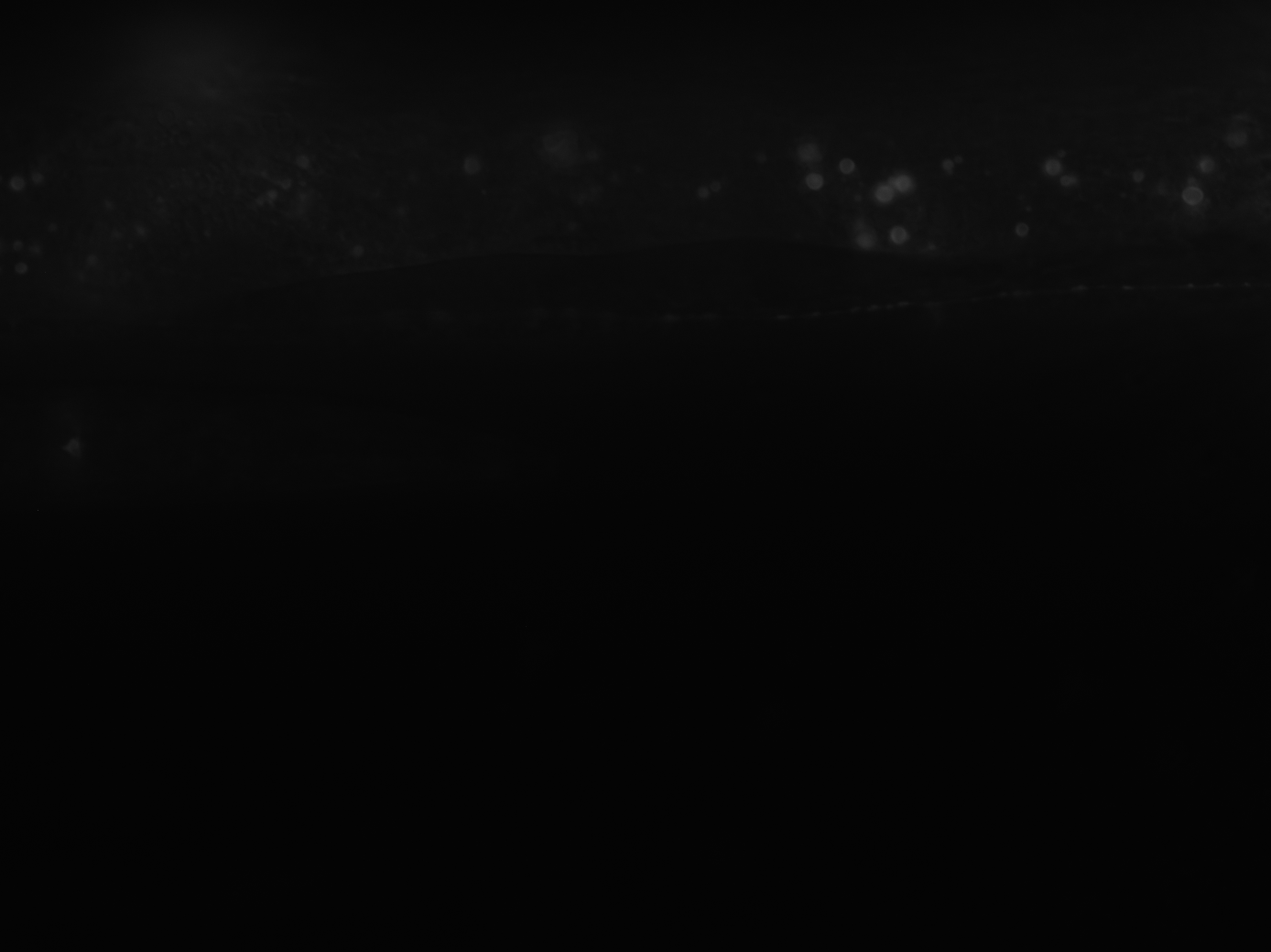

Supplement: Supplementary file 7 — Source data Fig. 6 [file 44319_2025_493_MOESM7_ESM.zip › Figure6/Fig6H/Experiment-19_GABA_OEskipped.tif_files/Experiment-19_z4c0x0-1388y0-1040.tif]

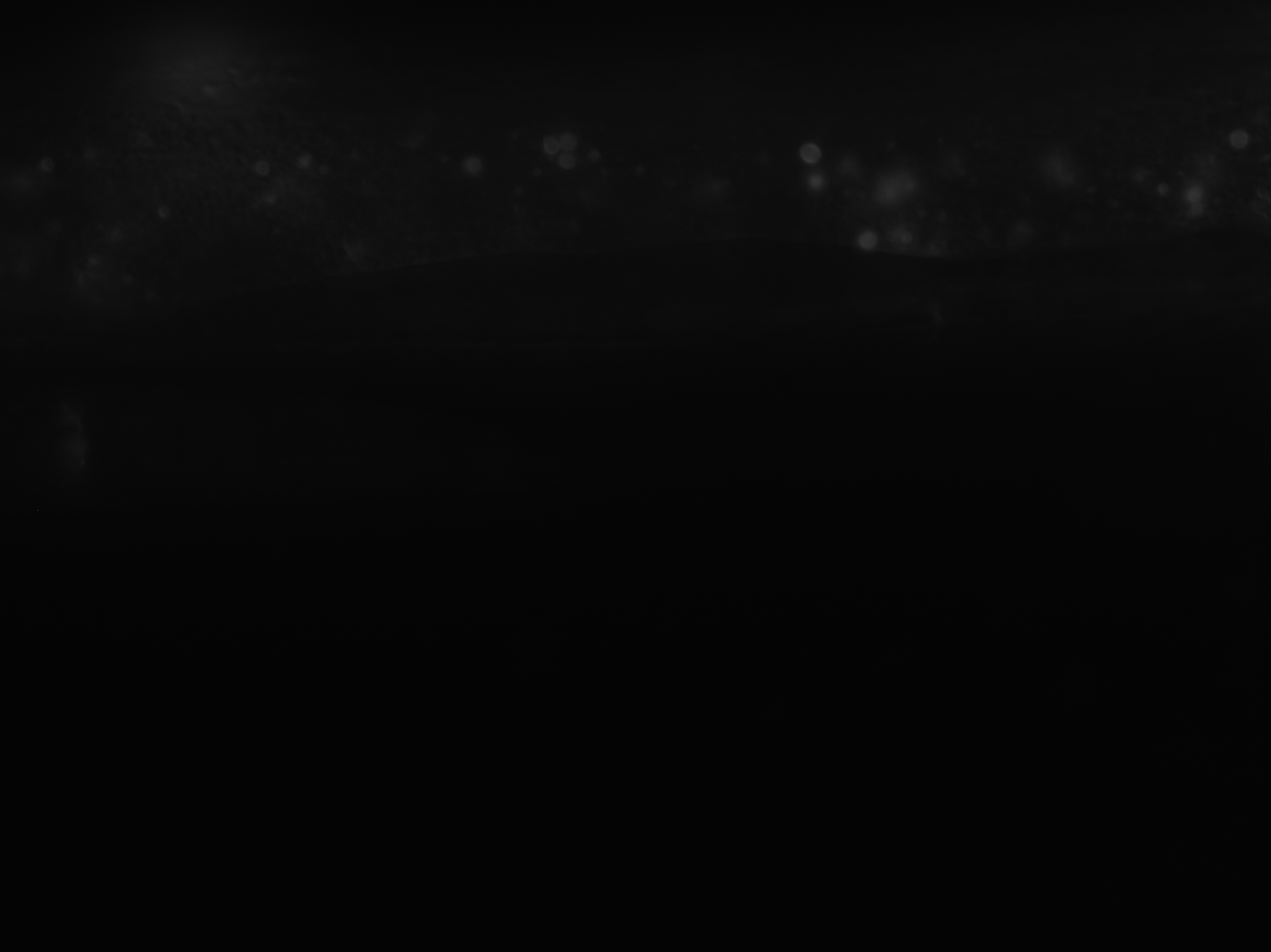

Supplement: Supplementary file 7 — Source data Fig. 6 [file 44319_2025_493_MOESM7_ESM.zip › Figure6/Fig6H/Experiment-19_GABA_OEskipped.tif_files/Experiment-19_z6c0x0-1388y0-1040.tif]

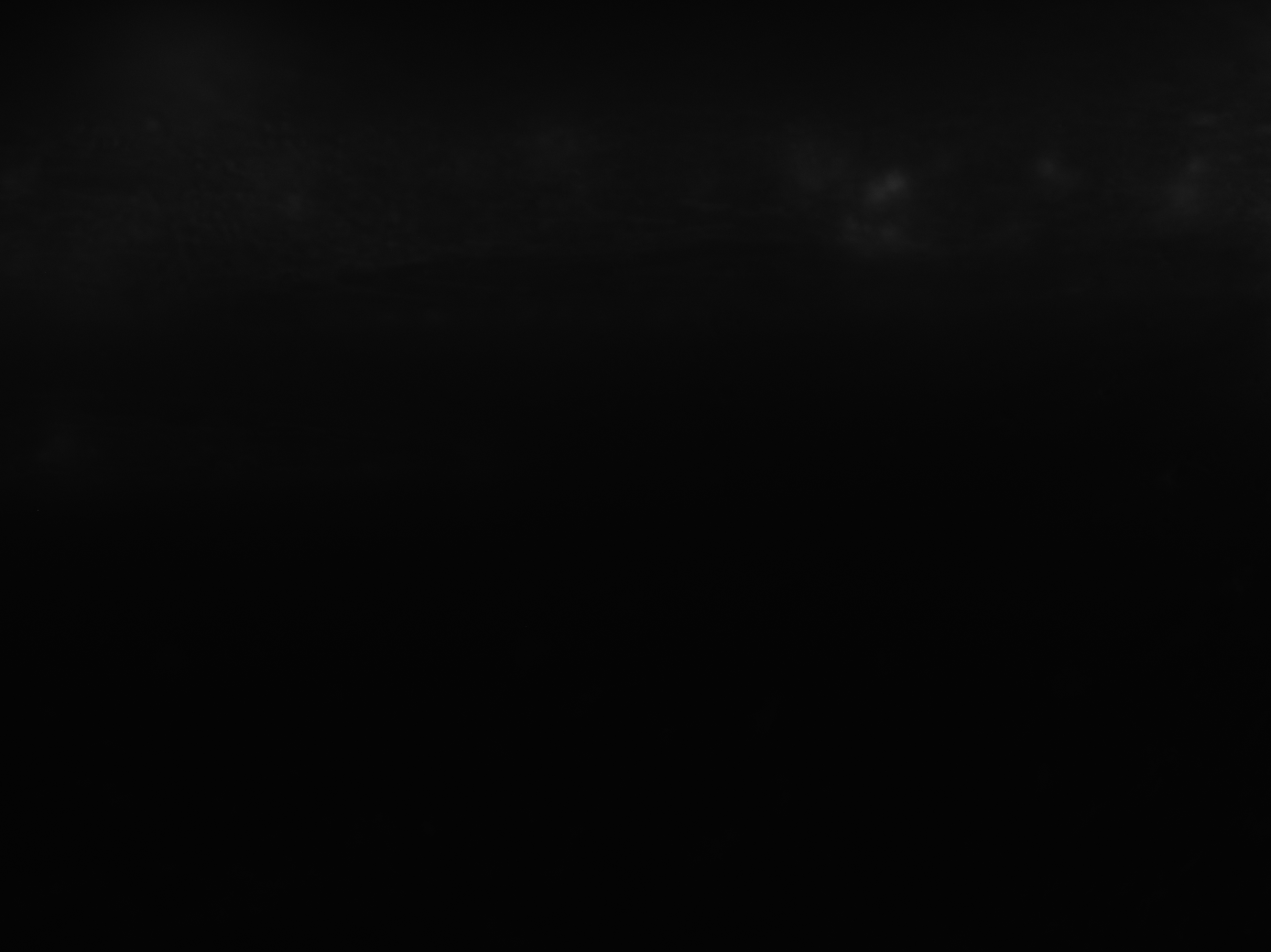

Supplement: Supplementary file 7 — Source data Fig. 6 [file 44319_2025_493_MOESM7_ESM.zip › Figure6/Fig6H/Experiment-19_GABA_OEskipped.tif_files/Experiment-19_z0c0x0-1388y0-1040.tif]

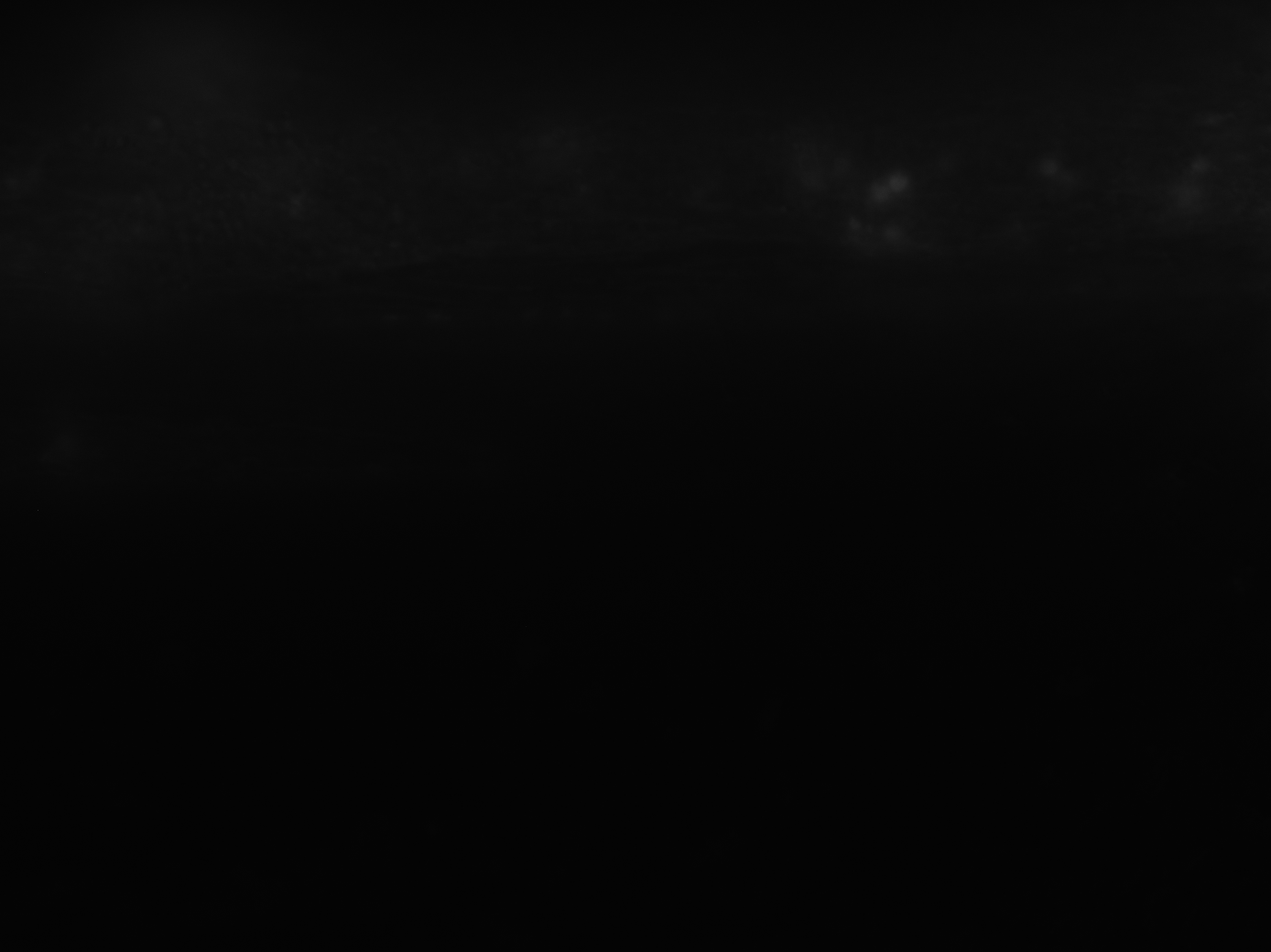

Supplement: Supplementary file 7 — Source data Fig. 6 [file 44319_2025_493_MOESM7_ESM.zip › Figure6/Fig6H/Experiment-19_GABA_OEskipped.tif_files/Experiment-19_z1c0x0-1388y0-1040.tif]

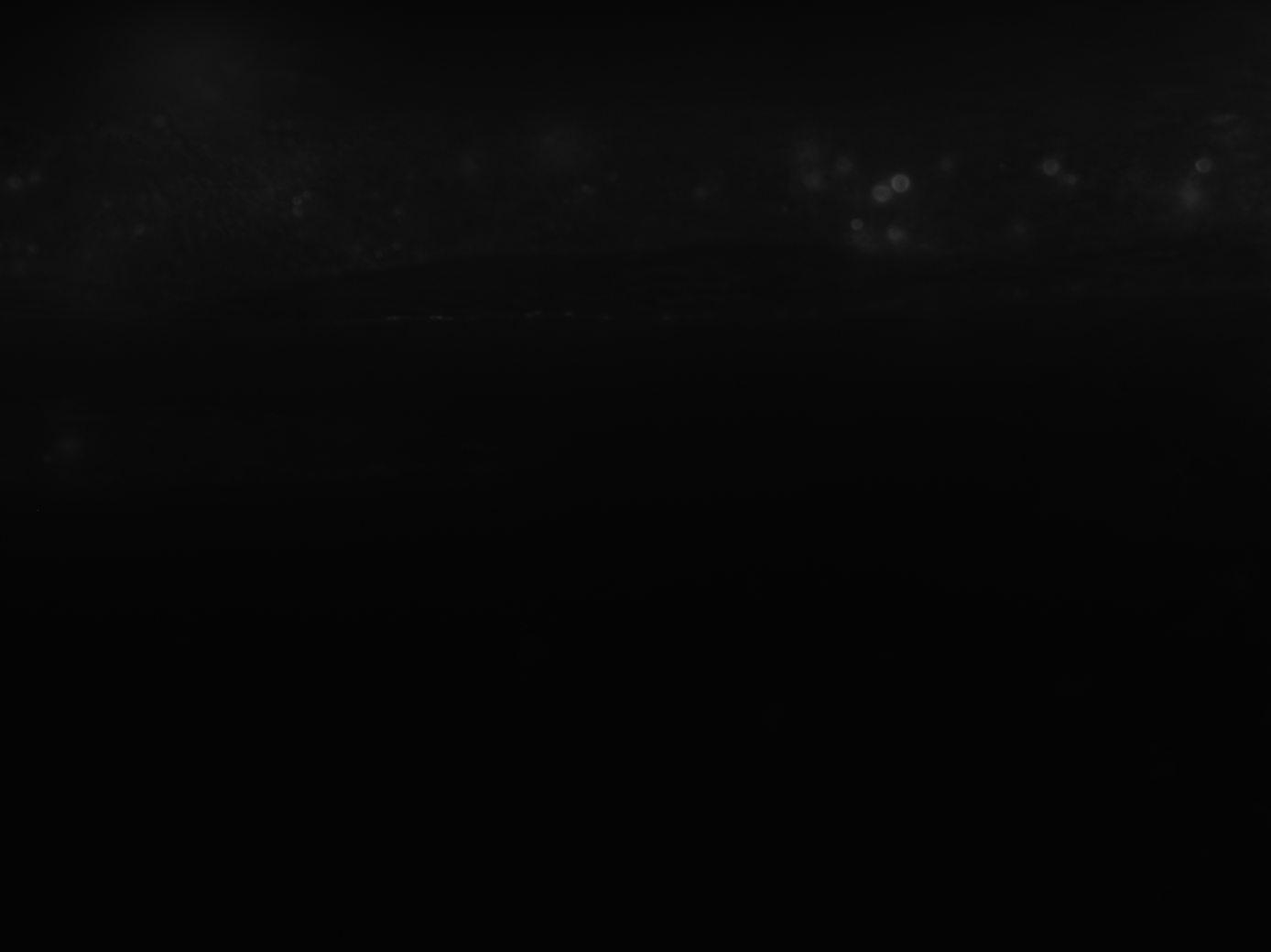

Supplement: Supplementary file 7 — Source data Fig. 6 [file 44319_2025_493_MOESM7_ESM.zip › Figure6/Fig6H/Experiment-19_GABA_OEskipped.tif_files/Experiment-19_z2c0x0-1388y0-1040.tif]

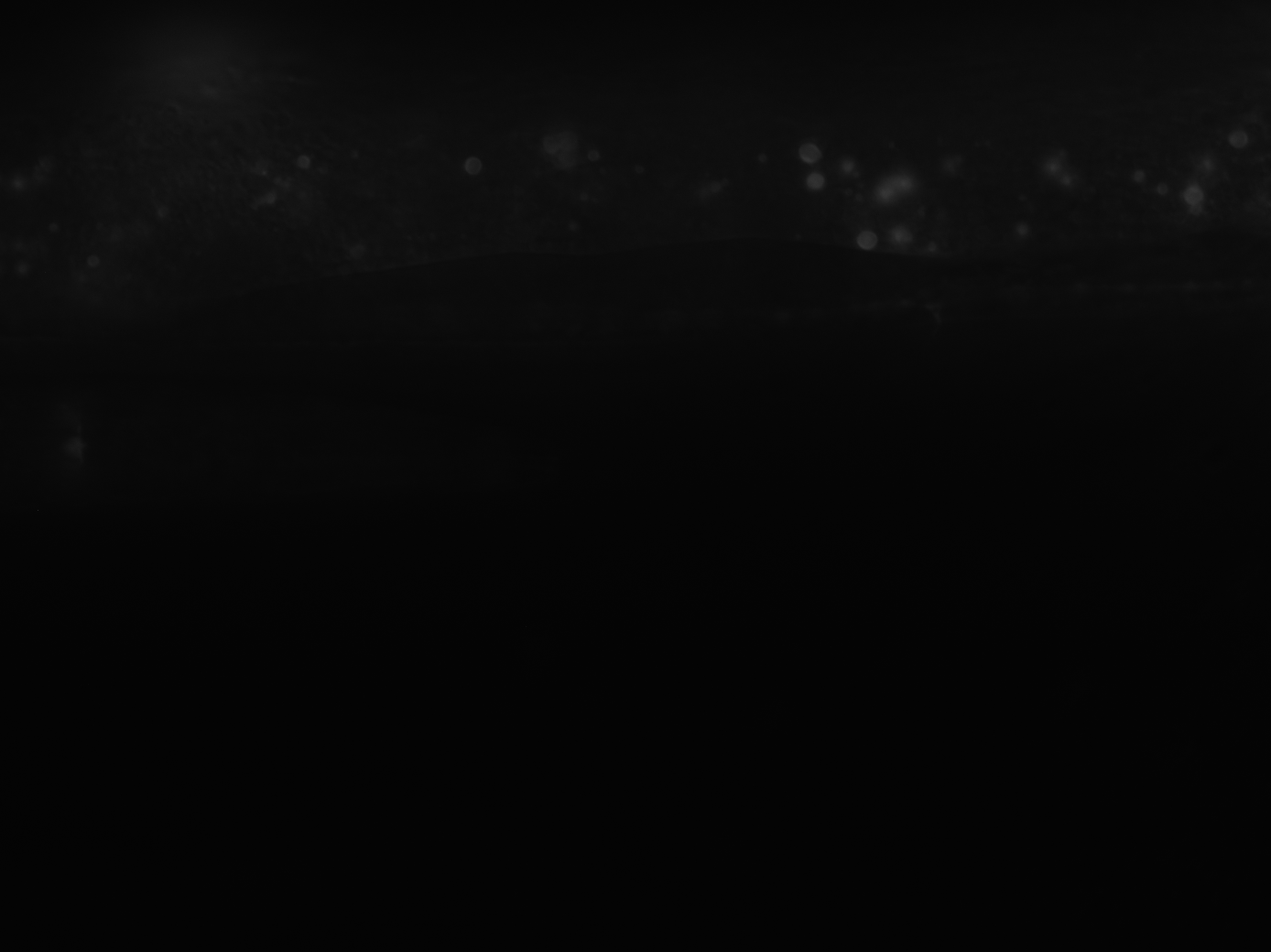

Supplement: Supplementary file 7 — Source data Fig. 6 [file 44319_2025_493_MOESM7_ESM.zip › Figure6/Fig6H/Experiment-19_GABA_OEskipped.tif_files/Experiment-19_z5c0x0-1388y0-1040.tif]

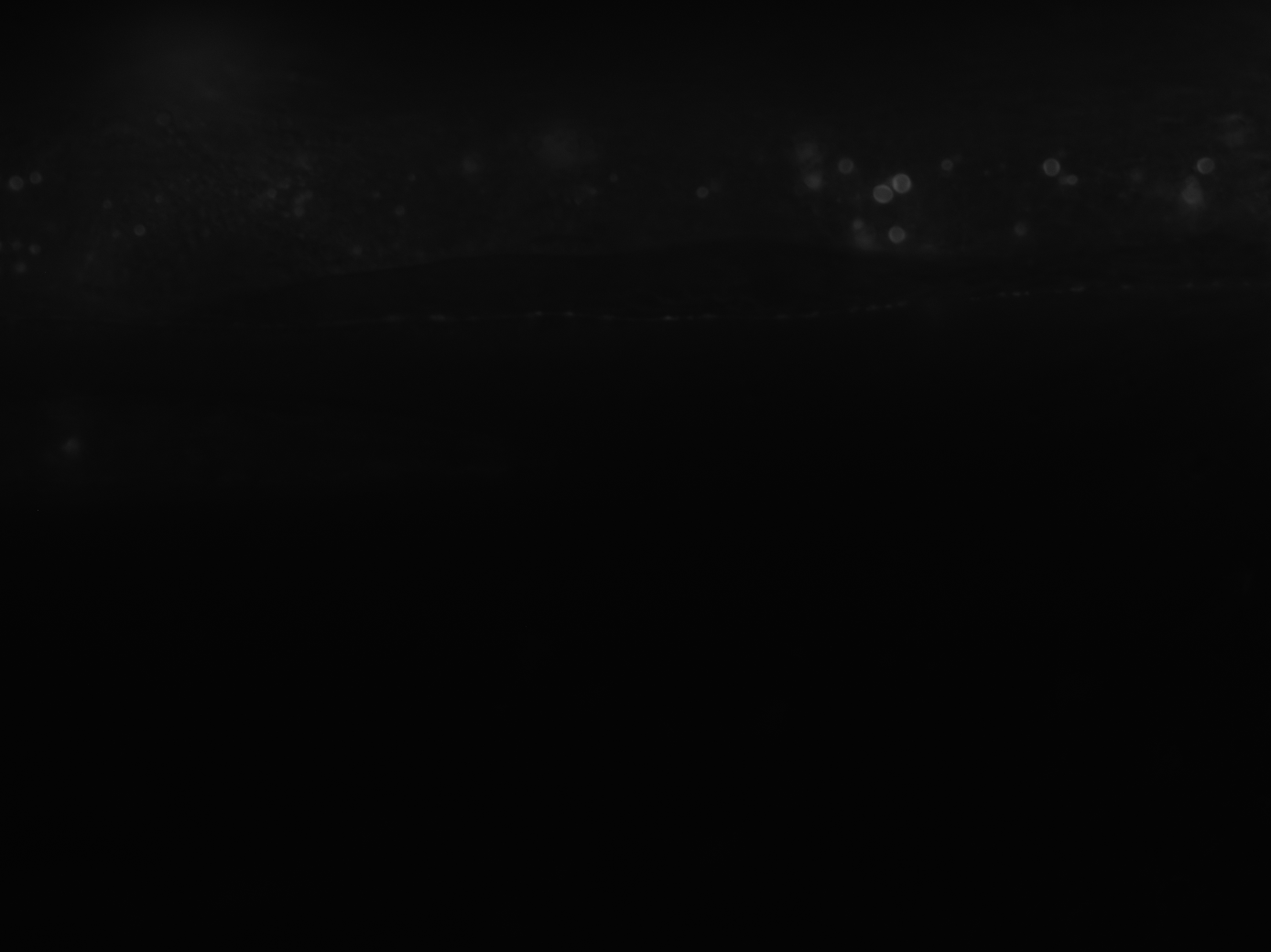

Supplement: Supplementary file 7 — Source data Fig. 6 [file 44319_2025_493_MOESM7_ESM.zip › Figure6/Fig6H/Experiment-19_GABA_OEskipped.tif_files/Experiment-19_z3c0x0-1388y0-1040.tif]

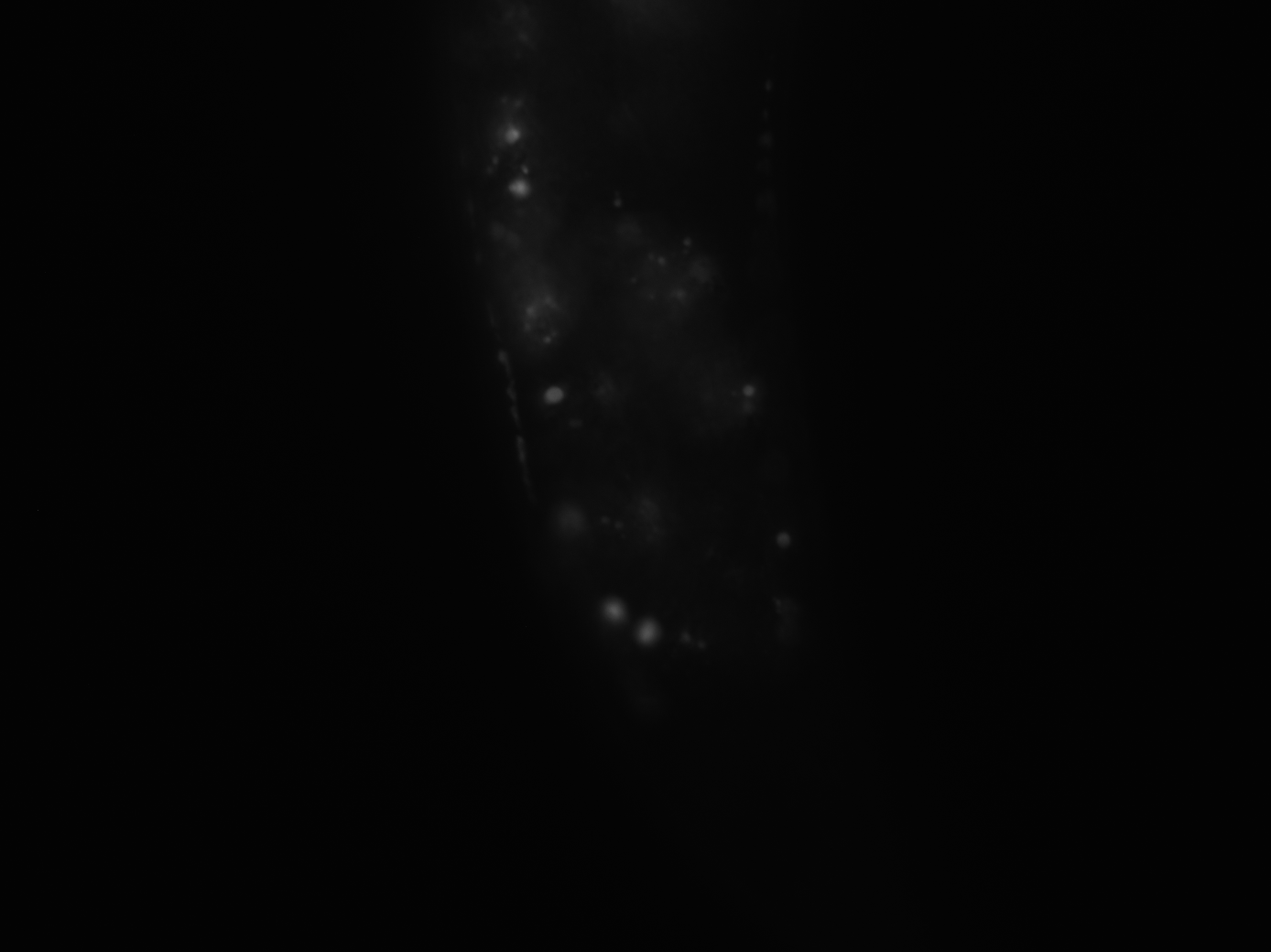

Supplement: Supplementary file 7 — Source data Fig. 6 [file 44319_2025_493_MOESM7_ESM.zip › Figure6/Fig6H/Experiment-07_GABA_s69.tif_files/Experiment-07_z7c0x0-1388y0-1040.tif]

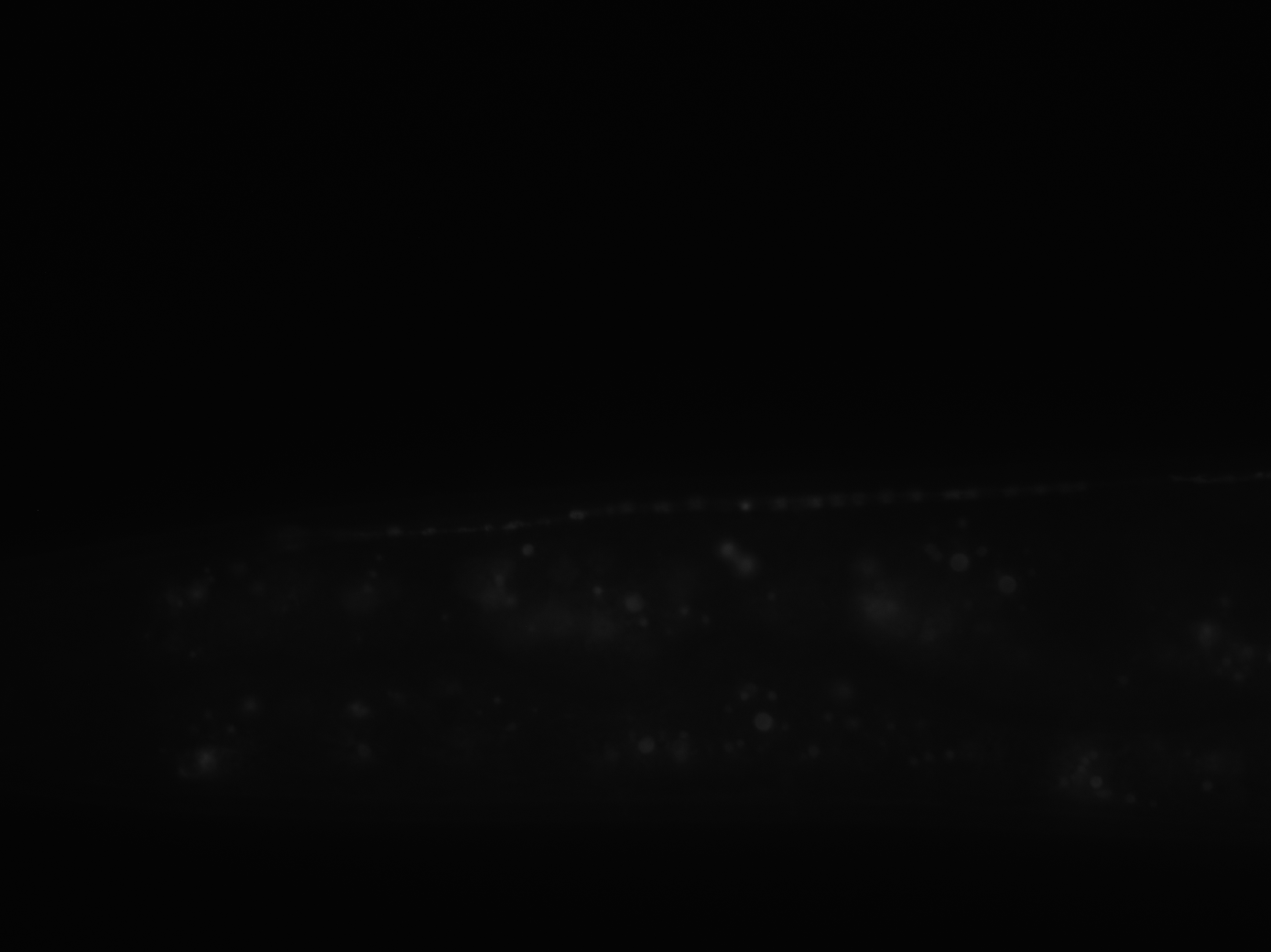

Supplement: Supplementary file 7 — Source data Fig. 6 [file 44319_2025_493_MOESM7_ESM.zip › Figure6/Fig6H/Experiment-03_GABA_wt.tif_files/Experiment-03_z4c0x0-1388y0-1040.tif]

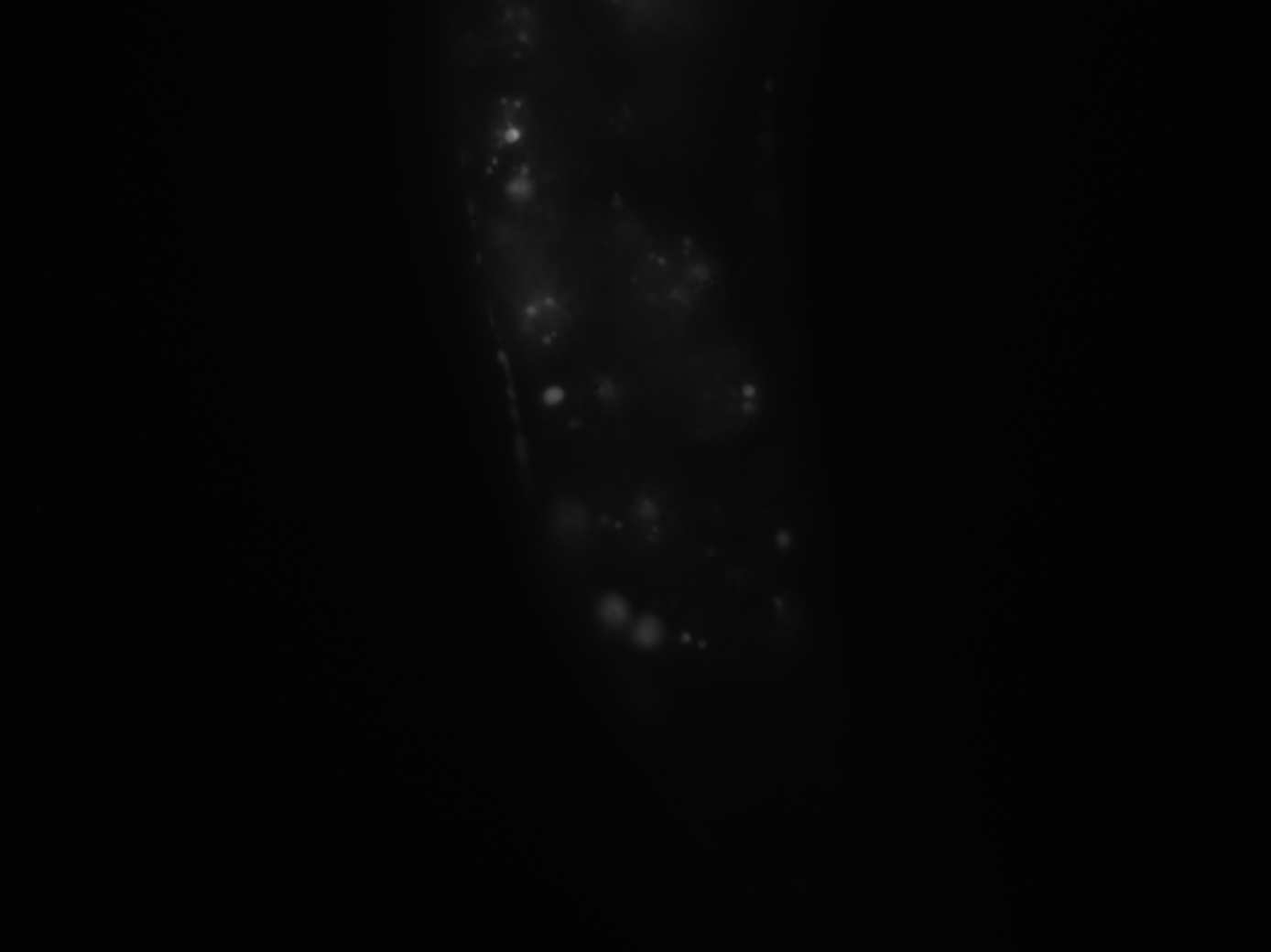

Supplement: Supplementary file 7 — Source data Fig. 6 [file 44319_2025_493_MOESM7_ESM.zip › Figure6/Fig6H/Experiment-07_GABA_s69.tif_files/Experiment-07_z8c0x0-1388y0-1040.tif]

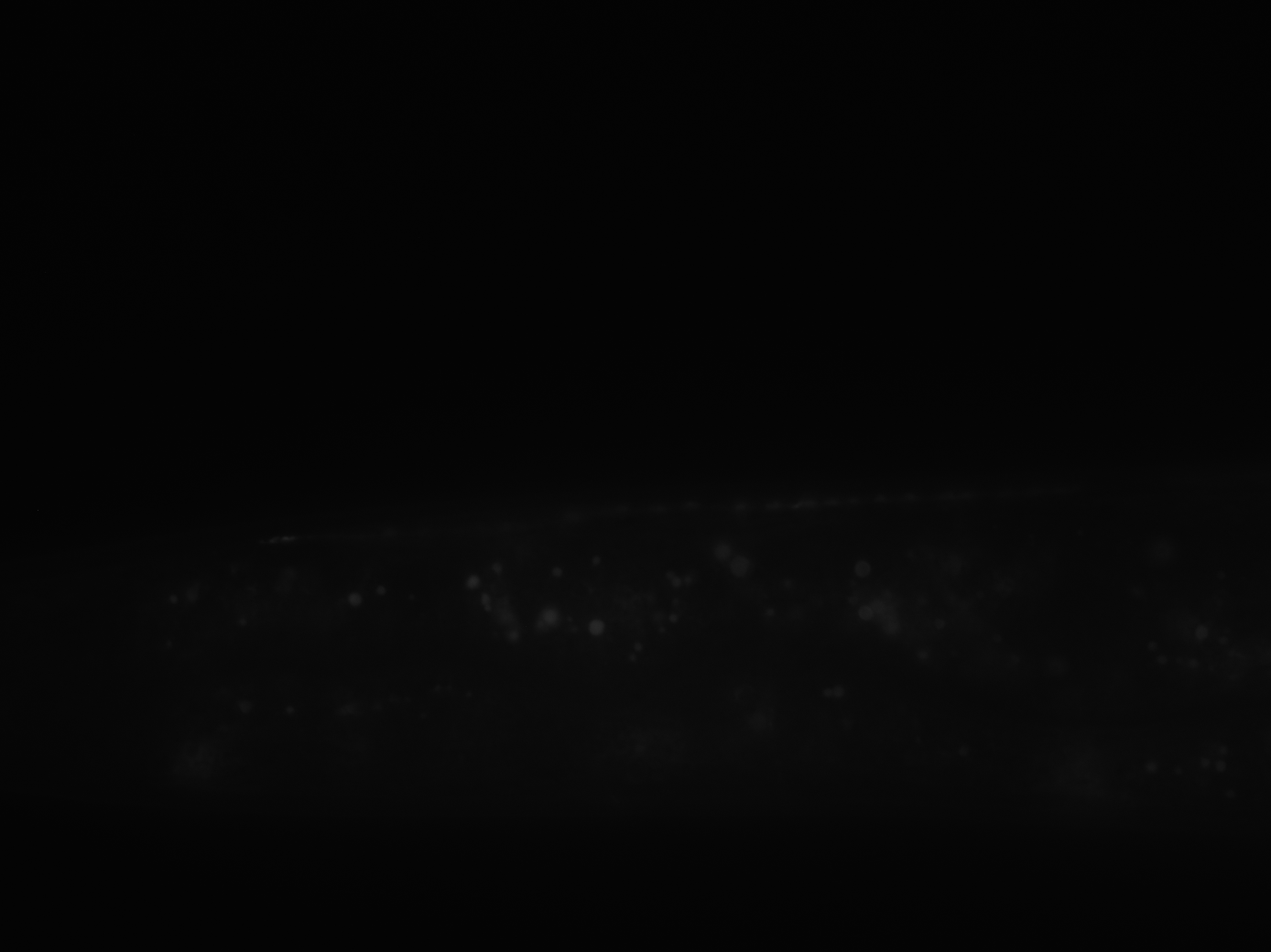

Supplement: Supplementary file 7 — Source data Fig. 6 [file 44319_2025_493_MOESM7_ESM.zip › Figure6/Fig6H/Experiment-03_GABA_wt.tif_files/Experiment-03_z1c0x0-1388y0-1040.tif]

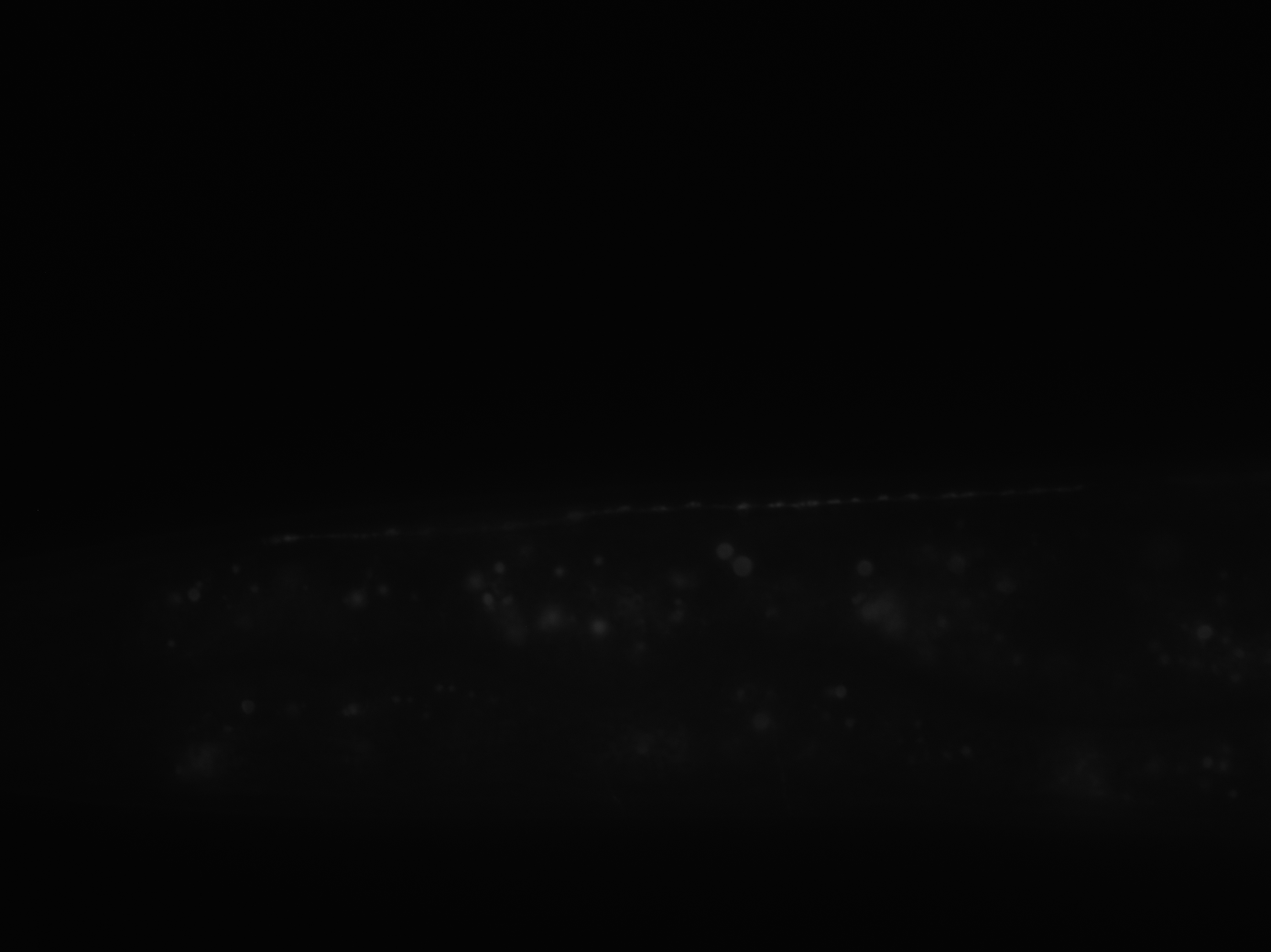

Supplement: Supplementary file 7 — Source data Fig. 6 [file 44319_2025_493_MOESM7_ESM.zip › Figure6/Fig6H/Experiment-03_GABA_wt.tif_files/Experiment-03_z2c0x0-1388y0-1040.tif]

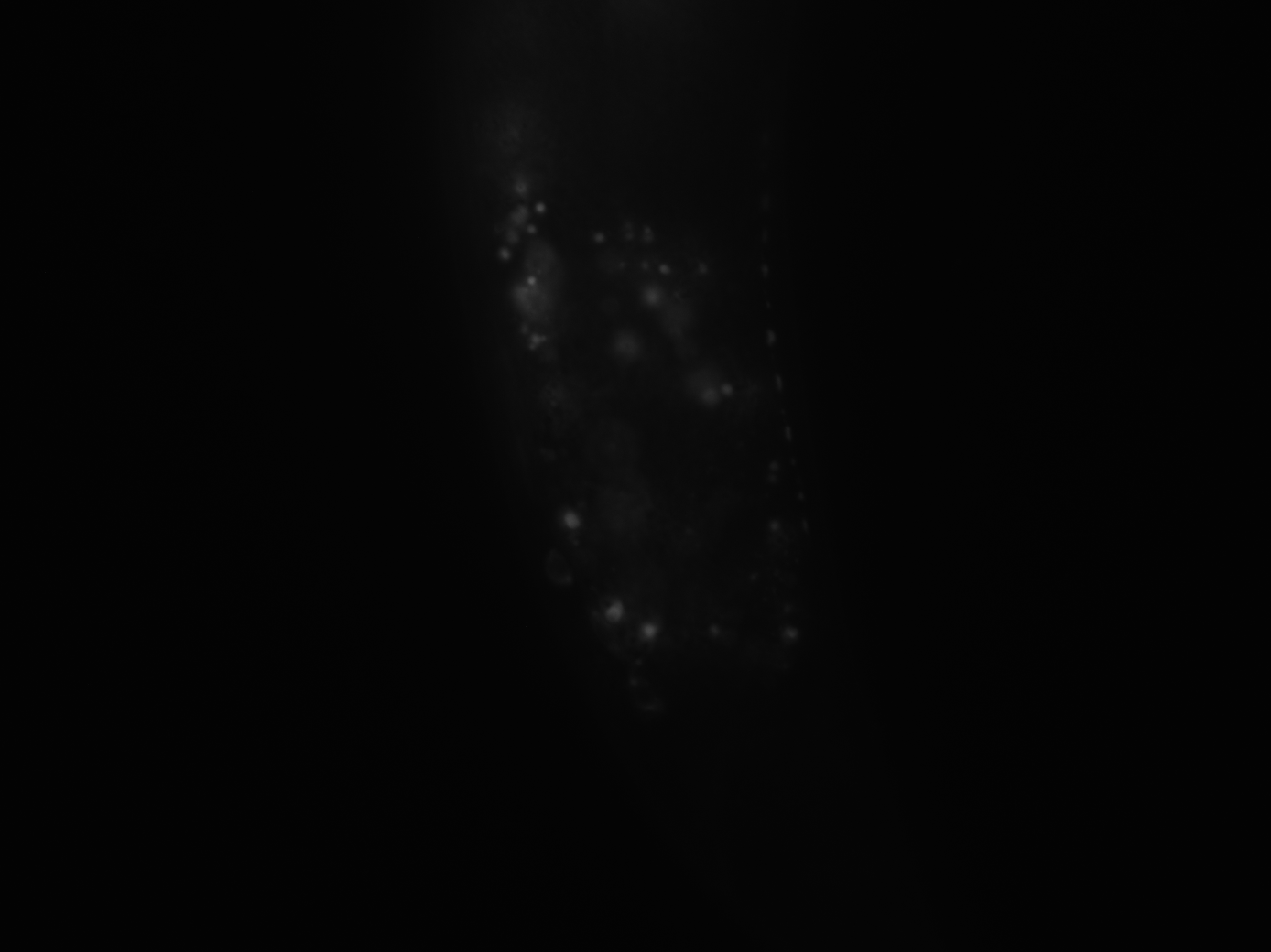

Supplement: Supplementary file 7 — Source data Fig. 6 [file 44319_2025_493_MOESM7_ESM.zip › Figure6/Fig6H/Experiment-07_GABA_s69.tif_files/Experiment-07_z3c0x0-1388y0-1040.tif]

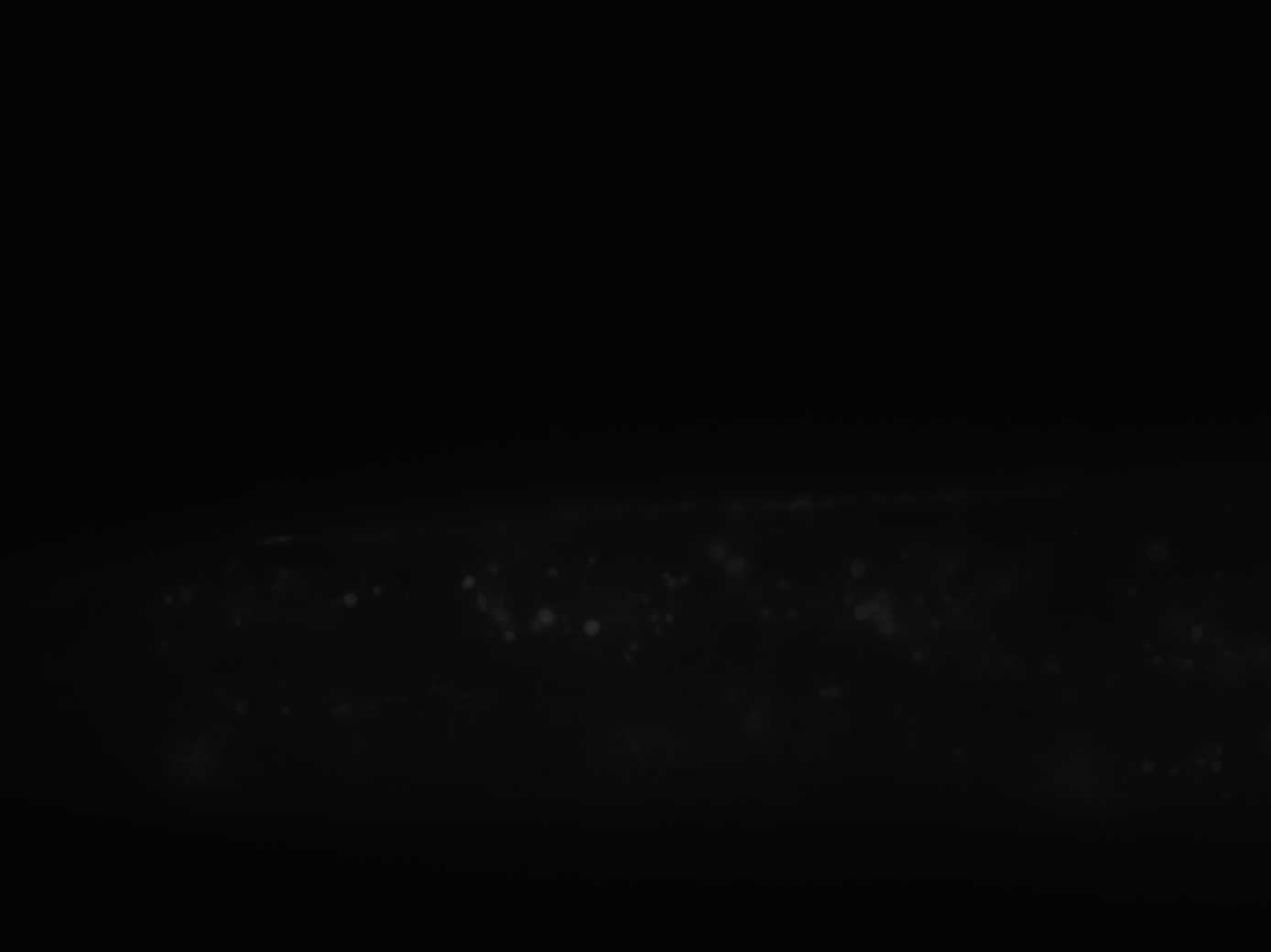

Supplement: Supplementary file 7 — Source data Fig. 6 [file 44319_2025_493_MOESM7_ESM.zip › Figure6/Fig6H/Experiment-03_GABA_wt.tif_files/Experiment-03_z0c0x0-1388y0-1040.tif]

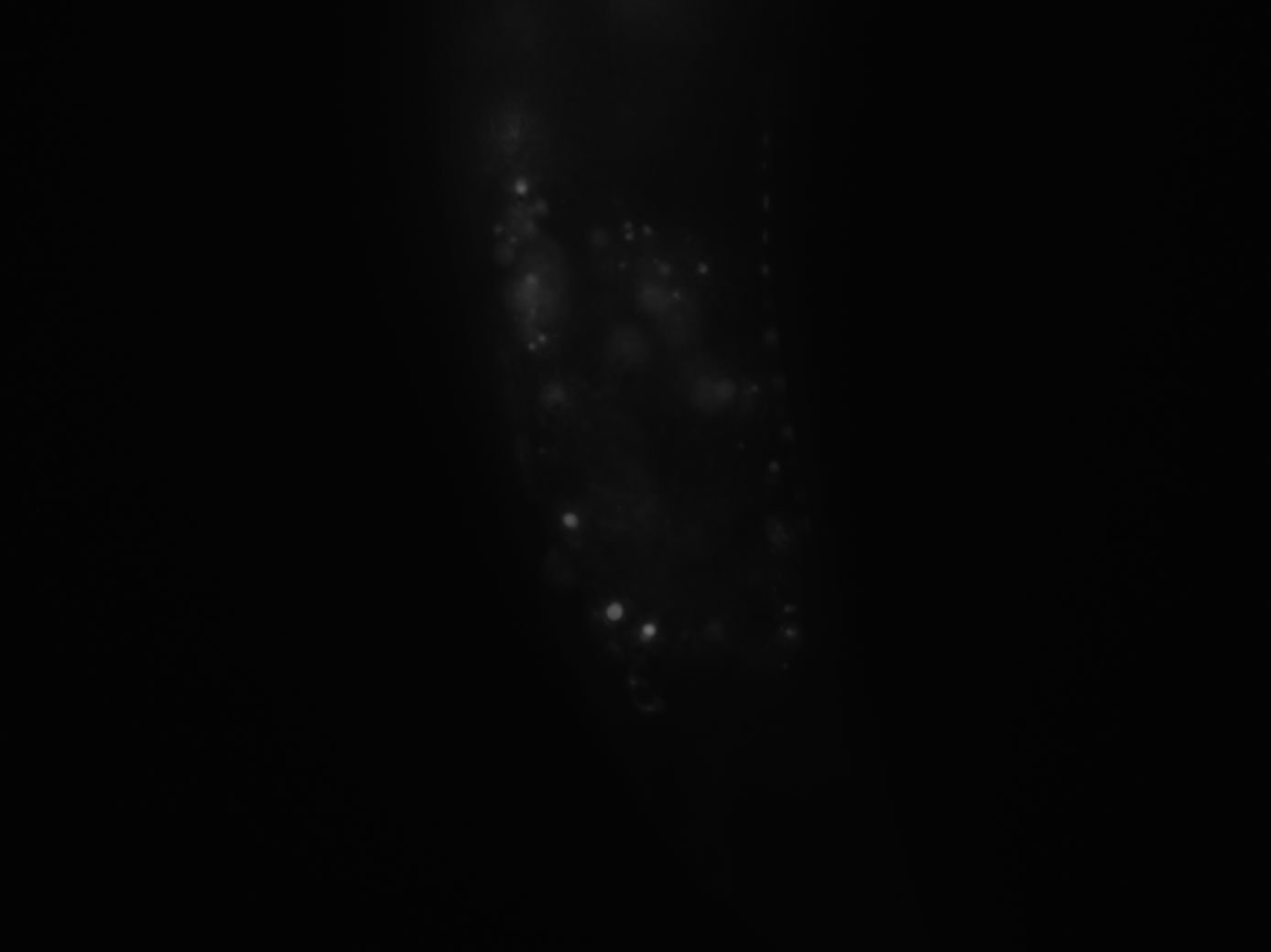

Supplement: Supplementary file 7 — Source data Fig. 6 [file 44319_2025_493_MOESM7_ESM.zip › Figure6/Fig6H/Experiment-07_GABA_s69.tif_files/Experiment-07_z4c0x0-1388y0-1040.tif]

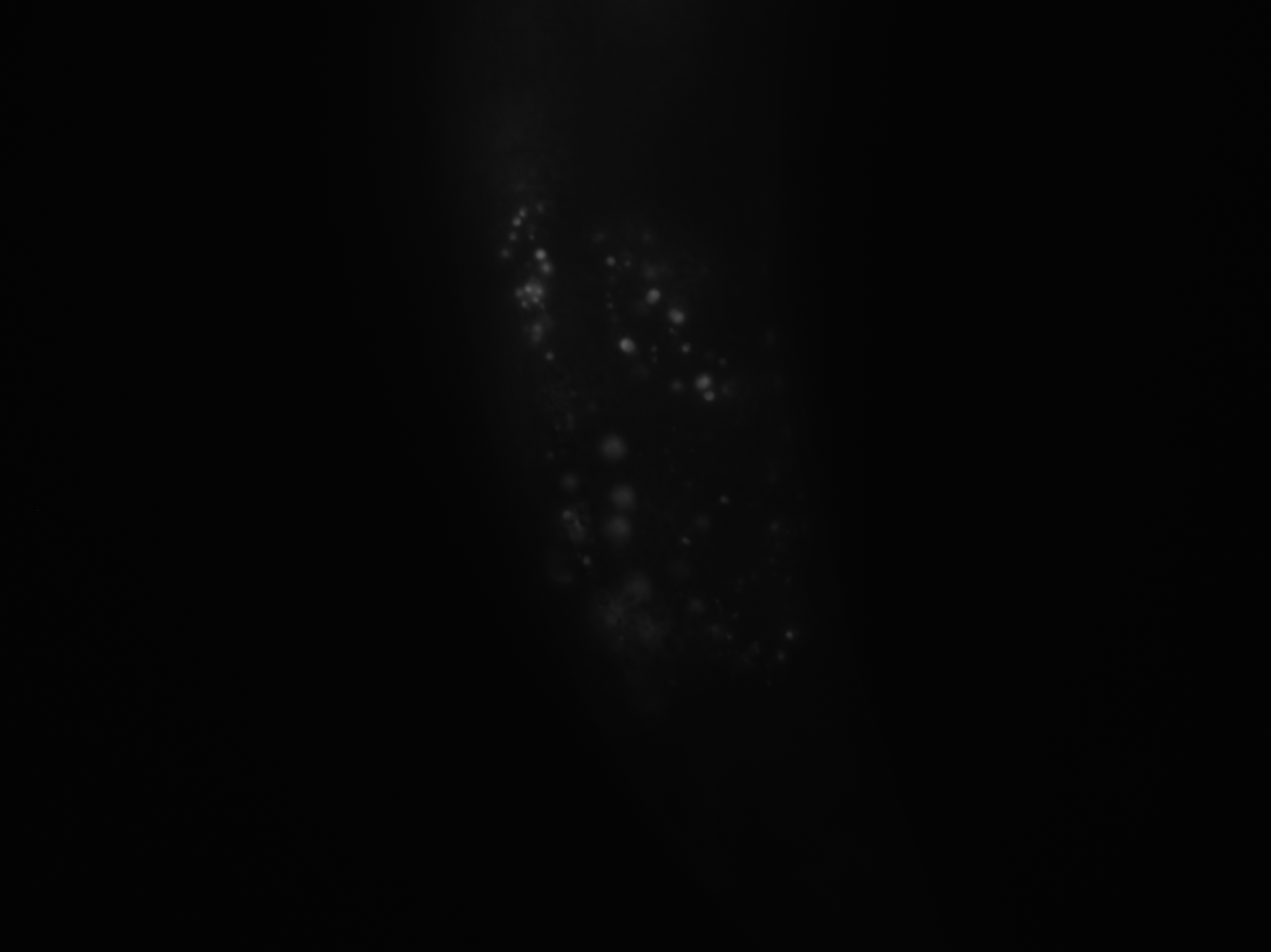

Supplement: Supplementary file 7 — Source data Fig. 6 [file 44319_2025_493_MOESM7_ESM.zip › Figure6/Fig6H/Experiment-07_GABA_s69.tif_files/Experiment-07_z0c0x0-1388y0-1040.tif]

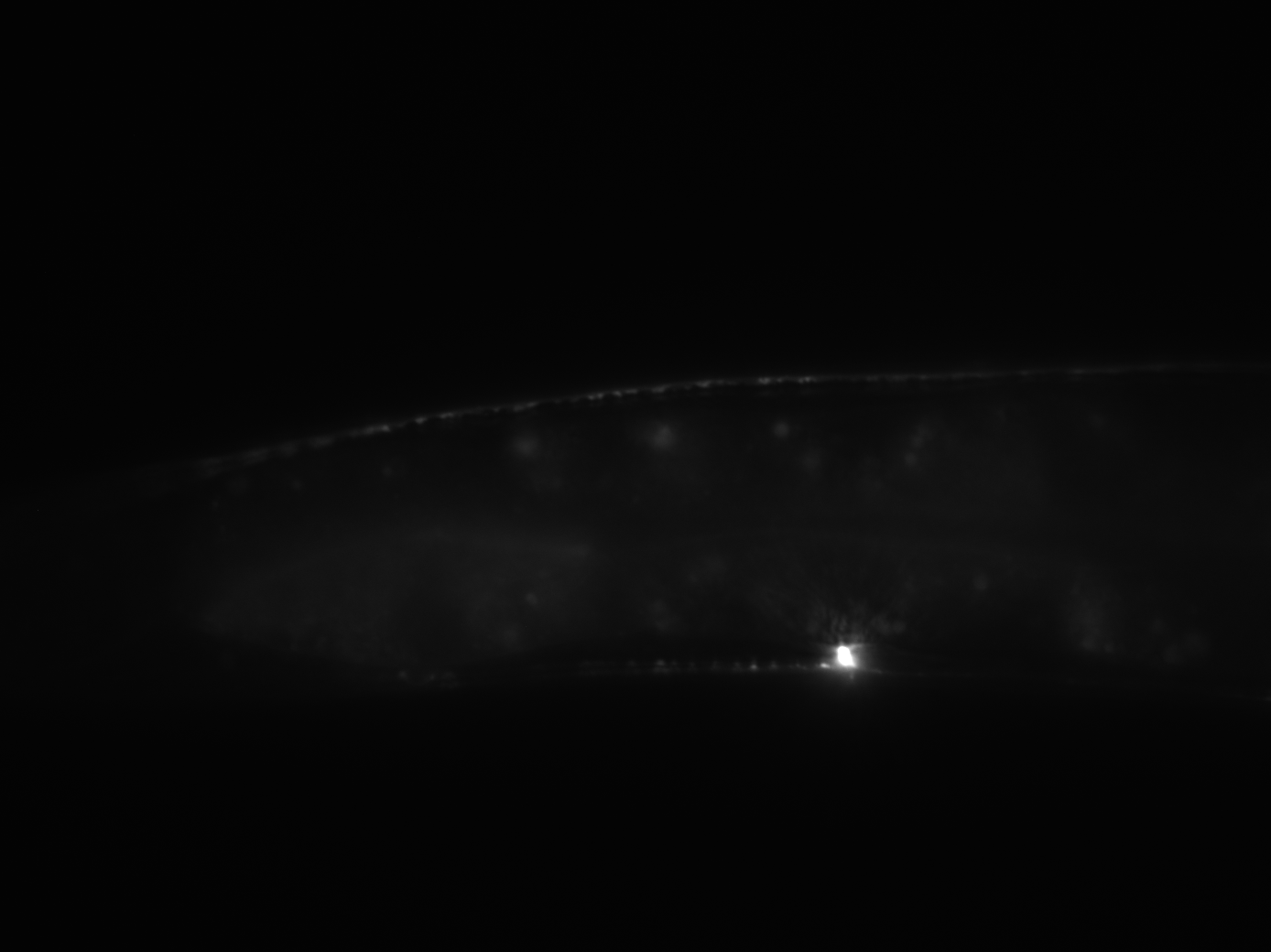

Supplement: Supplementary file 7 — Source data Fig. 6 [file 44319_2025_493_MOESM7_ESM.zip › Figure6/Fig6H/Experiment-69_wt_cholinergic.tif_files/Experiment-69_z7c0x0-1388y0-1040.tif]

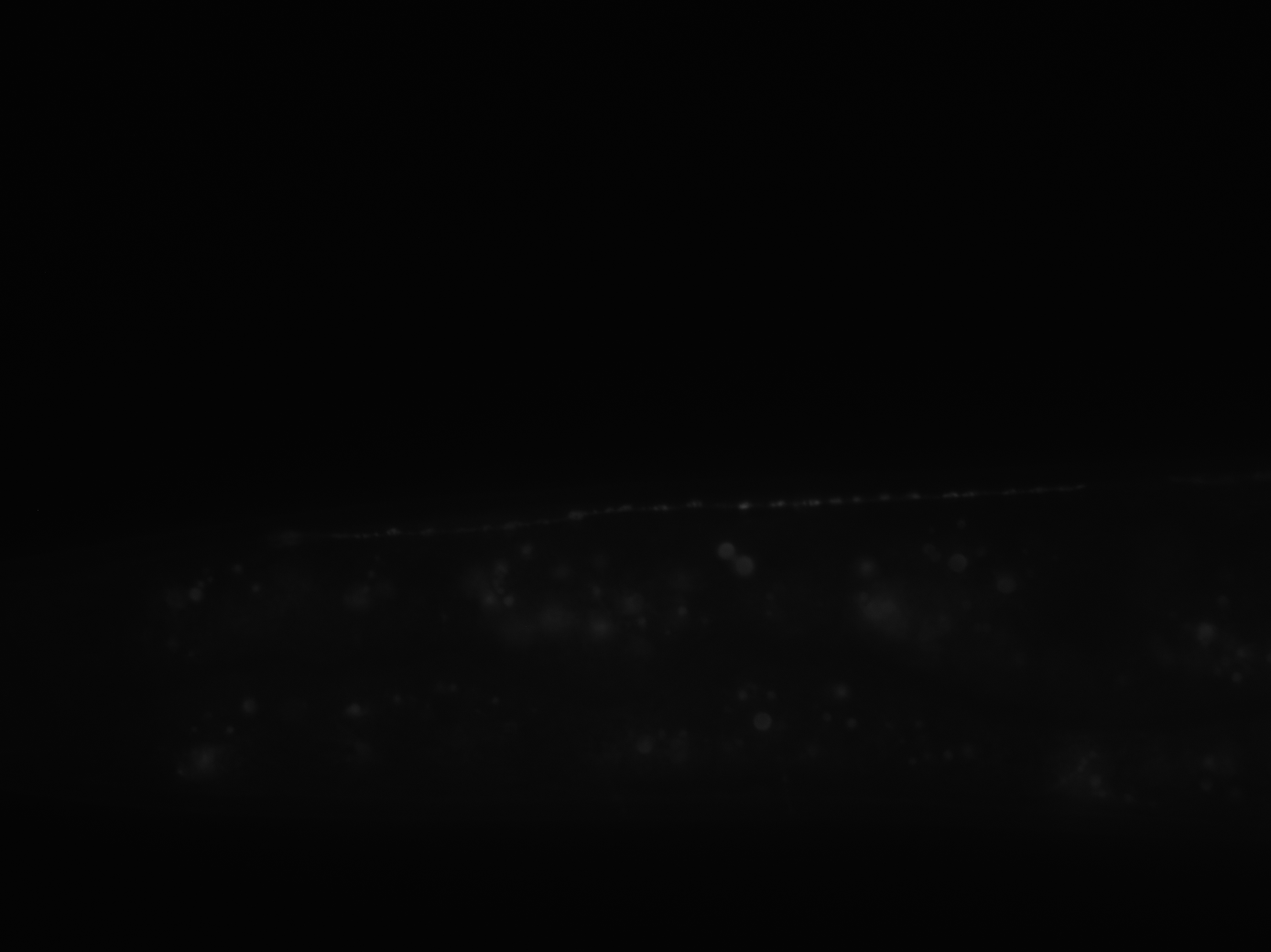

Supplement: Supplementary file 7 — Source data Fig. 6 [file 44319_2025_493_MOESM7_ESM.zip › Figure6/Fig6H/Experiment-03_GABA_wt.tif_files/Experiment-03_z3c0x0-1388y0-1040.tif]

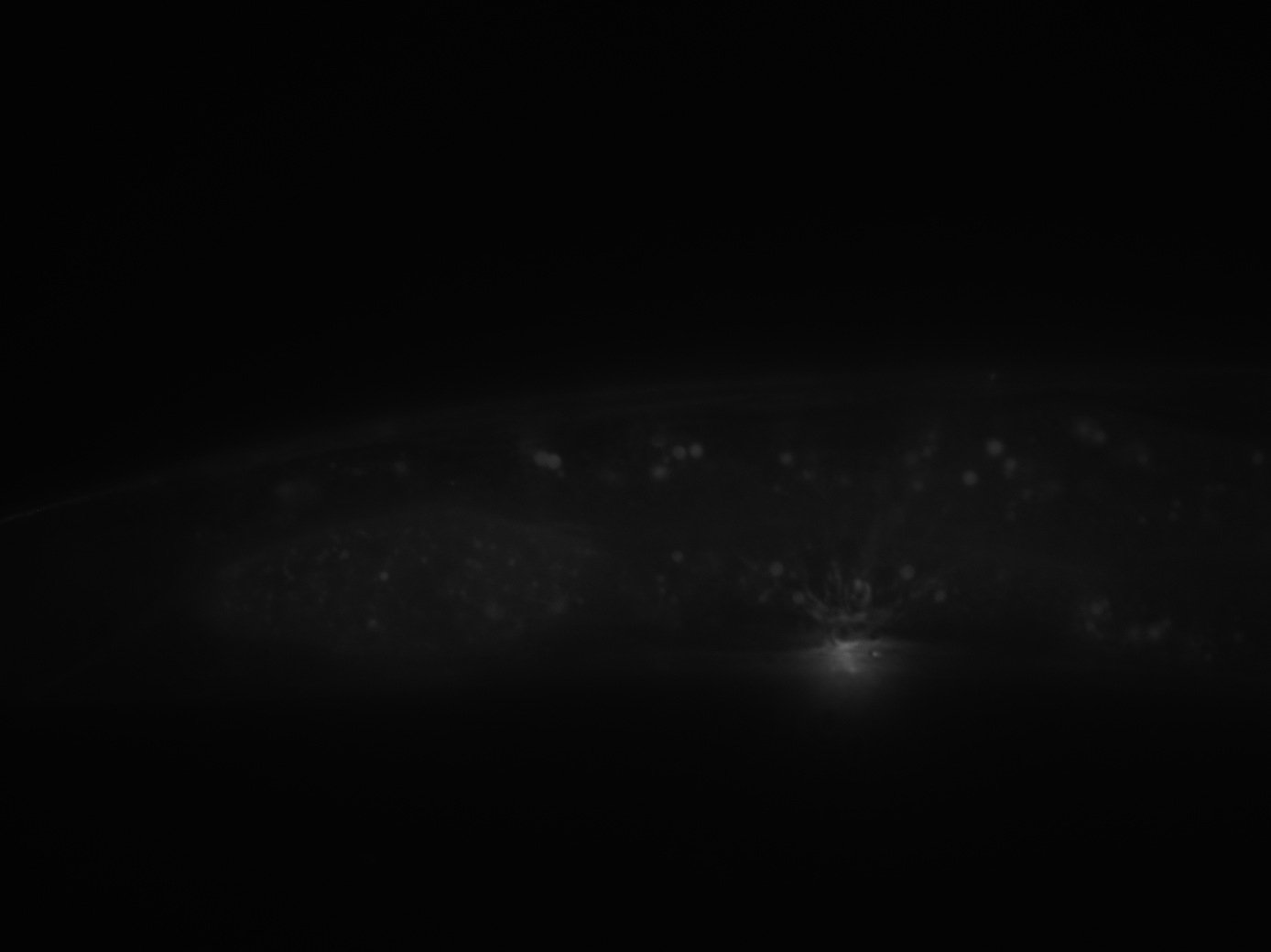

Supplement: Supplementary file 7 — Source data Fig. 6 [file 44319_2025_493_MOESM7_ESM.zip › Figure6/Fig6H/Experiment-69_wt_cholinergic.tif_files/Experiment-69_z1c0x0-1388y0-1040.tif]

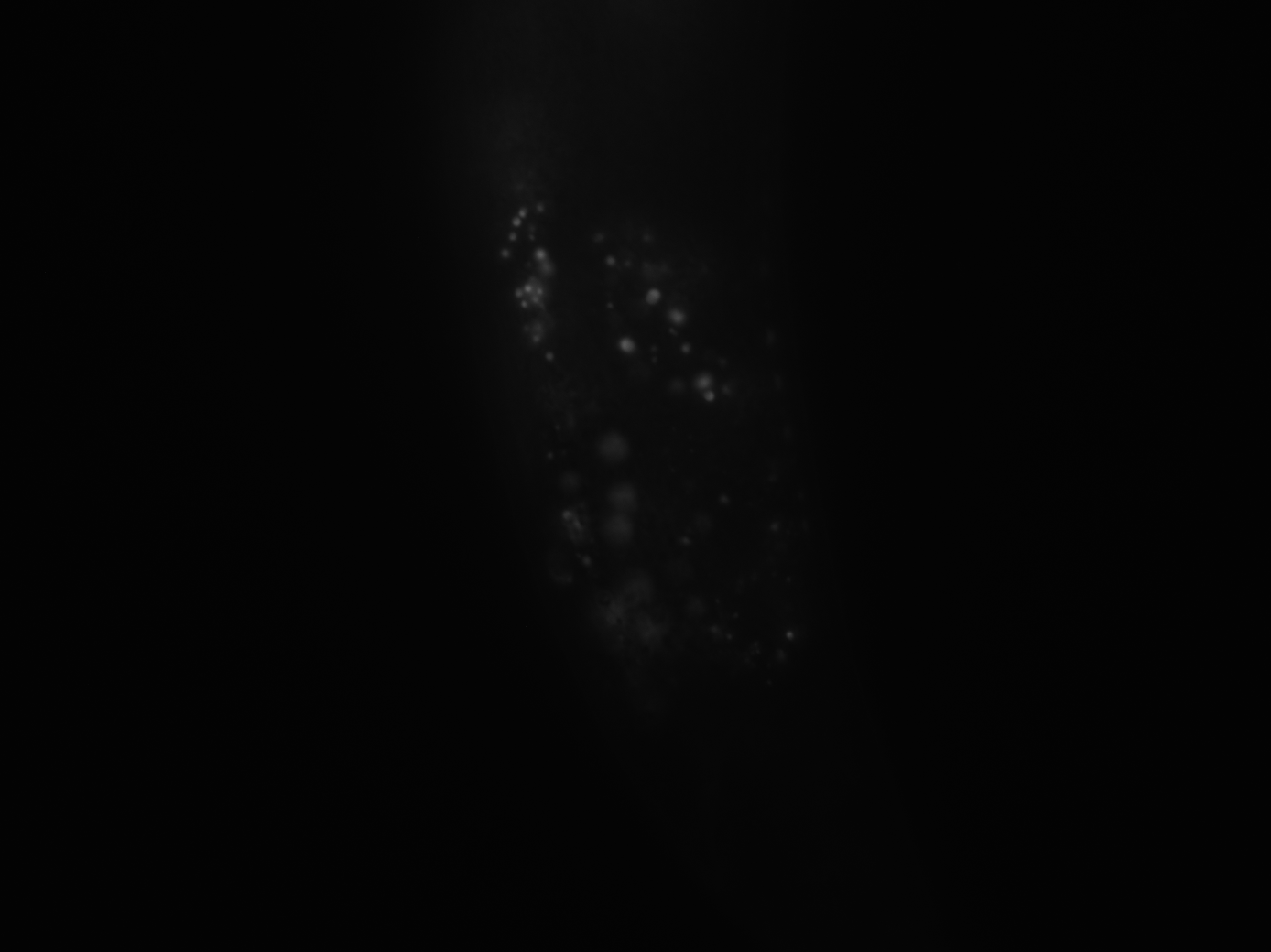

Supplement: Supplementary file 7 — Source data Fig. 6 [file 44319_2025_493_MOESM7_ESM.zip › Figure6/Fig6H/Experiment-07_GABA_s69.tif_files/Experiment-07_z1c0x0-1388y0-1040.tif]

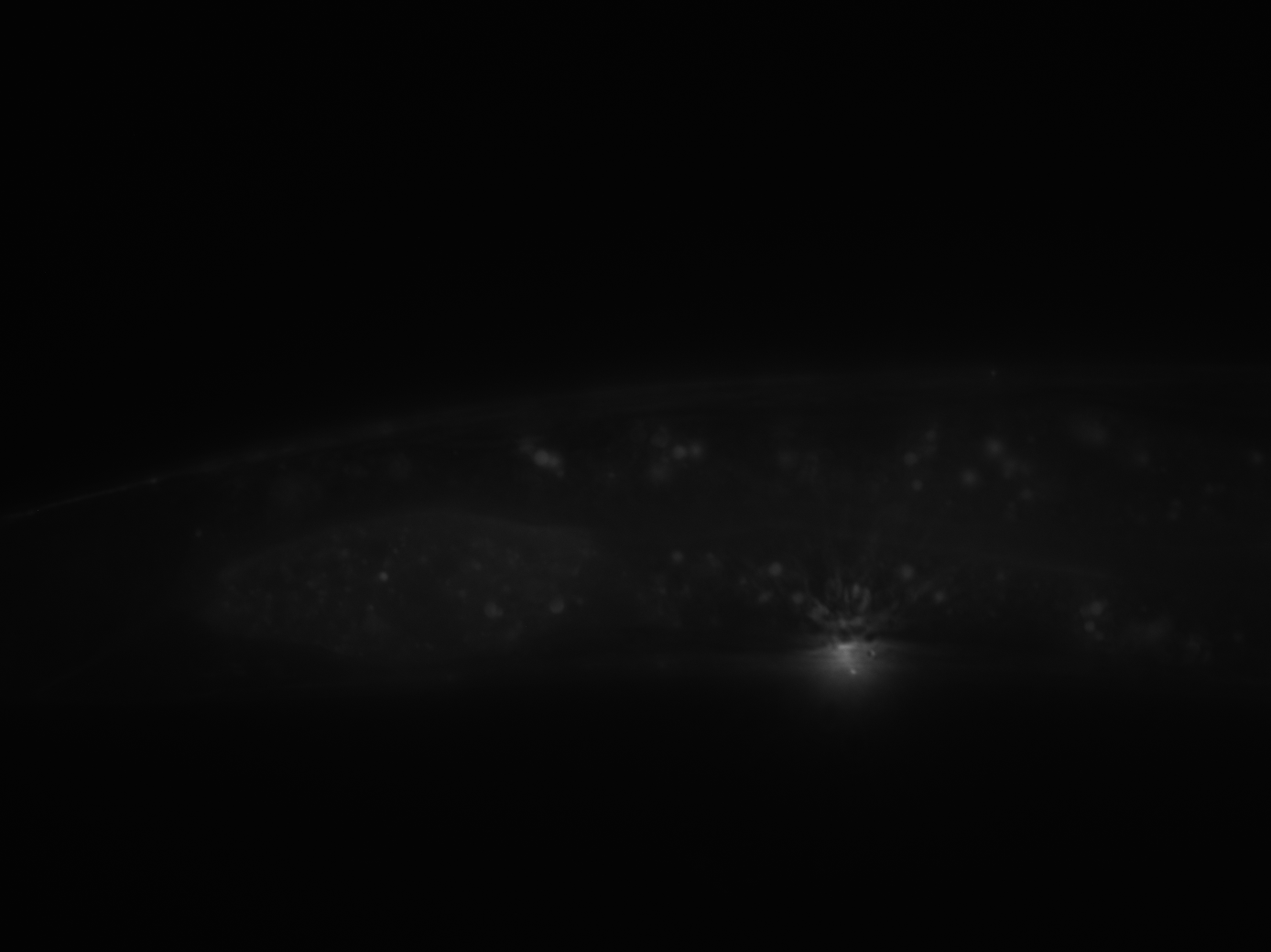

Supplement: Supplementary file 7 — Source data Fig. 6 [file 44319_2025_493_MOESM7_ESM.zip › Figure6/Fig6H/Experiment-69_wt_cholinergic.tif_files/Experiment-69_z2c0x0-1388y0-1040.tif]

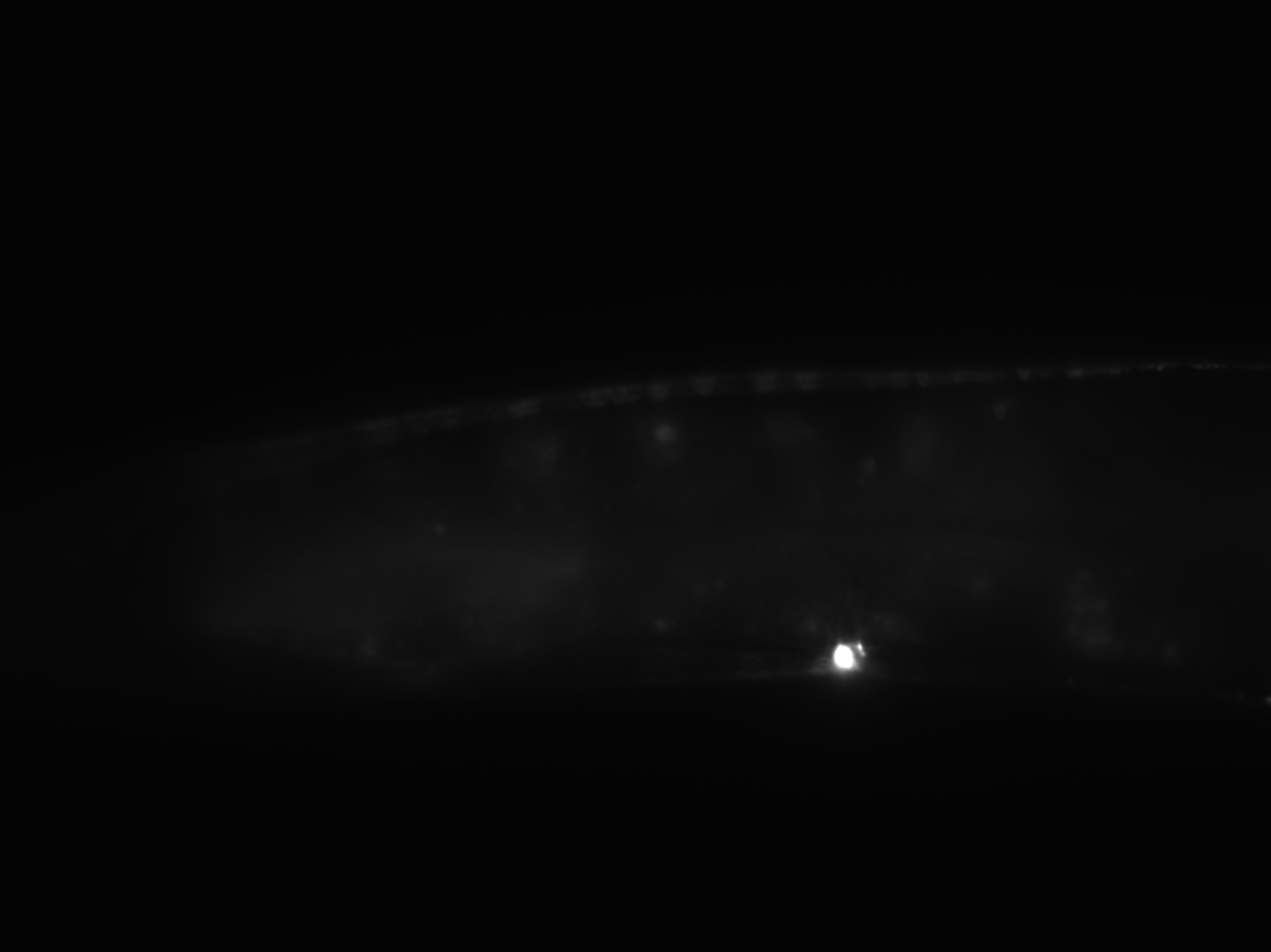

Supplement: Supplementary file 7 — Source data Fig. 6 [file 44319_2025_493_MOESM7_ESM.zip › Figure6/Fig6H/Experiment-69_wt_cholinergic.tif_files/Experiment-69_z10c0x0-1388y0-1040.tif]

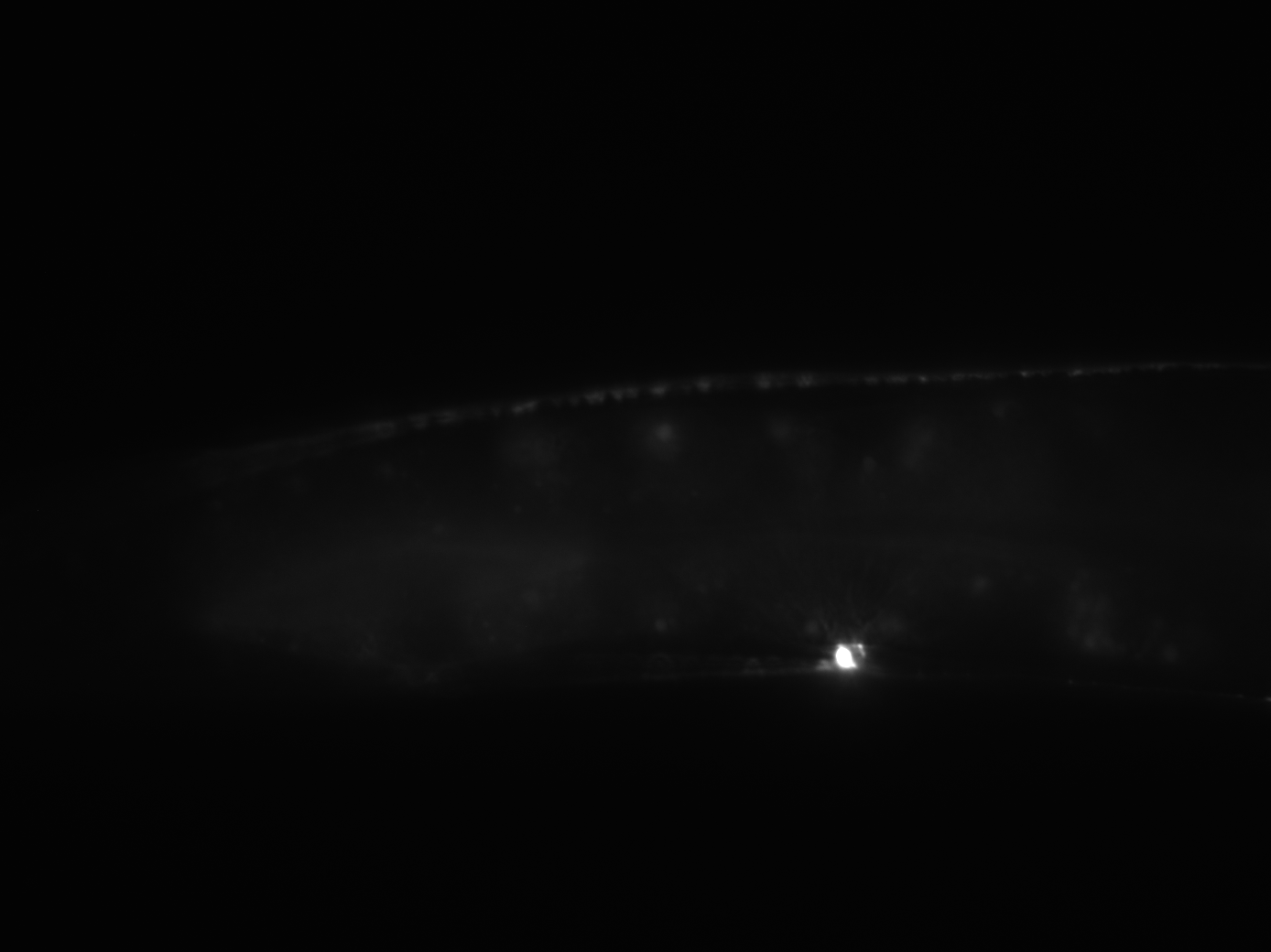

Supplement: Supplementary file 7 — Source data Fig. 6 [file 44319_2025_493_MOESM7_ESM.zip › Figure6/Fig6H/Experiment-69_wt_cholinergic.tif_files/Experiment-69_z9c0x0-1388y0-1040.tif]

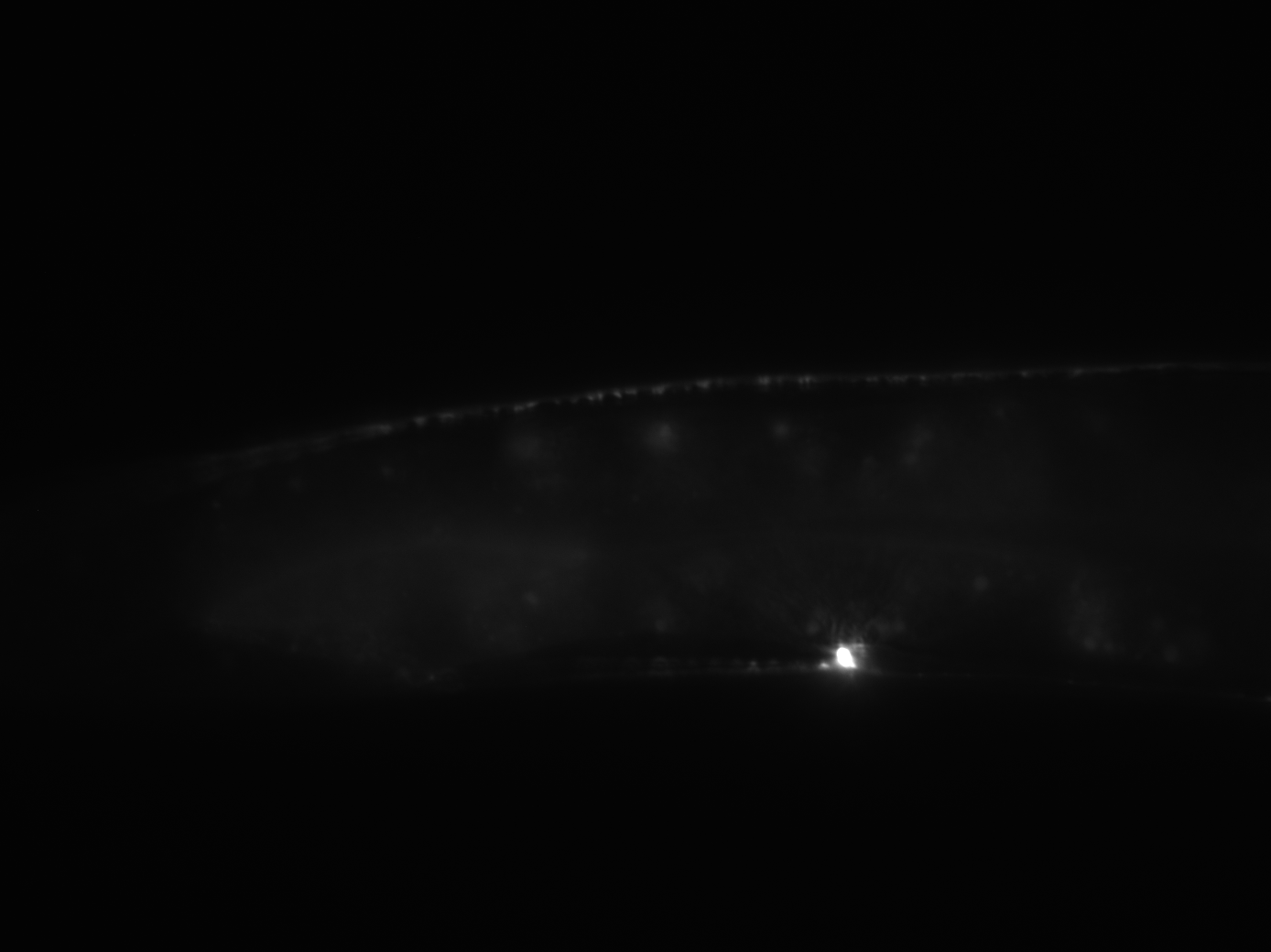

Supplement: Supplementary file 7 — Source data Fig. 6 [file 44319_2025_493_MOESM7_ESM.zip › Figure6/Fig6H/Experiment-69_wt_cholinergic.tif_files/Experiment-69_z8c0x0-1388y0-1040.tif]

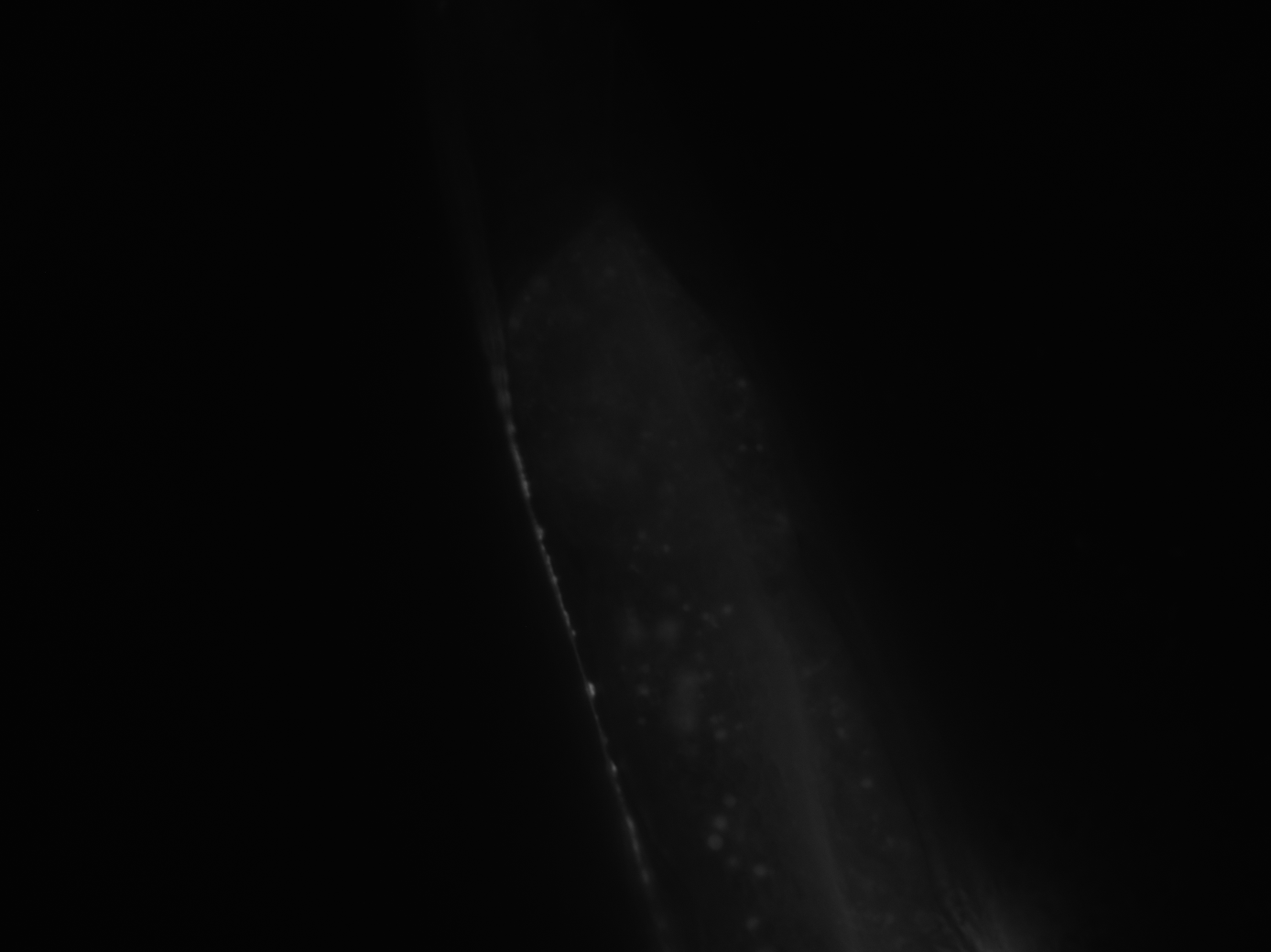

Supplement: Supplementary file 7 — Source data Fig. 6 [file 44319_2025_493_MOESM7_ESM.zip › Figure6/Fig6H/Experiment-74_OEskipped_cholinergic.tif_files/Experiment-74_z8c0x0-1388y0-1040.tif]

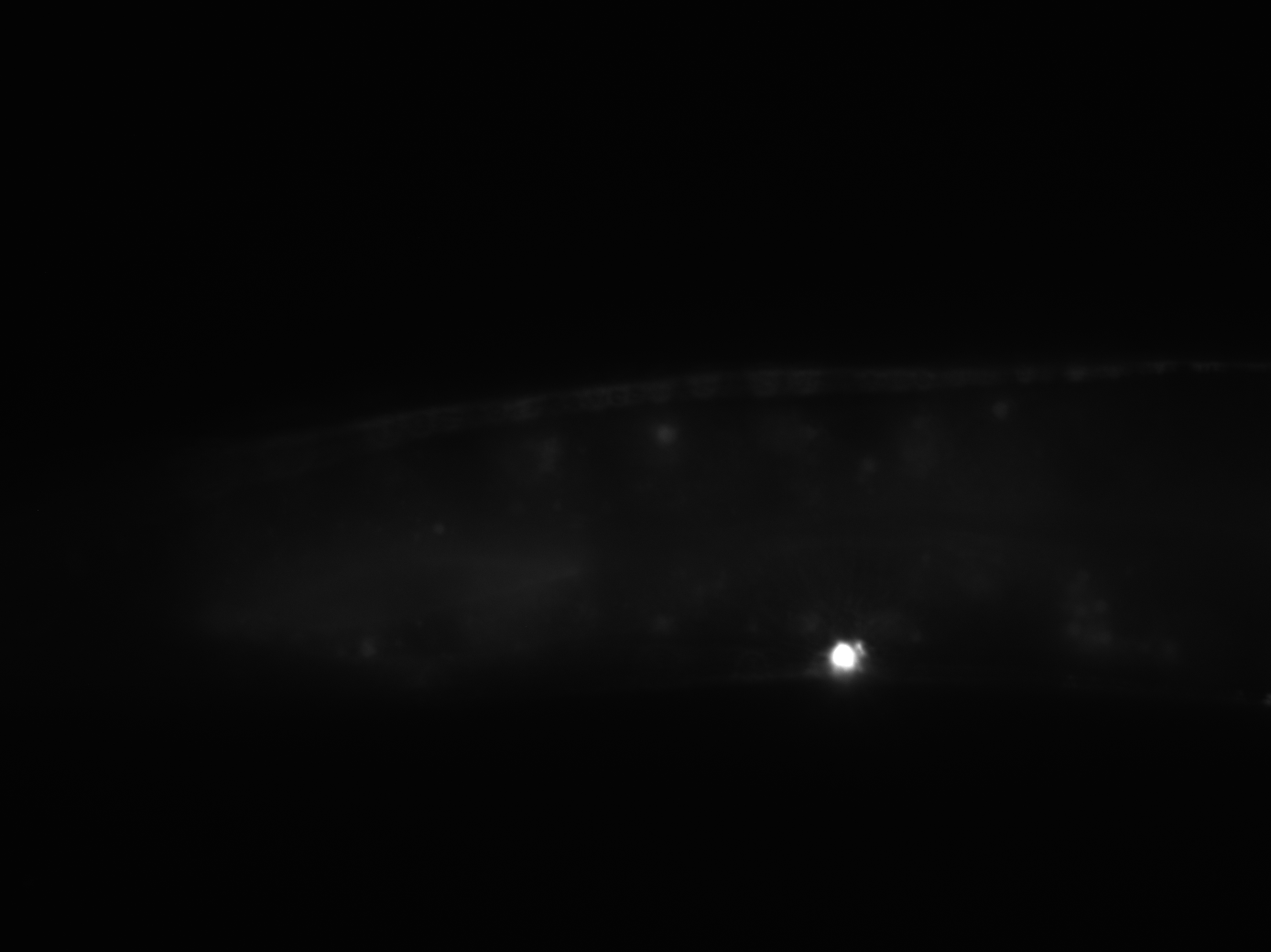

Supplement: Supplementary file 7 — Source data Fig. 6 [file 44319_2025_493_MOESM7_ESM.zip › Figure6/Fig6H/Experiment-69_wt_cholinergic.tif_files/Experiment-69_z11c0x0-1388y0-1040.tif]

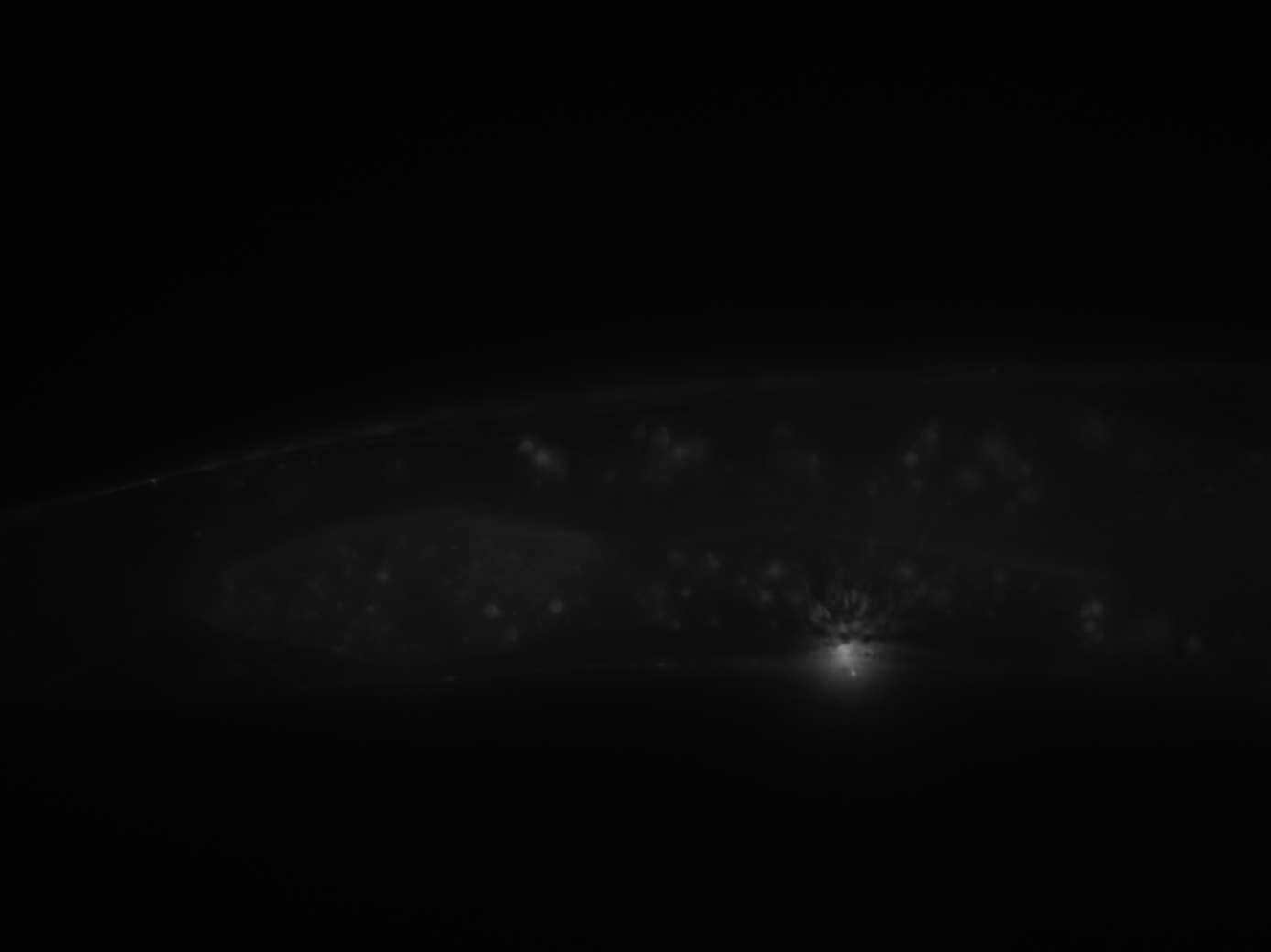

Supplement: Supplementary file 7 — Source data Fig. 6 [file 44319_2025_493_MOESM7_ESM.zip › Figure6/Fig6H/Experiment-69_wt_cholinergic.tif_files/Experiment-69_z3c0x0-1388y0-1040.tif]

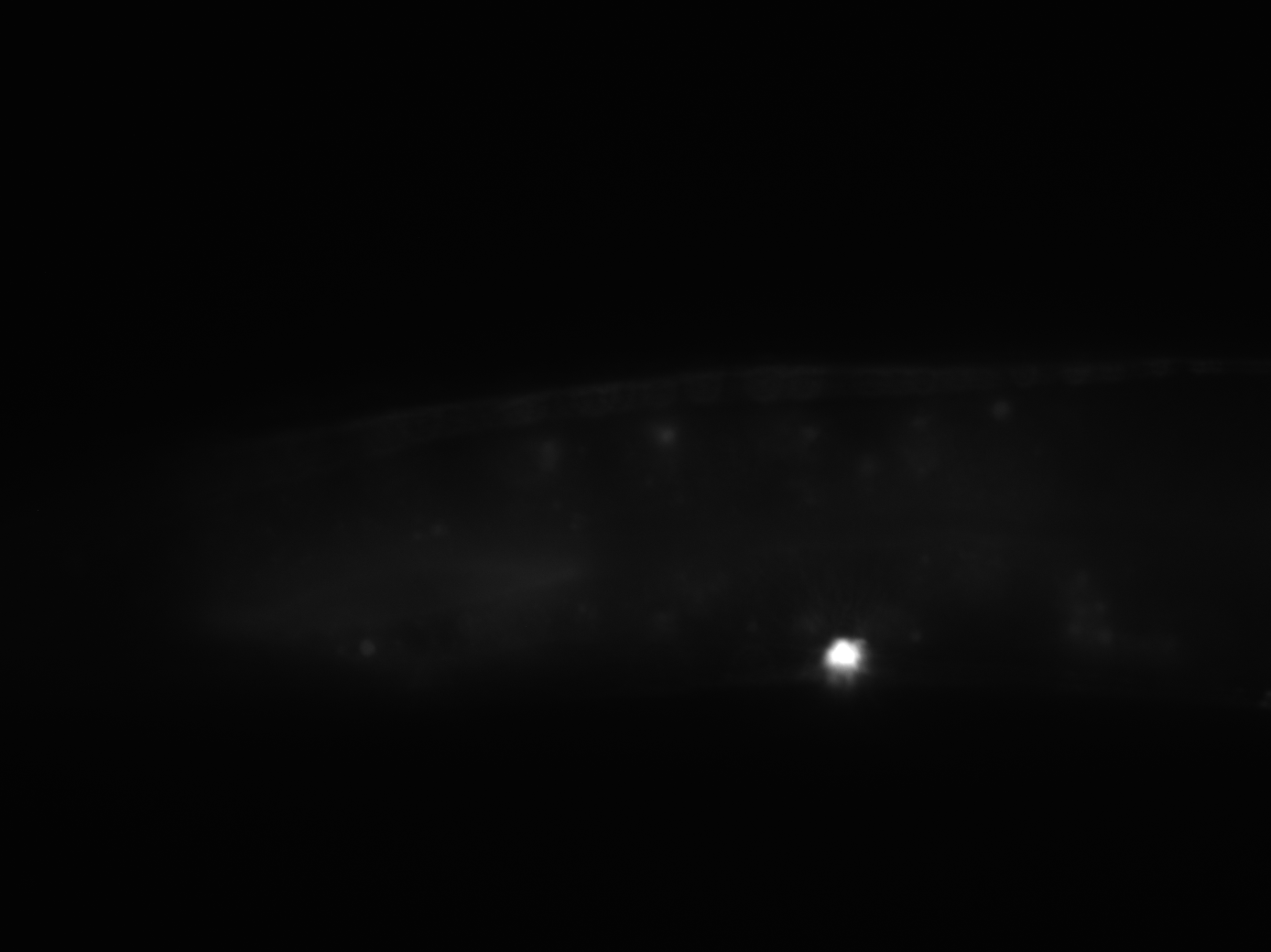

Supplement: Supplementary file 7 — Source data Fig. 6 [file 44319_2025_493_MOESM7_ESM.zip › Figure6/Fig6H/Experiment-69_wt_cholinergic.tif_files/Experiment-69_z12c0x0-1388y0-1040.tif]

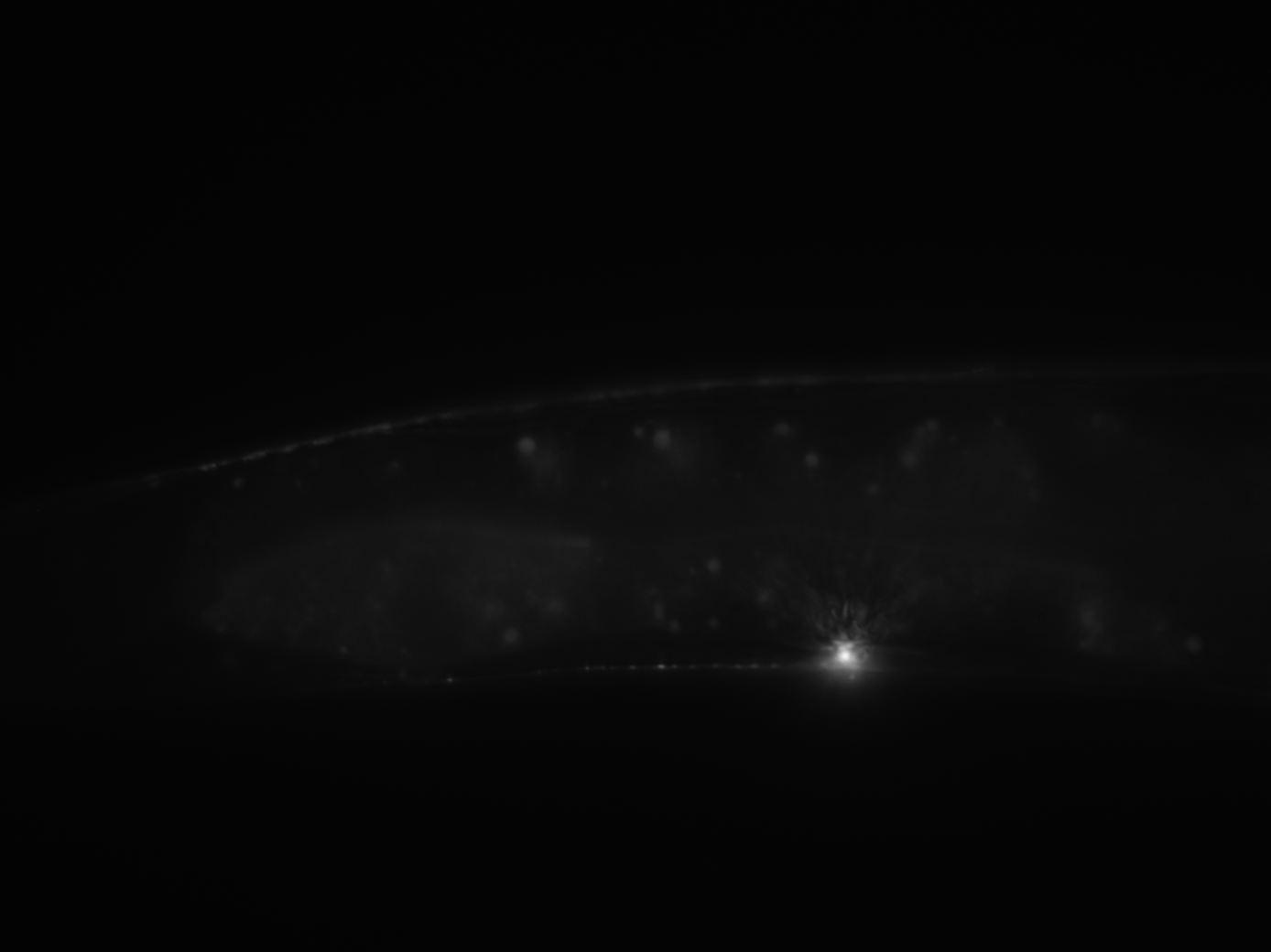

Supplement: Supplementary file 7 — Source data Fig. 6 [file 44319_2025_493_MOESM7_ESM.zip › Figure6/Fig6H/Experiment-69_wt_cholinergic.tif_files/Experiment-69_z5c0x0-1388y0-1040.tif]

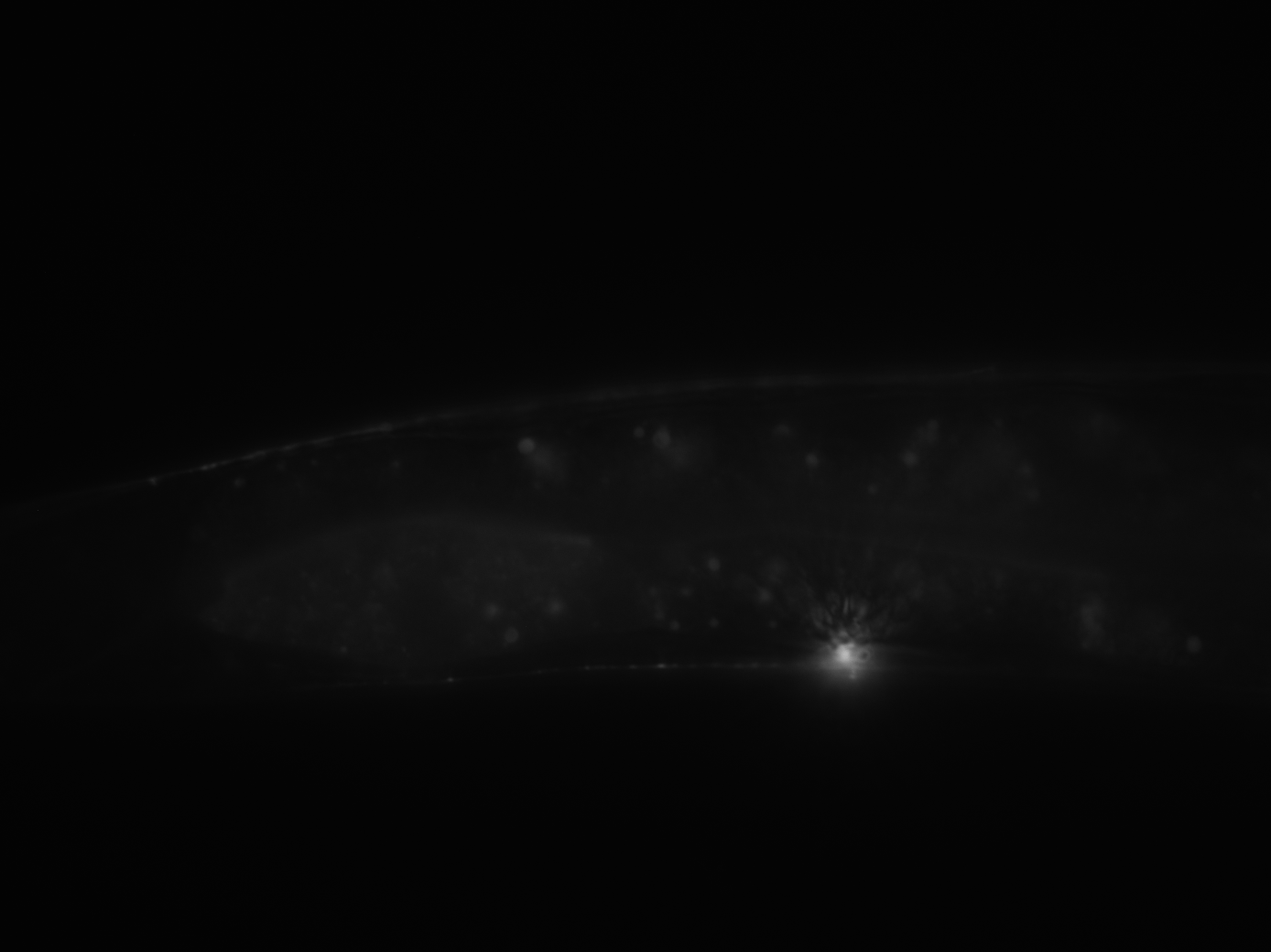

Supplement: Supplementary file 7 — Source data Fig. 6 [file 44319_2025_493_MOESM7_ESM.zip › Figure6/Fig6H/Experiment-69_wt_cholinergic.tif_files/Experiment-69_z4c0x0-1388y0-1040.tif]

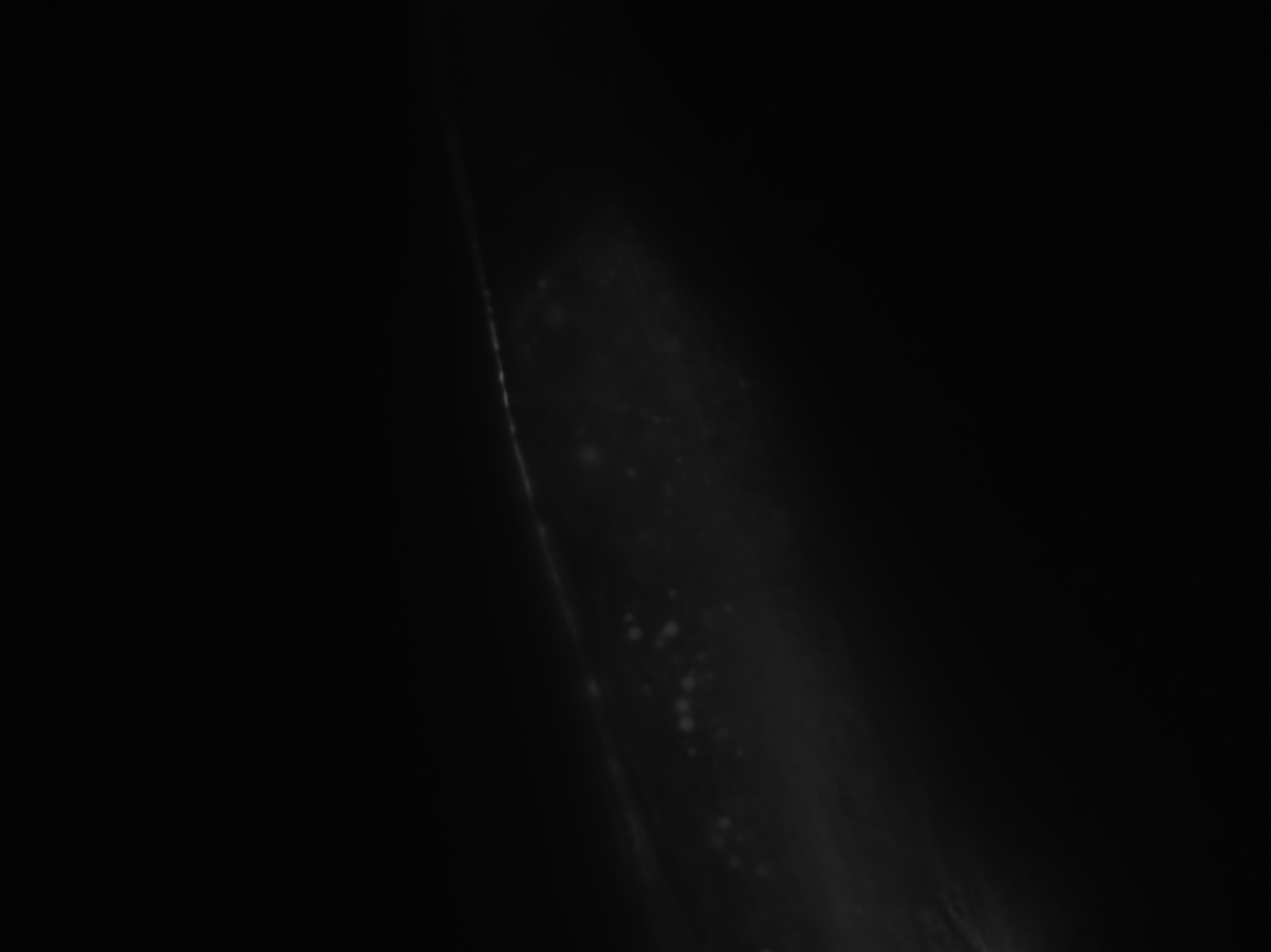

Supplement: Supplementary file 7 — Source data Fig. 6 [file 44319_2025_493_MOESM7_ESM.zip › Figure6/Fig6H/Experiment-74_OEskipped_cholinergic.tif_files/Experiment-74_z6c0x0-1388y0-1040.tif]

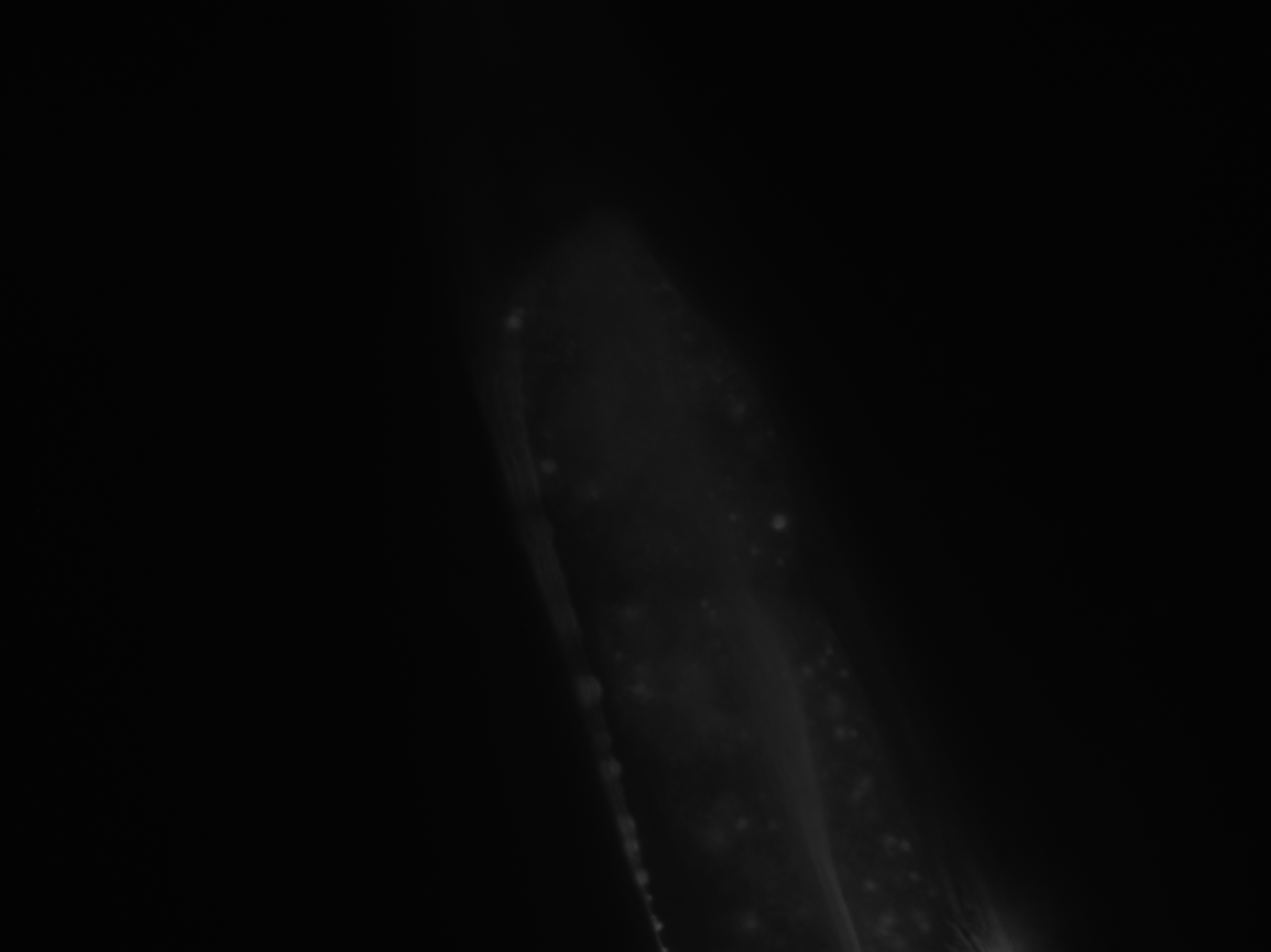

Supplement: Supplementary file 7 — Source data Fig. 6 [file 44319_2025_493_MOESM7_ESM.zip › Figure6/Fig6H/Experiment-74_OEskipped_cholinergic.tif_files/Experiment-74_z11c0x0-1388y0-1040.tif]

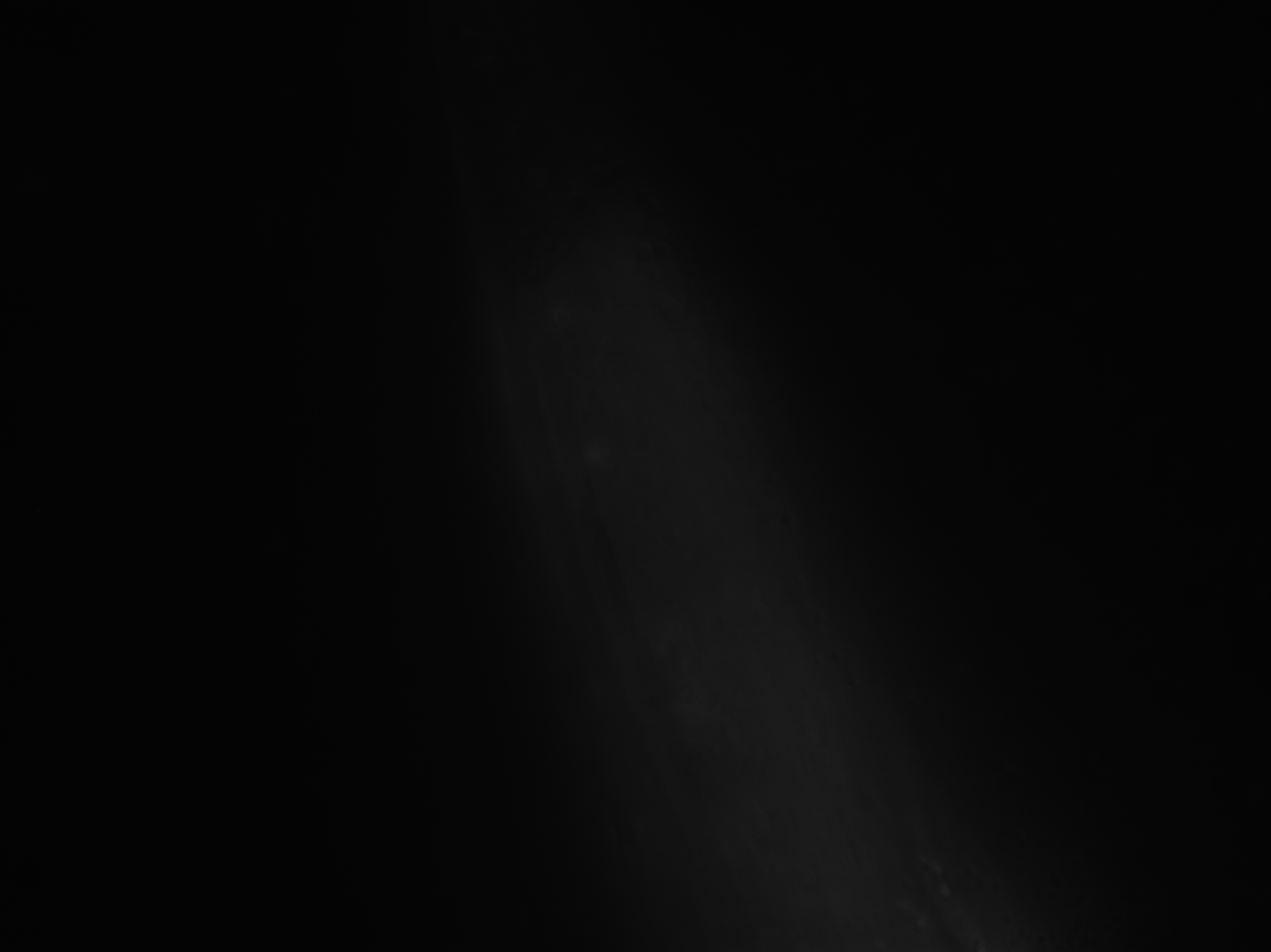

Supplement: Supplementary file 7 — Source data Fig. 6 [file 44319_2025_493_MOESM7_ESM.zip › Figure6/Fig6H/Experiment-74_OEskipped_cholinergic.tif_files/Experiment-74_z1c0x0-1388y0-1040.tif]

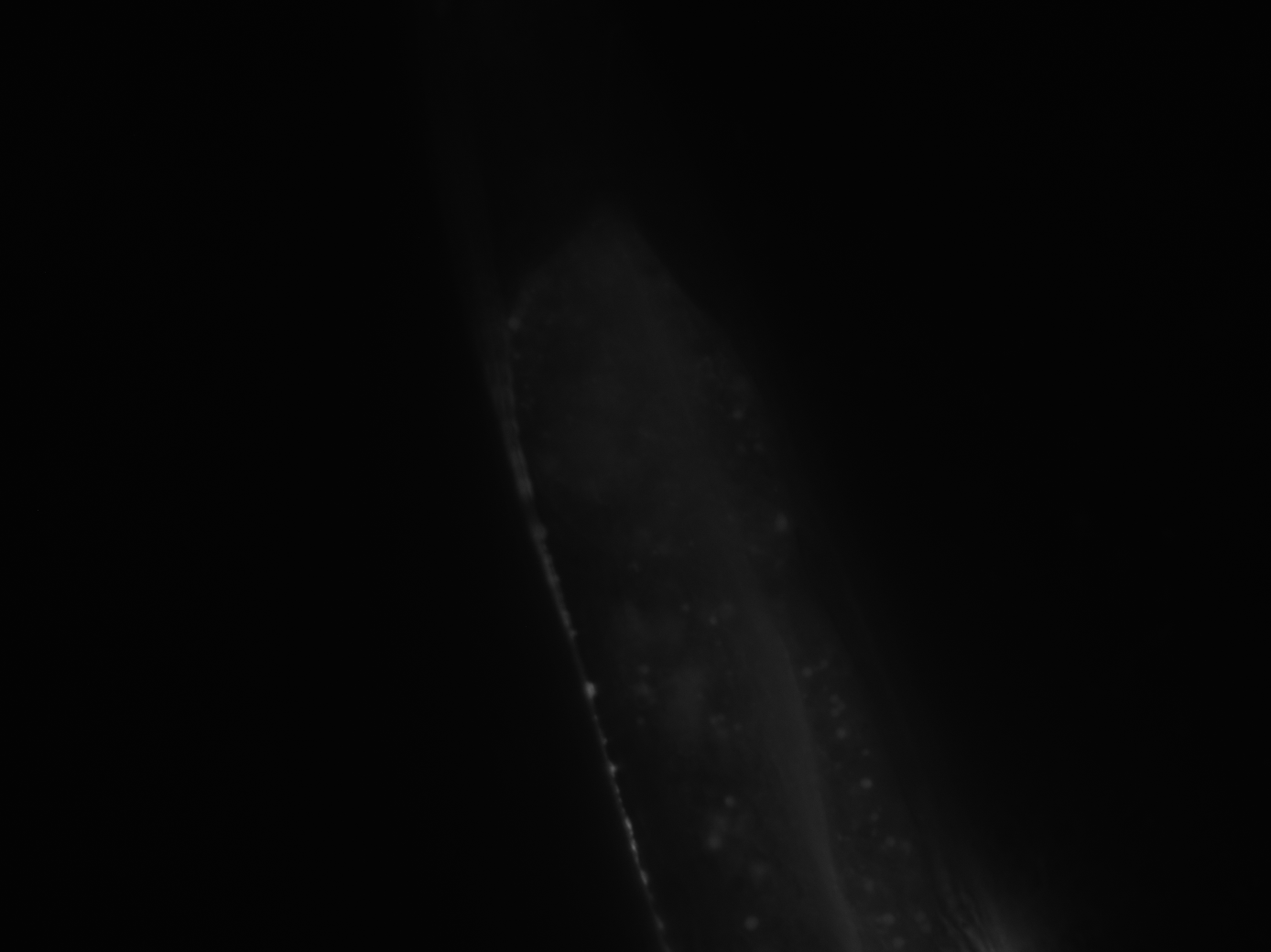

Supplement: Supplementary file 7 — Source data Fig. 6 [file 44319_2025_493_MOESM7_ESM.zip › Figure6/Fig6H/Experiment-74_OEskipped_cholinergic.tif_files/Experiment-74_z9c0x0-1388y0-1040.tif]

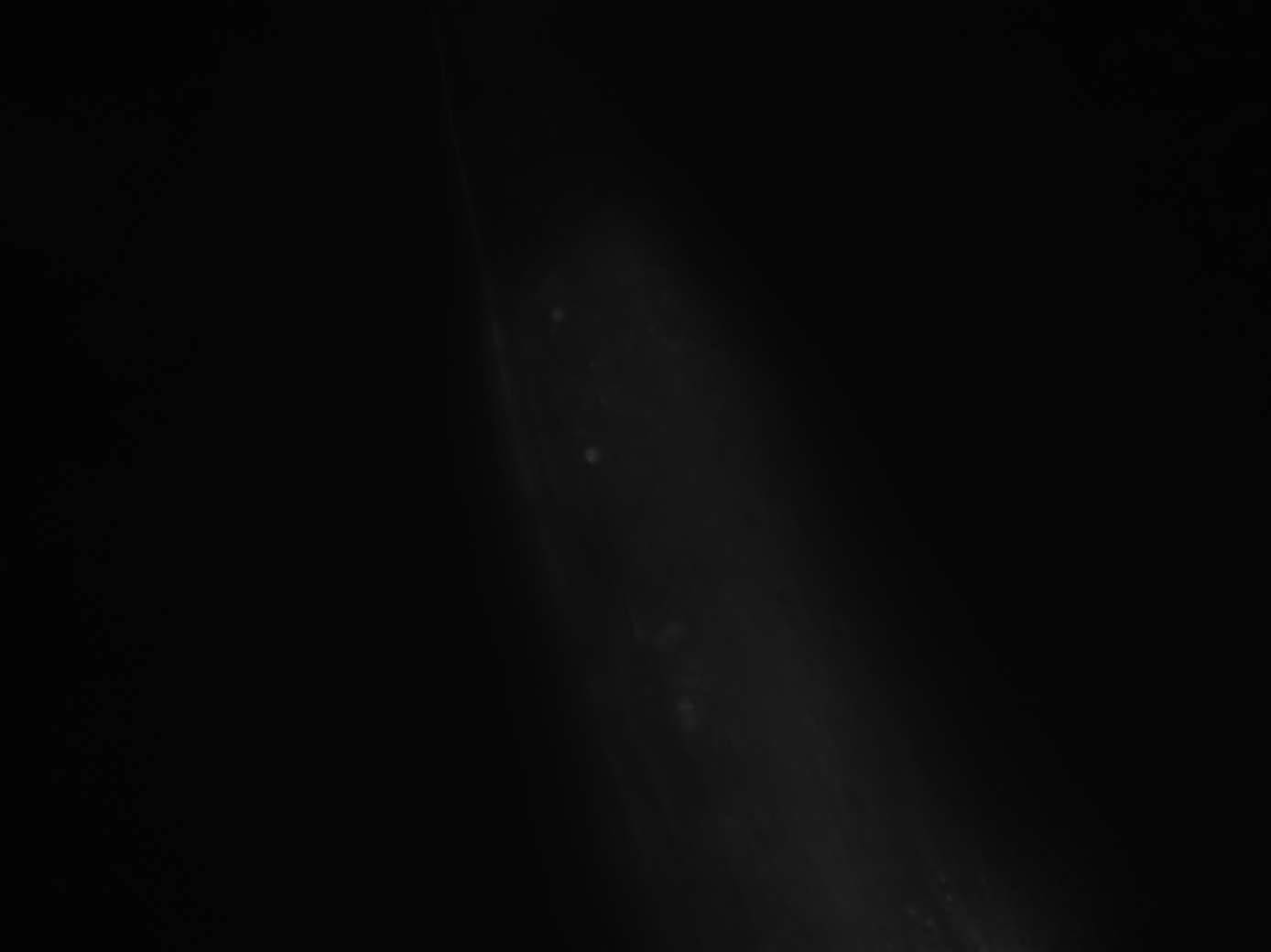

Supplement: Supplementary file 7 — Source data Fig. 6 [file 44319_2025_493_MOESM7_ESM.zip › Figure6/Fig6H/Experiment-74_OEskipped_cholinergic.tif_files/Experiment-74_z3c0x0-1388y0-1040.tif]

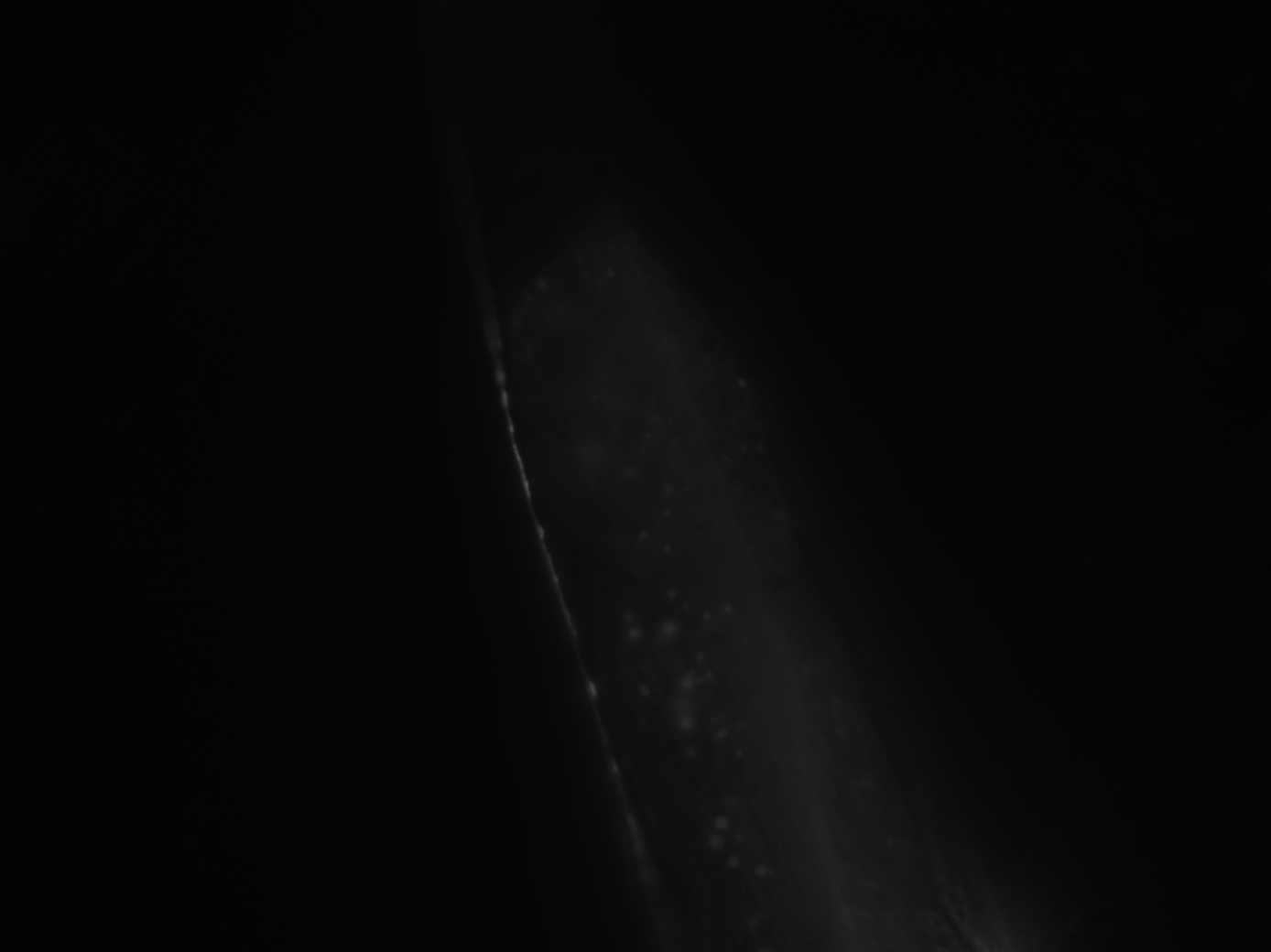

Supplement: Supplementary file 7 — Source data Fig. 6 [file 44319_2025_493_MOESM7_ESM.zip › Figure6/Fig6H/Experiment-74_OEskipped_cholinergic.tif_files/Experiment-74_z7c0x0-1388y0-1040.tif]

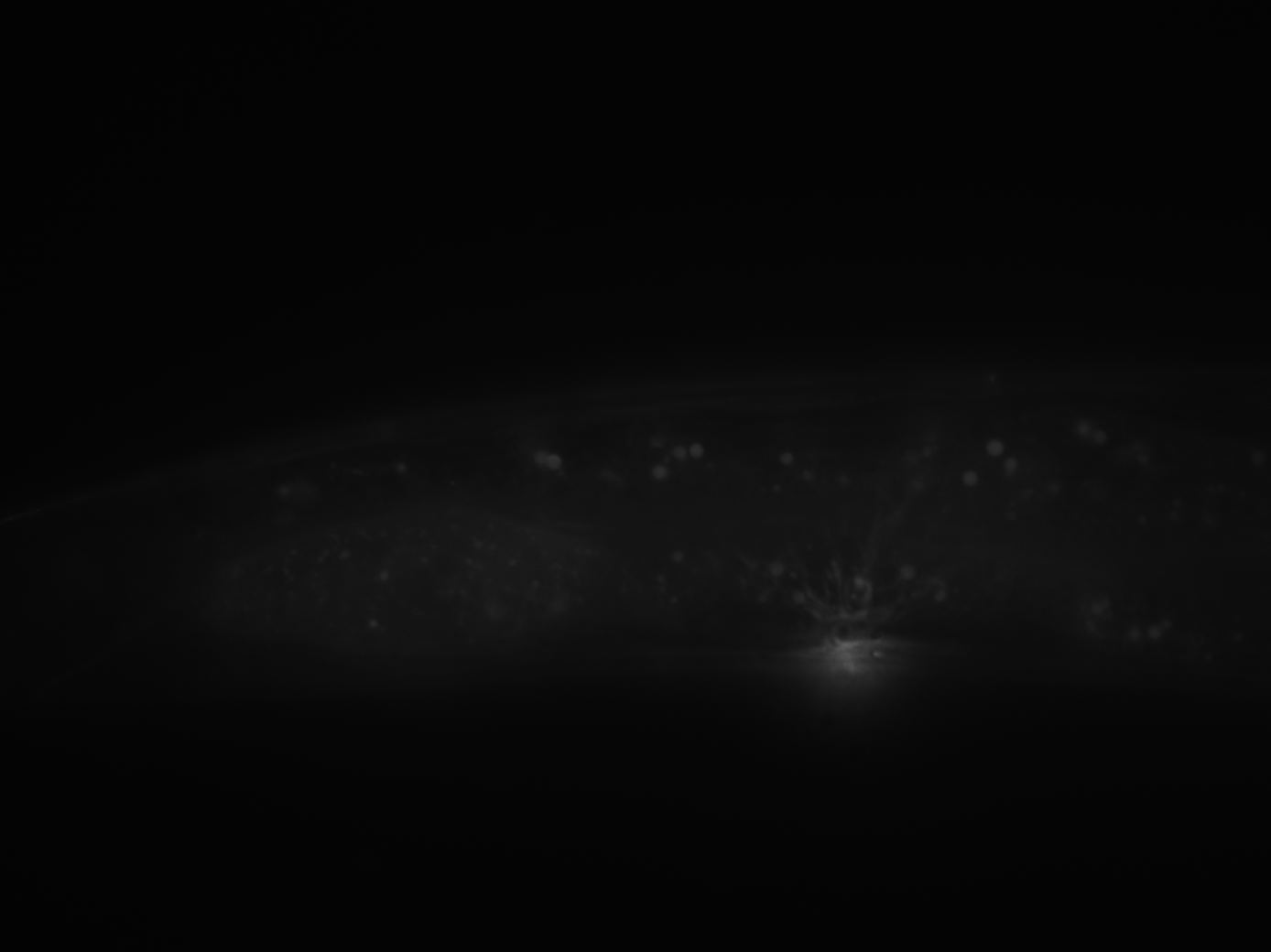

Supplement: Supplementary file 7 — Source data Fig. 6 [file 44319_2025_493_MOESM7_ESM.zip › Figure6/Fig6H/Experiment-69_wt_cholinergic.tif_files/Experiment-69_z0c0x0-1388y0-1040.tif]

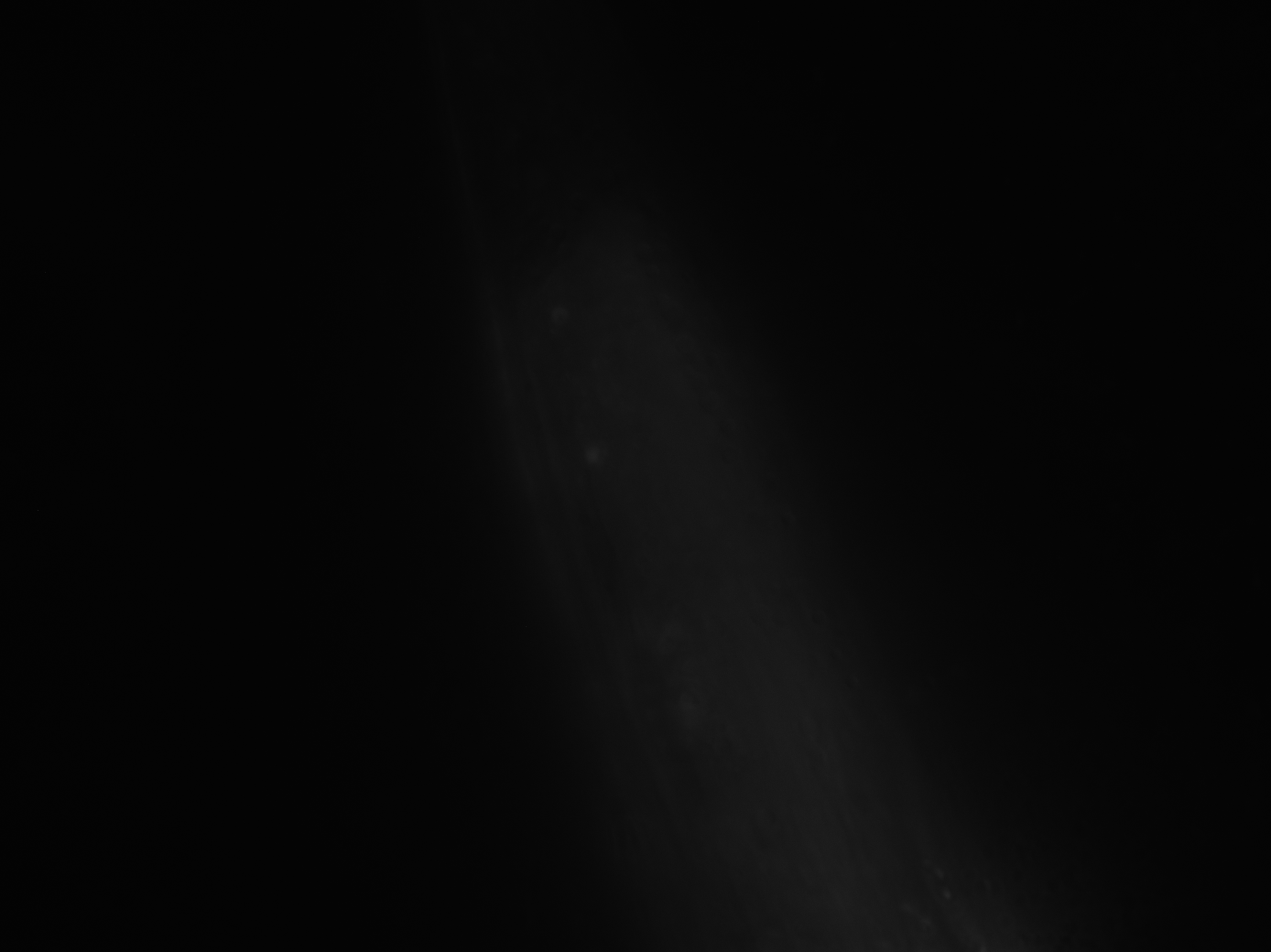

Supplement: Supplementary file 7 — Source data Fig. 6 [file 44319_2025_493_MOESM7_ESM.zip › Figure6/Fig6H/Experiment-74_OEskipped_cholinergic.tif_files/Experiment-74_z2c0x0-1388y0-1040.tif]

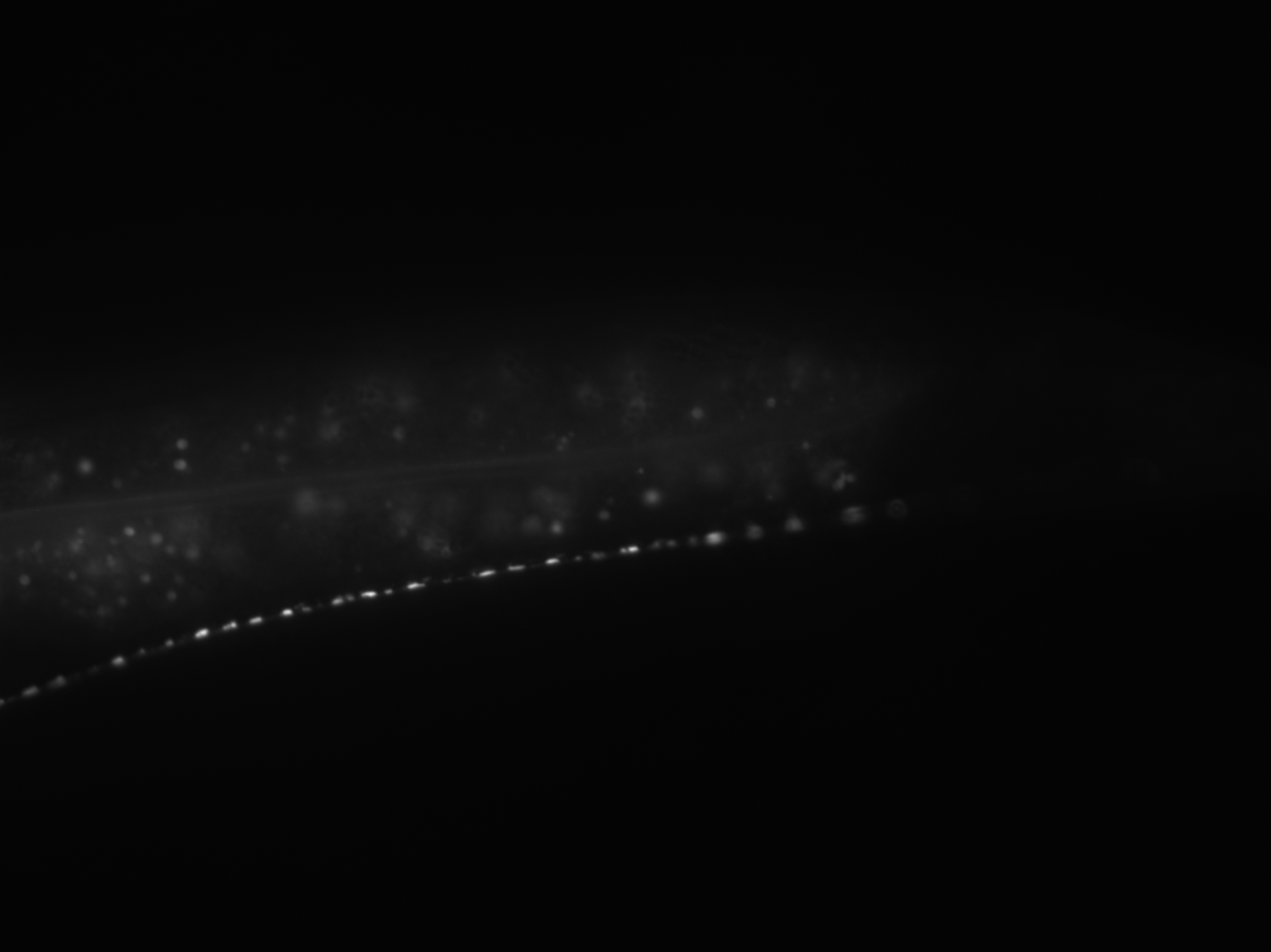

Supplement: Supplementary file 7 — Source data Fig. 6 [file 44319_2025_493_MOESM7_ESM.zip › Figure6/Fig6H/Experiment-50_cholinergic_s69.tif_files/Experiment-50_z3c0x0-1388y0-1040.tif]

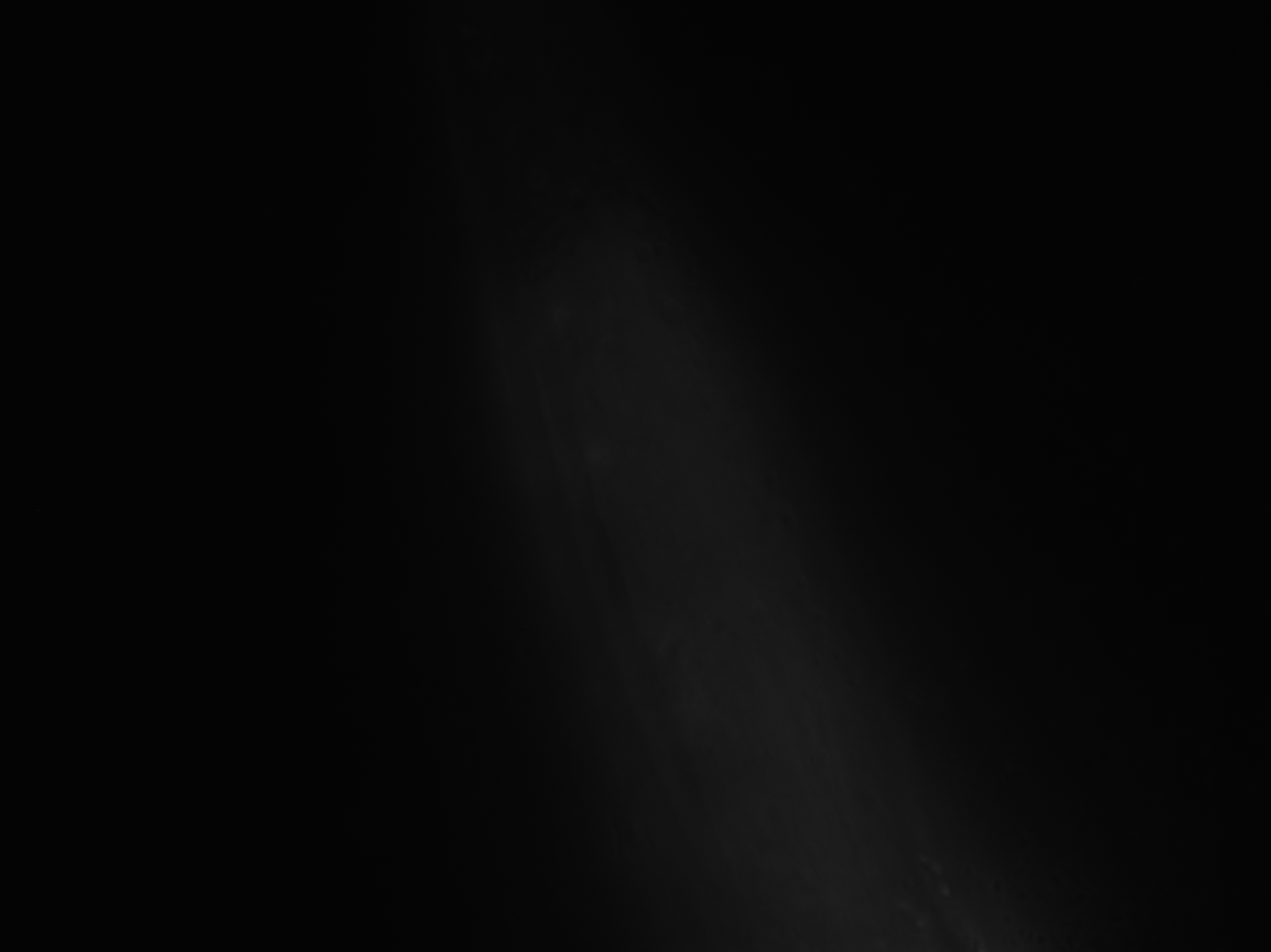

Supplement: Supplementary file 7 — Source data Fig. 6 [file 44319_2025_493_MOESM7_ESM.zip › Figure6/Fig6H/Experiment-74_OEskipped_cholinergic.tif_files/Experiment-74_z0c0x0-1388y0-1040.tif]

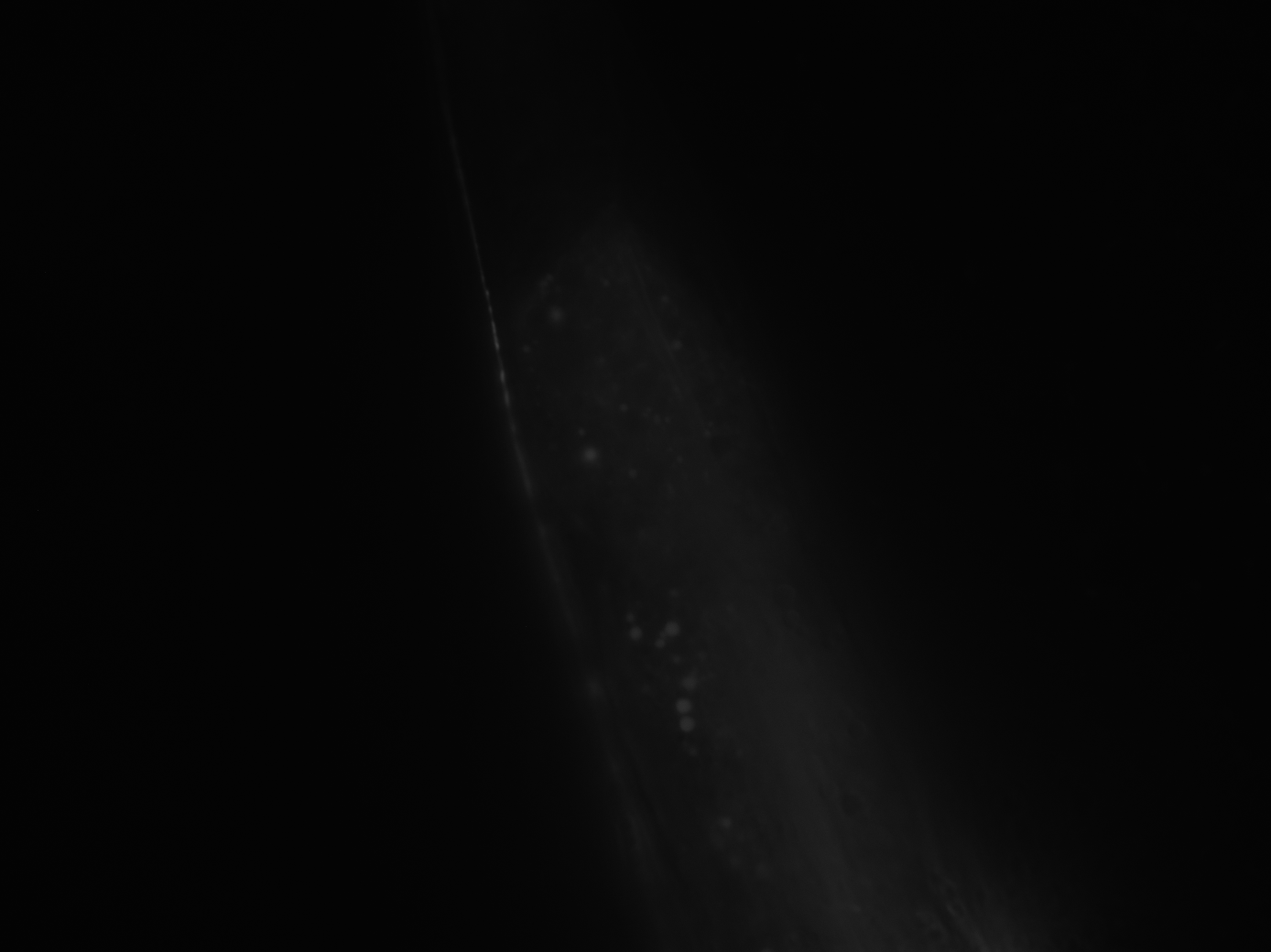

Supplement: Supplementary file 7 — Source data Fig. 6 [file 44319_2025_493_MOESM7_ESM.zip › Figure6/Fig6H/Experiment-74_OEskipped_cholinergic.tif_files/Experiment-74_z5c0x0-1388y0-1040.tif]

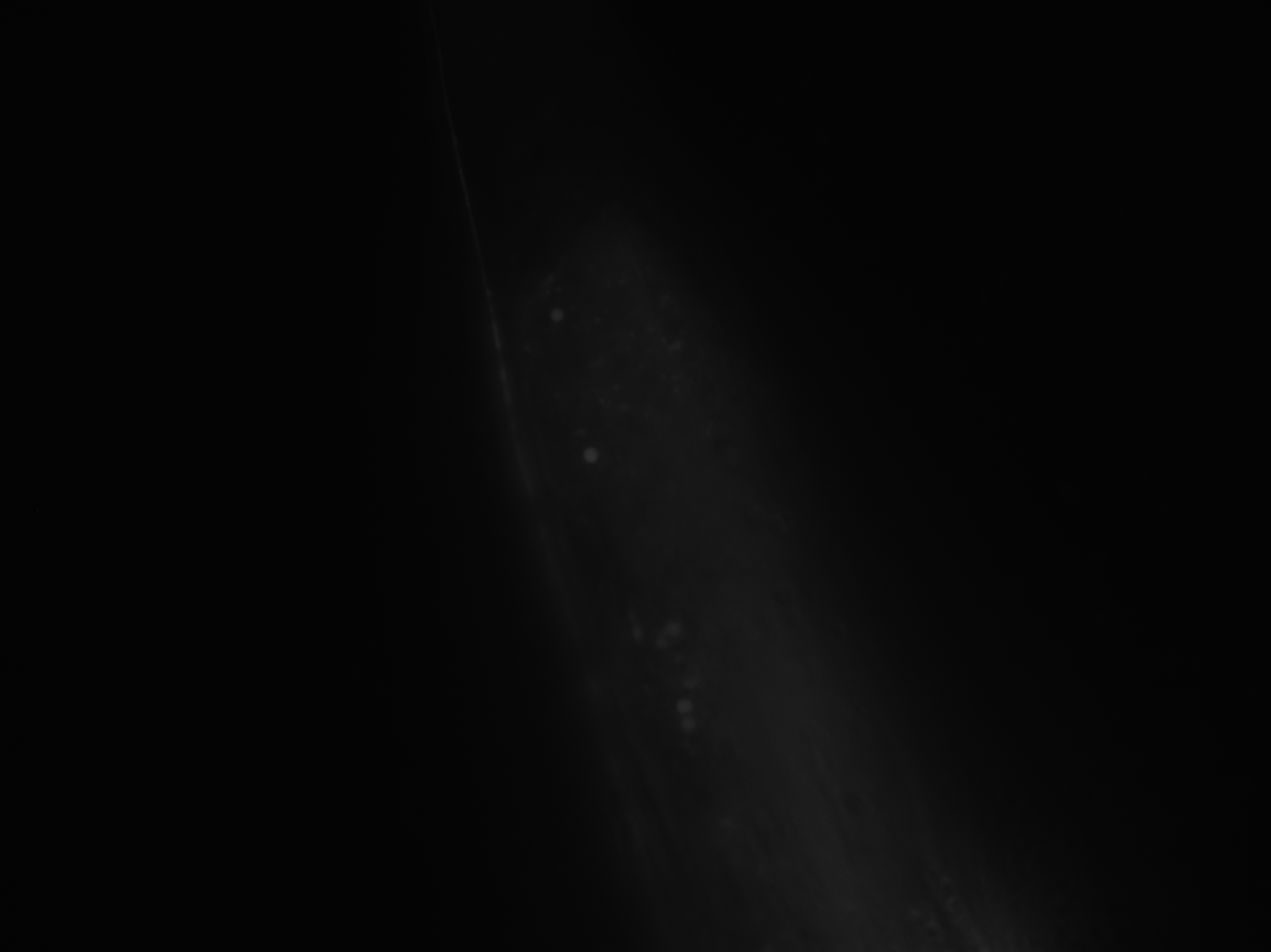

Supplement: Supplementary file 7 — Source data Fig. 6 [file 44319_2025_493_MOESM7_ESM.zip › Figure6/Fig6H/Experiment-74_OEskipped_cholinergic.tif_files/Experiment-74_z4c0x0-1388y0-1040.tif]

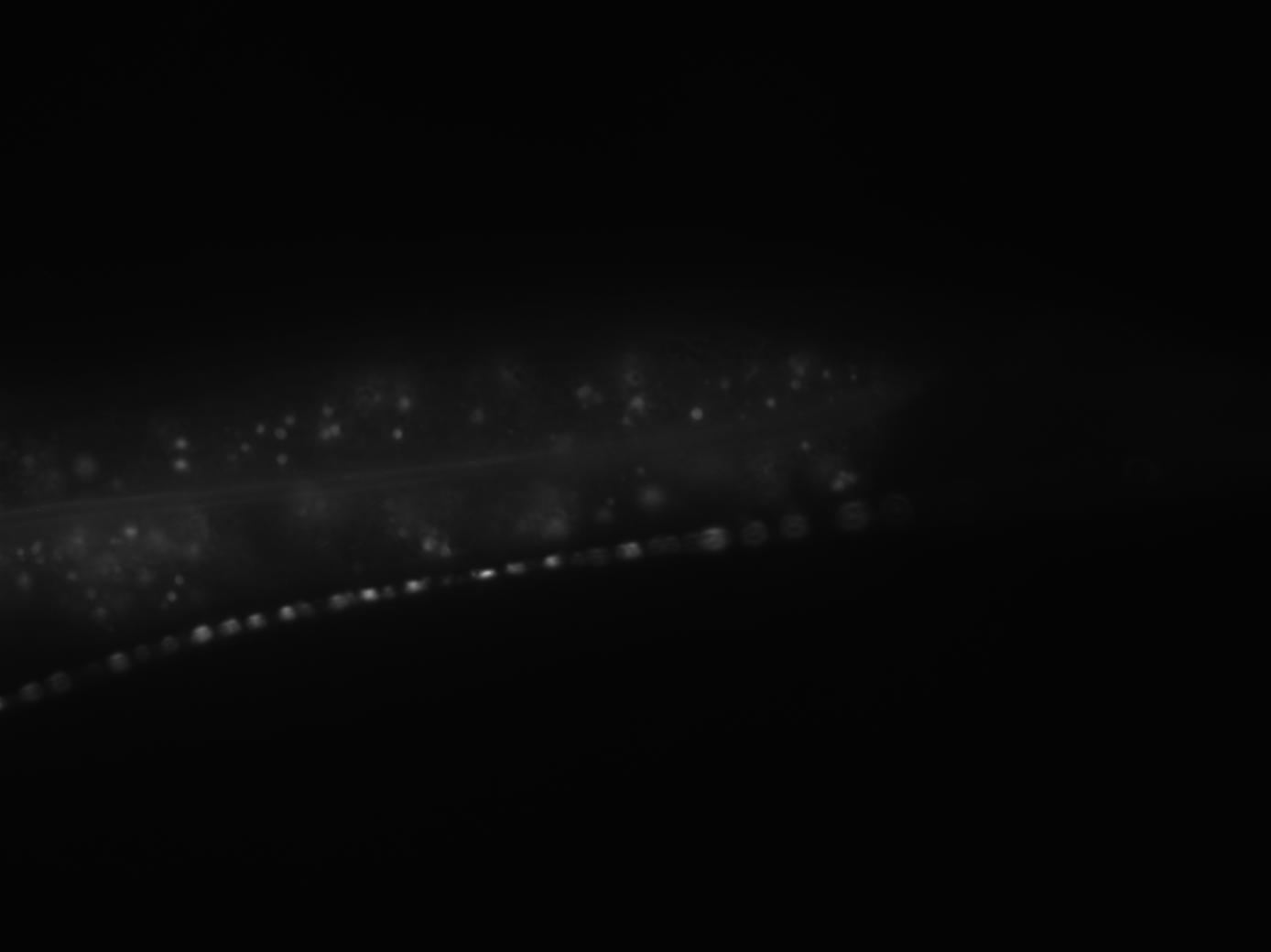

Supplement: Supplementary file 7 — Source data Fig. 6 [file 44319_2025_493_MOESM7_ESM.zip › Figure6/Fig6H/Experiment-50_cholinergic_s69.tif_files/Experiment-50_z4c0x0-1388y0-1040.tif]

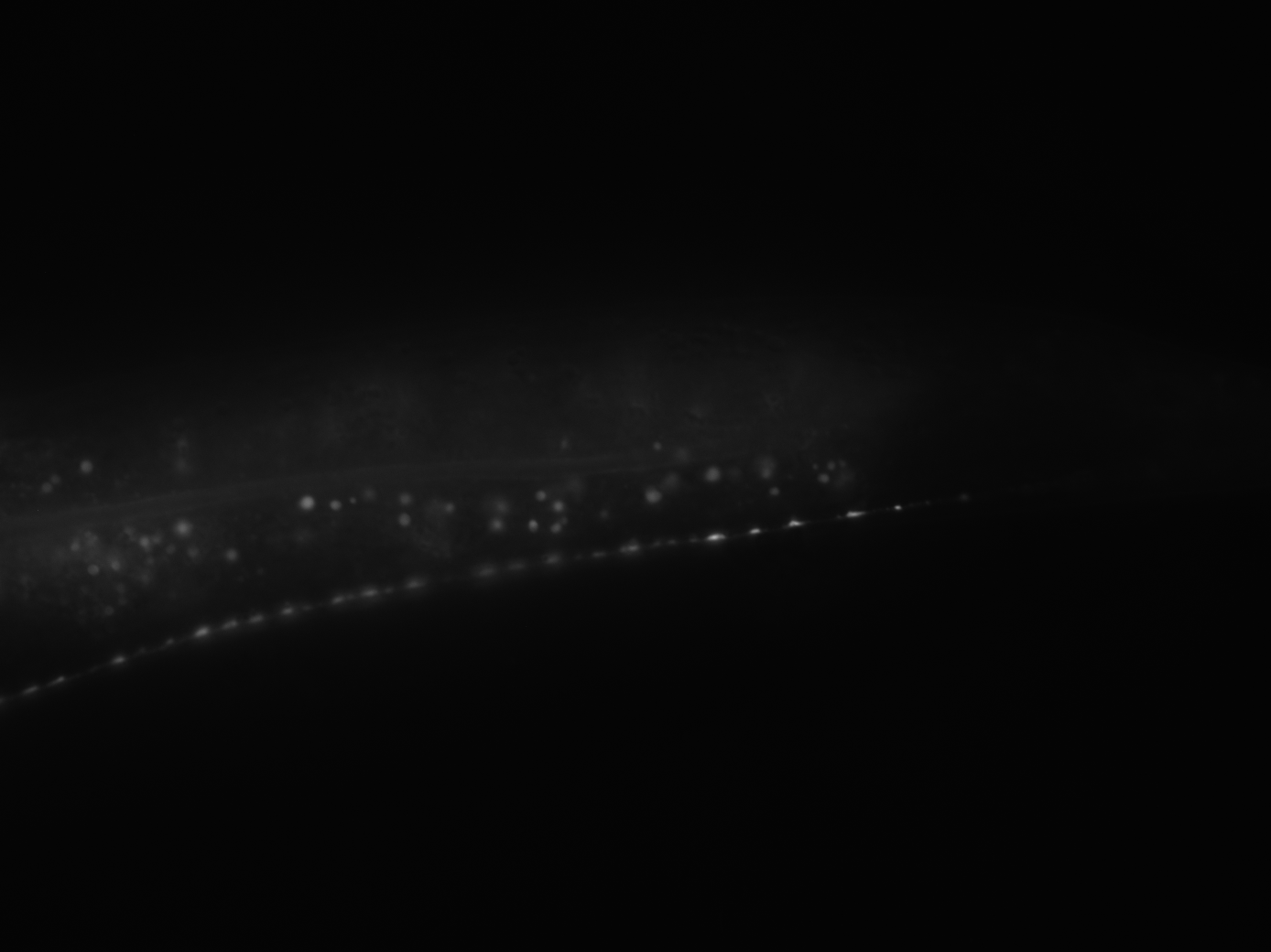

Supplement: Supplementary file 7 — Source data Fig. 6 [file 44319_2025_493_MOESM7_ESM.zip › Figure6/Fig6H/Experiment-50_cholinergic_s69.tif_files/Experiment-50_z1c0x0-1388y0-1040.tif]

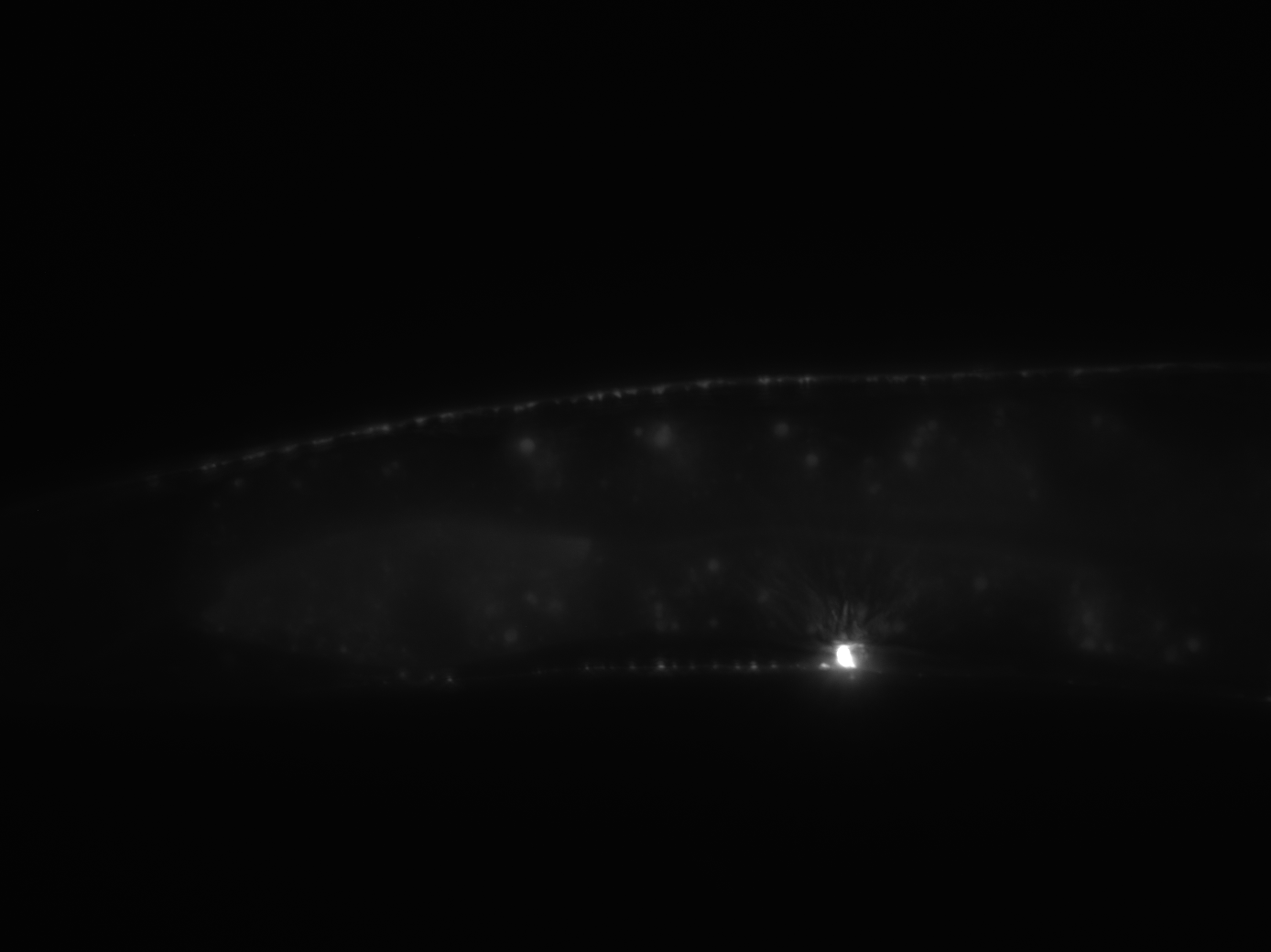

Supplement: Supplementary file 7 — Source data Fig. 6 [file 44319_2025_493_MOESM7_ESM.zip › Figure6/Fig6H/Experiment-69_wt_cholinergic.tif_files/processed/MAX_Experiment-69.tif_files.tif]

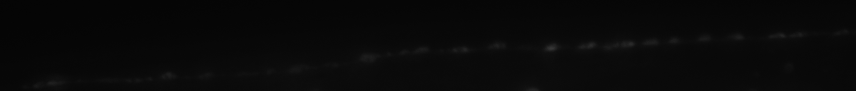

Supplement: Supplementary file 7 — Source data Fig. 6 [file 44319_2025_493_MOESM7_ESM.zip › Figure6/Fig6H/Experiment-03_GABA_wt.tif_files/processed/MAX_Experiment-03.tif_files.tif]

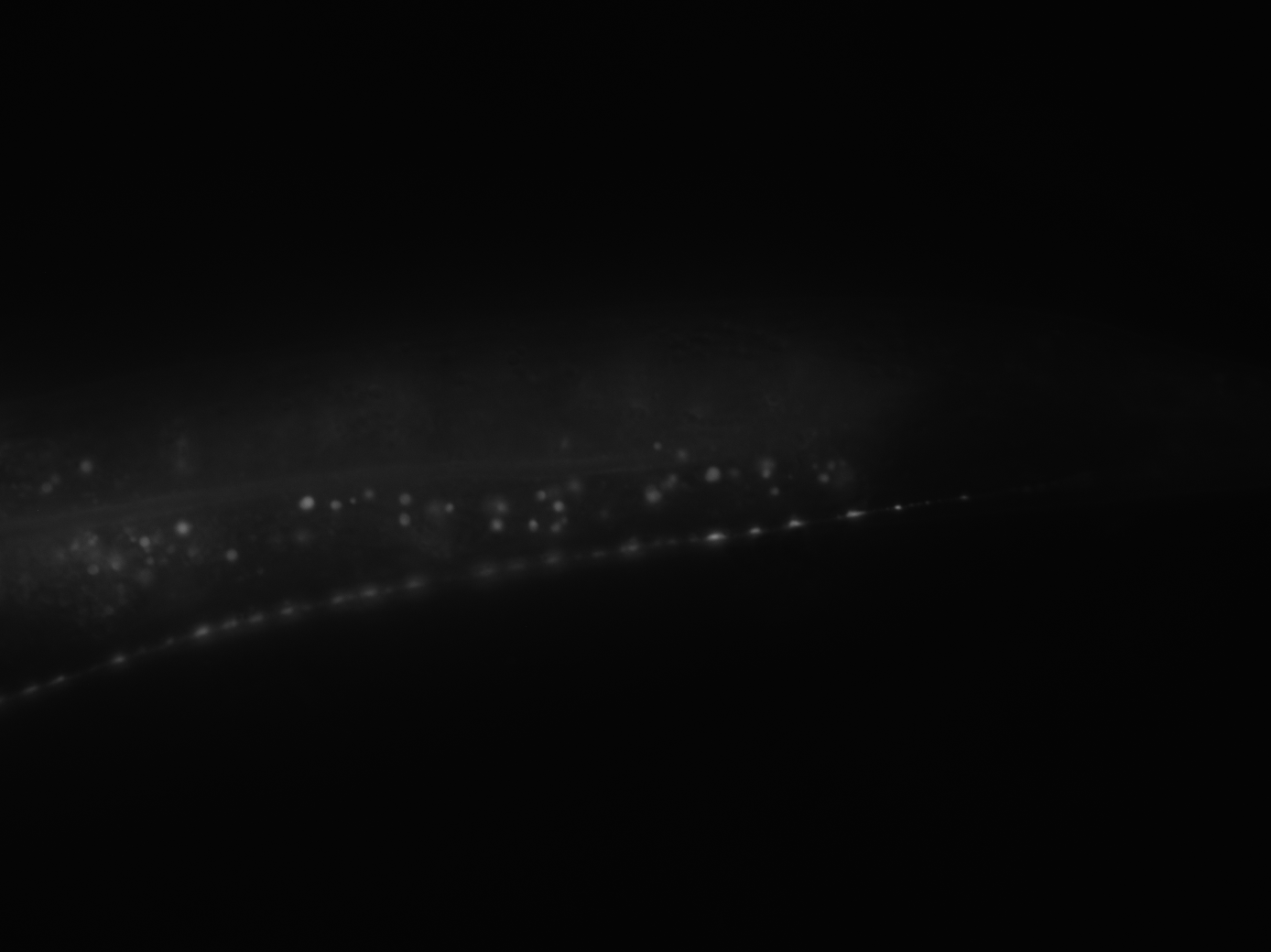

Supplement: Supplementary file 7 — Source data Fig. 6 [file 44319_2025_493_MOESM7_ESM.zip › Figure6/Fig6H/Experiment-50_cholinergic_s69.tif_files/Experiment-50_z0c0x0-1388y0-1040.tif]

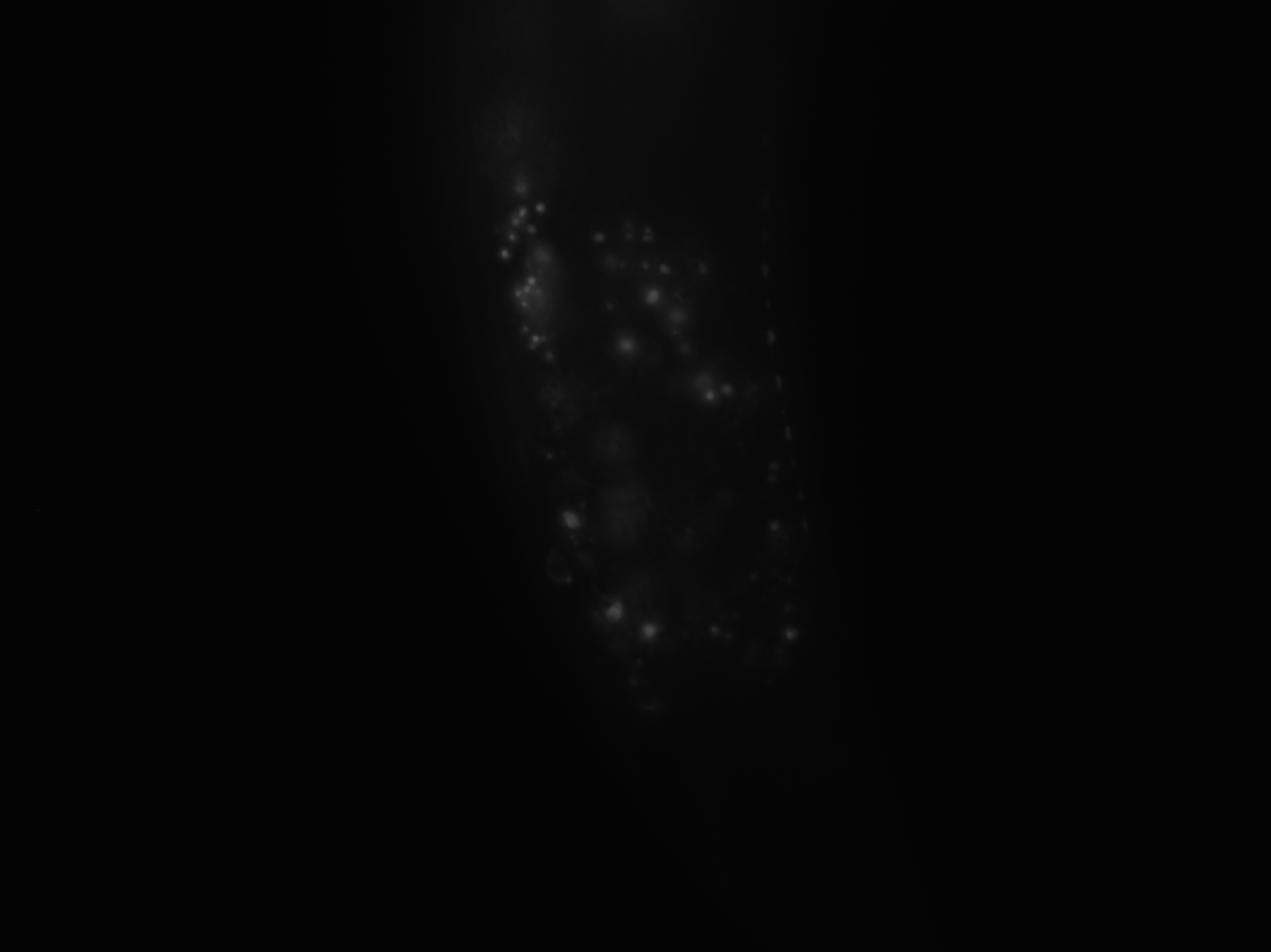

Supplement: Supplementary file 7 — Source data Fig. 6 [file 44319_2025_493_MOESM7_ESM.zip › Figure6/Fig6H/Experiment-07_GABA_s69.tif_files/vc/MAX_Experiment-07.tif_files.tif]

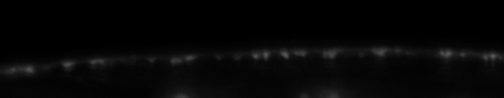

Supplement: Supplementary file 7 — Source data Fig. 6 [file 44319_2025_493_MOESM7_ESM.zip › Figure6/Fig6H/Experiment-69_wt_cholinergic.tif_files/processed/wt1.tif]

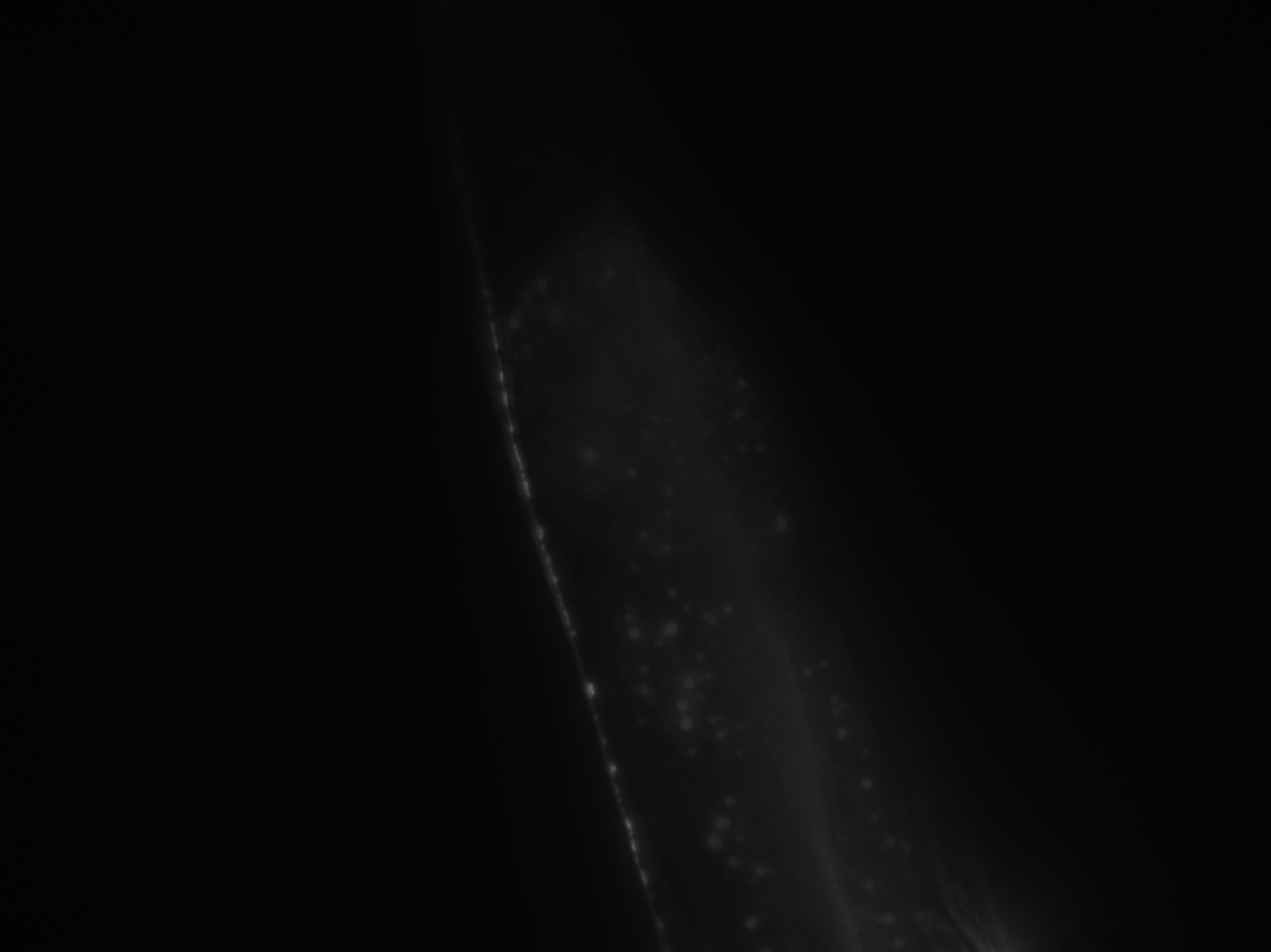

Supplement: Supplementary file 7 — Source data Fig. 6 [file 44319_2025_493_MOESM7_ESM.zip › Figure6/Fig6H/Experiment-74_OEskipped_cholinergic.tif_files/processed/MAX_Experiment-74.tif_files.tif]

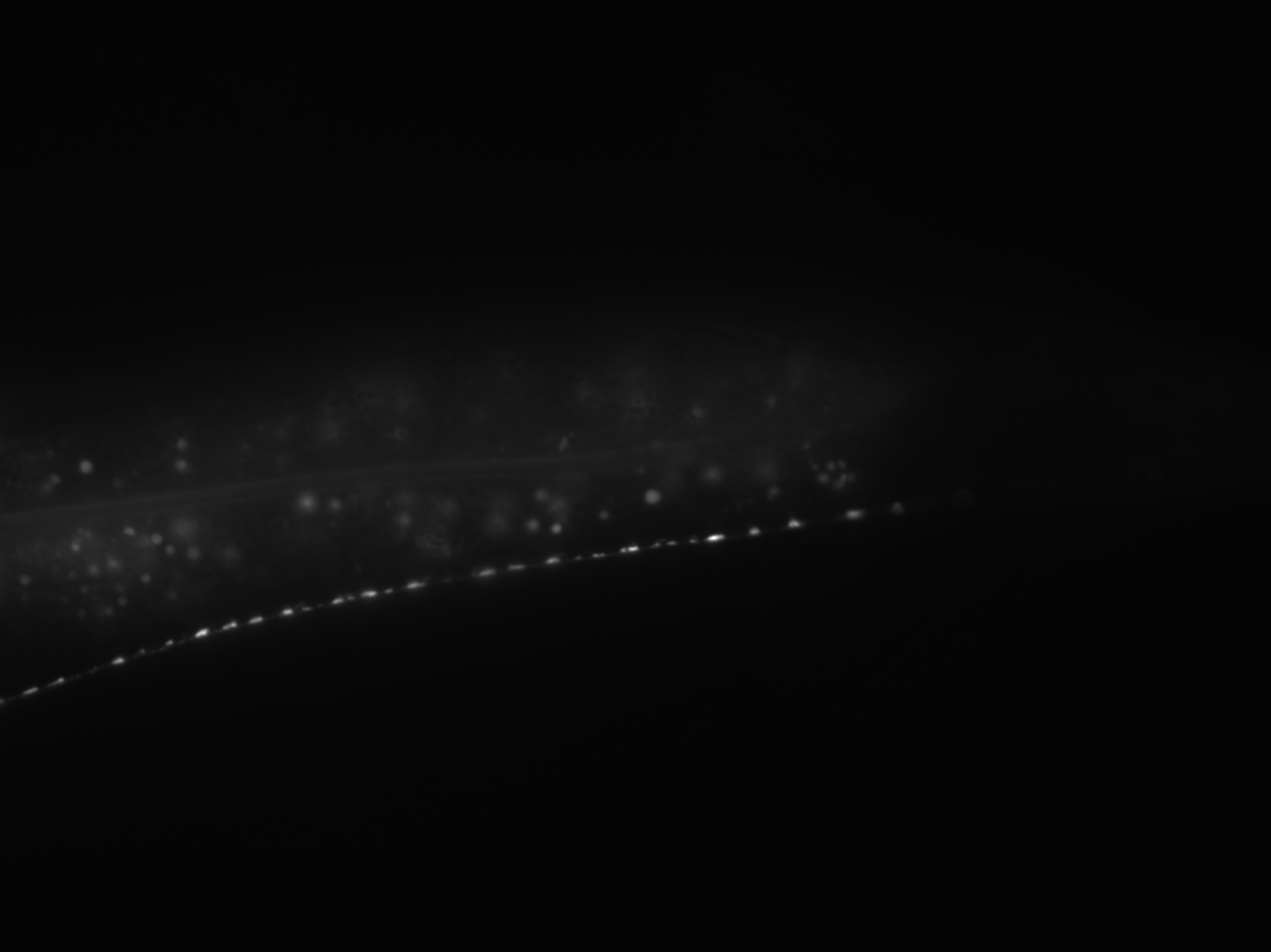

Supplement: Supplementary file 7 — Source data Fig. 6 [file 44319_2025_493_MOESM7_ESM.zip › Figure6/Fig6H/Experiment-50_cholinergic_s69.tif_files/Experiment-50_z2c0x0-1388y0-1040.tif]

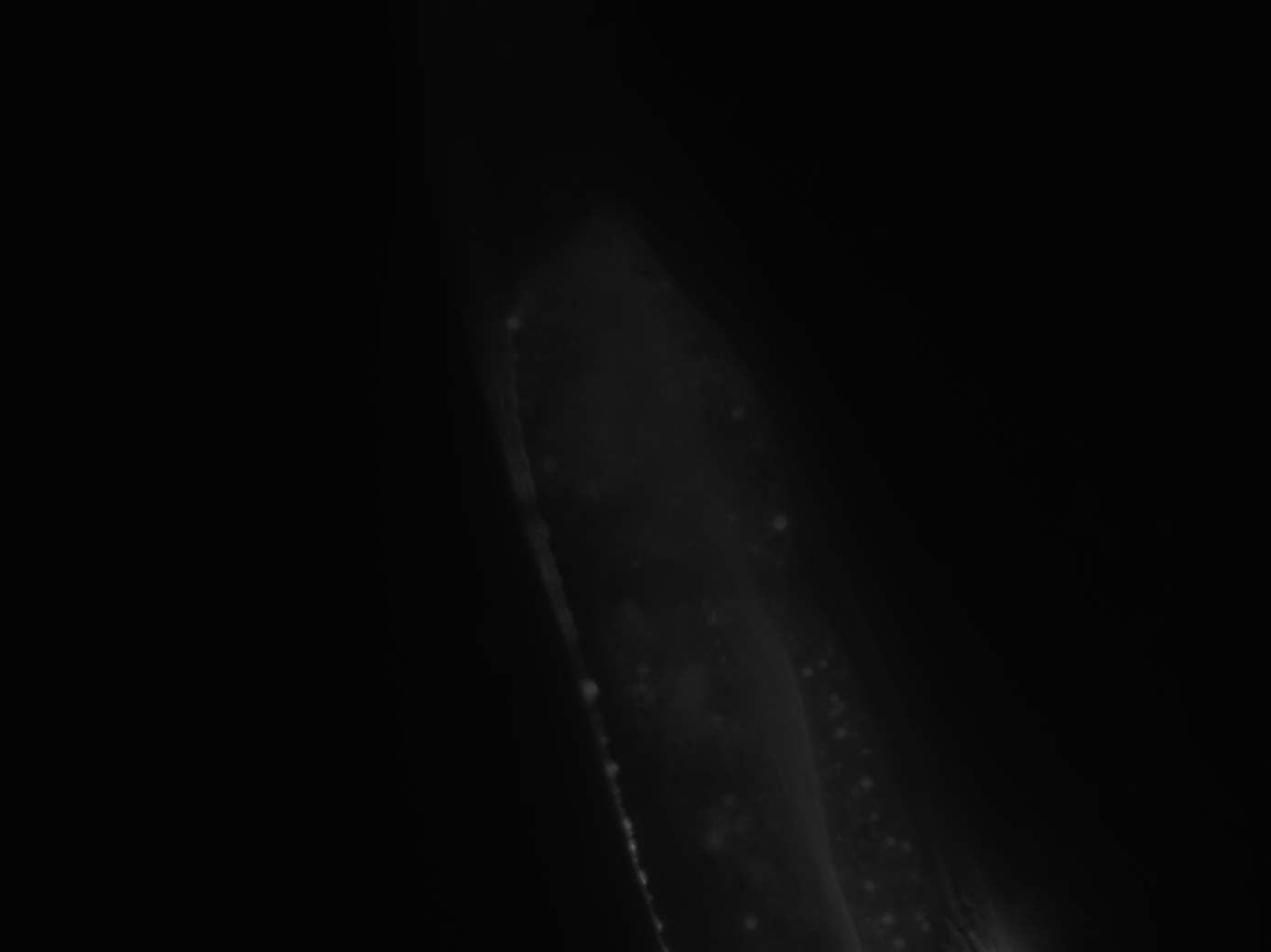

Supplement: Supplementary file 7 — Source data Fig. 6 [file 44319_2025_493_MOESM7_ESM.zip › Figure6/Fig6H/Experiment-74_OEskipped_cholinergic.tif_files/Experiment-74_z10c0x0-1388y0-1040.tif]

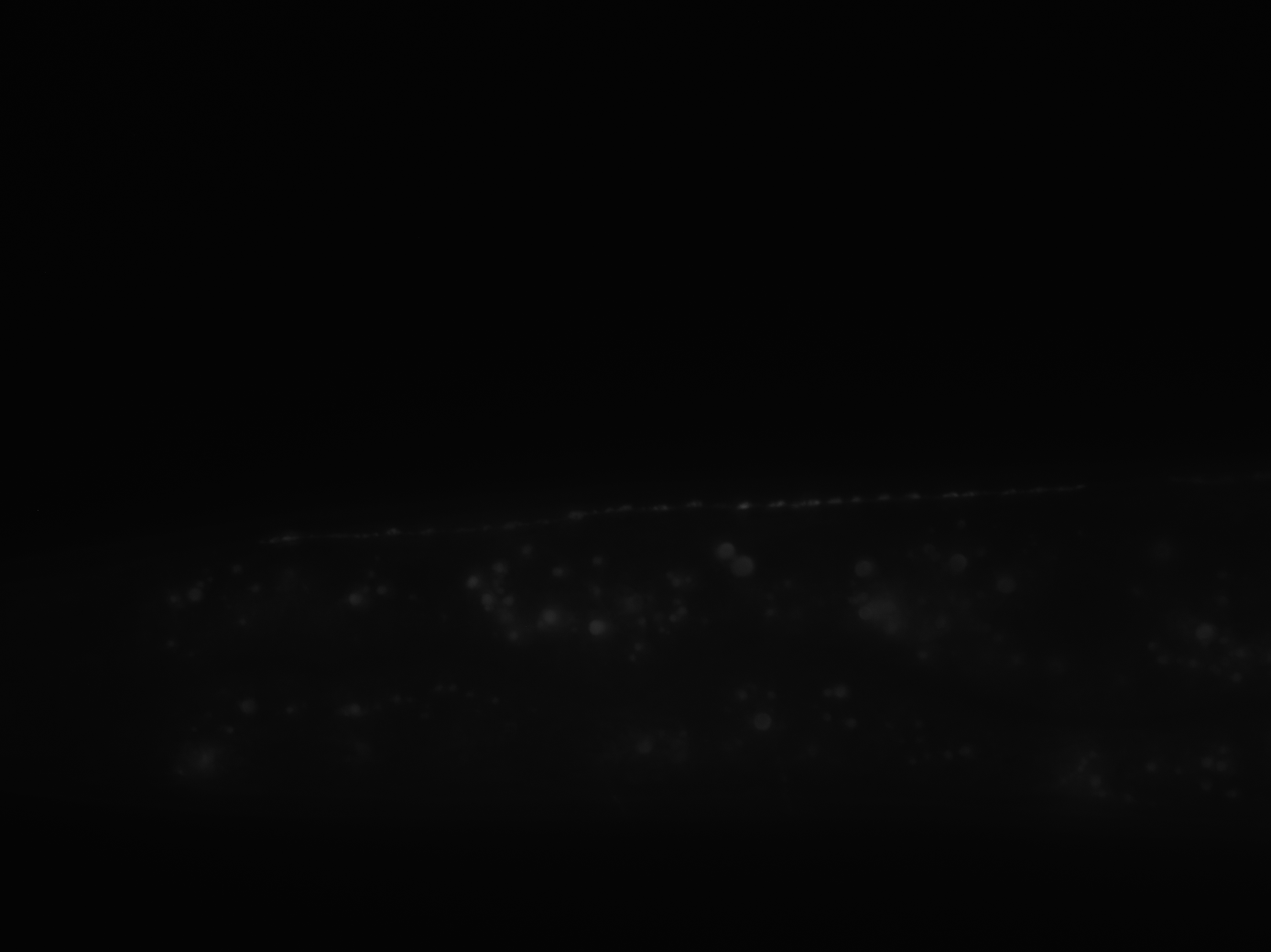

Supplement: Supplementary file 7 — Source data Fig. 6 [file 44319_2025_493_MOESM7_ESM.zip › Figure6/Fig6H/Experiment-03_GABA_wt.tif_files/vc/MAX_Experiment-03.tif_files.tif]

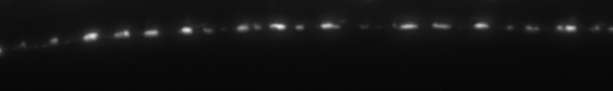

Supplement: Supplementary file 7 — Source data Fig. 6 [file 44319_2025_493_MOESM7_ESM.zip › Figure6/Fig6H/Experiment-50_cholinergic_s69.tif_files/processed/s69mutant.tif]

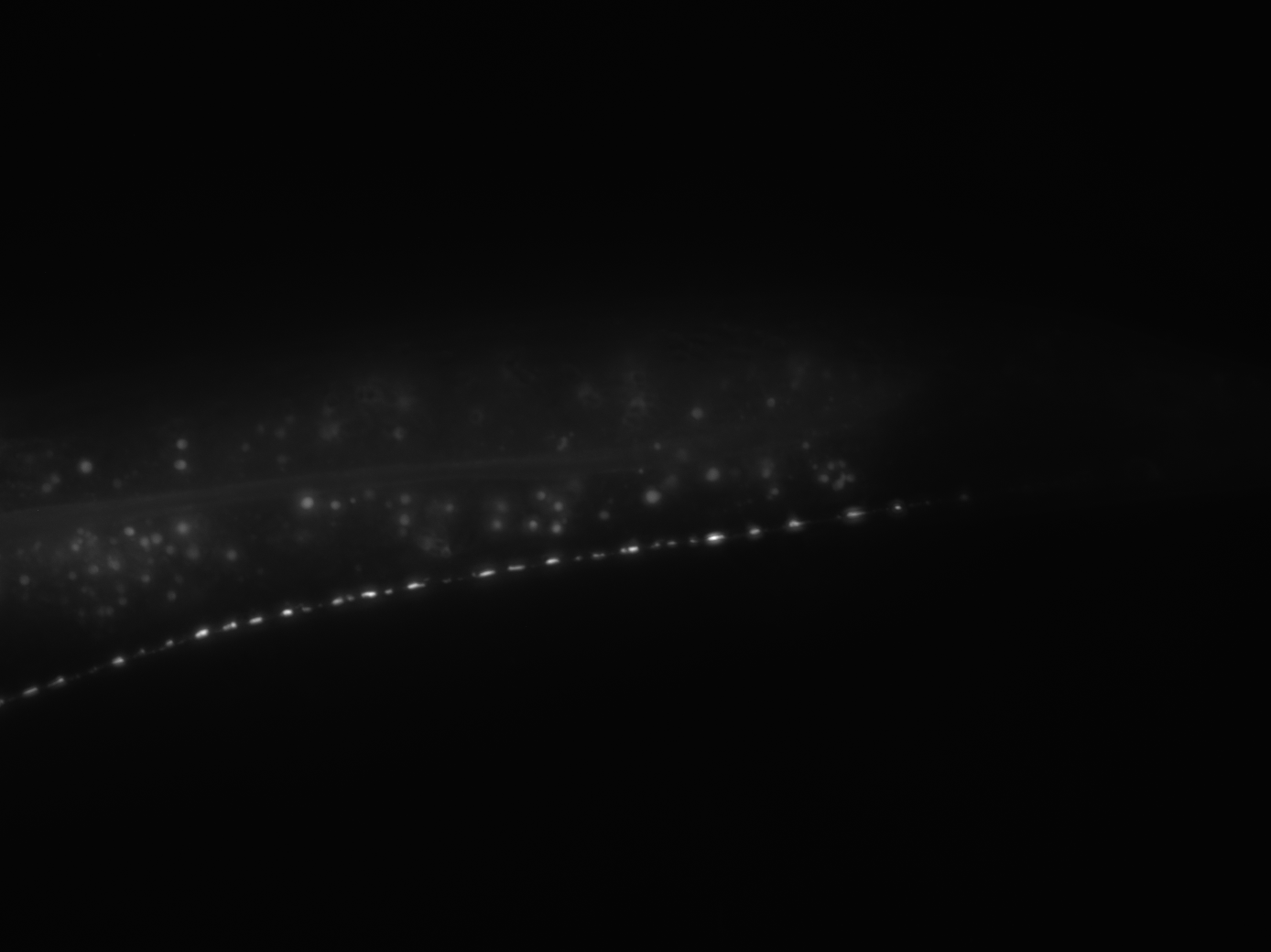

Supplement: Supplementary file 7 — Source data Fig. 6 [file 44319_2025_493_MOESM7_ESM.zip › Figure6/Fig6H/Experiment-50_cholinergic_s69.tif_files/processed/MAX_Experiment-50.tif_files.tif]

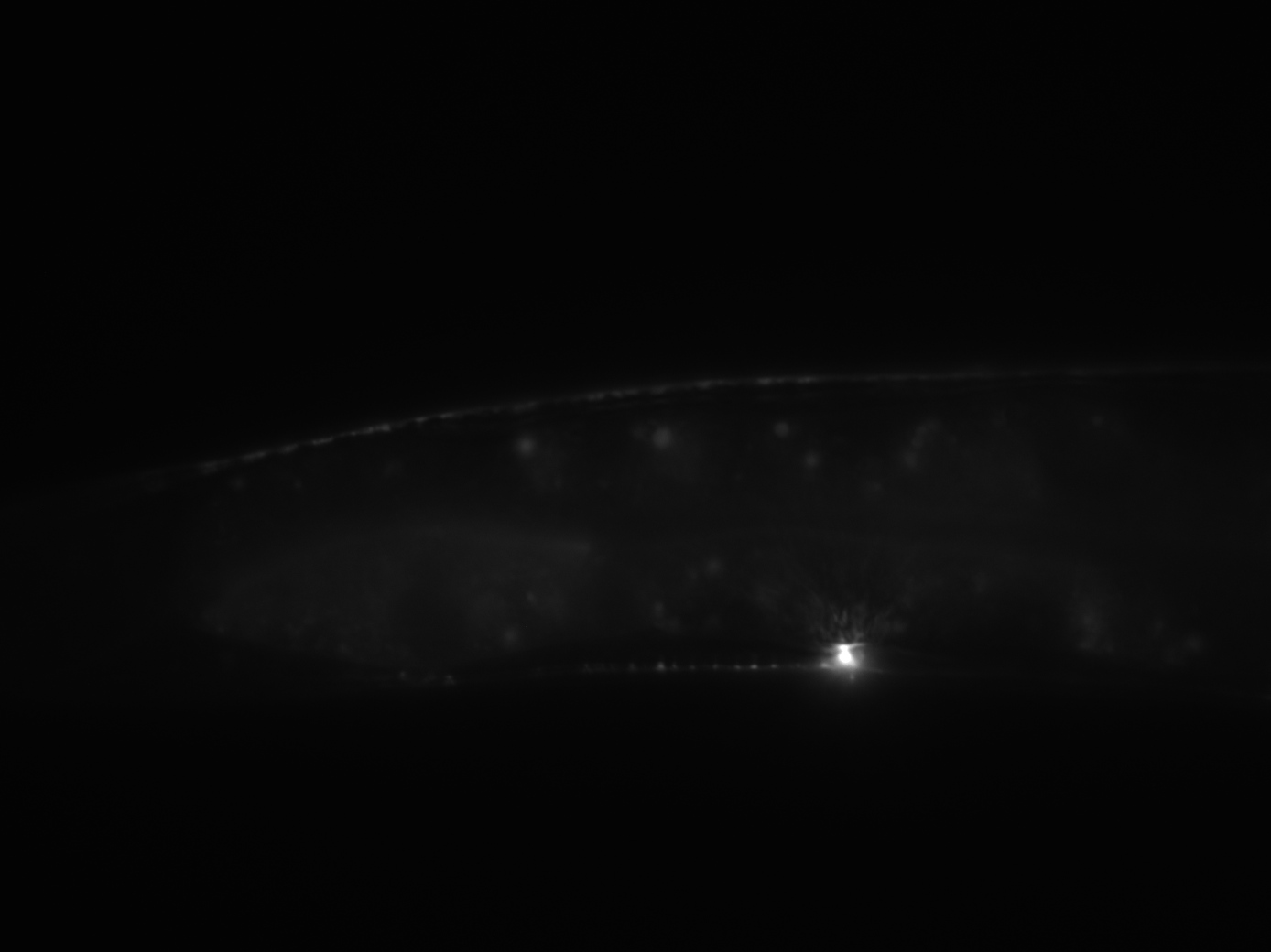

Supplement: Supplementary file 7 — Source data Fig. 6 [file 44319_2025_493_MOESM7_ESM.zip › Figure6/Fig6H/Experiment-69_wt_cholinergic.tif_files/Experiment-69_z6c0x0-1388y0-1040.tif]

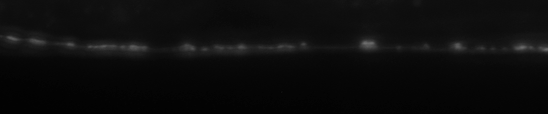

Supplement: Supplementary file 7 — Source data Fig. 6 [file 44319_2025_493_MOESM7_ESM.zip › Figure6/Fig6H/Experiment-74_OEskipped_cholinergic.tif_files/processed/skippedOE1.tif]

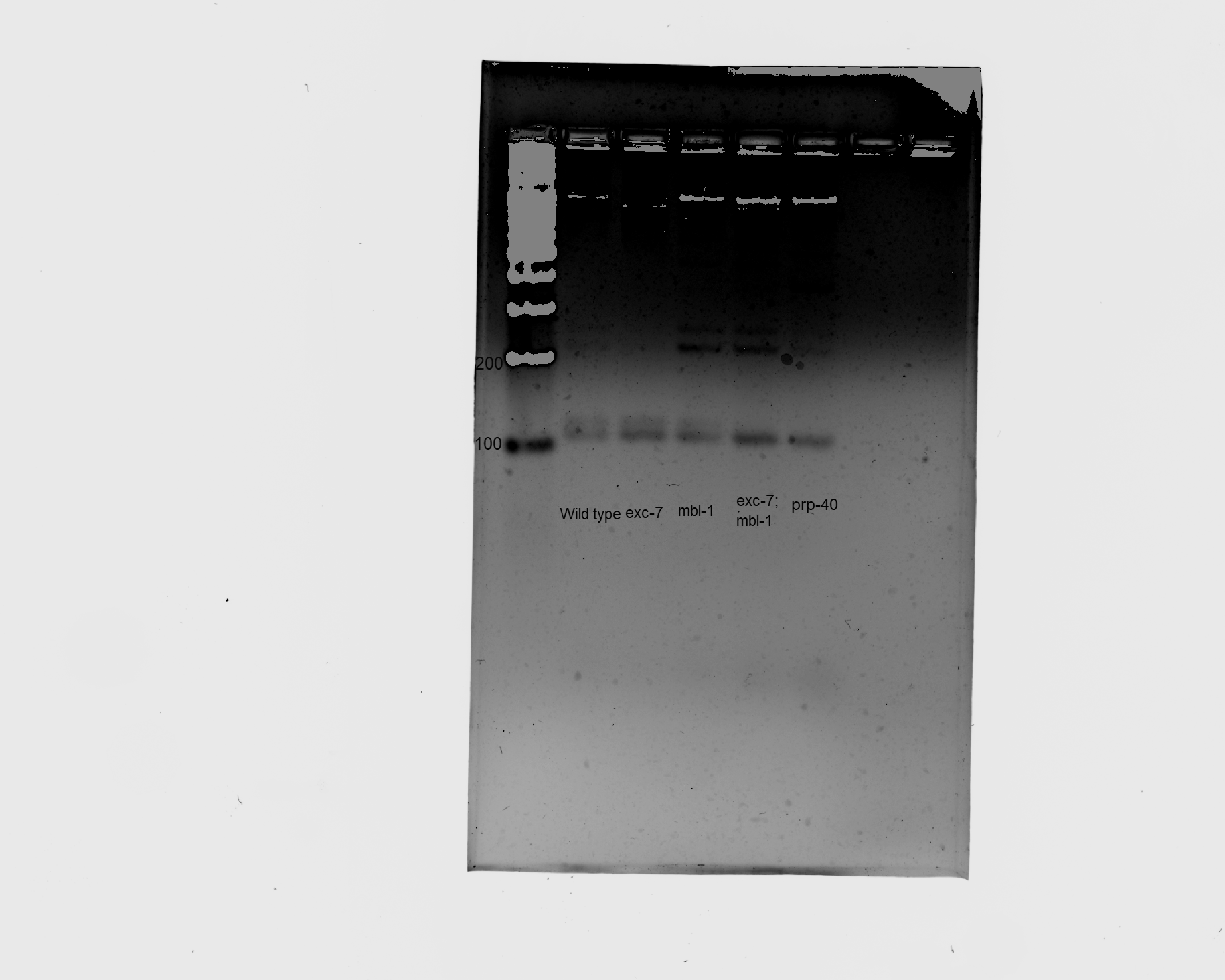

Supplement: Supplementary file 8 — Source data Fig. 7 [file 44319_2025_493_MOESM8_ESM.zip › Figure7/Fig7F/unc-31RTPCRb.tif]

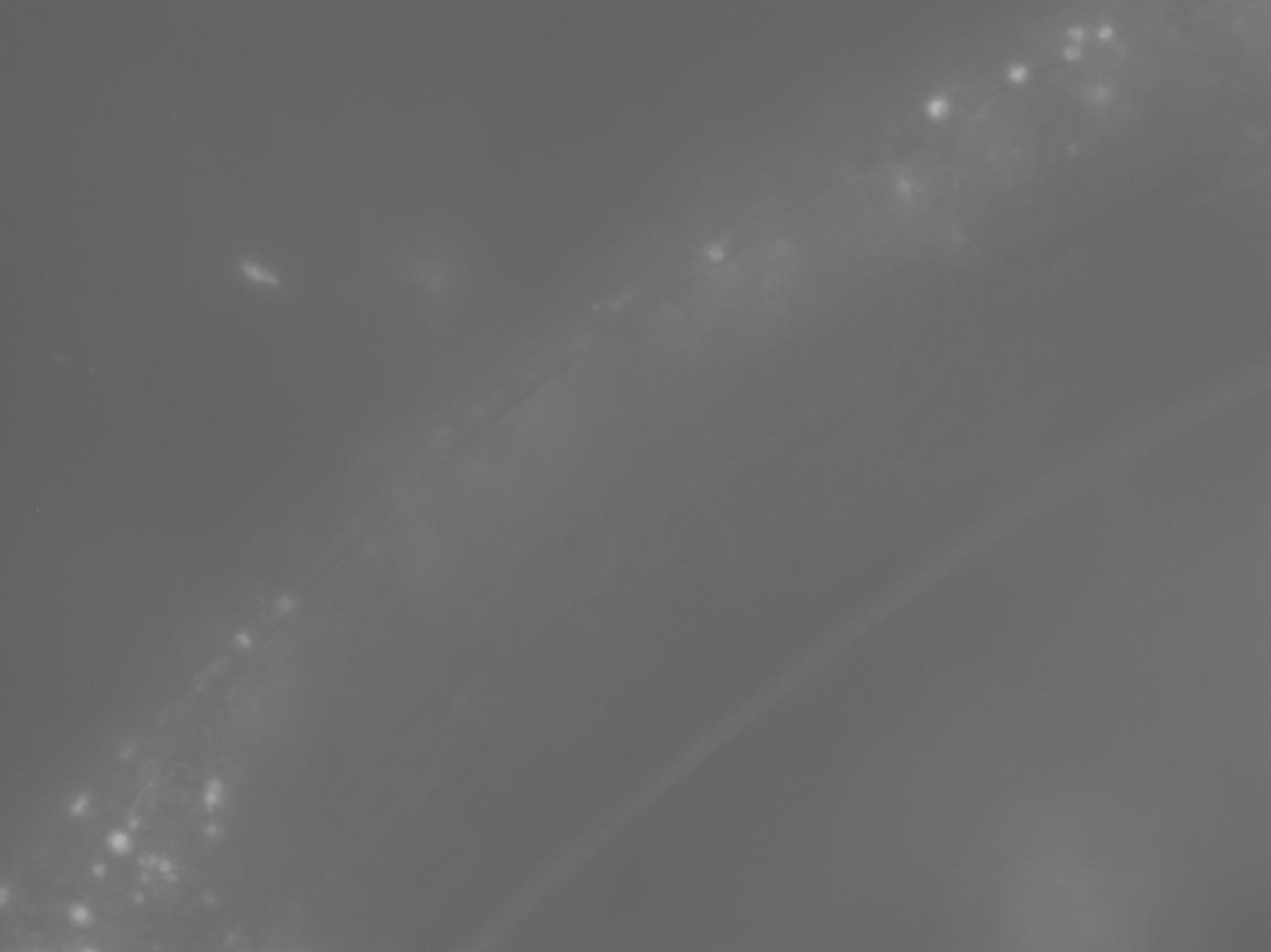

Supplement: Supplementary file 8 — Source data Fig. 7 [file 44319_2025_493_MOESM8_ESM.zip › Figure7/Fig7C/Experiment-48F54G21_VC.tif_files/red.tif]

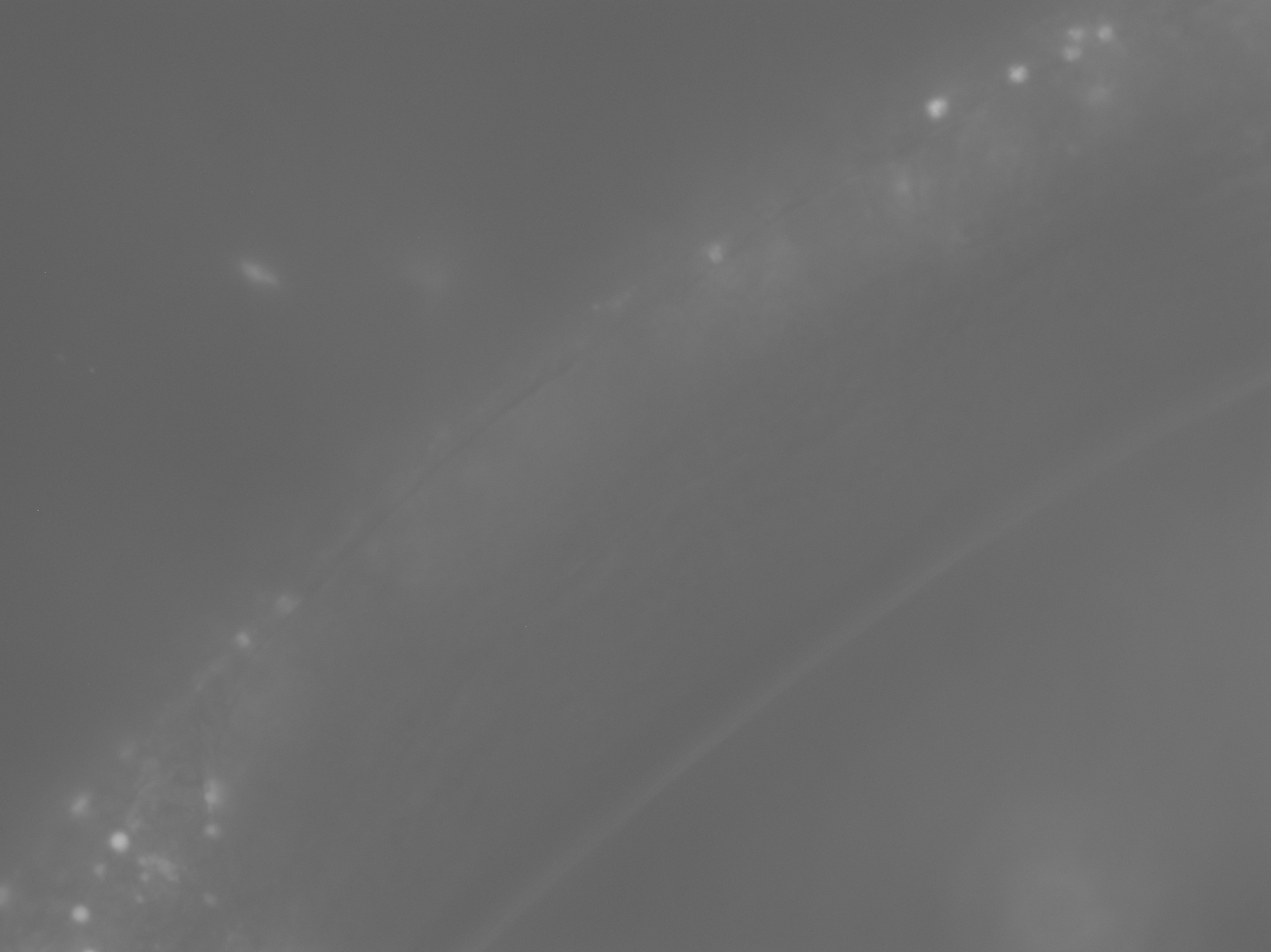

Supplement: Supplementary file 8 — Source data Fig. 7 [file 44319_2025_493_MOESM8_ESM.zip › Figure7/Fig7C/Experiment-48F54G21_VC.tif_files/Experiment-48_z5c1x0-1388y0-1040.tif]

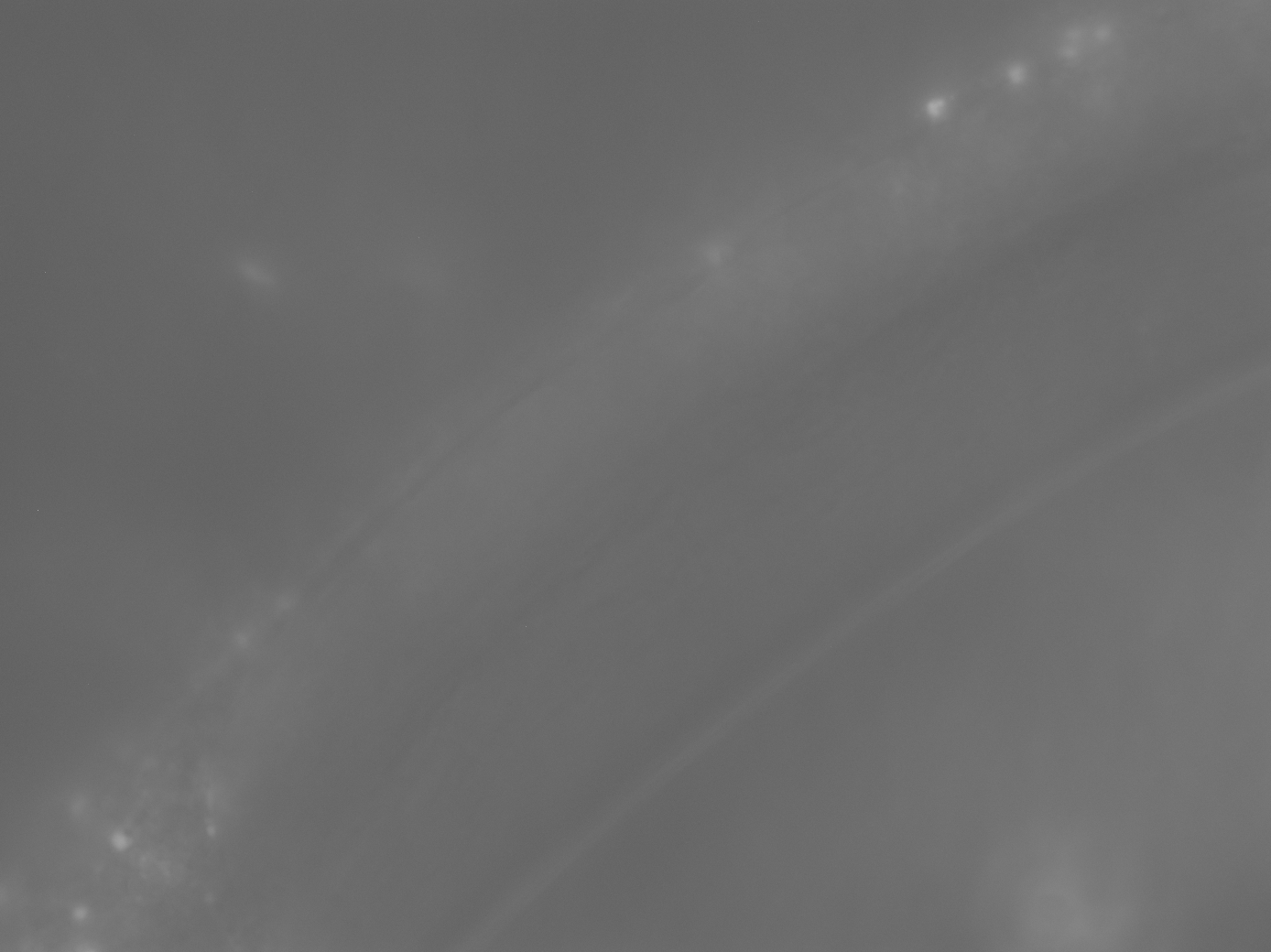

Supplement: Supplementary file 8 — Source data Fig. 7 [file 44319_2025_493_MOESM8_ESM.zip › Figure7/Fig7C/Experiment-48F54G21_VC.tif_files/Experiment-48_z3c1x0-1388y0-1040.tif]

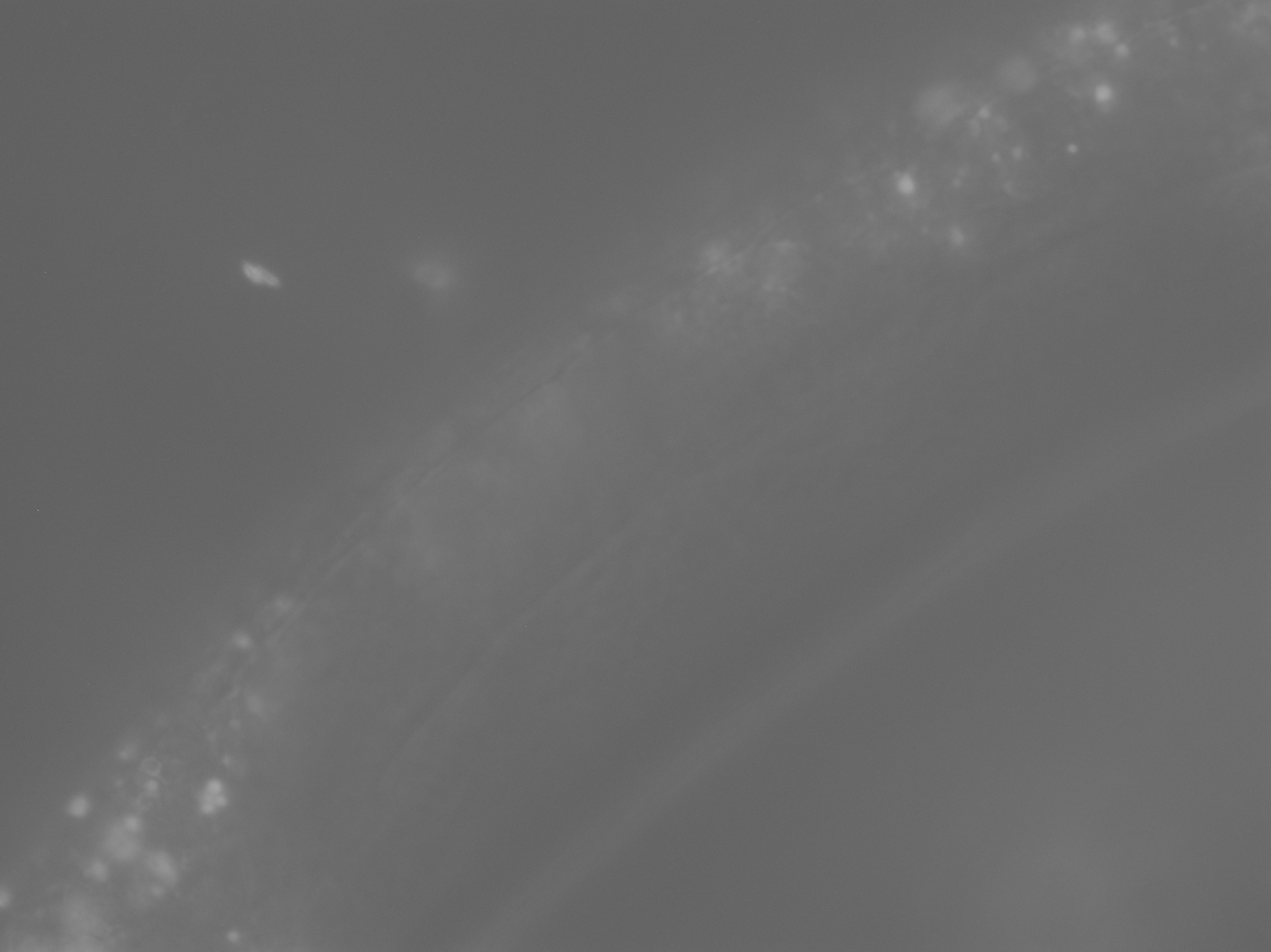

Supplement: Supplementary file 8 — Source data Fig. 7 [file 44319_2025_493_MOESM8_ESM.zip › Figure7/Fig7C/Experiment-48F54G21_VC.tif_files/Experiment-48_z8c1x0-1388y0-1040.tif]

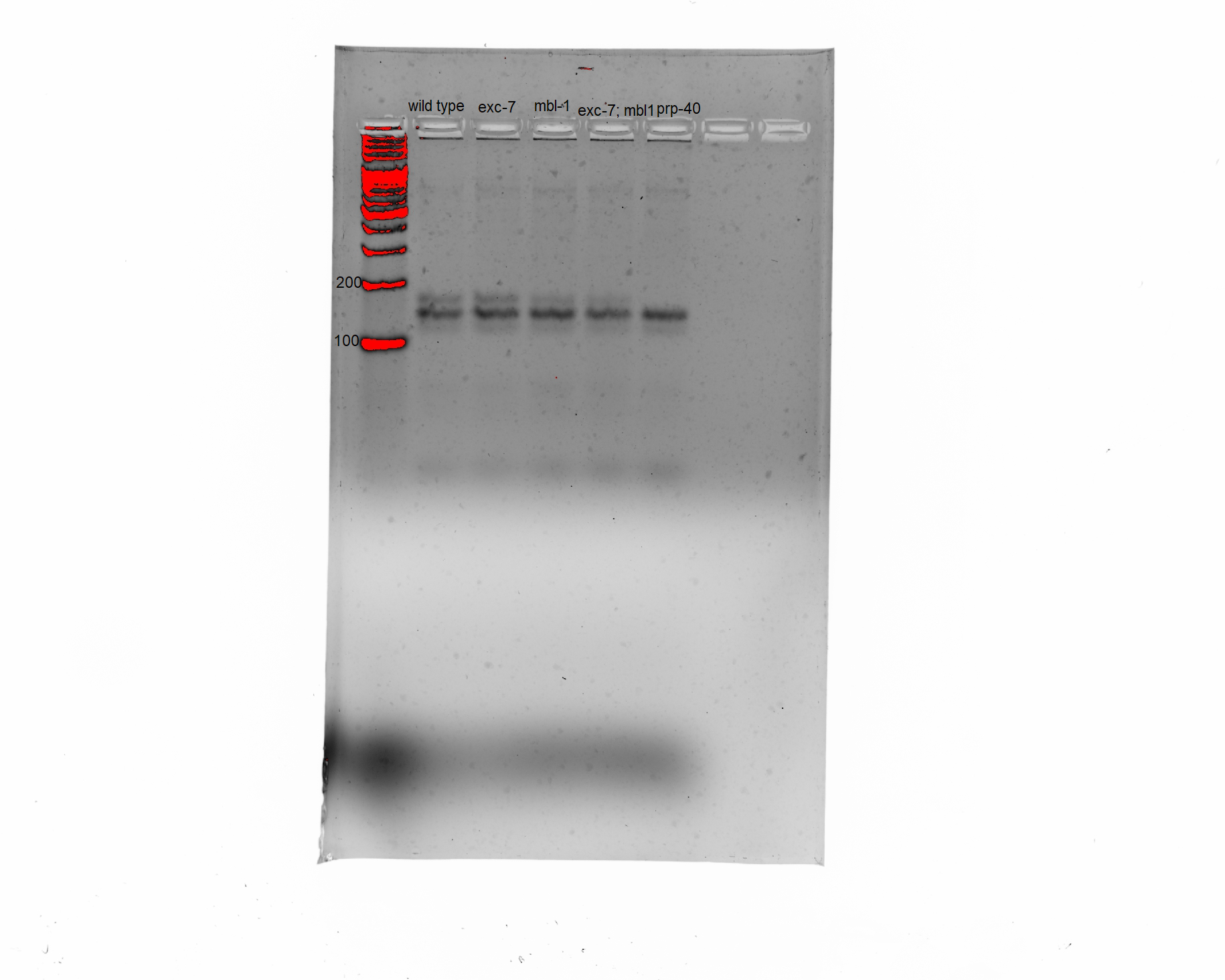

Supplement: Supplementary file 8 — Source data Fig. 7 [file 44319_2025_493_MOESM8_ESM.zip › Figure7/Fig7F/RTPCR_F54G21.tif]

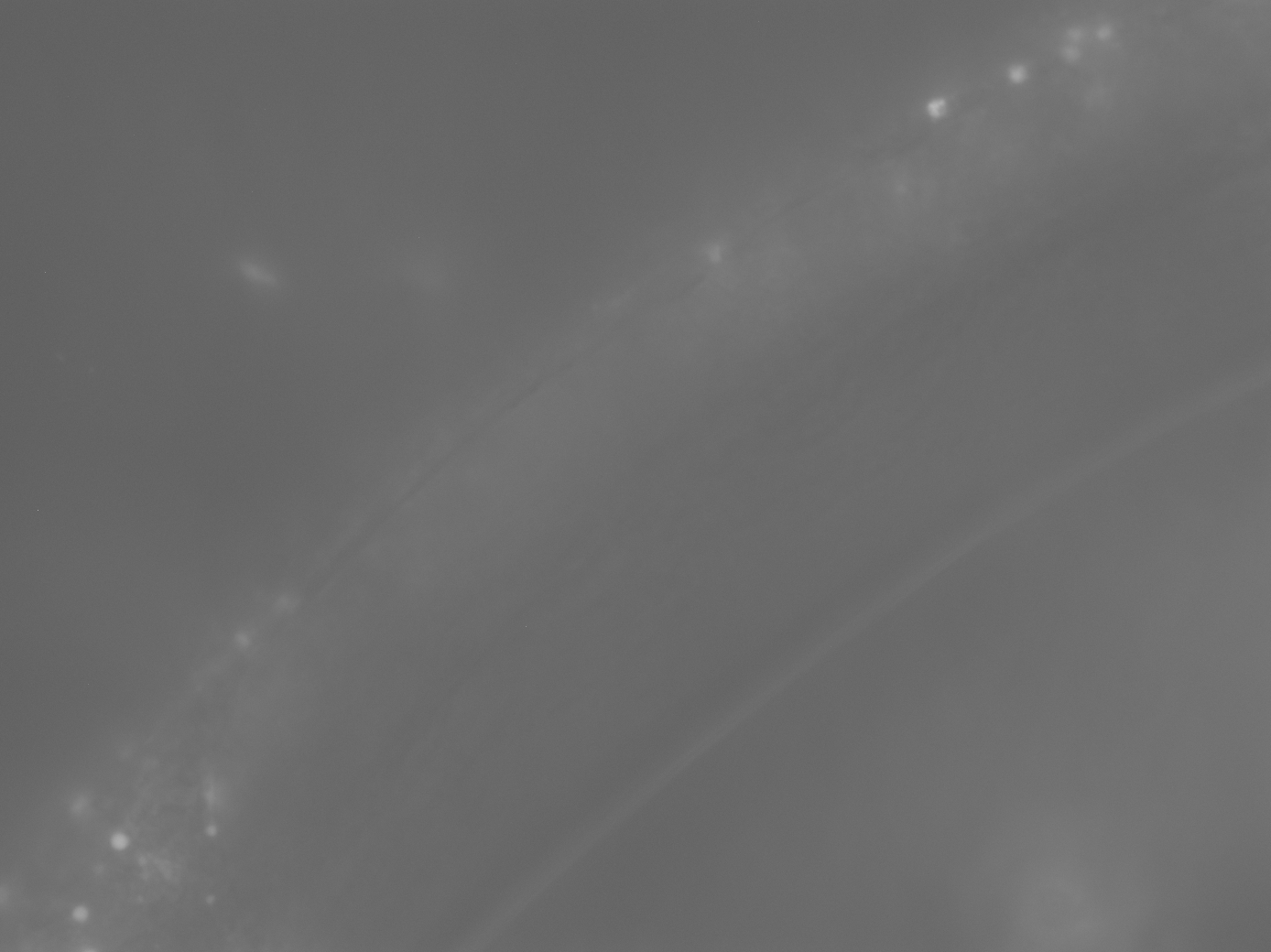

Supplement: Supplementary file 8 — Source data Fig. 7 [file 44319_2025_493_MOESM8_ESM.zip › Figure7/Fig7C/Experiment-48F54G21_VC.tif_files/Experiment-48_z4c1x0-1388y0-1040.tif]

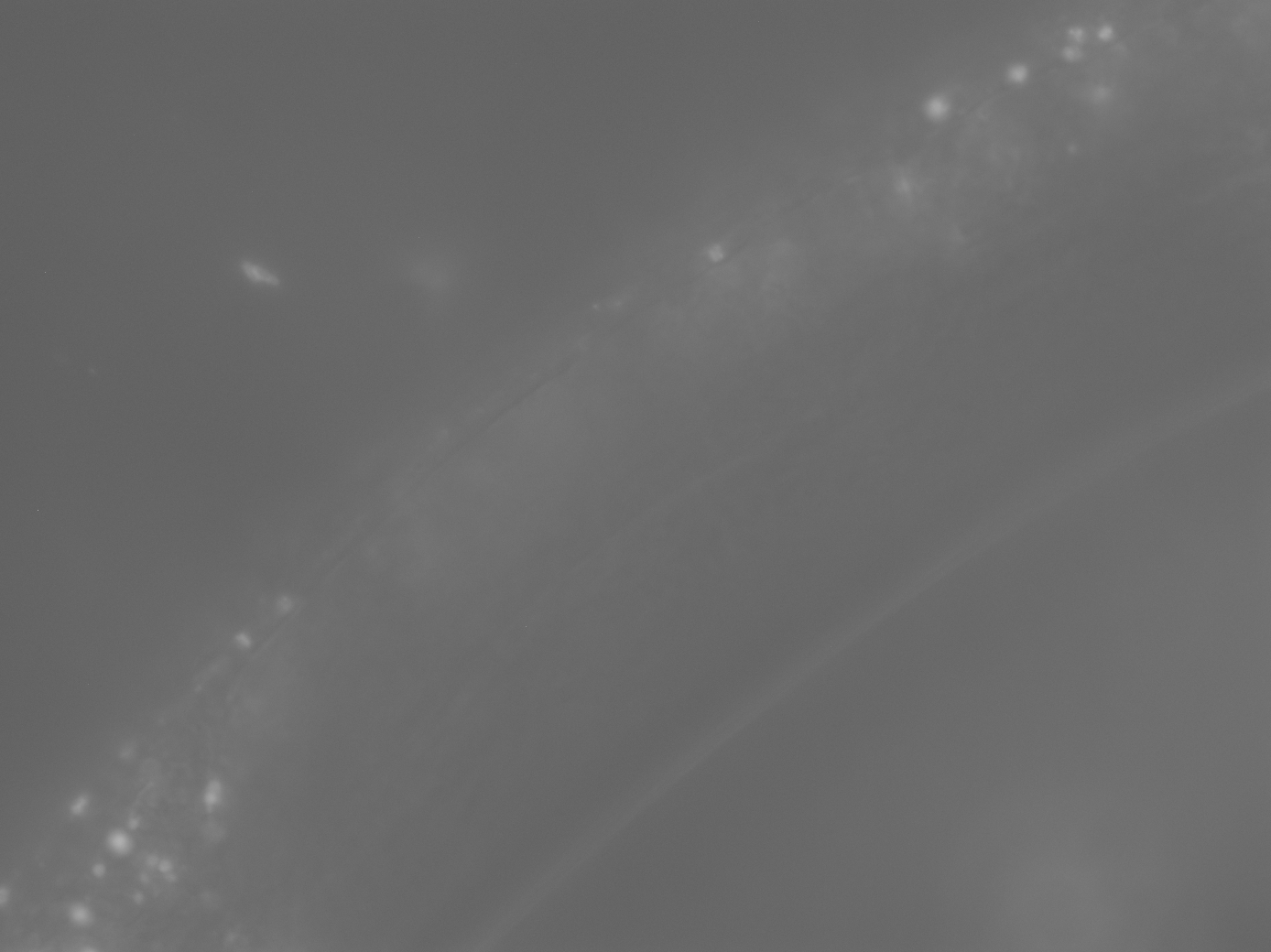

Supplement: Supplementary file 8 — Source data Fig. 7 [file 44319_2025_493_MOESM8_ESM.zip › Figure7/Fig7C/Experiment-48F54G21_VC.tif_files/Experiment-48_z6c1x0-1388y0-1040.tif]

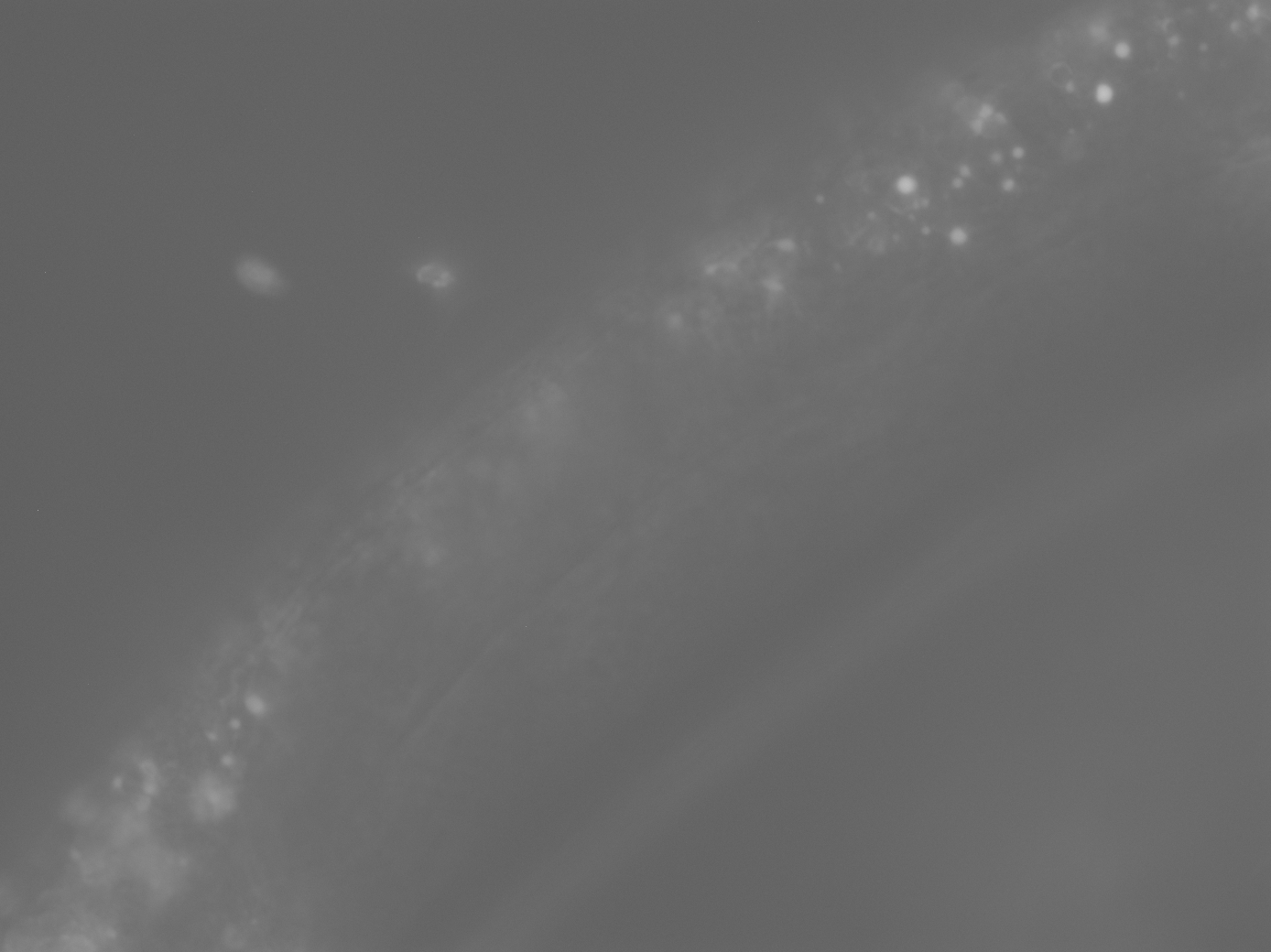

Supplement: Supplementary file 8 — Source data Fig. 7 [file 44319_2025_493_MOESM8_ESM.zip › Figure7/Fig7C/Experiment-48F54G21_VC.tif_files/Experiment-48_z10c1x0-1388y0-1040.tif]

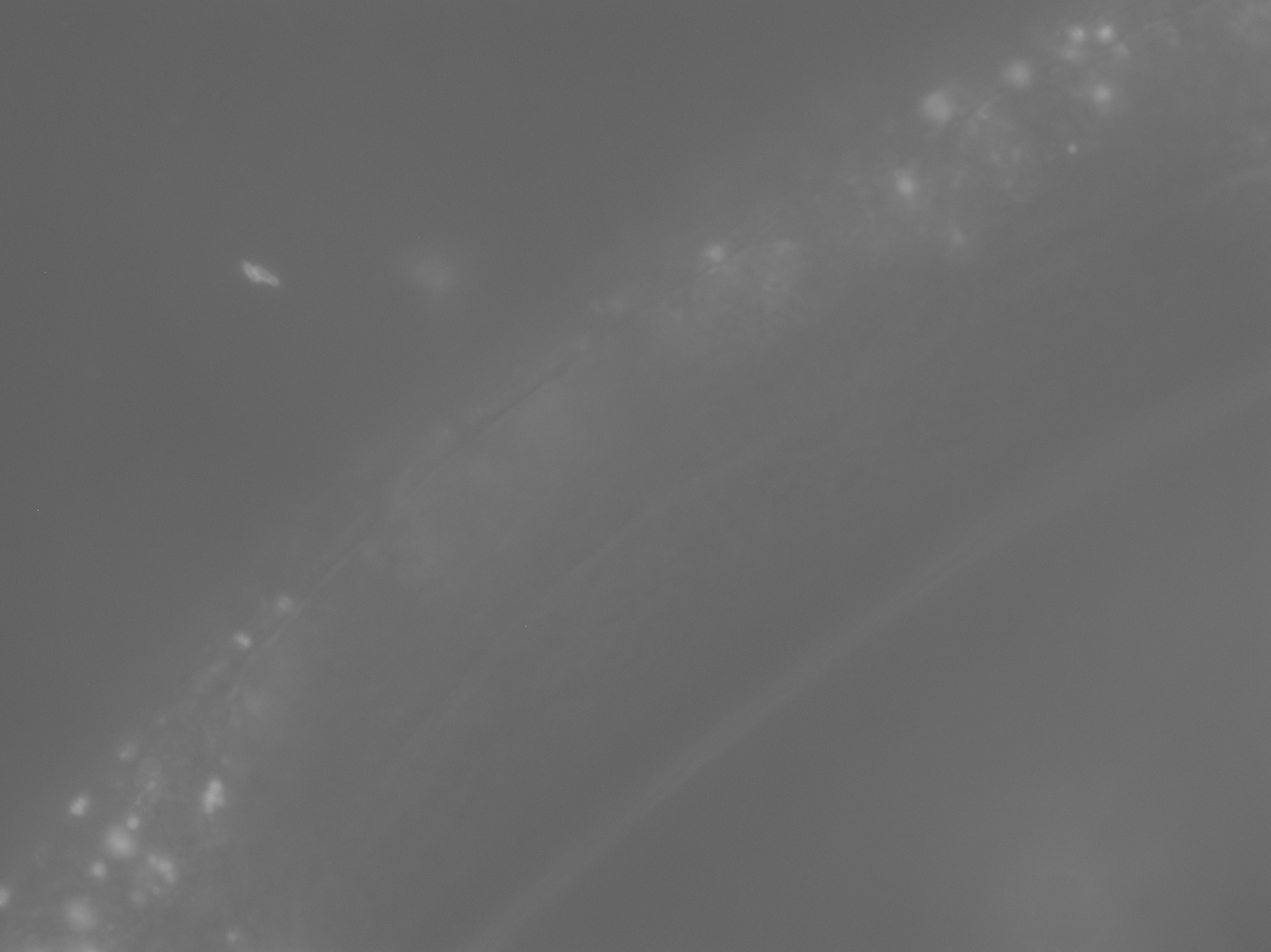

Supplement: Supplementary file 8 — Source data Fig. 7 [file 44319_2025_493_MOESM8_ESM.zip › Figure7/Fig7C/Experiment-48F54G21_VC.tif_files/Experiment-48_z7c1x0-1388y0-1040.tif]

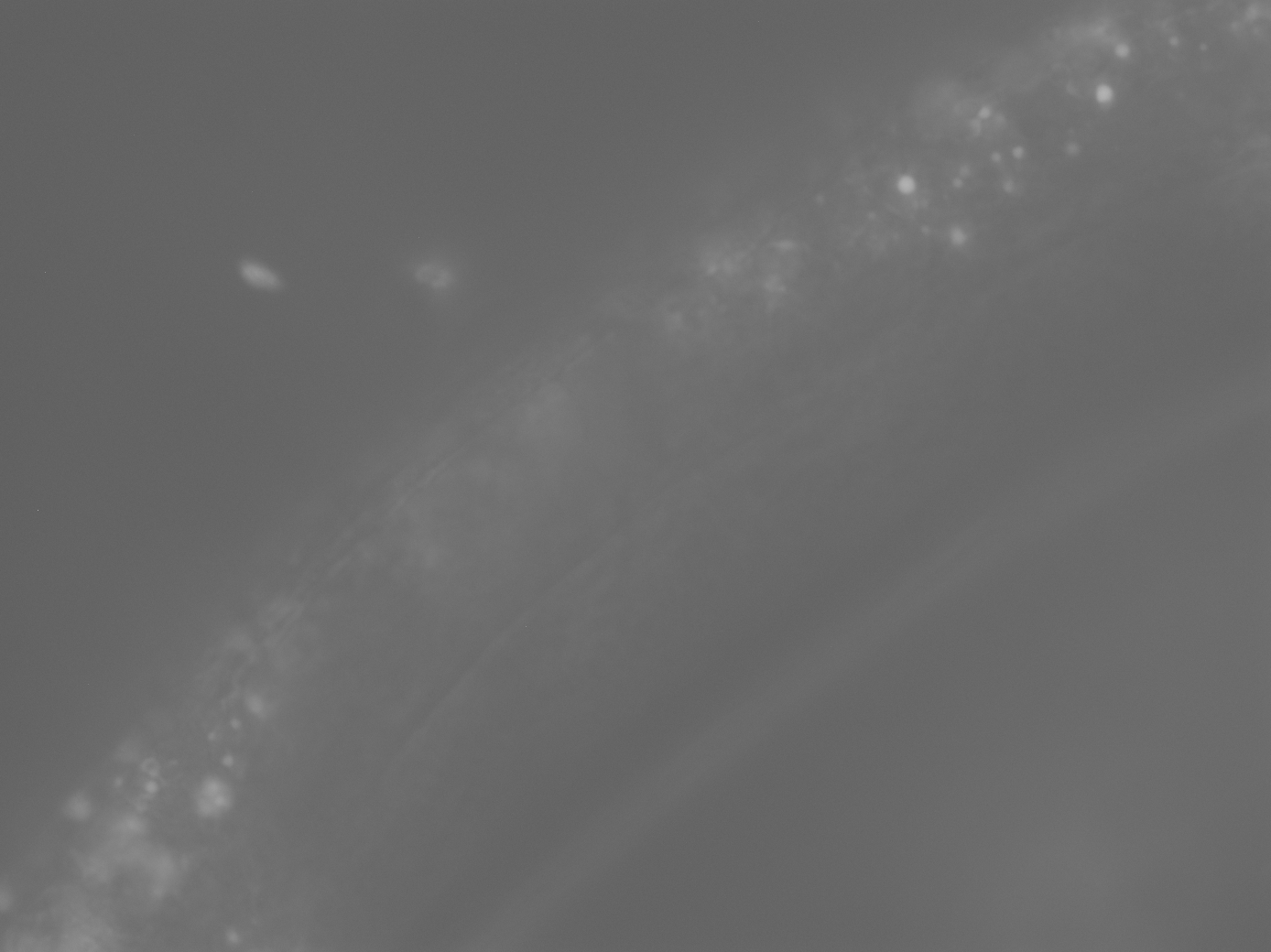

Supplement: Supplementary file 8 — Source data Fig. 7 [file 44319_2025_493_MOESM8_ESM.zip › Figure7/Fig7C/Experiment-48F54G21_VC.tif_files/Experiment-48_z9c1x0-1388y0-1040.tif]

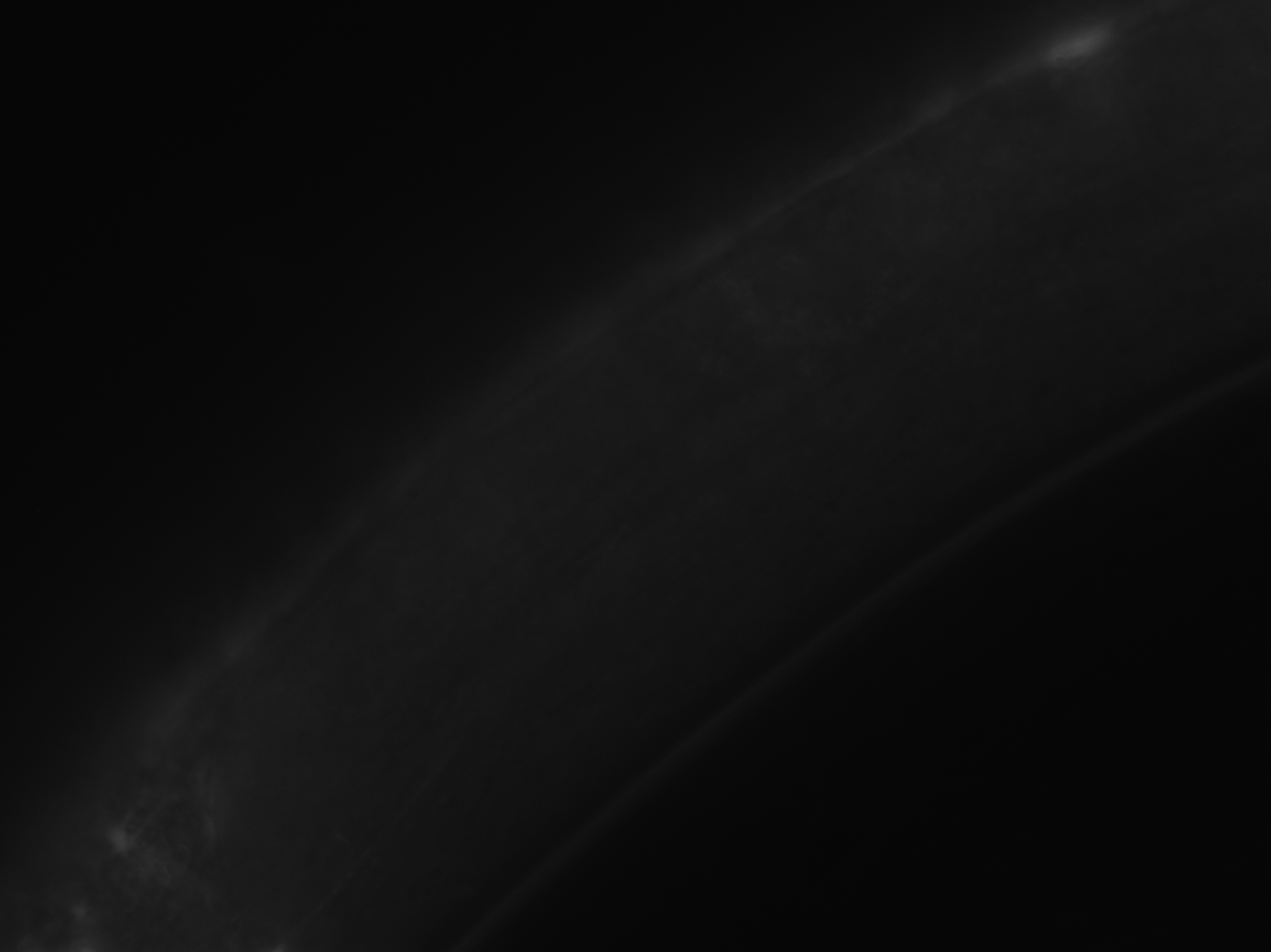

Supplement: Supplementary file 8 — Source data Fig. 7 [file 44319_2025_493_MOESM8_ESM.zip › Figure7/Fig7C/Experiment-48F54G21_VC.tif_files/Experiment-48_z2c0x0-1388y0-1040.tif]

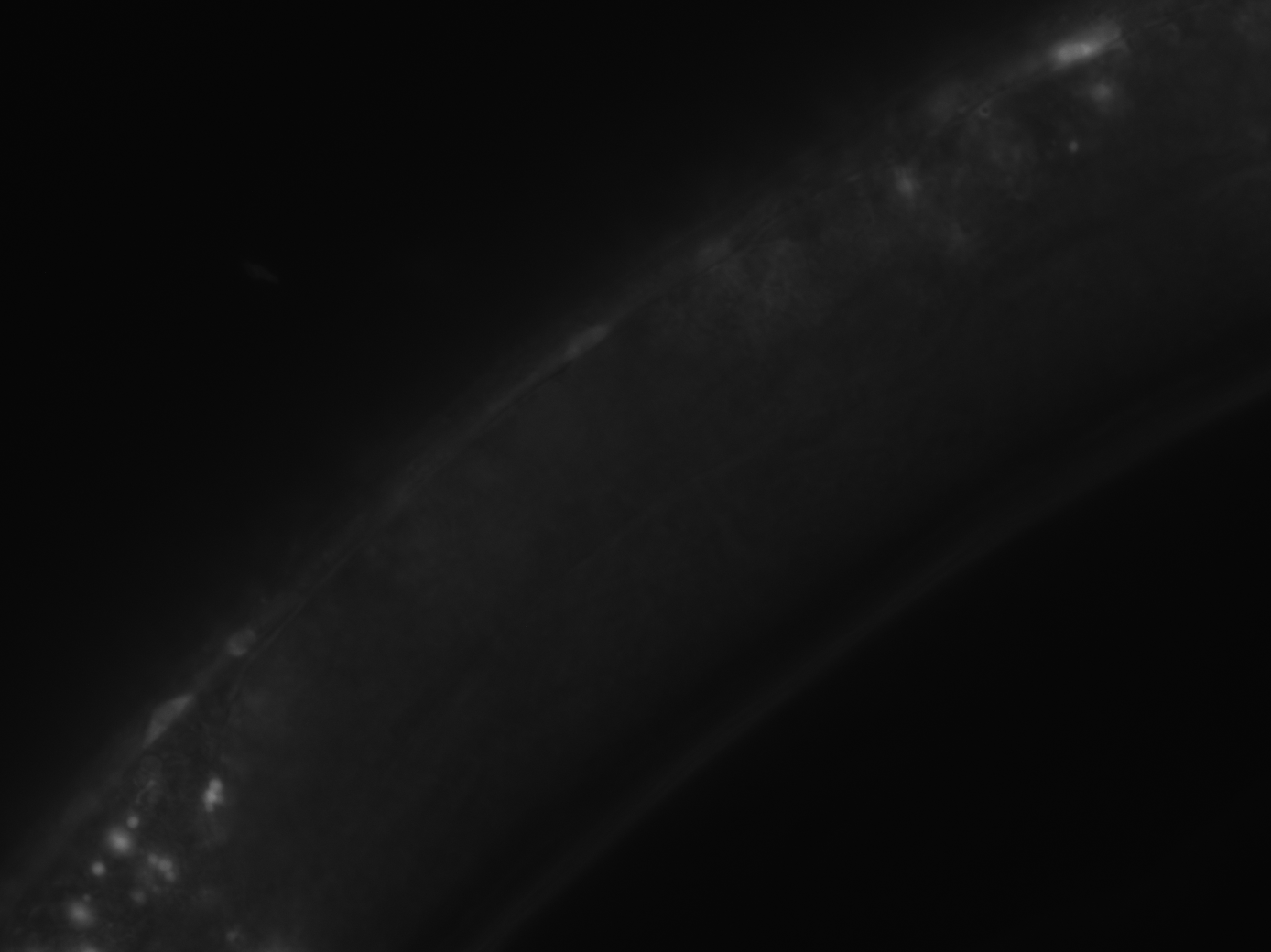

Supplement: Supplementary file 8 — Source data Fig. 7 [file 44319_2025_493_MOESM8_ESM.zip › Figure7/Fig7C/Experiment-48F54G21_VC.tif_files/Experiment-48_z7c0x0-1388y0-1040.tif]

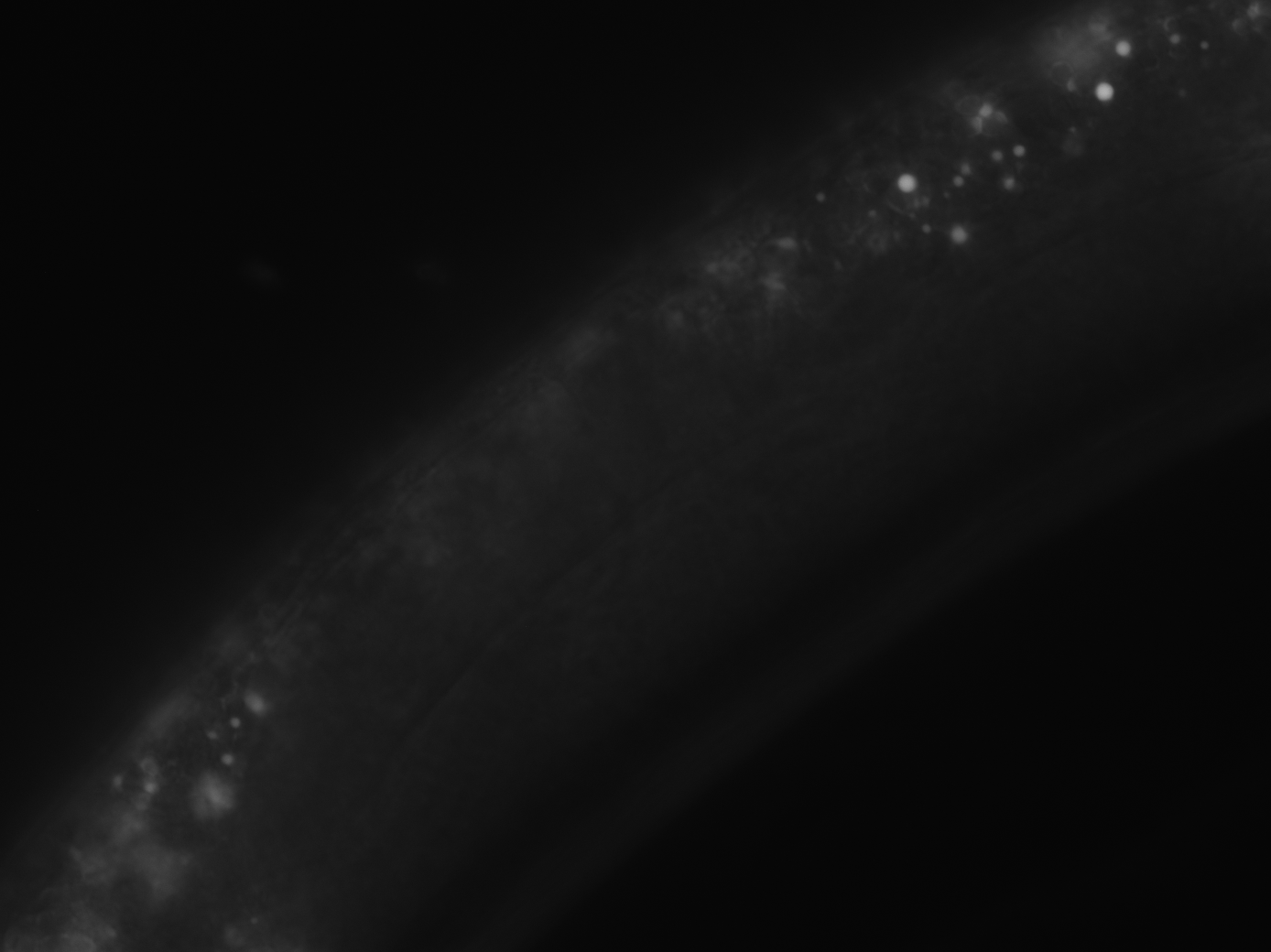

Supplement: Supplementary file 8 — Source data Fig. 7 [file 44319_2025_493_MOESM8_ESM.zip › Figure7/Fig7C/Experiment-48F54G21_VC.tif_files/Experiment-48_z10c0x0-1388y0-1040.tif]

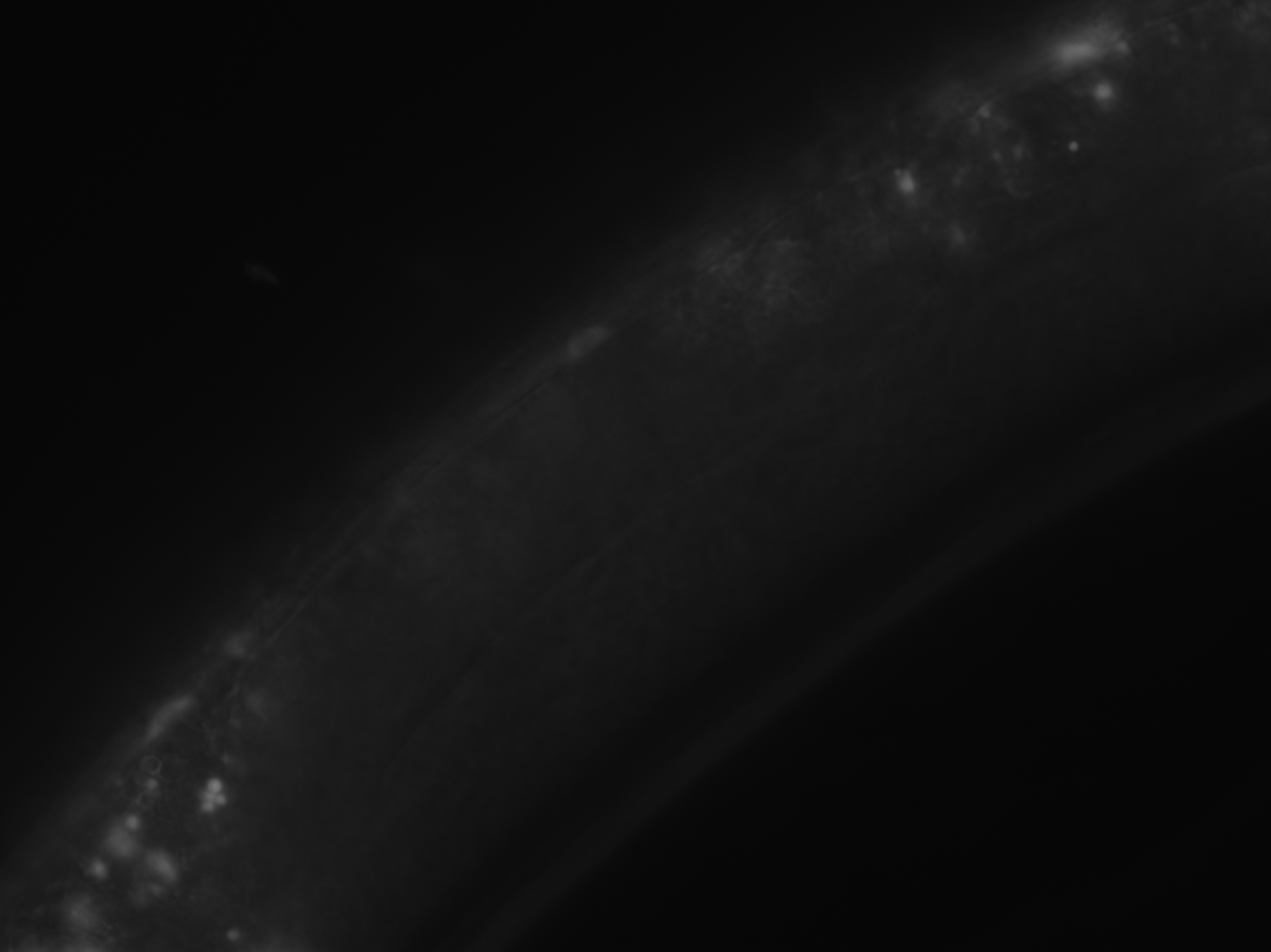

Supplement: Supplementary file 8 — Source data Fig. 7 [file 44319_2025_493_MOESM8_ESM.zip › Figure7/Fig7C/Experiment-48F54G21_VC.tif_files/Experiment-48_z8c0x0-1388y0-1040.tif]

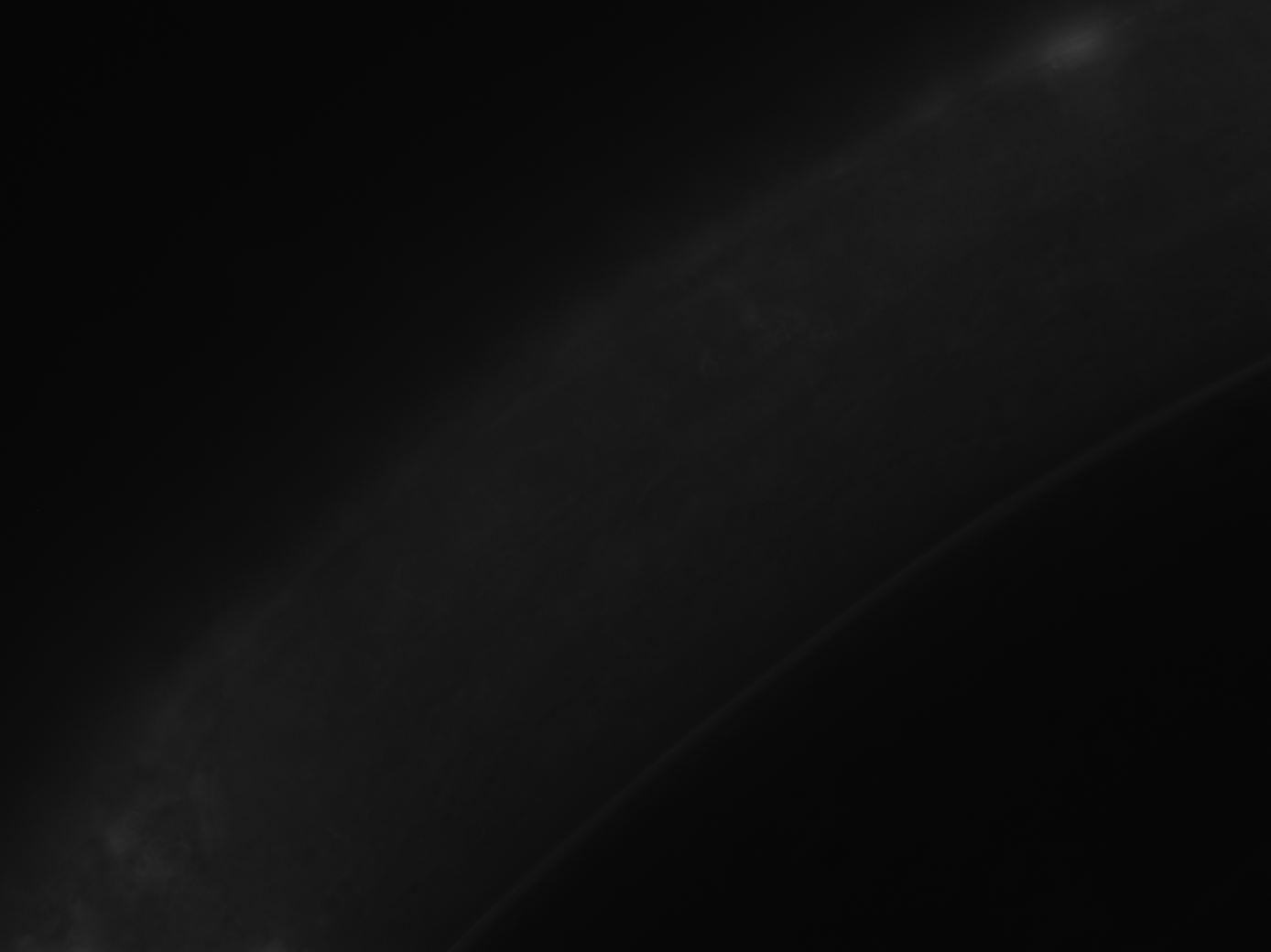

Supplement: Supplementary file 8 — Source data Fig. 7 [file 44319_2025_493_MOESM8_ESM.zip › Figure7/Fig7C/Experiment-48F54G21_VC.tif_files/Experiment-48_z0c0x0-1388y0-1040.tif]

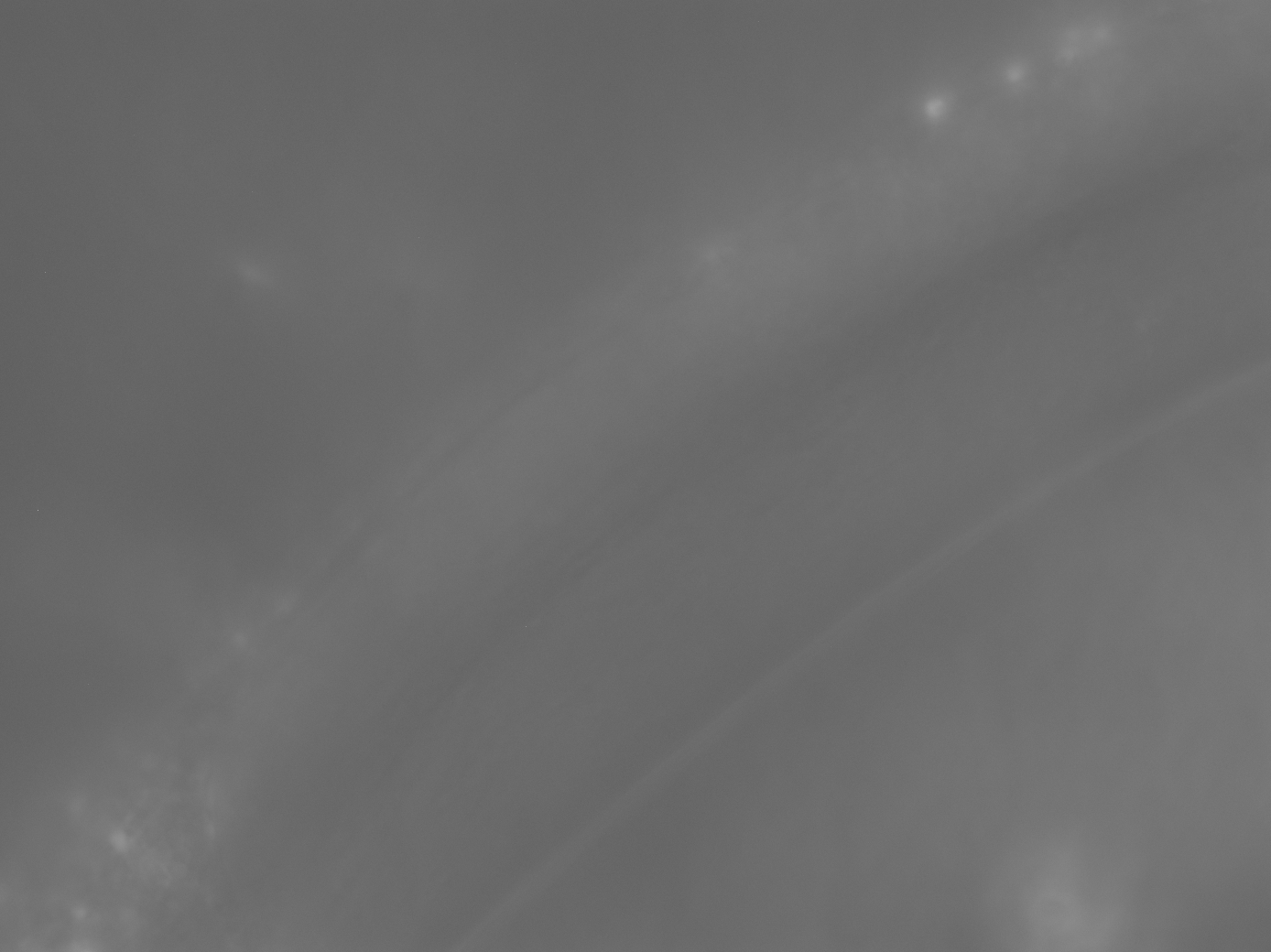

Supplement: Supplementary file 8 — Source data Fig. 7 [file 44319_2025_493_MOESM8_ESM.zip › Figure7/Fig7C/Experiment-48F54G21_VC.tif_files/Experiment-48_z2c1x0-1388y0-1040.tif]

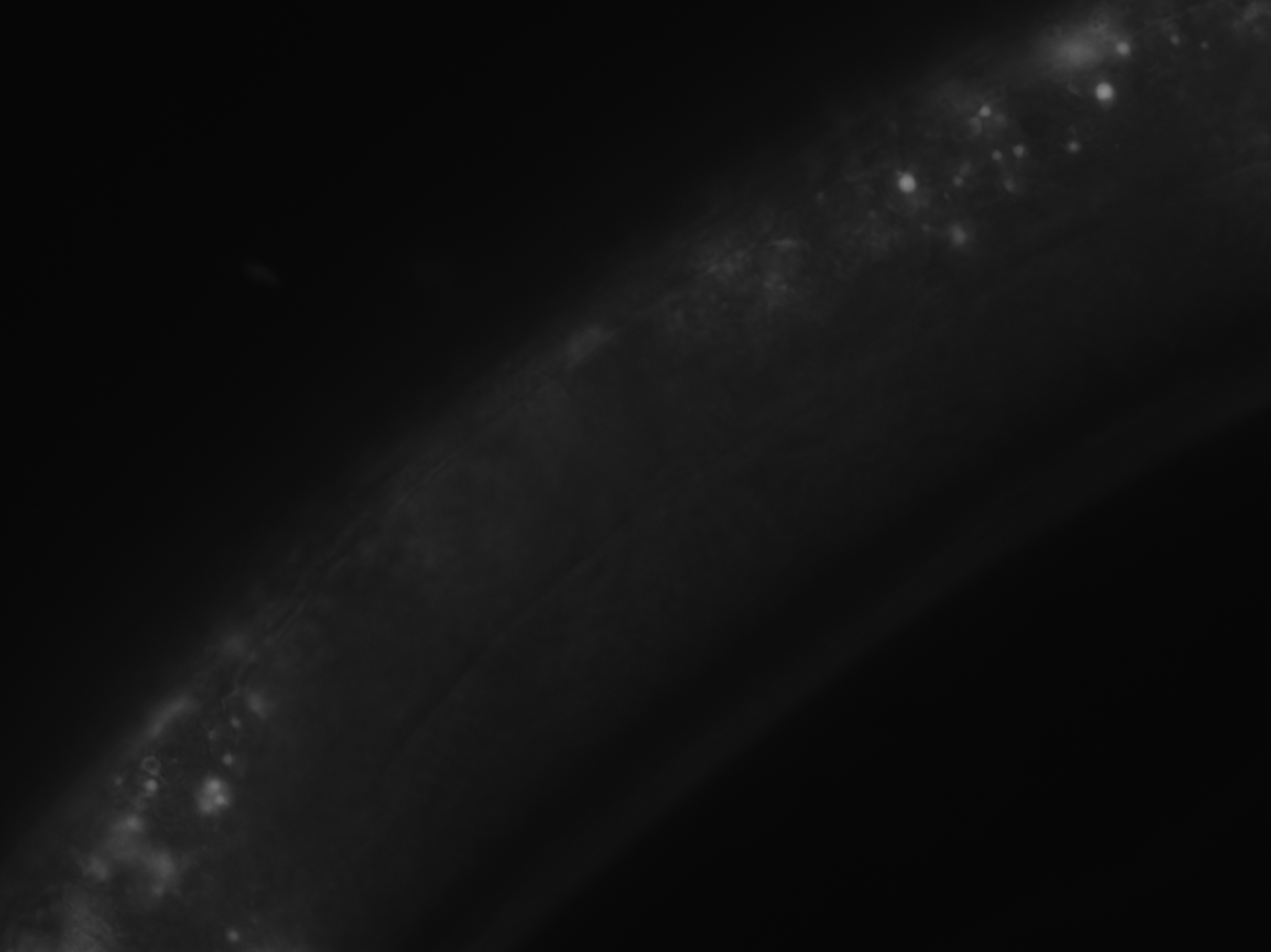

Supplement: Supplementary file 8 — Source data Fig. 7 [file 44319_2025_493_MOESM8_ESM.zip › Figure7/Fig7C/Experiment-48F54G21_VC.tif_files/Experiment-48_z9c0x0-1388y0-1040.tif]

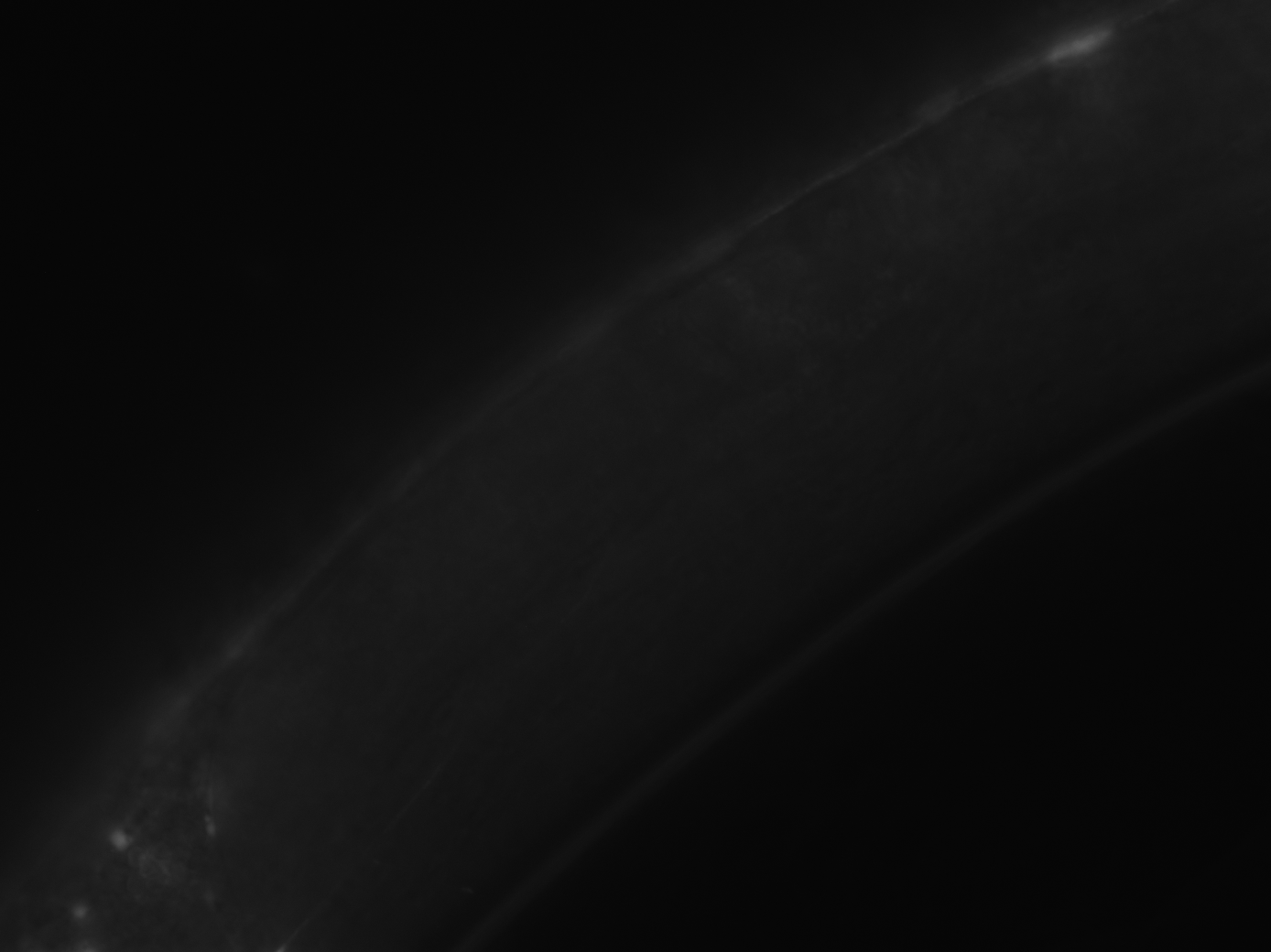

Supplement: Supplementary file 8 — Source data Fig. 7 [file 44319_2025_493_MOESM8_ESM.zip › Figure7/Fig7C/Experiment-48F54G21_VC.tif_files/Experiment-48_z3c0x0-1388y0-1040.tif]

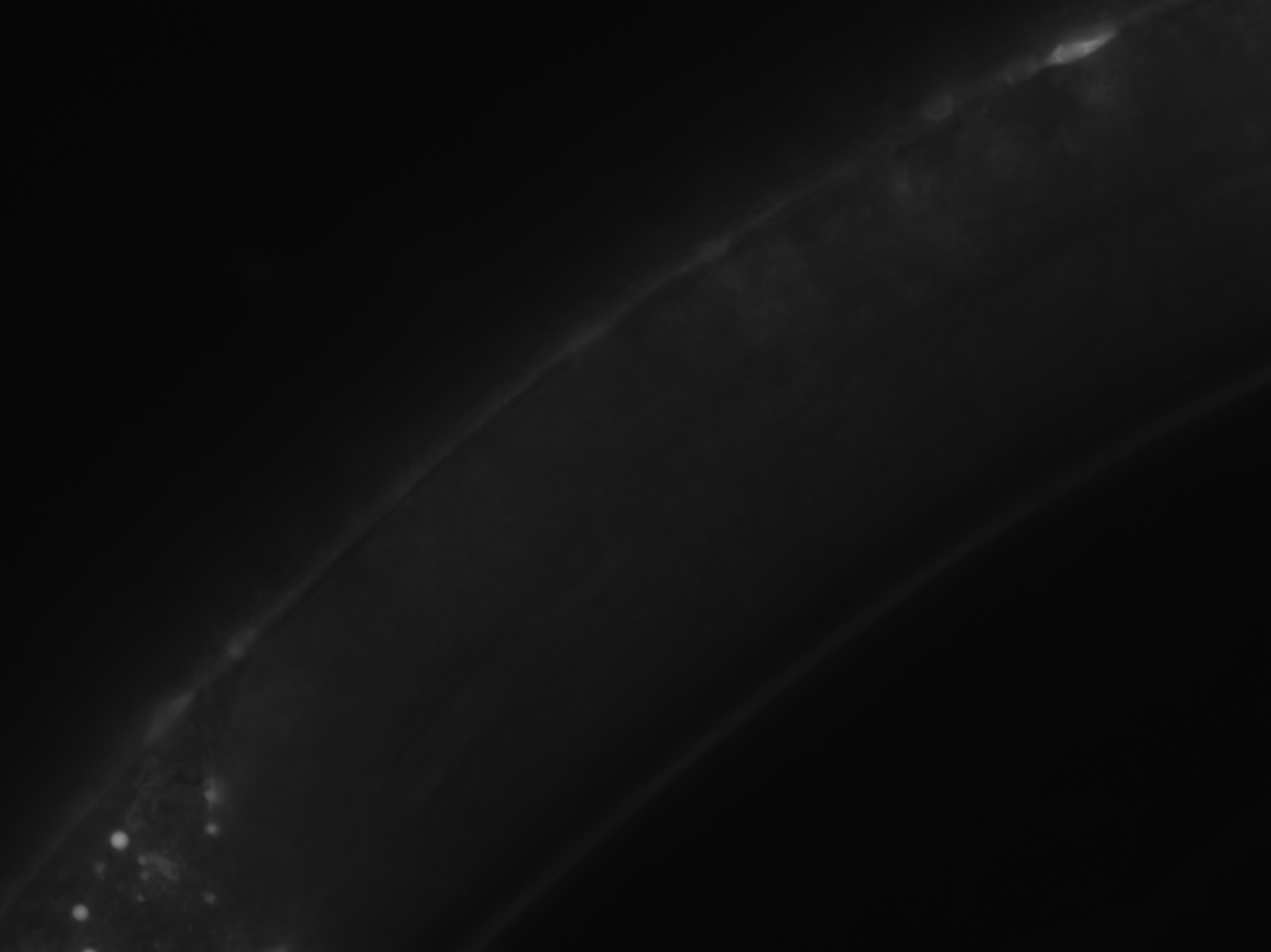

Supplement: Supplementary file 8 — Source data Fig. 7 [file 44319_2025_493_MOESM8_ESM.zip › Figure7/Fig7C/Experiment-48F54G21_VC.tif_files/Experiment-48_z5c0x0-1388y0-1040.tif]

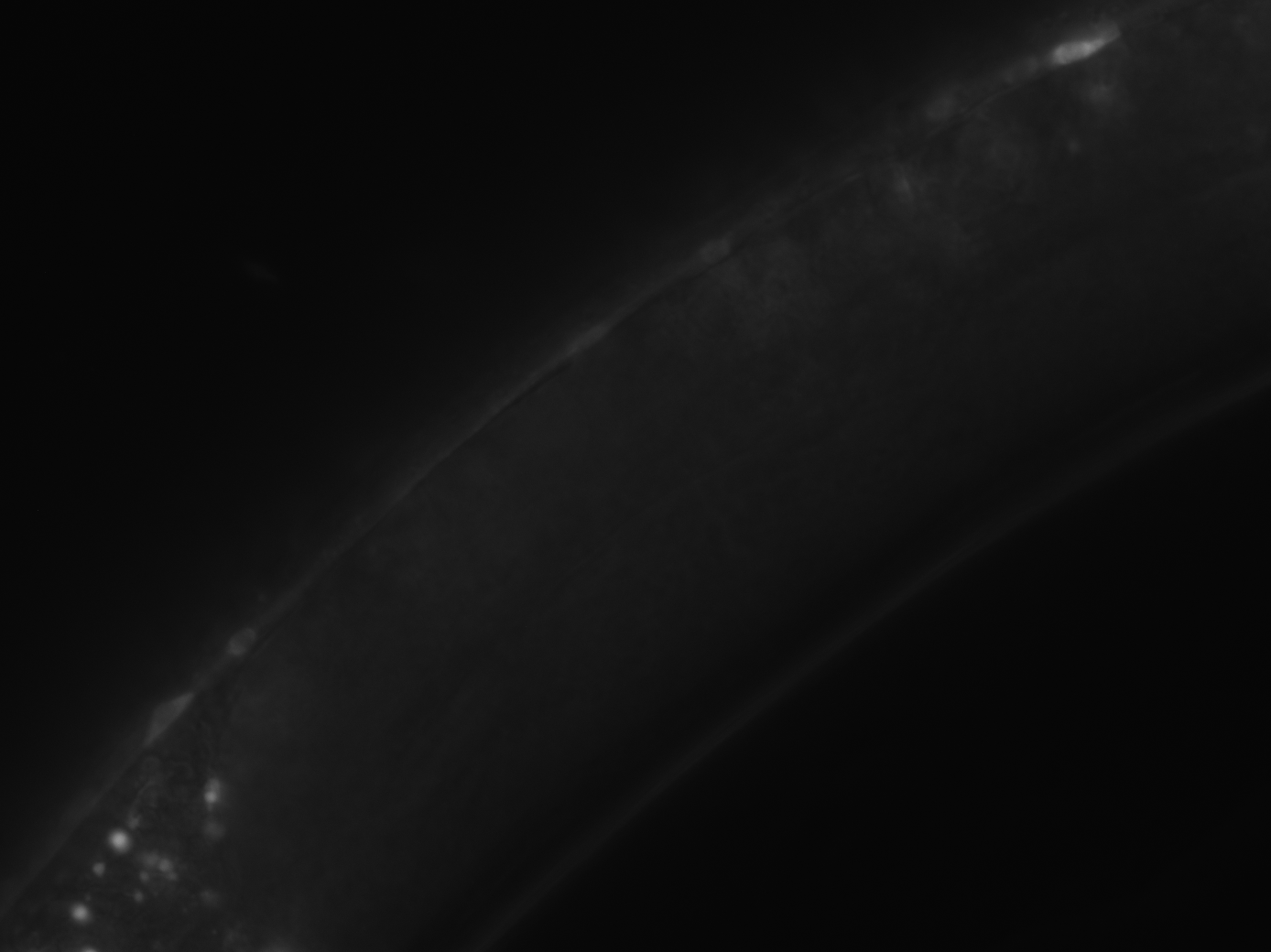

Supplement: Supplementary file 8 — Source data Fig. 7 [file 44319_2025_493_MOESM8_ESM.zip › Figure7/Fig7C/Experiment-48F54G21_VC.tif_files/Experiment-48_z6c0x0-1388y0-1040.tif]

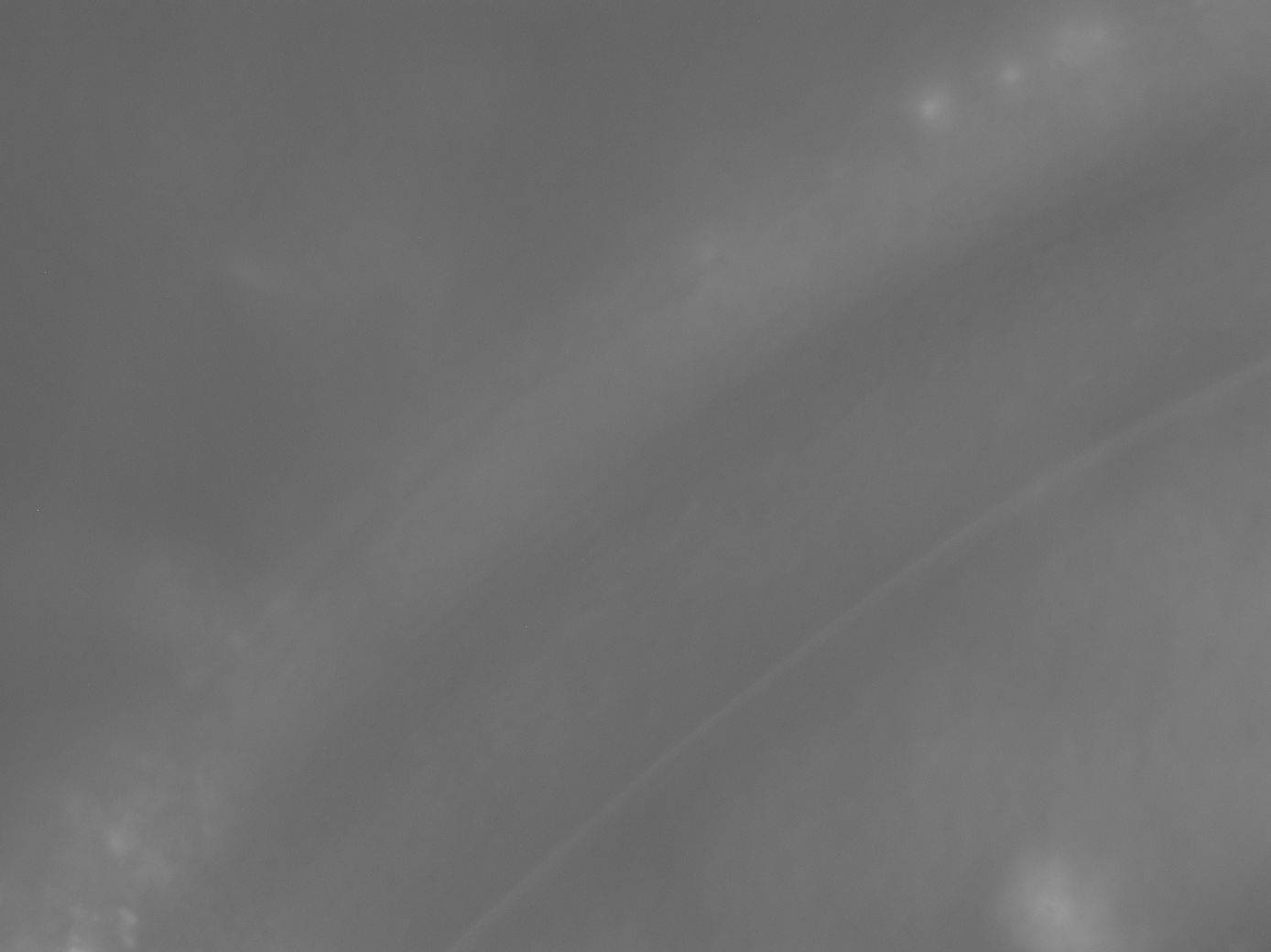

Supplement: Supplementary file 8 — Source data Fig. 7 [file 44319_2025_493_MOESM8_ESM.zip › Figure7/Fig7C/Experiment-48F54G21_VC.tif_files/Experiment-48_z0c1x0-1388y0-1040.tif]

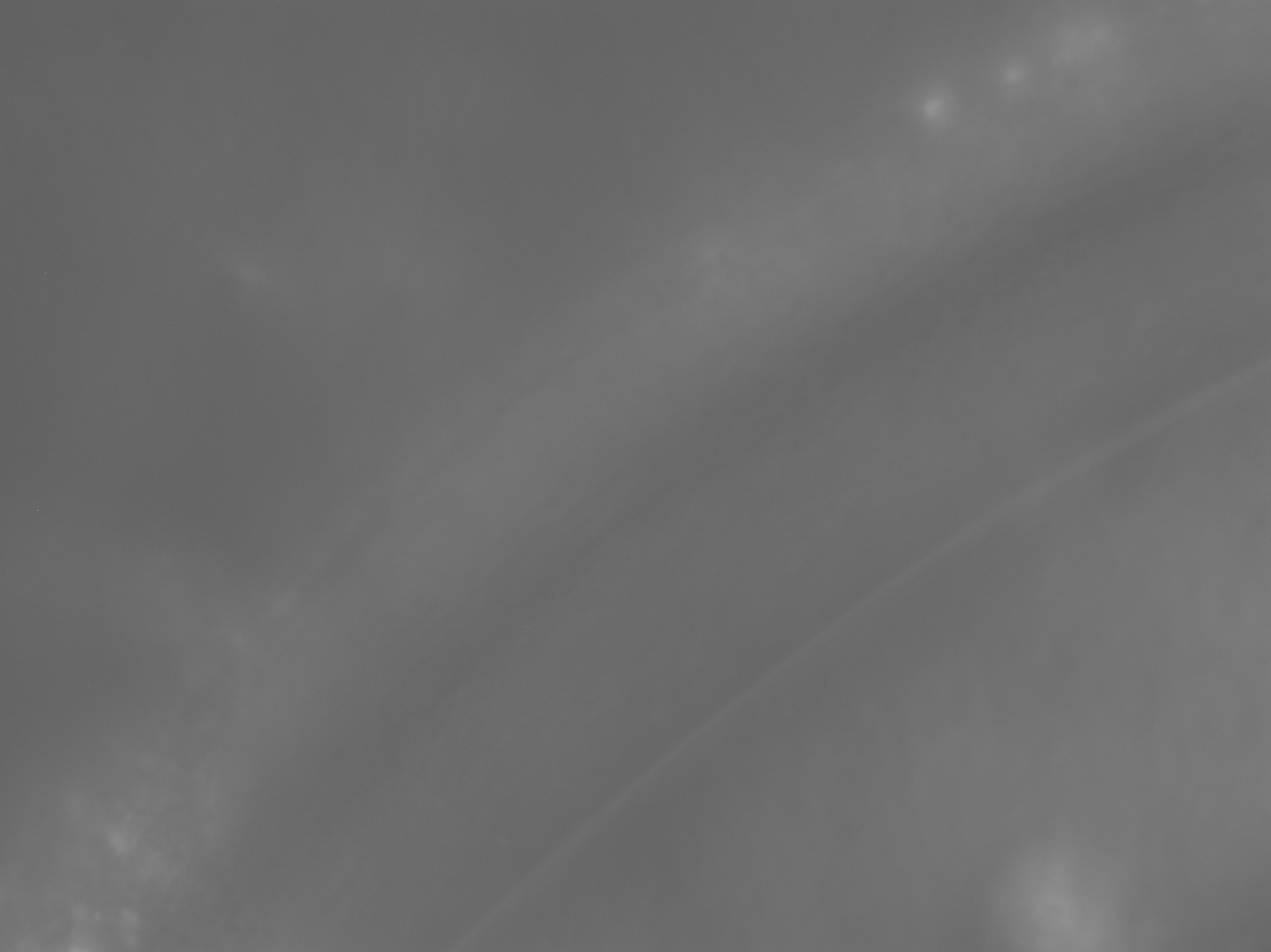

Supplement: Supplementary file 8 — Source data Fig. 7 [file 44319_2025_493_MOESM8_ESM.zip › Figure7/Fig7C/Experiment-48F54G21_VC.tif_files/Experiment-48_z1c1x0-1388y0-1040.tif]

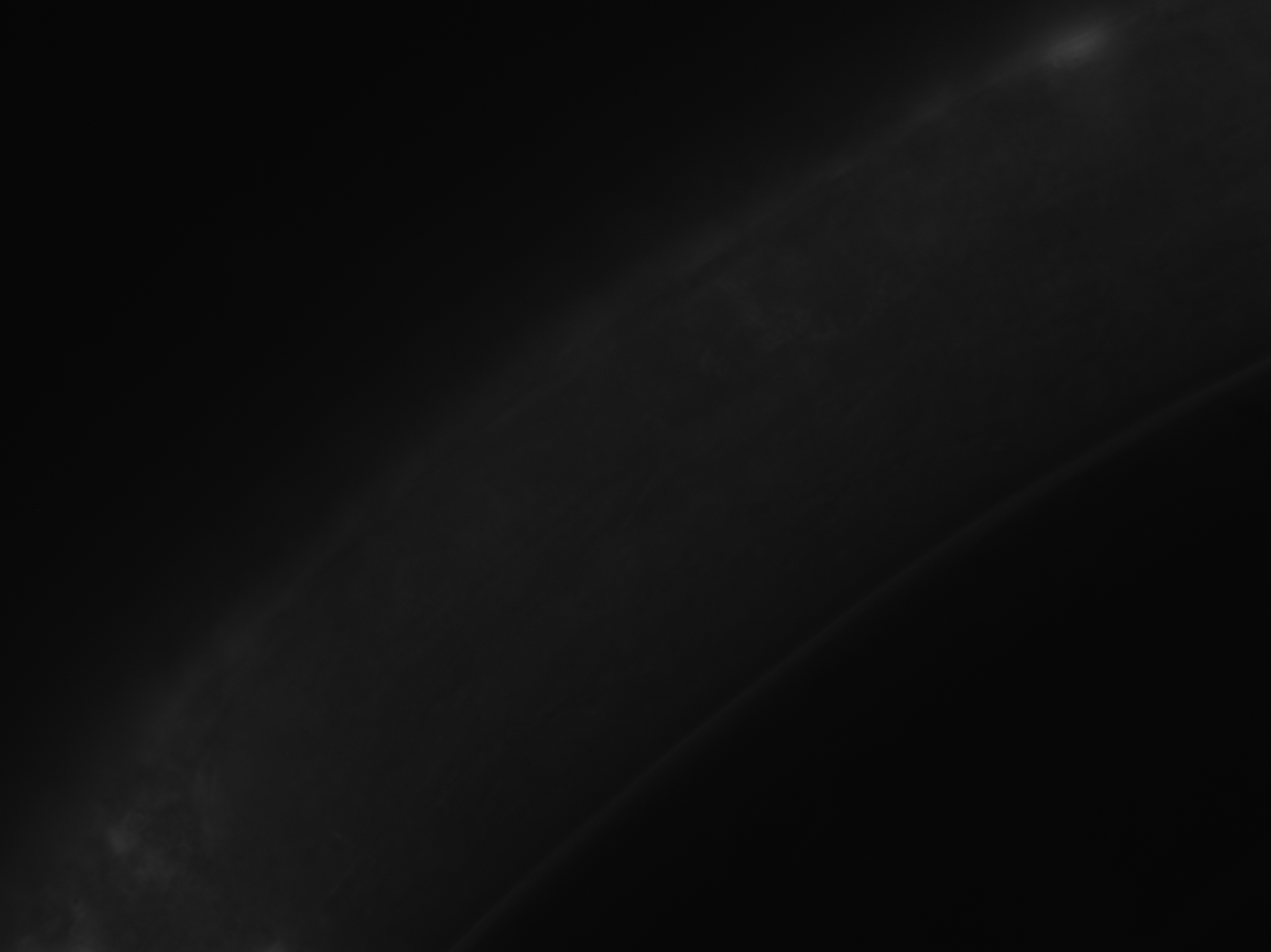

Supplement: Supplementary file 8 — Source data Fig. 7 [file 44319_2025_493_MOESM8_ESM.zip › Figure7/Fig7C/Experiment-48F54G21_VC.tif_files/Experiment-48_z1c0x0-1388y0-1040.tif]

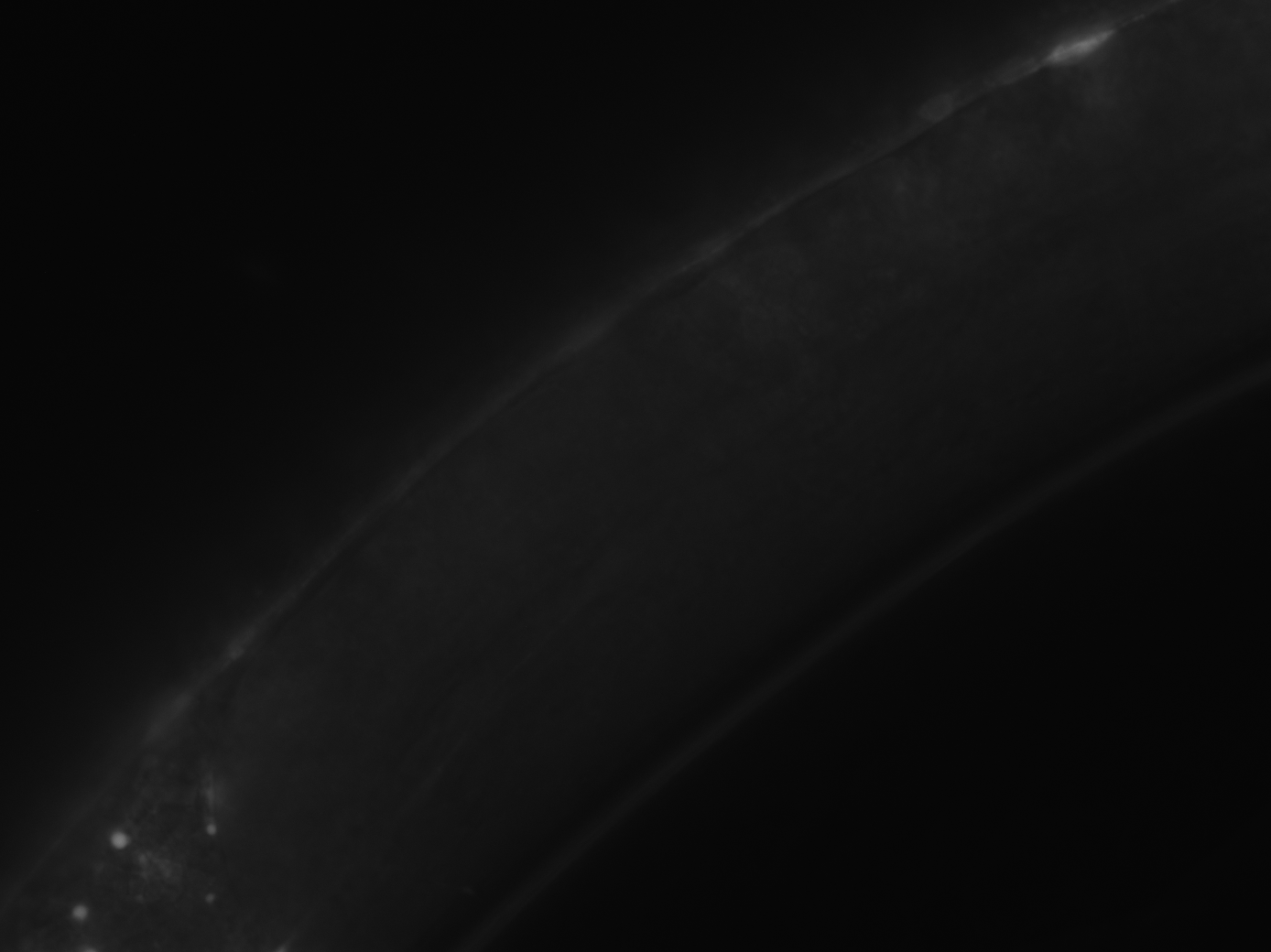

Supplement: Supplementary file 8 — Source data Fig. 7 [file 44319_2025_493_MOESM8_ESM.zip › Figure7/Fig7C/Experiment-48F54G21_VC.tif_files/Experiment-48_z4c0x0-1388y0-1040.tif]

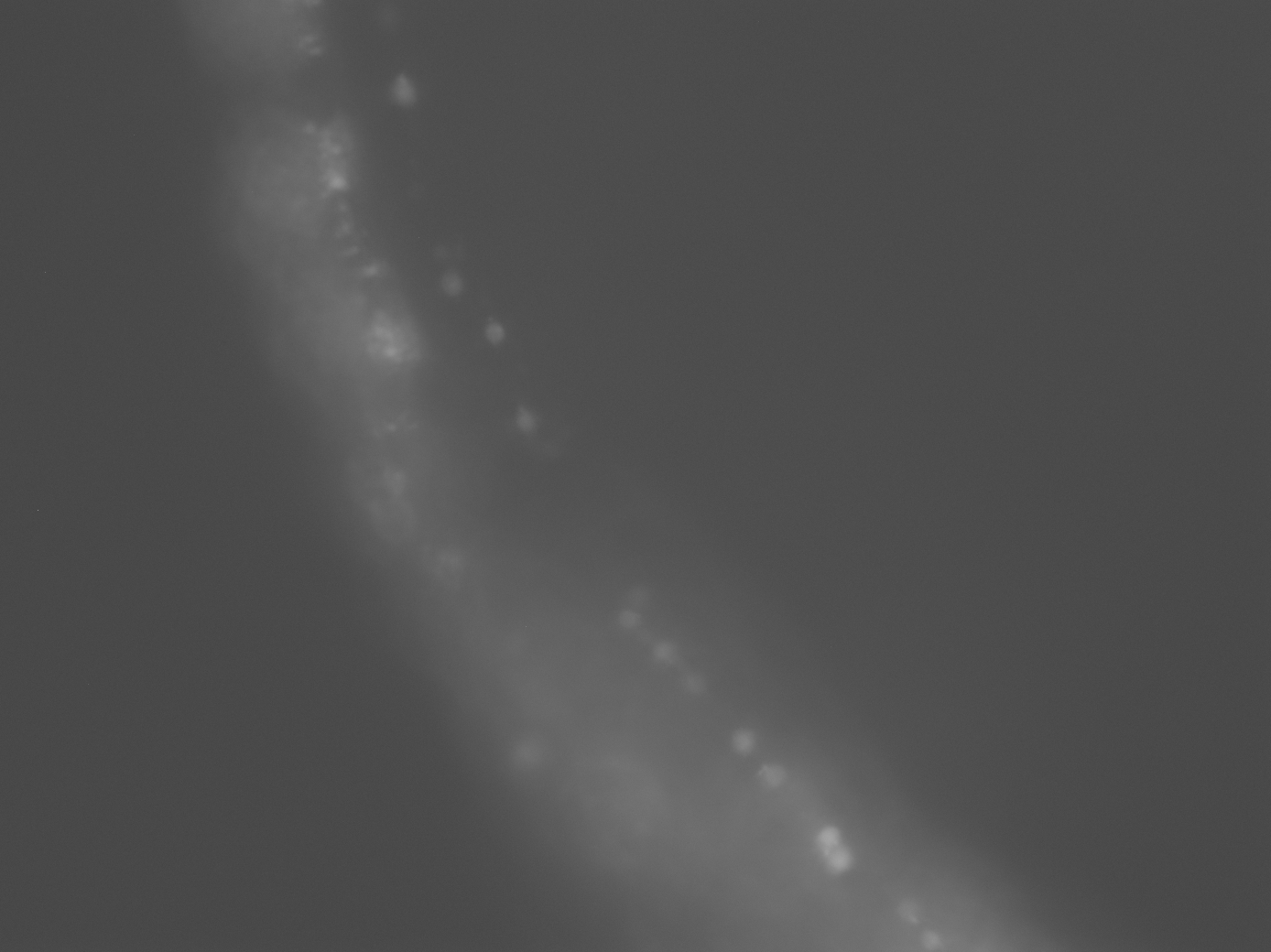

Supplement: Supplementary file 8 — Source data Fig. 7 [file 44319_2025_493_MOESM8_ESM.zip › Figure7/Fig7C/Experiment-72goodVCUNC31WTSAR.tif_files/Experiment-72good_z4c1x0-1388y0-1040.tif]

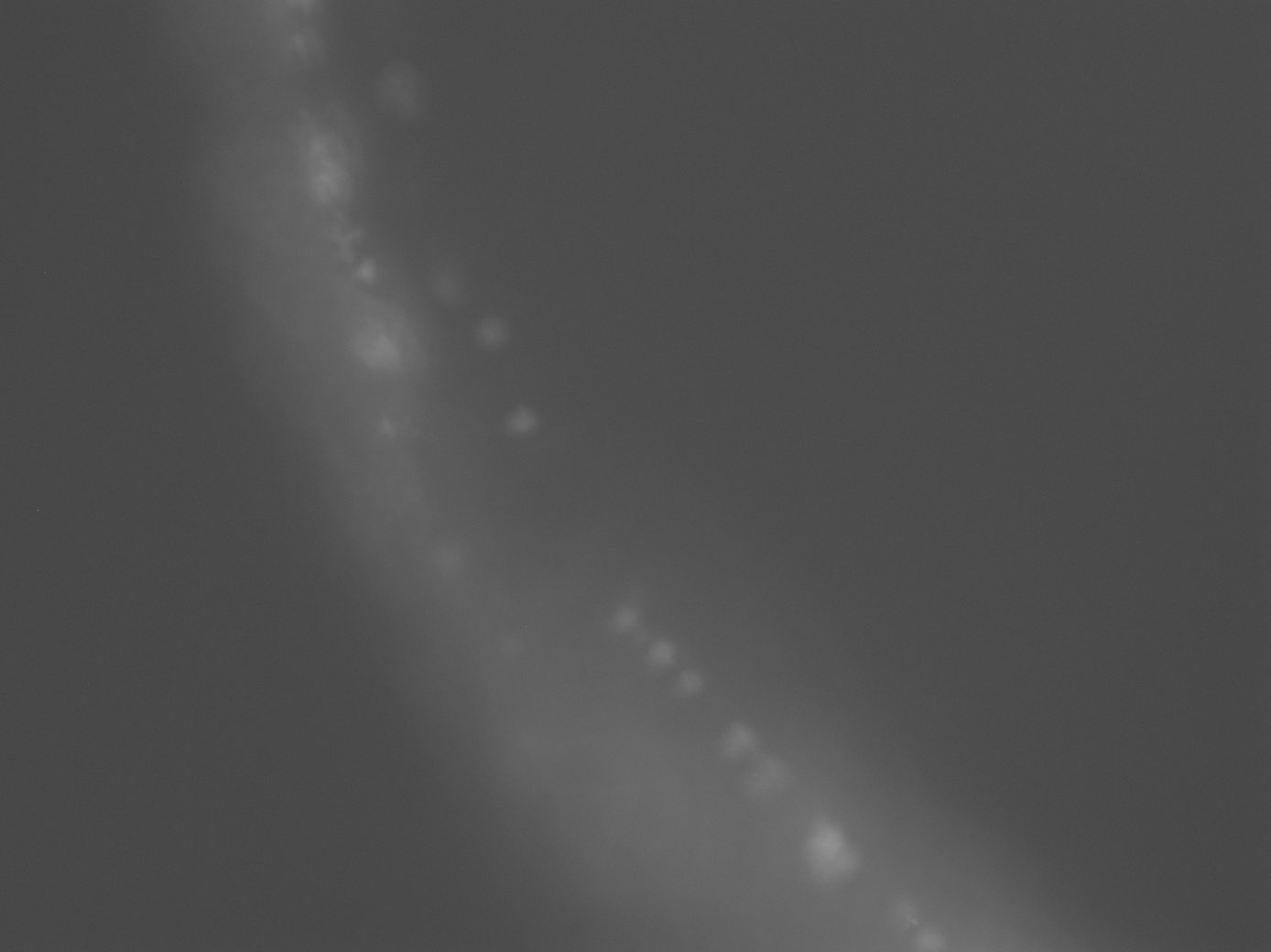

Supplement: Supplementary file 8 — Source data Fig. 7 [file 44319_2025_493_MOESM8_ESM.zip › Figure7/Fig7C/Experiment-72goodVCUNC31WTSAR.tif_files/Experiment-72good_z7c1x0-1388y0-1040.tif]

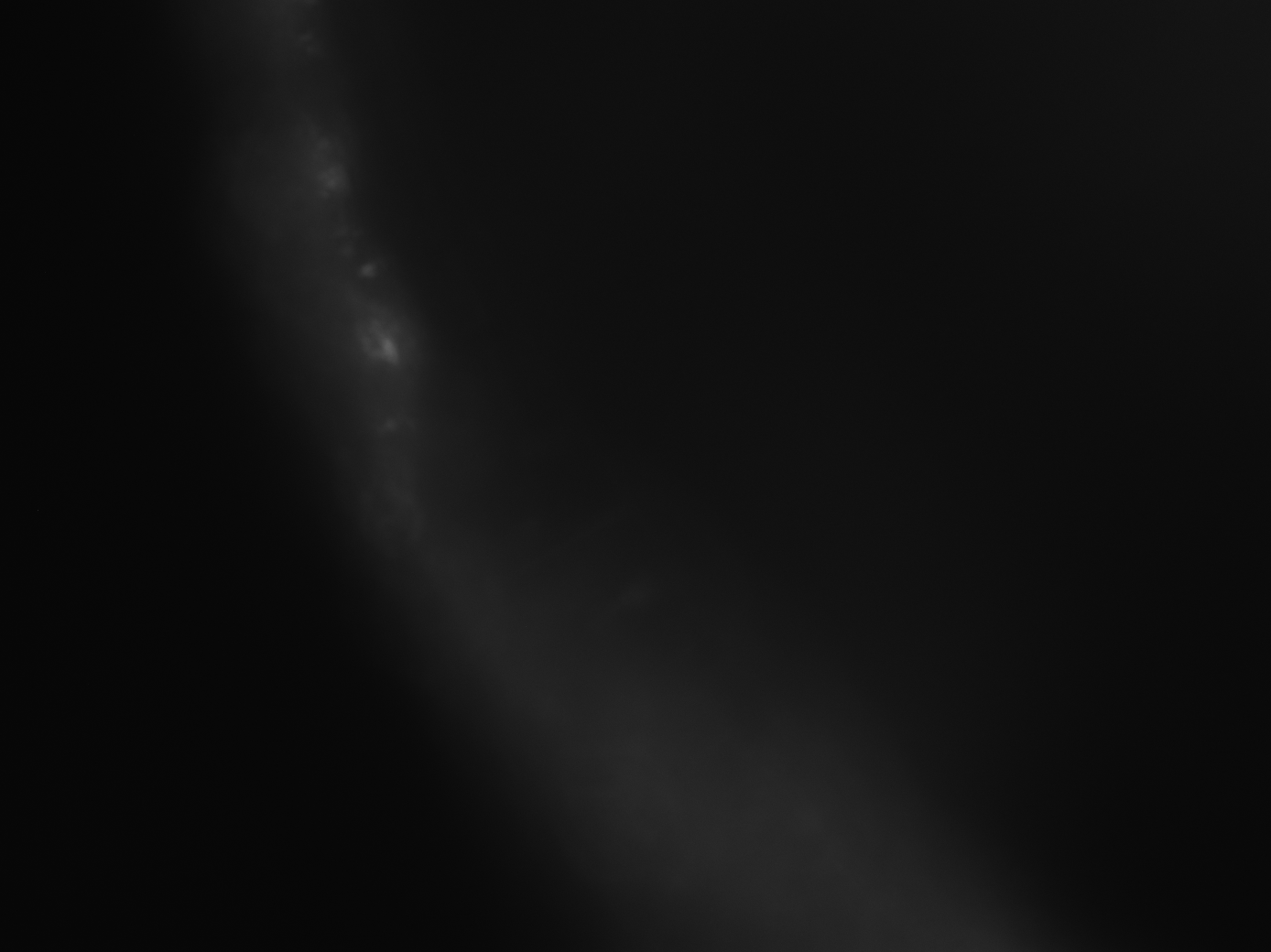

Supplement: Supplementary file 8 — Source data Fig. 7 [file 44319_2025_493_MOESM8_ESM.zip › Figure7/Fig7C/Experiment-72goodVCUNC31WTSAR.tif_files/Experiment-72good_z6c0x0-1388y0-1040.tif]

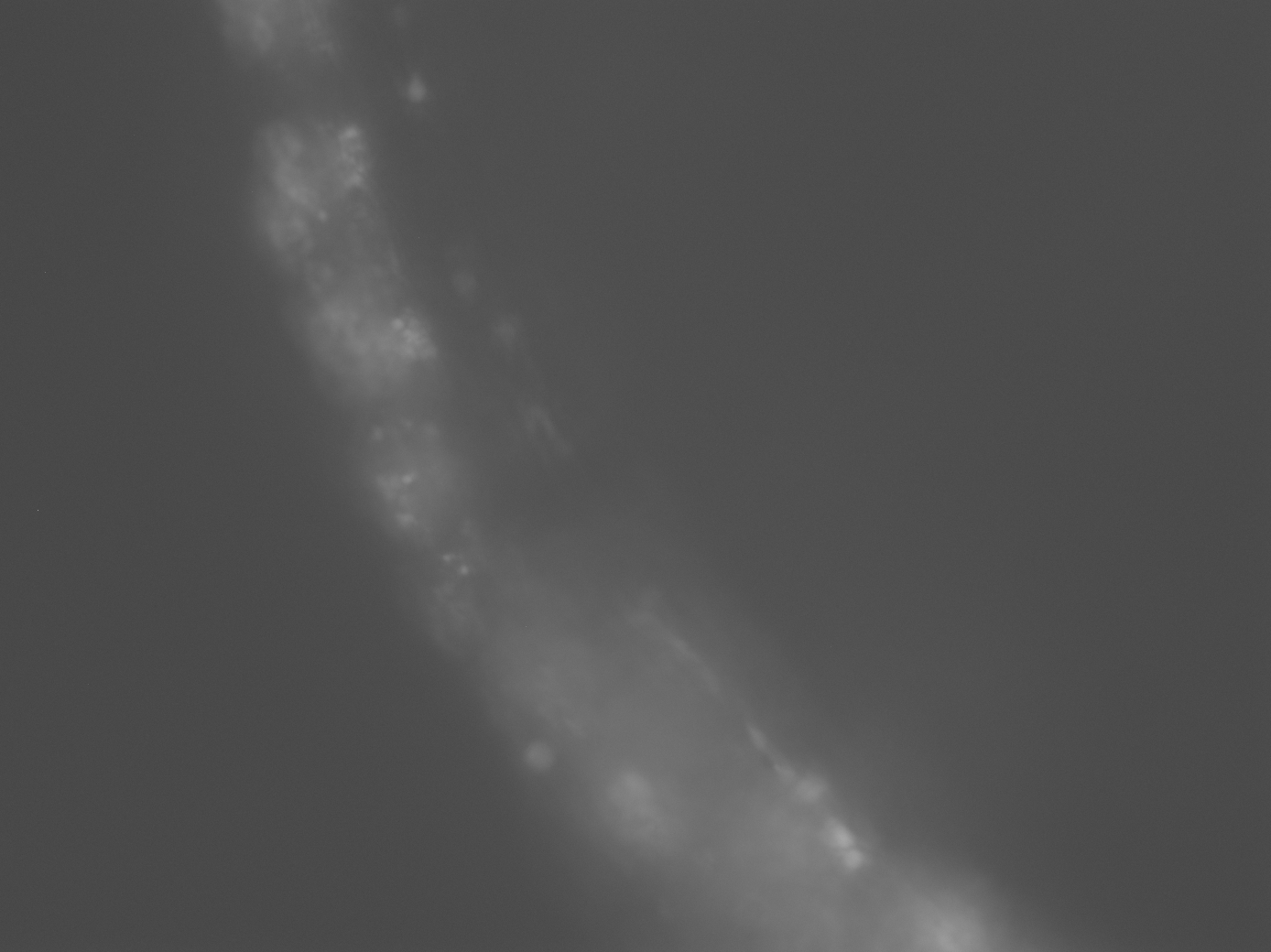

Supplement: Supplementary file 8 — Source data Fig. 7 [file 44319_2025_493_MOESM8_ESM.zip › Figure7/Fig7C/Experiment-72goodVCUNC31WTSAR.tif_files/Experiment-72good_z0c1x0-1388y0-1040.tif]

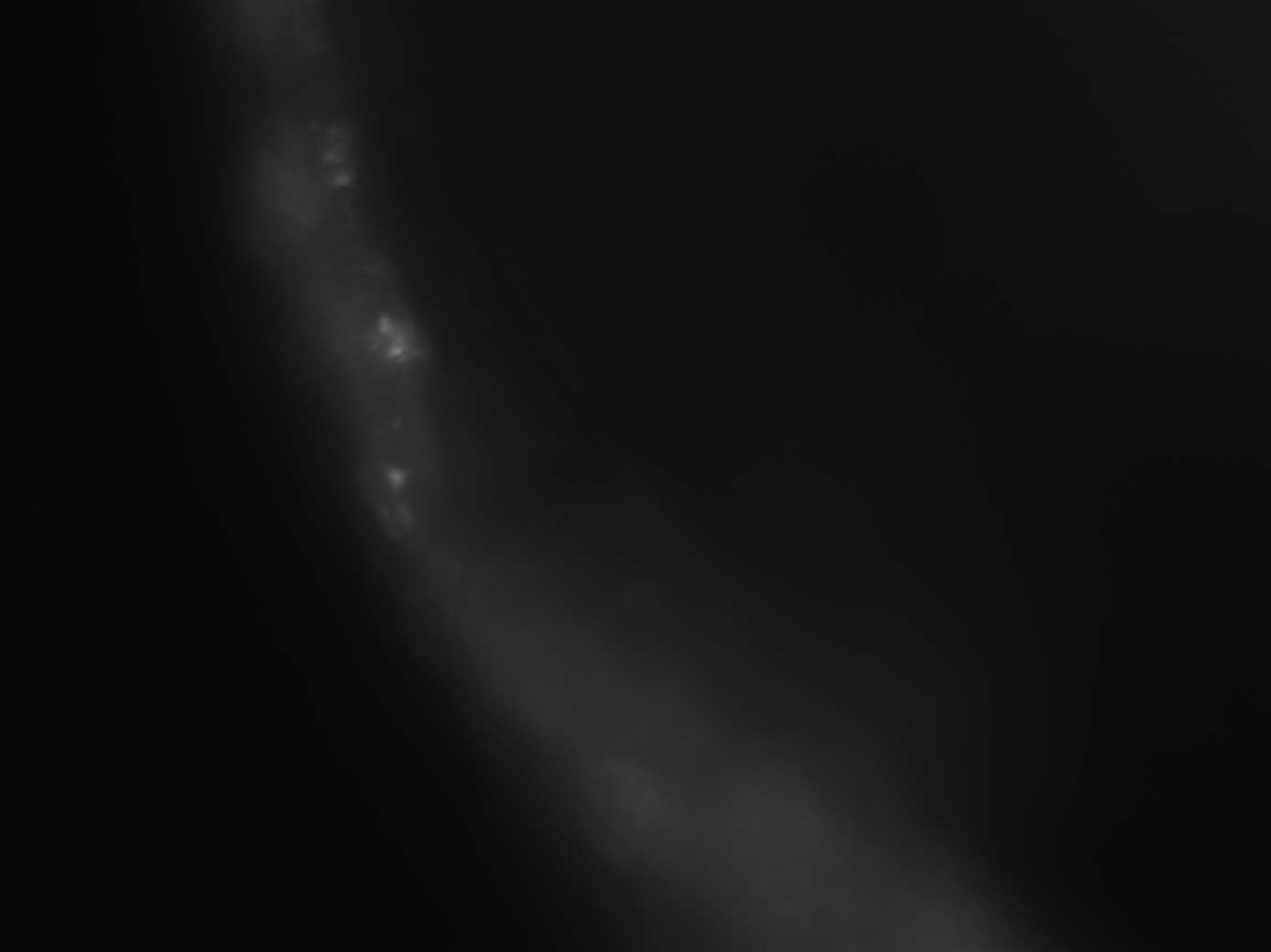

Supplement: Supplementary file 8 — Source data Fig. 7 [file 44319_2025_493_MOESM8_ESM.zip › Figure7/Fig7C/Experiment-72goodVCUNC31WTSAR.tif_files/Experiment-72good_z3c0x0-1388y0-1040.tif]

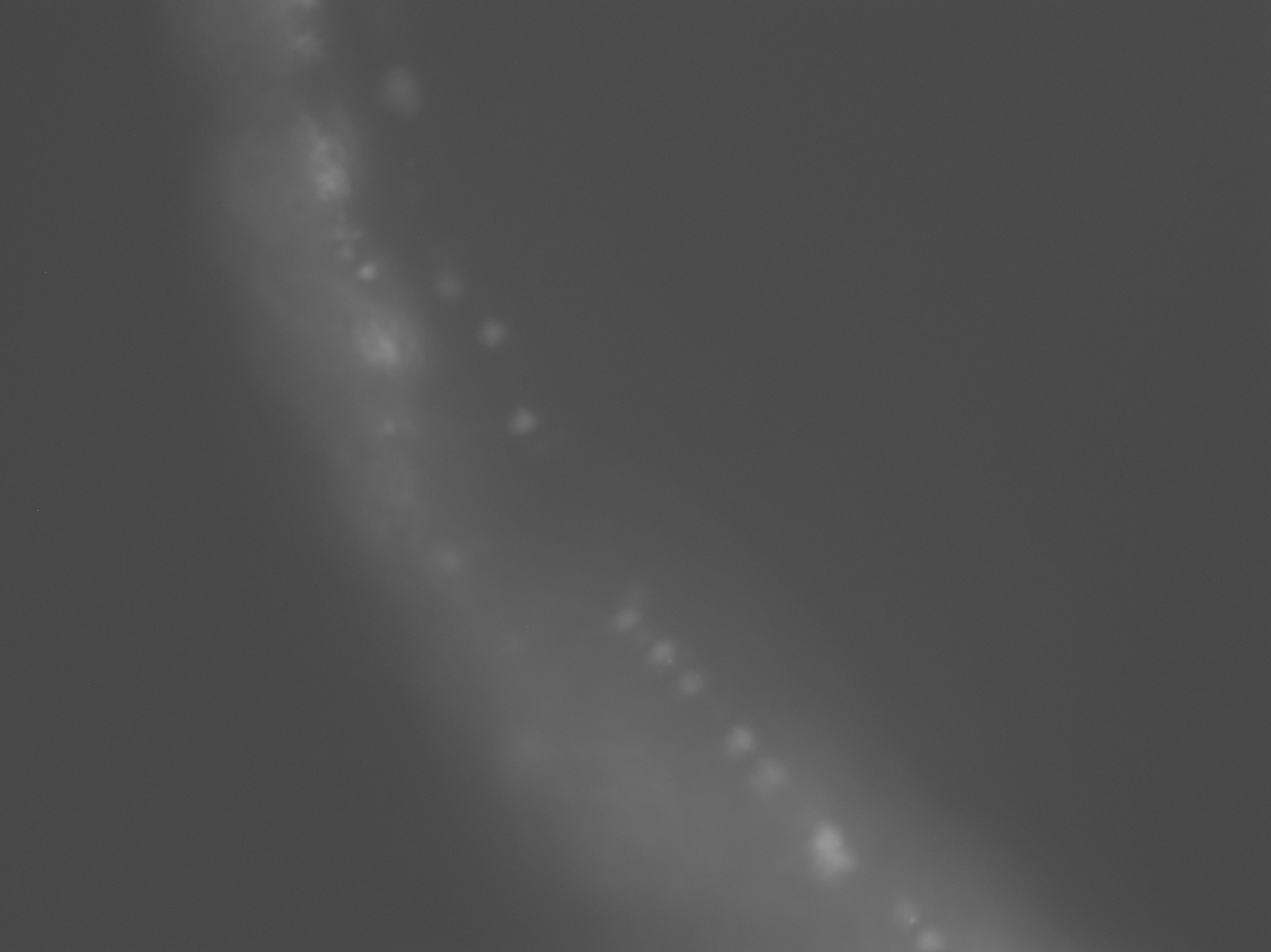

Supplement: Supplementary file 8 — Source data Fig. 7 [file 44319_2025_493_MOESM8_ESM.zip › Figure7/Fig7C/Experiment-72goodVCUNC31WTSAR.tif_files/Experiment-72good_z6c1x0-1388y0-1040.tif]

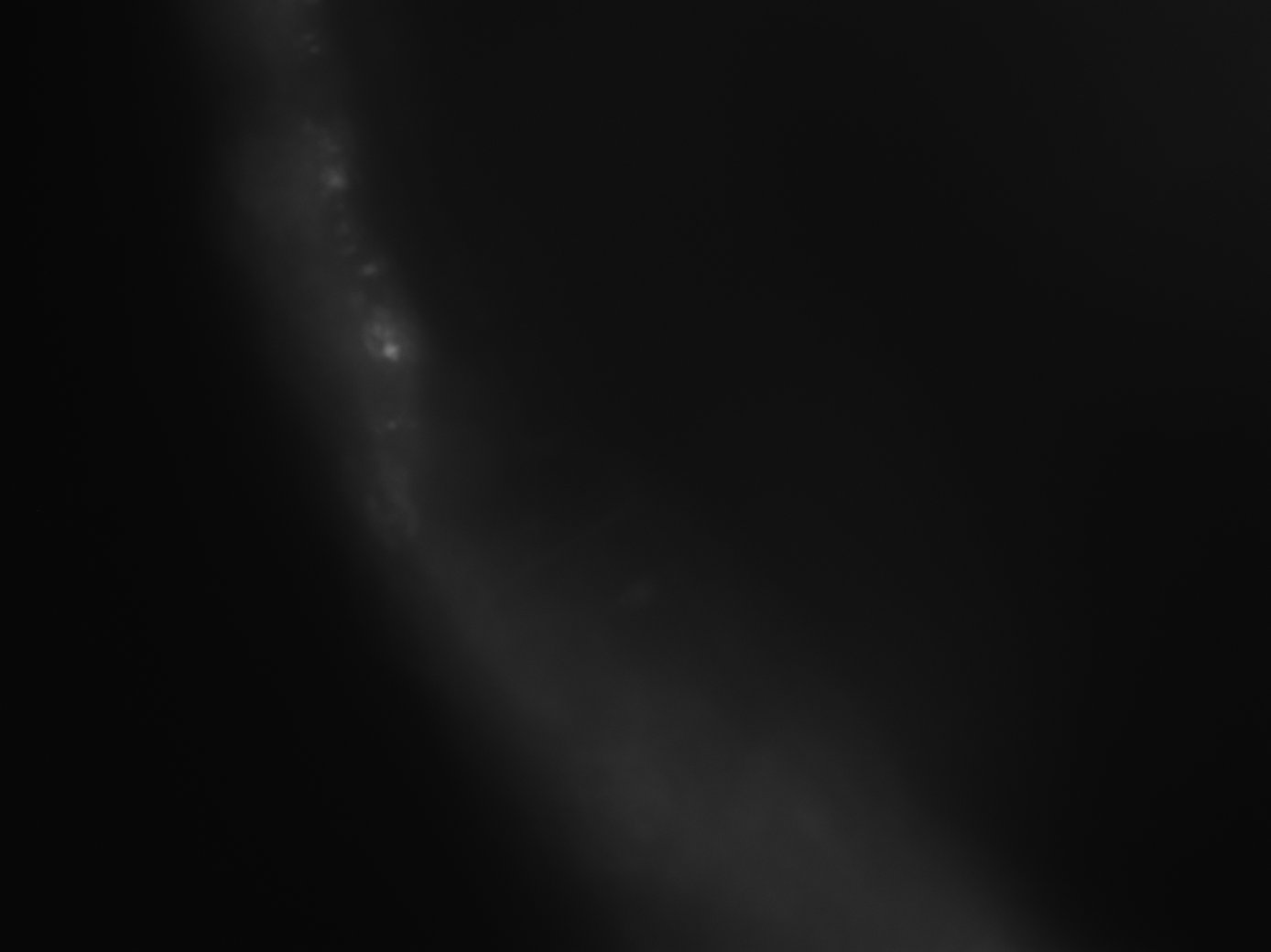

Supplement: Supplementary file 8 — Source data Fig. 7 [file 44319_2025_493_MOESM8_ESM.zip › Figure7/Fig7C/Experiment-72goodVCUNC31WTSAR.tif_files/Experiment-72good_z5c0x0-1388y0-1040.tif]
