# Supplementary figures and images for: Regulated microexon alternative splicing in single neurons tunes synaptic function (part 6 of 6)
Source: EMBO Rep. 2025 Jun 9;26(14):3640–62. doi: 10.1038/s44319-025-00493-7 (PMC12287369; doi:10.1038/s44319-025-00493-7)

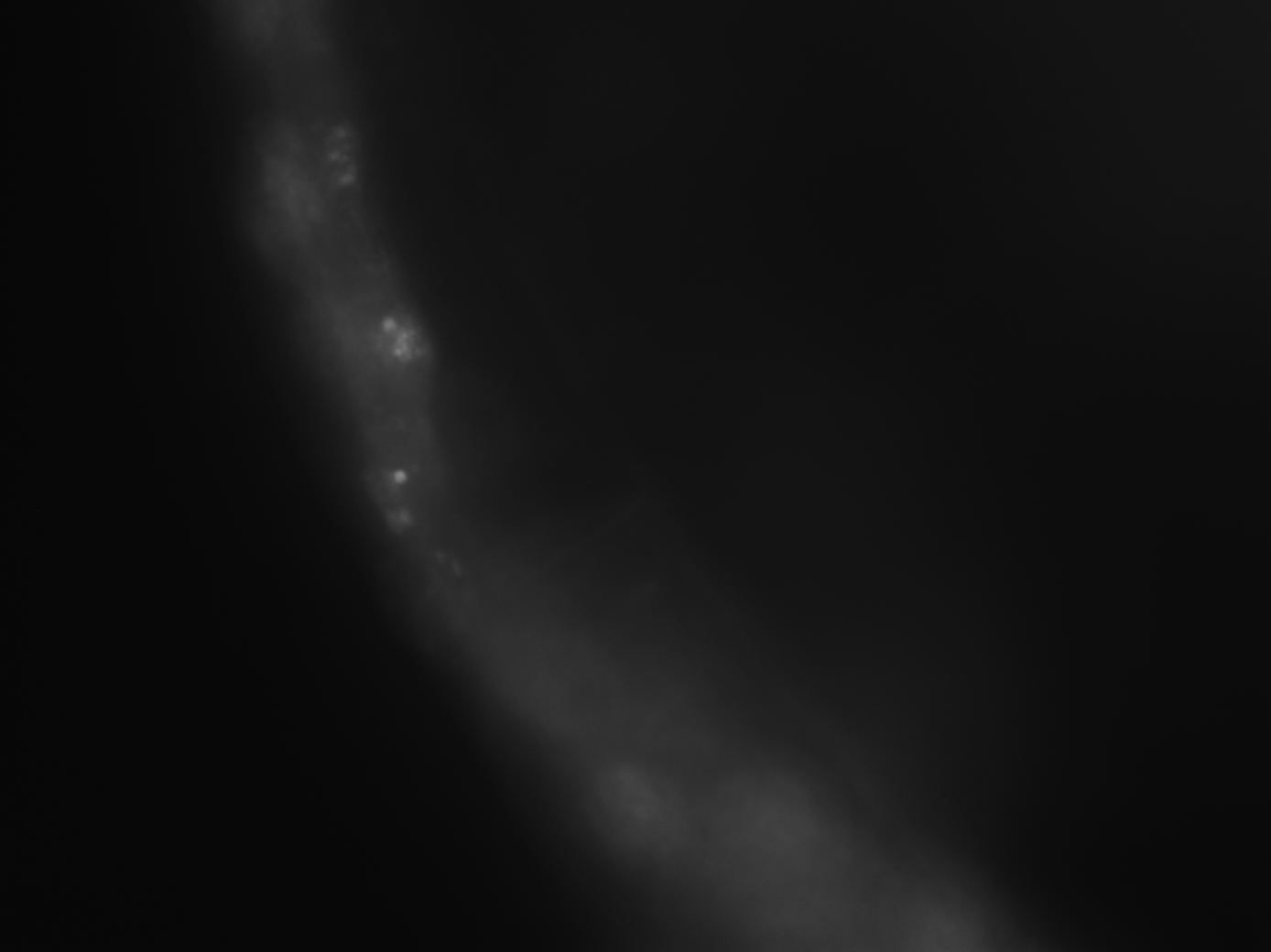

Supplement: Supplementary file 8 — Source data Fig. 7 [file 44319_2025_493_MOESM8_ESM.zip › Figure7/Fig7C/Experiment-72goodVCUNC31WTSAR.tif_files/Experiment-72good_z2c0x0-1388y0-1040.tif]

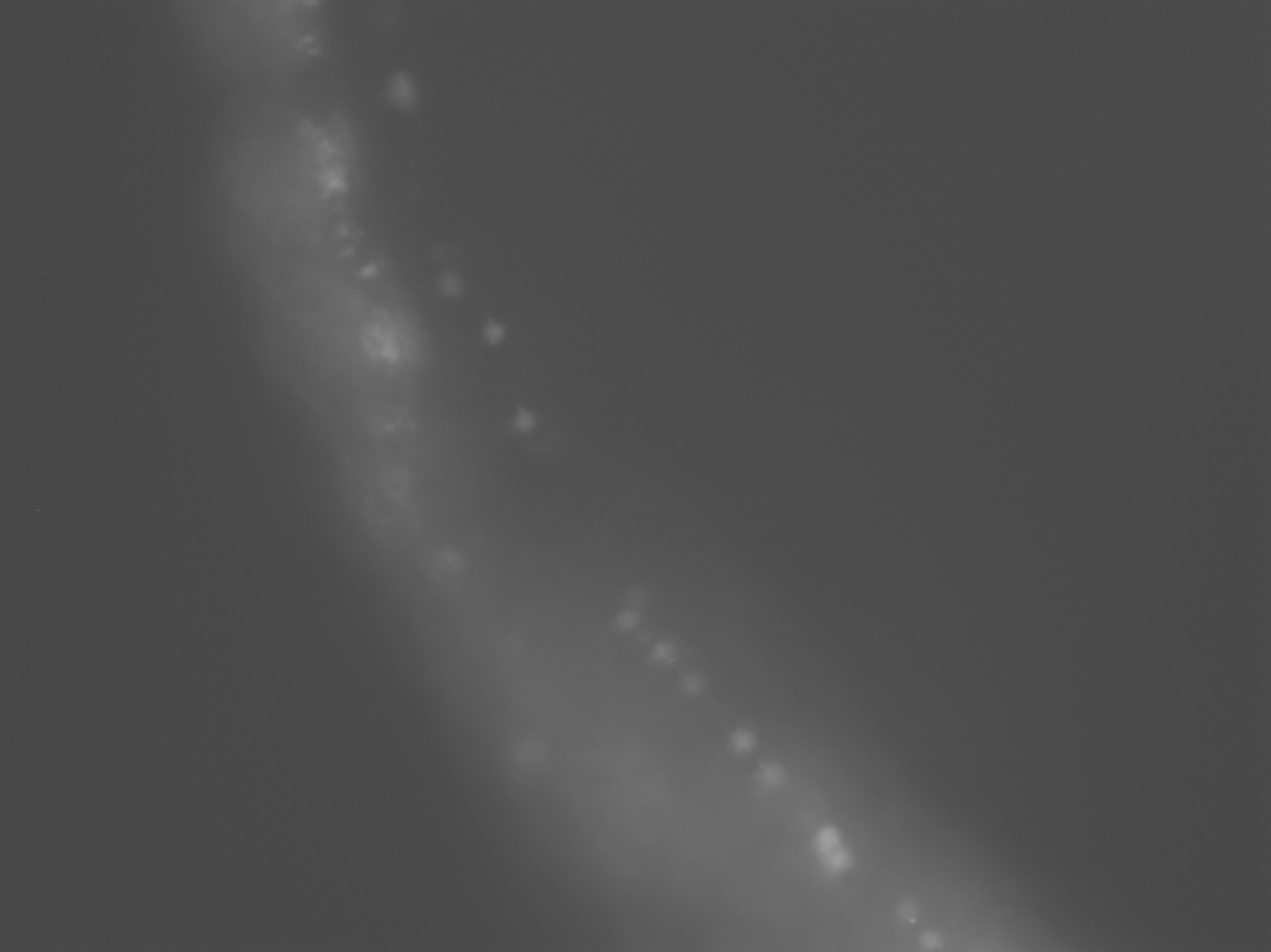

Supplement: Supplementary file 8 — Source data Fig. 7 [file 44319_2025_493_MOESM8_ESM.zip › Figure7/Fig7C/Experiment-72goodVCUNC31WTSAR.tif_files/Experiment-72good_z5c1x0-1388y0-1040.tif]

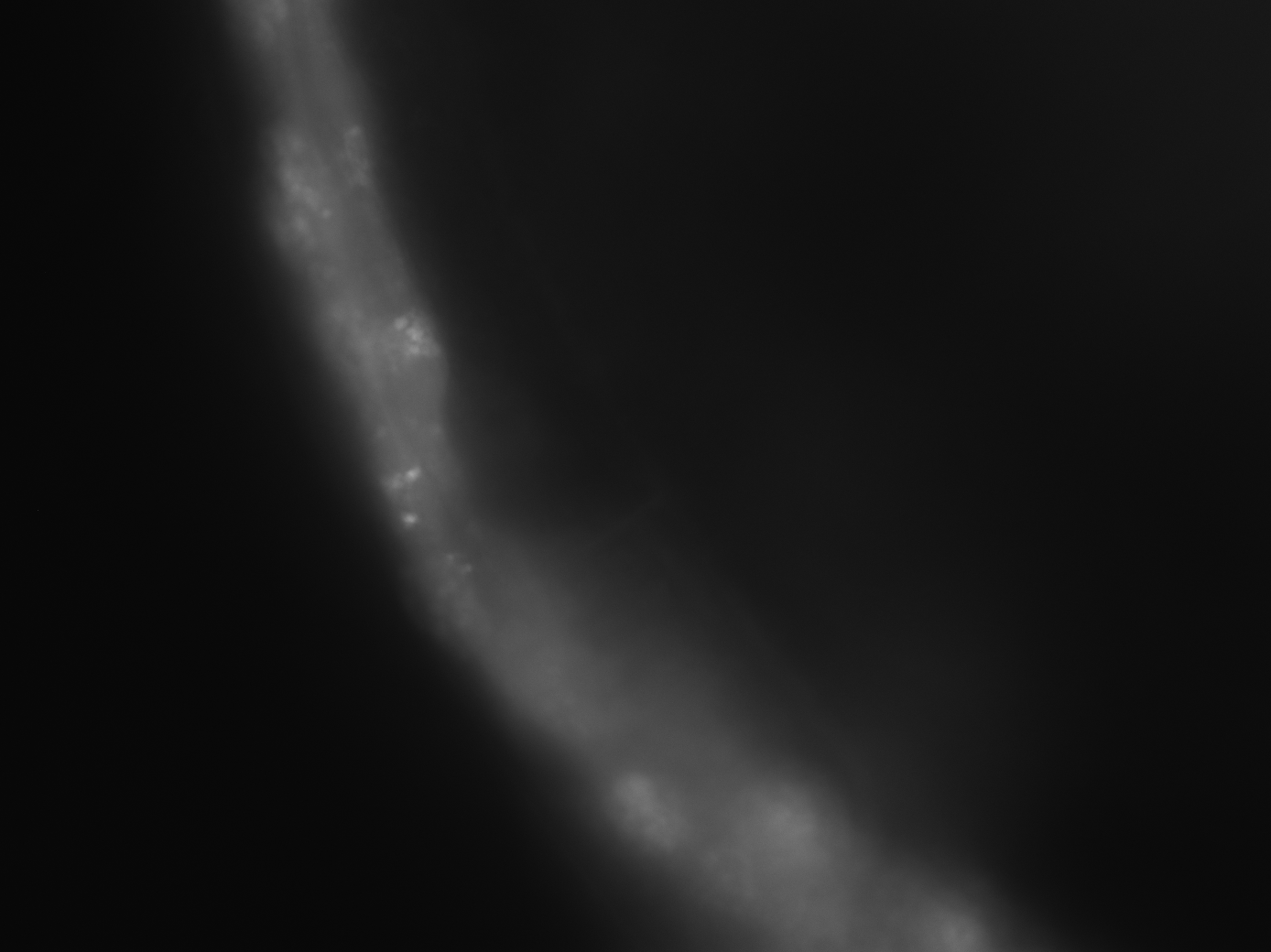

Supplement: Supplementary file 8 — Source data Fig. 7 [file 44319_2025_493_MOESM8_ESM.zip › Figure7/Fig7C/Experiment-72goodVCUNC31WTSAR.tif_files/Experiment-72good_z0c0x0-1388y0-1040.tif]

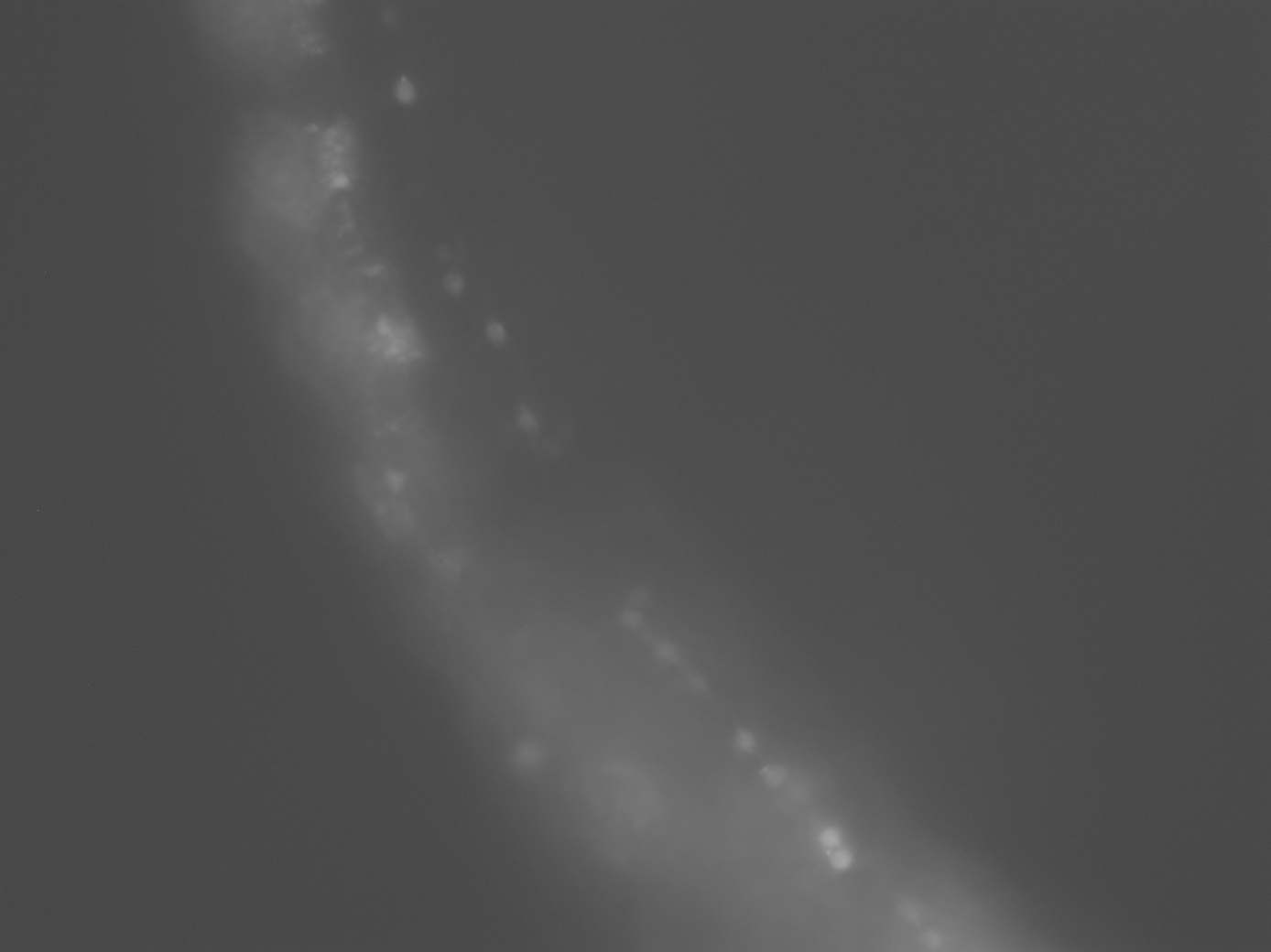

Supplement: Supplementary file 8 — Source data Fig. 7 [file 44319_2025_493_MOESM8_ESM.zip › Figure7/Fig7C/Experiment-72goodVCUNC31WTSAR.tif_files/Experiment-72good_z3c1x0-1388y0-1040.tif]

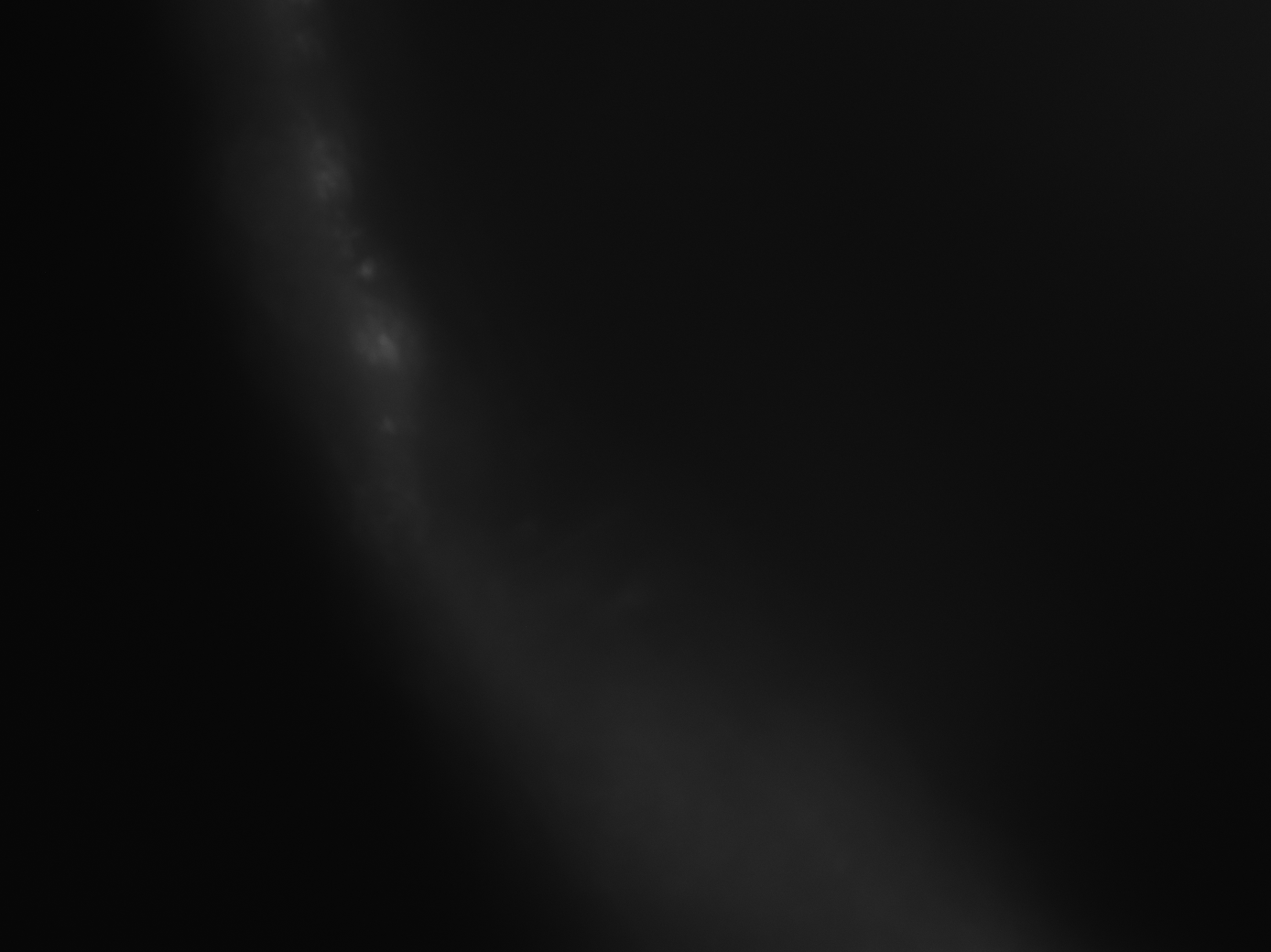

Supplement: Supplementary file 8 — Source data Fig. 7 [file 44319_2025_493_MOESM8_ESM.zip › Figure7/Fig7C/Experiment-72goodVCUNC31WTSAR.tif_files/Experiment-72good_z7c0x0-1388y0-1040.tif]

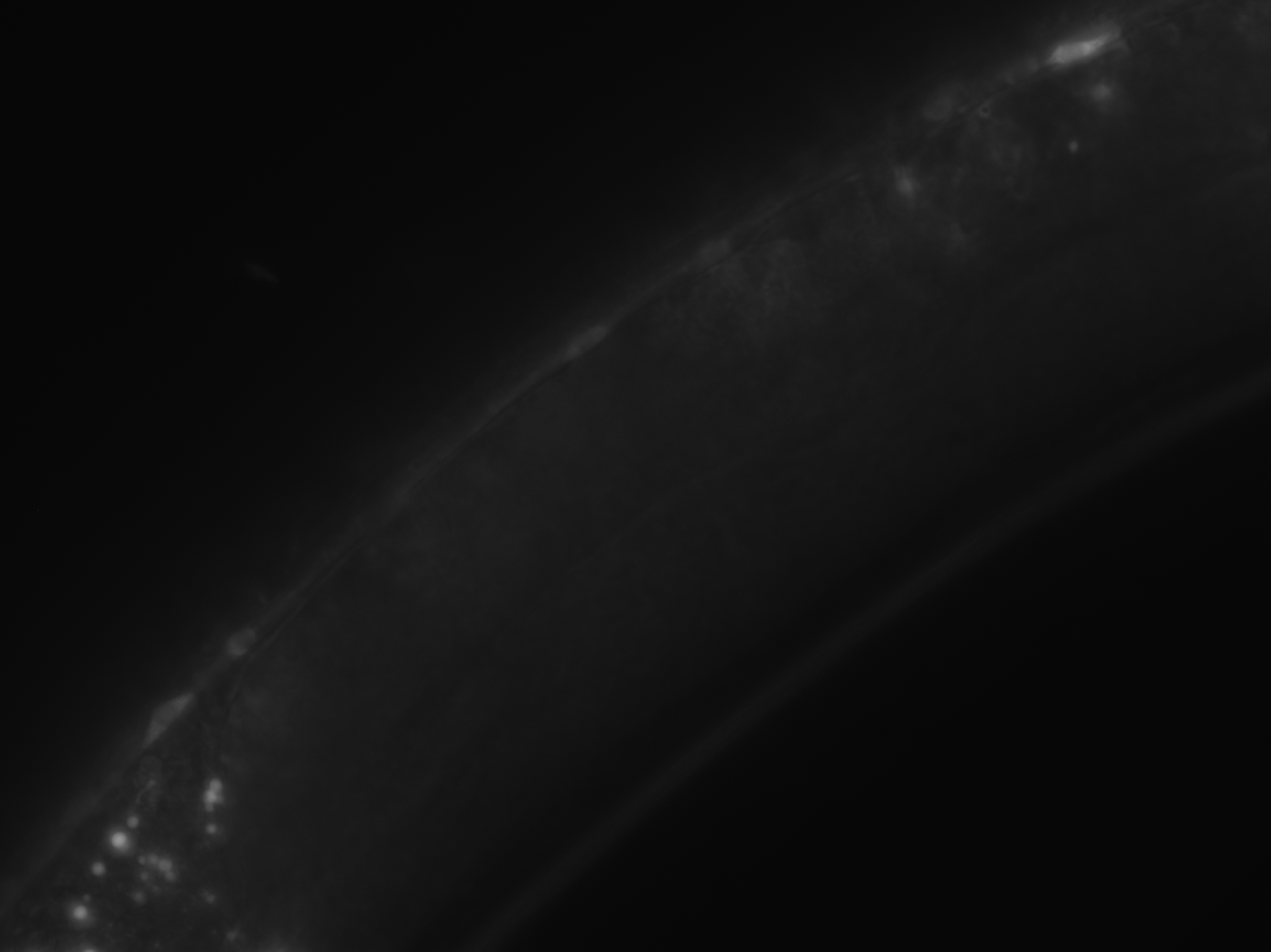

Supplement: Supplementary file 8 — Source data Fig. 7 [file 44319_2025_493_MOESM8_ESM.zip › Figure7/Fig7C/Experiment-48F54G21_VC.tif_files/VC/green.tif]

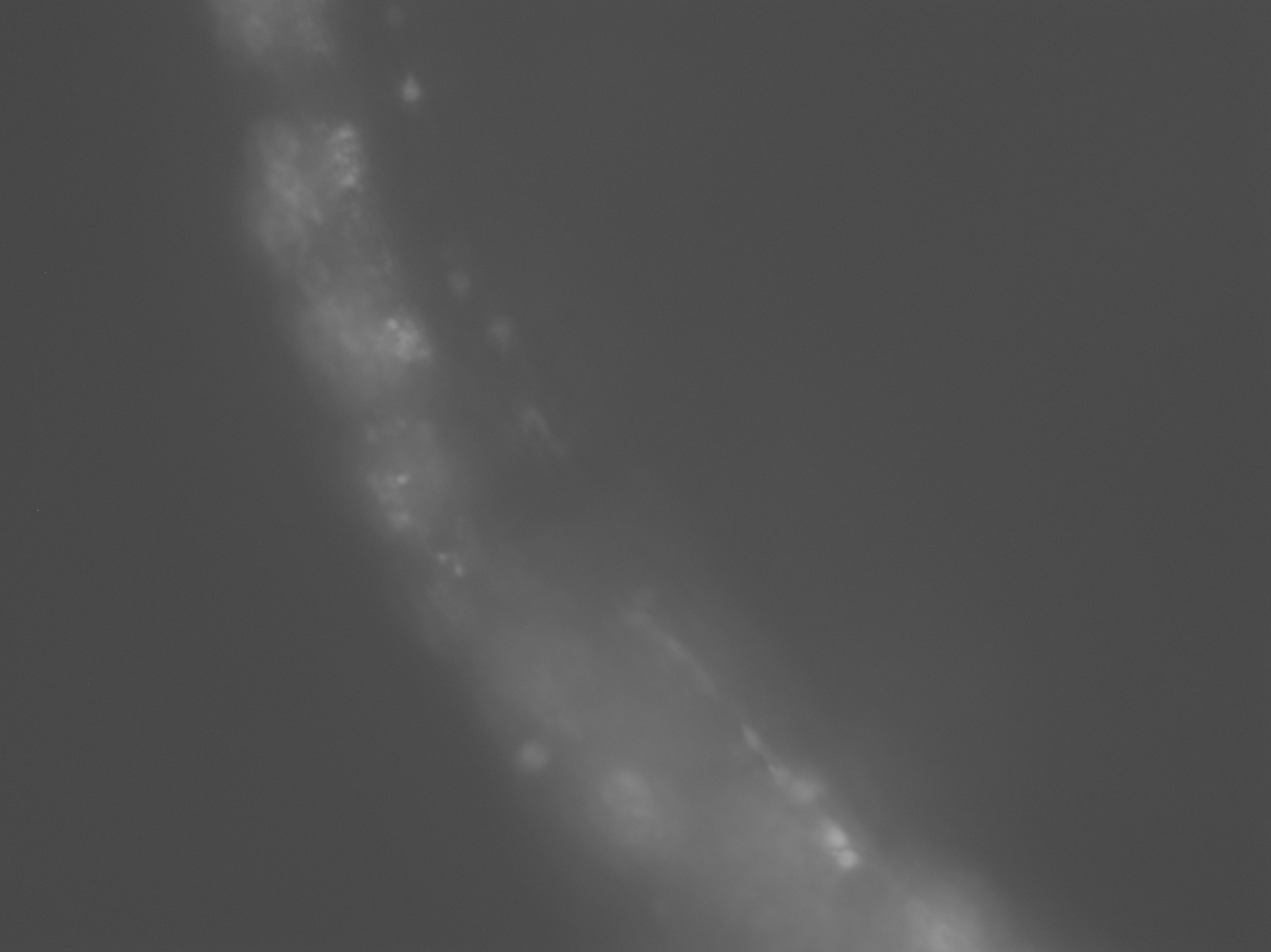

Supplement: Supplementary file 8 — Source data Fig. 7 [file 44319_2025_493_MOESM8_ESM.zip › Figure7/Fig7C/Experiment-72goodVCUNC31WTSAR.tif_files/Experiment-72good_z1c1x0-1388y0-1040.tif]

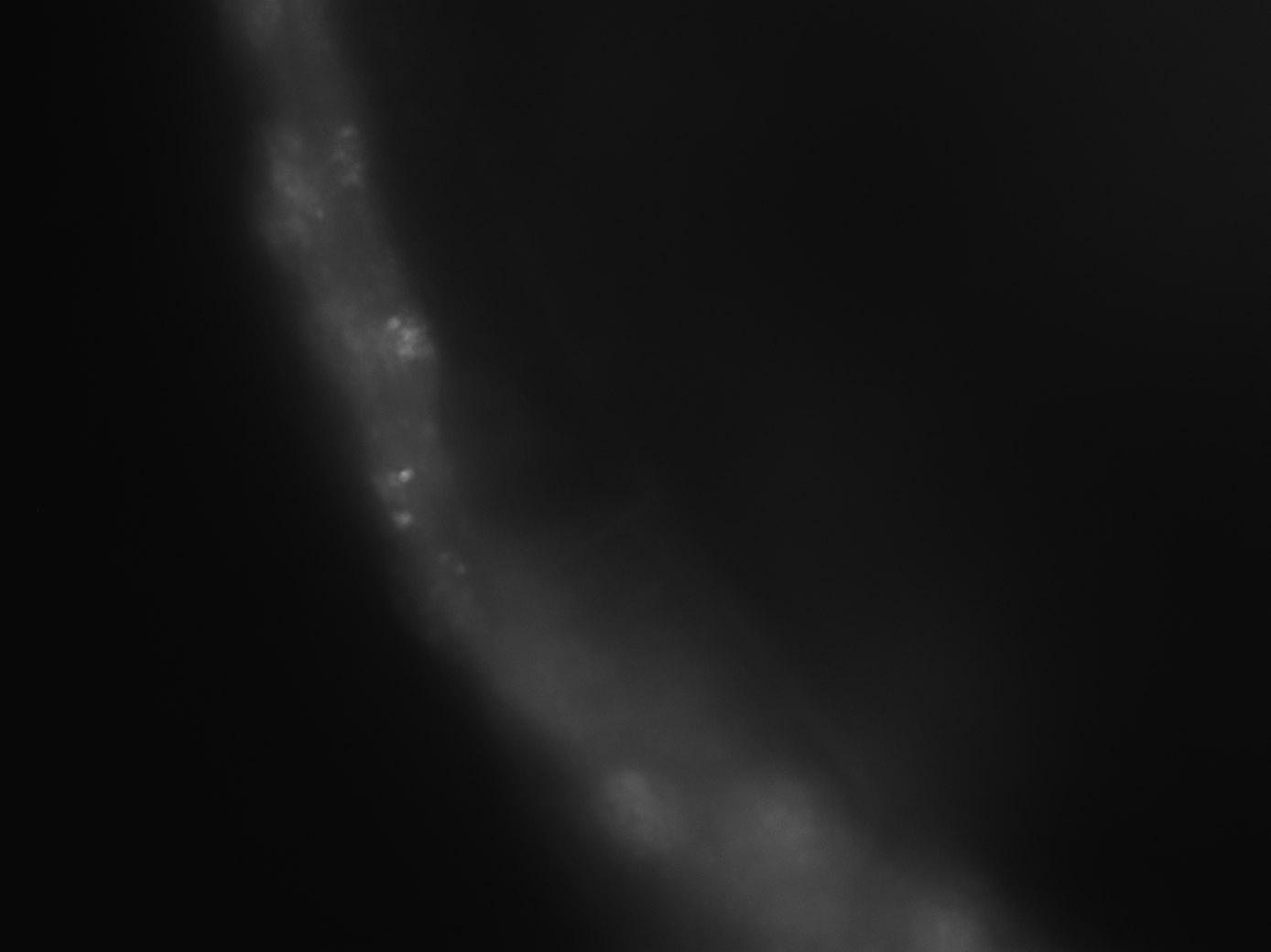

Supplement: Supplementary file 8 — Source data Fig. 7 [file 44319_2025_493_MOESM8_ESM.zip › Figure7/Fig7C/Experiment-72goodVCUNC31WTSAR.tif_files/Experiment-72good_z1c0x0-1388y0-1040.tif]

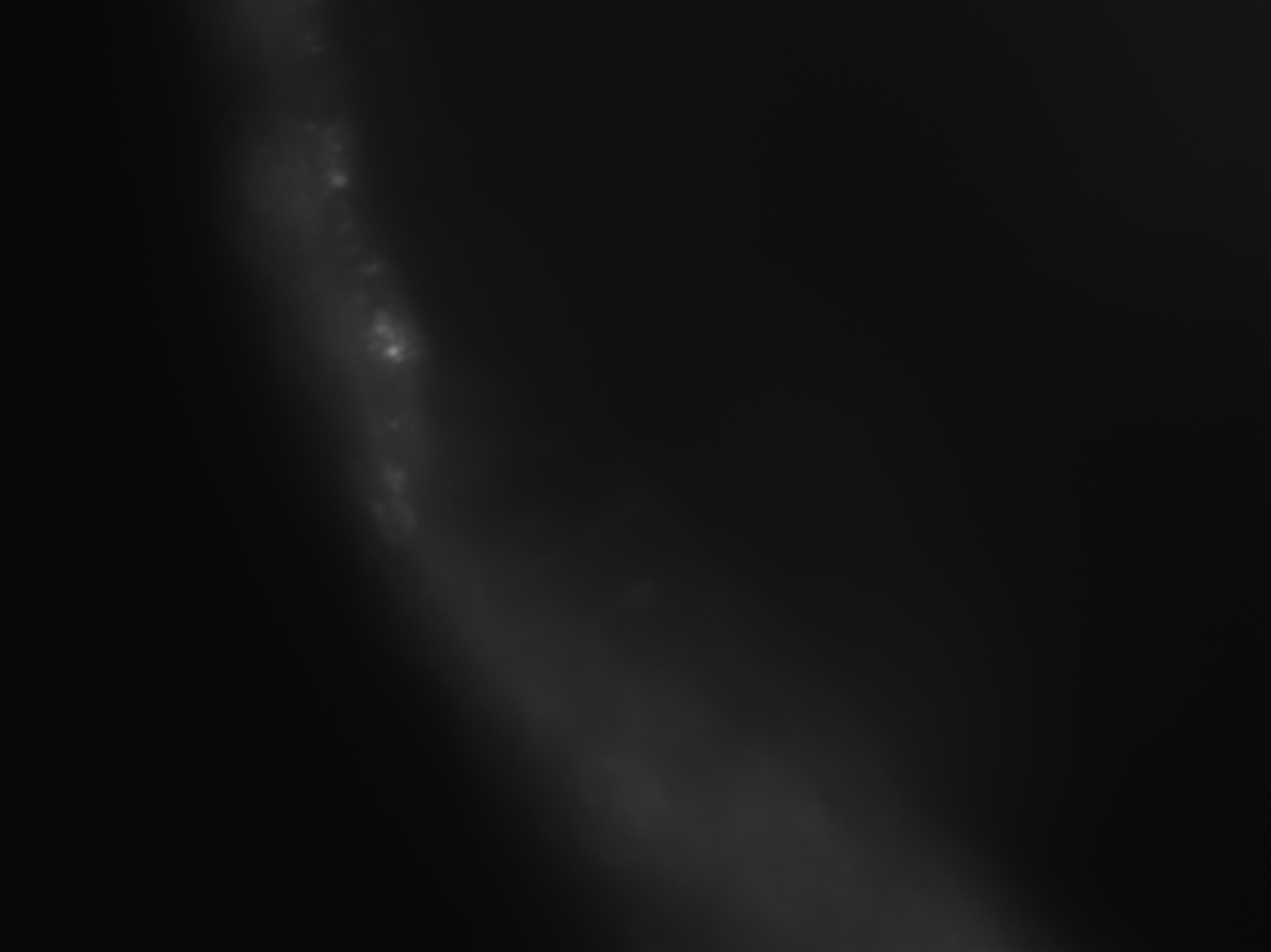

Supplement: Supplementary file 8 — Source data Fig. 7 [file 44319_2025_493_MOESM8_ESM.zip › Figure7/Fig7C/Experiment-72goodVCUNC31WTSAR.tif_files/Experiment-72good_z4c0x0-1388y0-1040.tif]

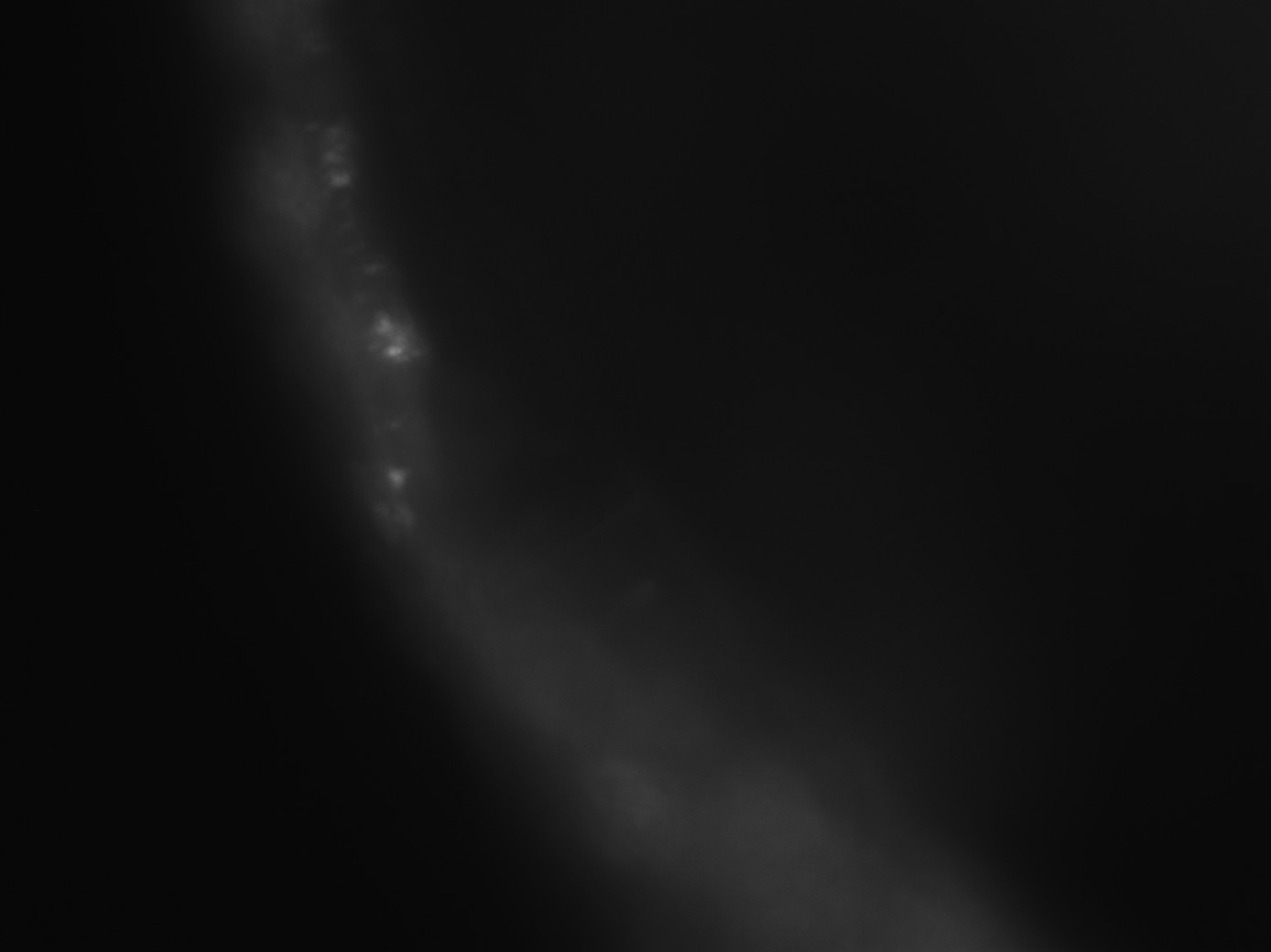

Supplement: Supplementary file 8 — Source data Fig. 7 [file 44319_2025_493_MOESM8_ESM.zip › Figure7/Fig7C/Experiment-72goodVCUNC31WTSAR.tif_files/vc/red.tif]

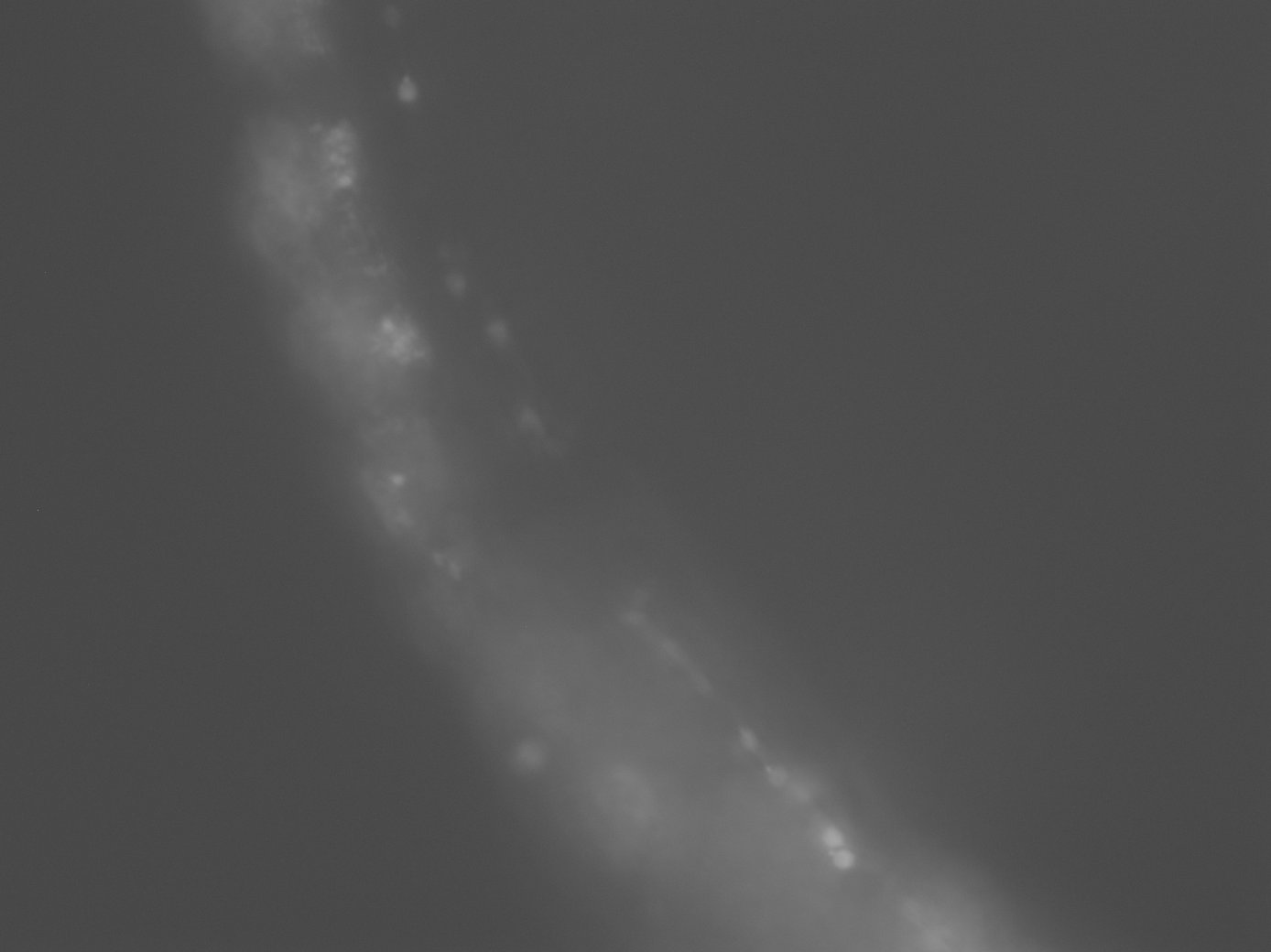

Supplement: Supplementary file 8 — Source data Fig. 7 [file 44319_2025_493_MOESM8_ESM.zip › Figure7/Fig7C/Experiment-72goodVCUNC31WTSAR.tif_files/Experiment-72good_z2c1x0-1388y0-1040.tif]

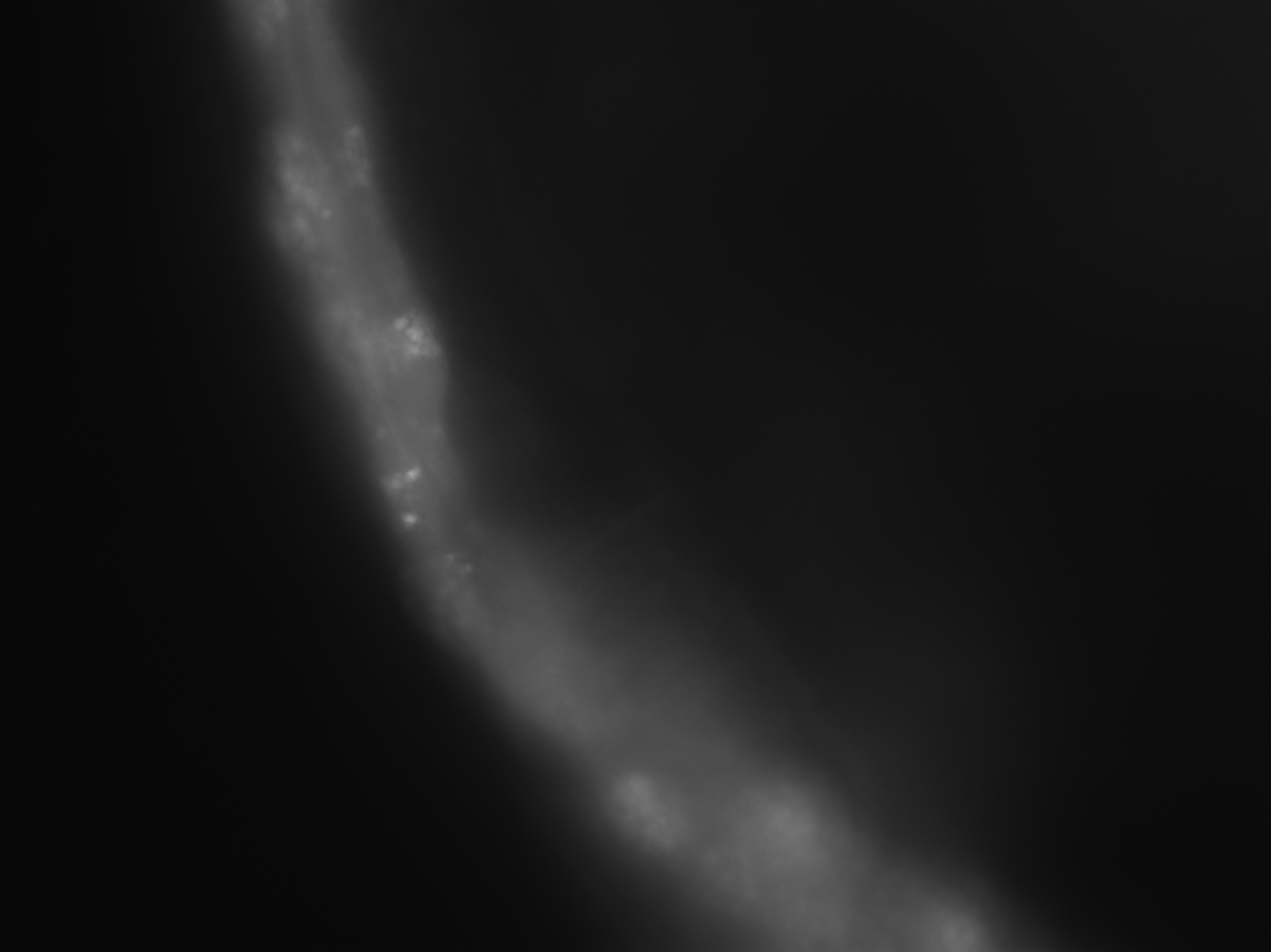

Supplement: Supplementary file 8 — Source data Fig. 7 [file 44319_2025_493_MOESM8_ESM.zip › Figure7/Fig7C/Experiment-72goodVCUNC31WTSAR.tif_files/vc/Experiment-72goodVCgreen.tif_files.tif]

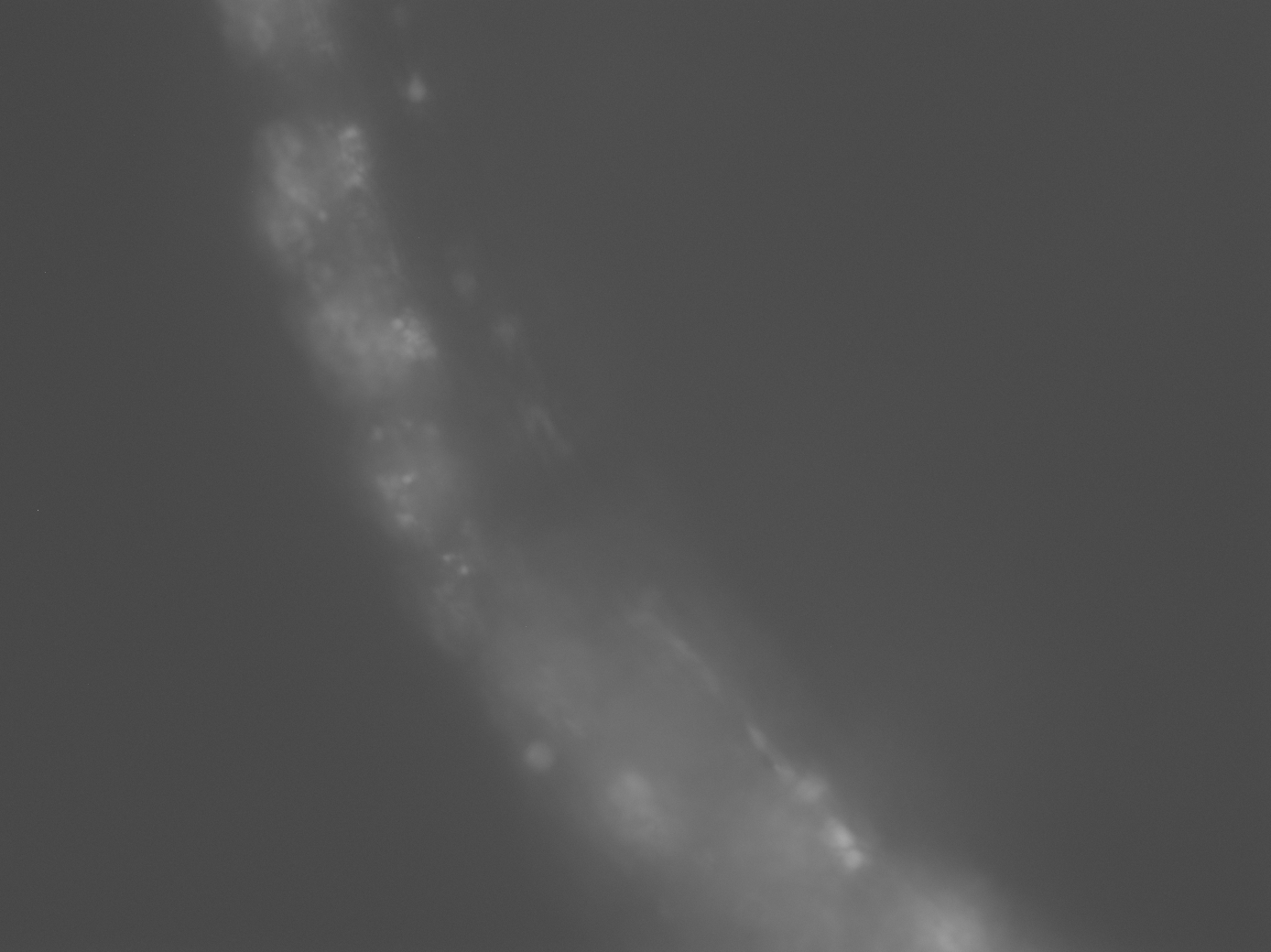

Supplement: Supplementary file 8 — Source data Fig. 7 [file 44319_2025_493_MOESM8_ESM.zip › Figure7/Fig7C/Experiment-72goodVCUNC31WTSAR.tif_files/vc/Experiment-72goodVCred.tif_files.tif]
