# Supplementary material for: Regulated microexon alternative splicing in single neurons tunes synaptic function
Source: EMBO Rep. 2025 Jun 9;26(14):3640–62. doi: 10.1038/s44319-025-00493-7 (PMC12287369; doi:10.1038/s44319-025-00493-7)
Supplement: Supplementary file 9 — Expanded View Figures [file 44319_2025_493_MOESM9_ESM.pdf]

Expanded View Figures

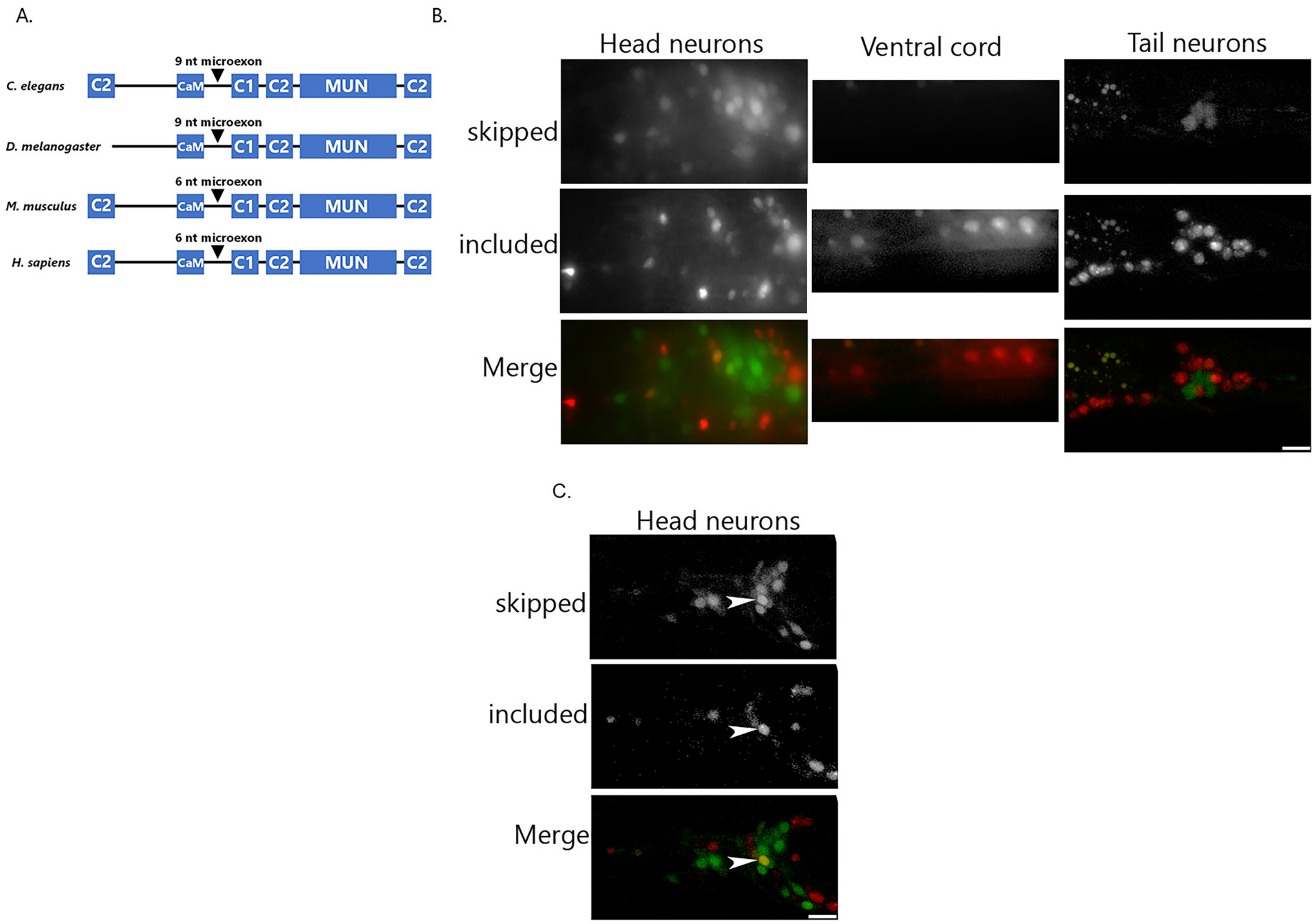

Figure EV1. The presence of an alternative microexon is conserved across species and its splicing pattern is invariant across multiple isolated independent integrated transgenic lines in *C. elegans*.

(**A**) Schematic of the position of the alternatively spliced microexon in the *unc-13* transcript across various species. In mice and humans, it's 6 nt microexon, whereas in *C. elegans* and *Drosophila*, it's 9 nt. Black arrowheads mark the position of the microexon in different species. (**B**) *unc-13* microexon-splicing pattern in various regions of the nervous system of an independently isolated integrated transgenic line. (**C**) A neuron marked by a white arrowhead expressing both skipped and included versions in the nerve ring neurons of the *unc-13* microexon splicing reporter expressing animal. Scale bar 20 µm.

 © The Author(s)

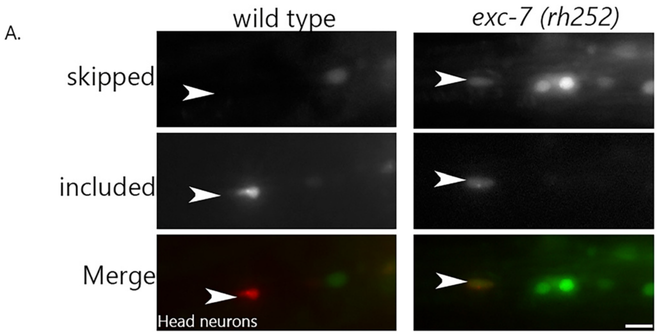

Figure EV2. Alternative splicing of the *unc-13* microexon is affected in a neuron-specific manner in *exc-7* animals.

(A) A region of the head neurons, where an identified neuron in wild type exclusively has RFP, whereas in *exc-7(rh252)* it has both, GFP and RFP signal. The white arrowhead marks that neuron. Scale bar 10 µm.

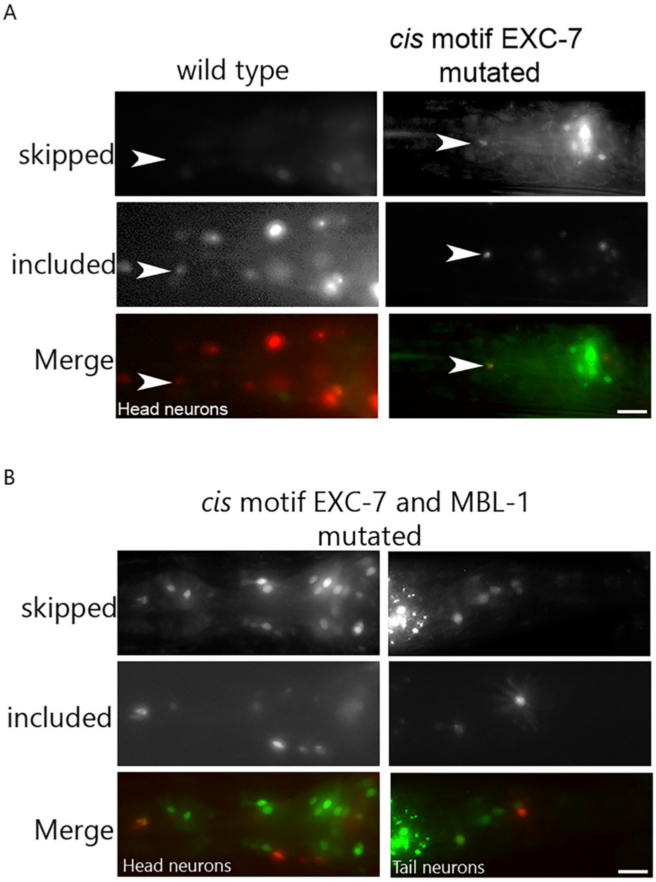

Figure EV3. Mutating *cis* motifs for EXC-7 and/or MBL-1 affect splicing in a neuronal-subtype-specific manner.

(**A**) A neuron in pharyngeal region shows the expression of GFP and RFP, whereas in wild type it expresses only included form. The white arrowhead marks that individual neuron. (**B**) Representative image showing *cis* motif mutation in both EXC-7 and MBL-1, where a set of animals (~20% of animals) has lesser RFP neuronal cell bodies in head and tail neurons compared to wild type. Scale bar 20 μm.

 © The Author(s)

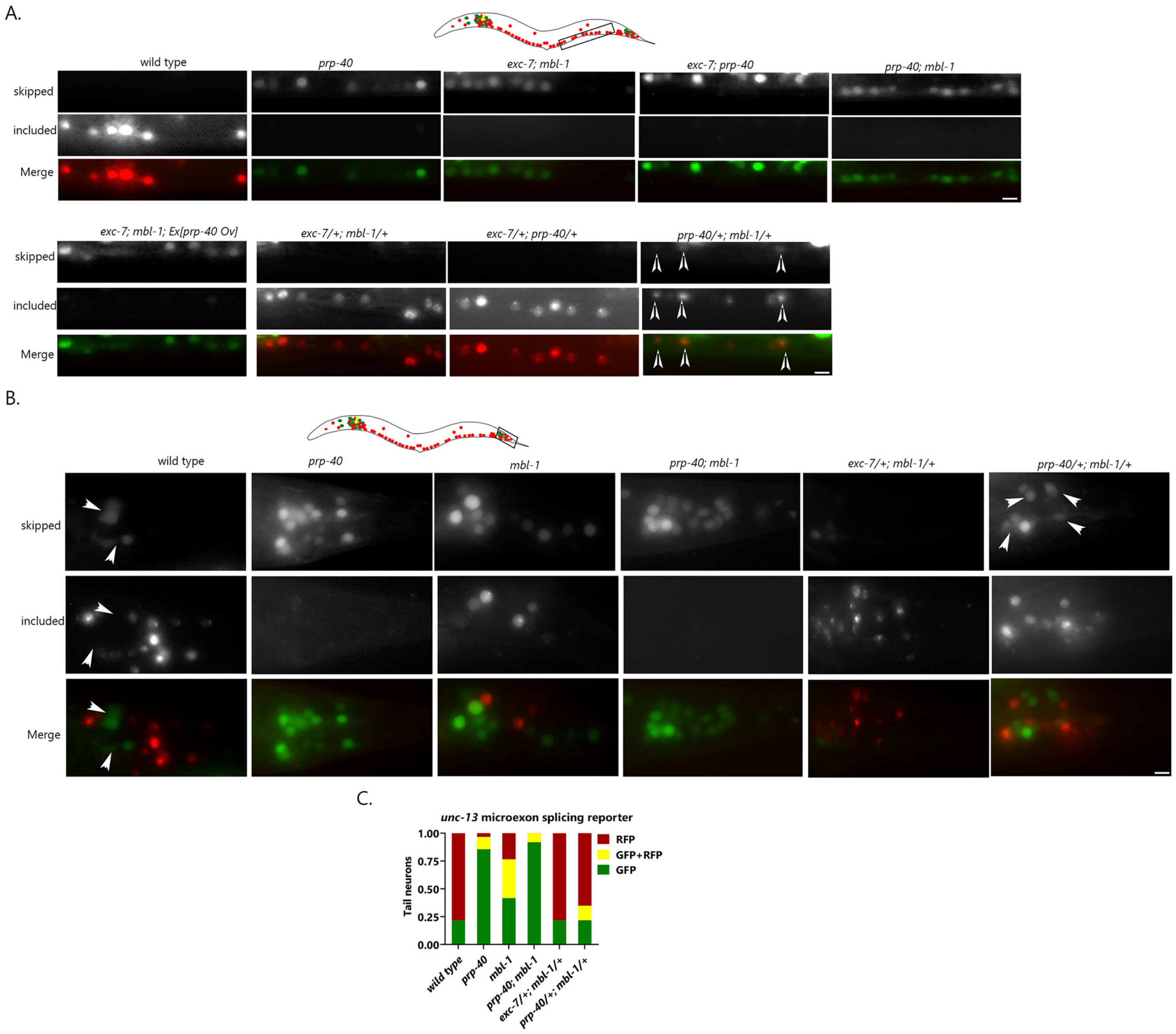

Figure EV4. Genetic interaction between splicing regulators for the alternative splicing of *unc-13* microexon in different neuronal subtypes (also see Fig. 3G, H in the main section for quantification).

(**A**) Upper panel: Worm schematic showing the splicing reporter pattern and the below images are from the area marked by dotted rectangle in the ventral cord region. Note that the wild-type control panel is the same worm as in Figs. 2D and 1F. Bottom panel: Representative images of the ventral cord region of the for various genotypes. White arrowheads mark the GFP appearance in the double heterozygous animals. (**B**) Upper panel: A worm schematic showing splicing reporter with the area marked by a dotted rectangle in the tail region is represented in the below mentioned images for various genotypes. Bottom panel: Representative images of the tail neurons in the mentioned genotypes. White arrowheads in the wild type panel marks a set of tail neurons expressing the skipped isoform (GFP) (**C**) Quantification of the splicing analysis of the tail neurons in the indicated genotypes. *n* = 15–20 animals. Scale bar 10 μm.

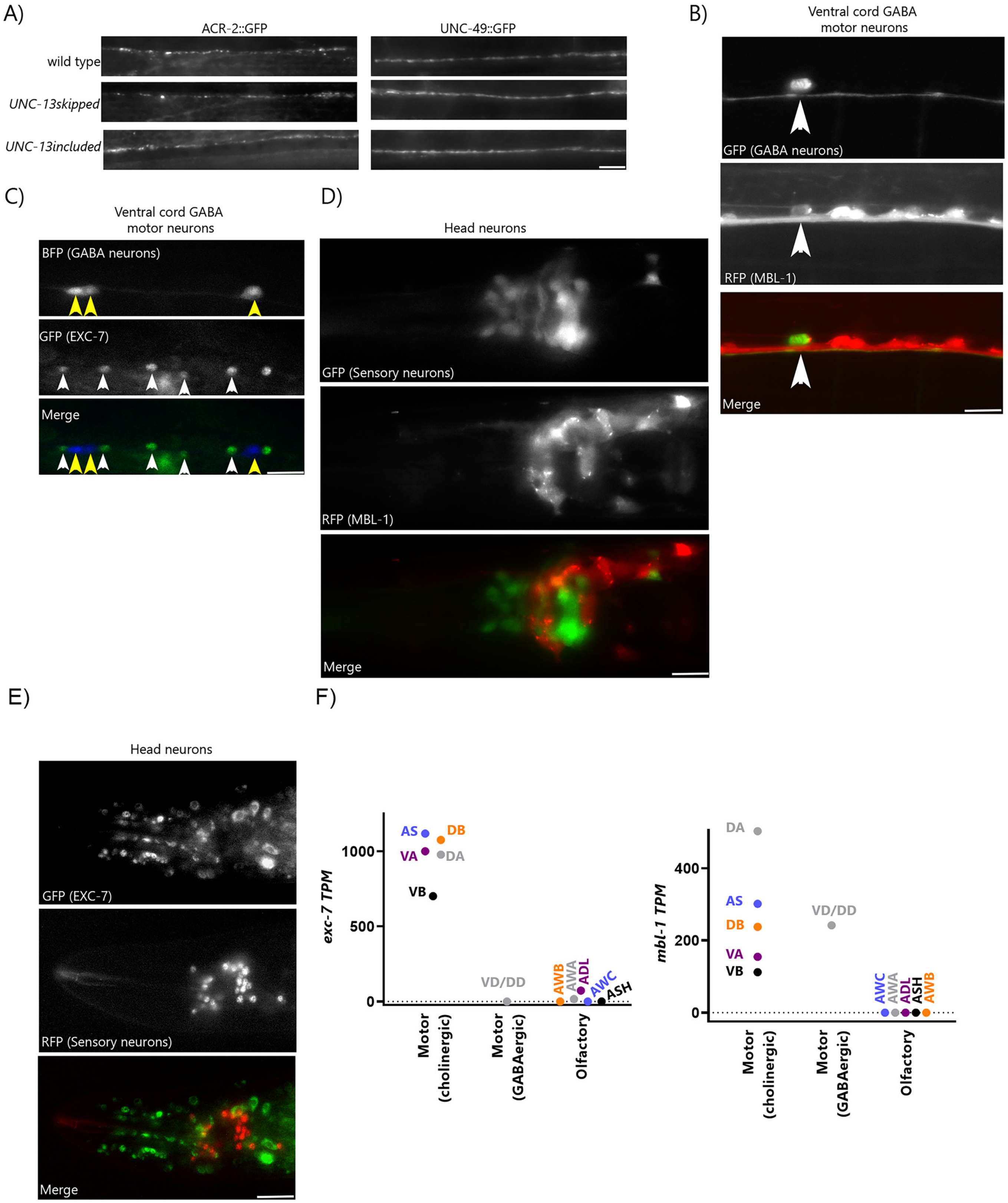
A)
ACR-2::GFP
UNC-49::GFP
wild type
UNC-13skipped
UNC-13included
B)
Ventral cord GABA motor neurons
GFP (GABA neurons)
RFP (MBL-1)
Merge
C)
Ventral cord GABA motor neurons
BFP (GABA neurons)
GFP (EXC-7)
Merge
D)
Head neurons
GFP (Sensory neurons)
RFP (MBL-1)
Merge
E)
Head neurons
GFP (EXC-7)
RFP (Sensory neurons)
Merge
F)
exc-7 TPM
1000
500
0
AS
DB
VA
DA
VB
VD/DD
AWB
AWA
ADL
AWC
ASH
Motor (cholinergic)
Motor (GABAergic)
Olfactory
mbl-1 TPM
400
200
0
DA
AS
DB
VA
VB
VD/DD
AWC
AWA
ADL
ASH
AWB
Motor (cholinergic)
Motor (GABAergic)
Olfactory

 © The Author(s)

Figure EV5. Localization of SNB-1::GFP in dorsal cords and expression analysis of RBPs EXC-7 and MBL-1 by using reporter strains in addition to the CeNGEN database.

(**A**) Representative images of localization of post-synaptic markers such as acetylcholine receptors (left panel) and GABA receptors (right panel) in the muscle compartment. (**B**) The upper panel shows GABA motor neurons (white arrowhead) in the ventral cord, which colocalize with MBL-1::RFP driven by a fosmid. (**C**) GABA motor neurons in the ventral cord marked by BFP (yellow arrowheads) driven under *unc-25* promoter does not colocalize with EXC-7::GFP (marked by white arrowheads) in ventral cord neurons. (**D**) Sensory neurons marked by GFP driven under *osm-6* promoter and MBL-1::RFP expression by the fosmid, indicates many of the sensory neurons does not have detectable MBL-1 expression. (**E**) The upper panel indicates EXC-7::GFP expression in the head region, and the sensory neurons marked by HIS-11::mcherry driven by *osm-6* promoter (middle panel). Sensory neurons have little to no detectable expression of EXC-7 as scored by the reporter analysis. (**F**) Single- cell RNA seq analysis for *exc-7* and *mbl-1* transcripts in multiple sensory and motor neurons (GABA and cholinergic neurons). Analysis indicates significant transcript level in motor neurons for *mbl-1*, whereas a set of sensory neurons lack the same, whereas exc-7 transcripts are lower or undetectable in both sensory and GABA motor neurons, but enriched in cholinergic motor neurons. Scale bar 20 µm.

© The Author(s)
